# Tet-mediated imprinting erasure in H19 locus following reprogramming of spermatogonial stem cells to induced pluripotent stem cells

**DOI:** 10.1038/srep13691

**Published:** 2015-09-02

**Authors:** P. Bermejo-Álvarez, P. Ramos-Ibeas, K.E. Park, A. P. Powell, L. Vansandt, Bickhart Derek, M. A. Ramirez, A. Gutiérrez-Adán, B. P. Telugu

**Affiliations:** 1 Department of Animal and Avian Sciences, University of Maryland, MD, USA; 2 Animal Bioscience and Biotechnology Laboratory, USDA-ARS, Beltsville, MD, USA; 3 Animal Improvement Program Laboratory, USDA-ARS, Beltsville, MD, USA; 4 Departamento de Reproducción Animal, INIA, Madrid, Spain

## Abstract

Selective methylation of CpG islands at imprinting control regions (ICR) determines the monoparental expression of a subset of genes. Currently, it is unclear whether artificial reprogramming induced by the expression of Yamanaka factors disrupts these marks and whether cell type of origin affects the dynamics of reprogramming. In this study, spermatogonial stem cells (SSC) that harbor paternalized imprinting marks, and fibroblasts were reprogrammed to iPSC (SSCiPSC and fiPSC). The SSCiPSC were able to form teratomas and generated chimeras with a higher skin chimerism than those derived from fiPSC. RNA-seq revealed extensive reprogramming at the transcriptional level with 8124 genes differentially expressed between SSC and SSCiPSC and only 490 between SSCiPSC and fiPSC. Likewise, reprogramming of SSC affected 26 of 41 imprinting gene clusters known in the mouse genome. A closer look at H19 ICR revealed complete erasure in SSCiPSC in contrast to fiPSC. Imprinting erasure in SSCiPSC was maintained even after *in vivo* differentiation into teratomas. Reprogramming of SSC from *Tet1* and *Tet2* double knockout mice however lacked demethylation of H19 ICR. These results suggest that imprinting erasure during reprogramming depends on the epigenetic landscape of the precursor cell and is mediated by TETs at the H19 locus.

Being one of the few exceptions to Mendelian rules, genomic imprinting is an epigenetic mechanism that allows for a subset of genes to be expressed either from the paternally or from the maternally inherited allele^1^. Regulation of this mono-allelic gene expression takes place at imprinting control regions (ICRs) by different patterns of DNA methylation. These methylation marks are established during gametogenesis in a sex-specific manner and remain unaltered after syngamy, escaping global demethylation taking place during preimplantation development^2^,^3^. Thus, under physiological conditions, imprinting marks are only erased during primordial germ cell (PGC) formation prior to the establishment of sex-specific methylation marks^4^, making it difficult to study the molecular mechanism underpinning the erasure and establishment of imprinting marks.

Cell reprogramming, either oocyte-mediated^5^ or artificially induced by the overexpression of Yamanaka factors^6^ is able to modify the epigenetic landscape of the precursor cell closely mimicking stem cells, and resulting in the acquisition of pluripotency. However, the reprogramming process does not recreate an exact replica of the epigenome of a stem cell^7^. Oocyte-mediated cell reprogramming by somatic cell nuclear transfer often results in aberrant imprinting patterns, and is one of the major factors contributing to growth abnormalities and low survival rates of cloned embryos^8^. Similarly, induced pluripotent stem cells (iPSC) retain some of the epigenetic marks of its original differentiated state, and the reprogramming process may disrupt other epigenetic marks, such as imprinting^9^.

In this article we have developed a Systems Biology approach to study the mechanisms involved in the erasure of genomic imprinting. In particular, we show that the reprogramming of spermatogonial stem cells (SSC), a germ-line committed stem cell type that exhibit an androgenetic imprinting pattern in H19 and Igf2r^10^ to SSCiPSC entails the erasure of the methylation mark of H19 ICR. Taking advantage of this model we have studied the role of ten-eleven translocation proteins (Tets) in imprinting erasure. Tets are the only known enzymes that convert 5-methylcytosine (5 mC) to 5-hydroxymethylcytosine (5 hmC)^11^, a critical step for the ultimate removal of the methyl mark, and have been reported to be involved in DNA demethylation in oocytes^12^ and PGCs^13^.

## Results

### Yamanaka factor mediated reprogramming of SSC and somatic cells

**Figure 1:**
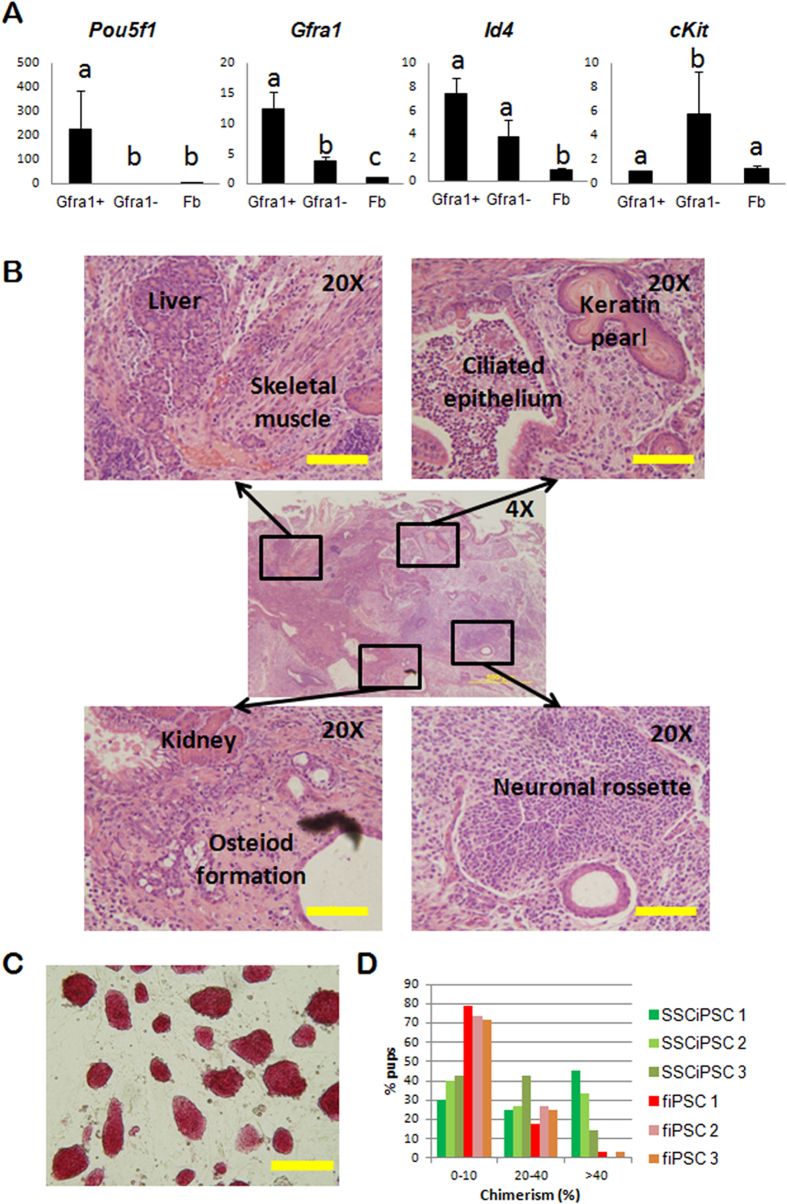
Reprogramming and characterization of iPSC derived from SSC. (**A**) Relative mRNA abundance determined by qPCR of three genes expressed in SSC (*Pou5f1*, *Gfra1* and *Id4*) and a marker of differentiated spermatogonia (*cKit*) in the testicular population sorted for Gfra1 (Gfra1+), unsorted (Gfa1−), and somatic fibroblast controls. Different letters indicate significant differences between treatments based on ANOVA (p < 0.05). (**B**) H&E histological section of a subcutaneous teratoma generated by SSCiPSC injection into immunocompromised mouse. The central image shows a 4X overview of the teratoma [Scale bar: 500 μM]. Four areas are amplified (20X) in the side images showing derivatives from the three germ layers [Scale bar: 100 M]. (**C**) Representative 10X light microscope image of SSCiPSC colony morphology after alkaline phosphatase staining [Scale bar: 200 μM]. (**D**) Percentage of skin chimerism in pups derived from 3 different SSCiPSC lines (green bars) or fiPSC (red-brown bars).

SSC and fetal fibroblast were obtained from a mouse model harboring Yamanaka factors under the control of a doxycycline inducible promoter (*Gt(ROSA)26Sor*^tm1(rtTA*M2)Jae^*Col1a1*^*tm3(tetO-Pou5f1,-Sox2,-Klf4,-Myc)Jae*^/J), which allows for a homogeneous and replicable expression of *Oct4*, *Sox2*, *Klf4* and *c-Myc* (OSKM) in the presence of doxycycline^14^. SSC were obtained from postnatal day (PND) 6 testis by sequential enzymatic digestion, differential plating followed by Magnetic Activated Cell Sorting (MACS) for the SSC surface marker Gfra1, which resulted in the selection of 3 ± 0.8% of differentially adherent cell population. The MACS enriched Gfra1+ cells showed a gene expression profile compatible with SSC as determined by Quantitative RT-PCR analysis (Fig. 1A). As expected, Gfra1+ cells but not Gfra1- expressed *Pou5f1*, a key marker for stemness in SSC^15^. Likewise, Gfra1 was upregulated in the cells sorted for Gfra1, whereas the expression of *Id4*, another marker of SSC, was expressed at a higher level in Gfra1+ compared to Gfra1- cells, but the differences were not statistically significant (ANOVA p<0.05). A marker of differentiated spermatogonia *cKit*^16^, was significantly upregulated in the Gfra1- population compared to Gfra1+ fraction.

Both SSC and fetal fibroblast were reprogrammed to iPSC in the presence of doxycycline and LIF. Colonies of iPSC began to emerge 10–12 days after doxycycline induction, as reported previously for this model^14^. Both SSCiPSC and fiPSC were morphologically indistinguishable, grow at a similar pace and were alkaline phosphatase positive (Fig. 1C), a distinguishing characteristic between SSC and Germinal Stem Cells (GSC)^17^. These characteristics were maintained following gradual withdrawal of doxycycline from the culture media. Doxycycline-independent SSC- and fiPSC were subjected to further pluripotency tests: teratoma formation and contribution to chimaeras. SSCiPSC were able to form teratomas following subcutaneous and intramuscular injection in immunocompromised mice. As shown in Fig. 1B and Supplementary Fig. S1A, the teratoma contained derivatives from the three germ layers, including neuronal rossettes and keratin pearls (ectoderm); skeletal muscle, osteoid formations, and kidney (mesoderm); and, liver and ciliated epithelium (endoderm).

The ability to generate chimeras was tested in 3 different cell lines from each cell type (SSCiPSC and fiPSC). Skin chimerism was detected based on the agouti coat color from injected iPSC, over the white coat color of blastocyst donor CD1 strain. All cell lines were able to contribute to chimeras to a certain degree. In terms of skin chimerism, the 3 SSCiPSC lines outperformed fiPSC lines, resulting in a significantly higher number of pups with a high skin chimerism (>40%) than fiPSC based on Chi-square test (Fig. 1D, Supplementary Fig. S1B, and Supplementary Table S1).

### Global transcriptome profile of SSC and reprogrammed iPSC

**Figure 2:**
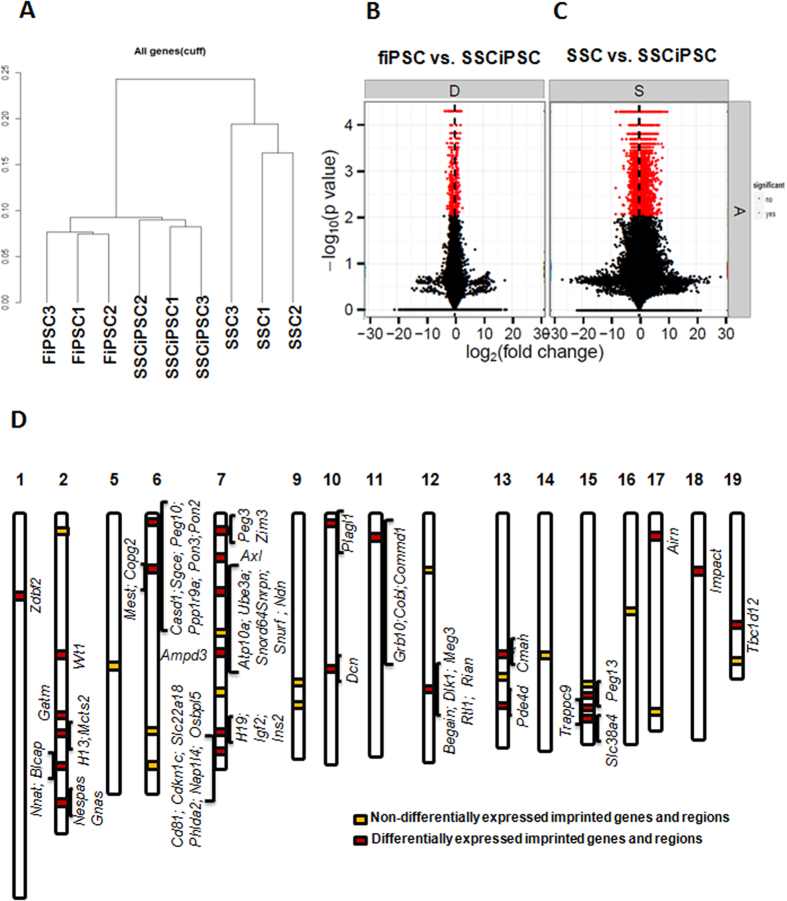
Global transcriptome profile of imprinted and non-imprinted loci as revealed by RNAseq analysis. (**A**) Hierarchical clustering of the nine biological samples analyzed; (**B,C**) Differentially expressed genes (FDR<0.05) between SSC and SSCiPSC (8124 genes, (B)) or fiPSC and SSCiPSC (490 genes, (**C**)); (**D**) Representation of the 41 known imprinting clusters along the mouse genome. Yellow squares depict clusters (15) with no differentially express genes, red squares denote clusters (26) with differentially expressed imprinted genes.

RNA-seq was performed to investigate the transcriptional differences between SSCiPSC, fiPSC and SSC (GEO Accession# GSE64856; Supplementary Table S2 online). Hierarchical clustering of the RNA-seq data revealed grouping of iPSC (SSCiPSC and fiPSC) distinctly from SSC (Fig. 2A and Supplementary Fig. S2). In particular, the comparison of SSCiPSC vs SSC revealed 8124 genes differentially expressed, whereas only 490 were differentially expressed between SSCiPSC and fiPSC (FDR<0.05, Fig. 2B,C; Supplementary Figs S2 & S3). The differentially expressed genes between SSC and SSCiPSC were similar to previous publications comparing SSC and GSC (pluripotent cells spontaneously derived from SSC) gene expression at the transcriptional level. In particular, 33 out of the 54 genes analyzed in these publications^17^,^18^,^19^ were regulated similarly between SSC and SSCiPSC or SSC and GSC, with only 3/54 regulated otherwise (Supplementary Table S3). The conversion of SSC into SSCiPSC entailed the complete transcriptional silencing of several genes essential for male fertility such as *Ins2*, *Lhcgr*, *Lhx1*, *Nanos2*, *Nr5a1*, *Serpina 5* and *Tcf21* (Fig. 2D; Supplementary Table S2). SSCiPSC expressed higher levels of genes involved in both DNA demethylation (*Tet1* and *Tet2*) and DNA methylation (*Dnmt1*, *Dnmt3a* and *Dnmt3b*) (Supplementary Fig. S3). Gene Ontology of the 3287 genes displaying a higher fold change and statistical significance between SSCiPSC and SSC revealed that regulation of transcription and transcription were the biological processes most affected by reprogramming, and non-membrane-bounded organelle and mitochondrion are the more abundant cellular components related to the differentially expressed genes (Supplementary Fig. S4 & Supplementary Table S4). KEGG pathway analysis suggested a reprogramming-mediated bias in pathways in cancer and MAPK signaling pathway, among others (see Supplementary Table S4 online). We next determined the extent of transcriptional reprogramming in imprinting genes by analyzing how many of the imprinting clusters along the mouse genome were affected by reprogramming of SSC to SSCiPSC (Fig. 2D). Out of 41 known imprinting clusters^20^, 26 of them were differentially expressed, showing extensive transcriptional changes at imprinting clusters during reprogramming.

### Imprinting erasure in SSCiPSC at H19 ICR

**Figure 3:**
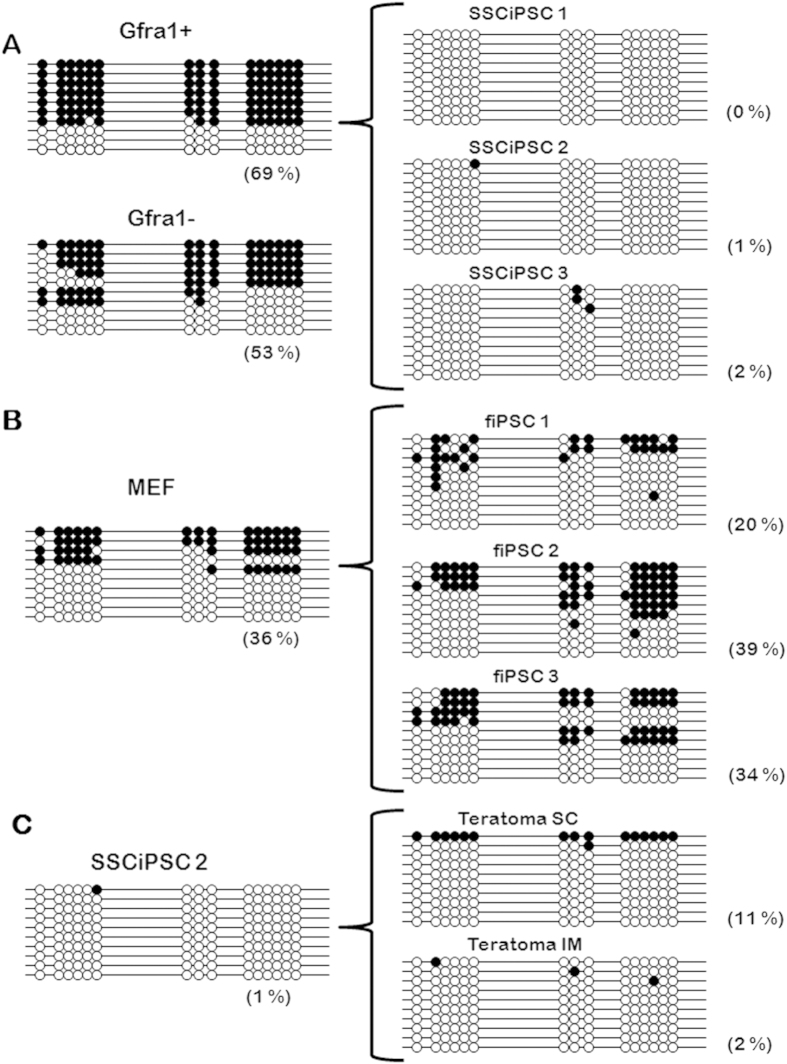
CpG methylation of H19 Imprinted Controlled region following reprogramming and differentiation. Bisulfite sequencing of H19 ICR revealed that CpG methylation was almost completely erased following reprogramming of (**A**) SSCiPSC (68% in SSC vs 0–2% in SSCiPSC) in comparison to (**B**) fiPSC (48% in fibroblast vs 20–39.3% in fiPSC). (**C**) The loss of imprinting erasure in SSCiPSC is maintained even following differentiation.

To investigate the extent and mechanism of imprinting erasure, the methylation profiles at H19 ICR in the original cells (Gfra1+ SSC, Gfra1-spermatogonia, and fetal fibroblast) and in 3 iPSC cell lines derived from each resultant cell type were analyzed (Fig. 3). As expected, H19 was highly, but not totally methylated in Gfra1+ cells, in agreement with previous findings in immature spermatogonia^10^,^21^, whereas fibroblast showed the typical pattern of methylation with a mix of methylated paternal alleles and unmethylated maternal alleles. The methylation profile of the differentiated spermatogonia (Gfra1-) did not differ from that of the SSC (Gfra1+). Following reprogramming of SSC to SSC-iPSC, methylation at H19 ICR was erased almost completely in all three representative SSC-iPSC cell lines (Fig. 3A). In contrast, the reprogramming of fibroblast to the fiPSC did not significantly alter the methylation of H19 ICR based on ANOVA (p < 0.05) (Fig. 3B). Next, we questioned whether the imprinting marks could be restored in SSC-iPSC following *in vivo* differentiation, by analyzing the methylation status of H19 ICR in teratomas. *In vivo* differentiation of iPSC did not affect the methylation levels at H19 ICR, as teratomas produced either by intramuscular or subcutaneous injections revealed similar methylation patterns as the SSCiPSC line from which they were derived (Fig. 3C).

### Erasure of imprinting following reprogramming of SSC to SSCiPSC is dependent on Tets

**Figure 4:**
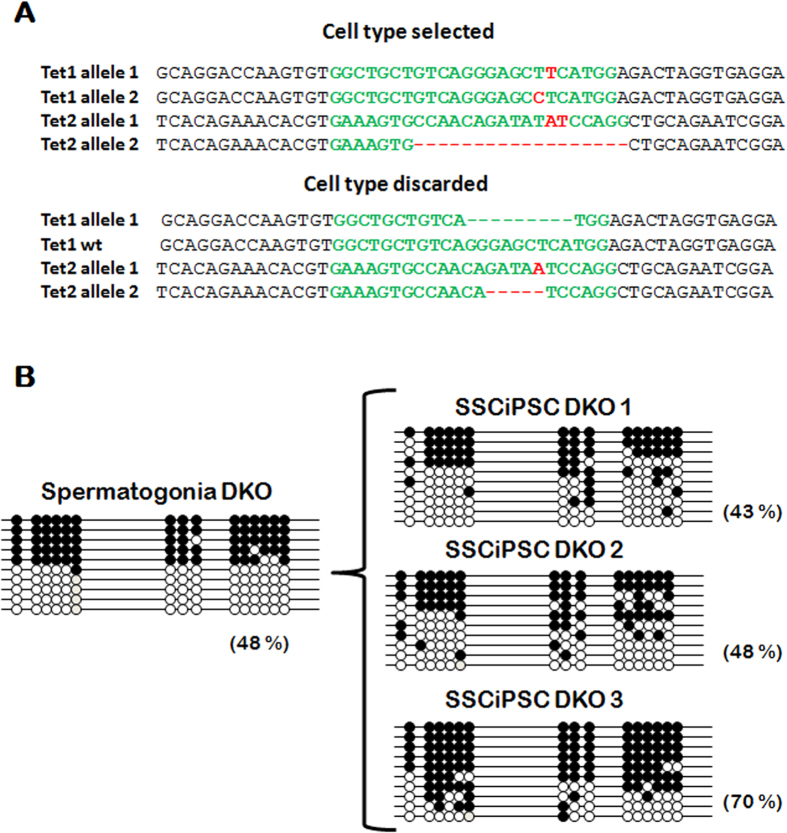
Methylation at H19 following reprogramming of *Tet1* and *Tet2* KO (DKO). (**A**) CRISPR/Cas mediated knockout of endogenous *Tet1* and *Tet2* loci by intra-cytoplasmic injection of *in vitro* transcribed Cas9 mRNA and sgRNA into mouse 2-cells embryos. CRISPR injection resulted in indels and two different genotypes. The CRISPR target sequence is indicated in green letters, whereas red letters indicates the indels generated. (**B**) Methylation at H19 ICR following reprogramming of Tet1 and Tet2 knockout SSC into three different lines of SSCiPSC.

In order to determine the molecular mechanism behind the erasure of H19 imprinting mark following reprogramming of SSC, we knocked-out *Tet1* and *Tet2* in *Gt(ROSA)26Sor*^tm1(rtTA*M2)Jae^*Col1a1*^*tm3(tetO-Pou5f1,-Sox2,-Klf4,-Myc)Jae*^/J mice by CRISPR/Cas9 system^22^. Initial attempts to microinject zygotes in this strain were unsuccessful because of zygote lysis, therefore the mRNA encoding for Cas9 and the sgRNA were co-injected into each blastomere at 2-cell embryos, an approach that resulted in mosaics. Other authors have reported that most *Tet1*+*Tet2* double knockout (DKO) embryos die in utero likely due to epigenetic defects^23^. Although this was not the aim of our study and we did not specifically test this possibility by analyzing embryo resorptions, we have noticed a lower than expected survival rate to term, with two recipients failing to deliver any offspring, despite gaining weight after embryo transfer, and a third delivering 3 pups out of 7 transferred embryos. Furthermore, only one of the three pups contained DKO cells. The low survival to term may be consistent with fetal mortality in the DKO, but may also be caused by the use of CRISPR. The male pup containing DKO cells was a mosaic individual consisted of two genotypes, one showing frame-disrupting mutations in both alleles of both *Tet1* and *Tet2* (DKO) and another showing frame-disrupting mutations in *Tet2* but an in-frame indel and a non-edited allele in *Tet1* (Tet2 KO cells) (Fig. 4A). The testicular morphology at PND6 and the percentage of Grfa1+ cells in the mosaic pup was similar to wild type (wt) individuals. As both DKO and Tet2 KO cells contributed to the germ line, individual SSCiPSC were clonally propagated, genotyped, and three clonal lines that are confirmed DKO for both alleles of both *Tet1* and *Tet2* genes were selected. The kinetics of colonies appearance and cell growth from Tet1 and Tet2 DKO SSCiPSC were similar to those of the wild type pup, with the colonies appearing 10–12 after doxycycline induction and being passaged every 2–3 days. Following the same procedures for reprogramming as described earlier, doxycycline was gradually withdrawn, and the methylation status was analyzed. The ablation of Tet1 and Tet2 did not alter the methylation status of H19 ICR in spermatogonia, which was similar to the wt SSC (Figs 3A and 4B). However, in contrast to SSC-iPSC from wt animals, SSC-iPSC lacking Tet1 and Tet2 retained the methylation levels of SSC, suggesting an essential role of these enzymes in imprinting erasure at H19 imprinting locus during iPSC derivation.

## Discussion

The regulation of DNA demethylation at ICR is uncoupled from the global demethylation changes leading to pluripotent states in early embryo. Although imprinting is erased during PGC formation^24^, the reprogramming of gamete genomes during early pre-implantation development does not affect imprinting marks, and evade the genome-wide demethylation events occurring in both pronuclei^2^,^3^. The molecular mechanism behind the protection of ICRs from demethylation remains unclear and it has been speculated that ICR demethylation might require mechanisms different from the demethylation occurring at other regions. The observation of the dynamics of methylation at ICR following artificially induced reprogramming may prove useful in understanding this process. In this regard, cell fusion-based reprogramming studies have been very informative. The fusion of mouse and human B cells with mouse EGC (Embryonic Germ Cells, pluripotent cells derived *in vitro* from PGC) resulted in the erasure of the imprinting marks, whereas the fusion with ESC, which also reprogram the B cell genome, does not erase the imprinting marks^25^,^26^. This result can be interpreted two ways: 1) EGC may contain specific factors required for imprinting erasure; or 2) EGC may harbor a higher demethylation ability compared to ESC that allows for overcoming the final barrier in demethylation of the genome, which is the demethylation of ICRs. Our results turn the balance towards the latter possibility, as we have observed that imprinting erasure following the same iPSC derivation conditions depends on the original cell type, with the iPSC derived from fibroblasts retaining the imprinting marks to some extent, whereas the iPSC from SSC lacking the imprints. The difference may be due to the active expression of *Pou5f1* before reprogramming in SSC compared to fibroblasts^27^. Importantly, both cell types also differ in their chromatin structure, with chromatin being more open and thereby more prone to demethylation in SSC compared to fibroblasts^28^. From this perspective, the imprinting erasure would be more of a dose or time-related effect of the reprogramming factors rather than a specific mechanism for imprinting erasure, which is distinct from demethylation at other genomic sites. In conclusion, a more intense demethylating process or the same process in an already partially reprogrammed genome leads to imprinting erasure.

The notion of a cumulative effect of demethylating agents acting on imprinting erasure rather than a specific mechanism for demethylation at ICR agrees with previous findings in different Systems Biology Approaches. The demethylation of ICR is a late event in cell fusion-based mechanism occurring much more slowly than the demethylation occurring at the *Pou5f1* locus, and in both cases it seems that there is a conversion of 5 mC to 5 hmC. Tets are the only enzymes known to catalyze 5 mC hydroxylation and both Tet1 and Tet2 are present in EGC and ESC, the two cell types differing in their ability to abolish imprinting marks following cell-fusion based reprogramming^26^. In agreement with our results, the spontaneous *in vitro* derivation of pluripotent cells from unipotent GSC (the *in vitro* equivalent of SSC) report different patterns of ICR methylation depending on the cell type of origin. Pluripotent stem cells derived from GSC of newborn testis (mGS) lose the imprinting of two paternally methylated ICR *H19*, *Meg3 IG* and keep the maternally methylated *Rasgrf1*, *Igf2r* and *Peg10*^17^ unmethylated, whereas multipotent SSC obtained from adult testes (called gPS) showed a gene expression pattern similar to ESC but exhibit an androgenetic imprinting pattern in H19 and Igf2r^18^. However, the study of the molecular mechanism behind the imprinting erasure during the conversion of GSC to mGS present some limitations, as mGS appeared spontaneously during GSC culture without the introduction of any particular molecule or exogenous reprogramming factor, and thereby driven by unknown factors^17^. In this regard, the use of Yamanaka factor based reprogramming of SSC provides a simple and highly reproducible means for studying imprinting erasure. During the revision of this manuscript, a novel type of EGCs (iEGC) derived from PGCs by culture of PGC with kenpaullone and an inhibitor of TGFβR have been reported^29^. The iEGC showed an increased methylation in DMR of H19 locus compared to PGCs as measured by combined bisulfite restriction analysis (COBRA). Assuming that the methylation of that single DMR is representative of the entire ICR, this result could be explained by the very distinct derivation process of iEGCs mediated by small molecules rather than by prolonged overexpression of Yamanaka factors as in SSCiPSC. This is in agreement with the notion that a more intense demethylating process rather than an imprinting-specific demethylating process mediates imprinting erasure.

A particularly relevant observation is that imprinting erasure was not reversed following *in vivo* differentiation into teratomas. Genomic imprinting is an epigenetic feature generally overlooked in iPSC and while the imprinting erasure did not impair the differentiation abilities of the SSCiPSC, the functionality of these differentiated cells could be affected. As imprinting has been shown to be affected in other types of iPSC^9^, these results raise a reason for caution for the use of iPSC in regenerative medicine. Alterations in the methylation of different ICRs are known to be responsible for disorders originated during development, such as Angelman, Beckwith-Wiedemann and Prader Willi syndromes, but they are also linked to the appearance of cancers, both spontaneous^30^,^31^,^32^ or derived from ESC with aberrant ICR methylation^33^.

As plausible drivers for imprinting erasure, Tets (Tet1, Tet2 and Tet3) are the only known enzymes that convert 5-methylcytosine (5 mC) to 5-hydroxymethylcytosine (5 hmC), an intermediate in the 5 mC demethylation process that undergo passive demethylation as cells divide^34^. It has been observed that somatic cells deficient in *Tet2*^35^ or all three *Tet* enzymes^36^ cannot be reprogrammed into iPSC. In contrast, we have been able to reprogram DKO SSC to SSCiPSC, with a similar efficiency and timing of colonies appearance as wt SSC, so *Tet1* and *Tet2* were dispensable for SSC reprogramming. A possible explanation for the differences between MEF and SSC reprogramming could be that SSC do not require a mesenchymal-to-epithelial transition, the critical step blocked by *Tet* deficiency^36^, but testing this hypothesis would require dedicated investigation. *Tet1* and *Tet2* are the only Tet members expressed in PGCs or ESC^26^, and the temporal expression of *Tet1* and *Tet2* coincides in time with the conversion of 5 mC to 5 hmC at ICR in PGCs, so they have been suggested to play a role in imprinting erasure^13^. *Tet1* paternal or maternal KO (i.e. pups derived from a *Tet1* KO male or female) E9.5–10.5 embryos were observed to dysregulate several imprinted genes by hypermethylation of paternal or maternal ICR, respectively^37^. Furthermore, the PGCs from *Tet1* and *Tet2* DKO embryos are deprived from 5 hmC. These pups were fertile and some of its progeny shows altered imprinting in *H19*, *Mest*, *Peg3* and *Igf2r*^23^. The presence of pups derived from DKO crosses with wt mouse with normal imprinting patterns was explained by the action of *Tet3*, not expressed in PGC but expressed later during gametogenesis and spermatogenesis^26^,^37^. In our system, we have observed that *Tet1* and *Tet2* DKO SSC does not lose their imprinting marks following reprogramming to SSCiPSC, suggesting an essential role of these enzymes in the process. Our findings are in agreement with the critical role for *Tet* enzymes in the erasure of imprinting mediated by cell fusion between B cells and EGC, where *Tet1* depleted EGC were unable to accumulate 5hmC and erase the imprinting marks on the B cell genome following fusion^26^. However, despite proving an essential role of *Tet* enzymes in imprinting erasure in our model, we cannot rule out that passive DNA demethylation may have also played a role.

Collectively, these results highlight that imprinting erasure following Yamanaka factors based reprogramming depends on the epigenetic status of the original cell, with iPSC obtained from highly differentiated cells (fiPSC) being more able to retain the imprinting mark than SSCiPSC. This observation proposes that imprinting marks are specially protected from demethylation and that reprogramming at imprinted loci is a late step in genome reprogramming driven by the cumulative action of demethylating agents, with Tet enzymes playing an essential role. In this regard, Tet mediated imprinting erasure does not seem to require a specific demethylation mechanism, in agreement with the lack of consensus target sequences at ICRs^37^, but from an overall demethylated genome, or a genome more prone to demethylation.

## Methods

### SSC enrichment and fetal fibroblasts culture

All experiments involving vertebrate animals were performed in accordance with the approved guidelines of Beltsville Area Animal Care and Use Committee (BAACUC). All experimental protocols involving vertebrate animals were approved by the Institutional BAACUC committee. Fetal fibroblast and SSC were obtained from the mouse model *Gt(ROSA)26Sor*^tm1(rtTA*M2)Jae^*Col1a1*^*tm3(tetO-Pou5f1,-Sox2,-Klf4,-Myc)Jae*^/J^14^. Germinal cells were enriched from PND6 testis by differential plating. Briefly, testicular stroma was minced and digested with 1 mg/ml type IA collagenase (Sigma) for 10 min at 37 °C, followed by digestion with 0.05% trypsin-EDTA (Gibco) for 5 min at 37 °C. Cell suspension was purified through a 70 μm filter and plated onto a gelatin coated dish for 2 hours, allowing testicular somatic cells to attach to the plate. Germinal floating cells were subjected to MACS (Miltenyi Biotech) for Gfra1, a SSC membrane marker^38^ for SSC enrichment following the manufacturer instructions, using polyclonal rabbit anti-Gfra1 antibody (SantaCruz Biotechnology) at 1:50 dilution. Fetal fibroblasts were obtained from single E13.5 mice fetuses following standard protocols and by PCR as previously described^39^ to select males in order to avoid any possible bias due to sex^40^.

### Derivation of iPSC

SSC and fetal fibroblasts were cultured following standard protocols in the presence of 2 μg/ml Doxycycline hyclate (Sigma). For the first passage, individual colonies were picked up and individually trypsinized to obtain clonal lines. Doxycycline concentration was kept at 2 μg/ml for the first 5 passages and then was gradually withdrawn (1, 0.5 and 0.25 μg/ml on passages 6, 7 and 8 respectively). After passage 8, cells were incubated without doxycycline.

### Teratoma formation and histology

To test teratoma formation ability, SSCiPSC were injected into NIH-bg-un-Xid immunocompromised mice. Two million cells were diluted in Matrigel (Corning) and injected subcutaneously. The same number of cells was injected intramuscularly diluted in culture media. One month after injection, teratoma growth was obvious and the mice were sacrificed and the teratomas collected and fixed in 4% paraformaldehyde. Fixed tissues were dehydrated through ethanol washes, paraffin embedded, sectioned and stained in hematoxylin/eosin (American Histolabs). An inner ~1 mm^3^ biopsy of the teratomas was collected to determine DNA methylation. As this procedure does not exclude the possibility of host cell contamination, the percentage of contamination was estimated by a PCR with three primers (Geno OSKM, Supplementary Table S5) designed to amplify a 650 bp band in wt mice (host cells) and 340 bp in the Gt(ROSA)^26Sortm1(rtTA*M2)Jae^Col1a1^tm3(tetO-Pou5f1,-Sox2,-Klf4,-Myc)Jae^/J iPSC derivatives. Comparison of band intensity between the PCR products amplified from teratomas or from standard mixes of known amounts of wt and transgenic DNA estimated an ~5% host cell contamination in the samples used for DNA methylation (Supplementary Fig. S5). This contamination may have been responsible for the fully methylated clone obtained in IM teratoma (Fig. 3C).

### Chimera formation

SSCiPSC and fiPSC were individualized and injected into CD1 blastocysts (10 iPSC/embryo). The injected blastocysts were transferred by uterotubal embryo transfer to pseudopregnant recipients. Three different lines from each cell type were tested, performing 3 to 5 embryo transfers and obtaining 15 to 30 weaned pups per cell line analyzed (Supplementary Table S1). Skin chimerism was estimated immediately after weaning (21 days after birth) and differences between treatments were determined by Chi-square (SigmaStat).

### Gene expression analysis

In order to minimize the contamination with the feeder cells, iPSC at passages 13-14 were purified by MACS for SSEA1 following a protocol similar to the one described for SSC enrichment for Gfra1, but using anti-SSEA-1 (CD15) microbeads (Miltenyi Biotech). The purified cells were used for gene expression and DNA methylation analysis. qPCR was performed as previously described^40^ and differences between groups were analyzed by ANOVA (SigmaStat). Global transcriptional differences between SSC, SSCiPSC and fiPSC were analyzed by deep sequencing of RNA libraries. Total RNA was extracted with miRNeasy Minikit following manufacturer instructions. RNA concentration, purity and integrity were verified by Nanodrop (Eppendorf) and Nanochip on Bioanalyzer (Agilent). The conversion of the mRNA in total RNA into a library of template molecules suitable for subsequent cluster generation and DNA sequencing was performed following the TruSeq® Stranded mRNA kit (Illumina, San Diego, CA, USA). Libraries were quantified by Quant-iT PicoGreen followed by qPCR. Libraries are clustered using TruSeq PE Cluster Kit v3 – cBot HS, and sequenced using TruSeq SBS Kit v3 - HS (200-cycles). Paired end 100 bp reads were processed and analyzed using Cufflinks and Tophat software platforms. Statistical analysis was performed using Cummerbund R software. Gene Ontology analysis was performed using DAVID Functional Annotation Tools (http://david.abcc.ncifcrf.gov/), and differentially expressed genes with p-value ≤ 5*10^−5^ and fold change ≥ 1.5 were categorized with respect to Molecular Function, Biological Process and Cellular Component. The annotated genes were also mapped into relevant functional groups in a pathway analysis according to the Kyoto Encyclopedia of Genes and Genomes (KEGG).

### DNA methylation analysis

200 ng of DNA from fibroblasts, FiPS, SSC, SSCiPS and teratomas were treated with bisulfite to convert all unmethylated cytosine to uracil using EZ DNA Methylation-Direct Kit (Zymo Research) according to the manufacturer instructions. A 422 bp fragment of the 2 kb ICR of *H19-Igf2*^21^, containing 2 of the 4 CCTF binding sites as previously described^41^ (Supplementary Fig. S6), was amplified in the modified DNA by nested PCR using primer sets specific for the mutagenized DNA shown in Supplementary Table S5. The amplified DNA fragments were verified gel extracted (Qiaex II) and cloned into the pCR 2.1 TOPO TA (Life Technologies) by Blunt/TA ligase (New England Biolabs). The ligation product was transformed into competent cells and the plasmids obtained from ten individual clones from each group were sequenced (Macrogen, Rockville, MD, USA). ANOVA (SigmaStat) was performed on the percentages of methylated CpG per clone to determine statistical differences between groups.

### Generation of Tet1+Tet2 DKO

The double KO for Tet1 and Tet2 was generated by CRISPR/Cas system. Capped polyadenylated Cas9 mRNA was produced by *in vitro* transcription (mMESSAGE mMACHINE T7 ULTRA kit, Life Technologies) from the plasmid pMJ920 (Addgene 42234) linearized with BstBI and treated with Antarctic phosphatase (NEB). Single guide RNAs (sgRNA) for *Tet1* and *Tet2*, previously described in^22^, were produced by *in vitro* transcription (MEGAshortscript T7 kit, Life Technologies) from g-Blocks containing T7 promoter. Both the Cas9 mRNA and the sgRNAs were purified using MEGAclear kit (Life Technologies) and eluted in TE buffer. Cas9 mRNA (100 ng/μl) and sgRNA (20 ng/μl) were injected into two cell embryos obtained from *Gt(ROSA)26Sor*^tm1(rtTA*M2)Jae^*Col1a1*^*tm3(tetO-Pou5f1,-Sox2,-Klf4,-Myc)Jae*^/J females. Injected embryos were allowed to develop to the blastocyst stage and transferred to CD1 foster mothers by utero-tubal embryo transfer. Genotyping was performed on DNA extracted from a tail biopsy by using specific primers spanning the target sequence (Supplementary Table S5). The PCR product was purified and cloned into pCR 2.1 TOPO TA (Life Technologies, Grand Island, NY, USA) and transformed into competent cells as above. The indels (insertion/deletion) produced by CRISPR were analyzed in 10 clones per pup.

## Additional Information

**How to cite this article**: Bermejo-Álvarez, P. *et al.* Tet-mediated imprinting erasure in H19 locus following reprogramming of spermatogonial stem cells to induced pluripotent stem cells. *Sci. Rep.* **5**, 13691; doi: 10.1038/srep13691 (2015).

## Supplementary Material

Supplementary Information

## References

[b1] Ferguson-Smith A. C. & Surani M. A. Imprinting and the epigenetic asymmetry between parental genomes. Science 293, 1086–1089 (2001). 11498578 10.1126/science.1064020

[b2] Smith Z. D. *et al.* A unique regulatory phase of DNA methylation in the early mammalian embryo. Nature 484, 339–344 (2012). 22456710 10.1038/nature10960 PMC3331945

[b3] Kobayashi H. *et al.* Contribution of intragenic DNA methylation in mouse gametic DNA methylomes to establish oocyte-specific heritable marks. PLoS genetics 8, e1002440 (2012). 22242016 10.1371/journal.pgen.1002440 PMC3252278

[b4] Labosky P. A., Barlow D. P. & Hogan B. L. Mouse embryonic germ (EG) cell lines: transmission through the germline and differences in the methylation imprint of insulin-like growth factor 2 receptor (Igf2r) gene compared with embryonic stem (ES) cell lines. Development 120, 3197–3204 (1994). 7720562 10.1242/dev.120.11.3197

[b5] Wilmut I., Schnieke A. E., McWhir J., Kind A. J. & Campbell K. H. Viable offspring derived from fetal and adult mammalian cells. Nature 385, 810–813 (1997). 9039911 10.1038/385810a0

[b6] Takahashi K. & Yamanaka S. Induction of pluripotent stem cells from mouse embryonic and adult fibroblast cultures by defined factors. Cell 126, 663–676 (2006). 16904174 10.1016/j.cell.2006.07.024

[b7] Kim K. *et al.* Epigenetic memory in induced pluripotent stem cells. Nature 467, 285–290 (2010). 20644535 10.1038/nature09342 PMC3150836

[b8] Young L. E. *et al.* Conservation of IGF2-H19 and IGF2R imprinting in sheep: effects of somatic cell nuclear transfer. Mech Dev 120, 1433–1442 (2003). 14654216 10.1016/j.mod.2003.09.006

[b9] Stadtfeld M. *et al.* Aberrant silencing of imprinted genes on chromosome 12qF1 in mouse induced pluripotent stem cells. Nature 465, 175–181 (2010). 20418860 10.1038/nature09017 PMC3987905

[b10] Li J. Y., Lees-Murdock D. J., Xu G. L. & Walsh C. P. Timing of establishment of paternal methylation imprints in the mouse. Genomics 84, 952–960 (2004). 15533712 10.1016/j.ygeno.2004.08.012

[b11] Tahiliani M. *et al.* Conversion of 5-methylcytosine to 5-hydroxymethylcytosine in mammalian DNA by MLL partner TET1. Science 324, 930–935 (2009). 19372391 10.1126/science.1170116 PMC2715015

[b12] Gu T. P. *et al.* The role of Tet3 DNA dioxygenase in epigenetic reprogramming by oocytes. Nature 477, 606–610 (2011). 21892189 10.1038/nature10443

[b13] Hackett J. A. *et al.* Germline DNA demethylation dynamics and imprint erasure through 5-hydroxymethylcytosine. Science 339, 448–452, 10.1126/science.1229277 (2013). 23223451 PMC3847602

[b14] Carey B. W., Markoulaki S., Beard C., Hanna J. & Jaenisch R. Single-gene transgenic mouse strains for reprogramming adult somatic cells. Nat Methods 7, 56–59 (2010). 20010831 10.1038/nmeth.1410 PMC3048025

[b15] Kubota H., Avarbock M. R. & Brinster R. L. Spermatogonial stem cells share some, but not all, phenotypic and functional characteristics with other stem cells. Proc Natl Acad Sci USA 100, 6487–6492 (2003). 12738887 10.1073/pnas.0631767100 PMC164473

[b16] Schrans-Stassen B. H., van de Kant H. J., de Rooij D. G. & van Pelt A. M. Differential expression of c-kit in mouse undifferentiated and differentiating type A spermatogonia. Endocrinology 140, 5894–5900 (1999). 10579355 10.1210/endo.140.12.7172

[b17] Kanatsu-Shinohara M. *et al.* Generation of pluripotent stem cells from neonatal mouse testis. Cell 119, 1001–1012 (2004). 15620358 10.1016/j.cell.2004.11.011

[b18] Ko K. *et al.* Induction of pluripotency in adult unipotent germline stem cells. Cell Stem Cell 5, 87–96 (2009). 19570517 10.1016/j.stem.2009.05.025

[b19] Fujino R. S. *et al.* Capillary morphogenesis gene (CMG)-1 is among the genes differentially expressed in mouse male germ line stem cells and embryonic stem cells. Mol Reprod Dev 73, 955–966 (2006). 16705683 10.1002/mrd.20504

[b20] Williamson C. M. *et al.* Uncoupling antisense-mediated silencing and DNA methylation in the imprinted Gnas cluster. PLoS genetics 7, e1001347 (2011). 21455290 10.1371/journal.pgen.1001347 PMC3063750

[b21] Davis T. L., Yang G. J., McCarrey J. R. & Bartolomei M. S. The H19 methylation imprint is erased and re-established differentially on the parental alleles during male germ cell development. Hum Mol Genet 9, 2885–2894 (2000). 11092765 10.1093/hmg/9.19.2885

[b22] Wang H. *et al.* One-Step Generation of Mice Carrying Mutations in Multiple Genes by CRISPR/Cas-Mediated Genome Engineering. Cell 153, 910–918 (2013). 23643243 10.1016/j.cell.2013.04.025 PMC3969854

[b23] Dawlaty M. M. *et al.* Combined deficiency of Tet1 and Tet2 causes epigenetic abnormalities but is compatible with postnatal development. Dev Cell 24, 310–323 (2013). 23352810 10.1016/j.devcel.2012.12.015 PMC3574201

[b24] Seisenberger S. *et al.* The dynamics of genome-wide DNA methylation reprogramming in mouse primordial germ cells. Mol Cell 48, 849–862 (2012). 23219530 10.1016/j.molcel.2012.11.001 PMC3533687

[b25] Tada M., Tada T., Lefebvre L., Barton S. C. & Surani M. A. Embryonic germ cells induce epigenetic reprogramming of somatic nucleus in hybrid cells. EMBO J. 16, 6510–6520 (1997). 9351832 10.1093/emboj/16.21.6510 PMC1170256

[b26] Piccolo F. M. *et al.* Different roles for Tet1 and Tet2 proteins in reprogramming-mediated erasure of imprints induced by EGC fusion. Mol Cell 49, 1023–1033 (2013). 23453809 10.1016/j.molcel.2013.01.032 PMC3613797

[b27] Dann C. T. *et al.* Spermatogonial stem cell self-renewal requires OCT4, a factor downregulated during retinoic acid-induced differentiation. Stem Cells 26, 2928–2937 (2008). 18719224 10.1634/stemcells.2008-0134

[b28] Shirakawa T. *et al.* An epigenetic switch is crucial for spermatogonia to exit the undifferentiated state toward a Kit-positive identity. Development 140, 3565–3576 (2013). 23903187 10.1242/dev.094045

[b29] Kimura T. *et al.* Pluripotent stem cells derived from mouse primordial germ cells by small molecule compounds. Stem Cells 33, 45–55 (2015). 25186651 10.1002/stem.1838

[b30] Fukuzawa R. *et al.* High frequency of inactivation of the imprinted H19 gene in “sporadic” hepatoblastoma. Int J Cancer 82, 490–497 (1999). 10404060 10.1002/(sici)1097-0215(19990812)82:4<490::aid-ijc4>3.0.co;2-i

[b31] Aiden A. P. *et al.* Wilms tumor chromatin profiles highlight stem cell properties and a renal developmental network. Cell Stem Cell 6, 591–602 (2010). 20569696 10.1016/j.stem.2010.03.016 PMC2897075

[b32] Ogawa O. *et al.* Relaxation of insulin-like growth factor II gene imprinting implicated in Wilms’ tumour. Nature 362, 749–751 (1993). 8097018 10.1038/362749a0

[b33] Holm T. M. *et al.* Global loss of imprinting leads to widespread tumorigenesis in adult mice. Cancer Cell 8, 275–285 (2005). 16226703 10.1016/j.ccr.2005.09.007

[b34] Kagiwada S., Kurimoto K., Hirota T., Yamaji M. & Saitou M. Replication-coupled passive DNA demethylation for the erasure of genome imprints in mice. The EMBO journal 32, 340–353 (2013). 23241950 10.1038/emboj.2012.331 PMC3567490

[b35] Doege C. A. *et al.* Early-stage epigenetic modification during somatic cell reprogramming by Parp1 and Tet2. Nature 488, 652–655 (2012). 22902501 10.1038/nature11333 PMC5176099

[b36] Hu X. *et al.* Tet and TDG mediate DNA demethylation essential for mesenchymal-to-epithelial transition in somatic cell reprogramming. Cell Stem Cell 14, 512–522 (2014). 24529596 10.1016/j.stem.2014.01.001

[b37] Yamaguchi S., Shen L., Liu Y., Sendler D. & Zhang Y. Role of Tet1 in erasure of genomic imprinting. Nature 504, 460–464 (2013). 24291790 10.1038/nature12805 PMC3957231

[b38] Hara K. *et al.* Mouse spermatogenic stem cells continually interconvert between equipotent singly isolated and syncytial states. Cell Stem Cell 14, 658–672 (2014). 24792118 10.1016/j.stem.2014.01.019 PMC4010676

[b39] Bermejo-Alvarez P., Roberts R. M. & Rosenfeld C. S. Effect of glucose concentration during *in vitro* culture of mouse embryos on development to blastocyst, success of embryo transfer, and litter sex ratio. Mol Reprod Dev 79, 329–336 (2012). 22461414 10.1002/mrd.22028 PMC4243512

[b40] Bermejo-Alvarez P., Rizos D., Rath D., Lonergan P. & Gutierrez-Adan A. Sex determines the expression level of one third of the actively expressed genes in bovine blastocysts. Proc Natl Acad Sci USA 107, 3394–3399 (2010). 20133684 10.1073/pnas.0913843107 PMC2840439

[b41] Engel N., Thorvaldsen J. L. & Bartolomei M. S. CTCF binding sites promote transcription initiation and prevent DNA methylation on the maternal allele at the imprinted H19/Igf2 locus. Hum Mol Genet 15, 2945–2954 (2006). 16928784 10.1093/hmg/ddl237

