# Supplementary material for: Tet-mediated imprinting erasure in H19 locus following reprogramming of spermatogonial stem cells to induced pluripotent stem cells
Source: Sci Rep. 2015 Sep 2;5:13691. doi: 10.1038/srep13691 (PMC4556992; doi:10.1038/srep13691)
Supplement: Supplementary Information [file srep13691-s1.pdf]

Tet-mediated imprinting erasure in H19 locus following reprogramming of spermatogonial stem cells to induced pluripotent stem cells

Bermejo-Álvarez P[1,2,4*+], Ramos-Ibeas P[4*], Park K-E[1,2], Powell AP[2], Vansandt L[1], Derek Bickhart[3], Ramirez MA[4], Gutiérrez-Adán A[4], Telugu BP[1,2+]

[1] Department of Animal and Avian Sciences, University of Maryland, MD, USA

[2] Animal Bioscience and Biotechnology Laboratory, USDA-ARS, Beltsville, MD, USA

[3] Animal Improvement Program Laboratory, USDA-ARS, Beltsville, MD, USA

[4] Departamento de Reproducción Animal, INIA, Madrid, Spain

[*] These authors contributed equally to this work.

[+] Corresponding authors.

Supplementary Fig. S1 (A) H&E histological section of an intramuscular teratoma generated by SSCiPSC injection into immunocompromised mouse. The central image shows a 4X overview of the teratoma. Four areas are amplified in the side images showing derivatives from the three germ layers. (B) Representative litters obtained from SSCiPSC (left) or fiPSC (right). In the figure scale bar is 100 μM.

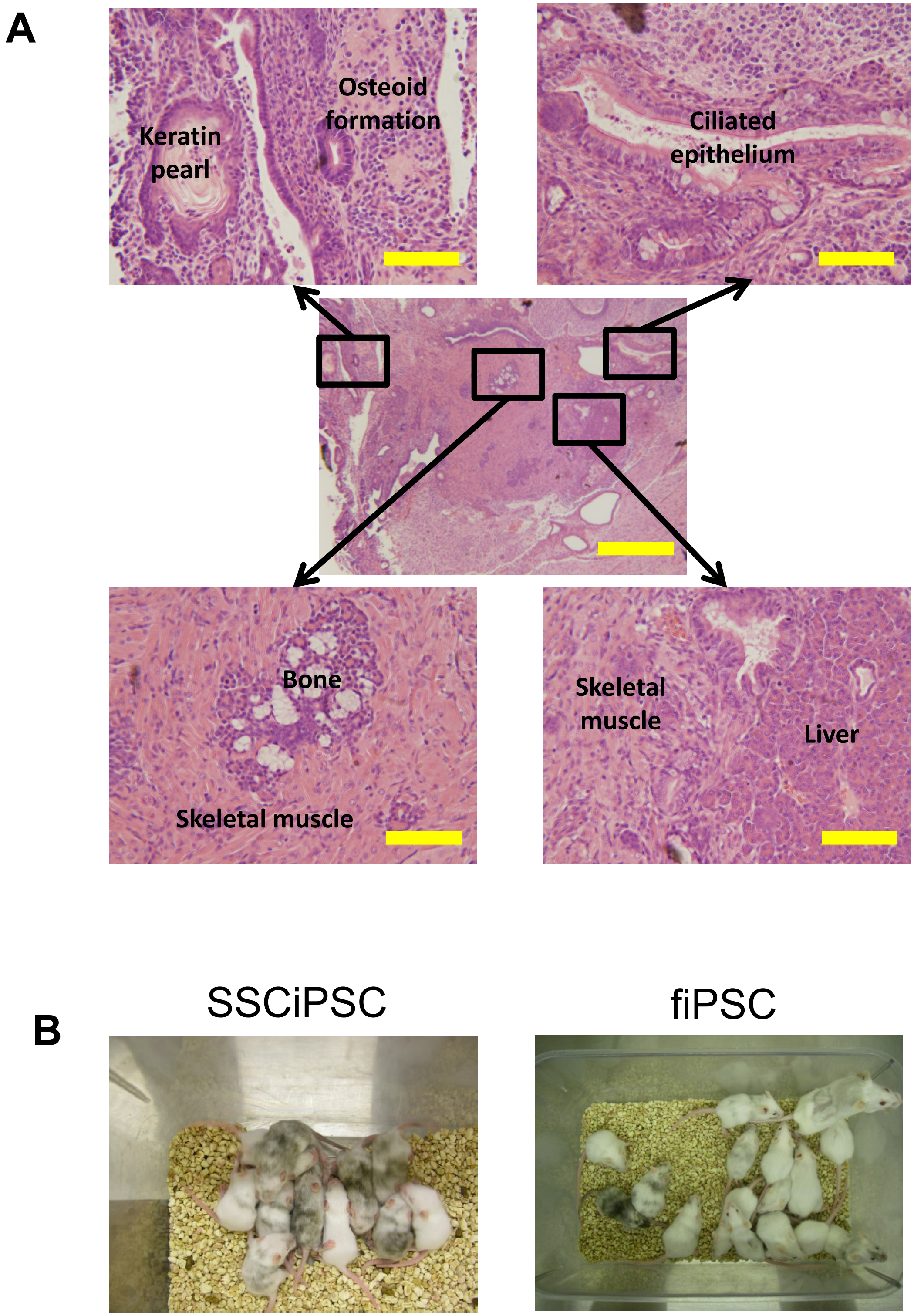

Supplementary Fig. S2: Venn diagram of the differentially expressed genes between SSC, SSCiPSC and fiPSC.

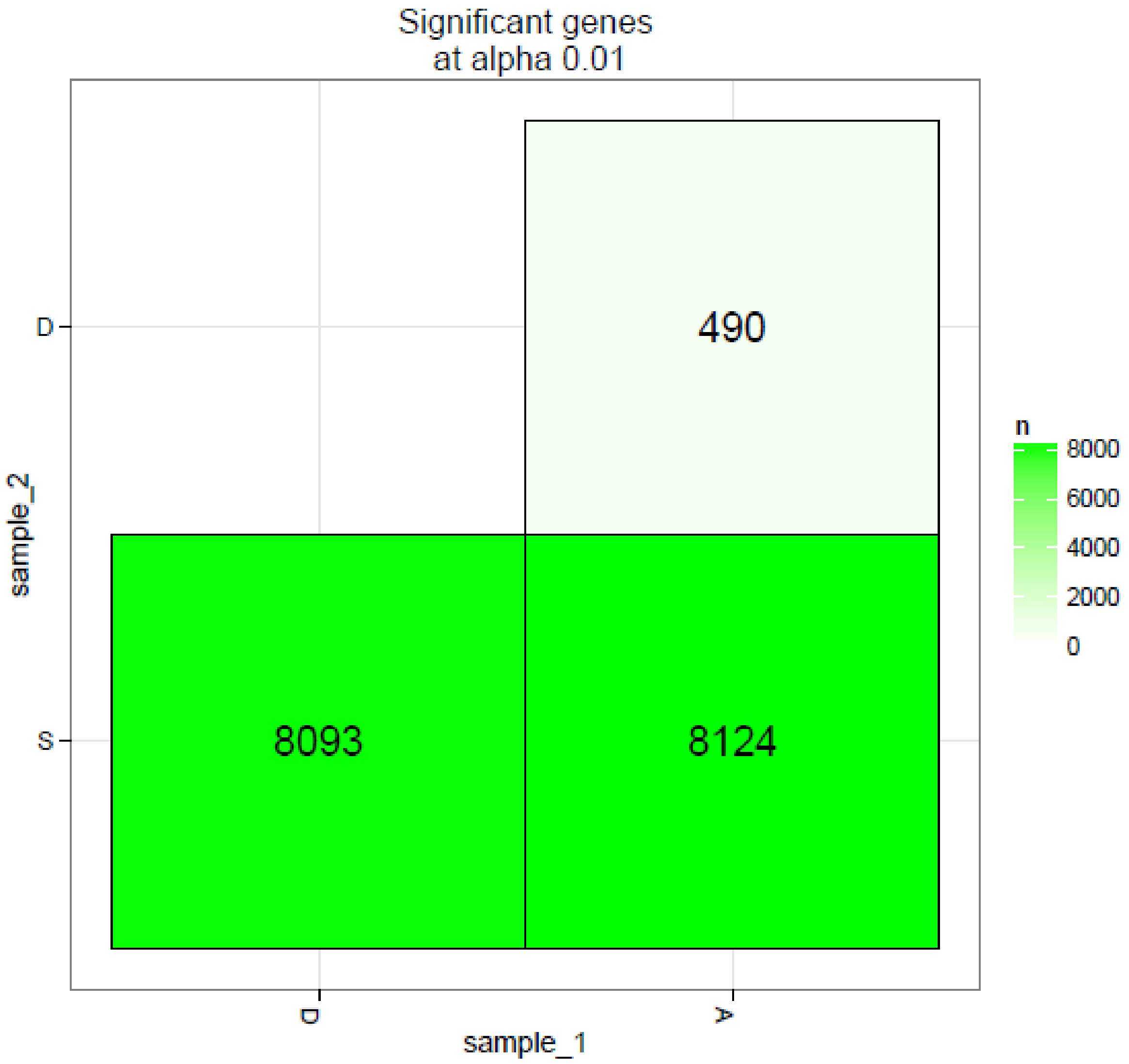

Supplementary Fig. S3. Heat map of differentially expressed pluripotent, methylating, demethylating and imprinted genes between the SSC and SSCiPSC

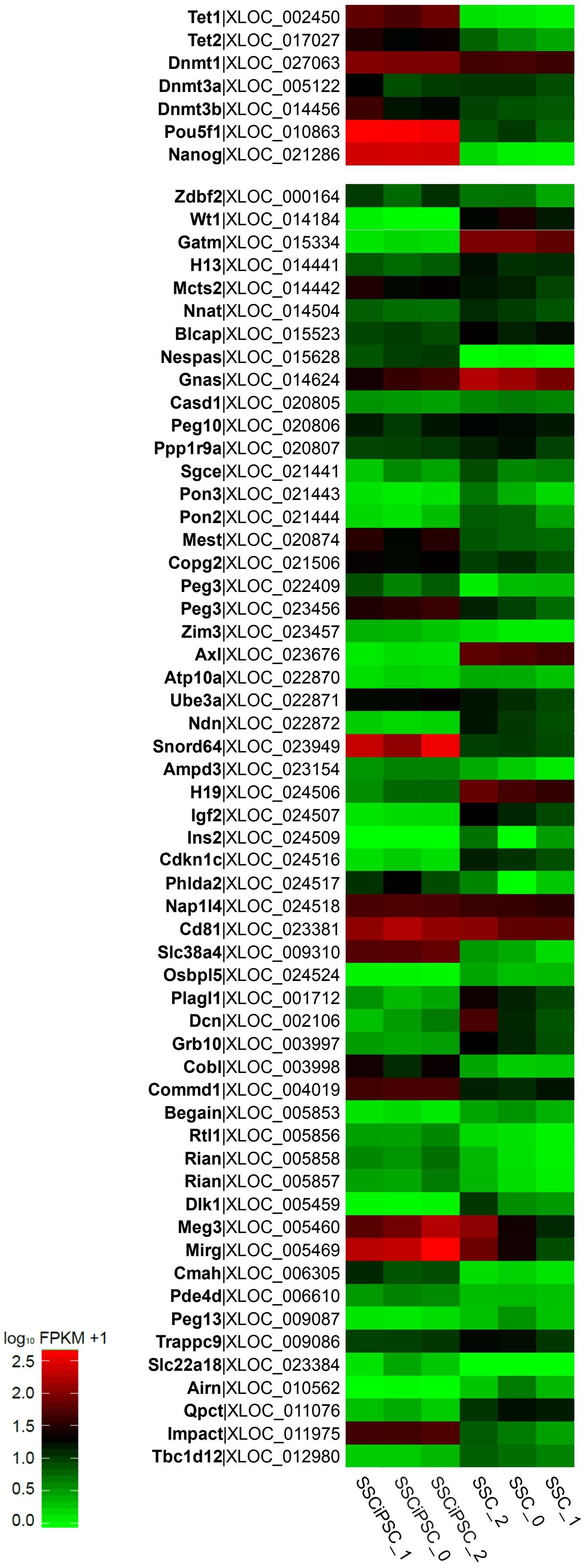

Supplementary. Fig. S4: DAVID gene ontology analysis: Percentage of differentially expressed genes between SSCiPSC and SSC classified in different categories according to: A) Biological Process, B) Cellular Component, C) Molecular Function, and D) KEEG Pathways.

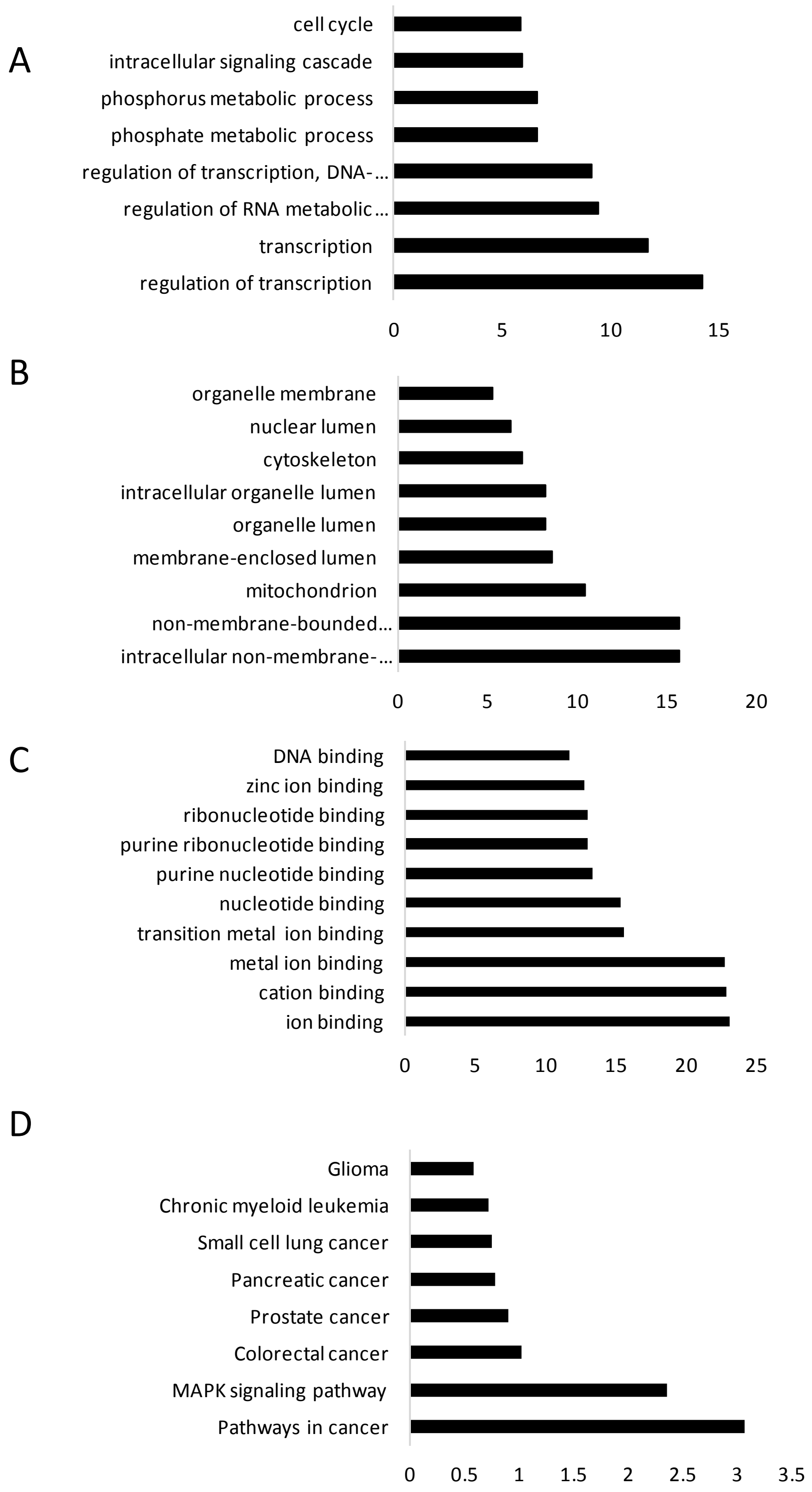

Supplementary Fig. S5. Genomic PCR to determine the percentage of contamination from wild type cells. In the picture, 650 bp band in wt mice (host cells) and 340 bp in the Gt(ROSA)26Sortm1(rtTA*M2)JaeCol1a1tm3(tetO-Pou5f1,-Sox2,-Klf4,-Myc)Jae/J iPSC derivatives.

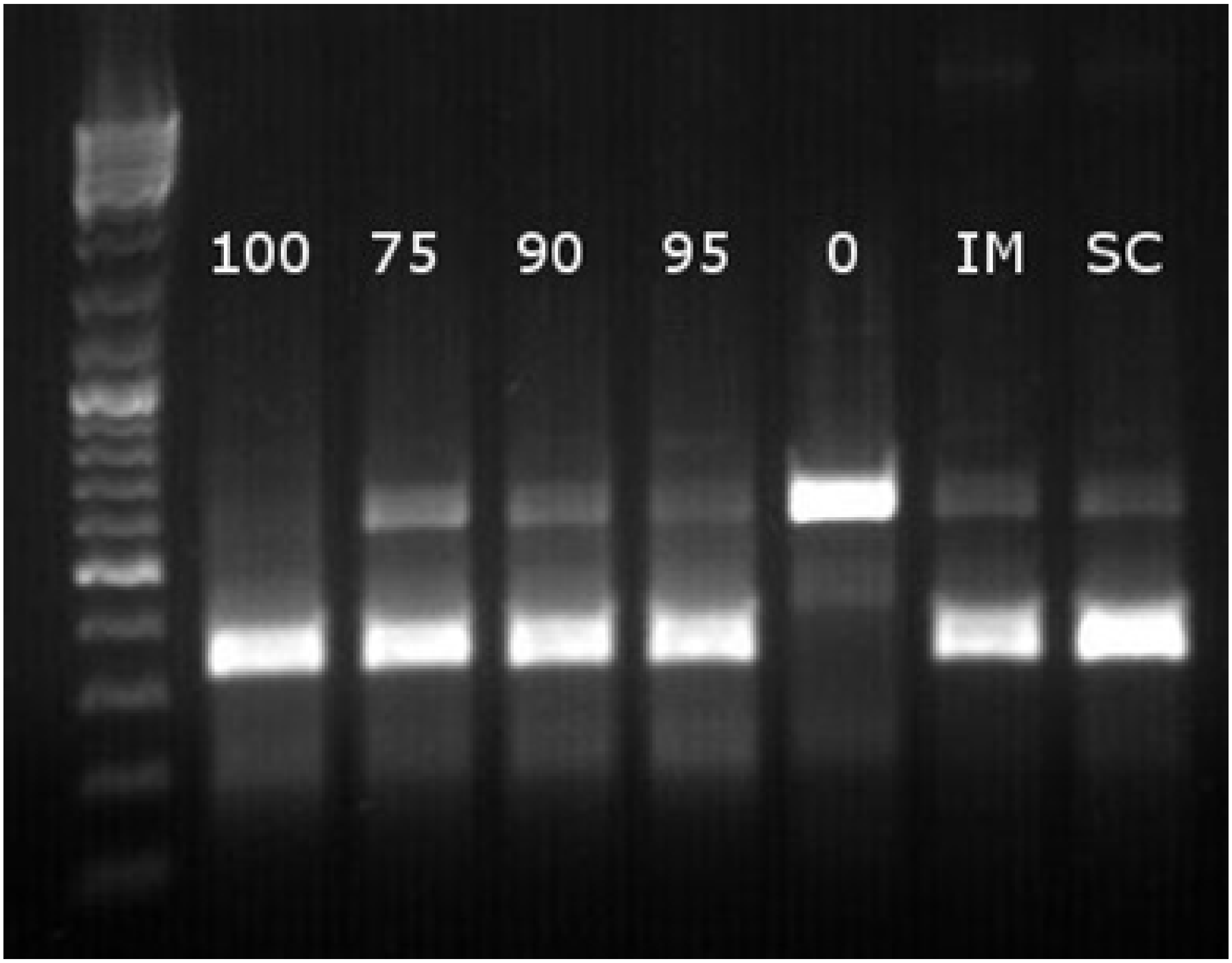

Supplementary Fig. S6. A 422 bp fragment of the 2 kb ICR of *H19-Igf2*[21], containing 2 of the 4 CCTF binding sites was used for methylation analysis

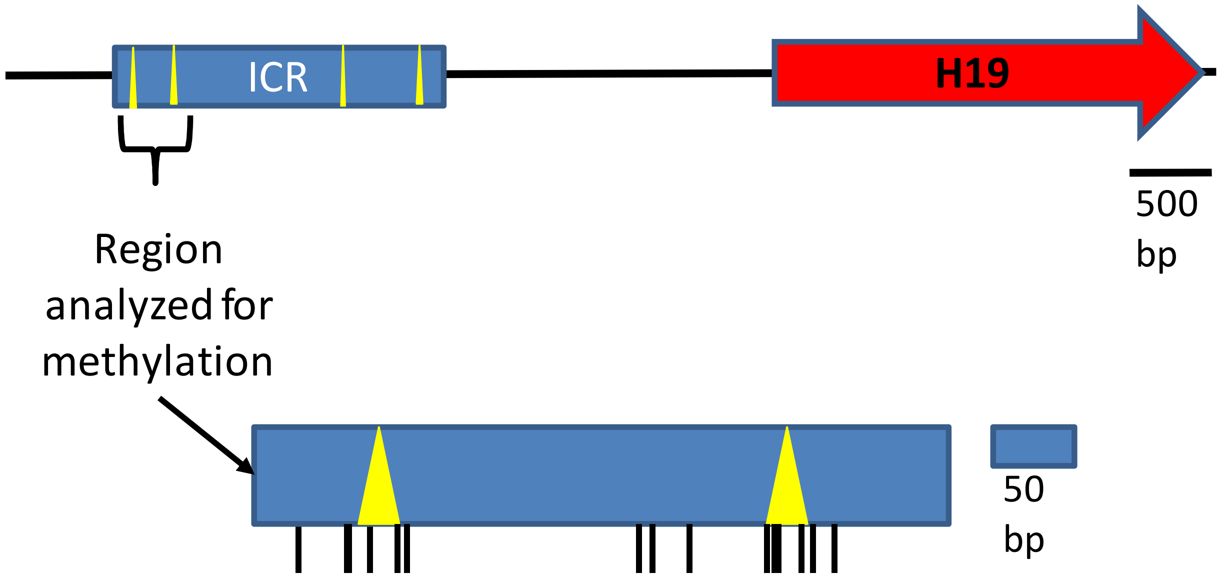

Supplementary Table. S1. Percentage of Skin Chimerism in pups derived from injection of iPSC from SSC and fibroblasts

iPSC line	Embryos transferred	Pups born	Pups weaned	Percentage of skin chimerism		
				0-10 %	20-40 %	>40 %
SSCiPSC #1	30	21	20	6 (30 %)	5 (25 %)	9 (45 %)
SSCiPSC #2	30	15	15	6 (40 %)	4 (27 %)	5 (33 %)
SSCiPSC #3	40	21	21	9 (43 %)	9 (43 %)	3 (14 %)
fiPSC #1	50	28	28	22 (79 %)	5 (18 %)	1 (3 %)
fiPSC #2	60	30	30	22 (73 %)	8 (27 %)	0 (0 %)
fiPSC #3	60	32	32	23 (72 %)	8 (25 %)	1 (3 %)

Supplementary Table S2. Normalized Expression of Genes from Deep Sequencing Analysis

Gene and Location Data					Sample RPKM values								
**LocName**	**GeneID**	**Chr**	**Start**	**End**	**SSC3**	**SSC2**	**SSC1**	**FiPSC3**	**FiPSC2**	**FiPSC1**	**SSCiPSC3**	**SSCiPSC2**	**SSCiPSC1**
XLOC_047926	A2LD1;PCCA	chr12	81026668	81186438	10.7847	9.30876	5.33739	9.42191	8.51891	9.70514	13.1262	10.6482	7.90426
XLOC_049793	A2LD1;PCCA	chr12	81026668	81186438	0.635264	0.380069	0.497021	0.740363	0.74641	1.12217	0.636395	0.127444	0.388808
XLOC_049794	A2LD1;PCCA	chr12	81026668	81186438	1.78817	0.534312	0.0	0.32023	0.416334	0.368965	0.318864	0.176857	0.622809
XLOC_040259	A4GNT	chr1	132064455	132065756	1.26496	2.64886	2.47425	0.113404	0.0	0.0	0.0	0.0	0.0
XLOC_086531	AAED1	chr8	84816107	84842678	3.47365	3.10318	0.808784	14.6658	11.2898	11.636	11.5759	12.2556	8.60944
XLOC_086983	AAED1	chr8	84816107	84842678	25.6257	26.7356	23.5597	63.2721	61.488	60.9356	59.428	46.3057	54.0247
XLOC_088456	AAED1	chr8	84815327	84815866	1.89598	1.13294	0.0	1.0185	1.61721	0.977379	0.168714	0.56182	0.66
XLOC_088457	AAED1	chr8	84816107	84842678	0.0	0.0	8.04438	8.31279	5.47448	8.89277	4.35419	0.985625	4.45096
XLOC_088458	AAED1	chr8	84816107	84842678	2.39115	3.57082	0.0	4.28031	4.98668	3.19518	4.00721	0.705174	4.15597
XLOC_087033	ABCA1	chr8	96371731	96376661	7.63355	6.08563	3.97894	0.0	0.0	0.0	0.0	0.0	0.0
XLOC_088633	ABCA1	chr8	96378994	96380043	2.43383	3.63971	3.8077	0.0	0.0	0.0	0.0	0.0	0.0
XLOC_065768	ABCA12	chr2	103566293	103719321	20.0606	23.4492	18.1639	1.64285	1.41856	1.15157	1.64546	1.28278	1.5757
XLOC_066439	ABCA12	chr2	103720226	103762509	17.3374	17.8663	16.0908	36.4629	29.3667	31.2716	36.1029	41.1688	29.813
XLOC_068160	ABCA12	chr2	103566293	103719321	6.33776	6.27204	7.62983	0.30599	0.229401	0.152115	0.267129	0.048991	0.298518
XLOC_068161	ABCA12	chr2	103566293	103719321	16.7755	8.10142	11.0982	0.115607	0.402195	0.267086	0.232212	0.0	0.225097
XLOC_068162	ABCA12	chr2	103566293	103719321	28.6437	6.84068	4.47202	0.512458	0.0	0.293604	0.0	0.279683	0.496857
XLOC_068163	ABCA12	chr2	103566293	103719321	11.732	2.10477	4.58696	0.0	0.0915007	0.1215	0.528932	0.0	0.102368
XLOC_068164	ABCA12	chr2	103566293	103719321	6.38538	12.3863	4.983	0.285535	0.980368	0.326642	0.832953	0.0	0.553195
XLOC_068165	ABCA12	chr2	103566293	103719321	6.55067	5.41398	7.56834	0.111898	0.244982	0.0974366	0.114322	0.0628294	0.191128
XLOC_068166	ABCA12	chr2	103566293	103719321	1.98452	7.11437	15.504	0.0	0.307537	0.204482	0.176301	0.195799	0.0
XLOC_068167	ABCA12	chr2	103566293	103719321	8.2093	3.7659	5.99527	0.196275	0.0429	0.227599	0.0499239	0.164869	0.047863
XLOC_068168	ABCA12	chr2	103566293	103719321	2.77637	3.82081	6.51715	0.199136	0.043523	0.230908	0.202579	0.0	0.0
XLOC_068169	ABCA12	chr2	103566293	103719321	8.98414	9.83258	25.7109	0.0	0.460473	0.613461	0.261322	0.291966	0.0
XLOC_068170	ABCA12	chr2	103566293	103719321	6.99483	4.29623	6.62672	0.165069	0.0866993	0.191591	0.0673905	0.0741	0.161088
XLOC_068171	ABCA12	chr2	103566293	103719321	11.9827	6.1454	9.3758	0.306948	0.178877	0.237262	0.104057	0.171844	0.049898
XLOC_068172	ABCA12	chr2	103566293	103719321	1.40043	1.25597	10.9476	0.250884	0.0	0.144822	0.125744	0.0	0.366265
XLOC_068173	ABCA12	chr2	103566293	103719321	2.92541	2.62336	12.5773	0.0	0.227626	0.0	0.0	0.145237	0.127492
XLOC_068174	ABCA12	chr2	103566293	103719321	8.06361	5.30188	3.78152	0.144431	0.0	0.0	0.0	0.319685	0.140493

XLOC_068175	ABCA12	chr2	103566293	103719321	6.46802	6.44015	8.42073	0.192976	0.0	0.0	0.190948	0.0	0.187449
XLOC_082988	ABCA7	chr7	45172057	45176141	0.900942	1.48259	0.705027	1.21178	1.76693	1.78078	0.823333	1.58511	0.906431
XLOC_066205	ABCB11	chr2	27188398	27197614	1.46651	0.73127	0.765041	0.0	0.0383365	0.0	0.0	0.0491227	0.0
XLOC_066206	ABCB11	chr2	27211018	27234420	0.739896	1.10657	1.73647	0.0	0.0	0.0	0.0	0.0	0.0
XLOC_066207	ABCB11	chr2	27239614	27244392	1.31625	0.786975	0.0	0.0	0.0	0.0	0.118374	0.0	0.0
XLOC_092190	ABCB7	chrX	81070694	81098154	5.39908	7.34946	9.54315	2.82746	2.46481	3.43028	3.00774	1.61642	1.55574
XLOC_093222	ABCB7	chrX	81098481	81099837	0.0	0.0	0.0	0.108203	0.141844	0.188153	0.10998	0.0	0.158294
XLOC_093223	ABCB7	chrX	81101967	81103402	1.13227	0.338736	0.0	0.0	0.0	0.117706	0.0516253	0.0	0.0
XLOC_072764	ABCB8;FASTK;TMUB1;CDK5;SLC4A2;ACCN3	chr4	114406769	114586533	0.0	0.0	1.45704	0.0	0.0727866	0.0	0.0	0.0	0.0
XLOC_074924	ABCB8;FASTK;TMUB1;CDK5;SLC4A2;ACCN3	chr4	114406769	114586533	0.0	0.290906	0.760824	0.0	0.0	0.0	0.0	0.0	0.0
XLOC_074925	ABCB8;FASTK;TMUB1;CDK5;SLC4A2;ACCN3	chr4	114406769	114586533	0.551038	0.164854	0.862332	0.0494049	0.0	0.0	0.0	0.0	0.0
XLOC_074927	ABCB8;FASTK;TMUB1;CDK5;SLC4A2;ACCN3	chr4	114406769	114586533	0.895674	0.535736	0.0	0.0	0.0700123	0.0	0.0	0.0	0.0
XLOC_074928	ABCB8;FASTK;TMUB1;CDK5;SLC4A2;ACCN3	chr4	114406769	114586533	0.0	0.188053	0.0	0.0	0.0492442	0.0	0.0	0.0	0.0
XLOC_074929	ABCB8;FASTK;TMUB1;CDK5;SLC4A2;ACCN3	chr4	114406769	114586533	1.49905	0.0	0.0	0.0	0.0	0.0	0.0	0.0	0.0
XLOC_074930	ABCB8;FASTK;TMUB1;CDK5;SLC4A2;ACCN3	chr4	114406769	114586533	0.0	0.3006	2.35853	0.0	0.0	0.0	0.0	0.0	0.0
XLOC_074931	ABCB8;FASTK;TMUB1;CDK5;SLC4A2;ACCN3	chr4	114406769	114586533	2.87876	0.0	0.0	0.0	0.0	0.0	0.0	0.0	0.0

XLOC_074932	ABCB8;FASTK;TMUB1;CDK5;SLC4A2;ACCN3	chr4	114406769	114586533	0.0	0.340334	0.445061	0.0	0.0	0.0	0.0	0.0	0.0
XLOC_074933	ABCB8;FASTK;TMUB1;CDK5;SLC4A2;ACCN3	chr4	114406769	114586533	1.20893	0.180828	0.472942	0.0	0.0	0.0	0.0	0.0	0.0
XLOC_074934	ABCB8;FASTK;TMUB1;CDK5;SLC4A2;ACCN3	chr4	114406769	114586533	0.0	0.392773	0.0	0.0	0.0	0.0	0.0	0.0	0.0
XLOC_074935	ABCB8;FASTK;TMUB1;CDK5;SLC4A2;ACCN3	chr4	114406769	114586533	0.0	0.350202	0.0	0.0	0.0	0.0	0.0	0.0	0.0
XLOC_074936	ABCB8;FASTK;TMUB1;CDK5;SLC4A2;ACCN3	chr4	114406769	114586533	0.966566	0.289187	0.378177	0.0	0.0	0.0	0.0	0.0	0.0
XLOC_074937	ABCB8;FASTK;TMUB1;CDK5;SLC4A2;ACCN3	chr4	114406769	114586533	1.64469	0.245955	0.0	0.0	0.0	0.0853508	0.0	0.082298	0.0
XLOC_074939	ABCB8;FASTK;TMUB1;CDK5;SLC4A2;ACCN3	chr4	114406769	114586533	3.3217	0.0	2.59606	0.0	0.0	0.0	0.0	0.0	0.0
XLOC_074940	ABCB8;FASTK;TMUB1;CDK5;SLC4A2;ACCN3	chr4	114406769	114586533	1.72924	0.0	0.676308	0.0	0.0676018	0.0	0.0	0.0	0.0
XLOC_074941	ABCB8;FASTK;TMUB1;CDK5;SLC4A2;ACCN3	chr4	114406769	114586533	1.40752	0.280748	0.367143	0.0	0.0	0.0	0.0	0.0	0.0
XLOC_074943	ABCB8;FASTK;TMUB1;CDK5;SLC4A2;ACCN3	chr4	114406769	114586533	1.79259	0.535634	2.80159	0.0	0.0	0.0	0.0	0.0	0.0
XLOC_074944	ABCB8;FASTK;TMUB1;CDK5;SLC4A2;ACCN3	chr4	114406769	114586533	1.60633	0.0	1.25656	0.0	0.0	0.0833702	0.0	0.0	0.0

XLOC_074945	ABCB8;FASTK;TMUB1;CDK5;SLC4A2;ACCN3	chr4	114406769	114586533	2.35786	0.0	0.0	0.0	0.0	0.0	0.0	0.0	0.0
XLOC_055432	ABCC8;B7H6;NUCB2;USH1C;KCNJ11	chr15	35370731	35931678	17.2419	22.7759	22.3538	15.585	15.5413	15.205	16.5536	12.4311	17.5001
XLOC_056315	ABCC8;B7H6;NUCB2;USH1C;KCNJ11	chr15	35370731	35931678	0.495047	1.03681	1.93705	1.28728	1.01115	0.979525	1.15785	0.947793	0.801641
XLOC_076837	ABCD2	chr5	41624996	41708161	6.91982	8.87376	4.80938	8.26916	6.92384	9.54654	7.23094	7.75896	7.82058
XLOC_069898	ABCD3;F3	chr3	49096187	49217459	0.0	0.0	8.22005	2.86032	3.12409	3.16575	1.68756	4.84618	9.03563
XLOC_070989	ABCD3;F3	chr3	49096187	49217459	2.48398	0.247643	0.0	0.964792	1.03612	1.20308	1.42761	1.07715	1.80846
XLOC_070990	ABCD3;F3	chr3	49096187	49217459	0.460511	0.826698	0.360366	0.991017	0.43348	1.1976	1.34644	1.06473	1.45033
XLOC_070991	ABCD3;F3	chr3	49096187	49217459	0.0	0.257665	0.673894	1.08105	0.606257	1.87732	1.25012	0.775603	1.12889
XLOC_070992	ABCD3;F3	chr3	49096187	49217459	0.0	0.45464	2.378	0.544969	0.709837	2.2007	1.63543	1.05653	1.5907
XLOC_070993	ABCD3;F3	chr3	49096187	49217459	0.0	0.324481	1.69726	1.06964	0.677565	1.91158	1.66615	2.16503	1.61021
XLOC_070994	ABCD3;F3	chr3	49096187	49217459	0.0	0.210551	0.0	0.694091	0.716378	1.24287	0.320113	1.05819	1.0459
XLOC_070995	ABCD3;F3	chr3	49096187	49217459	0.0	1.7244	0.0	0.645944	0.448971	1.0438	1.42347	0.716226	2.26298
XLOC_070996	ABCD3;F3	chr3	49096187	49217459	1.22894	0.857994	1.28232	0.734668	0.578638	1.32142	1.72336	0.824103	1.93556
XLOC_070997	ABCD3;F3	chr3	49096187	49217459	1.69226	0.379753	0.331077	0.60698	0.929489	1.10048	0.967048	1.23389	1.11048
XLOC_070998	ABCD3;F3	chr3	49096187	49217459	0.0	0.3466	1.81302	0.675169	0.499364	0.722579	0.897797	0.523323	0.658525
XLOC_070999	ABCD3;F3	chr3	49096187	49217459	3.3525	1.50279	1.31005	2.10157	0.520838	1.55752	0.748799	0.332025	1.60594
XLOC_071000	ABCD3;F3	chr3	49096187	49217459	0.0	0.748644	0.0	0.448676	0.193477	1.03011	1.1033	0.737495	2.39485
XLOC_071001	ABCD3;F3	chr3	49096187	49217459	1.68799	0.504431	0.0	0.604647	0.786654	0.348513	0.150776	0.334302	1.47011
XLOC_071002	ABCD3;F3	chr3	49096187	49217459	1.75773	0.0	0.0	0.157396	0.40935	1.81378	0.156806	0.0	0.459138
XLOC_071003	ABCD3;F3	chr3	49096187	49217459	0.610072	0.547514	0.477328	0.711028	0.238987	0.44382	0.389105	0.367284	0.426735
XLOC_041699	ABCD4	chr10	86021854	86049228	6.4146	7.62504	6.68519	11.1346	13.4571	11.0302	13.8007	11.7841	14.6673
XLOC_072765	ABCF2	chr4	114599872	114610630	0.792604	0.237067	0.0	0.0	0.062007	0.0	0.0	0.0	0.0
XLOC_038091	ABCG1	chr1	143999998	144004139	3.08549	0.149022	0.688027	0.0	0.0	0.0367324	0.0	0.0	0.0
XLOC_038575	ABCG1	chr1	143999998	144004139	18.5047	10.2837	11.8889	0.310758	0.203932	0.172362	0.356411	0.130686	0.22741
XLOC_080823	ABCG2	chr6	37966667	37967269	0.0	1.9452	0.0	3.64323	3.28771	2.35265	3.63773	3.0638	3.26024
XLOC_080824	ABCG2	chr6	37967365	37967620	0.0	4.33926	0.0	4.57446	4.91213	6.59785	8.38256	8.86964	3.12717
XLOC_080825	ABCG2	chr6	37968089	37970094	0.782926	1.28845	0.919069	1.40415	1.65911	1.62956	1.50417	1.92987	1.26744
XLOC_080826	ABCG2	chr6	37970642	37971244	0.0	1.4589	1.27179	1.45729	2.40256	2.01656	1.74611	1.29002	1.84274

XLOC_080827	ABCG2	chr6	37972212	37972657	2.51587	1.50248	1.96455	1.12558	2.32964	2.5841	4.64925	3.94657	1.96618
XLOC_080828	ABCG2	chr6	37983409	37983942	0.0	0.575506	0.0	1.2072	2.23999	1.38994	0.685264	1.52157	1.67618
XLOC_080829	ABCG2	chr6	37985261	37986075	0.0	0.658837	2.58462	1.4808	1.28962	0.570734	0.895241	1.31854	1.25002
XLOC_080830	ABCG2	chr6	37990445	37991174	0.0	0.756715	0.0	0.226769	0.98633	0.261985	0.341769	0.251939	0.110392
XLOC_044695	ABCG5;ABCG8;DYNC2LI1	chr11	25986732	26211006	0.0	0.492945	0.0	0.59088	0.256316	0.170323	1.47441	0.0	0.287356
XLOC_045692	ABCG5;ABCG8;DYNC2LI1	chr11	25986732	26211006	0.0	0.0	0.0	0.0	0.202325	0.0895021	0.234672	0.0	0.0
XLOC_050822	ABHD12;ANKRD16;GDI2	chr13	43197859	43304388	13.2533	12.6291	14.9483	7.20373	7.55183	6.8968	5.38086	7.8678	6.96358
XLOC_051721	ABHD12;ANKRD16;GDI2	chr13	43197859	43304388	1.40036	5.02315	2.18949	1.37986	0.981174	1.15857	0.754464	1.2524	0.976707
XLOC_051722	ABHD12;ANKRD16;GDI2	chr13	43197859	43304388	2.83446	3.10808	5.91183	2.37092	1.10737	1.66579	0.941093	1.13295	1.98023
XLOC_051723	ABHD12;ANKRD16;GDI2	chr13	43197859	43304388	5.12421	1.5276	7.98821	5.50201	2.33033	3.11718	3.89268	1.46601	1.32513
XLOC_051724	ABHD12;ANKRD16;GDI2	chr13	43197859	43304388	0.0	2.71643	1.18403	3.25614	2.8276	3.28735	2.44315	2.70563	2.2441
XLOC_041869	ABHD4	chr10	22055034	22058646	0.0	5.33558	1.55038	9.06026	8.91858	10.0196	5.99422	7.63616	9.84054
XLOC_042458	ABHD4	chr10	22046521	22046994	4.58527	2.05417	5.37189	3.89846	2.65828	4.24434	4.8613	4.28207	4.78165
XLOC_042459	ABHD4	chr10	22055034	22058646	0.0	0.0	0.0	1.98232	2.08934	2.28644	1.69792	1.56758	1.51519
XLOC_066411	ABI2	chr2	91982329	91991272	11.2086	7.54482	9.20875	1.55765	1.58133	1.51481	1.07332	0.900281	0.931199
XLOC_066412	ABI2	chr2	91991581	92024506	4.44556	5.48613	7.39173	0.548045	0.609788	1.2132	0.101366	0.223181	0.0971941
XLOC_068116	ABI2	chr2	91991581	92024506	2.22373	0.332454	2.60843	0.0996293	0.0867605	0.575962	0.0	0.0	0.0
XLOC_066410	ABI2;CYP20A1	chr2	91932839	91980999	25.1975	28.8606	26.6192	26.4417	30.7629	28.8589	25.2974	32.9931	25.7331
XLOC_068115	ABI2;CYP20A1	chr2	91932839	91980999	0.0	0.0	0.0	0.459914	0.398837	1.76705	0.152851	0.338952	0.0
XLOC_044449	ABL1	chr11	101008727	101026759	15.4635	13.7204	15.3602	9.14738	9.25489	9.19094	10.6585	9.26923	8.39228
XLOC_044450	ABL1	chr11	101027012	101064196	20.882	9.4118	19.0409	6.93666	8.48182	8.29202	6.2952	7.24076	6.20081
XLOC_044451	ABL1	chr11	101070097	101077797	6.11216	3.10251	3.10944	0.411093	0.39554	0.445482	0.111999	0.215443	0.427995
XLOC_044452	ABL1	chr11	101082578	101095436	15.9277	13.0157	10.6079	18.4002	20.482	18.5982	17.4345	19.4226	20.0026
XLOC_044453	ABL1	chr11	101096320	101126419	20.0863	26.6841	16.4458	20.7177	22.8863	23.5365	24.3175	26.3358	19.1855
XLOC_045090	ABL1	chr11	101082578	101095436	3.2651	7.45645	4.9285	16.3645	15.1151	16.4224	18.3782	20.8528	19.604
XLOC_045091	ABL1	chr11	101096320	101126419	19.569	26.1693	22.9985	11.0929	12.934	12.4958	12.2546	11.475	11.3042
XLOC_047277	ABL1	chr11	101008727	101026759	1.46267	3.49756	4.57354	1.31015	0.682879	1.05845	0.393567	0.726185	0.382475

XLOC_047278	ABL1	chr11	101008727	101026759	1.24596	1.11749	0.974207	0.893029	1.35967	1.28977	0.673182	0.744276	0.978192
XLOC_047279	ABL1	chr11	101008727	101026759	2.0893	2.29154	1.0897	1.31106	0.545256	0.940409	1.01365	0.13961	0.730483
XLOC_047280	ABL1	chr11	101008727	101026759	1.28178	2.49233	3.00854	1.20656	0.803189	0.932298	0.233443	0.707098	0.672359
XLOC_047281	ABL1	chr11	101027012	101064196	0.607623	0.181826	1.18891	0.463185	1.00219	0.727594	0.361923	0.520196	0.452091
XLOC_047282	ABL1	chr11	101027012	101064196	1.31625	0.0	0.0	0.353754	0.0	0.408584	0.118374	0.654678	0.0
XLOC_047283	ABL1	chr11	101027012	101064196	3.93444	0.784124	0.0	0.352473	0.510863	1.08564	0.235911	0.391412	0.457506
XLOC_047284	ABL1	chr11	101027012	101064196	0.0	0.0	5.95758	1.37376	1.71707	0.769299	0.0	0.714894	0.0
XLOC_064620	ACACA	chr19	13765845	13766539	0.0	0.0	0.0	1.57003	0.524993	0.976272	0.60586	0.134053	0.235118
XLOC_064621	ACACA	chr19	13768298	13769396	0.0	0.0	0.0	1.65436	0.541551	0.878253	0.908192	0.462062	0.739191
XLOC_059878	ACACB	chr17	66111533	66120161	18.4721	22.7799	13.6886	14.4145	14.96	14.5936	12.4098	18.2176	13.6048
XLOC_059879	ACACB	chr17	66123529	66154514	10.6335	19.3228	11.0171	28.3831	23.3388	24.8871	32.0401	35.0065	26.4145
XLOC_061835	ACACB	chr17	66123529	66154514	1.041	1.24578	2.03642	1.44672	2.6526	1.46135	2.37521	2.45736	1.91205
XLOC_061836	ACACB	chr17	66155186	66156652	0.552722	1.32286	0.864966	1.13979	1.08311	0.632095	0.907466	0.943479	1.45013
XLOC_061837	ACACB	chr17	66156783	66157568	2.30525	0.344627	1.80262	0.516385	0.62938	0.358155	0.519856	0.22977	1.10639
XLOC_040520	ACAD11	chr1	138063479	138064216	3.74432	1.49256	0.0	0.111821	0.0	0.0	0.0	0.0	0.0
XLOC_038567	ACAD11;DNAJC13	chr1	138080854	138175100	9.27927	0.666377	0.580964	0.0	0.0	0.0	0.03397	0.0	0.160423
XLOC_037904	ACAP2	chr1	72813363	72852498	52.8474	28.1139	24.3286	552.406	572.486	495.877	654.093	512.225	686.354
XLOC_038406	ACAP2	chr1	72862155	72875244	1.09331	2.07192	3.13736	0.326811	0.228875	0.379329	0.366938	0.110035	0.223253
XLOC_039658	ACAP2	chr1	72805534	72806240	0.0	0.0	0.0	0.945063	1.64361	0.955086	1.5416	0.524669	0.919993
XLOC_039659	ACAP2	chr1	72857933	72862026	5.69678	2.25489	3.16462	0.626336	0.563446	0.517029	0.489454	0.259509	0.305763
XLOC_063924	ACCN1	chr19	16383749	16390110	20.3518	16.8105	14.5869	6.72509	8.38983	6.90567	7.25123	6.50993	6.89448
XLOC_063925	ACCN1	chr19	16435343	16610029	5.20427	13.9043	9.63241	1.84108	0.603292	0.295299	1.96868	0.476764	1.34607
XLOC_063926	ACCN1	chr19	16955824	17222060	23.0687	21.4319	21.0489	33.9242	34.7868	31.2409	31.2644	37.1027	43.045
XLOC_063927	ACCN1	chr19	17394029	17401521	18.5367	13.1776	18.6583	12.484	13.9088	14.6197	18.4965	14.9659	14.2563
XLOC_064196	ACCN1	chr19	16614887	16641674	15.4565	18.287	19.7892	5.65404	6.67791	6.31075	7.87575	6.51608	8.1543
XLOC_064197	ACCN1	chr19	16643627	16780977	35.2661	33.0575	29.3506	9.83143	11.6936	10.5377	12.9661	10.6039	14.4107
XLOC_064198	ACCN1	chr19	17325934	17356676	2.32617	1.67088	3.27763	3.54702	2.73163	3.23255	2.79312	4.22503	3.30043
XLOC_064655	ACCN1	chr19	16391191	16391976	0.0	1.37851	3.60525	0.206554	0.359645	0.358155	0.415885	0.344656	0.100581
XLOC_064656	ACCN1	chr19	16435343	16610029	0.0	4.43311	0.0	0.0	0.0	0.0	0.0	0.0	0.0
XLOC_064657	ACCN1	chr19	16435343	16610029	2.66157	1.19348	1.04043	1.19218	0.414643	0.137687	0.358948	0.132354	0.232104
XLOC_064658	ACCN1	chr19	16610462	16610874	0.0	2.54503	4.43681	0.254222	0.437486	0.582673	0.0	0.27756	0.739491
XLOC_064659	ACCN1	chr19	16612142	16612601	4.79816	0.716442	1.87357	1.28813	1.29705	1.23293	1.05777	0.942146	2.29248

XLOC_064660	ACCN1	chr19	16612680	16614169	2.1727	2.27504	2.12508	1.21751	1.66059	1.24243	1.73434	1.30912	2.2327
XLOC_064661	ACCN1	chr19	16614382	16614805	5.44846	4.87984	0.0	1.94975	3.14815	0.558935	1.67193	3.19722	3.30942
XLOC_064662	ACCN1	chr19	16614887	16641674	0.0	1.59664	0.0	0.717679	0.206079	0.823158	0.234643	0.261668	0.928242
XLOC_064663	ACCN1	chr19	16643627	16780977	4.17771	7.4955	4.90079	1.96551	2.85398	2.59802	4.43698	2.60626	2.27938
XLOC_064664	ACCN1	chr19	16955824	17222060	4.91028	6.75436	8.44853	0.0	0.0	0.0	0.0888617	0.0	0.0857472
XLOC_064665	ACCN1	chr19	16955824	17222060	15.8977	10.8526	8.86902	0.203254	0.0	0.0	0.0	0.0	0.0
XLOC_064666	ACCN1	chr19	16955824	17222060	0.0	12.3827	11.774	0.0	0.0	0.0	0.0	0.0	0.0
XLOC_064667	ACCN1	chr19	16955824	17222060	4.45845	7.98925	3.48216	0.0	0.0	0.0	0.0	0.0	0.0
XLOC_064668	ACCN1	chr19	16955824	17222060	8.62042	3.85566	6.72111	0.0	0.0	0.0	0.0	0.0	0.0
XLOC_064669	ACCN1	chr19	16955824	17222060	0.0	1.4521	1.89869	0.0	0.0	0.0	0.0	0.0	0.0
XLOC_064670	ACCN1	chr19	16955824	17222060	0.0	19.1899	7.16637	0.0	0.0	0.0	0.0	0.0	0.0
XLOC_064671	ACCN1	chr19	16955824	17222060	5.7653	0.0	4.4927	0.0	0.0	0.583142	0.0	0.0	0.0
XLOC_064672	ACCN1	chr19	16955824	17222060	0.0	2.63248	3.44165	0.0	0.0	0.0	0.0	0.0	0.0
XLOC_064673	ACCN1	chr19	16955824	17222060	4.41483	7.89743	6.8833	0.0	0.0	0.0	0.0	0.0	0.0
XLOC_064674	ACCN1	chr19	16955824	17222060	3.20107	3.11281	3.13133	1.21993	1.82131	2.12356	1.17121	1.6906	1.40048
XLOC_064675	ACCN1	chr19	16955824	17222060	1.7369	2.07577	1.35715	0.777548	1.61801	1.61301	2.16977	1.0312	1.05854
XLOC_064676	ACCN1	chr19	16955824	17222060	1.49928	0.896106	2.34355	0.537074	0.583051	0.154933	0.134363	0.148784	0.130646
XLOC_064677	ACCN1	chr19	17222216	17224239	2.32611	2.90006	2.12381	5.97959	4.65559	5.7696	5.00135	6.98223	6.6493
XLOC_063923	ACCN1;CCL8;CCL2;CCL11	chr19	16133100	16383694	13.5242	19.3541	21.1752	6.87648	7.25838	7.1142	6.36024	6.63886	6.20579
XLOC_064645	ACCN1;CCL8;CCL2;CCL11	chr19	16133100	16383694	5.46458	3.59666	1.28275	1.46984	1.15656	1.81805	1.74472	0.82311	1.52931
XLOC_064646	ACCN1;CCL8;CCL2;CCL11	chr19	16133100	16383694	0.0	3.57552	0.0	1.07453	2.71473	3.6375	1.00106	1.13536	2.58113
XLOC_064647	ACCN1;CCL8;CCL2;CCL11	chr19	16133100	16383694	4.57405	2.39334	0.894195	0.512307	0.981278	0.829134	0.206333	0.569948	1.09768
XLOC_064648	ACCN1;CCL8;CCL2;CCL11	chr19	16133100	16383694	0.0	3.21699	4.20626	0.72301	0.207582	1.10558	0.236307	0.263554	1.6364
XLOC_064649	ACCN1;CCL8;CCL2;CCL11	chr19	16133100	16383694	14.3945	3.58221	5.62071	1.50281	0.74117	0.986344	0.634665	0.235536	0.625222

XLOC_064650	ACCN1;CCL8;CCL2;CCL11	chr19	16133100	16383694	5.28604	4.21212	8.26169	1.89334	1.231	2.54541	3.14352	0.348649	2.7615
XLOC_064651	ACCN1;CCL8;CCL2;CCL11	chr19	16133100	16383694	8.2976	3.30283	6.4777	0.494873	2.98223	1.98569	1.45392	1.0815	2.63963
XLOC_064652	ACCN1;CCL8;CCL2;CCL11	chr19	16133100	16383694	9.16028	8.19377	10.7123	1.63853	1.39311	4.65386	1.1688	0.439028	2.37159
XLOC_064653	ACCN1;CCL8;CCL2;CCL11	chr19	16133100	16383694	0.0	9.80441	14.649	2.10092	0.0	4.77098	1.59567	2.69873	2.83714
XLOC_064654	ACCN1;CCL8;CCL2;CCL11	chr19	16133100	16383694	33.8142	25.6226	31.1554	54.29	52.3366	50.7941	51.9659	53.5896	53.3219
XLOC_064200	ACCN1;CDK5R1;MYO1D;TMEM98;SPACA3	chr19	17515168	17837564	8.2533	6.82659	8.90565	0.0904898	0.0813988	0.0990724	0.0826958	0.0517373	0.0280889
XLOC_082806	ACER1	chr7	19344894	19356834	21.0228	16.883	13.6416	20.7803	15.7198	17.5455	18.9555	21.4284	15.5624
XLOC_067591	ACMSD	chr2	62692842	62695985	1.93795	1.2324	0.568825	1.28184	1.44671	1.56448	1.15565	1.53828	1.27255
XLOC_086304	ACO1	chr8	11497524	11550862	8.06857	7.58518	4.30207	15.6918	18.0989	15.8694	16.6255	12.5705	15.2422
XLOC_086779	ACO1	chr8	11497524	11550862	2.71503	7.83533	2.54989	27.5656	26.947	26.9749	19.4825	15.9907	18.5124
XLOC_076984	ACO2;XRCC6;CSDC2;PMM1;POLR3H	chr5	113089191	113228207	12.9281	18.2478	19.0176	3.52822	2.54438	3.34235	2.79889	5.4523	2.7913
XLOC_083095	ACOT12	chr7	83631995	83641848	7.97285	4.06835	4.26896	3.47849	5.02155	3.99508	2.74352	2.95412	3.67424
XLOC_085235	ACOT12	chr7	83624941	83625530	5.02875	5.51023	13.1005	1.05079	0.520838	0.519172	0.449279	0.0	0.291989
XLOC_064053	ACOX1	chr19	56287755	56320088	0.0	0.0	0.0	0.0	0.0	0.0360198	0.0	0.0696741	0.0
XLOC_055853	ACP2;DDB2	chr15	78294218	78313098	11.4351	3.76096	2.68258	0.0	0.0	0.118448	0.103166	0.0	0.0
XLOC_069849	ACP6	chr3	22076642	22211941	11.0359	11.5519	9.24543	32.001	32.3261	33.8706	35.0618	34.2191	36.6767
XLOC_070715	ACP6	chr3	22074318	22076186	0.845635	0.379531	0.99265	2.04736	1.79154	1.3198	1.04381	1.14813	1.55377
XLOC_070716	ACP6	chr3	22076642	22211941	2.96578	1.77414	2.32004	2.65844	3.71367	2.3097	2.62817	2.45101	1.81425
XLOC_070717	ACP6	chr3	22076642	22211941	0.0	1.30631	2.27757	1.30494	1.81376	1.05424	1.69878	2.31464	1.26982
XLOC_070718	ACP6	chr3	22076642	22211941	2.79016	0.0	2.18106	1.49956	0.977431	1.8755	2.25489	1.24769	1.21621
XLOC_065042	ACSF2	chr19	36895988	36896297	0.0	2.8376	3.70974	2.55398	3.97612	1.44953	2.01872	0.910733	1.64207
XLOC_065043	ACSF2	chr19	36896477	36897509	2.48114	0.494721	2.58778	0.88956	1.74646	1.03004	0.675477	0.662111	1.30061
XLOC_041662	ACTN1	chr10	81070022	81082767	26.2353	35.9599	30.3131	68.2166	49.8919	59.7549	55.1289	88.8969	56.596

XLOC_041663	ACTN1	chr10	81105635	81124524	24.7009	43.8791	33.8306	42.6697	22.4261	31.7093	32.8609	50.9017	22.243
XLOC_042064	ACTN1	chr10	81028824	81037272	2.06075	2.84295	2.15067	0.800886	0.675482	0.763274	0.588302	0.923308	0.801906
XLOC_042065	ACTN1	chr10	81044005	81060236	42.9194	50.0669	41.7706	53.8767	47.4214	44.9087	50.5674	60.8342	45.5515
XLOC_042066	ACTN1	chr10	81084483	81099080	0.0	0.25184	0.658684	0.377374	0.891612	0.743962	0.615693	0.550239	0.994206
XLOC_043092	ACTN1	chr10	81043431	81043859	2.67391	1.59664	2.08763	0.717679	0.0	0.548772	0.0	0.0	0.464121
XLOC_059684	ACTR3	chr17	28800093	28838883	30.5751	36.9252	35.7072	38.0668	41.7917	42.5489	33.0071	33.0591	28.3421
XLOC_061012	ACTR3	chr17	28800093	28838883	0.715731	1.07045	2.23974	2.5664	1.23261	1.56114	1.23663	1.50617	1.18856
XLOC_061013	ACTR3	chr17	28800093	28838883	2.4899	1.48701	1.94432	3.34196	2.30605	2.5582	0.876838	2.68609	1.5136
XLOC_061014	ACTR3	chr17	28800093	28838883	1.13632	1.69879	2.66574	2.34182	3.1028	2.70747	3.58861	2.0391	1.68579
XLOC_065664	ACTR3	chr2	65845748	66031318	0.179119	0.318178	0.210336	6.29378	6.23783	7.49597	9.42413	10.733	7.76246
XLOC_067757	ACTR3	chr2	65845748	66031318	0.426056	0.127479	0.0	0.993312	0.668598	0.975249	0.428485	0.428471	0.410051
XLOC_067758	ACTR3	chr2	65845748	66031318	0.0	0.0	0.0	1.8904	1.79829	1.52213	0.561582	2.91279	1.28553
XLOC_067759	ACTR3	chr2	65845748	66031318	0.0	0.0	0.0	3.29731	3.4031	2.35226	4.38027	4.85792	4.88567
XLOC_065628	ACVR1C	chr2	38511691	38586194	6.99476	9.03946	6.0166	11.8994	10.3769	12.3938	13.4323	14.5894	12.7632
XLOC_066270	ACVR1C	chr2	38586801	38643972	126.639	153.973	130.885	221.9	207.625	216.772	216.901	255.813	263.81
XLOC_067375	ACVR1C	chr2	38586338	38586660	4.38762	1.30835	3.42102	1.17713	2.33832	2.23101	2.24579	3.79239	2.27295
XLOC_042100	ACYP1	chr10	86698232	86705713	127.276	133.953	115.736	177.026	174.198	183.915	209.249	221.115	220.092
XLOC_074428	ADAM22	chr4	72542665	72543083	11.1059	8.28867	13.005	11.4256	7.26974	6.54903	5.59371	7.87	5.54019
XLOC_086485	ADAMDEC1	chr8	72492727	72548353	1.75705	1.27144	0.953504	1.24245	1.31102	1.8173	1.35715	1.38423	0.353268
XLOC_086945	ADAMDEC1	chr8	72492727	72548353	13.6924	13.5869	14.0412	12.072	10.2779	9.96187	11.1664	17.4305	8.31075
XLOC_088125	ADAMDEC1	chr8	72492727	72548353	0.867979	1.42834	0.679232	0.544806	0.646943	0.541823	0.39672	0.305479	0.531577
XLOC_088126	ADAMDEC1	chr8	72492727	72548353	1.01809	1.02538	2.19134	0.729427	0.598547	0.535628	0.489432	0.256438	0.43338
XLOC_088127	ADAMDEC1	chr8	72492727	72548353	2.6899	1.21268	4.03002	0.666292	1.96204	1.81833	2.93454	0.501926	0.266022
XLOC_088128	ADAMDEC1	chr8	72492727	72548353	4.74298	13.2301	12.3571	11.7523	13.0277	12.9021	15.4228	16.1461	17.2181
XLOC_088129	ADAMDEC1	chr8	72492727	72548353	0.0	1.51351	0.791673	1.63285	0.711466	0.73457	0.915469	0.303207	0.353511
XLOC_088130	ADAMDEC1	chr8	72492727	72548353	1.26562	3.40522	2.96856	1.70077	1.67676	0.654962	1.59492	0.503878	0.441567
XLOC_088131	ADAMDEC1	chr8	72492727	72548353	0.688501	0.823776	1.07726	0.740624	0.916405	1.57336	1.1275	1.10423	0.902699
XLOC_088132	ADAMDEC1	chr8	72492727	72548353	8.00439	14.3239	3.12119	2.50511	2.74929	2.85418	2.74764	1.54352	5.5343
XLOC_088133	ADAMDEC1	chr8	72492727	72548353	0.0	0.518946	1.35715	0.622042	1.61802	1.25457	0.774916	0.687464	0.45366
XLOC_088134	ADAMDEC1	chr8	72492727	72548353	3.5484	2.16298	2.75252	1.51878	1.28333	1.93351	1.796	1.87638	1.23665
XLOC_088135	ADAMDEC1	chr8	72492727	72548353	0.665199	1.2938	1.30151	1.25271	1.48863	1.24651	1.15753	0.703218	0.960628
XLOC_088136	ADAMDEC1	chr8	72492727	72548353	3.92567	1.87689	2.56218	1.99591	2.10721	1.90853	1.71009	1.44848	1.40015
XLOC_038836	ADAMTS1	chr1	8957297	8957566	0.0	0.0	0.0	0.574614	1.4478	1.94146	5.85582	0.604753	1.65425

XLOC_038837	ADAMTS1	chr1	8958114	8958748	0.0	0.0	0.0	1.08765	1.41673	1.56866	0.544031	1.05436	0.925972
XLOC_062130	ADAMTS18	chr18	4604875	4680553	30.0515	17.884	25.6304	1.93623	1.95527	1.68174	1.95066	1.26817	1.69368
XLOC_062550	ADAMTS18	chr18	4680638	4683327	16.57	8.89115	10.5094	0.871128	1.12208	0.773524	0.890408	1.00733	0.800287
XLOC_082848	ADAMTS19	chr7	25619216	25658935	16.4837	20.5562	17.0018	32.9464	31.0778	29.3699	28.6369	33.3728	31.4908
XLOC_082849	ADAMTS19	chr7	25619216	25658935	1.81487	0.434451	1.98849	0.618449	0.427422	0.717838	0.204578	0.450104	0.254129
XLOC_082850	ADAMTS19	chr7	25659119	25668093	17.941	16.0991	8.18724	72.9063	53.8813	62.3315	68.8894	88.9585	58.1427
XLOC_082851	ADAMTS19	chr7	25680537	25686791	0.551038	1.81339	2.15583	2.56906	3.45543	3.38003	4.52364	3.20919	2.024
XLOC_082852	ADAMTS19	chr7	25687034	25704924	11.0567	8.27086	7.10773	0.991485	0.929753	1.31501	0.650235	0.834277	0.172769
XLOC_083468	ADAMTS19	chr7	25614618	25615857	1.00523	0.0	0.0	0.0	0.0	0.0	0.0	0.0	0.0
XLOC_083469	ADAMTS19	chr7	25619216	25658935	25.6745	34.3247	28.8429	55.5329	45.4664	53.447	45.5577	63.2232	53.4837
XLOC_083470	ADAMTS19	chr7	25712826	25719677	16.331	18.3637	18.7842	6.87626	6.22923	5.95765	6.79732	6.5478	6.3738
XLOC_083471	ADAMTS19	chr7	25721840	25756249	154.522	204.713	163.413	130.093	144.894	128.145	128.699	137.548	141.906
XLOC_083472	ADAMTS19	chr7	25757210	25788690	109.523	95.6546	99.2683	1.46135	0.893546	1.10541	0.765335	0.5923	0.881965
XLOC_083473	ADAMTS19	chr7	25803394	25816731	0.0	1.61167	1.29701	6.42767	6.56776	7.15685	3.59958	2.79222	7.83082
XLOC_084396	ADAMTS19	chr7	25668607	25669656	0.0	2.66912	0.634617	3.41772	1.523	1.85257	0.589146	1.38042	1.70117
XLOC_084397	ADAMTS19	chr7	25670453	25670971	6.01931	2.99704	1.56755	2.69429	2.95351	1.86051	2.13823	1.97919	0.872715
XLOC_084398	ADAMTS19	chr7	25674353	25675099	3.68705	6.24648	0.96097	0.440452	1.24555	0.254474	0.553531	0.48954	0.536085
XLOC_084399	ADAMTS19	chr7	25710603	25712695	5.98172	4.69838	6.72942	2.74909	2.25969	2.25684	1.91599	2.67116	1.63583
XLOC_084400	ADAMTS19	chr7	25719936	25720274	4.00213	4.77463	12.4848	3.57872	2.44381	1.22322	2.40418	1.54352	2.76715
XLOC_084401	ADAMTS19	chr7	25720400	25721721	23.6211	20.4549	22.372	14.545	13.3916	12.5964	14.8358	15.0294	13.9675
XLOC_084402	ADAMTS19	chr7	25802104	25803256	1.45449	3.04546	7.39618	7.56221	7.05857	7.32596	4.36383	4.80916	9.3433
XLOC_076302	ADAMTS20	chr5	37028353	37125094	2.25191	2.55316	2.63049	16.1681	22.2147	16.8824	12.7357	21.3787	31.9747
XLOC_076303	ADAMTS20	chr5	37147997	37149971	0.970169	0.0	0.0	0.260836	1.66738	0.704206	0.0	0.581495	1.27073
XLOC_076304	ADAMTS20	chr5	37242225	37292466	0.0	0.577198	0.566124	23.2158	17.6753	21.4807	8.39444	15.7912	6.97371
XLOC_076305	ADAMTS20	chr5	37299169	37410722	4.14612	1.73127	1.5903	6.01134	6.583	6.40856	8.57764	7.26956	5.57667
XLOC_076829	ADAMTS20	chr5	37299169	37410722	2.24165	1.85191	0.877347	7.58953	10.2331	7.5681	9.78727	9.27915	9.84013
XLOC_077750	ADAMTS20	chr5	37125153	37125371	0.0	6.63872	0.0	5.0381	3.29105	5.56386	2.66113	6.11981	10.4863
XLOC_077751	ADAMTS20	chr5	37242225	37292466	0.0	0.0	0.0	0.545722	0.408033	0.902515	0.315488	1.30498	0.379904
XLOC_077752	ADAMTS20	chr5	37299169	37410722	0.0	0.162137	0.0	1.31195	2.33662	1.97216	2.07667	2.17699	1.84851
XLOC_080218	ADAMTS3	chr6	89315953	89595633	30.1797	37.7818	34.3781	28.8621	27.2491	24.1172	19.656	24.4658	19.8482
XLOC_081598	ADAMTS3	chr6	89200789	89201064	12.1906	7.26823	0.0	14.2	13.7893	9.85598	19.817	16.7193	15.2137
XLOC_081599	ADAMTS3	chr6	89288040	89288753	0.0	0.389242	0.0	0.699877	0.304334	0.538941	0.351379	1.42494	0.227113
XLOC_081600	ADAMTS3	chr6	89314701	89315884	0.705262	1.05481	1.65525	0.505777	0.607342	0.659269	0.256584	0.424094	0.55479

XLOC_070625	ADAMTS4	chr3	8327577	8328179	19.5266	11.1849	2.54358	19.8192	21.7495	23.5265	25.7552	28.3805	25.7984
XLOC_070626	ADAMTS4	chr3	8328571	8329185	9.50664	10.8913	8.66889	19.1571	20.0764	20.7863	23.8215	22.7788	23.1915
XLOC_045136	ADAMTSL2;FAM163B	chr11	104502320	104531856	24.8143	24.6491	21.0626	30.0434	28.5825	32.1345	37.0443	38.9924	36.6396
XLOC_050530	ADARB2	chr13	46418962	46429088	11.8758	1.16083	1.43998	0.353972	0.0	0.0	0.339949	0.381854	0.0
XLOC_051758	ADARB2	chr13	46418467	46418850	0.0	0.956967	0.0	0.0	0.0	0.328061	0.0	0.0	0.277809
XLOC_051759	ADARB2	chr13	46418962	46429088	0.0	0.0	1.1915	0.0	0.0	0.0	0.136565	0.0	0.0
XLOC_051760	ADARB2	chr13	46429269	46431074	0.877973	0.394038	1.03059	0.0	0.0	0.0	0.120377	0.088281	0.0768139
XLOC_065836	ADC	chr2	121413825	121437940	190.014	205.695	171.56	212.42	205.218	218.044	239.399	277.456	220.256
XLOC_066503	ADC	chr2	121438395	121442610	6.34255	7.51161	6.25299	6.25079	7.26111	7.29535	3.62329	5.09505	7.06799
XLOC_041717	ADCK1	chr10	90069468	90077288	0.0	8.23014	5.06442	0.725387	0.0	0.501903	0.289754	0.160545	0.423355
XLOC_043296	ADCK1	chr10	90113928	90114700	0.0	0.0	0.0	0.105459	0.0	0.0	0.0	0.0	0.1027
XLOC_043297	ADCK1	chr10	90133048	90134814	10.3415	11.7041	13.3705	1.37079	1.12874	1.35649	1.84907	2.26024	1.37678
XLOC_073265	ADCK2;NDUFB2	chr4	104817276	104876301	0.0	0.0	0.0	0.109088	0.0901466	0.239142	0.0741781	0.236993	0.106447
XLOC_058747	ADCK3	chr16	30697248	30698191	0.0	0.0	0.0	1.40287	0.431753	0.573041	0.834125	0.92036	0.96499
XLOC_074448	ADCY1	chr4	76927544	76927670	1198.56	707.46	506.329	925.735	734.008	763.216	469.731	863.811	792.93
XLOC_076792	ADCY6	chr5	31141692	31180721	38.5853	46.8721	42.1404	63.4511	65.494	61.8399	61.5163	81.5112	60.0333
XLOC_077587	ADCY6	chr5	31141692	31180721	0.0	2.69261	2.34702	2.68972	2.31179	1.23193	3.67291	2.93136	4.43162
XLOC_077588	ADCY6	chr5	31141692	31180721	3.09848	2.781	4.04087	3.00964	3.52265	2.79242	3.44084	3.6832	2.07773
XLOC_077589	ADCY6	chr5	31141692	31180721	11.3523	0.0	8.84779	9.65659	8.14978	6.89247	5.23186	3.77093	7.82109
XLOC_077590	ADCY6	chr5	31141692	31180721	4.31481	2.25783	5.06143	6.95956	8.75628	6.4832	7.21022	6.34924	6.40255
XLOC_077591	ADCY6	chr5	31141692	31180721	6.578	15.6891	15.3817	12.3887	12.3733	10.6223	5.4533	7.43873	7.35578
XLOC_077592	ADCY6	chr5	31141692	31180721	0.0	2.93262	0.0	5.71995	7.46478	10.4802	10.8248	5.16924	8.90696
XLOC_076452	ADIPOR2	chr5	108669304	108706970	14.3295	9.80938	9.41062	2.83282	5.19923	3.17786	2.77115	1.25793	2.91339
XLOC_078607	ADIPOR2	chr5	108669304	108706970	0.0	0.432824	0.0	0.389116	0.338061	0.449121	0.519642	0.143809	0.252444
XLOC_078608	ADIPOR2	chr5	108669304	108706970	1.4746	1.32227	1.15269	0.0	0.458922	0.0	0.528921	0.1464	0.257052
XLOC_078609	ADIPOR2	chr5	108669304	108706970	0.663052	1.78567	0.7784	0.148654	0.260326	0.207087	0.212549	0.100139	0.377216
XLOC_088193	ADRA1A	chr8	75362505	75362831	12.8537	6.38844	3.34087	5.36407	4.56986	6.10299	2.56256	3.29527	2.9602
XLOC_088194	ADRA1A	chr8	75363318	75363610	0.0	17.5351	16.6717	11.4864	8.50088	11.3762	7.65007	8.6521	8.29379
XLOC_085087	ADRA1B	chr7	73616321	73617825	0.0	0.48208	0.0	1.4929	1.47374	0.949397	0.784099	0.970971	1.31533
XLOC_085088	ADRA1B	chr7	73617887	73618368	2.23594	0.667825	0.0	4.00238	1.03748	0.690135	0.790985	0.659845	0.388695
XLOC_085089	ADRA1B	chr7	73618455	73619623	0.715731	0.428181	0.559935	2.5664	1.68083	1.78403	1.30171	1.57789	0.875785
XLOC_059881	ADRBK2	chr17	67695705	67822730	4.91083	5.72459	5.66795	2.65704	1.78331	1.95286	7.40185	8.43311	7.89436

XLOC_057879	ADSS;C16H1orf100	chr16	33517388	33663391	3.94965	2.3609	1.51937	7.21571	7.35898	7.28861	10.001	5.2796	5.59285
XLOC_058848	ADSS;C16H1orf100	chr16	33517388	33663391	0.0	0.42685	0.0	0.511661	0.555749	0.738279	1.28162	0.851134	0.248973
XLOC_076397	AEBP2	chr5	90786173	90905320	117.93	102.953	88.1181	2.01414	1.22302	1.90092	2.9908	1.15667	0.739655
XLOC_076398	AEBP2	chr5	90931290	91027536	2.84998	3.78454	4.04541	1.05195	0.702807	1.75881	1.60692	2.22195	1.04997
XLOC_078300	AEBP2	chr5	90786173	90905320	19.3935	52.036	60.4645	0.579637	0.0	0.65265	0.536607	0.0	0.0
XLOC_078301	AEBP2	chr5	90786173	90905320	32.4053	0.879328	0.0	0.263512	0.0	0.0	0.0	0.0	0.0
XLOC_078302	AEBP2	chr5	90905375	90905864	21.8199	1.95522	1.70439	0.585899	0.0	0.0	0.0	0.0	0.0
XLOC_078303	AEBP2	chr5	90931290	91027536	0.0	0.629017	1.64497	0.188496	0.163013	0.0	0.373342	0.414935	0.0
XLOC_078304	AEBP2	chr5	90931290	91027536	3.24713	0.970422	0.0	0.290809	0.252341	0.0	0.435579	0.482696	0.141438
XLOC_078305	AEBP2	chr5	90931290	91027536	0.0	0.623765	1.0876	0.124624	0.0544215	0.0	0.1897	0.487705	0.24303
XLOC_078306	AEBP2	chr5	90931290	91027536	0.0	0.0	0.0	0.549513	0.0	0.210766	0.0	0.201745	0.0
XLOC_083651	AFF4	chr7	46104579	46107073	0.0	5.06303	5.22809	0.0781481	0.0	0.0	0.0	0.0	0.0
XLOC_083652	AFF4	chr7	46108072	46180032	0.890989	2.2399	1.81334	0.0639324	0.0140084	0.0928362	0.0654636	0.143807	0.0624139
XLOC_084857	AFF4	chr7	46108072	46180032	0.0	0.0	1.33166	0.0	0.0	0.0	0.0	0.0	0.0
XLOC_080220	AFM	chr6	90309225	90325207	0.0	0.0	0.0	0.116228	0.0	0.0	0.116716	0.0	0.113151
XLOC_081608	AFM	chr6	90308341	90309063	0.0	0.0	0.0	0.0	0.0	0.0	0.115303	0.0	0.0
XLOC_072712	AGBL3	chr4	99649967	99652256	0.0	0.172472	0.0	2.1192	1.1747	1.67798	1.52429	1.67826	2.01656
XLOC_073254	AGBL3	chr4	99652730	99655681	0.0	0.229278	0.300822	7.00997	3.79506	4.67929	5.76088	7.66681	6.04521
XLOC_073255	AGBL3	chr4	99655918	99659008	0.0	0.0779778	0.815803	8.01576	2.90715	4.53232	6.90634	11.8427	5.10998
XLOC_073857	AGMO	chr4	23517071	23517827	3.62544	2.52915	3.77973	3.35653	4.42863	4.50456	4.79149	5.05555	3.37386
XLOC_073858	AGMO	chr4	23518018	23518524	14.5288	19.8403	9.72852	12.2623	13.6606	12.1838	18.9615	20.4564	12.8161
XLOC_089573	AGPAT4	chr9	98296925	98410921	1.88264	3.56684	3.79731	0.872924	1.8028	0.816218	0.628755	0.37772	1.53629
XLOC_065496	AGPS	chr2	19371638	19395199	0.783158	0.702732	1.349	15.6632	14.8174	15.8306	10.2697	11.7685	11.4077
XLOC_073096	AGR2;TSPAN13	chr4	25249420	25281824	6.77093	12.1424	9.25899	13.7647	13.7485	13.3093	14.9655	13.0379	14.6023
XLOC_073872	AGR2;TSPAN13	chr4	25249420	25281824	4.56342	2.72784	1.189	2.45236	1.89297	1.88631	2.04429	0.754711	1.06047
XLOC_057853	AHCTF1	chr16	31252966	31275443	7.47893	1.77141	7.39552	2.75649	2.35304	3.49741	2.03556	1.1943	1.65818
XLOC_058093	AHCTF1	chr16	31106361	31201301	23.9479	20.4545	18.25	19.0169	20.7427	19.437	20.1965	15.6437	22.5312
XLOC_058094	AHCTF1	chr16	31252966	31275443	32.9393	41.5833	34.8171	31.6739	29.3446	26.1008	30.8822	33.8905	29.2336
XLOC_058751	AHCTF1	chr16	31249311	31250022	1.30674	3.90647	4.08664	0.936537	1.83253	0.676091	1.17539	0.650006	0.683791
XLOC_058752	AHCTF1	chr16	31250826	31251356	0.0	2.32053	0.0	1.21691	1.20414	1.60114	2.07177	1.15015	1.18271
XLOC_058753	AHCTF1	chr16	31251582	31252271	2.72167	6.10196	7.44724	3.7791	4.66293	4.08229	5.01363	5.14115	3.08515

XLOC_058754	AHCTF1	chr16	31252485	31252744	7.00625	2.08913	5.46174	5.66037	5.78798	2.11918	4.04727	5.26594	4.21699
XLOC_058755	AHCTF1	chr16	31252966	31275443	3.91315	4.28938	13.2576	2.33712	3.04877	2.02464	4.3413	3.63357	2.73025
XLOC_058756	AHCTF1	chr16	31252966	31275443	4.6678	15.3089	0.0	5.42745	4.96651	8.05997	5.55165	3.57639	4.83299
XLOC_058757	AHCTF1	chr16	31252966	31275443	8.99129	20.1073	17.5252	4.42264	5.13051	6.39756	5.36049	4.74415	4.6566
XLOC_058758	AHCTF1	chr16	31252966	31275443	10.6239	3.70362	8.30207	3.8052	2.8862	2.55774	3.63218	4.02868	2.46661
XLOC_058759	AHCTF1	chr16	31252966	31275443	8.11783	2.42605	7.61365	2.32644	2.64958	1.34138	3.04904	2.09167	1.98007
XLOC_058760	AHCTF1	chr16	31252966	31275443	0.0	4.8545	15.867	2.91101	4.65687	2.48665	2.79076	3.92076	2.46141
XLOC_058761	AHCTF1	chr16	31252966	31275443	2.47288	4.43059	1.93106	1.77022	3.05412	4.57307	2.83109	1.69799	4.29526
XLOC_058762	AHCTF1	chr16	31252966	31275443	7.70747	3.44851	0.0	1.723	2.06114	2.75036	0.994504	1.11647	1.99924
XLOC_058763	AHCTF1	chr16	31252966	31275443	4.40383	2.63072	5.15977	2.95619	2.21426	3.62545	2.72798	2.38358	3.0626
XLOC_058764	AHCTF1	chr16	31252966	31275443	12.192	6.53537	2.75459	1.65708	3.52431	4.67853	3.16727	2.61173	1.90657
XLOC_058765	AHCTF1	chr16	31252966	31275443	6.54266	4.07787	5.97265	2.98191	2.69255	2.60756	2.93452	1.53302	2.86098
XLOC_050914	AHCY;ASIP	chr13	64189421	64275083	12.6645	15.8607	15.2884	5.67913	7.41999	6.56355	7.66099	6.90675	8.66928
XLOC_052244	AHCY;ASIP	chr13	64189421	64275083	5.17262	6.69808	1.34756	3.08799	4.0165	2.31342	2.30864	1.87729	4.80465
XLOC_052245	AHCY;ASIP	chr13	64189421	64275083	10.4982	11.2895	13.1218	7.89369	8.28994	10.3777	9.49357	10.5508	10.7727
XLOC_052246	AHCY;ASIP	chr13	64189421	64275083	3.18957	13.5184	15.3906	7.00643	7.4599	7.35146	7.37305	7.79514	7.90391
XLOC_073097	AHR	chr4	25618694	25799841	0.0	0.161824	0.0	11.0796	14.6531	14.0075	18.0283	8.50083	13.599
XLOC_073874	AHR	chr4	25618694	25799841	0.0	0.0	0.0	0.790535	1.35944	1.50912	3.08744	0.574753	0.510867
XLOC_073875	AHR	chr4	25618694	25799841	0.0	0.0	0.0	3.13304	1.66065	1.10558	0.0	0.790662	1.16886
XLOC_073876	AHR	chr4	25618694	25799841	0.0	0.0	0.0	1.61878	9.36583	1.80276	1.45479	0.832836	1.54124
XLOC_073877	AHR	chr4	25618694	25799841	0.0	0.0	0.0	1.17136	1.02217	1.52596	2.14991	1.14444	1.35586
XLOC_073878	AHR	chr4	25618694	25799841	0.0	0.0	0.0	0.864791	1.1718	1.77793	2.61546	0.74889	0.84239
XLOC_073879	AHR	chr4	25618694	25799841	0.0	0.0	0.0	0.0	1.32321	1.78254	8.63654	0.0	1.52378
XLOC_073880	AHR	chr4	25618694	25799841	0.0	0.0	0.0	0.543827	1.53479	2.19612	2.17612	1.95809	1.58738
XLOC_073881	AHR	chr4	25618694	25799841	0.0	0.0	0.0	2.66208	1.9731	1.09354	3.38853	1.46473	2.21661
XLOC_073882	AHR	chr4	25618694	25799841	0.0	0.0	0.0	1.34552	1.81921	2.08135	2.6593	1.14284	1.75037
XLOC_073883	AHR	chr4	25618694	25799841	0.0	0.0	0.0	1.00681	1.39176	5.3235	3.38175	1.32768	1.36883
XLOC_073884	AHR	chr4	25618694	25799841	0.0	0.0	0.0	1.17076	2.84186	2.56992	11.1749	4.68432	7.71587
XLOC_073885	AHR	chr4	25618694	25799841	0.0	0.0	0.0	1.77338	1.54812	2.21214	1.79754	0.762077	1.1304
XLOC_073886	AHR	chr4	25618694	25799841	0.0	0.0	0.0	2.95756	2.45575	1.65212	8.04022	0.765644	4.23363
XLOC_073887	AHR	chr4	25618694	25799841	0.0	0.0	0.0	1.80629	2.72068	1.42539	2.57291	1.73992	2.08025
XLOC_073888	AHR	chr4	25618694	25799841	0.0	0.0	0.0	3.11635	2.62948	1.91443	4.41688	1.19253	3.69953
XLOC_073889	AHR	chr4	25618694	25799841	0.0	0.0	0.0	2.59355	2.24107	2.06411	2.95742	1.75431	2.71255

XLOC_073890	AHR	chr4	25618694	25799841	0.0	0.0	1.4623	3.51872	4.35436	1.92976	3.49871	1.66415	2.28031
XLOC_073891	AHR	chr4	25618694	25799841	0.0	0.0	0.0	1.6293	1.97661	2.06465	3.08079	2.51861	2.21762
XLOC_073892	AHR	chr4	25618694	25799841	0.0	0.0	0.0	1.23441	3.17305	5.28857	4.48217	2.34302	3.88351
XLOC_073893	AHR	chr4	25618694	25799841	0.0	0.0	0.0	2.94444	3.44741	2.04447	4.16541	2.06376	2.29533
XLOC_073894	AHR	chr4	25618694	25799841	0.0	0.0	0.0	2.53461	3.15036	3.41011	3.33433	2.10146	2.50906
XLOC_073895	AHR	chr4	25799914	25800552	0.0	0.0	0.0	0.943786	1.28799	0.933432	1.07925	0.0	1.70542
XLOC_042111	AHSA1;C10H14orf133	chr10	89711981	89732363	12.1845	13.7265	20.9547	17.643	17.023	18.4705	22.1554	21.9453	25.0569
XLOC_044111	AHSA2	chr11	59662704	59777995	66.0565	73.5982	79.9512	24.038	17.5996	20.2102	20.719	25.5502	16.3786
XLOC_044801	AHSA2	chr11	59784313	59787657	4.23833	3.07921	3.31614	2.98758	2.1822	3.52988	1.76551	2.06917	2.27839
XLOC_046232	AHSA2	chr11	59662704	59777995	5.83886	5.67208	1.14108	0.915314	0.340762	0.754526	0.523733	0.869707	0.763424
XLOC_046233	AHSA2	chr11	59662704	59777995	8.77523	15.7002	20.5261	1.17731	2.00428	1.33862	1.49721	0.842788	0.757681
XLOC_046234	AHSA2	chr11	59662704	59777995	2.53349	1.51299	7.9131	0.226789	1.17282	0.260199	0.44576	0.248358	0.219998
XLOC_046235	AHSA2	chr11	59662704	59777995	6.11019	4.10903	9.55213	0.273694	0.475202	0.947106	0.957926	1.06093	0.399356
XLOC_046236	AHSA2	chr11	59662704	59777995	4.0434	2.11596	5.53398	0.181214	0.31577	0.314381	0.274269	0.302797	0.176516
XLOC_046237	AHSA2	chr11	59662704	59777995	0.791062	2.24863	2.47631	0.212825	0.155213	0.28813	0.144734	0.079588	0.173055
XLOC_046238	AHSA2	chr11	59662704	59777995	2.09794	1.88201	4.92209	0.28204	0.327578	0.543597	0.474017	0.732815	0.274718
XLOC_046239	AHSA2	chr11	59662704	59777995	0.0	0.0	6.20616	0.0	0.0	0.0	0.0	0.0	0.0
XLOC_046240	AHSA2	chr11	59662704	59777995	1.96821	1.76404	3.0755	0.352487	0.30507	0.811338	0.0	0.388493	0.0
XLOC_046241	AHSA2	chr11	59662704	59777995	0.0	0.961966	3.77387	0.144174	0.188694	0.166927	0.218998	0.402437	0.281019
XLOC_046242	AHSA2	chr11	59662704	59777995	1.3437	2.61265	2.62814	0.180713	0.210446	0.20939	0.0611483	0.0	0.352384
XLOC_046243	AHSA2	chr11	59662704	59777995	6.09532	7.26825	19.0027	0.546395	0.0	0.616014	0.0	0.0	1.04926
XLOC_046244	AHSA2	chr11	59662704	59777995	2.15418	1.28827	1.68463	0.0	0.0840727	0.223231	0.0	0.214921	0.188038
XLOC_046245	AHSA2	chr11	59662704	59777995	0.0	1.06091	5.54921	0.211999	0.0922442	0.0	0.106639	0.117858	0.206411
XLOC_046246	AHSA2	chr11	59662704	59777995	0.0	0.746076	1.95102	0.447229	0.192827	0.0	0.439876	0.0	0.0
XLOC_046247	AHSA2	chr11	59662704	59777995	2.86436	3.42017	6.70773	0.512571	0.0	0.0	0.50105	0.55939	0.0
XLOC_046248	AHSA2	chr11	59662704	59777995	0.0	2.39162	2.50201	0.0	0.125106	0.166009	0.145205	0.160098	0.0
XLOC_046249	AHSA2	chr11	59662704	59777995	1.19593	1.43108	0.935724	0.268073	0.0468548	0.1243	0.108994	0.0600133	0.104575
XLOC_046250	AHSA2	chr11	59662704	59777995	4.43124	3.21928	3.96226	0.454039	0.19835	0.394681	0.172965	0.317506	0.276729
XLOC_046251	AHSA2	chr11	59662704	59777995	3.754	3.45653	5.87634	0.362577	0.272197	0.511209	0.238225	0.116362	0.480289
XLOC_046252	AHSA2	chr11	59662704	59777995	7.46905	4.46781	9.08837	0.669497	0.259621	0.172263	0.0753117	0.249145	0.362525
XLOC_046253	AHSA2	chr11	59662704	59777995	2.65402	3.17697	4.91009	0.194762	0.189596	0.326725	0.309898	0.19456	0.253494
XLOC_046254	AHSA2	chr11	59662704	59777995	4.34085	3.319	3.01937	0.259499	0.151309	0.200663	0.220256	0.19389	0.0421945

XLOC_046255	AHSA2	chr11	59662704	59777995	0.682628	1.22517	2.13622	0.0612216	0.106903	0.141824	0.12423	0.205305	0.0
XLOC_046256	AHSA2	chr11	59662704	59777995	6.24215	2.5465	3.99613	0.356163	0.26685	0.294956	0.0517485	0.227881	0.0496283
XLOC_046257	AHSA2	chr11	59662704	59777995	2.63162	1.83645	1.37229	0.157278	0.274317	0.18203	0.0795354	0.0877366	0.229878
XLOC_046258	AHSA2	chr11	59662704	59777995	1.62722	1.94521	2.54358	0.145793	0.0	0.336097	0.0	0.0	0.567009
XLOC_046259	AHSA2	chr11	59662704	59777995	6.62591	4.6178	1.72517	0.593127	0.341681	0.909099	0.195438	0.217352	0.192007
XLOC_046260	AHSA2	chr11	59662704	59777995	0.827047	2.47361	5.17555	0.0741627	0.258736	0.343347	0.15011	0.165535	0.216773
XLOC_046261	AHSA2	chr11	59662704	59777995	1.34564	3.2181	1.05203	0.120601	0.314419	0.417634	0.0	0.0	0.11735
XLOC_046262	AHSA2	chr11	59662704	59777995	0.0	3.57555	18.6964	0.537501	0.0	0.0	0.0	0.0	0.516266
XLOC_046263	AHSA2	chr11	59662704	59777995	3.06997	1.83696	1.20112	0.275279	0.160478	0.212834	0.046708	0.15421	0.0895104
XLOC_046264	AHSA2	chr11	59662704	59777995	3.03227	1.81008	2.36661	0.271339	0.0	0.310524	1.05774	0.0	0.262865
XLOC_046265	AHSA2	chr11	59662704	59777995	2.70774	2.10621	2.96621	0.145685	0.339619	0.112612	0.14822	0.108773	0.284177
XLOC_046266	AHSA2	chr11	59662704	59777995	2.47486	1.84976	2.90262	0.110915	0.192924	0.128104	0.0	0.12322	0.107956
XLOC_046267	AHSA2	chr11	59662704	59777995	2.73452	6.53099	0.0	0.0	0.210658	0.0	0.23972	0.267432	0.0
XLOC_046268	AHSA2	chr11	59662704	59777995	18.5529	29.0412	47.0072	3.73377	2.46798	3.76937	3.15365	5.33216	4.40249
XLOC_046269	AHSA2	chr11	59662704	59777995	21.2259	29.8661	16.5663	2.98353	1.16532	2.17362	1.58661	1.18204	0.788553
XLOC_046270	AHSA2	chr11	59778491	59779361	11.1662	8.19588	3.96947	1.27356	1.42689	1.36801	2.20318	1.31754	0.709
XLOC_046271	AHSA2	chr11	59779494	59781163	3.34941	5.01056	5.24197	0.772262	0.863143	0.846064	0.437041	0.721332	0.418568
XLOC_046272	AHSA2	chr11	59782811	59783347	0.0	5.70952	10.452	1.36875	0.296334	0.788029	0.170005	0.377447	1.66299
XLOC_046273	AHSA2	chr11	59783747	59784136	0.0	5.59367	2.43784	1.67634	3.35905	2.23807	2.44787	1.52053	1.35348
XLOC_044460	AIF1L	chr11	101331902	101338211	6.71795	2.13551	4.60011	0.9789	1.08723	1.57024	0.4605	0.210907	0.771481
XLOC_091366	AIG1	chr9	81677434	81678396	136.65	151.64	104.775	1142.79	941.005	1110.21	1164.12	1508.38	1075.84
XLOC_057931	AJAP1	chr16	49855117	49911546	17.8277	11.2948	8.9727	2.54999	2.66923	2.54317	0.98934	0.734907	2.25124
XLOC_059126	AJAP1	chr16	49912014	49913773	4.51596	2.02676	1.41357	0.283454	0.460546	0.610732	0.206343	0.0454013	0.197542
XLOC_069930	AK5	chr3	67374109	67400049	21.8174	23.0585	26.7401	2.73805	3.52929	2.84655	2.64475	1.74917	2.6696
XLOC_069931	AK5	chr3	67429985	67475045	11.0762	11.3884	9.11375	49.1072	41.2508	46.8779	51.6014	55.7644	48.1822
XLOC_069932	AK5	chr3	67582644	67603473	29.1209	26.1419	19.9424	18.7555	15.669	17.9147	19.034	17.7255	17.2623
XLOC_070310	AK5	chr3	67429985	67475045	11.0861	8.94645	11.2982	3.06067	3.23677	3.2718	3.65181	2.48686	2.70867
XLOC_070311	AK5	chr3	67478674	67515690	6.20609	5.83588	3.46898	0.119247	0.13911	0.0461179	0.121548	0.0891421	0.077566
XLOC_071393	AK5	chr3	67357280	67358779	1.07825	2.41938	0.42185	4.10869	5.02931	5.10095	5.80407	4.11484	5.6581
XLOC_071394	AK5	chr3	67475538	67475750	0.0	3.61688	9.45423	8.80127	4.46799	4.84247	9.60377	5.52986	8.30346
XLOC_071395	AK5	chr3	67475889	67476950	0.8005	1.67599	0.0	1.5068	1.69076	1.91118	2.47111	2.24373	3.00756
XLOC_071396	AK5	chr3	67603620	67604046	13.4691	8.84672	6.30939	14.2192	13.2852	12.9906	16.0688	15.5497	16.1303
XLOC_071397	AK5	chr3	67606072	67607789	2.31879	1.38755	1.45163	1.16434	1.92799	1.44723	1.35586	1.77145	1.05484

XLOC_069929	AK5;ZZZ3;USP33	chr3	67082687	67357043	5.25059	8.21627	6.16035	9.20732	11.2802	10.8038	16.2471	11.9785	14.8124
XLOC_071380	AK5;ZZZ3;USP33	chr3	67082687	67357043	0.0	0.756784	0.659759	0.755987	0.593617	0.962825	0.765181	0.506326	0.957897
XLOC_071381	AK5;ZZZ3;USP33	chr3	67082687	67357043	1.30675	1.95324	5.10829	0.819473	0.916267	0.946532	0.94032	0.520004	0.797763
XLOC_071382	AK5;ZZZ3;USP33	chr3	67082687	67357043	3.10193	0.618377	0.808633	0.277974	0.484399	0.0	0.747786	0.412835	0.902666
XLOC_071383	AK5;ZZZ3;USP33	chr3	67082687	67357043	0.0	1.01836	1.33161	0.762923	0.793969	0.351769	0.456465	0.337387	0.296784
XLOC_071384	AK5;ZZZ3;USP33	chr3	67082687	67357043	0.0	0.85539	1.11854	0.384508	0.334096	0.591787	0.128415	0.142131	0.249471
XLOC_071385	AK5;ZZZ3;USP33	chr3	67082687	67357043	0.0	1.17284	1.53359	1.05437	1.21701	1.01145	0.523373	0.581171	1.19547
XLOC_071386	AK5;ZZZ3;USP33	chr3	67082687	67357043	0.0	2.38733	3.12119	0.715755	1.22191	0.815493	0.0	0.0	0.345916
XLOC_071387	AK5;ZZZ3;USP33	chr3	67082687	67357043	0.0	0.339104	0.886917	0.406508	0.593045	0.668384	0.483969	0.152053	0.628102
XLOC_071388	AK5;ZZZ3;USP33	chr3	67082687	67357043	0.622683	0.558822	0.97437	0.949008	0.243902	0.582374	0.170168	0.499754	0.871069
XLOC_071389	AK5;ZZZ3;USP33	chr3	67082687	67357043	0.0	0.0	0.0	0.395484	0.602733	0.685923	0.597724	0.330131	0.577813
XLOC_071390	AK5;ZZZ3;USP33	chr3	67082687	67357043	0.240634	0.360059	0.376694	0.388469	0.283638	0.200528	0.397383	0.242551	0.273888
XLOC_071391	AK5;ZZZ3;USP33	chr3	67082687	67357043	0.0	0.623722	0.0	0.280377	0.895683	0.540483	0.5656	0.624543	1.00149
XLOC_071392	AK5;ZZZ3;USP33	chr3	67082687	67357043	3.59541	0.0	0.0	0.214762	0.0934542	0.74459	1.18817	0.0	0.418261
XLOC_044484	AK8	chr11	102881227	102884346	1.87239	0.559868	0.721996	0.415718	0.216423	0.574473	0.678216	0.0934257	0.490464
XLOC_045121	AK8	chr11	102799255	102819816	24.3871	28.025	26.4557	31.4075	33.7035	31.4347	42.0477	46.5713	33.5617
XLOC_045122	AK8	chr11	102838354	102881206	30.8524	40.5721	36.7745	75.9513	70.9182	76.0234	82.9805	98.7563	61.7403
XLOC_045123	AK8	chr11	102905635	102925415	13.2415	18.6092	12.2211	30.3842	24.4773	27.2259	26.7792	35.4305	22.6474
XLOC_045124	AK8	chr11	102939211	102946292	6.70019	10.0206	6.98876	2.53588	1.74791	2.86029	1.48857	2.08811	1.69158
XLOC_047331	AK8	chr11	102799255	102819816	0.0	5.75293	7.51921	6.09351	10.0345	14.5025	6.99359	8.01939	10.753
XLOC_047332	AK8	chr11	102799255	102819816	12.7099	13.2614	29.7184	9.68446	8.13691	7.701	8.97884	10.7979	10.9354
XLOC_047333	AK8	chr11	102799255	102819816	12.416	2.46853	3.22735	7.77173	10.0995	6.3205	11.3412	5.57885	7.86625
XLOC_047334	AK8	chr11	102799255	102819816	3.14144	3.75543	4.91069	2.81347	5.37301	3.08301	5.90518	2.95946	3.4213
XLOC_047335	AK8	chr11	102799255	102819816	17.0514	8.48342	15.5288	10.6773	12.4685	10.1968	16.9082	11.1025	11.5854
XLOC_047336	AK8	chr11	102799255	102819816	6.25053	2.49336	2.85304	5.23064	4.94149	5.09133	4.23092	4.60433	4.78356
XLOC_047337	AK8	chr11	102799255	102819816	0.0	6.20649	3.24574	10.0494	11.1077	5.93217	9.26398	8.01325	6.1128

XLOC_047338	AK8	chr11	102799255	102819816	1.41968	2.97038	2.21953	4.57787	5.96709	4.69741	5.47941	4.79526	4.45526
XLOC_047339	AK8	chr11	102799255	102819816	3.50104	2.089	0.0	1.56532	5.36322	2.86064	3.33185	8.14517	5.15116
XLOC_047340	AK8	chr11	102799255	102819816	4.51596	2.24958	5.88323	8.35925	8.43055	7.31188	7.95954	7.46966	7.60881
XLOC_047342	AK8	chr11	102903274	102904120	0.0	0.628244	2.46462	0.658952	0.82012	0.326627	0.284812	0.524184	1.00873
XLOC_078543	AKAP3	chr5	105914945	105916187	2.00067	2.59338	1.56526	1.73376	2.40236	2.35563	2.30657	1.73858	2.21531
XLOC_078544	AKAP3	chr5	105917167	105918346	0.0	0.847146	1.66173	0.571226	0.332567	0.147076	0.515151	0.21287	0.371305
XLOC_089493	AKAP7	chr9	70207125	70399917	13.782	11.3297	15.1702	16.7838	15.0424	16.3237	16.8447	17.5477	19.739
XLOC_091206	AKAP7	chr9	70181781	70182328	0.0	0.0	0.0	0.49881	1.44044	0.766026	0.0	0.0	0.646533
XLOC_091207	AKAP7	chr9	70187551	70188145	0.0	0.0	0.0	0.445187	0.514951	2.05314	0.296192	0.820745	0.144332
XLOC_091208	AKAP7	chr9	70189748	70190409	0.0	0.0	0.0	1.41544	2.12424	1.0397	0.257811	0.0	0.500898
XLOC_091209	AKAP7	chr9	70194927	70195462	0.0	0.0	0.0	1.71546	2.82252	1.77771	0.681759	1.32448	1.00042
XLOC_072709	AKR1B1;AKR1B10	chr4	98923618	99122353	12.1255	13.2419	8.92963	33.6562	33.3126	35.1653	44.6006	45.1428	40.8631
XLOC_073253	AKR1B1;AKR1B10	chr4	98923618	99122353	37.0664	49.7705	40.7941	9.70753	11.9312	11.2762	13.7163	10.7009	10.6956
XLOC_074650	AKR1B1;AKR1B10	chr4	98923618	99122353	0.0	2.07366	4.06757	5.5153	5.42104	5.5756	6.52322	4.33445	4.92106
XLOC_074651	AKR1B1;AKR1B10	chr4	98923618	99122353	8.97417	2.6782	0.0	6.47514	6.69017	11.718	2.18219	8.32836	3.8531
XLOC_072725	AKR1D1	chr4	102570503	102605801	9.1099	4.84618	7.58176	0.211792	0.0264821	0.421499	0.0617982	0.0	0.147641
XLOC_074778	AKR1D1	chr4	102605859	102607253	1.75472	4.02456	1.83061	0.10488	0.0916705	0.182392	0.213274	0.0	0.0511463
XLOC_063268	AKT1S1	chr18	56675823	56676026	13.8915	0.0	0.0	20.3392	11.2547	15.2929	2.18858	3.78897	8.35405
XLOC_063269	AKT1S1	chr18	56677341	56677799	0.0	0.0	0.0	2.8002	0.929487	1.48437	2.12228	2.83557	1.46367
XLOC_062233	AKT2;TTC9B	chr18	49852347	49963119	21.6644	23.3484	23.6135	17.5988	20.3628	20.8882	23.9866	19.6786	24.5897
XLOC_058106	AKT3	chr16	34009302	34243958	5.55769	3.42429	6.19059	8.62719	6.27724	6.52475	5.04155	7.73834	10.2875
XLOC_058107	AKT3	chr16	34252226	34314229	2.34691	1.40397	3.52338	0.233215	0.117513	0.268032	0.071018	0.0783028	0.136684
XLOC_058859	AKT3	chr16	34009302	34243958	0.0	2.45036	0.0	6.65452	3.68369	3.30423	2.01006	7.65659	1.4113
XLOC_058108	AKT3;SDCCAG8	chr16	34314332	34573192	3.92855	2.21767	4.6401	0.211262	0.0614458	0.0815425	0.0	0.0786427	0.0
XLOC_058861	AKT3;SDCCAG8	chr16	34314332	34573192	0.796094	0.23811	0.622752	0.0	0.0	0.0	0.0	0.0	0.0695594
XLOC_058862	AKT3;SDCCAG8	chr16	34314332	34573192	1.2105	0.361911	0.0	0.0	0.0	0.0	0.0	0.0	0.0
XLOC_058864	AKT3;SDCCAG8	chr16	34314332	34573192	0.683273	0.613164	0.0	0.0	0.0	0.0	0.0	0.0	0.0
XLOC_058865	AKT3;SDCCAG8	chr16	34314332	34573192	0.622679	0.186273	0.487185	0.0	0.0	0.0	0.0	0.0	0.0

XLOC_058866	AKT3;SDCCAG8	chr16	34314332	34573192	0.0	0.415383	2.17269	0.0	0.0	0.0	0.0	0.0	0.0
XLOC_058867	AKT3;SDCCAG8	chr16	34314332	34573192	3.27926	0.0	0.0	0.0	0.0	0.0	0.0	0.0	0.0
XLOC_058868	AKT3;SDCCAG8	chr16	34314332	34573192	0.0	0.0	1.01799	0.0	0.0	0.0	0.0	0.0	0.113557
XLOC_086606	ALAD	chr8	104340654	104348611	13.3099	12.5043	10.1751	3.98224	4.78237	5.04439	4.40904	3.52638	2.79774
XLOC_064237	ALDH3A2;SLC47A2	chr19	34493672	34527461	10.5505	5.996	4.31262	3.27944	2.63235	3.02213	2.60739	1.25851	2.58568
XLOC_041698	ALDH6A1;LIN52	chr10	85829801	85888445	48.6447	45.0311	42.3411	95.4996	72.901	82.0375	83.0125	113.038	90.5292
XLOC_083503	ALDH7A1	chr7	28607257	28630878	1.08079	1.7248	1.97364	0.193841	0.22626	0.299994	0.131913	0.290079	0.346812
XLOC_070275	ALG14	chr3	48605774	48609106	25.5393	21.803	25.6587	10.4537	3.82152	6.20939	9.59	4.8693	3.96996
XLOC_086884	ALG2;SEC61B	chr8	64718085	64733737	85.7538	102.734	91.1557	72.2429	110.627	102.646	90.4572	55.2832	93.2415
XLOC_041716	ALKBH1;SNW1;SLIRP;ADCK1	chr10	89873514	90050084	0.0	4.38931	0.883038	0.303549	0.61672	0.584893	0.407618	0.225171	0.591285
XLOC_055807	ALKBH3	chr15	74834895	74856205	0.663656	0.49647	0.232988	0.0	0.0	0.0	0.0911708	0.0	0.0
XLOC_063953	ALOX15	chr19	27322172	27334302	0.0	0.0985716	0.0	0.0	0.0	0.0	0.0	0.0	0.0
XLOC_064830	ALOX15	chr19	27336143	27336929	0.0	0.344079	1.79976	0.0	0.0	0.0	0.0	0.0	0.0
XLOC_066565	ALPL	chr2	131856634	131860241	0.517521	0.0	0.0	0.649621	0.60871	0.592065	0.377887	0.939767	0.737197
XLOC_068635	ALPL	chr2	131853677	131854891	0.0	0.0	0.0	0.73642	0.696822	0.711118	0.373726	0.617631	0.359034
XLOC_068636	ALPL	chr2	131855389	131856444	0.0	0.0	0.0	0.794549	0.75644	0.585533	0.146316	0.403319	0.281638
XLOC_068081	ALS2CR11	chr2	90547231	90547969	2.49191	0.372498	0.0	0.111629	0.0	0.0	0.112197	0.372138	0.0
XLOC_068082	ALS2CR11	chr2	90550686	90551625	0.925475	0.276772	0.0	0.165887	0.144649	0.287979	0.0838322	0.0925017	0.242478
XLOC_068083	ALS2CR11	chr2	90554692	90555208	4.03527	0.602747	0.0	1.08372	0.156301	0.207863	0.179144	0.397993	0.351019
XLOC_068084	ALS2CR11	chr2	90570174	90570920	3.68705	0.73488	0.0	0.220226	0.0958116	0.127237	0.110706	0.0	0.214434
XLOC_066398	ALS2CR11;STRADB	chr2	90450667	90474992	14.9471	6.55708	6.00251	5.23388	5.64713	5.05743	4.54091	5.39826	5.22109
XLOC_065753	ALS2CR8	chr2	91711779	91721101	2.29163	2.76225	1.01285	16.1351	12.6038	12.4455	11.5169	12.1234	14.7031
XLOC_066408	ALS2CR8	chr2	91675918	91710725	11.3048	14.4047	11.3307	21.9001	18.3316	22.7749	15.5019	21.8739	16.2898
XLOC_068111	ALS2CR8	chr2	91673403	91675835	1.90782	3.61566	4.23063	3.6785	2.29733	2.74779	2.09736	3.202	2.14303
XLOC_068112	ALS2CR8	chr2	91721816	91722050	0.0	0.0	0.0	1.63803	3.38262	9.11706	0.735241	0.0	2.33861
XLOC_055521	AMBRA1	chr15	77356474	77360080	0.0	0.146949	0.0	5.64124	6.49378	5.10612	5.82315	7.05759	6.47996
XLOC_055847	AMBRA1	chr15	77403850	77411071	0.0	0.0	0.0	0.0530296	0.0927369	0.0	0.0	0.0	0.0517307
XLOC_057023	AMBRA1	chr15	77306870	77307112	16.6024	19.8146	12.9498	12.7077	7.44415	6.67854	9.47389	7.73474	9.27111

XLOC_057024	AMBRA1	chr15	77335848	77336926	0.785715	0.0	0.0	0.84514	1.10649	1.06038	0.285427	0.629341	1.09848
XLOC_057025	AMBRA1	chr15	77354538	77354825	0.0	0.0	0.0	1.98687	4.61516	0.561689	0.0	1.58146	1.43376
XLOC_057026	AMBRA1	chr15	77366650	77367354	0.0	0.0	0.0	0.948517	1.34027	1.23242	0.833059	1.05312	0.230836
XLOC_057027	AMBRA1	chr15	77368136	77368995	0.0	0.0	0.0	1.10872	0.724542	0.641225	0.466056	0.205837	0.270029
XLOC_057028	AMBRA1	chr15	77388255	77389798	0.0	0.0	0.0	0.0	0.0	0.0	0.0	0.104868	0.0
XLOC_055846	AMBRA1;CREB3L1;CHRM4;MDK	chr15	77084018	77306630	79.7306	91.0399	103.614	44.8325	44.1031	45.7955	46.4788	58.0701	42.5537
XLOC_057019	AMBRA1;CREB3L1;CHRM4;MDK	chr15	77084018	77306630	0.0	6.54813	17.1162	4.96822	2.43585	2.19572	12.2626	1.00795	4.70208
XLOC_057020	AMBRA1;CREB3L1;CHRM4;MDK	chr15	77084018	77306630	0.0	0.0	24.3381	9.40759	7.71191	3.12515	4.16871	2.87291	2.67565
XLOC_057021	AMBRA1;CREB3L1;CHRM4;MDK	chr15	77084018	77306630	6.19419	3.69732	9.66823	4.15522	2.85514	4.43854	2.6981	2.41312	4.0261
XLOC_057022	AMBRA1;CREB3L1;CHRM4;MDK	chr15	77084018	77306630	0.0	2.97239	0.0	5.35218	1.89076	2.02281	5.05954	2.38062	2.14924
XLOC_062809	AMFR	chr18	24290378	24290783	0.0	0.872249	2.2809	0.784167	0.449571	1.49714	1.27658	0.855407	1.77375
XLOC_076283	AMIGO2;FAM113B	chr5	33335334	33373461	30.7625	36.8217	29.5595	36.9025	35.5957	34.41	39.6883	43.7448	39.1913
XLOC_070243	AMPD1	chr3	28763529	28765367	6.12503	8.23975	10.3788	14.3618	14.9866	15.5558	16.7072	15.9997	21.3005
XLOC_073222	AMPH	chr4	82897882	83104133	0.709141	1.14311	1.5977	0.123264	0.079926	0.0377133	0.0621922	0.175114	0.188333
XLOC_073223	AMPH	chr4	83225337	83324308	3.54306	4.66204	3.92315	21.3701	20.4734	19.3648	28.0029	22.3187	22.55
XLOC_074482	AMPH	chr4	82897882	83104133	0.0	0.0	0.0	0.0	0.0	0.0615923	0.0540099	0.118943	0.0
XLOC_074483	AMPH	chr4	83217020	83217826	0.0	0.333478	0.0	0.599617	0.870257	1.15545	1.00673	0.556077	1.16804
XLOC_074484	AMPH	chr4	83219123	83219515	0.0	0.0	0.0	1.10332	2.13245	1.57856	2.95566	0.901078	2.1382
XLOC_074485	AMPH	chr4	83219983	83223642	1.23927	1.17445	0.970033	8.50301	10.358	8.65092	12.3819	10.539	11.8825
XLOC_074486	AMPH	chr4	83223738	83224838	2.30212	2.06574	1.80091	15.2704	15.7919	13.9427	20.5659	16.3687	17.9042
XLOC_074487	AMPH	chr4	83225337	83324308	1.57409	2.35224	0.0	4.37079	4.65027	6.34204	8.0317	6.40028	5.34945
XLOC_059969	ANAPC10;ABCE1;OTUD4	chr17	13109294	13425235	1.52826	0.228555	1.19554	0.206157	0.237471	0.555838	0.138846	0.0765241	0.0383737
XLOC_060599	ANAPC10;ABCE1;OTUD4	chr17	13109294	13425235	0.0	0.0	0.0	0.0	0.919287	0.0	0.508129	5.18876	1.57384
XLOC_059855	ANAPC5	chr17	56057865	56100578	20.3398	30.4056	28.4424	69.0066	55.406	63.6724	76.7733	104.826	56.9978

XLOC_059856	ANAPC5	chr17	56100628	56106908	0.298848	0.275669	0.924295	0.136513	0.052123	0.174304	0.0	5.49157E-4	0.0468981
XLOC_060245	ANAPC5	chr17	56057865	56100578	23.46	22.8545	17.9628	14.4573	11.4105	13.1529	11.2987	18.6008	13.1521
XLOC_060246	ANAPC5	chr17	56100628	56106908	0.637509	0.819037	1.37915E	0.339565	0.358643	0.222151	0.157342	0.241511	0.125764
XLOC_060247	ANAPC5	chr17	56106989	56109673	0.550618	0.823642	0.430838	0.296204	0.17264	0.0572452	0.10045	0.110578	0.144462
XLOC_061636	ANAPC5	chr17	56057865	56100578	3.23426	3.38572	3.16256	6.37781	6.70415	6.54628	5.79873	7.12206	5.86354
XLOC_061637	ANAPC5	chr17	56057865	56100578	4.11829	5.84956	10.4675	8.21145	5.78815	6.5099	5.02656	8.53031	8.17926
XLOC_061638	ANAPC5	chr17	56057865	56100578	6.40956	9.5541	14.9875	4.59779	8.6868	6.47152	4.79214	4.23386	7.71985
XLOC_060257	ANAPC7	chr17	56551192	56555330	1.89615	1.89134	3.2154	0.595164	1.04238	0.855499	0.434293	0.254599	0.829844
XLOC_061655	ANAPC7	chr17	56556892	56557476	5.08771	2.02717	3.97613	1.51869	2.5026	1.57559	0.454384	0.503757	1.92005
XLOC_061656	ANAPC7	chr17	56558611	56559719	5.3264	2.50355	4.76203	1.36414	1.30995	0.711046	1.45186	0.152415	0.997412
XLOC_053348	ANGPT1	chr14	59200570	59235870	9.89158	16.3907	14.3967	45.8029	59.4325	46.4608	52.4703	54.3793	72.9504
XLOC_053349	ANGPT1	chr14	59236013	59272831	6.7618	10.0973	9.6945	44.4144	56.5332	49.2523	43.9974	49.623	72.8108
XLOC_053653	ANGPT1	chr14	59321190	59440867	16.1617	25.0065	22.9339	36.9637	43.6523	36.553	60.0826	44.9972	74.8649
XLOC_054533	ANGPT1	chr14	59200570	59235870	17.9443	2.67823	0.0	4.04696	15.3867	10.8177	6.54697	8.32955	10.0181
XLOC_054534	ANGPT1	chr14	59200570	59235870	0.0	3.75286	4.36213	11.7461	8.14526	8.94479	11.9008	13.1699	11.3106
XLOC_054535	ANGPT1	chr14	59321190	59440867	0.0	0.385089	1.00712	0.115401	1.00372	0.533268	0.811329	0.512711	1.46054
XLOC_054536	ANGPT1	chr14	59321190	59440867	0.0	0.311861	3.26249	1.02804	1.38424	2.16195	0.377064	0.624543	1.82089
XLOC_054537	ANGPT1	chr14	59321190	59440867	1.25683	1.50292	2.94795	1.12597	1.17545	1.04075	0.226299	0.625537	1.09629
XLOC_054538	ANGPT1	chr14	59321190	59440867	1.01944	0.914501	0.0	0.36541	1.27367	0.317052	0.36872	0.814178	1.15699
XLOC_050592	ANGPT4	chr13	60601800	60763039	1.87504	1.48312	2.48293	16.9288	12.3483	11.7774	9.02325	17.6858	9.06304
XLOC_044420	ANGPTL2;RALGPS1	chr11	97840419	97843047	0.682628	0.407372	1.44476	0.611014	1.16875	0.907562	1.54202	1.31918	1.13719
XLOC_045051	ANGPTL2;RALGPS1	chr11	97853892	97879642	0.909439	2.04077	1.06751	0.6116	0.99878	0.993371	1.45431	0.685704	1.31278
XLOC_047193	ANGPTL2;RALGPS1	chr11	97836710	97839734	21.6979	14.7231	15.7982	40.3454	32.1351	37.4482	35.5003	59.7041	28.5388
XLOC_047194	ANGPTL2;RALGPS1	chr11	97850695	97851061	0.0	0.0	0.0	0.620149	1.06261	1.4169	0.600313	0.336255	3.00144
XLOC_047195	ANGPTL2;RALGPS1	chr11	97852725	97853293	0.0	0.526515	0.0	0.788893	1.64133	1.27271	0.785881	0.348649	1.841
XLOC_055379	ANGPTL5	chr15	7118111	7192605	24.7824	22.9316	26.019	24.4435	23.2616	21.9856	34.8601	29.6897	28.9637
XLOC_057910	ANGPTL7;EXOSC10;TARDBP;MASP2	chr16	43193392	43619064	24.9917	23.1392	23.4647	1.03895	1.78542	0.84105	1.57103	0.684233	1.88805

XLOC_058998	ANGPTL7;EXOSC10;TARDBP;MASP2	chr16	43193392	43619064	0.85445	1.27768	0.66833	0.30632	0.668087	0.443303	0.387483	0.256419	0.895671
XLOC_058999	ANGPTL7;EXOSC10;TARDBP;MASP2	chr16	43193392	43619064	0.82709	1.73152	1.29389	0.44478	1.09962	0.600854	0.375266	0.0827638	0.65031
XLOC_059000	ANGPTL7;EXOSC10;TARDBP;MASP2	chr16	43193392	43619064	0.0	0.536963	1.40428	0.321818	0.976206	0.556167	0.0	0.0	1.56448
XLOC_059001	ANGPTL7;EXOSC10;TARDBP;MASP2	chr16	43193392	43619064	0.0	2.05921	1.26725	0.326716	0.698938	0.421266	0.555409	0.366509	1.20439
XLOC_059002	ANGPTL7;EXOSC10;TARDBP;MASP2	chr16	43193392	43619064	0.0	1.0423	2.27174	0.156183	0.591573	0.482876	0.211751	0.408002	1.16789
XLOC_059003	ANGPTL7;EXOSC10;TARDBP;MASP2	chr16	43193392	43619064	0.0	2.65193	4.16121	1.11257	0.688855	0.732556	0.949779	0.526741	1.23633
XLOC_059004	ANGPTL7;EXOSC10;TARDBP;MASP2	chr16	43193392	43619064	4.29563	0.321111	0.0	0.673613	0.419115	1.11285	1.45515	0.321423	0.937383
XLOC_059005	ANGPTL7;EXOSC10;TARDBP;MASP2	chr16	43193392	43619064	0.0	0.0	1.054	0.120772	0.419996	0.139467	0.969378	0.134053	1.1756
XLOC_059006	ANGPTL7;EXOSC10;TARDBP;MASP2	chr16	43193392	43619064	0.0	0.974903	1.69981	0.194772	0.254466	0.225227	0.0981546	0.108412	0.284589
XLOC_059007	ANGPTL7;EXOSC10;TARDBP;MASP2	chr16	43193392	43619064	5.13254	4.09341	4.68381	0.230011	0.267549	0.088765	0.232761	0.0855729	0.448367
XLOC_059008	ANGPTL7;EXOSC10;TARDBP;MASP2	chr16	43193392	43619064	0.0	12.7236	4.75308	0.544715	0.46804	0.935399	0.26549	0.0	0.791841

XLOC_059009	ANGPTL7;EXOSC10;TARDBP;MASP2	chr16	43193392	43619064	9.28842	6.24145	0.0	0.415622	0.897266	0.477564	0.0	0.0	2.01775
XLOC_059010	ANGPTL7;EXOSC10;TARDBP;MASP2	chr16	43193392	43619064	2.84486	4.25173	4.44779	0.382238	0.221431	0.735401	0.255344	0.565229	0.372011
XLOC_059011	ANGPTL7;EXOSC10;TARDBP;MASP2	chr16	43193392	43619064	4.14806	1.77274	2.31824	0.212508	0.603633	0.246365	0.0540108	0.0594713	0.155451
XLOC_059012	ANGPTL7;EXOSC10;TARDBP;MASP2	chr16	43193392	43619064	5.20997	4.67048	14.9286	0.15551	0.134836	0.537671	0.774919	0.0	0.302455
XLOC_059013	ANGPTL7;EXOSC10;TARDBP;MASP2	chr16	43193392	43619064	1.44725	0.0	1.69828	0.129731	0.169923	0.0	0.131586	0.145009	0.189735
XLOC_059014	ANGPTL7;EXOSC10;TARDBP;MASP2	chr16	43193392	43619064	3.97937	2.38073	3.11331	0.0594562	0.207758	0.206711	0.241479	0.0665034	0.869672
XLOC_059015	ANGPTL7;EXOSC10;TARDBP;MASP2	chr16	43193392	43619064	3.91474	3.70831	5.6151	0.175474	0.357703	0.338947	0.118803	0.0654322	0.570391
XLOC_059016	ANGPTL7;EXOSC10;TARDBP;MASP2	chr16	43193392	43619064	2.35408	0.703819	10.1239	0.0	0.183592	0.243785	0.106125	0.0	0.616208
XLOC_059017	ANGPTL7;EXOSC10;TARDBP;MASP2	chr16	43193392	43619064	3.72579	1.67132	13.1133	0.166955	0.145574	0.0	0.0843642	0.186181	0.0
XLOC_064203	ANKRD13B;MIR2334;CORO6	chr19	21393547	21472590	0.981468	0.220285	0.384104	0.220062	0.154244	0.076675	0.0675277	0.123655	0.0859295
XLOC_081601	ANKRD17	chr6	90021389	90023014	0.0	0.0	0.0	0.751216	0.347838	0.615073	0.18001	0.49522	0.387986
XLOC_081602	ANKRD17	chr6	90023648	90024897	0.0	0.0	0.0	1.60385	0.570812	0.481886	0.844407	0.863759	0.521323
XLOC_081603	ANKRD17	chr6	90035933	90037339	0.0	0.0	0.0	0.415489	0.226984	0.481719	0.633739	0.116294	0.101312
XLOC_050749	ANKRD26	chr13	17882070	17944117	83.5915	85.7196	72.2499	55.017	57.2341	54.213	51.4682	54.8033	61.0994

XLOC_051260	ANKRD26	chr13	17944593	17945503	0.0	1.14972	0.0	1.29206	1.12635	0.598023	0.783052	0.288074	0.671442
XLOC_051261	ANKRD26	chr13	17949946	17950815	1.01653	3.03977	0.795008	3.82603	2.77838	2.95059	2.66585	2.74022	3.63872
XLOC_062220	ANKRD27	chr18	43310443	43315295	0.0	0.435436	1.13878	0.0	0.0	0.0	0.130675	0.0	0.0
XLOC_063071	ANKRD27	chr18	43315454	43316810	1.20691	0.180526	0.472154	0.108203	0.0	0.0	0.0	0.0605549	0.0
XLOC_063072	ANKRD27	chr18	43321069	43322564	11.3555	7.60338	5.92355	4.46034	3.00962	4.27283	4.48927	8.74285	5.81686
XLOC_062219	ANKRD27;PDCD5	chr18	43207709	43297521	2.55802	0.0	1.00001	0.0	0.0	0.132371	0.0	0.0	0.0
XLOC_062428	ANKRD27;RGS9BP;NUDT19;SLC7A9	chr18	43322697	43438293	11.7235	5.95007	6.68615	6.30962	2.86803	4.54055	4.44723	8.18801	6.15203
XLOC_063977	ANKRD40	chr19	36554758	36620976	6.68944	7.05182	6.63455	0.884601	1.25492	1.08057	1.11062	0.556404	1.28886
XLOC_053676	ANKRD46	chr14	65986792	66009232	1.54329	1.84712	1.67533	1.28252	2.37914	1.52684	1.79566	1.05875	4.25845
XLOC_059716	ANKRD50	chr17	33524093	33553787	7.77761	6.76184	8.94932	1.75774	2.8253	2.76903	3.41948	2.3475	2.98335
XLOC_061244	ANKRD50	chr17	33524093	33553787	3.64848	1.09024	0.0	0.16334	0.283082	0.752661	0.325085	0.180344	0.158798
XLOC_055463	ANO3	chr15	57912169	57970060	13.459	12.2734	9.2703	16.7684	18.1117	17.7906	18.3837	15.395	15.9561
XLOC_055771	ANO3	chr15	57869535	57892561	29.2999	23.6135	26.1081	13.5533	12.5748	13.6194	15.4715	13.0875	13.7607
XLOC_055773	ANO3	chr15	58094937	58097453	18.8092	17.6793	11.2092	23.5207	28.495	25.274	30.4416	25.9712	31.2928
XLOC_055774	ANO3	chr15	58098405	58122522	33.5574	43.7673	33.7431	73.9822	81.2882	79.6685	83.8794	74.7039	96.9554
XLOC_055775	ANO3	chr15	58125711	58135225	40.4101	50.394	46.1654	68.4539	78.7686	77.3454	86.493	69.303	90.9771
XLOC_056643	ANO3	chr15	57868296	57869199	2.91051	1.1605	2.27635	1.30418	0.909479	0.603605	1.0537	0.678411	0.931871
XLOC_056644	ANO3	chr15	57869535	57892561	6.50705	1.29568	3.38874	2.32957	0.503452	0.669715	1.34434	0.42705	1.69708
XLOC_056645	ANO3	chr15	58017997	58018653	0.0	1.30107	0.0	0.0	0.11291	0.0	0.0	0.0	0.0
XLOC_056646	ANO3	chr15	58020575	58021322	0.0	0.366817	0.959342	0.549632	0.286952	0.127023	0.0	0.12218	0.107036
XLOC_056647	ANO3	chr15	58086465	58087227	2.39297	0.0	0.0	0.321606	0.373199	0.371682	0.10784	0.357578	1.1483
XLOC_056648	ANO3	chr15	58091190	58091496	0.0	0.0	3.78328	0.8683	1.47374	1.47775	0.411207	0.927991	2.09293
XLOC_056649	ANO3	chr15	58093031	58093851	1.09173	1.30575	0.853746	1.07609	1.53365	1.58368	1.18306	0.871171	1.42931
XLOC_056650	ANO3	chr15	58094038	58094154	0.0	0.0	467.503	121.845	0.0	0.0	39.6635	0.0	99.2588
XLOC_056651	ANO3	chr15	58094283	58094679	21.2259	28.961	33.1325	77.0263	88.5635	84.7711	86.205	82.7414	105.663
XLOC_056652	ANO3	chr15	58097583	58098219	0.0	0.451794	1.18156	2.5724	3.87992	2.65568	3.65728	2.55008	3.29329
XLOC_055772	ANO3;MUC15	chr15	58022257	58076470	41.9054	53.2101	44.2363	59.2322	44.0183	47.1541	50.0024	68.5107	46.6023
XLOC_076342	ANO4	chr5	64970099	65030134	3.2016	6.17743	6.51276	2.00699	3.27534	1.92952	2.06734	0.644335	4.03077
XLOC_076343	ANO4	chr5	65030194	65031367	0.0	0.0	0.0	0.0	0.0	0.0	9.66189	0.0	0.0
XLOC_076344	ANO4	chr5	65034192	65041186	3.0496	2.73579	0.795008	1.00206	1.58765	1.47529	1.83851	0.405959	1.86373

XLOC_076345	ANO4	chr5	65107827	65168197	7.70861	5.88735	6.38876	1.50888	2.03512	1.40218	2.35153	1.25379	2.0183
XLOC_076346	ANO4	chr5	65199711	65260653	5.55134	5.20492	4.6545	6.34087	6.87331	5.77263	5.70114	5.98143	7.29079
XLOC_076347	ANO4	chr5	65348251	65384046	0.415224	0.186397	0.487522	0.0744832	0.0652753	0.086523	0.0762368	0.167483	0.0181794
XLOC_076857	ANO4	chr5	65261606	65336123	22.7166	41.3411	31.3082	79.062	83.7804	83.2955	86.7215	83.8316	85.6271
XLOC_078035	ANO4	chr5	65041295	65042013	1.29042	3.08619	2.01783	0.346821	1.20659	1.06834	1.27717	0.128403	1.12548
XLOC_078036	ANO4	chr5	65199711	65260653	0.0	1.61457	0.0	2.90284	1.45842	1.66448	0.948854	1.58704	2.11174
XLOC_044155	ANTXR1;GFPT1	chr11	67455483	67685979	18.2246	19.1456	16.3387	0.490252	0.318413	0.512497	0.53997	0.646515	0.313423
XLOC_046423	ANTXR1;GFPT1	chr11	67455483	67685979	0.933962	0.558628	0.0	0.0	0.0729732	0.193721	0.0845768	0.0	0.0
XLOC_046424	ANTXR1;GFPT1	chr11	67455483	67685979	0.0	0.0	0.0	0.0	0.17187	0.0	0.19658	0.218633	0.193163
XLOC_046425	ANTXR1;GFPT1	chr11	67455483	67685979	0.0	0.602978	0.525628	0.0	0.105222	0.0698035	0.0611448	0.0	0.0
XLOC_046426	ANTXR1;GFPT1	chr11	67455483	67685979	4.29941	0.0	0.0	0.0	0.166347	0.0	0.0	0.0	0.0
XLOC_046427	ANTXR1;GFPT1	chr11	67455483	67685979	0.0	0.0	0.0	0.0	0.0	0.0	0.0	0.0	0.108132
XLOC_079839	ANXA3	chr6	95117868	95129834	6.56544	9.35687	6.60773	13.4182	11.0876	13.1639	13.2008	16.688	14.0602
XLOC_044156	ANXA4;GMCL1	chr11	67966444	68204328	6.50688	6.87438	5.59993	12.8939	11.9886	11.1012	10.7229	13.9136	14.4226
XLOC_079584	ANXA5	chr6	3498380	3603465	10.6087	12.9488	11.8096	22.8531	19.4724	20.1313	22.7983	28.677	25.6544
XLOC_083039	ANXA6	chr7	64392616	64412118	2.9899	9.29203	0.817978	14.0956	16.3611	14.052	10.5929	14.9414	19.6198
XLOC_083676	ANXA6	chr7	64372535	64374261	4.167	3.74003	3.6682	2.47521	4.61475	4.00806	3.70798	3.34858	4.28243
XLOC_083677	ANXA6	chr7	64376578	64389458	30.7001	37.5407	27.1579	44.9227	42.1308	46.8431	61.2405	68.444	64.656
XLOC_083678	ANXA6	chr7	64389574	64392456	22.1652	21.3264	18.8433	35.4967	29.2874	32.9784	49.4614	51.5086	49.5662
XLOC_064273	AOC3;AOC2	chr19	43499893	43524631	23.9677	19.6841	14.336	125.074	102.701	109.914	124.097	152.271	120.018
XLOC_060297	AP1B1	chr17	70765845	70779850	1.10337	1.39826	3.45141	1.42884	1.15021	0.58253	1.09646	1.16439	0.898262
XLOC_061856	AP1B1	chr17	70747313	70749671	1.31465	0.885129	1.02891	1.8274	1.93563	2.0188	0.933212	0.827312	1.06436
XLOC_061857	AP1B1	chr17	70750548	70751764	0.0	0.408776	1.60368	1.47006	1.12352	1.63249	0.683885	0.47948	0.47781
XLOC_061858	AP1B1	chr17	70753706	70754625	1.89957	1.42018	0.742856	1.61728	0.964724	1.18199	0.429977	0.284709	0.165883
XLOC_061859	AP1B1	chr17	70755190	70755899	1.31148	0.392062	1.02536	0.822438	1.53259	0.542818	0.471821	0.26093	0.228753
XLOC_062424	AP1G1	chr18	39410140	39490631	10.0202	10.2368	9.29442	3.03865	2.8273	2.80403	1.66262	1.60562	2.20355
XLOC_082779	AP1M2	chr7	16309766	16318257	5.97934	12.1494	13.9874	21.9517	24.192	24.7558	10.6762	22.8943	14.1872
XLOC_084262	AP1M2	chr7	16308548	16309459	1.91974	0.574096	1.50147	2.58069	1.64981	2.38894	1.04271	1.15079	1.34111
XLOC_066459	AP1S3	chr2	112630419	112686650	1.20726	1.9108	1.45248	4.43628	3.40701	5.45302	5.37117	7.28129	2.79272

XLOC_041448	AP3S1;AQPEP	chr10	4712686	4857106	5.16167	3.47197	3.02675	0.0	0.0	0.0	0.0	0.0	0.0
XLOC_086393	APBA1	chr8	45885488	45919522	2.59689	7.3032	4.47048	0.605384	0.325774	0.864127	0.0	0.208689	0.454271
XLOC_062276	APEG3	chr18	64265403	64272254	0.0	0.143159	0.375958	0.0860078	0.0376155	0.299777	0.0438055	0.0482008	0.0
XLOC_042691	APH1B	chr10	46812889	46813188	0.0	0.0	0.0	3.64249	3.08623	5.67605	0.859472	3.39812	1.31605
XLOC_042692	APH1B	chr10	46813454	46813943	0.0	0.0	0.0	2.5389	1.68812	2.47024	0.193162	1.28868	0.758775
XLOC_056836	APIP	chr15	66222646	66223674	0.0	0.0	0.0	0.0744703	0.0649789	0.172457	0.0	0.0	0.0
XLOC_044149	APLF	chr11	66911945	66928063	0.0	0.0	0.0	2.30923	2.32102	2.35868	2.08564	1.89071	1.60425
XLOC_046416	APLF	chr11	66928196	66930058	0.0	0.0	0.0	1.48385	2.82991	2.51643	2.32767	2.51765	1.78198
XLOC_092382	APLN;ZDHHC9;<br>XPNPEP2;SASH3	chrX	13518981	13714699	5.51201	12.0728	7.00503	7.8861	7.91001	9.93142	8.85219	13.7219	7.20325
XLOC_055568	APLNR	chr15	81711424	81796457	69.2649	84.8692	72.3856	43.2679	37.6431	43.2194	41.034	47.5665	40.1098
XLOC_055886	APLNR	chr15	81711424	81796457	47.3103	60.9697	50.8796	123.442	90.2546	108.642	101.84	123.027	88.6328
XLOC_055724	APOA5;APOA4;<br>BUD13;ZNF259;<br>APOC3;APOA1	chr15	27829966	28025388	23.6564	23.9471	21.9387	5.25553	2.57549	3.83357	2.31711	1.89628	1.7557
XLOC_056247	APOA5;APOA4;<br>BUD13;ZNF259;<br>APOC3;APOA1	chr15	27829966	28025388	5.01927	0.0	0.0	0.898122	0.761393	0.0	0.84855	0.0	0.43275
XLOC_056248	APOA5;APOA4;<br>BUD13;ZNF259;<br>APOC3;APOA1	chr15	27829966	28025388	8.73123	1.73767	2.27176	1.04136	0.671747	1.19298	0.763025	0.0	0.0
XLOC_056249	APOA5;APOA4;<br>BUD13;ZNF259;<br>APOC3;APOA1	chr15	27829966	28025388	6.30192	3.76165	7.37694	0.0	0.0	0.322458	0.0	0.0	0.0
XLOC_056250	APOA5;APOA4;<br>BUD13;ZNF259;<br>APOC3;APOA1	chr15	27829966	28025388	0.0	1.04729	0.0	0.0	0.0	0.241832	0.0	0.116363	0.0
XLOC_056251	APOA5;APOA4;<br>BUD13;ZNF259;<br>APOC3;APOA1	chr15	27829966	28025388	4.41415	0.0	0.0	0.0	0.0	0.0	0.0	0.423868	0.381091
XLOC_056252	APOA5;APOA4;<br>BUD13;ZNF259;<br>APOC3;APOA1	chr15	27829966	28025388	1.53729	0.459555	8.41132	0.137688	0.0	0.317696	0.0	0.0	0.0

XLOC_056253	APOA5;APOA4;<br>BUD13;ZNF259;<br>APOC3;APOA1	chr15	27829966	28025388	2.14956	3.21051	3.35824	0.577212	0.166377	0.0	0.0	0.0	0.0
XLOC_056254	APOA5;APOA4;<br>BUD13;ZNF259;<br>APOC3;APOA1	chr15	27829966	28025388	0.0	0.0	7.05336	0.0	0.0	0.459744	0.0	0.0	0.0
XLOC_056255	APOA5;APOA4;<br>BUD13;ZNF259;<br>APOC3;APOA1	chr15	27829966	28025388	4.44099	1.32448	13.8501	0.397159	0.0	0.0	0.0	0.0	0.0
XLOC_056256	APOA5;APOA4;<br>BUD13;ZNF259;<br>APOC3;APOA1	chr15	27829966	28025388	2.62541	3.13564	0.0	0.469792	0.202454	0.269488	0.230584	0.257102	0.227889
XLOC_056257	APOA5;APOA4;<br>BUD13;ZNF259;<br>APOC3;APOA1	chr15	27829966	28025388	3.6782	2.19477	0.0	0.32891	0.0	0.375349	0.0	0.0	0.31819
XLOC_056258	APOA5;APOA4;<br>BUD13;ZNF259;<br>APOC3;APOA1	chr15	27829966	28025388	0.0	0.0	0.0	0.355914	0.0	0.0	0.0	0.0	0.0
XLOC_056259	APOA5;APOA4;<br>BUD13;ZNF259;<br>APOC3;APOA1	chr15	27829966	28025388	4.58264	2.73944	7.1641	0.0	0.356421	0.157852	0.136846	0.303139	0.26623
XLOC_056260	APOA5;APOA4;<br>BUD13;ZNF259;<br>APOC3;APOA1	chr15	27829966	28025388	4.7509	1.41894	1.85516	0.425155	0.183535	0.244194	0.0	0.0	0.206372
XLOC_056261	APOA5;APOA4;<br>BUD13;ZNF259;<br>APOC3;APOA1	chr15	27829966	28025388	0.0	5.48336	7.16637	0.0	0.684348	0.0	0.0	0.0	0.0
XLOC_056262	APOA5;APOA4;<br>BUD13;ZNF259;<br>APOC3;APOA1	chr15	27829966	28025388	11.7997	2.11448	5.52925	0.63358	0.18236	0.0	0.0	0.463637	0.410073
XLOC_056263	APOA5;APOA4;<br>BUD13;ZNF259;<br>APOC3;APOA1	chr15	27829966	28025388	2.39355	2.86366	1.87237	0.0	0.124841	0.165643	0.28977	0.0	0.27885

XLOC_056264	APOA5;APOA4;BUD13;ZNF259;APOC3;APOA1	chr15	27829966	28025388	2.74489	4.09746	0.0	0.0	0.422908	0.0	0.0	0.268427	0.238163
XLOC_056265	APOA5;APOA4;BUD13;ZNF259;APOC3;APOA1	chr15	27829966	28025388	2.59343	2.71534	3.55084	0.232498	0.101572	0.269463	0.0	0.0650336	0.113363
XLOC_056266	APOA5;APOA4;BUD13;ZNF259;APOC3;APOA1	chr15	27829966	28025388	2.83076	3.3803	4.41951	0.506459	0.0	0.0	0.247725	0.0	0.0
XLOC_076885	APOL3	chr5	75004479	75044438	21.5317	27.7311	26.6657	4.10655	4.73208	4.31457	4.87315	5.38254	5.03451
XLOC_037764	APP	chr1	9798189	9900838	56.1063	64.062	54.8205	1.73433	1.54478	2.13453	1.61285	1.68316	1.6111
XLOC_037765	APP	chr1	9911674	9937212	5.21939	3.08081	1.41357	3.34753	1.98767	2.01594	3.71505	2.14209	2.92794
XLOC_038262	APP	chr1	9667242	9700211	2.64238	3.00019	2.54655	6.61586	7.03124	7.25505	5.16993	3.55566	6.2817
XLOC_038263	APP	chr1	9722618	9748543	27.2673	23.994	20.8534	7.66433	9.28052	9.02492	10.4441	9.62706	7.33016
XLOC_038843	APP	chr1	9667242	9700211	0.0	0.0	0.0	4.25756	2.41214	3.58406	5.05115	2.16948	3.18401
XLOC_038844	APP	chr1	9667242	9700211	0.0	0.750159	0.0	1.23643	2.54244	2.85704	2.48508	1.8734	2.4077
XLOC_038845	APP	chr1	9719935	9722473	7.59528	5.00023	5.23122	1.60752	2.4339	2.81547	2.75618	2.96816	2.52639
XLOC_038846	APP	chr1	9782104	9782345	16.7826	5.00769	26.182	84.6499	54.5388	89.4378	78.6054	116.437	98.0388
XLOC_038847	APP	chr1	9798189	9900838	0.0	0.881523	2.30544	0.264165	0.229476	0.0	0.0	0.0	0.0
XLOC_038848	APP	chr1	9798189	9900838	0.0	1.2436	0.0	0.0	0.161189	0.0	0.0	0.205148	0.0
XLOC_038849	APP	chr1	9798189	9900838	7.15433	6.41541	8.94834	19.4815	20.157	24.8549	22.6	23.7359	29.1872
XLOC_038850	APP	chr1	9911674	9937212	4.44099	1.32423	3.46253	2.78012	0.675998	0.903044	1.51418	0.426279	4.21722
XLOC_076788	ARF3	chr5	31035953	31039414	5.59661	5.71603	4.86252	3.62032	2.75201	2.75037	4.61305	5.26437	2.98159
XLOC_064023	ARF4	chr19	46152522	46286687	14.5544	16.1113	13.4068	12.9553	10.2351	11.3898	12.2425	15.4186	9.6673
XLOC_053541	ARFGEF1	chr14	33376629	33447197	38.5333	50.1958	38.7598	20.6632	25.9749	21.9744	25.2981	20.606	35.6094
XLOC_054171	ARFGEF1	chr14	33370656	33371199	0.0	0.560602	0.0	0.0	0.145516	0.193471	0.0	0.0	0.163299
XLOC_054172	ARFGEF1	chr14	33371433	33372375	1.84387	1.37857	1.44219	0.578388	0.504349	0.573768	0.417585	0.368608	0.724662
XLOC_054173	ARFGEF1	chr14	33373280	33374657	0.593062	2.48387	1.39208	0.478534	0.975894	0.986266	0.486483	0.238079	1.3483
XLOC_054174	ARFGEF1	chr14	33375385	33376430	2.4448	1.94992	1.91243	0.365225	1.08362	1.18419	0.887642	0.652509	1.28161
XLOC_050646	ARFGEF2;CSE1L;ZNFX1;DDX27	chr13	77708695	78166289	8.28489	20.8898	23.1345	9.80148	8.78707	8.65444	12.0448	9.67595	10.776

XLOC_052558	ARFGEF2;CSE1L;ZNFX1;DDX27	chr13	77708695	78166289	3.26456	0.0	0.0	0.583975	0.751455	0.333871	0.567128	0.0	0.848284
XLOC_050559	ARFRP1;ZGPAT;RTEL1;RTEL1;STMN3;TNFRSF6B	chr13	54503749	54551589	18.1199	16.1121	14.3771	12.939	11.3628	11.7399	13.6624	14.1726	9.68702
XLOC_051866	ARFRP1;ZGPAT;RTEL1;RTEL1;STMN3;TNFRSF6B	chr13	54503749	54551589	4.04662	8.4483	9.46741	3.98112	3.39681	5.35839	5.55248	4.29022	2.09871
XLOC_089979	ARG1	chr9	70424946	70503304	14.813	12.903	14.63	11.3479	9.47251	11.0493	12.0186	16.1146	9.49076
XLOC_091211	ARG1	chr9	70503458	70503688	9.51565	5.68313	7.42804	22.353	16.2914	18.151	9.98806	15.8539	9.80645
XLOC_042040	ARG2	chr10	80069848	80106102	19.2828	32.7751	26.5363	17.2752	20.0146	23.641	22.2133	20.6614	16.5222
XLOC_043049	ARG2	chr10	80069848	80106102	1.15857	3.9859	8.61185	0.882913	1.08952	1.14408	1.10904	0.930352	0.861148
XLOC_059958	ARHGAP10	chr17	10204816	10318897	132.218	144.164	119.199	99.6965	96.3466	104.655	132.005	138.705	128.496
XLOC_060507	ARHGAP10	chr17	10192350	10192940	0.0	0.999546	0.0	0.299532	1.16921	0.172661	1.64366	0.993818	3.35016
XLOC_060508	ARHGAP10	chr17	10193323	10194047	1.27675	1.14508	0.0	0.457536	0.795958	0.264279	0.804335	0.508256	2.3386
XLOC_060509	ARHGAP10	chr17	10194828	10196105	1.29208	0.579773	2.52726	0.579169	1.31562	0.805511	1.35296	0.583149	2.31565
XLOC_060510	ARHGAP10	chr17	10196386	10196970	3.39181	3.54755	0.0	1.21495	1.05372	1.05039	2.27192	1.84711	2.95392
XLOC_060511	ARHGAP10	chr17	10197160	10197484	4.33549	3.87851	3.38048	2.32625	5.28302	4.41006	9.25359	5.41565	10.1081
XLOC_060512	ARHGAP10	chr17	10197864	10199408	2.60813	1.56059	0.816331	1.35631	3.02639	2.16964	2.99918	1.30991	4.74483
XLOC_060513	ARHGAP10	chr17	10200056	10200218	34.6592	0.0	27.6921	3.33582	15.0069	17.4176	15.7053	6.0832	30.1139
XLOC_060514	ARHGAP10	chr17	10200322	10200461	0.0	27.2366	0.0	26.6537	55.6246	26.5383	102.719	22.3361	61.4911
XLOC_060515	ARHGAP10	chr17	10200785	10201328	5.6289	2.24241	2.93216	2.85587	2.91033	2.32165	3.34046	2.22449	3.42928
XLOC_060516	ARHGAP10	chr17	10201512	10202124	1.59141	1.90247	4.97541	3.27814	6.18517	3.61647	6.40802	4.57542	5.12999
XLOC_060517	ARHGAP10	chr17	10202263	10204725	5.96279	2.44168	3.92995	3.26475	3.59842	4.31321	4.91677	3.60295	6.04332
XLOC_060518	ARHGAP10	chr17	10204816	10318897	42.7158	32.1014	35.2072	14.0681	10.1765	14.3489	19.5771	30.0322	16.0178
XLOC_060519	ARHGAP10	chr17	10204816	10318897	52.2552	51.0568	43.8454	17.5841	10.5285	16.8843	19.2708	28.7935	17.4532
XLOC_060520	ARHGAP10	chr17	10204816	10318897	77.956	51.7104	45.0738	19.8867	12.6639	19.8257	23.727	30.1568	24.0376
XLOC_060521	ARHGAP10	chr17	10320213	10321008	1.13454	0.678453	1.77438	0.304976	0.442569	0.587626	0.307123	1.35729	1.18811
XLOC_060522	ARHGAP10	chr17	10433932	10434793	0.0	0.0	0.0	0.644916	0.321111	1.06569	0.929513	0.513147	0.897547
XLOC_060523	ARHGAP10	chr17	10441687	10442724	0.0	0.0	0.0	0.737089	0.192955	0.341403	0.447797	0.41149	0.0718463
XLOC_065651	ARHGAP15	chr2	53108342	53133909	7.96279	6.57135	5.08001	6.90053	6.67004	7.95132	8.51557	8.65909	8.10422

XLOC_065652	ARHGAP15	chr2	53137082	53220276	13.3321	11.2568	13.3428	16.3718	16.4475	17.6474	20.7037	25.046	21.532
XLOC_066295	ARHGAP15	chr2	53137082	53220276	52.5015	78.1199	47.6638	111.266	115.864	120.843	141.324	175.128	126.266
XLOC_067533	ARHGAP15	chr2	53108342	53133909	7.60047	0.756637	1.97836	0.906758	1.17281	2.86226	2.00586	0.993346	1.75984
XLOC_067534	ARHGAP15	chr2	53134007	53136967	2.58062	1.31285	2.22185	2.52275	2.59534	2.66098	4.21295	2.88672	3.54708
XLOC_067535	ARHGAP15	chr2	53137082	53220276	2.56134	2.29857	5.51079	1.95175	3.56101	4.59084	4.37332	2.24788	3.97472
XLOC_067536	ARHGAP15	chr2	53137082	53220276	5.99763	1.78776	4.67427	3.76085	4.52454	3.03125	2.50352	3.97376	3.61459
XLOC_067537	ARHGAP15	chr2	53137082	53220276	1.96303	1.17284	4.60081	2.81164	2.43401	2.62976	3.4894	2.51841	1.87891
XLOC_067538	ARHGAP15	chr2	53137082	53220276	5.19449	6.20362	6.08361	4.1827	2.60366	4.79932	2.73898	3.81577	4.05868
XLOC_067539	ARHGAP15	chr2	53137082	53220276	0.341898	0.409192	0.802686	1.47159	1.90582	1.45915	2.41058	1.75524	1.52602
XLOC_067540	ARHGAP15	chr2	53137082	53220276	0.913497	0.956567	1.78706	0.941931	1.57642	1.33033	1.62769	0.964205	1.71822
XLOC_067541	ARHGAP15	chr2	53137082	53220276	1.15272	0.689253	4.50659	1.3426	2.06796	1.552	1.97563	1.60839	1.81065
XLOC_067542	ARHGAP15	chr2	53137082	53220276	0.0	1.03047	1.3475	2.31599	2.4099	3.73707	4.61755	3.07192	4.05422
XLOC_067543	ARHGAP15	chr2	53220386	53221255	0.0	1.21591	0.795008	1.18425	0.793823	1.36992	0.459629	0.10149	0.798742
XLOC_067544	ARHGAP15	chr2	53695702	53696859	18.8138	21.4281	20.3793	19.3948	21.7501	21.8684	19.6719	18.5612	21.9452
XLOC_091177	ARHGAP18	chr9	68729412	68730123	0.0	0.390647	0.0	0.0	0.0	0.0	0.235079	0.0	0.0
XLOC_091178	ARHGAP18	chr9	68739365	68740341	1.76727	0.0	0.691169	0.0791975	0.138156	0.0916772	0.0	0.0883653	0.0771839
XLOC_091179	ARHGAP18	chr9	68765914	68766406	0.0	0.0	0.0	0.0	0.167324	0.0	0.0	0.212903	0.0
XLOC_091180	ARHGAP18	chr9	68767549	68768355	1.1153	0.333478	0.0	0.0999362	0.0	0.115545	0.100673	0.111215	0.0973366
XLOC_091181	ARHGAP18	chr9	68769439	68770213	0.0	0.0	0.0	0.0	0.0915007	0.0	0.0	0.116906	0.102368
XLOC_091182	ARHGAP18	chr9	68780285	68781167	0.0	0.597055	0.0	0.0	0.155938	0.0	0.0	0.0996908	0.0871624
XLOC_091183	ARHGAP18	chr9	68790414	68791070	0.0	0.867382	1.13422	0.389895	0.11291	0.300009	0.0	0.144092	0.379422
XLOC_091184	ARHGAP18	chr9	68791928	68793172	0.665664	0.398248	0.0	0.11935	0.260648	0.276625	0.0605889	0.0	0.0581907
XLOC_056209	ARHGAP20	chr15	20920370	20920641	0.0	1.8785	0.0	2.25926	0.949411	0.636435	0.0	1.1901	0.0
XLOC_044152	ARHGAP25	chr11	67081573	67083391	0.0	0.703819	0.0	0.0	0.0	0.0	0.0	0.0	0.0
XLOC_044153	ARHGAP25	chr11	67083758	67091896	0.415291	0.0	0.0	0.446874	0.162941	0.17285	0.417744	0.0835879	0.181685
XLOC_046421	ARHGAP25	chr11	67066244	67066928	4.12128	2.87459	1.07398	4.55331	3.85096	3.12607	2.3449	5.0526	3.23408
XLOC_046422	ARHGAP25	chr11	67067093	67068052	0.0	1.61923	0.0	0.889634	1.41065	0.561665	1.06308	0.721751	0.945788
XLOC_044154	ARHGAP25;ANTXR1;BMP10;GKN1	chr11	67092065	67371591	1.00379	1.90599	1.72288	6.07404	2.80899	4.6812	4.17335	5.86877	3.47207
XLOC_083027	ARHGAP26	chr7	55794163	55830973	16.3409	21.8276	26.2352	17.2592	16.29	16.7148	18.7015	21.7292	16.7964
XLOC_083028	ARHGAP26	chr7	55842039	55930750	28.5349	27.974	26.424	39.6451	39.8298	39.8239	42.3068	41.2602	44.4137
XLOC_083029	ARHGAP26	chr7	55975715	56019520	0.0	0.0	0.57016	0.160466	0.325322	0.198801	0.119728	0.127757	0.227038

XLOC_083030	ARHGAP26	chr7	56050131	56204742	53.3866	42.0766	46.5737	13.4881	11.3723	11.9603	12.8859	13.1933	10.7751
XLOC_083666	ARHGAP26	chr7	55931257	55962982	21.9802	25.0146	30.5404	15.7915	14.4947	16.3688	19.4408	19.7012	16.4309
XLOC_083667	ARHGAP26	chr7	55975715	56019520	1.66021	2.48375	2.27364	1.53168	1.259	1.24192	0.776465	0.977776	0.540924
XLOC_084918	ARHGAP26	chr7	55794163	55830973	3.29416	4.91497	12.8524	2.65145	2.02159	3.70528	4.28986	3.20082	1.71187
XLOC_084919	ARHGAP26	chr7	55794163	55830973	2.77648	0.82888	2.16773	2.98062	3.42106	1.99318	3.40487	2.71386	0.963555
XLOC_084920	ARHGAP26	chr7	55831239	55831667	2.67391	14.3697	8.35052	5.98066	7.62493	5.21333	8.44714	8.8967	6.72976
XLOC_084921	ARHGAP26	chr7	55931257	55962982	0.0	6.91463	3.61593	4.14843	3.87845	5.18287	8.27828	4.44343	6.00346
XLOC_084922	ARHGAP26	chr7	55963170	55963730	1.79704	0.0	0.0	0.482728	0.278916	0.185389	0.640793	0.177714	0.469339
XLOC_084923	ARHGAP26	chr7	55973485	55974347	1.02664	0.0	0.0	0.184	0.160329	0.319256	0.278466	0.307457	0.268883
XLOC_084924	ARHGAP26	chr7	55974908	55975445	0.0	0.0	0.0	0.682573	0.0	0.196494	0.0	0.376475	0.0
XLOC_084925	ARHGAP26	chr7	55975715	56019520	0.0	2.78141	2.90956	0.833492	0.577637	0.383995	0.49733	0.367957	1.13433
XLOC_037887	ARHGAP31	chr1	64691088	64718395	13.1848	18.1565	14.9616	3.99128	4.62296	4.53529	6.55511	3.69739	4.88873
XLOC_037888	ARHGAP31	chr1	64718582	64723957	15.2022	9.70677	13.3854	3.59989	4.6434	4.71344	5.03129	4.49074	5.47064
XLOC_038389	ARHGAP31	chr1	64679863	64690664	8.95306	8.79444	15.0001	14.0941	11.8606	14.1637	10.5895	12.474	13.0523
XLOC_038390	ARHGAP31	chr1	64734409	64737753	8.78353	11.2854	7.98245	21.3401	19.1541	18.474	21.9508	31.4382	20.1973
XLOC_039557	ARHGAP31	chr1	64691088	64718395	3.464	2.81305	3.09785	0.709929	0.62091	0.720529	1.12964	0.49724	0.649294
XLOC_039558	ARHGAP31	chr1	64691088	64718395	1.34314	1.6061	1.05011	0.360974	0.418457	0.416878	0.724398	0.0	0.351376
XLOC_039559	ARHGAP31	chr1	64691088	64718395	3.61203	5.93609	0.0	0.485137	0.560584	0.931547	0.804861	0.535747	0.628894
XLOC_039560	ARHGAP31	chr1	64691088	64718395	6.14209	5.77573	5.14985	1.02283	0.963764	1.68882	1.08274	0.882277	0.844442
XLOC_039561	ARHGAP31	chr1	64691088	64718395	0.918423	3.02134	5.02849	0.411563	0.861332	0.857403	1.08166	0.826244	0.962549
XLOC_039562	ARHGAP31	chr1	64691088	64718395	1.16188	3.47395	2.72565	0.416424	1.26878	0.481363	0.943096	0.0	0.811084
XLOC_039563	ARHGAP31	chr1	64691088	64718395	2.76934	2.76069	2.16607	0.661859	0.144286	0.191511	1.00346	0.0	0.564345
XLOC_039564	ARHGAP31	chr1	64724294	64725791	15.118	12.438	14.7868	4.84096	4.5712	6.06288	7.8323	6.99475	6.09152
XLOC_039565	ARHGAP31	chr1	64726000	64726580	3.42392	2.04634	0.0	1.68635	2.39302	1.41364	1.83421	1.35581	3.72714
XLOC_039566	ARHGAP31	chr1	64726728	64727150	2.73452	0.816372	0.0	5.62667	2.73849	4.76861	3.8353	2.67409	5.9318
XLOC_039567	ARHGAP31	chr1	64727280	64728756	2.74265	0.984624	0.429205	1.57377	1.93487	1.88195	2.30167	1.32194	2.63844
XLOC_039568	ARHGAP31	chr1	64729623	64734320	7.96579	8.91632	6.98377	15.6188	14.9077	13.2997	19.9965	25.4144	18.7526
XLOC_039569	ARHGAP31	chr1	64788997	64793836	3.08967	3.23643	2.53949	1.78748	1.70072	1.57787	1.67513	2.12	1.29886
XLOC_038391	ARHGAP31;TIMMDC1;TMEM39A;POGLUT1;ADPRH;CD80;PLA1A	chr1	64794013	64956801	5.1554	5.20484	6.78194	2.42649	2.47159	2.22478	2.36927	3.65793	2.34175

XLOC_065905	ARHGEF10L	chr2	135648905	135651789	0.0	0.0	0.0	14.9919	9.25351	8.76776	7.78429	15.8943	15.0698
XLOC_068664	ARHGEF10L	chr2	135647688	135647928	0.0	0.0	0.0	7.64268	3.16776	5.9701	2.07144	11.844	3.64372
XLOC_068665	ARHGEF10L	chr2	135651902	135652270	3.43455	0.0	0.0	13.8214	7.63279	10.8789	8.03189	18.992	13.9746
XLOC_068666	ARHGEF10L	chr2	135652475	135653987	0.0	0.0	0.417803	3.87778	2.59527	2.83143	1.90007	3.48571	2.66185
XLOC_068667	ARHGEF10L	chr2	135655131	135656447	0.0	0.0	0.976892	1.56711	0.978117	0.843373	1.25107	1.81626	0.654987
XLOC_068668	ARHGEF10L	chr2	135656889	135657701	0.0	0.660848	0.0	1.68336	0.862349	1.48841	1.89561	0.771467	1.83251
XLOC_068669	ARHGEF10L	chr2	135658362	135659164	0.0	0.335546	0.0	1.40778	0.96317	0.93006	0.810281	1.56655	1.46907
XLOC_068670	ARHGEF10L	chr2	135659671	135663886	2.49538	1.92039	4.88327	1.16705	0.966935	1.18874	0.965944	0.952953	0.905336
XLOC_068671	ARHGEF10L	chr2	135664243	135665337	2.31687	3.00294	3.02073	1.17684	0.966843	1.20271	0.982171	1.54672	1.07977
XLOC_068672	ARHGEF10L	chr2	135667160	135667665	6.24453	3.10895	4.87823	1.86326	1.61168	1.50051	0.553805	0.820551	1.81024
XLOC_068673	ARHGEF10L	chr2	135669684	135671653	2.7947	2.38918	2.49954	0.680219	0.532658	1.16331	0.693881	0.682787	0.558957
XLOC_055421	ARHGEF12	chr15	31568780	31590161	5.36992	5.3565	6.36845	1.15323	1.54791	1.85102	1.26481	1.88674	2.39254
XLOC_055422	ARHGEF12	chr15	31602124	31618967	8.13398	3.55383	2.15863	1.83663	1.26363	1.46724	1.39715	1.37412	1.35542
XLOC_055726	ARHGEF12	chr15	31455896	31531016	23.5406	35.1692	26.9286	15.104	14.906	15.2523	19.3584	19.982	17.7603
XLOC_055727	ARHGEF12	chr15	31590893	31601878	200.434	264.283	239.948	603.781	503.134	569.988	574.271	655.531	599.047
XLOC_056276	ARHGEF12	chr15	31455896	31531016	2.48335	0.37122	0.0	0.778721	0.580733	0.257075	0.559103	0.494506	0.433266
XLOC_056277	ARHGEF12	chr15	31455896	31531016	1.49286	0.892412	1.16695	0.133715	0.232269	0.154299	0.936762	0.0	0.130109
XLOC_056278	ARHGEF12	chr15	31455896	31531016	0.0	4.66139	9.75136	0.279391	0.959728	1.2789	0.543972	1.52053	0.812089
XLOC_056279	ARHGEF12	chr15	31455896	31531016	0.0	7.91622	6.90066	0.9884	1.53755	0.68182	1.75884	1.52133	1.34395
XLOC_056280	ARHGEF12	chr15	31455896	31531016	7.33669	8.04272	10.5171	1.64333	2.00194	1.89895	1.87267	1.33951	2.77356
XLOC_056281	ARHGEF12	chr15	31531353	31531845	0.0	0.645908	1.68914	0.774207	1.00395	0.66774	1.34048	0.212903	0.188006
XLOC_056282	ARHGEF12	chr15	31533367	31533826	0.0	0.0	0.0	0.0	0.370585	0.0	0.846219	0.0	0.0
XLOC_056283	ARHGEF12	chr15	31568780	31590161	0.0	5.41893	0.0	0.0	0.676529	0.0	0.0	1.68405	1.55908
XLOC_069842	ARHGEF2;RXFP4	chr3	14863483	14872348	3.09847	0.0	0.0	0.463021	0.04049	0.375903	0.377078	0.207504	0.0903362
XLOC_092227	ARHGEF9	chrX	101794619	101798545	14.6558	35.8216	26.3881	6.16147	10.6903	7.0482	10.0345	7.96874	13.9866
XLOC_092228	ARHGEF9	chrX	102005063	102084703	3.08856	2.69011	2.41028	1.10803	0.87407	0.837946	0.475708	0.946869	1.19502
XLOC_092545	ARHGEF9	chrX	101790442	101793254	60.5799	57.1442	54.3993	10.9041	18.4023	10.5551	16.0145	13.8313	20.7505
XLOC_093365	ARHGEF9	chrX	101793420	101794341	43.5756	43.0604	43.7138	8.23504	12.0647	10.512	13.7239	12.5894	15.7178
XLOC_093366	ARHGEF9	chrX	101851649	101852590	1.84622	2.76066	0.0	0.0	0.0	0.0	0.0	0.0	0.0
XLOC_093367	ARHGEF9	chrX	101902032	101902552	0.0	0.0	1.55892	0.0	0.0	0.0	0.0	0.0	0.0
XLOC_093368	ARHGEF9	chrX	101957817	101958641	0.0	1.94688	0.0	0.0	0.0	0.0	0.196017	0.0	0.0
XLOC_093369	ARHGEF9	chrX	102005063	102084703	0.0	0.281085	2.20542	0.252708	0.0734438	0.0	0.0	0.0	0.0

XLOC_093370	ARHGEF9	chrX	102005063	102084703	3.48565	0.347392	2.72563	0.104106	0.0	0.120337	0.0	0.0	0.101385
XLOC_093371	ARHGEF9	chrX	102005063	102084703	0.0	0.461424	0.0	0.0	0.0	0.0	0.0	0.0	0.0
XLOC_065861	ARID1A	chr2	127008599	127065190	9.91475	11.6906	10.5746	7.65395	8.26325	6.44262	7.22607	8.17617	7.81791
XLOC_068527	ARID1A	chr2	127066540	127067766	4.06127	3.84705	6.88429	5.09708	5.98933	4.78152	4.55816	5.76822	5.85769
XLOC_083620	ARID3A	chr7	45082912	45092215	22.288	15.9214	16.2507	46.8988	55.0263	50.5834	40.02	38.6332	50.4938
XLOC_083621	ARID3A	chr7	45092928	45106577	16.1968	4.577	3.87292	4.02606	4.16305	5.04316	3.0503	3.84142	5.38969
XLOC_041586	ARID4A	chr10	70847380	70854327	0.0	0.829275	0.0	4.847	2.4582	2.17467	5.67878	9.46136	7.46284
XLOC_041983	ARID4A	chr10	70830708	70845155	0.0	0.0	0.68781	1.97031	0.916049	1.58248	1.80478	3.84436	2.53471
XLOC_042920	ARID4A	chr10	70830708	70845155	0.0	0.0	0.636759	4.15889	3.18355	1.68981	4.3594	6.2734	4.83622
XLOC_042921	ARID4A	chr10	70847380	70854327	0.0	0.0	0.0	1.16062	0.900884	0.522919	2.55079	2.0902	2.32605
XLOC_042922	ARID4A	chr10	70847380	70854327	0.0	0.68908	0.0	3.5103	3.38827	1.89824	5.70619	8.3905	5.81438
XLOC_042923	ARID4A	chr10	70847380	70854327	0.0	0.354213	0.0	2.01684	0.923925	0.858796	1.28164	3.42311	2.79098
XLOC_042924	ARID4A	chr10	70855677	70856289	0.0	0.0	0.0	0.570111	0.247407	0.493155	0.427202	1.7355	1.38648
XLOC_041587	ARID4A;TIMM9	chr10	70872037	71159636	0.0	0.491548	0.738711	0.59698	0.477538	0.49021	0.600474	1.54395	2.24183
XLOC_041984	ARID4A;TIMM9	chr10	70872037	71159636	14.7751	14.9176	15.5042	0.959876	0.685248	0.905007	0.746465	0.938885	0.724584
XLOC_042925	ARID4A;TIMM9	chr10	70872037	71159636	3.13155	2.24898	2.4509	0.0280835	0.0491869	0.0326052	0.172147	0.126147	0.0548205
XLOC_054127	ARMC1	chr14	31696482	31697854	2.97752	0.890745	0.931874	0.64067	1.49317	1.54736	0.922653	0.657393	1.14556
XLOC_054128	ARMC1	chr14	31698000	31699477	9.31794	5.73929	5.14656	5.2583	9.62409	9.34567	8.44979	7.26518	10.0664
XLOC_053533	ARMC1;MTFR1	chr14	31699689	31830435	33.6667	23.5345	24.2188	28.1585	35.9623	32.6129	38.6197	38.4565	30.9352
XLOC_054129	ARMC1;MTFR1	chr14	31699689	31830435	2.66406	3.18155	0.0	3.81356	2.2587	3.00731	1.87059	1.30378	4.8555
XLOC_054130	ARMC1;MTFR1	chr14	31699689	31830435	0.0	1.18715	3.10423	0.711829	2.73466	1.62221	1.36682	0.767882	3.78425
XLOC_054131	ARMC1;MTFR1	chr14	31699689	31830435	10.4982	4.39036	24.6031	2.06739	4.38879	2.81062	3.35069	1.862	4.19955
XLOC_054132	ARMC1;MTFR1	chr14	31699689	31830435	6.6174	2.37387	7.24312	2.60842	3.60842	3.28647	2.38018	1.57973	2.88545
XLOC_054133	ARMC1;MTFR1	chr14	31699689	31830435	4.81412	6.46939	3.75961	1.9386	2.97436	4.94789	1.91008	1.65418	3.76371
XLOC_054134	ARMC1;MTFR1	chr14	31699689	31830435	5.93179	5.57572	5.3028	2.27857	4.17466	3.69337	2.9211	2.54341	4.14559
XLOC_054135	ARMC1;MTFR1	chr14	31699689	31830435	6.92987	5.17701	4.06173	6.9812	5.78419	5.72157	4.79335	2.57202	4.97838
XLOC_054136	ARMC1;MTFR1	chr14	31699689	31830435	5.48346	6.88635	6.86102	4.81528	5.56314	5.56792	3.46599	2.84406	4.97741

XLOC_050775	ARMC3;PIP4K2A	chr13	24010640	24281010	13.7546	20.1614	16.881	7.78944	5.88714	7.1386	6.65382	6.37525	5.37071
XLOC_050810	ARMC4	chr13	36867618	37047408	14.6528	2.29692	8.10917	0.14602	0.0371634	0.290745	0.0216841	0.0	0.0
XLOC_038042	ARMC8	chr1	131909871	131931371	70.322	49.8795	40.1126	206.831	190.904	203.283	248.889	249.014	209.091
XLOC_038043	ARMC8	chr1	131962995	131983034	7.08198	4.7556	5.17358	2.25111	0.946129	1.94243	0.692563	1.37949	1.34611
XLOC_040242	ARMC8	chr1	131887057	131887377	0.0	0.0	0.0	0.397159	0.337993	0.903044	1.51418	4.26274	1.15015
XLOC_040243	ARMC8	chr1	131892549	131892712	0.0	0.0	0.0	35.521	2.42728	3.37759	7.62448	11.8123	8.75765
XLOC_040244	ARMC8	chr1	131931582	131936482	23.1794	19.8078	15.2797	93.7505	93.3092	98.4939	106.156	101.13	100.597
XLOC_040245	ARMC8	chr1	131936859	131937328	0.0	2.08048	5.44071	4.77965	5.38359	5.01442	5.94635	3.1946	3.83368
XLOC_040246	ARMC8	chr1	131937833	131938304	0.0	2.06724	0.0	2.27137	3.74491	2.3728	2.24172	2.49447	2.60645
XLOC_040247	ARMC8	chr1	131938607	131939351	0.0	0.737383	1.92849	2.09927	1.15362	1.91501	1.99935	0.736776	2.04404
XLOC_040248	ARMC8	chr1	131940611	131942011	1.16417	1.39309	0.910886	1.5656	2.05267	2.23867	1.64498	0.467406	1.8833
XLOC_040249	ARMC8	chr1	131942530	131943106	0.0	0.0	1.35067	1.85721	2.41558	1.78376	1.23413	0.684247	2.10703
XLOC_040250	ARMC8	chr1	131945084	131945874	0.0	0.341905	1.78839	1.84431	1.2489	1.06603	0.618999	0.455958	0.997888
XLOC_065819	ARMC9	chr2	119798442	119818105	12.2874	19.5279	9.44272	30.6389	25.3397	26.0984	27.5933	32.7118	28.5401
XLOC_065820	ARMC9	chr2	119872521	119926638	52.9907	50.4637	48.5752	66.7146	68.7309	71.8926	80.5254	68.8345	77.8471
XLOC_065821	ARMC9	chr2	119931379	119965421	45.237	54.2008	45.7669	70.5033	70.0647	75.7447	77.891	76.2576	76.3754
XLOC_066485	ARMC9	chr2	119763829	119788258	8.70514	10.6091	11.0468	48.0907	40.1567	42.0952	53.5067	67.7789	44.1618
XLOC_068382	ARMC9	chr2	119857933	119858508	0.0	0.0	0.0	1.2411	1.07613	0.536397	1.23699	1.20024	2.41376
XLOC_068383	ARMC9	chr2	119872521	119926638	0.0	1.45656	0.0	3.49644	5.19146	2.97593	4.13756	6.07028	2.10696
XLOC_068384	ARMC9	chr2	119927735	119928087	0.0	2.21695	2.89847	2.32591	3.12602	3.79112	2.24246	1.43748	3.2141
XLOC_068385	ARMC9	chr2	119928552	119928977	0.0	2.42173	2.11097	2.17712	2.29175	3.05154	1.42288	0.529015	3.28492
XLOC_068386	ARMC9	chr2	119929131	119929383	7.49037	0.0	0.0	0.0	2.24576	1.50883	6.76661	2.10433	4.50669
XLOC_068387	ARMC9	chr2	119931037	119931290	0.0	6.63635	5.78316	9.99707	7.78582	5.97743	7.92462	2.77977	2.5503
XLOC_068388	ARMC9	chr2	119931379	119965421	5.34745	6.37639	4.16792	9.09553	7.69268	11.3762	13.0501	8.6521	6.91149
XLOC_068389	ARMC9	chr2	119931379	119965421	4.38762	1.30835	0.0	2.356	3.34162	2.23101	0.374298	2.10688	4.92473
XLOC_069846	ARNT	chr3	19957079	19977209	6.2364	6.53113	8.54101	0.139809	0.0	0.162255	0.0356603	0.0784321	0.102323
XLOC_070709	ARNT	chr3	19933996	19934765	3.54836	0.0	2.7746	24.3745	19.0018	21.0676	17.5944	24.1588	23.4267
XLOC_070710	ARNT	chr3	19936027	19936417	6.22077	5.56972	14.5644	247.036	174.894	197.711	150.61	250.151	239.087
XLOC_069847	ARNT;CTSK;CTSS	chr3	19978860	20035518	10.5407	6.88073	4.49907	0.128881	0.112732	0.0498342	0.131284	0.0963043	0.12574
XLOC_070238	ARNT;CTSK;CTSS	chr3	19978860	20035518	6.24235	6.745	5.94423	7.01605	5.36084	5.93224	4.81128	4.04924	4.28249
XLOC_055755	ARNTL	chr15	39841962	39857216	1.60912	0.0	0.0	1.44111	1.50074	0.830977	0.287876	0.159494	1.542

XLOC_055756	ARNTL	chr15	39870639	39943763	8.76113	7.20454	5.35914	2.36483	2.59639	2.1056	3.39562	3.07594	2.82915
XLOC_056433	ARNTL	chr15	39870639	39943763	2.33502	1.39629	2.73881	0.523046	0.182123	0.604578	0.315855	0.116348	0.101876
XLOC_056434	ARNTL	chr15	39870639	39943763	7.36827	2.93372	1.91798	1.31867	0.758446	1.51416	2.81271	0.24097	1.06659
XLOC_056435	ARNTL	chr15	39870639	39943763	1.12048	2.01016	0.0	0.903601	0.874265	1.04471	1.01131	0.111725	0.391151
XLOC_044387	ARPC5L	chr11	95858850	95860169	2.83817	4.92294	4.53805	9.29807	5.00764	9.99024	9.03444	9.63714	7.14058
XLOC_047075	ARPC5L	chr11	95861451	95861853	0.0	0.0	0.0	4.49796	2.50226	2.12125	3.61598	1.44255	1.02587
XLOC_047076	ARPC5L	chr11	95862815	95863034	0.0	3.27406	0.0	13.9098	7.30755	5.4893	6.13088	5.03414	6.58283
XLOC_047077	ARPC5L	chr11	95863862	95864025	0.0	0.0	0.0	9.68756	14.5637	10.1328	12.7075	5.90616	52.5459
XLOC_047078	ARPC5L	chr11	95864211	95864405	0.0	0.0	12.7498	7.47919	10.718	1.6241	6.33708	4.39765	5.58397
XLOC_044388	ARPC5L;GOLGA1	chr11	95865930	95914954	0.606839	1.18051E-	1.40974	1.96956	1.34389	1.93103	1.23046	1.35392	1.28659
XLOC_045024	ARPC5L;GOLGA1	chr11	95865930	95914954	1.14293	1.28615	0.0	1.44522	4.78694	2.2468	1.6207	0.922337	4.56464
XLOC_056624	ARRB1	chr15	55255859	55256131	0.0	5.5883	9.74032	12.8808	15.5381	16.4113	13.519	14.7578	24.1961
XLOC_056625	ARRB1	chr15	55256238	55256582	0.0	4.62245	3.02172	4.50367	7.1034	7.89922	11.6605	12.7179	14.0684
XLOC_056626	ARRB1	chr15	55256986	55257132	0.0	38.6239	50.5665	62.2677	40.0344	37.8266	32.3873	26.8599	65.6253
XLOC_056627	ARRB1	chr15	55257465	55257866	0.0	4.43261	16.2275	9.82975	16.4441	16.7342	10.8901	12.4551	12.1029
XLOC_056628	ARRB1	chr15	55258017	55258164	0.0	18.4742	48.3669	59.4727	42.6367	30.1747	44.3585	30.9062	26.1685
XLOC_056629	ARRB1	chr15	55258272	55258557	0.0	6.71663	0.0	6.55646	9.79248	9.12133	8.50075	12.3004	16.4945
XLOC_056630	ARRB1	chr15	55258646	55259311	9.97638	5.11212	3.3424	6.38317	9.20725	10.6105	9.46578	10.0522	11.1818
XLOC_056631	ARRB1	chr15	55265818	55266438	0.0	0.0	0.0	1.54073	1.45908	1.13098	1.40008	0.465287	0.408791
XLOC_076520	ARSA;ACR;RABL2B	chr5	120143073	120255209	14.5965	16.4531	12.0014	25.2241	18.8283	20.566	22.558	34.0787	21.4676
XLOC_041842	ARSB	chr10	9757875	9901211	8.5057	9.84136	9.93584	2.27968	3.31765	2.34941	2.20574	1.5706	2.83996
XLOC_042314	ARSB	chr10	9757875	9901211	0.0	1.65433	5.04769	0.578389	1.0087	1.14754	0.4176	0.645068	0.8857
XLOC_042315	ARSB	chr10	9757875	9901211	3.63218	1.62942	4.26146	0.651066	0.851666	0.659367	0.658253	0.36312	0.951708
XLOC_042316	ARSB	chr10	9757875	9901211	1.4196	1.69742	0.0	0.127165	0.552501	0.293589	0.50973	0.141041	0.247517
XLOC_042317	ARSB	chr10	9757875	9901211	1.49354	2.68045	0.0	0.535524	0.525987	1.08561	0.610834	0.149612	0.456833
XLOC_081700	ART3	chr6	92700703	92701864	0.0	0.431166	1.12768	0.0	0.0	0.074851	0.0	0.0	0.125983
XLOC_056571	ART5	chr15	52259377	52259514	0.0	60.8588	79.7296	49.8119	96.219	19.7333	92.7931	57.9192	51.4704
XLOC_065881	ASAP3;TCEA3	chr2	130012335	130049111	0.0	0.640669	0.375169	1.59609	0.449453	0.891894	3.16702	6.95714	2.44062
XLOC_050525	ASB13	chr13	43398303	43480605	19.4642	34.9802	16.0898	37.7809	28.6912	36.5461	27.8179	39.7474	33.3838
XLOC_050823	ASB13	chr13	43398303	43480605	14.0964	18.5498	12.5268	8.85294	10.1017	9.70525	11.9349	10.9212	9.19938
XLOC_051725	ASB13	chr13	43398303	43480605	0.0	4.19843	0.0	1.25832	1.61674	1.43712	3.6514	1.36382	2.74016

XLOC_051726	ASB13	chr13	43398303	43480605	1.86664	3.3464	2.91712	3.17541	2.31655	2.88725	3.65629	1.47551	2.11201
XLOC_051727	ASB13	chr13	43398303	43480605	4.07654	3.6583	0.531574	1.40087	1.48968	2.47029	1.8548	1.63474	2.19745
XLOC_051728	ASB13	chr13	43398303	43480605	0.0	2.73531	0.0	1.53692	1.42739	1.53982	0.619064	0.569948	1.39704
XLOC_051729	ASB13	chr13	43398303	43480605	2.29996	2.74772	7.18533	0.411666	2.48896	1.65575	1.42233	1.80855	1.59891
XLOC_051730	ASB13	chr13	43398303	43480605	0.0	3.12876	5.4548	0.781293	2.84512	2.88127	2.95854	1.89946	2.73501
XLOC_051731	ASB13	chr13	43398303	43480605	11.5951	4.32659	6.78818	1.5558	4.01465	2.67364	4.81428	1.9805	3.0166
XLOC_051732	ASB13	chr13	43398303	43480605	4.34867	1.30103	1.70136	0.77979	0.980091	1.35639	1.23977	1.03716	1.47374
XLOC_051733	ASB13	chr13	43398303	43480605	0.0	2.65573	4.16739	0.557102	0.416549	0.92125	0.885455	0.443973	1.08586
XLOC_051734	ASB13	chr13	43398303	43480605	0.991462	2.37193	1.55086	0.444252	0.697019	0.822351	0.627949	0.198032	0.519402
XLOC_051735	ASB13	chr13	43398303	43480605	4.12302	3.49444	4.83848	0.739216	1.72178	1.28486	1.81316	0.826612	1.86204
XLOC_051736	ASB13	chr13	43398303	43480605	3.60054	1.88446	2.11221	0.403373	0.985077	0.840413	1.22368	0.449984	1.10068
XLOC_051737	ASB13	chr13	43398303	43480605	3.99107	2.55857	2.6767	1.4313	1.87678	1.06684	0.9878	1.0875	1.6952
XLOC_073233	ASB15	chr4	88718168	88722525	29.5333	29.2578	29.927	36.8773	27.2839	34.8368	30.6	32.3439	34.5831
XLOC_074566	ASB15	chr4	88714992	88715614	0.0	2.32696	1.21712	0.418391	0.0	0.643519	0.278844	0.154441	0.27136
XLOC_092269	ASB9;ASB11;PIR;FIGF	chrX	135302323	135550128	5.34269	8.56487	3.62708	3.41887	0.167773	0.482611	1.61987	0.32481	0.685773
XLOC_092270	ASB9;ASB11;PIR;FIGF	chrX	135302323	135550128	0.0	4.22485	0.0	0.140673	0.0	0.0	0.0	0.155757	0.136852
XLOC_093597	ASB9;ASB11;PIR;FIGF	chrX	135302323	135550128	6.61381	6.21615	2.21693	1.27014	0.0	0.391951	0.427769	0.472064	0.330039
XLOC_089881	ASCC3	chr9	49504705	49799041	4.03102	2.62267	4.48493	4.92575	4.0966	3.50809	11.7754	9.19636	10.4258
XLOC_073087	ASNS	chr4	15051505	15111232	0.0	0.0	0.0	1.15834	0.202403	0.402755	0.176473	0.259177	0.112959
XLOC_086546	ASPN;CENPP	chr8	85492482	85527601	8.18933	9.00103	9.84493	25.6942	47.0784	31.5311	41.6391	18.8579	33.5827
XLOC_045087	ASS1	chr11	100780694	100850547	8.87306	7.54292	7.33911	1.80243	2.40137	1.85172	2.06145	1.56268	2.39797
XLOC_044240	ATAD2B	chr11	75193821	75266123	3.43884	1.41476	1.00916	0.462537	0.640812	0.626131	0.0392977	0.259363	0.225655
XLOC_044909	ATAD2B	chr11	75177589	75190537	6.69588	8.11422	8.54882	17.0383	13.5405	12.981	15.8163	16.469	14.1834
XLOC_046570	ATAD2B	chr11	75175457	75176474	0.841482	3.27176	2.6329	1.96099	1.64512	2.35782	1.60319	2.5258	2.42593
XLOC_046571	ATAD2B	chr11	75176817	75177456	9.01298	6.28576	11.7421	10.091	7.36151	9.47036	6.19317	12.8215	9.81866
XLOC_044238	ATAD2B;UBXN2A;C11H2orf84;LOC100125925;SF3B14;PFN4;TP53I3;FKBP1B	chr11	74897211	75169164	23.9337	21.6927	19.2392	47.6723	41.013	45.4388	56.1326	58.0584	44.0248

XLOC_044239	ATAD2B;UBXN2A;C11H2orf84;LOC100125925;SF3B14;PFN4;TP53I3;FKBP1B	chr11	74897211	75169164	31.2294	37.6629	36.4191	17.6225	16.2827	15.8378	18.5666	16.4328	15.4508
XLOC_044907	ATAD2B;UBXN2A;C11H2orf84;LOC100125925;SF3B14;PFN4;TP53I3;FKBP1B	chr11	74897211	75169164	3.81785	4.13654	4.98347	3.76447	3.33093	2.54259	4.02041	2.27503	3.48878
XLOC_044908	ATAD2B;UBXN2A;C11H2orf84;LOC100125925;SF3B14;PFN4;TP53I3;FKBP1B	chr11	74897211	75169164	0.0	0.0	0.0	0.0	0.108038	0.0	0.129713	0.0	0.0
XLOC_046567	ATAD2B;UBXN2A;C11H2orf84;LOC100125925;SF3B14;PFN4;TP53I3;FKBP1B	chr11	74897211	75169164	6.46494	1.92799	5.03872	2.89818	0.973309	1.30764	0.536607	2.43896	2.22486
XLOC_046568	ATAD2B;UBXN2A;C11H2orf84;LOC100125925;SF3B14;PFN4;TP53I3;FKBP1B	chr11	74897211	75169164	0.0	2.44819	2.56084	0.880306	0.381902	0.508141	0.878812	0.811655	0.999025
XLOC_046569	ATAD2B;UBXN2A;C11H2orf84;LOC100125925;SF3B14;PFN4;TP53I3;FKBP1B	chr11	74897211	75169164	4.97125	2.97139	5.18026	1.18717	1.93107	1.02719	0.74048	0.984915	1.29907
XLOC_076780	ATF1;DIP2B	chr5	29230861	29378616	7.05427	11.2102	10.878	5.97917	7.77818	5.29621	7.99477	3.19	10.0201
XLOC_083097	ATG10	chr7	84304961	84409907	4.77393	6.7928	7.14462	0.0	0.155247	0.063886	0.126067	0.071135	0.115751

XLOC_083741	ATG10	chr7	84248904	84286025	4.16586	3.56174	3.72628	0.293544	0.373952	0.216895	0.654497	0.479558	0.520942
XLOC_083742	ATG10	chr7	84304961	84409907	10.7942	7.32169	2.65145	0.146087	0.946607	0.422929	0.929135	0.395315	0.643997
XLOC_085244	ATG10	chr7	84246103	84246744	1.49595	1.78851	2.33871	0.535965	0.116372	0.0	0.402277	0.0	0.130377
XLOC_085245	ATG10	chr7	84246858	84248677	2.61173	6.12127	3.06574	0.585476	0.443972	0.543449	0.756018	0.656558	0.418922
XLOC_085246	ATG10	chr7	84291054	84291977	0.0	0.0	2.21693	0.0846758	0.147649	0.0979877	0.0	0.0	0.0
XLOC_085247	ATG10	chr7	84304961	84409907	0.9847	5.00594	4.62082	0.0	0.0	0.204197	0.0	0.0	0.17196
XLOC_085248	ATG10	chr7	84304961	84409907	0.0	1.48649	2.33262	0.0	0.0776506	0.0	0.0	0.0992847	0.0868042
XLOC_085249	ATG10	chr7	84304961	84409907	0.792604	0.7112	2.4801	0.0710466	0.124014	0.164558	0.0719732	0.158701	0.207766
XLOC_085250	ATG10	chr7	84304961	84409907	1.01796	0.913214	2.38838	0.364898	0.556452	0.316577	0.460263	0.203262	0.177747
XLOC_042885	ATG14	chr10	68090655	68091845	0.70048	0.209532	1.09602	0.439557	0.877451	0.218273	0.637167	0.491451	1.1633
XLOC_041979	ATG14;KTN1	chr10	68092366	68278990	91.1085	69.3054	76.1024	29.381	29.919	26.3891	46.5594	60.3318	29.7282
XLOC_041980	ATG14;KTN1	chr10	68092366	68278990	9.03554	6.14202	7.06796	0.0	0.0642458	0.0	0.0	0.0	0.0
XLOC_042886	ATG14;KTN1	chr10	68092366	68278990	3.85381	26.4391	12.0231	4.48014	7.3612	7.07242	11.6025	7.81542	10.9959
XLOC_042887	ATG14;KTN1	chr10	68092366	68278990	0.0	37.8444	9.89227	6.91529	13.9999	5.06268	6.00879	12.6961	10.8556
XLOC_042888	ATG14;KTN1	chr10	68092366	68278990	0.0	0.0	4.75089	6.00823	5.51572	4.9281	6.60567	2.30633	4.19708
XLOC_042889	ATG14;KTN1	chr10	68092366	68278990	11.8451	5.75345	9.25929	5.57024	8.06363	6.12192	12.3464	5.87961	8.25921
XLOC_042890	ATG14;KTN1	chr10	68092366	68278990	17.5506	10.4674	6.84218	12.5565	9.35328	9.81654	17.592	8.42769	13.6379
XLOC_042891	ATG14;KTN1	chr10	68092366	68278990	15.4169	7.67246	0.0	6.89729	8.91856	7.65117	9.71348	12.8397	6.24625
XLOC_042892	ATG14;KTN1	chr10	68092366	68278990	0.0	9.1919	16.0161	12.9971	11.4238	3.08569	18.9433	26.4771	17.6092
XLOC_042893	ATG14;KTN1	chr10	68092366	68278990	0.0	3.97329	3.46268	0.0	0.0	0.0	0.0	0.0	0.0
XLOC_042894	ATG14;KTN1	chr10	68092366	68278990	0.0	10.334	3.8599	0.0	0.0	0.0	0.0	0.0	0.0
XLOC_042895	ATG14;KTN1	chr10	68092366	68278990	0.0	9.2201	7.09176	0.0	0.0	0.0	0.0	0.0	0.0
XLOC_070503	ATG16L1	chr3	113605490	113631155	27.8599	28.4493	36.0169	25.8939	28.6989	28.9734	33.1844	27.1351	29.3815
XLOC_072101	ATG16L1	chr3	113594859	113595647	1.14714	0.685977	0.0	0.411144	0.7159	1.66349	0.931382	0.0	1.20125
XLOC_072102	ATG16L1	chr3	113600536	113600720	0.0	5.96173	0.0	9.20099	7.21803	19.7863	10.6956	1.77093	5.11038
XLOC_072103	ATG16L1	chr3	113602331	113602915	1.6959	2.02717	1.32538	0.911216	2.37088	1.05039	1.51461	1.51127	0.886175
XLOC_072104	ATG16L1	chr3	113603713	113603990	5.99713	8.9388	14.0223	2.14905	4.07209	3.6375	2.00212	1.13536	7.74339

XLOC_072105	ATG16L1	chr3	113604165	113605352	2.10756	0.840568	4.39686	0.881673	1.75999	2.335	2.4282	0.704101	2.27196
XLOC_072106	ATG16L1	chr3	113605490	113631155	3.46893	5.87737	5.42514	2.38296	4.05883	3.23362	3.96327	3.80316	4.43964
XLOC_072107	ATG16L1	chr3	113605490	113631155	9.85782	5.88738	5.7735	5.29261	8.18053	9.36864	10.4192	5.56065	7.9194
XLOC_070520	ATG4B	chr3	121155437	121171711	0.0	0.692415	0.901434	1.67788	1.87311	1.36211	0.911605	0.987482	1.9136
XLOC_083414	ATG4D	chr7	16276066	16286839	13.63	17.6016	21.2871	15.0677	14.3027	15.6502	14.9722	24.7385	12.4013
XLOC_089850	ATG5	chr9	44149581	44234021	15.5222	13.0047	15.6964	13.9951	13.8058	14.4995	14.831	16.9851	11.1494
XLOC_089851	ATG5	chr9	44247501	44251512	0.0	0.140183	0.893886	0.230855	0.0734116	0.19495	0.21402	0.309965	0.163953
XLOC_089852	ATG5	chr9	44252612	44268647	0.874634	1.505	2.05383	1.49031	1.32993	1.4122	1.94671	3.48337	1.55083
XLOC_090690	ATG5	chr9	44149581	44234021	0.0	1.66484	0.0	1.49643	2.88088	1.34055	2.81121	1.4681	1.13143
XLOC_041523	ATL1	chr10	43524080	43524617	14.7365	19.0692	15.3439	41.9777	37.3534	41.8917	36.9979	37.8324	37.3306
XLOC_042661	ATL1	chr10	43512649	43515776	2.19194	3.57134	2.28756	17.4527	9.76037	16.008	13.7187	20.9152	8.25214
XLOC_041522	ATL1;MAP4K5	chr10	43479317	43512443	2.15122	5.8001	3.57816	19.2901	13.8734	17.4306	15.7262	20.0441	9.9004
XLOC_042656	ATL1;MAP4K5	chr10	43479317	43512443	0.0	1.53988	2.01344	5.07584	4.17653	3.70661	3.17328	4.54728	2.0148
XLOC_042657	ATL1;MAP4K5	chr10	43479317	43512443	8.7228	10.4044	0.0	14.041	11.6237	9.75897	11.1663	6.28453	4.14248
XLOC_042658	ATL1;MAP4K5	chr10	43479317	43512443	0.0	1.03455	5.41039	3.10075	3.71913	4.2507	1.80094	2.69004	2.70132
XLOC_042659	ATL1;MAP4K5	chr10	43479317	43512443	0.0	0.338166	3.53767	3.54694	3.61782	2.57752	2.04121	2.14242	1.97403
XLOC_042660	ATL1;MAP4K5	chr10	43479317	43512443	0.0	1.67763	1.09687	4.1476	3.82299	3.0471	4.40936	4.18263	2.56881
XLOC_041524	ATL1;SAV1	chr10	43525130	43584271	50.4212	17.9916	27.1458	11.8626	7.59446	12.7424	6.54973	7.68883	6.84592
XLOC_041926	ATL1;SAV1	chr10	43525130	43584271	17.4515	23.1436	15.2536	45.1723	47.7635	52.2755	43.0884	53.9918	57.5278
XLOC_044679	ATL2	chr11	20684915	20741503	26.7346	28.0865	30.8363	18.7531	18.1364	19.0589	16.7797	16.27	18.638
XLOC_044742	ATOH8	chr11	48986370	48992241	4.76654	0.674323	0.352733	0.834542	0.254815	0.291877	0.414207	0.135951	2.0713
XLOC_070657	ATP1A4	chr3	9610312	9611624	0.626454	0.562207	0.490137	0.561622	0.637968	0.716086	0.970048	1.00552	1.15019
XLOC_057898	ATP1B1	chr16	37509728	37574561	8.46767	13.6108	6.92605	8.54358	8.15515	8.4407	8.60866	10.8143	7.82674
XLOC_058123	ATP1B1	chr16	37509728	37574561	27.3132	31.2653	27.6485	63.2976	49.6658	53.9531	62.6259	74.4828	73.6339
XLOC_038522	ATP1B3;TFDP2	chr1	127954202	128103048	4.70205	6.01384	4.18461	19.1238	20.8037	18.8583	17.1995	17.3275	21.8327
XLOC_040200	ATP1B3;TFDP2	chr1	127954202	128103048	171.065	172.222	123.733	524.208	515.783	577.541	467.236	525.551	435.978
XLOC_040201	ATP1B3;TFDP2	chr1	127954202	128103048	0.0	1.43744	7.51729	0.0	1.46433	4.89407	2.04344	0.461083	1.66345
XLOC_040202	ATP1B3;TFDP2	chr1	127954202	128103048	2.05852	2.45891	1.60778	3.68419	2.86854	3.17925	3.28642	3.85436	3.57959

XLOC_060256	ATP2A2	chr17	56417235	56476745	41.5039	41.9389	55.9594	22.0206	29.092	24.5248	18.6645	18.3498	22.7021
XLOC_061654	ATP2A2	chr17	56417235	56476745	1.28824	5.00647	6.04288	2.07722	1.40534	3.06606	0.695428	1.28182	1.01114
XLOC_063942	ATP2A3;GSG2;P2RX1;CAMKK1	chr19	24999472	25201984	2.86278	5.61819	2.60328	0.0641981	0.0643815	0.0688129	0.0658323	0.0103235	0.0626935
XLOC_064812	ATP2A3;GSG2;P2RX1;CAMKK1	chr19	24999472	25201984	0.0	0.0	1.02093	0.0584913	0.0511003	0.0677894	0.059401	0.0	0.0
XLOC_064813	ATP2A3;GSG2;P2RX1;CAMKK1	chr19	24999472	25201984	3.88359	0.0	0.0	0.0	0.301034	0.0	0.0	0.191692	0.0
XLOC_064814	ATP2A3;GSG2;P2RX1;CAMKK1	chr19	24999472	25201984	1.52113	0.0	0.0	0.0	0.0	0.0	0.0	0.0	0.0
XLOC_064816	ATP2A3;GSG2;P2RX1;CAMKK1	chr19	24999472	25201984	0.0	0.0	3.9941	0.0	0.0	0.0	0.0	0.0	0.0
XLOC_038086	ATP2C1	chr1	140245927	140610649	1.18404	0.919024	0.47443	1.06487	1.28087	0.693491	0.929261	0.399064	0.973534
XLOC_040531	ATP2C1	chr1	140245927	140610649	0.0	0.0	0.0	0.252839	0.109861	0.437826	0.1267	0.140223	0.246073
XLOC_040532	ATP2C1	chr1	140245927	140610649	0.0	0.0	1.62608	0.372651	1.77284	0.428718	0.184602	1.02569	0.724097
XLOC_040533	ATP2C1	chr1	140245927	140610649	0.0	0.0	0.0	0.525588	0.366003	0.364499	0.423145	0.0	0.204736
XLOC_040534	ATP2C1	chr1	140245927	140610649	0.0	0.327715	0.0	0.294635	0.343458	0.0569428	0.199845	0.0549979	0.383193
XLOC_040535	ATP2C1	chr1	140245927	140610649	0.0	0.528549	0.0	0.158395	0.276312	0.0916772	0.56073	0.0883654	0.231552
XLOC_040536	ATP2C1	chr1	140245927	140610649	0.0	0.200791	1.05028	0.240692	0.262813	0.278926	0.305444	0.0672993	0.352053
XLOC_040537	ATP2C1	chr1	140245927	140610649	1.45972	0.872638	1.14108	0.130751	0.340759	0.301809	0.130931	0.289898	0.254471
XLOC_040538	ATP2C1	chr1	140245927	140610649	0.0	0.215374	0.0	0.129086	0.0563605	0.0747768	0.0	0.0	0.251715
XLOC_040539	ATP2C1	chr1	140245927	140610649	0.0	0.419475	0.0	0.0628546	0.164681	0.291312	0.318888	0.0	0.0612857
XLOC_040540	ATP2C1	chr1	140245927	140610649	0.0	0.354222	0.0	0.0	0.184783	0.24537	0.106803	0.0	0.516848
XLOC_040541	ATP2C1	chr1	140245927	140610649	0.0	0.448994	0.0	0.0	0.116849	0.155252	0.269268	0.149087	0.392746
XLOC_040542	ATP2C1	chr1	140245927	140610649	0.0	0.0	0.0	0.114789	0.0998436	0.132604	0.115303	0.0	0.447018
XLOC_040543	ATP2C1	chr1	140245927	140610649	0.0	0.230755	0.0	0.0	0.0603637	0.0800955	0.0700813	0.0	0.134827
XLOC_040544	ATP2C1	chr1	140245927	140610649	0.0	0.0	0.0	0.137106	0.238096	0.316363	0.0	0.151874	0.133395
XLOC_040545	ATP2C1	chr1	140245927	140610649	0.0	0.0	0.0	0.234134	0.509036	0.0	0.0	0.130001	0.113965
XLOC_040546	ATP2C1	chr1	140245927	140610649	0.0	0.0	0.0	0.312317	0.271881	0.240674	0.0	0.115795	0.101385
XLOC_040547	ATP2C1	chr1	140245927	140610649	0.0	0.0	1.06589	0.366408	0.212347	0.423095	0.367525	0.0	1.18882
XLOC_074792	ATP6V0A4	chr4	103215160	103216038	1.00383	0.300184	0.0	0.719674	0.391993	0.72849	0.817188	0.601425	0.788801
XLOC_053367	ATP6V1C1	chr14	63607223	63666620	2.86638	6.52282	11.7796	1.91565	1.24512	1.8745	0.501539	1.48633	1.15788

XLOC_054621	ATP6V1C1	chr14	63606049	63606384	8.13847	4.8545	6.34679	4.00263	1.24183	2.48665	0.697691	1.17623	0.35163
XLOC_054622	ATP6V1C1	chr14	63607223	63666620	4.1702	7.55051	5.54914	1.51481	1.62254	1.73793	0.976206	1.03026	1.18677
XLOC_054623	ATP6V1C1	chr14	63607223	63666620	4.97287	4.83479	8.26792	0.947376	1.60699	1.09815	0.736129	0.87308	0.543482
XLOC_054624	ATP6V1C1	chr14	63607223	63666620	28.7174	8.61207	0.0	10.5261	10.5664	1.43683	6.77319	6.51769	1.23377
XLOC_054625	ATP6V1C1	chr14	63607223	63666620	4.03527	10.8494	12.6102	2.34806	3.28232	2.91008	0.716575	0.795994	1.40408
XLOC_054626	ATP6V1C1	chr14	63607223	63666620	5.29122	2.7699	2.06985	1.36374	2.1755	1.30559	0.602058	1.19381	1.2142
XLOC_054627	ATP6V1C1	chr14	63607223	63666620	2.12615	5.29985	6.65342	1.3977	0.943192	1.10414	1.0313	0.781285	0.867224
XLOC_054628	ATP6V1C1	chr14	63607223	63666620	4.05474	3.63751	7.13504	0.817568	0.47497	1.05085	0.183346	0.607257	1.06202
XLOC_054629	ATP6V1C1	chr14	63607223	63666620	1.18418	4.07396	6.48576	0.690085	1.06709	0.923116	0.215864	0.653655	0.67305
XLOC_054630	ATP6V1C1	chr14	63607223	63666620	6.13611	5.04341	4.79629	0.412189	0.47719	0.158514	0.0	0.456577	0.935738
XLOC_054631	ATP6V1C1	chr14	63607223	63666620	3.91118	2.92498	5.10004	0.584386	0.408436	0.745013	0.118696	0.392245	0.398911
XLOC_054632	ATP6V1C1	chr14	63607223	63666620	4.40781	4.39482	6.32183	0.395119	0.804983	1.06805	0.267124	0.368001	0.642038
XLOC_054633	ATP6V1C1	chr14	63607223	63666620	5.82537	5.22119	7.58575	2.08613	1.8062	1.60114	0.863236	0.766777	0.506877
XLOC_054634	ATP6V1C1	chr14	63607223	63666620	2.22481	3.32875	4.72628	0.769581	0.57408	1.09202	0.0291173	0.41608	0.278193
XLOC_054635	ATP6V1C1	chr14	63607223	63666620	2.58545	7.34279	7.07502	0.926506	0.705087	0.802678	0.232617	0.257263	0.676492
XLOC_054636	ATP6V1C1	chr14	63607223	63666620	1.63089	7.31094	10.1972	0.730286	1.26732	0.673686	0.291655	0.484834	0.710337
XLOC_054637	ATP6V1C1	chr14	63607223	63666620	4.67759	2.79709	4.57206	0.419112	0.0912075	0.968877	0.210903	0.233071	0.102038
XLOC_054638	ATP6V1C1	chr14	63607223	63666620	1.61271	7.71152	6.30234	0.866588	0.752023	0.666249	0.14425	0.319692	0.561971
XLOC_054639	ATP6V1C1	chr14	63607223	63666620	1.01368	4.24376	7.92782	0.545045	0.633293	0.945762	0.366691	0.30363	0.354005
XLOC_054640	ATP6V1C1	chr14	63607223	63666620	4.33264	3.51801	6.77976	0.610387	0.387915	0.385931	0.281929	0.186296	0.270581
XLOC_054641	ATP6V1C1	chr14	63607223	63666620	5.24486	3.13667	7.38314	0.751997	0.982728	1.08719	0.0948023	0.418751	0.457851
XLOC_054642	ATP6V1C1	chr14	63607223	63666620	2.56255	5.36261	5.00888	0.573943	0.499218	0.795624	0.115303	1.02006	0.335263
XLOC_054643	ATP6V1C1	chr14	63607223	63666620	11.8804	15.9574	6.95466	1.06268	0.45678	0.304257	0.0	0.868971	0.515016
XLOC_088663	ATP6V1G1	chr8	105494607	105495807	0.69376	0.830092	0.542759	0.373151	0.271588	0.360314	0.441793	0.0695395	0.363844
XLOC_047828	ATP8A2;ATP8A2	chr12	33958281	33959832	8.38555	8.52718	8.52719	1.42805	0.786931	0.78322	0.456486	0.671228	1.17213
XLOC_049032	ATP8A2;ATP8A2	chr12	33957893	33958104	0.0	3.67118	0.0	1.11708	0.906494	0.0	0.0	1.12162	0.0
XLOC_083643	ATP8B3	chr7	45731349	45749756	0.0	0.79129	0.0	0.711388	0.927878	0.684681	0.357025	0.263279	0.692508
XLOC_063063	ATXN1L	chr18	39373395	39375684	12.2149	7.71631	6.90433	0.699844	1.19887	1.09499	0.714655	0.785663	0.801805
XLOC_073166	ATXN7L1	chr4	47015613	47057073	0.712326	0.709944	1.08647	0.188758	0.328897	0.363962	0.11877	0.0778257	0.172201
XLOC_072625	ATXN7L1;ATXN7L1	chr4	47091911	47123095	7.3929	8.99061	7.36294	1.2143	1.76696	1.48438	1.05365	1.18761	1.55082
XLOC_072626	ATXN7L1;ATXN7L1	chr4	47208304	47298728	8.37857	7.21776	8.46097	0.0974011	0.042657	0.0565514	0.0248904	0.0273524	0.0237641

XLOC_070798	ATXN7L2	chr3	34070985	34071174	0.0	5.37302	28.0919	4.95823	15.6796	19.6479	20.8014	4.81703	10.7555
XLOC_070799	ATXN7L2	chr3	34071316	34072789	3.84847	3.12508	3.87165	5.86579	5.99017	6.97334	9.9797	5.74137	8.5584
XLOC_070800	ATXN7L2	chr3	34072974	34074254	2.57726	1.73467	3.02461	4.44769	5.09709	6.22609	6.57092	4.71744	7.77337
XLOC_070801	ATXN7L2	chr3	34074354	34074798	2.52465	1.50771	0.0	5.8734	6.62322	6.74175	6.21966	4.45493	7.67269
XLOC_070802	ATXN7L2	chr3	34075023	34075747	3.83025	3.05354	2.99473	4.68974	6.06918	6.21056	7.23902	4.70137	7.79532
XLOC_070803	ATXN7L2	chr3	34075945	34076938	4.32828	3.36567	3.38559	4.73283	5.54994	7.36538	7.14354	3.72321	6.2007
XLOC_069872	ATXN7L2;AMIGO1;CYB561D1;GPR61	chr3	34020068	34070929	164.0	195.864	168.514	454.025	413.76	444.699	539.757	534.356	566.246
XLOC_070795	ATXN7L2;AMIGO1;CYB561D1;GPR61	chr3	34020068	34070929	11.3146	4.83092	12.6332	15.0544	17.3347	20.5325	19.8051	20.3445	27.3169
XLOC_070796	ATXN7L2;AMIGO1;CYB561D1;GPR61	chr3	34020068	34070929	7.49216	2.2361	0.0	15.4826	18.5275	27.159	22.7604	18.2375	24.4682
XLOC_070797	ATXN7L2;AMIGO1;CYB561D1;GPR61	chr3	34020068	34070929	19.4184	6.96215	7.58621	11.4738	11.8908	18.6133	16.4015	15.1437	16.052
XLOC_070253	ATXN7L2;SYPL2	chr3	34077027	34082579	4.47575	3.43623	4.38076	6.27896	7.44089	8.42303	10.4397	7.42131	9.08926
XLOC_076196	ATXN7L3B	chr5	3928272	4081720	20.4899	24.7314	22.1186	11.9387	12.9114	13.7498	13.8294	11.2279	12.5377
XLOC_077241	ATXN7L3B	chr5	3928272	4081720	0.0	1.78885	0.0	2.6787	4.60473	2.14715	3.13587	1.45991	2.07727
XLOC_077242	ATXN7L3B	chr5	3928272	4081720	0.0	3.03607	0.0	0.455462	1.9289	3.61209	3.00816	1.45648	5.26476
XLOC_088508	AUH	chr8	87959735	87959912	0.0	0.0	0.0	0.0	3.3647	2.31571	0.0	2.05856	0.0
XLOC_088509	AUH	chr8	87959987	87960304	0.0	0.0	0.0	3.23642	3.09682	1.83896	1.15531	3.47069	0.0
XLOC_086551	AUH;AUH	chr8	88118780	88135119	8.72748	11.3558	10.0646	9.14369	14.4488	12.1602	15.5538	9.26268	10.5284
XLOC_087000	AUH;NFIL3	chr8	87661730	87959440	1.98706	0.644238	1.14013	8.2173	4.68423	6.52964	6.50805	12.6481	3.21621
XLOC_062474	AURKC	chr18	64530655	64661143	2.64791	0.506835	1.38787	0.258362	0.21583	0.0809972	0.0622299	0.0625545	0.0275556
XLOC_058230	AVPR1B	chr16	3730351	3731132	0.0	0.0	0.0	485.394	442.009	425.194	546.974	439.787	783.67
XLOC_038444	B3GNT5;LAMP3;MCCC1	chr1	84369840	84585540	0.925425	1.07284	0.863387	0.321526	0.671605	0.459527	1.28175	0.361213	1.56923

XLOC_062395	B3GNT9;FBXL8;TRADD;NOL3;C18H16orf70;HSF4	chr18	34902334	34932927	60.6903	58.8458	49.3486	107.292	86.545	99.1115	125.197	154.137	104.199
XLOC_076442	B4GALNT3	chr5	107902934	107911940	21.5386	13.7559	16.8739	44.3836	40.8258	42.1232	48.4692	52.6125	50.5404
XLOC_078578	B4GALNT3	chr5	107902934	107911940	2.36557	6.71908	7.39908	13.5649	13.4673	8.20656	16.6347	18.1486	10.2169
XLOC_063182	B9D2	chr18	50789871	50790707	0.0	0.0	0.0	0.47761	0.416052	1.32564	0.577855	0.106362	0.651344
XLOC_053366	BAALC	chr14	63436571	63464599	0.0	0.36114	0.188912	0.822563	0.512079	0.729089	0.442856	0.583867	0.633942
XLOC_053667	BAALC	chr14	63492205	63509484	0.460437	0.964409	0.720658	154.859	93.1003	133.714	163.555	285.166	140.618
XLOC_053668	BAALC	chr14	63522856	63544113	13.2197	16.4407	12.6786	17.8298	20.4453	17.2477	16.7244	20.2866	17.6577
XLOC_054613	BAALC	chr14	63489428	63489843	0.0	0.0	0.0	1.50761	1.94626	0.864002	4.91793	1.37217	2.43655
XLOC_054614	BAALC	chr14	63490504	63490757	0.0	0.0	0.0	1.99941	3.33678	3.7359	3.65752	1.38988	8.28847
XLOC_054615	BAALC	chr14	63490861	63491237	0.0	0.987466	0.0	2.66351	4.56884	2.36847	6.03168	9.00145	6.30503
XLOC_054616	BAALC	chr14	63491654	63492144	0.0	0.0	0.0	11.2934	4.88109	8.28433	11.171	21.6292	10.9694
XLOC_054617	BAALC	chr14	63492205	63509484	0.0	0.330929	0.0	10.5123	11.2275	8.14138	14.4882	21.0818	14.1028
XLOC_054618	BAALC	chr14	63492205	63509484	0.0	0.0	0.759803	13.3205	14.7228	12.8941	23.7395	27.2695	18.4927
XLOC_054619	BAALC	chr14	63519544	63522297	3.62238	4.16921	3.48946	4.34823	5.86639	4.78806	5.44131	6.31654	5.95187
XLOC_054620	BAALC	chr14	63522398	63522742	11.6228	8.08929	18.1303	14.8968	15.3907	14.6136	13.6595	16.8326	13.3985
XLOC_055415	BACE1	chr15	28409011	28448453	0.374826	0.448624	1.46671	0.0672247	0.0	0.0390117	0.0	0.0	0.0
XLOC_050507	BAMBI	chr13	35979421	36537621	15.6575	21.3093	15.8881	14.6678	12.2662	11.9948	11.0298	13.3853	14.6336
XLOC_050808	BAMBI	chr13	35979421	36537621	30.054	34.2852	24.7244	23.1108	17.3411	24.1738	24.6484	26.3487	21.7313
XLOC_051618	BAMBI	chr13	35979421	36537621	16.6927	16.9011	12.0555	17.3247	20.2661	21.5475	22.9175	22.4738	16.0529
XLOC_051619	BAMBI	chr13	35979421	36537621	1.10848	1.65718	5.2009	0.993242	1.03796	1.3781	0.70048	1.43706	1.25765
XLOC_059767	BBS12;CETN4;IL21	chr17	35262739	35474227	79.3817	34.813	51.561	13.4004	13.3429	10.8404	7.09321	4.93853	12.0206
XLOC_059768	BBS12;CETN4;IL21	chr17	35262739	35474227	11.4175	6.30402	6.86977	2.44024	1.09859	2.73372	0.796271	1.05403	0.690461
XLOC_059769	BBS12;CETN4;IL21	chr17	35262739	35474227	29.9616	0.0	29.2012	2.01951	0.561512	1.50891	0.0	0.701539	0.0
XLOC_059770	BBS12;CETN4;IL21	chr17	35262739	35474227	0.0	0.85708	3.36227	0.256858	0.161935	0.214971	0.0	0.0	0.124996
XLOC_059771	BBS12;CETN4;IL21	chr17	35262739	35474227	3.13228	2.06142	3.43097	0.112331	0.245378	0.781192	0.285317	0.0628536	0.10955
XLOC_059772	BBS12;CETN4;IL21	chr17	35262739	35474227	3.23264	4.34741	2.52658	0.289524	0.251243	0.166949	0.144586	0.4806	0.422435
XLOC_061316	BBS12;CETN4;IL21	chr17	35262739	35474227	16.7827	12.5192	0.0	1.51169	0.626962	0.843842	0.0	0.0	0.0

XLOC_061317	BBS12;CETN4;IL21	chr17	35262739	35474227	5.58143	6.39849	5.82067	17.8412	11.2675	14.4726	13.9023	19.4312	14.9483
XLOC_079994	BBS7	chr6	3372225	3399552	4.18567	3.38237	3.71342	5.25616	3.88438	4.23194	2.28281	2.15441	3.996
XLOC_073206	BBS9	chr4	63959935	64046924	4.29798	1.3934	3.38132	3.49984	0.897315	2.80706	6.44677	2.89614	4.04341
XLOC_037841	BBX	chr1	52630709	52651313	3.09962	3.71943	4.399	9.31626	10.4939	10.4882	9.0409	6.13589	11.3783
XLOC_038346	BBX	chr1	52658208	52661178	3.93444	2.74443	8.20287	0.352473	0.919554	0.678523	0.235911	0.391395	0.343129
XLOC_038347	BBX	chr1	52663380	52727045	4.46026	6.13876	5.58459	0.239965	0.27993	0.417614	0.52993	0.269066	0.234131
XLOC_038348	BBX	chr1	52789292	52817770	5.80256	5.64291	6.80874	1.82179	2.19922	2.11153	2.11888	1.31325	1.31142
XLOC_039291	BBX	chr1	52630709	52651313	6.35724	2.84582	0.0	2.84285	2.19638	4.87852	3.31799	0.927746	3.30476
XLOC_039292	BBX	chr1	52651477	52653568	2.9924	1.23116	0.58547	2.8176	3.64085	3.11449	2.32776	2.29612	4.22262
XLOC_039293	BBX	chr1	52656601	52658063	4.43534	1.99037	3.03666	0.497078	0.782218	0.518749	0.758517	0.389675	0.43637
XLOC_039294	BBX	chr1	52786760	52787138	3.27926	0.978557	2.55882	0.0	1.25792	0.670695	0.569537	0.0	1.70416
XLOC_063186	BCKDHA	chr18	50819617	50820331	0.0	0.0	0.0	1.16437	0.405056	0.672475	0.0	0.129311	0.113354
XLOC_063187	BCKDHA	chr18	50827011	50829289	0.0	0.0	0.0	1.10109	0.642708	1.10066	0.374786	0.4807	1.04476
XLOC_063188	BCKDHA	chr18	50830432	50831147	0.0	0.0	0.0	1.39474	0.505422	0.671279	0.466864	0.258166	0.226302
XLOC_063189	BCKDHA	chr18	50833931	50834474	0.0	0.0	0.0	1.84791	0.291033	0.773883	0.334046	0.185374	0.979795
XLOC_063190	BCKDHA	chr18	50836195	50836718	0.0	0.0	0.0	1.94884	0.460064	1.01965	0.175836	0.195276	0.344344
XLOC_063191	BCKDHA;B3GNT8	chr18	50838315	50840165	0.0	0.0	0.0	1.03456	0.335283	1.73394	0.390674	0.300811	0.859909
XLOC_063265	BCL2L12	chr18	56524760	56524975	0.0	0.0	0.0	0.0	0.0	1.15981	0.922339	0.0	0.0
XLOC_089514	BCLAF1	chr9	75271286	75305414	0.90308	0.484381	1.22752	4.65839	6.1384	5.01426	3.69649	9.37337	9.36222
XLOC_091287	BCLAF1	chr9	75271286	75305414	0.0	0.0	0.0	1.16259	2.7432	0.332395	0.282356	0.315927	2.53356
XLOC_089513	BCLAF1;FAM54A	chr9	75232023	75270361	0.0	0.22748	0.0	3.67333	4.09003	3.7306	3.13937	4.62327	6.25714
XLOC_091285	BCLAF1;FAM54A	chr9	75232023	75270361	0.0	0.0	0.0	0.265461	0.385622	0.511877	0.0893382	0.0986151	1.12078
XLOC_091286	BCLAF1;FAM54A	chr9	75232023	75270361	0.0	0.0	0.0	0.543372	0.626926	0.0	0.0	0.399079	2.11197
XLOC_037902	BDH1	chr1	72583175	72633778	25.5846	17.9082	13.4195	29.4903	28.2566	28.6905	34.0452	32.1201	27.3033
XLOC_079618	BDH2	chr6	22961594	23052760	5.99643	3.23547	5.38977	0.427069	0.0740393	0.203207	0.156026	0.458893	0.0607858
XLOC_070072	BEND5	chr3	98013480	98139091	19.3958	20.2783	15.6961	5.9128	5.33925	5.50524	4.59035	5.6747	3.89128
XLOC_070451	BEST4;RPS8;KIF2C	chr3	101813723	101848937	0.291786	0.174621	0.456765	3.76736	3.88264	3.95791	2.89413	2.24595	4.82946
XLOC_072531	BET1	chr4	11156389	11172480	27.6496	30.2669	28.2523	63.5113	53.8749	61.3014	44.4733	51.5029	56.2391
XLOC_073721	BET1	chr4	11156389	11172480	0.0	5.10046	5.33413	9.7823	8.90803	12.5767	11.2539	5.97134	7.39994
XLOC_073722	BET1	chr4	11156389	11172480	9.75811	8.7424	1.90508	9.60517	10.1715	13.2873	10.5334	7.90072	14.6214

XLOC_073723	BET1	chr4	11172552	11174510	16.0673	25.48	24.5193	52.0844	43.345	48.3624	37.6233	46.9138	50.4373
XLOC_073724	BET1	chr4	11175054	11175236	0.0	6.22897	0.0	7.70236	16.5614	8.26374	14.3264	11.0739	16.013
XLOC_073725	BET1	chr4	11175481	11175851	0.0	5.07595	0.0	2.43412	2.08633	2.08621	5.60207	0.660371	2.35658
XLOC_050512	BFSP1	chr13	38204915	38227814	28.5499	22.3541	22.3395	38.9295	44.8697	43.2346	47.3689	39.8113	50.4843
XLOC_051647	BFSP1	chr13	38204915	38227814	0.0	10.5691	9.21217	4.22478	5.11114	2.80881	5.41168	4.93849	4.08413
XLOC_051648	BFSP1	chr13	38204915	38227814	1.38694	2.48758	1.08433	1.36666	1.29585	1.14758	1.74398	0.827093	0.967402
XLOC_051649	BFSP1	chr13	38204915	38227814	22.3614	3.33316	0.0	20.0201	20.7093	19.2392	15.0065	23.3619	16.8525
XLOC_051650	BFSP1	chr13	38204915	38227814	8.97898	13.6952	12.6417	16.4973	16.9848	17.1394	15.7869	17.7756	17.1752
XLOC_051651	BFSP1	chr13	38204915	38227814	20.2494	18.1682	17.9049	17.5177	22.4378	21.1919	19.3954	19.8104	23.0709
XLOC_050511	BFSP1;SLC6A9	chr13	38108236	38198988	8.91805	5.93895	6.58749	13.4911	16.3426	15.3798	10.0996	9.51945	15.2049
XLOC_051646	BFSP1;SLC6A9	chr13	38108236	38198988	0.0	0.0	0.0	1.36971	3.93646	1.5731	1.12203	0.25051	2.21524
XLOC_086995	BICD2	chr8	85636610	85700854	8.05231	9.14442	7.79106	0.740693	0.997441	0.763108	0.638145	0.677405	0.642002
XLOC_088490	BICD2	chr8	85717165	85718172	2.55416	2.54634	2.66387	3.20501	3.72816	2.38543	1.46731	2.04418	3.49566
XLOC_086909	BIN3	chr8	70450824	70476961	3.25889	5.98693	6.43481	7.70797	9.06349	6.15122	5.06999	4.97752	5.82606
XLOC_088012	BIN3	chr8	70450236	70450708	2.29993	3.43447	5.38893	7.20416	5.68871	3.31151	3.86026	2.26069	4.59686
XLOC_088013	BIN3	chr8	70450824	70476961	0.0	1.02897	5.38218	1.23343	0.7159	0.475284	0.413947	0.114346	0.100104
XLOC_088014	BIN3	chr8	70450824	70476961	0.0	0.462512	1.20968	0.877861	1.25252	0.643032	0.517384	0.414127	0.27043
XLOC_088015	BIN3	chr8	70450824	70476961	1.98784	1.18928	0.518415	0.950428	0.726488	0.413046	0.361889	0.265772	0.347549
XLOC_088016	BIN3	chr8	70450824	70476961	3.33873	0.0	2.61166	1.34665	1.17498	1.0395	0.454411	0.584641	0.437526
XLOC_088017	BIN3	chr8	70450824	70476961	2.18663	1.63573	1.9965	1.69942	1.28742	1.06212	0.767234	0.440139	0.924907
XLOC_088018	BIN3	chr8	70450824	70476961	2.98415	1.14799	3.00252	1.22326	1.23763	0.887111	0.935424	0.771701	0.783286
XLOC_088019	BIN3	chr8	70450824	70476961	0.0	0.901282	2.3568	1.35045	0.696337	1.85547	0.790162	0.294316	0.523519
XLOC_088020	BIN3	chr8	70450824	70476961	2.61348	4.29712	3.06499	2.69254	2.34157	2.9748	1.99817	1.56001	1.02569
XLOC_056079	BIRC3	chr15	6653731	6654863	63.0847	70.1502	59.2217	237.174	177.683	219.49	200.01	277.912	230.451
XLOC_045524	BIRC6	chr11	15049204	15049420	57.0651	58.0288	26.7673	103.686	91.2816	84.6365	77.374	82.7307	94.0871
XLOC_050472	BMI1;COMMD3;SPAG6	chr13	23531007	23764773	411.287	376.136	324.868	1136.13	1475.46	1399.4	1127.5	975.002	1157.12
XLOC_050473	BMI1;COMMD3;SPAG6	chr13	23531007	23764773	76.3958	53.498	30.9193	745.912	296.439	737.49	420.917	442.067	166.719
XLOC_050474	BMI1;COMMD3;SPAG6	chr13	23531007	23764773	306.645	262.039	272.246	1081.59	1128.87	890.522	948.241	884.468	974.038
XLOC_050475	BMI1;COMMD3;SPAG6	chr13	23531007	23764773	107.104	90.867	88.2585	345.82	358.414	378.57	265.134	258.408	276.366

XLOC_050769	BMI1;COMMD3;SPAG6	chr13	23531007	23764773	84.5687	95.804	62.926	741.824	643.84	663.759	446.657	431.232	588.787
XLOC_050770	BMI1;COMMD3;SPAG6	chr13	23531007	23764773	91.3284	76.9706	50.5347	698.038	869.657	838.469	752.584	614.212	679.772
XLOC_050771	BMI1;COMMD3;SPAG6	chr13	23531007	23764773	427.85	227.295	242.71	748.53	820.578	872.403	713.508	615.359	597.314
XLOC_050772	BMI1;COMMD3;SPAG6	chr13	23531007	23764773	12.7013	10.2479	11.0856	228.293	258.444	228.583	169.283	140.266	169.695
XLOC_050773	BMI1;COMMD3;SPAG6	chr13	23531007	23764773	142.435	112.465	103.012	954.616	977.397	1031.94	870.453	869.835	856.934
XLOC_050774	BMI1;COMMD3;SPAG6	chr13	23531007	23764773	109.724	103.508	95.5148	171.422	150.826	261.648	112.245	110.013	76.6754
XLOC_051361	BMI1;COMMD3;SPAG6	chr13	23531007	23764773	5.49081	7.48277	5.72797	80.2368	95.7861	89.0178	66.4266	65.9275	86.6805
XLOC_051362	BMI1;COMMD3;SPAG6	chr13	23531007	23764773	0.0	0.0	0.0	4.335	4.40517	3.58304	0.0	0.0	1.0229
XLOC_051363	BMI1;COMMD3;SPAG6	chr13	23531007	23764773	0.0	0.0	0.0	0.540241	0.0	0.619347	0.267744	0.0	0.261759
XLOC_051364	BMI1;COMMD3;SPAG6	chr13	23531007	23764773	50.2046	40.4632	33.2737	352.458	288.894	381.544	271.008	245.887	198.013
XLOC_051365	BMI1;COMMD3;SPAG6	chr13	23531007	23764773	26.6368	30.0279	26.0548	147.909	173.364	159.457	139.555	116.933	137.852
XLOC_051366	BMI1;COMMD3;SPAG6	chr13	23531007	23764773	0.922904	0.828345	0.722137	0.372351	0.180961	0.288106	0.29579	0.139191	0.0403639
XLOC_051367	BMI1;COMMD3;SPAG6	chr13	23531007	23764773	4.30224	6.43192	5.88751	28.912	20.399	30.3144	20.2091	13.0905	9.48149
XLOC_051368	BMI1;COMMD3;SPAG6	chr13	23531007	23764773	0.0	0.0	0.0	2.14683	1.17951	1.95845	0.342483	0.878893	0.990077
XLOC_051369	BMI1;COMMD3;SPAG6	chr13	23531007	23764773	0.0	0.0	0.0	2.22058	1.57802	0.843903	0.714677	2.39126	1.07267
XLOC_092195	BMP15	chrX	93969513	93974714	42.3876	56.1822	41.4567	6.12261	4.79776	6.6087	1.39521	2.59982	4.42717
XLOC_093259	BMP15	chrX	93975157	93975469	74.6848	115.512	54.5842	12.1074	11.7068	10.9047	1.58619	8.49393	6.04123
XLOC_050540	BMP2	chr13	49463750	49652940	4.52404	1.96893	5.14968	0.811356	1.06505	1.19828	0.752253	0.868746	0.899655
XLOC_050541	BMP2	chr13	49463750	49652940	8.49199	9.46471	7.91498	2.221	2.49015	3.00876	3.96416	2.06782	4.84011
XLOC_050542	BMP2	chr13	49463750	49652940	42.731	19.7441	27.0982	6.93878	8.05769	9.13637	6.6625	7.12841	8.8914
XLOC_050846	BMP2	chr13	49463750	49652940	14.2348	28.0011	18.3866	26.5247	20.9807	25.0438	27.5253	32.7629	31.2607
XLOC_050847	BMP2	chr13	49463750	49652940	2.56065	3.83121	2.75564	2.84178	3.36826	3.0326	3.51867	2.77194	4.28646
XLOC_051802	BMP2	chr13	49463750	49652940	2.78893	2.2762	1.78602	0.545736	1.13552	1.21478	0.906962	0.664361	0.86569
XLOC_051803	BMP2	chr13	49463750	49652940	3.46263	2.84788	4.06271	0.698298	1.1506	1.07786	0.549517	0.7793	1.05866
XLOC_051804	BMP2	chr13	49463750	49652940	10.0044	7.17304	7.81613	1.97039	3.10051	2.88622	3.02112	2.36867	2.08876

XLOC_051805	BMP2	chr13	49463750	49652940	0.0	3.83851	2.31672	0.619421	0.848378	1.53563	0.357368	1.18341	0.775924
XLOC_051806	BMP2	chr13	49463750	49652940	0.0	2.47683	6.47491	1.49513	4.3425	0.0	2.03024	3.86758	7.84478
XLOC_051807	BMP2	chr13	49463750	49652940	3.84233	2.29592	0.0	0.860027	0.893671	0.990212	0.854414	0.379456	0.835881
XLOC_051808	BMP2	chr13	49463750	49652940	7.41744	8.84847	0.0	1.33304	3.33685	2.24156	1.82886	0.695126	2.5503
XLOC_051809	BMP2	chr13	49463750	49652940	4.1608	3.3167	8.67412	1.61515	2.05174	1.86479	1.49485	1.37848	3.26494
XLOC_051810	BMP2	chr13	49463750	49652940	2.12639	4.29409	8.7352	1.76353	2.08378	2.87425	1.60074	1.06784	1.48779
XLOC_051811	BMP2	chr13	49463750	49652940	4.60902	4.12265	3.59322	0.412282	1.75209	0.468272	0.0	0.0	0.397729
XLOC_051812	BMP2	chr13	49463750	49652940	6.8346	10.2063	5.33814	2.03895	1.23288	2.57766	1.20802	1.12002	2.57388
XLOC_051813	BMP2	chr13	49463750	49652940	1.60913	3.3663	3.77301	1.72935	2.37619	1.82815	2.73485	0.638018	1.96255
XLOC_051814	BMP2	chr13	49463750	49652940	17.0949	10.8509	5.00767	2.48653	3.63839	5.27962	3.78645	1.89412	5.57402
XLOC_080240	BMP3;C6H4orf22	chr6	97286832	97617565	18.8764	13.0566	14.2318	0.482898	0.730432	0.996773	0.407848	3.38564	2.23153
XLOC_081914	BMP3;C6H4orf22	chr6	97286832	97617565	5.31193	4.23271	4.15104	0.0	0.0	0.0	0.157921	0.0	0.0
XLOC_081915	BMP3;C6H4orf22	chr6	97286832	97617565	1.44225	0.4311	0.0	0.0	0.0	0.0	0.0	0.0	0.0
XLOC_081916	BMP3;C6H4orf22	chr6	97286832	97617565	0.0	0.448053	0.0	0.0	0.116614	0.0	0.0	0.0	0.0
XLOC_081917	BMP3;C6H4orf22	chr6	97286832	97617565	1.37827	0.41228	1.07828	0.0	0.0	0.0	0.0	0.0	0.0
XLOC_081918	BMP3;C6H4orf22	chr6	97286832	97617565	0.0	0.0	3.66233	0.0	0.0	0.0	0.0	0.0	0.0
XLOC_081919	BMP3;C6H4orf22	chr6	97286832	97617565	1.11016	0.331944	0.868146	0.0994823	0.0866315	0.0	0.0	0.0	0.0
XLOC_081920	BMP3;C6H4orf22	chr6	97286832	97617565	0.0	1.41454	0.0	0.0	0.092245	0.122486	0.0	0.0	0.0
XLOC_081921	BMP3;C6H4orf22	chr6	97286832	97617565	12.929	7.70904	5.03871	0.0	0.0	0.0	0.0	0.0	0.0
XLOC_081922	BMP3;C6H4orf22	chr6	97286832	97617565	4.0902	0.0	1.06589	0.0	0.0	0.0	0.0	0.0	0.0
XLOC_081923	BMP3;C6H4orf22	chr6	97286832	97617565	2.30702	0.460028	1.20316	0.0	0.0	0.0798412	0.0	0.0	0.0
XLOC_081924	BMP3;C6H4orf22	chr6	97286832	97617565	3.33703	0.0	0.0	0.0	0.0	0.0	0.0	0.0	0.0
XLOC_081925	BMP3;C6H4orf22	chr6	97286832	97617565	1.95756	0.0	1.52945	0.0	0.0	0.201748	0.0	0.0	0.0
XLOC_081926	BMP3;C6H4orf22	chr6	97286832	97617565	0.0	1.26288	3.30217	0.0	0.0	0.0	0.0	0.0	0.0
XLOC_081927	BMP3;C6H4orf22	chr6	97286832	97617565	1.09173	0.326438	0.853746	0.0	0.0	0.0	0.0	0.0	0.190586

XLOC_081928	BMP3;C6H4orf22	chr6	97286832	97617565	0.0	0.392774	1.02722	0.0	0.0	0.0	0.0	0.0	0.0
XLOC_081929	BMP3;C6H4orf22	chr6	97286832	97617565	0.0	0.0	0.0	0.0	0.0	0.0	0.0	0.179901	0.0
XLOC_081932	BMP3;C6H4orf22	chr6	97286832	97617565	1.4058	0.420221	2.19799	0.0	0.0	0.0	0.12622	0.0	0.0
XLOC_081934	BMP3;C6H4orf22	chr6	97286832	97617565	3.92574	0.0	3.06717	0.0	0.0	0.0	0.174455	0.0	0.0
XLOC_081935	BMP3;C6H4orf22	chr6	97286832	97617565	1.25915	0.470983	0.492741	0.0	0.0247224	0.0327752	0.0	0.0	0.0275565
XLOC_081936	BMP3;C6H4orf22	chr6	97286832	97617565	1.55047	0.0	1.21191	0.0	0.0	0.0	0.0	0.0	0.0
XLOC_081937	BMP3;C6H4orf22	chr6	97286832	97617565	6.94188	0.0	5.41144	0.0	0.0	0.0	0.0	0.0	0.0
XLOC_081938	BMP3;C6H4orf22	chr6	97286832	97617565	0.0	0.0	0.0	0.116444	0.0	0.0	0.0	0.0	0.113367
XLOC_081939	BMP3;C6H4orf22	chr6	97286832	97617565	0.0	1.29588	2.25939	0.0	0.0	0.0	0.0	0.0	0.0
XLOC_081940	BMP3;C6H4orf22	chr6	97286832	97617565	0.0	0.771548	0.0	0.0	0.0	0.133543	0.116106	0.128403	0.112561
XLOC_081941	BMP3;C6H4orf22	chr6	97286832	97617565	2.34465	0.0	1.83116	0.0	0.0	0.0	0.0	0.0	0.0
XLOC_081942	BMP3;C6H4orf22	chr6	97286832	97617565	2.97843	0.29689	0.0	0.0	0.0	0.0	0.0	0.0	0.0
XLOC_050890	BMP7	chr13	59496408	59557347	16.1336	16.7957	15.2033	6.04082	5.85415	6.44374	5.52062	6.18651	5.5106
XLOC_050889	BMP7;MIR2307	chr13	59449534	59495650	7.34005	14.8464	16.5435	11.6623	13.3816	12.9646	17.4958	23.0679	20.8965
XLOC_051951	BMP7;MIR2307	chr13	59449534	59495650	1.45093	2.60216	2.26844	3.37909	2.82276	3.15009	4.81608	4.89913	3.66775
XLOC_072679	BMPER	chr4	62965602	63133860	7.96422	6.16508	4.46379	0.157681	0.222711	0.0358338	0.0307681	0.227835	0.0602726
XLOC_072680	BMPER	chr4	63214331	63245701	2.33718	0.874222	1.82925	0.235777	0.413036	0.243351	0.160635	0.0293968	0.153414
XLOC_072681	BMPER	chr4	63254285	63277827	4.57178	0.0	0.655938	0.242287	0.144776	0.0	0.16619	0.0	0.162462
XLOC_072682	BMPER	chr4	63281065	63324664	1.52298	1.26008	1.42195	0.753965	0.218031	0.277386	0.253088	0.114936	0.242453
XLOC_073203	BMPER	chr4	63165411	63172355	0.253806	0.0	0.213395	0.0	0.0	0.0	0.0	0.0	0.0
XLOC_074390	BMPER	chr4	62965602	63133860	0.0	0.0	0.0	0.0562112	0.0491168	0.0	0.114221	0.125799	0.0
XLOC_074391	BMPER	chr4	63134206	63139696	4.06871	4.05911	4.14051	0.0851552	0.0746659	0.141367	0.112206	0.109507	0.0237578
XLOC_074392	BMPER	chr4	63252957	63253592	0.0	0.0	0.0	0.0	0.117817	0.0	0.0	0.0	0.0
XLOC_079638	BMPR1B	chr6	30737975	30896951	7.88587	9.68759	7.75229	31.7392	33.9197	30.112	44.1812	48.5724	30.7735
XLOC_080050	BMPR1B	chr6	30737975	30896951	25.423	42.0722	36.8383	49.3151	56.1439	50.127	47.7428	51.6363	60.7863
XLOC_092584	BMX	chrX	135208687	135210671	1.53965	0.690776	0.602219	0.0	0.0	0.0	0.0	0.0	0.0

XLOC_037858	BOC	chr1	58430992	58462115	9.63613	8.59713	7.46752	32.0785	22.4683	25.1673	32.5092	38.2319	28.568
XLOC_038372	BOC	chr1	58430992	58462115	8.25247	15.4724	10.1282	34.003	31.0603	33.4543	30.9381	34.2871	38.7358
XLOC_038373	BOC	chr1	58463448	58505235	20.5344	26.5955	20.728	49.3371	42.5052	43.5597	56.106	62.4463	54.9741
XLOC_039373	BOC	chr1	58429063	58429730	0.0	0.42434	0.0	0.508652	1.989	1.02756	0.254854	0.846208	1.23757
XLOC_039374	BOC	chr1	58430992	58462115	3.20673	2.87099	0.0	2.58116	2.21536	2.62449	3.06694	2.49509	3.33371
XLOC_039375	BOC	chr1	58462272	58462924	0.0	1.31158	0.0	3.5374	3.30058	4.53621	3.67329	3.34045	4.5897
XLOC_079865	BOD1L	chr6	113638536	113712254	47.3017	49.8687	53.8368	35.2874	28.6298	30.5518	36.4367	45.9547	32.4002
XLOC_082199	BOD1L	chr6	113638536	113712254	3.24988	0.0	0.0	2.32541	5.9854	7.31279	2.82383	3.79127	2.81529
XLOC_072710	BPGM	chr4	99194172	99259168	1.38782	2.57423	1.98947	3.31313	4.31377	3.27896	3.50446	3.99084	4.16335
XLOC_050619	BPI	chr13	67833805	67843617	0.0	0.407563	1.06589	2.14121	0.719387	1.2544	2.22397	0.406632	1.25007
XLOC_050600	BPIFA2A;BPIFA2B;BPIFA2C;BPIFA3;BPIFA1;BPIFB1	chr13	62964898	63307362	6.88393	8.61186	10.4242	8.26521	6.92135	7.44886	5.67249	5.23207	6.23472
XLOC_052182	BPIFA2A;BPIFA2B;BPIFA2C;BPIFA3;BPIFA1;BPIFB1	chr13	62964898	63307362	4.65717	5.84961	8.01371	1.41913	1.01901	1.06268	0.674902	0.837815	1.30152
XLOC_052183	BPIFA2A;BPIFA2B;BPIFA2C;BPIFA3;BPIFA1;BPIFB1	chr13	62964898	63307362	0.0	0.965179	1.89324	1.66319	1.51456	1.33985	2.19716	0.807528	1.55074
XLOC_052184	BPIFA2A;BPIFA2B;BPIFA2C;BPIFA3;BPIFA1;BPIFB1	chr13	62964898	63307362	1.68296	0.503348	1.31645	1.65931	1.71093	1.92119	2.13759	0.926127	1.24973
XLOC_052185	BPIFA2A;BPIFA2B;BPIFA2C;BPIFA3;BPIFA1;BPIFB1	chr13	62964898	63307362	1.54617	1.61945	2.21868	1.77959	1.74126	1.69094	1.79624	0.856995	1.19572
XLOC_052186	BPIFA2A;BPIFA2B;BPIFA2C;BPIFA3;BPIFA1;BPIFB1	chr13	62964898	63307362	9.68802	3.56654	7.57895	4.07496	3.44107	3.40492	3.86067	3.13536	2.86561

XLOC_052187	BPIFA2A;BPIFA2B;BPIFA2C;BPIFA3;BPIFA1;BPIFB1	chr13	62964898	63307362	5.23136	6.39619	3.68411	5.913	4.06439	3.92088	2.847	3.48897	2.97408
XLOC_052188	BPIFA2A;BPIFA2B;BPIFA2C;BPIFA3;BPIFA1;BPIFB1	chr13	62964898	63307362	4.59936	3.20996	2.39867	2.95466	2.63921	3.26313	4.03923	1.61212	3.34931
XLOC_052189	BPIFA2A;BPIFA2B;BPIFA2C;BPIFA3;BPIFA1;BPIFB1	chr13	62964898	63307362	8.90214	6.54626	5.66141	5.67061	5.3296	3.5161	3.80845	3.32477	6.73618
XLOC_052190	BPIFA2A;BPIFA2B;BPIFA2C;BPIFA3;BPIFA1;BPIFB1	chr13	62964898	63307362	8.21449	6.25432	4.0894	2.74456	3.27281	2.94665	2.78263	2.39374	1.76207
XLOC_050907	BPIFB2	chr13	62864783	62867408	0.0	0.0	0.0	0.218299	0.188357	0.0	0.0	0.0	0.0
XLOC_041884	BRB	chr10	26280329	26376029	7.10795	4.93352	7.815	2.48479	2.00237	2.15495	1.93038	1.84667	1.53087
XLOC_064010	BRCA1	chr19	43736268	43768676	1.74365	1.70761	1.40079	2.83583	3.93621	4.54389	5.44534	2.42626	5.60178
XLOC_064275	BRCA1	chr19	43736268	43768676	3.30735	6.76078	5.5996	18.6409	18.3272	17.2784	20.6723	20.0424	19.6225
XLOC_065188	BRCA1	chr19	43731172	43731802	3.06156	2.28761	4.78616	1.50817	1.66667	1.42363	1.09702	1.21499	1.86754
XLOC_065189	BRCA1	chr19	43732101	43733919	1.7422	0.521272	2.04505	0.390552	0.512583	0.271888	0.47777	0.437968	0.571601
XLOC_065190	BRCA1	chr19	43734261	43734931	0.0	0.0	1.10328	0.379259	0.659164	0.583769	0.1267	0.560893	0.861255
XLOC_065191	BRCA1	chr19	43734982	43735288	9.70667	2.89387	0.0	7.8147	11.4215	6.89616	12.7474	12.5279	10.4646
XLOC_065192	BRCA1	chr19	43735428	43736074	4.44191	11.0639	13.8889	24.535	26.61	24.6406	30.9324	26.752	28.5199
XLOC_064011	BRCA1;NBR1;TMEM106A	chr19	43782212	43837787	0.443191	0.0	0.0	0.344521	0.487939	0.477323	0.353006	0.253436	0.310529
XLOC_065196	BRCA1;NBR1;TMEM106A	chr19	43782212	43837787	0.0	0.0	0.0	0.0	0.0	0.0	0.141494	0.0	0.0
XLOC_065197	BRCA1;NBR1;TMEM106A	chr19	43782212	43837787	0.0	0.0	0.0	0.350167	0.0	0.0	0.0	0.0	0.0
XLOC_064009	BRCA1;RND2;VAT1	chr19	43689650	43730931	2.86423	3.03993	1.42662	1.42389	1.24765	1.47746	1.05865	2.67653	1.53727
XLOC_070281	BRDT;EPHX4	chr3	51521249	51560658	17.4048	14.7795	7.31445	8.18505	8.7921	10.8431	4.43894	4.51885	4.5891

XLOC_071035	BRDT;EPHX4	chr3	51521249	51560658	0.0	0.0	2.2809	0.522778	0.674356	1.19771	0.765948	0.285136	0.506784
XLOC_044214	BRE	chr11	71750995	71789642	0.537911	1.69048	1.05255	1.23086	2.11418	1.51261	0.764531	1.00272	1.79046
XLOC_092073	BRS3;HTATSF1	chrX	20059549	20097671	0.327473	0.685941	1.28148	0.264307	0.154291	0.10228	0.0599905	0.0329731	0.114649
XLOC_065841	BSDC1;BSDC1	chr2	121956261	122014537	27.0919	26.2836	22.1945	29.8257	36.7014	35.1059	37.4143	32.3407	44.8234
XLOC_068440	BSDC1;BSDC1	chr2	121956261	122014537	3.12453	3.72943	0.0	1.67634	4.79864	2.23807	4.35178	2.73695	2.97861
XLOC_068441	BSDC1;BSDC1	chr2	121956261	122014537	0.0	0.0	0.0	2.71978	2.83424	1.52359	6.20807	2.83226	2.60295
XLOC_068442	BSDC1;BSDC1	chr2	121956261	122014537	5.59654	2.50605	0.0	1.7521	1.72347	3.15597	2.20493	1.91386	0.486294
XLOC_068443	BSDC1;BSDC1	chr2	121956261	122014537	2.03514	1.82367	3.17911	3.64282	3.62478	3.14392	4.515	3.00952	3.00928
XLOC_068444	BSDC1;BSDC1	chr2	121956261	122014537	0.0	1.0759	0.0	1.9343	1.10374	4.78443	1.24549	2.09452	4.68019
XLOC_068445	BSDC1;BSDC1	chr2	122014808	122015195	9.45288	5.6422	17.2129	7.32723	7.985	9.99613	10.1463	7.66639	12.0132
XLOC_068446	BSDC1;BSDC1	chr2	122015766	122016036	6.35475	5.68347	0.0	1.13935	5.74346	3.20875	1.05631	1.79959	6.56121
XLOC_068447	BSDC1;BSDC1	chr2	122016234	122016517	5.71993	1.7051	4.45803	2.0488	4.32142	1.73617	2.87418	1.08533	1.47768
XLOC_082978	BSG	chr7	44818362	44831217	43.0862	38.9498	32.4938	62.8011	69.6702	67.6394	68.6505	74.7851	62.9267
XLOC_084802	BSG	chr7	44816719	44817071	3.71583	0.0	5.79694	8.30681	6.82041	8.71959	5.12561	7.90614	4.17833
XLOC_084803	BSG	chr7	44817176	44817553	3.29416	3.93196	0.0	12.9625	9.09717	15.158	8.29372	7.3617	5.99138
XLOC_084804	BSG	chr7	44818050	44818215	0.0	48.2762	0.0	42.4436	25.1895	57.256	31.2012	36.2498	38.4738
XLOC_084805	BSG	chr7	44818362	44831217	0.0	2.42439	6.33787	16.0915	12.7607	10.6271	12.6057	9.09557	9.77474
XLOC_084806	BSG	chr7	44818362	44831217	0.0	2.03038	0.0	3.04265	1.82554	3.47702	3.2433	2.97167	3.2403
XLOC_084807	BSG	chr7	44818362	44831217	5.08875	4.55112	0.0	5.46374	5.78668	4.64404	1.71894	7.28169	6.58024
XLOC_084808	BSG	chr7	44818362	44831217	0.729448	3.27286	1.71197	5.23109	4.28214	5.52996	4.31054	4.82358	3.25134
XLOC_084809	BSG	chr7	44818362	44831217	0.0	3.14688	4.11461	2.3575	3.85964	4.59768	3.70215	1.28987	2.0584
XLOC_084810	BSG	chr7	44818362	44831217	3.19234	3.34344	5.99638	4.75238	4.28695	5.31795	4.09431	3.82546	3.38098
XLOC_084811	BSG	chr7	44818362	44831217	0.0	0.0	6.4057	7.39389	11.0509	9.08664	4.02011	3.06258	4.23363
XLOC_055753	BTBD10	chr15	39782194	39784791	0.737632	0.0	0.0	14.0839	10.6804	14.7836	4.96168	6.94632	11.6682
XLOC_055754	BTBD10	chr15	39793233	39828098	0.0	0.0	0.0	1.28841	0.869703	1.81615	0.429035	0.316914	1.11406
XLOC_056432	BTBD10	chr15	39780221	39780504	0.0	0.0	0.0	3.0732	6.04998	3.47235	3.83224	1.08533	3.44792
XLOC_076869	BTBD11	chr5	70961558	70973484	0.0	0.488496	0.0	0.0	0.0	0.0	0.0	0.0	0.0

XLOC_069900	BTBD8	chr3	51415092	51476158	29.0586	31.4155	21.5647	37.4286	47.0349	47.6073	68.4339	54.2714	39.2725
XLOC_071019	BTBD8	chr3	51415092	51476158	3.83438	3.15335	4.4984	2.835	2.09696	2.94061	2.25626	3.91751	3.18049
XLOC_071020	BTBD8	chr3	51415092	51476158	14.9807	4.46791	5.84026	4.71202	6.73731	2.26325	9.2272	7.01444	7.08195
XLOC_071021	BTBD8	chr3	51415092	51476158	4.88566	2.91312	19.0422	2.18527	1.48329	2.9748	2.89629	3.73556	1.68538
XLOC_071022	BTBD8	chr3	51415092	51476158	0.0	4.07984	8.00121	3.97406	1.30996	0.69862	2.96156	2.32178	1.77568
XLOC_071023	BTBD8	chr3	51415092	51476158	2.41293	3.60705	3.77344	2.48618	2.63398	1.74887	3.37031	2.88413	2.63145
XLOC_071024	BTBD8	chr3	51415092	51476158	9.72531	7.98657	15.1896	5.65675	5.44435	5.74661	5.57116	5.48806	3.37896
XLOC_071025	BTBD8	chr3	51415092	51476158	15.1737	5.65779	5.91767	3.39208	2.60965	3.48188	2.61303	3.66567	3.60852
XLOC_071026	BTBD8	chr3	51415092	51476158	5.94019	1.77305	13.9093	2.65669	3.88264	4.56386	3.11147	4.05514	3.60511
XLOC_071027	BTBD8	chr3	51415092	51476158	7.21004	4.31257	4.22963	3.39255	3.02915	3.17883	2.85862	3.33399	3.85717
XLOC_079830	BTC	chr6	91440984	91441751	0.0	1.9851	0.0	1.38799	1.54217	1.36776	1.56801	0.871925	0.962879
XLOC_079831	BTC	chr6	91464145	91488460	15.1554	15.3741	16.8887	12.0487	14.4104	12.0664	9.42381	10.2771	11.9864
XLOC_080223	BTC	chr6	91464145	91488460	15.7876	15.6354	14.9089	53.7889	42.2908	48.0543	46.1373	58.5503	47.3256
XLOC_081666	BTC	chr6	91463005	91463660	1.45385	2.17281	0.0	2.34407	0.905078	0.901819	1.82587	2.4544	1.9009
XLOC_076753	BTG1	chr5	21884451	22344688	7.33286	13.5261	15.1092	0.0865562	0.0556749	0.0861171	0.0356659	0.0718285	0.0441332
XLOC_082943	BTNL9	chr7	41599206	41611752	0.0	2.89369	14.5462	41.0773	38.0885	29.5002	33.6237	31.5571	35.1071
XLOC_084695	BTNL9	chr7	41599206	41611752	0.0	3.4413	1.00001	3.89592	6.47848	5.29485	2.9927	2.41842	6.35879
XLOC_084702	BTNL9	chr7	41644097	41644959	0.0	0.306995	0.0	1.472	1.60329	0.957768	1.67079	1.02486	1.34442
XLOC_084703	BTNL9	chr7	41647951	41648435	8.8616	1.3234	3.46087	5.94853	6.8541	5.92696	6.27206	7.84732	10.3991
XLOC_041706	C10H14orf1	chr10	87859172	87931994	16.1963	4.34907	4.87494	0.297916	0.16294	0.475336	0.531673	0.16708	0.436044
XLOC_041578	C10H14orf101	chr10	69249620	69291782	3.53397	7.98564	8.30159	1.05684	1.02108	2.15884	0.913711	1.24626	1.64994
XLOC_041710	C10H14orf118	chr10	88452955	88503820	56.7694	78.2646	45.6182	14.3728	19.7335	13.5229	24.0051	13.978	29.6637
XLOC_041711	C10H14orf118	chr10	88452955	88503820	19.4167	29.9969	18.5586	5.56	9.76659	5.81252	9.0615	6.54983	11.1103
XLOC_042106	C10H14orf118	chr10	88452955	88503820	14.1942	15.3736	10.7804	5.4306	6.71481	5.82274	6.82798	5.42543	6.70338
XLOC_043288	C10H14orf118	chr10	88452955	88503820	3.58956	2.14635	1.87116	0.428808	0.653098	0.247788	0.862722	1.07274	0.83513
XLOC_041882	C10H14orf176;ARHGEF40	chr10	25991755	26079022	3.40956	0.784865	1.23169	0.13524	0.0824232	0.136581	0.0721559	0.0792865	0.0918428
XLOC_041585	C10H14orf37	chr10	70440844	70599205	0.245426	0.384118	1.02136	1.79246	1.49884	2.08397	1.63981	2.32443	1.08821
XLOC_041982	C10H14orf37	chr10	70440844	70599205	0.210329	1.39195	0.74218	1.70697	1.70619	2.11885	1.45716	1.68423	1.47506

XLOC_042919	C10H14orf37	chr10	70664512	70665820	8.80085	5.2655	7.3776	5.4103	5.51535	5.9445	5.9548	3.78371	5.22129
XLOC_044221	C11H2orf18;CENPA	chr11	72777929	72916590	9.6145	12.6684	11.3565	4.20673	3.23487	3.75955	3.75971	4.24102	3.47506
XLOC_044901	C11H2orf18;CENPA	chr11	72777929	72916590	0.0	1.59837	2.03793	1.23464	1.02492	1.35492	1.48507	0.927371	1.65623
XLOC_046536	C11H2orf18;CENPA	chr11	72777929	72916590	1.31639	3.1479	10.291	0.353825	1.12795	0.272389	1.06537	0.785599	0.45921
XLOC_046537	C11H2orf18;CENPA	chr11	72777929	72916590	11.3616	12.805	13.1978	12.6937	10.8452	11.6653	11.1274	14.5843	11.1436
XLOC_073191	C11H2orf28	chr4	59314396	59438540	9.19848	6.84793	2.35504	7.77293	5.01284	8.20429	2.60443	2.83289	4.53312
XLOC_044899	C11H2orf28;CAD	chr11	72390863	72411742	2.17596	5.5336	2.97958	38.1292	30.779	36.072	33.2831	49.6997	34.3075
XLOC_044276	C11H2orf43	chr11	78159142	78182352	3.33781	4.66315	2.63788	5.09112	5.20986	6.21532	7.15705	11.5724	5.17591
XLOC_044277	C11H2orf43	chr11	78188352	78192187	22.6209	20.3293	23.6358	20.1955	17.2247	17.9258	17.4765	18.1962	15.7364
XLOC_044930	C11H2orf43	chr11	78159142	78182352	13.4925	27.2613	29.9424	36.353	29.4715	30.6454	27.2939	37.6246	24.0869
XLOC_044507	C11H9orf116;MRPS2	chr11	106411480	106613460	2.13472	3.85326	1.38543	5.8565	5.5398	8.46709	3.17975	5.61891	4.62264
XLOC_045148	C11H9orf116;MRPS2	chr11	106411480	106613460	25.2034	23.0422	25.9979	18.0704	14.1597	16.2437	13.1957	17.6824	12.9643
XLOC_047408	C11H9orf116;MRPS2	chr11	106411480	106613460	4.22421	1.0832	2.83292	0.378716	0.283689	0.439035	0.439921	0.181673	0.26386
XLOC_044429	C11H9orf16	chr11	98795638	98808335	54.512	56.6111	57.187	23.2204	29.7024	26.1963	33.2698	24.1903	26.8463
XLOC_047233	C11H9orf16	chr11	98795638	98808335	7.2243	1.61923	2.82288	1.4554	1.54159	2.04935	3.05847	0.535747	2.98723
XLOC_047234	C11H9orf16	chr11	98795638	98808335	0.0	2.0997	2.74509	3.14579	2.15534	1.07784	1.52133	1.70478	3.04462
XLOC_050607	C13H20orf118;DSN1;RPN2;SAMHD1;NDRG3;RBL1	chr13	66430786	66846017	20.1282	15.255	18.327	59.6607	38.0346	52.821	47.3497	65.755	63.5919
XLOC_050608	C13H20orf118;DSN1;RPN2;SAMHD1;NDRG3;RBL1	chr13	66430786	66846017	1.16749	1.5161	0.0	8.41702	3.76454	4.64857	3.52372	5.26405	5.56678
XLOC_050920	C13H20orf118;DSN1;RPN2;SAMHD1;NDRG3;RBL1	chr13	66430786	66846017	0.0	0.549224	0.0	1.15208	3.13722	2.65414	1.47366	0.726755	0.800005

XLOC_050921	C13H20orf118;DSN1;RPN2;SAMHD1;NDRG3;RBL1	chr13	66430786	66846017	0.0	0.29527	0.0	1.06184	0.848369	0.614252	0.178676	0.493076	1.46563
XLOC_050922	C13H20orf118;DSN1;RPN2;SAMHD1;NDRG3;RBL1	chr13	66430786	66846017	1.51146	1.35526	1.1814	3.45522	2.65485	4.30448	2.81925	3.8723	3.8923
XLOC_052311	C13H20orf118;DSN1;RPN2;SAMHD1;NDRG3;RBL1	chr13	66430786	66846017	2.1119	0.0	0.0	2.07944	2.28874	3.26172	1.68475	1.4564	2.02012
XLOC_052312	C13H20orf118;DSN1;RPN2;SAMHD1;NDRG3;RBL1	chr13	66430786	66846017	0.0	0.0	0.0	7.19977	3.96965	8.9127	7.91683	5.76685	5.33324
XLOC_052313	C13H20orf118;DSN1;RPN2;SAMHD1;NDRG3;RBL1	chr13	66430786	66846017	0.0	0.0	0.0	1.26615	1.74325	1.45108	1.48635	1.10604	1.96434
XLOC_052314	C13H20orf118;DSN1;RPN2;SAMHD1;NDRG3;RBL1	chr13	66430786	66846017	0.0	0.645908	0.0	2.12907	0.501974	0.22258	0.57449	1.27742	0.376011
XLOC_052315	C13H20orf118;DSN1;RPN2;SAMHD1;NDRG3;RBL1	chr13	66430786	66846017	0.0	0.0	0.0	3.4771	4.24006	4.46618	2.62522	2.2468	4.36944
XLOC_052316	C13H20orf118;DSN1;RPN2;SAMHD1;NDRG3;RBL1	chr13	66430786	66846017	0.0	1.69007	0.0	2.27907	2.17906	2.32173	0.990898	5.5302	1.96434
XLOC_052317	C13H20orf118;DSN1;RPN2;SAMHD1;NDRG3;RBL1	chr13	66430786	66846017	16.4257	2.45035	0.0	29.5756	19.646	24.7818	19.4305	28.3288	29.6354

XLOC_052318	C13H20orf118;DSN1;RPN2;SAMHD1;NDRG3;RBL1	chr13	66430786	66846017	1.45972	3.05419	2.28215	17.1284	11.6994	11.7706	14.1406	14.9298	14.2504
XLOC_052319	C13H20orf118;DSN1;RPN2;SAMHD1;NDRG3;RBL1	chr13	66430786	66846017	0.70048	0.628595	0.548012	4.0816	3.12592	3.2741	2.73982	3.36995	4.10217
XLOC_052320	C13H20orf118;DSN1;RPN2;SAMHD1;NDRG3;RBL1	chr13	66430786	66846017	1.11123	0.665012	0.579774	3.05592	2.96584	3.16136	2.33916	2.38571	2.98221
XLOC_052321	C13H20orf118;DSN1;RPN2;SAMHD1;NDRG3;RBL1	chr13	66430786	66846017	1.25897	1.88192	0.98436	9.92579	8.34046	7.42788	8.84066	11.0283	13.7272
XLOC_052322	C13H20orf118;DSN1;RPN2;SAMHD1;NDRG3;RBL1	chr13	66430786	66846017	0.0	0.0	0.0	6.86799	3.7379	4.41678	1.59051	2.29356	3.26122
XLOC_052323	C13H20orf118;DSN1;RPN2;SAMHD1;NDRG3;RBL1	chr13	66430786	66846017	0.0	1.54204	5.37703	12.3226	10.9527	13.4928	7.98486	13.1104	15.8778
XLOC_052324	C13H20orf118;DSN1;RPN2;SAMHD1;NDRG3;RBL1	chr13	66430786	66846017	0.0	0.0950653	0.0	1.22509	0.673625	1.09154	1.01866	1.47163	1.22351
XLOC_052325	C13H20orf118;DSN1;RPN2;SAMHD1;NDRG3;RBL1	chr13	66430786	66846017	0.0	0.0	0.0	3.16875	3.21384	2.63595	1.39992	2.50577	5.85964
XLOC_052326	C13H20orf118;DSN1;RPN2;SAMHD1;NDRG3;RBL1	chr13	66430786	66846017	0.0	1.44694	0.0	6.51225	3.31593	4.92583	3.28966	3.24797	5.8602

XLOC_052327	C13H20orf118;DSN1;RPN2;SAMHD1;NDRG3;RBL1	chr13	66430786	66846017	0.0	0.0	0.0	0.97835	0.485325	0.322447	0.977998	0.773837	0.679856
XLOC_052328	C13H20orf118;DSN1;RPN2;SAMHD1;NDRG3;RBL1	chr13	66430786	66846017	0.95354	0.0	0.0	0.0854567	0.521506	0.0	0.0	0.0	0.166537
XLOC_052329	C13H20orf118;DSN1;RPN2;SAMHD1;NDRG3;RBL1	chr13	66430786	66846017	0.0	0.0	0.0	0.394879	0.773771	0.570734	0.198943	0.109878	0.288465
XLOC_052330	C13H20orf118;DSN1;RPN2;SAMHD1;NDRG3;RBL1	chr13	66430786	66846017	0.0	0.0	0.0	3.05697	2.09592	0.69862	0.888468	0.331682	1.18379
XLOC_052331	C13H20orf118;DSN1;RPN2;SAMHD1;NDRG3;RBL1	chr13	66430786	66846017	0.0	1.1199	0.0	3.69275	1.14817	2.29773	0.32346	3.62933	0.649389
XLOC_052332	C13H20orf118;DSN1;RPN2;SAMHD1;NDRG3;RBL1	chr13	66430786	66846017	0.0	0.0	0.0	7.56331	4.6405	4.77098	0.398917	2.24894	3.24245
XLOC_052333	C13H20orf118;DSN1;RPN2;SAMHD1;NDRG3;RBL1	chr13	66430786	66846017	0.0	0.0	0.0	2.05366	1.79036	2.07935	0.518649	0.57239	1.08392
XLOC_052334	C13H20orf118;DSN1;RPN2;SAMHD1;NDRG3;RBL1	chr13	66430786	66846017	0.0	0.0	1.3335	0.381998	0.599871	0.265358	0.0	0.255821	0.521242
XLOC_052335	C13H20orf118;DSN1;RPN2;SAMHD1;NDRG3;RBL1	chr13	66430786	66846017	0.0	0.0	0.0	0.903601	1.01449	1.12165	0.392815	0.793617	0.440501

XLOC_052336	C13H20orf118;DSN1;RPN2;SAMHD1;NDRG3;RBL1	chr13	66430786	66846017	0.0	0.0	0.0	0.407785	0.880614	1.64033	0.0	0.223991	0.593971
XLOC_052337	C13H20orf118;DSN1;RPN2;SAMHD1;NDRG3;RBL1	chr13	66430786	66846017	0.0	0.299178	0.0	0.448304	0.862567	0.363986	0.502147	0.703311	0.524812
XLOC_052338	C13H20orf118;DSN1;RPN2;SAMHD1;NDRG3;RBL1	chr13	66430786	66846017	1.6451	0.0	0.965565	3.06101	2.32373	2.73892	1.73017	2.19252	2.05121
XLOC_052339	C13H20orf118;DSN1;RPN2;SAMHD1;NDRG3;RBL1	chr13	66430786	66846017	0.0	1.35234	0.0	1.0131	1.40032	1.16443	0.600364	2.4489	0.983805
XLOC_052340	C13H20orf118;DSN1;RPN2;SAMHD1;NDRG3;RBL1	chr13	66430786	66846017	0.0	0.0	0.0	8.19016	12.854	9.11706	10.2934	10.1043	7.79538
XLOC_052341	C13H20orf118;DSN1;RPN2;SAMHD1;NDRG3;RBL1	chr13	66430786	66846017	0.0	0.0	0.0	42.1045	33.8125	30.1734	23.7062	23.4635	55.5195
XLOC_052342	C13H20orf118;DSN1;RPN2;SAMHD1;NDRG3;RBL1	chr13	66430786	66846017	0.0	3.56401	0.0	7.58584	11.4534	5.96621	10.4211	2.18145	9.20608
XLOC_052343	C13H20orf118;DSN1;RPN2;SAMHD1;NDRG3;RBL1	chr13	66430786	66846017	0.0	0.0	2.39662	2.1973	0.943832	1.88638	1.60571	0.598262	2.39538
XLOC_052344	C13H20orf118;DSN1;RPN2;SAMHD1;NDRG3;RBL1	chr13	66430786	66846017	0.0	0.561362	0.293646	0.874828	0.323982	0.390521	0.171688	0.490853	0.525364

XLOC_052345	C13H20orf118;DSN1;RPN2;SAMHD1;NDRG3;RBL1	chr13	66430786	66846017	0.0	0.508732	0.665292	0.889374	1.0461	1.09174	0.649354	0.599441	1.21537
XLOC_052346	C13H20orf118;DSN1;RPN2;SAMHD1;NDRG3;RBL1	chr13	66430786	66846017	0.0	1.65851	0.722938	0.911216	1.87805	1.24632	1.2559	0.739079	1.45302
XLOC_052347	C13H20orf118;DSN1;RPN2;SAMHD1;NDRG3;RBL1	chr13	66430786	66846017	1.60554	1.91933	2.50976	1.86929	1.62224	1.49247	2.72893	0.954881	2.79745
XLOC_052348	C13H20orf118;DSN1;RPN2;SAMHD1;NDRG3;RBL1	chr13	66430786	66846017	0.831804	0.248782	0.0	0.820115	0.975797	1.12226	1.28312	0.749114	1.30806
XLOC_052349	C13H20orf118;DSN1;RPN2;SAMHD1;NDRG3;RBL1	chr13	66430786	66846017	2.71402	0.810265	2.11886	0.728421	2.09106	1.11374	1.66596	1.06187	1.64856
XLOC_052350	C13H20orf118;DSN1;RPN2;SAMHD1;NDRG3;RBL1	chr13	66430786	66846017	0.0	0.343044	0.299074	0.788193	0.809889	1.15341	0.629426	0.615247	1.10357
XLOC_052351	C13H20orf118;DSN1;RPN2;SAMHD1;NDRG3;RBL1	chr13	66430786	66846017	0.0	2.23395	0.0	0.673146	3.93009	7.54417	7.38176	0.701444	3.21907
XLOC_052352	C13H20orf118;DSN1;RPN2;SAMHD1;NDRG3;RBL1	chr13	66430786	66846017	0.0	0.0	0.0	1.09162	1.42689	0.84185	0.183598	1.52024	0.3545
XLOC_052353	C13H20orf118;DSN1;RPN2;SAMHD1;NDRG3;RBL1	chr13	66430786	66846017	0.935047	0.279632	0.731342	0.167602	0.511468	0.4849	0.0846843	0.186895	0.898251

XLOC_052354	C13H20orf118;DSN1;RPN2;SAMHD1;NDRG3;RBL1	chr13	66430786	66846017	0.0	0.703819	1.84072	1.68735	1.19334	0.609461	0.31837	0.234562	1.027
XLOC_050561	C13H20orf195	chr13	54585118	54591182	25.1134	19.4086	19.3762	22.8082	20.3228	22.0577	20.5779	20.1262	23.1222
XLOC_050860	C13H20orf195	chr13	54585118	54591182	24.6392	39.8466	24.0757	43.6205	34.1484	40.0411	46.0116	57.4778	44.9473
XLOC_050428	C13H20orf94	chr13	3633941	3650976	2.53016	1.19502	3.47102	3.46128	3.79122	3.27694	2.41444	2.22254	2.65267
XLOC_051086	C13H20orf94	chr13	3613018	3613323	0.0	0.0	3.80843	2.62232	5.19146	1.9832	4.55132	6.53722	3.79211
XLOC_051087	C13H20orf94	chr13	3651258	3651909	2.93127	3.50464	0.0	3.01944	2.28082	3.63625	2.36606	2.47394	2.17173
XLOC_053269	C14H8orf34	chr14	34673898	34748450	105.182	111.103	110.472	104.505	120.568	111.936	120.682	120.167	145.115
XLOC_053270	C14H8orf34	chr14	34774577	34778138	0.0	0.250207	0.654409	3.30459	4.16243	3.59216	1.6026	2.4292	5.84549
XLOC_053271	C14H8orf34	chr14	34820155	34946047	1.62722	0.486301	0.0	0.0	0.505802	0.168046	0.145509	0.322506	0.566998
XLOC_053547	C14H8orf34	chr14	34412496	34502419	52.7862	61.1225	51.2311	27.7004	38.3332	33.7579	34.2349	25.3027	46.6975
XLOC_053548	C14H8orf34	chr14	34527232	34542650	0.275986	0.165178	0.216011	0.717793	2.31988	0.919751	0.556679	0.806391	1.86035
XLOC_053549	C14H8orf34	chr14	34543992	34549639	0.0	0.201719	0.0	0.55715	2.11808	1.13388	0.544773	0.300876	0.956648
XLOC_054192	C14H8orf34	chr14	34412496	34502419	5.41775	4.21194	5.9315	0.776756	1.35317	1.34736	1.6637	0.540457	1.32409
XLOC_054193	C14H8orf34	chr14	34502863	34504320	3.89585	1.66502	2.17738	0.698585	0.43623	0.405019	0.253802	0.167642	0.535389
XLOC_054194	C14H8orf34	chr14	34670114	34670581	0.0	0.0	1.82526	0.0	0.0	0.240303	0.0	0.0	0.0
XLOC_054195	C14H8orf34	chr14	34672157	34673787	0.982321	1.17559	1.53735	0.484431	0.231108	0.357578	0.44851	0.493548	0.257781
XLOC_054196	C14H8orf34	chr14	34673898	34748450	0.0	1.27104	6.64281	2.28712	0.649131	1.73373	1.45833	0.819406	1.83948
XLOC_054197	C14H8orf34	chr14	34748891	34749915	3.33873	4.24392	5.2233	3.36662	3.6555	3.72486	3.10514	1.41984	4.01065
XLOC_054198	C14H8orf34	chr14	34750065	34750291	19.9917	2.98607	0.0	2.71307	9.65934	3.00894	4.02179	2.76906	4.29192
XLOC_054199	C14H8orf34	chr14	34753841	34754218	3.29416	0.982991	7.71124	3.53524	6.31748	6.06319	2.00193	1.2803	5.42077
XLOC_054200	C14H8orf34	chr14	34756317	34756886	0.0	1.05048	0.0	0.629583	1.3645	0.90689	0.313613	0.17391	2.60178
XLOC_054201	C14H8orf34	chr14	34768868	34769942	0.0	0.472066	0.0	1.13177	0.987809	0.90114	0.501646	1.0271	1.37908
XLOC_054202	C14H8orf34	chr14	34773421	34773854	0.0	0.0	0.0	0.939585	1.2145	1.34743	0.922336	0.771203	2.50678
XLOC_054203	C14H8orf34	chr14	34773980	34774269	0.0	0.0	0.0	2.93587	7.03006	2.76766	1.83714	2.5986	6.12184
XLOC_054204	C14H8orf34	chr14	34774577	34778138	0.0	0.0	0.0	0.631524	1.99918	0.483687	0.207601	0.23107	0.817501
XLOC_055770	C15H11orf30	chr15	56664724	56694565	0.72213	0.561927	0.791394	0.259089	0.0681483	0.451603	0.106195	0.0437255	0.0632473
XLOC_055523	C15H11orf49	chr15	78046969	78052031	0.0	0.0	0.0	1.43149	3.36024	2.0387	0.68691	1.92941	2.76715

XLOC_055852	C15H11orf49	chr15	78159426	78174166	9.48811	4.30321	5.95257	11.5975	9.13262	13.4478	10.2727	11.4259	10.7484
XLOC_057036	C15H11orf49	chr15	78099262	78099923	0.0	0.0	1.12297	0.386029	0.782614	0.891167	0.515622	0.142686	1.37747
XLOC_055434	C15H11orf58	chr15	36177159	36235607	5.94902	5.83259	9.74417	8.52093	10.1645	6.33201	8.35542	9.87767	8.85972
XLOC_055738	C15H11orf58	chr15	36239886	36266365	27.9889	26.9573	26.6892	8.76367	9.80298	10.6773	8.89812	6.28058	11.2011
XLOC_056320	C15H11orf58	chr15	36236400	36237750	2.42596	1.0886	1.42358	0.380615	0.332628	0.441225	0.165791	0.304287	0.424235
XLOC_056296	C15H11orf63	chr15	34055048	34055291	0.0	0.0	0.0	7.39389	1.22787	1.65212	1.34004	0.765644	0.705605
XLOC_056297	C15H11orf63	chr15	34064579	34065269	0.0	0.0	0.0	1.46013	0.317337	1.40506	0.488238	0.675217	0.0
XLOC_056298	C15H11orf63	chr15	34066104	34066495	0.0	0.924329	0.0	3.32408	0.237928	0.317038	0.0	0.0	0.0
XLOC_055427	C15H11orf63;BSX	chr15	34082047	34143701	51.7605	45.1284	58.7327	86.799	76.7713	86.3838	98.9952	104.301	91.4437
XLOC_056299	C15H11orf63;BSX	chr15	34082047	34143701	1.68044	4.01712	2.62617	1.95599	1.30568	2.25563	2.10136	2.16307	3.2193
XLOC_056300	C15H11orf63;BSX	chr15	34082047	34143701	0.0	0.0	0.0	0.936195	1.00942	2.68638	1.14895	0.512334	0.681219
XLOC_057900	C16H1orf114	chr16	37867988	37882181	40.9	39.5474	44.83	16.3711	15.8368	15.6818	21.5276	19.05	15.1134
XLOC_058930	C16H1orf114	chr16	37882303	37884324	91.9801	84.3081	91.1181	61.5306	55.1958	54.4054	64.5186	74.2703	53.8287
XLOC_057752	C16H1orf116	chr16	4683624	4707648	13.4085	23.3702	22.6871	50.7646	39.254	43.491	45.7152	57.4623	40.7826
XLOC_057935	C16H1orf158	chr16	56075367	56190379	3.00533	10.2107	6.08848	4.18405	6.49695	5.54649	2.62911	2.65357	5.51314
XLOC_059200	C16H1orf158	chr16	56075367	56190379	3.51811	4.19821	0.0	0.0	0.538836	1.07784	0.608557	0.0	0.0
XLOC_058088	C16H1orf95	chr16	30315022	30388471	60.8027	48.1125	48.2909	32.0666	30.7375	34.8641	38.4938	35.5763	30.4605
XLOC_058742	C16H1orf95	chr16	30312446	30314760	19.7868	21.6764	22.8352	19.6693	19.4079	19.3422	19.1041	19.7187	17.9638
XLOC_076364	C17H12orf34	chr5	73292723	73314067	37.9204	47.3664	44.4812	62.5081	76.9981	71.4048	71.7462	66.2916	80.0371
XLOC_059877	C17H12orf34;TRPV4;MVK	chr17	65651655	65864520	33.4253	38.6141	40.1828	16.4753	15.1718	15.8048	21.8568	19.6739	15.201
XLOC_060284	C17H12orf34;TRPV4;MVK	chr17	65651655	65864520	30.4791	31.3558	30.3836	5.7385	4.94894	5.16248	7.00567	5.36178	6.7856

XLOC_060285	C17H12orf34;TRPV4;MVK	chr17	65651655	65864520	29.6771	39.7496	40.1338	29.9814	23.9949	26.5769	34.0422	38.8908	35.716
XLOC_061823	C17H12orf34;TRPV4;MVK	chr17	65651655	65864520	1.97225	0.884611	3.85593	0.530266	0.924337	0.715689	0.713749	0.394023	0.860985
XLOC_061824	C17H12orf34;TRPV4;MVK	chr17	65651655	65864520	0.0	1.46728	2.87803	0.659643	0.669673	0.508131	0.442091	0.488839	1.28445
XLOC_061819	C17H12orf43	chr17	65438144	65438698	18.2424	21.2579	14.2548	39.2016	41.4705	41.7722	42.0985	38.774	35.2532
XLOC_060306	C17H22orf13;UPB1;SNRPD3	chr17	73493751	73574973	0.0	0.363181	0.0	0.0	0.0317461	0.0	0.0	0.0406944	0.0
XLOC_059884	C17H22orf31;EMID1	chr17	70516700	70657796	1.03843	3.72621	1.62422	0.0	0.0	0.0	0.0	0.0	0.0
XLOC_059794	C17H4orf45	chr17	40923415	40961027	13.3235	11.4987	11.6603	25.8084	21.1649	23.7622	29.0735	31.0477	28.3999
XLOC_060184	C17H4orf45	chr17	40923415	40961027	50.6949	30.6802	42.0009	27.0137	25.8094	26.8826	28.3159	31.1566	36.9021
XLOC_061426	C17H4orf45	chr17	40961219	40962549	2.46735	0.738108	1.44785	3.42863	2.46456	3.33336	2.41641	2.78494	4.0449
XLOC_062888	C18H16orf70	chr18	34896915	34897791	1.00663	1.20407	1.57454	0.992303	1.25784	1.46101	0.910467	1.00513	1.75775
XLOC_062889	C18H16orf70	chr18	34899153	34899503	0.0	1.1199	5.85668	1.67852	2.58339	2.29773	1.94076	1.45173	3.89634
XLOC_062890	C18H16orf70	chr18	34899651	34899882	47.0108	8.42231	7.33889	5.09524	11.2018	13.2168	3.03912	6.96619	8.88223
XLOC_062891	C18H16orf70	chr18	34899989	34900731	11.1369	4.06947	8.70785	2.99337	4.63012	3.45873	4.79221	2.09457	3.34637
XLOC_062892	C18H16orf70	chr18	34900821	34901719	1.95344	2.62875	4.58342	3.32621	4.12007	2.22807	3.18205	2.14637	2.89971
XLOC_063145	C18H19orf47	chr18	49963195	49965134	4.05966	3.40116	1.90621	4.07721	5.19298	4.52016	4.71556	5.39047	5.75461
XLOC_062234	C18H19orf47;SERTAD1;SERTAD3;PLD3;SPTBN4;BLVRB	chr18	49979456	50092590	3.00903	1.48785	1.41305	2.95561	5.15219	4.0732	3.65177	2.6509	3.97724
XLOC_063146	C18H19orf47;SERTAD1;SERTAD3;PLD3;SPTBN4;BLVRB	chr18	49979456	50092590	1.14171	0.341366	0.0	0.409199	1.24698	1.41918	0.824056	0.341437	0.59782
XLOC_044840	C1D	chr11	66217488	66525667	2.57845	8.36439	8.1893	0.0604534	0.067198	0.0	0.0779442	0.135221	0.134021
XLOC_046413	C1D	chr11	66217488	66525667	0.0	1.09176	2.85478	0.0	0.0	0.0	0.0	0.0	0.0

XLOC_046414	C1D	chr11	66217488	66525667	0.0	0.435436	0.0	0.0	0.0	0.0	0.0	0.0	0.0
XLOC_046415	C1D	chr11	66217488	66525667	0.0	0.788408	1.03096	0.0	0.102726	0.136441	0.0	0.0	0.0
XLOC_037826	C1H3orf26	chr1	43933088	44005046	35.3309	37.348	29.8458	30.0367	28.1924	30.3821	32.5376	28.6275	30.312
XLOC_038333	C1H3orf26	chr1	43748031	43827784	27.0777	42.7169	45.8558	15.2289	14.0112	11.0581	15.6777	11.7601	16.2744
XLOC_038334	C1H3orf26	chr1	43748031	43827784	2.0404	0.609565	0.0	0.365307	0.474109	0.0	0.181091	0.0	0.354951
XLOC_038335	C1H3orf26	chr1	43881627	43888431	40.3443	21.8112	15.0106	25.9721	25.9159	29.5082	27.5112	22.954	22.5688
XLOC_038336	C1H3orf26	chr1	44086630	44102486	46.8202	59.1286	52.7434	23.4548	22.6416	22.9738	32.7891	24.2086	31.6487
XLOC_039224	C1H3orf26	chr1	43747124	43747797	0.0	2.09704	3.2906	0.25137	0.327685	0.2902	0.629909	0.975948	0.61162
XLOC_039225	C1H3orf26	chr1	43748031	43827784	3.56585	3.55378	1.85884	2.02346	2.13192	2.21554	3.4287	2.60525	2.28157
XLOC_039226	C1H3orf26	chr1	43748031	43827784	0.876143	1.04814	0.0	0.314106	0.342482	0.45452	0.635488	0.262872	0.306126
XLOC_039227	C1H3orf26	chr1	44005221	44005592	0.0	4.04185	2.64223	1.21138	1.29802	2.76886	0.880642	0.657404	1.17285
XLOC_039228	C1H3orf26	chr1	44006866	44007443	1.72421	1.54571	2.69491	0.926396	1.33883	1.42364	1.07737	0.853312	1.05102
XLOC_039229	C1H3orf26	chr1	44008317	44008715	3.00709	0.897537	2.34701	0.537943	1.38702	0.307982	1.0494	0.586272	1.5641
XLOC_039230	C1H3orf26	chr1	44009359	44011377	1.55491	1.86103	3.34638	0.801744	1.12888	1.1732	1.45817	0.625779	1.15655
XLOC_039231	C1H3orf26	chr1	44011548	44015425	1.94439	1.92029	1.06539	1.01149	1.3298	1.49924	0.928423	0.607917	1.12376
XLOC_039232	C1H3orf26	chr1	44015658	44016102	2.52465	3.01543	7.88555	0.6777	1.3636	0.777894	1.55492	0.0	2.63064
XLOC_039233	C1H3orf26	chr1	44016627	44017927	0.633034	1.13622	0.495282	0.454014	0.743809	0.723578	0.518886	0.3175	1.32828
XLOC_039234	C1H3orf26	chr1	44018090	44018549	0.0	0.716442	0.0	0.0	2.03822	0.0	0.42311	0.0	0.0
XLOC_039235	C1H3orf26	chr1	44018621	44019598	1.76512	1.05579	0.0	0.158202	0.896926	0.549396	0.400041	0.264776	1.00217
XLOC_039236	C1H3orf26	chr1	44020417	44021042	3.09431	1.38723	2.41863	0.692851	0.721835	0.639428	0.277105	0.460404	1.48296
XLOC_039237	C1H3orf26	chr1	44103433	44104547	1.51228	0.226169	0.591523	0.271118	0.295859	0.0785119	0.343543	0.227202	0.594703
XLOC_039238	C1H3orf26	chr1	44106124	44106647	1.97899	0.591223	0.0	0.354334	0.613418	1.01965	0.879178	0.195276	1.03303
XLOC_037899	C1H3orf43	chr1	71660693	71684490	0.0	0.264594	0.0	0.0792943	1.03743	0.642521	0.641605	0.530833	0.618224
XLOC_039631	C1H3orf43	chr1	71654673	71655545	0.0	0.302702	1.58335	0.362856	1.42293	0.209877	0.549282	0.707482	0.883779
XLOC_039632	C1H3orf43	chr1	71656484	71658420	0.0	0.365025	0.318237	0.7293	1.21267	0.761681	0.595081	0.818109	0.996299
XLOC_039633	C1H3orf43	chr1	71660693	71684490	0.910331	0.272247	0.0	0.163175	0.426897	1.13317	0.164967	0.36402	0.318031
XLOC_039634	C1H3orf43	chr1	71660693	71684490	0.0	0.287811	0.752732	0.690014	0.977463	0.798419	1.13255	0.480755	1.0085
XLOC_038024	C1H3orf58	chr1	125676967	125910497	2.83895	1.84316	1.47878	1.66123E-5	1.53135E-5	0.0	0.0260249	1.86523E-5	0.0
XLOC_038518	C1H3orf58	chr1	125676967	125910497	0.0	0.0528487	1.65766E	0.0520406	0.0454867	0.0	0.0	0.116549	0.0
XLOC_037940	C1H3orf70	chr1	82653323	82664585	6.24453	12.4358	17.8868	0.0	0.0	0.0	0.0	0.0	0.0
XLOC_037941	C1H3orf70	chr1	82670233	82696452	2.65544	7.9456	9.20325	0.0	0.0	0.0	0.0	0.0	0.0
XLOC_066339	C1QL2	chr2	71274111	71367576	3.40739	2.81533	2.8575	29.5719	20.1072	28.6522	24.6613	31.1277	23.3498
XLOC_067832	C1QL2	chr2	71274111	71367576	0.0	0.0	0.0	45.3429	13.1642	17.8044	28.3854	30.599	19.0659

XLOC_067833	C1QL2	chr2	71274111	71367576	0.0	0.0	0.0	11.4113	9.34432	10.4887	10.6161	14.2231	11.7802
XLOC_067834	C1QL2	chr2	71274111	71367576	0.0	0.0	0.0	10.1667	8.5447	9.54653	7.86038	13.6862	14.6392
XLOC_067835	C1QL2	chr2	71274111	71367576	0.0	0.0	0.915891	11.3343	10.2316	12.373	6.97084	14.0063	11.9577
XLOC_067836	C1QL2	chr2	71274111	71367576	0.0	0.0	0.0	8.02953	8.32513	6.23022	5.35504	7.72142	7.79781
XLOC_067837	C1QL2	chr2	71274111	71367576	0.0	0.0	0.0	5.81185	7.69289	5.99394	4.11753	7.2706	6.99058
XLOC_067838	C1QL2	chr2	71274111	71367576	0.0	0.0	0.0	12.1409	11.3728	7.21103	3.63698	11.6597	9.24745
XLOC_067839	C1QL2	chr2	71274111	71367576	0.0	0.0	0.0	8.84527	7.86679	6.39756	6.50916	11.2134	6.2088
XLOC_067840	C1QL2	chr2	71274111	71367576	0.0	0.0	0.0	6.26245	9.22352	7.58586	3.56892	3.12934	10.0687
XLOC_067841	C1QL2	chr2	71274111	71367576	0.0	0.0	0.0	4.90404	3.61477	3.97127	3.60224	4.00171	4.05921
XLOC_067842	C1QL2	chr2	71274111	71367576	0.0	0.0	0.0	3.50351	2.95846	3.96099	7.03427	2.1238	3.37049
XLOC_064049	C1QTNF1;RBFOX3	chr19	53931131	54034022	21.7218	26.1442	24.7696	16.2535	14.3689	16.77	15.8356	18.0884	17.6028
XLOC_064318	C1QTNF1;RBFOX3	chr19	53931131	54034022	16.6704	16.7822	21.1481	14.2386	14.6857	14.8928	14.3986	14.4201	18.1377
XLOC_065314	C1QTNF1;RBFOX3	chr19	53931131	54034022	8.53684	3.12029	2.22565	1.10511	1.11169	1.67231	0.858846	1.04258	1.57383
XLOC_065315	C1QTNF1;RBFOX3	chr19	53931131	54034022	2.7951	4.1814	4.00997	1.08604	0.767352	1.45374	0.766078	0.608739	0.815065
XLOC_065316	C1QTNF1;RBFOX3	chr19	53931131	54034022	3.75963	1.68651	0.73514	0.758124	0.58755	0.974813	0.425584	0.563566	0.902905
XLOC_065317	C1QTNF1;RBFOX3	chr19	53931131	54034022	1.68784	2.3564	3.52173	0.605305	0.44096	0.701853	0.564381	0.33891	0.295205
XLOC_079871	C1QTNF7	chr6	115361260	115441663	2.01757	3.98612	0.0	0.0	0.0	0.207804	0.358224	0.0	0.0
XLOC_082213	C1QTNF7	chr6	115275417	115276456	0.0	2.94477	0.641811	0.0	0.0	0.0	0.0	0.0	0.0716838
XLOC_048125	C1QTNF9;TNFRSF19	chr12	34393168	34714631	3.68776	1.94965	1.32881	0.199411	0.449135	0.347468	0.18	0.0	0.194503
XLOC_049036	C1QTNF9;TNFRSF19	chr12	34393168	34714631	0.0	0.0	1.06589	0.0	0.0	0.0	0.0	0.0	0.0
XLOC_049038	C1QTNF9;TNFRSF19	chr12	34393168	34714631	0.0	0.0	0.0	0.0	0.0	0.0	0.107666	0.0	0.0
XLOC_049039	C1QTNF9;TNFRSF19	chr12	34393168	34714631	1.75773	0.0	0.0	0.157396	0.0	0.0	0.0	0.0	0.0
XLOC_049040	C1QTNF9;TNFRSF19	chr12	34393168	34714631	0.0	0.377697	1.97559	0.0	0.0	0.0	0.0	0.0	0.0
XLOC_049041	C1QTNF9;TNFRSF19	chr12	34393168	34714631	0.0	1.34876	0.0	0.0	0.0	0.0	0.0	0.0	0.0
XLOC_049042	C1QTNF9;TNFRSF19	chr12	34393168	34714631	0.0	0.42939	0.0	0.0	0.0	0.148528	0.0	0.0	0.0
XLOC_049043	C1QTNF9;TNFRSF19	chr12	34393168	34714631	1.68799	0.504431	0.0	0.0	0.0	0.0	0.0	0.0	0.0

XLOC_049044	C1QTNF9;TNFRSF19	chr12	34393168	34714631	0.0	0.58103	0.759803	0.0	0.0	0.0	0.0	0.0	0.0
XLOC_049045	C1QTNF9;TNFRSF19	chr12	34393168	34714631	0.0	0.721215	1.88605	0.0	0.0	0.0	0.0	0.0	0.0
XLOC_049046	C1QTNF9;TNFRSF19	chr12	34393168	34714631	0.0	0.0	3.9291	0.0	0.0	0.0	0.0	0.0	0.0
XLOC_049048	C1QTNF9;TNFRSF19	chr12	34393168	34714631	0.0	0.591223	6.1846	0.0	0.0	0.20393	0.0	0.0	0.0
XLOC_049049	C1QTNF9;TNFRSF19	chr12	34393168	34714631	0.0	0.250509	0.655179	0.0	0.0	0.0	0.0	0.0	0.0
XLOC_049050	C1QTNF9;TNFRSF19	chr12	34393168	34714631	1.67237	0.499773	0.0	0.0	0.0	0.0	0.0	0.0	0.0
XLOC_049051	C1QTNF9;TNFRSF19	chr12	34393168	34714631	0.0	0.0	0.0	0.0	0.0	0.0	0.0	0.0	0.565522
XLOC_049052	C1QTNF9;TNFRSF19	chr12	34393168	34714631	2.43953	0.728495	0.0	0.0	0.0	0.0	0.0	0.0	0.211891
XLOC_049053	C1QTNF9;TNFRSF19	chr12	34393168	34714631	1.13454	0.339226	2.66157	0.101659	0.0885137	0.0	0.0	0.0	0.0
XLOC_049054	C1QTNF9;TNFRSF19	chr12	34393168	34714631	0.0	0.545075	1.42548	0.0	0.0	0.0	0.0	0.0	0.0
XLOC_049055	C1QTNF9;TNFRSF19	chr12	34393168	34714631	2.20006	1.31567	3.44093	0.0	0.0858449	0.0	0.0	0.0	0.0
XLOC_049056	C1QTNF9;TNFRSF19	chr12	34393168	34714631	0.0	1.86003	3.24284	0.0	0.0	0.0	0.0	0.0	0.0
XLOC_049057	C1QTNF9;TNFRSF19	chr12	34393168	34714631	0.0	1.10971	1.45105	0.0	0.0	0.0	0.0	0.0	0.161633
XLOC_076426	C1S;C1R;EMG1;PHB2;LPCAT3;C1RL	chr5	103692314	103856144	26.2155	23.7197	23.2769	20.6546	18.3821	19.1485	20.9576	32.6775	17.685
XLOC_078470	C1S;C1R;EMG1;PHB2;LPCAT3;C1RL	chr5	103692314	103856144	0.0	0.0	0.0	4.67795	4.41676	2.63289	7.57316	8.60673	4.48757
XLOC_038087	C2CD2	chr1	143600438	143702637	3.3705	2.97498	3.72889	1.39767	1.39136	1.72367	1.37386	1.14183	2.1761
XLOC_038088	C2CD2	chr1	143600438	143702637	5.26012	1.88748	10.6955	0.754183	1.39622	0.872267	0.665509	0.314966	0.642907
XLOC_038573	C2CD2	chr1	143600438	143702637	17.6015	15.7916	17.2642	12.4075	17.3917	18.8389	22.7601	20.3347	10.4862
XLOC_040552	C2CD2	chr1	143600438	143702637	3.03586	2.72214	3.55954	0.815741	0.472244	1.4118	0.408023	0.602497	1.45518
XLOC_040553	C2CD2	chr1	143600438	143702637	1.72011	6.16817	1.34427	1.69436	1.60283	1.06522	2.61043	0.851333	2.24695
XLOC_066540	C2H1orf135;PAQR7	chr2	127800965	127831882	18.9849	26.5469	19.4835	102.617	106.474	117.099	112.415	89.8634	139.026
XLOC_065876	C2H1orf201	chr2	129100910	129126277	16.8945	21.5313	21.4849	57.8594	33.22	45.6375	50.1718	74.9366	35.8114
XLOC_065877	C2H1orf201	chr2	129129442	129133676	5.98654	5.37193	2.92704	26.6973	25.2408	24.1657	18.362	15.891	25.8315
XLOC_068574	C2H1orf201	chr2	129095348	129096883	1.5751	1.41371	0.410831	1.36517	1.72886	1.52864	1.62908	1.47663	1.9746

XLOC_068575	C2H1orf201	chr2	129126414	129126612	30.2271	49.8937	11.856	73.5651	44.4279	80.1431	58.049	93.0876	42.8668
XLOC_068576	C2H1orf201	chr2	129126709	129127224	2.02328	0.60443	0.0	1.63012	2.03751	1.04218	1.07776	2.99309	0.175997
XLOC_068577	C2H1orf201	chr2	129127442	129127667	10.1229	0.0	0.0	1.83237	1.5044	5.07795	1.62776	3.73672	1.73857
XLOC_065687	C2H2orf76	chr2	71453332	71525165	16.4311	13.9227	16.952	15.8058	14.3996	13.9073	12.0796	13.782	16.9333
XLOC_067843	C2H2orf76	chr2	71453332	71525165	1.12348	1.34443	2.19767	2.11528	2.33347	2.80307	1.63932	1.80473	2.25972
XLOC_067844	C2H2orf76	chr2	71453332	71525165	0.0	0.0	0.0	1.54593	3.20179	1.72437	3.48791	2.39371	0.736781
XLOC_067845	C2H2orf76	chr2	71453332	71525165	0.784011	1.40699	3.06654	2.03801	1.77883	2.11618	1.70891	2.19796	2.12371
XLOC_067846	C2H2orf76	chr2	71453332	71525165	2.27265	0.679517	1.77716	1.42547	1.86168	1.17709	1.33292	1.69925	1.09081
XLOC_065473	C2H2orf88	chr2	6039644	6056796	19.9371	26.2313	21.2807	18.6401	19.4619	20.9303	25.9632	21.1727	23.2658
XLOC_066127	C2H2orf88	chr2	6098494	6130151	4.44575	4.85678	5.91573	1.50688	1.13647	1.39227	1.06082	0.597958	1.25017
XLOC_082794	C3	chr7	18991287	19001123	4.05825	10.6792	12.7011	0.67571	0.711584	0.421838	0.316964	0.988357	0.760441
XLOC_082795	C3	chr7	19003780	19008081	5.65772	6.61422	4.01351	6.12122	4.33701	5.3615	4.31992	7.24121	3.56118
XLOC_083426	C3	chr7	19013145	19023561	0.0	0.11274	0.294869	0.608173	0.532354	0.35293	0.310317	0.189573	0.296747
XLOC_084305	C3	chr7	19001229	19001954	1.2745	8.76348	8.96838	0.456731	0.496606	0.395725	0.688244	0.761062	0.333499
XLOC_070419	C3H1orf185	chr3	95734091	95756271	9.78814	13.2018	23.8779	53.8622	47.9649	44.1011	47.0856	61.4814	49.401
XLOC_070420	C3H1orf185	chr3	95756271	95820032	16.0554	21.3075	19.2537	12.8381	12.2852	12.5668	13.5156	16.4978	9.77694
XLOC_071752	C3H1orf185	chr3	95756271	95820032	2.18372	3.34909	4.70032	3.2804	3.02366	3.46823	2.12738	3.30039	2.36573
XLOC_071753	C3H1orf185	chr3	95756271	95820032	8.40072	7.53137	13.1313	6.92118	7.04743	4.16292	6.45417	6.32283	3.65829
XLOC_057753	C4BPB;C4BPA;PFKFB2	chr16	4726361	4790372	10.9402	14.2547	12.1611	7.39906	6.43332	7.42591	7.14017	7.54161	8.10501
XLOC_058003	C4BPB;C4BPA;PFKFB2	chr16	4726361	4790372	18.5058	16.0487	18.4473	5.6457	6.08197	5.36369	5.61276	5.4835	5.59686
XLOC_058284	C4BPB;C4BPA;PFKFB2	chr16	4726361	4790372	1.44152	2.15587	2.25541	0.71071	1.46686	1.1228	0.655396	0.0	0.755927
XLOC_058285	C4BPB;C4BPA;PFKFB2	chr16	4726361	4790372	5.02726	3.00592	2.94806	1.01343	1.07755	0.260234	0.679002	0.37539	0.438566
XLOC_058286	C4BPB;C4BPA;PFKFB2	chr16	4726361	4790372	2.35467	2.81709	3.6839	0.703553	0.982516	0.651871	0.49903	0.471541	0.617262
XLOC_058287	C4BPB;C4BPA;PFKFB2	chr16	4726361	4790372	7.0348	4.9968	3.43912	1.0246	1.16869	1.09484	0.637843	1.05533	0.768135
XLOC_058288	C4BPB;C4BPA;PFKFB2	chr16	4726361	4790372	4.90547	2.20123	5.27735	0.549751	0.528459	0.764718	0.44698	0.615264	0.268089
XLOC_058289	C4BPB;C4BPA;PFKFB2	chr16	4726361	4790372	2.41226	3.60741	6.28984	0.504533	1.13219	0.667736	0.803046	0.643933	0.913329
XLOC_058290	C4BPB;C4BPA;PFKFB2	chr16	4726361	4790372	4.71852	3.45075	5.74334	0.658115	0.94541	0.872268	0.430652	0.526636	0.733693
XLOC_058291	C4BPB;C4BPA;PFKFB2	chr16	4726361	4790372	4.77396	5.50812	6.93624	0.855947	1.06804	0.495944	1.17911	0.957204	0.834624

XLOC_072690	C4H7orf10	chr4	80910662	80954568	0.394883	0.354465	1.23613	1.41641	1.1157	1.76704	0.614111	0.595912	1.07117
XLOC_073218	C4H7orf10	chr4	81278263	81442817	9.07869	12.2717	12.5508	2.9035	2.66752	3.24142	3.94081	3.6533	3.17011
XLOC_072575	C4H7orf23	chr4	33310149	33502844	12.4931	16.525	16.1087	21.8205	18.5258	20.8668	24.3611	23.034	22.3622
XLOC_076316	C5H12orf28	chr5	43818880	43842725	0.0	0.448457	0.0	1.56798	0.822792	1.61083	1.18608	2.20889	1.0926
XLOC_076839	C5H12orf28	chr5	43744524	43782123	32.7748	38.5477	31.1073	43.3422	46.1355	43.4859	51.9924	52.6877	56.1995
XLOC_077808	C5H12orf28	chr5	43744524	43782123	16.9627	8.68354	9.46175	9.75781	17.5899	10.4592	16.4463	11.1783	18.9437
XLOC_077809	C5H12orf28	chr5	43744524	43782123	4.19962	6.27198	8.20104	4.69863	5.3647	5.83746	6.51519	4.75828	10.4077
XLOC_077810	C5H12orf28	chr5	43744524	43782123	0.0	24.9844	21.7686	21.6104	14.3281	15.293	22.98	12.6303	25.0628
XLOC_076392	C5H12orf39;GOLT1B;GYS2;PYROXD1;IAPP;RECQL;LDHB	chr5	88887274	89239319	4.35181	2.31999	4.82617	0.207671	0.146442	0.32749	0.362923	0.18216	0.164169
XLOC_078238	C5H12orf39;GOLT1B;GYS2;PYROXD1;IAPP;RECQL;LDHB	chr5	88887274	89239319	0.0	1.11845	0.0	0.0	0.0	0.0	0.0	0.0	0.0
XLOC_078239	C5H12orf39;GOLT1B;GYS2;PYROXD1;IAPP;RECQL;LDHB	chr5	88887274	89239319	1.29969	0.777183	0.0	0.0	0.0	0.0	0.0	0.0	0.0
XLOC_078240	C5H12orf39;GOLT1B;GYS2;PYROXD1;IAPP;RECQL;LDHB	chr5	88887274	89239319	1.13632	0.679601	0.0	0.0	0.0886515	0.0	0.0	0.0	0.0
XLOC_076275	C5H12orf54	chr5	31624226	32084977	18.1954	20.1423	16.5764	34.1276	35.7148	32.7173	34.6115	33.9655	38.2116
XLOC_076798	C5H12orf54	chr5	31624226	32084977	0.0	3.45325	2.53289	2.65949	2.13326	1.58947	1.17569	1.02778	1.49764
XLOC_077609	C5H12orf54	chr5	31624226	32084977	0.0	1.20748	2.36846	1.26645	0.945994	1.77905	0.821665	0.705554	0.705056
XLOC_077610	C5H12orf54	chr5	31624226	32084977	0.0	1.73935	1.01094	1.85334	1.51802	1.27542	0.529418	0.712752	0.621273
XLOC_076940	C5H12orf57;LRRC23;ENO2;ATN1;PTPN6	chr5	103862073	103911255	2.77527	4.96015	4.68383	17.4204	16.5347	16.9854	14.371	14.1232	18.0097
XLOC_076867	C5H12orf75	chr5	69109999	69340820	0.641457	1.72699	0.501867	0.172519	0.0502436	0.133303	0.175236	0.0643382	0.112159
XLOC_076506	C5H22orf40;PPARA	chr5	117126458	117273859	20.1414	18.9473	23.2326	6.81446	13.0606	7.76852	8.40935	6.31056	10.6215

XLOC_078835	C5H22orf40;PPARA	chr5	117126458	117273859	2.15101	5.14542	3.36427	0.674616	1.67893	1.33739	0.485785	0.536548	0.469396
XLOC_079840	C6H4orf22	chr6	97155244	97233260	34.5599	19.9577	24.6755	25.3381	22.3335	23.7001	31.2237	28.1156	27.6813
XLOC_080237	C6H4orf22	chr6	96831265	97060399	0.0	0.0	0.0	1.52562	1.33549	1.53977	0.94778	0.744395	0.906239
XLOC_080238	C6H4orf22	chr6	97111960	97149024	8.20116	3.84062	3.57265	2.66602	4.18196	2.67828	3.63065	2.59011	3.37743
XLOC_080239	C6H4orf22	chr6	97155244	97233260	77.7883	76.1122	74.7475	91.8145	92.3544	87.3438	120.316	107.195	127.07
XLOC_081908	C6H4orf22	chr6	97109928	97111396	5.51879	3.46721	7.77282	1.53389	2.11966	1.37702	2.61761	1.66244	2.60625
XLOC_081909	C6H4orf22	chr6	97111509	97111852	7.79003	3.48536	0.0	0.696583	3.27269	2.38221	1.33932	1.12793	0.673472
XLOC_081910	C6H4orf22	chr6	97152847	97153699	2.08284	4.04834	5.70114	1.39985	1.95142	1.51118	2.25919	1.14337	2.63649
XLOC_081911	C6H4orf22	chr6	97154098	97155036	4.6333	3.87977	2.17437	1.91015	3.76565	2.88346	2.93782	5.92761	4.12739
XLOC_081912	C6H4orf22	chr6	97155244	97233260	0.0	2.59136	0.0	0.970655	2.0139	0.669866	0.192048	0.427446	0.565713
XLOC_081913	C6H4orf22	chr6	97155244	97233260	1.36801	0.0	17.5583	0.0	0.0	0.0	0.0	0.0	0.0
XLOC_081184	C6H4orf34	chr6	60320113	60320730	0.0	0.0	0.0	0.281958	0.367123	0.97568	0.422686	1.09262	1.64578
XLOC_082983	C7H19orf21	chr7	44950729	44958055	1.22971	0.36798	1.04159	1.1884	1.23479	0.482167	0.703471	1.12358	2.16041
XLOC_083630	C7H19orf24;CIRBP	chr7	45347731	45360772	0.39836	0.238389	0.0	0.975715	0.656923	0.994414	0.582547	1.3678	0.403687
XLOC_083381	C7H19orf42	chr7	6317409	6331407	1.61631	8.30485	4.37328	3.6357	4.49668	4.16163	2.2749	3.83468	4.06278
XLOC_084050	C7H19orf60	chr7	4531536	4532050	0.0	0.0	0.0	0.726524	0.471492	1.04507	0.720448	0.800342	0.705949
XLOC_085530	C7H5orf30	chr7	104692821	104693074	0.0	0.0	0.0	9.99707	26.1381	11.2077	14.6301	25.0179	22.3151
XLOC_069994	C8A	chr3	89831682	89855929	1.84065	1.46884	1.68071	0.192899	0.530077	0.479139	0.871419	1.8966	0.966705
XLOC_071633	C8A	chr3	89856104	89857057	1.81837	1.35953	1.42227	0.0814853	0.284243	0.282939	1.48287	1.45427	0.952898
XLOC_071634	C8A	chr3	89857688	89858836	2.19055	1.31046	2.85616	0.261818	0.628678	0.379145	1.19488	1.60947	0.574343
XLOC_071635	C8A	chr3	89863804	89864173	0.0	1.01996	0.0	0.305697	0.26199	0.0	0.296156	0.0	0.0
XLOC_071636	C8A	chr3	89867399	89867924	0.0	1.76403	1.53775	0.352409	0.0	0.202833	0.0	0.0	0.171242
XLOC_071637	C8A	chr3	89868345	89870023	3.80524	1.56543	2.97769	0.298547	0.11192	0.148424	0.347581	0.286835	0.374485
XLOC_069992	C8B	chr3	89715029	89754281	10.8833	12.9989	10.2168	9.89005	10.0944	9.41723	9.77232	11.2544	8.95489
XLOC_069993	C8B	chr3	89773850	89784090	20.7537	24.7121	18.8723	30.4441	28.5239	28.0453	32.4726	36.7341	28.315
XLOC_070380	C8B	chr3	89754834	89760832	1.48232	0.621005	0.232033	0.664685	0.768438	0.555675	0.624925	0.388209	0.93424
XLOC_070381	C8B	chr3	89760970	89762442	3.89209	1.16354	2.02868	1.16228	1.01084	0.671279	0.700296	0.774498	0.90521
XLOC_070382	C8B	chr3	89763186	89764798	1.51475	0.0	0.0	0.135672	0.235633	0.0	0.0	0.150313	0.396018
XLOC_070383	C8B	chr3	89765567	89773009	4.98547	2.80766	0.0	1.76826	2.44134	2.64265	2.81254	1.97386	3.00957
XLOC_071629	C8B	chr3	89715029	89754281	2.23634	3.33918	1.74645	1.80116	1.90205	0.920181	1.97746	1.53964	2.72086
XLOC_071630	C8B	chr3	89715029	89754281	1.65355	5.43481	0.0	1.62869	2.56895	1.19496	1.92098	1.47403	1.72805
XLOC_071631	C8B	chr3	89715029	89754281	3.32496	2.97603	5.18783	1.48664	3.05952	1.35951	3.46159	3.22899	2.87892

XLOC_071632	C8B	chr3	89765567	89773009	1.83452	1.37159	2.15233	1.23312	0.788557	1.23689	0.914106	1.00859	0.480665
XLOC_044506	C8G;FBXW5	chr11	106284090	106287630	2.1018	1.0062	1.6448	0.490019	0.791628	0.874768	0.192183	0.338218	0.551697
XLOC_086965	C8H9orf3	chr8	82763381	82774924	11.9779	12.653	11.1733	21.7511	25.3283	23.1673	20.1047	18.0636	26.3553
XLOC_088342	C8H9orf3	chr8	82591343	82591996	0.0	5.67208	5.70539	0.0	0.0	0.0	0.0	0.0	0.0
XLOC_088343	C8H9orf3	chr8	82629485	82630178	2.70134	4.44138	6.33573	0.0	0.0	0.0	0.0	0.0	0.0
XLOC_088344	C8H9orf3	chr8	82660720	82661942	3.39711	4.47121	8.50479	0.0	0.0	0.0	0.0	0.0	0.0
XLOC_088345	C8H9orf3	chr8	82667071	82667797	0.0	2.66246	12.9316	0.0	0.0	0.0	0.0	0.0	0.0
XLOC_088346	C8H9orf3	chr8	82669544	82669982	5.15726	9.23926	10.0672	0.0	0.0	0.0	0.0	0.0	0.0
XLOC_088347	C8H9orf3	chr8	82672214	82673393	6.37221	6.14181	11.0782	0.0	0.0	0.0	0.0	0.0	0.0
XLOC_088348	C8H9orf3	chr8	82673562	82673982	16.532	9.87087	21.5104	0.0	0.0	0.0	0.0	0.0	0.0
XLOC_088349	C8H9orf3	chr8	82674120	82674808	6.817	4.89075	10.6589	0.0	0.0	0.0	0.0	0.0	0.0
XLOC_088350	C8H9orf3	chr8	82677758	82678362	3.23986	1.45238	20.2577	0.0	0.0	0.0	0.0	0.0	0.0
XLOC_088351	C8H9orf3	chr8	82679622	82680019	6.03926	9.91396	11.7839	0.0	0.0	0.0	0.0	0.0	0.0
XLOC_088352	C8H9orf3	chr8	82685822	82686784	1.79803	6.45278	13.3605	0.0	0.0	0.0	0.0	0.0	0.0
XLOC_088353	C8H9orf3	chr8	82691746	82692543	7.91696	3.0435	13.2663	0.0	0.0	0.0	0.0	0.0	0.0
XLOC_088354	C8H9orf3	chr8	82692847	82693373	0.0	7.62344	4.60076	0.0	0.0	0.0	0.0	0.0	0.0
XLOC_088355	C8H9orf3	chr8	82693514	82694752	2.67744	4.40504	7.85525	0.0	0.0	0.0	0.0	0.0	0.0
XLOC_088356	C8H9orf3	chr8	82695705	82696169	9.4398	8.45738	7.37233	0.0	0.0	0.0	0.0	0.0	0.0
XLOC_088357	C8H9orf3	chr8	82696672	82697402	2.52674	6.42085	1.97559	0.0	0.0	0.0	0.0	0.0	0.0
XLOC_088358	C8H9orf3	chr8	82697685	82698425	7.45006	5.56831	5.82514	0.0	0.0	0.0	0.0	0.0	0.0
XLOC_088359	C8H9orf3	chr8	82699251	82700034	6.93786	3.803	10.8503	0.0	0.0	0.0	0.0	0.0	0.0
XLOC_088360	C8H9orf3	chr8	82701014	82701278	0.0	21.9577	31.3124	0.0	0.503486	0.0	0.0	0.0	0.0
XLOC_088361	C8H9orf3	chr8	82702139	82702764	0.0	6.01132	3.62795	0.0	0.0	0.0	0.0	0.0	0.0
XLOC_088362	C8H9orf3	chr8	82703407	82704183	4.67759	4.54527	9.14412	0.0	0.0	0.0	0.0	0.0	0.0
XLOC_088363	C8H9orf3	chr8	82706964	82707445	11.1797	10.6852	8.73224	0.0	0.0	0.0	0.0	0.0	0.0
XLOC_088364	C8H9orf3	chr8	82720108	82720442	20.4605	14.6452	31.9118	0.0	0.0	0.0	0.0	0.0	0.0
XLOC_088365	C8H9orf3	chr8	82729836	82730564	10.1424	9.85451	17.8425	0.0	0.0	0.0	0.0	0.126187	0.0
XLOC_088366	C8H9orf3	chr8	82731643	82732300	7.24013	4.76106	9.05562	0.0	0.0	0.0	0.129911	0.0	0.0
XLOC_088367	C8H9orf3	chr8	82734382	82735299	9.52285	6.55	9.68254	0.0	0.0	0.0	0.0	0.0	0.0
XLOC_088368	C8H9orf3	chr8	82745829	82746083	44.0746	48.195	28.6357	0.0	0.0	0.0	0.604122	0.688555	0.631449
XLOC_088369	C8H9orf3	chr8	82747489	82748459	11.5721	12.5124	15.3179	0.319127	0.0695845	0.184701	0.0	0.0	0.155504
XLOC_088370	C8H9orf3	chr8	82759831	82761352	0.0	0.158691	0.0	0.570695	0.665346	0.551529	0.677617	0.159824	0.556698
XLOC_088371	C8H9orf3	chr8	82761950	82762320	0.0	0.0	2.65459	0.60853	1.56475	0.695403	0.0	1.32074	0.0

XLOC_088372	C8H9orf3	chr8	82762549	82763063	4.05791	0.0	0.0	0.908155	2.35746	1.4631	0.720448	1.4006	3.00028
XLOC_086507	C8H9orf3;MIR2475;MIR23B;MIR24-1;MIR27B;FANCC	chr8	82864399	83036118	0.0	0.0	0.0	0.149421	0.0	0.172267	0.0	0.0	0.0
XLOC_087038	C8H9orf43;POLE3;ALAD	chr8	104348733	104395691	4.40689	7.07391	6.71116	14.8744	15.3148	15.9463	13.0108	10.2921	9.7076
XLOC_086499	C8H9orf64;MIR7-2;RMI1;HNRNPK;KIF27	chr8	78509013	78734498	6.76251	7.28821	9.51764	18.3526	23.1265	20.8406	16.7868	15.7059	17.7777
XLOC_086872	C8H9orf85;GDA;FAM108B1;TMEM2	chr8	48225471	48836901	5.54886	10.7322	8.04222	4.25143	5.34012	4.94677	7.78502	6.0911	4.00986
XLOC_087772	C8H9orf85;GDA;FAM108B1;TMEM2	chr8	48225471	48836901	0.0	0.0	2.24477	0.257245	0.442585	2.06325	0.50297	0.280838	1.49644
XLOC_087773	C8H9orf85;GDA;FAM108B1;TMEM2	chr8	48225471	48836901	0.794538	0.832083	3.41988	0.320617	0.467677	0.578785	0.726823	0.479608	0.2781
XLOC_087774	C8H9orf85;GDA;FAM108B1;TMEM2	chr8	48225471	48836901	0.0	0.0	0.0	0.0	1.23175	1.64863	0.0	0.516229	1.40246
XLOC_087775	C8H9orf85;GDA;FAM108B1;TMEM2	chr8	48225471	48836901	1.57068	0.469433	1.22767	0.42202	0.488447	0.0	0.140623	0.155802	0.136857
XLOC_087776	C8H9orf85;GDA;FAM108B1;TMEM2	chr8	48225471	48836901	3.02317	1.35539	1.18156	0.406168	0.823013	0.468649	1.35457	0.300053	0.131736
XLOC_087777	C8H9orf85;GDA;FAM108B1;TMEM2	chr8	48225471	48836901	0.0	1.31625	3.44166	0.394754	1.00802	0.897695	0.0	1.27156	0.381105
XLOC_087778	C8H9orf85;GDA;FAM108B1;TMEM2	chr8	48225471	48836901	1.10846	0.994311	0.866818	0.297973	0.086497	0.574209	1.30091	0.110575	0.193488
XLOC_087779	C8H9orf85;GDA;FAM108B1;TMEM2	chr8	48225471	48836901	2.65181	1.18911	4.14649	0.475127	0.413139	1.50904	0.357685	0.527546	0.34689
XLOC_087780	C8H9orf85;GDA;FAM108B1;TMEM2	chr8	48225471	48836901	2.45247	1.174	1.53527	0.439795	0.423126	0.918244	1.34373	0.295744	0.901012

XLOC_087781	C8H9orf85;GDA;FAM108B1;TMEM2	chr8	48225471	48836901	4.47208	2.0813	1.5553	0.757407	0.818288	0.775161	1.31574	0.848786	0.825824
XLOC_086620	C8H9orf91	chr8	105518136	105524047	1.31429	1.51762	1.14954	0.309655	0.662995	0.347848	0.178951	0.417514	0.44805
XLOC_086621	C8H9orf91	chr8	105524413	105581718	1.11358	2.33076	0.870816	1.29729	1.82483	0.461479	0.402086	0.111128	0.874686
XLOC_087051	C8H9orf91	chr8	105524413	105581718	28.6621	28.464	22.647	44.0626	46.6057	46.4476	44.2547	37.1658	34.7919
XLOC_088664	C8H9orf91	chr8	105524413	105581718	0.0	0.642076	0.0	1.34705	2.49535	2.21275	0.571208	0.0	0.747587
XLOC_090061	C9H6orf118	chr9	101921919	102354519	4.29871	5.19557	5.73156	0.0261585	0.022911	0.0	0.0	0.0	0.0255332
XLOC_091619	C9H6orf118	chr9	101921919	102354519	0.801399	0.479383	2.50761	0.0	0.0	0.0	0.0	0.0	0.0
XLOC_091620	C9H6orf118	chr9	101921919	102354519	0.0	1.45845	0.762938	0.0	0.0	0.0	0.0	0.0	0.0
XLOC_091621	C9H6orf118	chr9	101921919	102354519	1.16377	2.0877	5.4601	0.0	0.0	0.0	0.0	0.0	0.0
XLOC_091622	C9H6orf118	chr9	101921919	102354519	0.0	1.30772	1.71014	0.0	0.0	0.0	0.0	0.0	0.0
XLOC_091623	C9H6orf118	chr9	101921919	102354519	1.11704	0.667985	4.36759	0.0	0.0	0.0	0.100826	0.0	0.0
XLOC_089592	C9H6orf120;PHF10	chr9	105053240	105077518	33.4854	37.6317	37.9121	37.6169	33.7298	37.0047	43.5848	50.0086	39.2876
XLOC_091055	C9H6orf163	chr9	63443260	63443709	2.48136	1.48192	3.87534	9.76953	9.95928	10.1967	7.64715	11.4387	9.26621
XLOC_091056	C9H6orf163	chr9	63443805	63444083	5.94916	7.0938	9.27342	10.1255	12.1214	12.0298	11.4268	10.7038	13.8275
XLOC_090070	C9H6orf70	chr9	105129697	105131800	1.57784	1.19805	1.7565	1.39574	2.30304	0.970859	0.940793	1.01638	1.17278
XLOC_053427	CA1	chr14	79481584	79581277	22.352	15.0268	16.4457	7.08176	7.73122	7.35706	6.4591	4.04514	6.68453
XLOC_054925	CA1	chr14	79481584	79581277	2.69135	0.804523	2.10406	0.482189	0.209615	0.974533	1.57238	0.401423	0.352024
XLOC_054926	CA1	chr14	79481584	79581277	13.1361	14.7213	10.2666	9.85237	9.95155	10.3431	7.63314	11.7135	8.15267
XLOC_053425	CA3	chr14	79397766	79413107	1.77482	2.47777	0.46289	7.6908	10.7086	8.7932	8.89721	7.30309	14.4329
XLOC_053426	CA3	chr14	79415796	79432391	1.99537	0.0	0.0	13.911	20.5568	13.4079	11.1972	10.4433	22.5829
XLOC_054923	CA3	chr14	79415796	79432391	3.74994	4.39946	3.4051	4.2246	4.14028	3.28306	2.61914	3.14837	3.59959
XLOC_065813	CAB39	chr2	119351212	119353634	32.8535	23.1171	22.6724	8.65971	17.1359	11.022	11.4576	4.63353	7.7627
XLOC_066478	CAB39	chr2	119373019	119379999	24.3134	23.4217	23.7609	23.1346	22.6969	24.5098	23.1157	27.094	20.2695
XLOC_066479	CAB39	chr2	119381662	119418850	12.5666	20.6274	16.8103	22.1281	20.1939	23.7579	25.6254	28.9311	21.6807
XLOC_066480	CAB39	chr2	119423329	119427068	10.5446	18.6334	12.7456	19.6732	15.0534	17.5952	16.3145	15.1322	13.8119
XLOC_068360	CAB39	chr2	119353705	119355472	9.88573	8.87342	9.49422	1.85344	5.28767	2.57106	4.47611	1.08425	2.5553
XLOC_068361	CAB39	chr2	119355646	119356109	2.36767	0.707084	0.0	0.635649	4.02405	0.973616	1.88012	0.0	1.02851
XLOC_068362	CAB39	chr2	119419450	119419706	0.0	6.44688	0.0	1.94155	7.02987	7.26241	1.18696	4.05668	6.8148
XLOC_068363	CAB39	chr2	119419907	119420636	3.79674	1.89179	2.96856	2.38107	2.36719	3.27481	2.62023	1.13373	2.31823
XLOC_068364	CAB39	chr2	119421949	119422898	1.82756	1.91295	1.42945	0.900864	1.35693	1.51659	1.49024	1.64423	1.9952
XLOC_082772	CACNA1A	chr7	13258859	13325698	29.8308	35.6391	28.0265	96.076	107.271	96.5453	132.136	128.903	100.874

XLOC_082773	CACNA1A	chr7	13340613	13383131	2.91547	3.92932	2.95665	0.89313	1.86359	2.53305	2.35736	2.75393	1.54029
XLOC_083403	CACNA1A	chr7	13258859	13325698	0.859571	5.56202	9.08837	2.18512	2.9227	3.84488	2.47473	1.46651	2.01713
XLOC_084220	CACNA1A	chr7	13258859	13325698	0.0	0.788003	0.0	0.393586	0.206033	0.182263	0.557382	0.175791	0.153468
XLOC_084221	CACNA1A	chr7	13258859	13325698	1.44513	0.43196	0.0	0.388341	0.562402	1.49413	0.388975	0.430779	0.629913
XLOC_084222	CACNA1A	chr7	13258859	13325698	0.0	0.680418	1.77941	1.22336	0.528493	0.468706	0.805324	0.224303	1.38602
XLOC_084223	CACNA1A	chr7	13258859	13325698	1.41129	0.843718	1.1033	1.64346	1.97757	1.02162	1.3937	1.12198	1.47649
XLOC_084224	CACNA1A	chr7	13258859	13325698	3.04224	0.45464	0.0	1.90739	2.12959	1.10038	1.908	1.05674	1.59076
XLOC_084225	CACNA1A	chr7	13258859	13325698	0.772061	0.693044	0.302111	1.21158	1.03019	0.602632	0.706404	1.00992	1.08099
XLOC_084226	CACNA1A	chr7	13258859	13325698	0.0	0.627195	0.0	0.939727	1.95069	1.94585	1.30304	0.414046	2.00856
XLOC_044496	CACNA1B	chr11	105167872	105350460	51.4633	55.8698	45.8202	34.9313	37.3229	38.1541	42.7507	38.9358	42.9824
XLOC_045138	CACNA1B	chr11	105167872	105350460	0.0	0.0	0.0	0.0	0.0	0.0	0.0	0.0	0.410105
XLOC_047380	CACNA1B	chr11	105167872	105350460	0.0	1.72665	0.0	0.689831	1.04533	0.794252	0.685433	0.0	0.335371
XLOC_047381	CACNA1B	chr11	105167872	105350460	8.30131	12.3847	0.0	7.47512	6.20349	3.33927	5.41432	4.64085	3.56638
XLOC_047382	CACNA1B	chr11	105167872	105350460	0.64375	1.34804	1.00732	0.923374	1.91602	1.47153	0.820701	0.968482	1.1819
XLOC_047383	CACNA1B	chr11	105167872	105350460	1.19453	1.07149	0.934049	1.2843	0.745181	0.371073	0.646103	1.54698	0.83384
XLOC_047384	CACNA1B	chr11	105167872	105350460	0.0	0.0	2.72129	0.311816	0.271452	0.600727	0.627847	0.693671	0.303751
XLOC_047385	CACNA1B	chr11	105167872	105350460	3.32442	0.0	0.0	0.0	2.03969	1.01963	0.865683	1.6145	0.576015
XLOC_047386	CACNA1B	chr11	105167872	105350460	0.0	0.0	1.55043	0.355304	2.4603	1.84034	0.529076	0.587397	0.863343
XLOC_047387	CACNA1B	chr11	105167872	105350460	1.51546	0.0	0.0	0.815057	0.770838	0.236027	0.137769	0.531239	0.529783
XLOC_047388	CACNA1B	chr11	105167872	105350460	0.0	0.308818	0.807503	0.277575	0.241859	0.214046	0.653485	0.103063	0.270485
XLOC_047389	CACNA1B	chr11	105350535	105351263	1.2678	0.75804	1.98251	1.02225	0.296412	0.656101	0.228236	0.378561	0.221169
XLOC_064240	CACNA1G	chr19	36732849	36736612	0.513844	0.922392	0.804157	0.46072	0.604342	1.06868	0.891149	0.154859	0.943822
XLOC_065041	CACNA1G	chr19	36731743	36732596	0.0	0.621927	0.813279	0.559137	0.324774	0.215574	0.564	0.103795	0.453916
XLOC_064251	CACNB1	chr19	40293387	40327760	4.65079	4.93317	8.10246	4.29762	6.11591	4.94766	4.87471	5.49918	5.30379
XLOC_065115	CACNB1	chr19	40293387	40327760	0.951032	0.853227	0.743862	1.10802	0.743078	0.394513	0.516639	1.33037	0.913554
XLOC_065116	CACNB1	chr19	40293387	40327760	0.0	0.355379	0.0	0.319506	1.20502	0.984683	0.535734	0.118421	0.622247
XLOC_065117	CACNB1	chr19	40293387	40327760	0.0	5.05154	3.3023	0.757427	0.96826	2.58579	0.362105	0.407332	0.0
XLOC_050498	CACNB2	chr13	33003461	33260827	27.0532	11.9654	18.8764	3.72809	1.49121	4.06559	0.999454	4.37263	2.12606
XLOC_051540	CACNB2	chr13	33003461	33260827	21.4051	5.47592	11.9329	0.0	0.0	0.0	0.533095	0.0	0.0
XLOC_051541	CACNB2	chr13	33003461	33260827	0.0	0.0	0.0	0.747512	0.0	0.0	0.0	0.773474	0.0
XLOC_051542	CACNB2	chr13	33003461	33260827	0.615116	0.460171	1.20359	0.0	0.0	0.0320242	0.0	0.0	0.0
XLOC_051543	CACNB2	chr13	33003461	33260827	0.0	0.0	1.49954	0.0	0.0	0.0	0.0	0.0	0.0
XLOC_076268	CACNB3	chr5	31116529	31137658	0.0	0.14537	0.0	1.21985	1.25748	0.758027	0.843092	0.341792	1.53009

XLOC_058166	CACYBP;MRPS14	chr16	57624830	57754716	0.996049	0.447151	0.779687	2.01015	0.678758	1.76466	3.61467	1.59113	2.35525
XLOC_046530	CAD	chr11	72387892	72388855	0.0	0.0	0.0	0.88522	0.701849	0.0	0.325503	0.359103	0.235277
XLOC_046531	CAD	chr11	72389161	72389956	1.13454	0.339226	0.0	3.15142	3.00947	2.3505	2.7641	3.05391	2.37622
XLOC_046532	CAD	chr11	72390033	72390721	5.4536	3.2605	4.26358	32.4881	29.7285	34.4117	28.6669	38.359	30.3149
XLOC_073228	CADPS2	chr4	87769223	87794289	2.53191	1.7715	3.092	0.885648	1.2011	1.20764	0.646124	0.730961	1.13349
XLOC_073229	CADPS2	chr4	87834094	87841242	6.19344	5.20889	5.75035	1.75029	1.99848	2.50947	1.98981	0.890427	2.18402
XLOC_074562	CADPS2	chr4	87801407	87801852	2.51587	0.751241	0.0	0.0	0.0	0.0	0.0	0.0	0.0
XLOC_074563	CADPS2	chr4	87809818	87811331	1.06711	0.319253	2.08748	0.0	0.0	0.221909	0.0	0.0	0.139994
XLOC_074564	CADPS2	chr4	87817030	87818721	0.471622	0.423318	0.369057	0.169153	0.22195	0.0490565	0.0861651	0.0474029	0.0
XLOC_074565	CADPS2	chr4	87830521	87831074	1.82885	1.0929	0.0	0.0	0.14189	0.188634	0.0	0.0	0.0
XLOC_073230	CADPS2;RNF148	chr4	87841367	88033283	13.7771	7.77796	21.1661	3.42765	3.57144	5.85553	2.65389	1.8225	4.26893
XLOC_072699	CADPS2;TAS2R16;SLC13A1	chr4	88094602	88413662	10.8008	10.4306	9.67494	7.31597	6.46852	6.22572	7.06508	5.93579	6.94872
XLOC_053714	CALB1	chr14	75896846	76011016	16.7504	26.6491	17.8949	26.5464	34.2436	31.9468	38.0376	32.9276	46.0662
XLOC_054838	CALB1	chr14	75896846	76011016	8.10366	11.8764	15.2138	20.8466	21.6785	22.0378	22.8778	25.2256	20.1788
XLOC_056395	CALCA	chr15	38042438	38044748	0.335993	1.70918	3.68146	1.29564	1.53018	1.43412	3.01553	2.13113	1.76443
XLOC_055748	CALCA;INSC	chr15	37967077	38042146	74.6577	96.4503	87.7312	128.089	91.6335	108.751	137.622	168.832	103.764
XLOC_065077	CALCOCO2	chr19	38320396	38320957	0.0	0.0	0.0	2.56818	1.39117	2.21922	0.159814	2.1274	1.09243
XLOC_065078	CALCOCO2	chr19	38321455	38321793	0.0	0.0	0.0	4.65234	0.610953	0.815479	0.0	1.54352	0.345894
XLOC_065079	CALCOCO2	chr19	38322013	38322853	0.0	0.0	0.0	1.61437	0.413634	0.98844	0.766045	1.79769	1.38759
XLOC_065080	CALCOCO2	chr19	38322987	38324502	1.06554	0.637567	0.0	2.19733	1.16948	1.71727	0.291678	3.26402	1.35129
XLOC_065081	CALCOCO2	chr19	38324644	38325505	0.0	0.0	0.80404	2.48753	1.12389	3.51677	1.39427	5.74724	1.61558
XLOC_072711	CALD1	chr4	99569546	99575487	0.0	0.0844578	0.220899	4.62736	1.73614	3.01837	2.63922	4.38197	2.09483
XLOC_044004	CALM;CALM2	chr11	29373563	29510070	34.7012	26.8534	28.3538	12.008	19.9976	12.4952	23.233	14.6763	19.2533
XLOC_045703	CALM;CALM2	chr11	29373563	29510070	0.0	0.419407	0.0	1.38255	2.40305	1.74128	0.756022	1.25479	1.22324
XLOC_079602	CAMK2D	chr6	13069743	13086942	13.6879	13.3864	16.1529	7.35027	9.74475	8.07442	7.04675	6.56483	10.6377
XLOC_080012	CAMK2D	chr6	13185148	13187854	0.0	0.489685	0.0	0.0	0.0	0.0	0.0	0.0	0.0
XLOC_080539	CAMK2D	chr6	13087100	13089447	5.28504	3.26177	2.84371	2.72524	2.77534	2.78547	2.2993	1.79594	3.78731
XLOC_059857	CAMKK2	chr17	56162488	56172422	2.13826	1.27876	3.34439	0.670629	1.00145	0.664766	1.93177	1.17339	1.67982
XLOC_060250	CAMKK2	chr17	56123084	56138478	0.82686	0.928003	0.323464	0.778675	0.730886	0.604171	0.176255	0.562681	0.452325

XLOC_084298	CAMSAP3	chr7	17686155	17686297	298.701	209.928	0.0	189.217	127.87	91.1118	83.0537	102.799	72.5183
XLOC_044164	CAPN13	chr11	69045626	69051147	12.9095	15.0798	8.17662	44.3105	47.3426	39.9288	48.3898	44.9638	44.0742
XLOC_044165	CAPN13	chr11	69070046	69072984	15.9921	8.57045	9.09917	15.6821	18.7573	15.3678	18.2135	21.1822	16.6198
XLOC_044166	CAPN13	chr11	69089107	69093080	1.62913	3.15185	4.54434	3.1646	2.65177	3.63864	2.86878	2.31532	2.13529
XLOC_044167	CAPN13	chr11	69095075	69105419	20.0901	37.9603	29.6161	5.6704	5.04626	5.18134	4.74406	6.37457	4.13071
XLOC_044168	CAPN13	chr11	69105481	69109942	16.3041	21.6319	19.3781	3.32651	3.12949	3.03187	3.02065	3.75928	3.12868
XLOC_044169	CAPN13	chr11	69119063	69123483	0.0	0.204774	0.0	0.0	0.105985	0.0355316	0.0615747	0.0688456	0.0
XLOC_046458	CAPN13	chr11	69051472	69051647	0.0	29.3846	19.2093	43.3997	26.4496	12.1521	35.2921	51.743	46.1054
XLOC_046459	CAPN13	chr11	69059558	69061794	6.2632	4.99769	4.35712	2.52749	2.15821	2.39059	2.86732	2.7671	2.40577
XLOC_046460	CAPN13	chr11	69073563	69074108	7.46646	3.904	0.0	5.51518	6.65972	4.42712	4.81951	1.10663	4.7114
XLOC_046461	CAPN13	chr11	69088631	69088885	0.0	0.0	0.0	1.97977	0.550915	1.48015	0.0	0.0	1.2629
XLOC_046462	CAPN13	chr11	69110538	69110717	0.0	0.0	17.4403	10.3343	0.0	2.20991	1.69694	0.0	3.80844
XLOC_044159	CAPN14;GALNT 14	chr11	68692016	68871828	16.4051	16.4427	14.2257	16.3985	20.222	18.9123	23.3626	19.8561	19.5525
XLOC_044846	CAPN14;GALNT 14	chr11	68692016	68871828	75.1164	75.2036	61.1511	19.5609	23.5577	17.8903	22.927	20.0491	25.6864
XLOC_046445	CAPN14;GALNT 14	chr11	68692016	68871828	0.0	4.75647	4.66392	0.712594	1.54185	1.6403	1.94446	0.785304	0.865563
XLOC_046446	CAPN14;GALNT 14	chr11	68692016	68871828	2.80953	3.35497	2.19265	1.00512	0.432503	0.576002	0.245912	0.823311	0.97462
XLOC_046447	CAPN14;GALNT 14	chr11	68692016	68871828	1.15881	2.31152	2.41814	0.76198	0.545675	0.56275	0.530086	0.582927	0.506975
XLOC_046448	CAPN14;GALNT 14	chr11	68692016	68871828	7.92413	10.1967	10.2609	2.03319	2.04654	1.89585	1.10661	0.875287	3.13529
XLOC_046449	CAPN14;GALNT 14	chr11	68692016	68871828	0.0	5.31063	3.47148	1.19339	1.7187	0.685952	0.786332	1.3118	0.579488
XLOC_046450	CAPN14;GALNT 14	chr11	68692016	68871828	9.17115	6.84803	8.95315	1.43631	1.41775	1.65058	1.21534	1.12687	1.99235
XLOC_062230	CAPNS1;COX7A 1;POLR2I;ZNF56 5;TBCB	chr18	46958684	47087361	30.9634	47.4053	28.676	90.067	83.8016	83.7276	81.6617	88.6928	79.3176
XLOC_069860	CAPZA1	chr3	30809998	30821378	42.4196	39.9528	33.141	17.672	20.6335	19.7013	22.9118	20.7858	23.4493
XLOC_070759	CAPZA1	chr3	30822116	30822919	4.48192	2.01016	2.62862	0.502001	0.174853	0.348236	0.0	0.670351	0.391149
XLOC_065287	CARD14	chr19	53059094	53059984	0.987389	0.29527	0.0	0.884869	0.462746	0.409502	0.178676	0.19723	0.258641
XLOC_065141	CASC3	chr19	41082730	41084258	0.0	0.789402	0.0	0.0	0.0	0.0	0.0	0.0	0.0
XLOC_064258	CASC3;RAPGEF L1	chr19	41105421	41140146	0.0	0.254634	0.665967	0.0	0.0	0.0	0.0	0.0	0.0

XLOC_092568	CASK	chrX	107397086	107403381	15.7862	8.90661	8.82383	2.46702	2.01681	2.86218	2.3494	1.31501	2.88052
XLOC_055693	CASP4	chr15	3317665	3583124	2.88889	3.4043	3.25066	0.340085	0.227123	0.244594	0.281924	0.182131	0.173929
XLOC_056031	CASP4	chr15	3317665	3583124	4.9372	10.3283	6.43119	0.0	0.12787	0.0	0.0	0.0	0.0
XLOC_056032	CASP4	chr15	3317665	3583124	3.12101	2.33197	13.4171	0.139764	0.121331	0.0	0.0	0.0	0.0
XLOC_056033	CASP4	chr15	3317665	3583124	3.89112	3.19996	2.28247	0.174358	0.151984	0.0	0.0	0.0	0.0
XLOC_056034	CASP4	chr15	3317665	3583124	6.78148	5.47711	7.9583	0.0607933	0.0	0.0	0.0	0.0	0.0
XLOC_056035	CASP4	chr15	3317665	3583124	1.57067	3.75543	6.13836	0.0	0.122112	0.0	0.0	0.0	0.273704
XLOC_056036	CASP4	chr15	3317665	3583124	5.63115	6.85217	9.10774	0.0673293	0.117872	0.0781446	0.240487	0.0755549	0.0985567
XLOC_056037	CASP4	chr15	3317665	3583124	0.0	3.75313	8.17978	0.187456	0.0816591	0.0	0.0945333	0.0	0.0
XLOC_083124	CAST;CAST	chr7	98490021	98501109	4.30891	1.95897	3.79088	0.248376	0.0791215	0.183562	0.207838	0.202988	0.0881606
XLOC_085402	CAST;CAST	chr7	98501205	98501555	3.75422	1.1199	0.0	0.0	0.0	0.382955	0.323459	0.0	0.324695
XLOC_055476	CAT;ELF5	chr15	65787029	65873957	25.1181	30.6999	28.1646	22.8947	23.0343	23.1132	23.557	22.7671	27.6211
XLOC_062189	CBFB	chr18	34840589	34847475	24.4469	34.5113	25.1266	1.85883	3.29645	1.7661	1.19053	1.02867	1.79495
XLOC_062190	CBFB	chr18	34861340	34863456	224.718	134.343	158.282	41.0694	44.6512	46.1172	32.6333	44.6433	26.3955
XLOC_062885	CBFB	chr18	34859074	34859426	3.71583	2.21695	0.0	3.98727	1.13673	0.758225	0.961053	0.71874	1.60705
XLOC_062886	CBFB	chr18	34859787	34860661	1.00944	1.50929	0.0	1.35691	1.10366	1.36042	0.730369	0.20158	0.793189
XLOC_062887	CBFB	chr18	34863588	34866539	46.8598	29.6731	33.4351	7.43008	6.97705	7.1727	7.52689	10.7491	5.50773
XLOC_039250	CBLB	chr1	50821158	50822253	6.9432	6.69173	1.81051	0.138304	0.0603637	0.0	0.0	0.0	0.0
XLOC_039251	CBLB	chr1	50828517	50829199	1.379	4.94663	2.15614	0.123531	0.0	0.0	0.371623	0.0	0.0
XLOC_039252	CBLB	chr1	50830065	50831555	5.97043	5.68332	4.67167	0.0973276	0.0	0.0	0.0495159	0.0	0.0
XLOC_039254	CBLB	chr1	50846971	50847806	1.06755	6.06513	6.67891	0.0	0.0	0.0	0.0	0.0	0.0
XLOC_039255	CBLB	chr1	50848976	50849609	4.56336	7.72888	2.378	0.0	0.0	0.0	0.0	0.0	0.0
XLOC_039256	CBLB	chr1	50850330	50850781	0.0	8.09515	3.849	0.0	0.0	0.0	0.0	0.0	0.0
XLOC_039257	CBLB	chr1	50864398	50865537	2.94752	2.42453	2.88234	0.0	0.0	0.0	0.0	0.0	0.0
XLOC_039258	CBLB	chr1	50865662	50866125	2.36767	7.07084	3.6982	0.0	0.0	0.0	0.0	0.0	0.0
XLOC_065290	CBX2	chr19	53373867	53374437	0.0	0.523966	0.0	1.25612	1.90573	1.62849	0.93863	0.0	2.90087
XLOC_065291	CBX2	chr19	53374514	53375070	1.81508	1.62703	1.41834	0.975129	2.2534	2.24675	1.77929	0.358918	3.16013
XLOC_065292	CBX2	chr19	53375673	53376231	0.0	2.15856	1.41127	2.10225	2.5226	2.23566	1.93167	1.25008	3.45889
XLOC_065293	CBX2	chr19	53376740	53378421	1.42415	2.13048	3.34331	2.46881	3.76058	2.32075	3.0354	2.38561	3.07305
XLOC_077460	CBX5	chr5	25957254	25957524	38.1285	26.5228	19.8122	0.0	0.0	0.0	0.0	0.0	0.0
XLOC_064042	CBX8;CBX2	chr19	53310812	53373622	20.2538	24.0275	17.6777	21.8952	23.3546	23.4561	20.7201	19.2527	21.6537
XLOC_076976	CBY1	chr5	110784425	110809716	13.8722	13.9304	14.5497	31.5965	30.961	29.4013	36.3159	48.8506	25.2866
XLOC_078721	CBY1	chr5	110784425	110809716	5.19428	5.42817	4.0557	8.36541	11.8166	7.46561	11.8672	7.37715	10.822

XLOC_078722	CBY1	chr5	110784425	110809716	9.47988	4.53176	4.44427	8.65727	8.82116	7.62356	8.77313	8.24002	8.08499
XLOC_078723	CBY1	chr5	110784425	110809716	0.0	4.17828	32.7704	11.3207	14.733	11.3023	17.9236	9.2154	13.8558
XLOC_078724	CBY1	chr5	110809778	110810161	9.62019	2.8709	2.50238	18.9285	12.0614	8.85766	8.36438	15.2824	9.16769
XLOC_082766	CC2D1A	chr7	12885024	12890427	5.88862	1.17284	3.06717	2.28446	1.8255	1.01145	1.04673	1.74351	1.53702
XLOC_084197	CC2D1A	chr7	12881186	12881362	23.681	7.16659	0.0	8.90555	5.16604	2.37189	7.25968	4.21257	6.1346
XLOC_069910	CCBL2	chr3	55112057	55136375	9.61683	6.94036	6.02911	7.47305	7.70908	7.96929	7.52351	7.55457	7.51012
XLOC_071298	CCBL2	chr3	55136830	55137520	0.0	3.24827	1.0619	1.58181	2.32714	1.96709	2.07501	1.48548	1.65813
XLOC_084201	CCDC130	chr7	12926976	12930365	1.11901	1.74139	1.92695	2.36856	2.39212	1.77196	2.93712	2.75275	2.82181
XLOC_073687	CCDC132	chr4	10438706	10439016	0.0	4.22897	0.0	35.5241	39.5119	39.3697	38.125	18.1024	40.3816
XLOC_073688	CCDC132	chr4	10439467	10439835	0.0	0.0	0.0	14.4357	14.7392	15.792	13.089	8.9962	12.7853
XLOC_076474	CCDC134	chr5	113332553	113336276	0.0	0.0	0.0	6.06818	2.61167	6.35048	3.14133	3.13602	5.57119
XLOC_078749	CCDC134	chr5	113332553	113336276	0.0	0.0	0.0	0.802754	0.630116	0.836273	0.243532	0.895559	0.938783
XLOC_078750	CCDC134	chr5	113337314	113338246	0.0	0.279272	0.0	2.00862	1.8973	0.774845	0.592038	0.933282	1.0602
XLOC_078751	CCDC134	chr5	113338554	113341109	0.0	0.0	0.0	0.946856	0.900331	0.785266	1.07811	0.789968	0.660131
XLOC_078752	CCDC134	chr5	113341251	113343553	0.0	0.0	0.0	0.877064	1.16515	0.877716	0.895659	0.44139	0.678883
XLOC_078753	CCDC134	chr5	113343789	113345541	0.0	0.0	0.0	2.19624	1.85024	1.79303	1.45069	1.27679	1.19045
XLOC_078754	CCDC134	chr5	113346123	113347720	0.0	0.300632	0.0	1.89203	2.00927	1.51525	1.37609	1.26198	1.58208
XLOC_065490	CCDC141	chr2	17936229	17946602	0.0	0.0	0.0	3.15264	3.23592	2.43806	3.2522	3.7683	2.93365
XLOC_066932	CCDC141	chr2	17936229	17946602	0.0	0.0	0.0	6.45735	2.0978	2.80845	1.39705	6.32585	3.34543
XLOC_066933	CCDC141	chr2	17936229	17946602	0.457018	0.410216	1.0729	1.84407	1.00381	1.18855	1.33632	1.10264	1.67924
XLOC_066934	CCDC141	chr2	17946786	17948241	0.0	0.0	0.0	1.39932	1.09225	0.811282	1.11843	2.29461	1.31616
XLOC_066935	CCDC141	chr2	17962756	17963501	0.0	0.0	0.0	0.992702	0.383892	0.509807	0.221781	1.47108	0.75179
XLOC_066936	CCDC141	chr2	17965385	17965984	0.0	0.0	0.0	1.46718	0.763792	0.676707	0.439406	1.78559	0.285414
XLOC_066937	CCDC141	chr2	17971702	17972512	0.0	0.0	0.0	2.78108	2.16242	3.21557	1.10075	0.7738	1.93484
XLOC_066938	CCDC141	chr2	17981937	17982330	0.0	0.0	0.0	1.37331	1.6517	1.88638	1.60571	1.19652	3.72614
XLOC_073151	CCDC146;PION	chr4	44125655	44233640	19.5271	18.3014	19.9375	9.66915	9.80349	9.73299	11.2516	10.1679	10.1671
XLOC_074089	CCDC146;PION	chr4	44125655	44233640	2.55138	1.52363	3.98464	0.913138	0.787295	1.048	1.12187	0.250035	0.221518
XLOC_074090	CCDC146;PION	chr4	44125655	44233640	0.0	1.9912	1.73596	0.596676	0.687491	0.457302	1.17948	0.0	1.5453
XLOC_074091	CCDC146;PION	chr4	44125655	44233640	0.0	1.84419	3.21542	1.65789	1.43428	0.63585	1.27805	1.21717	1.07388
XLOC_074092	CCDC146;PION	chr4	44125655	44233640	0.0	2.65314	0.99138	1.02225	1.28446	1.44342	0.342354	0.504748	1.4376

XLOC_074093	CCDC146;PION	chr4	44125655	44233640	4.75092	3.54701	1.8554	2.97608	2.93603	1.70934	1.88603	1.39969	3.30195
XLOC_074094	CCDC146;PION	chr4	44125655	44233640	0.0	2.02637	1.06004	1.2145	1.68933	2.10368	1.70571	0.404383	1.4186
XLOC_074095	CCDC146;PION	chr4	44125655	44233640	0.0	5.36343	2.4755	2.08005	1.97683	2.73373	2.38362	0.421158	2.21046
XLOC_074096	CCDC146;PION	chr4	44125655	44233640	1.83817	0.0	0.0	0.329166	1.14082	0.568745	0.654961	0.181689	0.800005
XLOC_074097	CCDC146;PION	chr4	44125655	44233640	0.0	1.31179	1.7155	0.0	0.457683	0.607242	0.332241	0.439386	0.319401
XLOC_074098	CCDC146;PION	chr4	44125655	44233640	0.0	3.09833	4.05097	0.0	0.393739	0.526727	0.438196	0.9905	0.895775
XLOC_074099	CCDC146;PION	chr4	44125655	44233640	13.0339	3.90378	10.2054	0.0	0.0	0.0	2.06145	1.18847	1.11948
XLOC_074100	CCDC146;PION	chr4	44125655	44233640	2.5422	0.506887	1.32578	0.30381	0.397591	0.263812	0.230612	0.423896	0.296113
XLOC_074101	CCDC146;PION	chr4	44125655	44233640	1.44023	0.574537	2.25406	1.07614	1.01661	0.699064	0.61387	0.38598	0.755918
XLOC_074102	CCDC146;PION	chr4	44125655	44233640	0.0	0.735929	7.69825	0.882102	0.570748	0.253207	0.434133	0.967069	0.642114
XLOC_074103	CCDC146;PION	chr4	44125655	44233640	2.99469	3.57535	0.0	1.07145	1.1512	0.920192	0.261322	0.583932	1.03847
XLOC_074104	CCDC146;PION	chr4	44125655	44233640	1.51546	1.35986	7.70598	1.49427	1.42309	1.10146	1.03276	0.607131	0.52973
XLOC_084293	CCDC151	chr7	17031434	17032357	2.83446	1.69531	2.95591	2.03222	2.80534	3.13561	1.96774	0.66089	1.15514
XLOC_084294	CCDC151	chr7	17033233	17033599	3.46738	1.03455	0.0	2.79067	4.25044	0.354225	0.90047	0.67251	1.20058
XLOC_084295	CCDC151	chr7	17033831	17034314	2.22221	4.64611	3.47148	3.77895	2.74992	4.11571	1.57264	1.53043	1.73846
XLOC_083420	CCDC151;RGL3	chr7	17004515	17027915	12.4235	17.6372	13.9136	60.999	52.6089	55.906	51.136	60.906	41.7572
XLOC_046540	CCDC164	chr11	73085451	73086299	1.04745	0.0	0.0	0.469318	0.654211	0.542812	0.37867	0.209074	0.457191
XLOC_070459	CCDC23;C3H1orf50;YBX1;PPIH;CLDN19;LEPRE1	chr3	104013363	104219992	59.0957	58.7443	64.7543	5.02569	4.98313	5.66931	5.28977	5.27224	4.23647
XLOC_071932	CCDC23;C3H1orf50;YBX1;PPIH;CLDN19;LEPRE1	chr3	104013363	104219992	13.6328	24.3891	53.1342	0.0	2.04934	2.06268	1.69092	0.0	1.75862

XLOC_071933	CCDC23;C3H1orf50;YBX1;PPIH;CLDN19;LEPRE1	chr3	104013363	104219992	22.4782	8.04321	21.0302	0.804116	0.0	0.913937	1.14868	0.0	1.55221
XLOC_071934	CCDC23;C3H1orf50;YBX1;PPIH;CLDN19;LEPRE1	chr3	104013363	104219992	15.6639	9.34446	27.4876	1.75083	1.1963	0.798263	3.02869	0.377918	2.36971
XLOC_071935	CCDC23;C3H1orf50;YBX1;PPIH;CLDN19;LEPRE1	chr3	104013363	104219992	22.0707	2.63276	17.2082	0.0	0.336008	0.448847	0.37641	0.423811	1.14328
XLOC_071936	CCDC23;C3H1orf50;YBX1;PPIH;CLDN19;LEPRE1	chr3	104013363	104219992	32.3017	15.1599	39.6446	1.65191	0.534987	2.13552	3.87206	0.22677	2.20546
XLOC_071937	CCDC23;C3H1orf50;YBX1;PPIH;CLDN19;LEPRE1	chr3	104013363	104219992	13.777	8.23551	16.7515	0.822636	0.476193	0.316363	2.19403	0.151874	1.20056
XLOC_071938	CCDC23;C3H1orf50;YBX1;PPIH;CLDN19;LEPRE1	chr3	104013363	104219992	18.6113	18.2021	29.0902	1.66666	1.44553	1.22262	1.81341	1.17274	0.736765
XLOC_071939	CCDC23;C3H1orf50;YBX1;PPIH;CLDN19;LEPRE1	chr3	104013363	104219992	8.2795	13.0845	21.272	1.48367	1.47587	1.34735	0.853061	0.942784	2.16723
XLOC_071940	CCDC23;C3H1orf50;YBX1;PPIH;CLDN19;LEPRE1	chr3	104013363	104219992	5.7482	9.15952	14.9709	1.37237	1.48554	1.38266	1.7044	0.378424	2.16757
XLOC_071941	CCDC23;C3H1orf50;YBX1;PPIH;CLDN19;LEPRE1	chr3	104013363	104219992	21.9462	10.9291	10.0035	1.96503	1.56079	1.88634	0.977639	0.723161	0.636791

XLOC_050733	CCDC3	chr13	11572246	11587440	0.0	0.496336	0.0	1.63609	0.387088	0.342968	0.296851	0.98711	0.578647
XLOC_050734	CCDC3	chr13	11594690	11640373	0.335525	0.602402	0.0	0.812412	0.579608	0.873247	0.737816	0.912495	0.587597
XLOC_063954	CCDC42;MFSD6L	chr19	28834869	28913150	1.42906	0.704843	0.614504	0.72991	0.451991	0.463833	0.288779	0.432219	0.435687
XLOC_064946	CCDC42;MFSD6L	chr19	28834869	28913150	41.8084	44.6985	24.4633	83.4985	88.6095	87.1941	95.2785	88.5055	89.8678
XLOC_064018	CCDC43	chr19	45047722	45055705	10.9386	11.7427	8.27543	0.821147	0.449214	0.607309	0.567374	0.77064	0.893466
XLOC_060266	CCDC63	chr17	56891884	56927537	15.5196	22.4232	25.3926	11.6308	11.0216	12.655	10.1659	15.9855	10.9191
XLOC_061669	CCDC63	chr17	56891884	56927537	1.95608	2.04734	0.764935	0.613624	1.22244	1.11553	0.973686	1.36782	0.597908
XLOC_061670	CCDC63	chr17	56933767	56935179	2.30606	1.37977	2.25544	0.46519	0.225903	0.359568	0.262808	0.289354	0.201656
XLOC_048102	CCDC70	chr12	21324575	21374518	35.3574	36.4694	28.1043	22.0959	20.8716	20.7393	18.4646	19.1358	25.4066
XLOC_048791	CCDC70	chr12	21375718	21375947	0.0	0.0	7.51921	0.0	2.15014	0.966831	8.54733	1.78197	2.48146
XLOC_048792	CCDC70	chr12	21377042	21377502	2.39115	0.71408	3.73478	0.641939	2.2163	0.983131	2.31974	1.1739	2.70042
XLOC_048793	CCDC70	chr12	21378138	21378998	3.08867	1.84721	2.41555	0.276786	1.68821	1.0672	2.14087	0.924963	2.24705
XLOC_048794	CCDC70	chr12	21379379	21379793	2.81973	2.52525	2.20117	1.51347	2.17076	0.867312	2.46807	1.10187	1.22298
XLOC_048795	CCDC70	chr12	21380557	21381226	1.41405	0.845366	2.21087	0.886665	0.770511	1.02357	1.01553	0.983449	2.34224
XLOC_076385	CCDC91	chr5	81646371	81795875	1.15144	2.47493	1.65593	0.482984	0.211678	0.340668	0.212931	0.329756	0.30437
XLOC_065676	CCDC93	chr2	69897321	70024315	38.3708	55.6269	45.8104	61.2393	57.0118	62.1137	82.8002	74.4876	61.4285
XLOC_067789	CCDC93	chr2	69897321	70024315	4.85979	2.42064	8.86273	4.20724	1.76252	2.50952	2.60779	1.92654	2.54022
XLOC_067790	CCDC93	chr2	69897321	70024315	0.0	7.83931	11.7134	2.68564	1.43532	3.44659	4.20498	3.62934	4.87063
XLOC_067791	CCDC93	chr2	69897321	70024315	2.63497	2.36018	2.05731	2.829	2.64088	3.51587	3.23939	2.83773	2.74469
XLOC_067792	CCDC93	chr2	69897321	70024315	0.0	4.50282	8.83051	2.69958	2.88505	3.46415	2.6003	1.45897	2.61111
XLOC_067793	CCDC93	chr2	69897321	70024315	0.0	4.80915	6.86029	0.917105	2.95918	3.02414	2.36141	3.04998	3.69734
XLOC_067794	CCDC93	chr2	69897321	70024315	0.0	2.16969	5.67213	3.26747	4.91233	6.59785	10.7776	6.82282	5.00388
XLOC_067795	CCDC93	chr2	70025434	70026328	1.96405	0.587336	0.768048	0.440034	1.22734	0.916405	1.33293	1.3732	1.02897
XLOC_063173	CCDC97	chr18	50755305	50756526	0.0	0.0	0.0	0.365788	0.319505	0.98904	0.433179	0.6136	1.07003
XLOC_063174	CCDC97	chr18	50759039	50759922	0.0	0.0	0.0	0.982738	0.778626	1.03357	0.450922	0.19911	0.609299
XLOC_063175	CCDC97	chr18	50759991	50760614	0.0	0.0	0.0	1.80914	0.724898	1.12376	0.278262	0.30823	0.541561
XLOC_063176	CCDC97	chr18	50764202	50764779	0.0	0.0	0.0	1.85279	0.535534	0.889777	0.0	0.170662	0.750726
XLOC_063177	CCDC97	chr18	50765520	50766066	0.0	0.0	0.0	1.00018	0.866456	1.34396	0.331553	0.183975	1.94456
XLOC_076749	CCER1;EPYC	chr5	20895423	20921207	8.65149	9.10245	6.51055	10.1361	12.6251	12.3896	13.198	10.3558	9.23156
XLOC_079679	CCKAR	chr6	47453671	47526915	45.114	54.0814	39.7735	59.5698	50.5595	58.4212	65.5941	74.7755	55.9125
XLOC_083101	CCNH	chr7	89404359	89413219	5.83558	2.10347	3.54311	0.654339	0.513902	0.703854	0.482263	0.577429	0.626161

XLOC_085257	CCNH	chr7	89401056	89401632	0.0	0.0	0.0	0.0	0.0	0.0	0.0	0.171062	0.0
XLOC_085258	CCNH	chr7	89402391	89403111	0.0	0.384404	0.0	0.0	0.100195	0.0	0.0	0.0	0.2243
XLOC_081732	CCNI	chr6	93648628	93649374	0.0	0.0	0.0	1.10113	1.14974	0.636184	0.221412	0.122385	0.428868
XLOC_081733	CCNI	chr6	93649447	93650602	0.72506	0.0	0.0	0.0649956	0.737799	0.301197	0.26369	0.0	0.253477
XLOC_076269	CCNT1	chr5	31187475	31221632	6.77339	7.27984	6.13149	6.79878	5.37305	6.72769	7.43079	9.19492	6.85861
XLOC_076793	CCNT1	chr5	31187475	31221632	14.1521	24.8587	13.4386	35.8514	25.0188	32.8379	34.7716	47.986	29.087
XLOC_076794	CCNT1	chr5	31187475	31221632	28.3636	34.946	27.5614	58.2847	49.5428	61.993	74.6145	100.723	50.9533
XLOC_076270	CCNT1;MIR1291;KANSL2	chr5	31240878	31291510	59.783	82.2722	63.9384	31.375	27.2326	28.9315	30.7886	31.5907	29.7371
XLOC_076795	CCNT1;MIR1291;KANSL2	chr5	31240878	31291510	5.55788	7.10011	5.44749	3.91696	4.64524	4.59799	4.56624	4.73102	4.23143
XLOC_065660	CCNT2	chr2	62664197	62692274	13.7414	10.7614	16.5845	14.2799	9.2816	11.4332	8.97223	11.3935	10.4461
XLOC_050752	CCNY	chr13	18378369	18489143	0.0	0.0	0.0	1.42799	0.310417	0.687175	0.955479	0.396346	0.23168
XLOC_038566	CCRL1;ACAD11	chr1	138064332	138080266	5.29649	1.58458	0.0	0.0	0.0	0.110145	0.0	0.0	0.0926473
XLOC_069840	CCT3;C3H1orf182;BGLAP;PAQR6;C3H1orf85;SMG5;PMF1;TMEM79	chr3	14533797	14611459	7.78	14.8723	7.70665	17.3348	18.5581	19.2878	21.6324	21.1855	24.8289
XLOC_070234	CCT3;C3H1orf182;BGLAP;PAQR6;C3H1orf85;SMG5;PMF1;TMEM79	chr3	14533797	14611459	9.57532	7.72195	11.4219	12.0547	10.0459	11.4713	13.5244	16.8364	13.0398
XLOC_044808	CCT4	chr11	60400813	60417081	9.01059	9.9047	8.96463	22.7446	18.072	19.3778	19.4386	24.6426	18.5716
XLOC_046308	CCT4	chr11	60400813	60417081	4.8536	11.5755	0.0	5.64428	5.89498	6.40393	3.28966	6.03226	2.9301
XLOC_037854	CD200R1L	chr1	58113161	58142764	0.0	0.0	0.0	2.75039	0.59987	1.59215	0.23195	1.02328	1.71265
XLOC_065757	CD28	chr2	92184299	92361568	31.8975	49.7938	47.7539	38.3561	36.6984	41.636	25.1052	30.5461	33.9632
XLOC_068118	CD28	chr2	92184299	92361568	4.49715	4.92859	2.34355	2.1483	2.3322	1.70426	1.61235	1.19027	1.4371
XLOC_068119	CD28	chr2	92184299	92361568	13.7404	5.46251	3.57076	2.86744	1.74139	7.91157	2.3376	1.31708	0.395265
XLOC_068120	CD28	chr2	92184299	92361568	7.00625	4.17827	0.0	3.14465	5.2618	9.88952	4.04727	6.58243	6.6267
XLOC_064060	CD300A	chr19	57611000	57642624	10.6621	8.50474	9.45345	2.99479	3.28303	3.02685	3.36147	2.63563	2.91996
XLOC_079875	CD38	chr6	115774537	115805120	3.62354	2.76514	2.03314	5.8471	5.64605	5.70539	5.40163	8.34736	4.88511

XLOC_079876	CD38	chr6	115853505	115926694	14.8361	17.864	12.1145	5.83441	6.21613	5.34854	5.45017	3.47622	5.92625
XLOC_080275	CD38	chr6	115805441	115809236	262.674	294.545	232.492	549.421	579.491	598.919	559.858	552.706	671.581
XLOC_080276	CD38	chr6	115810368	115838645	2.47307	2.59922	2.9146	1.91622	1.692	1.60056	2.09655	2.2591	1.37611
XLOC_080277	CD38	chr6	115842076	115851027	4.07033	11.0541	7.29121	12.6005	12.8079	13.6584	13.6107	13.2861	15.3174
XLOC_082219	CD38	chr6	115774537	115805120	0.0	0.90127	0.0	1.08036	2.08901	0.927861	0.526787	2.94316	0.0
XLOC_082220	CD38	chr6	115810368	115838645	0.0	1.08032	0.9418	1.07915	0.563492	0.748295	0.759799	0.719851	0.525418
XLOC_082221	CD38	chr6	115839480	115840686	1.37958	1.03168	1.07931	0.185509	0.756118	0.716521	0.251027	0.414869	0.422061
XLOC_082222	CD38	chr6	115841802	115841969	0.0	45.5227	47.6204	65.615	45.4654	36.0269	45.4283	31.6626	54.4478
XLOC_082223	CD38	chr6	115842076	115851027	2.75916	3.30138	1.07931	2.72079	2.64641	2.22122	2.51027	2.97323	3.0148
XLOC_082224	CD38	chr6	115852822	115853374	3.66698	4.93051	4.29808	7.87999	6.68551	5.8623	5.88021	4.34972	6.70312
XLOC_055788	CD44	chr15	66447596	66489556	0.0	0.0	0.842315	0.0	0.168141	0.0	0.0	0.0	0.0940155
XLOC_038350	CD47	chr1	53158580	53187610	0.0	1.65184	0.0	10.2703	6.48608	11.2522	8.63774	5.813	7.57735
XLOC_039301	CD47	chr1	53158580	53187610	0.0	1.58263	0.0	0.475132	0.803947	3.22738	1.34098	6.06544	0.914958
XLOC_070249	CD53	chr3	32584149	32617217	19.6719	16.2198	15.9895	4.01375	4.06787	3.0991	4.82418	3.76909	5.06499
XLOC_057757	CD55	chr16	5008678	5132429	2.29519	0.0	3.669	0.0	3.90832	3.12126	1.30337	0.0	0.0
XLOC_057758	CD55	chr16	5008678	5132429	26.3977	28.2154	31.9544	9.40694	9.72253	9.53646	12.5925	8.80769	9.78813
XLOC_058006	CD55	chr16	5008678	5132429	77.8704	83.7455	72.541	80.454	68.5922	73.6079	82.8124	99.1298	80.607
XLOC_058007	CD55	chr16	5008678	5132429	1.07142	0.67658	1.39947	0.719627	0.721317	0.544222	0.309099	0.298938	0.300071
XLOC_058315	CD55	chr16	5008678	5132429	18.5825	5.54814	14.5034	9.23037	6.92287	5.59872	16.532	3.44554	4.78836
XLOC_055809	CD82	chr15	75564917	75565841	0.0	0.411436	0.0	0.0	0.0	0.0	0.0	0.273625	0.120008
XLOC_078483	CD9	chr5	104505132	104506019	7.9316	9.19099	10.8557	4.62022	4.49147	3.80337	4.03645	5.34685	4.67462
XLOC_078484	CD9	chr5	104508627	104509500	4.0434	2.72051	0.790568	2.62703	1.89461	1.4671	1.82843	1.41301	1.41208
XLOC_078485	CD9	chr5	104509665	104510207	1.88118	2.24823	5.87955	2.358	2.04245	1.55175	2.51164	1.30092	1.80093
XLOC_078486	CD9	chr5	104510986	104512071	3.11914	0.233236	1.83001	1.11835	1.28122	1.13337	2.12478	1.17119	1.70346
XLOC_078487	CD9	chr5	104512449	104513317	2.03593	1.21762	2.38838	1.27714	1.58986	1.26631	1.19668	0.813046	0.799862
XLOC_078488	CD9	chr5	104513641	104513970	0.0	6.27803	0.0	0.753006	1.6046	5.1423	6.12255	1.21524	2.18211
XLOC_078489	CD9	chr5	104514527	104516180	2.41803	0.28938	2.64901	0.953969	0.948197	1.30778	1.10419	0.388802	0.972988
XLOC_078490	CD9	chr5	104516380	104521951	8.81723	9.91598	10.7716	5.27254	7.17637	7.0741	8.73197	5.48793	6.658
XLOC_037849	CD96	chr1	56796467	56974782	1.04594	0.312761	0.0	0.0	0.163318	0.108406	0.0	0.313163	0.0
XLOC_038366	CD96	chr1	56796467	56974782	1.10775	1.07736	2.38431	0.422225	0.326336	0.346093	0.457026	0.418531	0.436385
XLOC_039338	CD96	chr1	56793673	56795603	0.0	0.610433	0.957942	0.21953	0.1601	0.127375	0.261222	0.369389	0.107107
XLOC_039339	CD96	chr1	56795721	56796389	0.0	2.11755	2.21519	0.0	0.220572	0.146509	0.508735	0.563053	0.123516
XLOC_039340	CD96	chr1	56796467	56974782	0.548946	0.492686	0.42953	0.0984351	0.0860593	0.0	0.0	0.0	0.0960161

XLOC_039341	CD96	chr1	56796467	56974782	0.0	0.111291	0.0	0.0333532	0.0	0.0774229	0.136158	0.0748587	0.0325485
XLOC_039342	CD96	chr1	56796467	56974782	0.0	0.0	0.310898	0.0	0.0935353	0.0413417	0.0	0.0799327	0.0695247
XLOC_039343	CD96	chr1	56796467	56974782	0.0	0.103819	0.271536	0.0622277	0.0272407	0.108351	0.0953041	0.174631	0.182191
XLOC_039344	CD96	chr1	56796467	56974782	0.0	0.335585	0.0	0.201143	0.263759	0.058307	0.306899	0.112622	0.0
XLOC_086528	CDC14B	chr8	84667667	84668948	1.93468	2.50789	5.0455	2.02348	2.27301	2.74727	1.4093	1.68169	1.635
XLOC_086529	CDC14B	chr8	84669680	84672242	13.7601	17.9406	19.7437	41.8382	37.8442	40.5211	42.6724	49.515	30.5105
XLOC_086530	CDC14B	chr8	84672357	84699811	21.4333	23.5432	27.8625	45.7788	45.1246	44.7863	45.4772	50.064	35.7254
XLOC_086981	CDC14B	chr8	84672357	84699811	40.6241	45.7976	38.2063	85.221	82.3092	84.1437	76.9374	95.9755	61.2121
XLOC_086982	CDC14B	chr8	84707755	84772321	12.1679	14.2979	12.4653	0.155347	0.163368	0.413132	0.300926	0.132456	0.0909678
XLOC_088442	CDC14B	chr8	84639649	84640427	2.33126	4.18212	1.82294	13.8906	17.0923	15.9353	17.8702	18.7022	14.748
XLOC_088443	CDC14B	chr8	84655942	84657245	0.631376	0.566621	0.987972	1.47168	0.593497	0.590474	0.460038	1.0767	0.828005
XLOC_088444	CDC14B	chr8	84659207	84659796	3.3525	1.50279	1.31005	2.4018	2.21356	1.73057	1.94688	0.996075	2.18992
XLOC_088445	CDC14B	chr8	84659907	84660746	3.18386	1.26938	2.48991	2.56775	3.1482	4.1795	0.958934	1.2708	1.48226
XLOC_088446	CDC14B	chr8	84660887	84663673	15.133	13.3387	11.8445	36.5947	38.3888	40.8045	39.4802	41.1675	22.2323
XLOC_088447	CDC14B	chr8	84663854	84664396	11.2871	0.562058	5.87955	2.86328	2.62601	3.10349	1.67443	0.929226	1.80093
XLOC_088448	CDC14B	chr8	84665021	84665902	2.99902	2.39152	2.3455	2.77718	1.87383	2.07282	1.89901	1.99654	1.22195
XLOC_088449	CDC14B	chr8	84666840	84667115	0.0	1.81706	9.50136	4.91539	4.59643	7.39199	2.54064	2.88263	0.524611
XLOC_088450	CDC14B	chr8	84672357	84699811	0.0	1.78482	1.16695	2.94173	2.20656	2.77739	3.74705	2.22271	1.30109
XLOC_088451	CDC14B	chr8	84672357	84699811	3.66903	2.74317	2.86977	2.63066	1.79217	2.37864	2.16061	2.01717	1.84255
XLOC_088452	CDC14B	chr8	84702645	84703283	0.0	0.0	0.0	0.0	0.234179	0.0	0.134907	0.149392	0.0
XLOC_088453	CDC14B	chr8	84704056	84704707	0.0	0.438079	0.0	0.26256	0.342123	0.303021	0.394343	0.145526	0.127749
XLOC_088454	CDC14B	chr8	84704956	84706234	6.45464	6.37182	6.56501	0.0	0.0	0.0	0.0587724	0.0	0.112858
XLOC_088455	CDC14B	chr8	84706956	84707544	3.36029	10.0418	3.93925	0.0	0.261016	0.0	0.0	0.16639	0.0
XLOC_086527	CDC14B;HABP4	chr8	84559535	84638714	3.36128	4.60288	8.39963	16.1664	16.9332	14.8866	12.6719	10.7982	11.2429
XLOC_088438	CDC14B;HABP4	chr8	84559535	84638714	0.0	2.88363	0.0	2.59262	2.22497	1.64747	3.35981	1.56611	1.39515
XLOC_088439	CDC14B;HABP4	chr8	84559535	84638714	0.0	0.0	0.0	1.71383	5.09264	4.86272	1.21861	2.74925	2.06586
XLOC_088440	CDC14B;HABP4	chr8	84559535	84638714	0.0	0.526515	0.0	1.89335	2.59878	2.3636	1.10023	1.56892	1.53417
XLOC_088441	CDC14B;HABP4	chr8	84559535	84638714	0.0	0.0	1.14802	0.131548	1.25697	0.151815	0.395123	0.729083	0.640034
XLOC_086604	CDC26	chr8	104215060	104217358	0.0	0.572462	0.0	0.0	0.0	0.0	0.0	0.0	0.0
XLOC_088642	CDC26	chr8	104217608	104218256	1.4746	0.88151	2.30539	0.0	0.0	0.0	0.0	0.1464	0.0

XLOC_088643	CDC26	chr8	104218309	104218962	0.0	0.436314	2.28215	0.0	0.113586	0.150904	0.0	0.0	0.0
XLOC_064033	CDC27;ITGB3;MYL4	chr19	46761485	47025401	9.63837	11.4488	4.33851	13.2	13.5287	12.8912	11.7055	17.338	13.247
XLOC_064034	CDC27;ITGB3;MYL4	chr19	46761485	47025401	2.73558	3.07001	2.67652	14.0871	8.85032	13.394	4.35128	9.2847	3.59281
XLOC_064301	CDC27;ITGB3;MYL4	chr19	46761485	47025401	32.8539	49.1247	39.4307	35.1499	24.4644	30.0811	24.958	29.8051	21.6432
XLOC_065249	CDC27;ITGB3;MYL4	chr19	46761485	47025401	2.12647	2.29117	1.6646	3.4587	3.16632	3.01437	2.43668	3.67692	2.74405
XLOC_065250	CDC27;ITGB3;MYL4	chr19	46761485	47025401	1.3093	1.3713	1.2809	1.7319	1.95347	1.90836	1.07934	1.45016	1.00273
XLOC_065251	CDC27;ITGB3;MYL4	chr19	46761485	47025401	0.0	0.69381	0.604793	0.900899	1.27033	1.44479	0.421374	0.774181	0.540459
XLOC_065252	CDC27;ITGB3;MYL4	chr19	46761485	47025401	0.659593	0.19738	0.0	1.36001	1.13645	2.26143	0.900646	0.925995	0.46129
XLOC_059168	CDC2L1	chr16	52249112	52249570	0.0	1.43764	5.63936	0.2154	0.185897	0.0	0.424457	0.0	0.209095
XLOC_090643	CDC40	chr9	40489563	40489912	0.0	0.0	2.9435	0.0	0.0	0.0	0.0	0.0	0.0
XLOC_089838	CDC40;C9H6orf186	chr9	40315127	40489424	24.3621	15.7582	14.6585	5.37004	5.98797	5.45293	5.20988	5.85349	7.67547
XLOC_090640	CDC40;C9H6orf186	chr9	40315127	40489424	1.78815	1.60318	2.79476	0.160123	0.0	0.184507	0.0	0.0	0.0
XLOC_090641	CDC40;C9H6orf186	chr9	40315127	40489424	0.0	0.0	1.27761	0.0	0.0	0.0	0.0	0.0	0.0
XLOC_066564	CDC42	chr2	131301963	131352836	14.3976	9.6963	15.4965	1.97559	1.88694	1.92932	2.01468	2.03611	1.16776
XLOC_068633	CDC42	chr2	131301963	131352836	35.9772	53.3183	29.2945	118.291	96.6699	111.397	130.869	147.742	146.976
XLOC_068634	CDC42	chr2	131301963	131352836	0.0	8.15353	1.6403	0.0	0.487823	0.432404	0.558455	0.620904	0.182646
XLOC_058091	CDC42BPA	chr16	30955944	31081086	4.42205	3.44502	3.92084	11.1828	9.58657	10.6997	11.7919	12.176	9.74425
XLOC_058748	CDC42BPA	chr16	30868758	30869028	0.0	0.0	0.0	0.0	0.0	0.0	0.528154	1.19973	0.0
XLOC_058749	CDC42BPA	chr16	30955342	30955560	22.2028	0.0	8.6765	18.1372	9.87315	16.6916	10.6445	17.3395	13.3462
XLOC_044676	CDC42EP3	chr11	20062601	20113382	20.4509	18.4978	21.0819	60.233	62.2834	64.1876	71.0838	77.7975	56.1342
XLOC_082823	CDC42SE2	chr7	24370199	24385214	0.777873	0.465458	1.16432	0.279017	0.152633	0.242861	0.0711192	0.0390814	0.068018
XLOC_082824	CDC42SE2	chr7	24399137	24422850	10.7045	9.51552	8.35536	14.4134	17.8187	14.1674	14.269	12.7813	13.5757
XLOC_070284	CDC7	chr3	51856113	52104873	7.01791	10.2758	9.86763	0.122583	0.0567621	0.254914	0.0992788	0.0	0.0316359
XLOC_071135	CDC7	chr3	51856113	52104873	0.0	0.948594	0.0	0.0	0.0	0.0	0.0	0.0	0.0
XLOC_071136	CDC7	chr3	51856113	52104873	0.0	1.36967	0.0	0.0	0.0	0.315701	0.0	0.0	0.0
XLOC_071137	CDC7	chr3	51856113	52104873	0.0	3.00496	0.0	0.0	0.0	0.25841	0.0	0.0	0.0
XLOC_071139	CDC7	chr3	51856113	52104873	0.0	0.0	1.79063	0.0	0.0	0.0	0.0	0.0	0.0
XLOC_071141	CDC7	chr3	51856113	52104873	0.0	5.98536	0.0	0.0	0.0	0.0	0.0	0.0	0.0

XLOC_071144	CDC7	chr3	51856113	52104873	0.0	0.28425	0.0	0.0	0.0	0.0494099	0.0	0.0	0.0
XLOC_071145	CDC7	chr3	51856113	52104873	0.0	0.656914	0.859061	0.0492175	0.0	0.0	0.0	0.0	0.0
XLOC_071146	CDC7	chr3	51856113	52104873	0.0	0.67197	0.0	0.0	0.0	0.0	0.0	0.0	0.0
XLOC_071147	CDC7	chr3	51856113	52104873	0.976721	0.584166	0.0	0.0	0.0	0.0	0.0	0.0	0.0
XLOC_071148	CDC7	chr3	51856113	52104873	0.0	0.982991	0.0	0.0	0.0	0.0	0.0	0.0	0.0
XLOC_071150	CDC7	chr3	51856113	52104873	0.0	2.12866	0.0	0.0	0.0	0.0	0.0	0.0	0.0
XLOC_071151	CDC7	chr3	51856113	52104873	0.0	1.24002	3.24284	0.0	0.0	0.0	0.0	0.0	0.0
XLOC_071152	CDC7	chr3	51856113	52104873	0.0	0.0	2.42381	0.0	0.120561	0.0	0.0	0.0	0.0
XLOC_071153	CDC7	chr3	51856113	52104873	1.13494	0.5093	1.77606	0.0	0.0444748	0.0	0.0	0.0	0.0
XLOC_071154	CDC7	chr3	51856113	52104873	0.0	1.66647	0.0	0.0	0.0	0.0	0.0	0.0	0.0
XLOC_071155	CDC7	chr3	51856113	52104873	0.0	0.389242	2.03597	0.0	0.0	0.0	0.0	0.0	0.0
XLOC_071156	CDC7	chr3	51856113	52104873	0.0	0.475327	0.994569	0.0	0.0249491	0.0	0.0	0.0	0.0
XLOC_071158	CDC7	chr3	51856113	52104873	0.0	0.721215	1.88605	0.0	0.0	0.0	0.0	0.0	0.0
XLOC_071159	CDC7	chr3	51856113	52104873	0.827994	0.495287	1.94305	0.0	0.0	0.0	0.0	0.0	0.0
XLOC_071160	CDC7	chr3	51856113	52104873	0.0	0.879328	0.0	0.0	0.0	0.0	0.0	0.0	0.0
XLOC_071161	CDC7	chr3	51856113	52104873	0.0	0.293668	2.30414	0.0	0.0	0.0	0.0	0.0	0.0
XLOC_071162	CDC7	chr3	51856113	52104873	0.0	0.0	4.99553	0.0	0.0	0.0	0.0	0.0	0.0
XLOC_071163	CDC7	chr3	51856113	52104873	0.0	0.438147	0.0	0.0	0.0	0.0	0.0	0.0	0.0
XLOC_071164	CDC7	chr3	51856113	52104873	0.0	1.06338	0.0	0.0	0.0	0.0	0.0	0.0	0.0
XLOC_071165	CDC7	chr3	51856113	52104873	0.0	0.254335	1.99556	0.0762201	0.0	0.0	0.0	0.0850751	0.0
XLOC_071166	CDC7	chr3	51856113	52104873	2.37545	0.7094	0.0	0.0	0.0	0.0	0.0	0.0	0.0
XLOC_071167	CDC7	chr3	51856113	52104873	0.0	2.32696	0.0	0.0	0.0	0.0	0.0	0.0	0.0
XLOC_071168	CDC7	chr3	51856113	52104873	0.390406	0.350449	0.611059	0.0	0.0306416	0.0	0.0357177	0.0	0.0
XLOC_071170	CDC7	chr3	51856113	52104873	1.02956	1.53934	0.0	0.0	0.0	0.0	0.0	0.0	0.089882
XLOC_071171	CDC7	chr3	51856113	52104873	0.0	0.700223	1.83116	0.0	0.0	0.0	0.0	0.0	0.0
XLOC_071172	CDC7	chr3	51856113	52104873	5.30859	0.792477	0.0	0.0	0.0	0.0	0.0	0.0	0.0
XLOC_071173	CDC7	chr3	51856113	52104873	0.0	2.19286	3.82302	0.0	0.0	0.0	0.0	0.0	0.0
XLOC_071175	CDC7	chr3	51856113	52104873	8.67274	2.5883	6.76619	0.0	0.0	0.0	0.0	0.0	0.0
XLOC_071176	CDC7	chr3	51856113	52104873	1.24381	1.11557	0.0	0.0	0.0969536	0.0	0.0	0.0	0.0
XLOC_071177	CDC7	chr3	51856113	52104873	12.2656	3.67118	0.0	0.0	0.0	0.0	0.0	0.0	0.0
XLOC_071178	CDC7	chr3	51856113	52104873	0.0	0.0	2.95882	0.0	0.0	0.0	0.0	0.0	0.0
XLOC_071179	CDC7	chr3	51856113	52104873	3.18854	0.953435	0.831187	0.0	0.0	0.110147	0.0	0.0	0.0
XLOC_071181	CDC7	chr3	51856113	52104873	0.0	0.547834	1.43269	0.0	0.0	0.0	0.0	0.0	0.0

XLOC_071182	CDC7	chr3	51856113	52104873	0.0	0.580252	1.51757	0.0	0.0757899	0.0	0.0	0.0	0.0
XLOC_071184	CDC7	chr3	51856113	52104873	0.952285	0.569565	2.23443	0.0	0.0	0.0	0.0	0.0	0.0
XLOC_071185	CDC7	chr3	51856113	52104873	0.0	0.523966	1.37028	0.0	0.0	0.0	0.0	0.0	0.0
XLOC_071187	CDC7	chr3	51856113	52104873	0.0	0.250509	0.655179	0.0	0.0	0.0869242	0.0759943	0.0	0.0
XLOC_071188	CDC7	chr3	51856113	52104873	0.0	1.4162	3.0866	0.0	0.0	0.0819218	0.0	0.0	0.0
XLOC_071189	CDC7	chr3	51856113	52104873	0.789144	1.18016	0.61732	0.0	0.0	0.0	0.0	0.0	0.0
XLOC_071190	CDC7	chr3	51856113	52104873	0.730184	0.436821	1.7137	0.0	0.0	0.0	0.066382	0.0	0.0
XLOC_071191	CDC7	chr3	51856113	52104873	2.2154	0.661701	1.73044	0.198284	0.0	0.22796	0.0	0.0	0.0
XLOC_071192	CDC7	chr3	51856113	52104873	0.0	0.891893	0.0	0.0	0.0	0.103075	0.0	0.0	0.0
XLOC_071193	CDC7	chr3	51856113	52104873	0.0	0.602037	0.78727	0.0	0.0	0.0	0.0	0.0	0.0
XLOC_071194	CDC7	chr3	51856113	52104873	0.0	0.946487	0.82513	0.0	0.0	0.218698	0.0	0.0	0.0
XLOC_071195	CDC7	chr3	51856113	52104873	0.804955	0.240758	3.14839	0.0	0.0	0.0	0.0	0.0	0.0
XLOC_071196	CDC7	chr3	51856113	52104873	0.0	1.42816	0.0	0.0	0.0	0.0	0.0	0.0	0.0
XLOC_071197	CDC7	chr3	51856113	52104873	0.0	1.87116	3.26249	0.0	0.0	0.0	0.0	0.0	0.0
XLOC_071199	CDC7	chr3	51856113	52104873	0.0	0.821311	2.14796	0.0	0.0	0.0	0.0	0.0	0.0
XLOC_071200	CDC7	chr3	51856113	52104873	2.37545	1.4188	3.71031	0.212577	0.0	0.0	0.209559	0.0	0.206372
XLOC_071201	CDC7	chr3	51856113	52104873	0.0	0.466974	0.610662	0.0	0.0	0.0	0.0	0.0	0.0
XLOC_071202	CDC7	chr3	51856113	52104873	0.0	1.96598	2.57041	0.0	0.252699	0.0	0.0	0.0	0.0
XLOC_071203	CDC7	chr3	51856113	52104873	0.0	0.0	0.0	0.0	0.150517	0.0	0.0	0.0	0.168959
XLOC_071204	CDC7	chr3	51856113	52104873	0.0	2.27496	11.8972	0.0	0.0	0.0	0.0	0.0	0.0
XLOC_071205	CDC7	chr3	51856113	52104873	6.75526	4.02823	5.26567	0.0	0.0	0.0	0.0	0.0	0.0
XLOC_071206	CDC7	chr3	51856113	52104873	0.760914	0.455191	0.595254	0.0	0.0595431	0.0	0.0	0.0	0.0
XLOC_071207	CDC7	chr3	51856113	52104873	0.0	0.15428	1.61405	0.0924723	0.0	0.107248	0.0	0.0	0.0
XLOC_071208	CDC7	chr3	51856113	52104873	0.0	1.5941	8.33584	0.0	0.0	0.541724	0.0	0.508947	0.460766
XLOC_071209	CDC7	chr3	51856113	52104873	0.0	1.2413	3.24574	0.0	0.0	0.0	0.0	0.0	0.0
XLOC_071210	CDC7	chr3	51856113	52104873	0.0	0.602037	0.0	0.0	0.0	0.0	0.0	0.0	0.0
XLOC_071211	CDC7	chr3	51856113	52104873	0.0	0.694784	0.0	0.0	0.0	0.0	0.0	0.0	0.0
XLOC_071212	CDC7	chr3	51856113	52104873	2.5545	0.382081	1.49896	0.114505	0.0	0.0	0.0	0.0	0.0
XLOC_071213	CDC7	chr3	51856113	52104873	0.0	0.512889	0.670701	0.0	0.0	0.0	0.0	0.0	0.0
XLOC_071215	CDC7	chr3	51856113	52104873	8.67274	0.0	0.0	0.0	0.0	0.0	0.0	0.0	0.0
XLOC_071216	CDC7	chr3	51856113	52104873	0.0	0.376384	1.96872	0.0	0.0	0.130314	0.0	0.0	0.0
XLOC_071218	CDC7	chr3	51856113	52104873	0.0	1.07673	0.0	0.0	0.0	0.0	0.0	0.0	0.0
XLOC_071219	CDC7	chr3	51856113	52104873	0.0	0.0	0.809789	0.0	0.0	0.0	0.0	0.0	0.0

XLOC_071220	CDC7	chr3	51856113	52104873	0.0	0.592843	0.0	0.0	0.0	0.0	0.0	0.0	0.0
XLOC_071221	CDC7	chr3	51856113	52104873	0.638061	0.763489	0.998424	0.0	0.0	0.0663001	0.0	0.0	0.0
XLOC_071222	CDC7	chr3	51856113	52104873	0.0	0.985026	0.0	0.110702	0.0645848	0.0	0.0	0.0	0.0
XLOC_071223	CDC7	chr3	51856113	52104873	0.0	2.93262	0.0	0.0	0.373232	0.499058	0.0	0.469931	0.0
XLOC_071224	CDC7	chr3	51856113	52104873	0.597334	1.07249	0.701273	0.16071	0.0469174	0.0621998	0.0	0.0300834	0.0261438
XLOC_071225	CDC7	chr3	51856113	52104873	0.0	1.21355	0.0	0.0	0.105387	0.0	0.0	0.0	0.0
XLOC_071227	CDC7	chr3	51856113	52104873	0.0	4.28417	0.0	0.0	0.0	0.0	0.0	0.0	0.0
XLOC_071228	CDC7	chr3	51856113	52104873	0.615262	0.0	1.44416	0.110319	0.0482021	0.0	0.0	0.0	0.0
XLOC_071229	CDC7	chr3	51856113	52104873	0.975403	0.583379	0.762874	0.0	0.0761956	0.0	0.0	0.0	0.0
XLOC_071230	CDC7	chr3	51856113	52104873	0.0	0.0	3.38048	0.0	0.0	0.0	0.0	0.0	0.0
XLOC_071231	CDC7	chr3	51856113	52104873	0.0	0.549331	0.0	0.0	0.0	0.0	0.0	0.0	0.0
XLOC_071232	CDC7	chr3	51856113	52104873	0.0	0.583259	1.52532	0.0	0.0	0.0	0.0	0.0	0.0
XLOC_071233	CDC7	chr3	51856113	52104873	0.0	4.38136	5.72713	0.0	0.0	0.0	0.0	0.0	0.0
XLOC_071234	CDC7	chr3	51856113	52104873	1.76632	1.0556	5.52122	0.0	0.0	0.0	0.0	0.0	0.0
XLOC_071235	CDC7	chr3	51856113	52104873	0.275146	1.81143	0.646061	0.0	0.0216153	0.0	0.0	0.0	0.0
XLOC_071236	CDC7	chr3	51856113	52104873	0.0	0.0	0.0	0.0	0.219586	0.0	0.0	0.0	0.0
XLOC_071237	CDC7	chr3	51856113	52104873	0.0	1.31978	0.0	0.0	0.0	0.0763662	0.0	0.0	0.0
XLOC_071238	CDC7	chr3	51856113	52104873	1.44513	0.863921	0.0	0.0	0.0	0.0	0.0	0.0	0.0
XLOC_071239	CDC7	chr3	51856113	52104873	4.99242	0.74628	0.975877	0.0	0.0	0.0	0.0	0.0	0.0
XLOC_071240	CDC7	chr3	51856113	52104873	0.0	5.23528	6.84286	0.79076	0.0	0.0	0.0	0.0	0.0
XLOC_071241	CDC7	chr3	51856113	52104873	2.27265	0.679517	3.55433	0.0	0.0	0.0	0.0	0.0	0.0
XLOC_071242	CDC7	chr3	51856113	52104873	5.94019	1.77305	4.63644	0.0	0.0	0.0	0.0	0.0	0.0
XLOC_071243	CDC7	chr3	51856113	52104873	0.0	0.897537	7.04104	0.0	0.0	0.0	0.0	0.0	0.0
XLOC_071244	CDC7	chr3	51856113	52104873	0.0	1.04904	0.914564	0.0523975	0.0	0.0	0.0	0.0	0.0
XLOC_071246	CDC7	chr3	51856113	52104873	0.856429	0.768423	1.33981	0.0	0.0669658	0.0888695	0.0	0.0	0.0
XLOC_071247	CDC7	chr3	51856113	52104873	1.15816	1.7314	2.71691	0.0	0.0	0.119954	0.0	0.0	0.0
XLOC_071248	CDC7	chr3	51856113	52104873	1.39227	0.832364	4.35373	0.124719	0.0	0.0	0.250073	0.0	0.0
XLOC_071249	CDC7	chr3	51856113	52104873	3.35624	0.334509	0.874852	0.0	0.0	0.0	0.0	0.0	0.0
XLOC_071250	CDC7	chr3	51856113	52104873	0.975403	0.583379	2.28862	0.0	0.0	0.303421	0.0	0.0	0.0
XLOC_071251	CDC7	chr3	51856113	52104873	3.05975	0.915194	0.5984	0.0685675	0.0	0.0	0.0694967	0.0	0.0
XLOC_071252	CDC7	chr3	51856113	52104873	0.0	0.0	5.00476	0.0	0.0	0.0	0.0	0.0	0.0
XLOC_071253	CDC7	chr3	51856113	52104873	0.0	0.835259	0.0	0.0	0.0	0.0	0.0	0.0	0.0
XLOC_071254	CDC7	chr3	51856113	52104873	0.0	0.488496	1.27753	0.0	0.254029	0.0	0.0	0.0	0.0

XLOC_071255	CDC7	chr3	51856113	52104873	0.0	0.0	6.04344	0.0	0.0	0.0	0.0	0.0	0.0
XLOC_071256	CDC7	chr3	51856113	52104873	0.760115	0.909426	1.18926	0.0	0.0594809	0.0	0.0	0.0	0.0664244
XLOC_071257	CDC7	chr3	51856113	52104873	1.37192	0.684126	3.93647	0.0410053	0.035873	0.142715	0.0	0.0	0.0400072
XLOC_071258	CDC7	chr3	51856113	52104873	1.15501	0.6912	0.903908	0.0345246	0.0	0.0	0.0352268	0.0387382	0.0336905
XLOC_071259	CDC7	chr3	51856113	52104873	0.0	1.8696	1.22237	0.0	0.0	0.161569	0.0	0.0	0.0
XLOC_071260	CDC7	chr3	51856113	52104873	0.0	1.03388	0.0	0.0	0.0	0.23877	0.0	0.0	0.0
XLOC_071261	CDC7	chr3	51856113	52104873	0.0	0.0	8.22005	0.0	0.0	0.0	0.0	0.0	0.0
XLOC_071262	CDC7	chr3	51856113	52104873	9.51761	0.0	0.0	0.0	0.0	0.483175	0.0	0.0	0.821034
XLOC_071263	CDC7	chr3	51856113	52104873	1.19451	0.357135	4.67011	0.0	0.0	0.0	0.0	0.0	0.0
XLOC_071264	CDC7	chr3	51856113	52104873	1.23367	1.66074	2.8957	0.0	0.0483248	0.0641032	0.0561957	0.0	0.0
XLOC_071265	CDC7	chr3	51856113	52104873	2.28756	0.912301	0.596509	0.136702	0.0	0.0	0.06928	0.0	0.0
XLOC_071266	CDC7	chr3	51856113	52104873	3.36447	1.51022	3.07221	0.0754343	0.0	0.0583965	0.0771111	0.0	0.0
XLOC_070494	CDCA8;C3H1orf 109	chr3	108793179	108813165	17.6531	19.3332	17.473	6.87396	7.4967	6.54108	5.96466	5.89764	7.66138
XLOC_072088	CDCA8;C3H1orf 109	chr3	108793179	108813165	2.22906	0.665802	3.48216	0.0	1.03434	0.458692	1.38013	0.0	0.387532
XLOC_062180	CDH11	chr18	32837182	32866400	36.7722	45.8216	40.2163	72.6277	61.293	71.6573	76.2851	82.4805	67.3022
XLOC_062858	CDH11	chr18	32867242	32867992	2.44144	2.18977	0.954491	0.656223	1.04688	0.88469	0.879804	0.850997	1.27796
XLOC_062859	CDH11	chr18	32869018	32869863	1.05202	0.314579	0.0	0.65991	0.739172	0.436132	0.665509	0.314962	1.10203
XLOC_062860	CDH11	chr18	32870661	32871312	1.46563	0.0	1.1457	1.05024	0.798288	1.66662	0.0	0.145526	0.383246
XLOC_062136	CDH13	chr18	9707642	9877338	16.2775	7.23561	7.88528	3.09782	1.05129	2.0263	2.58579	2.00085	2.72138
XLOC_062137	CDH13	chr18	9957758	10030117	14.9939	17.6215	16.7102	19.2779	18.4179	19.1906	24.8263	29.6653	27.4677
XLOC_062354	CDH13	chr18	9311878	9450140	17.7834	19.0741	17.0026	11.7565	10.8508	11.6951	13.3677	11.7258	12.0591
XLOC_062355	CDH13	chr18	9957758	10030117	6.70133	7.6736	6.61983	15.1476	16.5686	16.9375	20.8107	16.1785	15.4498
XLOC_062616	CDH13	chr18	9212122	9213926	5.27104	2.89137	4.81235	3.62362	2.51571	2.42204	1.56585	1.81085	1.76779
XLOC_062617	CDH13	chr18	9214033	9218129	1.83627	2.03336	1.86858	0.971729	0.851744	0.82279	0.99503	0.592713	0.691464
XLOC_062618	CDH13	chr18	9309143	9311195	0.763502	0.342682	0.597518	0.308098	0.539358	0.278115	0.419177	0.3073	0.567909
XLOC_062619	CDH13	chr18	9495635	9496666	0.827994	2.22879	0.0	0.593718	0.4533	0.515605	0.826513	0.33143	0.578707
XLOC_062620	CDH13	chr18	9592929	9593142	0.0	0.0	0.0	1.08369	0.881033	2.38648	1.89475	0.0	0.0
XLOC_062621	CDH13	chr18	9877729	9878144	0.0	1.67698	0.0	0.251269	0.216251	0.864002	0.491793	0.274435	0.243655
XLOC_062622	CDH13	chr18	9880196	9880574	0.0	0.0	0.0	0.0	0.251584	0.670695	0.284769	0.318679	0.0
XLOC_062623	CDH13	chr18	9916838	9917261	2.72423	1.62661	2.12681	40.2136	30.8518	36.6102	31.289	41.2974	36.8764
XLOC_062624	CDH13	chr18	9917610	9917901	0.0	1.60573	0.0	7.71392	6.52277	7.63847	8.60806	7.17485	6.0333
XLOC_062625	CDH13	chr18	9957758	10030117	10.3948	7.76552	18.9546	6.9812	8.20548	10.1915	11.5968	8.05876	13.2757

XLOC_062626	CDH13	chr18	9957758	10030117	0.0	3.14263	0.0	2.83027	3.19346	3.73885	6.21631	4.51806	4.99661
XLOC_062627	CDH13	chr18	9957758	10030117	4.48576	2.67956	5.25555	3.41255	6.41729	3.23048	3.37169	2.42673	3.31407
XLOC_062628	CDH13	chr18	9957758	10030117	2.13686	2.55316	8.3461	4.59047	3.63837	5.05963	7.00487	4.41949	5.75982
XLOC_062629	CDH13	chr18	9957758	10030117	0.0	5.5883	0.0	3.92025	3.29596	7.57445	4.67964	3.54187	8.60307
XLOC_062630	CDH13	chr18	9957758	10030117	3.09709	2.77299	4.83411	1.10803	2.61721	1.58519	4.04676	2.71438	4.02608
XLOC_062631	CDH13	chr18	10063093	10063395	4.9852	0.0	0.0	0.891997	2.26892	2.52852	0.843191	0.476017	1.28953
XLOC_052529	CDH22	chr13	75644862	75645228	662.27	787.29	684.414	2883.69	2258.58	2525.27	2351.43	2669.53	2874.48
XLOC_062405	CDH3	chr18	36018547	36196854	9.34042	13.4554	7.28026	0.74798	0.78664	0.560405	0.923902	1.27379	0.644083
XLOC_062966	CDH3	chr18	36018547	36196854	9.21267	10.0816	2.39662	0.0	0.0	0.0	0.535235	0.0	0.0
XLOC_062967	CDH3	chr18	36018547	36196854	0.0	4.52314	11.8283	0.0	0.1948	0.0	0.222131	0.247496	0.0
XLOC_062182	CDH5	chr18	34220691	34320012	47.6638	48.1597	49.8782	31.2516	27.3348	28.0223	28.4784	32.7205	28.2463
XLOC_062825	CDH8	chr18	29639926	29640529	1.62357	5.33732	3.80683	0.0	0.0	0.0	0.0	0.0	0.0
XLOC_062826	CDH8	chr18	29648657	29649266	6.40795	6.22404	2.50421	0.0	0.0	0.0	0.0	0.0	0.0
XLOC_062827	CDH8	chr18	29652787	29653453	1.42239	5.52725	4.44778	0.0	0.0	0.0	0.0	0.0	0.0
XLOC_062828	CDH8	chr18	29663836	29664381	3.73323	5.57714	1.45853	0.0	0.0	0.0	0.0	0.0	0.0
XLOC_062829	CDH8	chr18	29675643	29676982	1.83641	2.74683	0.957886	0.0	0.0	0.0	0.0	0.0	0.0
XLOC_062830	CDH8	chr18	29678982	29679941	1.80476	2.96859	2.82326	0.0	0.0	0.0	0.0	0.0	0.0
XLOC_062831	CDH8	chr18	29700219	29700637	0.0	8.28867	0.0	0.0	0.0	0.0	0.0	0.0	0.0
XLOC_062832	CDH8	chr18	29728242	29728988	2.45803	5.5116	2.88291	0.0	0.0	0.0	0.0	0.0	0.0
XLOC_062833	CDH8	chr18	29730225	29730821	3.29898	5.42239	1.28917	0.0	0.0	0.0	0.0	0.0	0.0
XLOC_064253	CDK12	chr19	40550111	40588666	14.8825	14.9006	12.7761	19.085	27.116	21.8435	34.2346	22.2666	22.8702
XLOC_073075	CDK14	chr4	8144853	8238876	4.55249	4.29666	0.892785	0.408181	0.355094	0.354552	0.51056	0.225221	0.199264
XLOC_073654	CDK14	chr4	8119164	8119334	0.0	0.0	0.0	5.22947	1.99773	0.0	23.0896	17.0638	4.77045
XLOC_089835	CDK19	chr9	40058715	40152221	0.0	0.0	0.0	3.82337	4.09441	2.93874	3.76206	3.04287	5.08355
XLOC_090598	CDK19	chr9	40050773	40051860	0.0	0.0	0.0	0.0	0.73056	0.242345	0.353379	0.233739	0.407954
XLOC_090599	CDK19	chr9	40052699	40054352	0.0	0.0	0.0	0.173449	0.265495	0.201197	0.220837	0.0972005	0.803773
XLOC_090600	CDK19	chr9	40058715	40152221	0.0	0.0	0.0	0.183931	0.803269	0.426269	0.186693	0.47993	0.478262
XLOC_090601	CDK19	chr9	40058715	40152221	0.0	0.0	0.0	1.28774	0.916265	0.135218	0.235088	0.130002	0.569829
XLOC_090602	CDK19	chr9	40058715	40152221	0.0	0.0	0.0	0.162971	0.426364	0.094313	0.906203	0.45446	0.635268
XLOC_086568	CDK20;FBXW12	chr8	91313415	91667342	14.0554	18.9785	13.1068	15.7309	17.7665	16.2318	18.3108	15.002	13.3233
XLOC_088567	CDK20;FBXW12	chr8	91313415	91667342	0.0	2.17261	2.84051	1.62807	1.11453	1.48661	0.314309	1.05735	0.945103

XLOC_088568	CDK20;FBXW12	chr8	91313415	91667342	1.97899	2.36489	3.0923	1.063	1.07348	0.611789	0.879178	0.585828	0.688688
XLOC_064678	CDK5R1;MYO1D	chr19	17926676	17926928	352.047	402.112	297.851	1164.54	897.182	1024.5	994.077	1005.17	965.72
XLOC_050908	CDK5RAP1;BPIFB5	chr13	63310149	63403132	6.31973	6.76851	6.04667	8.93772	8.45587	9.26176	7.06624	7.70398	8.57367
XLOC_052193	CDK5RAP1;BPIFB5	chr13	63310149	63403132	4.94576	6.64589	5.79318	3.983	3.81765	3.55683	3.48443	5.09396	4.29526
XLOC_087090	CDK5RAP2	chr8	111710502	111743859	94.3365	82.1193	87.1002	23.0229	18.839	21.6	19.8105	25.8446	11.6434
XLOC_087091	CDK5RAP2	chr8	111768470	111854527	112.774	129.823	97.9577	269.659	273.246	281.297	320.813	289.041	314.339
XLOC_087092	CDK5RAP2	chr8	111865777	111876777	6.46081	7.73019	8.44002	18.063	16.3611	18.0484	14.4265	15.5168	14.7724
XLOC_088872	CDK5RAP2	chr8	111710502	111743859	4.5453	3.39759	4.44291	0.305686	0.62068	0.706259	0.0	0.22661	0.396657
XLOC_088873	CDK5RAP2	chr8	111763922	111764480	5.41805	0.53964	1.41127	1.77882	1.2613	0.745219	1.28778	0.892912	0.62889
XLOC_088874	CDK5RAP2	chr8	111765292	111766081	0.0	0.0	0.0	0.410494	1.16151	0.830438	0.0	0.570838	0.499731
XLOC_088875	CDK5RAP2	chr8	111766134	111767988	0.426307	0.510215	0.0	1.49085	1.63903	1.1976	1.01338	0.986062	0.522191
XLOC_088876	CDK5RAP2	chr8	111863116	111863898	3.47449	4.84793	5.43382	12.4527	11.5634	14.0347	8.8791	10.2732	10.3083
XLOC_088877	CDK5RAP2	chr8	111864064	111864380	4.55163	5.42858	3.54859	12.6194	11.7698	18.0393	12.0075	8.29192	5.1068
XLOC_088878	CDK5RAP2	chr8	111864476	111865613	8.86062	8.17188	8.66465	16.4811	15.8924	16.7922	13.8933	13.8327	16.5844
XLOC_088879	CDK5RAP2	chr8	111889137	111890239	0.765748	0.687121	0.0	0.960961	0.719028	0.556533	0.417415	0.996935	0.401492
XLOC_088880	CDK5RAP2	chr8	111890446	111891018	0.0	1.04288	0.0	0.937552	0.406428	0.540238	0.467122	1.89946	0.30389
XLOC_072529	CDK6	chr4	9844365	9862548	0.201819	0.0603987	0.0	0.0181013	0.0158642	0.0	0.0	0.0	0.0
XLOC_073686	CDK6	chr4	9820126	9820489	3.51781	12.5946	5.48885	0.0	0.269418	0.0	0.304267	0.0	0.0
XLOC_080227	CDKL2	chr6	92169077	92184369	8.3134	8.3694	6.84603	13.3259	12.536	12.4827	13.1275	15.1846	13.2782
XLOC_081694	CDKL2	chr6	92186673	92187458	3.45787	5.1694	7.21049	2.89175	3.9561	5.13355	3.32708	4.36564	4.72729
XLOC_080228	CDKL2;G3BP2	chr6	92187624	92214883	7.61589	11.69	11.9352	8.72793	10.318	7.8733	10.4815	10.7503	9.28715
XLOC_044682	CDKL4	chr11	21557185	21572001	55.7755	56.5879	47.9853	112.49	101.781	105.584	116.696	119.788	152.723
XLOC_086802	CDKN2B	chr8	22107704	22125041	0.0	0.0	1.45704	0.333909	1.31016	0.676253	0.253088	0.279272	1.6269
XLOC_064235	CDRT4;ZNF286A;FAM18B2;ZNF287	chr19	33514346	33785700	4.90385	2.9419	4.66137	5.70076	4.90601	4.97641	6.02194	4.82909	5.3485
XLOC_065021	CDRT4;ZNF286A;FAM18B2;ZNF287	chr19	33514346	33785700	0.0	0.939654	0.0	1.59573	1.0631	0.759937	1.23068	0.83632	1.55445
XLOC_051774	CDS2	chr13	47855563	47856807	0.0	0.0	0.0	0.954799	0.364907	0.899033	2.06002	1.33493	0.989242
XLOC_062353	CDYL2;DYNLRB2	chr18	7350389	7626860	6.99048	4.32627	5.92109	10.7425	9.19322	11.8606	11.693	9.95857	9.15494

XLOC_053496	CEBPD	chr14	20734871	20794080	9.88549	8.39051	11.4984	8.33372	12.4121	9.69136	11.4486	8.63346	12.5966
XLOC_053937	CEBPD	chr14	20734871	20794080	0.0	3.54232	0.0	1.41591	1.8136	1.21033	1.35983	0.381859	0.342559
XLOC_055856	CELF1	chr15	78479329	78511595	0.398501	0.0596127	0.0	0.0535953	0.0615714	0.0207531	0.0365704	0.0200756	0.117944
XLOC_055857	CELF1	chr15	78523233	78529631	1.04739	1.56667	1.19797	0.74125	1.25025	1.03756	0.841971	0.462486	0.671811
XLOC_057038	CELF1	chr15	78554196	78554942	1.22902	0.36744	0.0	1.76181	1.53299	1.52684	0.774944	1.10146	1.71547
XLOC_051182	CELF2	chr13	13163085	13163805	0.0	0.384404	0.0	0.0	0.0	0.0	0.0	0.0	0.0
XLOC_057948	CENPF	chr16	70313571	70569713	6.47959	8.66251	8.83608	5.15937	6.48727	6.41661	5.94954	3.11815	7.78612
XLOC_086993	CENPP	chr8	85408706	85432893	7.0377	6.5976	12.1157	12.1937	15.9714	14.9287	16.001	11.9314	15.6539
XLOC_088480	CENPP	chr8	85408706	85432893	10.3203	1.5383	0.0	5.54082	4.69239	6.27725	1.30602	3.93551	3.11344
XLOC_088481	CENPP	chr8	85528762	85529320	0.0	0.53964	1.41127	0.0	1.82188	0.558914	2.09264	0.357165	0.943335
XLOC_088482	CENPP	chr8	85531297	85531518	0.0	0.0	0.0	4.8324	3.95544	8.55289	8.54217	1.96295	10.071
XLOC_088483	CENPP	chr8	85531839	85532810	3.55627	0.797682	1.39083	1.67336	2.22399	2.67489	2.8206	1.06683	2.09673
XLOC_088484	CENPP	chr8	85532905	85533375	2.31464	0.69128	1.80778	1.03574	3.04111	1.19014	1.63534	0.454951	2.21245
XLOC_088485	CENPP	chr8	85534472	85534724	0.0	2.23395	0.0	3.36572	7.86017	6.03533	5.53632	2.10433	3.21907
XLOC_088486	CENPP	chr8	85535480	85535859	0.0	2.92248	7.64199	2.33563	6.01147	6.34343	6.52181	1.58649	2.26209
XLOC_088487	CENPP	chr8	85536045	85536370	0.0	0.0	0.0	6.16663	4.26781	3.06917	4.41721	2.48541	5.21062
XLOC_088488	CENPP	chr8	85536941	85537195	0.0	0.0	5.72713	1.97977	4.40732	2.22022	1.20824	0.688555	6.94594
XLOC_089318	CENPW	chr9	25252897	25308686	110.751	112.22	92.0804	76.4135	76.4971	76.1731	96.0044	90.8973	103.708
XLOC_090443	CENPW	chr9	25252897	25308686	3.19263	0.0	14.9483	0.571076	0.490192	1.30657	0.0	0.621129	0.276607
XLOC_090444	CENPW	chr9	25252897	25308686	5.58222	2.92213	3.2754	1.12593	1.20187	1.15965	1.52338	1.2589	0.365947
XLOC_090445	CENPW	chr9	25252897	25308686	0.0	1.77347	0.0	1.06586	0.448955	1.80448	1.49046	1.69008	0.0
XLOC_090446	CENPW	chr9	25252897	25308686	0.0	0.563526	0.0	0.337737	0.292533	0.194473	0.0	0.186322	0.0
XLOC_090447	CENPW	chr9	25252897	25308686	3.73324	4.46171	10.2097	1.16989	0.868663	2.30981	2.16047	0.922197	0.974778
XLOC_090448	CENPW	chr9	25252897	25308686	3.33704	5.48485	2.60803	2.24132	1.55541	1.55041	1.19272	0.826313	0.581305
XLOC_090449	CENPW	chr9	25252897	25308686	3.58737	3.21074	5.59706	1.28317	0.823831	0.732505	0.619858	0.694865	0.931256
XLOC_090450	CENPW	chr9	25252897	25308686	0.0	2.05143	4.47098	0.614771	1.2489	1.53982	1.03167	0.56995	0.498947
XLOC_080166	CEP135	chr6	73025427	73035433	0.0	0.0	0.729458	0.334339	0.14576	0.290194	0.168939	0.0	0.407247
XLOC_080167	CEP135	chr6	73037629	73103558	0.0	0.0	0.0	2.66911	2.80952	2.14123	2.11787	1.42088	2.49958
XLOC_081403	CEP135	chr6	73037629	73103558	0.0	0.0	0.0	0.736838	1.91237	0.21195	1.27806	0.811445	1.07388
XLOC_057883	CEP170	chr16	34690568	34725333	2.63072	2.71265	2.69609	12.1144	14.3423	12.1624	13.3279	12.2823	14.7097
XLOC_070248	CEPT1	chr3	32340569	32366604	1.65924	0.840658	0.942575	16.8086	14.2442	16.162	12.7536	13.0542	10.3545
XLOC_070768	CEPT1	chr3	32338752	32340384	0.980988	0.586999	1.53527	8.66396	7.38546	8.41723	6.67382	7.59039	5.44897
XLOC_070769	CEPT1	chr3	32340569	32366604	0.0	0.997244	1.30401	2.98842	1.94427	2.92855	3.42906	1.81788	2.32523

XLOC_070770	CEPT1	chr3	32366851	32367397	0.0	0.556281	0.0	2.16706	0.722046	0.575982	2.1551	2.57565	0.48614
XLOC_065539	CERS6	chr2	27442532	27445426	1.92969	1.81488	1.72611	0.494463	0.714662	0.45935	0.909893	0.722147	0.989445
XLOC_065540	CERS6	chr2	27515359	27536616	42.2225	53.2812	51.0825	92.586	86.3342	83.5844	115.875	127.458	119.256
XLOC_065541	CERS6	chr2	27677392	27763134	11.8532	16.5567	16.0298	2.28055	2.11441	2.08261	1.76948	2.68792	1.69983
XLOC_066209	CERS6	chr2	27445480	27475699	23.8156	31.681	32.3332	20.333	23.863	22.6903	23.4603	30.5686	17.5236
XLOC_066210	CERS6	chr2	27513752	27515050	0.811797	0.24238	1.27031	1.23758	0.444303	0.842665	1.0318	0.649948	0.922144
XLOC_067140	CERS6	chr2	27426678	27427044	13.8695	18.6218	13.526	8.68209	6.64131	7.43873	3.90204	8.40637	5.70274
XLOC_067141	CERS6	chr2	27445480	27475699	0.0	3.99118	1.15986	1.06315	1.15428	1.22687	2.26137	1.62014	0.387959
XLOC_067142	CERS6	chr2	27445480	27475699	1.56941	1.87813	0.409377	1.40714	0.943345	0.870358	1.05033	0.735661	0.686334
XLOC_067143	CERS6	chr2	27445480	27475699	0.0	6.46419	0.0	2.32625	0.330189	0.882012	2.22086	1.24977	0.748808
XLOC_067144	CERS6	chr2	27445480	27475699	0.0	3.30751	4.94293	1.98232	1.35193	0.979905	1.13195	1.09731	1.23972
XLOC_067145	CERS6	chr2	27445480	27475699	1.86804	2.23528	4.3847	1.3956	0.926823	1.22945	1.3046	0.8121	1.08884
XLOC_067146	CERS6	chr2	27539776	27540102	0.0	3.83306	3.34087	0.766295	1.63209	3.0515	1.46432	1.23572	0.0
XLOC_066208	CERS6;NOSTRIN;G6PC2;SPC25	chr2	27261615	27426567	8.67825	19.5502	17.4617	11.7245	9.53245	11.2084	11.4471	9.36234	9.22192
XLOC_062187	CES2	chr18	34759083	34771241	10.5338	18.6737	12.5722	10.5871	8.75208	9.56497	10.0353	12.5327	6.111
XLOC_062188	CES4A;CBFB	chr18	34776920	34839118	18.9381	19.8885	19.0368	28.6313	25.466	26.3896	31.2303	37.7005	23.8327
XLOC_062883	CES4A;CBFB	chr18	34776920	34839118	4.6678	2.78343	14.5558	6.67994	3.54751	4.26704	7.53438	4.91754	5.23573
XLOC_062884	CES4A;CBFB	chr18	34776920	34839118	7.3564	2.19455	2.8692	2.30237	7.31589	4.50415	7.29793	6.76164	3.50009
XLOC_083617	CFD	chr7	45027170	45043573	2.15812	4.36913	3.53203	2.66645	3.04491	2.90297	2.70201	4.44024	1.3285
XLOC_084824	CFD	chr7	45027170	45043573	2.66648	0.797117	1.04238	0.716626	0.519249	0.551754	1.67811	0.397826	0.69759
XLOC_084825	CFD	chr7	45027170	45043573	2.16385	0.970533	1.69221	0.775596	0.76	0.896895	0.0977304	0.755558	0.283307
XLOC_084826	CFD	chr7	45027170	45043573	0.0	1.2617	1.64981	0.756161	0.163481	0.434902	0.0	0.832293	0.550941
XLOC_068079	CFLAR	chr2	90148419	90148828	0.0	0.0	0.0	0.0	0.221292	0.29475	0.0	0.280757	0.0
XLOC_089954	CGA	chr9	63645523	63758080	28.6933	22.669	24.3681	5.67754	5.84311	6.13161	6.52007	6.73483	7.2873
XLOC_041977	CGRRF1	chr10	67266206	67285326	8.03339	6.09448	6.80748	5.86184	5.34108	7.09847	6.44003	5.54607	6.48674
XLOC_042875	CGRRF1	chr10	67258170	67258838	0.0	0.42351	1.1076	1.39606	2.53658	2.05113	1.14465	1.5484	1.23516
XLOC_042876	CGRRF1	chr10	67259207	67259602	3.04502	0.0	2.37653	2.17886	0.936077	4.05339	1.32743	1.78022	1.05575
XLOC_042877	CGRRF1	chr10	67266206	67285326	1.37376	1.23197	1.07398	1.35369	1.71154	0.426282	1.11074	1.50213	1.67693
XLOC_042878	CGRRF1	chr10	67266206	67285326	3.04224	0.90928	0.0	0.272485	0.473224	0.0	0.545147	0.150935	0.530234
XLOC_038093	CHAF1B	chr1	150361276	150439212	73.1333	88.1077	74.7806	81.0221	79.3133	89.1723	110.425	109.567	81.704
XLOC_038579	CHAF1B	chr1	150361276	150439212	11.0508	11.268	10.8835	10.942	16.5525	14.1765	11.8181	11.0874	15.556

XLOC_040574	CHAF1B	chr1	150361276	150439212	1.83349	0.0	2.86539	3.11916	3.98285	1.51285	1.96007	1.99362	2.71346
XLOC_040575	CHAF1B	chr1	150361276	150439212	2.85307	1.70335	2.22712	0.510443	0.439172	0.292466	0.0	1.11447	0.247906
XLOC_040576	CHAF1B	chr1	150361276	150439212	1.50529	2.69949	1.17665	0.943786	0.936718	0.311144	0.944346	0.448175	0.26261
XLOC_048253	CHAMP1;CDC16;MIR2303	chr12	90998474	91384408	1.16579	2.56201	2.92485	5.38417	5.46886	5.80184	6.54579	5.59239	5.15377
XLOC_050013	CHAMP1;CDC16;MIR2303	chr12	90998474	91384408	6.24759	6.52605	4.87598	11.1756	11.7567	11.1903	16.3192	11.556	13.5348
XLOC_073249	CHCHD3	chr4	97520818	97522549	24.6186	21.1946	14.5464	60.4098	69.5759	70.6943	86.5101	77.94	80.3344
XLOC_074627	CHCHD3	chr4	97523451	97523618	0.0	9.10454	47.6204	11.4113	25.9802	6.00448	18.1713	13.1928	7.77826
XLOC_045894	CHCHD5	chr11	46153101	46153448	3.81331	1.13748	0.0	3.06888	0.291441	3.88866	1.31295	2.57888	0.989247
XLOC_083135	CHD1	chr7	100479586	100499570	45.4117	42.602	33.2024	60.7269	59.5006	61.776	74.9429	84.0659	59.0247
XLOC_083136	CHD1	chr7	100536932	100539879	1.00523	3.006	3.14471	6.03398	5.25811	5.16978	3.64264	6.77854	4.2064
XLOC_083137	CHD1	chr7	100549117	100642174	40.1942	44.1489	47.334	26.5234	25.0736	25.6268	31.2266	28.5255	27.8885
XLOC_083781	CHD1	chr7	100502367	100514806	0.416006	0.91471	0.651113	3.68836	3.1178	2.7119	3.58038	3.57887	6.18222
XLOC_083782	CHD1	chr7	100549117	100642174	15.7889	17.8318	19.4522	26.5931	22.4835	25.5973	22.8615	24.7389	29.1248
XLOC_085463	CHD1	chr7	100479586	100499570	0.0	1.44731	0.0	6.51225	5.15811	5.911	6.99052	6.03194	7.53455
XLOC_085464	CHD1	chr7	100499621	100500163	1.88118	4.49646	0.0	3.87385	7.29446	3.29746	4.68839	8.17719	7.53117
XLOC_085465	CHD1	chr7	100500325	100501388	1.59746	1.19449	3.74888	3.86608	4.18638	2.98484	4.56885	4.23778	5.58314
XLOC_085466	CHD1	chr7	100547855	100548382	0.0	0.584835	0.0	0.0	0.7586	0.201748	0.521998	0.579636	0.681285
XLOC_085467	CHD1	chr7	100549117	100642174	1.63458	0.488496	0.0	0.0	0.0	0.675193	0.58459	0.485902	0.0
XLOC_085468	CHD1	chr7	100549117	100642174	4.79816	3.58221	1.87374	2.57625	1.85312	2.71245	2.11555	3.53305	1.66726
XLOC_050949	CHD6	chr13	70797838	70836374	5.7357	4.11586	2.69108	0.0	0.0	0.0	0.0	0.0	0.0
XLOC_062372	CHD9	chr18	21558054	21652416	1.10629	0.165484	0.0	0.0495938	0.0	0.230028	0.0	0.0	0.0
XLOC_062784	CHD9	chr18	21652505	21653516	5.08439	1.26721	1.98855	0.0	0.0	0.0879373	0.0	0.0	0.0
XLOC_062785	CHD9	chr18	21655038	21656218	1.41466	0.423159	1.10674	0.0	0.0	0.0	0.0	0.0	0.0
XLOC_059804	CHFR	chr17	45391681	45435151	1.07848	0.0	0.0	0.426866	0.0	0.553435	0.766218	0.0	0.0
XLOC_060190	CHFR	chr17	45391681	45435151	77.4405	92.707	113.787	183.088	162.313	165.737	266.082	272.332	222.941
XLOC_061453	CHFR	chr17	45383611	45384232	0.0	0.932788	0.0	1.53741	1.5773	2.90202	1.39714	1.39291	1.49568
XLOC_061454	CHFR	chr17	45387573	45389176	0.500335	0.449078	0.0	2.06362	2.27561	2.60169	2.23837	2.16165	2.31952
XLOC_061455	CHFR	chr17	45390457	45390989	5.79426	1.73112	3.01813	5.18752	4.04258	5.17625	4.63745	5.91146	3.36126
XLOC_061456	CHFR	chr17	45391681	45435151	0.0	1.39641	1.82608	1.25544	2.49608	2.1811	3.81649	1.63618	1.56383
XLOC_061457	CHFR	chr17	45391681	45435151	0.455579	2.18093	2.13905	1.87912	2.82329	1.61135	3.03895	2.93117	2.35152

XLOC_061458	CHFR	chr17	45391681	45435151	7.07985	2.11435	1.84308	2.53432	3.4642	0.970485	6.45575	3.94065	5.33098
XLOC_061459	CHFR	chr17	45391681	45435151	0.933504	1.6758	2.92198	1.79962	2.08683	2.23337	2.81434	2.0174	3.02157
XLOC_061460	CHFR	chr17	45391681	45435151	4.16302	1.86537	1.62608	2.04958	2.73986	3.42974	6.83026	4.71817	5.24974
XLOC_061461	CHFR	chr17	45391681	45435151	1.72011	3.5981	2.68852	2.31049	2.6714	1.59783	4.76021	2.38371	3.89458
XLOC_061462	CHFR	chr17	45391681	45435151	0.0	2.1618	2.82637	0.971972	0.831848	1.47934	4.06685	2.45532	4.38877
XLOC_080148	CHIC2	chr6	71213943	71262281	0.0	0.295951	0.0	0.443882	0.162164	0.199137	0.146991	0.461379	0.0874028
XLOC_092173	CHM	chrX	74177206	74208509	19.4768	35.9171	25.9719	1.18739	0.707666	0.570591	0.96845	0.84792	1.25057
XLOC_092174	CHM	chrX	74254539	74258091	15.7707	18.1492	16.1394	27.0871	31.6075	28.7404	29.7369	30.7525	27.3514
XLOC_092175	CHM	chrX	74269735	74271949	7.42392	15.8542	9.98573	43.3043	42.9512	46.0805	50.2488	56.3427	46.9714
XLOC_092176	CHM	chrX	74273215	74290336	7.91909	9.62285	4.34141	11.4634	12.9885	13.067	12.8472	9.10256	17.7649
XLOC_092177	CHM	chrX	74296403	74304756	33.9094	31.686	30.1748	20.9719	23.8731	23.7642	22.3396	19.4253	26.8985
XLOC_092178	CHM	chrX	74305319	74311177	28.9286	20.6247	19.6109	36.4263	45.5495	37.719	43.1278	33.8121	49.461
XLOC_092179	CHM	chrX	74313172	74320198	69.73	28.0962	31.9306	77.6257	70.3858	100.898	83.3954	101.654	72.686
XLOC_092180	CHM	chrX	74329079	74344617	3.45214	2.88024	4.47452	2.55911	2.46487	3.33763	2.54341	2.03088	2.69924
XLOC_092181	CHM	chrX	74384248	74449952	4.10994	4.75212	6.43373	7.04246	7.03044	6.11169	7.05955	5.69689	8.91172
XLOC_092490	CHM	chrX	74223282	74253619	102.557	98.0908	107.28	81.7787	72.7388	73.3001	89.044	109.57	69.0226
XLOC_092491	CHM	chrX	74273215	74290336	1.3389	0.667668	0.0	1.60076	1.96071	1.34647	1.67234	1.30129	2.14754
XLOC_092492	CHM	chrX	74352168	74353630	13.5566	11.3062	15.9375	67.2079	56.4281	74.8027	60.8275	72.7058	74.275
XLOC_092493	CHM	chrX	74366030	74368516	15.5447	13.0283	7.48622	9.36436	9.69718	9.20814	11.209	8.60159	10.0795
XLOC_092494	CHM	chrX	74368573	74373574	7.2428	4.56407	6.78819	1.73856	1.22997	1.65685	2.30684	2.43978	2.11174
XLOC_092495	CHM	chrX	74384248	74449952	0.948885	3.55167	1.23774	2.68903	3.1153	2.19112	2.50011	2.08023	2.70659
XLOC_092496	CHM	chrX	74384248	74449952	21.5067	23.9822	25.5519	32.8692	30.351	32.9052	29.2517	30.1935	29.9013
XLOC_093142	CHM	chrX	74170632	74170900	0.0	0.0	5.0387	1.15927	0.0	1.30528	1.07321	1.21944	0.0
XLOC_093143	CHM	chrX	74217182	74217571	9.37138	11.1873	7.31352	1.11756	0.479864	0.319724	0.0	0.0	0.0
XLOC_093144	CHM	chrX	74218132	74218622	4.35085	7.14764	6.79711	0.194714	0.50494	0.0	0.192603	0.21415	0.378255
XLOC_093145	CHM	chrX	74219500	74220496	6.90046	4.12756	8.77106	0.69579	1.07907	0.537012	0.625792	0.690238	0.753485
XLOC_093146	CHM	chrX	74221156	74221516	3.56971	2.12999	2.7848	1.27686	0.273291	0.364481	1.54246	0.691576	0.0
XLOC_093147	CHM	chrX	74221727	74223008	2.57496	3.27369	2.51826	0.69253	0.756324	0.468215	0.351704	0.258262	0.506509
XLOC_093148	CHM	chrX	74260867	74261540	0.0	0.0	0.0	0.377055	0.546142	0.870601	1.3858	0.975948	0.61162
XLOC_093149	CHM	chrX	74305009	74305244	0.0	0.0	0.0	4.04696	7.35887	3.60552	4.36438	7.49553	6.16497
XLOC_093150	CHM	chrX	74311293	74312022	20.2493	18.5395	13.8533	24.491	31.2667	27.7704	28.9364	24.3121	31.6825
XLOC_093151	CHM	chrX	74329079	74344617	0.0	0.0	0.0	1.1256	1.94136	1.29205	0.221393	0.246661	0.218464
XLOC_093152	CHM	chrX	74384248	74449952	1.94745	1.89365	0.380976	1.65885	1.56547	2.22803	1.6451	1.95707	1.74611

XLOC_050601	CHMP4B	chr13	63815195	63900363	12.0222	10.8318	8.51569	12.5025	13.6287	15.0079	16.1118	14.4678	15.2372
XLOC_086925	CHMP7	chr8	71251206	71272555	29.0453	17.6793	13.8309	34.6652	43.3837	37.8152	38.2247	30.8394	40.9157
XLOC_066986	CHN1	chr2	22010353	22010957	0.0	0.0	0.0	0.290155	0.503566	0.167301	0.43463	0.481634	1.97566
XLOC_066987	CHN1;CHN1	chr2	22067864	22068110	0.0	0.0	0.0	1.43198	0.595438	0.800794	1.3014	0.743036	0.683807
XLOC_066988	CHN1;CHN1	chr2	22068531	22068919	0.0	1.87261	2.44836	3.08658	4.57799	3.21084	3.27721	3.96988	1.63114
XLOC_074946	CHPF2	chr4	114610943	114611896	1.81837	0.0	0.0	0.0	0.0710607	0.0	0.164763	0.0	0.0
XLOC_074947	CHPF2	chr4	114612745	114614537	1.32743	0.397171	0.346262	0.0	0.0	0.0	0.0	0.0	0.0
XLOC_072722	CHRM2	chr4	101496671	101642793	0.29673	0.222019	0.0	5.98799	5.23859	4.87242	6.94988	5.42098	6.90907
XLOC_088200	CHRNA2	chr8	75871904	75872059	87.3376	13.4244	35.1189	29.8828	31.4018	8.80731	32.7573	15.2262	30.4995
XLOC_050564	CHRNA4	chr13	54701433	54737727	0.142829	0.0854944	0.111806	0.0768673	0.0561629	0.119097	0.118151	0.0288285	0.0
XLOC_064834	CHRNB1	chr19	27761701	27762884	0.0	0.632883	0.55175	0.948332	1.38032	0.366261	0.833897	0.777506	1.23287
XLOC_055512	CHST1	chr15	76576367	76607618	3.65953	4.14459	3.6349	5.13386	4.2731	4.73763	4.7587	4.80903	4.30365
XLOC_055834	CHST1	chr15	76576367	76607618	19.8602	23.4256	23.8313	31.2515	26.7232	28.0228	29.1956	33.6821	25.0653
XLOC_056989	CHST1	chr15	76576367	76607618	0.939916	0.281344	0.0	0.673889	0.514294	0.585047	0.0851175	0.563573	0.738804
XLOC_082841	CHSY3	chr7	25221433	25226639	1.96928	3.61889	4.92342	2.20105	1.79207	1.77047	1.87877	3.13878	1.2678
XLOC_082842	CHSY3	chr7	25267731	25294786	30.6919	37.2631	33.4177	18.9573	15.8778	17.6865	15.7359	24.8717	11.7902
XLOC_082843	CHSY3	chr7	25301298	25304110	3.70102	10.8871	9.16973	4.25814	4.83248	4.23081	2.47261	4.70346	2.75053
XLOC_082844	CHSY3	chr7	25306237	25308834	0.0	0.720207	0.0	1.6004	1.61944	0.748274	0.330546	0.18201	0.538244
XLOC_082845	CHSY3	chr7	25311364	25316120	0.0	0.0	0.0	0.751562	1.31437	0.92609	0.430299	0.36836	0.320749
XLOC_082846	CHSY3	chr7	25316793	25364714	5.76798	7.65362	5.6053	7.05058	8.57583	6.85298	5.70183	5.32037	5.217
XLOC_082847	CHSY3	chr7	25396294	25419833	3.43884	2.31507	2.01833	5.39627	4.11469	4.9196	5.77677	6.7002	6.39357
XLOC_083462	CHSY3	chr7	25202628	25220637	10.6324	9.4181	11.6518	14.0147	7.04375	10.5769	8.12789	13.1754	6.69411
XLOC_083463	CHSY3	chr7	25227867	25237418	36.1552	24.5987	31.6244	36.7962	31.9254	32.9906	36.5767	40.5135	28.3473
XLOC_083464	CHSY3	chr7	25242910	25250621	49.6661	60.2929	54.9933	74.835	57.2521	66.6897	71.8837	120.551	56.4027
XLOC_083465	CHSY3	chr7	25295019	25297986	35.1212	53.4764	31.6082	44.9832	47.7541	46.4733	45.712	36.397	45.9553
XLOC_083466	CHSY3	chr7	25379772	25395771	4.98468	6.19804	5.43895	3.09159	2.91103	3.59002	2.85459	4.41055	2.76047
XLOC_083467	CHSY3	chr7	25463671	25539859	2.0358	1.0724	0.502755	3.7253	1.9555	4.35756	4.03172	1.89664	3.55866
XLOC_084376	CHSY3	chr7	25200070	25200244	24.8656	15.0668	19.6995	0.0	10.8379	0.0	7.60722	6.62304	6.44611
XLOC_084377	CHSY3	chr7	25200374	25201524	6.55841	3.7055	6.27091	3.33146	2.3956	3.40546	1.25875	1.97131	2.22907
XLOC_084378	CHSY3	chr7	25201640	25202291	1.46563	3.94271	5.72848	0.91896	1.14041	1.96964	1.70882	3.78368	1.14974
XLOC_084379	CHSY3	chr7	25226711	25227762	1.61892	3.14736	1.89959	2.46687	2.02615	1.59638	2.05746	3.07881	1.62665
XLOC_084380	CHSY3	chr7	25238026	25239021	0.0	1.03312	0.675502	0.309609	0.0675216	0.179217	0.234948	0.0863817	0.150876
XLOC_084381	CHSY3	chr7	25241194	25241724	0.0	0.0	0.0	0.521532	0.602068	0.800571	0.690589	0.0	0.0

XLOC_084382	CHSY3	chr7	25242910	25250621	2.934	7.00619	4.58057	4.46158	2.25676	3.30684	3.84447	3.14877	2.03527
XLOC_084383	CHSY3	chr7	25242910	25250621	10.9326	4.88852	4.26108	4.40381	2.4812	4.9818	4.59284	5.71692	3.29637
XLOC_084384	CHSY3	chr7	25266913	25267400	0.0	0.0	1.71472	2.16131	1.69819	1.12956	1.36001	0.216049	1.14502
XLOC_084385	CHSY3	chr7	25267731	25294786	0.878456	0.788003	2.7479	1.02333	0.549292	1.09348	0.238877	0.790511	0.306869
XLOC_084386	CHSY3	chr7	25316793	25364714	5.44524	9.2199	8.51003	10.0765	12.5345	7.48917	6.30839	7.89621	7.90032
XLOC_084387	CHSY3	chr7	25365032	25366255	3.39392	5.48225	2.65525	5.05057	4.78362	4.23082	4.63261	4.015	4.21277
XLOC_084388	CHSY3	chr7	25420132	25420501	0.0	0.0	0.0	1.22279	2.6199	1.39724	0.592312	0.331682	0.887839
XLOC_084389	CHSY3	chr7	25420736	25421280	0.0	0.0	4.38689	0.670233	0.435436	2.12274	0.333211	0.739621	0.488639
XLOC_084390	CHSY3	chr7	25421593	25422890	0.634701	0.189868	0.993171	1.19492	1.04406	0.791428	0.520236	1.01865	0.776864
XLOC_084391	CHSY3	chr7	25433817	25434542	0.0	0.0	1.99297	1.02764	1.58914	0.791451	0.688244	0.507375	0.555832
XLOC_084392	CHSY3	chr7	25435136	25435636	2.1119	0.0	0.0	0.18904	1.63481	0.217448	0.0	0.416113	0.0
XLOC_084393	CHSY3	chr7	25436010	25436975	0.895674	0.0	0.0	0.401377	0.91016	0.371677	0.162355	0.268668	0.234696
XLOC_084394	CHSY3	chr7	25442573	25443142	3.51546	1.05048	0.0	1.41656	1.3645	1.08827	2.3521	1.56519	3.06092
XLOC_084395	CHSY3	chr7	25462383	25463607	0.0	0.202856	1.59166	1.763	1.32755	1.40896	0.863952	1.49573	1.95622
XLOC_042012	CHURC1;RAB15;GPX2	chr10	77292338	77418186	21.3686	15.3044	22.7452	3.40413	4.70663	3.86115	3.48334	2.89433	2.38706
XLOC_044222	CIB4	chr11	72916980	72927169	6.62961	7.43996	7.7837	3.18532	2.15835	2.88573	1.93345	2.64926	2.69496
XLOC_046538	CIB4	chr11	72916980	72927169	3.21109	1.91933	0.0	0.862747	0.873515	0.994981	0.574512	1.27317	0.279745
XLOC_046539	CIB4	chr11	72961187	72961660	0.0	0.684722	0.0	0.820728	1.41775	2.35797	1.01277	2.47909	1.19541
XLOC_044223	CIB4;C11H2orf70	chr11	72970846	72993038	3.04587	1.93036	1.10157	2.09711	3.19009	1.96971	2.34344	3.5482	3.91171
XLOC_082725	CILP2	chr7	3703719	3706898	290.758	237.513	240.265	1035.93	773.034	866.967	913.059	1082.45	821.905
XLOC_084040	CILP2	chr7	3707528	3707855	4.25953	2.54049	3.32141	7.61814	8.76327	11.7027	9.46613	11.8786	6.62197
XLOC_084041	CILP2	chr7	3707944	3708454	2.05198	0.612987	0.0	5.69433	7.31025	7.39747	7.82945	8.2943	4.10493
XLOC_084042	CILP2	chr7	3708633	3708877	0.0	0.0	6.33787	5.12002	7.89946	5.7223	12.6057	5.30575	5.58556
XLOC_084043	CILP2	chr7	3709025	3709169	0.0	63.5202	0.0	47.9389	43.7135	48.3169	65.6461	17.5841	29.9522
XLOC_084044	CILP2	chr7	3709802	3710179	0.0	0.982991	0.0	3.24064	2.02159	2.69475	2.8599	0.960221	1.71182
XLOC_080025	CISD2;UBE2D3;UBE2D3;MANBA	chr6	23262435	23390489	1.49952	1.2839	1.17362	0.0537813	0.0240593	0.0319036	0.0	0.0	0.078746
XLOC_083376	CIST1	chr7	4898915	4911888	0.375215	0.901705	1.1791	3.26655	5.81309	3.35849	2.98913	2.8833	3.4477
XLOC_084075	CIST1	chr7	4898915	4911888	0.0	0.0	0.0	0.849135	2.43002	1.29536	0.275357	1.84768	3.29051
XLOC_070099	CITED4	chr3	105942641	105975038	32.1251	35.2948	35.9226	55.0598	46.6051	49.7316	64.3722	69.2836	49.1194
XLOC_070467	CITED4	chr3	105942641	105975038	44.2149	41.8678	40.3131	100.617	93.0711	98.438	102.984	108.861	107.245

XLOC_072016	CITED4	chr3	105942641	105975038	11.1808	6.66589	17.4284	7.00725	8.03042	11.3174	7.50328	9.55709	8.66708
XLOC_072017	CITED4	chr3	105942641	105975038	5.60352	4.07025	10.6453	2.94188	3.31894	4.23787	5.52366	3.76626	4.19661
XLOC_072018	CITED4	chr3	105942641	105975038	4.01699	3.30554	3.66783	0.960644	1.76111	0.836396	1.65551	0.370729	1.08404
XLOC_048819	CKAP2	chr12	21601635	21602543	0.0	0.0	0.0	0.431833	0.752888	0.399738	0.2617	0.288833	0.504919
XLOC_048820	CKAP2	chr12	21624314	21624866	0.0	0.0	0.0	1.14917	0.0	0.378213	0.163339	0.906192	0.478794
XLOC_045897	CKAP2L	chr11	46325198	46326079	0.0	0.29894	0.781835	0.0	0.0	0.0	0.0	0.0	0.0
XLOC_063213	CKM	chr18	53390369	53391135	2.37724	1.77688	0.929422	2.12996	1.57577	2.09245	1.17862	3.31578	1.86674
XLOC_062240	CKM;MARK4;LOC508455	chr18	53245744	53390129	1.52986	1.37279	2.41212	4.45655	3.93699	4.60646	3.75284	5.79381	3.32723
XLOC_085233	CKMT2	chr7	83568006	83568628	1.55715	1.39617	0.0	1.11571	1.45288	0.482639	1.39422	0.154441	1.3568
XLOC_066346	CLASP1	chr2	73611821	73775100	22.8404	29.9687	21.2455	20.7158	18.0344	14.5722	20.648	25.1932	19.5813
XLOC_067911	CLASP1	chr2	73529374	73529861	0.0	1.31138	0.0	0.0	0.0	0.0	0.0	0.0	0.190836
XLOC_067912	CLASP1	chr2	73611292	73611584	0.0	3.18819	0.0	2.393	2.02402	0.541724	6.30006	3.56263	1.84306
XLOC_071309	CLCA1	chr3	57717761	57718077	40.9647	70.5716	85.166	119.681	124.622	124.425	131.308	132.671	106.457
XLOC_069913	CLCA1;ODF2L	chr3	57735897	57835231	13.9125	11.0072	10.663	5.96299	8.73173	6.50599	8.00046	6.26857	11.4409
XLOC_070297	CLCA3;LOC784768;SH3GLB1	chr3	57455458	57578754	68.2685	65.1242	64.2678	8.47136	10.2578	8.35555	12.6166	9.98102	14.9284
XLOC_070298	CLCA4	chr3	57646090	57651846	12.5968	11.5524	14.4861	16.0303	13.2932	14.8626	17.9348	19.9321	16.244
XLOC_071305	CLCA4	chr3	57644825	57645200	0.0	1.98396	0.0	0.0	0.509919	1.01963	0.865397	0.645798	1.15157
XLOC_071306	CLCA4	chr3	57645332	57645621	0.0	0.0	0.0	1.95725	1.2406	1.10707	1.83714	2.5986	1.88364
XLOC_092298	CLCN4	chrX	142681431	142842454	22.3001	18.4232	24.4135	6.18677	5.7812	8.72097	5.28193	5.74643	6.40587
XLOC_093703	CLCN4	chrX	142681431	142842454	13.134	11.1744	10.134	4.71208	4.7644	4.18395	3.34284	3.16048	4.81422
XLOC_093704	CLCN4	chrX	142681431	142842454	1.32528	1.38749	2.59206	0.534616	0.311352	0.344204	0.0603148	0.0664486	0.405473
XLOC_093705	CLCN4	chrX	142681431	142842454	1.24176	0.742441	1.94174	0.0	0.193578	0.128538	0.559103	0.123637	0.108316
XLOC_047903	CLDN10	chr12	76765421	76782962	12.1564	8.82202	6.78573	31.4129	28.9894	27.4212	29.7568	27.4986	29.0343
XLOC_048199	CLDN10	chr12	76792331	76795538	0.0	0.0	0.0	56.2038	38.725	52.835	25.1222	19.3882	33.4448
XLOC_048200	CLDN10	chr12	76800578	76822452	0.0	2.20555	0.0	13.4546	12.5983	15.3005	4.06451	6.37801	7.46557
XLOC_049635	CLDN10	chr12	76797777	76800404	1.46431	0.438188	0.229216	8.85113	6.83212	9.11845	2.44266	2.44856	3.23016
XLOC_047904	CLDN10;DZIP1;CLDN10;DNAJC3	chr12	76837214	76962124	3.35974	6.02993	5.24382	23.3784	17.6276	22.9556	21.9956	29.2667	21.3285

XLOC_048201	CLDN10;DZIP1;CLDN10;DNAJC3	chr12	76837214	76962124	35.6687	41.7275	41.4752	48.823	42.7829	46.073	53.8278	63.9715	48.394
XLOC_049636	CLDN10;DZIP1;CLDN10;DNAJC3	chr12	76837214	76962124	2.50413	0.249741	0.0	0.299255	0.522215	0.346499	0.0	0.16704	0.218763
XLOC_049637	CLDN10;DZIP1;CLDN10;DNAJC3	chr12	76837214	76962124	0.0	0.937009	0.0	0.421107	0.731103	0.485746	0.0	0.0	0.409672
XLOC_049638	CLDN10;DZIP1;CLDN10;DNAJC3	chr12	76837214	76962124	2.51806	1.1293	0.0	0.563965	0.981231	0.521254	0.340244	0.0	0.0
XLOC_038428	CLDN16	chr1	77367096	77515127	1.18684	1.10119	0.74315	20.587	12.385	16.2038	24.4925	31.3332	26.5206
XLOC_039688	CLDN16	chr1	77367096	77515127	0.0	0.0	0.0	2.44655	1.53766	3.4378	2.8182	1.92379	2.34482
XLOC_040260	CLDN18	chr1	132188770	132189257	0.0	0.0	0.0	0.0	0.0	0.225912	0.0	0.0	0.0
XLOC_040261	CLDN18	chr1	132190765	132191280	0.0	0.60443	0.0	0.181124	0.0	0.208437	0.0	0.0	0.175997
XLOC_040262	CLDN18	chr1	132191466	132194736	0.0	0.0	0.0	0.291758	0.182604	0.145232	0.234533	0.421627	0.284823
XLOC_092464	CLDN2	chrX	60055961	60179368	0.0	0.0590713	0.171824	20.3188	15.2935	20.5006	18.7079	30.2464	27.2922
XLOC_076930	CLEC12B;CLEC12A	chr5	100361609	100416272	93.729	92.3807	83.5146	86.0456	71.4318	81.4921	84.6751	121.175	84.152
XLOC_062347	CLEC3A;VAT1L;WWOX	chr18	5210415	5334793	3.54876	4.58142	3.44435	2.27058	2.67256	2.09907	2.06784	2.27846	2.4494
XLOC_062553	CLEC3A;VAT1L;WWOX	chr18	5210415	5334793	0.0	0.0	0.0	0.658333	0.427818	0.758327	0.327481	0.0	0.640004
XLOC_062554	CLEC3A;VAT1L;WWOX	chr18	5210415	5334793	0.555269	0.664475	1.30343	0.547625	1.04456	0.865906	0.65838	0.446022	0.679837
XLOC_059984	CLGN	chr17	17523374	17625284	41.3301	33.5337	37.7453	15.2695	16.9202	16.4481	20.3288	15.8131	19.3274
XLOC_092414	CLIC2	chrX	38484369	38552161	51.2507	59.321	53.5359	105.802	96.9668	109.811	112.412	129.857	120.67
XLOC_065870	CLIC4	chr2	128698852	128801725	7.92274	5.69152	8.7769	1.75963	2.84671	2.35144	0.779249	0.829166	1.76194
XLOC_061630	CLIP1	chr17	55321607	55322631	0.0	1.24821	0.0	2.6933	2.67635	3.03187	2.87794	3.50785	2.98976
XLOC_062228	CLIP3;SYNE4;SDHAF1;ALKBH6	chr18	46850800	46878341	2.421	0.723822	0.946513	0.0	0.0943823	0.0	0.0	0.0	0.0
XLOC_044206	CLIP4	chr11	70646669	70662804	20.6964	23.9999	18.548	14.1628	13.3243	13.8703	14.1942	15.9664	12.3977
XLOC_044207	CLIP4	chr11	70646669	70662804	2.72306	3.11715	2.13367	14.311	15.8009	14.2122	10.2189	12.6402	13.9931
XLOC_044208	CLIP4	chr11	70700611	70729162	25.4209	27.1023	26.6239	14.337	12.68	12.9963	10.3072	15.0762	7.84653
XLOC_044887	CLIP4	chr11	70646669	70662804	591.602	619.538	505.668	846.629	611.458	803.657	653.957	1020.06	580.968
XLOC_044888	CLIP4	chr11	70663788	70688097	41.3073	42.4592	36.8673	90.3775	94.0216	94.6926	102.289	93.5949	111.884
XLOC_044889	CLIP4	chr11	70688157	70700131	1.9698	3.14262	2.56853	0.647491	0.630807	0.244899	0.418416	0.202218	0.806817

XLOC_044890	CLIP4	chr11	70700611	70729162	0.0	0.565275	0.0	0.254027	0.0738247	0.195976	0.0	0.283259	0.16502
XLOC_046508	CLIP4	chr11	70646669	70662804	8.05308	8.81472	4.1907	2.16181	3.93009	3.85598	3.76755	3.93969	4.89118
XLOC_046509	CLIP4	chr11	70729288	70729914	0.0	5.53708	6.0337	4.28652	3.0013	2.39279	1.52091	2.45034	1.88339
XLOC_046510	CLIP4	chr11	70730176	70731058	0.998292	1.49264	0.780757	0.626241	0.857662	0.517495	0.541839	0.498454	0.522974
XLOC_046511	CLIP4	chr11	70731250	70731657	2.89876	0.865282	0.0	0.0	0.223025	0.297071	0.253375	0.848788	0.251383
XLOC_055428	CLMP	chr15	34237586	34284514	24.4544	33.5477	36.8263	53.5155	60.3092	51.2641	54.278	36.4813	46.4872
XLOC_055429	CLMP	chr15	34306701	34436289	1.57431	2.76643	3.10039	1.73862	2.2727	1.35741	3.72411	1.82512	3.13782
XLOC_056304	CLMP	chr15	34237586	34284514	2.53349	4.53923	3.95694	25.6159	20.5243	23.1571	27.8597	19.1219	28.1575
XLOC_056305	CLMP	chr15	34306701	34436289	0.0	2.87129	0.938667	1.82847	2.62098	1.24302	3.02933	2.51124	3.24682
XLOC_055453	CLPB	chr15	52712419	52730367	6.44626	12.9087	12.0011	38.3168	44.6446	37.1602	49.054	40.8646	49.624
XLOC_083433	CLPP	chr7	19303306	19310225	142.552	62.2215	96.1346	38.3576	55.7695	54.7919	30.781	29.0729	38.6584
XLOC_084316	CLPP	chr7	19302364	19303232	73.2935	26.7876	31.0489	7.11548	11.606	10.7636	10.1258	5.89459	9.4206
XLOC_040211	CLSTN2	chr1	129796326	129797747	16.5998	18.3231	16.1236	23.6071	26.2881	25.8241	32.3064	31.2591	28.6819
XLOC_078469	CLSTN3	chr5	103605729	103606688	0.0	0.0	0.0	0.0808758	0.0	0.0936109	0.0817751	0.0	0.0788157
XLOC_064182	CLTC	chr19	10856413	10869988	6.75543	4.16956	3.74636	4.80765	4.09826	5.24477	3.42476	3.70518	3.68214
XLOC_064183	CLTC	chr19	10870454	10881892	7.82232	6.30968	7.45291	11.0103	8.11805	10.9048	5.79289	7.84079	7.82851
XLOC_047917	CLYBL	chr12	80462993	80475886	7.39337	7.35922	3.849	5.95419	9.13175	6.58337	2.82187	5.31888	5.77902
XLOC_047918	CLYBL	chr12	80475977	80680618	9.88894	8.36705	12.2708	8.22384	8.9449	7.94198	6.98631	6.99945	7.729
XLOC_047919	CLYBL	chr12	80475977	80680618	12.5836	21.5241	11.5895	80.5802	62.8747	70.5975	84.0321	108.077	73.3208
XLOC_047920	CLYBL	chr12	80475977	80680618	7.61082	3.97356	4.07524	0.64656	0.75448	0.833687	0.73281	0.322387	1.05152
XLOC_047921	CLYBL	chr12	80475977	80680618	8.24447	5.75654	6.21366	0.492916	1.2231	0.667685	0.867395	0.676598	0.694933
XLOC_047922	CLYBL	chr12	80475977	80680618	8.82114	6.18895	6.062	5.12811	6.32639	4.85142	5.3629	6.91347	6.97343
XLOC_048215	CLYBL	chr12	80475977	80680618	4.54596	4.25527	2.69648	8.79955	7.49762	9.98622	9.72649	9.89256	7.90211
XLOC_049761	CLYBL	chr12	80475977	80680618	0.0	0.0	3.08732	0.707967	3.62692	1.61356	0.679905	1.52755	1.36869
XLOC_049762	CLYBL	chr12	80475977	80680618	0.0	1.0863	2.84051	3.25615	1.39316	2.22992	3.4574	3.17215	4.72551
XLOC_049763	CLYBL	chr12	80475977	80680618	11.2658	1.67916	0.0	2.52173	4.25761	5.70083	1.88906	1.60457	3.88105
XLOC_049764	CLYBL	chr12	80475977	80680618	2.76856	1.65518	0.0	4.34012	5.60462	4.43835	4.10311	3.99	2.53456
XLOC_049765	CLYBL	chr12	80475977	80680618	1.86661	2.78857	5.83411	4.17817	5.93584	5.38954	4.65332	6.27098	6.01109
XLOC_049766	CLYBL	chr12	80475977	80680618	8.12627	2.42439	12.6757	10.2401	10.3301	9.80966	11.9422	14.4016	3.49098
XLOC_049767	CLYBL	chr12	80475977	80680618	7.63324	3.65305	7.76279	9.5792	9.6166	9.27273	10.1948	7.79759	8.80494
XLOC_049768	CLYBL	chr12	80475977	80680618	3.77372	7.87988	5.88701	9.44852	11.8283	11.5472	9.7511	10.9423	7.83269
XLOC_049769	CLYBL	chr12	80475977	80680618	11.2552	3.36581	0.0	19.419	11.6738	16.9209	13.4778	7.23433	10.6317
XLOC_049770	CLYBL	chr12	80475977	80680618	8.43483	5.8831	6.59398	12.0892	12.1477	11.3396	11.8647	10.8957	11.8884

XLOC_049771	CLYBL	chr12	80475977	80680618	2.91355	4.7898	4.55513	8.48172	10.2024	10.0904	9.40858	9.69242	6.72998
XLOC_048214	CLYBL;TM9SF2	chr12	80335776	80436401	10.6304	11.487	10.8059	4.66607	5.09024	5.19469	4.18584	3.83302	3.59885
XLOC_076389	CMAS	chr5	88564453	88650257	13.1736	10.8977	11.4417	13.7983	10.9624	11.2118	10.9382	6.15616	11.6388
XLOC_078224	CMAS	chr5	88564453	88650257	0.0	4.21497	3.67421	2.31559	1.81744	0.967374	2.28361	0.924281	2.04375
XLOC_078225	CMAS	chr5	88564453	88650257	15.058	4.48902	0.0	4.49061	0.380662	0.509092	0.0	0.47912	0.43275
XLOC_071874	CMPK1	chr3	99500477	99501473	17.2512	20.3798	13.4939	19.6367	19.4906	16.1999	22.9196	22.0876	21.5497
XLOC_062184	CMTM4	chr18	34542123	34548062	0.0	0.154501	1.21383	0.660625	0.282226	0.569611	0.141412	0.0366048	0.0
XLOC_062876	CMTM4	chr18	34542123	34548062	0.0	0.0	0.0	1.06766	0.868796	1.17631	0.0	0.0	0.0
XLOC_062877	CMTM4	chr18	34542123	34548062	0.0	0.0	0.0	0.0	0.494933	0.663896	0.0	0.619894	0.565829
XLOC_062878	CMTM4	chr18	34548264	34548656	0.0	0.920404	0.0	0.275829	0.0	1.26285	0.268697	0.0	0.0
XLOC_062879	CMTM4	chr18	34549137	34549653	0.0	0.0	0.0	0.0	0.156301	0.207863	0.0	0.0	0.0
XLOC_062880	CMTM4	chr18	34550031	34550753	1.28127	0.0	0.0	0.114789	0.0	0.132604	0.0	0.0	0.0
XLOC_062388	CMTM4;CMTM3;CMTM2	chr18	34440700	34523024	38.6777	51.8712	52.797	8.24227	8.35396	9.87686	11.1414	15.2232	9.79938
XLOC_062870	CMTM4;CMTM3;CMTM2	chr18	34440700	34523024	5.42341	5.5617	12.1217	1.80566	2.97041	4.26316	2.11133	2.63781	3.38509
XLOC_062871	CMTM4;CMTM3;CMTM2	chr18	34440700	34523024	0.0	9.80566	5.12721	3.53969	4.94927	2.65558	3.81737	4.95919	9.05334
XLOC_062872	CMTM4;CMTM3;CMTM2	chr18	34440700	34523024	1.89598	7.36411	7.40712	1.69752	2.79337	2.15023	2.86816	2.99638	2.31002
XLOC_062873	CMTM4;CMTM3;CMTM2	chr18	34440700	34523024	12.1906	21.8047	47.5068	1.63852	4.59643	3.07999	3.55696	5.18877	5.24617
XLOC_062874	CMTM4;CMTM3;CMTM2	chr18	34440700	34523024	8.87389	3.53164	13.8527	0.793784	2.95722	1.81821	2.84115	2.3081	3.59057
XLOC_062875	CMTM4;CMTM3;CMTM2	chr18	34440700	34523024	16.0424	15.941	16.6717	0.957249	3.23843	0.541724	3.15008	2.03582	1.38236
XLOC_066532	CNKSR1;CNKSR1	chr2	127458204	127510893	0.0	0.343526	0.898446	0.308841	1.07147	1.06575	0.10364	0.0	0.499744
XLOC_082987	CNN2	chr7	45141475	45160069	13.1347	16.9474	7.17342	36.7225	27.1465	30.0105	29.7011	33.2456	29.5766
XLOC_083624	CNN2	chr7	45141475	45160069	10.8225	11.3076	8.62576	22.3369	17.2116	19.4932	19.8523	23.549	19.4625
XLOC_062821	CNOT1	chr18	26445620	26445949	0.0	0.0	0.0	3.76503	0.641842	0.857049	0.720299	0.40508	0.363685

XLOC_074660	CNOT4	chr4	99988272	99989538	0.0	0.195174	0.0	0.233965	0.562103	0.745683	0.059401	0.130864	0.285193
XLOC_081866	CNOT6L	chr6	94284084	94284597	0.0	0.607824	0.0	0.546423	0.315198	0.41919	0.0	0.802538	0.35396
XLOC_080234	CNOT6L;CXCL13	chr6	93757295	94283926	13.9623	9.99997	12.7372	12.6861	12.3028	12.3698	11.655	13.1393	11.2776
XLOC_081735	CNOT6L;CXCL13	chr6	93757295	94283926	0.0	0.0	0.0	0.383747	0.111147	0.442968	0.64081	0.425567	0.248973
XLOC_081736	CNOT6L;CXCL13	chr6	93757295	94283926	0.0	0.62179	1.62608	0.186328	0.322335	0.214359	0.184602	0.205138	0.543073
XLOC_081737	CNOT6L;CXCL13	chr6	93757295	94283926	0.0	0.280385	0.733334	0.336115	0.330775	0.292438	0.128418	0.18839	0.286933
XLOC_081738	CNOT6L;CXCL13	chr6	93757295	94283926	0.0	0.0	0.0	0.0	0.427083	0.567308	0.123188	0.136302	0.0
XLOC_081739	CNOT6L;CXCL13	chr6	93757295	94283926	1.08517	0.0	0.848628	0.194482	0.169391	1.01201	0.0980086	0.108251	0.47359
XLOC_081740	CNOT6L;CXCL13	chr6	93757295	94283926	0.0	0.0	0.0	0.489279	0.631958	1.12203	0.479412	0.267409	0.0
XLOC_081741	CNOT6L;CXCL13	chr6	93757295	94283926	0.0	0.484209	0.0	0.290221	0.696487	0.504121	0.367404	0.162043	0.636514
XLOC_081742	CNOT6L;CXCL13	chr6	93757295	94283926	0.0	0.310998	0.0	0.186407	0.733515	0.594513	0.237183	0.208838	0.454597
XLOC_081743	CNOT6L;CXCL13	chr6	93757295	94283926	0.0	0.0	0.528079	0.665608	0.739975	0.490843	0.429986	0.473711	0.472027
XLOC_081744	CNOT6L;CXCL13	chr6	93757295	94283926	0.0	0.0	0.0	0.200802	0.349706	0.580393	0.202263	0.111725	0.293361
XLOC_081745	CNOT6L;CXCL13	chr6	93757295	94283926	0.0	0.917132	1.799	0.549703	0.539839	0.955062	0.417852	0.614143	0.669861
XLOC_081746	CNOT6L;CXCL13	chr6	93757295	94283926	0.0	0.530385	1.38707	1.11257	0.413312	0.366278	0.316592	0.351161	0.772694
XLOC_081747	CNOT6L;CXCL13	chr6	93757295	94283926	1.37376	0.821311	2.14796	0.492252	0.962741	1.13675	0.740494	0.273114	0.598904
XLOC_081748	CNOT6L;CXCL13	chr6	93757295	94283926	0.0	0.777087	2.03232	0.465749	0.810113	0.53798	0.350762	0.387934	0.680122
XLOC_081749	CNOT6L;CXCL13	chr6	93757295	94283926	0.647197	0.0	2.53178	0.522186	0.5576	0.60521	0.117857	0.324547	0.565802
XLOC_081750	CNOT6L;CXCL13	chr6	93757295	94283926	0.0	1.05303	0.0	0.31556	0.273556	0.72726	0.157176	0.348649	0.460249
XLOC_081751	CNOT6L;CXCL13	chr6	93757295	94283926	0.0	4.25732	0.0	0.640998	0.535815	0.719508	0.0	0.0	2.4549
XLOC_081752	CNOT6L;CXCL13	chr6	93757295	94283926	0.0	0.381693	1.99649	0.457537	0.696463	0.396418	0.114905	0.127064	0.222723
XLOC_081753	CNOT6L;CXCL13	chr6	93757295	94283926	0.874025	0.261399	0.683658	0.156675	0.273328	0.272056	0.158494	0.437072	0.381735
XLOC_081754	CNOT6L;CXCL13	chr6	93757295	94283926	0.521627	0.0	1.2245	0.280617	0.408972	0.379686	0.0476061	0.261983	0.41061

XLOC_081755	CNOT6L;CXCL13	chr6	93757295	94283926	0.0	0.0	0.763903	0.0875331	0.381488	0.405104	0.0883904	0.0	0.170571
XLOC_081756	CNOT6L;CXCL13	chr6	93757295	94283926	0.829895	0.744634	1.94751	0.520696	0.259619	0.0861303	0.301229	0.166092	0.435023
XLOC_081757	CNOT6L;CXCL13	chr6	93757295	94283926	0.0	0.498739	0.869616	0.298934	0.392006	0.346627	0.0506831	0.223181	0.0
XLOC_081758	CNOT6L;CXCL13	chr6	93757295	94283926	1.55715	1.39617	0.0	1.39464	0.484293	0.16088	0.975953	0.772203	0.13568
XLOC_081759	CNOT6L;CXCL13	chr6	93757295	94283926	5.23351	1.56016	0.0	0.468346	0.396429	1.5912	0.0	0.498606	0.902081
XLOC_081760	CNOT6L;CXCL13	chr6	93757295	94283926	0.0	0.891893	0.0	0.0890957	0.854153	0.721523	0.179882	0.0992847	0.868042
XLOC_081761	CNOT6L;CXCL13	chr6	93757295	94283926	0.50242	0.601265	0.0	0.585629	0.472769	0.57475	0.642175	0.201917	0.263679
XLOC_081762	CNOT6L;CXCL13	chr6	93757295	94283926	1.85808	1.11135	1.4533	0.582843	0.435606	0.481804	0.336599	0.371417	0.405682
XLOC_081763	CNOT6L;CXCL13	chr6	93757295	94283926	0.627541	0.375455	1.47296	0.506337	0.540751	0.717324	0.400117	0.37772	0.713255
XLOC_081764	CNOT6L;CXCL13	chr6	93757295	94283926	1.10003	0.657836	1.72046	0.887129	0.944293	0.683846	0.695261	0.877707	0.768076
XLOC_081765	CNOT6L;CXCL13	chr6	93757295	94283926	0.0	0.190536	0.498333	0.513913	0.798263	0.330919	0.580052	0.638884	1.00234
XLOC_081766	CNOT6L;CXCL13	chr6	93757295	94283926	0.900133	0.538401	2.11218	0.726073	0.42215	1.02717	0.16315	0.629978	1.02206
XLOC_081767	CNOT6L;CXCL13	chr6	93757295	94283926	0.476281	0.284999	0.745402	1.11035	1.23274	0.990794	0.435044	0.478684	1.58324
XLOC_081768	CNOT6L;CXCL13	chr6	93757295	94283926	0.884415	1.05847	0.34605	0.475824	0.867318	1.15013	0.444595	0.578014	0.541636
XLOC_081769	CNOT6L;CXCL13	chr6	93757295	94283926	0.0	0.453405	1.18588	0.291179	0.595389	0.766657	0.317854	0.305518	0.549589
XLOC_081770	CNOT6L;CXCL13	chr6	93757295	94283926	0.555269	0.332238	1.7379	0.398273	0.609322	1.09681	0.303868	0.557527	0.825517
XLOC_081771	CNOT6L;CXCL13	chr6	93757295	94283926	1.08148	0.80887	1.69244	0.921158	0.805391	0.562214	0.443994	0.760248	0.756665
XLOC_081772	CNOT6L;CXCL13	chr6	93757295	94283926	0.0	0.520317	1.36086	0.4678	0.863226	0.783419	0.422828	0.407353	0.811141
XLOC_081773	CNOT6L;CXCL13	chr6	93757295	94283926	0.0	0.699764	0.610072	0.908762	0.520067	0.649021	0.249622	0.470593	0.648044
XLOC_081774	CNOT6L;CXCL13	chr6	93757295	94283926	0.320934	0.384141	1.00472	0.575624	0.554467	0.568035	0.382198	0.420124	0.730356
XLOC_081775	CNOT6L;CXCL13	chr6	93757295	94283926	0.0	0.788518	1.54673	0.768004	0.464488	0.34233	0.479912	0.396496	0.403262
XLOC_081776	CNOT6L;CXCL13	chr6	93757295	94283926	0.0	0.136308	1.42604	0.490205	0.50033	0.56871	0.457924	0.412195	0.51813

XLOC_081777	CNOT6L;CXCL13	chr6	93757295	94283926	0.0	0.56647	1.48142	1.52776	1.17615	1.36833	0.84357	0.374547	0.165
XLOC_081778	CNOT6L;CXCL13	chr6	93757295	94283926	1.73131	1.03559	1.35423	0.853462	0.270729	0.179643	0.785004	0.692691	0.226855
XLOC_081779	CNOT6L;CXCL13	chr6	93757295	94283926	1.73247	1.5531	0.0	0.930828	0.67258	0.715196	0.927742	0.685852	0.60344
XLOC_081780	CNOT6L;CXCL13	chr6	93757295	94283926	0.828943	0.247927	1.29685	0.668699	0.518647	0.516193	0.601779	0.248855	0.579368
XLOC_081781	CNOT6L;CXCL13	chr6	93757295	94283926	0.0	0.467504	1.63031	0.747234	0.612578	0.974935	0.665534	0.523216	0.865597
XLOC_081782	CNOT6L;CXCL13	chr6	93757295	94283926	0.0	0.0	4.45803	1.53661	0.432142	0.578725	0.95806	1.62799	1.47768
XLOC_081783	CNOT6L;CXCL13	chr6	93757295	94283926	0.0	2.24593	1.95776	0.673017	0.773907	0.772584	0.0	0.737495	0.653141
XLOC_081784	CNOT6L;CXCL13	chr6	93757295	94283926	0.0	0.868586	1.36304	0.832979	0.8191	0.784673	0.264687	0.408002	0.81244
XLOC_081785	CNOT6L;CXCL13	chr6	93757295	94283926	0.533557	0.159627	0.0	0.526224	0.752913	0.499296	0.340788	0.321522	0.839965
XLOC_081786	CNOT6L;CXCL13	chr6	93757295	94283926	0.543877	0.406891	1.27707	0.70727	0.726373	0.793	0.374016	0.465803	0.666581
XLOC_081787	CNOT6L;CXCL13	chr6	93757295	94283926	0.0	3.01324	0.0	0.452172	1.53281	0.512525	0.853976	0.964524	0.435697
XLOC_081788	CNOT6L;CXCL13	chr6	93757295	94283926	1.97878	1.57847	1.54814	0.650442	1.03313	1.16498	0.540388	0.26457	1.15323
XLOC_081789	CNOT6L;CXCL13	chr6	93757295	94283926	0.660932	0.593321	1.55182	0.711258	0.570891	1.06653	0.272409	0.232912	0.636327
XLOC_081790	CNOT6L;CXCL13	chr6	93757295	94283926	0.0	0.290515	0.759803	0.522373	0.531236	1.00735	0.527544	0.388178	0.933119
XLOC_081791	CNOT6L;CXCL13	chr6	93757295	94283926	0.553992	0.497212	1.30043	0.546367	0.694775	0.979112	0.404236	0.333753	0.823623
XLOC_081792	CNOT6L;CXCL13	chr6	93757295	94283926	0.0	1.02214	2.6733	1.1487	0.668086	1.06393	0.619971	0.341892	0.821025
XLOC_081793	CNOT6L;CXCL13	chr6	93757295	94283926	0.0	0.0	5.41143	1.24613	0.521486	0.0	1.14651	0.652487	0.596926
XLOC_081794	CNOT6L;CXCL13	chr6	93757295	94283926	0.0	0.0	0.0	0.367996	0.31387	0.0	0.705068	1.18895	0.355559
XLOC_081795	CNOT6L;CXCL13	chr6	93757295	94283926	1.79259	0.0	1.40079	0.481536	0.834702	0.184935	0.319628	0.177283	0.156061
XLOC_081796	CNOT6L;CXCL13	chr6	93757295	94283926	1.22072	3.28466	0.954491	0.765595	0.475855	1.01107	0.329927	0.486284	0.425985
XLOC_081797	CNOT6L;CXCL13	chr6	93757295	94283926	1.24168	0.742441	0.0	1.33495	1.06468	0.899763	0.223641	0.618133	0.649899
XLOC_081798	CNOT6L;CXCL13	chr6	93757295	94283926	0.0	0.319689	0.836096	0.383218	0.500726	0.664766	0.193177	0.106672	0.186646

XLOC_081799	CNOT6L;CXCL13	chr6	93757295	94283926	0.0	0.634548	0.0	1.14089	0.493276	0.656126	0.376503	0.418495	0.738871
XLOC_081800	CNOT6L;CXCL13	chr6	93757295	94283926	0.944476	0.989032	2.21723	0.465778	0.666715	0.196482	0.215692	0.42718	0.660977
XLOC_081801	CNOT6L;CXCL13	chr6	93757295	94283926	0.683919	0.204581	1.07013	1.10359	0.642615	0.284179	0.497835	0.617052	0.836956
XLOC_081802	CNOT6L;CXCL13	chr6	93757295	94283926	0.0	0.436314	0.0	1.04601	0.908689	0.452713	0.523725	0.434847	0.254471
XLOC_081803	CNOT6L;CXCL13	chr6	93757295	94283926	1.15562	0.576306	1.50731	0.725402	0.725635	0.681523	0.669664	0.31007	0.910128
XLOC_081804	CNOT6L;CXCL13	chr6	93757295	94283926	0.783774	1.17259	0.920064	0.843401	0.799693	0.815661	0.537782	0.354848	0.994472
XLOC_081805	CNOT6L;CXCL13	chr6	93757295	94283926	2.49828	0.996272	0.65141	0.447851	0.78153	0.864263	0.226691	0.333319	0.945796
XLOC_081806	CNOT6L;CXCL13	chr6	93757295	94283926	0.0	0.0	4.08494	0.468101	0.6051	0.537053	1.14895	0.0	1.13536
XLOC_081807	CNOT6L;CXCL13	chr6	93757295	94283926	3.73144	1.85929	0.972524	0.78006	1.55126	1.54508	1.23209	0.61918	1.95303
XLOC_081808	CNOT6L;CXCL13	chr6	93757295	94283926	0.0	1.09595	3.82185	1.25903	1.43513	0.82493	0.611961	0.428857	1.6016
XLOC_081809	CNOT6L;CXCL13	chr6	93757295	94283926	0.0	0.959884	0.836842	0.863003	0.796497	0.6116	0.292746	0.322231	1.07567
XLOC_081810	CNOT6L;CXCL13	chr6	93757295	94283926	0.0	0.865648	0.0	0.907939	1.57762	0.898241	0.649553	0.862852	1.38844
XLOC_081811	CNOT6L;CXCL13	chr6	93757295	94283926	2.42318	0.0	0.0	1.51788	2.24543	1.24514	0.213589	0.47567	2.94677
XLOC_081812	CNOT6L;CXCL13	chr6	93757295	94283926	0.661402	0.3957	2.06985	0.889398	0.517976	0.412293	0.602058	0.397935	0.867286
XLOC_081813	CNOT6L;CXCL13	chr6	93757295	94283926	1.78374	0.532997	0.0	1.11805	1.10752	0.73613	0.318103	0.352856	1.24237
XLOC_081814	CNOT6L;CXCL13	chr6	93757295	94283926	0.0	0.0	0.461757	0.687834	0.693671	0.368046	0.430335	0.236922	0.980485
XLOC_081815	CNOT6L;CXCL13	chr6	93757295	94283926	1.19847	1.07495	0.937113	0.536898	0.467265	0.496391	0.0	0.358162	0.731944
XLOC_081816	CNOT6L;CXCL13	chr6	93757295	94283926	0.0	0.576173	2.00925	0.920917	1.30749	1.13408	0.701557	0.579554	0.67355
XLOC_081817	CNOT6L;CXCL13	chr6	93757295	94283926	1.54056	0.920884	1.20417	1.51779	0.958396	1.11428	0.689893	0.152826	0.805459
XLOC_081818	CNOT6L;CXCL13	chr6	93757295	94283926	0.0	0.504551	3.519	0.907252	0.837175	0.93508	0.512679	0.677296	1.22906
XLOC_081819	CNOT6L;CXCL13	chr6	93757295	94283926	0.0	1.42806	0.0	2.99919	1.8188	0.972543	0.0	1.37462	3.30538
XLOC_081820	CNOT6L;CXCL13	chr6	93757295	94283926	1.47677	0.883447	0.577643	0.529514	0.462325	0.46006	0.604056	0.665746	1.35514

XLOC_081821	CNOT6L;CXCL13	chr6	93757295	94283926	1.18862	0.710752	0.929422	0.851985	1.205	0.861598	0.642881	0.473682	1.14079
XLOC_081822	CNOT6L;CXCL13	chr6	93757295	94283926	0.0	0.635623	0.831187	0.190485	0.663744	0.330442	0.0960316	0.212106	0.371107
XLOC_081823	CNOT6L;CXCL13	chr6	93757295	94283926	0.0	0.438079	1.1457	0.393842	1.25445	0.757553	0.262896	0.145526	0.638743
XLOC_081824	CNOT6L;CXCL13	chr6	93757295	94283926	3.32437	1.98396	0.0	0.891907	0.0	1.01963	0.288466	0.0	0.0
XLOC_081825	CNOT6L;CXCL13	chr6	93757295	94283926	2.00273	0.599184	1.17535	0.628495	0.628186	0.885185	0.548548	0.402445	1.05107
XLOC_081826	CNOT6L;CXCL13	chr6	93757295	94283926	0.963705	0.576389	0.753734	1.20913	0.903466	1.29915	0.2617	0.962777	1.00984
XLOC_081827	CNOT6L;CXCL13	chr6	93757295	94283926	0.0	2.38732	3.12119	0.715749	0.305477	1.22322	0.68691	0.771762	0.0
XLOC_081828	CNOT6L;CXCL13	chr6	93757295	94283926	1.05049	0.942367	0.821539	0.28241	0.492072	0.544379	0.0	0.419347	0.0
XLOC_081829	CNOT6L;CXCL13	chr6	93757295	94283926	0.0	2.29901	0.0	1.72301	2.06114	0.785817	0.663003	0.744312	1.99924
XLOC_081830	CNOT6L;CXCL13	chr6	93757295	94283926	0.0	1.32422	0.0	1.58864	2.02796	1.80609	1.13564	0.426274	0.766767
XLOC_081831	CNOT6L;CXCL13	chr6	93757295	94283926	0.0	0.645094	3.37428	0.773286	0.336779	0.894234	0.487177	0.0	0.753241
XLOC_081832	CNOT6L;CXCL13	chr6	93757295	94283926	0.0	1.10419	0.962604	0.551503	0.863758	0.764711	0.554452	0.12259	0.322196
XLOC_081833	CNOT6L;CXCL13	chr6	93757295	94283926	3.50084	0.0	5.46245	0.939194	2.14521	2.86064	1.81731	0.339374	1.81805
XLOC_081834	CNOT6L;CXCL13	chr6	93757295	94283926	0.0	0.639741	0.557729	0.575165	0.279041	0.370215	0.453826	0.428652	0.37386
XLOC_081835	CNOT6L;CXCL13	chr6	93757295	94283926	0.0	0.334807	1.3135	0.250846	0.48244	0.698065	0.510324	0.393268	0.831891
XLOC_081836	CNOT6L;CXCL13	chr6	93757295	94283926	0.870867	0.260455	1.36238	0.858596	1.29365	1.08432	0.86862	0.958123	1.21715
XLOC_081837	CNOT6L;CXCL13	chr6	93757295	94283926	0.0	0.0	0.0	1.23667	1.75205	1.40478	1.56755	0.883339	3.18183
XLOC_081838	CNOT6L;CXCL13	chr6	93757295	94283926	0.0	0.0	2.04701	0.820953	1.22388	0.541844	0.353241	0.520931	1.37004
XLOC_081839	CNOT6L;CXCL13	chr6	93757295	94283926	1.40132	0.838688	0.877434	0.728916	0.682511	0.817235	0.719426	0.677667	0.711502
XLOC_081840	CNOT6L;CXCL13	chr6	93757295	94283926	2.20191	1.31536	0.0	0.591241	0.851637	0.906363	0.389712	0.433378	0.191413
XLOC_081841	CNOT6L;CXCL13	chr6	93757295	94283926	0.0	1.38386	2.17159	0.497663	0.578595	0.383972	0.754489	0.277505	0.80826
XLOC_081842	CNOT6L;CXCL13	chr6	93757295	94283926	4.7823	0.71408	0.0	0.855921	0.184691	0.245783	0.210885	0.0	1.03862

XLOC_081843	CNOT6L;CXCL13	chr6	93757295	94283926	0.0	0.0	0.0	1.30246	1.47374	0.985166	0.0	0.0	0.418586
XLOC_081844	CNOT6L;CXCL13	chr6	93757295	94283926	3.14691	0.627334	1.6407	0.563999	1.3922	0.217438	0.758418	0.732806	1.00727
XLOC_081845	CNOT6L;CXCL13	chr6	93757295	94283926	0.0	0.0	0.0	1.04914	1.81166	0.0	0.62087	0.230342	0.814873
XLOC_081846	CNOT6L;CXCL13	chr6	93757295	94283926	0.719291	0.430309	0.0	0.451352	0.844572	0.373514	0.196215	0.144152	0.502931
XLOC_081847	CNOT6L;CXCL13	chr6	93757295	94283926	0.0	0.910221	1.9045	0.381896	0.667483	0.695678	0.443581	0.305303	0.266037
XLOC_081848	CNOT6L;CXCL13	chr6	93757295	94283926	1.41949	0.56627	0.740527	0.806104	1.07625	0.935114	0.994112	0.190228	0.62088
XLOC_081849	CNOT6L;CXCL13	chr6	93757295	94283926	0.0	0.0	0.0	1.92849	1.63069	0.545605	0.906111	0.512489	1.3923
XLOC_081850	CNOT6L;CXCL13	chr6	93757295	94283926	11.8038	1.75936	0.0	1.05731	1.78192	0.0	0.493155	0.0	1.01619
XLOC_081851	CNOT6L;CXCL13	chr6	93757295	94283926	0.0	1.06338	0.0	0.318659	1.24294	0.734339	0.0	0.528009	0.46475
XLOC_081852	CNOT6L;CXCL13	chr6	93757295	94283926	1.95081	0.875069	3.81437	0.786726	0.838151	1.82052	0.52964	0.38973	0.681368
XLOC_081853	CNOT6L;CXCL13	chr6	93757295	94283926	0.0	0.333478	0.872157	0.399746	1.21836	1.61763	0.0	0.333646	0.778693
XLOC_081854	CNOT6L;CXCL13	chr6	93757295	94283926	0.0	1.70069	2.22389	1.78378	0.775006	1.32372	1.40438	0.847844	1.11599
XLOC_081855	CNOT6L;CXCL13	chr6	93757295	94283926	0.0	0.897537	2.34701	0.806919	1.15585	2.46386	0.524701	0.586272	2.60684
XLOC_081856	CNOT6L;CXCL13	chr6	93757295	94283926	4.30988	3.85566	0.0	3.85415	3.28293	3.06917	1.4724	2.07118	2.60531
XLOC_081857	CNOT6L;CXCL13	chr6	93757295	94283926	0.336932	1.00821	1.84587	1.08776	1.00533	1.08736	0.586268	0.780196	0.825698
XLOC_081858	CNOT6L;CXCL13	chr6	93757295	94283926	0.0	0.0	10.2544	1.17989	0.989853	1.99169	0.54533	0.619894	0.565829
XLOC_081859	CNOT6L;CXCL13	chr6	93757295	94283926	1.68915	1.17912	2.2028	1.06011	0.882603	0.877997	0.410775	0.847929	1.32945
XLOC_081860	CNOT6L;CXCL13	chr6	93757295	94283926	0.0	0.941085	1.96907	1.29735	1.1336	1.63452	0.630376	0.504931	1.21019
XLOC_081861	CNOT6L;CXCL13	chr6	93757295	94283926	3.59408	2.14785	1.40427	2.57455	1.53404	2.22467	1.92238	1.06629	1.87736
XLOC_081862	CNOT6L;CXCL13	chr6	93757295	94283926	0.0	0.564651	1.4768	1.29735	0.985737	1.50376	0.515763	0.883629	1.21019
XLOC_081863	CNOT6L;CXCL13	chr6	93757295	94283926	0.0	0.0	0.0	3.69389	2.73723	6.27725	3.04737	3.93551	1.33433
XLOC_081864	CNOT6L;CXCL13	chr6	93757295	94283926	1.40504	0.0	1.09922	1.63739	1.53999	0.875625	0.766799	0.915331	1.04388

XLOC_081865	CNOT6L;CXCL13	chr6	93757295	94283926	1.32662	0.793856	1.73025	1.46712	1.49179	1.19614	1.29337	0.978177	1.47016
XLOC_083052	CNOT8;MRPL22	chr7	67951931	68232058	89.9378	103.523	100.539	7.3135	10.4585	8.85257	8.2877	7.82071	8.07366
XLOC_085026	CNOT8;MRPL22	chr7	67951931	68232058	26.6543	23.7257	29.5008	2.38953	2.9023	3.10714	2.54517	2.34716	2.44386
XLOC_065879	CNR2	chr2	129592848	129632415	18.1025	5.85802	8.32611	2.43733	3.03108	2.59389	1.30758	0.937361	2.3555
XLOC_057854	CNST	chr16	31428488	31457279	4.96756	4.86422	2.82715	9.43497	8.89208	9.16098	9.94574	16.8439	12.0896
XLOC_058766	CNST	chr16	31457421	31458026	0.0	0.0	0.0	0.289507	0.628068	0.834654	0.433688	1.76212	0.985634
XLOC_058767	CNST	chr16	31458086	31458900	0.0	0.658837	0.0	2.07311	1.54754	1.25562	1.19366	3.95562	1.9231
XLOC_086362	CNTLN	chr8	26977437	26982654	17.2719	10.8483	12.5542	0.0631791	0.0138103	0.110148	0.0161349	0.0	0.0
XLOC_086363	CNTLN	chr8	27008553	27054164	39.5757	43.1485	42.5838	34.7086	38.467	37.6293	38.2353	34.2582	35.452
XLOC_086364	CNTLN	chr8	27085317	27128709	1.62038	0.582852	0.491684	0.233731	0.301619	0.263045	0.190969	0.21454	0.256046
XLOC_086365	CNTLN	chr8	27260332	27316276	203.231	202.153	159.679	536.124	503.913	496.64	536.049	465.411	395.004
XLOC_086827	CNTLN	chr8	26969015	26976946	2.46945	0.77085	1.61291	0.0	0.0483606	0.0641523	0.0	0.0619345	0.0539725
XLOC_086828	CNTLN	chr8	27008553	27054164	7.77892	4.12066	5.28963	0.0	1.06287	1.3142	0.388724	0.434993	0.42687
XLOC_086829	CNTLN	chr8	27085317	27128709	0.833001	3.7406	3.19437	3.74605	3.63017	3.36447	3.63963	2.65568	3.34741
XLOC_086830	CNTLN	chr8	27142725	27174762	11.4084	9.68318	9.46211	11.7013	11.4086	13.1141	7.06967	7.6013	7.23329
XLOC_086831	CNTLN	chr8	27197459	27202868	0.644311	2.63747	2.64212	16.9092	28.8803	15.7364	16.2646	6.43253	19.6816
XLOC_086832	CNTLN	chr8	27225636	27229352	0.0	0.0866408	0.0	10.788	6.35422	7.0227	7.47135	12.025	6.72912
XLOC_086833	CNTLN	chr8	27260332	27316276	12.929	3.85452	0.0	6.376	2.91985	3.91585	3.21964	2.43888	2.22457
XLOC_087534	CNTLN	chr8	27008553	27054164	0.0	0.0	3.5037	0.0	0.0	0.0	0.0	0.0	0.0
XLOC_087535	CNTLN	chr8	27008553	27054164	0.0	6.90592	0.0	3.46972	1.7339	4.6617	3.16219	1.44334	0.0
XLOC_087536	CNTLN	chr8	27008553	27054164	35.6412	22.1631	18.5457	3.45369	5.25297	4.56386	3.11147	4.63445	6.18019
XLOC_087537	CNTLN	chr8	27054278	27054753	43.2857	25.8559	24.9113	25.2827	28.8841	28.1199	22.9517	22.6231	23.1649
XLOC_087538	CNTLN	chr8	27054872	27055554	9.65299	1.64888	4.31228	3.953	5.58348	3.85105	2.47749	2.60431	4.92965
XLOC_087539	CNTLN	chr8	27085317	27128709	0.556979	0.0	0.0	0.0	0.0	0.0	0.0	0.0	0.0
XLOC_087540	CNTLN	chr8	27085317	27128709	0.0	0.0	0.962706	0.0	0.0	0.0	0.0	0.0	0.0
XLOC_087541	CNTLN	chr8	27139750	27141268	4.25277	2.22657	5.82346	1.71586	2.7089	2.81898	1.4067	1.65513	1.95273
XLOC_087542	CNTLN	chr8	27141527	27141968	7.65406	6.0945	7.96876	9.13138	11.0219	9.43198	5.60935	6.75095	7.08858
XLOC_087543	CNTLN	chr8	27142467	27142598	139.048	88.5135	0.0	205.276	88.6298	128.446	71.6096	106.504	49.7444
XLOC_087544	CNTLN	chr8	27207594	27208174	0.0	0.0	0.0	1.53305	0.531783	0.70682	0.305701	0.508427	0.596343
XLOC_087545	CNTLN	chr8	27213156	27213605	0.0	0.740959	0.0	4.88477	2.2983	1.78442	0.65547	1.94701	1.29296
XLOC_087546	CNTLN	chr8	27214766	27215207	2.55135	0.761812	0.0	2.28285	2.75548	2.358	1.57062	2.00028	1.32911

XLOC_087547	CNTLN	chr8	27215976	27216252	0.0	1.80229	4.7121	5.95841	2.73613	3.66652	1.5129	4.00449	2.60193
XLOC_087548	CNTLN	chr8	27218327	27218750	0.0	0.0	0.0	2.43719	2.93827	1.95627	2.62732	1.86504	1.65471
XLOC_087549	CNTLN	chr8	27221740	27221971	0.0	2.80744	0.0	5.09524	4.90081	2.83216	7.5978	4.35387	4.84486
XLOC_087550	CNTLN	chr8	27222626	27222953	8.51906	1.27024	0.0	5.71361	6.16674	9.10208	4.0049	4.91528	3.31099
XLOC_087551	CNTLN	chr8	27230411	27231686	0.0	0.193603	0.0	1.85666	1.31796	1.4794	0.530355	1.75255	0.282901
XLOC_087552	CNTLN	chr8	27231931	27232517	0.0	0.0	0.0	3.17439	1.70442	1.91682	0.603104	2.84157	1.91114
XLOC_087553	CNTLN	chr8	27232988	27233409	0.0	0.0	0.0	4.66571	2.74865	1.68932	1.92457	2.9523	1.42898
XLOC_087554	CNTLN	chr8	27233780	27234206	0.0	0.0	2.10313	3.13304	2.49098	4.69873	4.72613	4.21686	1.6364
XLOC_087555	CNTLN	chr8	27234265	27235790	0.0	0.0	0.413834	2.98739	1.49267	2.0897	1.88218	1.85919	1.1564
XLOC_087556	CNTLN	chr8	27235981	27236450	0.0	0.693495	0.0	1.66249	1.79453	1.19391	1.64037	1.5973	1.41241
XLOC_087557	CNTLN	chr8	27238883	27239080	0.0	0.0	0.0	5.65469	3.38947	4.61644	1.20442	13.9207	3.96632
XLOC_087558	CNTLN	chr8	27246602	27247009	0.0	0.0	0.0	3.1116	2.89933	3.56485	0.760124	3.96101	1.25692
XLOC_087559	CNTLN	chr8	27259698	27260201	0.0	0.0	4.90641	1.87402	0.486255	0.0	0.55689	0.825182	0.546193
XLOC_087560	CNTLN	chr8	27260332	27316276	8.35205	5.62219	6.53536	2.24655	2.41467	1.73696	2.23908	1.73033	1.41534
XLOC_087561	CNTLN	chr8	27260332	27316276	10.8766	10.2166	18.2176	2.64413	3.50367	1.2843	3.89566	1.69526	2.70781
XLOC_087562	CNTLN	chr8	27260332	27316276	9.09186	4.35049	2.84454	0.244456	0.92379	0.848817	0.576671	0.454459	0.635266
XLOC_055389	CNTN5	chr15	9436040	9466299	0.0	0.879833	1.72586	12.0953	11.7118	12.688	12.6587	12.0513	14.17
XLOC_055707	CNTN5	chr15	9716333	9725251	0.0	0.516466	0.0	0.154768	0.402597	0.0	0.462797	0.171062	0.150502
XLOC_056126	CNTN5	chr15	9471046	9471562	0.0	0.0	0.0	0.541859	1.87561	1.03931	0.358288	0.596989	0.351019
XLOC_056127	CNTN5	chr15	9472698	9473549	0.0	0.31186	0.0	0.186916	0.977107	0.108096	0.282798	0.624543	0.546265
XLOC_056128	CNTN5	chr15	9560599	9561572	0.886887	0.265242	1.38742	0.715397	1.5946	0.644088	1.44709	0.620808	0.464801
XLOC_056129	CNTN5	chr15	9562379	9563418	0.0	0.245398	0.0	1.10313	1.15512	1.19221	1.34039	0.903245	0.645154
XLOC_056130	CNTN5	chr15	9566838	9567166	0.0	0.0	0.0	0.0	0.0	1.29289	3.62105	0.0	0.365774
XLOC_086419	CNTNAP3	chr8	66511719	66515630	1.90214	6.09878	3.82832	5.14213	2.36962	3.67902	3.81211	3.0939	2.4978
XLOC_086887	CNTNAP3	chr8	66476615	66486550	24.2134	21.3847	22.9184	38.2036	40.5064	38.5304	45.9124	40.4692	38.5334
XLOC_087864	CNTNAP3	chr8	66467962	66468875	2.87198	1.14516	2.24625	1.02955	0.673149	0.595662	0.779997	0.0	1.25397
XLOC_087865	CNTNAP3	chr8	66469685	66470146	0.0	0.711732	1.86125	0.426552	1.84094	0.489969	2.1022	0.0	0.207046
XLOC_087866	CNTNAP3	chr8	66470299	66471233	1.86287	0.835659	1.45704	0.417387	0.43672	0.676253	0.759264	0.186181	0.244034
XLOC_087867	CNTNAP3	chr8	66472177	66474320	2.18552	1.52591	0.855213	2.31919	2.63074	2.12318	3.33413	2.19959	1.94452
XLOC_087868	CNTNAP3	chr8	66475047	66475386	15.921	13.0586	12.4167	20.2871	16.408	13.3833	18.4515	17.6575	18.2332
XLOC_087869	CNTNAP3	chr8	66475732	66475890	78.7965	36.2312	94.773	22.9456	28.3726	47.6001	23.7086	20.6642	30.8876
XLOC_087870	CNTNAP3	chr8	66476007	66476468	16.6829	7.11732	20.4738	9.59742	14.7275	11.2693	15.1359	10.9993	12.4227
XLOC_087871	CNTNAP3	chr8	66515740	66516784	0.0	1.95212	0.638194	1.75506	1.53153	1.35488	1.11078	1.38813	1.49689

XLOC_087872	CNTNAP3	chr8	66518536	66518756	0.0	3.22987	0.0	9.79849	3.20558	3.24993	1.7298	0.994001	2.78347
XLOC_062126	CNTNAP4	chr18	3507779	3512726	18.323	10.3372	10.6507	9.76323	10.9179	9.9619	13.5686	11.2006	16.1632
XLOC_062344	CNTNAP4	chr18	3440914	3470881	0.0	0.0	0.0	0.221353	0.192595	0.0	1.11261	0.49201	0.538819
XLOC_062529	CNTNAP4	chr18	3473381	3473546	0.0	0.0	0.0	0.0	0.0	0.0	2.40009	8.36533	5.49626
XLOC_062530	CNTNAP4	chr18	3512953	3513873	0.0	1.41832	2.22565	0.850086	0.889355	0.590226	0.601192	0.09478	0.911166
XLOC_062531	CNTNAP4	chr18	3514235	3515391	0.0	0.433324	0.566659	0.714236	0.396881	0.526569	0.0	0.0	0.189918
XLOC_047805	COG6;LHFP	chr12	22592898	22877806	0.0	0.339759	0.0	0.0	0.0886515	0.941668	0.0	0.0	0.69415
XLOC_048859	COG6;LHFP	chr12	22592898	22877806	0.0	1.01258	0.0	0.303454	0.992811	0.52701	0.307129	0.508086	0.591536
XLOC_048860	COG6;LHFP	chr12	22592898	22877806	2.10575	0.629017	0.0	0.565473	0.489039	0.216823	1.12002	0.207466	1.46493
XLOC_048861	COG6;LHFP	chr12	22592898	22877806	0.0	0.700223	0.0	1.25896	1.08699	0.723212	1.03478	0.230342	2.03718
XLOC_048862	COG6;LHFP	chr12	22592898	22877806	0.0	0.0	1.43633	2.13958	2.85202	1.70623	3.60229	2.90702	3.04002
XLOC_048863	COG6;LHFP	chr12	22592898	22877806	0.0	0.601801	0.524654	1.68328	0.840226	1.74168	0.793426	1.6137	0.996562
XLOC_048864	COG6;LHFP	chr12	22592898	22877806	0.0	0.0	0.0	0.249245	0.272107	0.288803	0.126466	0.418032	0.546812
XLOC_048866	COG6;LHFP	chr12	22592898	22877806	0.0	0.0	0.0	0.0	0.0	0.0	0.0	0.0996908	0.0
XLOC_048867	COG6;LHFP	chr12	22592898	22877806	0.0	0.162381	0.0	0.583965	0.63821	0.395022	0.297095	0.599564	0.284812
XLOC_069888	COL11A1	chr3	40540780	40702129	3.64596	5.66255	4.75627	7.01324	6.47326	6.83303	6.85294	7.41824	6.64576
XLOC_090282	COL12A1	chr9	14869416	14870635	0.0	0.203811	0.0	0.0610794	0.0	0.0	0.0	0.13661	0.0
XLOC_086612	COL27A1	chr8	105224615	105255613	15.5636	21.242	22.3937	45.727	47.9017	47.5287	44.794	45.8754	55.1856
XLOC_086613	COL27A1	chr8	105266865	105269321	0.0	0.446308	0.396962	0.312089	0.506906	0.723981	0.0	0.203842	0.355019
XLOC_087043	COL27A1	chr8	105224615	105255613	2.29023	1.00838	0.322706	1.41363	1.85898	0.959776	0.909286	0.892488	1.31414
XLOC_087044	COL27A1	chr8	105258305	105264605	1.39196	1.46004	0.831762	3.28391	4.58048	3.68112	3.41928	2.79255	3.53269
XLOC_088653	COL27A1	chr8	105264754	105265582	0.0	0.0	0.0	0.0	0.0	0.0	0.0	0.0	0.0941551
XLOC_037825	COL8A1	chr1	43445847	43570617	35.8189	37.9955	26.2567	9.73252	9.23093	11.9947	7.02458	6.70402	7.01274
XLOC_039204	COL8A1	chr1	43445847	43570617	0.990392	3.11127	2.32497	0.754815	0.854331	0.772516	0.497378	0.149274	0.649737
XLOC_039205	COL8A1	chr1	43445847	43570617	4.23272	4.11343	4.96525	0.94824	0.495641	0.877337	0.669328	0.316781	1.10845
XLOC_039206	COL8A1	chr1	43445847	43570617	2.17955	2.17314	3.4102	0.781512	0.568671	0.377249	0.330265	0.21838	0.634962
XLOC_039207	COL8A1	chr1	43445847	43570617	3.98218	1.19083	1.55722	0.267651	0.466538	1.23859	0.540375	0.298259	0.434616
XLOC_039208	COL8A1	chr1	43445847	43570617	1.89708	1.41832	2.22565	0.170017	0.148226	0.393484	0.0858846	0.09478	0.2485
XLOC_039209	COL8A1	chr1	43445847	43570617	1.11358	0.332965	2.61245	0.0997825	0.0868929	0.346106	0.100521	0.111046	0.0971873
XLOC_039210	COL8A1	chr1	43445847	43570617	5.0493	1.50771	0.0	0.4518	0.1948	0.259299	0.666392	0.989984	0.43844
XLOC_039211	COL8A1	chr1	43445847	43570617	0.648939	1.35887	1.01543	0.407236	0.60992	0.471981	0.177255	0.325414	0.510589
XLOC_039212	COL8A1	chr1	43445847	43570617	0.674353	2.42066	0.527587	0.302267	0.739289	0.280222	0.30685	0.0676104	0.117897
XLOC_039213	COL8A1	chr1	43445847	43570617	0.0	1.36394	1.42693	0.613142	0.393364	0.474223	0.083311	0.320797	0.159525

XLOC_039214	COL8A1	chr1	43445847	43570617	3.64132	1.08899	0.0	0.571114	0.426897	0.377725	0.742351	0.18201	0.238523
XLOC_039215	COL8A1	chr1	43445847	43570617	0.0	3.74019	4.19201	0.160115	0.138778	0.368966	0.159432	0.353709	0.311355
XLOC_039216	COL8A1	chr1	43445847	43570617	0.821151	0.98279	1.92784	0.257718	0.128877	0.512676	0.187744	0.123895	0.21555
XLOC_039217	COL8A1	chr1	43445847	43570617	4.16522	2.32669	4.99877	1.07085	0.894376	0.925391	0.891038	0.643467	0.729261
XLOC_039218	COL8A1	chr1	43445847	43570617	6.78209	3.47685	5.30438	1.38926	1.05965	0.703268	1.14	1.06466	1.01523
XLOC_039219	COL8A1	chr1	43445847	43570617	4.30956	3.00573	1.12297	1.15809	0.894416	1.93086	1.28906	0.428058	0.626122
XLOC_039220	COL8A1	chr1	43445847	43570617	1.48661	3.26166	3.8776	0.844194	0.660615	1.08226	0.452469	0.647293	0.390109
XLOC_039221	COL8A1	chr1	43445847	43570617	3.54128	1.05818	1.38368	0.792749	0.274876	0.548087	0.157921	0.17516	0.308326
XLOC_039222	COL8A1	chr1	43445847	43570617	2.05782	3.07364	4.82283	0.921047	1.43427	1.05975	0.912895	0.202861	0.536938
XLOC_039223	COL8A1	chr1	43571142	43571691	0.0	1.65607	2.88731	0.0	0.143319	0.190539	0.0	0.0	0.321626
XLOC_053286	COLEC10	chr14	47240742	47292074	4.15603	5.96444	4.43981	5.30608	7.36755	5.96692	5.92953	5.53474	7.19745
XLOC_053287	COLEC10	chr14	47297957	47322293	26.9788	26.8343	27.4224	46.1552	54.3503	50.1461	54.8495	57.5709	49.8215
XLOC_053576	COLEC10	chr14	47240742	47292074	18.3282	25.8581	18.4527	31.5141	40.8215	32.2532	36.844	35.7652	50.9696
XLOC_054304	COLEC10	chr14	47240742	47292074	0.855542	0.511676	0.0	1.07339	0.46821	0.17753	0.620693	0.342292	0.747266
XLOC_054305	COLEC10	chr14	47292962	47294104	3.67317	1.97767	2.87356	2.76583	3.85242	3.12785	2.93837	2.79678	3.72382
XLOC_054306	COLEC10	chr14	47294253	47295240	6.10342	3.91154	2.04603	5.54852	8.79371	5.24714	5.92943	4.18589	6.62623
XLOC_054307	COLEC10	chr14	47295334	47296436	0.765748	1.37424	0.599033	1.3728	2.39676	2.54415	1.73923	1.38037	2.40895
XLOC_054308	COLEC10	chr14	47297957	47322293	10.6753	7.98263	12.5267	16.4042	17.7214	15.2008	13.4403	14.8915	17.8579
XLOC_054309	COLEC10	chr14	47297957	47322293	9.87939	7.87952	12.236	12.471	14.5524	13.5007	13.3742	14.336	13.5944
XLOC_054310	COLEC10	chr14	47297957	47322293	7.00638	12.5348	10.9235	16.3522	17.3639	16.2471	24.2836	11.8484	16.2655
XLOC_054311	COLEC10	chr14	47297957	47322293	14.359	8.61207	11.2555	26.3153	29.5859	24.4261	22.5773	16.9458	17.2727
XLOC_054312	COLEC10	chr14	47297957	47322293	13.2722	15.828	25.8629	19.6424	17.471	15.4023	24.1911	20.0034	22.8318
XLOC_054313	COLEC10	chr14	47297957	47322293	8.51914	3.81073	6.64281	12.189	11.0352	9.96894	13.8351	14.3362	14.3476
XLOC_054314	COLEC10	chr14	47297957	47322293	13.8918	12.4921	43.5373	25.424	35.8105	18.0734	29.5458	26.5228	29.8359
XLOC_054315	COLEC10	chr14	47297957	47322293	33.933	10.1257	26.4702	16.0496	22.1743	16.2045	15.1905	16.5816	18.2186
XLOC_054316	COLEC10	chr14	47297957	47322293	3.22036	7.70755	8.81942	6.15966	8.75176	7.64537	8.8636	6.89959	9.57602
XLOC_054317	COLEC10	chr14	47297957	47322293	0.0	3.72695	19.484	5.67237	10.1171	7.48072	5.92445	12.5144	11.7615
XLOC_054318	COLEC10	chr14	47297957	47322293	0.0	8.22572	4.30207	8.38088	12.9456	9.32605	9.17482	7.00387	11.2359
XLOC_044117	COMMD1	chr11	60417450	60444150	11.8413	12.4025	12.089	18.1924	16.1603	18.0175	15.7476	20.0531	15.0995
XLOC_044118	COMMD1	chr11	60454545	60468893	14.349	17.6507	17.1559	50.2173	37.0682	48.7783	44.7366	61.069	46.9098
XLOC_044119	COMMD1	chr11	60537090	60557200	25.5706	29.5189	25.0759	31.4929	30.2314	33.6088	31.2783	27.7208	25.9894
XLOC_046309	COMMD1	chr11	60444299	60445552	3.96117	6.91212	5.68169	9.70633	10.1856	10.6315	9.97614	11.5192	9.92677
XLOC_046310	COMMD1	chr11	60468969	60470365	0.0	0.698794	1.37074	1.0471	0.732185	0.546294	0.532334	0.527518	0.357447

XLOC_046311	COMMD1	chr11	60557479	60557646	89.7498	100.15	71.4306	193.992	153.716	186.139	120.385	131.928	134.823
XLOC_046312	COMMD1	chr11	60557971	60558307	20.2331	12.069	12.6232	31.843	26.5551	30.5016	29.1505	28.0792	31.123
XLOC_046313	COMMD1	chr11	60559230	60559899	0.0	0.0	0.0	0.633332	0.440292	1.46225	0.634707	0.140493	0.246552
XLOC_050599	COMMD7;DNMT3B	chr13	62411476	62712975	6.78348	3.47466	5.06743	8.61779	7.91842	9.91685	7.29587	6.03113	7.43866
XLOC_050903	COMMD7;DNMT3B	chr13	62411476	62712975	0.0	0.0	0.0	1.60269	1.40342	1.20559	0.697774	0.9004	1.64256
XLOC_050904	COMMD7;DNMT3B	chr13	62411476	62712975	0.999978	2.26754	1.59871	2.78001	2.32719	3.12452	1.89761	1.76162	2.2287
XLOC_050905	COMMD7;DNMT3B	chr13	62411476	62712975	0.0	3.60459	0.0	2.70837	5.47226	7.33305	1.00861	2.28828	3.64271
XLOC_052126	COMMD7;DNMT3B	chr13	62411476	62712975	0.0	0.538298	0.0	0.967855	1.1184	1.48677	1.44526	0.356295	0.156836
XLOC_052127	COMMD7;DNMT3B	chr13	62411476	62712975	0.0	0.0	0.0	1.93219	1.11175	0.0	0.423112	0.235537	0.625225
XLOC_052128	COMMD7;DNMT3B	chr13	62411476	62712975	0.0	0.0	0.0	0.871942	0.498764	0.332396	0.0	0.631854	1.12603
XLOC_052129	COMMD7;DNMT3B	chr13	62411476	62712975	1.9694	0.588934	0.0	0.705969	0.76916	1.22518	0.623696	0.885148	1.46166
XLOC_052130	COMMD7;DNMT3B	chr13	62411476	62712975	0.0	1.43764	0.0	1.077	0.371795	0.247396	0.848917	1.18149	0.0
XLOC_052131	COMMD7;DNMT3B	chr13	62411476	62712975	0.0	2.70381	7.07033	1.08036	1.62479	2.47396	2.37049	2.64884	1.57056
XLOC_052132	COMMD7;DNMT3B	chr13	62411476	62712975	1.77064	0.529089	0.0	0.792749	0.824629	0.913479	0.473765	0.52548	1.07914
XLOC_052133	COMMD7;DNMT3B	chr13	62411476	62712975	1.76201	1.05303	0.0	1.42001	1.36778	1.09089	1.10023	1.91757	1.07392
XLOC_052134	COMMD7;DNMT3B	chr13	62411476	62712975	1.24596	3.72498	1.9484	9.60006	10.0033	11.221	12.9027	13.7691	18.9117
XLOC_052135	COMMD7;DNMT3B	chr13	62411476	62712975	3.50253	0.0	0.0	0.941482	0.54637	0.967325	0.736997	0.930782	0.509372
XLOC_052136	COMMD7;DNMT3B	chr13	62411476	62712975	0.0	0.0	0.0	0.0	0.815066	1.95141	0.560012	0.622399	0.732468
XLOC_052137	COMMD7;DNMT3B	chr13	62411476	62712975	0.385208	2.88153	2.11024	1.76169	1.66291	2.24502	2.29096	1.93794	1.34834
XLOC_052138	COMMD7;DNMT3B	chr13	62411476	62712975	1.1118	1.32159	1.97606	1.86108	2.35643	2.66152	1.59415	1.86478	1.81617
XLOC_052139	COMMD7;DNMT3B	chr13	62411476	62712975	5.12076	1.529	1.99923	1.83272	0.197503	1.05165	0.450267	0.752679	0.444593
XLOC_052140	COMMD7;DNMT3B	chr13	62411476	62712975	0.284341	0.59562	0.667647	1.02002	1.13914	1.42132	0.46918	0.716136	0.721842
XLOC_052141	COMMD7;DNMT3B	chr13	62411476	62712975	0.0	0.426914	1.11656	1.40734	1.0614	1.4082	0.973432	0.572095	1.12269

XLOC_052142	COMMD7;DNMT3B	chr13	62411476	62712975	0.751427	0.67428	0.0	0.538858	0.764467	1.56049	0.75115	0.903196	0.591007
XLOC_052143	COMMD7;DNMT3B	chr13	62411476	62712975	0.0	0.0	0.0	0.449061	0.380662	1.01819	0.42428	1.91648	3.02926
XLOC_052144	COMMD7;DNMT3B	chr13	62411476	62712975	0.0	3.82787	5.00478	3.72835	4.18456	2.95255	1.11525	3.11886	3.0559
XLOC_052145	COMMD7;DNMT3B	chr13	62411476	62712975	0.0	0.0	3.10419	6.40646	5.16547	4.86664	6.49221	11.8996	4.81633
XLOC_052146	COMMD7;DNMT3B	chr13	62411476	62712975	1.23743	1.84976	1.93508	1.99558	2.12214	1.79342	1.89459	1.35531	1.29537
XLOC_052147	COMMD7;DNMT3B	chr13	62411476	62712975	1.0723	1.28254	0.838579	0.576528	1.1718	0.777846	1.16243	1.06984	1.21679
XLOC_052148	COMMD7;DNMT3B	chr13	62411476	62712975	4.04656	0.60443	3.16137	1.81124	1.56731	0.833748	1.25739	0.399079	1.23198
XLOC_052149	COMMD7;DNMT3B	chr13	62411476	62712975	0.0	0.0	0.0	1.19765	1.62984	1.37905	1.19004	0.754894	1.66299
XLOC_052150	COMMD7;DNMT3B	chr13	62411476	62712975	0.0	0.0	1.35391	0.775688	0.134516	0.1788	0.309249	0.342926	0.452582
XLOC_052151	COMMD7;DNMT3B	chr13	62411476	62712975	0.774772	0.231738	0.60609	0.347241	0.181862	0.160875	0.0	0.543077	0.203106
XLOC_052152	COMMD7;DNMT3B	chr13	62411476	62712975	0.0	0.230504	0.0	0.276314	0.542698	0.400053	0.560062	0.540211	0.134685
XLOC_052153	COMMD7;DNMT3B	chr13	62411476	62712975	0.0	0.0	0.0	4.06829	2.79674	3.7262	1.32217	2.36404	1.57707
XLOC_052154	COMMD7;DNMT3B	chr13	62411476	62712975	2.90537	1.49025	1.62405	3.57294	4.33174	3.45501	4.06119	4.71733	3.52267
XLOC_052155	COMMD7;DNMT3B	chr13	62411476	62712975	1.02084	1.83158	0.0	5.21444	4.78294	4.55033	3.78461	3.87274	4.09969
XLOC_052156	COMMD7;DNMT3B	chr13	62411476	62712975	2.38327	2.1352	3.72252	8.74432	9.02062	11.5143	6.93727	9.12712	8.48888
XLOC_052157	COMMD7;DNMT3B	chr13	62411476	62712975	0.0	2.40048	6.27683	2.15911	0.921358	0.819901	3.1071	0.387925	1.39114
XLOC_052158	COMMD7;DNMT3B	chr13	62411476	62712975	0.0	4.07984	0.0	1.52848	3.66786	2.79448	0.29616	3.31682	1.77568
XLOC_052159	COMMD7;DNMT3B	chr13	62411476	62712975	0.0	1.30295	1.27793	1.56193	1.45095	1.75463	1.14226	1.25738	1.04745
XLOC_052160	COMMD7;DNMT3B	chr13	62411476	62712975	2.08151	1.24358	0.0	1.11795	2.09518	2.78667	1.29221	1.02569	0.905124
XLOC_052161	COMMD7;DNMT3B	chr13	62411476	62712975	0.828943	1.23964	0.64843	1.6346	1.55594	1.46255	0.60178	0.995419	1.08632
XLOC_052162	COMMD7;DNMT3B	chr13	62411476	62712975	3.32437	0.0	5.18785	1.4865	1.78472	2.03926	0.865401	1.9374	2.30314
XLOC_052163	COMMD7;DNMT3B	chr13	62411476	62712975	1.85227	0.0	1.44735	0.66338	1.43681	2.48328	0.824783	0.549161	1.12856

XLOC_052164	COMMD7;DNMT3B	chr13	62411476	62712975	0.0	1.42816	1.8674	2.1398	1.10815	1.22892	1.05443	0.23478	1.03862
XLOC_052165	COMMD7;DNMT3B	chr13	62411476	62712975	0.0	0.0	4.87939	1.00639	0.875564	1.29198	0.674319	0.994055	0.870999
XLOC_052166	COMMD7;DNMT3B	chr13	62411476	62712975	1.33817	0.800063	4.18481	1.79819	0.938069	1.66133	0.360893	1.06462	1.40032
XLOC_052167	COMMD7;DNMT3B	chr13	62411476	62712975	3.96302	0.237067	1.86008	1.42091	1.86021	1.64558	1.29552	1.11091	0.831065
XLOC_052168	COMMD7;DNMT3B	chr13	62411476	62712975	3.6782	0.0	14.346	1.97346	2.8138	1.12604	3.49032	0.355876	1.27277
XLOC_052169	COMMD7;DNMT3B	chr13	62411476	62712975	1.1153	3.0013	1.74432	0.799489	1.39241	1.84873	0.805387	0.444862	0.973367
XLOC_052170	COMMD7;DNMT3B	chr13	62411476	62712975	0.0	1.48416	0.646949	1.55673	1.16431	1.54505	0.675478	0.827638	1.15609
XLOC_052171	COMMD7;DNMT3B	chr13	62411476	62712975	0.0	1.63333	2.13582	0.734197	1.80832	1.55425	1.35007	0.950571	1.90569
XLOC_052172	COMMD7;DNMT3B	chr13	62411476	62712975	0.346124	0.310712	1.62532	0.465591	0.597865	0.468401	0.633851	0.313587	0.333219
XLOC_070227	COPA;PEX19;NCSTN;DCAF8;PEA15;ATP1A4;CASQ1	chr3	9432263	9609333	9.322	11.696	12.7114	8.67047	8.14896	8.56086	11.2441	12.266	8.1001
XLOC_070651	COPA;PEX19;NCSTN;DCAF8;PEA15;ATP1A4;CASQ1	chr3	9432263	9609333	2.2238	2.32718	0.0	2.29147	2.68959	2.18871	3.11148	2.43936	2.32904
XLOC_070652	COPA;PEX19;NCSTN;DCAF8;PEA15;ATP1A4;CASQ1	chr3	9432263	9609333	3.18293	1.90247	7.46312	1.5678	2.5978	3.94531	0.712012	4.25993	3.74368
XLOC_070653	COPA;PEX19;NCSTN;DCAF8;PEA15;ATP1A4;CASQ1	chr3	9432263	9609333	2.64704	4.74774	4.13891	1.77847	3.50534	1.91716	3.09423	2.89612	4.15519
XLOC_070654	COPA;PEX19;NCSTN;DCAF8;PEA15;ATP1A4;CASQ1	chr3	9432263	9609333	0.0	2.27887	5.95758	8.92941	4.57895	3.84681	10.0252	3.57428	8.5372

XLOC_070655	COPA;PEX19;NCSTN;DCAF8;PEA15;ATP1A4;CASQ1	chr3	9432263	9609333	12.208	4.8545	6.34679	5.82201	6.51968	4.97347	8.02347	8.62582	6.6814
XLOC_070656	COPA;PEX19;NCSTN;DCAF8;PEA15;ATP1A4;CASQ1	chr3	9432263	9609333	4.85364	13.0224	0.0	7.38055	3.68443	5.41862	11.1026	7.88809	10.0466
XLOC_041953	COPS2	chr10	61319890	61383502	16.6164	20.056	19.7681	32.6548	32.2164	31.886	31.6451	34.6125	37.2314
XLOC_082036	COPS4	chr6	99625242	99626702	6.66322	4.98357	3.47579	0.547624	1.00103	0.692724	0.65838	0.278764	0.582718
XLOC_053261	COPS5;ARFGEF1;CSPP1	chr14	33217067	33370269	1.09806	0.624691	1.20664	2.27154	2.55299	2.75033	1.60504	1.38066	2.45735
XLOC_054166	COPS5;ARFGEF1;CSPP1	chr14	33217067	33370269	0.0	0.0	0.0	0.747908	0.523077	0.751602	0.405781	0.0	0.437709
XLOC_054167	COPS5;ARFGEF1;CSPP1	chr14	33217067	33370269	0.0	0.0	0.0	0.0	0.620366	0.0	0.676806	0.0	0.713162
XLOC_054168	COPS5;ARFGEF1;CSPP1	chr14	33217067	33370269	1.80152	0.0	0.0	0.645237	0.419405	0.92923	0.321192	0.17815	0.784167
XLOC_054169	COPS5;ARFGEF1;CSPP1	chr14	33217067	33370269	0.0	0.0	0.0	0.766295	0.0	0.871858	1.0983	0.0	0.370025
XLOC_054170	COPS5;ARFGEF1;CSPP1	chr14	33217067	33370269	0.0	0.0	0.0	0.0	0.0	0.0	0.947514	0.0	0.0
XLOC_076942	COPS7A;COPS7A	chr5	104079110	104114062	44.304	26.4201	30.7109	0.198734	0.149513	0.230728	0.261027	0.12752	0.166257
XLOC_066492	COPS7B	chr2	120316333	120353247	8.85405	9.94839	8.89079	11.3709	14.858	12.7581	12.4595	10.4575	14.6583
XLOC_066491	COPS7B;PDE6D	chr2	120302730	120314146	0.505935	0.209433	0.0	0.413203	0.595477	0.503327	0.391179	0.557507	0.542152
XLOC_068399	COPS7B;PDE6D	chr2	120302730	120314146	0.0	0.394204	0.0	0.118133	0.308177	0.272882	0.0	0.131167	0.344999
XLOC_089891	COQ3	chr9	51057388	51077027	2.33683	1.86388	1.2187	0.69822	0.487561	0.242604	1.06126	0.233989	0.612588
XLOC_060293	CORO1C	chr17	66494440	66519592	36.1814	39.5673	27.9437	11.1222	10.5574	11.6679	16.8541	15.2777	13.2537
XLOC_061842	CORO1C	chr17	66475247	66475461	0.0	0.0	0.0	2.13532	8.68786	4.70525	1.8694	1.07586	2.0165
XLOC_061843	CORO1C	chr17	66477959	66478536	1.72421	0.0	0.0	1.54399	2.00825	1.95751	0.615638	2.90126	1.6516
XLOC_061844	CORO1C	chr17	66479027	66479703	0.0	0.0	0.0	2.74909	2.49788	2.88535	1.00217	2.49531	1.94592
XLOC_061845	CORO1C	chr17	66479809	66481121	0.0	0.187402	0.0	3.53822	5.05467	3.45023	2.2254	1.69682	2.62901
XLOC_061846	CORO1C	chr17	66482296	66482775	0.0	0.0	0.0	1.81225	5.2191	3.47185	1.98927	2.2128	2.15102
XLOC_061847	CORO1C	chr17	66483777	66483920	0.0	0.0	0.0	71.9487	71.1748	21.7044	15.8533	6.13293	18.8412
XLOC_061848	CORO1C	chr17	66484236	66485059	0.0	0.0	0.0	2.23988	0.848218	1.23875	0.68707	0.433648	0.758878

XLOC_061849	CORO1C	chr17	66485711	66486969	0.0	0.196592	0.0	2.06207	2.11026	1.57044	0.71793	1.5817	1.83847
XLOC_061850	CORO1C	chr17	66494440	66519592	6.09532	21.8047	0.0	2.18462	1.83857	3.07999	1.01626	2.30611	1.57383
XLOC_061851	CORO1C	chr17	66520095	66521227	2.22652	3.55191	2.90303	0.0	0.0	0.0	0.0674569	0.0	0.129722
XLOC_062140	COTL1	chr18	10726287	10818864	45.5391	47.0253	47.9456	37.6581	37.6896	38.5602	44.1753	32.9444	41.1733
XLOC_062661	COTL1	chr18	10726287	10818864	31.5168	22.9804	30.8224	23.604	22.2663	23.2625	28.3926	19.49	24.9117
XLOC_062662	COTL1	chr18	10726287	10818864	24.4497	20.9012	25.2833	17.6173	17.5383	19.2142	19.6355	13.3023	18.1443
XLOC_062663	COTL1	chr18	10818944	10819384	7.68115	0.0	3.99844	0.916362	2.17253	1.57747	1.12566	0.501786	1.55607
XLOC_064233	COX10	chr19	32672485	32712659	30.6043	28.8701	33.4229	36.7494	43.4504	41.7997	52.9293	44.3589	55.8132
XLOC_064104	COX11;TOM1L1	chr19	5273897	5295922	87.2985	97.4701	86.8119	158.126	135.935	147.249	155.052	192.183	140.79
XLOC_089743	COX7A2	chr9	15006197	15045486	5.77633	6.37252	5.74696	5.09931	7.1078	4.59744	6.46842	5.90233	5.22706
XLOC_079749	COX7B2	chr6	66896578	67020371	24.6128	25.9319	19.0001	1.50901	1.52343	1.56067	1.69409	1.38542	2.08401
XLOC_079750	COX7B2	chr6	67073627	67074415	2.91943	0.436314	1.14108	0.392253	0.795103	0.301809	0.654657	0.144949	1.01789
XLOC_079751	COX7B2	chr6	67075965	67078340	2.78355	1.94237	0.0	0.0831563	0.290034	0.0962378	0.0	0.0927364	0.243098
XLOC_080143	COX7B2	chr6	66896578	67020371	3.46543	1.93577	3.64038	1.99281	3.28403	2.20921	1.38345	1.00503	1.48766
XLOC_080144	COX7B2	chr6	67034273	67038640	18.5808	13.8835	17.429	16.2578	16.5407	13.6488	19.7984	19.0469	16.9653
XLOC_081265	COX7B2	chr6	66896578	67020371	1.2678	3.41118	1.98251	0.227189	0.197609	0.13122	0.570606	0.378561	0.331754
XLOC_081266	COX7B2	chr6	66896578	67020371	2.01129	2.00614	2.36117	0.120252	0.315858	0.104693	0.153504	0.269995	0.0880171
XLOC_081267	COX7B2	chr6	66896578	67020371	2.36202	2.38553	2.77304	0.1324	0.0463825	0.18447	0.135305	0.208185	0.0775359
XLOC_081268	COX7B2	chr6	66896578	67020371	1.78032	2.92843	1.39254	0.319144	0.417508	0.0923505	0.0806973	0.178022	0.544264
XLOC_081269	COX7B2	chr6	66896578	67020371	1.92116	1.14796	6.00425	0.172036	0.148944	0.0	0.0	0.379405	0.0
XLOC_081270	COX7B2	chr6	66896578	67020371	6.46524	5.3135	3.78984	0.43429	0.125615	0.834654	0.57827	0.160193	0.140805
XLOC_081271	COX7B2	chr6	66896578	67020371	1.85227	0.553438	2.89469	0.165879	1.72417	0.764087	0.0	0.183053	0.644889
XLOC_081272	COX7B2	chr6	66896578	67020371	0.0	2.41357	4.20812	1.32605	1.04806	0.41763	0.604777	0.401423	0.352024
XLOC_081273	COX7B2	chr6	66896578	67020371	0.0	1.98304	3.70456	0.594299	1.85042	0.785943	0.428882	0.378629	0.330901
XLOC_081274	COX7B2	chr6	66896578	67020371	2.09333	1.46107	4.36719	0.750632	2.67688	1.23213	0.571274	0.559509	1.15882
XLOC_081275	COX7B2	chr6	66896578	67020371	1.15919	0.97133	1.63319	2.74469	2.38675	2.65662	2.489	1.75285	2.35467
XLOC_081276	COX7B2	chr6	66896578	67020371	1.73547	0.778557	2.71496	2.64431	2.84942	4.05163	3.46218	3.55847	3.56256
XLOC_081277	COX7B2	chr6	67020502	67022347	10.7145	8.97637	5.36626	0.65332	0.941566	0.267549	0.35264	0.129299	0.449976
XLOC_081278	COX7B2	chr6	67022408	67023306	0.976721	0.876249	2.29171	0.175064	0.0	0.101276	0.0	0.0975624	0.0
XLOC_081279	COX7B2	chr6	67033401	67034098	4.02196	1.20232	0.0	1.68141	1.56632	0.554793	1.08463	0.399959	0.818359
XLOC_081280	COX7B2	chr6	67041747	67042380	3.04224	3.63712	5.94501	0.272485	1.18306	0.943156	1.49914	0.754668	0.79535
XLOC_081281	COX7B2	chr6	67046425	67047412	0.871917	1.56461	2.72804	0.468889	1.22703	0.361872	0.790591	0.610442	1.06629

XLOC_081282	COX7B2	chr6	67048011	67049785	2.23703	1.20477	3.50116	0.200589	0.456287	0.372357	0.327111	0.0449823	0.665419
XLOC_081283	COX7B2	chr6	67053640	67054456	1.09836	1.31368	0.858926	0.0984202	0.0857156	0.341407	0.0991752	0.109549	0.383457
XLOC_081284	COX7B2	chr6	67055437	67056735	2.53658	2.08672	1.9846	0.341107	0.347717	0.13179	0.288769	0.0636105	0.221767
XLOC_081285	COX7B2	chr6	67112035	67112664	0.0	0.458492	1.19907	2.06095	2.26665	2.21919	1.64892	1.06533	2.40618
XLOC_081286	COX7B2	chr6	67113619	67114423	2.23749	0.0	0.0	2.0049	1.74585	1.0431	0.302935	0.780883	0.781092
XLOC_038508	CP	chr1	119909909	119913385	3.77669	0.80706	0.844329	5.6597	5.83665	6.00228	5.02078	6.77284	6.7004
XLOC_040162	CP	chr1	119908579	119908828	7.71762	0.0	0.0	2.08183	6.93558	3.88475	4.42707	2.165	9.94864
XLOC_040163	CP	chr1	119909191	119909769	1.72011	0.514013	1.34426	2.61856	2.67139	4.26088	1.6891	3.4053	2.54644
XLOC_040164	CP	chr1	119940231	119942664	18.4342	12.0786	22.6364	14.6791	14.8762	16.7112	14.7916	12.0663	12.9638
XLOC_074620	CPA1	chr4	94942868	94944219	16.3615	17.0405	25.1289	12.9301	18.944	14.9261	16.2341	14.168	18.9157
XLOC_074621	CPA1	chr4	94944422	94944767	7.70747	16.0931	18.0345	15.8516	16.4891	9.82271	16.2436	10.4204	13.6615
XLOC_073242	CPA1;CEP41	chr4	94944875	94979076	28.098	33.9678	48.8482	21.0097	27.4059	24.3324	26.161	21.64	28.818
XLOC_074622	CPA1;CEP41	chr4	94944875	94979076	0.0	10.395	21.7452	3.42764	1.06758	3.91491	4.82423	2.36465	3.61888
XLOC_038511	CPA3	chr1	120226523	120271007	4.33672	3.28763	1.58398	5.96215	3.58777	5.87103	4.4279	8.06692	3.41622
XLOC_073241	CPA5	chr4	94907793	94921068	3.55753	2.12828	0.556632	0.0637815	0.111398	0.0	0.129413	0.0	0.248751
XLOC_041825	CPLX2	chr10	5205051	5228398	4.36105	4.93483	5.37583	0.390926	0.29463	0.200696	0.0749501	0.0823611	0.166962
XLOC_053391	CPQ	chr14	69716971	69732607	15.8084	10.3159	26.0279	1.83892	3.4254	4.74751	5.7692	2.24775	8.75753
XLOC_053392	CPQ	chr14	69732798	69734679	0.0	1.08626	0.0	0.162766	0.780694	0.376781	0.0822798	0.090779	0.317235
XLOC_053393	CPQ	chr14	69767489	69782535	1.28291	4.6053	4.5168	14.8941	16.5804	18.3292	10.8062	10.6801	12.2253
XLOC_053688	CPQ	chr14	69244198	69503472	9.03028	5.84237	6.10902	11.6831	13.4453	10.7451	9.47286	8.83696	11.4851
XLOC_053689	CPQ	chr14	69696621	69705175	5.86312	5.76171	9.28525	11.2186	16.5241	11.5125	12.1782	13.0045	11.7889
XLOC_053690	CPQ	chr14	69705365	69707672	4.58629	7.94833	6.45757	10.4255	15.3416	13.5601	12.3722	11.2246	15.3901
XLOC_053691	CPQ	chr14	69716971	69732607	21.2092	27.7361	22.2406	19.6843	21.0148	16.0758	17.7948	21.9852	23.7874
XLOC_053692	CPQ	chr14	69784924	69815902	1.4913	4.92047	3.35985	5.27099	5.64044	5.29587	3.88603	5.86503	4.79873
XLOC_054761	CPQ	chr14	69244198	69503472	0.0	4.10195	10.724	9.25979	4.13529	4.85642	7.95798	3.88128	5.32399
XLOC_054762	CPQ	chr14	69609688	69611603	0.0	0.0	0.322038	0.0369006	0.0322924	0.0428199	0.0376336	0.0	0.0
XLOC_054763	CPQ	chr14	69693305	69693694	18.7428	6.52595	9.75136	8.38172	14.156	11.8298	8.70355	2.73695	22.7385
XLOC_054764	CPQ	chr14	69694882	69695500	17.2774	11.7357	6.13836	6.04896	8.42571	9.08677	6.04572	3.11513	14.5063
XLOC_054765	CPQ	chr14	69782621	69783326	0.0	1.57969	3.09852	6.27246	6.3805	7.79122	4.27667	5.25612	5.41478
XLOC_054766	CPQ	chr14	69784924	69815902	0.0	0.0	0.0	0.0	0.0	0.205194	0.0	0.0	0.0
XLOC_054767	CPQ	chr14	69784924	69815902	0.0	0.394204	0.0	0.118133	0.0	0.0	0.0	0.0	0.115
XLOC_046944	CPSF3	chr11	87978102	87979446	0.0	0.911754	0.953852	0.491835	0.811875	0.633496	0.444321	0.611628	0.426376

XLOC_044988	CPSF3;ITGB1BP1	chr11	87979517	88031813	5.98094	11.4721	7.39109	11.2184	13.5523	13.1101	12.9512	13.1575	6.06918
XLOC_046945	CPSF3;ITGB1BP1	chr11	87979517	88031813	18.8048	0.0	7.33907	4.24632	9.80214	5.66432	3.03912	7.83721	5.65233
XLOC_046946	CPSF3;ITGB1BP1	chr11	87979517	88031813	0.0	3.06361	8.00808	9.28335	3.04692	2.057	4.1181	2.83707	1.76088
XLOC_050852	CPXM1	chr13	52835092	52930958	2.36671	3.09287	3.96224	18.5052	23.5527	19.5903	21.1615	20.7537	31.8113
XLOC_058010	CR2	chr16	5245239	5256011	2.76105	3.94635	2.28456	13.9755	9.97629	12.5209	10.7975	15.1716	18.91
XLOC_058318	CR2	chr16	5245239	5256011	8.21286	9.80139	0.0	3.69695	6.13937	3.30423	6.70018	4.59387	7.05605
XLOC_057953	CRB1;MIR2284N	chr16	78301677	78377631	50.3647	56.1785	41.3394	7.03792	9.55071	7.38633	7.83247	8.96836	9.6327
XLOC_058180	CRB1;MIR2284N	chr16	78301677	78377631	27.3359	15.2977	32.4076	8.82133	8.42559	9.40844	12.5317	10.2969	11.1958
XLOC_059323	CRB1;MIR2284N	chr16	78301677	78377631	26.5274	23.4566	26.2933	4.17156	3.52133	4.12872	4.01927	4.90051	5.92855
XLOC_059324	CRB1;MIR2284N	chr16	78301677	78377631	1.59845	2.3887	0.0	0.715783	0.745476	0.165104	1.14409	0.950742	1.6711
XLOC_059325	CRB1;MIR2284N	chr16	78301677	78377631	2.11227	3.37135	3.67408	0.631483	0.774638	0.806801	0.839853	0.993664	1.19184
XLOC_059326	CRB1;MIR2284N	chr16	78301677	78377631	0.988739	2.36546	3.86659	0.531646	0.617842	1.23017	1.25241	0.691238	0.690652
XLOC_059327	CRB1;MIR2284N	chr16	78301677	78377631	3.4925	1.91555	2.73273	0.365307	0.273695	0.423533	0.795958	0.525832	0.6108
XLOC_059328	CRB1;MIR2284N	chr16	78301677	78377631	2.92216	1.74472	2.28128	0.784167	1.57353	0.598857	1.02126	1.71081	0.760185
XLOC_059329	CRB1;MIR2284N	chr16	78301677	78377631	2.17542	0.649949	6.79739	1.363	1.0099	2.46291	1.73343	1.07075	1.70215
XLOC_059330	CRB1;MIR2284N	chr16	78301677	78377631	4.78477	2.8601	11.2195	2.4283	1.85961	1.15321	3.28223	1.73926	2.3622
XLOC_059331	CRB1;MIR2284N	chr16	78301677	78377631	7.72248	4.79822	9.29587	3.24872	3.49104	4.44546	5.36001	2.74237	3.94766
XLOC_059332	CRB1;MIR2284N	chr16	78301677	78377631	5.7073	2.27646	8.93079	1.98979	1.78826	2.504	3.0032	1.65387	2.49488
XLOC_059333	CRB1;MIR2284N	chr16	78301677	78377631	5.10721	4.07444	5.32824	1.32281	1.82347	1.76972	2.17331	1.6521	2.18348
XLOC_059334	CRB1;MIR2284N	chr16	78301677	78377631	12.497	3.726	4.87097	5.60036	5.17942	4.41843	5.71956	8.26437	7.5277
XLOC_059335	CRB1;MIR2284N	chr16	78301677	78377631	8.80199	2.62754	6.87074	1.57468	2.70814	2.40497	2.56298	2.57627	2.03528
XLOC_059336	CRB1;MIR2284N	chr16	78301677	78377631	4.42705	3.6403	5.19309	2.08262	1.55459	2.63735	4.09666	3.53203	3.96039
XLOC_059337	CRB1;MIR2284N	chr16	78301677	78377631	10.2446	4.59804	6.78394	2.26132	2.22656	3.19837	3.31623	2.45776	2.24112

XLOC_082804	CRB3;DENND1C;SLC25A41	chr7	19228242	19272882	7.27131	7.12974	6.39631	9.00934	8.24501	8.71282	7.49479	11.6861	6.61657
XLOC_083431	CRB3;DENND1C;SLC25A41	chr7	19228242	19272882	25.1596	21.1887	26.1579	27.3782	25.1239	24.6204	27.7294	30.8727	20.4534
XLOC_084312	CRB3;DENND1C;SLC25A41	chr7	19228242	19272882	15.7529	16.4472	42.9955	14.1703	18.8642	18.2321	17.4016	27.225	14.8901
XLOC_084313	CRB3;DENND1C;SLC25A41	chr7	19228242	19272882	9.12317	10.3064	7.13504	9.72003	5.69965	8.19661	7.88388	11.9428	7.43411
XLOC_070235	CRCT1	chr3	18071907	18243321	1.42262	0.772788	0.306848	1.48888	1.45898	1.55213	1.64039	0.513718	1.28795
XLOC_064027	CRHR1	chr19	46396911	46488828	3.1419	3.0677	2.07109	2.74406	2.52265	3.10206	2.82573	3.63714	1.70928
XLOC_065237	CRHR1	chr19	46396911	46488828	0.0	1.79966	1.17665	1.07861	0.819628	0.77786	0.269813	1.04574	0.918305
XLOC_044675	CRIM1	chr11	18880344	19019513	3.36943	1.79257	2.24147	0.139658	0.218095	0.117006	0.178726	0.169902	0.388702
XLOC_045551	CRIM1	chr11	19020055	19020528	2.29264	0.684722	0.0	0.0	0.886094	0.0	0.202554	0.0	0.0
XLOC_054210	CRISPLD1	chr14	40456176	40456847	0.0	0.0	0.0	1.13556	0.657894	1.60226	0.379379	1.95937	1.35078
XLOC_054211	CRISPLD1	chr14	40456997	40457861	0.0	0.0	0.0	1.28436	0.159879	0.318358	0.370256	1.32859	0.3575
XLOC_062141	CRISPLD2	chr18	11046980	11086218	8.07739	4.92076	7.25631	0.20912	0.0940798	0.0570703	0.524988	0.409755	0.523284
XLOC_062359	CRISPLD2	chr18	11046980	11086218	0.743726	1.20628	0.0	0.0	0.0	0.0	0.0	0.0	0.0
XLOC_062664	CRISPLD2	chr18	11013823	11014298	2.2782	0.0	0.0	0.0	0.0	0.0	0.0	0.0	0.19799
XLOC_062665	CRISPLD2	chr18	11046980	11086218	1.07666	0.805259	0.842454	0.0	0.0	0.0559712	0.196458	0.270313	0.188323
XLOC_072574	CROT	chr4	33209297	33231962	1.66198	0.828687	0.433476	0.248348	0.738198	0.633543	0.0	0.111251	0.436036
XLOC_073103	CROT	chr4	33245179	33248764	84.3632	90.8321	110.755	37.9229	31.4885	35.5713	30.0549	34.5929	30.8157
XLOC_073939	CROT	chr4	33232922	33233274	0.0	0.0	0.0	0.332273	0.852551	0.379112	0.0	0.71874	0.64282
XLOC_073940	CROT	chr4	33241627	33242018	3.09709	2.77299	0.0	1.38503	2.37928	0.951115	1.34892	0.301598	0.536811
XLOC_073941	CROT	chr4	33243111	33243370	0.0	4.17827	5.46174	1.88679	1.57854	2.82558	7.51636	0.658243	4.21699
XLOC_073942	CROT	chr4	33243848	33244062	46.9634	0.0	0.0	4.27065	6.95029	0.0	1.8694	4.30343	12.099
XLOC_073943	CROT	chr4	33244347	33244726	0.0	8.76745	15.284	5.83909	5.76099	5.6757	4.82047	5.71135	4.24142
XLOC_073944	CROT	chr4	33244831	33245006	0.0	29.3846	38.4187	6.85259	17.633	14.5825	11.1449	2.15596	16.7656
XLOC_073945	CROT	chr4	33248829	33249390	1.79259	3.21381	1.40079	0.802555	0.417351	1.10961	0.319628	0.354567	0.780305
XLOC_083364	CRTC1	chr7	4427042	4445685	9.51354	10.01	9.28756	14.2879	12.6323	12.473	13.9844	14.8104	15.9001
XLOC_084049	CRTC1	chr7	4426192	4426489	15.4804	13.8447	28.1546	26.7806	18.7695	23.0166	18.7196	29.5163	27.5762
XLOC_076927	CSDA;T2R10C;TAS2R10	chr5	99217175	99377135	13.0201	13.261	11.2959	0.6055	0.986264	0.680737	0.599537	0.561219	0.911275

XLOC_069854	CSDE1;NRAS	chr3	28697887	28728979	0.474787	0.4973	1.4865	0.0425824	0.0	0.0	0.0	0.0	0.0207849
XLOC_064256	CSF3;PSMD3;GSDMA	chr19	40928925	40994253	1.74441	0.988762	1.36513	1.99802	1.41729	1.71133	2.90252	4.28021	2.83476
XLOC_080203	CSN2;STATH;CSN1S2;HSTN;ODAM	chr6	87180121	87335809	9.523	11.8527	9.623	1.28286	0.449359	0.310664	0.703361	0.289979	0.358764
XLOC_076465	CSNK1E;CSNK1E	chr5	110544823	110621332	0.0	0.295335	0.0	1.60984	1.39152	1.80289	1.25699	1.80946	1.11734
XLOC_083007	CSNK1G2	chr7	45872697	45884041	13.8739	13.2798	10.4789	17.9322	19.2829	18.5218	22.8088	22.7665	20.1881
XLOC_083646	CSNK1G2	chr7	45886866	45896696	1.14896	1.71766	2.69535	0.720644	0.896283	0.595041	0.725534	0.801677	1.30341
XLOC_065575	CSRNP3	chr2	30807866	30828565	5.02216	3.37235	4.49237	10.0013	9.23888	10.0708	8.67206	9.1972	10.5483
XLOC_066226	CSRNP3	chr2	30795619	30801705	0.0	0.181282	0.0	1.41253	0.712179	1.00767	0.993924	0.608067	0.31791
XLOC_066227	CSRNP3	chr2	30807866	30828565	4.98718	7.10422	6.10725	7.70874	5.36664	6.40341	8.2768	9.97686	6.70072
XLOC_067204	CSRNP3	chr2	30807866	30828565	4.17771	2.81079	3.26725	3.65012	2.93552	4.22178	2.07679	1.98054	2.46173
XLOC_067205	CSRNP3	chr2	30807866	30828565	0.0	4.07144	3.54881	8.54862	3.46172	4.16292	2.32403	4.80058	4.71397
XLOC_067206	CSRNP3	chr2	30807866	30828565	6.23786	3.73184	2.71123	4.0386	3.36448	3.81568	2.45909	4.02946	3.63496
XLOC_067207	CSRNP3	chr2	30807866	30828565	0.0	0.781045	4.08507	2.34049	3.0255	1.61116	0.689367	0.512334	2.4978
XLOC_050814	CSRP2BP	chr13	38645623	38659087	46.9822	46.9064	25.1483	131.484	131.054	121.053	138.429	144.667	147.467
XLOC_051665	CSRP2BP	chr13	38640748	38642405	0.482316	0.432912	0.377421	1.72986	0.945678	1.25414	0.925064	0.775542	1.05478
XLOC_051666	CSRP2BP	chr13	38643923	38644287	3.50084	0.0	0.0	1.56532	0.0	0.715159	3.63461	1.3575	1.21204
XLOC_051667	CSRP2BP	chr13	38644347	38645165	1.09503	0.654851	1.71266	2.06057	1.62371	2.2692	1.08769	0.764552	1.33805
XLOC_062898	CTCF	chr18	35255714	35256516	0.0	0.335546	0.0	0.201111	0.612927	0.581288	0.20257	0.223792	0.489691
XLOC_062899	CTCF	chr18	35256696	35257975	1.93467	1.35039	0.50455	0.578137	0.606132	0.737074	0.469765	0.646797	1.01482
XLOC_062900	CTCF	chr18	35264286	35264990	0.0	0.0	2.06946	1.30421	0.206195	0.821617	0.476034	0.263279	0.57709
XLOC_089981	CTGF;ENPP1	chr9	70798069	70934853	0.0	0.0	0.865447	0.40461	0.178735	0.341418	0.993385	0.547326	0.94811
XLOC_091230	CTGF;ENPP1	chr9	70798069	70934853	11.6052	18.5945	15.5622	28.4568	22.4315	25.4655	28.6597	37.2453	23.9714
XLOC_070317	CTH;ANKRD13C	chr3	75071729	75165002	0.0	0.151468	0.0	1.91154	1.36887	1.60745	1.04352	1.93189	1.44232
XLOC_083025	CTNNA1	chr7	51862024	51864125	2.44556	2.00809	0.93039	5.14536	6.99958	6.07354	6.08237	4.12785	5.70264
XLOC_084903	CTNNA1	chr7	51855137	51855802	0.0	1.70404	0.0	0.255327	0.443723	0.147367	0.0	0.141581	0.49697
XLOC_084904	CTNNA1	chr7	51855948	51856293	3.85374	3.44851	0.0	2.0676	1.17779	1.57163	0.331501	0.0	1.99924
XLOC_084905	CTNNA1	chr7	51856431	51857513	3.12926	2.57391	1.22396	0.771362	0.612072	0.974596	0.781578	0.469983	0.273436
XLOC_084906	CTNNA1	chr7	51858238	51859655	2.29691	2.06145	2.2465	0.926689	0.495023	1.07444	0.99475	0.461142	0.703
XLOC_084907	CTNNA1	chr7	51859829	51860070	8.39128	5.00769	0.0	8.31383	0.626883	4.21877	0.683526	1.56292	0.0

XLOC_084908	CTNNA1	chr7	51860295	51860888	3.3217	7.44503	7.78819	4.61081	2.96768	6.00194	4.15592	2.96133	2.31459
XLOC_084909	CTNNA1	chr7	51861022	51861947	3.76943	4.22725	5.15938	8.52999	4.49166	6.74363	7.08279	9.13446	4.93773
XLOC_084910	CTNNA1	chr7	51864489	51864846	0.0	1.0809	0.0	0.0	0.831832	1.84917	3.44118	0.35076	0.940439
XLOC_083026	CTNNA1;SIL1	chr7	51864947	51995130	2.61246	2.95336	2.2719	7.90239	8.88078	11.2097	9.46488	7.30696	9.55416
XLOC_084911	CTNNA1;SIL1	chr7	51864947	51995130	2.08651	0.468178	2.04083	0.888619	0.940635	0.976336	1.04733	0.576362	0.41061
XLOC_084912	CTNNA1;SIL1	chr7	51864947	51995130	0.0	0.0	2.33262	0.178189	1.0871	0.412299	0.089941	0.496423	0.694434
XLOC_084913	CTNNA1;SIL1	chr7	51864947	51995130	0.0	0.318279	0.832409	0.381526	0.249267	0.66185	0.865531	0.424829	1.11495
XLOC_044086	CTNNA2	chr11	54866291	54870497	148.365	144.152	112.001	289.33	286.648	298.63	296.046	292.247	312.696
XLOC_044087	CTNNA2	chr11	54902680	54963052	1.10388	0.126942	0.843318	1.68638	1.14577	1.28304	0.990493	0.796175	0.978941
XLOC_044088	CTNNA2	chr11	55087365	55110181	66.3102	62.9947	68.6571	27.6242	39.9085	32.8881	27.7085	19.4171	45.2784
XLOC_044089	CTNNA2	chr11	55204466	55233485	2.02348	2.68417	1.90544	1.30047	2.20704	1.87071	1.857	1.76556	1.48962
XLOC_044090	CTNNA2	chr11	55463339	55501019	75.2834	72.8227	70.49	272.011	227.633	247.81	305.649	357.201	265.93
XLOC_044771	CTNNA2	chr11	54717419	54787733	21.3103	27.0113	26.6965	28.9765	27.9467	26.0801	31.8967	31.4217	29.3913
XLOC_044772	CTNNA2	chr11	54840656	54860594	1.27218	0.380293	0.0	2.16855	3.26213	3.76118	1.58923	1.77295	1.82419
XLOC_044773	CTNNA2	chr11	54902680	54963052	16.8108	14.8146	16.2274	9.46162	9.74399	9.59325	14.1507	12.5581	11.8309
XLOC_044774	CTNNA2	chr11	54979027	55021559	74.9597	55.5254	48.5024	82.7992	77.2747	88.0801	61.4726	93.3509	84.8516
XLOC_044775	CTNNA2	chr11	55049941	55078122	2.75916	3.30138	2.15862	0.556526	0.702109	0.358261	0.815838	0.345725	0.602945
XLOC_044776	CTNNA2	chr11	55394426	55410092	417.365	340.457	371.526	126.684	136.664	129.058	149.68	175.973	169.387
XLOC_044777	CTNNA2	chr11	55446599	55461364	36.8979	27.3553	24.8141	144.699	90.0025	120.794	82.9486	108.637	88.6757
XLOC_044778	CTNNA2	chr11	55463339	55501019	5.89793	3.23507	6.15359	11.5462	11.6789	11.8081	14.362	13.7793	9.8453
XLOC_046114	CTNNA2	chr11	54871010	54871385	0.0	2.97594	0.0	1.1892	1.78472	0.679753	1.73079	2.26029	1.72735
XLOC_046115	CTNNA2	chr11	54872529	54872856	0.0	2.54049	3.32141	1.90454	1.62283	0.0	1.09225	0.819214	0.367887
XLOC_046116	CTNNA2	chr11	54902680	54963052	2.65675	0.794194	1.03864	0.832997	1.34514	1.09948	1.79153	1.18904	0.5792
XLOC_046117	CTNNA2	chr11	54902680	54963052	1.39829	0.418254	3.82873	0.0626725	0.492617	0.0726198	0.763144	0.840872	0.916626
XLOC_046118	CTNNA2	chr11	54973547	54974300	0.0	0.0	0.0	0.21764	0.662874	0.377257	0.109434	0.120968	1.05962
XLOC_046119	CTNNA2	chr11	55022350	55023819	1.10292	0.989877	1.72598	0.395541	0.648384	0.401326	0.452706	0.664472	0.434045
XLOC_046120	CTNNA2	chr11	55029486	55029851	3.48403	0.0	2.71815	0.623123	0.266896	0.711789	1.20606	3.37807	0.60314
XLOC_046121	CTNNA2	chr11	55032083	55032711	0.0	0.459465	6.00809	1.65226	0.478192	0.794237	1.10155	0.762538	0.535837
XLOC_046122	CTNNA2	chr11	55110497	55111212	27.2446	32.5792	46.6595	14.9934	24.3613	16.782	17.9743	11.8756	22.5171
XLOC_046123	CTNNA2	chr11	55111567	55111812	0.0	2.39895	0.0	1.44726	0.0	0.0	0.0	0.0	0.690931
XLOC_046124	CTNNA2	chr11	55112325	55112600	0.0	0.0	0.0	0.0	1.83857	0.0	0.0	0.0	0.0

XLOC_046125	CTNNA2	chr11	55112683	55113017	0.0	1.22043	0.0	0.365922	0.312155	0.0	0.0	0.0	0.0
XLOC_046126	CTNNA2	chr11	55117993	55118796	85.1565	69.3505	58.7058	148.191	132.539	142.661	148.663	170.828	183.546
XLOC_046127	CTNNA2	chr11	55233629	55236473	0.538411	1.45009	1.89635	1.08646	1.41703	1.09345	1.80201	1.30203	1.0841
XLOC_046128	CTNNA2	chr11	55411139	55411463	0.0	5.17135	3.38048	1.55083	0.330189	1.32302	0.740287	1.24977	1.12312
XLOC_046129	CTNNA2	chr11	55411606	55412595	4.3491	2.86157	5.44297	0.545722	0.340027	0.180503	0.630976	1.13098	0.531866
XLOC_046130	CTNNA2	chr11	55412709	55413299	5.01712	0.999546	2.61404	0.299532	1.0393	1.72661	0.298848	0.165636	0.291318
XLOC_046131	CTNNA2	chr11	55414066	55414501	0.0	1.55648	0.0	0.466415	0.200988	0.535149	0.457999	0.765818	0.226262
XLOC_046132	CTNNA2	chr11	55414822	55415648	0.0	3.23511	5.92264	0.290848	0.675556	0.560557	0.684048	0.755523	0.566614
XLOC_046133	CTNNA2	chr11	55417693	55418157	0.0	2.11435	0.0	0.63358	0.364652	0.727864	1.45775	0.0	0.615109
XLOC_046134	CTNNA2	chr11	55418247	55419769	2.65019	2.53719	4.56217	0.522754	0.789522	0.606239	0.773857	0.585595	0.649005
XLOC_046135	CTNNA2	chr11	55463339	55501019	0.0	0.0	0.0	1.0738	0.741325	0.986474	1.05787	3.53321	0.625222
XLOC_046136	CTNNA2	chr11	55463339	55501019	7.3564	9.87549	2.86923	18.4195	14.0693	17.2661	24.115	23.488	24.1825
XLOC_046137	CTNNA2	chr11	55501070	55501232	34.6592	0.0	0.0	60.0448	30.0139	13.9341	57.5862	79.0817	60.2277
XLOC_046138	CTNNA2	chr11	55501341	55501610	0.0	3.82148	0.0	25.8576	24.13	23.9446	30.8762	30.8424	35.2907
XLOC_046139	CTNNA2	chr11	55501874	55502605	2.52233	7.16375	3.9443	16.0445	13.8592	11.618	16.6896	25.7351	21.0116
XLOC_046140	CTNNA2	chr11	55502762	55503388	3.0877	2.76854	2.41348	17.4226	12.2453	14.0377	20.1867	17.9181	19.7756
XLOC_046141	CTNNA2	chr11	55503529	55503697	29.0943	17.6964	23.1393	69.2421	50.5617	29.1901	70.7638	79.6263	68.05
XLOC_046142	CTNNA2	chr11	55503810	55506264	2.83432	3.0156	2.2183	9.46122	9.74453	9.11545	10.2715	13.0981	11.5223
XLOC_046143	CTNNA2	chr11	55506442	55507502	0.0	0.958767	0.626889	0.933815	1.1911	1.16461	1.16414	0.320885	1.12033
XLOC_046144	CTNNA2	chr11	55507638	55508609	0.0	0.265894	1.39083	1.35462	1.59849	1.01461	0.402943	0.889025	1.39782
XLOC_046145	CTNNA2	chr11	55508661	55509523	0.0	0.306995	0.802899	0.736002	1.68346	0.851349	0.278466	0.307457	0.896277
XLOC_058139	CTNNBIP1	chr16	44484772	44558886	2.69863	2.23313	1.9495	0.401899	0.249572	0.46416	0.30614	0.128065	0.212955
XLOC_059040	CTNNBIP1	chr16	44484772	44558886	0.0	0.226169	0.0	0.0	0.0	0.0	0.0	0.0757346	0.0
XLOC_050928	CTNNBL1	chr13	67315573	67342683	4.21033	2.28038	3.28314	10.2744	11.4405	10.0554	9.21361	13.7134	13.9679
XLOC_050929	CTNNBL1	chr13	67315573	67342683	0.0	4.92495	0.0	3.9439	4.16525	5.01824	9.24924	6.80434	3.79511
XLOC_050930	CTNNBL1	chr13	67365365	67389314	1.11358	1.33186	0.870816	0.698477	1.12961	1.03832	1.20626	1.6657	1.45781
XLOC_050931	CTNNBL1	chr13	67408670	67413458	0.0	2.88742	0.472942	34.1699	18.8368	22.522	30.2538	38.7282	27.415
XLOC_050932	CTNNBL1	chr13	67418143	67422113	0.189696	0.810651	0.593963	5.87238	3.46368	3.54937	5.41673	9.28941	4.20995
XLOC_052381	CTNNBL1	chr13	67268478	67269105	3.08112	0.0	0.0	0.827885	0.598998	0.318365	0.551915	0.916959	0.402729
XLOC_052382	CTNNBL1	chr13	67269788	67270422	1.51793	0.0	0.0	0.0	0.0	0.156866	0.0	0.0	0.0
XLOC_052383	CTNNBL1	chr13	67314982	67315245	6.75526	4.02823	5.26567	6.06068	5.58661	8.85941	6.70739	7.62945	8.13373
XLOC_052384	CTNNBL1	chr13	67315573	67342683	1.53078	0.0	2.39308	0.68553	2.14287	1.58181	1.64552	0.607496	1.06716
XLOC_052385	CTNNBL1	chr13	67315573	67342683	0.0	0.438968	1.14802	0.263092	0.0	0.0	0.395123	0.583265	0.128007

XLOC_052386	CTNNBL1	chr13	67315573	67342683	7.9581	9.49598	6.20616	22.1956	20.2449	15.2151	16.9182	25.2632	19.8304
XLOC_052387	CTNNBL1	chr13	67364327	67364913	0.0	0.0	1.3192	0.755808	0.786654	1.21979	0.452328	1.17006	0.294021
XLOC_052388	CTNNBL1	chr13	67389700	67389860	0.0	22.5871	0.0	7.13366	5.32116	3.71247	8.34505	3.23253	28.8958
XLOC_052389	CTNNBL1	chr13	67397560	67398583	0.0	0.0	0.0	0.449401	0.71887	0.0867243	0.0758214	0.167231	0.43803
XLOC_052390	CTNNBL1	chr13	67398820	67399481	1.43652	0.42939	0.0	0.772058	0.782614	0.594112	0.386717	0.142686	1.25224
XLOC_052391	CTNNBL1	chr13	67399580	67400354	0.0	0.0	0.0	0.525588	1.00651	1.0935	0.317359	0.233812	1.02368
XLOC_052392	CTNNBL1	chr13	67401970	67402415	0.0	0.0	0.0	1.3507	2.71791	2.84251	0.442786	0.493322	2.62157
XLOC_052393	CTNNBL1	chr13	67403180	67403463	0.0	0.0	0.0	2.0488	2.59285	3.47235	1.43709	1.62799	7.38839
XLOC_052394	CTNNBL1	chr13	67403981	67404264	0.0	1.7051	0.0	2.561	0.432142	0.0	0.47903	0.542665	4.92559
XLOC_052395	CTNNBL1	chr13	67404764	67405072	0.0	0.0	0.0	1.71382	3.6376	5.34899	0.812403	0.458207	1.65269
XLOC_052396	CTNNBL1	chr13	67405838	67406375	0.0	0.0	0.0	10.2386	7.83247	9.62821	9.83521	21.4591	9.62009
XLOC_052397	CTNNBL1	chr13	67406798	67406910	0.0	0.0	0.0	992.412	403.601	600.994	1269.01	884.219	532.78
XLOC_052398	CTNNBL1	chr13	67407436	67407558	0.0	0.0	0.0	92.7223	218.489	175.275	165.484	190.484	307.155
XLOC_052399	CTNNBL1	chr13	67408085	67408209	0.0	76.1976	200.045	1075.8	598.58	950.693	1159.25	1196.48	1087.66
XLOC_052400	CTNNBL1	chr13	67408670	67413458	0.0	0.0	0.0	5.92096	5.35531	2.38221	4.68763	7.14357	5.05104
XLOC_052401	CTNNBL1	chr13	67408670	67413458	0.388728	0.232628	0.0	5.57735	3.63071	4.32869	6.04608	6.45334	4.35405
XLOC_052402	CTNNBL1	chr13	67413600	67413814	0.0	0.0	0.0	12.8119	6.0815	8.23418	19.6287	16.1379	6.04951
XLOC_052403	CTNNBL1	chr13	67414415	67415661	0.0	0.795034	1.03967	8.81567	5.35953	5.3153	8.89041	11.1263	5.86649
XLOC_052404	CTNNBL1	chr13	67416068	67416300	0.0	0.0	7.25168	11.7448	4.1525	3.73215	6.76285	5.16592	5.58572
XLOC_052405	CTNNBL1	chr13	67417684	67418045	0.0	2.1196	0.0	7.62372	7.07169	5.44097	6.75554	15.4873	7.68525
XLOC_052406	CTNNBL1	chr13	67423044	67424148	0.0	0.228557	0.0	0.821941	0.478345	1.42807	1.24964	0.535687	0.667744
XLOC_052407	CTNNBL1	chr13	67424968	67425645	0.0	0.416182	0.0	0.498874	2.16793	1.58392	1.25036	0.276731	2.18496
XLOC_052408	CTNNBL1	chr13	67426244	67427313	0.793473	0.237327	0.0	1.28022	0.558671	0.576583	0.648459	0.23831	0.693311
XLOC_055576	CTNND1	chr15	82275370	82294540	0.636127	1.14914	1.11445	4.88689	4.0329	4.14941	3.81278	5.8446	4.79948
XLOC_055577	CTNND1	chr15	82298960	82314715	3.52696	2.3801	0.0	4.66004	1.77651	2.74871	2.24742	3.46852	2.36236
XLOC_057201	CTNND1	chr15	82275370	82294540	1.18862	0.355382	0.0	1.3845	0.463461	1.10778	0.214294	0.355271	0.207416
XLOC_057202	CTNND1	chr15	82275370	82294540	1.28355	0.383729	0.0	3.44979	1.90036	2.78959	2.07904	3.06553	2.23904
XLOC_070466	CTPS;SCMH1	chr3	105729898	105801308	18.0809	12.4218	16.4612	5.55389	6.5115	6.24899	5.78786	4.16681	6.65021
XLOC_072002	CTPS;SCMH1	chr3	105729898	105801308	6.49972	2.90945	0.0	0.87197	0.997544	1.32958	1.69415	0.315933	1.40754
XLOC_072003	CTPS;SCMH1	chr3	105729898	105801308	5.55295	6.63097	15.1725	0.49679	1.06909	2.27792	2.43206	0.542763	1.44527
XLOC_072004	CTPS;SCMH1	chr3	105729898	105801308	3.38768	0.675289	0.883065	0.0	0.176212	0.116979	0.509527	0.112588	0.197096

XLOC_072005	CTPS;SCMH1	chr3	105729898	105801308	4.11173	0.983829	4.5029	0.147425	0.257278	0.0	0.0746357	0.246896	0.287386
XLOC_072006	CTPS;SCMH1	chr3	105729898	105801308	2.8829	3.66492	5.6384	0.258435	0.282086	0.0	0.52427	0.144438	0.377949
XLOC_072007	CTPS;SCMH1	chr3	105729898	105801308	3.89636	2.33039	3.04741	0.523786	0.0760988	0.404019	0.176317	0.0	0.340228
XLOC_072008	CTPS;SCMH1	chr3	105729898	105801308	2.00808	1.80207	1.57107	0.300036	0.209673	0.139078	0.0	0.268457	0.234053
XLOC_072009	CTPS;SCMH1	chr3	105729898	105801308	9.4091	7.72747	3.67426	0.421036	0.726988	1.20922	0.415211	0.0	0.0
XLOC_072010	CTPS;SCMH1	chr3	105729898	105801308	0.0	1.1937	3.12129	0.0	0.305497	0.815479	0.686923	0.0	0.0
XLOC_072011	CTPS;SCMH1	chr3	105729898	105801308	1.43715	1.93447	2.81081	0.128836	0.506246	0.373144	0.588069	0.216016	0.439629
XLOC_072012	CTPS;SCMH1	chr3	105729898	105801308	3.3822	2.81119	3.52925	0.3033	0.501479	0.743143	0.41269	0.453799	0.263091
XLOC_072013	CTPS;SCMH1	chr3	105729898	105801308	0.0	0.0	3.70985	0.425703	0.361489	0.966351	0.40376	0.455375	0.0
XLOC_072014	CTPS;SCMH1	chr3	105729898	105801308	5.21775	3.64222	4.98982	0.675715	0.681497	0.723156	0.528537	0.698321	0.405571
XLOC_092261	CTPS2	chrX	134059252	134086959	29.7948	32.3176	35.7891	54.3288	56.8963	54.7586	62.5376	53.7516	64.7655
XLOC_055757	CTR9	chr15	42468204	42523590	0.0	0.467401	0.0	0.0	0.0	0.323137	0.0	0.0	0.136264
XLOC_086537	CTSL2	chr8	84957573	84997409	77.2884	67.7397	61.0121	168.834	189.92	186.253	186.494	155.474	164.101
XLOC_086538	CTSL2	chr8	84957573	84997409	4.97151	5.66003	6.50307	39.6424	33.489	36.1632	36.8903	31.4154	29.1907
XLOC_086987	CTSL2	chr8	84957573	84997409	101.019	109.361	89.3836	132.877	169.594	142.04	143.854	120.01	117.873
XLOC_088461	CTSL2	chr8	84957573	84997409	3.27928	3.91423	2.55882	5.86577	5.03167	5.36556	3.70199	4.46152	2.55633
XLOC_088462	CTSL2	chr8	84957573	84997409	0.982032	1.76201	2.30414	1.14418	1.30405	1.42552	0.710894	0.392346	0.857499
XLOC_088463	CTSL2	chr8	84957573	84997409	1.71286	0.512282	1.33981	1.84235	1.20538	2.13287	1.0098	1.11375	1.42151
XLOC_088464	CTSL2	chr8	84957573	84997409	0.564813	0.337943	0.0	1.46858	1.06242	0.7633	0.669573	0.793856	0.642117
XLOC_088465	CTSL2	chr8	84957573	84997409	6.94192	6.20966	0.0	4.98515	2.60743	4.20004	4.01277	6.52488	6.56637
XLOC_088466	CTSL2	chr8	84957573	84997409	0.455295	0.54489	1.06885	2.2454	1.46433	1.37352	0.499242	1.23581	0.318662
XLOC_088467	CTSL2	chr8	84957573	84997409	1.19451	0.357135	0.0	2.14061	1.67665	1.97905	0.861329	1.18999	0.521131
XLOC_069862	CTTNBP2NL	chr3	30995948	31052690	49.1068	53.7462	48.1531	81.0751	73.6374	81.0369	88.6224	91.8298	97.317
XLOC_041961	CTXN2	chr10	62382911	62422455	0.82191	0.24398	0.321647	0.0183881	0.0161152	0.0213633	0.0	0.0206776	0.0719794
XLOC_082862	CTXN3	chr7	27499148	27520146	0.700184	1.0735	1.37022	9.04685	11.0309	9.22772	7.62492	8.85765	9.90861
XLOC_050494	CUBN	chr13	31626176	31631439	0.0	0.0	0.0	0.0604534	0.0544734	0.0	0.0	0.0	0.0
XLOC_050495	CUBN	chr13	31797688	31802372	0.0	0.0	0.0	0.0	0.0342544	0.0	0.0	0.0	0.0

XLOC_050797	CUBN	chr13	31673023	31681479	0.0	0.0	0.0	0.0	0.0	0.0	2.81337	3.31969	1.72172
XLOC_051529	CUBN	chr13	31559310	31559744	5.23183	8.5915	2.04247	0.0	0.2017	0.0	0.459578	0.0	0.0
XLOC_051530	CUBN	chr13	31559915	31560665	1.22072	5.83939	9.54491	0.218741	0.0951711	0.126384	0.0	0.121571	0.212993
XLOC_051531	CUBN	chr13	31671891	31672331	0.0	0.0	0.0	0.229091	0.0	0.0	2.25132	1.00357	0.666885
XLOC_051532	CUBN	chr13	31673023	31681479	0.0	0.0	0.0	0.0	0.0	0.0	0.114905	0.63532	1.22498
XLOC_051533	CUBN	chr13	31682038	31682942	0.0	0.0	0.0	0.0	0.0	0.0	4.03386	3.77471	3.80711
XLOC_051534	CUBN	chr13	31684130	31684764	0.0	0.0	0.0	0.0	0.0	0.0	2.72016	0.903736	2.11651
XLOC_051535	CUBN	chr13	31807192	31811101	1.34946	0.461555	1.509	0.0691632	0.0606194	0.0803493	0.0708102	0.0388867	0.101289
XLOC_051536	CUBN	chr13	31814325	31814960	0.0	0.0	1.18403	0.135672	0.0	0.15654	0.0	0.0	0.0
XLOC_063899	CUEDC1	chr19	8920513	8952509	55.4448	49.2349	36.363	75.5044	59.8465	66.6075	95.0172	105.137	66.4123
XLOC_063900	CUEDC1	chr19	8953743	8978796	30.6375	34.0401	32.1352	105.289	85.9596	97.1647	108.288	123.685	90.59
XLOC_064541	CUEDC1	chr19	8918369	8918710	3.93717	1.17433	0.0	0.352062	0.901954	0.401249	0.0	0.0	0.340341
XLOC_064542	CUEDC1	chr19	8919254	8919617	0.0	1.04955	0.0	0.314579	0.0	0.35928	1.21707	0.0	0.304462
XLOC_064543	CUEDC1	chr19	8953743	8978796	4.33553	1.72784	1.12969	4.14229	4.94851	3.43646	3.76008	8.18161	4.78702
XLOC_064544	CUEDC1	chr19	8953743	8978796	0.0	4.23398	22.1342	50.4327	47.8249	50.8754	61.123	64.1537	38.8268
XLOC_064545	CUEDC1	chr19	8953743	8978796	10.4639	8.5915	4.08494	35.5754	28.4398	29.538	35.1577	41.7563	30.2009
XLOC_064546	CUEDC1	chr19	8953743	8978796	4.84659	6.51263	0.0	18.2146	17.7764	19.1752	24.3491	31.6331	21.0486
XLOC_064547	CUEDC1	chr19	8953743	8978796	24.7449	3.16395	11.031	34.456	27.6113	29.9628	33.9285	42.4778	31.8169
XLOC_064548	CUEDC1	chr19	8953743	8978796	5.42828	8.10265	6.35658	33.5074	30.3205	23.3887	34.9851	40.8831	37.9172
XLOC_064549	CUEDC1	chr19	8953743	8978796	15.4359	13.8118	6.01795	23.5941	23.697	18.6469	29.0921	23.0964	23.8774
XLOC_063898	CUEDC1;MRPS23	chr19	8880024	8916822	47.6499	44.6569	40.6779	59.6293	55.8484	57.1435	59.2314	67.6661	59.5488
XLOC_064163	CUEDC1;MRPS23	chr19	8880024	8916822	7.83123	6.12234	7.87385	27.4094	18.0169	22.7653	20.5948	33.0391	24.3504
XLOC_064164	CUEDC1;MRPS23	chr19	8880024	8916822	2.67812	1.18973	1.0799	1.90378	1.28038	1.15798	1.3168	1.32543	1.51369
XLOC_064538	CUEDC1;MRPS23	chr19	8880024	8916822	0.0	0.0	0.0	0.981761	2.24148	0.374436	1.57921	1.06246	0.633257
XLOC_064539	CUEDC1;MRPS23	chr19	8880024	8916822	0.0	1.43004	1.24685	3.14251	4.3397	2.30683	3.56773	6.95703	3.89069
XLOC_064540	CUEDC1;MRPS23	chr19	8880024	8916822	0.0	2.83774	0.0	13.1955	6.86976	10.6311	11.3051	18.2147	13.1366
XLOC_064215	CUEDC2;ALOX12	chr19	27386042	27429843	37.8469	36.1494	34.3833	90.9046	75.8449	97.447	102.089	129.077	90.7027
XLOC_064833	CUEDC2;ALOX12	chr19	27386042	27429843	0.0	0.0	0.0	0.0	0.766406	2.0501	0.0	0.964524	1.74279
XLOC_065790	CUL3	chr2	113440745	113525502	5.0564	2.83701	4.55064	1.44654	1.61334	1.53068	1.34682	0.92939	1.8599

XLOC_059870	CUX2	chr17	57062140	57070709	0.0	0.35709	0.644831	6.07015	6.01177	6.83967	2.42118	4.32325	4.02773
XLOC_059871	CUX2	chr17	57105324	57107695	4.35471	0.434176	0.528572	1.10601	0.635161	0.829102	1.18776	2.09981	1.41771
XLOC_059872	CUX2	chr17	57249436	57263395	25.8355	15.9781	13.9826	47.5135	38.811	43.4966	47.9857	65.1579	33.5269
XLOC_060271	CUX2	chr17	57062140	57070709	1.8371	0.0	1.367	1.96823	2.8157	2.4796	1.90475	1.97902	2.52678
XLOC_060272	CUX2	chr17	57079868	57087681	0.0	0.209636	0.54832	0.125648	0.275027	0.474048	0.0	0.168049	0.398555
XLOC_060273	CUX2	chr17	57203969	57223166	4.40922	0.628356	1.47912	0.828589	0.396079	0.459382	0.404757	0.0	0.533167
XLOC_060274	CUX2	chr17	57223233	57228084	5.7514	1.20442	0.450013	0.56721	0.180293	0.358713	0.681689	0.115468	0.452646
XLOC_060275	CUX2	chr17	57234732	57248164	7.41408	8.7737	9.80926	7.03988	7.20764	7.51994	6.64714	6.41348	8.25428
XLOC_061672	CUX2	chr17	57263457	57263671	0.0	0.0	0.0	1.06766	1.73757	1.17631	7.4776	10.7586	0.0
XLOC_064824	CXCL16	chr19	27250072	27250340	0.0	0.0	0.0	0.579637	0.973285	0.0	1.07321	0.60972	1.11228
XLOC_064825	CXCL16	chr19	27250519	27251164	5.93469	0.886928	1.15978	0.797361	1.38514	0.460075	1.06417	0.0	1.42244
XLOC_064826	CXCL16	chr19	27251380	27253271	1.66883	1.12349	0.326495	0.673402	1.6369	0.520935	0.915613	0.0839237	0.949115
XLOC_080221	CXCL2;PF4;CXCL2;CXCL3;GRO1;MTHFD2L	chr6	90662691	90988309	8.93116	9.51911	9.39154	5.53311	5.60464	5.33718	4.08118	5.19839	4.15876
XLOC_081616	CXCL2;PF4;CXCL2;CXCL3;GRO1;MTHFD2L	chr6	90662691	90988309	1.01085	0.906841	0.790568	1.08705	1.26307	0.419173	0.548529	0.403721	0.882548
XLOC_081617	CXCL2;PF4;CXCL2;CXCL3;GRO1;MTHFD2L	chr6	90662691	90988309	0.688622	1.54543	2.42522	1.79086	1.62203	2.11471	1.29263	1.17848	1.92859
XLOC_081618	CXCL2;PF4;CXCL2;CXCL3;GRO1;MTHFD2L	chr6	90662691	90988309	1.64949	0.0	0.0	0.590886	0.384475	0.170326	0.294883	0.326836	0.143678
XLOC_081619	CXCL2;PF4;CXCL2;CXCL3;GRO1;MTHFD2L	chr6	90662691	90988309	1.32591	1.18911	0.0	0.237568	0.103285	1.09748	0.119222	0.131881	0.346886
XLOC_081620	CXCL2;PF4;CXCL2;CXCL3;GRO1;MTHFD2L	chr6	90662691	90988309	0.0	0.0	0.0	0.0	0.224785	0.0	0.255318	0.285145	0.0
XLOC_081621	CXCL2;PF4;CXCL2;CXCL3;GRO1;MTHFD2L	chr6	90662691	90988309	2.27104	1.35658	1.7738	0.406516	0.0	0.233612	0.0	1.11654	0.986869

XLOC_081622	CXCL2;PF4;CXCL2;CXCL3;GRO1;MTHFD2L	chr6	90662691	90988309	0.0	0.0	0.0	0.535734	0.0	0.0	0.261325	0.583941	0.259617
XLOC_081623	CXCL2;PF4;CXCL2;CXCL3;GRO1;MTHFD2L	chr6	90662691	90988309	0.0	0.0	0.0	0.347695	0.903102	0.40029	0.0	0.383391	0.168959
XLOC_081624	CXCL2;PF4;CXCL2;CXCL3;GRO1;MTHFD2L	chr6	90662691	90988309	0.760115	0.0	0.594629	0.476951	0.178443	0.394614	0.13813	0.152259	0.464971
XLOC_081625	CXCL2;PF4;CXCL2;CXCL3;GRO1;MTHFD2L	chr6	90662691	90988309	1.43084	0.0	0.0	0.384509	0.111365	0.147949	0.256819	0.142136	0.623658
XLOC_081626	CXCL2;PF4;CXCL2;CXCL3;GRO1;MTHFD2L	chr6	90662691	90988309	0.610072	0.182506	0.954656	0.21878	0.143392	0.126807	0.0555869	0.183644	0.160026
XLOC_081627	CXCL2;PF4;CXCL2;CXCL3;GRO1;MTHFD2L	chr6	90662691	90988309	0.0	1.69007	0.0	0.25324	0.653718	0.0	0.495451	0.0	0.0
XLOC_081628	CXCL2;PF4;CXCL2;CXCL3;GRO1;MTHFD2L	chr6	90662691	90988309	0.0	0.0	0.0	0.234139	0.0	0.0	0.0	0.0	0.0
XLOC_081629	CXCL2;PF4;CXCL2;CXCL3;GRO1;MTHFD2L	chr6	90662691	90988309	0.0	0.0	0.0	0.0	0.0	0.305496	0.0	0.0	0.0
XLOC_081630	CXCL2;PF4;CXCL2;CXCL3;GRO1;MTHFD2L	chr6	90662691	90988309	3.07083	0.0	0.0	0.549336	0.0	0.0	0.0	0.0	0.532307
XLOC_081631	CXCL2;PF4;CXCL2;CXCL3;GRO1;MTHFD2L	chr6	90662691	90988309	9.14257	9.57218	9.19664	4.27372	4.60316	5.36018	6.12376	4.71534	3.76793
XLOC_081632	CXCL2;PF4;CXCL2;CXCL3;GRO1;MTHFD2L	chr6	90662691	90988309	0.0	0.0	0.0	0.341951	0.0	0.263359	0.229022	0.126628	0.221944

XLOC_081633	CXCL2;PF4;CXCL2;CXCL3;GRO1;MTHFD2L	chr6	90662691	90988309	0.0	0.0	0.924868	0.10598	0.184484	0.0	0.106634	0.0	0.0
XLOC_081634	CXCL2;PF4;CXCL2;CXCL3;GRO1;MTHFD2L	chr6	90662691	90988309	0.0	0.74372	0.0	0.111441	0.193907	0.0	0.0	0.0	0.108502
XLOC_081635	CXCL2;PF4;CXCL2;CXCL3;GRO1;MTHFD2L	chr6	90662691	90988309	0.0	0.599414	1.56755	0.0	0.0	0.0	0.356374	0.0	0.0
XLOC_081636	CXCL2;PF4;CXCL2;CXCL3;GRO1;MTHFD2L	chr6	90662691	90988309	0.0	0.0	0.0	0.216217	0.125795	0.0834643	0.0	0.0	0.0
XLOC_081637	CXCL2;PF4;CXCL2;CXCL3;GRO1;MTHFD2L	chr6	90662691	90988309	0.0	0.39783	0.0	0.35766	0.310982	0.0	0.0	0.132359	0.232104
XLOC_081638	CXCL2;PF4;CXCL2;CXCL3;GRO1;MTHFD2L	chr6	90662691	90988309	0.0	0.0	0.0	0.0	0.0	0.256658	0.0	0.245016	0.0
XLOC_081639	CXCL2;PF4;CXCL2;CXCL3;GRO1;MTHFD2L	chr6	90662691	90988309	0.659593	0.39462	0.0	0.0591334	0.15497	0.0	0.0	0.0661447	0.115323
XLOC_081640	CXCL2;PF4;CXCL2;CXCL3;GRO1;MTHFD2L	chr6	90662691	90988309	0.705414	0.105541	0.276037	0.189779	0.304602	0.330432	0.193748	0.035504	0.277813
XLOC_081641	CXCL2;PF4;CXCL2;CXCL3;GRO1;MTHFD2L	chr6	90662691	90988309	0.0	1.00977	0.880293	0.0	0.0878311	0.349853	0.101595	0.224483	0.294726
XLOC_081642	CXCL2;PF4;CXCL2;CXCL3;GRO1;MTHFD2L	chr6	90662691	90988309	0.566579	0.169502	2.21659	0.101597	0.133216	0.235596	0.154997	0.113763	0.148641
XLOC_081643	CXCL2;PF4;CXCL2;CXCL3;GRO1;MTHFD2L	chr6	90662691	90988309	0.828943	0.743784	0.0	0.297202	0.129662	0.086034	0.0752231	0.0829544	0.144842

XLOC_081644	CXCL2;PF4;CXCL2;CXCL3;GRO1;MTHFD2L	chr6	90662691	90988309	0.0	0.196952	0.515108	0.354143	0.0515631	0.136809	0.179806	0.0660252	0.0575566
XLOC_081645	CXCL2;PF4;CXCL2;CXCL3;GRO1;MTHFD2L	chr6	90662691	90988309	1.22693	0.0	0.0	0.109931	0.382603	0.0	0.110524	0.0	0.0
XLOC_081646	CXCL2;PF4;CXCL2;CXCL3;GRO1;MTHFD2L	chr6	90662691	90988309	0.0	0.444379	2.32431	0.53267	0.115663	0.307345	0.799729	0.147587	0.518308
XLOC_081647	CXCL2;PF4;CXCL2;CXCL3;GRO1;MTHFD2L	chr6	90662691	90988309	0.0	0.0	0.0	0.0	0.0	0.712909	0.0	0.0	0.0
XLOC_081648	CXCL2;PF4;CXCL2;CXCL3;GRO1;MTHFD2L	chr6	90662691	90988309	0.0	0.266551	1.39425	0.239643	0.0696697	0.0	0.242353	0.178241	0.0
XLOC_081649	CXCL2;PF4;CXCL2;CXCL3;GRO1;MTHFD2L	chr6	90662691	90988309	0.0	0.22019	1.15176	0.197964	0.11523	0.305776	0.267661	0.0	0.128668
XLOC_081650	CXCL2;PF4;CXCL2;CXCL3;GRO1;MTHFD2L	chr6	90662691	90988309	0.0	1.15717	1.5131	0.346766	0.15012	0.199618	0.0	0.0	0.337019
XLOC_081651	CXCL2;PF4;CXCL2;CXCL3;GRO1;MTHFD2L	chr6	90662691	90988309	0.0	0.351955	0.460256	0.0527403	0.138285	0.24457	0.321713	0.177119	0.154311
XLOC_081652	CXCL2;PF4;CXCL2;CXCL3;GRO1;MTHFD2L	chr6	90662691	90988309	0.0	0.533002	0.0	0.159727	0.0	0.184037	0.318105	0.176434	0.0
XLOC_081653	CXCL2;PF4;CXCL2;CXCL3;GRO1;MTHFD2L	chr6	90662691	90988309	0.0	0.0	1.5719	0.180125	0.155873	0.207296	0.178665	0.0	0.35005
XLOC_081654	CXCL2;PF4;CXCL2;CXCL3;GRO1;MTHFD2L	chr6	90662691	90988309	0.0	0.0	0.0	0.386034	0.335406	0.445587	0.0	0.0	0.250449

XLOC_081655	CXCL2;PF4;CXCL2;CXCL3;GRO1;MTHFD2L	chr6	90662691	90988309	0.0	0.70709	1.8491	0.211892	0.182912	0.0	0.417807	0.465088	0.617106
XLOC_081656	CXCL2;PF4;CXCL2;CXCL3;GRO1;MTHFD2L	chr6	90662691	90988309	0.0	0.932288	0.0	0.279402	0.479864	0.639456	0.271989	0.0	0.270697
XLOC_081657	CXCL2;PF4;CXCL2;CXCL3;GRO1;MTHFD2L	chr6	90662691	90988309	1.75347	0.523971	0.0	0.157021	0.272248	0.542833	0.15644	0.173503	0.0
XLOC_081658	CXCL2;PF4;CXCL2;CXCL3;GRO1;MTHFD2L	chr6	90662691	90988309	0.0	0.408336	1.0679	0.122371	0.212744	0.141299	0.0	0.271596	0.119106
XLOC_081659	CXCL2;PF4;CXCL2;CXCL3;GRO1;MTHFD2L	chr6	90662691	90988309	3.1699	0.631915	0.0	0.568117	0.164974	0.657049	0.190964	0.210886	0.27671
XLOC_081660	CXCL2;PF4;CXCL2;CXCL3;GRO1;MTHFD2L	chr6	90662691	90988309	0.0	0.0	10.367	1.20923	1.95275	0.0	1.04597	1.20637	2.27438
XLOC_081661	CXCL2;PF4;CXCL2;CXCL3;GRO1;MTHFD2L	chr6	90662691	90988309	0.27567	0.494968	0.0	0.395572	0.30319	0.172257	0.278023	0.416624	0.241328
XLOC_081662	CXCL2;PF4;CXCL2;CXCL3;GRO1;MTHFD2L	chr6	90662691	90988309	0.0	1.05967	0.69286	0.555742	0.34623	0.367604	0.883271	0.354321	0.232117
XLOC_081663	CXCL2;PF4;CXCL2;CXCL3;GRO1;MTHFD2L	chr6	90662691	90988309	0.0	0.944471	0.0	0.0	0.486003	1.29536	0.826074	1.2318	0.0
XLOC_081664	CXCL2;PF4;CXCL2;CXCL3;GRO1;MTHFD2L	chr6	90662691	90988309	1.10208	0.659417	2.15583	0.444646	0.734278	0.630177	0.603153	0.276656	0.385525
XLOC_079826	CXCL6	chr6	90604989	90646446	5.48547	6.78475	6.75167	1.44737	2.50491	1.30083	1.83048	2.55166	2.94139
XLOC_070511	CXCR7	chr3	116630346	116712464	27.5829	32.7855	27.7195	37.6969	35.2087	37.6503	40.1449	43.0883	41.6802
XLOC_070512	CXCR7	chr3	116630346	116712464	5.11402	4.19651	2.49259	6.30627	7.94374	8.95632	5.91109	7.352	9.51157

XLOC_072120	CXCR7	chr3	116630346	116712464	3.19602	3.05935	1.50034	4.6989	6.25836	4.7156	5.99551	5.77018	5.75583
XLOC_072121	CXCR7	chr3	116630346	116712464	0.0	0.861841	0.0	0.0	0.888623	0.887725	0.252877	0.0	0.500842
XLOC_072122	CXCR7	chr3	116630346	116712464	0.0	0.0	0.0	0.708258	0.906731	0.403398	1.70038	1.52777	2.39529
XLOC_092557	CXHXorf36	chrX	103960992	103981347	62.3864	56.3537	74.3093	78.501	90.558	88.4078	83.226	54.0859	74.8178
XLOC_093450	CXHXorf36	chrX	103960992	103981347	11.0635	3.30283	6.4777	8.41283	15.9762	16.4529	10.6621	5.94827	9.59865
XLOC_093451	CXHXorf36	chrX	103960992	103981347	10.9515	6.54813	8.55811	14.9034	14.6151	13.1743	14.0134	10.0683	7.52323
XLOC_093452	CXHXorf36	chrX	103985897	103987330	0.0	0.0	0.0	0.915071	1.5554	1.11995	0.67217	0.0	0.297514
XLOC_062201	CYB5B	chr18	36667302	36683977	13.3464	16.0131	12.7115	13.7612	10.056	11.2841	11.0014	15.4203	10.6041
XLOC_062408	CYB5B	chr18	36667302	36683977	28.6679	35.7726	32.5776	62.6056	61.1307	62.9687	59.3327	59.9182	65.169
XLOC_062990	CYB5B	chr18	36667302	36683977	2.0073	3.00305	2.19924	0.97199	1.00823	0.96087	0.771154	0.686564	1.05386
XLOC_089482	CYB5R4	chr9	66350483	66593080	64.8511	68.9876	65.2213	17.345	17.6813	17.3773	19.1462	17.2107	16.9842
XLOC_089967	CYB5R4	chr9	66350483	66593080	4.72941	5.39656	6.45121	3.13027	2.67396	2.45127	1.75141	1.61659	1.88621
XLOC_089968	CYB5R4	chr9	66350483	66593080	37.9783	49.6039	51.6447	4.84751	6.69142	6.17017	10.1643	7.38215	8.98514
XLOC_091140	CYB5R4	chr9	66350483	66593080	1.53443	1.834	7.19444	1.09918	1.78946	2.85326	2.88561	1.06535	2.00515
XLOC_091141	CYB5R4	chr9	66350483	66593080	2.23653	3.33917	10.4787	2.0012	0.864569	0.460116	0.593247	0.659873	2.33217
XLOC_091142	CYB5R4	chr9	66350483	66593080	10.2757	4.09642	5.35671	0.306905	1.15788	1.3009	1.13308	0.569075	0.49816
XLOC_091143	CYB5R4	chr9	66350483	66593080	0.0	3.3255	8.69541	0.664562	0.852552	0.379155	1.92212	2.51563	1.60705
XLOC_091144	CYB5R4	chr9	66350483	66593080	2.56743	3.45353	3.01065	0.804951	0.500096	0.664202	0.808521	0.255476	1.23147
XLOC_091145	CYB5R4	chr9	66350483	66593080	4.24666	4.60542	5.39957	0.713895	0.707447	0.772711	0.629675	0.746396	0.742807
XLOC_091146	CYB5R4	chr9	66350483	66593080	4.26169	5.09135	4.99294	0.762839	0.0	0.0	0.943931	1.04927	0.741031
XLOC_091147	CYB5R4	chr9	66350483	66593080	2.82364	0.84437	4.41658	0.0843498	0.220616	0.292833	0.255681	0.188109	0.246566
XLOC_091148	CYB5R4	chr9	66350483	66593080	0.0	4.54999	8.92291	0.0	0.582883	0.0	0.32825	0.0	0.659498
XLOC_091149	CYB5R4	chr9	66350483	66593080	2.14563	3.31702	6.15687	0.288607	0.336881	0.335	0.0327367	0.179965	0.312949
XLOC_091150	CYB5R4	chr9	66350483	66593080	2.48873	2.41949	4.3809	0.167331	0.341169	0.129312	0.28337	0.0624239	0.489554
XLOC_091151	CYB5R4	chr9	66350483	66593080	14.8643	8.86786	20.2893	0.996835	0.568369	1.51649	2.56282	0.718786	1.60705
XLOC_091152	CYB5R4	chr9	66350483	66593080	19.2292	5.73235	14.9866	0.0	0.965202	1.29438	2.12941	0.60483	0.551417
XLOC_091153	CYB5R4	chr9	66350483	66593080	3.0535	2.96812	2.98569	0.342118	0.657035	0.79255	0.138707	0.305797	0.533633
XLOC_091154	CYB5R4	chr9	66350483	66593080	3.50176	2.08908	16.3873	0.626142	0.536304	0.357619	0.605781	0.0	0.303009
XLOC_091155	CYB5R4	chr9	66350483	66593080	7.01669	6.29347	9.14412	0.419118	0.456038	0.726671	0.421811	0.582681	0.510191
XLOC_091156	CYB5R4	chr9	66350483	66593080	6.56897	6.15832	10.9664	0.667558	0.446651	0.637857	0.840618	0.616484	0.344827
XLOC_091157	CYB5R4	chr9	66350483	66593080	6.29495	7.80271	7.38899	0.725713	0.493822	0.795189	0.65745	0.632874	0.904744
XLOC_091158	CYB5R4	chr9	66350483	66593080	11.785	10.4242	18.8442	1.14854	1.04457	1.06567	1.35636	0.720648	1.12042
XLOC_091159	CYB5R4	chr9	66350483	66593080	8.44452	7.57667	10.5685	0.378437	0.726373	1.75263	0.842666	0.675893	0.295079

XLOC_062364	CYBA;RNF166;MVD;SNAI3	chr18	13687163	13972666	2.14357	2.17087	2.74827	0.291445	0.320241	0.47931	0.579655	0.254286	0.405755
XLOC_062693	CYBA;RNF166;MVD;SNAI3	chr18	13687163	13972666	4.92891	2.20777	7.698	0.0	0.190245	0.0	0.217067	0.0	0.0
XLOC_065509	CYBRD1	chr2	24949948	24959573	34.6836	31.4044	35.9203	46.3059	35.7608	41.6249	41.6202	48.7226	38.4039
XLOC_065510	CYBRD1	chr2	24962193	24972120	3.23898	4.82156	3.53684	15.9336	18.4655	15.6614	17.3424	18.2784	21.0866
XLOC_066169	CYBRD1	chr2	24973847	24975081	22.3814	22.6133	19.4988	26.9584	22.5384	22.6787	30.3262	30.3519	36.4606
XLOC_067081	CYBRD1	chr2	24949948	24959573	6.71025	9.01023	0.0	7.20124	7.20342	7.54538	6.98376	11.0765	4.358
XLOC_067082	CYBRD1	chr2	24959679	24959846	29.9166	36.4181	71.4306	74.1735	43.3004	36.0269	20.4427	50.1325	57.0406
XLOC_067083	CYBRD1	chr2	24960193	24961896	3.74368	4.48035	2.92954	9.2312	8.33214	10.3195	10.9442	10.207	8.63781
XLOC_065511	CYBRD1;DCAF17	chr2	24975413	25054124	9.04804	10.6716	8.39799	4.01501	4.3007	4.36971	2.03841	2.59713	3.14377
XLOC_041866	CYP11A1	chr10	21126002	21160853	1.40534	0.893803	0.274962	12.9766	10.4304	14.1007	3.75682	4.97993	5.59134
XLOC_065755	CYP20A1	chr2	91911460	91918848	18.661	19.4696	17.598	14.2868	10.2827	12.1435	14.466	17.0715	9.41777
XLOC_065756	CYP20A1	chr2	91922304	91929645	0.313016	0.187334	0.489969	0.757927	0.614571	0.912551	0.889042	0.630461	0.493171
XLOC_066409	CYP20A1	chr2	91929828	91932022	80.3156	41.5522	40.7004	306.02	444.63	357.68	250.47	215.021	350.415
XLOC_068113	CYP20A1	chr2	91910686	91911344	1.44513	0.863921	0.0	0.776679	0.899716	0.747051	0.648289	0.287053	1.63763
XLOC_068114	CYP20A1	chr2	91919960	91921248	0.639754	0.765514	1.50161	0.573537	0.50111	0.465327	0.174776	0.449182	0.447448
XLOC_044670	CYP26B1	chr11	12236606	12464936	3.17747	2.19409	1.80656	19.394	19.7848	22.1745	41.7345	51.0675	23.4624
XLOC_045488	CYP26B1	chr11	12236606	12464936	0.0	0.746064	0.0	1.78852	3.85643	2.82318	5.27837	3.1851	6.07512
XLOC_045489	CYP26B1	chr11	12236606	12464936	1.74923	0.0	2.73395	2.97608	2.17279	3.42969	6.08681	3.46173	4.11238
XLOC_045490	CYP26B1	chr11	12236606	12464936	0.0	0.50561	1.32228	1.66666	2.49682	2.27058	3.17348	3.01561	3.68381
XLOC_045491	CYP26B1	chr11	12236606	12464936	0.0	0.0	0.926381	1.06149	2.49457	3.06713	4.05853	3.06899	2.68761
XLOC_045492	CYP26B1	chr11	12236606	12464936	2.62525	0.52343	1.36897	2.35295	2.80497	2.08828	3.64971	3.06317	5.88581
XLOC_045493	CYP26B1	chr11	12236606	12464936	0.0	0.485211	1.26894	0.872415	2.77575	1.00604	4.06539	1.76988	4.10156
XLOC_045494	CYP26B1	chr11	12236606	12464936	0.0	0.0	0.0	3.45369	3.42585	3.04258	7.77867	2.89653	5.92269
XLOC_045495	CYP26B1	chr11	12236606	12464936	0.736131	0.0	1.15176	2.37554	3.51452	2.29331	3.81416	2.72867	4.31039
XLOC_045496	CYP26B1	chr11	12236606	12464936	0.0	0.0	1.05991	1.82175	3.27307	2.52441	4.26428	2.69589	4.84689
XLOC_045497	CYP26B1	chr11	12236606	12464936	1.56633	0.0	0.0	1.61459	2.63474	2.43912	3.41416	2.35243	3.7638
XLOC_045498	CYP26B1	chr11	12236606	12464936	2.75534	1.64514	0.0	2.71146	3.60778	3.39129	4.58742	5.38759	9.32339
XLOC_045499	CYP26B1	chr11	12236606	12464936	0.0	0.0	0.0	1.29385	4.393	2.44704	4.90427	0.922167	2.49517
XLOC_045500	CYP26B1	chr11	12236606	12464936	0.0	1.66647	8.71418	1.5015	5.07165	0.0	7.03428	3.1857	5.77799
XLOC_045501	CYP26B1	chr11	12236606	12464936	4.13861	0.0	0.0	2.2205	4.73405	2.94957	3.18967	2.39084	6.43602

XLOC_045502	CYP26B1	chr11	12236606	12464936	0.409646	0.612853	0.961739	2.05707	2.70031	3.45274	4.45826	3.54364	5.87837
XLOC_045503	CYP26B1	chr11	12236606	12464936	1.60067	0.159627	0.0	2.15273	3.38811	2.99578	4.52762	3.6975	3.68651
XLOC_045504	CYP26B1	chr11	12236606	12464936	0.0	0.596108	0.0	2.67946	3.86509	3.08395	6.02611	5.11818	7.29064
XLOC_045505	CYP26B1	chr11	12236606	12464936	1.34815	0.0	0.0	2.53621	4.09495	3.62615	6.30094	4.82591	5.76039
XLOC_045506	CYP26B1	chr11	12236606	12464936	0.0	0.0	0.0	2.17712	1.66673	3.88378	4.03151	3.17409	4.92738
XLOC_045507	CYP26B1	chr11	12236606	12464936	0.0	0.0	0.0	3.10008	2.47897	3.43615	6.0925	5.09063	5.79328
XLOC_045508	CYP26B1	chr11	12236606	12464936	0.0	1.0929	0.0	2.62003	3.83104	3.77268	2.76998	5.06213	4.13913
XLOC_045509	CYP26B1	chr11	12236606	12464936	0.0	0.569565	0.744811	1.70688	2.97614	3.45651	5.08686	3.2351	4.07481
XLOC_045510	CYP26B1	chr11	12236606	12464936	0.0	0.0	3.32141	3.80907	2.27196	3.03403	3.64083	7.78253	7.35775
XLOC_045511	CYP26B1	chr11	12236606	12464936	0.0	1.35715	0.0	2.84954	3.46172	2.31274	6.58475	4.80058	5.89246
XLOC_045512	CYP26B1	chr11	12236606	12464936	0.0	0.0	0.0	3.48369	3.8359	3.67188	5.46782	4.21762	6.38648
XLOC_045513	CYP26B1	chr11	12236606	12464936	0.0	1.01996	2.66706	2.44557	3.40587	2.44517	3.55387	4.64355	5.03109
XLOC_045514	CYP26B1	chr11	12236606	12464936	0.0	1.43764	0.0	4.30801	2.97436	4.45311	6.57909	4.72595	6.69104
XLOC_045515	CYP26B1	chr11	12236606	12464936	0.0	0.0	0.0	2.63153	4.22656	4.31051	12.4175	5.2141	4.93507
XLOC_045516	CYP26B1	chr11	12236606	12464936	0.0	2.50385	6.5455	3.77901	5.64195	4.21878	9.56937	1.56292	5.76699
XLOC_045517	CYP26B1	chr11	12236606	12464936	0.0	0.0	2.67965	3.99285	5.0008	4.56214	3.56973	5.33108	5.35199
XLOC_045518	CYP26B1	chr11	12236606	12464936	0.0	0.0	5.12721	5.30945	8.90868	6.63896	9.81595	9.91831	9.6191
XLOC_045519	CYP26B1	chr11	12236606	12464936	1.51793	0.0	0.0	4.89444	6.37528	3.60792	7.34442	9.03736	7.14321
XLOC_045520	CYP26B1	chr11	12236606	12464936	0.0	3.22987	8.44265	10.7783	5.60976	7.58319	12.1086	8.94601	21.3399
XLOC_045521	CYP26B1	chr11	12236606	12464936	3.0295	0.905477	0.0	10.4468	7.18681	6.10507	10.1798	10.071	8.97642
XLOC_063149	CYP2B6	chr18	50568016	50568788	0.0	0.35191	0.0	0.949134	1.00975	1.09703	0.742863	0.469123	1.1297
XLOC_080020	CYP2U1;LEF1;HADH	chr6	18366675	18514830	0.259239	0.764175	1.01467	0.110278	0.0	0.13015	0.0476719	0.131876	0.0572818
XLOC_070301	CYR61	chr3	58674756	58691659	24.7918	25.7001	21.8855	2.31426	3.25957	3.18916	4.46299	2.63674	2.36442
XLOC_048564	CYSLTR2	chr12	18399107	18400026	0.0	0.0	0.0	0.17024	0.148419	0.196999	0.0859954	0.189806	0.0829417
XLOC_048565	CYSLTR2	chr12	18503574	18503795	0.0	0.0	0.0	2.89944	3.16435	2.13822	0.854217	2.94442	2.74664
XLOC_048566	CYSLTR2	chr12	18514462	18515747	0.0	0.0	0.0	0.172519	0.0502436	0.133303	0.0584119	0.0643382	0.0
XLOC_048567	CYSLTR2	chr12	18516004	18517744	0.0	0.410216	0.0	0.409794	0.322653	0.332793	0.0835198	0.13783	0.399819
XLOC_048568	CYSLTR2	chr12	18525961	18527172	0.0	0.0	0.0	0.0	0.0537535	0.0	0.0	0.137639	0.0
XLOC_065629	CYTIP	chr2	38669383	38683698	1.81033	0.107266	1.76455	4.57278	3.28138	3.56769	2.25448	2.07897	4.29782
XLOC_066271	CYTIP	chr2	38692657	38702180	7.98486	6.33134	4.68663	0.071602	0.0313328	0.0415466	0.0	0.0	0.0
XLOC_066272	CYTIP	chr2	38707970	38714534	18.7526	7.90297	4.66726	0.0	0.0	0.0	0.0	0.0	0.0
XLOC_067376	CYTIP	chr2	38669383	38683698	0.0	0.0	0.0	2.34219	1.65119	1.35039	0.146149	0.161967	1.42387

XLOC_067377	CYTIP	chr2	38669383	38683698	0.0	0.0	0.0	1.09902	0.950859	2.10766	0.0	0.403483	0.88987
XLOC_067378	CYTIP	chr2	38669383	38683698	0.0	0.0	0.0	1.29931	1.31917	1.25127	0.326693	0.24074	0.3163
XLOC_067379	CYTIP	chr2	38683752	38684585	1.07071	0.0	0.0	1.2473	0.167154	0.776701	0.386914	0.213656	0.560765
XLOC_067380	CYTIP	chr2	38685391	38686237	2.10099	0.314122	1.64308	0.941361	1.14817	0.544379	0.569624	0.104837	1.37554
XLOC_070521	D2HGDH	chr3	121206692	121228885	2.62207	4.44378	2.05097	0.470021	0.0	0.181371	0.0792471	0.0874145	0.076347
XLOC_060291	DAO;CORO1C;SVOP	chr17	66336836	66473048	3.91216	6.50969	7.94751	31.6149	24.9021	27.4098	27.6837	35.4621	23.8472
XLOC_060292	DAO;CORO1C;SVOP	chr17	66336836	66473048	0.0	0.260143	0.680372	2.80663	2.24418	1.98553	1.81406	1.39199	1.2917
XLOC_061838	DAO;CORO1C;SVOP	chr17	66336836	66473048	0.0	0.721216	0.0	2.16134	0.373012	0.496417	0.425813	1.18537	0.209866
XLOC_061839	DAO;CORO1C;SVOP	chr17	66336836	66473048	0.0	0.271224	0.0	3.08874	1.70121	2.63418	2.87624	2.26668	1.98027
XLOC_061840	DAO;CORO1C;SVOP	chr17	66336836	66473048	1.15446	1.03553	0.0	2.68956	3.15187	3.7068	1.77027	2.41641	2.01485
XLOC_061841	DAO;CORO1C;SVOP	chr17	66336836	66473048	0.0	0.0	0.0	3.54637	2.84583	3.13029	1.41199	2.70253	2.00013
XLOC_086506	DAPK1	chr8	81856708	82124273	2.28474	0.570403	0.897697	0.0	0.0	0.0	0.0	0.0	0.0334593
XLOC_088336	DAPK1	chr8	82124431	82125611	1.41466	1.48106	0.0	0.0	0.0	0.0	0.0	0.0	0.0
XLOC_088337	DAPK1	chr8	82126024	82127714	0.94386	1.41198	1.10789	0.0	0.0370156	0.0	0.0	0.0	0.0
XLOC_066268	DAPL1	chr2	37575326	37703871	53.5647	61.3635	49.7784	72.4229	75.5926	66.0736	71.4007	66.9586	77.5801
XLOC_067368	DAPL1	chr2	37575326	37703871	0.862565	2.06378	6.07227	0.85041	0.8093	1.34253	1.09514	1.12176	1.58232
XLOC_067369	DAPL1	chr2	37575326	37703871	0.830854	3.23045	1.29983	0.893644	0.779747	1.12097	0.829322	0.582101	1.95985
XLOC_065659	DARS	chr2	61711680	61800381	43.0065	57.8285	42.0311	108.737	88.2167	102.597	117.793	133.58	119.032
XLOC_059201	DARS2	chr16	56686378	56688408	1.93118	1.04012	1.51134	0.277081	0.697251	0.522553	0.388728	0.349755	0.709765
XLOC_082994	DAZAP1	chr7	45459379	45461449	41.4755	32.3377	32.7703	132.604	139.121	133.01	146.066	134.281	125.313
XLOC_083634	DAZAP1	chr7	45456785	45459265	152.262	89.8984	79.6774	472.797	506.5	477.056	469.534	434.961	434.512
XLOC_084840	DAZAP1	chr7	45456080	45456675	1.65326	2.96442	5.16843	17.1746	21.9645	20.1435	15.8111	19.1624	24.7687
XLOC_082993	DAZAP1;GAMT	chr7	45434818	45455872	2.86076	4.24923	1.45864	19.5134	24.5097	21.762	16.6368	19.9625	24.4429
XLOC_083635	DAZAP1;RPS15	chr7	45461755	45466992	38.0097	46.3545	28.411	214.068	164.987	200.969	171.501	224.065	155.967
XLOC_038603	DAZL	chr1	155275492	155417417	40.9459	14.181	34.5888	4.95105	5.80997	5.98226	4.1737	6.58988	4.87242
XLOC_086667	DBC1	chr8	110500560	110565171	0.0	0.28145	0.736096	0.674764	0.661848	1.07368	0.511354	0.940485	2.13691
XLOC_086668	DBC1	chr8	110500560	110565171	0.0	0.732044	1.91458	0.804401	1.21246	1.01616	0.666471	1.30647	1.92458
XLOC_086669	DBC1	chr8	110500560	110565171	0.861488	1.54947	0.919053	5.88782	4.2098	4.29931	4.32309	6.07873	5.56724
XLOC_087080	DBC1	chr8	110500560	110565171	0.0	0.0	0.0	6.17297	1.55062	2.77492	2.27362	5.17462	1.18304

XLOC_088822	DBC1	chr8	110495316	110495750	0.0	0.0	0.0	12.8727	3.4289	8.0558	8.04262	15.6262	6.81219
XLOC_088823	DBC1	chr8	110500560	110565171	0.0	0.0	0.0	6.15994	4.10556	1.99623	4.16337	4.69931	4.66555
XLOC_072567	DBF4;CCDC126;SLC25A40;TRA2A	chr4	32238925	32502013	0.0	6.55815	5.04458	0.462426	0.280751	0.934792	0.928826	0.898813	0.288877
XLOC_073907	DBF4;CCDC126;SLC25A40;TRA2A	chr4	32238925	32502013	2.80877	4.7599	2.92915	0.419546	0.292643	0.291315	0.593546	0.561403	0.981177
XLOC_073908	DBF4;CCDC126;SLC25A40;TRA2A	chr4	32238925	32502013	3.14819	1.88179	9.84271	0.140979	0.244748	0.325228	0.140899	0.468268	0.411448
XLOC_073909	DBF4;CCDC126;SLC25A40;TRA2A	chr4	32238925	32502013	1.83452	3.01749	5.02209	0.328832	0.215061	0.0951461	0.415505	0.27507	0.400556
XLOC_073910	DBF4;CCDC126;SLC25A40;TRA2A	chr4	32238925	32502013	4.42379	6.17057	5.76346	0.264164	0.11473	0.15243	0.0	0.439202	0.0
XLOC_073911	DBF4;CCDC126;SLC25A40;TRA2A	chr4	32238925	32502013	3.53264	5.80579	4.14092	0.158164	0.0	0.182255	0.0	0.174742	0.153793
XLOC_073912	DBF4;CCDC126;SLC25A40;TRA2A	chr4	32238925	32502013	0.0	5.79167	1.37695	0.0	0.0	0.0	0.0	0.0	0.15342
XLOC_073913	DBF4;CCDC126;SLC25A40;TRA2A	chr4	32238925	32502013	2.8644	3.99747	4.48065	0.171139	0.372992	0.0990171	0.0864439	0.476992	0.333515
XLOC_073914	DBF4;CCDC126;SLC25A40;TRA2A	chr4	32238925	32502013	1.22485	5.12675	1.91544	0.109741	0.286471	0.380428	0.220682	0.243954	0.213713
XLOC_073915	DBF4;CCDC126;SLC25A40;TRA2A	chr4	32238925	32502013	2.97028	3.84901	0.77435	0.088729	0.0	0.513271	0.0	0.197764	0.1729
XLOC_073916	DBF4;CCDC126;SLC25A40;TRA2A	chr4	32238925	32502013	2.97345	5.33249	6.97294	0.0	0.0	0.307342	0.399867	0.0	0.12958
XLOC_073917	DBF4;CCDC126;SLC25A40;TRA2A	chr4	32238925	32502013	1.99621	3.10594	2.49954	0.143204	0.125331	0.0	0.146081	0.120492	0.20961
XLOC_073918	DBF4;CCDC126;SLC25A40;TRA2A	chr4	32238925	32502013	2.02118	3.62761	4.74386	0.0	0.158272	0.0	0.0	0.135096	0.0588952
XLOC_047368	DBH	chr11	104571029	104571645	4.73258	4.71473	3.69906	14.2699	10.9148	14.5039	9.3188	11.7317	10.171
XLOC_052514	DBNDD2	chr13	74458323	74460122	1.32177	0.527304	0.689572	0.829649	1.27896	0.825078	0.845696	1.19613	1.34915

XLOC_052515	DBNDD2	chr13	74461759	74462999	2.00437	1.19916	1.56815	1.49738	1.15106	1.73527	1.09458	1.07185	2.04418
XLOC_038044	DBR1	chr1	132007310	132024031	35.9635	34.5741	30.8373	5.98001	8.75402	5.96676	7.28027	7.68618	6.00533
XLOC_076817	DBX2	chr5	35502219	35511733	2.49254	1.24248	1.94974	1.04258	0.909705	1.98326	0.452355	0.249421	0.290348
XLOC_082764	DCAF15	chr7	12834687	12836284	4.95046	4.73725	3.0974	9.84891	7.03738	6.56986	5.91217	7.51502	8.55845
XLOC_082765	DCAF15	chr7	12836527	12847961	3.04231	3.64143	2.91014	9.7915	10.5638	9.99411	8.57496	8.95044	12.0709
XLOC_084195	DCAF15	chr7	12836527	12847961	18.1375	5.40688	0.0	6.50008	4.56021	5.49978	9.58173	6.86484	8.32617
XLOC_080077	DCAF16;NCAPG;LCORL	chr6	38692238	38875967	30.8203	24.4222	32.3689	3.991	2.53504	3.12433	3.18368	5.47733	2.603
XLOC_080887	DCAF16;NCAPG;LCORL	chr6	38692238	38875967	4.59985	2.06124	14.3705	0.823332	0.355545	0.473079	1.01586	0.226079	1.1992
XLOC_065512	DCAF17	chr2	25054206	25063291	11.5339	12.1852	11.6241	1.17343	1.60575	1.497	0.916342	0.496213	1.04296
XLOC_067084	DCAF17	chr2	25054206	25063291	0.0	1.46191	0.0	0.219036	0.377964	0.754555	0.0	0.0	0.425204
XLOC_071331	DDAH1	chr3	58923720	58924360	0.0	0.0	0.0	0.0	0.0	0.0	0.134363	0.0	0.0
XLOC_089837	DDO	chr9	40293235	40313519	34.828	18.1721	18.0452	7.66271	6.25021	6.92283	8.10511	9.89664	8.14391
XLOC_090636	DDO	chr9	40288338	40289485	0.730922	0.218631	0.0	0.393124	0.457679	0.683147	0.531585	0.292924	0.702682
XLOC_090637	DDO	chr9	40289946	40290855	1.92485	0.0	0.0	1.12127	0.676705	0.698617	0.348478	0.288453	1.0085
XLOC_090638	DDO	chr9	40293235	40313519	27.9823	8.37353	11.1834	3.07013	3.32019	3.65773	3.001	4.73758	3.04876
XLOC_070606	DDR2	chr3	6759133	6759619	0.0	7.23449	0.0	0.591239	0.0	0.226591	0.0	0.0	0.382825
XLOC_070607	DDR2	chr3	6762059	6763045	0.0	4.96059	3.41417	0.156485	0.1365	0.362306	0.237459	0.17462	0.762552
XLOC_070608	DDR2	chr3	6763737	6764235	6.37293	6.34548	4.97831	1.14089	0.164425	0.656126	0.188251	0.209247	0.923588
XLOC_070609	DDR2	chr3	6764985	6765314	16.8413	12.5561	16.4157	0.376503	0.320921	0.0	0.720299	0.40508	1.45474
XLOC_070610	DDR2	chr3	6766360	6766758	6.01419	17.0532	14.0821	0.268972	0.462341	1.23193	0.787052	0.0	1.30342
XLOC_070611	DDR2	chr3	6772656	6774124	1.65564	6.60421	4.31823	0.148441	0.0865169	0.286879	0.503386	0.0554147	0.627431
XLOC_070612	DDR2	chr3	6782356	6783067	3.92022	4.68777	3.06498	0.0	0.0	0.270436	0.117539	0.0	0.113965
XLOC_070613	DDR2	chr3	6783208	6783718	6.15594	6.74286	4.80917	0.367376	0.0	0.211356	0.36416	0.0	0.713901
XLOC_070614	DDR2	chr3	6787874	6788534	7.1969	8.60487	3.37561	0.386796	0.448088	0.446463	1.54986	0.714826	0.752834
XLOC_070615	DDR2	chr3	6788776	6790544	7.63423	9.00228	7.02839	0.523474	0.669357	0.467176	1.27224	0.45149	1.65009
XLOC_070616	DDR2	chr3	6796621	6798368	5.46007	10.8909	7.47727	1.22397	1.5348	0.851995	2.28677	1.23504	3.2243
XLOC_044310	DDX1	chr11	82780194	82871192	25.3966	29.9475	25.897	34.6165	28.2863	30.4388	31.2756	36.4202	33.9778
XLOC_044948	DDX1	chr11	82780194	82871192	7.64131	11.7888	9.67538	7.24476	6.75325	7.18551	6.0081	7.38187	5.09791
XLOC_046742	DDX1	chr11	82780194	82871192	1.57485	1.64871	3.08002	1.83519	2.52579	2.53407	1.14416	1.18258	1.85771
XLOC_046743	DDX1	chr11	82780194	82871192	1.99537	1.78851	1.55926	0.178812	0.77304	1.23358	0.0	0.787456	0.173588
XLOC_046744	DDX1	chr11	82780194	82871192	1.7039	1.52769	0.0	0.915657	0.793985	0.703525	0.152153	0.674812	0.445163
XLOC_055402	DDX10	chr15	18818949	18966936	0.773116	0.0	0.0	0.2079	0.0604919	0.0	0.070229	0.0	0.0

XLOC_076975	DDX17;DMC1	chr5	110676694	110780644	71.9841	78.6603	97.2143	57.9648	50.0819	46.8024	69.0965	65.6471	46.4775
XLOC_078717	DDX17;DMC1	chr5	110676694	110780644	8.93872	8.01522	15.1391	5.47104	4.17234	5.08148	4.67442	5.17583	4.80417
XLOC_078718	DDX17;DMC1	chr5	110676694	110780644	3.19898	2.87117	3.3375	0.956065	1.33753	1.77397	1.45945	0.963861	1.30565
XLOC_078719	DDX17;DMC1	chr5	110676694	110780644	0.0	2.54056	3.32141	11.4272	9.41242	11.7027	16.3837	15.5651	10.3008
XLOC_078720	DDX17;DMC1	chr5	110676694	110780644	0.0	16.8292	0.0	7.15434	6.6708	1.12806	3.59409	5.16715	4.83258
XLOC_076974	DDX17;KDELR3	chr5	110664200	110676512	32.9434	30.532	32.7204	14.8461	12.936	15.0574	15.1708	17.6604	11.6077
XLOC_078715	DDX17;KDELR3	chr5	110664200	110676512	4.86318	11.6178	9.49385	4.13405	2.25283	3.74779	5.57147	4.29531	3.80171
XLOC_078716	DDX17;KDELR3	chr5	110664200	110676512	7.22446	7.55567	4.23409	2.58758	2.10216	2.23566	3.38065	3.03613	3.30195
XLOC_076267	DDX23	chr5	31110208	31112525	0.767373	0.688578	1.20061	1.10057	1.74132	1.43411	0.976007	0.691637	0.469399
XLOC_045120	DDX31;BARHL1	chr11	102685916	102698259	5.96687	4.463	5.44729	30.9858	32.286	32.1654	31.5537	34.3238	40.8845
XLOC_092616	DDX3Y	chrX	143291102	143394238	4.67891	4.51753	1.93873	0.0	0.0485284	0.0	0.132363	0.072931	0.0
XLOC_093710	DDX3Y	chrX	143291102	143394238	1.91102	0.570958	0.0	0.0	0.0	0.0	0.0	0.0	0.0
XLOC_062152	DEF8;DBNDD1;GAS8	chr18	14783106	14899763	31.5095	43.7488	33.8022	10.4691	9.63091	10.2632	14.0082	13.1053	9.93819
XLOC_051969	DEFB119;DEFB119	chr13	61532072	61532535	0.0	0.707084	1.8491	2.75448	3.29241	2.19064	3.13354	2.3254	3.08553
XLOC_044373	DENND1A	chr11	94643109	94659914	2.63506	7.08048	4.11461	22.3299	18.0311	17.2353	19.8991	19.3114	18.0442
XLOC_044374	DENND1A	chr11	94936258	94941124	35.8482	10.1725	11.7275	4.77568	1.40339	5.22434	5.96311	2.22612	2.06134
XLOC_044375	DENND1A	chr11	94941233	94952579	24.4819	9.91887	12.3834	3.16685	1.04749	2.60345	4.86565	1.94888	1.85214
XLOC_044376	DENND1A	chr11	94989301	95007072	16.3131	29.1078	33.7008	17.0987	14.0046	14.6352	8.85695	15.8711	13.8031
XLOC_044377	DENND1A	chr11	95010300	95028999	0.324066	0.457375	0.573242	0.0268128	0.0214689	0.0	0.0	0.0	0.0
XLOC_045010	DENND1A	chr11	94542081	94549173	0.768777	2.27613	2.02675	0.309645	0.203203	0.269454	0.236758	0.39066	0.0755266
XLOC_045011	DENND1A	chr11	94559464	94561429	0.0	1.24841	0.0	0.748677	1.59564	1.70445	1.43286	1.61141	1.44648
XLOC_045012	DENND1A	chr11	94571268	94571740	0.0	2.27496	0.0	0.681973	1.16576	1.1666	1.96942	0.0	0.0
XLOC_045013	DENND1A	chr11	94643109	94659914	24.14	23.6105	21.6949	50.4288	42.0936	46.7723	53.3578	65.7655	48.1854
XLOC_045014	DENND1A	chr11	94663847	94676984	4.49531	7.40117	5.47643	12.6406	9.96745	10.078	10.244	11.3144	9.29709
XLOC_045015	DENND1A	chr11	94755717	94862289	0.471294	1.02619	0.35448	0.149661	0.0	0.174461	0.154117	0.0	0.0
XLOC_045016	DENND1A	chr11	95010300	95028999	1.42044	0.980287	0.796001	0.830505	1.11337	0.814281	0.829201	0.640191	0.68442
XLOC_045017	DENND1A	chr11	95029670	95041381	1.13811	2.38205	1.77996	2.03957	2.5749	2.35785	0.410759	1.9288	1.88707

XLOC_045018	DENND1A	chr11	95044429	95076719	14.8864	9.11794	13.3468	6.22715	6.97293	7.7293	5.08488	8.18775	4.27428
XLOC_047031	DENND1A	chr11	94539772	94540302	1.94179	0.580133	1.51715	0.173844	0.0	0.400286	0.0	0.0	0.0
XLOC_047032	DENND1A	chr11	94557713	94558387	8.40216	14.2323	8.75797	3.51237	6.2141	5.64804	5.40699	6.12285	5.37189
XLOC_047033	DENND1A	chr11	94565962	94566777	0.0	0.0	0.860231	0.394279	0.17169	0.569872	0.297969	0.438854	0.192019
XLOC_047034	DENND1A	chr11	94570239	94570758	0.0	0.597754	1.56323	0.0	0.620098	0.206159	0.0	0.592154	0.522191
XLOC_047035	DENND1A	chr11	94598903	94599339	0.0	0.775452	0.0	1.16186	0.400563	0.0	1.59751	0.508769	0.901831
XLOC_047036	DENND1A	chr11	94601358	94601787	0.0	0.795387	0.0	0.95339	0.410673	0.0	0.0	0.0	0.231214
XLOC_047037	DENND1A	chr11	94643109	94659914	0.0	0.0	0.0	8.19016	12.854	10.0288	6.61717	6.73619	7.79538
XLOC_047038	DENND1A	chr11	94643109	94659914	0.0	2.34952	6.14221	7.08495	2.35801	0.0	2.578	0.735781	3.3841
XLOC_047039	DENND1A	chr11	94660559	94661680	2.25194	1.34716	1.17446	1.54761	0.881167	0.623553	0.886813	0.601511	0.590394
XLOC_047040	DENND1A	chr11	94662514	94663535	3.35033	1.50306	0.655179	0.825809	0.91703	0.347697	0.53196	0.838074	0.585389
XLOC_047041	DENND1A	chr11	94663847	94676984	1.91212	0.857733	0.747762	0.68546	0.971051	1.28891	0.77903	1.05075	0.584416
XLOC_047042	DENND1A	chr11	94663847	94676984	0.0	0.347714	0.909423	1.14627	0.728671	0.785301	1.21856	0.641658	0.813092
XLOC_047043	DENND1A	chr11	94663847	94676984	1.04855	0.784255	0.820471	1.08115	0.86318	1.03579	1.00478	0.895232	0.871234
XLOC_047044	DENND1A	chr11	94677289	94677511	0.0	12.5787	0.0	19.0688	14.8394	16.884	11.8129	12.6001	14.457
XLOC_047045	DENND1A	chr11	94742017	94742802	2.30525	1.72313	2.70394	10.3277	8.18194	10.864	14.556	12.2927	7.04065
XLOC_047046	DENND1A	chr11	94755145	94755560	0.0	0.0	0.0	0.0	0.216251	0.0	0.0	0.0	0.243655
XLOC_047047	DENND1A	chr11	94952710	94953040	20.9308	9.9873	0.0	1.12301	1.27652	0.426112	3.94037	0.805707	0.0
XLOC_082803	DENND1C	chr7	19212320	19223528	1.07432	1.17871	1.40133	1.73417	0.927754	1.34193	0.819554	1.65805	0.376057
XLOC_084309	DENND1C	chr7	19223762	19224189	0.0	1.60254	0.0	0.480223	0.413655	0.275387	0.706415	0.525215	0.465826
XLOC_084310	DENND1C	chr7	19224842	19225545	0.0	0.0	1.03662	0.118782	0.413138	0.137185	0.834549	0.0	0.578143
XLOC_084311	DENND1C	chr7	19226973	19227493	0.0	2.98054	1.55892	2.50083	1.54604	2.05597	0.354477	0.393706	0.694347
XLOC_070766	DENND2D	chr3	32334108	32335882	0.447406	0.937047	1.05035	7.10086	7.3708	8.6573	5.888	6.52244	5.28421
XLOC_070767	DENND2D	chr3	32336052	32338555	1.85007	1.29177	1.20665	12.8308	11.7452	14.8007	11.2448	13.1671	7.93506
XLOC_059895	DERL3;ZNF70;CHCHD10;SMARCB1;IGLL1	chr17	73108043	73243561	9.9369	12.0121	11.5292	1.98528	2.25751	1.74501	1.3253	1.79659	1.88892
XLOC_061898	DERL3;ZNF70;CHCHD10;SMARCB1;IGLL1	chr17	73108043	73243561	1.27245	1.1412	0.0	0.227964	0.0991484	0.0	0.0	0.0	0.110972
XLOC_092192	DGAT2L6;IGBP1	chrX	85574024	85634638	10.9305	14.4874	12.6503	6.62208	7.29786	7.06317	8.5213	7.3355	7.16934
XLOC_093235	DGAT2L6;IGBP1	chrX	85574024	85634638	1.46862	3.51174	3.44406	2.10492	1.37124	1.36634	0.921953	0.145816	0.76804

XLOC_072560	DGKB	chr4	22357596	22434088	2.75594	5.86586	3.6745	4.94352	6.23241	5.89936	4.99584	2.75089	5.70603
XLOC_065865	DHDDS	chr2	127269866	127299136	12.7859	14.9077	14.29	25.7836	22.6017	24.6904	28.6357	27.4742	24.6339
XLOC_083092	DHFR;MSH3	chr7	83011146	83048119	0.0	0.0	0.0	0.0473497	0.0	0.0	0.0	0.186656	0.0
XLOC_047801	DHRS12	chr12	21270687	21315065	28.8126	39.2938	29.986	56.087	56.6993	60.3418	67.5748	68.1156	66.3267
XLOC_048101	DHRS12	chr12	21270687	21315065	12.8971	11.0974	5.97384	10.4447	8.15909	9.48279	15.1044	8.743	13.7486
XLOC_063959	DHRS7C	chr19	29520309	29606159	12.2112	10.6312	10.0671	13.0766	12.1888	13.3123	13.5397	11.5644	13.6479
XLOC_064222	DHRS7C	chr19	29520309	29606159	0.0	1.07123	0.329535	0.596542	0.700399	0.394332	0.413683	0.254147	0.290658
XLOC_064970	DHRS7C	chr19	29520309	29606159	0.0	4.10286	1.78839	2.1517	2.4978	3.43498	2.68233	2.05193	2.59451
XLOC_064971	DHRS7C	chr19	29520309	29606159	3.29021	3.65144	0.64371	2.5068	2.16236	4.381	1.92287	1.53553	1.66784
XLOC_050738	DHTKD1	chr13	12306287	12340494	4.03527	6.63021	9.45768	0.0	0.781506	0.415725	0.179144	0.397993	0.351019
XLOC_050737	DHTKD1;SEC61A2	chr13	12269428	12301128	3.60891	5.8328	7.06101	1.26248	1.38837	1.23963	0.660712	0.690136	1.16888
XLOC_062214	DHX38;HP;TXNL4B;DHODH;DHODH;ZNF821;MIR2328;IST1	chr18	38993095	39373298	18.9456	22.3235	21.6725	2.09772	1.8881	1.47873	2.37832	1.99801	1.81613
XLOC_063057	DHX38;HP;TXNL4B;DHODH;DHODH;ZNF821;MIR2328;IST1	chr18	38993095	39373298	3.72006	3.70951	7.76157	0.889357	0.291433	0.38659	0.790739	0.311017	0.704714
XLOC_063058	DHX38;HP;TXNL4B;DHODH;DHODH;ZNF821;MIR2328;IST1	chr18	38993095	39373298	13.7173	7.79619	12.3415	0.614846	0.483325	0.427477	0.811255	0.275019	0.659475
XLOC_063059	DHX38;HP;TXNL4B;DHODH;DHODH;ZNF821;MIR2328;IST1	chr18	38993095	39373298	4.53475	10.3913	11.8156	0.541557	0.117573	0.312432	0.54182	0.150005	0.0
XLOC_063060	DHX38;HP;TXNL4B;DHODH;DHODH;ZNF821;MIR2328;IST1	chr18	38993095	39373298	13.519	6.21927	9.75934	0.186379	0.0811934	0.323361	0.376	0.20759	0.544699

XLOC_063061	DHX38;HP;TXNL4B;DHODH;DHODH;ZNF821;MIR2328;IST1	chr18	38993095	39373298	0.0	8.35259	6.55261	0.0	0.215433	0.0	0.244993	0.0	0.0
XLOC_063062	DHX38;HP;TXNL4B;DHODH;DHODH;ZNF821;MIR2328;IST1	chr18	38993095	39373298	7.05597	6.98237	10.1927	0.243319	0.0850947	0.225727	0.396127	0.218023	0.237343
XLOC_064181	DHX40	chr19	10794569	10798749	0.0	0.0	0.0	0.546116	0.118552	0.157521	0.409695	0.302492	0.0
XLOC_064590	DHX40	chr19	10789346	10789881	0.0	0.0	0.0	0.171546	0.0	0.592569	0.0	0.189212	0.333473
XLOC_064278	DHX8	chr19	44034939	44069750	8.83318	9.61909	5.76153	6.21435	10.744	9.22655	12.6342	9.06989	10.3669
XLOC_092420	DIAPH2	chrX	47809380	47855244	24.6649	25.4818	25.4556	6.8375	4.29109	3.95676	5.19921	5.86571	8.05249
XLOC_076244	DIP2B	chr5	29434671	29451469	27.8498	24.9929	28.2643	24.9953	16.9994	22.3387	24.4811	35.5426	23.1871
XLOC_076781	DIP2B	chr5	29473478	29477828	0.0	0.0	0.0	0.388805	0.285924	0.531222	0.0662936	0.0730785	0.383161
XLOC_077503	DIP2B	chr5	29451616	29451747	139.048	0.0	0.0	43.9877	29.5433	28.5435	10.2299	118.338	62.1805
XLOC_077504	DIP2B	chr5	29452106	29452498	0.0	3.68162	2.4068	3.86161	2.84327	2.5257	2.95566	3.30395	3.47457
XLOC_077505	DIP2B	chr5	29454122	29454372	0.0	4.55774	0.0	0.0	0.0	0.769299	0.62657	2.85922	0.0
XLOC_077506	DIP2B	chr5	29456799	29457689	0.0	0.885811	0.0	0.884869	0.69412	0.409502	0.536029	0.591691	0.258641
XLOC_077507	DIP2B	chr5	29457868	29458920	0.0	0.483668	0.632491	0.0724738	0.695714	0.335707	0.0733999	0.242795	0.28258
XLOC_077508	DIP2B	chr5	29538185	29538546	0.0	0.0	0.0	192.499	159.113	178.464	195.604	179.653	214.572
XLOC_077509	DIP2B	chr5	29569213	29569395	20.642	6.22897	0.0	25.0327	7.52789	12.3956	3.18363	12.9195	21.3507
XLOC_050533	DIP2C	chr13	47043490	47102467	9.9351	14.5338	14.2995	5.54159	5.3527	5.64422	4.63277	5.97776	6.24992
XLOC_050830	DIP2C	chr13	47025356	47043264	5.55076	8.62675	4.34069	7.93812	13.0367	15.4971	10.2707	11.6668	15.0053
XLOC_050831	DIP2C	chr13	47043490	47102467	27.4997	29.9786	19.8513	59.9995	57.1158	60.7315	77.3499	78.5999	65.819
XLOC_050832	DIP2C	chr13	47104531	47106245	10.0656	5.31386	6.94903	17.5706	14.7537	16.8623	12.1424	19.0748	15.5837
XLOC_051765	DIP2C	chr13	47013458	47014672	0.684567	1.22865	0.535572	1.10463	1.23284	0.640007	0.934314	0.686257	0.837746
XLOC_051766	DIP2C	chr13	47023625	47024021	3.03227	0.0	0.0	1.62732	0.699186	1.55258	1.85103	1.47752	2.89129
XLOC_051767	DIP2C	chr13	47024437	47024999	3.57631	1.60294	0.0	2.24161	1.66534	1.84482	1.43489	1.41484	2.02381
XLOC_051768	DIP2C	chr13	47043490	47102467	0.0	1.17791	0.770137	0.44126	0.769187	0.306333	0.44552	0.688475	0.343968
XLOC_051769	DIP2C	chr13	47043490	47102467	1.60207	0.957619	0.0	0.860887	2.49034	0.661927	2.00651	1.11165	2.09355
XLOC_051770	DIP2C	chr13	47043490	47102467	10.5027	10.4452	16.3873	14.0879	17.6981	20.0246	23.0193	14.9325	24.8469
XLOC_051771	DIP2C	chr13	47102556	47102898	11.7479	11.6803	6.10835	10.8552	14.6547	14.7679	21.8739	13.605	13.5412
XLOC_051772	DIP2C	chr13	47103374	47104296	11.3527	7.63888	8.87933	21.1118	21.1413	19.7211	26.2131	19.8523	20.3238

XLOC_051773	DIP2C	chr13	47106400	47107634	0.0	0.602918	0.525628	1.32504	0.841779	0.348981	0.428013	0.53889	0.23492
XLOC_086557	DIRAS2	chr8	88697045	88747813	27.413	22.069	24.0663	5.69691	6.96629	5.65548	4.78652	4.31854	6.26827
XLOC_088527	DIRAS2	chr8	88687672	88688528	0.0	0.0	0.0	0.463949	0.808475	0.429308	0.093604	0.516774	0.903948
XLOC_088528	DIRAS2	chr8	88697045	88747813	0.657793	2.55802	1.02928	0.0589698	0.309098	0.136684	0.47905	0.131927	0.230017
XLOC_041847	DIS3L	chr10	13177104	13193172	19.657	20.6323	17.768	34.9554	28.953	33.5409	36.0852	46.7132	31.4158
XLOC_065825	DIS3L2	chr2	120567703	120600812	21.2049	18.2032	16.0514	17.3042	19.4684	20.3005	19.0972	21.8072	21.792
XLOC_065826	DIS3L2	chr2	120604583	120619291	4.109	12.8543	5.78987	36.8052	32.5493	37.6314	40.9791	32.3014	41.4088
XLOC_065827	DIS3L2	chr2	120629149	120665020	4.96272	3.70479	7.75067	1.11017	1.34067	1.01967	0.87396	1.70364	0.861973
XLOC_065828	DIS3L2	chr2	120666283	120673721	4.40383	1.31536	1.71992	0.394159	0.851637	0.0	0.584567	0.433378	0.382825
XLOC_065829	DIS3L2	chr2	120674147	120850289	7.06556	7.98308	6.18221	1.02089	1.37338	0.979903	1.19363	0.96603	0.984654
XLOC_066494	DIS3L2	chr2	120506569	120563794	20.5966	21.5176	20.9118	81.3149	84.7524	86.8016	94.7048	94.3621	81.1396
XLOC_066495	DIS3L2	chr2	120604583	120619291	5.07527	5.18442	3.97113	8.10512	10.283	8.84796	10.2412	10.7824	11.9891
XLOC_066496	DIS3L2	chr2	120674147	120850289	6.7667	9.65212	9.44891	10.3358	9.1069	9.79589	9.57281	8.52766	7.90467
XLOC_066497	DIS3L2	chr2	120674147	120850289	8.15349	10.3449	8.81357	2.92699	3.79502	3.72174	4.33022	3.16245	3.80853
XLOC_068401	DIS3L2	chr2	120503981	120505034	0.807651	0.483128	0.0	0.868715	1.38988	0.922168	0.293277	0.565895	0.987928
XLOC_068402	DIS3L2	chr2	120600959	120601745	3.45237	4.12895	2.69964	3.29961	1.97493	3.57589	3.73713	5.16172	3.21348
XLOC_068403	DIS3L2	chr2	120603576	120604425	0.0	0.312761	1.63596	0.374912	0.816591	0.650439	0.189067	0.835101	0.913064
XLOC_068404	DIS3L2	chr2	120619776	120619975	0.0	4.45685	11.6497	21.8108	13.1057	17.8352	13.9862	12.1184	28.0849
XLOC_068405	DIS3L2	chr2	120620387	120620592	0.0	16.1206	10.5345	63.9206	60.4836	74.0484	46.7115	67.3581	98.2091
XLOC_068406	DIS3L2	chr2	120620785	120621139	0.0	10.9728	5.73839	16.7744	14.6318	10.5097	20.3073	17.082	12.4094
XLOC_068407	DIS3L2	chr2	120621301	120621762	0.0	0.0	0.0	3.19914	0.736377	0.979939	1.89198	1.40417	1.86341
XLOC_068408	DIS3L2	chr2	120674147	120850289	0.0	3.5941	0.0	1.07701	1.11541	0.0	0.212228	0.236309	0.627285
XLOC_068409	DIS3L2	chr2	120674147	120850289	0.0	6.54813	0.0	2.98069	2.43595	3.29358	5.25504	3.02054	1.88081
XLOC_068410	DIS3L2	chr2	120674147	120850289	10.0192	0.0	2.60583	0.597341	1.28056	0.682826	2.8972	0.648677	0.578408
XLOC_068411	DIS3L2	chr2	120674147	120850289	9.05281	7.03285	4.24439	2.43173	3.0908	1.30736	1.12953	1.61119	4.09795
XLOC_068412	DIS3L2	chr2	120674147	120850289	3.83975	4.75902	3.00443	1.42623	2.06387	1.42572	1.6512	1.37702	2.25467
XLOC_068413	DIS3L2	chr2	120674147	120850289	7.16846	3.85686	4.483	1.7979	2.678	1.33412	2.44448	0.854456	3.49937
XLOC_068414	DIS3L2	chr2	120674147	120850289	7.58403	7.93058	10.37	3.56476	3.6755	1.75928	2.53071	2.62183	3.63
XLOC_092510	DLG3	chrX	85237499	85270554	0.0	0.207922	0.543802	0.186934	0.108843	0.216602	0.0	0.069672	0.303784
XLOC_052305	DLGAP4	chr13	66275716	66275915	0.0	0.0	0.0	2.72635	1.09214	2.97253	3.49655	1.34648	7.65952
XLOC_041978	DLGAP5	chr10	67888504	67912662	6.06274	4.92932	5.53938	11.6406	7.64518	8.40213	8.39247	9.44363	4.63968
XLOC_042881	DLGAP5	chr10	67886046	67888334	4.07355	3.14892	1.85973	5.66226	4.05143	4.59405	3.79255	5.80979	2.25799
XLOC_063094	DMKN	chr18	46356160	46356443	0.0	1.7051	0.0	6.1464	4.75356	5.20852	2.39515	2.17066	1.47768

XLOC_071808	DMRTA2	chr3	96454549	96455946	8.16889	8.37877	4.56545	8.57933	8.36764	7.39937	10.1063	10.7176	4.28592
XLOC_065724	DNAH7	chr2	84798470	84809594	5.53375	3.60191	6.00787	9.23043	7.41875	7.73751	7.18963	5.27153	9.87614
XLOC_065725	DNAH7	chr2	84826735	84830218	10.8692	14.086	11.3354	32.0659	19.8228	25.1815	25.1156	33.2334	28.7963
XLOC_065726	DNAH7	chr2	84839019	84863667	2.93265	2.43581	1.0201	8.73772	8.54848	7.38887	4.13442	4.85913	7.18199
XLOC_065727	DNAH7	chr2	84934559	84948811	8.45904	13.0322	13.3262	1.21346	1.63019	1.19304	1.08001	1.67525	1.27237
XLOC_065728	DNAH7	chr2	84949831	84958279	19.9529	20.0775	19.018	2.69201	2.69029	3.42428	1.53196	0.94631	1.86229
XLOC_066392	DNAH7	chr2	84811030	84818857	0.0	0.0	0.0	15.7951	6.3186	11.5565	5.59312	8.74783	5.94685
XLOC_066393	DNAH7	chr2	84872299	84886842	0.0	1.43974	1.25517	0.143823	0.188276	0.0832782	0.291354	0.0803093	0.0
XLOC_066394	DNAH7	chr2	84998787	85000755	0.0	0.509177	1.33161	0.152584	0.529311	0.175881	0.304306	0.0	0.0
XLOC_068066	DNAH7	chr2	84810590	84810835	0.0	0.0	0.0	10.8545	2.40594	5.66336	0.0	2.25129	3.45466
XLOC_068067	DNAH7	chr2	84871339	84872232	0.0	0.882201	0.0	0.0881263	0.0	0.0	0.0	0.0982177	0.0858634
XLOC_068068	DNAH7	chr2	84996661	84997005	0.0	0.0	0.0	0.346436	0.0	0.394961	0.0	0.374057	0.334962
XLOC_066391	DNAH7;SLC39A10	chr2	84765360	84775450	16.5751	6.59774	6.0402	6.46233	3.87383	5.34565	4.28374	4.877	6.51305
XLOC_064992	DNAH9	chr19	31062087	31062761	1.40036	0.837192	1.09475	1.25442	0.436077	0.579286	0.377232	0.556623	0.244177
XLOC_064993	DNAH9	chr19	31082847	31084605	24.8533	30.2851	27.9355	31.8477	24.8141	27.03	28.3691	33.1636	30.5985
XLOC_064994	DNAH9	chr19	31084766	31087907	6.30256	9.21291	12.1431	10.8703	8.34318	9.24164	10.43	12.3389	10.357
XLOC_064995	DNAH9	chr19	31114521	31116097	1.52958	1.06778	0.398962	1.09716	0.319827	0.583232	0.232711	0.25611	0.0
XLOC_048089	DNAJC15	chr12	13201720	13219237	0.0	0.878135	0.861281	0.789555	0.903611	1.12326	1.74825	1.00036	1.35192
XLOC_048090	DNAJC15	chr12	13219290	13249412	49.1115	64.9989	50.088	108.604	96.9945	104.18	113.374	124.44	109.227
XLOC_048483	DNAJC15	chr12	13201720	13219237	0.0	0.533576	0.930346	0.852824	0.791949	0.556138	0.270927	0.298327	0.363898
XLOC_048484	DNAJC15	chr12	13201720	13219237	0.0	0.0	0.0	0.249338	1.50235	0.857457	0.732286	1.63434	0.967187
XLOC_038457	DNAJC19	chr1	86666613	86670565	2.60407	3.11394	0.0	0.0	0.202889	0.134735	0.351379	0.0	0.227113
XLOC_055475	DNAJC24	chr15	62980661	62981688	0.0480099	6.62584	23.8065	14.8035	16.2165	19.1054	12.4223	14.6097	14.5989
XLOC_055784	DNAJC24	chr15	62980661	62981688	22.2741	27.5763	9.07904	23.7983	26.3902	25.1751	24.7793	26.3253	25.3238
XLOC_087035	DNAJC25;PTGR1;ZNF483;GNG10	chr8	102632225	102785096	6.67183	4.19222	3.85389	1.16889	0.486225	0.995423	1.38112	0.935319	1.05463
XLOC_051862	DNAJC5	chr13	54383053	54383903	0.0	1.24924	0.0	0.0	0.0815422	0.0	0.283198	0.0	0.0911751
XLOC_053254	DNAJC5B	chr14	32082853	32103282	4.81432	6.82235	8.5463	20.5742	26.7626	21.9975	18.6901	19.8641	25.7742
XLOC_053534	DNAJC5B	chr14	32082853	32103282	44.4805	34.8344	27.0335	74.4993	74.4911	74.4998	56.0523	81.6354	73.4294
XLOC_054137	DNAJC5B	chr14	32080394	32081562	2.14719	2.56909	2.23974	2.11728	3.30564	2.23004	2.40816	2.58201	2.31457
XLOC_054138	DNAJC5B	chr14	32081773	32082647	3.02832	6.03716	7.10519	4.61351	5.2818	5.23237	5.84295	3.83003	4.58287
XLOC_054139	DNAJC5B	chr14	32142123	32142468	0.0	0.0	0.0	0.344601	2.94448	1.57163	0.0	0.0	0.666412

XLOC_065182	DNAJC7	chr19	42787282	42788335	0.807651	5.07284	1.89535	0.0	0.0631765	0.167667	0.146638	0.0	0.0
XLOC_070119	DNALI1;GNL2;SNIP1;MEAF6	chr3	108904495	108960327	10.8802	10.8359	10.3014	4.39977	4.76046	4.52947	5.42247	5.3872	4.75903
XLOC_070497	DNALI1;GNL2;SNIP1;MEAF6	chr3	108904495	108960327	76.1817	87.0071	63.6365	74.276	73.3604	80.5542	97.1146	110.692	83.6038
XLOC_044431	DNM1	chr11	98837279	98840400	0.0	0.572462	0.0	0.0	0.0	0.592569	0.0	0.756847	0.0
XLOC_044432	DNM1	chr11	98842193	98846167	0.0	1.82531	0.0	0.273505	0.704953	0.313093	0.266547	0.595825	0.795123
XLOC_044433	DNM1	chr11	98849262	98851609	0.0	2.55968	0.0	0.340928	1.04029	1.08491	0.344426	0.380106	0.332201
XLOC_047236	DNM1	chr11	98852025	98853053	3.32339	0.993985	1.94974	0.893644	0.519831	0.431144	0.30157	0.249421	0.362935
XLOC_083408	DNMT1	chr7	15937726	15946222	43.6842	45.3787	41.5098	114.291	95.3977	107.713	107.959	127.056	93.3053
XLOC_083409	DNMT1	chr7	15949920	15967511	5.27485	3.72037	5.89733	0.540542	0.26612	0.352874	0.272594	0.341175	0.0658823
XLOC_084238	DNMT1	chr7	15936816	15937033	0.0	0.0	0.0	5.11024	3.33537	5.6403	2.69556	3.10029	3.86607
XLOC_084239	DNMT1	chr7	15948345	15949662	5.61377	2.79892	5.36827	0.223681	0.0977278	0.324096	0.397729	0.187728	0.0
XLOC_083407	DNMT1;MIR2454	chr7	15917216	15922458	42.6885	47.9425	45.4418	104.304	109.916	108.499	112.192	155.047	122.431
XLOC_065791	DOCK10	chr2	113525811	113709981	3.27531	2.45002	4.80597	1.02795	0.963852	1.06506	1.46029	0.658911	1.32547
XLOC_066460	DOCK10	chr2	113831362	113848716	8.94116	7.57114	8.27038	17.1961	19.937	20.5208	26.7819	21.6407	16.5332
XLOC_068295	DOCK10	chr2	113712234	113712946	2.60877	0.389943	0.0	0.350569	0.304877	0.269953	0.117332	0.389311	1.59265
XLOC_068296	DOCK10	chr2	113715176	113716177	0.857445	0.769333	1.3414	0.307409	0.335223	0.0	0.466606	0.0857737	0.898839
XLOC_068297	DOCK10	chr2	113748859	113749395	0.0	0.0	0.0	1.71093	0.0	0.0	0.850025	0.377447	0.0
XLOC_068298	DOCK10	chr2	113749646	113749975	0.0	0.0	0.0	0.753006	1.28368	0.428525	3.6015	0.40508	0.363685
XLOC_068299	DOCK10	chr2	113830628	113831141	0.0	0.0	0.0	1.09285	0.945594	0.41919	1.0836	1.20381	0.35396
XLOC_068300	DOCK10	chr2	113831362	113848716	0.0	2.0511	5.362	4.32108	5.68559	6.9373	6.82088	3.881	8.28126
XLOC_082789	DOCK6	chr7	16875418	16885339	0.0	1.06964	0.0	6.41093	5.95168	6.17442	4.62218	3.68297	5.30647
XLOC_082790	DOCK6	chr7	16891807	16892641	0.0	0.0	0.0	1.22161	0.289874	1.02655	0.446533	0.493672	0.216263
XLOC_084288	DOCK6	chr7	16885684	16887334	1.93831	0.869884	0.37919	8.34227	8.85489	7.66082	5.62044	4.57755	6.90936
XLOC_047916	DOCK9	chr12	79657823	79814192	0.0	2.13199	0.0	9.85173	8.60997	7.10248	3.8822	4.7509	12.1131
XLOC_065665	DPP10	chr2	67519247	67525954	0.0	0.0	0.0	1.31797	0.200268	1.26192	0.291039	0.25645	0.167645
XLOC_067761	DPP10	chr2	67567181	67568196	0.0	0.504521	0.0	0.377992	0.197872	0.0	0.0	0.168775	0.294736
XLOC_067762	DPP10	chr2	67568346	67568984	1.50529	0.449916	3.52994	0.0	0.0	0.155572	0.134907	0.298783	0.262373
XLOC_067763	DPP10	chr2	67570499	67571269	0.0	2.11835	1.84672	0.105803	0.276278	0.0	0.212925	0.117658	0.103033
XLOC_067764	DPP10	chr2	67573574	67574077	0.0	1.87615	0.0	0.374804	0.0	0.0	0.37126	0.0	0.0
XLOC_067765	DPP10	chr2	67594980	67595819	1.06129	0.0	0.0	0.190204	0.0828472	0.0	0.0	0.1059	0.0926411

XLOC_067766	DPP10	chr2	67607144	67607746	0.0	0.486301	1.27179	0.145729	0.0	0.0	0.0	0.0	0.0
XLOC_067767	DPP10	chr2	67609976	67610253	0.0	0.0	0.0	1.07453	0.0	0.0	0.0	0.0	0.0
XLOC_067768	DPP10	chr2	67611324	67612376	0.0	0.483668	0.0	0.0	0.0	0.0839267	0.1468	0.242795	0.070645
XLOC_065616	DPP4;DPP4;SLC4A10;GCG	chr2	34406755	34624395	28.9904	31.3123	31.6919	19.5565	18.8015	22.5459	18.6108	20.6201	18.928
XLOC_072785	DPP6	chr4	116877952	116989876	7.02151	9.0395	6.94773	2.9112	3.30276	3.12066	3.11862	2.68754	2.56423
XLOC_072786	DPP6	chr4	116995317	117005235	4.67611	6.1242	4.05535	9.40452	7.88923	8.47393	7.88713	9.19092	8.60776
XLOC_072787	DPP6	chr4	117019415	117086847	11.0186	20.019	17.2424	33.6577	37.4726	34.8059	40.602	41.4972	43.4097
XLOC_072788	DPP6	chr4	117096007	117115650	4.2032	4.1553	3.31804	4.50208	4.25314	4.56449	3.95812	6.21417	1.95065
XLOC_072789	DPP6	chr4	117145311	117146719	0.0	0.213444	0.0	0.789579	0.912377	0.745685	1.49705	0.261729	0.798539
XLOC_072790	DPP6	chr4	117252042	117268583	1.94294	1.16206	1.51961	0.522372	0.607127	0.20147	0.087924	0.388178	0.84829
XLOC_072791	DPP6	chr4	117550229	117682264	30.6011	23.8703	18.7743	25.0135	25.0477	21.7832	22.8771	24.6893	21.2767
XLOC_073310	DPP6	chr4	116990130	116994487	8.16889	19.2014	17.3487	41.5365	33.1962	32.8726	35.7975	42.2848	40.5121
XLOC_073311	DPP6	chr4	117120037	117127401	0.0	1.0124	2.36348	13.9655	12.5991	14.9337	6.89965	12.5667	9.53621
XLOC_073312	DPP6	chr4	117127811	117133780	9.67611	8.7529	10.0463	14.2335	17.4053	14.3004	16.3338	15.0884	13.3292
XLOC_073313	DPP6	chr4	117153828	117156257	100.799	177.653	137.677	471.774	495.59	579.665	570.529	592.206	586.976
XLOC_073314	DPP6	chr4	117159633	117182728	19.3665	31.2595	22.3606	88.6416	94.7234	86.3995	110.015	103.471	123.256
XLOC_073315	DPP6	chr4	117271461	117496764	42.159	44.9179	31.1707	73.3059	58.5514	64.9615	72.9549	89.5421	62.0784
XLOC_073316	DPP6	chr4	117550229	117682264	5.51678	11.8103	6.71019	16.1155	13.6702	14.225	14.8937	19.7321	16.5095
XLOC_075004	DPP6	chr4	117087638	117088618	0.0	1.84091	0.68781	0.945752	0.893677	0.638636	0.637768	0.615581	0.921716
XLOC_075005	DPP6	chr4	117118145	117118471	0.0	1.27769	0.0	0.383148	3.26419	0.871856	0.732161	1.23572	1.4801
XLOC_075006	DPP6	chr4	117147515	117149372	0.0	0.891301	0.999072	2.13692	3.03855	2.1696	3.15153	2.26824	3.05315
XLOC_075007	DPP6	chr4	117149560	117150125	0.0	1.59116	0.0	2.86089	2.61764	2.74709	2.84933	2.63371	2.16354
XLOC_075008	DPP6	chr4	117151293	117151506	0.0	0.0	0.0	9.75322	6.16723	3.57972	2.84213	9.81652	7.16028
XLOC_075009	DPP6	chr4	117152663	117152946	11.4399	3.41019	0.0	6.1464	3.02499	4.6298	6.70642	10.3106	9.35863
XLOC_075010	DPP6	chr4	117190058	117190462	0.0	0.875774	0.0	1.31223	2.48243	1.80373	3.33187	0.858755	1.01764
XLOC_075011	DPP6	chr4	117202646	117202898	0.0	6.70186	0.0	5.38516	3.93008	3.77208	1.84544	1.40289	3.86288
XLOC_075012	DPP6	chr4	117271461	117496764	0.0	0.0	0.0	0.0	0.206828	0.275414	0.0	0.0	0.465826
XLOC_075013	DPP6	chr4	117271461	117496764	0.0	0.0	0.0	1.78488	0.774176	1.02892	0.742128	3.29037	1.01263
XLOC_075014	DPP6	chr4	117271461	117496764	0.0	0.201159	0.0	1.02486	0.631918	0.838336	1.16282	2.56209	1.23447
XLOC_075015	DPP6	chr4	117271461	117496764	0.0	0.0	0.0	0.816455	0.395258	0.524703	1.00702	2.02138	0.707023
XLOC_075016	DPP6	chr4	117271461	117496764	0.0	0.0	0.0	2.57253	0.221292	1.76853	0.251463	1.40378	0.748217

XLOC_075017	DPP6	chr4	117271461	117496764	0.0	0.0	0.0	1.56213	2.68682	1.49126	2.79776	4.54445	1.26192
XLOC_075018	DPP6	chr4	117271461	117496764	0.0	0.0	0.0	0.72874	0.422331	1.68296	0.731019	1.48274	0.35465
XLOC_075019	DPP6	chr4	117271461	117496764	0.0	0.0	0.0	0.74662	0.406547	0.323834	0.753062	1.66308	0.636394
XLOC_075020	DPP6	chr4	117271461	117496764	0.0	0.210756	0.551213	1.01059	0.717072	0.731818	1.34577	1.55351	0.615834
XLOC_075021	DPP6	chr4	117271461	117496764	0.0	0.0	0.0	0.426027	0.370769	0.246183	0.642881	0.592103	1.45191
XLOC_075022	DPP6	chr4	117271461	117496764	0.0	0.0	0.0	0.212682	0.0925417	0.0	0.21395	0.0	0.103538
XLOC_075023	DPP6	chr4	117271461	117496764	0.0	0.0	0.0	0.0	0.0	0.0	0.130419	0.144377	0.253454
XLOC_075024	DPP6	chr4	117271461	117496764	0.0	0.0	0.846092	0.0969814	0.0844445	0.0	0.195442	0.107932	0.188871
XLOC_075025	DPP6	chr4	117271461	117496764	1.86661	0.0	1.45853	0.167182	0.289553	0.192502	0.0	0.184439	0.162462
XLOC_075026	DPP6	chr4	117271461	117496764	0.0	0.545075	1.42548	0.490073	0.0	0.0	0.162542	0.0	0.0
XLOC_075027	DPP6	chr4	117271461	117496764	0.0	0.0	0.417495	0.19137	0.083657	0.277392	0.292104	0.160761	0.0466647
XLOC_075028	DPP6	chr4	117271461	117496764	0.0	0.0	0.0	0.0	0.264042	0.0	0.151806	0.168305	0.0
XLOC_075029	DPP6	chr4	117271461	117496764	0.0	0.313213	0.819162	0.281622	0.0	0.217135	0.0946676	0.313611	0.0914382
XLOC_075030	DPP6	chr4	117271461	117496764	0.0	0.928786	0.0	0.0	0.120816	0.32109	0.139131	0.154115	0.0
XLOC_075031	DPP6	chr4	117271461	117496764	0.0	0.418658	0.0	0.18822	0.109576	0.0726947	0.509247	0.0701399	0.0611674
XLOC_075032	DPP6	chr4	117271461	117496764	0.0	0.0	0.0	0.206556	0.0	0.237303	0.0	0.0	0.0
XLOC_075033	DPP6	chr4	117271461	117496764	0.0	0.0	0.0	0.295293	0.428578	0.113814	0.0991752	0.766843	0.383457
XLOC_075034	DPP6	chr4	117271461	117496764	0.0	0.56664	0.0	0.127377	0.111409	0.0492535	0.216252	0.285528	0.289934
XLOC_075035	DPP6	chr4	117271461	117496764	0.37079	0.443796	0.580369	0.232765	0.203746	0.23156	0.407225	0.63435	0.356934
XLOC_075036	DPP6	chr4	117271461	117496764	0.0	0.625799	0.148799	0.119355	0.119552	0.0594254	0.261852	0.115039	0.0832328
XLOC_075037	DPP6	chr4	117550229	117682264	1.99936	3.58728	2.34555	1.88132	0.780762	1.7619	0.904297	0.898442	1.30924
XLOC_075038	DPP6	chr4	117550229	117682264	1.14664	1.54364	0.897199	1.43923	1.16821	1.54951	1.41138	0.920832	1.0027
XLOC_075039	DPP6	chr4	117550229	117682264	2.49306	2.23765	5.07216	1.38591	1.173	1.71122	1.41128	1.05208	1.2648
XLOC_072792	DPP6;HTR5A	chr4	117716899	117725701	0.0	0.77085	1.6129	0.0	0.0	0.0	0.0	0.0	0.0
XLOC_073317	DPP6;HTR5A	chr4	117728019	117729482	8.86388	5.73494	4.76824	8.59286	8.64576	8.69687	10.207	11.737	7.26727
XLOC_041844	DPP8;PTPLAD1;C10H15orf44;SLC24A1;DENND4A	chr10	12253286	12643865	14.1729	15.1773	17.3236	44.1872	43.9334	44.8511	56.5578	46.8329	40.7872
XLOC_076422	DPPA3;DPPA3	chr5	101755993	101822069	8.10844	9.56734	6.64207	6.14765	7.3779	9.22412	3.74149	3.69462	4.70231
XLOC_078442	DPPA3;DPPA3	chr5	101755993	101822069	1.71602	0.0	2.68215	1.53668	0.666284	0.8856	0.153202	0.509609	0.896623

XLOC_078443	DPPA3;DPPA3	chr5	101755993	101822069	0.0	0.335029	0.0	0.2008	0.437133	1.27687	0.70792	0.335176	0.68451
XLOC_038360	DPPA4;DPPA2	chr1	54478157	54557824	14.578	15.6441	11.026	11.5356	12.4298	11.7798	10.948	10.2609	12.7135
XLOC_039313	DPPA4;DPPA2	chr1	54478157	54557824	0.0	1.46631	0.0	5.71995	5.22551	3.99254	2.08168	1.40979	2.96899
XLOC_058923	DPT	chr16	37113895	37114957	0.0	3.58743	0.625503	0.0	0.0	0.24901	0.0726004	0.0	0.279465
XLOC_072670	DPY19L2	chr4	62286417	62298112	14.2755	5.14557	5.27573	6.91025	6.08748	7.2054	5.9759	5.24253	9.24004
XLOC_072671	DPY19L2	chr4	62300134	62389591	13.343	10.6354	11.0069	11.7137	15.8905	14.2234	16.4566	12.6368	16.4499
XLOC_073195	DPY19L2	chr4	62300134	62389591	15.4872	15.6025	9.46803	9.58697	9.73539	10.2957	11.2705	11.3921	14.9101
XLOC_074368	DPY19L2	chr4	62298280	62298939	1.44225	0.8622	0.0	1.55026	1.12243	1.19292	0.647031	0.859471	1.38293
XLOC_074369	DPY19L2	chr4	62300134	62389591	1.80602	0.53964	0.0	2.10228	2.24231	2.98095	3.70259	2.67892	3.93056
XLOC_074370	DPY19L2	chr4	62300134	62389591	2.56947	3.06884	4.0126	1.83927	0.396383	1.84692	1.58159	1.5108	1.56154
XLOC_074371	DPY19L2	chr4	62300134	62389591	3.59408	0.0	4.21281	1.1264	0.697291	0.741628	0.961417	1.77733	0.469339
XLOC_070273	DPYD	chr3	46442544	46443138	2.08752	1.24716	0.0	0.934311	0.484875	0.859878	0.370229	0.617145	0.726171
XLOC_070274	DPYD	chr3	46445691	46447946	1.3815	1.84436	1.08107	3.26477	2.22229	2.58681	1.43056	1.59676	2.0546
XLOC_070986	DPYD	chr3	46443247	46444610	0.59995	0.717913	0.938827	1.07575	0.658116	0.997678	0.164023	0.180618	0.944258
XLOC_070987	DPYD	chr3	46444673	46445639	0.0	1.07015	0.0	1.44317	1.88801	1.57768	0.324315	0.447232	0.625084
XLOC_053361	DPYS	chr14	62332032	62377969	22.5267	33.2503	16.1924	64.0715	79.187	68.606	68.8359	75.2216	101.613
XLOC_086493	DPYSL2	chr8	75093475	75100510	180.725	67.4807	93.0788	59.5962	72.9577	67.709	73.2252	37.424	40.7593
XLOC_086494	DPYSL2	chr8	75109479	75127620	45.1779	55.3545	45.9804	240.421	249.431	240.466	241.59	242.091	212.896
XLOC_088184	DPYSL2	chr8	75101884	75102063	0.0	0.0	0.0	2.06686	1.60757	0.0	0.0	1.96852	1.90422
XLOC_088185	DPYSL2	chr8	75108244	75108947	1.32591	0.0	2.07325	1.66294	3.20182	2.46933	1.6691	0.659385	1.6188
XLOC_088186	DPYSL2	chr8	75128449	75129441	1.73339	1.03683	2.03378	3.72865	4.60786	3.23744	4.95136	2.68736	3.25547
XLOC_088187	DPYSL2	chr8	75131245	75131785	0.0	0.0	1.47756	1.18515	1.46641	1.16982	1.68288	0.37359	0.822857
XLOC_088188	DPYSL2	chr8	75131966	75132484	0.0	0.0	1.56755	2.33505	2.33172	1.44706	2.13823	0.395838	1.39634
XLOC_088189	DPYSL2	chr8	75132967	75133938	1.77814	0.265894	0.0	0.956206	1.59849	0.737901	0.967063	0.444513	1.32016
XLOC_088190	DPYSL2	chr8	75135374	75136322	0.0	0.0	0.0	0.656002	1.35863	1.23378	1.16051	0.274381	1.35845
XLOC_044220	DPYSL5	chr11	72689446	72771324	16.944	20.2996	18.3246	15.2785	15.8342	15.4813	16.495	16.5819	15.4757
XLOC_046533	DPYSL5	chr11	72688129	72688406	0.0	3.57552	9.34822	2.14905	0.904909	3.6375	5.50584	2.27072	3.61358
XLOC_046534	DPYSL5	chr11	72688507	72689197	4.07484	1.62414	1.0619	1.0951	1.90402	1.40506	0.854417	0.675217	2.72407
XLOC_046535	DPYSL5	chr11	72689446	72771324	6.09003	6.36179	7.12959	5.71951	3.27627	2.4944	3.45131	2.96704	4.75089
XLOC_044219	DPYSL5;MAPRE3	chr11	72606758	72687705	11.1775	13.8209	12.4607	16.1882	14.5815	17.6091	19.2045	20.852	17.408
XLOC_045466	DQX1	chr11	10098810	10099681	0.0	0.0	0.0	0.0	0.0791616	0.0	0.0	0.0	0.0

XLOC_076862	DRAM1	chr5	66016279	66151795	9.53801	5.54627	13.5232	11.0653	7.04914	11.1865	4.63935	9.89736	4.34166
XLOC_069865	DRAM2	chr3	32397444	32465053	34.8443	33.167	30.2189	16.5906	16.4964	16.9331	18.0946	16.8336	18.7238
XLOC_070771	DRAM2	chr3	32397444	32465053	1.46563	5.69503	10.3113	2.36304	2.05274	2.27266	1.84031	2.61947	1.27749
XLOC_070772	DRAM2	chr3	32397444	32465053	6.2953	7.82372	7.57873	2.04075	1.78499	2.11539	1.76906	1.21662	1.31317
XLOC_070773	DRAM2	chr3	32397444	32465053	7.20007	7.32253	7.3226	1.54903	1.97262	1.49554	1.37487	0.865764	1.3215
XLOC_070774	DRAM2	chr3	32397444	32465053	1.36598	4.49165	3.20371	0.611831	0.957346	0.282592	0.859176	0.543184	1.07195
XLOC_050815	DTD1	chr13	38932178	38961166	6.67991	8.41753	9.37454	4.16776	4.78692	4.70639	6.75158	4.99599	7.09739
XLOC_062399	DUS2L	chr18	35618995	35630234	0.849345	0.770686	0.443172	2.03039	1.18235	2.52301	0.441216	0.47294	0.663973
XLOC_062918	DUS2L	chr18	35617602	35617758	0.0	0.0	33.8757	8.22425	0.0	12.7494	6.33179	0.0	0.0
XLOC_062919	DUS2L	chr18	35618282	35618807	0.0	1.76403	1.53775	1.05723	1.22026	0.608499	1.04948	0.582715	1.19869
XLOC_062920	DUS2L	chr18	35618995	35630234	2.45309	0.244566	1.27927	1.53914	0.959357	0.763839	0.964826	0.245512	0.857296
XLOC_062921	DUS2L	chr18	35618995	35630234	0.0	0.0	0.0	0.0	1.16516	0.519542	0.0	0.488671	0.0
XLOC_062193	DUS2L;DDX28;SLC12A4	chr18	35557393	35617441	199.675	236.676	198.535	276.261	298.747	286.901	346.44	324.397	374.768
XLOC_062398	DUS2L;DDX28;SLC12A4	chr18	35557393	35617441	0.0	0.407716	0.0	0.319814	0.205737	0.653438	0.220812	0.0	0.546599
XLOC_062902	DUS2L;DDX28;SLC12A4	chr18	35557393	35617441	58.5539	17.4548	4.56387	45.6292	41.9996	45.6001	38.1876	43.282	41.8453
XLOC_062903	DUS2L;DDX28;SLC12A4	chr18	35557393	35617441	2.91819	1.64943	1.77639	12.0093	14.3354	14.7185	11.2272	13.027	10.8697
XLOC_062904	DUS2L;DDX28;SLC12A4	chr18	35557393	35617441	0.77229	1.84797	0.0	7.68444	10.8774	9.06074	7.01592	8.04297	8.57105
XLOC_062905	DUS2L;DDX28;SLC12A4	chr18	35557393	35617441	19.5227	9.33214	16.7787	26.7424	36.0144	30.1824	33.4955	19.8484	28.3684
XLOC_062906	DUS2L;DDX28;SLC12A4	chr18	35557393	35617441	0.0	16.2591	5.31373	17.7406	18.9597	17.8793	24.8034	12.184	15.831
XLOC_062907	DUS2L;DDX28;SLC12A4	chr18	35557393	35617441	10.3203	18.4596	12.0665	20.7805	23.8555	20.4032	14.8041	15.742	20.0176
XLOC_062908	DUS2L;DDX28;SLC12A4	chr18	35557393	35617441	3.65967	4.36705	14.2741	7.2013	8.96177	8.21832	12.6338	2.83323	9.50003
XLOC_062909	DUS2L;DDX28;SLC12A4	chr18	35557393	35617441	7.71762	11.5099	12.0363	13.1886	13.2969	16.3191	7.59299	10.1034	14.5953
XLOC_062910	DUS2L;DDX28;SLC12A4	chr18	35557393	35617441	2.20191	4.60377	5.15987	2.76015	5.6219	4.53274	6.04168	3.9004	5.93493
XLOC_062911	DUS2L;DDX28;SLC12A4	chr18	35557393	35617441	1.24596	2.97998	2.92265	2.23316	4.66233	4.51472	4.71294	4.21757	5.76111
XLOC_062912	DUS2L;DDX28;SLC12A4	chr18	35557393	35617441	0.0	0.0	0.0	2.66938	7.23326	5.98048	1.22278	1.38989	3.82926
XLOC_062913	DUS2L;DDX28;SLC12A4	chr18	35557393	35617441	2.62541	2.35162	2.04998	1.8804	3.23997	1.8875	2.99895	0.771204	2.50814

XLOC_062914	DUS2L;DDX28;SLC12A4	chr18	35557393	35617441	5.76441	3.43671	4.49296	4.13245	5.22763	4.66752	11.1	3.2801	8.93739
XLOC_062915	DUS2L;DDX28;SLC12A4	chr18	35557393	35617441	0.0	3.14468	8.22053	1.91188	0.786031	4.2253	9.28657	5.81542	5.42678
XLOC_062916	DUS2L;DDX28;SLC12A4	chr18	35557393	35617441	5.25081	5.48711	4.09984	3.52467	2.02547	4.85184	4.38246	2.82775	2.96392
XLOC_062917	DUS2L;DDX28;SLC12A4	chr18	35557393	35617441	7.41863	3.69216	0.0	5.09054	2.67358	6.35252	1.30794	2.91083	2.7932
XLOC_083447	DUS3L	chr7	19756099	19770750	9.61095	4.6974	7.2713	34.9684	30.8805	30.653	32.2242	50.4451	32.5034
XLOC_066148	DUSP19	chr2	13511076	13544848	2.39881	4.72922	3.94255	5.94435	5.2215	5.26773	6.88035	7.90731	6.09978
XLOC_041960	DUT	chr10	62269002	62340510	15.8318	15.8815	12.5443	35.9658	46.7652	36.4762	41.6708	33.2716	48.5947
XLOC_072543	DYNC1I1	chr4	12906862	12981479	1.20892	1.15486	0.945875	0.446003	0.433243	0.654238	0.302499	0.554988	0.482848
XLOC_066167	DYNC1I2	chr2	24790089	24919576	27.9116	33.5242	29.9356	37.3896	29.7495	34.0559	32.849	36.4011	24.6393
XLOC_067069	DYNC1I2	chr2	24790089	24919576	0.0	0.317346	3.31987	0.665749	0.0	0.549934	0.28768	0.423598	0.0926432
XLOC_067070	DYNC1I2	chr2	24790089	24919576	0.0	0.0	0.0	0.504531	0.85152	0.570083	0.0	0.0	0.0
XLOC_067071	DYNC1I2	chr2	24790089	24919576	7.42813	2.21942	2.90211	0.831413	0.864264	0.383013	0.496096	0.0	0.32327
XLOC_067072	DYNC1I2	chr2	24790089	24919576	4.39048	0.655689	10.2883	0.196556	0.509458	0.677737	0.194288	0.864195	0.954185
XLOC_067073	DYNC1I2	chr2	24790089	24919576	0.0	2.71429	14.1943	0.814307	0.692343	0.462546	0.387338	0.0	0.0
XLOC_067074	DYNC1I2	chr2	24790089	24919576	5.40269	4.03762	5.27978	0.847024	0.736349	0.97808	0.364178	0.805796	0.58889
XLOC_067075	DYNC1I2	chr2	24790089	24919576	6.94195	2.06989	5.41143	0.623295	2.60743	0.700007	2.29301	0.0	0.596939
XLOC_067076	DYNC1I2	chr2	24790089	24919576	6.06986	1.20886	1.58068	0.543439	0.626926	0.208437	0.0	0.598618	0.351998
XLOC_067077	DYNC1I2	chr2	24790089	24919576	5.01433	0.748644	5.87328	0.224422	0.773907	1.28764	0.22066	1.22916	1.08857
XLOC_067078	DYNC1I2	chr2	24790089	24919576	0.0	3.63026	3.79739	1.30549	1.68962	0.749557	1.07138	1.67028	0.63356
XLOC_067079	DYNC1I2	chr2	24790089	24919576	1.32836	5.95645	9.34672	2.49904	2.37986	0.962045	1.43322	2.77442	1.39008
XLOC_067080	DYNC1I2	chr2	24790089	24919576	5.57985	8.33967	9.81477	2.62418	1.95486	2.88535	0.751628	1.80217	1.8243
XLOC_062389	DYNC1LI2	chr18	34562648	34579101	0.0	1.22524	1.55169	0.316374	0.15936	0.160703	0.140606	0.38992	0.357955
XLOC_064167	DYNLL2	chr19	9111986	9135714	3.47241	4.85177	4.52917	23.519	27.9546	24.3582	26.6355	21.2555	31.2582
XLOC_064555	DYNLL2	chr19	9111986	9135714	0.0	0.0	0.0	0.522778	1.34871	1.19771	2.29784	1.14054	0.506784
XLOC_038097	DYRK1A	chr1	151298378	151364469	83.2942	78.4285	79.4345	122.602	114.039	121.196	148.527	149.549	153.816
XLOC_038098	DYRK1A	chr1	151379382	151458028	11.2978	13.9755	12.0155	18.2681	14.9186	19.2339	12.1753	19.4874	13.3715
XLOC_038589	DYRK1A	chr1	151298378	151364469	0.0	0.508381	1.38034	0.811777	1.31399	0.982219	0.652179	0.379773	0.986113
XLOC_038590	DYRK1A	chr1	151367487	151372377	16.765	17.2516	11.5424	11.6026	13.2336	11.9131	12.4507	10.8252	17.9967
XLOC_038591	DYRK1A	chr1	151379382	151458028	25.3256	40.3802	36.6219	24.5323	27.5817	23.1031	27.4043	23.283	35.0083
XLOC_040585	DYRK1A	chr1	151298378	151364469	0.0	0.39783	0.0	0.477282	0.829454	1.23917	0.598247	1.85296	0.232122
XLOC_040586	DYRK1A	chr1	151364765	151365094	12.631	1.25561	6.56629	3.38853	6.0975	3.85672	3.24135	2.43048	3.63685

XLOC_040587	DYRK1A	chr1	151365190	151365630	5.12076	3.82251	1.99922	1.37454	2.37004	2.36621	2.47645	1.25446	2.00066
XLOC_040588	DYRK1A	chr1	151365806	151366290	0.0	0.661701	1.73044	1.78456	1.71353	1.59572	1.56801	1.08991	2.88864
XLOC_040589	DYRK1A	chr1	151366419	151367012	1.66085	2.48168	2.59606	1.18989	2.1935	2.40077	2.22639	0.822592	2.89323
XLOC_040590	DYRK1A	chr1	151379382	151458028	0.0	3.00274	1.96326	0.524912	0.457988	0.520982	2.12542	1.17196	1.97352
XLOC_057749	DYRK3;IL10	chr16	4278013	4420371	0.0	0.550604	2.99103	1.87807	0.989285	0.879592	0.389287	1.72676	0.886316
XLOC_057998	DYRK3;IL10	chr16	4278013	4420371	7.26393	6.07894	5.37378	0.966489	1.83518	0.920021	0.901036	0.815779	1.30154
XLOC_058261	DYRK3;IL10	chr16	4278013	4420371	8.56807	19.6352	7.81431	30.6997	31.1213	29.3835	33.3221	35.322	40.2091
XLOC_038353	DZIP3	chr1	53755105	53785218	36.3028	23.4442	13.464	11.5573	12.6778	17.0109	18.8016	15.0957	10.4261
XLOC_038354	DZIP3	chr1	53811551	53830730	1.51263	2.60251	2.07164	0.915597	0.356198	0.432934	0.27684	0.684948	0.0992772
XLOC_038355	DZIP3	chr1	53831508	53925347	1.57523	4.47768	4.31467	1.44523	0.332135	1.213	0.144106	1.04524	0.1723
XLOC_046873	E2F6	chr11	86357893	86358415	43.6572	34.9777	55.8138	17.9429	17.0683	17.5855	13.9278	14.2933	15.883
XLOC_083693	EBF1	chr7	72672775	72674880	17.6349	26.0315	21.4682	31.6978	26.4821	30.5465	38.0033	38.2841	30.2114
XLOC_048618	EBPL	chr12	19304765	19305055	0.0	0.0	0.0	10.6852	7.39049	9.3422	5.47379	8.25727	11.2195
XLOC_048619	EBPL	chr12	19305709	19305978	0.0	5.73222	4.99553	22.4099	8.6868	18.1203	13.841	16.3283	11.0283
XLOC_048620	EBPL	chr12	19307260	19307826	0.0	1.05818	0.0	3.32955	2.47389	3.83661	3.0005	5.42996	3.69991
XLOC_048621	EBPL	chr12	19308550	19308732	0.0	6.22897	16.2863	23.1071	24.0893	26.8572	17.51	11.0739	12.4546
XLOC_048622	EBPL	chr12	19309415	19309604	0.0	0.0	0.0	1.65274	7.83978	7.1447	11.0941	6.42271	6.14601
XLOC_048623	EBPL	chr12	19310118	19310693	0.0	0.0	0.0	1.55138	2.5558	2.32439	2.31936	1.71463	2.11204
XLOC_048624	EBPL	chr12	19312852	19313968	0.0	0.902789	0.59029	0.541106	0.767638	0.470093	0.617105	0.529043	0.923169
XLOC_065899	ECE1	chr2	132052463	132157679	4.61739	2.70732	4.53967	1.00277	0.664607	0.198502	0.549752	1.56062	0.25318
XLOC_066567	ECE1	chr2	132002935	132030280	2.48738	1.84988	4.6114	2.71286	2.68725	2.03923	1.88512	1.85732	2.86584
XLOC_066568	ECE1	chr2	132052463	132157679	17.3715	20.2941	21.0415	5.66131	5.85424	5.6612	5.52729	7.04104	5.29248
XLOC_050440	ECHDC3	chr13	12565890	12593557	3.84969	2.87811	3.76366	1.98379	2.33087	3.19368	2.17799	0.86536	3.52974
XLOC_070240	ECM1;TARS2	chr3	20215636	20242167	105.574	31.2405	4.48943	0.0	0.0	0.0	0.0	0.0	0.0501745
XLOC_086994	ECM2;CENPP	chr8	85537587	85555390	87.2174	98.1801	84.1414	105.213	106.679	110.069	126.09	111.279	106.635
XLOC_088489	ECM2;CENPP	chr8	85555511	85556445	0.931435	0.835659	2.18556	0.834773	0.509506	0.38643	0.168725	0.186181	0.0813448
XLOC_039985	ECT2	chr1	95498584	95499175	488.875	561.448	393.811	1397.98	1560.46	1532.83	1234.16	993.225	1431.74
XLOC_092511	EDA	chrX	85702541	85776669	2.11002	0.803946	0.335288	3.08683	3.60286	3.78337	4.64648	3.89967	4.44558
XLOC_050878	EDN3;SLMO2;CTSZ;ATP5E;TUBB1	chr13	57429137	57908322	0.99722	0.149177	0.390165	0.223534	0.0	0.155565	0.1821	0.100198	0.174456
XLOC_049360	EDNRB	chr12	53382856	53383719	0.0	0.0	0.0	0.09187	0.0800519	0.0	0.0	0.102342	0.0

XLOC_049361	EDNRB	chr12	53389842	53390476	0.0	0.0	0.0	0.0	0.0	0.156866	0.0	0.0	0.0
XLOC_049362	EDNRB	chr12	53396046	53397781	0.0	0.137172	0.0	0.0	0.0	0.0	0.0	0.0	0.0
XLOC_049365	EDNRB	chr12	53406247	53407574	0.0	0.185	0.0	0.0	0.0	0.0	0.0	0.0	0.0
XLOC_050562	EEF1A2	chr13	54621653	54655481	22.997	22.4076	17.9716	26.6485	25.8466	24.147	25.7691	22.7972	27.3876
XLOC_053947	EFCAB1	chr14	21459300	21459583	0.0	0.0	0.0	1.5366	1.29642	1.15745	5.26933	2.71332	0.985119
XLOC_053948	EFCAB1	chr14	21459852	21460194	0.0	0.0	0.0	0.350167	1.1963	0.0	4.03825	2.64542	1.69264
XLOC_057872	EFCAB2	chr16	32913912	33006107	19.9483	17.6498	15.9212	10.258	12.2112	10.3699	11.9497	10.2873	11.0254
XLOC_057873	EFCAB2	chr16	33008398	33012909	10.2883	8.70741	6.19102	6.07589	4.86761	4.31639	4.39999	4.90505	3.63028
XLOC_057874	EFCAB2	chr16	33016304	33060312	38.7108	48.3966	30.1208	80.4696	81.867	99.2711	97.2911	92.4796	87.949
XLOC_057875	EFCAB2	chr16	33061920	33124828	5.19537	6.10582	6.60922	3.18124	3.44453	3.15011	3.61186	4.73289	4.01003
XLOC_058103	EFCAB2	chr16	33016304	33060312	3.39729	4.95565	2.74843	24.0518	26.8022	24.474	27.204	20.872	41.955
XLOC_058826	EFCAB2	chr16	32913912	33006107	10.5419	7.85656	4.10836	1.41513	2.39509	2.13649	3.10815	2.00803	2.27119
XLOC_058827	EFCAB2	chr16	32913912	33006107	15.1823	3.88704	11.8594	1.94131	0.67127	2.23239	1.72844	1.49468	1.13139
XLOC_058828	EFCAB2	chr16	32913912	33006107	5.98461	5.72906	7.02374	1.60962	1.7819	1.92822	1.19988	0.96092	1.46521
XLOC_058829	EFCAB2	chr16	33006601	33008242	3.90015	3.79236	3.8149	1.18025	1.83521	1.26762	1.06852	0.685875	0.895554
XLOC_058830	EFCAB2	chr16	33008398	33012909	0.0	1.7051	0.0	2.0488	1.72857	1.15745	2.87418	0.542665	1.47768
XLOC_058831	EFCAB2	chr16	33008398	33012909	0.0	9.24329	0.0	3.87809	2.85514	2.85335	2.15827	2.41278	1.87884
XLOC_058832	EFCAB2	chr16	33012977	33013819	20.0761	14.534	20.6584	10.6995	11.7132	10.6223	11.8397	10.5441	11.2529
XLOC_058833	EFCAB2	chr16	33014165	33014643	4.51375	31.0069	14.1021	10.9074	12.0407	10.6798	8.9785	12.2073	12.1614
XLOC_058834	EFCAB2	chr16	33014759	33015789	9.11838	8.67745	5.83583	7.20708	9.40049	7.48479	8.12402	6.22137	11.2253
XLOC_058835	EFCAB2	chr16	33016304	33060312	6.94188	6.20975	10.8229	19.9382	23.4677	18.2002	20.6381	19.5746	26.2647
XLOC_058836	EFCAB2	chr16	33061000	33061391	3.09709	2.77299	2.41706	8.03319	7.37578	7.60892	9.71223	7.53994	10.1994
XLOC_058837	EFCAB2	chr16	33061920	33124828	5.42812	0.0	2.11886	0.485614	0.418229	1.67062	1.42804	1.32734	1.41305
XLOC_058838	EFCAB2	chr16	33061920	33124828	7.03404	2.80089	3.66233	1.88845	1.26817	2.16964	1.86267	2.76411	1.22231
XLOC_065274	EFCAB3	chr19	47496840	47497729	0.0	0.0	0.773293	0.0	0.926748	0.410058	0.268374	0.296245	0.345325
XLOC_065275	EFCAB3	chr19	47499389	47500347	0.0	0.810625	0.0	0.404884	0.494341	0.468636	0.245627	0.0	0.394569
XLOC_064306	EFCAB3;METTL2B	chr19	47501081	47537082	4.73164	6.79635	7.02655	12.7226	15.3527	12.2235	15.9074	15.5496	18.4034
XLOC_037829	EFHB;RAB5A	chr1	44551439	44796853	1.67444	1.00168	0.873708	0.250523	0.329794	0.813172	0.153509	0.0843474	0.366113
XLOC_083628	EFNA2;ATP5D	chr7	45303224	45333792	2.40248	1.18175	0.834975	6.82351	7.90071	7.35323	3.37256	4.44034	7.67766
XLOC_084833	EFNA2;ATP5D	chr7	45303224	45333792	2.84561	0.567326	1.48377	1.78518	2.96453	2.95113	2.14712	1.13736	2.31933

XLOC_084834	EFNA2;ATP5D	chr7	45303224	45333792	1.77064	0.0	1.38369	1.9026	2.61135	2.19235	1.89505	1.22612	1.84995
XLOC_083820	EFNA5	chr7	109036827	109083325	1.78903	1.99937	1.75208	4.29996	4.27597	3.99225	4.51171	3.98797	4.67295
XLOC_085600	EFNA5	chr7	109036827	109083325	1.51158	0.903587	0.0	0.676949	0.823013	0.468649	0.812732	0.600019	1.31736
XLOC_053826	EFR3A	chr14	10208200	10208885	1.37116	1.22963	0.0	0.0	0.0	0.141827	0.0	0.0	0.0
XLOC_038438	EHHADH	chr1	82471023	82491610	8.10878	5.97103	8.78445	0.0	0.0	0.0	0.0	0.0	0.0
XLOC_038439	EHHADH	chr1	82498933	82563469	4.58227	7.31164	9.96002	0.0	0.0	0.0	0.0	0.0	0.0
XLOC_060241	EIF2B1;GTF2H3	chr17	54275904	54298382	0.586807	0.0928234	0.0	0.139093	0.024362	0.161491	0.0568439	0.0588957	0.102291
XLOC_050911	EIF2S2	chr13	64058566	64089400	32.1598	30.5325	11.486	0.313362	0.0547352	0.0726176	0.0	0.490509	0.183325
XLOC_052229	EIF2S2	chr13	64058279	64058506	9.87168	47.179	23.1237	0.0	0.0	0.0	0.0	0.0	0.0
XLOC_052230	EIF2S2	chr13	64058566	64089400	4.86266	7.98657	0.0	0.0	0.0	0.0	0.0	0.0	0.0
XLOC_082774	EIF3G;P2RY11	chr7	15896107	15904064	0.0	0.230259	0.0	1.06214	1.66325	1.42651	1.51586	1.49811	1.61381
XLOC_053583	EIF3H	chr14	49768334	49783456	0.7516	0.899579	0.294103	4.65055	10.531	6.21894	4.95226	2.23117	9.6357
XLOC_076461	EIF3L;ANKRD54;MIR658;MICALL1	chr5	110135720	110223245	26.7949	29.9187	34.6255	21.2384	23.9455	21.8328	24.5395	27.2485	22.1467
XLOC_055786	EIF3M;PRRG4;CCDC73	chr15	64086676	64382754	13.5558	17.4784	15.0307	2.54728	3.28667	3.0449	3.98146	3.50908	2.32878
XLOC_056828	EIF3M;PRRG4;CCDC73	chr15	64086676	64382754	3.09431	0.462409	6.04659	0.277141	0.842173	0.959142	0.692777	0.306936	0.808886
XLOC_056829	EIF3M;PRRG4;CCDC73	chr15	64086676	64382754	3.29416	2.94897	7.71124	0.589207	0.0	1.34738	0.572012	0.960221	0.570608
XLOC_056830	EIF3M;PRRG4;CCDC73	chr15	64086676	64382754	2.59714	6.20362	4.0557	0.92949	0.80118	0.533258	0.684671	0.254385	0.901831
XLOC_056831	EIF3M;PRRG4;CCDC73	chr15	64086676	64382754	3.47828	4.16261	3.49943	0.623747	0.467604	0.620129	1.04352	0.0499278	0.738894
XLOC_056832	EIF3M;PRRG4;CCDC73	chr15	64086676	64382754	3.53454	2.11417	8.29402	0.475185	0.345409	0.275032	0.160217	0.0883653	0.154368
XLOC_056833	EIF3M;PRRG4;CCDC73	chr15	64086676	64382754	0.0	0.810265	0.0	0.0	0.0	0.278436	0.0	0.0	0.23551
XLOC_066499	EIF4E2	chr2	121019323	121036799	0.0	0.167792	0.0	0.0502807	0.0	0.0583057	0.0588683	0.0	0.0562311
XLOC_068415	EIF4E2	chr2	121018623	121019128	0.0	0.0	0.0	0.372651	0.322335	0.0	0.0	0.0	0.0
XLOC_065831	EIF4E2;EIF4E2	chr2	121008372	121016903	13.2404	18.0815	15.9562	14.6318	15.458	14.411	16.0222	17.9087	15.4146
XLOC_065830	EIF4E2;EIF4E2;CHRNG;CHRND	chr2	120977082	121007646	3.67033	2.60822	2.4788	0.209449	0.314393	0.312875	0.122225	0.235149	0.154657
XLOC_084821	ELANE	chr7	44988144	44988572	0.0	0.798319	2.08763	1.67458	1.0304	0.0	0.703928	0.0	1.1603

XLOC_084822	ELANE	chr7	44989974	44990329	0.0	2.18353	0.0	1.63627	1.12	1.12047	0.631596	0.0	1.58302
XLOC_070064	ELAVL4	chr3	96679091	96691072	1.01522	1.09177	0.375196	6.62406	6.8766	5.45615	6.2336	8.27723	10.1363
XLOC_070065	ELAVL4	chr3	96696272	96706084	2.11881	2.39723	3.34593	8.93248	9.6677	8.12491	8.36723	7.56558	10.5182
XLOC_070066	ELAVL4	chr3	96711967	96791510	28.4961	29.6035	25.1919	19.5808	20.1463	20.2676	22.5691	24.0931	23.1265
XLOC_070433	ELAVL4	chr3	96706202	96708538	1.75772	1.54477	1.98598	10.41	9.8487	6.45839	7.16803	5.88251	12.4304
XLOC_070434	ELAVL4	chr3	96711967	96791510	8.48642	9.39595	7.82778	14.3732	13.4863	16.6422	15.2244	17.1678	10.7204
XLOC_071832	ELAVL4	chr3	96679091	96691072	0.0	0.0	0.0	1.58864	0.675987	2.25761	3.78546	1.27882	0.383383
XLOC_071833	ELAVL4	chr3	96711452	96711654	0.0	4.23398	11.0671	5.17257	4.15862	1.4132	4.44531	3.8489	2.42661
XLOC_048085	ELF1	chr12	11196371	11209178	0.254341	0.345176	0.0	0.0456217	0.0900536	0.0	0.0	0.11164	0.0
XLOC_048086	ELF1	chr12	11241127	11266001	3.47584	3.29295	3.22265	9.20726	6.52638	7.54703	7.42506	9.85805	7.63852
XLOC_048460	ELF1	chr12	11237662	11238876	1.36913	0.819099	0.0	0.981893	0.857627	1.2089	0.622876	0.48038	0.837746
XLOC_048461	ELF1	chr12	11240138	11240719	1.70792	1.53113	2.6695	3.36475	1.98954	2.2918	3.66002	5.07251	3.42092
XLOC_048462	ELF1	chr12	11241127	11266001	3.93717	0.0	0.0	4.22475	1.20261	3.61125	2.70583	2.27946	3.06307
XLOC_048463	ELF1	chr12	11241127	11266001	0.0	1.20215	0.0	1.9813	3.58509	1.86562	3.57327	2.38157	1.92528
XLOC_047787	ELF1;WBP4	chr12	11266128	11320254	25.5034	28.111	27.1893	23.07	26.6206	26.3267	31.0159	28.9292	28.9741
XLOC_083370	ELL	chr7	4627942	4629860	8.43833	4.59362	2.9607	1.60131	1.58537	1.31223	1.19589	2.29623	1.08515
XLOC_084051	ELL	chr7	4629980	4630624	0.0	3.11062	1.16216	1.06533	1.27229	0.461012	0.799727	1.62341	0.777462
XLOC_084052	ELL	chr7	4631148	4631882	18.819	18.0038	26.4856	15.8487	11.1476	12.5969	14.6845	19.4834	12.5855
XLOC_083121	ELL2	chr7	97579951	97687090	20.2478	16.1018	17.5652	19.5952	22.2565	26.4994	25.8595	22.2188	22.6881
XLOC_085385	ELL2	chr7	97687164	97693127	2.98977	1.71508	1.85284	2.72646	3.22367	3.2075	3.6309	3.20658	3.57897
XLOC_062396	ELMO3;KIAA0895L;EXOC3L1;E2F4	chr18	34936935	34955192	92.596	99.9806	79.3734	320.032	306.795	318.404	382.489	381.563	349.43
XLOC_060677	ELMOD2	chr17	17491427	17491949	0.0	0.0	0.0	1.95417	1.07638	2.04482	0.881503	1.37059	1.72641
XLOC_060678	ELMOD2	chr17	17493592	17494478	0.0	0.0	0.0	0.622806	1.78352	1.64695	0.538918	0.9915	1.04023
XLOC_060679	ELMOD2	chr17	17501847	17502876	0.0	0.0	0.0	0.74385	0.713953	0.947433	0.376537	0.332183	0.290015
XLOC_070309	ELTD1	chr3	65945813	65958802	84.3117	82.1749	76.3969	62.0277	59.5036	63.2004	77.2438	66.9324	69.5727
XLOC_071374	ELTD1	chr3	65945221	65945358	0.0	30.4294	79.7296	29.8872	82.4735	29.5999	35.6897	8.27417	25.7352
XLOC_071375	ELTD1	chr3	65959070	65959377	4.82142	5.74974	7.5169	2.5877	0.732167	1.46822	1.63476	0.922167	1.24759
XLOC_071376	ELTD1	chr3	65959758	65959956	0.0	9.07157	0.0	4.16406	1.1107	0.0	0.0	0.0	0.0
XLOC_071377	ELTD1	chr3	65974839	65975807	0.0	0.0	0.697985	0.239936	0.209265	0.0925771	0.0	0.089228	0.0
XLOC_080032	EMCN	chr6	25668398	25689572	9.68307	13.627	6.7596	0.0	0.0	0.0	0.0	0.0	0.0

XLOC_063979	EME1;MRPL27;LRRC59;XYLT2	chr19	36926104	37012427	23.8044	31.6041	29.5865	30.1874	25.1294	31.4009	34.132	36.9241	28.5277
XLOC_065044	EME1;MRPL27;LRRC59;XYLT2	chr19	36926104	37012427	6.41059	6.3829	1.66922	1.53016	1.98456	1.53989	2.27203	2.10597	0.929393
XLOC_077628	ENDOU	chr5	32733368	32734078	0.0	1.17406	0.0	0.234557	0.713927	0.948227	0.588735	0.781396	0.570852
XLOC_077629	ENDOU	chr5	32734199	32734698	2.11809	3.16347	0.0	1.32715	0.819759	0.872305	1.12633	1.2519	1.47345
XLOC_076799	ENDOU;RPAP3	chr5	32734860	32785769	12.0432	13.732	12.1452	30.8259	27.02	28.7792	31.1508	35.7006	31.9133
XLOC_058144	ENO1	chr16	45396966	45409053	4.80597	6.70281	6.11778	0.35049	0.151704	0.0	0.0	0.193196	0.170305
XLOC_089495	ENPP1	chr9	70679033	70780503	19.8576	18.3277	17.0353	4.9887	5.68552	6.19856	8.5972	5.39309	5.20109
XLOC_091226	ENPP1	chr9	70780688	70780942	0.0	8.76272	5.72713	0.659922	1.10183	3.70036	0.0	0.0	1.2629
XLOC_091227	ENPP1	chr9	70781090	70781812	1.28127	0.766087	1.00178	0.459155	0.0998436	0.132604	0.230605	0.0	0.111754
XLOC_091228	ENPP1	chr9	70781984	70782900	0.95354	0.285158	2.23737	0.170913	0.223502	0.0988867	0.258989	0.0952741	0.166537
XLOC_091229	ENPP1	chr9	70784323	70784903	1.71196	2.04634	1.33791	0.0	0.265891	0.0	0.152851	0.0	0.149086
XLOC_089980	ENPP3	chr9	70604596	70656876	1.41783	2.96669	1.66459	1.52632	2.03232	1.80605	1.13542	0.713348	1.0544
XLOC_091219	ENPP3	chr9	70592659	70593125	0.0	1.40045	0.0	1.67862	2.71749	1.44642	0.206957	0.921369	2.2409
XLOC_091220	ENPP3	chr9	70594601	70595300	1.3357	1.19788	1.04427	0.358974	1.14443	0.552761	0.960644	0.398506	0.582393
XLOC_091221	ENPP3	chr9	70598926	70601394	1.25206	2.248	2.44984	2.04921	2.33537	2.41174	1.69205	1.29245	2.00008
XLOC_091222	ENPP3	chr9	70601468	70601750	0.0	0.0	0.0	2.06487	3.48322	4.66514	0.482486	1.64005	3.4745
XLOC_091223	ENPP3	chr9	70601851	70602179	8.46958	1.26288	0.0	2.27215	2.9046	3.01675	2.89684	0.814662	3.29197
XLOC_091224	ENPP3	chr9	70602479	70604543	1.51739	1.58912	2.07815	1.56481	1.87591	1.57925	1.11082	0.725266	1.39425
XLOC_091225	ENPP3	chr9	70604596	70656876	0.0	0.0	0.0	0.597336	2.56105	1.02424	0.869159	0.972992	1.15682
XLOC_070239	ENSA;GOLPH3L	chr3	20122041	20155207	3.41051	5.34398	4.41496	2.31678	2.43138	2.79802	1.07451	2.07764	1.68435
XLOC_078733	ENTHD1	chr5	111740698	111740908	0.0	0.0	0.0	0.0	0.919732	1.24679	0.0	0.0	1.06923
XLOC_045141	ENTPD2;NPDC1;NPDC1	chr11	106163316	106193593	11.5821	15.2358	14.2954	19.0554	16.5437	16.046	16.3146	20.4774	15.2326
XLOC_086927	ENTPD4	chr8	71456738	71463776	3.03319	2.38243	1.48362	5.54198	7.82707	6.03747	4.75322	3.39553	5.07639
XLOC_059809	EP400	chr17	46110720	46144147	3.98531	20.6008	24.0348	59.6679	49.7716	59.4042	58.9655	61.826	48.0705
XLOC_060198	EP400	chr17	46110720	46144147	1.3209	1.67873	0.844132	1.43091	1.34787	1.62118	1.01783	1.46314	1.23968
XLOC_061484	EP400	chr17	46110720	46144147	3.44903	3.50772	2.15863	0.432865	0.486085	0.286621	0.251035	0.276599	0.844129
XLOC_044697	EPAS1;PRKCE	chr11	28387658	28583904	0.260385	0.120321	0.0	1.41784	1.5698	1.12856	1.95237	2.46158	0.938551
XLOC_065854	EPB41	chr2	125067830	125114453	9.6538	10.6135	9.26956	4.36238	6.85785	5.6033	6.96198	4.32138	6.38353

XLOC_066514	EPB41	chr2	125067830	125114453	35.2073	38.1624	40.4295	48.6183	46.0279	56.7393	64.6985	50.938	49.4374
XLOC_068474	EPB41	chr2	125067830	125114453	0.458471	0.699712	0.717551	0.212764	0.143858	0.0953842	0.320667	0.0460904	0.207482
XLOC_068475	EPB41	chr2	125067830	125114453	0.524278	2.19594	0.410253	0.141044	0.164419	0.327092	0.382762	0.0	0.41269
XLOC_068476	EPB41	chr2	125067830	125114453	3.39979	0.0	3.98552	0.0	0.132033	0.526417	0.151806	0.0	0.148041
XLOC_068477	EPB41	chr2	125067830	125114453	0.0	0.879467	4.59892	1.5812	2.49233	1.50912	2.83016	0.574767	2.55434
XLOC_068478	EPB41	chr2	125067830	125114453	3.14109	3.44575	2.86751	2.30002	3.24252	2.8307	3.05771	1.78789	3.4799
XLOC_068479	EPB41	chr2	125067830	125114453	4.08252	0.813658	2.12783	1.82866	4.02709	2.95614	2.69024	2.02941	2.25453
XLOC_068480	EPB41	chr2	125067830	125114453	3.13945	4.46035	6.13976	4.0101	4.6668	5.45912	6.77164	4.00774	4.04627
XLOC_068481	EPB41	chr2	125067830	125114453	7.13851	4.25766	11.1303	5.12823	5.89401	3.59754	2.35317	4.02035	4.29608
XLOC_068482	EPB41	chr2	125067830	125114453	2.22907	3.32896	0.0	2.19464	3.96499	4.81626	4.73187	3.07005	3.29381
XLOC_065691	EPB41L5	chr2	72174355	72195786	0.0	0.821311	3.22194	1.47675	0.213942	0.284188	0.370247	0.40967	0.0
XLOC_065692	EPB41L5	chr2	72205676	72226044	4.73535	0.707084	0.0	0.847532	0.0	0.973616	0.0	3.25556	0.0
XLOC_086894	EPB49	chr8	69887639	69889527	0.0	1.28308	0.0	2.97333	4.21293	2.1022	1.79774	0.710661	1.62151
XLOC_086895	EPB49	chr8	69894169	69903123	204.546	175.418	151.911	271.761	304.517	265.071	244.679	203.655	294.636
XLOC_087977	EPB49	chr8	69882527	69883496	0.0	1.06619	0.0	0.15976	0.0	0.184927	0.0	0.0891192	0.0
XLOC_087978	EPB49	chr8	69891777	69892347	0.0	0.523966	0.0	2.19821	1.08899	0.723772	0.93863	0.0	0.305355
XLOC_037800	EPHA3	chr1	36767524	36782986	0.0	0.444162	0.0	5.44622	6.74907	4.85797	4.00191	3.61224	9.18702
XLOC_037801	EPHA3	chr1	37100480	37157409	0.0	0.0	0.0	14.4213	13.6323	14.3357	16.0617	17.0901	17.3927
XLOC_038316	EPHA3	chr1	36792208	36939823	47.0645	64.5355	47.0314	36.2343	38.1753	34.1558	49.9687	50.6071	32.9401
XLOC_039130	EPHA3	chr1	36791497	36792087	0.0	0.499773	0.0	1.79719	1.29913	1.72661	0.74712	1.65636	1.01961
XLOC_039131	EPHA3	chr1	36792208	36939823	6.19418	1.84868	2.4171	6.37115	6.66199	2.85335	6.7446	6.6353	4.29449
XLOC_039132	EPHA3	chr1	36792208	36939823	2.91041	2.60628	6.81532	5.20682	5.59754	5.07017	3.30644	6.53278	6.81436
XLOC_039133	EPHA3	chr1	36792208	36939823	1.3896	1.24616	0.0	3.73438	3.89487	3.73667	4.61768	5.80045	5.69428
XLOC_039134	EPHA3	chr1	36985113	36985958	0.0	0.314579	0.0	2.82819	1.889	1.3084	2.37682	1.99476	1.56121
XLOC_089428	EPHA7	chr9	57701949	57702643	0.0	1.37422	35.9321	0.0	0.0	0.0	0.0	0.0	0.0
XLOC_089429	EPHA7	chr9	57708556	57750341	6.84091	7.56622	5.53429	6.4034	8.04954	8.39067	9.95012	8.19734	5.46059
XLOC_089926	EPHA7	chr9	57626554	57652605	19.985	22.6656	23.3354	14.9363	13.983	13.395	13.6284	14.6116	11.1709
XLOC_089927	EPHA7	chr9	57750537	57836841	52.6873	71.0386	60.5547	30.3594	32.0691	32.0649	31.8921	29.7951	30.8263
XLOC_090940	EPHA7	chr9	57625003	57626142	1.47376	2.64494	1.15293	0.132109	0.346041	0.0	0.0669824	0.0	0.193199
XLOC_090941	EPHA7	chr9	57750537	57836841	17.0445	16.5468	24.9647	2.47919	3.29803	3.50958	3.58692	2.09848	3.14942
XLOC_038068	EPHB1	chr1	135224395	135233408	77.4322	86.813	59.0541	287.876	263.428	322.486	398.762	413.077	240.908
XLOC_038555	EPHB1	chr1	135253511	135261512	1.59518	1.167	0.583597	34.6391	26.2675	31.9217	28.1286	17.003	27.3516
XLOC_038556	EPHB1	chr1	135262705	135284378	7.65265	6.65455	4.44642	29.396	23.8975	30.9203	17.5948	22.0352	20.1446

XLOC_038557	EPHB1	chr1	135301367	135313792	34.1561	30.6031	37.3712	62.3236	57.7956	64.2079	61.0275	66.994	62.1644
XLOC_038558	EPHB1	chr1	135371177	135374924	9.20167	5.97388	12.7349	0.664171	0.688271	1.52353	1.36224	0.599557	0.947589
XLOC_038559	EPHB1	chr1	135382241	135430508	17.0872	18.6401	24.5472	50.8661	48.4046	51.1164	48.7672	54.9238	41.1093
XLOC_038560	EPHB1	chr1	135437436	135688152	14.0204	9.88074	7.45267	7.19421	6.39204	8.05256	9.88091	13.1139	8.06705
XLOC_040383	EPHB1	chr1	135296110	135296478	6.86911	1.02478	0.0	2.45714	3.1584	3.50933	1.78487	1.33277	2.37866
XLOC_040384	EPHB1	chr1	135297663	135298027	3.50084	3.13351	0.0	4.06982	2.14521	1.7879	0.605769	1.01812	2.12106
XLOC_040385	EPHB1	chr1	135300602	135301211	0.0	0.0	0.0	2.00862	0.871605	1.4892	2.29311	0.635205	0.697822
XLOC_040386	EPHB1	chr1	135370360	135371033	1.40308	1.25822	1.09687	0.125685	0.218457	0.0	0.0	0.0	0.0
XLOC_040387	EPHB1	chr1	135380114	135380934	2.18345	1.30575	1.70749	0.684786	1.19284	0.22624	0.394354	0.544482	0.571726
XLOC_040388	EPHB1	chr1	135434589	135436401	0.874259	1.70028	1.71039	0.313574	0.617323	0.454785	0.639318	0.351638	0.803139
XLOC_040389	EPHB1	chr1	135436919	135437363	0.0	1.50771	0.0	0.9036	1.1688	0.518596	1.55492	1.48498	1.31532
XLOC_040390	EPHB1	chr1	135437436	135688152	0.0	0.0	0.0	0.641586	0.274607	0.732497	0.619851	0.694859	0.310415
XLOC_040391	EPHB1	chr1	135437436	135688152	0.0	0.625385	0.0	1.31182	0.0	0.862335	0.74252	0.618886	0.546193
XLOC_040392	EPHB1	chr1	135437436	135688152	1.6844	0.50326	1.31614	0.301622	0.130808	0.695419	0.0	0.16677	0.0
XLOC_040393	EPHB1	chr1	135437436	135688152	0.0	0.0	0.0	0.580657	1.67324	0.0	0.382994	0.212903	0.564017
XLOC_040394	EPHB1	chr1	135437436	135688152	1.36927	0.0	0.0	0.0613688	0.107203	0.142224	0.311438	0.274503	0.299195
XLOC_040395	EPHB1	chr1	135437436	135688152	0.0	0.0	0.0	0.0	0.277277	0.369834	0.312835	0.0	0.31348
XLOC_040396	EPHB1	chr1	135437436	135688152	0.0	0.0	1.77938	0.203894	0.528368	0.468664	1.00665	0.895965	0.19799
XLOC_040397	EPHB1	chr1	135437436	135688152	0.0	0.0	0.0	0.287584	0.249576	0.33166	0.143628	0.0	0.419617
XLOC_040398	EPHB1	chr1	135437436	135688152	0.0	0.171109	0.0	0.205118	0.627549	0.594557	0.156451	0.287082	0.300097
XLOC_040399	EPHB1	chr1	135437436	135688152	0.0	0.0	0.0	0.230557	0.201134	0.444872	0.155535	0.0857737	0.374516
XLOC_040400	EPHB1	chr1	135437436	135688152	0.0	0.0	0.0	0.588202	1.40342	0.508671	0.0	0.488064	0.858175
XLOC_040401	EPHB1	chr1	135437436	135688152	0.0	0.0	0.0	0.387938	1.1506	0.269465	0.0785006	0.606104	0.529328
XLOC_040402	EPHB1	chr1	135437436	135688152	0.0	0.359852	0.941168	0.485296	0.5655	0.625098	0.0548094	0.181065	0.105179
XLOC_040403	EPHB1	chr1	135437436	135688152	0.0	0.0	0.0	0.618678	0.0	0.715177	0.207619	0.114705	0.301264
XLOC_040404	EPHB1	chr1	135437436	135688152	0.824041	0.0	1.28962	0.295541	0.452607	0.60016	0.226057	0.372948	0.180238
XLOC_040405	EPHB1	chr1	135437436	135688152	0.0	0.0	0.0	2.24603	0.957387	0.0	1.07465	0.0	0.0
XLOC_040406	EPHB1	chr1	135437436	135688152	0.0	0.611258	0.0	0.54951	0.316952	0.421532	0.181584	0.0	0.177974
XLOC_040407	EPHB1	chr1	135437436	135688152	0.0	0.469429	1.22767	0.281348	0.366335	0.486791	0.562393	0.0	0.68426
XLOC_065884	EPHB2	chr2	130417659	130554373	47.8682	58.4136	48.5485	57.607	59.0932	58.2688	69.417	61.7552	66.9187
XLOC_065885	EPHB2	chr2	130563538	130565350	9.00994	10.7671	13.5466	21.471	17.7501	21.0321	22.4304	24.0602	14.246
XLOC_065886	EPHB2	chr2	130667666	130681278	13.6626	22.4136	17.3832	44.5334	36.5858	37.433	41.2653	44.7872	41.4254
XLOC_066552	EPHB2	chr2	130417659	130554373	4.49518	3.14938	4.23614	4.01541	3.38765	3.47051	3.50772	3.45667	2.56301

XLOC_066554	EPHB2	chr2	130633612	130637973	0.92855	1.50142	1.81704	2.00583	3.21858	2.93197	1.89243	1.65265	2.41871
XLOC_066555	EPHB2	chr2	130653852	130664639	29.6688	30.8274	21.133	19.5509	22.0518	18.3291	23.4368	21.4353	20.8718
XLOC_066556	EPHB2	chr2	130683546	130685892	1.40387	1.0476	1.09857	4.4112	3.89334	3.36952	2.90159	3.21256	2.71867
XLOC_066557	EPHB2	chr2	130686032	130697926	1.621	1.77957	2.15131	8.26748	7.64767	7.97025	3.75097	6.17299	5.58432
XLOC_068609	EPHB2	chr2	130556161	130556629	2.32955	1.39145	1.8194	0.208479	1.08012	0.23954	0.617038	0.2289	0.202417
XLOC_068610	EPHB2	chr2	130589081	130589897	0.0	1.64209	0.858926	0.492101	0.600009	1.13802	0.297526	0.109549	0.383457
XLOC_068611	EPHB2	chr2	130590125	130590929	4.47499	5.01763	1.7497	1.70417	1.39668	1.5067	1.4137	2.78887	1.85509
XLOC_068614	EPHB2	chr2	130632890	130633518	0.0	2.29732	1.20162	2.89145	4.42328	3.33579	2.2031	2.13511	4.55462
XLOC_068615	EPHB2	chr2	130667666	130681278	0.0	6.54173	6.84206	3.13902	2.33832	2.67731	4.49158	2.52828	2.27295
XLOC_068616	EPHB2	chr2	130667666	130681278	2.63497	2.36016	2.05732	2.59325	2.03139	1.89322	0.925538	2.83774	1.82969
XLOC_068617	EPHB2	chr2	130681423	130681751	8.46958	10.1031	6.60433	15.9051	14.8457	10.3432	23.8989	17.1079	17.9229
XLOC_068618	EPHB2	chr2	130681860	130683154	0.636377	0.0	0.497895	0.684614	0.44863	0.46288	0.405683	0.382996	0.278182
XLOC_068619	EPHB2	chr2	130705980	130707815	8.19158	6.1919	6.07302	1.85566	1.75901	1.79426	1.6948	1.51743	1.92378
XLOC_066558	EPHB2;C1QB;C1QC;C1QA	chr2	130707993	130840129	14.3261	16.7018	16.9496	3.7572	3.77786	3.42604	4.10407	3.93824	4.78914
XLOC_066553	EPHB2;EPHB2	chr2	130591085	130630191	10.7351	11.0443	9.18757	16.9576	15.9489	16.8371	15.726	17.4052	17.3128
XLOC_068612	EPHB2;EPHB2	chr2	130591085	130630191	0.799618	1.9133	1.87651	0.573404	0.625519	0.415017	0.290402	0.400228	0.628804
XLOC_068613	EPHB2;EPHB2	chr2	130591085	130630191	0.0	2.47949	3.70553	1.06152	1.01631	0.613425	1.49525	1.06234	1.24045
XLOC_072736	EPHB6;TRPV6	chr4	106910406	107048490	61.4715	82.9881	66.4358	29.5946	26.2172	31.2071	23.8837	30.4773	21.8346
XLOC_057850	EPHX1;TMEM63A	chr16	29579259	29662926	17.513	16.235	16.256	31.8264	28.1312	29.9534	34.8008	34.5011	30.7623
XLOC_058727	EPHX1;TMEM63A	chr16	29579259	29662926	1.64185	3.27463	2.56939	7.7038	7.97939	7.62468	8.53698	8.40836	8.51961
XLOC_069901	EPHX4	chr3	51483997	51494985	5.2558	10.357	6.05944	3.26789	3.82451	3.79483	3.50433	3.18485	3.21958
XLOC_071031	EPHX4	chr3	51495119	51496016	3.91217	6.72699	9.94416	2.2789	2.9796	2.73813	1.77015	3.22386	1.62261
XLOC_071032	EPHX4	chr3	51515310	51518438	3.16507	4.08016	3.23961	2.86049	2.77408	3.11937	1.42949	1.03068	1.51345
XLOC_071033	EPHX4	chr3	51518581	51518921	7.91718	11.807	9.26197	12.7432	11.183	8.87457	4.07943	4.20044	2.3952
XLOC_071034	EPHX4	chr3	51519086	51521115	6.18308	6.47532	5.74615	4.05448	3.39712	3.82069	2.22753	1.43865	2.53623
XLOC_089540	EPM2A	chr9	83925139	84124358	12.1444	11.9452	9.33529	35.1755	26.884	32.6624	32.9297	37.2098	34.4885
XLOC_084292	EPOR	chr7	16999900	17000289	0.0	0.0	0.0	0.0	0.479864	0.0	0.271986	0.0	0.0
XLOC_070041	EPS15	chr3	95427577	95497507	11.8314	13.0038	10.8636	15.4324	19.1219	18.2984	20.0349	18.2025	14.3427

XLOC_070042	EPS15	chr3	95498719	95509386	3.52413	1.92164	2.06947	0.508573	0.385479	0.576334	0.782462	0.354453	0.895898
XLOC_070043	EPS15	chr3	95526842	95556391	25.6355	4.69772	8.5242	1.2828	0.316228	1.66482	0.525257	0.38569	0.39221
XLOC_044229	EPT1	chr11	73135324	73147426	2.80277	2.87586	2.97739	9.03107	7.77678	7.98878	9.07873	10.5985	6.98509
XLOC_044230	EPT1	chr11	73170357	73172184	3.77766	0.756715	0.0	0.715772	1.15243	1.29188	1.43743	0.842276	0.186018
XLOC_046541	EPT1	chr11	73133647	73134252	0.0	2.41523	1.26328	1.8818	0.376841	1.16852	1.73475	0.961157	0.563219
XLOC_046542	EPT1	chr11	73172249	73172732	0.0	1.99119	0.0	0.397784	1.7187	0.457302	0.78632	1.53043	0.965814
XLOC_046543	EPT1	chr11	73173321	73174246	0.942358	0.281817	2.94822	0.591187	1.69358	1.36827	0.512009	1.41255	0.822954
XLOC_077347	EPYC	chr5	20924562	20925163	4.89267	0.974791	2.54931	1.60663	1.01386	1.01053	2.6249	0.484828	0.426202
XLOC_077348	EPYC	chr5	20925371	20925632	0.0	0.0	0.0	1.23459	1.55062	1.38746	4.54725	6.46827	0.591519
XLOC_077349	EPYC	chr5	20926642	20927591	0.0	0.546557	0.714725	1.06466	0.357086	0.663509	0.579536	0.45673	0.718274
XLOC_077350	EPYC	chr5	20933840	20935049	2.75129	0.822992	1.61435	0.431619	0.484696	1.07173	0.81354	0.344746	0.0601229
XLOC_077351	EPYC	chr5	20940681	20941265	6.78362	1.01359	0.0	0.911216	0.922009	1.92572	0.151461	0.671675	0.590783
XLOC_077352	EPYC	chr5	20944535	20944772	0.0	2.61764	0.0	1.58152	1.96313	0.0	1.42436	9.7802	0.0
XLOC_077353	EPYC	chr5	20945029	20945445	5.59588	2.50578	0.0	4.00479	2.5852	3.15596	1.95994	1.09363	1.69906
XLOC_077354	EPYC	chr5	20949690	20950487	1.13099	0.676333	0.0	1.7228	0.882394	1.05444	1.02061	1.12759	0.592205
XLOC_083767	ERAP1	chr7	98590612	98648269	28.9591	28.0769	26.352	26.4445	27.2733	27.8309	25.2739	29.5311	25.7555
XLOC_085403	ERAP1	chr7	98585476	98586079	3.24713	1.45563	2.53788	1.01782	1.51404	0.670691	0.58077	0.80449	0.707166
XLOC_085404	ERAP1	chr7	98588690	98589009	4.46815	1.33231	10.451	6.3935	3.40002	6.81344	5.32989	5.14519	2.31422
XLOC_085405	ERAP1	chr7	98589161	98590474	15.0219	9.92373	12.7325	12.5133	11.4735	13.2687	12.8279	14.5047	12.0939
XLOC_085406	ERAP1	chr7	98590612	98648269	1.77523	0.530432	0.0	2.22523	0.964404	1.83147	2.84935	1.58026	1.7
XLOC_085407	ERAP1	chr7	98590612	98648269	11.2632	6.71948	17.5701	3.69294	6.31496	5.36154	4.20502	1.81473	3.89648
XLOC_085408	ERAP1	chr7	98590612	98648269	2.95364	2.20864	2.88823	2.71379	1.96488	1.68692	0.738301	0.961647	1.67783
XLOC_085409	ERAP1	chr7	98590612	98648269	0.0	5.03762	0.0	4.53937	2.98035	2.85067	5.19498	1.6045	3.88127
XLOC_085410	ERAP1	chr7	98590612	98648269	0.0	4.82753	0.0	2.89369	2.23468	4.30234	2.81169	3.14574	4.20407
XLOC_083125	ERAP2	chr7	98701867	98717162	21.4335	23.9227	23.4793	20.0343	17.6972	20.882	22.3253	25.6338	22.0491
XLOC_085417	ERAP2	chr7	98717342	98718791	6.71998	6.53379	2.19086	5.7739	4.60866	4.59914	4.90286	5.28508	4.16267
XLOC_076449	ERC1	chr5	108312800	108349341	23.7782	9.74679	15.1867	11.4149	12.2541	11.3758	8.04406	12.0546	13.6292
XLOC_076450	ERC1	chr5	108448986	108504533	20.017	22.7397	22.5326	15.3864	15.4914	16.7828	17.1088	16.4707	14.3201
XLOC_076955	ERC1	chr5	108430203	108432501	0.851608	0.382209	0.52887	0.307509	0.735991	0.175871	0.583288	0.428218	0.335613
XLOC_076956	ERC1	chr5	108433215	108440621	50.8042	67.8571	50.1031	194.969	171.046	185.759	217.514	237.594	200.268
XLOC_076957	ERC1	chr5	108441464	108443475	0.0	0.0	0.722938	0.0828378	0.216698	0.191742	0.0837269	0.0	0.161446
XLOC_076958	ERC1	chr5	108443538	108444783	0.0	0.0	0.0	0.0	0.0	0.0	0.225132	0.0	0.0
XLOC_076959	ERC1	chr5	108445224	108448698	0.251336	0.451286	0.0	0.0225414	0.0789946	0.104716	0.138325	0.0	0.088018

XLOC_076960	ERC1	chr5	108518558	108548758	0.354609	1.06109	0.832577	0.540602	1.30848	0.922805	1.52582	0.606789	1.48965
XLOC_078595	ERC1	chr5	108366124	108366892	2.36945	4.25055	6.48467	0.0	0.0	0.0	0.0	0.0	0.0
XLOC_078596	ERC1	chr5	108426814	108428687	0.843171	0.126142	0.0	0.0756072	0.0330803	0.131597	0.0	0.1272	0.0
XLOC_078597	ERC1	chr5	108448986	108504533	1.27245	3.04611	2.24075	1.8258	1.64881	1.58979	2.15658	1.15324	1.6428
XLOC_078598	ERC1	chr5	108448986	108504533	28.8222	6.87352	4.49297	1.03256	0.870913	2.3327	1.93017	2.73362	2.97838
XLOC_078599	ERC1	chr5	108448986	108504533	5.09523	1.52432	3.10083	0.862894	1.24239	1.00051	0.826029	0.966236	1.28724
XLOC_078600	ERC1	chr5	108448986	108504533	4.26117	3.18211	3.32873	0.953583	1.64906	0.438736	1.1328	1.25917	1.85266
XLOC_078601	ERC1	chr5	108448986	108504533	3.08588	1.15376	1.81054	0.691539	0.845106	0.881068	0.981171	1.15885	0.674168
XLOC_078602	ERC1	chr5	108448986	108504533	7.41753	2.21224	17.3498	2.66605	2.22466	0.0	2.43863	2.08508	0.0
XLOC_078603	ERC1	chr5	108504900	108505681	1.16002	1.38734	7.2567	1.8709	1.08579	2.16262	2.30173	1.27173	2.53058
XLOC_078604	ERC1	chr5	108517110	108517539	0.0	0.795387	8.31986	4.05191	1.64269	3.82748	4.67639	3.38955	2.77457
XLOC_062241	ERCC1	chr18	53463703	53464684	0.0	0.676168	0.0	0.0	0.0	0.0	0.0	0.0	0.0
XLOC_062242	ERCC1;RTN2;VASP;FOSB;RTN2;PPM1N;OPA3	chr18	53465012	53576550	0.308473	0.0772543	0.248942	0.227966	0.0249537	0.0993596	0.0	0.032006	0.0278156
XLOC_063217	ERCC1;RTN2;VASP;FOSB;RTN2;PPM1N;OPA3	chr18	53465012	53576550	0.0	0.0	0.0	0.0	0.0	0.0	0.0	0.168694	0.0
XLOC_063218	ERCC1;RTN2;VASP;FOSB;RTN2;PPM1N;OPA3	chr18	53465012	53576550	0.0	0.0792046	0.0	0.0	0.0	0.0275668	0.0	0.0	0.0
XLOC_063221	ERCC1;RTN2;VASP;FOSB;RTN2;PPM1N;OPA3	chr18	53465012	53576550	0.0	0.0	1.70954	0.0	0.0	0.0	0.0	0.0	0.0
XLOC_063222	ERCC1;RTN2;VASP;FOSB;RTN2;PPM1N;OPA3	chr18	53465012	53576550	0.0	0.0	0.0	0.0	0.0	0.0	0.356301	0.0	0.0
XLOC_063223	ERCC1;RTN2;VASP;FOSB;RTN2;PPM1N;OPA3	chr18	53465012	53576550	0.0	0.0	0.0	0.0	0.0	0.0420338	0.0	0.0	0.0
XLOC_063225	ERCC1;RTN2;VASP;FOSB;RTN2;PPM1N;OPA3	chr18	53465012	53576550	0.0	0.0	0.0	0.0	0.0	0.0	0.123651	0.0	0.0

XLOC_063229	ERCC1;RTN2;VASP;FOSB;RTN2;PPM1N;OPA3	chr18	53465012	53576550	0.0	0.0	0.0	0.13144	0.0	0.0	0.0	0.0	0.0
XLOC_063231	ERCC1;RTN2;VASP;FOSB;RTN2;PPM1N;OPA3	chr18	53465012	53576550	0.0	0.29894	0.0	0.0	0.0	0.0	0.0	0.0	0.0
XLOC_063238	ERCC1;RTN2;VASP;FOSB;RTN2;PPM1N;OPA3	chr18	53465012	53576550	0.0	0.0	0.0	0.0	0.0	0.0	0.0	0.0	0.227073
XLOC_065469	ERCC3	chr2	5137997	5230901	0.707134	2.24511	1.66027	1.5139	0.491027	0.95837	1.19714	3.49455	1.09508
XLOC_038594	ERG	chr1	152333677	152386695	5.76328	8.6006	5.50279	9.12945	9.19421	10.5899	8.99345	8.54939	10.2258
XLOC_040609	ERG	chr1	152333677	152386695	0.463165	0.554308	0.0	0.539893	0.581292	0.289083	1.18487	0.41902	0.445715
XLOC_040610	ERG	chr1	152333677	152386695	2.48114	2.22624	1.29389	2.29803	1.29367	1.80257	2.77696	0.910402	1.73416
XLOC_042079	ERH	chr10	81512340	81524137	3.73787	0.0	0.0	1.11629	0.194238	0.386931	0.0	0.248092	0.217376
XLOC_042080	ERH;SLC39A9	chr10	81528635	81545012	12.5618	17.4295	10.9051	12.1268	9.89018	11.5159	12.1353	15.2658	10.9188
XLOC_066273	ERMN;GALNT5	chr2	38723344	38926360	8.08012	10.403	11.5222	17.0264	11.456	15.7524	9.04794	16.4149	9.39219
XLOC_067382	ERMN;GALNT5	chr2	38723344	38926360	1.71602	0.0	2.68218	0.0	0.133257	0.35424	0.612808	0.169869	0.149437
XLOC_067383	ERMN;GALNT5	chr2	38723344	38926360	13.0415	0.0	0.0	0.0	0.0	0.0	0.0	0.0	0.0
XLOC_067384	ERMN;GALNT5	chr2	38723344	38926360	0.0	0.37508	0.980974	0.786816	0.0977861	0.389595	0.0	0.0	0.328316
XLOC_067385	ERMN;GALNT5	chr2	38723344	38926360	1.84109	0.367176	0.960336	0.0550191	0.14424	0.191334	0.0559128	0.0615749	0.053658
XLOC_067386	ERMN;GALNT5	chr2	38723344	38926360	0.995544	1.78624	1.55724	0.089217	0.0777563	0.103216	0.0	0.198839	0.0869233
XLOC_067387	ERMN;GALNT5	chr2	38723344	38926360	1.81154	0.812654	1.41695	0.0811794	0.141591	0.0939606	0.0820772	0.0	0.0791108
XLOC_067388	ERMN;GALNT5	chr2	38723344	38926360	0.0	1.49932	1.30705	0.149766	0.259825	0.172661	0.149424	0.165636	0.291318
XLOC_067389	ERMN;GALNT5	chr2	38723344	38926360	1.1131	0.832511	1.30644	0.199596	0.218115	0.231439	0.0507603	0.0	0.0973434
XLOC_067390	ERMN;GALNT5	chr2	38723344	38926360	0.0	0.0	4.48959	0.257245	0.0	0.589501	0.0	0.0	0.997623
XLOC_067391	ERMN;GALNT5	chr2	38723344	38926360	0.0	0.807245	4.22198	0.0	0.208341	0.0	0.0	0.0	0.234637
XLOC_067392	ERMN;GALNT5	chr2	38723344	38926360	2.6963	0.806021	0.0	0.120772	0.0	0.139467	0.0	0.0	0.0

XLOC_067393	ERMN;GALNT5	chr2	38723344	38926360	0.0	1.55683	0.0	0.311019	0.134835	0.537671	0.309966	0.0	0.0
XLOC_067394	ERMN;GALNT5	chr2	38723344	38926360	3.41837	2.03992	0.0	0.0	0.0	0.0	0.0	0.0	0.295946
XLOC_067395	ERMN;GALNT5	chr2	38723344	38926360	2.01069	1.72993	1.37707	0.540993	0.197486	0.366508	0.138325	0.126654	0.24205
XLOC_067396	ERMN;GALNT5	chr2	38723344	38926360	2.00089	2.39101	4.68971	0.537371	0.310049	0.824634	0.0	0.197385	0.348127
XLOC_067397	ERMN;GALNT5	chr2	38723344	38926360	0.0	0.0	19.8528	1.52854	0.0	2.55861	0.0	1.5792	0.0
XLOC_067398	ERMN;GALNT5	chr2	38723344	38926360	7.79627	6.97656	0.0	0.701148	0.58368	2.35424	0.0	0.0	0.669967
XLOC_067399	ERMN;GALNT5	chr2	38723344	38926360	1.89598	1.13294	1.48146	0.509251	0.294039	0.0	0.168714	0.0	0.33
XLOC_067400	ERMN;GALNT5	chr2	38723344	38926360	2.6849	1.4055	1.57543	0.481384	0.262813	0.488121	0.305444	0.336496	0.293378
XLOC_067401	ERMN;GALNT5	chr2	38723344	38926360	7.07177	2.10872	0.0	0.0	0.0	0.0	0.0	0.0	0.608027
XLOC_067402	ERMN;GALNT5	chr2	38723344	38926360	7.79003	2.32357	0.0	0.348292	0.297517	0.0	0.0	0.0	0.336736
XLOC_067403	ERMN;GALNT5	chr2	38723344	38926360	1.81965	3.26224	5.68764	0.0	0.42356	0.37539	0.324294	0.359801	0.475204
XLOC_067404	ERMN;GALNT5	chr2	38723344	38926360	1.93702	1.7386	3.78938	0.521043	0.455742	0.302199	0.22113	0.19466	0.0423604
XLOC_067405	ERMN;GALNT5	chr2	38723344	38926360	0.0	1.4862	3.88599	0.445999	0.378153	0.505703	0.421595	0.0	0.429843
XLOC_067406	ERMN;GALNT5	chr2	38723344	38926360	0.0	5.11914	6.69128	0.772912	1.28071	0.0	0.0	0.0	0.0
XLOC_067407	ERMN;GALNT5	chr2	38723344	38926360	0.0	0.0	4.08126	0.0	0.270309	0.0	0.0	0.172271	0.0
XLOC_067408	ERMN;GALNT5	chr2	38723344	38926360	1.527	2.05609	2.9876	0.410797	0.359572	0.357576	0.209589	0.15365	0.400877
XLOC_067409	ERMN;GALNT5	chr2	38723344	38926360	1.80027	2.4228	0.704078	0.403373	0.281433	0.280138	0.0815749	0.26999	0.550341
XLOC_067410	ERMN;GALNT5	chr2	38723344	38926360	1.27838	2.1033	2.50048	0.171909	0.0500669	0.730585	0.0582078	0.192338	0.0558817
XLOC_067411	ERMN;GALNT5	chr2	38723344	38926360	1.50216	1.34695	4.69686	0.941829	0.584247	0.621007	0.0	0.149087	0.785492
XLOC_067412	ERMN;GALNT5	chr2	38723344	38926360	0.0	2.47683	6.47506	0.747512	1.24069	2.50445	1.35341	0.773474	0.0
XLOC_067413	ERMN;GALNT5	chr2	38723344	38926360	4.91483	3.9168	3.84129	0.586871	0.509194	0.338354	0.146469	0.0	0.0
XLOC_067414	ERMN;GALNT5	chr2	38723344	38926360	0.0	4.02124	8.41257	0.964013	0.622745	0.829188	0.70892	0.527108	0.467544

XLOC_067415	ERMN;GALNT5	chr2	38723344	38926360	8.93631	1.33231	6.9674	0.0	0.680004	0.454229	0.0	0.428766	0.385703
XLOC_067416	ERMN;GALNT5	chr2	38723344	38926360	0.0	5.49687	3.5933	1.64889	0.700819	0.468261	0.783777	0.883339	0.397729
XLOC_067417	ERMN;GALNT5	chr2	38723344	38926360	0.0	1.34049	7.01016	0.0	0.342034	0.0	0.382892	0.0	0.38805
XLOC_067418	ERMN;GALNT5	chr2	38723344	38926360	14.0933	11.205	0.0	1.26055	1.42785	3.81679	0.0	1.34936	1.21592
XLOC_067419	ERMN;GALNT5	chr2	38723344	38926360	0.757725	1.5865	2.96382	0.543372	0.533655	0.314702	0.137701	0.0758913	0.397297
XLOC_067420	ERMN;GALNT5	chr2	38723344	38926360	5.99713	16.0898	9.34834	1.61179	1.35736	3.6375	0.500531	0.0	0.0
XLOC_067421	ERMN;GALNT5	chr2	38723344	38926360	6.33571	0.63085	0.0	0.0	0.163481	0.0	0.0	0.0	0.0
XLOC_067422	ERMN;GALNT5	chr2	38723344	38926360	0.878271	2.10134	2.74792	0.314868	0.0686615	0.455618	0.0	0.263504	0.306867
XLOC_067423	ERMN;GALNT5	chr2	38723344	38926360	0.0	0.0	3.94283	0.2259	0.0	0.0	0.222131	0.0	0.43844
XLOC_067424	ERMN;GALNT5	chr2	38723344	38926360	4.36397	2.60696	5.11322	0.585899	0.67525	0.449135	0.0	1.28868	0.758775
XLOC_067425	ERMN;GALNT5	chr2	38723344	38926360	0.0	0.0	2.67972	0.921427	0.2632	0.350933	0.297478	0.0	0.891998
XLOC_042337	ERO1L	chr10	11458188	11458832	5.94689	1.7775	2.32431	1.06533	2.66024	2.45873	3.99864	0.590329	4.01689
XLOC_041457	ERO1L;PSMC6;GNPNAT1;STYX	chr10	11343487	11458049	0.0	1.49177	1.95082	0.715309	2.85429	0.570405	1.86653	0.651285	2.39876
XLOC_048093	ESD	chr12	16671645	16800984	2.89463	2.09918	2.33235	0.109835	0.140132	0.341541	0.0921538	0.113533	0.13589
XLOC_048500	ESD	chr12	16671645	16800984	0.0	0.0	0.638194	0.0	0.0	0.0	0.0	0.0	0.0
XLOC_048502	ESD	chr12	16671645	16800984	0.0	0.0	0.970857	0.0	0.0	0.0	0.0	0.0	0.0
XLOC_048507	ESD	chr12	16671645	16800984	0.0	0.340829	0.89138	0.0	0.0	0.0	0.0	0.0	0.0
XLOC_048508	ESD	chr12	16671645	16800984	1.95228	0.0	0.0	0.0	0.0	0.0	0.0	0.0	0.0
XLOC_048510	ESD	chr12	16671645	16800984	0.0	3.31936	8.67651	0.0	0.0	0.0	0.0	0.0	0.0
XLOC_048511	ESD	chr12	16671645	16800984	0.0	0.0	0.0	0.0	0.0	0.0	0.833621	0.0	0.891864
XLOC_048512	ESD	chr12	16671645	16800984	0.0	0.0	0.0	0.0	0.35477	0.0	0.0	0.0	0.0
XLOC_048513	ESD	chr12	16671645	16800984	3.72362	0.0	0.0	0.0	0.0	0.0	0.0	0.0	0.0
XLOC_048514	ESD	chr12	16671645	16800984	1.72268	0.515362	0.898599	0.0	0.0	0.0	0.157068	0.115285	0.0
XLOC_048515	ESD	chr12	16671645	16800984	0.0	1.00114	0.0	0.0	0.0	0.0	0.0	0.0	0.0
XLOC_077463	ESPL1	chr5	26906764	26907765	0.0	0.0	0.0	0.614817	0.335223	0.711795	0.233303	0.0	1.12355
XLOC_077464	ESPL1	chr5	26908248	26909158	0.0	0.0	0.0	0.602961	0.675812	0.797364	0.348023	0.19205	1.34288

XLOC_077465	ESPL1	chr5	26909498	26909962	0.0	0.0	0.0	0.63358	0.364652	0.485243	0.20825	0.231803	0.410073
XLOC_077466	ESPL1	chr5	26910145	26910724	0.0	0.0	0.0	1.69035	0.666284	1.06272	0.306404	0.509608	0.597749
XLOC_089556	ESR1	chr9	90054148	90076101	27.2315	18.6076	18.2515	0.232476	0.152322	0.393727	0.413208	0.195054	0.226705
XLOC_089557	ESR1	chr9	90118881	90121069	4.95231	7.97907	6.26072	4.9875	5.59156	4.9559	4.84531	6.13312	6.03384
XLOC_089558	ESR1	chr9	90163057	90173788	1.16188	2.43174	3.63417	0.520528	0.271881	0.361011	0.0	0.115795	0.0
XLOC_089559	ESR1	chr9	90174535	90200094	4.78563	2.78731	2.70837	0.424568	0.392645	0.521759	0.254269	0.43042	0.335055
XLOC_090031	ESR1	chr9	90095016	90114564	9.57477	13.557	10.4241	12.0814	12.8937	13.9616	20.0329	14.7769	16.1637
XLOC_090032	ESR1	chr9	90201895	90270611	13.3573	10.2351	14.4246	6.42703	3.87917	4.12226	3.9371	5.04017	3.44127
XLOC_091456	ESR1	chr9	90084856	90085103	0.0	0.0	0.0	2.12549	6.48454	2.37808	0.6445	3.6789	2.03046
XLOC_091457	ESR1	chr9	90087762	90088059	5.16015	1.5383	0.0	1.84694	4.69239	5.23104	1.74136	0.983878	2.66866
XLOC_091458	ESR1	chr9	90088404	90089958	3.62524	2.94391	5.67342	3.06469	3.6951	5.0084	3.63967	2.185	3.3067
XLOC_091459	ESR1	chr9	90090033	90090469	0.0	5.42816	10.1392	5.80931	4.40619	3.99943	4.10788	4.32454	3.83278
XLOC_091460	ESR1	chr9	90090602	90090830	9.75032	0.0	15.2249	2.64457	8.70346	1.95722	2.35782	1.80286	1.67465
XLOC_091461	ESR1	chr9	90091048	90092991	1.62023	3.02995	3.48689	3.41428	3.36949	3.45631	3.89002	3.01519	2.33923
XLOC_091462	ESR1	chr9	90093170	90094648	2.1908	4.09639	5.57122	3.83025	3.9927	4.44154	5.24594	4.01484	4.02353
XLOC_091463	ESR1	chr9	90095016	90114564	4.58014	4.09689	0.0	4.09634	3.48278	3.25772	1.55841	4.39028	2.76685
XLOC_091464	ESR1	chr9	90095016	90114564	6.13853	6.11256	6.39413	3.48022	3.328	5.90145	5.26594	5.85051	4.8053
XLOC_091465	ESR1	chr9	90095016	90114564	0.0	4.44575	1.93767	2.66442	1.91525	2.03935	3.05886	3.1639	2.15493
XLOC_091466	ESR1	chr9	90095016	90114564	0.0	0.916514	4.79325	4.3946	2.83149	2.82958	4.28189	1.49565	3.45999
XLOC_091467	ESR1	chr9	90095016	90114564	1.80152	6.45958	8.44656	3.5488	5.03281	4.832	8.1898	5.34442	5.646
XLOC_091468	ESR1	chr9	90095016	90114564	0.0	1.17433	9.21199	2.8165	5.71237	3.21001	4.39697	1.89939	4.76477
XLOC_091469	ESR1	chr9	90095016	90114564	0.0	3.85246	0.0	3.27087	3.49328	3.09785	4.95047	1.27002	0.56069
XLOC_091470	ESR1	chr9	90095016	90114564	1.9829	2.37187	2.32623	2.84321	2.55549	3.3922	3.85705	2.77244	2.77015
XLOC_091471	ESR1	chr9	90095016	90114564	1.14714	1.02897	1.79406	1.33622	1.07385	2.01996	2.58717	2.28692	1.00104
XLOC_042007	ESR2	chr10	76708801	76712403	47.4248	20.2386	24.0331	3.03269	0.762755	1.9823	2.24061	0.899557	1.59875
XLOC_042008	ESR2	chr10	76713184	76721109	103.629	35.0545	20.3272	4.02315	1.29369	1.6671	3.37416	1.02488	1.85771
XLOC_042009	ESR2	chr10	76721400	76726018	63.2246	29.3352	25.5738	2.53966	1.19105	2.25901	2.55951	1.19608	1.80775
XLOC_062404	ESRP2	chr18	35734398	35739945	1.30726	0.782108	1.53416	1.05475	0.819075	0.271646	0.773591	0.393297	1.19997
XLOC_044148	ETAA1	chr11	65807188	65828449	10.358	13.4601	13.0218	8.91217	8.18725	8.52286	6.95166	5.12414	7.7346
XLOC_046409	ETAA1	chr11	65807188	65828449	0.0	0.360751	1.88704	0.594621	0.566907	0.0626656	0.274724	0.363029	0.369046
XLOC_046410	ETAA1	chr11	65807188	65828449	0.0	0.449916	2.3533	0.269653	0.351269	0.311144	0.269813	0.597566	0.262377
XLOC_038103	ETS2	chr1	152791092	152902588	2.45806	1.69871	0.96097	0.660691	0.670729	0.935608	0.553817	0.855805	1.2842
XLOC_038596	ETS2	chr1	152791092	152902588	62.5553	70.5525	62.0264	64.213	56.1846	59.2331	68.4556	85.3419	59.4419

XLOC_040613	ETS2	chr1	152791092	152902588	0.0	0.0	0.0	0.234077	0.0	0.0	0.0	0.0	0.0
XLOC_040614	ETS2	chr1	152791092	152902588	1.70393	0.0	0.0	0.152602	0.132394	0.175894	0.152546	0.168694	0.148472
XLOC_040615	ETS2	chr1	152791092	152902588	2.48685	1.48765	1.29685	1.04021	0.972497	0.688263	0.376306	0.829516	1.5933
XLOC_040616	ETS2	chr1	152791092	152902588	2.19722	0.657295	1.14593	0.919154	1.54781	0.68453	0.799143	1.3208	1.47225
XLOC_040617	ETS2	chr1	152791092	152902588	6.10017	5.01773	7.75461	2.46064	3.04309	4.27524	4.01842	3.81835	4.26461
XLOC_040618	ETS2	chr1	152791092	152902588	3.2313	2.89871	1.68463	1.83383	2.6903	2.23229	2.33529	1.82675	3.76067
XLOC_040619	ETS2	chr1	152791092	152902588	4.58132	1.3699	4.47805	2.87347	3.84197	3.55903	4.75328	3.5392	4.69753
XLOC_040620	ETS2	chr1	152791092	152902588	1.94183	2.90086	6.0686	2.78153	3.31145	2.20159	5.17986	2.87538	3.54823
XLOC_040621	ETS2	chr1	152791092	152902588	4.63831	2.37862	6.73932	3.2671	3.16544	4.26814	4.2825	2.12618	4.86571
XLOC_040622	ETS2	chr1	152791092	152902588	0.0	2.29376	7.99688	2.52002	2.96264	3.41788	4.72835	3.5125	6.22439
XLOC_040623	ETS2	chr1	152791092	152902588	8.7544	7.84473	10.2572	5.48493	6.26472	5.63096	6.97453	4.30825	6.2788
XLOC_040624	ETS2	chr1	152791092	152902588	0.631942	2.07949	6.92184	2.60603	3.01959	3.15194	2.93542	2.28204	3.09394
XLOC_040625	ETS2	chr1	152791092	152902588	0.978062	1.46249	3.82468	2.19126	1.98644	2.33249	2.56695	1.85616	3.33067
XLOC_040626	ETS2	chr1	152791092	152902588	0.0	0.388675	1.01616	1.63013	3.34177	3.0934	3.04024	2.19829	2.38049
XLOC_040627	ETS2	chr1	152791092	152902588	3.03424	2.8533	6.78418	3.18719	3.09483	3.56274	3.76446	1.91787	3.45113
XLOC_040628	ETS2	chr1	152791092	152902588	7.40321	4.86828	3.47222	6.49848	6.33577	6.27495	5.31062	3.52773	5.80729
XLOC_040629	ETS2	chr1	152791092	152902588	7.8729	6.46833	4.61326	3.34791	3.05073	4.05668	4.37327	4.27325	4.45238
XLOC_039716	ETV5	chr1	81741592	81742832	0.0	0.399719	0.522716	1.55728	0.889459	1.59645	1.70268	1.80875	1.69375
XLOC_076416	ETV6	chr5	98329355	98737814	0.0	0.0	0.0	0.308799	0.40165	0.0	0.15391	0.0	0.150145
XLOC_078392	ETV6	chr5	98329355	98737814	0.0	11.4675	7.99688	43.2981	33.1805	31.2866	54.0317	54.4438	43.7921
XLOC_073250	EXOC4	chr4	98355343	98383239	9.02262	7.42603	9.9693	6.23807	5.59083	6.96997	4.82249	6.12109	4.73161
XLOC_074630	EXOC4	chr4	98111480	98111950	11.5732	12.443	9.0389	0.207147	0.0	0.0	0.0	0.0	0.0
XLOC_074631	EXOC4	chr4	98112434	98112785	22.4096	23.3973	2.91333	0.0	0.0	0.0	0.321898	0.0	0.0
XLOC_074632	EXOC4	chr4	98112923	98113637	14.2966	12.8219	6.09695	0.116437	0.0	0.134495	0.116921	0.0	0.113354
XLOC_074633	EXOC4	chr4	98113719	98114030	37.582	32.2145	21.9735	0.0	0.0	0.0	0.797834	0.0	0.405306
XLOC_074634	EXOC4	chr4	98114812	98115888	7.87426	5.41694	2.46391	0.0	0.0	0.0	0.0715099	0.0	0.0
XLOC_074635	EXOC4	chr4	98116368	98116938	1.75347	5.76363	2.74057	0.0	0.0	0.0	0.0	0.0	0.0
XLOC_074636	EXOC4	chr4	98117210	98117586	6.61839	10.8621	7.74634	0.0	0.0	0.338353	0.287223	0.0	0.286592
XLOC_074637	EXOC4	chr4	98355343	98383239	0.0	0.897965	1.17421	0.134547	0.233699	0.621007	0.67317	0.0	0.654577
XLOC_072707	EXOC4;LRGUK	chr4	98395798	98719592	13.2124	9.61569	21.6376	5.87703	3.57491	4.53917	2.62873	1.53807	3.04885
XLOC_072706	EXOC4;MIR2423	chr4	97772843	98111411	14.4636	17.6004	9.91482	0.181243	0.197319	0.236313	0.0808846	0.137198	0.342194

XLOC_074628	EXOC4;MIR2423	chr4	97772843	98111411	3.12251	0.46698	1.83199	0.0699728	0.0	0.0	0.0	0.0	0.0682135
XLOC_074629	EXOC4;MIR2423	chr4	97772843	98111411	0.739139	0.221093	2.89117	0.0662569	0.0	0.0	0.0	0.0	0.0
XLOC_041579	EXOC5;AP5M1; NAA30	chr10	69785533	70027317	1.65649	2.32769	3.35941	5.53995	7.09428	5.14424	3.78531	4.18952	6.66834
XLOC_042905	EXOC5;AP5M1; NAA30	chr10	69785533	70027317	0.0	1.26805	1.1055	1.0134	1.65118	2.04715	1.01553	1.40493	2.21896
XLOC_043972	EXOC6B	chr11	11684987	11771117	2.2534	1.5748	0.783886	0.176597	0.152382	0.151294	0.26333	0.192869	0.321645
XLOC_043973	EXOC6B	chr11	12037712	12039475	0.904637	0.270546	0.0	0.0	0.0	0.0	0.0	0.0	0.0
XLOC_043974	EXOC6B	chr11	12044536	12046198	0.0	0.52836	0.0	0.0527811	0.0	0.0611917	0.0	0.0	0.0
XLOC_044666	EXOC6B	chr11	11800318	11810553	11.4791	11.8115	11.5591	62.1163	68.427	67.0207	65.1326	69.4443	83.2282
XLOC_044667	EXOC6B	chr11	11800318	11810553	0.0	0.35596	0.93095	4.3736	4.2708	5.79447	4.93675	5.10034	3.32409
XLOC_044668	EXOC6B	chr11	11813964	11898177	2.57233	2.09271	0.0	11.7144	11.1526	11.3729	13.0646	19.1188	15.8082
XLOC_044669	EXOC6B	chr11	11930301	12037398	38.0529	21.866	40.6602	5.29605	4.29191	4.76887	4.60243	3.28458	3.66355
XLOC_045470	EXOC6B	chr11	11684987	11771117	0.0	0.481989	0.0	0.144434	0.250677	0.166563	0.288503	0.0	0.0
XLOC_045471	EXOC6B	chr11	11684987	11771117	0.0	0.0	0.0	0.0	0.160557	0.106569	0.185905	0.0	0.0897555
XLOC_045472	EXOC6B	chr11	11684987	11771117	0.822346	0.0	1.28654	0.0737101	0.192956	0.085351	0.149267	0.082298	0.0
XLOC_045473	EXOC6B	chr11	11684987	11771117	0.309533	0.277878	0.0	0.0277596	0.0486204	0.0322292	0.113447	0.0311736	0.0270941
XLOC_045474	EXOC6B	chr11	11684987	11771117	1.32591	0.0	0.0	0.0	0.0	0.0	0.0	0.0	0.11563
XLOC_045475	EXOC6B	chr11	11771232	11772915	3.31867	0.567383	0.370992	0.0850197	0.111555	0.147939	0.0433065	0.0953001	0.0
XLOC_045476	EXOC6B	chr11	11793460	11794121	1.43652	0.42939	0.0	0.257353	0.782614	0.148528	0.257811	0.285372	1.25224
XLOC_045477	EXOC6B	chr11	11798358	11798768	0.0	1.71008	2.23591	2.30606	2.64523	1.17441	2.75569	1.95778	0.993714
XLOC_045478	EXOC6B	chr11	11810907	11811694	0.0	0.0	0.0	0.617695	1.34442	1.07107	0.0	0.572626	1.40367
XLOC_045479	EXOC6B	chr11	11907702	11908085	0.0	0.0	0.0	2.00757	1.23075	1.31225	0.278813	0.0	0.555618
XLOC_045480	EXOC6B	chr11	11922522	11923162	0.0	0.0	0.0	0.134269	0.816271	0.154933	0.806176	0.148784	0.783873
XLOC_045481	EXOC6B	chr11	11924597	11925127	0.0	0.0	0.0	1.04306	0.0	0.600428	0.172647	0.191692	0.337918
XLOC_053292	EXT1	chr14	48446333	48515223	2.72798	4.08039	2.72677	2.4144	2.64805	1.55746	1.92633	1.1202	2.60929
XLOC_055492	EXT2;ACCS;AL X4	chr15	74994825	75239093	0.0	0.0	0.965888	0.0	0.0	0.0	0.0	0.0	0.0
XLOC_055808	EXT2;ACCS;AL X4	chr15	74994825	75239093	4.62496	5.63914	13.745	4.24037	1.26332	4.74539	3.53809	1.59889	2.61113
XLOC_056943	EXT2;ACCS;AL X4	chr15	74994825	75239093	0.0	1.30837	0.0	115.359	51.4431	88.7943	35.5583	58.5713	37.8826
XLOC_066536	EXTL1;SLC30A2	chr2	127633729	127656202	0.890953	0.0	1.04582	0.0399449	0.139793	0.0	0.0814281	0.0	0.428712
XLOC_052549	EYA2	chr13	76439375	76439669	10.5419	10.9992	32.8669	13.2079	14.7697	16.0237	11.9886	18.0722	18.1695

XLOC_065856	EYA3;SMPDL3B	chr2	125827532	125983563	3.60377	4.71732	4.2302	15.3094	13.4694	14.6547	14.3374	17.3989	18.289
XLOC_066519	EYA3;SMPDL3B	chr2	125827532	125983563	72.0108	75.8449	68.2949	95.9986	89.5246	102.002	109.314	125.798	121.585
XLOC_068509	EYA3;SMPDL3B	chr2	125827532	125983563	4.72238	3.24968	2.95635	9.06153	9.66743	10.168	12.5965	11.8662	13.2196
XLOC_090042	EZR	chr9	96540634	96631529	33.2185	34.6853	21.0237	9.01007	10.1645	9.8013	8.72908	8.5365	7.56778
XLOC_091520	EZR	chr9	96540634	96631529	4.80572	1.43715	3.75866	0.344549	1.12635	0.398682	0.957063	0.480124	0.755372
XLOC_091521	EZR	chr9	96540634	96631529	4.51375	2.02219	1.76276	1.61591	1.22152	0.696507	0.798089	2.44147	1.37306
XLOC_041454	F2RL2	chr10	7767944	7821615	49.3009	47.2644	43.0741	33.5161	29.3445	36.2154	39.7562	47.563	41.4105
XLOC_041838	F2RL2	chr10	7767944	7821615	20.6916	19.4422	20.5033	7.26218	8.56417	8.30836	8.59586	7.00478	10.6332
XLOC_042282	F2RL2	chr10	7767944	7821615	0.0	0.0	2.57088	0.883825	2.52699	2.02106	1.71595	2.24061	0.855911
XLOC_042283	F2RL2	chr10	7767944	7821615	0.0	11.5302	0.0	9.36615	5.68372	3.85488	4.06364	3.51357	5.51134
XLOC_042284	F2RL2	chr10	7767944	7821615	0.0	1.62677	0.0	2.19348	2.51852	2.23574	3.10502	1.59868	1.41832
XLOC_082746	F2RL3	chr7	6045182	6109001	27.3923	37.341	30.2623	4.16843	2.36858	2.15141	4.1448	3.18825	2.37568
XLOC_058933	F5	chr16	38004950	38005469	0.0	1.79326	0.0	0.0	0.0	0.0	0.0	0.0	0.0
XLOC_092899	F8	chrX	38969784	38970228	2.52465	3.01543	0.0	2.9367	2.14281	2.33368	3.33196	3.71244	4.3844
XLOC_065851	FABP3	chr2	122738464	122747227	3.25053	4.85035	8.61574	8.3095	9.54568	7.87837	7.84683	7.08141	8.61019
XLOC_068472	FABP3	chr2	122747507	122748573	6.36875	3.09543	1.86825	4.5669	4.91998	6.61117	4.55398	4.14422	5.98211
XLOC_065852	FABP3;ZCCHC17	chr2	122765356	122809535	1.93831	1.15985	2.27514	0.217247	0.0760077	0.252001	0.0885109	0.0	0.0423887
XLOC_053284	FABP4	chr14	46832138	46854295	15.6031	12.2455	12.1794	1.75739	2.75602	2.39712	1.73454	1.16015	2.62153
XLOC_053574	FABP9	chr14	46810479	46822379	28.9261	31.9447	21.4645	37.5385	45.7552	40.0073	36.311	34.2345	47.7595
XLOC_070050	FAF1	chr3	95929258	95947459	56.1657	45.7325	37.5228	82.8352	98.487	105.793	133.676	108.373	91.0951
XLOC_070051	FAF1	chr3	96104498	96157935	20.2204	16.5392	17.7126	7.01933	7.31566	6.42248	8.47717	8.69537	6.27897
XLOC_070052	FAF1	chr3	96161968	96171938	4.34657	3.26094	2.43288	0.991434	1.46052	0.946946	0.833007	0.597109	1.08239
XLOC_070053	FAF1	chr3	96172508	96177967	16.267	16.1275	14.5878	20.5928	14.075	17.3049	17.7388	30.9337	11.6915
XLOC_070054	FAF1	chr3	96181186	96195728	0.0	0.298714	0.390647	0.462583	0.693022	0.502752	0.427927	0.335663	0.378798
XLOC_070055	FAF1	chr3	96267928	96274231	43.2808	46.2475	30.4315	163.953	135.2	168.937	161.782	160.183	138.643
XLOC_070427	FAF1	chr3	95988799	95991177	6.86516	9.44645	8.59353	1.60012	1.98888	1.64021	2.74836	2.33986	2.28034
XLOC_070428	FAF1	chr3	95991315	95995850	24.4568	12.8726	30.0717	2.37122	2.91499	2.52637	3.93738	2.42415	3.08972
XLOC_070429	FAF1	chr3	95999733	96057985	3.79463	4.7795	4.11435	5.24404	4.99599	4.55754	6.24155	6.87459	5.69915
XLOC_070430	FAF1	chr3	96196516	96198472	2.29792	4.46101	3.59381	19.6623	15.5012	17.1682	18.9391	18.6672	19.0331
XLOC_070431	FAF1	chr3	96209671	96222454	4.09276	4.55045	4.12012	34.2989	30.4311	31.7809	25.8812	31.2443	25.6145
XLOC_071755	FAF1	chr3	95929258	95947459	0.0	0.560855	2.93216	3.02434	3.20143	2.70868	3.3405	2.96604	2.123

XLOC_071756	FAF1	chr3	95929258	95947459	0.0	1.09777	2.8692	0.987671	1.9698	2.25224	1.9039	2.13537	0.318411
XLOC_071757	FAF1	chr3	95929258	95947459	3.48423	0.0	0.0	2.49339	1.33461	2.13553	3.01523	1.01353	1.20649
XLOC_071758	FAF1	chr3	95995996	95997065	0.0	0.474653	1.24141	0.142246	0.0620746	0.411845	0.432306	0.23831	0.138662
XLOC_071759	FAF1	chr3	95999733	96057985	11.3759	16.9941	8.88855	2.54626	2.20529	2.93214	1.68714	2.43455	2.475
XLOC_071760	FAF1	chr3	96104498	96157935	6.68519	2.24939	1.961	0.599203	0.784221	0.867244	1.28896	0.501701	0.73006
XLOC_071761	FAF1	chr3	96104498	96157935	4.96897	4.31128	2.72178	0.668301	1.20795	0.930194	0.862031	0.499282	0.825829
XLOC_071762	FAF1	chr3	96104498	96157935	2.71656	1.62415	1.0619	0.0	0.95201	0.702533	0.36618	0.675228	0.710643
XLOC_071763	FAF1	chr3	96104498	96157935	12.208	4.25479	6.35823	1.63927	1.41839	1.46717	0.722401	1.40446	0.530966
XLOC_071764	FAF1	chr3	96104498	96157935	0.0	5.36305	4.67468	0.80359	1.61165	0.920195	1.30661	0.291989	1.55774
XLOC_071765	FAF1	chr3	96104498	96157935	14.6387	9.82591	17.1287	0.654513	1.68	1.12048	1.26319	1.41664	0.949859
XLOC_071766	FAF1	chr3	96104498	96157935	7.98471	3.18263	7.28303	0.238439	0.829285	0.413058	0.358949	0.661782	1.27659
XLOC_071767	FAF1	chr3	96104498	96157935	3.54836	2.82909	1.84974	0.529882	0.553451	0.122487	0.639796	0.235705	1.03203
XLOC_071768	FAF1	chr3	96104498	96157935	3.16003	1.65404	3.08997	0.283252	0.679856	0.90212	0.358705	0.158192	0.483213
XLOC_071769	FAF1	chr3	96104498	96157935	4.29447	2.33633	2.13871	0.595153	0.949888	0.65007	0.6072	0.510634	1.05905
XLOC_071770	FAF1	chr3	96104498	96157935	3.82065	0.914237	1.79331	0.547962	0.597932	0.6347	0.347122	0.229586	0.400656
XLOC_071771	FAF1	chr3	96104498	96157935	6.98003	3.65012	1.36369	1.40633	1.89667	1.26056	0.62283	1.03608	1.21558
XLOC_071772	FAF1	chr3	96104498	96157935	0.0	2.50467	2.62009	0.600452	0.651048	0.346116	1.34784	0.498051	0.875989
XLOC_071773	FAF1	chr3	96104498	96157935	5.18038	1.20539	2.25186	0.206423	0.496199	0.119666	0.104958	0.0577839	0.503346
XLOC_071774	FAF1	chr3	96104498	96157935	3.50084	4.17805	0.0	0.313069	0.268151	1.07274	0.302886	0.678774	0.0
XLOC_071775	FAF1	chr3	96104498	96157935	1.63458	0.977012	3.8326	0.0	0.508059	0.506396	0.730738	0.485914	0.142407
XLOC_071776	FAF1	chr3	96104498	96157935	1.75773	1.0505	2.74722	0.157399	0.40935	0.906891	0.627226	0.521744	0.153068
XLOC_071777	FAF1	chr3	96104498	96157935	3.32526	0.596832	2.08126	0.178861	0.416655	0.414559	0.423738	0.400118	0.348832
XLOC_071778	FAF1	chr3	96158565	96159501	6.50329	5.00108	4.3599	0.666104	2.68624	1.15633	1.09395	1.48567	1.13591
XLOC_071779	FAF1	chr3	96159645	96161208	9.26235	4.46457	3.2212	1.79936	2.17872	1.97987	1.54997	1.29232	1.89032
XLOC_071780	FAF1	chr3	96172070	96172447	0.0	3.93196	5.14083	1.17841	0.758097	2.02106	0.857971	0.640148	1.14121
XLOC_071781	FAF1	chr3	96172508	96177967	7.73597	6.15962	8.05383	2.99963	1.98893	3.44201	4.30676	4.29477	3.35815
XLOC_071782	FAF1	chr3	96223135	96223593	2.40706	0.718821	0.0	1.077	0.929487	0.494789	0.212228	0.236298	0.83638
XLOC_071783	FAF1	chr3	96231198	96232012	0.0	0.0	1.72308	0.0987197	0.0859745	0.114147	0.0	0.109878	0.288465
XLOC_071784	FAF1	chr3	96235418	96235903	0.0	0.659685	0.0	0.59304	2.2209	0.909093	0.781708	0.0	0.959962
XLOC_071785	FAF1	chr3	96236114	96236359	0.0	2.39895	0.0	2.1709	3.60891	1.6181	1.97106	2.25129	1.38186
XLOC_071786	FAF1	chr3	96236605	96237234	1.53403	0.458492	0.0	1.92355	1.78946	0.634055	1.3741	0.456571	0.802061
XLOC_071787	FAF1	chr3	96237334	96238075	0.0	4.07643	0.969195	4.77538	6.08735	3.46461	3.0141	2.34494	4.32526
XLOC_071788	FAF1	chr3	96240519	96241341	2.17688	0.0	0.0	3.41363	1.86886	2.70676	1.57276	1.30288	1.99504

XLOC_071789	FAF1	chr3	96244303	96245367	0.0	0.715904	1.24825	2.64606	1.18588	0.331284	1.88351	1.11819	0.83655
XLOC_071790	FAF1	chr3	96247477	96248224	0.0	3.30135	2.87803	3.95735	3.63473	4.19175	3.97882	2.44361	3.10404
XLOC_071791	FAF1	chr3	96248481	96249934	0.0	0.501048	0.0	1.20127	0.875142	0.406265	0.967394	0.448415	0.634682
XLOC_071792	FAF1	chr3	96262315	96264807	9.91353	10.9391	9.94113	52.7597	44.7199	57.9975	51.1226	47.3931	36.147
XLOC_071793	FAF1	chr3	96310731	96311175	0.0	0.0	0.0	0.2259	0.0	0.0	0.0	0.0	0.21922
XLOC_071795	FAF1	chr3	96311941	96312556	0.0	0.0	0.0	0.141594	0.0	0.0	0.0	0.0	0.0
XLOC_071796	FAF1	chr3	96322147	96322292	0.0	20.2094	0.0	39.159	4.64562	19.7788	28.9857	39.258	28.6025
XLOC_071797	FAF1	chr3	96323104	96323251	0.0	0.0	48.3669	29.7363	8.52734	12.0699	8.87171	15.4531	15.7011
XLOC_071798	FAF1	chr3	96328589	96328759	0.0	0.0	21.8877	18.3031	25.9705	27.636	8.39623	12.1885	14.3113
XLOC_071799	FAF1	chr3	96340921	96341156	0.0	0.0	0.0	1.61878	1.33798	0.901379	0.0	2.49851	0.770621
XLOC_071801	FAF1	chr3	96398933	96399060	0.0	0.0	0.0	257.768	117.585	57.1803	176.582	125.45	149.77
XLOC_071802	FAF1	chr3	96402289	96402591	0.0	4.45859	3.88589	7.58198	2.64707	2.02281	2.10798	2.38009	1.71937
XLOC_071803	FAF1	chr3	96406337	96406544	13.0337	0.0	10.2041	3.56908	0.0	2.60969	0.0	1.18847	0.0
XLOC_071804	FAF1	chr3	96408337	96408751	2.81973	1.6835	4.40234	3.53143	6.07812	4.04746	5.18295	6.61125	5.1365
XLOC_071805	FAF1	chr3	96412924	96413700	4.67759	2.79709	6.40089	4.08634	1.36811	2.17997	1.15997	3.02986	1.12242
XLOC_071806	FAF1	chr3	96413910	96414091	42.2023	6.37033	16.6562	27.5916	9.22987	8.44841	3.25111	11.3105	0.0
XLOC_071807	FAF1	chr3	96414191	96414952	21.5724	28.307	43.1072	166.223	141.488	150.034	109.203	104.583	118.68
XLOC_076250	FAIM2	chr5	30119280	30177167	120.442	22.9456	8.58322	18.8923	15.8682	17.7576	16.8967	11.0647	11.2114
XLOC_076251	FAIM2	chr5	30177829	30180473	0.0	0.0	0.0	0.87261	1.01533	0.673661	0.441859	0.324805	0.425292
XLOC_076252	FAIM2	chr5	30183941	30184720	29.6593	33.4499	33.7277	15.7003	15.7498	14.2257	15.9947	17.695	23.982
XLOC_076783	FAIM2	chr5	30119280	30177167	47.9779	60.3321	51.5956	40.5317	35.3624	40.2628	39.7689	43.3106	42.3232
XLOC_077551	FAIM2	chr5	30119280	30177167	3.39978	5.07982	9.29941	1.37003	2.37638	1.40378	1.36626	1.00983	1.62845
XLOC_077552	FAIM2	chr5	30119280	30177167	2.95803	3.18571	7.86913	1.21992	1.2053	1.41429	1.72552	1.18749	0.982886
XLOC_077553	FAIM2	chr5	30119280	30177167	1.31865	1.18261	4.12384	0.590664	1.54088	0.818645	2.13453	1.04934	1.26499
XLOC_077554	FAIM2	chr5	30119280	30177167	1.50843	0.901705	2.35819	1.08086	0.469324	0.155893	0.811081	0.748487	0.525834
XLOC_077555	FAIM2	chr5	30177829	30180473	0.0	4.96336	9.08623	1.63609	2.06447	1.88632	1.92953	0.329037	0.723308
XLOC_077556	FAIM2	chr5	30180720	30181640	0.0	1.70198	4.4513	1.87019	2.2975	1.27882	0.687077	1.80082	1.57383
XLOC_077557	FAIM2	chr5	30181799	30182442	2.97957	1.78115	4.65819	2.0016	3.01335	0.923906	1.4691	1.77457	1.8178
XLOC_077558	FAIM2	chr5	30182877	30183360	0.0	2.65492	8.67871	2.3867	2.06244	1.82921	2.35896	2.6236	2.51112
XLOC_059845	FAM101A	chr17	53716470	53728725	0.0	0.0727621	0.0	0.0	0.0191059	0.0558056	0.0	0.0	0.0425751
XLOC_047229	FAM102A	chr11	98624758	98625528	36.6065	32.1282	26.7774	95.5404	80.2127	90.3704	137.336	148.602	137.653

XLOC_050793	FAM107B;HSPA14;SUV39H2;CDNF;DCLRE1C	chr13	29325136	29855662	26.5445	20.1713	17.3345	30.1254	21.6764	25.4531	31.8655	23.5818	33.1275
XLOC_083644	FAM108A	chr7	45805880	45814392	26.7185	27.3567	20.5084	17.2321	12.5249	15.1028	14.1729	18.2798	12.251
XLOC_084852	FAM108A	chr7	45805880	45814392	5.80942	11.4639	15.4456	4.06015	4.35009	3.61011	5.34421	4.28442	2.83879
XLOC_084853	FAM108A	chr7	45805880	45814392	8.48321	12.6571	0.0	6.87863	3.16776	3.41148	10.3572	5.52725	2.18623
XLOC_084854	FAM108A	chr7	45816552	45818388	0.0	0.386763	0.337189	0.193183	0.169035	0.35864	0.157562	0.259978	0.0376987
XLOC_076476	FAM109B;NDUFA6;C5H22orf32;NAGA	chr5	113558098	113590700	0.0	0.369503	1.86692	3.22109	3.41717	2.2478	2.22385	2.62459	4.20327
XLOC_076986	FAM109B;NDUFA6;C5H22orf32;NAGA	chr5	113558098	113590700	0.0	0.0	0.0	44.8271	38.6826	21.5702	21.5803	15.6733	55.9516
XLOC_078756	FAM109B;NDUFA6;C5H22orf32;NAGA	chr5	113558098	113590700	0.0	0.821311	0.0	0.615313	0.213942	0.142094	0.493663	0.682784	0.239562
XLOC_078757	FAM109B;NDUFA6;C5H22orf32;NAGA	chr5	113558098	113590700	0.0	0.0	0.0	0.219481	0.190981	0.634046	0.551698	0.975813	1.28226
XLOC_078758	FAM109B;NDUFA6;C5H22orf32;NAGA	chr5	113558098	113590700	0.0	0.0	0.837333	1.53513	0.668615	0.665744	0.290185	0.747796	0.654226
XLOC_053219	FAM110B	chr14	25842124	26165543	7.91945	10.8722	10.2072	20.0172	17.561	21.725	15.1794	21.0398	16.1154
XLOC_054010	FAM110B	chr14	25842124	26165543	0.0	0.0	0.0	4.1223	6.04728	3.65391	2.21091	0.52548	3.08326
XLOC_066533	FAM110D	chr2	127526962	127541438	0.0	0.0838323	0.139189	0.698279	0.483903	0.695204	0.0326467	0.255114	0.0934236
XLOC_055582	FAM111B	chr15	83563576	83575093	26.8694	10.4161	11.5551	17.0401	24.0037	16.8575	23.4801	14.8937	28.9627
XLOC_055912	FAM111B	chr15	83576868	83595360	9.72379	18.3886	12.3878	22.1202	19.2712	20.1264	19.4742	27.5707	16.4937
XLOC_057314	FAM111B	chr15	83575267	83576769	5.91714	8.36847	4.20909	8.63311	5.60851	7.60644	8.29424	11.5068	5.78663
XLOC_076801	FAM113B	chr5	33213897	33218251	1.6893	0.252725	0.991491	0.681657	1.15983	0.351538	0.540606	0.382264	0.665125
XLOC_076802	FAM113B	chr5	33247647	33274914	101.648	68.5309	72.2742	87.0698	67.9557	67.795	68.7911	93.9622	65.7379
XLOC_077639	FAM113B	chr5	33245606	33246191	1.69194	1.51683	1.32228	0.757573	0.525646	0.17466	1.20894	0.167534	0.58941
XLOC_077640	FAM113B	chr5	33282409	33282593	0.0	0.0	0.0	5.52059	0.0	0.0	0.0	0.0	0.0
XLOC_074867	FAM115A	chr4	107872492	107873403	12.4783	21.2416	29.2787	1.37637	3.74957	2.58801	3.99705	1.82208	3.9395

XLOC_074868	FAM115A	chr4	107873568	107876354	7.15379	7.73974	15.5055	0.296114	0.670075	0.573097	0.428859	0.304945	0.602174
XLOC_074869	FAM115A	chr4	107876453	107876840	6.30192	6.58256	7.37694	0.0	0.48394	0.644912	0.274224	0.0	0.273027
XLOC_072743	FAM115A;FAM115C	chr4	107802477	107872413	24.8366	34.5735	50.7302	4.82307	7.60933	5.41529	6.45185	2.94409	5.79696
XLOC_073276	FAM115A;FAM115C	chr4	107802477	107872413	0.0	0.564488	2.6185	0.453485	1.31109	0.391449	0.256333	0.334302	0.882071
XLOC_074864	FAM115A;FAM115C	chr4	107802477	107872413	3.04046	2.95573	3.56778	1.56712	1.30858	1.026	1.10503	1.5987	0.99637
XLOC_074865	FAM115A;FAM115C	chr4	107802477	107872413	2.09333	0.208813	1.6377	1.12593	1.0926	1.0147	0.698214	1.18896	0.792883
XLOC_074866	FAM115A;FAM115C	chr4	107802477	107872413	0.0	0.0	2.2184	0.254222	0.0	0.0	0.0	0.0	0.246508
XLOC_076505	FAM118A	chr5	116439030	116462674	0.0	0.323028	0.844829	1.79521	4.40837	2.71322	2.91658	0.991991	5.54551
XLOC_078824	FAM118A	chr5	116439030	116462674	0.0	0.0	0.0	0.0	0.594571	0.527664	2.25895	0.251757	0.669232
XLOC_078825	FAM118A	chr5	116439030	116462674	1.90875	3.7695	2.39007	0.513496	0.659215	0.55623	0.558903	0.3073	0.267251
XLOC_092387	FAM122B	chrX	18418137	18420560	0.0	0.371588	0.0	0.0	0.0	0.0	0.0	0.0	0.0
XLOC_079652	FAM13A	chr6	37442985	37446929	1.31386	0.0	2.05444	3.17801	3.07069	3.12683	0.827151	0.914888	3.32291
XLOC_080070	FAM13A	chr6	37330947	37442169	30.6554	25.4532	29.2602	1.91527	2.30539	2.2005	2.0226	1.27706	1.81532
XLOC_080801	FAM13A	chr6	37442492	37442830	0.0	2.38732	6.24239	2.14723	1.83286	2.85418	1.37382	1.15764	4.15073
XLOC_080802	FAM13A	chr6	37447184	37447418	0.0	2.70947	0.0	8.19016	2.02957	3.64682	2.20572	2.52607	3.89769
XLOC_086818	FAM154A;RRAGA	chr8	25159081	25240898	32.297	24.1864	22.1495	14.9948	14.1699	16.0331	12.6686	14.2773	12.9222
XLOC_087473	FAM154A;RRAGA	chr8	25159081	25240898	3.88514	3.87156	2.02505	3.36459	2.11901	2.81436	3.14582	2.57718	1.24244
XLOC_087474	FAM154A;RRAGA	chr8	25159081	25240898	2.0233	3.62661	6.32272	3.8036	2.35097	1.25062	1.97589	1.9955	1.58397
XLOC_047963	FAM155A	chr12	86826173	86827501	0.0	0.613116	0.589675	1.19618	1.47791	1.42813	1.22888	2.04602	1.51308
XLOC_047964	FAM155A	chr12	86829708	86844488	0.0	0.0	0.0	1.31186	0.318	0.590773	0.442838	0.732438	0.284164
XLOC_047965	FAM155A	chr12	86947354	86963458	14.2895	12.5067	16.4953	14.6723	13.7424	13.7585	14.9454	18.3739	16.6324
XLOC_047966	FAM155A	chr12	87069151	87075719	0.0	3.81021	2.98946	0.456731	0.297963	0.263817	0.573536	1.77581	0.333499
XLOC_047967	FAM155A	chr12	87077665	87086419	0.967573	4.0509	2.27027	0.260139	0.302355	0.301	0.0875774	0.289979	0.253469
XLOC_047968	FAM155A	chr12	87106732	87173203	6.64646	12.8441	13.0945	17.8433	22.6894	18.7191	20.4253	18.0224	22.3827
XLOC_047969	FAM155A	chr12	87266425	87274055	62.9288	75.2986	77.8644	322.062	279.252	317.748	331.185	391.352	338.644
XLOC_048245	FAM155A	chr12	87106732	87173203	5.64172	6.89833	7.35377	5.50968	6.4832	5.15016	5.47783	5.41914	4.69492
XLOC_048246	FAM155A	chr12	87173857	87200295	1.67509	2.00399	3.27581	5.96371	4.04751	4.9546	5.54751	5.36359	4.53668
XLOC_048247	FAM155A	chr12	87221036	87225162	1.26999	1.13902	1.98595	10.4267	8.62928	8.61352	6.46251	11.7989	12.2225
XLOC_048248	FAM155A	chr12	87238579	87266333	12.5203	15.9222	11.0767	16.0988	14.2459	13.6169	15.3013	20.3521	16.3025

XLOC_048249	FAM155A	chr12	87278068	87301021	0.507429	0.659432	0.198581	4.46937	5.67853	4.26299	7.58625	6.95919	6.06376
XLOC_048250	FAM155A	chr12	87308407	87388661	28.0341	33.4975	31.6246	18.6915	17.217	17.6049	24.0547	22.2001	26.5805
XLOC_049961	FAM155A	chr12	86829708	86844488	0.0	0.0	0.0	1.61821	0.344091	0.459741	0.770204	0.867673	1.17128
XLOC_049962	FAM155A	chr12	86867821	86868308	0.0	0.0	0.0	2.35779	0.169819	2.03321	0.388576	1.08024	1.33585
XLOC_049963	FAM155A	chr12	86879452	86880042	0.0	0.499773	0.0	0.599063	1.29913	1.03597	0.74712	0.496909	0.728296
XLOC_049964	FAM155A	chr12	86880313	86880995	1.379	4.94663	4.31228	3.953	2.46962	3.70841	5.07885	7.67585	4.44871
XLOC_049965	FAM155A	chr12	86881141	86884964	24.8603	27.162	29.6525	26.0668	20.7367	22.1616	25.381	40.0953	18.1758
XLOC_049966	FAM155A	chr12	86885063	86885888	6.50128	1.94397	2.54207	2.52446	1.09941	2.13329	2.44662	2.4861	3.31018
XLOC_049967	FAM155A	chr12	86888372	86888751	0.0	1.94832	0.0	2.04368	2.75526	1.33546	0.850671	0.634594	1.41381
XLOC_049968	FAM155A	chr12	86894860	86895603	0.0	1.10796	0.0	0.553383	0.962977	0.0	0.333782	0.246005	0.431056
XLOC_049969	FAM155A	chr12	86908674	86909407	0.0	0.0	0.0	0.675584	0.587726	0.260178	0.452598	1.25107	0.109628
XLOC_049970	FAM155A	chr12	86911306	86913077	0.0	0.0	0.0	0.763655	0.843937	0.792726	0.901214	1.35197	0.745085
XLOC_049971	FAM155A	chr12	86914150	86915226	0.0	0.235519	0.0	0.988142	0.55444	0.245233	0.929629	1.41907	1.44489
XLOC_049972	FAM155A	chr12	86915452	86916604	0.0	0.217533	0.0	2.41208	2.67543	1.81261	4.42994	3.8619	4.51275
XLOC_049973	FAM155A	chr12	86917146	86917898	0.0	0.363735	0.0	0.545015	0.379416	0.377887	0.109614	1.21168	0.318419
XLOC_049974	FAM155A	chr12	86918219	86918867	0.0	0.0	0.0	0.396245	0.344191	0.457287	1.3223	0.292801	0.514104
XLOC_049975	FAM155A	chr12	86945438	86946407	1.78252	0.799646	0.0	1.03844	1.04504	0.739709	0.323136	0.802073	0.700628
XLOC_049976	FAM155A	chr12	86946558	86947047	0.0	0.651741	0.0	1.7577	0.67525	1.3474	2.12478	3.22169	2.46602
XLOC_049977	FAM155A	chr12	86963581	86965402	8.69528	6.89441	7.48502	8.45971	8.15248	8.09673	7.71013	12.7657	10.0801
XLOC_049978	FAM155A	chr12	87106732	87173203	40.2068	16.7926	25.0862	20.2617	30.0743	24.2716	28.2518	16.5096	39.3831
XLOC_049979	FAM155A	chr12	87173857	87200295	0.0	0.0	2.66706	1.22279	0.785971	1.74655	1.18462	3.31682	0.591893
XLOC_049980	FAM155A	chr12	87173857	87200295	3.91512	2.33934	7.64722	1.22677	1.36548	1.81574	1.91399	1.15927	1.70321
XLOC_049981	FAM155A	chr12	87200484	87201961	3.28869	1.47582	2.1444	0.638859	0.472612	0.569858	0.649984	0.660471	0.62316
XLOC_049982	FAM155A	chr12	87274826	87275822	0.862558	1.28986	0.0	1.00503	2.4279	1.25303	1.32981	1.2942	0.828834
XLOC_049983	FAM155A	chr12	87278068	87301021	0.0	0.29527	1.54448	0.796383	1.07974	1.02376	2.23345	0.986151	1.8967
XLOC_049984	FAM155A	chr12	87278068	87301021	0.0	1.05465	0.0	2.52888	2.97767	1.44403	0.305662	3.42551	1.83558
XLOC_049985	FAM155A	chr12	87278068	87301021	0.0	0.0	0.0	3.2097	4.06321	1.7205	2.10885	2.3478	1.86952
XLOC_049986	FAM155A	chr12	87301200	87301550	0.0	2.23979	0.0	2.34993	4.30564	1.91477	0.970378	1.45173	1.62347
XLOC_049987	FAM155A	chr12	87305058	87306782	3.23181	2.07202	2.529	1.65591	1.52104	1.96907	2.4466	2.97024	3.47351
XLOC_049988	FAM155A	chr12	87306886	87307375	0.0	1.95522	3.40878	1.953	3.03862	3.36851	1.54529	3.43647	3.60418
XLOC_049989	FAM155A	chr12	87307536	87308315	15.1288	6.61105	3.64001	2.294	3.35854	4.33905	5.87739	6.95871	6.49905
XLOC_049990	FAM155A	chr12	87419742	87420354	0.0	0.951233	0.0	0.855166	2.47407	1.15069	1.1392	0.157773	2.21838
XLOC_049991	FAM155A	chr12	87421743	87422342	1.63828	0.0	1.28042	1.76061	1.78218	1.01506	2.05056	0.48698	1.9979

XLOC_049992	FAM155A	chr12	87423634	87424793	0.0	1.72811	1.12993	1.03578	2.03502	1.35	2.29819	0.57889	1.5148
XLOC_083150	FAM174A	chr7	102210473	102238141	7.0075	10.8201	8.48762	8.91947	10.307	10.0536	6.14091	9.12174	8.5899
XLOC_083796	FAM174A	chr7	102238358	102250896	9.43596	10.2348	7.3845	3.06728	2.7463	3.06867	2.19405	2.01732	1.8922
XLOC_085510	FAM174A	chr7	102238358	102250896	3.90455	1.74978	0.0	0.174781	0.151317	0.603631	0.347093	0.192703	0.169865
XLOC_085511	FAM174A	chr7	102238358	102250896	0.0	1.16652	4.57597	0.349561	0.0	0.603631	0.347093	0.0	0.0
XLOC_050493	FAM188A	chr13	30660086	30711365	7.55879	10.6185	6.46967	2.31612	2.81245	3.59868	2.3449	2.08067	2.18151
XLOC_051526	FAM188A	chr13	30660086	30711365	0.0	3.72181	5.56193	0.955971	0.690524	0.183585	0.476025	0.352006	0.309833
XLOC_051527	FAM188A	chr13	30660086	30711365	0.0	6.65084	5.797	1.99364	2.27347	1.13734	0.0	0.71874	1.28564
XLOC_079650	FAM190A	chr6	35177413	35247628	31.0257	41.2762	33.1354	68.842	69.2102	69.7634	66.5083	70.5495	68.4552
XLOC_079651	FAM190A	chr6	35252670	35262069	23.9449	31.29	16.6133	29.0941	29.7848	27.7277	27.8799	37.1007	31.2989
XLOC_080063	FAM190A	chr6	35021962	35134793	56.5486	95.6753	78.253	34.709	36.52	40.3827	39.5405	42.5773	40.0032
XLOC_080064	FAM190A	chr6	35267877	35308145	0.668718	0.533666	0.872378	0.339867	0.350375	0.255441	0.204573	0.426993	0.351314
XLOC_080065	FAM190A	chr6	35312835	35315742	0.634696	0.163115	0.496579	0.0	0.0	0.0	0.289014	0.0	0.0554838
XLOC_080066	FAM190A	chr6	35508780	35539896	40.0613	35.5833	31.0708	30.9872	30.8017	32.8026	31.747	30.307	30.2384
XLOC_080778	FAM190A	chr6	35021962	35134793	0.0	4.26033	0.0	0.957718	2.73305	1.45795	2.77642	2.42057	3.39805
XLOC_080779	FAM190A	chr6	35021962	35134793	0.0	6.16414	5.37366	0.923634	1.96568	2.01678	2.37651	0.79919	1.59917
XLOC_080780	FAM190A	chr6	35021962	35134793	4.82737	3.84735	2.51534	1.15292	1.37574	2.16055	1.15151	0.637999	1.68222
XLOC_080781	FAM190A	chr6	35021962	35134793	3.37599	2.52232	2.6384	1.36049	1.70448	0.697036	1.50776	1.33723	1.32314
XLOC_080782	FAM190A	chr6	35021962	35134793	3.28897	2.75486	5.1464	2.41777	3.50314	2.32363	2.45513	2.37469	2.99024
XLOC_080783	FAM190A	chr6	35021962	35134793	3.01198	5.18031	1.76718	2.36243	3.35886	3.28383	3.42132	2.11182	2.82958
XLOC_080784	FAM190A	chr6	35021962	35134793	2.36404	3.53701	3.43605	1.93831	2.0949	1.89852	2.13402	1.83603	2.30553
XLOC_080785	FAM190A	chr6	35021962	35134793	3.76925	3.85371	3.4417	1.46479	2.07213	1.83143	2.35978	2.08773	2.74936
XLOC_080786	FAM190A	chr6	35021962	35134793	10.2891	12.2948	8.03804	2.02635	3.8248	3.60316	4.01674	2.43436	5.72739
XLOC_080787	FAM190A	chr6	35021962	35134793	0.0	3.93229	0.0	0.589272	2.02172	2.02108	2.28792	0.960268	0.855995
XLOC_080788	FAM190A	chr6	35262339	35262489	0.0	16.2749	42.5933	5.21456	11.33	15.9776	0.0	22.8403	4.61552
XLOC_080789	FAM190A	chr6	35262901	35263643	1.23743	1.10985	1.93508	0.776059	0.771687	0.384303	0.891573	0.985679	0.43179
XLOC_080790	FAM190A	chr6	35265520	35266099	0.0	0.0	0.0	0.0	0.0	0.0	0.0	0.0	0.298874
XLOC_080791	FAM190A	chr6	35337719	35338317	0.0	0.0	0.0	0.735251	0.637919	0.339114	0.440374	0.488064	1.43029
XLOC_080792	FAM190A	chr6	35338531	35338930	0.0	0.0	0.0	0.267862	2.30236	0.613461	0.522644	0.291966	0.259616
XLOC_080793	FAM190A	chr6	35504723	35508094	1.12528	2.49201	2.1139	0.827584	0.955123	0.937826	0.929423	0.431101	0.768518
XLOC_080794	FAM190A	chr6	35926707	35927123	39.1711	43.4335	43.684	24.279	21.5433	26.1084	24.9893	33.6293	19.4178
XLOC_078864	FAM19A5	chr5	119585735	119586227	0.0	0.0	0.0	1.93552	0.669297	1.1129	0.765987	0.63871	0.752022
XLOC_060259	FAM216A	chr17	56607515	56610379	43.2803	48.2951	24.034	45.3113	35.885	35.1087	41.1327	44.2018	47.5538

XLOC_059861	FAM216A;RAD9B;VPS29	chr17	56613268	56627761	3.65278	3.46827	2.6964	4.06974	4.4483	4.14449	3.65533	4.35477	3.87527
XLOC_060260	FAM216A;RAD9B;VPS29	chr17	56613268	56627761	56.6076	63.8602	57.3269	82.6826	71.0595	75.7048	82.696	96.6709	76.5153
XLOC_073227	FAM3C	chr4	86648427	86670207	12.9194	7.13864	8.38227	23.9387	31.5394	26.9924	23.8092	15.71	40.8939
XLOC_066524	FAM46B	chr2	126791526	126840967	23.0256	28.1547	23.0254	24.411	30.0956	27.3215	32.7336	22.3131	32.0345
XLOC_068522	FAM46B	chr2	126791526	126840967	4.75881	9.93159	3.70974	6.38494	4.69905	10.1467	12.1123	9.56269	8.62086
XLOC_048951	FAM48A	chr12	24706723	24707221	0.0	0.0	0.0	0.380297	0.164425	1.09354	0.0	0.83699	0.554153
XLOC_047809	FAM48A;EXOSC8	chr12	24707369	24716291	4.17748	4.23421	2.17117	119.667	130.126	122.897	146.381	125.828	126.753
XLOC_057786	FAM5C	chr16	15623363	15816671	11.14	13.6101	14.1782	10.68	11.5381	11.8862	11.2821	11.6885	10.5466
XLOC_057787	FAM5C	chr16	15816863	15842081	10.8646	13.7409	15.071	12.2655	10.4557	11.0426	10.4618	11.6117	12.3538
XLOC_057788	FAM5C	chr16	15887047	16146838	11.5188	17.4453	25.7013	1.27462	1.21474	1.10208	0.651677	0.816103	0.965551
XLOC_057789	FAM5C	chr16	16213148	16272826	12.5027	17.3468	14.7729	29.9585	19.2603	29.697	12.2329	14.665	15.3543
XLOC_058034	FAM5C	chr16	15623363	15816671	33.7766	35.0743	25.5629	104.253	104.29	102.63	121.233	128.728	112.747
XLOC_058035	FAM5C	chr16	15848439	15863423	4.79769	3.23853	1.70235	10.5243	11.3116	9.54161	10.4934	11.0235	9.90352
XLOC_058036	FAM5C	chr16	15887047	16146838	6.09446	11.26	11.7022	29.4971	37.2217	33.6472	30.0051	23.7619	42.6869
XLOC_058469	FAM5C	chr16	16197568	16197950	32.2098	29.7975	25.1347	72.5933	78.121	76.7719	81.1953	84.2565	75.3383
XLOC_058470	FAM5C	chr16	16198056	16198222	184.664	206.213	147.081	261.666	204.796	262.603	228.758	303.719	277.535
XLOC_057747	FAM72A;SRGAP2	chr16	3847292	3854624	0.0	0.741794	0.388026	1.33385	1.94431	2.21757	0.679164	0.69756	2.25551
XLOC_047293	FAM78A	chr11	101481186	101481644	4.81412	0.718821	0.0	0.646201	1.85897	0.494789	0.848914	0.0	0.83638
XLOC_047294	FAM78A	chr11	101485429	101486276	2.09794	1.882	0.0	1.87999	1.14652	1.63078	0.758418	0.837493	1.73984
XLOC_047295	FAM78A	chr11	101487352	101487743	0.0	1.84866	0.0	1.66204	2.85514	0.951115	0.539568	0.904793	1.07362
XLOC_045100	FAM78A;PPAPDC3	chr11	101488742	101551974	21.0819	30.2943	20.6547	65.4107	75.1001	70.0942	65.6604	63.5575	95.2162
XLOC_083115	FAM81B;ARSK	chr7	97081714	97348922	14.1162	13.8121	12.9585	1.58693	1.10932	1.31535	1.00729	1.03343	1.0896
XLOC_083759	FAM81B;ARSK	chr7	97081714	97348922	3.8249	8.18557	5.4437	1.60386	1.45913	2.14197	1.24344	1.28731	1.89088
XLOC_085360	FAM81B;ARSK	chr7	97081714	97348922	5.0139	2.99917	7.19033	0.0	0.0653523	0.0	0.0	0.0	0.0
XLOC_085361	FAM81B;ARSK	chr7	97081714	97348922	10.1511	6.07102	13.4962	0.0909722	0.317088	0.315694	0.0917991	0.0	0.0
XLOC_085362	FAM81B;ARSK	chr7	97081714	97348922	3.08682	2.58558	4.34726	0.0	0.0483662	0.0641577	0.0562431	0.0	0.0
XLOC_085363	FAM81B;ARSK	chr7	97081714	97348922	0.0	4.74788	24.8276	0.0	0.0	0.0	0.0	0.0	0.0

XLOC_085364	FAM81B;ARSK	chr7	97081714	97348922	11.3197	4.05701	0.0	0.0	0.0	0.0	0.0	0.0	0.0
XLOC_085365	FAM81B;ARSK	chr7	97081714	97348922	3.69246	1.93246	5.77613	0.0827357	0.0	0.0	0.0	0.0	0.0
XLOC_085366	FAM81B;ARSK	chr7	97081714	97348922	4.02944	0.803031	3.15025	0.0	0.0	0.138954	0.0	0.0	0.0
XLOC_085367	FAM81B;ARSK	chr7	97081714	97348922	1.62953	1.13752	4.25017	0.0	0.0	0.056474	0.0	0.0	0.0
XLOC_085368	FAM81B;ARSK	chr7	97081714	97348922	2.028	1.21348	3.17381	0.0	0.0	0.0	0.0	0.0	0.0
XLOC_085369	FAM81B;ARSK	chr7	97081714	97348922	2.11373	1.26453	2.75607	0.0	0.0	0.0	0.0	0.0	0.0
XLOC_085370	FAM81B;ARSK	chr7	97081714	97348922	9.04697	6.74382	10.5801	0.0	0.0	0.459741	0.0	0.0	0.0
XLOC_085371	FAM81B;ARSK	chr7	97081714	97348922	14.4643	2.26797	11.2754	0.0	0.0	0.0	0.0	0.0	0.0
XLOC_085372	FAM81B;ARSK	chr7	97081714	97348922	0.890169	1.59733	3.48135	0.0	0.0	0.0923505	0.0	0.0	0.0
XLOC_085373	FAM81B;ARSK	chr7	97081714	97348922	2.10962	2.31381	1.10029	0.0	0.0	0.0	0.127923	0.0	0.0
XLOC_085374	FAM81B;ARSK	chr7	97081714	97348922	0.0	0.783872	4.09973	0.0	0.0	0.0	0.0	0.0	0.0
XLOC_085375	FAM81B;ARSK	chr7	97081714	97348922	0.0	0.831232	0.869618	0.0996467	0.0	0.0	0.0	0.0	0.0
XLOC_085376	FAM81B;ARSK	chr7	97081714	97348922	0.0	0.567326	1.48377	0.0	0.0	0.0	0.0	0.0	0.0
XLOC_085377	FAM81B;ARSK	chr7	97081714	97348922	1.96623	1.76452	3.07665	0.0	0.0	0.0	0.0	0.0	0.0
XLOC_085378	FAM81B;ARSK	chr7	97081714	97348922	1.88036	0.984534	2.57501	0.0	0.0	0.0	0.0	0.0	0.0
XLOC_085379	FAM81B;ARSK	chr7	97081714	97348922	0.534745	0.959884	2.92895	0.0	0.0419212	0.0	0.0	0.0537073	0.0467682
XLOC_085380	FAM81B;ARSK	chr7	97081714	97348922	1.4741	1.65406	1.44205	0.0	0.0	0.0	0.0	0.0	0.0
XLOC_045587	FAM82A1	chr11	20395737	20396625	0.0	0.0	0.0	0.79856	0.464003	1.23185	0.179157	0.395527	0.259347
XLOC_053721	FAM82B;WWP1;CPNE3	chr14	78494769	78732402	45.1704	60.0046	53.9315	16.6921	23.9234	17.7998	22.9188	20.0807	26.4699
XLOC_054905	FAM82B;WWP1;CPNE3	chr14	78494769	78732402	5.27096	7.85707	4.10836	0.471712	3.59264	2.67063	1.33233	1.00403	2.27123
XLOC_054906	FAM82B;WWP1;CPNE3	chr14	78494769	78732402	3.43369	2.56884	4.47914	2.64318	3.64129	3.45656	3.88201	2.59435	6.31222
XLOC_054907	FAM82B;WWP1;CPNE3	chr14	78494769	78732402	2.96448	2.798	3.39132	2.98603	4.05021	3.70589	3.91321	3.33343	6.18957

XLOC_054908	FAM82B;WWP1;CPNE3	chr14	78494769	78732402	0.0	0.73251	2.87316	2.63377	3.34216	1.90214	2.64822	3.78128	6.09077
XLOC_054909	FAM82B;WWP1;CPNE3	chr14	78494769	78732402	3.69508	1.65625	5.77461	3.47387	3.0097	1.9054	2.46833	2.00857	4.98522
XLOC_054910	FAM82B;WWP1;CPNE3	chr14	78494769	78732402	1.64096	2.69945	3.20905	3.67709	4.87717	3.40633	4.02121	3.44876	7.88522
XLOC_054911	FAM82B;WWP1;CPNE3	chr14	78494769	78732402	0.0	0.481328	0.629399	1.15391	1.19923	1.29723	0.772445	1.29449	1.54823
XLOC_054912	FAM82B;WWP1;CPNE3	chr14	78494769	78732402	0.852112	1.01987	0.333416	2.02483	1.90551	1.5072	2.06454	1.4568	2.60942
XLOC_062186	FAM96B;CES2	chr18	34754519	34758981	0.0	0.309601	0.80976	0.433569	0.404212	0.401335	0.655203	0.413394	0.903948
XLOC_062365	FANCA	chr18	14625637	14682977	34.0805	31.819	35.8899	69.4435	59.948	64.9628	72.3053	69.4809	63.2532
XLOC_062698	FANCA	chr18	14625637	14682977	2.63731	0.788476	1.03108	1.06326	0.92461	1.36442	0.592932	0.918181	1.15002
XLOC_062699	FANCA	chr18	14625637	14682977	1.74501	2.6073	0.0	1.71893	0.948437	0.90041	1.08996	1.55411	0.607821
XLOC_062700	FANCA	chr18	14625637	14682977	1.98835	3.27033	2.33271	1.42556	1.55307	2.37073	1.529	0.89357	1.38889
XLOC_062701	FANCA	chr18	14625637	14682977	2.39884	0.717528	1.87658	0.43008	0.750671	0.498026	1.01641	0.0800523	0.838412
XLOC_062702	FANCA	chr18	14625637	14682977	0.59123	0.442322	1.15688	0.530242	0.580506	0.492525	0.785708	0.297773	0.362284
XLOC_062703	FANCA	chr18	14625637	14682977	2.31464	0.6914	0.0	0.62156	0.179027	1.42819	2.04419	1.36487	0.60345
XLOC_062704	FANCA	chr18	14625637	14682977	1.09669	1.31174	0.0	0.687953	1.02711	0.681795	0.990285	0.328165	0.95722
XLOC_062705	FANCA	chr18	14625637	14682977	0.0	0.0	10.8235	1.86954	2.60783	0.700061	1.7198	0.652542	1.19401
XLOC_062706	FANCA	chr18	14625637	14682977	2.80192	0.838163	1.09609	0.816355	0.932333	1.60067	1.01947	0.631871	1.46945
XLOC_062707	FANCA	chr18	14625637	14682977	0.0	1.54001	4.0271	2.53805	3.38116	1.85332	2.26665	1.26315	1.56712
XLOC_062708	FANCA	chr18	14625637	14682977	0.680842	2.13918	5.8614	2.04541	1.55031	1.80736	1.87042	1.33659	1.5791
XLOC_062709	FANCA	chr18	14625637	14682977	0.0	4.16476	0.0	6.35672	1.02394	13.9027	12.0372	15.156	4.77406
XLOC_062710	FANCA	chr18	14625637	14682977	5.36446	1.60303	0.0	4.80354	2.08178	2.76725	2.07263	1.76856	2.49088
XLOC_062711	FANCA	chr18	14625637	14682977	2.29725	3.20661	2.3962	2.53972	2.21705	2.94168	2.92191	2.99081	2.81046
XLOC_086509	FANCC	chr8	83389892	83427527	1.02713	2.1516	1.80884	0.748337	0.61452	0.588394	0.894968	1.65099	0.943822
XLOC_086966	FANCC	chr8	83314449	83332464	3.73965	3.37741	5.48646	6.08491	11.4959	8.17913	6.90749	3.65748	10.8883
XLOC_088384	FANCC	chr8	83312597	83314290	2.35504	4.08675	2.58004	3.25195	7.13011	4.89928	3.44216	1.98834	6.09814
XLOC_044026	FANCL	chr11	40702669	40739932	13.3833	19.765	19.935	90.8987	85.6579	89.5318	109.817	106.745	118.288
XLOC_044724	FANCL	chr11	40679315	40695105	31.5546	41.2072	27.1185	59.5765	51.5957	56.543	61.1713	65.4823	73.0511
XLOC_044725	FANCL	chr11	40702669	40739932	12.6431	13.7985	10.8084	74.0543	75.5046	76.0481	102.171	82.0938	116.718
XLOC_044726	FANCL	chr11	40702669	40739932	0.0	0.0	0.0	0.0	0.717807	0.0	0.0	0.416773	0.394369
XLOC_045850	FANCL	chr11	40678739	40679134	0.0	4.54414	0.0	0.817072	1.87215	2.4944	2.65486	1.48352	1.05575
XLOC_045851	FANCL	chr11	40702669	40739932	0.0	0.0	0.0	4.75962	2.26739	2.28537	2.48323	2.83226	1.95051

XLOC_045852	FANCL	chr11	40740150	40740679	0.0	1.16338	0.0	0.697244	2.41465	2.00675	1.03857	0.96098	2.37175
XLOC_045853	FANCL	chr11	40740936	40741881	0.918423	1.37333	0.718352	1.317	0.933106	0.952657	1.33127	1.28527	1.68446
XLOC_066252	FAP	chr2	34171291	34371945	6.0085	4.10985	4.27168	3.59382	4.28445	4.40744	3.26567	2.54231	3.27366
XLOC_067288	FAP	chr2	34171291	34371945	0.0	1.35715	0.0	0.814202	0.346194	0.462548	0.387338	0.0	0.0
XLOC_067289	FAP	chr2	34171291	34371945	0.0	0.213037	0.557202	0.446918	0.780553	0.517793	0.777231	0.214117	0.871487
XLOC_067290	FAP	chr2	34171291	34371945	0.0	1.75508	2.14219	0.350661	0.705897	0.610426	0.572403	0.432769	0.444837
XLOC_067291	FAP	chr2	34171291	34371945	0.611638	0.548902	0.478557	0.438672	0.718775	0.889884	0.390081	0.368209	0.588248
XLOC_067292	FAP	chr2	34171291	34371945	2.78277	1.16552	0.870971	0.898186	0.872463	1.1572	1.11673	0.447044	0.681406
XLOC_067293	FAP	chr2	34171291	34371945	1.21198	2.35666	0.0	0.706271	0.569746	0.377877	0.331308	0.36484	0.529853
XLOC_067294	FAP	chr2	34171291	34371945	2.04046	2.43814	3.18813	1.2786	1.42234	0.630537	1.08654	0.804746	0.532436
XLOC_067295	FAP	chr2	34171291	34371945	1.83023	1.27753	0.0	0.929812	0.81256	1.14125	0.61145	0.30607	0.480079
XLOC_047914	FARP1	chr12	79208858	79222654	0.528868	0.474674	0.0	0.497408	0.580476	0.624391	0.48185	0.212473	0.27753
XLOC_047915	FARP1	chr12	79297338	79342192	0.0	2.09488	2.57292	8.22749	11.0789	8.77939	9.43151	6.67989	12.1425
XLOC_048207	FARP1	chr12	79174890	79192026	12.6763	11.8956	10.241	19.8137	24.7671	24.9065	21.0078	14.8948	22.9445
XLOC_048208	FARP1	chr12	79232346	79255359	6.66487	12.2601	12.3938	7.04301	5.82751	6.07913	5.19561	9.53536	4.81884
XLOC_048209	FARP1	chr12	79257132	79296411	11.8488	13.9826	15.5705	7.59225	6.79651	6.81505	7.77003	8.74644	6.78092
XLOC_049657	FARP1	chr12	79257132	79296411	0.0	2.31433	1.5131	0.693518	0.30024	2.39536	1.37761	0.955955	1.01106
XLOC_049658	FARP1	chr12	79257132	79296411	5.28318	1.26382	0.0	1.04154	1.07233	1.53311	2.76896	1.68706	1.29131
XLOC_049659	FARP1	chr12	79297338	79342192	0.0	2.01882	1.05596	2.90393	2.52462	5.03015	2.30649	0.805796	2.70889
XLOC_049660	FARP1	chr12	79297338	79342192	0.0	0.0	0.0	1.48879	0.634713	1.27125	0.712674	0.801302	1.79792
XLOC_049661	FARP1	chr12	79297338	79342192	0.0	0.667843	0.0	1.20071	2.42079	2.76058	4.15271	2.41943	3.69262
XLOC_049662	FARP1	chr12	79297338	79342192	0.604463	0.36166	1.89177	2.33025	3.74145	3.26676	2.3685	1.88032	4.38676
XLOC_049663	FARP1	chr12	79297338	79342192	22.2028	3.31945	0.0	9.06858	11.5187	11.1279	4.4354	6.11981	5.71989
XLOC_049664	FARP1	chr12	79297338	79342192	1.76615	1.23284	0.460631	2.16403	3.18313	2.20291	2.14649	1.94986	3.19169
XLOC_049665	FARP1	chr12	79297338	79342192	0.0	1.36332	0.89138	1.63422	1.68964	1.6531	0.925654	1.02272	1.89005
XLOC_049666	FARP1	chr12	79297338	79342192	0.0	0.0	0.0	1.10547	1.70833	2.41108	1.60146	0.545209	2.152
XLOC_049667	FARP1	chr12	79344567	79344986	0.0	1.65142	0.0	1.97949	3.19524	4.53872	1.69624	0.811127	4.07943
XLOC_049668	FARP1	chr12	79345903	79349393	0.434039	0.324735	0.169869	1.65447	2.3368	1.56006	1.53379	0.853378	1.99526
XLOC_049669	FARP1	chr12	79350911	79351125	0.0	0.0	0.0	4.27065	6.0815	12.9394	7.4776	3.22758	10.0825
XLOC_049670	FARP1	chr12	79351339	79351812	0.0	1.36944	1.79063	1.43627	1.41775	3.30116	1.21532	1.12686	1.59388
XLOC_049671	FARP1	chr12	79352265	79353672	0.578822	0.519484	0.452893	1.24547	2.44947	1.50417	1.31924	1.04581	1.87277
XLOC_049672	FARP1	chr12	79354022	79357844	0.197358	0.413447	0.463447	1.62851	1.55141	1.54227	1.61279	0.855866	1.88375
XLOC_049673	FARP1	chr12	79360861	79361358	0.0	0.636414	0.0	0.762829	1.97882	1.31607	1.32149	0.209848	1.2968

XLOC_049674	FARP1	chr12	79361804	79362299	0.0	0.640178	0.0	0.76734	0.829311	1.32376	1.13916	1.0553	1.49078
XLOC_049675	FARP1	chr12	79364123	79366860	0.560745	0.503408	1.31666	1.05608	1.3215	1.25553	1.15667	0.649682	1.57082
XLOC_049676	FARP1	chr12	79372437	79373590	0.726516	0.651943	0.568366	0.846639	1.99035	1.28265	1.25501	0.436761	1.90489
XLOC_049677	FARP1	chr12	79375173	79379057	0.582222	0.290407	0.455738	0.870339	1.00692	1.03132	0.552447	0.411043	1.15564
XLOC_049678	FARP1	chr12	79380155	79381347	0.0	0.836507	0.546953	1.06543	1.36838	1.52497	0.953926	0.700727	1.64993
XLOC_049679	FARP1	chr12	79381990	79382721	1.26116	0.377039	0.986074	0.677937	1.17951	1.04431	1.02181	0.251074	2.42019
XLOC_049680	FARP1	chr12	79383985	79385599	0.993112	0.891375	1.16567	0.979501	1.63546	1.23941	1.22416	0.548831	1.34647
XLOC_049681	FARP1	chr12	79386956	79388690	0.458758	0.411777	0.717991	0.863841	0.971631	1.09761	0.67068	0.599524	1.00335
XLOC_049682	FARP1	chr12	79388903	79389616	0.0	0.778485	0.0	1.04982	2.02889	1.61682	1.28839	1.16586	1.81691
XLOC_049683	FARP1	chr12	79393523	79394217	0.0	1.20903	0.0	0.72463	1.67998	1.53414	0.484688	0.536212	1.17559
XLOC_049684	FARP1	chr12	79404043	79405643	0.0	0.300007	0.392328	1.03396	1.33674	1.14712	0.732402	0.654874	1.22795
XLOC_049685	FARP1	chr12	79408700	79409537	0.0	0.0	0.0	0.953816	1.41251	1.76493	1.73106	0.318622	1.4866
XLOC_049686	FARP1	chr12	79410193	79410515	0.0	2.61669	0.0	2.35426	6.34687	1.78481	4.49158	2.10688	3.40943
XLOC_049687	FARP1	chr12	79410746	79412014	0.0	0.194823	0.509545	1.05095	1.42825	1.28569	0.652248	0.587837	1.70808
XLOC_049688	FARP1	chr12	79412132	79414135	0.0	0.234518	0.0	0.808258	0.922722	1.06036	0.717043	0.591413	1.47456
XLOC_049689	FARP1	chr12	79416810	79417349	0.0	0.0	0.0	1.52775	0.882116	1.75928	1.34971	1.31091	1.65
XLOC_049690	FARP1	chr12	79417453	79417871	0.0	2.4866	0.0	2.48383	2.56579	1.70844	1.45923	0.814138	2.89054
XLOC_049691	FARP1	chr12	79420358	79421212	0.0	0.0	0.0	0.930559	0.891856	0.968698	1.31415	0.621887	0.815881
XLOC_049692	FARP1	chr12	79421356	79421815	0.0	2.14933	0.0	1.28813	2.22351	1.7261	1.90399	1.17768	1.87567
XLOC_049693	FARP1	chr12	79422467	79423436	0.891259	0.799646	0.697125	0.7988	1.39339	1.66435	1.05019	1.33679	1.94619
XLOC_049694	FARP1	chr12	79423845	79424815	0.0	0.532442	1.39254	1.11695	1.39169	1.38526	1.53304	0.623075	1.39954
XLOC_049695	FARP1	chr12	79425430	79426190	0.0	1.43564	0.0	1.39825	1.68492	1.61593	1.0819	0.956661	2.61841
XLOC_049696	FARP1	chr12	79426364	79426764	0.0	0.890164	0.0	1.06704	1.83448	3.36038	0.780905	2.32644	1.55135
XLOC_049697	FARP1	chr12	79428107	79428877	0.0	1.41223	1.84672	1.05803	1.47348	0.978299	1.38401	1.05892	2.1637
XLOC_049698	FARP1	chr12	79429074	79430901	1.29961	0.388848	0.0	1.00996	1.15561	1.53241	1.42565	0.566305	1.21285
XLOC_084986	FAT2	chr7	64753444	64754126	0.0	0.412219	1.07807	0.370594	0.0	0.142631	0.0	0.0	0.0
XLOC_078831	FBLN1	chr5	116632779	116633604	93.1851	104.974	98.2935	8.8356	12.7701	9.54365	9.29714	6.2693	11.3492
XLOC_041564	FBN1	chr10	61838176	61895667	0.0	4.23398	11.0671	7.75886	4.15862	5.6528	1.11137	11.5467	3.63991
XLOC_041565	FBN1	chr10	62071016	62072475	14.8421	7.77	8.70938	71.8532	59.7366	65.9379	63.2677	116.397	70.4532
XLOC_041566	FBN1	chr10	62108405	62134980	0.859719	0.523014	0.0	0.985649	0.900077	0.969267	0.126837	0.347042	0.486729
XLOC_041956	FBN1	chr10	61838176	61895667	10.1742	9.02407	21.3644	2.53566	5.98676	2.76988	2.51529	3.55051	2.81496
XLOC_041957	FBN1	chr10	61961138	61979150	0.836615	3.00264	2.61769	0.524907	0.850549	1.12871	0.531354	1.50681	0.584713
XLOC_041958	FBN1	chr10	62108405	62134980	5.69868	9.12186	5.19999	8.41866	10.095	9.6791	5.20779	6.22626	10.8137

XLOC_042784	FBN1	chr10	61838176	61895667	0.0	1.52141	0.0	1.36778	0.594889	1.05343	0.114515	0.126625	0.443887
XLOC_042785	FBN1	chr10	61838176	61895667	2.85311	1.70336	0.0	0.0	0.219586	0.0	0.0	0.557238	0.742377
XLOC_042786	FBN1	chr10	61922181	61923108	0.0	0.0	0.0	1.01083	0.440663	0.584888	0.0851169	0.469639	0.328329
XLOC_042787	FBN1	chr10	61926651	61927374	0.0	1.52947	0.0	1.48962	0.697682	0.397114	0.460414	0.890997	0.669347
XLOC_042788	FBN1	chr10	61927821	61928439	0.0	0.469428	0.0	1.54741	1.09901	1.46037	0.562393	0.934539	0.957965
XLOC_042789	FBN1	chr10	61928756	61929639	0.0	0.89435	0.779682	0.804058	0.934352	1.447	0.450922	0.59733	0.696341
XLOC_042790	FBN1	chr10	61959621	61960335	2.59939	2.71981	0.0	0.116437	0.911377	0.134495	0.467683	0.258623	1.92701
XLOC_088341	FBP2	chr8	82416356	82417584	0.675613	0.0	0.528572	0.0	0.0	0.0	0.0	0.0	0.0
XLOC_086964	FBP2;CTSL1	chr8	82331644	82402439	2.25493	1.40947	0.768048	0.0	0.2359	0.116788	0.0	0.0	0.0
XLOC_088338	FBP2;CTSL1	chr8	82331644	82402439	0.0	0.432824	2.26391	0.0	0.0	0.0	0.0	0.0	0.0
XLOC_088339	FBP2;CTSL1	chr8	82331644	82402439	0.0	0.0	0.0	0.0	0.0	0.0	0.0	0.0	0.186897
XLOC_088340	FBP2;CTSL1	chr8	82331644	82402439	0.0	0.0	0.0	0.0	0.263087	0.116437	0.101439	0.0	0.0
XLOC_076451	FBXL14;WNT5B	chr5	108564298	108660794	14.3007	7.98801	9.06228	4.83377	7.03023	8.75396	4.55596	5.77131	9.32797
XLOC_076961	FBXL14;WNT5B	chr5	108564298	108660794	13.5768	15.271	12.6174	11.437	10.9629	11.6018	12.4264	12.2355	11.1542
XLOC_076962	FBXL14;WNT5B	chr5	108564298	108660794	3.68395	5.31241	3.94229	5.368	3.57609	4.49072	4.23256	4.26866	4.87675
XLOC_064252	FBXL20;MED1	chr19	40339934	40513796	4.61615	9.63488	5.63405	8.30598	8.70352	7.88465	10.2513	7.22799	7.89518
XLOC_065124	FBXL20;MED1	chr19	40339934	40513796	0.0	0.268534	1.40463	0.402373	0.421111	0.558902	0.406885	0.0897758	0.235277
XLOC_065125	FBXL20;MED1	chr19	40339934	40513796	0.0	0.0	0.0	0.0	0.352575	0.0	0.788441	0.0	0.0
XLOC_065126	FBXL20;MED1	chr19	40339934	40513796	0.0	0.0	1.28624	0.442153	0.639355	0.679774	0.441358	0.163051	0.43006
XLOC_065127	FBXL20;MED1	chr19	40339934	40513796	0.0	0.0	2.38654	0.547011	0.704958	0.626221	0.0	0.595825	0.530085
XLOC_079874	FBXL5	chr6	115601931	115676893	11.6134	16.3996	15.0879	13.8507	14.9866	13.7772	13.9442	16.4655	16.6755
XLOC_080272	FBXL5	chr6	115585272	115592580	0.0	0.361891	0.0	0.0	0.0	0.125314	0.0	0.0	0.105587
XLOC_080273	FBXL5	chr6	115601931	115676893	20.8117	24.2142	18.4375	30.181	26.5229	27.6375	32.3464	36.5669	29.6907
XLOC_082214	FBXL5	chr6	115578616	115579293	0.0	1.24855	0.0	0.623593	0.867173	0.57597	0.0	0.830194	1.33526
XLOC_082215	FBXL5	chr6	115601931	115676893	6.35012	2.27811	1.98598	2.27566	2.57337	2.10318	2.05765	1.26405	2.88022
XLOC_082216	FBXL5	chr6	115601931	115676893	8.80199	11.3851	13.7407	8.9232	6.77028	9.01864	6.15114	8.01505	7.88667
XLOC_082217	FBXL5	chr6	115601931	115676893	5.76441	5.15507	22.4635	8.25956	5.22483	5.24828	6.7548	7.65356	8.43806
XLOC_079873	FBXL5;MIR2448	chr6	115544635	115578348	1.27892	5.67801	4.44878	17.9601	17.148	18.2304	14.8462	20.3741	18.6351

XLOC_044704	FBXO11	chr11	30099359	30268141	64.3779	63.0103	63.0294	59.0628	57.7731	58.4924	63.1801	69.7565	57.8956
XLOC_044703	FBXO11;MSH6	chr11	29854063	30025963	1.46949	0.732755	0.0	0.30744	0.614625	0.560391	0.447306	0.24611	0.599861
XLOC_058972	FBXO2	chr16	42908410	42909332	0.0	2.26337	4.43966	0.508718	0.443523	0.294345	0.256992	0.18907	0.90879
XLOC_058973	FBXO2	chr16	42909664	42910571	0.964991	2.30863	0.754739	0.691854	0.452331	0.400268	0.349391	0.0	0.84265
XLOC_058974	FBXO2	chr16	42910799	42915337	2.31138	1.28469	3.10166	0.503486	0.88275	0.825884	0.288201	0.266514	0.824161
XLOC_041576	FBXO34	chr10	67979575	68042923	3.8043	5.6905	4.76272	10.7969	12.6721	10.4673	11.8648	11.6728	12.4381
XLOC_042882	FBXO34	chr10	68043071	68043565	4.29913	2.56831	3.35824	4.04048	4.9904	3.98294	4.18886	2.75172	4.67242
XLOC_042883	FBXO34	chr10	68043855	68045298	1.68784	0.168315	0.440217	1.10973	1.0583	1.28673	1.28268	0.677819	1.32842
XLOC_042884	FBXO34	chr10	68046192	68047190	0.860505	0.772077	0.673093	0.694136	0.87467	0.803612	0.390204	0.946843	0.902037
XLOC_065801	FBXO36	chr2	118814198	118837232	9.75827	18.8088	6.60417	37.3278	38.7862	38.848	45.2886	44.7589	43.0051
XLOC_065802	FBXO36	chr2	118861968	118881514	5.86171	4.02385	5.06326	12.5087	13.4279	12.3474	12.4484	14.7871	14.1259
XLOC_068342	FBXO36	chr2	118790800	118791658	0.0	0.0	0.0	0.0	0.0806186	0.0	0.0	0.0	0.0
XLOC_068343	FBXO36	chr2	118792173	118794928	0.55686	0.0	0.0	0.0499412	0.0	0.0	0.0	0.028052	0.0
XLOC_068344	FBXO36	chr2	118814198	118837232	0.835648	0.999723	1.30735	2.17211	4.05181	4.68311	3.94271	2.17401	5.54838
XLOC_058971	FBXO44	chr16	42901339	42902556	0.682628	1.22517	2.13622	0.673141	0.855212	0.638202	0.248454	0.410598	1.13373
XLOC_039636	FBXO45	chr1	71714020	71714465	7.54761	6.00993	0.0	34.4428	36.1094	34.3685	38.9651	37.9858	38.4497
XLOC_058964	FBXO6	chr16	42882179	42883040	1.02809	1.53715	1.60808	0.552785	0.883054	1.17226	0.185903	0.513147	0.897547
XLOC_058965	FBXO6	chr16	42883290	42885632	1.98643	1.18881	3.10932	0.65318	0.727916	0.654875	0.181937	0.300005	0.608513
XLOC_058966	FBXO6	chr16	42886101	42887291	0.70048	1.25719	1.64404	1.06749	0.877451	0.94585	0.3823	0.491451	0.734718
XLOC_058967	FBXO6	chr16	42888784	42889499	2.59473	1.16354	2.02868	0.232456	1.01084	0.805534	0.700296	0.0	0.678907
XLOC_058968	FBXO6	chr16	42889601	42890362	4.79386	2.1499	1.87423	0.859034	1.40179	2.35786	1.08015	0.358162	1.1502
XLOC_058969	FBXO6	chr16	42893124	42894107	0.876143	1.31016	4.11188	0.549685	0.821956	0.818136	0.556052	0.350496	0.765314
XLOC_058970	FBXO6	chr16	42898427	42899146	1.28812	0.0	3.02136	1.03861	1.00372	0.533227	0.347712	0.128178	1.23584
XLOC_076871	FBXO7;SYN3	chr5	71442931	71548267	17.0949	45.3186	28.3767	17.4055	3.96913	7.91942	6.81555	2.73588	5.20242
XLOC_088569	FBXW12	chr8	91667603	91668040	15.5271	14.681	24.2474	15.9765	15.3676	17.2698	19.1047	12.4216	11.9069
XLOC_088570	FBXW12	chr8	91668189	91668611	2.73452	2.44912	2.13483	2.69102	4.84501	5.61013	4.79412	5.08078	4.27089
XLOC_059549	FBXW7	chr17	4994312	5343849	14.4074	15.7477	15.0109	7.00312	6.89576	7.86699	7.92765	8.05158	5.60484
XLOC_060390	FBXW7	chr17	4994312	5343849	2.15108	2.8943	1.68213	1.34923	1.0913	1.56031	2.42879	1.93137	1.5959
XLOC_060391	FBXW7	chr17	4994312	5343849	7.79038	1.16179	0.0	1.04487	1.19007	0.794162	1.00449	0.375977	1.34694
XLOC_060392	FBXW7	chr17	4994312	5343849	0.850464	0.254335	2.66074	0.762201	0.465476	0.529495	0.848514	0.680601	1.3372
XLOC_060393	FBXW7	chr17	4994312	5343849	0.0	1.12524	3.92379	1.12402	0.977861	0.649355	1.01662	0.624468	0.87551
XLOC_060394	FBXW7	chr17	4994312	5343849	0.0	3.12344	1.16695	0.401145	1.16134	1.38873	1.20441	0.740902	0.910764

XLOC_045152	FCN1	chr11	106821624	106920240	26.89	26.1445	28.5095	17.1637	17.7191	18.8384	17.1404	15.5344	18.8726
XLOC_041445	FEM1C	chr10	4265398	4358087	70.6802	83.3731	75.5823	270.631	246.372	265.763	339.774	404.299	296.817
XLOC_083172	FER	chr7	110627595	110630679	5.85329	1.33937	3.23363	5.83574	5.46082	7.49109	3.08971	1.31713	4.97214
XLOC_083173	FER	chr7	110768275	110812556	3.041	0.50839	1.03423	3.12228	2.74447	2.74895	5.04306	4.04693	5.19849
XLOC_083832	FER	chr7	110428223	110614938	29.8487	31.0633	29.6803	22.9113	23.8036	22.0964	23.896	18.8567	23.9696
XLOC_083833	FER	chr7	110814988	110844526	16.4257	24.5329	18.8074	14.3652	15.6186	14.0345	14.6258	13.4492	20.9798
XLOC_083834	FER	chr7	110851510	110863311	2.27968	0.682125	1.78408	0.0	0.0	0.0	0.0	0.0	0.0
XLOC_085639	FER	chr7	110615448	110616003	0.0	1.08741	0.0	0.488789	0.0	0.187695	0.162147	0.0	0.158401
XLOC_085640	FER	chr7	110630778	110632217	0.0	0.0	0.0	0.0	0.044233	0.0	0.0	0.0	0.0987079
XLOC_085641	FER	chr7	110632410	110633420	0.0	0.0	0.0	0.0	0.0	0.0880399	0.0	0.0	0.0
XLOC_085642	FER	chr7	110768275	110812556	4.00229	0.0	0.0	0.0	0.0	0.0	0.0	0.0	0.0
XLOC_085643	FER	chr7	110768275	110812556	0.0	0.0	0.0	0.0	0.0	0.0	0.315095	0.0	0.0
XLOC_085644	FER	chr7	110768275	110812556	0.0	0.0	0.0	0.0	0.0	0.261531	0.113729	0.0	0.0
XLOC_085645	FER	chr7	110812726	110814575	2.13784	4.86137	4.01518	2.53042	3.45545	2.84712	2.85359	3.61186	4.52654
XLOC_085646	FER	chr7	110814988	110844526	0.0	8.56196	8.39557	3.52871	3.84456	4.02873	2.16948	3.4743	2.48332
XLOC_085647	FER	chr7	110850523	110851430	1.92998	1.73147	0.0	0.0	0.0	0.0	0.0	0.0	0.0
XLOC_050536	FERMT1	chr13	48665041	48666504	2.46423	3.68514	4.49778	0.368126	0.44972	0.170509	0.0	0.0822055	0.28706
XLOC_050837	FERMT1	chr13	48590523	48625560	1.54056	9.66929	3.61252	1.79375	2.15639	0.63673	0.551915	0.458479	1.34243
XLOC_051788	FERMT1	chr13	48590523	48625560	1.05202	1.88747	1.64547	0.942728	0.903433	0.218066	0.475364	0.314962	0.64285
XLOC_062434	FFAR3	chr18	46240558	46280980	8.78068	13.6903	14.243	9.12716	8.99806	9.20855	10.1399	10.5383	10.2332
XLOC_092531	FGD1;TSR2	chrX	97336097	97377224	2.18649	1.69818	1.69	22.5816	22.8662	23.769	17.2019	14.7294	16.5678
XLOC_086996	FGD3;BICD2	chr8	85718414	85840958	41.7936	53.1814	36.2032	31.5677	46.7265	36.7218	45.0467	26.4828	42.1554
XLOC_086547	FGD3;C8H9orf89;NINJ1	chr8	85841013	86062232	13.4802	13.5831	6.51855	12.8163	20.9855	19.4518	18.5633	12.1402	15.9598
XLOC_037927	FGF12	chr1	75360082	75367810	4.62123	1.93601	2.89349	4.69694	8.85586	4.7476	3.58513	5.92636	7.98224
XLOC_037928	FGF12	chr1	75382142	75432270	4.35334	1.26498	1.48298	2.63887	3.88817	2.55101	1.48988	1.86012	1.68105
XLOC_037929	FGF12	chr1	75435867	75443212	8.49218	13.9217	13.7166	23.0815	26.6603	22.8959	22.5927	25.4069	30.2904
XLOC_037930	FGF12	chr1	75450647	75473937	0.741201	0.979203	1.40079	0.193745	0.139117	0.223508	0.0	0.0372957	0.0324319
XLOC_037931	FGF12	chr1	75474460	75512035	1.85689	0.624815	2.76938	0.499409	0.835575	0.808745	0.61933	0.777772	0.639191
XLOC_037932	FGF12	chr1	75474460	75512035	117.168	138.675	120.067	0.606702	1.24657	0.719101	0.819024	0.534317	1.79736
XLOC_037933	FGF12	chr1	75521126	75529173	4.2577	6.48571	6.66412	10.7457	10.0254	8.1773	9.73885	10.6319	9.31441
XLOC_037934	FGF12	chr1	75529267	75535425	5.60868	10.9569	10.7197	12.4877	13.5233	13.3517	12.747	14.26	13.5002
XLOC_037935	FGF12	chr1	75546306	75560597	1.31783	3.80729	3.96373	0.9526	1.48493	1.74419	1.10145	0.594482	1.22437

XLOC_038426	FGF12	chr1	75308437	75317627	13.2628	17.8597	20.3986	38.3209	45.6831	36.435	38.2192	40.7267	50.0496
XLOC_038427	FGF12	chr1	75474460	75512035	37.4788	39.4744	42.102	16.5008	16.6704	16.8873	14.213	18.7018	19.1499
XLOC_039682	FGF12	chr1	75382142	75432270	1.11187	0.997362	0.0	0.199259	0.260281	0.0	0.0	0.221756	0.194077
XLOC_039683	FGF12	chr1	75450647	75473937	0.0	0.0	0.0	0.0834773	0.0727866	0.0	0.0	0.0	0.16269
XLOC_039684	FGF12	chr1	75450647	75473937	0.0	0.0	0.0	0.0	0.0	0.0	0.0	0.0837114	0.0
XLOC_039685	FGF12	chr1	75474460	75512035	0.67756	2.63482	2.12023	0.364419	0.477476	0.140778	0.184972	0.271714	0.473796
XLOC_039686	FGF12	chr1	75535868	75536712	4.21422	7.56088	4.11965	11.8957	11.6793	9.82718	10.4729	12.8269	10.6684
XLOC_047929	FGF14	chr12	82169427	82450213	28.2805	44.4408	36.101	11.7631	12.2657	13.2379	16.183	15.674	15.5837
XLOC_049803	FGF14	chr12	82169427	82450213	2.56952	6.13792	6.01891	0.459966	0.991019	0.527781	0.677717	0.25176	1.78462
XLOC_049804	FGF14	chr12	82450303	82451300	0.861531	0.515331	1.34779	0.38609	0.875704	0.536376	1.09385	0.43089	1.05363
XLOC_049805	FGF14	chr12	82496115	82496273	0.0	36.2312	0.0	53.5396	42.5589	19.8334	23.7086	37.8843	17.1598
XLOC_059766	FGF2	chr17	35240715	35253861	73.723	88.2514	72.4498	191.059	132.139	166.943	188.936	262.378	157.683
XLOC_060157	FGF2	chr17	35220031	35236499	2.23289	1.60343	2.79696	4.63481	6.09116	4.39253	3.88621	4.81933	3.67995
XLOC_060158	FGF2	chr17	35240715	35253861	16.7552	21.4392	16.1222	20.908	19.7514	19.5227	23.2407	21.9425	21.4357
XLOC_061313	FGF2	chr17	35220031	35236499	0.393165	0.941132	0.0	0.634613	0.864008	1.26839	0.827269	0.316444	0.894499
XLOC_061314	FGF2	chr17	35238328	35238857	0.0	0.0	0.0	0.522933	0.754579	0.802701	0.865478	0.768784	0.508232
XLOC_061315	FGF2	chr17	35254048	35254795	0.0	0.0	0.0	0.549632	0.478253	0.762136	0.442091	0.366541	0.642215
XLOC_041950	FGF7	chr10	61043131	61054865	0.0	0.0	0.0	2.39835	1.45656	1.8934	2.40681	4.60423	1.8937
XLOC_042765	FGF7	chr10	61024721	61025423	0.0	0.0	0.0	0.119	0.206945	0.27487	0.0	0.26423	1.1584
XLOC_042766	FGF7	chr10	61033185	61033617	0.0	0.0	0.0	1.4145	0.609417	0.811355	0.0	0.773924	2.28712
XLOC_042767	FGF7	chr10	61036466	61037243	0.0	0.0	0.0	0.627655	0.364247	0.241831	0.21057	0.349043	1.01874
XLOC_042768	FGF7	chr10	61038220	61038514	0.0	0.0	0.0	1.88685	2.79428	1.06824	0.888044	0.0	5.90509
XLOC_042769	FGF7	chr10	61043131	61054865	0.0	0.0	0.0	1.25634	0.0	0.576001	2.70486	3.56765	1.94924
XLOC_042770	FGF7	chr10	61043131	61054865	0.0	0.0	0.0	0.226268	0.39483	0.0873266	0.992452	0.420967	0.808644
XLOC_042771	FGF7	chr10	61055275	61056193	0.0	0.0	0.0	0.596624	0.965983	0.493141	0.861065	0.760211	0.166101
XLOC_090065	FGFR1OP	chr9	103352996	103365669	75.6783	78.4816	79.241	117.414	97.0098	114.257	132.088	145.255	119.44
XLOC_091644	FGFR1OP	chr9	103352996	103365669	15.2251	4.54414	11.8827	13.8906	6.55254	13.0956	7.16811	12.7583	9.76571
XLOC_091645	FGFR1OP	chr9	103352996	103365669	0.0	12.5143	9.34825	11.2833	10.4064	10.3063	17.0181	10.2182	14.9706
XLOC_091646	FGFR1OP	chr9	103352996	103365669	17.6566	9.21367	10.325	22.8963	15.7924	15.7096	22.5846	22.0382	19.4356
XLOC_091647	FGFR1OP	chr9	103365903	103366579	0.0	2.08492	4.36212	1.62446	0.32581	0.865606	0.375814	0.970397	1.33782
XLOC_082147	FGFRL1	chr6	108859950	108860246	0.0	1.54916	0.0	2.79008	1.57485	0.526727	0.438196	1.48575	0.895775
XLOC_082148	FGFRL1	chr6	108860366	108860684	4.49564	1.34049	0.0	1.60823	2.0522	1.37091	1.91446	0.862573	1.94025
XLOC_082149	FGFRL1	chr6	108860891	108861056	0.0	9.65524	0.0	9.09505	9.15983	3.18089	2.40009	0.0	16.4888

XLOC_082150	FGFRL1	chr6	108861133	108862194	0.8005	0.71828	0.626195	0.932782	1.44028	0.997137	1.45359	0.7212	1.04915
XLOC_079848	FGFRL1;PDE6B;ATP5I	chr6	108783002	108859801	67.5008	45.4004	42.3682	42.9302	49.888	42.6192	58.2423	47.8363	47.1448
XLOC_082143	FGFRL1;PDE6B;ATP5I	chr6	108783002	108859801	0.0	0.0	4.26083	0.489312	0.827066	1.66063	1.37792	0.0	2.35455
XLOC_082144	FGFRL1;PDE6B;ATP5I	chr6	108783002	108859801	0.530428	0.793453	0.830129	0.285348	0.540594	0.716991	0.629223	0.479472	0.32474
XLOC_082145	FGFRL1;PDE6B;ATP5I	chr6	108783002	108859801	0.0	0.0	1.08644	0.248958	0.540954	0.862318	0.499227	0.552418	0.0
XLOC_082146	FGFRL1;PDE6B;ATP5I	chr6	108783002	108859801	1.15447	0.345176	0.902825	0.310325	0.270161	0.35873	0.41655	0.345198	0.201481
XLOC_069955	FGGY	chr3	86988358	86989561	0.0	1.19222	0.0	1.96494	1.54604	1.23358	2.12686	2.36224	1.38869
XLOC_070338	FGGY	chr3	86785603	86920906	5.91896	6.37697	6.3252	35.4209	32.3825	34.695	40.0174	40.44	35.6461
XLOC_070339	FGGY	chr3	86995710	86999332	4.81484	1.75138	2.10603	3.05563	4.68133	2.80832	4.31375	3.88055	5.00991
XLOC_070340	FGGY	chr3	87080732	87112135	3.5359	2.57703	2.24676	4.6438	4.41013	4.25915	4.55615	5.3503	2.97253
XLOC_070341	FGGY	chr3	87141054	87174519	0.0	0.0	0.0	4.66614	3.7048	10.1143	6.56421	12.1528	4.34819
XLOC_070342	FGGY	chr3	87252457	87256882	20.2711	2.7531	1.43998	0.0	0.0	0.380118	0.0	0.0	0.0
XLOC_071539	FGGY	chr3	87078705	87080617	5.35878	2.2201	2.25812	3.43762	4.26986	3.86037	2.60112	4.51938	2.4887
XLOC_071540	FGGY	chr3	87117231	87117991	0.0	0.717822	1.87733	0.215115	0.0936065	0.124302	0.216381	0.0	0.104736
XLOC_071541	FGGY	chr3	87121480	87122348	0.0	0.0	0.0	0.45612	0.476959	0.422102	0.0920527	0.101631	0.0888736
XLOC_071542	FGGY	chr3	87123936	87124833	0.0	0.0	1.52987	0.7012	0.1528	1.11553	0.265523	0.390771	0.256202
XLOC_071543	FGGY	chr3	87130870	87132784	0.0	0.123198	0.0	0.664588	0.743147	0.642664	0.414203	0.372737	0.720542
XLOC_071544	FGGY	chr3	87133955	87134988	0.826103	0.0	0.646206	0.296182	0.452272	0.428694	0.149937	0.330681	0.433041
XLOC_071545	FGGY	chr3	87140073	87140424	0.0	0.0	0.0	1.00194	0.285606	0.381024	0.321898	0.361143	0.0
XLOC_071546	FGGY	chr3	87141054	87174519	0.0	0.264594	0.0	0.872237	0.899105	0.826098	2.00502	1.32708	1.23645
XLOC_071547	FGGY	chr3	87141054	87174519	1.8763	0.0	0.0	0.839961	1.89171	1.54777	2.50534	1.29762	0.489898
XLOC_071548	FGGY	chr3	87207337	87208002	24.2283	34.5068	28.9675	50.6824	40.6007	56.2944	42.0844	46.4385	47.0879
XLOC_072085	FHL3	chr3	108554052	108554879	97.2279	95.6163	97.1553	174.249	130.526	152.021	105.092	120.813	117.398
XLOC_089841	FIG4	chr9	41011021	41157746	5.22171	3.39933	3.39941	0.105384	0.215644	0.127658	0.101336	0.0790556	0.114342
XLOC_090646	FIG4	chr9	41011021	41157746	3.23988	0.0	2.53221	0.0	0.0	0.0	0.0	0.0	0.0
XLOC_090647	FIG4	chr9	41011021	41157746	2.48139	0.740959	1.93767	0.0	0.0	0.0	0.0	0.0	0.0
XLOC_090648	FIG4	chr9	41011021	41157746	1.24383	0.743716	3.8901	0.0	0.0	0.0	0.0	0.0	0.0
XLOC_090649	FIG4	chr9	41011021	41157746	5.50711	0.0	0.0	0.0	0.0	0.0	0.0	0.0	0.0
XLOC_090650	FIG4	chr9	41011021	41157746	2.62544	1.56774	2.04986	0.0	0.0	0.0	0.0	0.0	0.0
XLOC_090651	FIG4	chr9	41011021	41157746	0.0	1.81706	4.75068	0.0	0.0	0.0	0.0	0.0	0.0

XLOC_090652	FIG4	chr9	41011021	41157746	0.0	1.2617	1.64977	0.0	0.0	0.0	0.0	0.0	0.0
XLOC_090653	FIG4	chr9	41011021	41157746	4.36145	0.0	0.0	0.0	0.0	0.0	0.0	0.0	0.0
XLOC_090654	FIG4	chr9	41011021	41157746	1.67907	2.5109	2.62679	0.0	0.0	0.0	0.0	0.0	0.0
XLOC_090655	FIG4	chr9	41011021	41157746	2.958	4.41455	2.30878	0.0	0.0	0.0	0.0	0.0	0.0
XLOC_090656	FIG4	chr9	41011021	41157746	0.0	1.53489	1.00355	0.0	0.0	0.132837	0.0	0.0	0.111952
XLOC_090657	FIG4	chr9	41011021	41157746	4.4957	5.36194	7.01007	0.0	0.0	0.0	0.0	0.0	0.0
XLOC_090658	FIG4	chr9	41011021	41157746	11.8984	0.0	9.27342	0.0	0.0	0.601488	0.0	0.0	0.0
XLOC_090659	FIG4	chr9	41011021	41157746	0.0	3.41243	0.0	0.0	0.0	0.0	0.0	0.0	0.0
XLOC_090660	FIG4	chr9	41011021	41157746	4.70183	0.936825	2.45003	0.0	0.0	0.0	0.140302	0.0	0.136557
XLOC_077489	FIGNL2	chr5	28197612	28200070	0.0	0.0	0.0	0.789414	0.938198	0.78558	1.15211	0.443234	0.825521
XLOC_089279	FILIP1	chr9	15239094	15259207	1.37802	0.549733	0.359451	4.20113	1.18905	4.34823	0.923346	1.01586	1.64757
XLOC_090286	FILIP1	chr9	15239094	15259207	0.0	0.0	0.0	1.21301	0.454196	0.736419	0.0	0.129245	0.168986
XLOC_090287	FILIP1	chr9	15239094	15259207	0.0	0.886928	0.0	1.06315	0.346285	0.766792	0.133022	0.29457	0.0
XLOC_090288	FILIP1	chr9	15239094	15259207	0.0	0.0	0.0	1.17934	0.321592	0.512105	0.149266	0.246894	0.359231
XLOC_050627	FITM2	chr13	73467416	73516522	5.96029	6.94381	4.66536	17.011	16.4468	16.6775	15.3973	18.6951	15.6383
XLOC_052477	FITM2	chr13	73467416	73516522	8.56912	1.27769	0.0	3.83148	2.28493	6.53892	1.46432	3.29527	4.07027
XLOC_076266	FKBP11;CCDC65	chr5	31048293	31078683	35.5088	32.9986	37.9609	74.4267	54.9674	66.4256	60.2822	76.9847	46.2498
XLOC_076439	FKBP4;WASH1	chr5	107437917	107518155	2.40831	2.14127	2.75276	2.24395	3.28764	2.40519	2.28042	1.96375	2.86141
XLOC_078568	FKBP4;WASH1	chr5	107437917	107518155	66.9249	49.7412	53.643	58.2401	46.3734	55.0843	56.6848	77.1208	54.694
XLOC_047822	FLT1;MIR2300B;<br>MIR2300A	chr12	31640576	31950275	8.27548	11.4747	10.8383	12.6039	14.327	13.5823	15.063	13.554	16.6382
XLOC_048119	FLT1;MIR2300B;<br>MIR2300A	chr12	31640576	31950275	2.66021	5.25055	2.08126	4.47355	5.70372	6.02145	4.5173	5.25015	6.3079
XLOC_048120	FLT1;MIR2300B;<br>MIR2300A	chr12	31640576	31950275	71.7809	97.5397	80.1912	37.2465	37.6013	39.9157	34.5733	32.7469	44.904
XLOC_048976	FLT1;MIR2300B;<br>MIR2300A	chr12	31640576	31950275	0.0	1.60307	1.39734	1.92138	1.24928	1.4762	1.11603	1.06113	2.02401
XLOC_048977	FLT1;MIR2300B;<br>MIR2300A	chr12	31640576	31950275	5.47424	4.09114	6.41954	2.94234	2.55792	2.97304	4.05769	2.85702	4.1767

XLOC_048978	FLT1;MIR2300B;<br>MIR2300A	chr12	31640576	31950275	0.0	4.55149	3.9665	1.36593	0.77234	1.54896	1.28922	0.970891	2.63267
XLOC_048979	FLT1;MIR2300B;<br>MIR2300A	chr12	31640576	31950275	13.1411	28.4143	33.9944	5.07288	3.86832	5.23983	6.12523	3.33067	5.73668
XLOC_048980	FLT1;MIR2300B;<br>MIR2300A	chr12	31640576	31950275	13.2115	9.86538	17.1992	3.15327	3.23657	3.17269	3.70226	1.73351	4.21133
XLOC_065636	FMNL2;ARL6IP6	chr2	43555299	43681451	0.0	0.0	0.0	0.0	0.0	0.056096	0.0	0.0541827	0.0
XLOC_044439	FNBP1	chr11	100319990	100322353	28.1926	18.9064	18.2862	29.2613	37.273	38.6442	44.085	38.6945	29.4386
XLOC_044440	FNBP1	chr11	100368822	100397925	3.46864	4.98797	4.66727	10.0946	6.83053	8.13911	7.95283	16.668	5.55591
XLOC_045075	FNBP1	chr11	100348485	100356230	5.85703	9.46454	5.62618	21.6598	25.7459	19.1896	30.4413	30.9123	38.9111
XLOC_045076	FNBP1	chr11	100368822	100397925	25.1227	24.6375	21.5959	48.19	27.0677	41.0104	36.8878	70.8459	25.6643
XLOC_047261	FNBP1	chr11	100322415	100322637	21.0445	25.1574	57.5404	40.0445	34.365	46.431	38.8139	26.1694	44.2746
XLOC_047262	FNBP1	chr11	100347321	100348315	2.59386	7.49907	2.02893	13.4066	19.8073	13.0087	22.0327	23.0047	28.7007
XLOC_047263	FNBP1	chr11	100348485	100356230	0.0	3.03139	2.64225	5.14838	5.45169	4.1533	4.99094	6.90274	10.8489
XLOC_047264	FNBP1	chr11	100361397	100362159	51.4488	72.9759	54.2628	163.376	117.838	159.204	142.133	185.583	142.181
XLOC_047265	FNBP1	chr11	100367902	100368437	1.91607	0.572462	0.0	1.20082	0.742768	1.18514	1.02264	1.13527	1.50063
XLOC_048587	FNDC3A	chr12	18853379	18855083	0.0	0.0	0.0	2.09664	1.72404	1.75123	1.06809	1.22217	1.96373
XLOC_048588	FNDC3A	chr12	18855173	18856348	0.0	0.0	0.0	0.57347	0.500801	0.959733	0.258575	0.569866	0.434887
XLOC_048589	FNDC3A	chr12	18857343	18859106	0.0	0.0	0.0	0.646281	0.918805	1.07784	0.823333	1.53982	0.709381
XLOC_048590	FNDC3A	chr12	18859374	18859702	0.0	0.0	0.0	3.02954	0.968199	3.44772	2.17263	4.07331	2.56042
XLOC_048591	FNDC3A	chr12	18861260	18861772	4.08081	1.21907	3.18806	3.65307	3.95091	3.36286	3.44072	2.61543	3.54951
XLOC_048592	FNDC3A	chr12	18863956	18864493	0.0	0.0	0.0	1.36515	0.886694	1.96494	1.01744	0.75295	1.32691
XLOC_048593	FNDC3A	chr12	18864940	18865234	0.0	0.0	4.10836	4.71711	3.99182	6.40946	3.9962	4.01605	5.45085
XLOC_048594	FNDC3A	chr12	18865454	18866510	0.0	0.0	0.0	0.577211	0.440769	1.16977	0.73078	1.04747	0.703314
XLOC_048595	FNDC3A	chr12	18872211	18872421	12.4499	0.0	9.74202	102.103	67.1405	92.2622	87.8794	104.666	112.269
XLOC_048596	FNDC3A	chr12	18877024	18877523	0.0	0.0	1.65459	1.70633	1.31161	1.74461	0.938606	1.2519	0.920904
XLOC_048597	FNDC3A	chr12	18882147	18882682	1.91607	0.0	0.0	2.23009	1.48554	1.97523	2.04528	2.45975	2.50104
XLOC_048598	FNDC3A	chr12	18882856	18886279	0.0	0.0662793	0.0	0.774681	0.835503	1.08442	0.589463	0.893168	1.10535
XLOC_048599	FNDC3A	chr12	18887046	18887599	0.0	0.0	0.0	0.81876	0.851342	0.754535	0.32588	0.18079	0.477592
XLOC_048600	FNDC3A	chr12	18890873	18892133	0.0	0.0	0.0	0.99976	0.976162	1.22682	0.955525	0.592069	1.6631
XLOC_048601	FNDC3A	chr12	18897136	18897542	2.91041	0.868752	0.0	0.260341	1.56731	1.49123	1.01737	1.70417	1.00953

XLOC_048602	FNDC3A	chr12	18898037	18898457	0.0	1.64514	4.30207	2.21847	2.97112	4.52172	1.44866	1.88566	1.91249
XLOC_048603	FNDC3A	chr12	18898596	18899310	0.0	0.0	0.0	1.16437	2.12655	1.34495	1.98765	1.68105	0.680122
XLOC_048604	FNDC3A	chr12	18905843	18906454	0.0	0.476664	0.0	1.85694	1.98356	1.97693	2.14058	1.73926	1.25057
XLOC_048605	FNDC3A	chr12	18911166	18912560	0.584908	0.349962	0.457652	1.20612	1.37506	1.39834	1.97279	1.52639	1.79012
XLOC_044216	FNDC4	chr11	72153973	72209499	82.2488	93.1485	89.2699	185.175	154.359	187.948	178.116	222.6	222.758
XLOC_044895	FNDC4	chr11	72153973	72209499	3.73752	5.1716	7.64316	3.8539	3.88279	3.45455	3.78733	3.8815	4.62177
XLOC_046525	FNDC4	chr11	72153973	72209499	1.33818	0.0	1.0462	0.479516	0.938069	0.830756	0.481189	0.532308	0.350079
XLOC_043001	FNTB	chr10	77485838	77487350	3.2037	2.71566	2.92462	3.15967	3.59989	3.10902	2.4847	3.91472	3.64253
XLOC_042013	FNTB;MAX	chr10	77487475	77515855	14.0622	13.3493	17.4545	19.8706	13.8821	15.8403	17.2295	22.369	13.065
XLOC_042102	FOS	chr10	86864668	86885692	1.67481	4.95537	2.42757	0.111265	0.14584	0.0	0.169587	0.0	0.0976436
XLOC_060289	FOXN4	chr17	66037188	66076917	10.6267	10.3245	14.5393	1.19	1.75903	3.57331	3.10531	2.51019	3.12768
XLOC_061829	FOXN4	chr17	66037188	66076917	5.79081	4.15537	3.62255	0.0	0.271018	0.359863	0.5223	0.230859	1.01062
XLOC_061830	FOXN4	chr17	66037188	66076917	3.96833	2.84972	3.10557	0.320265	0.342588	0.289075	1.23424	0.359304	0.312519
XLOC_061831	FOXN4	chr17	66037188	66076917	2.10575	5.03214	11.5148	1.13095	0.815066	0.433646	1.8667	0.622399	0.549349
XLOC_061832	FOXN4	chr17	66037188	66076917	10.1043	6.0391	2.63227	0.603241	0.523231	0.34771	1.50436	1.16739	0.440011
XLOC_061833	FOXN4	chr17	66037188	66076917	3.75079	5.60676	7.82049	1.23216	0.779611	0.905936	2.70183	0.995747	0.872496
XLOC_061834	FOXN4	chr17	66037188	66076917	4.56683	6.48595	2.67835	1.8414	0.979743	0.946096	1.64811	1.13812	1.19558
XLOC_060290	FOXN4;ACACB	chr17	66079212	66101529	66.5612	55.6615	33.9833	138.124	135.343	134.029	148.158	170.578	204.309
XLOC_089842	FOXO3	chr9	41964701	42124160	3.03584	2.17844	2.09583	8.02395	8.16902	7.62832	6.2869	7.93443	10.4182
XLOC_087564	FREM1	chr8	29407757	29407909	97.4648	285.547	235.94	67.2072	31.4924	19.6926	18.23	33.8882	34.118
XLOC_050491	FRMD4A	chr13	28950164	28957929	49.9204	63.2328	40.7516	15.4717	13.6079	17.1225	15.8695	20.9561	14.2873
XLOC_050792	FRMD4A	chr13	28950164	28957929	23.7225	28.8353	31.1943	3.51777	3.9907	0.792659	1.67968	1.87162	2.87788
XLOC_051491	FRMD4A	chr13	28947624	28948255	0.0	0.913116	1.19402	0.273634	0.475199	0.631401	0.136845	0.0	0.0
XLOC_051492	FRMD4A	chr13	28948370	28949057	4.09793	2.04166	6.40743	0.97893	0.850974	0.98907	0.981871	0.950571	0.238211
XLOC_051493	FRMD4A	chr13	28949192	28949993	56.1981	65.533	58.0091	16.2146	13.7682	15.2532	13.9986	23.7584	13.6345
XLOC_051494	FRMD4A	chr13	28958945	28959586	0.0	0.894256	0.0	0.133991	0.0	0.0	0.0	0.296963	0.130377
XLOC_041526	FRMD6	chr10	44268335	44364305	13.294	20.0927	18.1	19.981	17.6963	17.9172	22.7109	23.3743	25.2985
XLOC_041930	FRMD6	chr10	44437697	44528663	2.68571	1.30615	2.75929	6.27323	6.48993	6.49208	6.92893	5.06288	6.45533
XLOC_042667	FRMD6	chr10	44366797	44367874	0.0	0.235263	0.0	0.987068	0.738455	0.489935	0.500033	0.866275	1.30586
XLOC_042668	FRMD6	chr10	44389099	44389778	0.0	0.0	0.0	1.36665	0.647913	0.0	0.124569	0.137844	0.604618
XLOC_042669	FRMD6	chr10	44436997	44437593	1.64949	0.98589	0.0	1.4772	2.05053	2.04387	1.47441	0.490246	1.2931
XLOC_042670	FRMD6	chr10	44437697	44528663	1.12924	0.0	0.883049	2.02366	1.58585	1.52072	1.32476	0.900683	1.67531

XLOC_042671	FRMD6	chr10	44437697	44528663	1.43085	0.427711	0.0	1.40985	0.668188	0.443836	0.513635	0.142131	0.873121
XLOC_047816	FRY	chr12	28751813	28867830	6.51384	8.48416	9.59572	14.6717	10.9473	12.7609	13.8838	16.2699	13.7138
XLOC_081306	FRYL	chr6	68830450	68831400	0.0	0.0	0.0	1.88125	0.64195	0.662676	0.661505	0.912321	0.797078
XLOC_081307	FRYL	chr6	68834612	68835655	0.0	0.0	0.0	2.41592	0.83051	1.35641	2.00164	1.0627	0.927692
XLOC_081308	FRYL	chr6	68842919	68843181	0.0	0.0	0.0	6.11633	0.0	0.687559	1.69092	1.28252	1.17241
XLOC_066149	FRZB	chr2	13672622	13793465	0.0	0.0	2.29941	1.05405	0.226573	0.905471	2.57287	0.0	0.0
XLOC_082982	FSTL3	chr7	44895433	44929487	5.00126	2.22371	3.36134	6.29147	5.75077	5.28292	4.48424	6.16668	4.22415
XLOC_083612	FSTL3	chr7	44895433	44929487	17.9071	22.6101	19.8826	22.8831	17.1175	21.3862	22.5329	30.5452	14.6889
XLOC_059791	FSTL5	chr17	37270235	37272742	1.68492	1.5118	1.31798	0.377552	0.461167	0.524567	0.22929	0.505743	0.367992
XLOC_061385	FSTL5	chr17	37191887	37192003	0.0	0.0	0.0	60.9224	75.9149	56.2877	237.981	454.194	99.2588
XLOC_061386	FSTL5	chr17	37712266	37713940	4.29218	3.56718	1.49277	4.2762	6.09696	4.91088	4.66105	5.17657	6.46645
XLOC_061387	FSTL5	chr17	37860729	37861418	0.0	0.406797	0.0	1.09716	0.953782	0.98538	0.978268	2.0294	1.77989
XLOC_061388	FSTL5	chr17	37995848	37997333	1.08962	1.46693	0.426296	3.90776	3.24573	3.39861	3.28023	5.47098	3.47824
XLOC_061389	FSTL5	chr17	38002828	38003646	1.09503	2.29198	0.856328	2.15869	2.30738	4.19803	3.36195	4.80576	4.30088
XLOC_061390	FSTL5	chr17	38003822	38004605	0.0	0.691455	0.0	1.24328	0.811766	0.838347	1.35585	1.61346	2.01802
XLOC_061391	FSTL5	chr17	38012650	38012922	0.0	1.86277	4.87016	13.4409	11.3004	15.7801	16.6387	17.7094	17.7438
XLOC_061392	FSTL5	chr17	38013188	38013385	15.3807	4.61722	0.0	36.7555	23.7263	30.7763	26.4971	38.978	42.3074
XLOC_061393	FSTL5	chr17	38013505	38014578	1.58001	1.89032	3.08997	14.9415	12.361	13.4498	11.1198	18.3496	14.0819
XLOC_061394	FSTL5	chr17	38014878	38015359	0.0	0.0	1.74645	8.405	10.7206	12.8825	9.29408	14.0767	11.4665
XLOC_061395	FSTL5	chr17	38017948	38018213	0.0	0.0	5.17258	0.595223	5.49088	2.00899	1.09959	0.0	0.570795
XLOC_061396	FSTL5	chr17	38018613	38019226	0.0	2.37287	0.0	0.568861	1.35779	1.14819	0.284194	0.629734	0.138346
XLOC_062159	FTO	chr18	22517421	22522527	0.746001	2.00824	1.16719	0.401228	0.175149	0.38732	0.1356	0.0	0.0651945
XLOC_062786	FTO	chr18	22516064	22517179	0.0	0.903732	1.77272	0.270835	0.177331	0.313721	0.0686379	0.151311	0.0
XLOC_062787	FTO	chr18	22523229	22523925	1.34314	1.60606	1.05008	0.240648	0.313839	0.416861	0.241466	0.40069	0.0
XLOC_069928	FUBP1	chr3	66985683	67082615	13.7775	32.9287	23.0663	29.6024	26.0135	29.8165	36.2049	26.7572	41.717
XLOC_071379	FUBP1	chr3	66972042	66972907	0.0	0.0	0.0	0.183221	0.159655	0.0	0.0	0.0	0.0892491
XLOC_045088	FUBP3	chr11	100886647	100939539	61.9232	60.4932	59.1819	22.8213	19.1539	16.5481	30.9261	29.3874	24.8028
XLOC_093465	FUNDC1	chrX	104536973	104540331	0.677911	0.473376	0.707495	2.04696	2.04231	2.25991	2.42627	2.41483	2.21598
XLOC_045142	FUT7	chr11	106199231	106216360	26.5667	22.6419	23.0051	27.5753	36.1109	33.3894	34.3868	29.7252	38.3897
XLOC_041614	FUT8	chr10	78049810	78063999	0.0	0.0	0.622398	124.801	54.1072	86.1012	81.799	109.557	62.0119
XLOC_041615	FUT8	chr10	78067555	78079666	2.46973	0.865042	0.964808	4.58376	4.67571	4.12057	5.211	4.05563	4.34581
XLOC_042016	FUT8	chr10	77987821	78009768	9.24939	7.48961	7.45235	193.987	206.243	189.938	174.482	115.453	200.454
XLOC_042017	FUT8	chr10	78025619	78043853	0.0	0.0	0.0	52.0367	40.5267	45.3759	34.9866	24.7308	42.1123

XLOC_042018	FUT8	chr10	78159834	78169853	3.0797	3.19511	4.82126	8.61806	7.6819	7.67957	7.52075	8.69576	9.81612
XLOC_043006	FUT8	chr10	78016289	78016892	0.0	0.0	0.0	0.436207	0.883192	0.167673	0.0	0.321796	1.6972
XLOC_043007	FUT8	chr10	78018818	78019293	0.0	0.0	0.0	1.22335	1.93735	0.0	0.603991	0.0	0.791962
XLOC_043008	FUT8	chr10	78025619	78043853	0.0	0.0	0.0	1.65085	1.12795	0.817167	0.355123	0.130932	0.459163
XLOC_089906	FUT9	chr9	54419807	54501631	26.5734	28.0044	19.601	6.96111	6.87589	5.91387	7.04804	8.30391	6.58981
XLOC_065739	FZD7	chr2	90940537	91019531	42.8056	48.336	38.3033	80.3725	73.295	76.6094	91.3642	102.883	73.5183
XLOC_059973	GAB1	chr17	14666561	14680040	10.8612	3.49401	5.95069	2.82483	0.682621	1.83813	3.13704	1.3406	1.68772
XLOC_059974	GAB1	chr17	14680298	14694081	7.24722	3.82661	2.00168	1.83489	0.501743	1.68551	2.7673	0.900317	1.11898
XLOC_060619	GAB1	chr17	14665732	14666399	4.25881	2.97038	0.0	1.65312	0.331501	0.0	0.764561	0.564139	0.247514
XLOC_041554	GABPB1	chr10	60121121	60131383	43.2021	48.2266	47.6767	2.62778	3.13891	3.04363	2.31268	3.24469	2.89879
XLOC_041945	GABPB1	chr10	60184940	60219477	0.291211	0.731998	0.659567	0.835118	0.332364	0.793637	1.35254	1.30418	1.05294
XLOC_042755	GABPB1	chr10	60105932	60106660	12.678	18.572	16.8513	0.0	0.296412	0.393661	0.0	0.252374	0.0
XLOC_042760	GABPB1	chr10	60181736	60184203	0.626303	0.187415	0.735271	0.365086	0.78699	0.554288	0.889425	1.5453	1.09641
XLOC_042761	GABPB1	chr10	60184940	60219477	0.0	0.0	0.0	0.0	0.391145	0.0	0.225915	1.37383	0.437755
XLOC_042762	GABPB1	chr10	60184940	60219477	0.0	0.0	0.591523	0.203339	0.236688	0.157029	0.274835	0.302938	0.396469
XLOC_083058	GABRA1	chr7	75704406	75754887	48.6351	55.5901	45.8747	5.32402	4.92432	5.70371	6.58901	7.45221	4.1422
XLOC_085100	GABRA1	chr7	75703096	75704330	6.04663	2.41167	3.6794	0.481831	0.631334	0.0697961	0.0	0.673612	0.5873
XLOC_079748	GABRA2	chr6	66535329	66541148	28.2305	37.3873	28.3046	78.6872	81.5669	81.2379	92.7192	85.7468	90.2604
XLOC_081258	GABRA2	chr6	66541538	66542211	0.0	1.25822	0.0	4.1476	2.94917	4.2079	2.39365	4.46148	3.18042
XLOC_081259	GABRA2	chr6	66543422	66543989	1.76632	1.5834	0.0	2.68878	2.19372	3.09832	2.52076	2.44638	1.84547
XLOC_081260	GABRA2	chr6	66544090	66544327	0.0	2.61764	6.84286	3.9538	5.88939	7.93233	2.84873	4.07508	3.01338
XLOC_081261	GABRA2	chr6	66544769	66545225	0.0	5.789	1.89235	2.38524	2.99391	5.22958	2.99024	2.61618	3.57822
XLOC_081262	GABRA2	chr6	66545305	66545720	0.0	0.838492	2.19265	1.50761	1.94626	3.16801	1.47538	1.64661	1.21828
XLOC_081263	GABRA2	chr6	66546637	66547216	1.71602	1.02559	4.02322	1.84401	3.19816	1.94832	1.83842	2.37817	1.94268
XLOC_079752	GABRA4	chr6	67205579	67206146	190.834	177.766	179.542	366.825	379.656	383.612	459.57	471.156	383.186
XLOC_081289	GABRA4	chr6	67221027	67221881	0.0	0.0	0.0	1.02361	0.7297	1.2916	0.938677	0.621887	0.725227
XLOC_083057	GABRA6	chr7	75455551	75697895	100.82	98.3512	87.5042	12.503	12.4976	13.8397	10.1711	10.0171	10.8248
XLOC_081291	GABRB1	chr6	67288386	67288732	7.66684	17.1519	14.9497	39.7629	48.0416	47.687	43.8715	45.5437	48.3944
XLOC_081292	GABRB1	chr6	67289295	67289454	38.1205	35.0246	0.0	88.6083	63.1725	115.086	51.6647	60.0397	96.2327
XLOC_081293	GABRB1	chr6	67289588	67291917	4.32969	6.77693	5.47388	11.4095	14.1483	14.1119	15.2821	11.0322	14.1378
XLOC_081294	GABRB1	chr6	67292006	67293223	2.73051	3.87971	3.20434	8.0165	12.454	10.8494	12.6711	6.70644	11.218
XLOC_081295	GABRB1	chr6	67293535	67294410	4.03213	5.42588	7.88366	6.32345	10.7064	7.94218	10.7582	5.73713	11.7933
XLOC_081296	GABRB1	chr6	67294614	67295934	3.11071	3.16393	2.9206	3.51388	5.70239	3.23262	5.44065	1.87248	6.90815

XLOC_081297	GABRB1	chr6	67297438	67297771	8.23044	6.13654	9.62749	3.31191	4.70805	6.28549	9.87095	0.396318	8.17786
XLOC_081298	GABRB1	chr6	67298054	67298358	0.0	4.39893	0.0	2.19998	4.85202	1.49717	1.24901	0.0	4.24141
XLOC_081299	GABRB1	chr6	67298593	67299152	1.80152	1.61489	1.40776	2.25833	2.9358	0.557537	3.69344	0.534442	3.45033
XLOC_081300	GABRB1	chr6	67299865	67300171	0.0	1.44694	0.0	2.17075	2.57905	3.44808	2.46724	0.0	5.8602
XLOC_081301	GABRB1	chr6	67300260	67303925	2.26811	2.53	1.93675	1.14659	1.73419	1.0097	2.46081	0.49902	2.61799
XLOC_081302	GABRB1	chr6	67304175	67304717	0.0	1.12412	2.93977	1.34743	0.729446	1.16381	2.00931	0.185845	1.63721
XLOC_081303	GABRB1	chr6	67305461	67306640	3.54012	1.69429	1.66173	0.190409	0.720562	0.588302	0.515151	0.141913	1.11391
XLOC_081304	GABRB1	chr6	67533963	67535912	2.4222	1.44951	0.315929	0.0362005	0.126726	0.0840186	0.0369247	0.0406101	0.0
XLOC_079753	GABRB1;LOC536190	chr6	67238027	67288273	157.993	192.343	169.287	870.274	764.227	849.386	870.92	1085.99	956.425
XLOC_085096	GABRB2	chr7	75116336	75117023	0.0	0.0	2.13581	0.0	0.0	0.141296	0.0	0.0	0.0
XLOC_061894	GAL3ST1	chr17	71659462	71660218	2.41696	2.16784	2.8348	2.70688	1.41339	2.00203	1.74236	1.2037	2.5304
XLOC_061895	GAL3ST1	chr17	71660847	71661075	0.0	2.91216	0.0	0.881522	7.97817	0.978608	2.35782	1.80286	0.837327
XLOC_041951	GALK2	chr10	61098622	61147651	58.5332	53.7693	51.2814	26.4422	27.8915	27.1054	27.9787	28.9822	28.3196
XLOC_041952	GALK2	chr10	61198095	61273402	1.86715	0.647026	0.423477	7.584	5.57526	7.07595	3.69285	5.86524	4.59168
XLOC_042773	GALK2	chr10	61195814	61196442	0.0	0.0	0.0	1.78994	0.358644	0.317695	0.413081	0.152508	0.401878
XLOC_042774	GALK2	chr10	61197066	61198040	0.885801	0.794753	0.0	3.41383	3.04682	3.12462	2.24832	3.4546	2.39854
XLOC_043986	GALM;SRSF7;GEMIN6	chr11	21091440	21150760	15.1466	13.5638	14.1224	6.816	7.86469	7.5439	9.00206	7.82746	7.73552
XLOC_045610	GALM;SRSF7;GEMIN6	chr11	21091440	21150760	4.67871	5.59765	8.54006	3.07548	2.3184	2.75247	3.8246	1.79583	2.72554
XLOC_045611	GALM;SRSF7;GEMIN6	chr11	21091440	21150760	5.21357	4.8524	4.98581	2.18132	2.22444	2.04731	2.48214	1.33738	2.33017
XLOC_045612	GALM;SRSF7;GEMIN6	chr11	21091440	21150760	6.40795	4.78772	5.00842	2.29557	1.99224	1.32373	3.58298	1.74681	2.09347
XLOC_045613	GALM;SRSF7;GEMIN6	chr11	21091440	21150760	0.0	2.71314	5.32141	0.406507	1.05348	0.467213	2.00724	0.893229	0.592122
XLOC_045614	GALM;SRSF7;GEMIN6	chr11	21091440	21150760	2.83699	1.33393	4.12318	1.12662	0.82694	1.13869	1.59394	0.896909	1.02842
XLOC_045615	GALM;SRSF7;GEMIN6	chr11	21091440	21150760	6.45082	4.33773	7.5628	0.866588	2.38141	1.49906	2.308	0.639371	1.26443
XLOC_045616	GALM;SRSF7;GEMIN6	chr11	21091440	21150760	10.8561	1.62053	2.11886	0.485614	1.25464	0.278436	0.713983	0.0	2.35509
XLOC_045617	GALM;SRSF7;GEMIN6	chr11	21091440	21150760	8.66234	1.0354	2.7078	1.39624	0.538064	0.178799	1.70086	1.02878	1.65946
XLOC_045618	GALM;SRSF7;GEMIN6	chr11	21091440	21150760	3.53166	1.6604	4.73747	0.588078	0.632987	0.996893	1.56603	0.456203	1.32391
XLOC_045619	GALM;SRSF7;GEMIN6	chr11	21091440	21150760	1.27002	1.13905	0.0	0.341346	0.296928	0.525795	0.800197	0.0	0.664667

XLOC_045620	GALM;SRSF7;GEMIN6	chr11	21091440	21150760	6.44196	3.84483	2.51347	0.288069	0.988874	1.97696	1.11993	0.626443	1.39515
XLOC_045621	GALM;SRSF7;GEMIN6	chr11	21091440	21150760	12.5243	6.36532	7.83402	2.35636	2.04999	2.07427	2.25533	2.49361	2.51275
XLOC_065634	GALNT13	chr2	41987025	41988857	53.7676	70.5845	58.4199	248.616	200.752	230.847	237.202	267.65	234.159
XLOC_044160	GALNT14	chr11	68901396	68906809	432.177	369.217	372.374	816.336	861.89	837.582	844.816	748.868	1077.13
XLOC_044847	GALNT14	chr11	68944627	68960597	4.38236	6.8036	5.85018	0.855422	1.75123	1.13392	1.26468	1.68739	0.592887
XLOC_046451	GALNT14	chr11	68901396	68906809	7.27578	6.50926	11.3443	7.84193	8.73267	5.13171	1.19751	5.45824	6.25434
XLOC_046452	GALNT14	chr11	68901396	68906809	0.0	3.92526	3.42108	7.06279	10.0214	7.13927	5.98877	8.42753	7.95534
XLOC_046453	GALNT14	chr11	68907371	68907969	3.28399	0.0	0.0	0.294101	1.65859	0.678228	1.02754	0.488064	1.14423
XLOC_046454	GALNT14	chr11	68943240	68943973	6.28391	6.01169	6.87856	1.01338	0.881589	0.780533	2.14984	1.50129	0.767396
XLOC_046455	GALNT14	chr11	68944037	68944568	5.80977	9.8359	18.1571	1.21366	1.5012	2.59497	0.516604	1.72072	0.674039
XLOC_042075	GALNTL1	chr10	81398972	81427151	6.45133	7.84171	8.24391	3.16556	2.63091	3.81191	3.63432	4.07941	2.1339
XLOC_042076	GALNTL1	chr10	81462675	81482612	0.0	0.230979	0.604146	3.62577	3.27124	3.71311	1.17715	2.32131	1.63766
XLOC_042077	GALNTL1	chr10	81486435	81496420	11.3588	12.5984	11.1162	22.096	16.2143	18.1855	18.5544	22.4856	15.2846
XLOC_043108	GALNTL1	chr10	81396277	81397246	4.45629	4.26478	4.18275	1.27808	2.22943	2.21913	1.53489	1.8715	0.62278
XLOC_043109	GALNTL1	chr10	81397371	81398768	5.25143	5.06217	5.47854	1.30782	1.60037	1.63757	1.91487	2.28408	1.17352
XLOC_043110	GALNTL1	chr10	81398972	81427151	0.0	0.462504	0.0	1.1088	0.907379	0.882925	1.19389	1.54836	0.945803
XLOC_043111	GALNTL1	chr10	81459179	81460127	0.914937	0.0	0.0	1.476	1.78767	1.42359	0.994726	1.55483	1.19863
XLOC_043112	GALNTL1	chr10	81460271	81462492	0.0	0.104875	0.548598	2.64015	3.71481	3.393	1.54028	2.15209	1.56437
XLOC_055447	GALNTL4	chr15	41535580	41861049	20.1603	21.9755	21.0852	28.1332	25.2141	27.6955	29.1239	27.0627	28.5878
XLOC_086296	GALNTL6	chr8	4166564	4208173	7.59089	2.9827	2.67761	1.95298	2.98483	2.18043	1.62147	2.17391	2.04929
XLOC_086297	GALNTL6	chr8	4226923	4236611	1.30805	1.25365	1.43334	0.200375	0.373783	0.29085	0.204832	0.168823	0.0488914
XLOC_086298	GALNTL6	chr8	4238916	4251762	9.34112	11.7228	10.7221	8.61644	10.3997	9.08632	8.63639	8.28059	7.04604
XLOC_086299	GALNTL6	chr8	4252857	4256326	10.1224	11.557	13.1606	15.6281	16.0308	14.7718	15.9463	14.798	12.7953
XLOC_086300	GALNTL6	chr8	4325063	4359538	19.807	12.4313	8.22343	6.78294	7.44461	7.74996	6.22592	4.46622	6.69983
XLOC_086301	GALNTL6	chr8	4493317	4524682	3.31982	3.07295	2.50094	10.8182	13.7493	12.1008	11.4664	8.347	8.09273
XLOC_086768	GALNTL6	chr8	3965754	3969496	0.0	0.0	0.0	2.78173	1.10815	0.737348	0.843542	0.23478	0.415449
XLOC_086769	GALNTL6	chr8	4256529	4275942	36.6241	49.2502	35.1947	83.8883	95.3958	90.735	90.6843	84.5024	86.0743
XLOC_086770	GALNTL6	chr8	4288435	4324959	54.5635	48.3116	39.2712	60.842	59.5202	62.9056	74.3597	73.8769	45.9389
XLOC_086771	GALNTL6	chr8	4735903	4779575	7.60748	8.54126	7.57763	29.7383	31.8162	32.0185	36.0639	31.9565	35.6821
XLOC_087176	GALNTL6	chr8	3994490	3997573	12.1134	11.7629	11.8033	20.3757	17.8907	19.0294	24.2652	23.3965	17.1419
XLOC_087177	GALNTL6	chr8	3998830	3999055	0.0	3.02433	15.8111	13.7428	6.0176	5.07795	10.5804	9.34181	6.95427
XLOC_087178	GALNTL6	chr8	4039915	4040419	29.2253	13.0952	11.4153	8.96939	10.1824	9.67363	12.7729	15.4286	10.8926

XLOC_087179	GALNTL6	chr8	4087497	4088859	10.2076	13.2924	9.86586	8.88231	12.6557	10.1098	13.3515	14.4011	11.498
XLOC_087180	GALNTL6	chr8	4210233	4210773	1.89102	2.82496	0.0	0.338615	0.733203	2.14468	0.0	0.560384	0.658286
XLOC_087181	GALNTL6	chr8	4238916	4251762	4.76659	12.0996	14.89	3.41257	4.05031	3.92014	4.83522	4.68077	4.96925
XLOC_087182	GALNTL6	chr8	4283794	4284194	0.0	0.890164	2.32774	2.13409	1.14655	3.05489	1.30151	1.16322	1.80991
XLOC_087183	GALNTL6	chr8	4284453	4287008	5.73148	7.04096	4.48585	2.97583	5.30722	3.95774	5.22471	3.00796	3.74954
XLOC_087184	GALNTL6	chr8	4287077	4288302	6.77512	7.09331	8.4809	4.13007	7.37427	5.84172	5.24057	3.73584	4.61946
XLOC_087185	GALNTL6	chr8	4325063	4359538	7.83024	12.2816	7.64722	2.10303	3.64128	3.22797	2.26201	1.5457	1.53289
XLOC_087186	GALNTL6	chr8	4325063	4359538	6.03927	5.40767	2.35678	2.70091	1.8569	1.54622	2.6339	2.06021	2.35584
XLOC_087187	GALNTL6	chr8	4325063	4359538	7.22119	3.59415	5.63937	0.430802	2.60256	1.97916	1.91008	0.945191	0.83638
XLOC_087188	GALNTL6	chr8	4325063	4359538	0.0	7.23476	18.9164	1.7366	1.84218	1.47775	1.23366	2.78397	2.09293
XLOC_087189	GALNTL6	chr8	4325063	4359538	4.36141	1.30062	0.0	0.390031	2.65685	1.77436	3.72213	1.25691	0.753174
XLOC_087190	GALNTL6	chr8	4325063	4359538	0.0	2.78351	3.63895	0.417498	0.709501	2.37058	1.98277	0.894099	1.611
XLOC_087191	GALNTL6	chr8	4325063	4359538	1.24322	1.85955	2.43174	0.390096	0.681754	0.710568	0.453008	0.311813	0.489134
XLOC_087192	GALNTL6	chr8	4391722	4392154	13.1748	12.5875	14.4011	24.0465	26.2049	27.5861	24.5268	25.7975	28.3602
XLOC_087193	GALNTL6	chr8	4524793	4525266	4.58527	4.79305	0.0	5.95028	7.08875	8.48869	6.27917	3.83133	5.18012
XLOC_087194	GALNTL6	chr8	4525374	4525620	0.0	2.37399	0.0	2.86395	8.93157	2.40238	5.20561	5.20125	3.41904
XLOC_087195	GALNTL6	chr8	4525767	4526030	0.0	2.01412	0.0	9.09102	5.58661	6.81493	9.50214	8.26523	6.39078
XLOC_087196	GALNTL6	chr8	4528465	4528989	0.0	0.589613	0.0	1.23679	1.98825	1.22028	1.57836	0.389512	0.686822
XLOC_087197	GALNTL6	chr8	4529768	4530262	2.14956	0.0	0.0	0.577212	0.99808	0.885097	0.76161	2.54005	0.747587
XLOC_087198	GALNTL6	chr8	4588099	4589019	0.0	0.0	0.0	0.765077	0.296452	1.47556	0.687077	1.61126	0.745499
XLOC_087199	GALNTL6	chr8	4613145	4613871	0.0	0.0	3.97894	1.02584	0.694038	1.97517	1.83216	1.01299	1.10972
XLOC_087200	GALNTL6	chr8	4615309	4616686	0.0	0.709676	0.928056	1.4356	0.604125	0.801341	1.08107	0.654717	0.622294
XLOC_087201	GALNTL6	chr8	4629467	4629612	0.0	20.2094	0.0	26.106	37.165	52.7436	38.6476	33.6497	45.7639
XLOC_087202	GALNTL6	chr8	4629818	4630019	0.0	8.61207	0.0	11.8419	7.39648	11.4946	12.4175	10.4282	16.039
XLOC_087203	GALNTL6	chr8	4630291	4630812	0.0	2.37788	0.0	2.6721	3.39207	1.6403	1.94445	3.73017	3.28914
XLOC_087204	GALNTL6	chr8	4631111	4632545	1.13316	1.017	0.443319	1.9303	1.643	1.59027	1.49829	2.10457	1.88278
XLOC_087205	GALNTL6	chr8	4633535	4634859	1.24002	1.4838	0.485098	1.00053	1.16573	0.902043	0.39537	0.435424	0.975758
XLOC_087206	GALNTL6	chr8	4637939	4638947	2.55116	0.254335	0.665185	0.381101	0.997448	0.794213	0.308551	0.255225	0.297155
XLOC_087207	GALNTL6	chr8	4641688	4643033	3.04522	5.10152	5.24177	2.07489	2.33815	1.96219	2.49722	1.89446	2.3431
XLOC_087208	GALNTL6	chr8	4652051	4652517	0.0	2.10067	3.66233	23.5007	13.2251	15.6696	14.073	21.6522	18.5384
XLOC_087209	GALNTL6	chr8	4714149	4714288	86.8017	163.42	142.702	337.613	265.762	336.152	410.876	506.285	476.556
XLOC_087210	GALNTL6	chr8	4714511	4714781	0.0	0.0	0.0	0.0	0.0	0.0	3.16893	2.39945	3.82737
XLOC_087211	GALNTL6	chr8	4812868	4813008	0.0	0.0	0.0	58.8705	58.7413	33.5807	42.717	21.2357	65.6342

XLOC_087212	GALNTL6	chr8	5117412	5117793	6.47072	0.0	0.0	0.868057	2.23466	1.32375	1.40582	0.314568	0.560526
XLOC_039465	GAP43	chr1	60900875	60901438	44.5936	33.5788	25.0902	76.3465	104.938	95.5129	90.8184	59.9855	87.8976
XLOC_044391	GAPVD1	chr11	96124754	96165508	0.0	0.564628	0.552344	7.84138	10.9517	8.42823	2.69079	2.64122	3.60068
XLOC_047088	GAPVD1	chr11	96124754	96165508	0.0	0.0	0.0	1.00686	3.13154	2.3146	0.198927	0.442559	0.391094
XLOC_047089	GAPVD1	chr11	96194357	96194895	1.90096	1.70387	1.48531	0.850978	1.474	0.195984	0.676569	0.187754	0.165431
XLOC_044421	GARNL3	chr11	98026660	98029901	54.9271	47.2883	51.8086	126.457	116.258	117.234	132.288	141.597	124.099
XLOC_045055	GARNL3	chr11	98036687	98053372	9.39699	12.8012	11.7594	2.98399	6.03484	3.85333	5.23333	4.47552	4.42663
XLOC_045056	GARNL3	chr11	98088039	98150179	11.773	13.22	16.2368	10.0447	12.152	10.3037	7.80596	8.29463	9.18629
XLOC_047199	GARNL3	chr11	98031001	98031761	0.0	0.0	0.0	1.82847	1.87213	1.36732	0.432762	1.55457	2.61841
XLOC_047200	GARNL3	chr11	98057001	98057625	0.0	0.463399	1.21191	1.80527	0.602802	1.28157	0.416523	0.461372	0.405305
XLOC_047201	GARNL3	chr11	98068046	98068321	60.9532	29.0729	28.5041	9.28462	1.83857	2.464	1.01626	2.30611	4.72149
XLOC_047202	GARNL3	chr11	98071995	98072215	64.8288	103.356	75.9839	29.3955	6.41116	10.8331	12.1086	14.91	9.27823
XLOC_047203	GARNL3	chr11	98074956	98075985	0.0	1.98569	3.24585	1.41331	1.2981	0.430652	0.903688	1.57787	0.797542
XLOC_047204	GARNL3	chr11	98076682	98077196	0.0	2.42449	0.0	0.544893	0.314328	0.836057	0.90056	0.200085	1.05892
XLOC_047205	GARNL3	chr11	98082659	98086046	4.03094	2.88177	4.20686	2.32986	2.6048	2.23965	1.6236	1.69331	3.07846
XLOC_047206	GARNL3	chr11	98086152	98087080	2.81606	3.08792	4.40512	2.35555	1.83373	3.69951	1.87018	1.68853	2.54126
XLOC_047207	GARNL3	chr11	98087331	98087867	3.82201	2.85476	5.97258	4.44843	5.48218	3.74314	2.38007	2.64213	3.99117
XLOC_073207	GARS;CRHR2;GGCT;NOD1;MIR2419;ZNRF2	chr4	66052974	66403992	0.0	0.0	0.0	0.103442	0.195246	0.0	0.104134	0.0	0.0
XLOC_063960	GAS7	chr19	29703854	29708527	0.0	0.0	1.06583	3.51202	2.13795	4.54659	1.71506	4.20181	2.27933
XLOC_064224	GAS7	chr19	29715550	29805985	31.8503	32.5235	32.5097	14.7191	15.725	15.656	17.0064	13.9559	16.5194
XLOC_064225	GAS7	chr19	29809433	29812977	8.73599	5.90484	7.50601	5.32306	4.81357	5.23866	5.59825	5.91976	5.5506
XLOC_064977	GAS7	chr19	29714289	29714753	0.0	1.40956	9.21541	2.32313	1.45861	2.18359	1.2495	0.695408	2.46044
XLOC_064978	GAS7	chr19	29715550	29805985	2.39115	1.42819	9.33696	1.28388	3.69388	1.96626	0.84358	1.40868	3.53131
XLOC_064979	GAS7	chr19	29715550	29805985	1.53078	2.28763	2.39308	2.05659	2.73814	2.21454	3.01682	1.82249	3.20149
XLOC_064980	GAS7	chr19	29715550	29805985	0.0	4.95995	5.18783	2.37841	2.03976	2.37914	2.01931	0.968698	4.89417
XLOC_064981	GAS7	chr19	29806671	29807793	0.749869	1.79435	1.17324	1.1427	0.821575	0.545046	0.954053	0.751117	0.393189
XLOC_064982	GAS7	chr19	29808175	29809107	1.86768	3.63053	5.11279	1.75755	1.45946	2.13082	2.45273	1.67991	3.42527
XLOC_064223	GAS7;RCVRN	chr19	29610130	29683196	10.5134	8.77461	7.89335	25.7397	26.2245	25.837	25.6737	26.8516	21.7076
XLOC_064975	GAS7;RCVRN	chr19	29610130	29683196	0.0	0.0	0.0	0.360971	0.941516	1.38954	0.0	0.0	0.0

XLOC_064976	GAS7;RCVRN	chr19	29610130	29683196	0.0	0.0	0.0	1.09778	0.398579	0.423286	0.0	0.306112	0.267371
XLOC_064008	GAST;EIF1	chr19	42518773	42592282	9.70861	12.6436	9.3771	8.30639	8.97826	9.28187	9.61181	10.0156	9.93963
XLOC_055909	GAT	chr15	83484001	83505368	4.44215	5.48192	2.60685	7.81609	7.96471	8.08178	7.90056	8.80893	7.23541
XLOC_080958	GBA3	chr6	43766961	43767126	31.6798	38.621	0.0	75.7921	43.5092	41.3515	50.402	64.1342	54.9626
XLOC_039053	GBE1	chr1	28800225	28800410	19.3621	11.6708	30.5126	37.7977	16.9711	19.3735	29.954	29.5042	23.3469
XLOC_069909	GBP6	chr3	54755552	54798092	0.372126	0.32518	0.582434	0.533902	0.146033	0.232366	0.238374	0.337012	0.253616
XLOC_071289	GBP6	chr3	54755552	54798092	0.0	0.0	0.0	0.0	0.0	0.0	0.0	0.0	0.108504
XLOC_079819	GC	chr6	88724428	88828424	1.66999	3.8825	4.79947	0.127349	0.0498263	0.1762	0.194059	0.168387	0.182777
XLOC_078663	GCAT	chr5	110129033	110129592	1.80152	5.92128	1.40776	6.45237	6.85021	7.80552	6.74454	5.16627	6.43017
XLOC_078664	GCAT	chr5	110130227	110130381	45.2587	27.8551	0.0	22.1775	9.75888	13.7001	6.78407	3.9415	27.6807
XLOC_078665	GCAT	chr5	110130584	110130981	0.0	0.0	0.0	2.70091	1.8569	3.09244	1.05355	1.47158	1.57056
XLOC_044036	GCC2;LIMS1;SULT1C3;SULT1C4;SLC5A7	chr11	44620412	45005170	5.77146	3.1761	3.6397	1.92136	1.79256	1.88974	5.66651	3.79193	4.30022
XLOC_070277	GCLM	chr3	49743134	49757377	2.62564	1.41492	2.11789	0.161961	0.0434755	0.164626	0.038108	0.0976527	0.0986463
XLOC_041531	GCNT3;BNIP2;GTF2A2	chr10	50592690	50854030	22.9444	27.9583	27.596	22.6212	19.9213	25.7535	20.9635	23.2734	21.2382
XLOC_082731	GDF15;LRRC25	chr7	4692878	4715653	2.05413	2.04887	2.67939	8.25872	7.01584	8.01885	8.87195	11.9594	7.07132
XLOC_084054	GDF15;LRRC25	chr7	4692878	4715653	0.0	2.52577	0.0	1.13608	0.968199	0.430965	1.44842	4.88797	1.09732
XLOC_053698	GDF6	chr14	70374117	70429493	47.8803	64.3177	67.5536	14.9783	19.4266	13.5842	16.9144	16.8571	18.9651
XLOC_044280	GDF7	chr11	78261676	78292626	63.1737	73.2743	73.0073	50.9909	49.2345	50.4019	56.0705	61.8964	47.7002
XLOC_064578	GDPD1	chr19	10449026	10450696	4.30353	5.72258	3.36761	1.15763	1.1251	1.09418	1.13556	1.87423	1.92414
XLOC_064579	GDPD1	chr19	10450928	10451701	5.87549	7.02677	4.59432	1.57932	1.92461	1.21696	1.1655	1.28802	1.2304
XLOC_064580	GDPD1	chr19	10456046	10457165	0.752209	0.674981	0.0	0.0674273	0.117732	0.0	0.0	0.150688	0.0
XLOC_055458	GDPD5;KLHL35;RPS3	chr15	55338512	55463749	1.65139	1.15319	1.5799	0.115202	0.0288507	0.133841	0.134817	0.0555246	0.0803417
XLOC_045622	GEMIN6	chr11	21151499	21152051	0.0	3.287	2.86539	0.656666	0.711224	0.567319	0.980034	0.543715	0.79799
XLOC_044485	GFI1B	chr11	103027856	103069232	13.5707	15.5884	12.1113	61.0411	45.2052	54.9777	50.0787	61.5215	47.3483
XLOC_046428	GFPT1	chr11	67686057	67686989	21.4783	24.8552	21.912	0.585849	0.729732	0.290567	0.592038	0.373313	0.815541
XLOC_046429	GFPT1	chr11	67687138	67689058	13.5413	12.2779	11.5605	0.18398	0.225408	0.298891	0.337748	0.454024	0.323142
XLOC_086893	GFRA2	chr8	69554804	69585157	0.921284	0.70667	1.0899	2.0886	3.52658	3.23598	1.60368	1.00105	1.36028
XLOC_087935	GFRA2	chr8	69553882	69554019	0.0	0.0	0.0	39.8495	13.7456	29.5999	28.5517	0.0	0.0

XLOC_087936	GFRA2	chr8	69554804	69585157	3.97772	2.77458	3.10988	6.77056	9.70875	8.77985	10.0146	5.01132	8.20963
XLOC_087937	GFRA2	chr8	69554804	69585157	0.0	0.0	0.0	1.10397	2.82483	0.838066	0.0	0.0	0.355559
XLOC_087938	GFRA2	chr8	69554804	69585157	0.0	0.0	0.0	0.304023	0.970633	1.28876	0.0	0.338277	0.0987009
XLOC_065374	GGA3	chr19	56869245	56870012	29.6667	32.9958	25.0533	0.106323	0.832875	0.122885	0.21395	0.472916	0.207077
XLOC_064056	GGA3;MIF4GD;SLC25A19;MRPS7	chr19	56826227	56868926	24.9714	37.1058	27.9061	0.418837	0.70058	0.474611	0.400047	0.339534	0.504956
XLOC_064057	GGA3;NUP85	chr19	56874359	56912328	3.4916	2.26048	2.96929	1.18386	0.524956	0.911741	0.237589	1.06066	0.423033
XLOC_050556	GINS1	chr13	54071872	54089020	3.60431	1.07462	5.62476	118.569	90.9432	105.179	94.0347	116.109	103.51
XLOC_061820	GIT2	chr17	65576095	65576348	0.0	6.63635	0.0	0.666471	1.11226	2.24154	2.43834	0.694942	1.91272
XLOC_061821	GIT2	chr17	65577160	65577567	0.0	4.32641	6.78807	1.0372	1.33815	2.0795	1.52025	0.565859	1.25692
XLOC_061822	GIT2	chr17	65577894	65579780	4.60256	1.50232	3.27439	1.38822	0.886474	1.82853	1.49214	0.757487	1.28134
XLOC_038026	GK5	chr1	127774168	127808457	11.4876	10.697	12.1003	27.1011	28.4437	27.9393	28.7225	27.3949	33.553
XLOC_038027	GK5	chr1	127774168	127808457	0.624303	0.0	0.0	0.0	0.0	0.0	0.0	0.0	0.0
XLOC_038520	GK5	chr1	127774168	127808457	3.79179	3.54311	3.32855	3.76891	3.11229	3.76027	4.09333	3.33865	3.74309
XLOC_040198	GK5	chr1	127771191	127771776	0.0	0.0	2.64457	1.36363	0.131412	0.17466	0.453354	0.502602	0.294705
XLOC_086498	GKAP1	chr8	78390994	78394322	0.0	1.85349	1.07726	2.6539	2.96484	2.50307	2.81875	3.3817	3.55062
XLOC_045066	GLE1;ODF2	chr11	99067642	99090657	0.197952	0.0592417	0.0	0.248563	0.357896	0.330008	0.0545269	0.159709	0.208009
XLOC_066341	GLI2	chr2	72936437	72981722	84.3321	77.1888	79.9707	85.4881	91.9271	89.7056	92.5403	95.6749	96.205
XLOC_066342	GLI2	chr2	73092320	73214439	23.867	34.2933	18.1167	84.3123	72.3946	79.8495	91.1115	101.15	81.0136
XLOC_067870	GLI2	chr2	72936437	72981722	4.53597	3.83267	5.63869	2.84772	3.39597	3.11252	3.08292	2.31222	2.40597
XLOC_067871	GLI2	chr2	72936437	72981722	4.04662	6.03449	15.779	14.4751	8.64584	10.3062	5.55248	8.18978	10.8406
XLOC_067872	GLI2	chr2	72982227	72982900	1.40308	0.419407	0.0	0.75411	0.436914	1.1608	0.503927	0.557684	0.733944
XLOC_067873	GLI2	chr2	72983580	72983916	0.0	1.2069	0.0	1.44741	0.30878	0.824367	0.0	0.0	0.349697
XLOC_067874	GLI2	chr2	72984727	72985986	1.3132	0.98207	1.54112	0.470902	0.514234	0.613966	0.239095	0.329223	0.229601
XLOC_067875	GLI2	chr2	72986257	72987065	0.0	0.997361	1.73896	0.298888	0.347042	0.230385	0.30111	0.0	0.194077
XLOC_067876	GLI2	chr2	73089240	73089981	2.4791	0.370585	0.0	0.111055	0.579747	0.256638	0.223267	0.123418	0.64879
XLOC_067877	GLI2	chr2	73091356	73092207	0.0	0.31186	0.0	0.654206	0.488554	0.648575	0.565596	1.0409	0.546265
XLOC_076733	GLIPR1;GLIPR1L1;KRR1	chr5	4803248	5380308	18.6773	16.5505	13.5532	0.346523	0.303974	0.876563	0.628477	0.315812	0.664433
XLOC_077245	GLIPR1;GLIPR1L1;KRR1	chr5	4803248	5380308	8.00425	1.19366	0.0	0.0	0.0	0.0	0.0	0.0	0.0
XLOC_070278	GLMN;C3H1orf146;RPAP2	chr3	51253528	51340664	43.4186	46.4031	45.1622	24.1212	28.2324	25.7125	30.2352	28.716	32.6305

XLOC_071015	GLMN;C3H1orf146;RPAP2	chr3	51253528	51340664	17.1412	14.5239	22.3443	10.0707	11.607	8.98691	11.3808	8.94411	12.3907
XLOC_092273	GLRA2	chrX	136138670	136140689	28.4265	8.253	10.8006	52.7789	53.6793	61.5397	20.7128	21.7809	11.8472
XLOC_092274	GLRA2	chrX	136221131	136226088	0.0	0.237587	0.0	0.712012	0.434996	0.577213	0.360645	0.636186	0.694071
XLOC_092587	GLRA2	chrX	136066562	136068189	32.0532	37.7208	16.1727	30.2989	31.4858	27.651	38.6977	26.7311	23.8302
XLOC_092588	GLRA2	chrX	136073335	136075889	0.0	3.32932	2.48771	2.70802	0.123703	0.65754	0.569602	0.315546	0.415945
XLOC_092589	GLRA2	chrX	136076080	136085151	2.4431	4.31343	4.19392	2.51652	0.353554	0.577115	1.26929	0.827393	0.504131
XLOC_092590	GLRA2	chrX	136170673	136172266	42.1071	37.3352	66.2335	117.312	108.053	124.974	145.038	143.102	146.692
XLOC_092591	GLRA2	chrX	136201790	136203851	0.0	7.16659	0.0	0.0	0.0	0.0	0.0	0.0	0.0
XLOC_092592	GLRA2	chrX	136213969	136215717	21.0152	30.6845	25.5623	121.051	139.678	139.16	155.381	169.265	111.037
XLOC_093616	GLRA2	chrX	136033027	136034947	1.23103	0.245558	0.963375	2.46533	1.86766	2.17764	1.65121	1.651	0.718093
XLOC_093617	GLRA2	chrX	136072131	136072557	5.38762	3.21699	12.6188	2.41003	0.415163	0.829188	0.236307	0.527108	0.701316
XLOC_093618	GLRA2	chrX	136073045	136073271	0.0	20.9025	15.6111	10.8523	2.22908	3.00894	4.02179	3.69208	3.43354
XLOC_093619	GLRA2	chrX	136169766	136170475	3.93444	6.66506	10.2536	17.3887	17.2672	16.0131	19.6985	24.2665	21.6172
XLOC_093620	GLRA2	chrX	136172414	136173165	0.0	0.0	0.0	0.327559	0.0950123	0.0	0.439178	0.0	0.106318
XLOC_093621	GLRA2	chrX	136201010	136201691	0.0	0.0	0.0	0.0	0.0	0.285803	0.0	0.137326	0.0
XLOC_093622	GLRA2	chrX	136212060	136212776	0.0	0.0	0.0	0.0	0.20181	0.134017	0.233024	0.128856	0.0
XLOC_059795	GLRB	chr17	43016431	43089765	1.68822	1.74029	2.20246	6.44776	6.0432	6.15254	6.78549	7.62655	4.52985
XLOC_083120	GLRX	chr7	97530339	97579502	23.8809	36.6686	36.7143	57.8661	63.2203	63.1093	55.6131	79.7794	71.0378
XLOC_083761	GLRX	chr7	97530339	97579502	7.51445	7.54824	5.87538	7.56806	11.5372	10.8252	8.21509	8.54496	13.3253
XLOC_055910	GLYATL2	chr15	83506286	83510888	0.0	4.70125	24.5772	12.9615	14.9441	17.2298	8.57339	2.83184	14.8055
XLOC_083040	GM2A;SLC36A2;FAT2;SLC36A1	chr7	64501645	64753300	2.87891	6.78787	7.81104	0.177243	0.105723	0.202862	0.254098	0.170148	0.171494
XLOC_046430	GMCL1	chr11	68205539	68206164	0.0	0.924818	0.0	0.0	0.360917	0.639428	0.554209	0.0	0.134814
XLOC_050560	GMEB2	chr13	54574579	54582222	4.83235	5.42447	3.78419	8.09789	7.77767	7.92392	6.59619	6.44498	7.75473
XLOC_051868	GMEB2	chr13	54582331	54583527	8.35719	4.16644	5.44848	3.55858	3.81679	2.82123	3.48443	3.21103	3.89591
XLOC_041574	GMFB;CNIH;CDKN3;CGRRF1	chr10	67012957	67256774	59.4115	64.7808	57.8195	56.92	46.2006	58.1713	57.2049	69.417	51.0004
XLOC_041976	GMFB;CNIH;CDKN3;CGRRF1	chr10	67012957	67256774	29.084	25.3911	20.2862	9.37119	8.41066	7.21958	6.73045	9.41912	7.31377
XLOC_042859	GMFB;CNIH;CDKN3;CGRRF1	chr10	67012957	67256774	4.09497	1.53064	2.40217	0.27522	0.799478	1.5918	0.370261	0.919846	0.357669

XLOC_042860	GMFB;CNIH;CDKN3;CGRRF1	chr10	67012957	67256774	3.77174	1.91858	4.42775	2.57052	2.22048	2.90489	2.58865	2.6568	1.98044
XLOC_042861	GMFB;CNIH;CDKN3;CGRRF1	chr10	67012957	67256774	3.41592	5.61416	8.00898	16.3649	10.8763	17.9818	12.3526	11.836	11.6017
XLOC_042862	GMFB;CNIH;CDKN3;CGRRF1	chr10	67012957	67256774	7.89489	4.98464	8.23396	13.6802	11.8642	17.3836	11.3729	12.1067	9.88463
XLOC_042863	GMFB;CNIH;CDKN3;CGRRF1	chr10	67012957	67256774	10.598	8.44479	17.9445	21.5102	16.453	18.9545	15.9123	16.6005	15.533
XLOC_042864	GMFB;CNIH;CDKN3;CGRRF1	chr10	67012957	67256774	8.25766	6.03939	11.847	15.7137	13.5309	15.2711	15.5933	11.806	10.1138
XLOC_042865	GMFB;CNIH;CDKN3;CGRRF1	chr10	67012957	67256774	8.48363	10.1257	6.61988	19.1067	12.6717	10.2345	9.66673	15.0028	8.7463
XLOC_042866	GMFB;CNIH;CDKN3;CGRRF1	chr10	67012957	67256774	8.33726	3.49097	9.78263	10.5366	9.25877	7.44119	8.39707	7.75856	6.55548
XLOC_042867	GMFB;CNIH;CDKN3;CGRRF1	chr10	67012957	67256774	7.12566	6.157	8.67106	8.58708	8.42384	10.7668	8.268	9.0362	5.94959
XLOC_042868	GMFB;CNIH;CDKN3;CGRRF1	chr10	67012957	67256774	7.37929	4.08879	9.34857	10.3389	7.57104	10.8992	7.41364	8.84834	8.21756
XLOC_042869	GMFB;CNIH;CDKN3;CGRRF1	chr10	67012957	67256774	2.334	2.79293	5.93524	5.33592	4.75543	5.94376	4.46804	4.80246	3.52067
XLOC_042870	GMFB;CNIH;CDKN3;CGRRF1	chr10	67012957	67256774	7.47004	7.79911	8.74101	4.00776	5.14121	8.00154	6.75987	6.8619	3.87714
XLOC_042871	GMFB;CNIH;CDKN3;CGRRF1	chr10	67012957	67256774	14.2355	10.4776	11.9889	7.35918	6.32401	7.94222	6.42727	8.62858	6.97716
XLOC_042872	GMFB;CNIH;CDKN3;CGRRF1	chr10	67012957	67256774	5.05225	6.54237	3.94887	2.8654	4.31679	3.65097	2.70785	4.50288	5.28041
XLOC_042873	GMFB;CNIH;CDKN3;CGRRF1	chr10	67012957	67256774	1.37122	3.68891	9.64791	8.22958	6.61989	5.95675	3.57245	7.633	6.33664
XLOC_042874	GMFB;CNIH;CDKN3;CGRRF1	chr10	67012957	67256774	8.82851	7.89745	10.3262	6.31606	8.40055	6.2839	6.39899	9.74789	8.76582

XLOC_082721	GMIP	chr7	3617292	3620176	201.327	191.172	153.973	279.254	238.114	226.612	221.998	269.899	340.802
XLOC_082722	GMIP;LPAR2;PBX4	chr7	3620291	3642140	8.82951	13.1228	11.7289	59.7371	33.07	46.3737	48.168	77.7164	43.8382
XLOC_083358	GMIP;LPAR2;PBX4	chr7	3620291	3642140	13.9635	14.837	19.7747	19.1243	14.0074	14.3779	19.0621	22.9568	17.9128
XLOC_084037	GMIP;LPAR2;PBX4	chr7	3620291	3642140	0.787426	1.88416	4.31192	1.05886	0.80086	0.735699	1.00114	0.867229	0.550452
XLOC_072585	GNAI1	chr4	41135921	41191237	58.0427	74.9588	69.6216	50.0091	46.3116	49.2185	55.2202	56.4151	43.9254
XLOC_073123	GNAI1	chr4	41191380	41275318	60.1979	57.163	51.7439	64.5099	62.4793	74.0107	75.587	87.0786	38.282
XLOC_073992	GNAI1	chr4	41191380	41275318	0.0	7.77622	0.0	7.01693	6.87051	9.87334	13.5234	6.76243	4.48757
XLOC_070794	GNAI3	chr3	34004814	34005525	15.6809	14.8446	14.3032	27.0425	29.6259	23.1223	21.2746	27.0402	26.098
XLOC_062165	GNAO1	chr18	24470689	24477882	7.27344	7.85408	4.93603	16.3378	16.9895	14.9763	19.2959	20.2348	15.1493
XLOC_062377	GNAO1	chr18	24485987	24530127	20.4221	15.0546	21.8435	24.7345	20.8484	22.4992	17.7432	23.1949	27.8784
XLOC_086403	GNAQ	chr8	54077352	54186696	1.54296	1.00094	2.74489	5.75676	4.28713	5.21053	6.59994	4.94077	4.61876
XLOC_087789	GNAQ	chr8	54077352	54186696	1.13991	0.340829	0.891379	0.919249	1.33393	1.41693	2.7769	1.47726	1.39266
XLOC_087790	GNAQ	chr8	54077352	54186696	0.533557	0.47888	0.0	0.765416	0.54377	1.22051	0.778945	0.643044	1.02662
XLOC_087791	GNAQ	chr8	54077352	54186696	0.0	0.143634	0.375668	0.774824	0.978953	0.948733	1.27159	0.57897	1.04989
XLOC_087792	GNAQ	chr8	54077352	54186696	0.0	0.0	0.0	1.36551	0.669631	0.790066	0.344872	0.66605	0.831593
XLOC_087793	GNAQ	chr8	54077352	54186696	0.0	0.339226	0.0	0.406635	0.265541	1.41031	0.307123	0.113108	0.396037
XLOC_087794	GNAQ	chr8	54077352	54186696	0.0	0.0	0.846091	0.290848	0.591111	0.11212	0.488606	0.107932	1.03879
XLOC_087795	GNAQ	chr8	54077352	54186696	0.575598	0.0	0.0	0.361239	0.676635	0.299169	0.47231	0.635573	0.805343
XLOC_050879	GNAS	chr13	58004474	58013988	0.0	0.221962	0.0	0.892993	1.2254	0.924788	0.582358	1.33826	0.995006
XLOC_073117	GNAT3	chr4	40804508	40854044	46.6717	35.5153	39.8073	14.2728	12.6249	12.3388	10.042	11.5104	10.4181
XLOC_073987	GNAT3	chr4	40800686	40801423	2.49621	0.37314	0.0	0.111821	0.19457	0.129198	0.0	0.124257	0.108875
XLOC_037969	GNB4;MFN1	chr1	88258692	88277636	10.4269	11.8223	14.0437	13.5269	13.8368	11.7641	10.5614	9.56469	12.6311
XLOC_038461	GNB4;MFN1	chr1	88258692	88277636	68.2622	88.56	87.8314	124.485	125.782	121.23	109.725	105.256	115.368
XLOC_039860	GNB4;MFN1	chr1	88258692	88277636	68.5378	88.4454	76.4604	129.67	142.682	124.753	113.87	112.164	141.593
XLOC_039861	GNB4;MFN1	chr1	88258692	88277636	8.02172	11.1765	6.26289	15.5497	11.1292	17.0119	10.794	12.5608	6.96216
XLOC_039862	GNB4;MFN1	chr1	88258692	88277636	7.10449	8.47839	8.31364	20.9652	8.97676	13.7838	14.7399	11.0142	6.45607
XLOC_039863	GNB4;MFN1	chr1	88258692	88277636	0.0	0.983925	0.0	7.54789	1.63409	4.67831	7.04327	2.67517	6.65161
XLOC_041544	GNB5	chr10	58255371	58305735	10.1717	13.1129	12.3265	7.95122	9.78891	9.4736	11.4724	11.0097	11.309
XLOC_041545	GNB5	chr10	58323425	58421889	32.0992	30.3515	30.1512	30.1669	27.6115	31.1463	21.9843	22.9346	29.8779
XLOC_086413	GNE	chr8	60890401	60907726	30.0514	30.8439	26.4996	50.9296	72.4009	62.507	67.9155	53.3851	54.8582
XLOC_084332	GNG7	chr7	22220260	22220462	0.0	8.46796	0.0	6.46572	5.19828	2.8264	3.33398	5.13187	9.70642

XLOC_073077	GNGT1;GNG11	chr4	11047494	11076366	0.744351	2.36866	4.65884	17.112	18.8448	18.2128	23.6455	24.7443	21.31
XLOC_073718	GNGT1;GNG11	chr4	11047494	11076366	0.0	0.0	0.0	0.784099	0.389843	0.6471	0.225184	0.248963	0.327189
XLOC_073719	GNGT1;GNG11	chr4	11047494	11076366	0.0	2.46853	3.22738	2.2205	0.631327	0.842742	0.708917	4.78176	1.43024
XLOC_072090	GNL2	chr3	108898518	108899027	2.05782	1.84418	1.60761	0.368419	1.59363	1.2717	1.82579	0.608584	1.78979
XLOC_072091	GNL2	chr3	108900286	108900536	22.9213	20.5098	0.0	6.1819	9.15769	5.38509	5.63913	4.28882	3.93987
XLOC_072092	GNL2	chr3	108901170	108904394	32.3108	28.3024	31.3822	22.6542	22.9263	25.5998	33.4981	32.9508	26.1835
XLOC_044743	GNLY	chr11	49058229	49064206	0.0	0.401095	0.0	0.0	0.0350558	0.0	0.0	0.0	0.039094
XLOC_076352	GNPTAB	chr5	65966612	65977490	0.0	0.0	0.632562	7.96969	5.38792	6.1759	3.55163	5.41811	4.7076
XLOC_044435	GOLGA2	chr11	98890431	98901192	12.7957	14.1384	14.9292	4.94136	4.917	5.42169	3.95738	6.81172	3.9522
XLOC_070711	GOLPH3L	chr3	20121361	20121915	0.0	0.0	0.0	0.0	0.141538	0.0	0.0	0.0	0.0
XLOC_057908	GORAB;PRRX1	chr16	38902350	39027620	18.7064	15.5121	16.3203	0.0873856	0.0765896	0.0406071	0.0894632	0.0589572	0.15357
XLOC_065525	GORASP2	chr2	25557752	25570319	3.54353	4.64249	3.80625	6.76084	5.41231	6.22266	6.07488	7.65707	6.00769
XLOC_066184	GORASP2	chr2	25557752	25570319	0.0	0.369153	0.0	0.118373	0.275438	0.249059	0.0675883	0.172287	0.059053
XLOC_066185	GORASP2	chr2	25572891	25575090	5.49993	6.76407	4.30321	13.8611	15.7997	16.2585	12.4165	15.5131	14.6403
XLOC_066186	GORASP2	chr2	25582638	25608670	17.2805	20.0223	15.3018	32.7564	46.0322	37.2235	36.1354	34.5075	41.2312
XLOC_067103	GORASP2	chr2	25570921	25571811	0.987389	2.06689	1.54448	0.265461	0.231373	0.819003	0.178676	0.0986151	0.344855
XLOC_057835	GPATCH2	chr16	21794092	21898625	2.54828	2.05673	3.70577	5.59201	4.21945	4.54177	3.73285	6.70512	5.00426
XLOC_058070	GPATCH2	chr16	21761623	21787893	0.643855	0.630297	0.471008	3.31897	2.24759	3.306	1.88863	2.12161	1.62609
XLOC_058627	GPATCH2	chr16	21794092	21898625	0.0	0.432026	0.0	1.22999	1.30015	1.2	1.18193	0.723612	1.57792
XLOC_058628	GPATCH2	chr16	21794092	21898625	0.0	2.37458	0.8872	3.25308	2.65541	2.23298	1.53561	1.58351	3.26731
XLOC_058629	GPATCH2	chr16	21794092	21898625	27.5426	36.8949	40.4924	80.7527	60.698	75.1086	67.541	86.8039	66.0586
XLOC_066526	GPATCH3;GPN2;SFN	chr2	126893663	126933534	21.2324	12.1455	15.4516	5.69152	7.68925	6.01857	7.74163	5.70906	7.6329
XLOC_070447	GPBP1L1;NASP;AKR1A1	chr3	101040106	101110723	6.93517	8.2125	8.6471	27.4658	24.9562	26.0965	36.5771	33.4031	28.2571
XLOC_092386	GPC3	chrX	17556143	17572310	3.43982	4.56227	3.23054	7.77747	7.48323	8.27482	7.72153	9.04141	8.97491
XLOC_092799	GPC3	chrX	17495024	17495709	0.0	0.819756	0.0	1.2283	0.640624	1.13462	0.739125	0.817813	1.19555
XLOC_092800	GPC3	chrX	17505265	17505732	0.0	0.697966	1.82526	0.627452	0.361182	0.240303	0.618948	0.688857	0.406131
XLOC_092801	GPC3	chrX	17505965	17506509	0.0	0.0	0.0	0.837792	0.870873	0.385952	0.333211	1.47924	0.977277
XLOC_092802	GPC3	chrX	17509409	17509718	4.75881	2.8376	3.70974	2.12831	0.0	0.966351	1.21123	0.0	0.410517
XLOC_092803	GPC3	chrX	17509789	17510049	0.0	0.0	0.0	3.11531	1.56446	3.50004	1.14651	1.95746	0.0

XLOC_092804	GPC3	chrX	17510792	17511606	0.0	0.988255	0.86154	0.789757	0.515847	0.114147	0.497356	0.109878	0.38462
XLOC_092805	GPC3	chrX	17512106	17512396	0.0	8.08767	0.0	1.94276	1.64233	1.64862	3.19304	1.54824	3.27237
XLOC_092806	GPC3	chrX	17512540	17512805	0.0	1.9785	0.0	2.38089	1.99669	2.00899	0.549797	0.0	2.28318
XLOC_092807	GPC3	chrX	17513339	17513678	0.0	0.0	3.10417	0.355914	1.2154	1.62221	0.68339	1.15158	0.344023
XLOC_092808	GPC3	chrX	17555608	17555846	0.0	2.5883	0.0	1.56348	2.58918	4.35837	3.52401	3.2255	1.48995
XLOC_092385	GPC4;GPC3	chrX	17131855	17319345	5.78506	7.56844	7.7997	23.7971	17.7801	16.5189	43.2102	8.18626	33.9334
XLOC_092790	GPC4;GPC3	chrX	17131855	17319345	7.31333	10.2591	12.1642	3.17712	1.88589	1.57134	4.17348	0.760316	3.54185
XLOC_092791	GPC4;GPC3	chrX	17131855	17319345	5.89728	6.04721	6.58992	0.981692	0.790578	1.92341	2.52219	0.842904	2.28155
XLOC_092792	GPC4;GPC3	chrX	17131855	17319345	4.13882	12.3427	6.45471	3.7011	1.26244	2.10683	4.25289	0.0	1.43023
XLOC_092793	GPC4;GPC3	chrX	17131855	17319345	9.63871	10.915	10.2339	1.66645	0.970315	1.07274	1.87916	0.690144	2.28684
XLOC_092794	GPC4;GPC3	chrX	17131855	17319345	10.4523	12.474	21.7452	4.05053	2.13519	1.77947	5.12574	0.0	1.80942
XLOC_092795	GPC4;GPC3	chrX	17131855	17319345	3.16204	2.83502	3.70713	0.849672	0.737424	0.65327	2.40539	0.0	0.275489
XLOC_092796	GPC4;GPC3	chrX	17131855	17319345	1.6164	0.966095	2.52656	0.289615	0.628078	0.500793	1.59019	0.160193	1.26724
XLOC_092797	GPC4;GPC3	chrX	17131855	17319345	0.0	1.79508	0.0	0.269171	0.462359	0.307982	2.88586	0.0	0.521367
XLOC_092798	GPC4;GPC3	chrX	17131855	17319345	1.12929	0.675281	0.0	0.50599	0.264315	0.233957	1.22286	0.0	0.985474
XLOC_044215	GPN1	chr11	72042474	72047845	2.39678	2.74908	2.50118	11.8205	17.045	14.3743	19.9756	11.8083	15.6842
XLOC_046524	GPN1	chr11	72048532	72049223	0.0	1.21582	0.0	0.36435	0.0	0.140245	0.243673	0.0	0.118217
XLOC_059860	GPN3;ARPC3	chr17	56562012	56607199	42.7766	40.5158	33.7013	28.6933	29.4783	27.6853	36.194	40.0234	24.7154
XLOC_060258	GPN3;ARPC3	chr17	56562012	56607199	32.7697	31.9412	42.8881	32.4825	28.3044	24.3772	39.1921	43.6587	24.2893
XLOC_061658	GPN3;ARPC3	chr17	56562012	56607199	10.8561	4.86167	8.47567	6.07017	3.76391	3.61967	3.09392	4.51309	3.53264
XLOC_061659	GPN3;ARPC3	chr17	56562012	56607199	0.0	2.04002	13.3356	4.27976	5.23981	1.74655	1.77693	1.32691	3.55136
XLOC_061660	GPN3;ARPC3	chr17	56562012	56607199	5.43402	3.57468	1.6999	3.70067	3.30805	3.71624	1.47229	2.27671	2.37149
XLOC_061661	GPN3;ARPC3	chr17	56562012	56607199	1.56727	1.8737	3.67518	1.26332	2.07146	0.971493	2.38514	1.55434	1.36557
XLOC_061662	GPN3;ARPC3	chr17	56562012	56607199	3.35924	2.41169	4.73071	0.963662	1.63095	1.8147	2.01778	1.61671	1.35079
XLOC_061663	GPN3;ARPC3	chr17	56562012	56607199	4.33549	3.87864	13.5223	2.71396	2.9717	2.64604	4.44172	4.16612	2.24624
XLOC_073906	GPNMB	chr4	32022767	32023414	4.43283	7.50813	8.08531	11.9116	13.4506	13.8991	13.9118	13.4958	13.5226
XLOC_044442	GPR107	chr11	100441167	100459048	15.2219	21.5363	21.3021	7.32292	7.5766	8.04914	8.8469	9.04467	9.69523
XLOC_045078	GPR107	chr11	100429533	100441092	25.7663	32.9896	22.5133	22.9828	22.2704	22.5551	23.0348	30.7818	24.2819
XLOC_045079	GPR107	chr11	100461695	100472683	42.5217	49.2925	48.0953	16.9729	16.7814	18.0102	23.6458	21.3616	20.2107

XLOC_045080	GPR107	chr11	100476270	100494198	96.0102	105.406	105.446	153.29	133.77	139.327	141.902	170.931	138.318
XLOC_047267	GPR107	chr11	100459166	100459709	61.9179	53.2572	46.9146	14.1113	13.0965	16.445	12.1927	15.7568	19.2693
XLOC_047268	GPR107	chr11	100459891	100460779	59.4055	71.0587	58.8506	17.8345	15.5441	20.7361	22.305	21.5562	20.4884
XLOC_047269	GPR107	chr11	100460911	100461595	26.1014	39.8336	33.2934	8.61438	8.23678	9.6624	10.3669	10.788	9.94181
XLOC_039241	GPR128	chr1	45441383	45442317	0.931435	0.278553	0.0	0.166955	0.0727866	0.0966075	0.0	0.0930906	0.0813448
XLOC_038339	GPR128;TFG	chr1	45442517	45503330	11.309	2.45945	0.80404	4.14589	0.802776	2.55765	2.32378	1.33418	2.60289
XLOC_038338	GPR128;TMEM45A	chr1	45351582	45437629	12.2908	3.70027	5.77256	4.14799	0.834297	3.61439	5.34999	3.56288	4.04912
XLOC_059822	GPR133	chr17	47141352	47313981	0.0	3.2295	0.0	7.34179	6.65495	6.5734	5.07093	7.84107	3.71159
XLOC_061515	GPR133	chr17	47141352	47313981	0.0	0.300184	0.0	0.629714	0.627189	0.93663	0.181597	0.100237	0.175289
XLOC_076941	GPR162;CD4;GNB3	chr5	103967092	104023802	9.43213	3.99087	5.86703	18.7731	22.7366	22.1158	21.151	24.1996	21.0365
XLOC_078474	GPR162;CD4;GNB3	chr5	103967092	104023802	2.53559	1.51608	4.95626	12.4941	16.7967	17.5835	15.1777	17.54	16.4771
XLOC_092528	GPR173	chrX	95937375	95940416	13.2721	35.6129	15.8037	71.6701	77.4545	81.314	88.1358	78.3051	83.5104
XLOC_092529	GPR173	chrX	95940693	95956948	8.41783	12.5928	10.6127	27.8015	30.5193	33.1761	38.6648	40.9899	32.3607
XLOC_093298	GPR173	chrX	95934374	95935775	1.16323	1.04398	0.910154	4.43231	5.51499	5.80379	6.30958	5.83791	4.32302
XLOC_093299	GPR173	chrX	95937012	95937305	0.0	1.58263	0.0	0.950263	1.60789	0.537897	0.893986	0.505453	0.457479
XLOC_066322	GPR39	chr2	65526491	65536719	0.0	0.0	0.0	0.526185	0.45708	0.151815	2.23903	3.20796	0.38402
XLOC_058164	GPR52	chr16	57168865	57231513	13.9824	20.6185	18.8722	12.3238	11.5803	11.7206	10.5728	11.3046	14.0565
XLOC_059206	GPR52	chr16	57168865	57231513	0.0	3.08201	3.58244	1.43681	1.34021	2.49133	1.23992	1.14168	1.79907
XLOC_059207	GPR52	chr16	57168865	57231513	0.0	15.7934	5.89835	7.47994	1.70054	8.37977	6.20807	3.54032	4.55147
XLOC_089346	GPR6	chr9	40685974	40785155	3.83281	3.0934	2.45958	7.97668	5.501	6.08477	7.28972	11.7867	5.71842
XLOC_089839	GPR6	chr9	40685974	40785155	0.0	0.264923	0.0	0.82945	0.201391	0.212411	0.25187	0.0	0.294785
XLOC_083038	GPX3;TNIP1;ANXA6	chr7	64287499	64372458	7.40953	4.70229	8.66219	5.31274	4.2473	4.56759	5.6604	4.33895	3.49547
XLOC_083675	GPX3;TNIP1;ANXA6	chr7	64287499	64372458	2.01205	2.51	2.107	2.90443	2.51302	2.34768	3.49663	3.3177	3.32791
XLOC_084985	GPX3;TNIP1;ANXA6	chr7	64287499	64372458	1.14174	0.341401	0.0	1.1253	0.445338	0.59131	0.721049	0.569061	0.996319
XLOC_070020	GPX7	chr3	94310114	94332205	0.0	2.251	0.905689	1.64354	1.17363	1.74474	1.42468	2.12775	1.35257
XLOC_037863	GRAMD1C	chr1	58973605	58995332	6.35302	6.02237	4.44994	1.88843	2.5234	2.32151	1.98287	2.15449	3.24243
XLOC_038375	GRAMD1C;ATP6V1A	chr1	58900269	58973495	16.2618	15.325	12.8323	9.11854	7.2769	7.50104	10.2734	12.2162	8.28339
XLOC_082881	GRAMD3	chr7	28692655	28698618	1.81837	0.0	0.481373	6.40682	4.91663	4.31787	5.276	3.06369	5.01398
XLOC_082883	GRAMD3	chr7	28732795	28737732	4.58527	3.38101	0.0	6.80927	4.51084	5.68417	5.90516	3.6875	4.82226

XLOC_084498	GRAMD3	chr7	28698981	28699374	0.0	0.0	0.0	3.29595	6.84277	4.40156	3.74665	2.09392	5.05691
XLOC_084499	GRAMD3	chr7	28732345	28732737	0.0	0.920404	0.0	3.03412	0.947755	1.57856	0.537393	1.20144	0.801824
XLOC_082882	GRAMD3;MIR2458	chr7	28716796	28721286	2.43953	1.45699	1.90508	1.5281	2.82536	4.01092	1.50477	1.43635	1.05946
XLOC_076512	GRAMD4	chr5	117976776	118010126	27.5129	33.6943	36.6435	23.2728	21.8702	24.638	25.0564	30.1941	23.2726
XLOC_065027	GRAP	chr19	34807112	34808321	0.687822	0.411496	0.538117	0.24664	0.161565	0.142897	0.12516	0.413696	0.84172
XLOC_065028	GRAP	chr19	34809005	34809746	1.23955	0.0	1.93839	0.555277	0.193249	0.769913	0.223267	0.123418	0.432526
XLOC_064238	GRAP;FAM83G;PRPSAP2;SLC5A10	chr19	34810346	34879801	4.45927	6.31108	5.57111	4.98443	3.98213	4.47988	4.13973	5.09134	3.83452
XLOC_065029	GRAP;FAM83G;PRPSAP2;SLC5A10	chr19	34810346	34879801	0.0	3.94871	3.44165	2.76347	3.36008	1.34669	0.75282	5.08573	1.52436
XLOC_065030	GRAP;FAM83G;PRPSAP2;SLC5A10	chr19	34810346	34879801	0.0	0.0	8.69541	1.32925	0.852551	1.13747	3.20351	2.15622	0.964229
XLOC_065588	GRB14	chr2	31688234	31804227	19.1971	22.1443	22.5788	23.0782	22.2636	22.4931	20.255	29.4526	23.3161
XLOC_065589	GRB14	chr2	31887258	31947377	7.62824	5.70782	7.84837	0.0391	0.0342901	0.0340827	0.0	0.0	0.0190915
XLOC_064322	GRB2	chr19	56790509	56822224	34.1552	38.1468	37.7133	11.4513	12.1854	13.2967	12.0648	12.2515	11.7307
XLOC_065372	GRB2	chr19	56787056	56789518	6.59045	7.13721	8.10553	2.95517	3.64771	3.75772	3.62288	2.71801	3.48864
XLOC_065373	GRB2	chr19	56789636	56790408	4.70804	15.484	20.2479	5.27297	4.95697	6.46029	6.36739	4.10483	4.92958
XLOC_044972	GREB1	chr11	86192774	86201217	3.61203	13.491	12.7014	14.554	13.0334	12.1098	15.6143	9.1077	14.15
XLOC_044973	GREB1	chr11	86212106	86257573	11.2515	16.0224	12.2808	24.01	24.6645	23.3564	28.0021	30.0885	28.1689
XLOC_046863	GREB1	chr11	86201349	86201848	2.11809	5.69425	3.30917	4.17104	2.13137	1.74461	2.81582	2.9211	2.94689
XLOC_046864	GREB1	chr11	86207197	86207348	151.901	31.2644	40.9067	65.0117	29.0696	71.6561	22.7093	30.7788	39.9122
XLOC_046865	GREB1	chr11	86208016	86208170	181.035	69.6378	109.311	57.6614	45.5415	82.2004	98.3691	63.0639	39.5438
XLOC_046866	GREB1	chr11	86209606	86210436	4.30196	2.57271	2.52319	2.50571	2.01472	1.44884	2.13733	1.39488	1.78365
XLOC_046867	GREB1	chr11	86210556	86211950	5.84908	8.39909	10.9836	14.2112	15.309	14.4089	17.9684	20.3127	19.3333
XLOC_046868	GREB1	chr11	86212106	86257573	4.2841	4.69535	5.58165	2.68622	3.66787	3.10077	3.71672	3.40453	3.23665
XLOC_046869	GREB1	chr11	86262569	86263090	0.0	0.594471	0.0	1.06884	0.30837	1.23023	0.176768	0.588974	0.865563
XLOC_046870	GREB1	chr11	86263211	86263499	5.50705	3.2833	4.29221	0.985975	1.24957	3.90307	3.6997	1.57023	1.89756
XLOC_046871	GREB1	chr11	86265408	86266184	2.3388	3.14673	0.914412	0.733446	1.36811	0.726658	0.527258	1.16533	1.42853
XLOC_046872	GREB1	chr11	86266323	86266691	3.43455	2.04955	2.67965	0.0	0.0	0.350933	0.0	0.333193	0.594665
XLOC_044974	GREB1;E2F6	chr11	86266935	86357641	65.2958	52.2753	62.7664	21.3178	21.5339	22.8935	28.4629	22.3971	21.6708
XLOC_044346	GRHL1	chr11	87578422	87591840	1.09828	2.66638	1.54172	0.545382	0.346746	0.622581	0.446744	0.769344	0.832114

XLOC_044347	GRHL1	chr11	87592000	87616580	9.07149	9.01481	9.124	10.3309	9.30776	10.209	8.21687	13.374	8.28457
XLOC_046932	GRHL1	chr11	87592000	87616580	0.0	0.770176	0.0	0.92321	1.00373	0.666594	1.04314	0.897245	1.01114
XLOC_086417	GRHPR;ZBTB5;POLR1E;FBXO10	chr8	61928131	62033473	0.709386	0.561815	1.01732	0.768512	1.37886	0.87366	0.390697	0.160306	1.38799
XLOC_060186	GRIA2;GRIA2	chr17	42792949	42876665	87.1983	110.384	94.3101	70.9138	69.1384	74.8664	74.7254	59.2728	75.5947
XLOC_061429	GRIA2;GRIA2	chr17	42789597	42790003	0.0	1.7375	0.0	0.520681	2.23902	0.894736	0.508683	0.284028	1.00953
XLOC_061430	GRIA2;GRIA2	chr17	42792949	42876665	31.3281	36.341	48.9594	38.7756	39.7429	38.582	40.3793	32.239	44.5929
XLOC_061431	GRIA2;GRIA2	chr17	42792949	42876665	0.0	4.61963	4.02687	2.07687	1.98923	1.58856	4.75993	1.01051	1.56708
XLOC_092359	GRIA3	chrX	7154195	7319385	14.3895	12.8514	14.3471	2.42203	2.50588	2.13144	1.34064	1.56672	1.84317
XLOC_092693	GRIA3	chrX	7371928	7372609	4.1449	3.30405	3.24038	0.990135	0.107578	0.285803	0.24821	0.137326	0.481856
XLOC_092694	GRIA3	chrX	7372829	7375192	4.26294	5.49499	3.59302	2.20555	1.41626	1.6387	0.991181	1.22166	1.00454
XLOC_092695	GRIA3	chrX	7428151	7428902	0.0	0.364348	0.0	1.20105	0.475061	0.883211	0.878355	0.606846	0.318953
XLOC_089386	GRIK2	chr9	48987194	49042523	25.1502	35.6055	28.5129	71.6429	67.3532	71.0802	63.7445	58.1829	61.8296
XLOC_089387	GRIK2	chr9	49055537	49078821	9.56179	14.7207	13.0142	17.5615	14.2454	17.1469	17.5143	22.5809	14.8807
XLOC_089388	GRIK2	chr9	49114384	49117414	0.0	0.277838	0.0	0.499578	0.145202	0.0963608	0.168299	0.0928542	0.811365
XLOC_089877	GRIK2	chr9	48468512	48496050	87.872	103.251	81.1274	81.7543	71.8955	80.25	77.9261	92.0042	80.8024
XLOC_089878	GRIK2	chr9	48657051	48837186	51.9201	77.379	72.0162	0.150902	0.122325	0.0700521	0.0307928	0.156878	0.029425
XLOC_089879	GRIK2	chr9	48899237	48908749	0.0	0.0	0.0	0.738051	0.917693	0.991561	0.396013	0.653429	1.02334
XLOC_090760	GRIK2	chr9	48468512	48496050	0.0	1.40499	0.0	1.26305	0.181744	0.967384	1.66104	0.462141	0.6133
XLOC_090761	GRIK2	chr9	48652303	48652474	53.6799	179.223	127.824	0.0	0.0	0.0	0.0	0.0	0.0
XLOC_090762	GRIK2	chr9	48652677	48653512	27.7564	39.9022	22.5413	0.0	0.0833322	0.110632	0.0	0.106517	0.0
XLOC_090763	GRIK2	chr9	48653612	48654191	37.7525	41.0237	29.5036	0.153668	0.0	0.17712	0.0	0.169869	0.0
XLOC_090764	GRIK2	chr9	48654256	48656359	51.3006	70.0774	53.822	0.0	0.0875435	0.0773832	0.0340221	0.112231	0.0
XLOC_090765	GRIK2	chr9	48656437	48656866	31.9688	30.2247	35.3594	0.0	0.0	0.0	0.0	0.0	0.0
XLOC_090766	GRIK2	chr9	48985574	48986359	3.45787	1.72313	7.21049	1.65243	0.809202	0.835694	0.519856	1.03397	0.804645
XLOC_090767	GRIK2	chr9	48986686	48987017	4.16224	1.2413	3.24574	1.86099	1.58678	0.847452	4.63192	2.00326	0.719153
XLOC_090768	GRIK2	chr9	48987194	49042523	4.03693	3.21822	4.20812	1.80821	1.67696	1.3921	1.57245	1.7395	0.704064
XLOC_090769	GRIK2	chr9	48987194	49042523	17.3232	10.3424	4.50738	4.9071	4.88758	8.58128	5.30088	5.35494	7.51175
XLOC_090770	GRIK2	chr9	48987194	49042523	9.75036	14.5617	22.8374	12.3413	7.9787	23.4866	5.50207	6.31001	9.21071
XLOC_090771	GRIK2	chr9	49043162	49043476	0.0	2.74843	3.59321	4.53444	3.50409	2.3413	2.74322	3.09169	2.38637

XLOC_090772	GRIK2	chr9	49078898	49079359	7.14982	6.40559	1.86125	16.209	13.2548	17.3939	15.9767	14.2757	18.6341
XLOC_090773	GRIK2	chr9	49080594	49081465	1.01368	0.909378	1.58556	0.0	1.0291	0.210169	0.0916728	0.101209	0.354005
XLOC_070121	GRIK3	chr3	109341043	109685693	2.66841	5.68924	2.2938	0.386782	0.195851	0.138165	0.0486627	0.197	0.131171
XLOC_076411	GRIN2B	chr5	96373841	96783123	4.03795	4.41767	2.55135	0.17794	0.0448458	0.15669	0.0715393	0.0261629	0.0620568
XLOC_086575	GRIN3A	chr8	92991369	92999015	0.0	0.0	0.0	0.0	0.0	0.0373878	0.0	0.0	0.0
XLOC_088583	GRIN3A	chr8	92999698	93001667	0.0	0.0	0.0	0.0	0.0	0.0415466	0.0	0.0	0.0
XLOC_086574	GRIN3A;PPP3R2	chr8	92961046	92990420	1.74501	0.0	0.0	0.0	0.0	0.0	0.122508	0.0	0.0
XLOC_038029	GRK7	chr1	128103298	128193596	46.5433	41.4073	40.49	35.0828	36.1728	39.0418	40.5019	30.3636	37.8118
XLOC_040203	GRK7	chr1	128103298	128193596	8.62749	6.86761	6.73432	5.14489	4.20455	5.30553	4.27503	2.52681	4.73871
XLOC_040204	GRK7	chr1	128103298	128193596	2.6089	3.50958	3.05946	3.85626	2.23576	3.91432	3.63738	3.11449	3.52658
XLOC_072576	GRM3	chr4	33925077	33944578	0.760914	2.27596	1.19051	0.136414	0.178629	0.237015	0.0	0.0	0.0664941
XLOC_073947	GRM3	chr4	33945545	33946884	1.22428	1.09873	0.478943	0.0548795	0.191834	0.0636167	0.0	0.0	0.107044
XLOC_073948	GRM3	chr4	33947392	33948050	0.0	3.02372	2.25939	0.0	0.0	0.14941	0.129658	0.0	0.125971
XLOC_073949	GRM3	chr4	33970860	33971126	0.0	5.8834	10.2544	1.76982	3.46449	3.98337	3.81731	4.33926	5.65829
XLOC_074603	GRM8	chr4	91805421	91806539	71.5342	86.4874	69.5093	201.952	184.5	204.301	169.698	214.65	212.743
XLOC_092266	GRPR	chrX	134589500	134607165	27.4615	38.0083	31.2651	44.2176	39.3124	41.1673	49.4386	53.6312	54.3393
XLOC_092580	GRPR	chrX	134589500	134607165	6.84515	4.23088	5.32468	7.29951	9.64556	8.11063	11.0341	7.8065	12.0876
XLOC_093589	GRPR	chrX	134589500	134607165	1.77949	4.78528	1.39047	3.18657	1.1049	2.01964	3.80823	2.81605	2.01392
XLOC_079815	GRSF1	chr6	87913817	87936730	8.51803	15.1714	14.3005	74.2645	45.2068	53.1341	66.3567	106.328	55.3695
XLOC_053190	GSDMC;FAM49B;ASAP1	chr14	11553500	12242337	54.1576	56.0217	65.8718	68.0128	58.259	58.0255	54.2682	64.293	44.0725
XLOC_053827	GSDMC;FAM49B;ASAP1	chr14	11553500	12242337	1.84188	1.70883	2.59517	3.28756	2.92504	2.68704	2.36829	1.96936	2.25816
XLOC_053828	GSDMC;FAM49B;ASAP1	chr14	11553500	12242337	0.0	2.02718	1.32538	1.21511	1.58059	0.875383	2.12054	0.671828	0.88623
XLOC_053829	GSDMC;FAM49B;ASAP1	chr14	11553500	12242337	4.94369	2.06972	3.09317	2.74693	3.08916	2.76793	1.6103	1.97506	1.64033
XLOC_053830	GSDMC;FAM49B;ASAP1	chr14	11553500	12242337	0.0	1.93468	2.52997	1.3046	2.46662	1.84641	1.24784	1.45684	1.62486
XLOC_053831	GSDMC;FAM49B;ASAP1	chr14	11553500	12242337	0.0	1.92244	0.0	0.288355	0.741667	2.96555	1.40007	0.939948	1.11623
XLOC_053832	GSDMC;FAM49B;ASAP1	chr14	11553500	12242337	0.964991	1.15432	0.0	1.47027	2.71399	1.90131	2.18374	0.964134	1.43254
XLOC_053833	GSDMC;FAM49B;ASAP1	chr14	11553500	12242337	0.0	1.18073	0.0	2.12422	1.81347	2.42047	1.36	0.764063	0.684471
XLOC_053834	GSDMC;FAM49B;ASAP1	chr14	11553500	12242337	0.0	0.916533	4.79326	2.47223	2.59554	3.45847	1.60585	1.49593	2.12932

XLOC_053835	GSDMC;FAM49B;ASAP1	chr14	11553500	12242337	0.0	2.3236	3.03788	2.78668	2.97519	1.98531	4.01816	1.50425	3.03075
XLOC_053836	GSDMC;FAM49B;ASAP1	chr14	11553500	12242337	0.0	4.09913	2.67967	1.84316	1.84241	1.75478	0.892597	1.33307	1.18944
XLOC_053837	GSDMC;FAM49B;ASAP1	chr14	11553500	12242337	3.32437	0.992004	5.18785	2.9733	2.80457	2.71912	0.865557	0.968991	2.59114
XLOC_053838	GSDMC;FAM49B;ASAP1	chr14	11553500	12242337	0.295012	0.264845	0.692695	1.03186	0.579308	0.76801	0.757089	0.950957	0.748912
XLOC_053839	GSDMC;FAM49B;ASAP1	chr14	11553500	12242337	0.387478	0.115943	0.0	0.833949	0.790725	0.524242	0.42544	0.389896	0.406895
XLOC_053840	GSDMC;FAM49B;ASAP1	chr14	11553500	12242337	0.0	0.0	0.0	3.42818	0.878824	0.391006	0.989766	0.740885	0.331591
XLOC_053841	GSDMC;FAM49B;ASAP1	chr14	11553500	12242337	0.0	0.0	0.0	0.638284	0.557602	0.874215	0.530387	1.03861	0.622403
XLOC_053842	GSDMC;FAM49B;ASAP1	chr14	11553500	12242337	0.0	0.244295	0.0	0.585752	0.511086	0.847782	0.370714	0.735788	0.214109
XLOC_053843	GSDMC;FAM49B;ASAP1	chr14	11553500	12242337	0.0	0.0	0.0	0.396766	0.514066	2.27967	0.588114	0.43616	0.770375
XLOC_053844	GSDMC;FAM49B;ASAP1	chr14	11553500	12242337	0.0	0.0	0.0	0.906254	0.337399	0.44828	0.389045	0.430709	0.755874
XLOC_037889	GSK3B;GSK3B;NR1I2	chr1	65186655	65276838	26.057	24.0617	24.9765	18.7275	19.5662	20.5221	24.7686	21.721	23.1942
XLOC_039574	GSK3B;GSK3B;NR1I2	chr1	65186655	65276838	4.6893	2.80089	5.49349	1.46879	0.905829	2.16964	0.62087	1.38209	2.85206
XLOC_072742	GSTK1;ZYX;FAM131B;TMEM139;CLCN1;CASP2;EPHA1	chr4	107434816	107635103	6.17629	8.50629	6.54026	0.536903	0.0	0.107633	0.0938677	0.214208	0.0906534
XLOC_074825	GSTK1;ZYX;FAM131B;TMEM139;CLCN1;CASP2;EPHA1	chr4	107434816	107635103	1.44731	0.606345	1.13278	0.0519195	0.0	0.0301432	0.0265325	0.0583191	0.0253389
XLOC_074826	GSTK1;ZYX;FAM131B;TMEM139;CLCN1;CASP2;EPHA1	chr4	107434816	107635103	0.0	1.27704	0.668013	0.0382719	0.0	0.0	0.0780433	0.0	0.0373433
XLOC_074827	GSTK1;ZYX;FAM131B;TMEM139;CLCN1;CASP2;EPHA1	chr4	107434816	107635103	0.459341	0.274866	0.359451	0.0	0.0	0.0	0.0419703	0.0461755	0.0401847

XLOC_074828	GSTK1;ZYX;FAM131B;TMEM139;CLCN1;CASP2;EPHA1	chr4	107434816	107635103	3.22098	0.0	0.0	0.0	0.0	0.0	0.0	0.313221	0.0
XLOC_074829	GSTK1;ZYX;FAM131B;TMEM139;CLCN1;CASP2;EPHA1	chr4	107434816	107635103	1.92437	0.191887	0.0	0.0	0.0	0.0	0.0584119	0.0643382	0.0560795
XLOC_074830	GSTK1;ZYX;FAM131B;TMEM139;CLCN1;CASP2;EPHA1	chr4	107434816	107635103	0.0	0.21241	0.55554	0.0	0.0	0.0	0.0	0.0	0.0
XLOC_074831	GSTK1;ZYX;FAM131B;TMEM139;CLCN1;CASP2;EPHA1	chr4	107434816	107635103	3.31409	0.0	2.59013	0.148396	0.0	0.0	0.0	0.0	0.0
XLOC_074832	GSTK1;ZYX;FAM131B;TMEM139;CLCN1;CASP2;EPHA1	chr4	107434816	107635103	0.0	0.681657	0.0	0.0	0.0	0.0	0.102848	0.0	0.0
XLOC_074833	GSTK1;ZYX;FAM131B;TMEM139;CLCN1;CASP2;EPHA1	chr4	107434816	107635103	0.842462	0.503934	1.97697	0.0755104	0.0	0.0	0.0	0.0	0.0735985
XLOC_074834	GSTK1;ZYX;FAM131B;TMEM139;CLCN1;CASP2;EPHA1	chr4	107434816	107635103	0.361884	0.21657	0.0	0.0	0.0	0.0	0.0331259	0.0	0.0
XLOC_074835	GSTK1;ZYX;FAM131B;TMEM139;CLCN1;CASP2;EPHA1	chr4	107434816	107635103	0.522003	0.312344	0.40846	0.0	0.0	0.0	0.0476401	0.0	0.0
XLOC_074836	GSTK1;ZYX;FAM131B;TMEM139;CLCN1;CASP2;EPHA1	chr4	107434816	107635103	0.0	0.275742	0.360596	0.0	0.0	0.0	0.0421028	0.0	0.0

XLOC_074837	GSTK1;ZYX;FAM131B;TMEM139;CLCN1;CASP2;EPHA1	chr4	107434816	107635103	0.470089	0.281295	0.735716	0.0	0.0	0.0	0.0	0.0	0.0
XLOC_074838	GSTK1;ZYX;FAM131B;TMEM139;CLCN1;CASP2;EPHA1	chr4	107434816	107635103	0.316995	0.474285	0.248097	0.0	0.0248945	0.0	0.0	0.0	0.0
XLOC_074839	GSTK1;ZYX;FAM131B;TMEM139;CLCN1;CASP2;EPHA1	chr4	107434816	107635103	0.0	0.29894	0.0	0.0	0.0	0.0	0.0	0.0	0.0
XLOC_074840	GSTK1;ZYX;FAM131B;TMEM139;CLCN1;CASP2;EPHA1	chr4	107434816	107635103	3.11038	0.928287	0.0	0.0	0.0	0.0	0.0	0.0	0.0
XLOC_074841	GSTK1;ZYX;FAM131B;TMEM139;CLCN1;CASP2;EPHA1	chr4	107434816	107635103	0.0	0.807524	0.0	0.0	0.0	0.0	0.0	0.0	0.0
XLOC_074842	GSTK1;ZYX;FAM131B;TMEM139;CLCN1;CASP2;EPHA1	chr4	107434816	107635103	0.0	0.260142	0.680372	0.0779603	0.0	0.0	0.0	0.0869984	0.0
XLOC_074843	GSTK1;ZYX;FAM131B;TMEM139;CLCN1;CASP2;EPHA1	chr4	107434816	107635103	0.50922	0.380969	0.199285	0.0	0.0400107	0.0	0.0	0.0	0.0
XLOC_074844	GSTK1;ZYX;FAM131B;TMEM139;CLCN1;CASP2;EPHA1	chr4	107434816	107635103	0.0	0.0	0.0	0.0	0.0	0.0	0.0	0.158801	0.0
XLOC_074845	GSTK1;ZYX;FAM131B;TMEM139;CLCN1;CASP2;EPHA1	chr4	107434816	107635103	0.0	0.452738	1.18403	0.0	0.0	0.0	0.0	0.0	0.0

XLOC_074846	GSTK1;ZYX;FAM131B;TMEM139;CLCN1;CASP2;EPHA1	chr4	107434816	107635103	2.065	0.0	0.0	0.0	0.0	0.0	0.0	0.0	0.0
XLOC_074847	GSTK1;ZYX;FAM131B;TMEM139;CLCN1;CASP2;EPHA1	chr4	107434816	107635103	2.31464	0.69128	0.0	0.0	0.0	0.0	0.0	0.227476	0.0
XLOC_074848	GSTK1;ZYX;FAM131B;TMEM139;CLCN1;CASP2;EPHA1	chr4	107434816	107635103	5.08478	0.0	0.0	0.0	0.0	0.0	0.0	0.0	0.0
XLOC_074849	GSTK1;ZYX;FAM131B;TMEM139;CLCN1;CASP2;EPHA1	chr4	107434816	107635103	0.0	0.250387	0.327439	0.0	0.0	0.0	0.0	0.0420826	0.0
XLOC_074850	GSTK1;ZYX;FAM131B;TMEM139;CLCN1;CASP2;EPHA1	chr4	107434816	107635103	0.295253	0.0	0.693259	0.0	0.0231911	0.0614901	0.0	0.0	0.0
XLOC_074851	GSTK1;ZYX;FAM131B;TMEM139;CLCN1;CASP2;EPHA1	chr4	107434816	107635103	0.987389	0.590541	0.772238	0.0884869	0.0	0.0	0.0893382	0.0	0.0
XLOC_074852	GSTK1;ZYX;FAM131B;TMEM139;CLCN1;CASP2;EPHA1	chr4	107434816	107635103	0.0	0.0	0.0	0.0	0.163013	0.0	0.0	0.0	0.0
XLOC_074853	GSTK1;ZYX;FAM131B;TMEM139;CLCN1;CASP2;EPHA1	chr4	107434816	107635103	0.0	0.7914	0.0	0.0	0.0	0.0	0.0	0.0	0.0
XLOC_074854	GSTK1;ZYX;FAM131B;TMEM139;CLCN1;CASP2;EPHA1	chr4	107434816	107635103	0.0	0.330929	1.73098	0.0	0.0	0.0	0.0999186	0.0	0.0

XLOC_074855	GSTK1;ZYX;FAM131B;TMEM139;CLCN1;CASP2;EPHA1	chr4	107434816	107635103	0.0	0.905477	0.0	0.0	0.0	0.0	0.0	0.0	0.0
XLOC_074856	GSTK1;ZYX;FAM131B;TMEM139;CLCN1;CASP2;EPHA1	chr4	107434816	107635103	0.827047	0.24736	0.646944	0.07413	0.129367	0.0	0.075053	0.0	0.0
XLOC_074857	GSTK1;ZYX;FAM131B;TMEM139;CLCN1;CASP2;EPHA1	chr4	107434816	107635103	0.0	0.0	1.66431	0.0	0.0	0.0	0.0	0.0	0.0
XLOC_074858	GSTK1;ZYX;FAM131B;TMEM139;CLCN1;CASP2;EPHA1	chr4	107434816	107635103	1.06598	0.318747	0.833634	0.0	0.0	0.0	0.0	0.0	0.0
XLOC_074860	GSTK1;ZYX;FAM131B;TMEM139;CLCN1;CASP2;EPHA1	chr4	107434816	107635103	0.0	0.0	0.0	0.0	0.111802	0.0	0.0	0.0	0.0
XLOC_074862	GSTK1;ZYX;FAM131B;TMEM139;CLCN1;CASP2;EPHA1	chr4	107434816	107635103	0.823281	0.738705	0.0	0.0	0.0	0.0	0.0	0.0	0.0
XLOC_070252	GSTM1	chr3	33811111	33844306	6.16916	5.80961	9.16216	5.1373	7.06589	6.09381	9.89226	7.89601	8.68007
XLOC_070790	GSTM1	chr3	33799497	33800123	0.0	0.922847	0.0	0.414825	1.20052	0.159519	0.27653	0.153147	0.134528
XLOC_070793	GSTM1	chr3	33810516	33810990	4.57079	11.6036	8.92493	8.79502	13.4268	10.8129	8.48149	8.98718	10.5264
XLOC_069871	GSTM1;GSTM1	chr3	33800195	33810380	29.5608	27.8078	32.2249	31.6789	37.5533	32.9885	36.7427	26.8285	42.4874
XLOC_070791	GSTM1;GSTM1	chr3	33800195	33810380	4.47189	8.68172	5.23939	6.40381	7.26237	8.28163	8.1076	6.59845	9.52303
XLOC_070792	GSTM1;GSTM1	chr3	33800195	33810380	16.6984	11.9585	13.0292	5.37603	6.91484	12.9737	7.53271	8.10827	11.5682
XLOC_079758	GSX2	chr6	71271733	71293659	12.9355	17.969	15.5452	15.7412	20.8732	15.1378	16.2084	12.8637	24.9363
XLOC_080149	GSX2	chr6	71271733	71293659	0.0	0.982705	2.57016	1.32525	0.835195	0.852544	0.745493	0.493233	1.14824
XLOC_081317	GSX2	chr6	71271733	71293659	0.0	2.88564	4.71679	3.78332	3.85689	3.24791	2.60928	1.44207	2.10516

XLOC_081318	GSX2	chr6	71271733	71293659	3.19323	4.4559	4.16204	1.43072	1.99414	1.21339	1.73106	0.955866	1.85826
XLOC_081319	GSX2	chr6	71271733	71293659	0.0	5.6355	0.0	1.69445	1.89882	1.90931	1.57208	0.0	1.08439
XLOC_081320	GSX2	chr6	71271733	71293659	0.0	1.79435	1.17324	0.537742	1.76052	0.467182	1.15849	0.45067	2.09701
XLOC_066294	GTDC1	chr2	52692092	52742227	32.4285	39.7032	31.2818	25.1626	23.5176	24.1434	23.5129	23.805	23.2368
XLOC_067523	GTDC1	chr2	52619736	52620573	7.45087	9.23008	7.49168	20.6978	20.7723	20.5174	18.8494	20.9228	16.5385
XLOC_067524	GTDC1	chr2	52742290	52742829	3.79195	0.0	1.48142	2.20675	0.882116	0.977379	0.0	0.56182	0.33
XLOC_065650	GTDC1;ARHGAP15	chr2	52857967	53108280	8.21113	12.717	7.18129	9.25318	8.92157	9.53134	10.0529	8.79409	9.34065
XLOC_067525	GTDC1;ARHGAP15	chr2	52857967	53108280	0.0	5.00568	7.85356	1.50024	0.771832	2.74371	0.290984	1.95465	0.581056
XLOC_067526	GTDC1;ARHGAP15	chr2	52857967	53108280	2.47832	1.48247	5.16965	0.888545	1.09839	0.943127	1.04956	0.909373	0.938257
XLOC_067527	GTDC1;ARHGAP15	chr2	52857967	53108280	3.88362	1.7404	3.0343	1.21691	0.90313	1.401	0.863236	0.766769	0.337918
XLOC_067528	GTDC1;ARHGAP15	chr2	52857967	53108280	3.78207	5.08492	2.95512	1.01584	1.02651	1.75474	0.168288	0.186795	0.987428
XLOC_067529	GTDC1;ARHGAP15	chr2	52857967	53108280	0.0	1.43288	1.87357	1.28813	0.926497	0.986344	2.3271	0.235537	0.416815
XLOC_067530	GTDC1;ARHGAP15	chr2	52857967	53108280	0.0	1.44694	7.56656	0.8683	1.10538	1.47775	5.34569	0.927991	0.0
XLOC_067531	GTDC1;ARHGAP15	chr2	52857967	53108280	0.0	4.47639	5.85305	1.34139	1.15697	1.28326	1.09966	0.490016	0.216969
XLOC_067532	GTDC1;ARHGAP15	chr2	52857967	53108280	0.0	0.748644	5.87328	0.0	0.580467	0.515056	0.0	0.245832	0.0
XLOC_045713	GTF2A1L	chr11	30770948	30771657	1.31148	0.392062	0.0	0.469964	0.408691	0.814228	0.589777	0.52186	0.343129
XLOC_069911	GTF2B	chr3	55181854	55209938	13.2663	22.004	14.3879	31.7367	34.7088	28.9267	56.6588	33.2931	43.4711
XLOC_082805	GTF2F1	chr7	19282720	19296235	2.27799	2.73945	2.81353	5.6843	6.73677	5.23382	6.64469	4.75355	3.80714
XLOC_044483	GTF3C4	chr11	102776370	102778559	0.0	0.356298	0.0	0.160168	0.139985	0.123789	0.325642	0.119526	0.104142
XLOC_047327	GTF3C4	chr11	102778660	102779674	0.0	0.50511	0.0	0.22706	0.330169	0.0	0.229817	0.16897	0.368849
XLOC_044486	GTF3C5;CEL	chr11	103069726	103112827	1.27446	1.3429	1.62158	4.66865	5.07299	4.14229	4.19795	3.27283	4.67819
XLOC_045126	GTF3C5;CEL	chr11	103069726	103112827	3.86748	2.50433	2.55815	5.79112	6.56624	6.72201	5.30168	4.87964	6.42936
XLOC_050872	GTPBP5	chr13	55529800	55536982	20.4234	24.9511	23.6942	32.3372	29.7158	31.8549	34.7253	39.6563	33.3974
XLOC_037855	GTPBP8	chr1	58146430	58172350	1.08838	0.434226	0.0	1.95202	0.598092	1.43496	0.19925	0.730268	0.603241
XLOC_060189	GUCY1A3	chr17	44607701	44735319	6.57219	4.82591	5.14223	0.0	0.0	0.0	0.0	0.0	0.0
XLOC_061447	GUCY1A3	chr17	44604464	44605098	3.03586	3.17581	5.93255	0.0	0.0	0.0	0.0	0.0	0.0
XLOC_061448	GUCY1A3	chr17	44605604	44606950	0.608532	2.36657	3.33287	0.109113	0.0	0.0	0.0	0.0	0.0532075

XLOC_064217	GUCY2D;HES7;TMEM107;VAMP2;ALOX12B;RANGRF;ODF4;AURKB;ARHGEF15;C19H17orf59;SLC25A35;PFAS;RPL26;NDEL1;ALOX15B	chr19	28262222	28680333	31.3907	37.4748	41.459	0.466491	0.239699	0.373247	0.29651	0.223694	0.298407
XLOC_064882	GUCY2D;HES7;TMEM107;VAMP2;ALOX12B;RANGRF;ODF4;AURKB;ARHGEF15;C19H17orf59;SLC25A35;PFAS;RPL26;NDEL1;ALOX15B	chr19	28262222	28680333	1.93142	6.34744	0.0	0.0	0.0	0.0	0.0	0.0	0.168063
XLOC_064883	GUCY2D;HES7;TMEM107;VAMP2;ALOX12B;RANGRF;ODF4;AURKB;ARHGEF15;C19H17orf59;SLC25A35;PFAS;RPL26;NDEL1;ALOX15B	chr19	28262222	28680333	2.53349	8.32139	9.89137	0.0	0.195469	0.0	0.0	0.0	0.219981

XLOC_064884	GUCY2D;HES7;TMEM107;VAMP2;ALOX12B;RANGRF;ODF4;AURKB;ARHGEF15;C19H17orf59;SLC25A35;PFAS;RPL26;NDEL1;ALOX15B	chr19	28262222	28680333	4.72809	14.0966	18.4292	0.0	0.0	0.0	0.0	0.45256	0.0
XLOC_064885	GUCY2D;HES7;TMEM107;VAMP2;ALOX12B;RANGRF;ODF4;AURKB;ARHGEF15;C19H17orf59;SLC25A35;PFAS;RPL26;NDEL1;ALOX15B	chr19	28262222	28680333	4.61452	8.26896	3.60405	0.0	0.178329	0.23728	0.0	0.0	0.200496
XLOC_064886	GUCY2D;HES7;TMEM107;VAMP2;ALOX12B;RANGRF;ODF4;AURKB;ARHGEF15;C19H17orf59;SLC25A35;PFAS;RPL26;NDEL1;ALOX15B	chr19	28262222	28680333	6.88187	8.64016	16.1403	0.0	0.107173	0.142362	0.0	0.0	0.120008

XLOC_064887	GUCY2D;HES7;TMEM107;VAMP2;ALOX12B;RANGRF;ODF4;AURKB;ARHGEF15;C19H17orf59;SLC25A35;PFAS;RPL26;NDEL1;ALOX15B	chr19	28262222	28680333	2.54239	5.31399	25.8077	0.0	0.0	0.0	0.0	0.249183	0.66224
XLOC_064888	GUCY2D;HES7;TMEM107;VAMP2;ALOX12B;RANGRF;ODF4;AURKB;ARHGEF15;C19H17orf59;SLC25A35;PFAS;RPL26;NDEL1;ALOX15B	chr19	28262222	28680333	15.4442	8.20473	12.0697	0.153668	0.0	0.0	0.0	0.0	0.149437
XLOC_064889	GUCY2D;HES7;TMEM107;VAMP2;ALOX12B;RANGRF;ODF4;AURKB;ARHGEF15;C19H17orf59;SLC25A35;PFAS;RPL26;NDEL1;ALOX15B	chr19	28262222	28680333	6.705	7.51394	14.4105	0.0	0.260419	0.0	0.14976	0.0	0.0

XLOC_064890	GUCY2D;HES7;TMEM107;VAMP2;ALOX12B;RANGRF;ODF4;AURKB;ARHGEF15;C19H17orf59;SLC25A35;PFAS;RPL26;NDEL1;ALOX15B	chr19	28262222	28680333	6.29892	8.15353	3.28042	0.0	0.162548	0.0	0.0	0.0	0.0
XLOC_064891	GUCY2D;HES7;TMEM107;VAMP2;ALOX12B;RANGRF;ODF4;AURKB;ARHGEF15;C19H17orf59;SLC25A35;PFAS;RPL26;NDEL1;ALOX15B	chr19	28262222	28680333	7.96048	11.8715	6.20834	0.0	0.0	0.405553	0.341695	0.0	0.0
XLOC_064892	GUCY2D;HES7;TMEM107;VAMP2;ALOX12B;RANGRF;ODF4;AURKB;ARHGEF15;C19H17orf59;SLC25A35;PFAS;RPL26;NDEL1;ALOX15B	chr19	28262222	28680333	5.46629	21.1836	17.0419	0.489312	0.0	0.553533	0.0	0.0	0.0

XLOC_064893	GUCY2D;HES7;TMEM107;VAMP2;ALOX12B;RANGRF;ODF4;AURKB;ARHGEF15;C19H17orf59;SLC25A35;PFAS;RPL26;NDEL1;ALOX15B	chr19	28262222	28680333	8.23044	15.955	9.62749	0.0	0.31387	0.0	0.0	0.0	0.0
XLOC_064894	GUCY2D;HES7;TMEM107;VAMP2;ALOX12B;RANGRF;ODF4;AURKB;ARHGEF15;C19H17orf59;SLC25A35;PFAS;RPL26;NDEL1;ALOX15B	chr19	28262222	28680333	14.1843	14.0966	3.68584	0.0	0.0	0.0	0.0	0.0	0.407895
XLOC_064895	GUCY2D;HES7;TMEM107;VAMP2;ALOX12B;RANGRF;ODF4;AURKB;ARHGEF15;C19H17orf59;SLC25A35;PFAS;RPL26;NDEL1;ALOX15B	chr19	28262222	28680333	0.0	9.36845	6.68137	0.0	0.219586	0.0	0.0	0.0	0.0

XLOC_064896	GUCY2D;HES7;TMEM107;VAMP2;ALOX12B;RANGRF;ODF4;AURKB;ARHGEF15;C19H17orf59;SLC25A35;PFAS;RPL26;NDEL1;ALOX15B	chr19	28262222	28680333	8.30118	27.2451	6.47489	0.0	0.0	0.0	0.0	1.54695	0.0
XLOC_064897	GUCY2D;HES7;TMEM107;VAMP2;ALOX12B;RANGRF;ODF4;AURKB;ARHGEF15;C19H17orf59;SLC25A35;PFAS;RPL26;NDEL1;ALOX15B	chr19	28262222	28680333	7.42811	9.98739	10.1574	0.16627	0.0	0.383013	0.0	0.0	0.161633
XLOC_064898	GUCY2D;HES7;TMEM107;VAMP2;ALOX12B;RANGRF;ODF4;AURKB;ARHGEF15;C19H17orf59;SLC25A35;PFAS;RPL26;NDEL1;ALOX15B	chr19	28262222	28680333	2.0404	10.3621	11.1582	0.0	0.158036	0.0	0.0	0.0	0.0

XLOC_064899	GUCY2D;HES7;TMEM107;VAMP2;ALOX12B;RANGRF;ODF4;AURKB;ARHGEF15;C19H17orf59;SLC25A35;PFAS;RPL26;NDEL1;ALOX15B	chr19	28262222	28680333	0.0	25.5957	20.0733	0.0	0.0	0.0	0.0	0.0	0.0
XLOC_064900	GUCY2D;HES7;TMEM107;VAMP2;ALOX12B;RANGRF;ODF4;AURKB;ARHGEF15;C19H17orf59;SLC25A35;PFAS;RPL26;NDEL1;ALOX15B	chr19	28262222	28680333	11.8772	14.188	5.56547	0.212577	0.0	0.244192	0.0	0.0	0.0
XLOC_064901	GUCY2D;HES7;TMEM107;VAMP2;ALOX12B;RANGRF;ODF4;AURKB;ARHGEF15;C19H17orf59;SLC25A35;PFAS;RPL26;NDEL1;ALOX15B	chr19	28262222	28680333	9.71265	6.84826	10.3124	0.186576	0.0	0.0720627	0.126227	0.0	0.0

XLOC_064902	GUCY2D;HES7;TMEM107;VAMP2;ALOX12B;RANGRF;ODF4;AURKB;ARHGEF15;C19H17orf59;SLC25A35;PFAS;RPL26;NDEL1;ALOX15B	chr19	28262222	28680333	3.64132	14.1219	8.52152	0.0	0.0	0.0	0.0	0.0	0.0
XLOC_064903	GUCY2D;HES7;TMEM107;VAMP2;ALOX12B;RANGRF;ODF4;AURKB;ARHGEF15;C19H17orf59;SLC25A35;PFAS;RPL26;NDEL1;ALOX15B	chr19	28262222	28680333	8.94077	8.01469	15.3707	0.0	0.0	0.0	0.0	0.176855	0.0
XLOC_064904	GUCY2D;HES7;TMEM107;VAMP2;ALOX12B;RANGRF;ODF4;AURKB;ARHGEF15;C19H17orf59;SLC25A35;PFAS;RPL26;NDEL1;ALOX15B	chr19	28262222	28680333	3.90455	8.16562	7.62662	0.0	0.151317	0.0	0.0	0.0	0.0

XLOC_064905	GUCY2D;HES7;TMEM107;VAMP2;ALOX12B;RANGRF;ODF4;AURKB;ARHGEF15;C19H17orf59;SLC25A35;PFAS;RPL26;NDEL1;ALOX15B	chr19	28262222	28680333	8.23044	12.2731	12.8367	0.0	0.0	0.0	0.0	0.0	0.0
XLOC_064906	GUCY2D;HES7;TMEM107;VAMP2;ALOX12B;RANGRF;ODF4;AURKB;ARHGEF15;C19H17orf59;SLC25A35;PFAS;RPL26;NDEL1;ALOX15B	chr19	28262222	28680333	1.30674	0.781294	11.2382	0.0	0.0	0.0	0.117539	0.0	0.0
XLOC_064907	GUCY2D;HES7;TMEM107;VAMP2;ALOX12B;RANGRF;ODF4;AURKB;ARHGEF15;C19H17orf59;SLC25A35;PFAS;RPL26;NDEL1;ALOX15B	chr19	28262222	28680333	2.05912	4.31015	7.24665	0.0	0.0	0.0	0.0	0.0	0.089882

XLOC_064908	GUCY2D;HES7;TMEM107;VAMP2;ALOX12B;RANGRF;ODF4;AURKB;ARHGEF15;C19H17orf59;SLC25A35;PFAS;RPL26;NDEL1;ALOX15B	chr19	28262222	28680333	2.75534	9.0483	4.30207	0.0	0.0	0.282607	0.0	0.0	0.0
XLOC_064909	GUCY2D;HES7;TMEM107;VAMP2;ALOX12B;RANGRF;ODF4;AURKB;ARHGEF15;C19H17orf59;SLC25A35;PFAS;RPL26;NDEL1;ALOX15B	chr19	28262222	28680333	8.42301	6.29017	4.93492	0.0	0.0	0.0	0.0	0.0	0.0
XLOC_064910	GUCY2D;HES7;TMEM107;VAMP2;ALOX12B;RANGRF;ODF4;AURKB;ARHGEF15;C19H17orf59;SLC25A35;PFAS;RPL26;NDEL1;ALOX15B	chr19	28262222	28680333	3.89501	11.6179	12.1515	0.348292	0.0	0.0	0.0	0.0	0.336736

XLOC_064911	GUCY2D;HES7;TMEM107;VAMP2;ALOX12B;RANGRF;ODF4;AURKB;ARHGEF15;C19H17orf59;SLC25A35;PFAS;RPL26;NDEL1;ALOX15B	chr19	28262222	28680333	3.57761	5.3482	8.39235	0.0	0.092995	0.0	0.0	0.0	0.0
XLOC_064912	GUCY2D;HES7;TMEM107;VAMP2;ALOX12B;RANGRF;ODF4;AURKB;ARHGEF15;C19H17orf59;SLC25A35;PFAS;RPL26;NDEL1;ALOX15B	chr19	28262222	28680333	3.23986	6.29363	15.1933	0.145077	0.0	0.167301	0.0	0.0	0.0
XLOC_064913	GUCY2D;HES7;TMEM107;VAMP2;ALOX12B;RANGRF;ODF4;AURKB;ARHGEF15;C19H17orf59;SLC25A35;PFAS;RPL26;NDEL1;ALOX15B	chr19	28262222	28680333	7.79142	11.6318	12.1671	0.0	0.0	0.0	0.0	0.0	0.0

XLOC_064914	GUCY2D;HES7;TMEM107;VAMP2;ALOX12B;RANGRF;ODF4;AURKB;ARHGEF15;C19H17orf59;SLC25A35;PFAS;RPL26;NDEL1;ALOX15B	chr19	28262222	28680333	29.8788	8.92203	11.6659	0.0	0.192171	0.255782	0.0	0.0	0.216228
XLOC_064915	GUCY2D;HES7;TMEM107;VAMP2;ALOX12B;RANGRF;ODF4;AURKB;ARHGEF15;C19H17orf59;SLC25A35;PFAS;RPL26;NDEL1;ALOX15B	chr19	28262222	28680333	1.43084	2.13847	13.4224	0.0	0.0	0.0	0.0	0.0	0.0
XLOC_064916	GUCY2D;HES7;TMEM107;VAMP2;ALOX12B;RANGRF;ODF4;AURKB;ARHGEF15;C19H17orf59;SLC25A35;PFAS;RPL26;NDEL1;ALOX15B	chr19	28262222	28680333	12.3341	7.37121	13.7695	0.0	0.0	0.0	0.0	0.0	0.0

XLOC_064917	GUCY2D;HES7;TMEM107;VAMP2;ALOX12B;RANGRF;ODF4;AURKB;ARHGEF15;C19H17orf59;SLC25A35;PFAS;RPL26;NDEL1;ALOX15B	chr19	28262222	28680333	3.12956	6.17893	9.30455	0.0561136	0.0	0.0650426	0.0	0.0627911	0.0547237
XLOC_064918	GUCY2D;HES7;TMEM107;VAMP2;ALOX12B;RANGRF;ODF4;AURKB;ARHGEF15;C19H17orf59;SLC25A35;PFAS;RPL26;NDEL1;ALOX15B	chr19	28262222	28680333	7.07985	7.7526	3.68616	0.0	0.0	0.485243	0.0	0.0	0.0
XLOC_064919	GUCY2D;HES7;TMEM107;VAMP2;ALOX12B;RANGRF;ODF4;AURKB;ARHGEF15;C19H17orf59;SLC25A35;PFAS;RPL26;NDEL1;ALOX15B	chr19	28262222	28680333	0.0	8.95804	5.40608	0.0	0.0	0.0	0.0	0.0	0.0

XLOC_064920	GUCY2D;HES7;TMEM107;VAMP2;ALOX12B;RANGRF;ODF4;AURKB;ARHGEF15;C19H17orf59;SLC25A35;PFAS;RPL26;NDEL1;ALOX15B	chr19	28262222	28680333	5.37745	5.467	10.9338	0.0	0.0	0.111449	0.0	0.0	0.0
XLOC_064921	GUCY2D;HES7;TMEM107;VAMP2;ALOX12B;RANGRF;ODF4;AURKB;ARHGEF15;C19H17orf59;SLC25A35;PFAS;RPL26;NDEL1;ALOX15B	chr19	28262222	28680333	8.09459	4.11577	14.5636	0.0	0.063317	0.0	0.0	0.0	0.0
XLOC_064922	GUCY2D;HES7;TMEM107;VAMP2;ALOX12B;RANGRF;ODF4;AURKB;ARHGEF15;C19H17orf59;SLC25A35;PFAS;RPL26;NDEL1;ALOX15B	chr19	28262222	28680333	12.1273	7.24781	5.4156	0.0	0.0	0.0	0.0	0.0	0.0

XLOC_064923	GUCY2D;HES7;TMEM107;VAMP2;ALOX12B;RANGRF;ODF4;AURKB;ARHGEF15;C19H17orf59;SLC25A35;PFAS;RPL26;NDEL1;ALOX15B	chr19	28262222	28680333	2.06369	3.08239	19.3462	0.0	0.0	0.0	0.0	0.0	0.0
XLOC_064924	GUCY2D;HES7;TMEM107;VAMP2;ALOX12B;RANGRF;ODF4;AURKB;ARHGEF15;C19H17orf59;SLC25A35;PFAS;RPL26;NDEL1;ALOX15B	chr19	28262222	28680333	4.91218	13.2017	1.91798	0.0	0.0	0.0	0.0	0.0	0.0
XLOC_064925	GUCY2D;HES7;TMEM107;VAMP2;ALOX12B;RANGRF;ODF4;AURKB;ARHGEF15;C19H17orf59;SLC25A35;PFAS;RPL26;NDEL1;ALOX15B	chr19	28262222	28680333	29.5754	12.1313	8.65128	0.0	0.0	0.0	0.0	0.0	0.0

XLOC_064926	GUCY2D;HES7;TMEM107;VAMP2;ALOX12B;RANGRF;ODF4;AURKB;ARHGEF15;C19H17orf59;SLC25A35;PFAS;RPL26;NDEL1;ALOX15B	chr19	28262222	28680333	9.62824	12.9388	18.7979	0.2154	0.185897	0.0	0.212228	0.0	0.0
XLOC_064927	GUCY2D;HES7;TMEM107;VAMP2;ALOX12B;RANGRF;ODF4;AURKB;ARHGEF15;C19H17orf59;SLC25A35;PFAS;RPL26;NDEL1;ALOX15B	chr19	28262222	28680333	1.80152	6.99788	22.5242	0.161309	0.1398	0.0	0.0	0.0	0.0
XLOC_064928	GUCY2D;HES7;TMEM107;VAMP2;ALOX12B;RANGRF;ODF4;AURKB;ARHGEF15;C19H17orf59;SLC25A35;PFAS;RPL26;NDEL1;ALOX15B	chr19	28262222	28680333	10.0882	7.23296	7.8814	0.361239	0.156301	0.0	0.0	0.0	0.0

XLOC_064929	GUCY2D;HES7;TMEM107;VAMP2;ALOX12B;RANGRF;ODF4;AURKB;ARHGEF15;C19H17orf59;SLC25A35;PFAS;RPL26;NDEL1;ALOX15B	chr19	28262222	28680333	6.56576	6.53709	8.54769	0.0	0.0	0.0	0.0	0.0	0.0
XLOC_064930	GUCY2D;HES7;TMEM107;VAMP2;ALOX12B;RANGRF;ODF4;AURKB;ARHGEF15;C19H17orf59;SLC25A35;PFAS;RPL26;NDEL1;ALOX15B	chr19	28262222	28680333	6.43592	4.49274	5.03594	0.0	0.0420447	0.0	0.0489342	0.0	0.187626
XLOC_064931	GUCY2D;HES7;TMEM107;VAMP2;ALOX12B;RANGRF;ODF4;AURKB;ARHGEF15;C19H17orf59;SLC25A35;PFAS;RPL26;NDEL1;ALOX15B	chr19	28262222	28680333	5.34745	19.1292	12.5038	0.0	0.0	0.0	0.0	0.0	0.0

XLOC_064932	GUCY2D;HES7;TMEM107;VAMP2;ALOX12B;RANGRF;ODF4;AURKB;ARHGEF15;C19H17orf59;SLC25A35;PFAS;RPL26;NDEL1;ALOX15B	chr19	28262222	28680333	9.74929	2.91478	14.2933	0.109186	0.0	0.0	0.0	0.0	0.106318
XLOC_064933	GUCY2D;HES7;TMEM107;VAMP2;ALOX12B;RANGRF;ODF4;AURKB;ARHGEF15;C19H17orf59;SLC25A35;PFAS;RPL26;NDEL1;ALOX15B	chr19	28262222	28680333	3.40155	4.06937	7.98222	0.0	0.0	0.0	0.0	0.0	0.0
XLOC_064934	GUCY2D;HES7;TMEM107;VAMP2;ALOX12B;RANGRF;ODF4;AURKB;ARHGEF15;C19H17orf59;SLC25A35;PFAS;RPL26;NDEL1;ALOX15B	chr19	28262222	28680333	6.18714	7.76898	17.4157	0.110866	0.0	0.0	0.0	0.0	0.0

XLOC_064935	GUCY2D;HES7;TMEM107;VAMP2;ALOX12B;RANGRF;ODF4;AURKB;ARHGEF15;C19H17orf59;SLC25A35;PFAS;RPL26;NDEL1;ALOX15B	chr19	28262222	28680333	7.49549	6.71458	19.5102	0.0	0.0	0.0	0.0	0.0	0.0
XLOC_064936	GUCY2D;HES7;TMEM107;VAMP2;ALOX12B;RANGRF;ODF4;AURKB;ARHGEF15;C19H17orf59;SLC25A35;PFAS;RPL26;NDEL1;ALOX15B	chr19	28262222	28680333	7.92589	4.57294	9.30241	0.0	0.0887417	0.0588528	0.0	0.0	0.0495082
XLOC_064937	GUCY2D;HES7;TMEM107;VAMP2;ALOX12B;RANGRF;ODF4;AURKB;ARHGEF15;C19H17orf59;SLC25A35;PFAS;RPL26;NDEL1;ALOX15B	chr19	28262222	28680333	14.4321	10.2903	18.231	0.0994765	0.0866285	0.0	0.100219	0.22142	0.0968901

XLOC_064938	GUCY2D;HES7;TMEM107;VAMP2;ALOX12B;RANGRF;ODF4;AURKB;ARHGEF15;C19H17orf59;SLC25A35;PFAS;RPL26;NDEL1;ALOX15B	chr19	28262222	28680333	6.81558	7.12848	17.311	0.0	0.0	0.0	0.0	0.0	0.0
XLOC_064939	GUCY2D;HES7;TMEM107;VAMP2;ALOX12B;RANGRF;ODF4;AURKB;ARHGEF15;C19H17orf59;SLC25A35;PFAS;RPL26;NDEL1;ALOX15B	chr19	28262222	28680333	2.05198	6.12987	16.0306	0.0	0.0	0.0	0.0	0.0	0.0
XLOC_064940	GUCY2D;HES7;TMEM107;VAMP2;ALOX12B;RANGRF;ODF4;AURKB;ARHGEF15;C19H17orf59;SLC25A35;PFAS;RPL26;NDEL1;ALOX15B	chr19	28262222	28680333	15.6673	7.80284	16.3249	0.0	0.0	0.0	0.0	0.0	0.151582

XLOC_064941	GUCY2D;HES7;TMEM107;VAMP2;ALOX12B;RANGRF;ODF4;AURKB;ARHGEF15;C19H17orf59;SLC25A35;PFAS;RPL26;NDEL1;ALOX15B	chr19	28262222	28680333	11.0337	5.81613	9.8671	0.141327	0.0823861	0.0	0.0479485	0.10555	0.137862
XLOC_064942	GUCY2D;HES7;TMEM107;VAMP2;ALOX12B;RANGRF;ODF4;AURKB;ARHGEF15;C19H17orf59;SLC25A35;PFAS;RPL26;NDEL1;ALOX15B	chr19	28262222	28680333	0.0	7.2828	38.0843	0.0	0.0	0.0	0.0	0.0	0.0
XLOC_064943	GUCY2D;HES7;TMEM107;VAMP2;ALOX12B;RANGRF;ODF4;AURKB;ARHGEF15;C19H17orf59;SLC25A35;PFAS;RPL26;NDEL1;ALOX15B	chr19	28262222	28680333	7.0762	7.97088	7.81785	0.0373251	0.0	0.0433116	0.0	0.0	0.0
XLOC_092141	GUCY2F	chrX	62283006	62292084	7.34128	16.741	8.30102	1.72675	1.13298	1.79441	0.335816	0.492477	0.749708
XLOC_038020	GYG1	chr1	120113283	120188226	0.879653	7.14846	7.74618	9.59789	7.94551	11.4707	6.78705	11.1173	9.37942
XLOC_038510	GYG1	chr1	120113283	120188226	155.27	157.437	143.921	252.578	269.088	270.05	277.752	290.978	309.703
XLOC_059577	GYPB	chr17	14279510	14404764	29.2011	22.5945	20.7459	0.222216	0.166949	0.170961	0.306524	0.165393	0.359413
XLOC_065712	GYPC	chr2	79255425	79327024	4.49823	1.44917	1.35638	0.101718	0.0445441	0.117995	0.103451	0.0611256	0.0176675
XLOC_076460	H1F0;GCAT	chr5	110123888	110127912	2.77386	4.14588	3.25279	5.34236	4.96734	6.02468	3.48793	5.10023	7.73911
XLOC_083662	H2AFY	chr7	48445907	48456283	6.98768	4.77931	1.56253	0.0	0.0	0.0	0.0	0.0	0.0

XLOC_053209	H3F3C;PRKDC	chr14	20929330	21039402	73.9152	72.5725	76.1813	16.7447	21.3671	19.0817	20.4481	22.374	25.1152
XLOC_053497	H3F3C;PRKDC	chr14	20929330	21039402	7.93041	3.14796	5.07992	3.0447	4.56375	4.43965	3.36388	2.81337	3.9151
XLOC_053938	H3F3C;PRKDC	chr14	20929330	21039402	4.9718	8.91934	9.33082	5.25657	6.98852	8.1429	5.57634	4.16996	6.51032
XLOC_053939	H3F3C;PRKDC	chr14	20929330	21039402	5.66414	5.91638	6.63021	4.05167	3.92231	4.64346	3.71587	3.59463	6.87519
XLOC_053940	H3F3C;PRKDC	chr14	20929330	21039402	3.09403	3.39179	4.83832	3.14137	4.10574	4.06109	2.33028	2.57296	3.33036
XLOC_053941	H3F3C;PRKDC	chr14	20929330	21039402	8.8047	13.1378	2.2911	6.56115	6.77028	6.31305	6.40744	5.72503	5.59699
XLOC_053942	H3F3C;PRKDC	chr14	20929330	21039402	6.69281	4.33567	5.23332	2.89815	3.7421	3.11972	2.01346	3.22525	4.18547
XLOC_089376	HACE1	chr9	45838550	45862749	5.14738	5.32004	6.03324	8.98794	7.8086	8.3687	6.36269	6.93642	6.22429
XLOC_089870	HACE1	chr9	45766840	45828676	19.29	11.4353	18.6166	20.4626	15.7322	19.9682	18.2693	17.3355	13.0242
XLOC_090722	HACE1	chr9	45766273	45766728	0.0	1.4521	0.0	0.652702	0.375472	0.0	0.214275	0.0	0.0
XLOC_090723	HACE1	chr9	45766840	45828676	0.0	2.81761	2.94743	2.53301	1.75517	2.72255	3.18942	2.23593	1.96974
XLOC_090724	HACE1	chr9	45766840	45828676	1.0194	0.304834	0.797247	1.00488	1.11446	0.63404	0.36872	1.22132	0.444992
XLOC_044232	HADHB	chr11	73199462	73217897	0.885801	0.529835	0.0	0.873304	0.969444	0.643304	0.481784	0.0	0.232117
XLOC_046547	HADHB	chr11	73223527	73224505	0.0	0.0	0.689485	0.948055	0.620197	1.006	0.399561	0.705218	0.384982
XLOC_062433	HAMP;MAG;USF2	chr18	46167265	46198821	2.35504	1.2013	1.05751	0.493349	0.35532	0.598474	0.433301	0.373884	0.341323
XLOC_083051	HAND1	chr7	67647275	67726645	4.42997	3.84841	3.62586	7.55918	11.9828	8.76954	8.36403	7.11835	9.36725
XLOC_085022	HAND1	chr7	67647275	67726645	0.0	0.761833	1.9922	7.07684	4.13323	4.716	4.48748	4.75067	4.20884
XLOC_050548	HAO1	chr13	51100899	51153591	23.8469	27.0538	25.2535	26.9212	21.5606	27.9067	27.5271	32.1714	21.2197
XLOC_050549	HAO1	chr13	51171552	51175022	4.62929	0.0	9.0389	0.207147	0.715556	1.42817	0.0	0.227476	0.0
XLOC_051827	HAO1	chr13	51100899	51153591	9.41197	6.55414	4.89675	3.64778	5.30083	6.1006	2.1848	3.05375	2.17485
XLOC_051828	HAO1	chr13	51100899	51153591	0.0	1.94406	10.1652	2.33898	6.37976	0.0	5.95027	1.8443	4.48757
XLOC_051829	HAO1	chr13	51154506	51155090	1.6959	0.506793	2.65076	0.911216	0.790294	1.05039	1.66607	1.84711	0.738479
XLOC_051830	HAO1	chr13	51155605	51156130	0.0	0.588012	0.0	1.40964	1.67786	0.608499	0.699651	0.776954	0.0
XLOC_053200	HAS2	chr14	19708666	19779738	30.3856	32.3453	28.1186	111.212	112.827	108.058	96.1963	117.183	99.217
XLOC_062406	HAS3	chr18	36403847	36454671	37.4701	37.1245	26.8691	90.2646	79.8872	90.7669	92.6681	109.819	97.8157
XLOC_066164	HAT1	chr2	24605643	24616849	0.711311	1.06422	1.67007	0.73356	0.502595	0.666376	0.227917	0.250588	0.217881
XLOC_072637	HBP1	chr4	48539856	48561738	14.2597	23.3097	16.2849	60.2373	56.4278	58.6602	57.0221	69.1075	72.4216
XLOC_074201	HBP1	chr4	48561935	48562328	3.07083	0.916513	2.39662	6.86656	7.55064	10.0607	6.95806	8.07653	8.5169
XLOC_074202	HBP1	chr4	48562481	48562986	0.0	0.62179	3.25215	4.28549	5.80203	5.35897	2.39982	4.30789	5.79278

XLOC_074203	HBP1	chr4	48563074	48563304	0.0	5.68313	0.0	16.3349	14.1665	14.3297	10.7564	20.2577	21.2473
XLOC_074204	HBP1	chr4	48563473	48564314	1.05818	1.26567	0.827542	9.4824	12.6389	9.76036	7.74508	10.0314	14.9641
XLOC_074205	HBP1	chr4	48564414	48565728	0.625371	0.561235	0.97858	6.72781	7.69143	6.82359	5.12677	6.14825	9.29499
XLOC_037895	HCLS1;FBXO40	chr1	66700501	66817461	3.17095	1.91034	2.1198	10.0186	6.99393	8.79188	9.69688	9.46811	9.86277
XLOC_038396	HCLS1;FBXO40	chr1	66700501	66817461	23.6093	20.7257	27.0948	6.69613	8.47517	7.08134	10.6629	4.87017	12.8898
XLOC_039577	HCLS1;FBXO40	chr1	66700501	66817461	2.86752	2.32884	2.56464	1.79994	2.76462	2.51497	2.8473	2.3487	3.79943
XLOC_039578	HCLS1;FBXO40	chr1	66700501	66817461	1.92093	2.41406	2.10464	1.10244	1.50775	1.43942	1.86309	0.811787	2.824
XLOC_039579	HCLS1;FBXO40	chr1	66700501	66817461	8.13853	6.06816	12.6936	4.36653	4.0361	3.31567	4.88394	1.96044	9.14237
XLOC_039580	HCLS1;FBXO40	chr1	66700501	66817461	0.0	2.54052	0.0	0.0	0.649286	0.867007	0.0	0.0	0.367887
XLOC_039581	HCLS1;FBXO40	chr1	66700501	66817461	2.93404	1.75157	2.29012	1.0498	1.12849	1.20258	1.53786	0.572544	2.7985
XLOC_039582	HCLS1;FBXO40	chr1	66700501	66817461	4.03695	2.81584	2.10406	0.964386	1.0481	0.835306	1.33051	0.802865	3.28556
XLOC_039583	HCLS1;FBXO40	chr1	66700501	66817461	0.0	1.98535	2.59606	0.743688	1.54841	0.514508	0.890596	0.658096	1.59128
XLOC_039584	HCLS1;FBXO40	chr1	66700501	66817461	5.68647	1.27572	0.556085	1.3381	1.55807	1.32888	2.00397	1.49591	1.80168
XLOC_039585	HCLS1;FBXO40	chr1	66700501	66817461	0.0	2.59031	1.50546	0.603767	0.977498	0.798452	1.48106	0.961525	1.59679
XLOC_039586	HCLS1;FBXO40	chr1	66700501	66817461	0.0	0.591237	3.0923	0.177177	0.0	0.203997	0.351721	0.195304	0.688688
XLOC_039587	HCLS1;FBXO40	chr1	66700501	66817461	0.762531	1.82461	4.77207	0.751863	1.7304	2.21681	1.4549	0.305478	2.19892
XLOC_039588	HCLS1;FBXO40	chr1	66700501	66817461	2.73052	1.42937	3.20434	0.550755	1.12249	1.06369	1.3044	0.479041	1.37241
XLOC_039589	HCLS1;FBXO40	chr1	66700501	66817461	1.96407	1.17468	2.30414	0.440039	0.920542	0.814615	1.06637	0.294272	0.685977
XLOC_039590	HCLS1;FBXO40	chr1	66700501	66817461	0.371367	1.44456	1.45316	0.370966	0.787098	0.463836	1.08069	0.261609	1.36605
XLOC_039591	HCLS1;FBXO40	chr1	66700501	66817461	4.1597	2.48772	1.62656	0.745522	1.29913	0.323396	1.31603	0.10381	1.90645
XLOC_039592	HCLS1;FBXO40	chr1	66700501	66817461	1.5548	2.0157	2.02767	0.371746	0.284461	0.53894	0.283825	0.260311	0.362637
XLOC_039593	HCLS1;FBXO40	chr1	66700501	66817461	1.44434	1.5121	1.69489	0.647365	0.452254	0.375024	0.78797	0.217094	0.315584
XLOC_039594	HCLS1;FBXO40	chr1	66700501	66817461	7.62668	2.27498	2.9743	1.02298	1.45734	1.16672	1.64128	0.0	0.659498

XLOC_062226	HCST;TYROBP;APLP1;NFKBID	chr18	46754202	46790971	6.81913	2.87467	3.33375	0.381986	1.05543	0.715569	0.833986	0.0852592	0.735284
XLOC_062227	HCST;TYROBP;APLP1;NFKBID	chr18	46754202	46790971	10.1879	13.3159	9.52594	4.93097	4.46279	5.31162	3.79317	5.44728	5.30031
XLOC_063115	HCST;TYROBP;APLP1;NFKBID	chr18	46754202	46790971	3.18283	0.951233	3.73156	0.570111	0.123703	0.164385	0.427202	0.473319	0.415948
XLOC_063116	HCST;TYROBP;APLP1;NFKBID	chr18	46754202	46790971	5.05378	1.50662	0.0	0.0	0.0	1.53758	0.426988	0.0	0.435704
XLOC_063117	HCST;TYROBP;APLP1;NFKBID	chr18	46754202	46790971	2.43443	1.69963	1.27009	0.181916	0.413932	0.379988	0.296878	0.163256	0.319522
XLOC_063118	HCST;TYROBP;APLP1;NFKBID	chr18	46754202	46790971	2.1429	1.92351	3.6893	0.345875	0.605292	0.401323	0.470187	0.387897	0.449976
XLOC_063119	HCST;TYROBP;APLP1;NFKBID	chr18	46754202	46790971	2.79008	1.5504	1.87156	0.178709	0.656905	0.539216	0.364601	0.441076	0.244141
XLOC_089337	HDAC2;MARCKS	chr9	36890400	37147309	0.0	0.0	0.0	0.827125	0.812393	0.75084	1.3775	1.04921	1.2779
XLOC_089826	HDAC2;MARCKS	chr9	36890400	37147309	0.815334	1.66704	2.42883	1.37577	1.1933	1.95964	1.91897	1.9784	4.78277
XLOC_064013	HDAC5	chr19	44562855	44571415	14.8167	18.2837	15.4569	11.4016	11.898	12.3824	13.4421	15.485	9.33818
XLOC_064286	HDAC5	chr19	44550227	44555477	94.9184	99.1296	64.3188	195.928	181.287	184.699	195.036	197.394	216.699
XLOC_065213	HDAC5	chr19	44548280	44548864	0.0	0.0	0.0	0.759347	0.658578	0.0	0.757307	0.335838	0.590783
XLOC_065214	HDAC5	chr19	44549264	44549663	0.0	0.893835	0.0	1.87503	1.84189	1.84038	1.30661	1.45983	1.29808
XLOC_065215	HDAC5	chr19	44571528	44576034	6.65255	9.30789	9.24327	7.89128	7.4016	6.72497	6.57029	7.18161	4.69003
XLOC_064285	HDAC5;G6PC3;LSM12	chr19	44527016	44543813	0.0	0.444785	0.0	0.532783	1.2354	0.740932	0.777014	0.433415	1.23362
XLOC_064192	HEATR6	chr19	14448068	14452491	21.8988	25.5325	21.8659	33.7902	21.8127	26.5249	32.8582	43.2365	27.2916
XLOC_064193	HEATR6	chr19	14452564	14597510	45.4595	50.8759	38.1606	64.6924	43.3543	53.068	59.6998	75.4681	51.0901
XLOC_064630	HEATR6	chr19	14447012	14447691	0.0	2.07294	1.08426	2.36058	3.13158	2.8689	2.74052	2.34335	3.14401
XLOC_064631	HEATR6	chr19	14452564	14597510	3.21691	0.641479	0.83859	1.72974	2.17633	2.11132	1.64682	1.39087	1.5912
XLOC_064632	HEATR6	chr19	14452564	14597510	3.02457	3.16651	2.3661	2.50796	2.89952	2.5124	1.92387	1.59047	2.51098
XLOC_064633	HEATR6	chr19	14452564	14597510	6.64849	0.568254	2.97144	2.04303	3.33955	4.137	2.14992	1.80323	1.65885
XLOC_064634	HEATR6	chr19	14452564	14597510	4.91014	3.81787	4.6083	3.16839	3.22189	3.97111	3.90996	2.55031	3.85864
XLOC_064635	HEATR6	chr19	14452564	14597510	1.90411	2.08867	3.47611	2.44684	2.48594	2.57216	2.94803	1.91002	2.60805

XLOC_064636	HEATR6	chr19	14452564	14597510	2.9406	4.79081	4.09147	3.66269	3.2072	3.0955	2.42417	2.33585	3.28873
XLOC_076924	HEBP1	chr5	97384195	97392380	2.27881	1.36348	0.0	7.50838	6.25089	5.80379	4.67525	5.09008	5.62961
XLOC_078390	HEBP1	chr5	97382156	97384048	1.66787	0.499041	0.652614	6.13191	6.74015	6.29101	5.75744	5.95517	8.1358
XLOC_080071	HERC3	chr6	37603454	37607909	0.0	0.0	0.0	0.0	0.0	0.241058	0.0	0.0	0.101548
XLOC_080803	HERC3	chr6	37542225	37542332	0.0	2730.39	0.0	1279.71	0.0	853.226	1000.97	1588.14	1518.06
XLOC_080804	HERC3	chr6	37603454	37607909	0.0	0.0	0.0	0.335117	0.435436	0.578928	0.0	0.184905	0.325759
XLOC_080805	HERC3	chr6	37613585	37614692	0.0	0.0	0.0	0.0682788	0.0596054	0.0	0.0	0.152574	0.0
XLOC_080808	HERC3	chr6	37623214	37623735	0.0	0.0	0.0	0.0	0.0	0.205038	0.0	0.0	0.173113
XLOC_080814	HERC5	chr6	37686003	37687200	41.05	44.9543	41.913	135.034	99.4685	120.618	126.08	170.441	105.454
XLOC_080815	HERC5	chr6	37689138	37690086	0.0	0.0	0.0	0.410001	0.92959	0.664343	1.3263	0.365841	1.19863
XLOC_080816	HERC5	chr6	37690207	37691016	0.0	0.0	0.0	1.49215	0.433142	1.15017	0.100219	0.664261	0.29067
XLOC_079653	HERC6;PPM1K;ABCG2	chr6	37790422	37966533	46.5026	67.8503	50.0902	82.0053	69.0755	80.2989	88.46	103.149	83.6453
XLOC_080817	HERC6;PPM1K;ABCG2	chr6	37790422	37966533	9.48523	7.09331	9.54107	15.7914	13.4222	15.6248	20.0375	18.3396	10.8972
XLOC_080818	HERC6;PPM1K;ABCG2	chr6	37790422	37966533	0.0	0.0	0.0	0.554281	1.32336	0.319724	0.692762	0.613877	0.404443
XLOC_080819	HERC6;PPM1K;ABCG2	chr6	37790422	37966533	0.0	1.11135	1.45337	2.91421	2.68624	2.2163	1.93544	3.2499	1.94728
XLOC_080820	HERC6;PPM1K;ABCG2	chr6	37790422	37966533	5.93482	7.98235	18.5565	14.7512	11.1965	10.1217	17.5589	11.4882	15.9055
XLOC_080821	HERC6;PPM1K;ABCG2	chr6	37790422	37966533	0.0	23.7963	20.7349	8.46425	14.6456	5.30127	23.0112	14.476	11.3719
XLOC_080822	HERC6;PPM1K;ABCG2	chr6	37790422	37966533	5.12465	9.1656	11.9827	7.79452	7.37937	8.31252	9.51543	9.77343	7.50906
XLOC_037905	HES1	chr1	73910083	74148528	1.18601	1.19265	0.891245	0.0353919	0.0619542	0.0	0.0	0.0860448	0.0747614
XLOC_038407	HES1	chr1	73910083	74148528	38.6122	14.4684	17.5468	6.22599	4.24068	5.76816	5.30499	9.66647	7.02386
XLOC_039663	HES1	chr1	73910083	74148528	3.24093	0.646056	1.68966	0.0	0.0	0.223892	0.0	0.0	0.0942955
XLOC_064020	HEXIM2;ACBD4;HEXIM1;PLCD3;NMT1;DCAKD	chr19	45363466	45532068	7.14706	9.21306	7.07157	17.2664	17.6576	15.7078	15.943	19.788	18.0431
XLOC_059576	HHIP	chr17	13727837	13906029	27.0938	30.799	32.8555	39.0683	35.9623	37.9697	36.8258	38.6451	33.9172
XLOC_059971	HHIP	chr17	13727837	13906029	2.19469	1.99444	4.0652	1.1687	1.38733	1.21619	1.38838	0.732199	1.04395
XLOC_060609	HHIP	chr17	13661882	13662725	0.0	0.0	0.82513	0.189095	0.164736	0.546746	0.0	0.0	0.36841
XLOC_060610	HHIP	chr17	13667669	13667963	0.0	0.0	0.0	4.71711	1.99591	3.20473	2.66413	0.502007	1.81695
XLOC_060611	HHIP	chr17	13682254	13683538	0.0	0.76823	2.51156	1.03603	1.10634	0.733815	0.818483	0.515159	0.67355

XLOC_060612	HHIP	chr17	13711942	13712913	0.0	1.32947	2.08624	0.637471	0.555996	0.276713	0.0805886	0.0889025	0.155313
XLOC_060613	HHIP	chr17	13716724	13717524	2.25142	0.673178	1.76059	1.21042	0.702649	0.466468	0.304782	1.01016	0.589451
XLOC_065471	HIBCH;HIBCH	chr2	5951475	5957650	48.5404	32.3032	15.1858	24.8837	24.133	40.1546	32.8797	23.685	24.0555
XLOC_065472	HIBCH;HIBCH	chr2	5957810	6034299	29.3703	33.1908	29.5884	18.6905	20.5768	20.6729	24.5264	20.8759	21.9076
XLOC_066126	HIBCH;HIBCH;INPP1	chr2	5898131	5942806	2.97002	3.83046	2.9008	7.17702	7.02898	5.27453	9.81143	9.36385	9.77909
XLOC_066809	HIBCH;HIBCH;INPP1	chr2	5898131	5942806	1.6309	0.0	0.0	2.62903	2.15444	2.69476	0.874972	0.969655	1.42067
XLOC_066125	HIBCH;INPP1	chr2	5869643	5895393	12.5591	11.4371	13.1507	18.6431	26.2604	24.1526	21.8168	15.6644	22.6507
XLOC_066800	HIBCH;INPP1	chr2	5869643	5895393	3.50084	5.22251	8.19375	6.26126	6.70378	5.72127	6.96634	2.37562	6.36332
XLOC_066801	HIBCH;INPP1	chr2	5869643	5895393	7.63839	7.13834	4.48067	5.90427	8.95181	6.73312	8.29839	4.67452	7.83758
XLOC_066802	HIBCH;INPP1	chr2	5869643	5895393	4.25046	2.54106	5.53801	5.07657	4.30116	3.66272	3.05241	2.25221	2.7174
XLOC_066803	HIBCH;INPP1	chr2	5869643	5895393	12.4158	3.70279	19.3642	5.18116	6.31207	12.2196	11.341	6.77404	7.86639
XLOC_066804	HIBCH;INPP1	chr2	5869643	5895393	0.0	1.84729	14.4892	2.22133	1.86823	4.38239	4.12765	4.09952	5.8661
XLOC_066805	HIBCH;INPP1	chr2	5869643	5895393	3.00433	7.6327	2.34845	3.22913	2.22014	4.03655	4.71219	2.83265	3.40385
XLOC_066806	HIBCH;INPP1	chr2	5869643	5895393	4.19928	5.01756	9.84129	6.57807	8.28994	7.78326	9.49357	4.34446	6.39069
XLOC_066807	HIBCH;INPP1	chr2	5896204	5896937	0.0	1.87865	1.9653	2.92753	3.52635	2.3416	4.07338	3.3779	3.72735
XLOC_066808	HIBCH;INPP1	chr2	5897076	5897493	2.78717	0.832051	0.0	5.98411	4.9363	7.71711	8.54334	8.1717	10.6391
XLOC_055787	HIPK3	chr15	64699144	64922358	0.817528	0.0611139	0.159846	0.0183151	0.0	0.0212762	0.0	0.0	0.0
XLOC_063856	HLF	chr19	5689503	5702898	22.1554	26.6751	26.772	6.95173	6.61489	5.91051	6.95226	7.59872	6.66651
XLOC_063857	HLF	chr19	5706351	5707103	0.0	0.0	0.0	0.543372	1.56731	1.66749	0.898133	0.399079	0.879985
XLOC_063858	HLF	chr19	5742413	5747852	11.0279	6.59798	5.27284	0.0570207	0.720024	0.330599	0.279097	0.0614713	0.33379
XLOC_064119	HLF	chr19	5707877	5726329	6.22563	3.62659	4.96314	2.20263	3.08235	1.98029	3.65929	2.57987	3.30643
XLOC_064120	HLF	chr19	5728092	5731771	13.3967	13.0496	9.48171	22.3793	13.1056	16.7949	16.2541	32.514	14.8364
XLOC_064440	HLF	chr19	5741920	5742315	0.0	2.72648	2.37653	0.272357	0.234019	0.0	0.0	0.0	0.0
XLOC_038509	HLTF	chr1	120067517	120088307	3.21205	0.960397	1.67457	9.51105	7.70526	9.65595	5.24239	7.48831	10.472
XLOC_040165	HLTF	chr1	120067517	120088307	1.45972	0.0	0.0	0.784505	1.13586	1.05633	0.0	0.869695	0.636179
XLOC_058706	HLX	chr16	25058113	25059820	1.40026	0.0	0.365248	0.795183	0.842054	0.67972	0.511699	0.281497	0.979968

XLOC_068591	HMGCL	chr2	129687236	129688393	0.0	0.0	0.0	0.324328	0.339845	0.300597	0.197377	0.652543	0.0
XLOC_065864	HMGN2	chr2	127242381	127261480	15.805	13.457	15.655	4.47521	4.70942	4.1193	3.74093	4.69637	4.6728
XLOC_066530	HMGN2	chr2	127242381	127261480	15.1379	19.557	15.5966	21.2443	18.0397	18.8629	20.9583	25.9869	23.2216
XLOC_068531	HMGN2	chr2	127242381	127261480	1.74349	2.78177	3.18298	1.3026	1.04748	0.664516	0.688749	0.408369	0.50819
XLOC_083625	HMHA1;POLR2E	chr7	45183313	45204618	2.72493	2.83747	2.81312	3.3027	3.85702	2.8772	2.84953	2.82621	2.3413
XLOC_060282	HNF1A;C17H12orf43	chr17	65382454	65436328	10.0635	15.7761	5.89365	0.0	0.0	0.0	0.0	0.0	0.0
XLOC_050628	HNF4A	chr13	73538467	73578665	0.0	0.0	0.312098	0.0	0.125194	0.0	0.0	0.0	0.0348964
XLOC_053556	HNF4G	chr14	40906336	40966877	22.7842	20.0334	18.1709	19.0791	25.3068	23.8175	22.6989	21.2304	29.2362
XLOC_054221	HNF4G	chr14	40906336	40966877	11.2658	6.71676	4.39024	2.0175	1.70305	1.71025	0.944669	0.0	1.94079
XLOC_067555	HNMT	chr2	59382908	59383828	0.0	0.0	0.0	1.0201	0.741129	0.590226	0.687077	0.37912	1.40817
XLOC_067556	HNMT	chr2	59384362	59384839	0.0	0.0	0.0	0.202619	0.700159	0.232886	0.600364	2.22627	0.393522
XLOC_081999	HNRNPD	chr6	98925180	98929990	5.90696	5.11752	5.59735	4.28043	3.92378	3.77482	3.15682	3.16841	2.55945
XLOC_080241	HNRNPD;TMEM150C;HNRPDL;ENOPH1	chr6	98930048	99075901	26.8585	38.7575	32.1968	16.4456	13.9232	16.2488	10.584	11.2311	9.41794
XLOC_082000	HNRNPD;TMEM150C;HNRPDL;ENOPH1	chr6	98930048	99075901	0.0	1.25815	1.64497	2.0734	0.326026	0.650491	0.373376	1.65977	1.28182
XLOC_057876	HNRNPU;COX20	chr16	33170230	33243367	17.4371	24.9825	21.1839	11.1222	11.7082	12.1926	12.2	14.1908	12.6156
XLOC_063158	HNRNPUL1	chr18	50718800	50719370	0.0	0.0	0.0	1.0991	0.408372	1.99037	0.0	0.520492	0.305355
XLOC_063159	HNRNPUL1	chr18	50719768	50720328	0.0	0.0	0.0	0.965455	0.418373	1.85389	0.0	0.355429	0.156446
XLOC_063160	HNRNPUL1	chr18	50721322	50722329	0.0	0.0	0.0	1.22096	0.732317	0.883492	0.386135	0.425871	0.89251
XLOC_063161	HNRNPUL1	chr18	50723346	50723780	0.0	0.0	0.0	1.17024	1.4119	0.268527	0.689367	0.512334	2.4978
XLOC_063162	HNRNPUL1	chr18	50724038	50724851	0.0	0.0	0.0	0.988701	0.688836	1.02888	0.0	0.770306	0.674109
XLOC_063163	HNRNPUL1	chr18	50725695	50726446	0.0	0.0	0.0	0.655119	0.760098	0.630865	0.329383	0.242738	1.16949
XLOC_063164	HNRNPUL1	chr18	50728306	50729708	0.0	0.0	0.0	1.19837	0.728671	1.14775	0.317877	0.408328	0.86391
XLOC_063165	HNRNPUL1	chr18	50730478	50732219	0.0	0.0	0.0	1.22861	0.394106	1.6154	0.33387	0.413231	0.879047
XLOC_063166	HNRNPUL1	chr18	50733124	50733802	0.0	0.0	0.0	0.497917	0.649144	2.01205	0.998418	0.552418	0.726929
XLOC_063167	HNRNPUL1	chr18	50736459	50737083	0.0	0.0	0.0	0.555467	0.482242	1.76216	0.0	0.153791	0.540407
XLOC_063168	HNRNPUL1	chr18	50739170	50739701	0.0	0.0	0.0	0.866897	0.30024	2.19575	0.861006	0.764764	0.842548
XLOC_063169	HNRNPUL1	chr18	50742835	50743402	0.0	0.0	0.0	0.632653	0.274214	1.27578	0.315095	0.174741	1.53789

XLOC_063170	HNRNPUL1	chr18	50745789	50746758	0.0	0.0	0.0	1.11832	0.557357	1.38695	0.40392	0.267358	0.778475
XLOC_063171	HNRNPUL1	chr18	50747271	50748386	0.0	0.0	0.0	1.15105	0.472883	1.09802	0.343189	0.226967	0.99014
XLOC_063172	HNRNPUL1	chr18	50751829	50752508	0.0	0.0	0.0	0.745445	0.431942	2.15167	0.124569	0.551376	0.725542
XLOC_069848	HORMAD1;GOLPH3L	chr3	20057732	20118445	15.4873	18.4156	13.6488	57.1368	58.4878	58.9922	63.7715	50.1575	69.4448
XLOC_060302	HORMAD2	chr17	71344405	71363422	35.3779	44.1596	41.4003	26.7709	26.0591	29.3235	31.6585	24.8984	27.1301
XLOC_061891	HORMAD2	chr17	71343312	71344063	7.31197	1.45739	4.76442	0.655119	1.23516	0.630865	0.548972	0.485477	0.956858
XLOC_060301	HORMAD2;MTMR3	chr17	71251321	71310972	6.48122	1.46178	1.26277	0.692208	0.636654	0.548862	0.70535	1.14421	0.568523
XLOC_066158	HOXD12;KIAA1715	chr2	20847877	20968843	42.3217	37.6625	37.7821	17.9565	17.3992	16.882	18.9597	18.2916	16.1702
XLOC_044986	HPCAL1	chr11	87226833	87358490	11.3903	9.89523	20.5205	1.81772	2.44688	2.92092	2.40922	1.55966	3.43901
XLOC_046909	HPCAL1	chr11	87219797	87220384	0.0	1.50978	2.63227	3.46864	4.9707	3.65095	4.36264	2.16801	5.28014
XLOC_046910	HPCAL1	chr11	87220495	87220682	0.0	22.3843	14.6298	13.7891	10.8687	20.4501	12.9629	30.0309	15.9975
XLOC_046911	HPCAL1	chr11	87225232	87226329	2.30947	0.690776	3.01109	0.966071	1.26495	1.11896	0.48954	0.693824	0.807246
XLOC_046912	HPCAL1	chr11	87226833	87358490	0.942016	3.24121	6.2658	1.05583	1.10831	1.17583	0.602377	0.426074	0.865277
XLOC_046913	HPCAL1	chr11	87226833	87358490	4.42516	2.64774	3.89532	1.14066	0.95386	1.03513	1.10997	0.666491	1.30611
XLOC_046914	HPCAL1	chr11	87226833	87358490	5.60144	0.418596	2.18949	0.501777	0.872155	1.30341	0.754465	0.278312	0.854619
XLOC_046915	HPCAL1	chr11	87226833	87358490	4.88166	2.9178	0.0	0.582927	0.885154	1.0083	1.45509	1.12877	1.55925
XLOC_046916	HPCAL1	chr11	87226833	87358490	1.88964	0.847657	7.38978	0.0	0.0	0.0	0.0	0.0	0.0
XLOC_046917	HPCAL1	chr11	87226833	87358490	0.0	6.51741	1.70439	0.0	0.0	0.224593	0.0	0.214779	0.0
XLOC_046918	HPCAL1	chr11	87226833	87358490	4.46118	5.00217	2.61647	0.19988	0.696206	0.346649	0.503367	0.222431	0.681356
XLOC_080244	HPSE	chr6	99877548	99884498	299.526	255.863	219.552	558.604	687.666	666.494	617.376	547.32	627.96
XLOC_086896	HR;SFTPC;REEP4	chr8	69906864	69996565	72.192	76.2673	71.0111	99.6877	92.2181	101.022	111.415	121.707	62.9754
XLOC_037918	HRASLS	chr1	74771887	74785385	4.69471	6.01049	5.01097	0.497824	0.335082	0.622071	0.117129	0.257683	0.485745
XLOC_037919	HRASLS	chr1	74792023	74804125	0.413628	0.123762	0.0	0.0370904	0.16229	0.129119	0.0	0.0832074	0.0
XLOC_044281	HS1BP3	chr11	78301554	78313910	12.4773	16.7445	13.8943	32.8769	33.7505	32.8592	37.4875	34.9427	37.8604
XLOC_044282	HS1BP3	chr11	78314062	78322217	17.852	19.4502	13.3784	36.0472	38.4366	35.0648	42.0005	36.6302	44.7631
XLOC_063965	HS3ST3A1	chr19	32123069	32389869	0.0	0.0	5.1181	0.87982	0.754794	0.0	0.0	0.0	0.284027
XLOC_064230	HS3ST3A1	chr19	32123069	32389869	51.2088	52.0932	40.2081	25.9817	22.812	25.174	22.7747	22.1114	31.7698
XLOC_064231	HS3ST3A1	chr19	32123069	32389869	3.62109	4.8689	11.3186	3.08019	2.95031	2.98825	0.968173	1.7902	2.67943
XLOC_065008	HS3ST3A1	chr19	32390050	32390945	0.980694	1.46635	3.06803	0.175775	0.612845	0.305057	0.443717	0.587727	0.428157
XLOC_063968	HS3ST3B1;COX10	chr19	32757550	32832823	40.0177	47.1051	41.6565	62.183	46.44	62.7051	55.7213	69.5124	47.8929

XLOC_065012	HS3ST3B1;COX10	chr19	32757550	32832823	0.0	1.58263	4.13793	10.928	5.22566	6.45476	4.46993	3.53817	2.74487
XLOC_065013	HS3ST3B1;COX10	chr19	32757550	32832823	5.81322	4.34606	9.09323	6.94631	8.09874	7.33408	7.01541	6.87191	7.27581
XLOC_065014	HS3ST3B1;COX10	chr19	32757550	32832823	9.77132	5.82624	11.4253	8.30402	9.27047	9.42019	6.20634	5.13639	6.32018
XLOC_065015	HS3ST3B1;COX10	chr19	32757550	32832823	0.0	0.835259	0.0	0.750898	2.15433	1.43453	0.244993	1.91386	0.728167
XLOC_065016	HS3ST3B1;COX10	chr19	32757550	32832823	0.0	1.62053	2.11886	1.21403	0.418212	3.34123	0.951977	3.45108	0.706527
XLOC_065017	HS3ST3B1;COX10	chr19	32757550	32832823	0.0	1.05573	2.76107	1.16005	1.92771	0.853246	0.955109	0.703685	1.84859
XLOC_089336	HS3ST5	chr9	36832750	36878996	0.915752	1.64396	0.716606	6.0841	4.59733	4.19121	6.7656	8.10729	11.1016
XLOC_089824	HS3ST5	chr9	36709095	36767941	55.1896	52.9118	44.9514	119.309	119.61	120.112	131.424	138.178	150.082
XLOC_089825	HS3ST5	chr9	36779126	36797611	19.9163	24.5039	27.1741	89.1814	86.4874	86.4261	76.5892	92.205	98.8095
XLOC_090558	HS3ST5	chr9	36692659	36693130	0.0	0.0	0.0	0.206488	0.356658	0.47456	0.407585	0.45354	2.00496
XLOC_090559	HS3ST5	chr9	36697696	36699055	1.80584	0.180075	0.0	0.323799	0.801788	0.688178	1.09709	0.604048	1.31583
XLOC_090560	HS3ST5	chr9	36703842	36705512	0.0	0.429193	0.0	2.058	2.70023	2.83493	2.4895	2.06646	3.05353
XLOC_090561	HS3ST5	chr9	36706183	36706603	5.51068	1.64514	0.0	1.97197	2.97112	3.6739	2.41443	1.3469	2.39061
XLOC_090562	HS3ST5	chr9	36709095	36767941	6.64945	1.98404	0.0	4.4603	2.04061	2.71984	4.03975	1.93879	5.75784
XLOC_090563	HS3ST5	chr9	36709095	36767941	3.70494	5.5344	7.23673	7.62931	11.7823	9.16951	7.75364	7.87209	8.54477
XLOC_090564	HS3ST5	chr9	36767992	36768776	0.0	0.690352	0.902749	0.827533	0.450268	0.717444	1.2496	0.230132	0.402962
XLOC_090565	HS3ST5	chr9	36813294	36813865	19.2415	29.794	24.6056	78.3179	57.1715	74.1896	119.707	108.006	84.6846
XLOC_092071	HS6ST2	chrX	16618757	16686878	5.41127	2.32125	2.25907	8.78486	8.72136	9.05977	7.63751	4.23944	6.34999
XLOC_092384	HS6ST2	chrX	16709283	16817355	4.84194	2.12503	1.51582	0.0868444	0.202791	0.0336073	0.147847	0.0	0.0282534
XLOC_092789	HS6ST2	chrX	16687447	16687808	0.0	1.0598	0.0	0.952965	2.71988	1.45092	2.14949	0.688324	2.45928
XLOC_047905	HS6ST3	chr12	77178516	77476051	18.6904	20.3604	22.2769	4.89561	5.50424	5.3578	7.70756	4.66051	5.10816
XLOC_047906	HS6ST3	chr12	77553775	77640099	0.0	0.0	0.572967	8.1468	5.24452	5.19913	4.77325	4.01341	5.86637
XLOC_049640	HS6ST3	chr12	77178516	77476051	0.0	1.92243	2.51366	0.0	0.247218	0.658986	0.279984	0.313221	0.279031
XLOC_049641	HS6ST3	chr12	77553775	77640099	0.0	0.278912	0.0	1.25378	0.728799	1.35424	1.52045	0.932093	0.977394
XLOC_049642	HS6ST3	chr12	77553775	77640099	0.0	0.0	0.0	1.30056	0.803565	1.06876	0.920462	0.409129	2.16611
XLOC_049643	HS6ST3	chr12	77553775	77640099	0.0	0.0	0.0	1.68955	1.00146	0.419553	0.551315	0.944798	0.941426
XLOC_049644	HS6ST3	chr12	77553775	77640099	0.0	0.0	0.0	0.874129	0.844884	1.33397	0.42153	0.257735	0.807845
XLOC_049645	HS6ST3	chr12	77553775	77640099	0.0	0.325456	0.0	0.780265	0.424743	1.2406	0.393192	0.325719	0.285008
XLOC_049646	HS6ST3	chr12	77553775	77640099	0.0	0.0	0.0	0.894575	0.486426	1.29198	0.33716	0.248514	0.435501
XLOC_049647	HS6ST3	chr12	77553775	77640099	0.0	0.49407	0.0	1.77669	2.05516	1.53637	2.51205	2.6205	0.864029

XLOC_062356	HSBP1;CDH13	chr18	10064343	10182238	33.3307	34.1339	35.2732	20.2907	20.1807	20.927	24.6329	21.6402	21.8216
XLOC_055488	HSD17B12	chr15	74714717	74717074	0.87297	0.0	0.682833	0.234727	0.1365	0.0	0.0	0.0	0.0
XLOC_055489	HSD17B12	chr15	74721101	74724234	0.546458	2.1253	2.5655	7.88812	5.91129	6.13593	6.13145	5.65202	4.97024
XLOC_055490	HSD17B12	chr15	74821279	74824430	1.88118	2.81029	1.46989	0.168428	0.0	0.193968	0.0	0.0	0.0
XLOC_055802	HSD17B12	chr15	74668991	74672753	14.948	12.4051	14.7622	10.1211	9.12117	8.74071	8.54725	7.77931	8.27571
XLOC_055803	HSD17B12	chr15	74707435	74709543	0.0	2.66246	1.98947	2.96353	1.18978	1.97517	2.06118	3.54547	2.77429
XLOC_055804	HSD17B12	chr15	74732252	74734341	0.0	0.0	0.0	1.79124	0.310049	2.06159	0.710844	0.394769	0.0
XLOC_055805	HSD17B12	chr15	74796844	74799808	1.49286	0.446206	1.16695	2.94173	1.74202	1.85159	3.34558	1.92635	2.73229
XLOC_055806	HSD17B12	chr15	74813466	74817600	0.906906	1.46726	1.91868	0.10992	0.0	0.0	0.0	0.0	0.0
XLOC_056937	HSD17B12	chr15	74651625	74652081	0.0	0.0	0.0	0.650521	0.374238	0.747083	1.06794	2.37835	1.68387
XLOC_056938	HSD17B12	chr15	74704357	74705882	2.1155	3.79744	2.06917	2.18127	2.81948	2.03471	2.31653	3.66527	2.26655
XLOC_056939	HSD17B12	chr15	74707435	74709543	0.0	0.0	4.41951	0.0	0.871624	0.580432	0.743173	0.27651	0.491085
XLOC_056940	HSD17B12	chr15	74749957	74750461	0.0	0.623582	1.63076	0.373725	0.6465	0.21497	0.370229	0.205715	0.181543
XLOC_056941	HSD17B12	chr15	74750511	74751478	0.893461	1.33603	0.698846	0.240232	0.698405	0.278073	0.323921	0.178674	0.390196
XLOC_062615	HSD17B2	chr18	8695767	8696356	13.41	8.01487	7.86027	1.65124	1.43231	1.73057	1.4976	1.3281	2.6279
XLOC_086526	HSD17B3;ZNF367;SLC35D2;LOC100140121	chr8	84415769	84559249	4.9536	3.06152	6.57329	7.37571	9.94356	8.19894	9.39956	6.67521	8.20416
XLOC_088435	HSD17B3;ZNF367;SLC35D2;LOC100140121	chr8	84415769	84559249	1.92116	0.57398	3.00212	2.40801	4.02144	1.98042	2.39227	1.89704	3.34352
XLOC_088436	HSD17B3;ZNF367;SLC35D2;LOC100140121	chr8	84415769	84559249	0.0	1.35657	1.7738	1.21952	3.16043	4.43853	3.61303	1.56316	3.7501
XLOC_088437	HSD17B3;ZNF367;SLC35D2;LOC100140121	chr8	84415769	84559249	0.0	0.0	0.0	0.499681	1.56646	1.50209	0.805386	1.00094	0.973366
XLOC_083578	HSD17B4	chr7	35690208	35754470	14.4997	22.5578	23.4039	5.21977	3.53525	3.32109	4.24343	5.03467	3.89931
XLOC_084638	HSD17B4	chr7	35685507	35685822	0.0	9.5594	14.283	0.819267	0.348278	0.465386	0.7792	0.439028	1.18579
XLOC_084639	HSD17B4	chr7	35685906	35686573	1.4196	2.97038	5.54883	0.254326	0.0	0.0	0.382281	0.141035	0.123757
XLOC_084640	HSD17B4	chr7	35687777	35688807	4.97366	5.70232	3.24213	0.371499	0.194493	0.860321	0.0752224	0.414758	0.362105
XLOC_084641	HSD17B4	chr7	35688875	35690056	14.8394	10.9914	9.9509	1.83702	1.383	1.54129	1.54247	2.62031	1.42056

XLOC_084642	HSD17B4	chr7	35690208	35754470	2.97345	0.88875	3.4865	0.399512	0.115663	0.307341	0.133288	0.442752	0.0
XLOC_084643	HSD17B4	chr7	35690208	35754470	2.60877	1.55978	2.03967	0.584293	0.101626	0.404929	0.586662	0.259546	0.0
XLOC_084644	HSD17B4	chr7	35690208	35754470	0.0	0.824441	1.0781	0.247075	0.0	0.142631	0.495498	0.0	0.0
XLOC_063367	HSPBP1	chr18	62648891	62649145	0.0	0.0	0.0	5.9393	1.10183	0.0	1.81237	1.37711	7.57739
XLOC_062272	HSPBP1;PPP6R1	chr18	62649914	62661202	0.0	0.0	0.0	3.07447	0.904513	2.07281	0.778824	0.394849	1.92894
XLOC_092388	HTATSF1;CD40LG;ARHGEF6	chrX	20098922	20291564	20.6266	18.8686	18.4455	0.385617	0.257535	0.58858	0.191237	0.0523898	0.122044
XLOC_092815	HTATSF1;CD40LG;ARHGEF6	chrX	20098922	20291564	11.3946	6.24919	8.91508	0.0567517	0.198349	0.19734	0.230617	0.127	0.0553453
XLOC_092816	HTATSF1;CD40LG;ARHGEF6	chrX	20098922	20291564	6.24058	4.85082	2.92763	0.223642	0.0972849	0.387594	0.0	0.0	0.0
XLOC_047791	HTR2A	chr12	16810961	16826397	36.6479	36.5874	28.7216	9.54154	9.56267	10.2254	11.848	9.4078	8.8695
XLOC_048516	HTR2A	chr12	16826585	16826794	37.9179	7.56852	9.89184	5.76181	1.86665	2.53122	7.01026	4.61661	5.4276
XLOC_092205	HUWE1	chrX	96456364	96574513	6.2269	6.02743	4.84103	0.710361	0.36598	0.694166	0.353901	0.420881	0.429394
XLOC_059865	HVCN1	chr17	56764734	56769855	10.5278	10.5599	7.43804	37.6001	24.5421	31.2828	30.3005	47.9223	28.5631
XLOC_059866	HVCN1	chr17	56770101	56772186	0.0	0.235412	0.518406	0.295068	0.290249	0.413792	0.18094	0.332211	0.133798
XLOC_086545	IARS	chr8	85260286	85308048	48.7272	45.7645	47.2005	104.029	120.953	113.368	113.885	101.026	117.755
XLOC_086991	IARS	chr8	85260286	85308048	38.0583	36.4721	35.5294	84.082	84.8087	83.5266	90.1697	77.5018	90.8525
XLOC_088478	IARS	chr8	85260286	85308048	9.89693	7.88712	14.1808	13.2948	19.8645	13.4555	16.9557	11.1122	11.6379
XLOC_088479	IARS	chr8	85308275	85308465	0.0	5.26716	0.0	1.61915	5.12775	1.75156	0.0	0.0	0.0
XLOC_080075	IBSP	chr6	38306508	38355559	24.3218	15.2594	13.356	21.0576	22.7313	20.8376	27.7311	29.0704	26.2399
XLOC_089792	IBTK	chr9	22279011	22283256	2.35228	1.40499	5.51131	2.7366	5.99755	4.35318	4.98243	3.92819	5.10938
XLOC_089793	IBTK	chr9	22290420	22307684	0.531187	0.660715	3.32525	2.70557	2.79815	2.92808	3.38107	1.80429	1.66481
XLOC_089794	IBTK	chr9	22331435	22390141	39.7571	39.8487	34.1714	113.748	128.657	126.309	134.441	107.14	137.822
XLOC_090422	IBTK	chr9	22276606	22276925	0.0	0.0	3.48365	0.799187	2.72001	2.72537	0.761413	3.00136	2.31422
XLOC_090423	IBTK	chr9	22277055	22277571	0.0	4.82197	1.57628	3.07053	3.90753	3.3258	4.29945	3.97993	4.73876
XLOC_090424	IBTK	chr9	22277703	22278217	2.02895	1.81837	1.58511	1.99794	2.67179	1.67211	2.70168	2.20094	3.17677
XLOC_084254	ICAM1	chr7	16048411	16049121	2.61821	2.34812	0.0	2.69741	1.52984	2.16738	0.353241	1.56279	1.71256
XLOC_064325	ICT1	chr19	57041722	57043062	0.0	1.33231	3.48365	1.59837	0.680004	0.454229	0.380707	0.428766	0.385703

XLOC_064326	ICT1;C19H17orf28;CDR2L;USH1G;OTOP2;OTOP3;FDXR;GRIN2C;FADS6	chr19	57044708	57197382	4.88076	3.16107	6.40784	1.12505	0.560108	0.750367	1.01321	0.29257	1.98648
XLOC_051976	ID1	chr13	61723248	61726561	2.52112	0.480125	1.43516	1.06891	0.864575	1.14605	0.778178	1.06285	1.14385
XLOC_044991	ID2	chr11	88343134	88718110	55.8688	73.1937	71.6438	48.3339	39.3245	48.8516	54.9775	48.0235	49.0502
XLOC_050853	IDH3B;IDH3B;IDH3B	chr13	52967143	52982382	41.298	29.3483	28.5604	3.35577	2.38243	2.9393	3.40185	3.44945	4.96528
XLOC_065187	IFI35	chr19	43675833	43676747	0.956061	1.14364	0.747762	0.428411	0.448178	0.694026	0.605877	0.859707	0.166976
XLOC_064274	IFI35;RPL27	chr19	43663772	43674775	28.7799	27.8612	30.7821	27.3763	36.6985	29.3867	33.6464	28.2185	30.6477
XLOC_044510	IFITM3;COX8B;PSMD13	chr11	107212124	107296575	3.53281	2.65216	3.3984	5.78978	6.28562	6.51059	7.77248	6.41677	8.27284
XLOC_045158	IFITM3;COX8B;PSMD13	chr11	107212124	107296575	8.45907	9.46565	14.5316	32.2979	29.941	33.2133	37.2792	43.7972	30.0555
XLOC_047435	IFITM3;COX8B;PSMD13	chr11	107212124	107296575	0.0	1.05468	2.75779	5.68997	5.68473	4.33197	1.83397	2.05531	2.75336
XLOC_047436	IFITM3;COX8B;PSMD13	chr11	107212124	107296575	0.0	2.14901	0.0	9.70775	10.8154	7.98865	7.12173	8.78948	9.91244
XLOC_086345	IFNAG;KLHL9;IFNAA	chr8	22859450	22935642	34.1762	37.6649	34.9612	20.9806	23.0066	22.7613	24.16	21.6793	17.2303
XLOC_087441	IFNAG;KLHL9;IFNAA	chr8	22859450	22935642	52.626	36.647	41.0572	94.8924	71.3271	108.409	72.6426	101.877	102.455
XLOC_087442	IFNAG;KLHL9;IFNAA	chr8	22859450	22935642	0.0	3.81073	9.96422	3.04784	4.86848	3.03403	6.18939	4.50567	4.04708
XLOC_086346	IFNAH	chr8	23058620	23098034	3.96623	5.33849	6.59644	0.0444314	0.0	0.0	0.0	0.0	0.0433455
XLOC_086811	IFNB3;IFNB1	chr8	23174049	23208857	22.5516	26.9651	26.5402	20.0694	23.8149	21.6449	24.435	18.5893	21.0054
XLOC_089521	IFNGR1	chr9	76014976	76134858	0.367961	0.110102	0.0	0.659939	0.462162	0.497884	0.202069	0.333281	0.579622
XLOC_086343	IFNT;IFNT3;IFNT2	chr8	22624057	22627095	0.0	0.0	0.0	0.0	0.0	0.217125	0.0	0.0	0.0
XLOC_086808	IFN-tau-c1;IFNT;IFNT3;IFNT2	chr8	22628008	22653625	22.6114	24.1178	32.0341	26.9111	32.2908	32.599	32.2069	27.4814	35.5236
XLOC_086344	IFNW1;IFNAA	chr8	22757657	22782839	9.36458	11.1242	13.5169	0.517258	0.197876	0.491536	0.428812	0.217447	0.30056
XLOC_041709	IFT43	chr10	88379247	88446828	13.8907	16.7618	11.954	14.4279	13.9482	13.6782	14.2294	14.945	11.0315
XLOC_042105	IFT43	chr10	88322694	88357088	1.16761	0.291332	0.685377	0.163824	0.0687365	0.135147	0.213999	0.10521	0.227002
XLOC_037843	IFT57	chr1	53297089	53326381	12.355	17.2958	7.95439	20.2579	26.0032	22.0334	25.5577	24.0848	30.918
XLOC_038351	IFT57	chr1	53188726	53297021	6.26139	11.0625	6.96612	14.5712	17.2785	16.0612	17.8637	17.3725	21.1824

XLOC_038352	IFT57	chr1	53344634	53352696	19.3035	28.2941	19.4609	45.8042	48.7379	47.7669	46.1036	51.6566	54.3171
XLOC_039302	IFT57	chr1	53297089	53326381	0.0	0.388548	4.06463	0.465747	1.01264	1.21046	0.935376	0.775868	1.3604
XLOC_039303	IFT57	chr1	53297089	53326381	2.9541	3.53361	0.0	1.05895	2.30748	1.63357	2.49479	1.08185	2.57951
XLOC_039304	IFT57	chr1	53342679	53343583	0.0	0.579475	3.03108	0.260487	0.302757	0.301401	0.0876926	0.0967875	0.169205
XLOC_059858	IFT81	chr17	56302971	56323480	39.4953	42.4123	40.7428	207.032	160.35	176.126	189.526	275.513	169.811
XLOC_059859	IFT81	chr17	56325753	56403391	10.2774	13.8405	12.4303	18.8101	14.6345	18.5327	12.1988	14.6353	15.1377
XLOC_061651	IFT81	chr17	56302971	56323480	0.0	3.25905	8.52095	8.8096	5.37593	3.87473	6.88926	4.67748	2.35455
XLOC_061652	IFT81	chr17	56403579	56404512	0.932636	1.95238	2.18837	1.0866	0.510158	0.580388	0.253409	1.21172	0.733044
XLOC_061653	IFT81	chr17	56405782	56406719	5.5671	1.94237	5.08003	1.41366	0.652577	0.673665	0.252131	0.370946	1.05343
XLOC_047830	IFT88;CRYL1	chr12	36090389	36197419	1.41172	2.89579	3.20767	21.1457	22.6325	22.7863	22.7198	22.1391	30.2986
XLOC_048128	IFT88;CRYL1	chr12	36090389	36197419	7.68727	6.18122	4.88873	77.7995	70.564	73.8699	90.7812	99.4705	87.3683
XLOC_063986	IGF2BP1	chr19	38161900	38184050	0.0	0.0	0.0	0.0	0.0509998	0.0	0.0	0.0	0.0
XLOC_065074	IGF2BP1	chr19	38161900	38184050	0.0	0.0	0.0	0.0	0.0	0.323831	0.0	0.155425	0.273114
XLOC_065075	IGF2BP1	chr19	38161900	38184050	0.0	0.0	0.0	0.265298	0.173724	0.153666	0.0	0.0741218	0.193987
XLOC_090048	IGF2R;PLG;SLC22A1	chr9	97582622	98032979	6.66662	0.66373	0.0	0.0	0.0	0.0	0.0	0.0	0.0
XLOC_065777	IGFBP2;IGFBP5	chr2	105224335	105379870	0.0	0.297125	0.777117	0.0445228	0.116818	0.051642	0.0453392	0.0498937	0.130304
XLOC_068246	IGFBP2;IGFBP5	chr2	105224335	105379870	0.0	0.218411	0.0	0.130909	0.22861	0.075829	0.132764	0.0	0.0638159
XLOC_063997	IGFBP4;TNS4	chr19	41273660	41385438	2.31445	0.0	0.603524	0.0	0.0	0.0	0.0	0.154509	0.0
XLOC_064260	IGFBP4;TNS4	chr19	41273660	41385438	5.5348	1.6629	2.28042	0.213799	0.121184	0.288719	0.0848289	0.310163	0.108049
XLOC_066451	IGFBP5	chr2	105386322	105399672	19.0902	9.71601	11.5589	28.4261	31.0994	27.5719	51.5441	55.6613	61.9549
XLOC_059893	IGLL1	chr17	72836608	72850498	40.1125	42.5768	41.8628	12.6812	11.4243	11.8844	11.8297	11.6816	15.5924
XLOC_059894	IGLL1	chr17	72918312	72939585	89.492	115.795	79.3645	1.4359	0.104039	0.552761	2.40161	1.06268	1.39774
XLOC_061897	IGLL1	chr17	72939744	72941602	63.3703	68.7171	64.2359	0.839013	0.233598	0.531018	0.622161	0.384947	0.669811
XLOC_070228	IGSF8;KCNJ9;KCNJ10;DCAF16;PIGM	chr3	9690224	9833616	17.3642	19.9142	27.0365	0.272827	0.415902	0.389129	0.275078	0.207002	0.262034
XLOC_070661	IGSF8;KCNJ9;KCNJ10;DCAF16;PIGM	chr3	9690224	9833616	5.35123	6.39596	0.0	0.159721	0.276881	0.0	0.0	0.0	0.310592
XLOC_057997	IKBKE;RASSF5;SRGAP2	chr16	4084025	4213253	76.8627	79.5631	74.7642	17.4259	14.4351	16.3428	16.5064	21.2813	10.4544

XLOC_063993	IKZF3	chr19	40831265	40848771	3.89503	3.2981	3.96352	2.13768	2.43927	2.35273	2.31893	3.15038	1.74378
XLOC_065135	IKZF3	chr19	40830236	40831091	0.0	0.310073	0.0	0.0	0.242887	0.322439	0.187471	0.207002	0.0
XLOC_045908	IL1F10	chr11	46636606	46637176	1.75347	0.0	0.0	0.0	0.0	0.0	0.0	0.0	0.0
XLOC_044660	IL1R1	chr11	6889613	6895551	0.0	0.0	0.0	9.57446	2.97636	6.6187	2.8702	5.27544	4.07746
XLOC_045442	IL1R1	chr11	6885014	6885413	0.0	0.0	0.0	1.07145	0.460473	3.06731	0.0	0.875897	0.519233
XLOC_045443	IL1R1	chr11	6885994	6886644	0.0	0.0	0.0	2.89402	2.2854	1.9736	0.790245	1.45816	1.15206
XLOC_045444	IL1R1	chr11	6888753	6889520	0.0	0.0	0.0	4.46558	1.01796	3.56366	0.641849	2.12812	0.931846
XLOC_045445	IL1R1	chr11	6889613	6895551	0.0	0.0	0.0	3.7764	3.20771	2.62058	1.61854	2.83873	1.37953
XLOC_045446	IL1R1	chr11	6896034	6896296	0.0	0.0	0.0	1.8349	2.04934	1.37512	5.63639	1.28252	1.17241
XLOC_043967	IL1RL1	chr11	7063790	7172182	0.924403	1.38359	1.34413	1.1018	1.16349	1.00505	0.886247	1.4398	0.844523
XLOC_038053	IL20RB	chr1	133163662	133303546	0.929237	1.46268	0.565755	0.540348	0.792901	0.694604	0.473721	0.848467	0.757644
XLOC_040306	IL20RB	chr1	133163662	133303546	0.0	0.0	0.899879	0.0	0.0897696	0.0	0.20762	0.0	0.0
XLOC_040307	IL20RB	chr1	133163662	133303546	0.0	0.0	0.0	0.0	0.268151	0.0	0.0	0.0	0.0
XLOC_040308	IL20RB	chr1	133163662	133303546	0.0	0.0	0.0	0.0	0.0	0.0	0.0	0.0	0.215895
XLOC_040310	IL20RB	chr1	133163662	133303546	0.0	0.0	0.0	0.0	0.0	0.0	0.0	0.0	0.361619
XLOC_040312	IL20RB	chr1	133163662	133303546	0.0	0.0	0.0	0.0	0.165694	0.0	0.0	0.211801	0.0
XLOC_040314	IL20RB	chr1	133163662	133303546	0.0	0.0	3.79739	0.0	0.0	0.0	0.0	0.0	0.0
XLOC_040316	IL20RB	chr1	133163662	133303546	0.0	0.550621	1.43998	0.0	0.0	0.0	0.0	0.0	0.0
XLOC_040318	IL20RB	chr1	133163662	133303546	0.0	0.0	0.0	0.0	0.0	0.282609	0.0	0.0	0.0
XLOC_040319	IL20RB	chr1	133163662	133303546	2.91047	0.0	0.0	0.0	0.0	0.0	0.0	0.0	0.252384
XLOC_040320	IL20RB	chr1	133163662	133303546	0.0	1.02964	0.0	0.0	0.0	0.0	0.0	0.0	0.0
XLOC_040321	IL20RB	chr1	133163662	133303546	6.09544	0.0	0.0	0.0	0.0	0.0	0.0	0.0	0.0
XLOC_040322	IL20RB	chr1	133163662	133303546	0.0	0.0	0.0	0.0	0.0	0.0	0.0	0.883346	0.0
XLOC_040323	IL20RB	chr1	133163662	133303546	0.0	0.0	0.0	0.0	0.0	0.0	0.0	0.0	0.347785
XLOC_040324	IL20RB	chr1	133163662	133303546	16.9666	0.0	0.0	0.0	0.0	1.70575	0.0	0.0	0.0
XLOC_040327	IL20RB	chr1	133163662	133303546	0.0	0.0	0.0	0.0	0.106701	0.0707786	0.0	0.0	0.0
XLOC_040330	IL20RB	chr1	133163662	133303546	0.0	0.0	0.0	0.0	0.0	0.258411	0.0	0.0	0.0
XLOC_040331	IL20RB	chr1	133163662	133303546	2.08756	0.0	0.0	0.0	0.161625	0.0	0.0	0.205718	0.0
XLOC_040332	IL20RB	chr1	133163662	133303546	0.0	0.0	0.0	0.0	0.933326	0.0	0.0	0.0	0.0
XLOC_040333	IL20RB	chr1	133163662	133303546	0.0	0.0	0.0	0.12472	0.0	0.287986	0.250075	0.0	0.0
XLOC_040334	IL20RB	chr1	133163662	133303546	0.0	0.0	0.480162	0.0550199	0.04808	0.255112	0.0	0.2463	0.160974
XLOC_076319	IL22;IL26;IFNG	chr5	45669824	45857430	16.4668	22.1323	17.6862	42.3229	45.8671	44.825	52.979	46.0976	55.7225

XLOC_076847	IL22;IL26;IFNG	chr5	45669824	45857430	8.712	10.1857	11.0464	5.8662	8.43959	7.40688	5.91691	3.04351	8.59452
XLOC_077836	IL22;IL26;IFNG	chr5	45669824	45857430	3.69716	8.82277	5.76752	3.63641	5.6555	4.52664	6.6952	4.29145	8.95418
XLOC_077837	IL22;IL26;IFNG	chr5	45669824	45857430	2.67635	1.74746	2.28525	1.44019	2.17953	1.92612	1.76334	1.15262	2.19409
XLOC_077838	IL22;IL26;IFNG	chr5	45669824	45857430	3.72511	1.48488	0.970857	1.11246	1.83899	1.15684	1.11821	0.61816	1.19148
XLOC_077839	IL22;IL26;IFNG	chr5	45669824	45857430	0.0	7.85292	0.0	1.58152	6.54377	6.16959	0.712182	0.0	2.26003
XLOC_077840	IL22;IL26;IFNG	chr5	45669824	45857430	2.61609	0.781046	0.0	0.936195	2.6221	0.537053	0.689367	1.02472	2.72488
XLOC_077841	IL22;IL26;IFNG	chr5	45669824	45857430	0.0	2.63798	4.59882	2.10809	2.71888	0.905471	2.57287	0.0	2.04347
XLOC_077842	IL22;IL26;IFNG	chr5	45669824	45857430	0.0	1.22963	0.0	0.0	0.427083	1.84375	0.985501	0.0	0.836884
XLOC_077843	IL22;IL26;IFNG	chr5	45669824	45857430	1.08151	0.80887	1.69244	0.24241	0.763002	1.18065	0.789322	0.325832	0.756665
XLOC_077844	IL22;IL26;IFNG	chr5	45669824	45857430	0.0	0.0	0.0	0.309312	0.582239	1.18699	0.133445	0.0	0.553252
XLOC_077845	IL22;IL26;IFNG	chr5	45669824	45857430	2.73203	2.36176	3.32616	0.980039	1.5974	1.70683	1.39085	0.79493	1.67399
XLOC_077846	IL22;IL26;IFNG	chr5	45669824	45857430	1.00251	0.899304	0.0	0.898346	1.40924	1.35105	0.634726	0.300323	1.22533
XLOC_077847	IL22;IL26;IFNG	chr5	45669824	45857430	1.87157	1.67746	7.31148	1.00535	1.5966	1.54381	1.16624	0.184946	0.977277
XLOC_077848	IL22;IL26;IFNG	chr5	45669824	45857430	1.3026	0.584469	0.509545	0.350316	0.357061	0.541342	0.592953	0.19596	0.740168
XLOC_077849	IL22;IL26;IFNG	chr5	45669824	45857430	0.0	1.65933	1.92883	0.773548	1.06224	1.79337	1.45986	0.494695	1.88601
XLOC_077850	IL22;IL26;IFNG	chr5	45669824	45857430	0.446055	0.800707	1.74518	0.759889	0.909771	1.06723	0.570694	0.179387	1.63892
XLOC_077851	IL22;IL26;IFNG	chr5	45669824	45857430	0.978105	0.877433	1.52987	0.78885	0.6112	0.507061	0.973585	0.2931	1.36641
XLOC_077852	IL22;IL26;IFNG	chr5	45669824	45857430	5.01228	2.99768	2.352	0.718677	0.626327	1.24712	0.544051	0.500523	0.875235
XLOC_077853	IL22;IL26;IFNG	chr5	45669824	45857430	1.02047	1.22118	1.59697	0.365977	0.760122	0.848935	0.465748	0.563847	0.580156
XLOC_077854	IL22;IL26;IFNG	chr5	45669824	45857430	1.86642	2.23386	2.92131	1.10463	1.23065	1.20438	0.785716	0.939107	0.751317
XLOC_066548	IL22RA1	chr2	129365883	129369879	20.5099	1.44282	0.0	0.0	0.0	0.0	0.0	0.120172	0.0
XLOC_069844	IL6R	chr3	16183122	16217198	44.0335	41.8196	48.6218	39.0593	41.6995	42.1322	44.776	48.6818	45.0616
XLOC_070684	IL6R	chr3	16183122	16217198	12.2683	11.6717	12.2102	5.69644	6.70139	9.01254	12.1815	7.00657	9.0523

XLOC_070685	IL6R	chr3	16183122	16217198	8.39138	15.0231	32.7275	9.07024	16.302	15.1876	19.8222	9.3775	7.20874
XLOC_070686	IL6R	chr3	16183122	16217198	20.323	14.3527	24.5421	7.27871	10.1763	7.43103	10.8602	7.66905	8.04065
XLOC_070687	IL6R	chr3	16183122	16217198	12.7792	11.2867	13.8076	4.96468	6.34132	6.76705	7.81812	4.45743	7.66188
XLOC_070688	IL6R	chr3	16183122	16217198	13.8838	14.4892	21.6458	3.1158	4.17439	3.50004	7.45229	3.26243	8.35696
XLOC_070689	IL6R	chr3	16217275	16218632	3.61771	2.16451	4.7176	1.08113	1.55899	1.06532	1.92307	0.484038	2.37243
XLOC_070690	IL6R	chr3	16219489	16220937	1.6813	2.17962	1.75405	0.602961	0.790666	0.990453	1.32888	0.731478	1.27427
XLOC_070691	IL6R	chr3	16221454	16222138	0.0	1.23197	1.07398	0.49225	0.427885	0.852565	1.23416	0.546227	0.718685
XLOC_070692	IL6R	chr3	16222691	16222930	0.0	0.0	6.69111	0.0	0.0	0.862176	2.09271	0.797904	0.736775
XLOC_070693	IL6R	chr3	16223065	16224531	1.10544	1.81893	3.45987	0.545115	1.21308	1.37912	0.705807	0.665985	1.5468
XLOC_070694	IL6R	chr3	16224798	16225357	7.20607	3.22979	1.40776	0.645237	0.838801	1.30092	1.76643	1.78147	2.66617
XLOC_070695	IL6R	chr3	16226250	16227727	2.74057	1.6398	1.28664	0.589716	0.945224	0.512872	0.999976	0.275196	1.486
XLOC_053276	IL7	chr14	44102673	44103531	1.34314	0.0	0.0	0.0	0.0	0.0	0.0	0.0	0.0
XLOC_079825	IL8	chr6	90486474	90599569	3.32873	2.22763	1.91067	3.92094	3.97518	2.84594	2.81198	2.67982	5.09865
XLOC_072656	IMMP2L	chr4	57567709	57855493	76.9498	60.5933	112.881	37.9775	33.5556	32.3079	44.2118	52.9969	43.9359
XLOC_072657	IMMP2L	chr4	57855604	57896973	53.6471	30.795	47.7985	28.5062	28.161	28.7973	35.2574	39.6497	30.8241
XLOC_073180	IMMP2L	chr4	56995725	57143020	25.4271	46.7817	43.7072	5.04011	4.67508	4.13319	3.64313	5.52667	7.0182
XLOC_073181	IMMP2L	chr4	57190468	57301874	9.00026	8.13022	8.49612	19.0572	16.7296	18.2617	15.2291	16.0193	13.9708
XLOC_073182	IMMP2L	chr4	57190468	57301874	5.96488	0.890164	0.0	2.40085	2.52243	2.7494	1.56181	1.16323	1.29279
XLOC_073183	IMMP2L	chr4	57909338	57915814	0.54236	0.324518	0.424379	1.41019	1.10541	1.35335	3.26562	3.26791	1.28069
XLOC_073184	IMMP2L	chr4	57943720	57957024	177.122	118.887	83.4237	610.225	620.114	596.546	640.59	616.376	746.575
XLOC_073185	IMMP2L	chr4	58046602	58108375	1.58421	0.328935	0.402329	0.306952	0.0510943	0.169215	0.283626	0.186863	0.280369
XLOC_074310	IMMP2L	chr4	56995725	57143020	10.6412	17.1883	12.2099	2.0986	2.55467	2.72621	1.41942	3.48364	3.16227
XLOC_074311	IMMP2L	chr4	57413766	57414237	13.8436	7.57988	7.20811	2.06488	1.06997	0.71184	3.66827	2.49447	1.20298
XLOC_074312	IMMP2L	chr4	57567709	57855493	0.0	11.3159	8.87647	0.0	0.0	0.0	0.0	0.0	0.0
XLOC_074313	IMMP2L	chr4	57567709	57855493	6.98003	8.34321	5.45473	0.0	0.0	0.0	0.0	0.0	0.0
XLOC_074314	IMMP2L	chr4	57567709	57855493	15.2856	11.6308	14.1225	0.0	0.0	0.143718	0.0	0.138104	0.0
XLOC_074315	IMMP2L	chr4	57567709	57855493	6.54898	8.8144	8.32467	0.0733754	0.064029	0.0	0.0743	0.0819292	0.0
XLOC_074316	IMMP2L	chr4	57567709	57855493	10.7822	10.2024	25.2768	0.160909	0.0	0.0	0.0	0.0	0.0
XLOC_074317	IMMP2L	chr4	57567709	57855493	7.17035	6.42775	7.00397	0.0	0.0	0.0	0.0	0.0	0.0
XLOC_074318	IMMP2L	chr4	57567709	57855493	10.3948	8.28335	10.8312	0.0	0.0	0.0	0.0	0.0	0.0
XLOC_074319	IMMP2L	chr4	57567709	57855493	21.7542	14.2954	11.8949	0.0	0.0	0.0	0.0	0.0	0.378255
XLOC_074320	IMMP2L	chr4	57567709	57855493	10.8361	5.39654	8.46761	0.0	0.0	0.0	0.0	0.0	0.0
XLOC_074321	IMMP2L	chr4	57567709	57855493	7.70129	4.60477	8.02841	0.0	0.0	0.0	0.0	0.0	0.0

XLOC_074322	IMMP2L	chr4	57567709	57855493	2.43953	9.47062	5.71524	0.218299	0.0	0.0	0.0	0.0	0.0
XLOC_074323	IMMP2L	chr4	57567709	57855493	10.1277	6.06096	11.6061	0.356796	0.312349	0.112947	0.132438	0.109213	0.0316544
XLOC_074324	IMMP2L	chr4	57567709	57855493	7.4312	5.88561	8.89403	0.235181	0.102887	0.545742	0.0	0.307683	0.152979
XLOC_074325	IMMP2L	chr4	57567709	57855493	37.9341	3.39496	17.7529	0.0	0.0	0.0	0.0	0.366567	0.0
XLOC_074326	IMMP2L	chr4	57908517	57909238	2.5671	0.383723	1.00355	1.72488	0.600114	1.0627	1.73254	2.17141	1.34342
XLOC_074327	IMMP2L	chr4	57941894	57942733	1.06129	0.317346	0.0	0.190204	0.662778	0.219974	0.863041	0.211799	1.29698
XLOC_074328	IMMP2L	chr4	57957180	57958040	0.0	1.53934	2.41555	1.47619	1.84899	1.38736	1.76854	0.513868	0.988702
XLOC_053216	IMPAD1	chr14	25459351	25645736	20.2079	10.701	12.3846	20.3827	20.8566	14.2818	12.9537	17.8365	19.8951
XLOC_053507	IMPAD1	chr14	25459351	25645736	2.86496	1.27303	4.92744	3.86532	6.73846	3.77954	2.88237	3.35324	5.58302
XLOC_053987	IMPAD1	chr14	25459351	25645736	0.0	0.586601	0.767114	0.659253	1.1532	0.815673	0.447607	0.738842	1.37204
XLOC_053988	IMPAD1	chr14	25459351	25645736	0.483285	1.30134	1.51271	0.346673	1.02337	0.753995	0.441383	1.11708	0.972339
XLOC_053989	IMPAD1	chr14	25459351	25645736	0.0	1.08439	2.83614	0.844952	1.07832	0.677704	1.58214	0.79916	1.39412
XLOC_053990	IMPAD1	chr14	25459351	25645736	3.7429	4.47323	0.0	1.17293	3.1932	1.54384	1.83266	1.2944	2.93183
XLOC_053991	IMPAD1	chr14	25459351	25645736	1.30138	1.16789	3.56361	2.81181	3.50958	3.47343	3.77735	2.43362	3.5081
XLOC_053992	IMPAD1	chr14	25459351	25645736	1.975	1.80819	2.18104	2.76354	4.69933	3.37522	3.03064	3.40235	4.28524
XLOC_053993	IMPAD1	chr14	25459351	25645736	2.62518	0.628314	0.821662	0.517831	1.02908	0.655138	0.862454	0.316437	1.05618
XLOC_053994	IMPAD1	chr14	25459351	25645736	3.58926	0.306811	4.01224	0.735592	1.32674	0.746501	0.655253	0.412102	0.941818
XLOC_053995	IMPAD1	chr14	25459351	25645736	0.0	0.0	0.0	1.13984	1.48722	1.05345	0.572553	0.126665	0.443887
XLOC_053996	IMPAD1	chr14	25459351	25645736	0.600311	0.718555	1.40953	2.88027	3.22981	2.37535	2.53065	2.08606	2.57484
XLOC_053997	IMPAD1	chr14	25459351	25645736	6.57794	1.96113	5.12721	5.89948	6.92898	3.98347	5.99864	6.19914	10.7508
XLOC_053998	IMPAD1	chr14	25459351	25645736	1.04142	0.311411	1.6289	2.09687	2.19535	2.50097	2.48622	3.12921	3.65024
XLOC_053999	IMPAD1	chr14	25459351	25645736	0.803018	0.540722	1.09998	1.90862	2.06726	1.35962	1.71417	1.43744	2.25015
XLOC_054000	IMPAD1	chr14	25459351	25645736	1.32178	0.922781	1.72393	1.1062	1.24439	1.14595	1.04705	0.841733	1.81171
XLOC_054001	IMPAD1	chr14	25459351	25645736	0.0	0.233236	0.610004	0.978572	1.46425	0.647652	0.566609	0.8589	1.02208
XLOC_054002	IMPAD1	chr14	25459351	25645736	0.975697	0.729791	1.14524	0.656141	0.726925	0.304442	0.846474	0.637326	1.19488
XLOC_054003	IMPAD1	chr14	25459351	25645736	0.546048	0.653444	1.70905	0.391668	0.299623	0.794808	0.796992	0.603179	0.716322
XLOC_054004	IMPAD1	chr14	25459351	25645736	2.57109	0.947167	2.25428	0.615629	0.788337	0.379577	0.514966	0.85988	0.719098
XLOC_054005	IMPAD1	chr14	25459351	25645736	0.409415	0.122501	0.961194	0.183569	0.353413	0.383427	0.149774	0.370651	0.358235
XLOC_054006	IMPAD1	chr14	25459351	25645736	0.0	1.65142	0.0	0.0	0.213017	0.283713	0.726965	0.0	0.959865
XLOC_054007	IMPAD1	chr14	25459351	25645736	2.51807	0.538195	1.68916	0.58066	0.395366	0.37444	0.790308	0.362072	0.629628
XLOC_089748	IMPG1	chr9	15925014	15944954	0.0	0.0	0.0	0.128422	0.0	0.0	0.0	0.0	0.0
XLOC_070332	INADL	chr3	84163775	84270758	8.30236	11.8765	14.8994	16.6	9.7603	14.4102	9.87175	17.744	10.8164
XLOC_070333	INADL	chr3	84442176	84480338	2.76539	1.94756	4.09417	0.397588	0.525418	0.417399	0.352468	0.213415	0.543077

XLOC_071486	INADL	chr3	84163097	84163646	1.84754	6.07227	10.1056	10.0908	7.5959	7.62157	5.75921	11.321	6.59333
XLOC_071487	INADL	chr3	84163775	84270758	0.0	0.0	0.0	0.0	0.144776	0.0	0.0	0.0	0.162462
XLOC_071488	INADL	chr3	84163775	84270758	0.0	0.0	0.0	0.0	0.0	0.106269	0.0	0.204684	0.0
XLOC_071490	INADL	chr3	84442176	84480338	0.0	0.128082	0.0	0.115155	0.0335877	0.0890777	0.0	0.0	0.0
XLOC_069948	INADL;LOC782781	chr3	84087833	84162593	4.03822	7.09975	10.4699	10.964	7.65689	9.67402	7.17661	10.481	8.9942
XLOC_070331	INADL;LOC782781	chr3	84087833	84162593	7.31879	3.91671	1.82608	16.0132	14.1612	13.9074	15.6948	15.2309	15.9888
XLOC_072150	ING5	chr3	121202838	121204050	0.685865	1.43614	0.536587	0.184454	0.0	0.213738	0.124808	0.0	0.0599522
XLOC_065470	INPP1	chr2	5794655	5868585	12.5092	18.275	16.7488	41.3303	38.7532	42.0681	48.3432	50.8841	51.2451
XLOC_066124	INPP1	chr2	5794655	5868585	33.0089	30.6839	23.545	58.6244	59.3064	58.3368	68.9243	74.284	69.8228
XLOC_066799	INPP1	chr2	5868914	5869061	0.0	73.897	0.0	77.3145	46.9004	78.4543	93.1529	36.0572	73.2718
XLOC_043936	INPP4A	chr11	3648449	3691000	26.5872	31.5118	30.0246	30.5767	24.9999	28.0812	34.6532	42.1857	25.8305
XLOC_045303	INPP4A	chr11	3648449	3691000	4.85337	4.34081	3.78334	4.3423	4.05395	3.94066	4.93448	5.56795	4.18586
XLOC_045304	INPP4A	chr11	3648449	3691000	11.7409	17.5626	18.363	6.40795	12.1657	7.05787	14.0205	7.53101	23.1898
XLOC_045305	INPP4A	chr11	3691109	3692375	0.652452	0.585523	0.510464	0.701896	1.02201	0.745683	0.475208	0.327161	0.855578
XLOC_044623	INPP4A;COA5;MGAT4A;UNC50	chr11	3726148	3820035	3.0877	0.0	0.0	0.0	0.0	0.0	0.0	0.153147	0.0
XLOC_064770	INPP5K	chr19	23199278	23199658	0.0	7.75844	5.07189	0.290647	1.24691	0.332395	0.847068	0.947781	2.81507
XLOC_064771	INPP5K	chr19	23205777	23206596	0.0	1.30772	0.855035	1.46961	1.10929	0.339869	0.691141	0.218118	0.668018
XLOC_055747	INSC	chr15	37929009	37961174	3.13604	3.92069	0.613298	2.44422	3.23689	2.22339	2.51276	2.27569	3.9229
XLOC_056385	INSC	chr15	37923981	37924291	0.0	2.81931	0.0	1.26872	1.0776	0.960236	0.401316	0.90512	2.85527
XLOC_056386	INSC	chr15	37924507	37925638	0.742936	2.66666	1.1624	1.26534	2.96538	2.23727	1.48556	1.2652	3.50608
XLOC_056387	INSC	chr15	37925765	37926708	1.84152	1.92755	1.44036	1.40287	2.51856	1.81463	1.75166	1.74868	3.69913
XLOC_056388	INSC	chr15	37926866	37927090	0.0	0.0	0.0	0.928303	0.761586	1.0285	1.64724	1.8912	2.64132
XLOC_056389	INSC	chr15	37927234	37927395	0.0	21.8636	0.0	17.2412	15.473	21.5702	13.4877	6.26931	31.0842
XLOC_056390	INSC	chr15	37962424	37963172	2.44971	2.19718	0.957719	1.20715	1.24137	2.53618	1.76543	0.853836	2.03025
XLOC_056391	INSC	chr15	37963281	37963727	0.0	0.0	0.0	0.897352	0.773907	2.57528	1.1033	1.22916	2.39485
XLOC_056392	INSC	chr15	37963958	37965208	0.0	1.98031	0.517935	1.60238	1.81457	2.68235	1.80781	2.38978	2.43062
XLOC_056393	INSC	chr15	37965349	37966362	3.38167	1.76995	1.9839	2.1217	1.45443	1.75466	2.07073	2.62207	2.73266
XLOC_056394	INSC	chr15	37966521	37966998	4.52787	0.676168	1.76826	4.66024	3.32575	3.95905	4.20255	6.01093	7.28016
XLOC_073322	INSIG1	chr4	117894382	117915166	10.3276	9.35543	8.88857	7.61795	5.64546	7.02826	7.41983	10.7898	4.28682
XLOC_048204	IPO5	chr12	78961763	79007213	2.06189	4.37647	4.84184	1.64186	1.87252	1.26744	0.860226	1.99253	1.82633

XLOC_070446	IPP	chr3	100939849	100970056	7.32442	8.87374	7.09744	35.8555	33.1188	33.1078	34.0358	37.4872	35.5462
XLOC_071883	IPP	chr3	100937178	100938344	0.717151	0.214515	0.0	0.321436	0.954351	0.372408	0.45649	0.43118	0.438759
XLOC_071884	IPP	chr3	100938534	100939326	5.69953	5.11243	1.78276	26.6582	21.165	21.1358	23.1408	23.1816	25.2668
XLOC_070125	IQCA1	chr3	116513057	116549203	6.48928	6.27257	4.76157	16.5588	18.0976	16.5508	20.5759	20.573	22.9661
XLOC_070126	IQCA1	chr3	116581074	116629250	3.83124	4.87893	4.38199	10.535	12.3023	10.1658	12.5795	11.2664	10.7377
XLOC_070507	IQCA1	chr3	116272523	116423997	2.70572	7.17148	3.64891	1.14288	0.871017	0.89493	0.87879	1.08471	1.31412
XLOC_070508	IQCA1	chr3	116488917	116508252	29.0859	45.6144	33.0151	45.4334	36.9303	42.2849	47.7136	54.1372	45.5349
XLOC_070509	IQCA1	chr3	116560626	116561534	65.6909	69.1385	60.9856	231.587	172.751	208.094	270.39	301.212	266.941
XLOC_070510	IQCA1	chr3	116581074	116629250	5.12309	7.83537	6.50102	6.73726	6.98583	7.37028	6.985	6.32852	7.69364
XLOC_072112	IQCA1	chr3	116513057	116549203	139.821	145.181	142.822	144.049	139.844	169.335	161.023	187.2	166.761
XLOC_072113	IQCA1	chr3	116549440	116549900	0.0	10.7112	0.0	10.485	7.94173	9.33975	14.3402	12.6781	14.7484
XLOC_072114	IQCA1	chr3	116553048	116553899	1.04292	1.24744	2.44687	0.93458	1.13996	0.432383	1.03693	0.208181	0.910441
XLOC_072115	IQCA1	chr3	116554269	116554509	0.0	0.0	26.4702	5.34987	7.60261	5.11723	4.14287	1.5792	2.91497
XLOC_072116	IQCA1	chr3	116577199	116577744	1.86661	0.0	0.0	1.00276	1.73732	0.577451	0.83095	0.368878	0.649848
XLOC_072117	IQCA1	chr3	116578993	116580015	1.67323	1.75154	0.654422	0.449921	0.850549	0.434121	0.759077	0.334846	0.657803
XLOC_072118	IQCA1	chr3	116581074	116629250	4.70181	4.21571	2.45003	2.10559	1.09668	1.45733	1.54333	2.95308	1.77524
XLOC_038397	IQCB1	chr1	66830827	66863130	8.36102	7.98152	8.41273	1.89211	2.27911	2.75832	3.79837	2.78555	1.82864
XLOC_065845	IQCC	chr2	122260027	122265591	5.22317	1.4356	0.923205	16.1702	12.4643	18.722	7.06252	7.38063	7.29504
XLOC_065846	IQCC	chr2	122279625	122302619	0.0	0.37448	0.0	0.0	0.0	0.0	0.0	0.0	0.0547237
XLOC_066510	IQCC	chr2	122279625	122302619	0.0	0.142319	0.113463	0.0	0.0124617	0.0362577	0.0	0.0159912	0.0
XLOC_068458	IQCC	chr2	122214604	122215373	3.54836	5.30451	0.924868	38.0454	26.1967	38.9505	30.177	41.836	32.6116
XLOC_068465	IQCC	chr2	122259347	122259903	0.0	1.08469	1.41834	0.812607	0.704188	1.12338	0.161753	0.0	0.158006
XLOC_068466	IQCC	chr2	122346145	122346895	0.0	0.729924	0.954491	0.328111	0.475855	0.505537	0.219951	0.243142	0.319489
XLOC_068467	IQCC	chr2	122347265	122348108	1.05509	1.26198	0.0	0.37819	0.247104	0.546746	0.381379	0.210579	0.36841
XLOC_068468	IQCC	chr2	122378037	122378358	8.82829	13.1624	10.3249	11.4479	13.7763	13.4654	15.0564	12.2905	9.90837
XLOC_065844	IQCC;KHDRBS1	chr2	122234781	122259137	0.622679	1.03827	6.54554	30.682	15.5817	24.0604	9.10435	17.549	15.4542
XLOC_066509	IQCC;KHDRBS1	chr2	122234781	122259137	0.0	0.29074	0.380204	0.0	0.0	0.0	0.177493	0.0	0.0425024
XLOC_068459	IQCC;KHDRBS1	chr2	122234781	122259137	0.0	0.0	0.0	1.15208	0.570404	0.379163	0.0	0.545067	0.160001
XLOC_068460	IQCC;KHDRBS1	chr2	122234781	122259137	0.0	0.0	0.0	0.879819	0.0	1.34139	0.0	0.637359	0.0
XLOC_068461	IQCC;KHDRBS1	chr2	122234781	122259137	0.0	0.0	0.0	1.08674	0.156731	0.416874	0.179627	0.199539	0.527991

XLOC_068462	IQCC;KHDRBS1	chr2	122234781	122259137	0.0	0.0	0.0	0.831472	0.103285	0.27437	0.119221	0.131877	0.231257
XLOC_068463	IQCC;KHDRBS1	chr2	122234781	122259137	0.0	0.0	3.96653	1.82125	0.771557	0.0	0.859472	0.0	0.438683
XLOC_068464	IQCC;KHDRBS1	chr2	122234781	122259137	0.0	0.0	0.0	0.310075	0.796957	2.12535	0.0	0.0	0.0
XLOC_066511	IQCC;PTP4A2	chr2	122348726	122369919	4.81716	4.43619	5.52521	7.08136	3.94488	4.88825	3.73229	7.092	2.44348
XLOC_065843	IQCC;TMEM39B	chr2	122186265	122205647	2.52445	2.55831	1.65164	4.62998	3.93594	4.92433	3.17401	3.84642	3.97281
XLOC_068457	IQCC;TMEM39B	chr2	122205753	122206393	0.0	0.448053	0.0	1.20842	2.91525	1.8592	1.61235	1.63662	2.22097
XLOC_073232	IQUB	chr4	88598966	88599875	12.4255	9.90814	16.1958	18.3724	19.1609	17.8163	18.8144	20.3609	23.0507
XLOC_062143	IRF8;MIR2325A	chr18	11839940	11944475	2.92686	3.16974	4.12219	2.11796	2.84831	1.92616	1.16017	1.4353	3.28774
XLOC_062160	IRX5	chr18	23310082	23659572	29.4861	44.1923	43.9926	6.74043	5.21849	7.55022	3.11227	5.15055	2.59021
XLOC_086962	ISCA1;ZCCHC6	chr8	80764350	80880252	20.1433	25.2099	24.609	102.586	120.796	110.18	112.497	108.731	97.764
XLOC_088296	ISCA1;ZCCHC6	chr8	80764350	80880252	0.0	1.05303	2.7539	1.42001	2.59878	1.81815	0.943057	1.22027	0.920499
XLOC_088297	ISCA1;ZCCHC6	chr8	80764350	80880252	1.37376	0.821311	1.07398	1.23063	0.962742	1.56303	0.493663	0.546227	0.838466
XLOC_088298	ISCA1;ZCCHC6	chr8	80764350	80880252	0.0	0.0	3.28314	2.25902	2.56737	1.28557	3.6015	0.0	0.72737
XLOC_088299	ISCA1;ZCCHC6	chr8	80764350	80880252	1.31148	0.392062	0.0	0.939929	0.510864	1.22134	0.707732	0.52186	0.228753
XLOC_088300	ISCA1;ZCCHC6	chr8	80764350	80880252	0.0	0.0	0.899879	0.928015	1.16701	0.95357	0.311428	0.917639	0.602528
XLOC_088301	ISCA1;ZCCHC6	chr8	80764350	80880252	1.15447	0.345176	0.902749	1.96539	2.5215	1.91318	1.66614	0.690396	1.71259
XLOC_088302	ISCA1;ZCCHC6	chr8	80764350	80880252	0.0	0.621973	2.16896	1.61544	1.73651	2.30381	1.00886	0.486314	1.51457
XLOC_088303	ISCA1;ZCCHC6	chr8	80764350	80880252	0.0	1.44042	1.88358	1.4029	3.38095	3.74139	1.41106	1.31973	0.945752
XLOC_088304	ISCA1;ZCCHC6	chr8	80764350	80880252	3.21109	0.479834	2.50976	3.01961	1.87182	3.48243	1.57991	1.75061	1.5386
XLOC_088305	ISCA1;ZCCHC6	chr8	80764350	80880252	5.28246	1.05323	0.688647	2.7618	2.95958	2.00957	0.638532	1.40874	2.07638
XLOC_088306	ISCA1;ZCCHC6	chr8	80764350	80880252	2.12569	1.58906	0.0	1.80959	1.74233	1.98265	1.92063	0.742373	1.2061
XLOC_088307	ISCA1;ZCCHC6	chr8	80764350	80880252	2.24289	2.67956	0.0	2.40886	2.94849	3.69198	3.57003	1.32367	0.974727
XLOC_088308	ISCA1;ZCCHC6	chr8	80764350	80880252	0.0	2.4045	6.28834	3.74687	3.37667	2.65913	2.15907	2.23292	1.96255

XLOC_088309	ISCA1;ZCCHC6	chr8	80764350	80880252	0.0	0.0	0.0	1.7366	1.84219	1.97033	0.822414	1.85598	1.25576
XLOC_088310	ISCA1;ZCCHC6	chr8	80764350	80880252	0.0	0.769939	4.02687	0.922879	1.59106	3.17709	2.4933	1.51576	0.671596
XLOC_088311	ISCA1;ZCCHC6	chr8	80764350	80880252	0.0	0.0	5.73839	1.97346	3.37657	4.50415	0.951904	0.355876	0.636381
XLOC_088312	ISCA1;ZCCHC6	chr8	80764350	80880252	0.0	7.34235	0.0	6.70248	3.62599	4.91384	1.94743	5.60808	1.05337
XLOC_088313	ISCA1;ZCCHC6	chr8	80764350	80880252	0.0	1.16983	3.05946	3.03826	4.3699	2.69953	0.703994	1.42747	1.93393
XLOC_088314	ISCA1;ZCCHC6	chr8	80764350	80880252	2.20391	0.439482	0.574712	2.17316	2.24245	2.21238	1.13528	1.03039	0.963057
XLOC_088315	ISCA1;ZCCHC6	chr8	80764350	80880252	0.0	0.0	0.0	2.52245	3.47321	3.75835	1.23404	1.37734	2.44595
XLOC_088316	ISCA1;ZCCHC6	chr8	80764350	80880252	0.0	0.45464	1.189	3.13357	2.48443	3.14385	2.18057	1.50934	1.5907
XLOC_088317	ISCA1;ZCCHC6	chr8	80764350	80880252	1.78375	3.73098	2.78779	2.55553	3.7379	2.20839	2.06767	1.05857	2.01885
XLOC_088318	ISCA1;ZCCHC6	chr8	80764350	80880252	0.918426	0.274665	2.15506	3.3748	3.87598	3.6201	2.08011	1.65249	2.80743
XLOC_088319	ISCA1;ZCCHC6	chr8	80764350	80880252	3.79914	1.70421	1.48571	1.87264	3.26522	2.46249	1.11794	0.759224	1.07824
XLOC_088320	ISCA1;ZCCHC6	chr8	80764350	80880252	0.0	0.382367	3.00002	1.71879	2.09305	2.91217	1.61145	1.78199	0.780904
XLOC_088321	ISCA1;ZCCHC6	chr8	80764350	80880252	0.0	1.94832	0.0	4.08736	2.2543	2.00319	0.850671	1.58649	0.565522
XLOC_088322	ISCA1;ZCCHC6	chr8	80764350	80880252	1.09873	0.49306	3.009	2.512	2.36842	1.59923	0.952119	1.10328	1.10502
XLOC_088323	ISCA1;ZCCHC6	chr8	80764350	80880252	0.0	0.0	0.0	7.40756	6.20247	4.85611	2.84203	0.646827	1.77456
XLOC_088324	ISCA1;ZCCHC6	chr8	80764350	80880252	0.962426	0.287812	4.51639	3.27757	3.08277	2.39526	2.43935	1.44227	0.924457
XLOC_088325	ISCA1;ZCCHC6	chr8	80764350	80880252	0.0	2.35737	1.23302	6.0753	4.5376	4.23708	2.2591	4.84911	2.33658
XLOC_088326	ISCA1;ZCCHC6	chr8	80764350	80880252	1.55381	1.39318	2.42901	4.17494	2.29551	1.44484	2.08696	1.84938	0.541561
XLOC_088327	ISCA1;ZCCHC6	chr8	80764350	80880252	0.0	0.691115	2.1691	2.5683	2.39172	2.8834	1.85722	1.43962	1.41454
XLOC_088328	ISCA1;ZCCHC6	chr8	80764350	80880252	0.0	0.699273	0.0	3.3529	0.912076	2.78552	1.37087	0.932265	1.02038
XLOC_088329	ISCA1;ZCCHC6	chr8	80764350	80880252	1.79803	1.6132	3.51593	2.82011	3.02167	3.45076	1.05918	1.70784	1.80602
XLOC_088330	ISCA1;ZCCHC6	chr8	80764350	80880252	1.51475	4.07465	2.36806	3.52748	1.88507	3.28735	1.62877	2.55532	1.18806

XLOC_088331	ISCA1;ZCCHC6	chr8	80764350	80880252	0.0	0.565142	0.985393	0.846831	0.887932	1.047	0.975057	0.568538	0.440451
XLOC_088332	ISCA1;ZCCHC6	chr8	80764350	80880252	1.32107	1.57969	3.09852	2.95871	2.16114	2.3237	1.54435	0.657015	1.26729
XLOC_088333	ISCA1;ZCCHC6	chr8	80764350	80880252	1.64096	1.71778	3.85087	0.80896	1.47599	2.72506	1.63825	0.410566	1.00357
XLOC_063363	ISOC2	chr18	62503148	62503547	2.99466	0.893835	0.0	0.803585	2.76284	0.920192	0.522644	0.0	0.259616
XLOC_062271	ISOC2;RPL28;UBE2S	chr18	62503925	62548947	2.29704	3.06958	4.25693	3.65813	2.18438	2.05081	3.86193	2.83035	1.51445
XLOC_062465	ISOC2;RPL28;UBE2S	chr18	62503925	62548947	24.4061	28.0686	22.1273	27.1746	27.4441	26.7911	27.7285	28.7355	28.3589
XLOC_063364	ISOC2;RPL28;UBE2S	chr18	62503925	62548947	0.0	6.60745	4.31995	3.30008	1.14393	0.760336	0.820863	1.09289	1.60409
XLOC_063365	ISOC2;RPL28;UBE2S	chr18	62503925	62548947	0.0	3.35969	2.92834	1.00722	0.287554	0.76611	0.970663	0.725953	1.29884
XLOC_083371	ISYNA1;SSBP4;ELL	chr7	4632004	4685145	98.7793	89.8456	93.2936	79.58	64.033	70.8257	67.4451	90.9646	60.2388
XLOC_084053	ISYNA1;SSBP4;ELL	chr7	4632004	4685145	3.50084	3.13351	5.46245	2.81811	1.07264	2.50307	2.72596	2.03624	0.606018
XLOC_050603	ITCH	chr13	64432379	64439749	2.50257	2.37978	1.85814	2.74435	2.17518	2.72627	2.0286	2.06189	2.39021
XLOC_050916	ITCH	chr13	64363917	64370721	372.026	412.352	297.844	70.7456	90.4541	75.3287	89.36	48.65	87.5641
XLOC_052251	ITCH	chr13	64361229	64361684	0.0	0.726052	0.0	0.870269	0.93868	0.0	0.428551	0.477222	2.11185
XLOC_052252	ITCH	chr13	64362431	64363019	6.72058	5.02091	2.62617	1.35414	2.34914	2.08146	2.55165	0.998342	3.07297
XLOC_066925	ITGA4	chr2	15128331	15128607	12.0917	18.0229	14.1363	94.7929	77.0676	85.5522	89.2613	94.9637	85.3432
XLOC_067057	ITGA6	chr2	24179110	24179510	8.94698	4.45082	4.65548	13.0713	13.0706	10.6921	19.2623	13.6678	10.0838
XLOC_067058	ITGA6	chr2	24179678	24179847	0.0	60.2244	0.0	45.7364	53.3263	45.4323	64.6188	62.5421	41.6711
XLOC_067059	ITGA6	chr2	24179967	24180109	74.6753	46.6506	0.0	37.8435	53.2792	60.7412	71.9798	128.498	52.7406
XLOC_050460	ITGB1	chr13	20090280	20606573	12.3523	13.1573	16.8322	19.1286	13.8988	19.2953	10.7607	7.71555	7.42014
XLOC_071481	ITGB3BP	chr3	82392493	82395560	0.994287	0.81826	1.36192	2.16247	3.30089	2.69273	3.12385	2.68067	2.95973
XLOC_069943	ITGB3BP;EFCAB7	chr3	82358100	82392319	2.03608	4.13698	2.37987	6.03257	6.49297	6.21711	7.88987	6.46792	5.01056
XLOC_065366	ITGB4	chr19	56485711	56486448	0.0	0.0	0.0	0.0	0.19457	0.258396	0.112386	0.372771	0.326624
XLOC_037897	ITGB5	chr1	69826979	69896583	0.0	0.777624	0.677928	0.543762	0.609864	0.71943	1.41467	0.520134	0.908503
XLOC_077468	ITGB7	chr5	26967151	26967670	0.0	0.0	0.0	0.537371	0.155025	0.412317	0.0	0.0	0.174064
XLOC_047928	ITGBL1	chr12	81860225	82001022	19.1239	22.9365	19.029	6.33422	5.9597	5.51348	5.77924	5.96604	5.12157
XLOC_069835	ITLN2	chr3	8509345	8561522	0.862285	0.0	0.674399	165.647	142.82	160.969	216.6	222.403	204.736
XLOC_070629	ITLN2	chr3	8509345	8561522	0.0	0.319217	0.0	22.8634	21.6664	22.5689	37.1327	41.9676	24.5079
XLOC_068374	ITM2C	chr2	119477614	119478340	3.81677	1.14105	3.97894	0.911856	1.28893	1.71182	0.801571	1.13962	0.887774

XLOC_066481	ITM2C;CAB39	chr2	119428290	119477406	20.1547	24.6395	21.5275	24.198	22.146	25.0917	24.4714	26.9855	21.559
XLOC_068365	ITM2C;CAB39	chr2	119428290	119477406	0.0	1.50627	2.62621	1.35414	0.522032	0.173455	1.35087	0.499171	0.292664
XLOC_068366	ITM2C;CAB39	chr2	119428290	119477406	9.33597	8.35029	7.27801	1.66999	3.90226	5.21528	6.34474	2.68229	2.81924
XLOC_068367	ITM2C;CAB39	chr2	119428290	119477406	0.0	1.86277	9.74048	3.92025	1.8834	3.15602	2.5998	1.77094	4.83923
XLOC_068368	ITM2C;CAB39	chr2	119428290	119477406	0.0	2.34952	12.2846	0.0	2.94752	1.58539	0.6445	0.0	2.03046
XLOC_068369	ITM2C;CAB39	chr2	119428290	119477406	7.9161	4.72979	3.09235	1.5945	1.84025	2.65109	2.4617	1.17166	2.23824
XLOC_068370	ITM2C;CAB39	chr2	119428290	119477406	12.8349	7.68634	0.0	7.02457	12.3147	7.70976	4.0636	7.02647	2.20453
XLOC_068371	ITM2C;CAB39	chr2	119428290	119477406	2.03483	2.43129	3.17917	2.36783	2.67918	1.46716	2.8896	1.80571	1.94678
XLOC_068372	ITM2C;CAB39	chr2	119428290	119477406	7.39031	6.07227	7.21831	5.78978	5.58944	4.76348	6.7465	5.66049	4.66358
XLOC_068373	ITM2C;CAB39	chr2	119428290	119477406	6.62247	2.26337	6.65952	3.64582	4.06563	3.04157	3.94054	2.74151	2.47852
XLOC_089552	IYD	chr9	88581010	88847482	1.27901	1.366	2.39316	0.363439	0.377802	0.369243	1.40381	0.737528	0.603832
XLOC_090026	IYD	chr9	88581010	88847482	1.31577	4.27318	1.72204	3.44108	3.01938	3.88017	3.44527	3.64544	3.08163
XLOC_091442	IYD	chr9	88581010	88847482	0.0	0.455597	1.19151	0.136529	0.47421	0.315042	1.09252	0.151253	0.0
XLOC_091443	IYD	chr9	88581010	88847482	0.0	0.0	0.0	0.0	0.0	0.0	0.0	0.270388	0.0
XLOC_091444	IYD	chr9	88581010	88847482	0.0	0.0	0.0	0.0	0.0	0.740073	0.0	0.0	0.0
XLOC_091445	IYD	chr9	88581010	88847482	0.931435	0.557106	0.0	0.166955	0.509506	0.289823	0.590539	0.0930949	0.894792
XLOC_062448	IZUMO2;VRK3	chr18	56775642	56812048	3.84503	6.21038	2.40631	1.24077	0.902584	0.558888	0.419166	0.616083	0.604793
XLOC_037767	JAM2	chr1	10112546	10136934	9.19431	9.57208	12.09	12.899	13.3597	13.5689	12.2866	12.3607	13.3929
XLOC_038874	JAM2	chr1	10112546	10136934	2.69131	3.21809	3.15615	0.482189	1.57208	1.11368	1.93523	1.33808	0.586706
XLOC_038875	JAM2	chr1	10112546	10136934	1.86184	3.33769	1.45485	1.33358	1.15527	0.959969	1.49199	0.735899	0.48614
XLOC_038876	JAM2	chr1	10112546	10136934	1.72045	1.02953	1.68295	0.424245	0.607445	0.626503	0.707776	0.778548	0.602105
XLOC_037766	JAM2;ATP5J;GABPA	chr1	10024695	10112493	19.7402	21.6687	19.0661	22.2473	22.8088	22.6126	24.7421	19.0341	23.07
XLOC_038266	JAM2;ATP5J;GABPA	chr1	10024695	10112493	23.9191	29.0836	30.1538	45.9142	40.7363	43.1174	49.3009	54.1865	50.7141
XLOC_038856	JAM2;ATP5J;GABPA	chr1	10024695	10112493	0.0	4.38911	0.0	1.64467	3.37657	2.62749	5.39412	4.27051	3.81828
XLOC_038857	JAM2;ATP5J;GABPA	chr1	10024695	10112493	3.75729	1.87215	1.95854	1.45874	1.17142	1.94465	1.3532	1.49617	1.748

XLOC_038858	JAM2;ATP5J;GABPA	chr1	10024695	10112493	0.0	1.45425	0.0	0.799025	0.570489	1.09349	1.25055	0.324445	0.424817
XLOC_038859	JAM2;ATP5J;GABPA	chr1	10024695	10112493	3.3354	4.43568	4.35055	2.82487	3.43282	3.2787	3.731	2.20045	2.56212
XLOC_038860	JAM2;ATP5J;GABPA	chr1	10024695	10112493	8.4592	8.4175	0.0	7.06297	4.55859	4.33661	8.88505	3.58109	8.07164
XLOC_038861	JAM2;ATP5J;GABPA	chr1	10024695	10112493	3.5809	3.20155	4.18606	3.02295	2.6096	3.68827	1.91331	2.4836	1.84282
XLOC_038862	JAM2;ATP5J;GABPA	chr1	10024695	10112493	0.0	6.42293	12.5952	6.26774	7.7458	4.36493	13.1386	4.09991	2.7846
XLOC_038863	JAM2;ATP5J;GABPA	chr1	10024695	10112493	3.71583	4.43389	14.4925	5.64876	8.24133	7.58231	5.12561	3.95307	7.71384
XLOC_038864	JAM2;ATP5J;GABPA	chr1	10024695	10112493	2.77386	2.90211	5.42136	3.35455	3.56353	3.87304	6.35302	3.30826	4.1114
XLOC_038865	JAM2;ATP5J;GABPA	chr1	10024695	10112493	5.76347	6.31378	4.50324	6.19208	5.36192	6.73344	5.98069	4.93227	4.34658
XLOC_038866	JAM2;ATP5J;GABPA	chr1	10024695	10112493	2.36228	4.94591	4.31186	3.03503	3.63467	3.51502	3.86153	3.31116	3.3026
XLOC_038867	JAM2;ATP5J;GABPA	chr1	10024695	10112493	6.40918	11.4644	14.9868	3.4479	6.27381	7.11878	3.19409	2.41901	3.85992
XLOC_038868	JAM2;ATP5J;GABPA	chr1	10024695	10112493	0.0	3.50901	1.5295	2.45361	2.73097	1.81577	1.91399	3.47781	1.36257
XLOC_038869	JAM2;ATP5J;GABPA	chr1	10024695	10112493	1.85227	1.66031	5.78943	1.49267	1.72417	0.573097	3.46408	1.09832	0.806111
XLOC_038870	JAM2;ATP5J;GABPA	chr1	10024695	10112493	2.05565	1.84472	0.536098	1.04427	0.858436	0.78298	1.55865	0.755592	0.898434
XLOC_038871	JAM2;ATP5J;GABPA	chr1	10024695	10112493	1.52112	2.2732	2.37805	0.681263	0.473227	0.471604	0.272572	1.20747	1.5907
XLOC_038872	JAM2;ATP5J;GABPA	chr1	10024695	10112493	0.859483	1.54232	0.0	0.462237	0.470422	1.07023	0.623597	0.601828	0.525565
XLOC_038873	JAM2;ATP5J;GABPA	chr1	10024695	10112493	1.01796	1.21762	0.796155	0.456155	0.874427	0.844222	0.552316	0.304892	1.24423
XLOC_038267	JAM2;MRPL39;MIR155	chr1	10137097	10232502	47.5692	49.7927	48.4635	13.6486	14.2695	15.574	16.2388	13.2625	13.7429
XLOC_038877	JAM2;MRPL39;MIR155	chr1	10137097	10232502	4.58967	3.43198	4.78721	0.754242	0.598569	0.953052	1.18155	0.0766075	1.00267
XLOC_038878	JAM2;MRPL39;MIR155	chr1	10137097	10232502	3.5524	7.41859	5.54247	2.22359	1.08799	1.81366	1.22876	1.72081	1.84446
XLOC_038879	JAM2;MRPL39;MIR155	chr1	10137097	10232502	3.52411	2.63258	5.50782	1.57779	1.09424	1.09089	2.67224	0.871624	0.920499
XLOC_038880	JAM2;MRPL39;MIR155	chr1	10137097	10232502	3.09438	1.84964	2.41865	0.27714	0.120324	0.319714	0.415873	0.0	0.0
XLOC_038881	JAM2;MRPL39;MIR155	chr1	10137097	10232502	0.0	2.15646	0.0	0.861601	0.185926	0.494789	0.424788	0.0	0.0

XLOC_073208	JAZF1;TAX1BP1	chr4	68761998	68798713	0.583492	0.0	0.0	0.052313	0.0914496	0.303253	0.159573	0.292831	0.0
XLOC_076977	JOSD1	chr5	110829072	110839896	11.4532	21.9421	14.3154	11.2441	13.5427	11.6395	11.5382	8.9254	14.8749
XLOC_083375	JUND	chr7	4849458	4866166	0.0	0.0	0.0	0.270215	0.586655	0.46768	0.13518	0.149697	0.92021
XLOC_038401	KALRN	chr1	69537255	69687280	0.711004	0.51592	0.371008	0.223608	0.469346	0.632573	0.519436	0.137095	1.14452
XLOC_039611	KALRN	chr1	69104511	69105313	2.24444	4.69764	3.51026	0.0	0.0	0.0	0.0	0.0	0.0
XLOC_039612	KALRN	chr1	69107397	69108046	4.41479	8.35733	6.9021	0.0	0.0	0.0	0.0	0.0	0.0
XLOC_039613	KALRN	chr1	69108114	69108449	16.2769	15.7771	25.3872	0.0	0.0	0.0	0.0	0.0	0.0
XLOC_039614	KALRN	chr1	69378075	69378711	7.55791	9.48767	7.08935	0.135389	0.235147	0.0	0.27091	0.150005	0.0
XLOC_039615	KALRN	chr1	69378801	69379220	2.76587	9.08279	17.2739	0.247436	0.0	0.0	0.0	0.0	0.0
XLOC_039616	KALRN	chr1	69532237	69537205	0.300681	0.359961	0.235371	0.296668	0.212807	0.156693	0.138164	0.106197	0.23701
XLOC_039617	KALRN	chr1	69537255	69687280	0.0	0.0	0.851179	0.0	0.0	0.112782	0.0982976	0.0	0.0
XLOC_039618	KALRN	chr1	69537255	69687280	0.929041	0.0	0.0	0.083263	0.072601	0.0	0.0	0.0	0.162273
XLOC_039619	KALRN	chr1	69537255	69687280	1.0723	0.0	0.0	0.192176	0.0836997	0.222241	0.0	0.106984	0.0935989
XLOC_039620	KALRN	chr1	69537255	69687280	0.0	0.572462	0.0	0.0	0.0	0.197523	0.0	0.0	0.0
XLOC_039621	KALRN	chr1	69537255	69687280	0.0	0.0	0.0	0.104441	0.0909163	0.0	0.210238	0.0	0.0
XLOC_082788	KANK2;DOCK6	chr7	16842956	16870194	2.9017	3.07499	3.18583	1.06112	1.67802	1.55419	1.64827	1.68789	0.940876
XLOC_082787	KANK2;SPC24	chr7	16809590	16841021	35.6071	45.7511	43.9751	48.0767	39.4025	37.6593	42.1593	50.312	37.6362
XLOC_083418	KANK2;SPC24	chr7	16809590	16841021	3.87373	3.49416	5.6144	5.27065	4.96899	5.99606	4.31084	5.18303	3.7285
XLOC_070329	KANK4	chr3	83836269	83841773	13.8864	3.42955	3.55724	2.9498	2.85394	2.24408	3.38117	4.68195	3.58097
XLOC_071484	KANK4	chr3	83858427	83859487	0.801387	0.0	0.0	1.36481	0.752276	0.499118	0.800348	1.28354	0.490144
XLOC_044049	KCMF1	chr11	49794128	49838784	14.5882	12.4711	11.2699	32.5314	35.422	32.7577	40.1152	38.3409	40.9966
XLOC_076434	KCNA5	chr5	105519385	105611275	15.0261	12.3199	9.92993	0.336927	0.52257	0.316169	0.451972	0.375963	0.444202
XLOC_078518	KCNA5	chr5	105519385	105611275	6.71908	7.46325	4.50442	0.258069	0.0749914	0.099542	0.0868923	0.0	0.0838192
XLOC_078519	KCNA5	chr5	105519385	105611275	4.91483	3.9168	2.56084	0.293435	0.0	0.0	0.0	0.162327	0.142707
XLOC_078520	KCNA5	chr5	105519385	105611275	10.9278	5.71664	5.33952	0.0	0.212743	0.0	0.0	0.0	0.0
XLOC_078521	KCNA5	chr5	105519385	105611275	6.50128	4.85993	9.32093	0.0970945	0.16914	0.112282	0.195729	0.324274	0.0
XLOC_078522	KCNA5	chr5	105519385	105611275	1.56727	6.55778	12.2502	0.140369	0.12185	0.16192	0.140302	0.155425	0.273114
XLOC_078523	KCNA5	chr5	105519385	105611275	8.15444	1.94958	16.5705	0.730286	0.63366	0.505269	0.0	0.161609	0.426202
XLOC_076435	KCNA6	chr5	105700218	105815513	9.32288	8.74061	8.75391	12.2556	14.7503	13.2472	16.5796	11.1338	9.80653
XLOC_078528	KCNA6	chr5	105700218	105815513	5.12682	3.94333	5.15673	5.97445	4.07008	3.49868	3.92827	3.37538	2.68839
XLOC_078529	KCNA6	chr5	105700218	105815513	8.08146	4.8255	10.5157	3.85605	3.52889	3.31675	3.5446	2.63554	3.03904

XLOC_078530	KCNA6	chr5	105700218	105815513	5.46635	8.14756	8.52111	8.80762	8.68419	8.30299	1.83714	3.63804	3.29637
XLOC_078531	KCNA6	chr5	105700218	105815513	0.0	3.02439	7.90586	11.9104	5.2654	5.07795	11.3943	4.67091	5.2157
XLOC_078532	KCNA6	chr5	105700218	105815513	0.0	2.00969	7.00748	3.21181	1.21408	0.922995	1.38834	0.882449	2.1444
XLOC_078533	KCNA6	chr5	105700218	105815513	9.16746	2.73918	2.86545	2.29833	1.84918	2.26928	1.63339	1.81239	1.43638
XLOC_078534	KCNA6	chr5	105700218	105815513	6.98005	1.56434	2.72742	0.937553	1.76119	0.720317	0.934244	0.518034	0.911671
XLOC_078535	KCNA6	chr5	105700218	105815513	3.85257	1.72653	7.52532	2.93178	2.38932	1.78706	1.19921	0.950982	1.17333
XLOC_078536	KCNA6	chr5	105700218	105815513	0.0	1.77751	1.1622	0.798998	1.15663	1.99772	0.266576	0.885495	1.03662
XLOC_078537	KCNA6	chr5	105700218	105815513	1.72102	1.80152	0.67312	0.61701	0.403694	0.625032	0.780408	0.77469	0.451019
XLOC_078538	KCNA6	chr5	105700218	105815513	38.5421	30.6884	30.0946	67.3614	67.7811	73.0814	75.223	74.7718	77.4078
XLOC_055431	KCNC1;KCNC1	chr15	35296082	35303260	7.99092	4.41193	4.2269	2.04963	2.85191	1.53039	3.4518	3.56347	3.65925
XLOC_058176	KCNH1	chr16	74351646	74411561	2.43307	0.485336	0.317345	0.0363628	0.0	0.0	0.0	0.0	0.0
XLOC_059249	KCNH1	chr16	74187360	74190524	0.481104	0.0	0.0	0.0	0.0	0.0	0.0	0.0	0.0
XLOC_059253	KCNH1	chr16	74291976	74293370	0.584908	0.0	0.457652	0.0	0.0	0.0	0.0	0.0	0.0
XLOC_059256	KCNH1	chr16	74299236	74299742	0.0	0.62001	0.0	0.0	0.0	0.0	0.0	0.0	0.0
XLOC_059258	KCNH1	chr16	74306709	74307228	2.00089	0.0	0.0	0.0	0.0	0.0	0.0	0.0	0.0
XLOC_059263	KCNH1	chr16	74320649	74322325	0.0	0.0	0.372701	0.0	0.0	0.0	0.0	0.0	0.0
XLOC_059265	KCNH1	chr16	74323136	74323592	0.0	0.723625	0.0	0.0	0.0	0.0	0.0	0.0	0.0
XLOC_059266	KCNH1	chr16	74329724	74331478	0.0	0.0	0.0	0.0406202	0.0	0.0	0.0	0.0	0.0
XLOC_059268	KCNH1	chr16	74333370	74334783	1.15211	0.172334	0.0	0.0	0.0	0.0	0.0	0.0	0.0
XLOC_059269	KCNH1	chr16	74335499	74336285	1.15079	0.344079	0.0	0.0	0.0	0.0	0.0	0.0	0.0
XLOC_059270	KCNH1	chr16	74338166	74339892	0.0	0.137959	0.0	0.0	0.0	0.047965	0.0	0.0	0.0
XLOC_059272	KCNH1	chr16	74344563	74345246	0.0	0.0	1.07602	0.0	0.0	0.0	0.0	0.0	0.0
XLOC_059274	KCNH1	chr16	74346607	74348182	1.02044	0.0	0.0	0.0	0.0	0.0	0.0	0.0	0.0
XLOC_059275	KCNH1	chr16	74348334	74348928	0.0	0.0	1.29506	0.0	0.0	0.0	0.148096	0.0	0.0
XLOC_059276	KCNH1	chr16	74349247	74351502	0.344803	0.0	0.0	0.0	0.0	0.0	0.0	0.0	0.0
XLOC_041595	KCNH5	chr10	75212064	75311995	32.8132	38.6646	43.4528	23.3545	23.2476	20.3427	22.3316	22.9796	21.5118
XLOC_041596	KCNH5	chr10	75506730	75535900	15.2286	17.9678	12.5288	46.0776	49.6899	57.6204	64.6006	65.8489	35.4361
XLOC_041597	KCNH5	chr10	75561740	75579210	0.0	0.0	0.58119	4.15644	4.25692	3.19466	3.18141	4.32915	4.27053
XLOC_041598	KCNH5	chr10	75579294	75586651	0.943792	0.278939	1.10783	3.29856	4.39558	3.82362	3.61226	4.59678	6.30531
XLOC_041599	KCNH5	chr10	75589557	75615932	3.39847	0.391157	0.0	0.0703367	0.0410787	0.108911	0.0958989	0.131719	0.0457731
XLOC_041989	KCNH5	chr10	75506730	75535900	6.04397	6.31482	6.02076	3.80818	4.44611	3.55127	3.66117	3.75653	4.85236
XLOC_041990	KCNH5	chr10	75636550	75644087	1.66443	2.82942	4.55817	6.50878	5.19786	5.11428	6.13986	7.7195	7.19035

XLOC_042959	KCNH5	chr10	75312185	75312817	4.57298	6.83395	4.76601	3.14017	3.79368	1.73273	2.45817	3.93239	3.18807
XLOC_042960	KCNH5	chr10	75506730	75535900	5.05348	1.13309	1.97559	0.33956	0.984624	0.523122	1.13751	0.377294	0.661206
XLOC_042961	KCNH5	chr10	75536075	75536385	0.0	4.22897	0.0	2.11453	2.8736	1.44035	5.21711	1.81024	3.26316
XLOC_042962	KCNH5	chr10	75536915	75537456	1.88609	2.25408	1.47371	1.85753	3.6566	3.6949	5.53952	4.84429	5.58094
XLOC_042963	KCNH5	chr10	75616449	75617412	0.0	0.268532	0.0	0.0804745	0.0	0.0	0.0	0.0	0.0
XLOC_042964	KCNH5	chr10	75635551	75636355	0.0	0.334509	0.0	0.60147	0.436462	0.231801	0.302935	0.22311	0.976365
XLOC_064338	KCNJ16	chr19	61224542	61228471	5.66686	7.97588	7.19374	5.94176	3.84029	4.66325	2.62904	3.51894	3.55249
XLOC_065455	KCNJ2	chr19	61185013	61185757	0.0	0.368691	0.0	0.0	0.288405	0.127667	0.0	0.0	0.0
XLOC_041442	KCNN2;KCNN2	chr10	3366758	3464966	2.06814	1.23762	0.323695	0.593447	0.129832	0.516477	0.113477	0.291226	0.0361917
XLOC_053825	KCNQ3	chr14	9885958	9886647	4.0825	12.2039	4.25557	24.2594	20.3474	26.3237	23.6007	22.9999	26.5797
XLOC_053341	KCNV1	chr14	56648266	56659493	1.42582	2.34572	2.23091	0.639072	0.61389	0.444258	0.388994	0.500094	0.56079
XLOC_060288	KCTD10	chr17	65967123	66027349	30.2902	30.4984	33.9153	6.87911	10.9374	5.59423	8.61696	7.28062	11.4523
XLOC_061827	KCTD10	chr17	65967123	66027349	32.2579	26.6747	34.878	2.22035	4.59663	3.82376	6.11772	5.84104	5.60283
XLOC_060287	KCTD10;UBE3B	chr17	65925776	65951194	25.6447	20.8546	21.9756	14.1285	16.8388	15.8641	17.9138	16.4191	23.2766
XLOC_062222	KCTD15	chr18	44380475	44421624	12.1831	11.0875	12.7159	22.5339	21.2941	23.4123	30.1072	40.8722	27.2654
XLOC_079744	KCTD8	chr6	64663183	64666390	0.0	0.0	0.0	0.545932	0.0950123	0.378519	0.0	0.242738	0.0
XLOC_081234	KCTD8	chr6	64667091	64667586	0.0	0.640178	0.0	0.0	0.0	0.441254	0.0	0.0	0.186347
XLOC_088162	KCTD9	chr8	73749536	73749681	0.0	0.0	0.0	6.5265	13.9369	13.1859	28.9857	11.2166	11.441
XLOC_088163	KCTD9	chr8	73749796	73750699	7.76135	6.09264	4.55271	7.65118	5.60845	5.73425	5.00506	5.81495	7.11611
XLOC_078714	KDELR3	chr5	110663599	110663834	0.0	5.3564	7.00117	3.23756	4.68292	1.80276	2.90959	5.82985	10.0181
XLOC_045918	KDM3A	chr11	48289791	48290316	1.96821	1.17602	0.0	4.93373	5.49119	4.46233	6.99651	4.27325	3.76731
XLOC_070455	KDM4A;MIR2415	chr3	102922384	102936149	16.0836	31.9083	14.0039	8.26255	3.8448	5.55687	7.25268	5.6081	6.98849
XLOC_078572	KDM5A	chr5	107744464	107746043	4.57904	6.24097	5.17557	6.56906	6.58256	7.14279	11.7041	10.1718	9.79045
XLOC_076953	KDM5A;CCDC77	chr5	107746148	107869290	22.4057	60.9448	97.8459	44.7983	49.4037	46.982	44.2822	33.2871	43.8764
XLOC_078573	KDM5A;CCDC77	chr5	107746148	107869290	0.0	0.874389	0.0	0.131015	0.569066	0.453621	0.655945	0.580948	0.382475
XLOC_078574	KDM5A;CCDC77	chr5	107746148	107869290	0.0	1.92606	0.629677	0.432909	0.503736	0.584884	0.292312	0.322299	0.421988
XLOC_078575	KDM5A;CCDC77	chr5	107746148	107869290	0.0	0.946195	0.618669	0.42534	0.309361	0.574701	0.359091	0.0	0.276417
XLOC_078576	KDM5A;CCDC77	chr5	107746148	107869290	0.0	0.463399	1.21191	0.138867	0.482242	0.320393	0.971887	0.307582	1.08081

XLOC_078577	KDM5A;CCDC77	chr5	107746148	107869290	0.0	3.12691	0.0	0.374804	1.29668	1.07792	1.48504	1.44407	1.82064
XLOC_092239	KDM6A	chrX	104078574	104200717	2.93652	3.89376	5.28959	1.85232	0.864304	0.988559	1.81334	2.49111	1.09567
XLOC_092558	KDM6A	chrX	104078574	104200717	12.2064	8.29551	7.74788	6.5514	4.94984	5.97456	6.48132	8.50284	5.87881
XLOC_093453	KDM6A	chrX	104077409	104078362	2.72756	3.80668	2.1334	0.896338	1.13697	1.4147	1.3181	2.09051	1.19112
XLOC_093454	KDM6A	chrX	104078574	104200717	2.31205	0.691525	1.20578	0.759864	0.783896	0.560074	0.770084	0.308692	0.202053
XLOC_093455	KDM6A	chrX	104078574	104200717	3.539	1.85217	2.07611	0.872237	1.03743	0.642521	0.802007	0.353889	0.463699
XLOC_093456	KDM6A	chrX	104078574	104200717	1.03413	1.85514	0.808718	0.741258	0.645863	0.857396	1.0282	1.34172	0.722164
XLOC_093457	KDM6A	chrX	104078574	104200717	0.0	0.950662	2.48635	1.42444	0.827269	0.98844	0.478778	0.951718	1.0176
XLOC_093458	KDM6A	chrX	104078574	104200717	2.19553	1.53287	1.43187	1.44379	0.77557	1.21876	0.97127	0.847	1.08877
XLOC_080154	KDR	chr6	72206029	72235674	5.31018	12.2232	8.2584	0.0265656	0.023267	0.0728915	0.127672	0.117448	0.0613392
XLOC_053801	KHDRBS3	chr14	7559552	7560767	0.0	0.0	0.0	0.306552	0.107103	0.0	0.0	0.0685614	0.0597826
XLOC_063933	KIAA0100;SPAG5	chr19	20601981	20643520	187.122	165.733	130.042	0.0671204	0.0	0.0779025	0.0684987	0.0376607	0.0327504
XLOC_038402	KIAA0226	chr1	71029629	71103119	7.47244	9.60718	6.28265	22.9577	26.7618	24.2144	25.876	25.0847	26.7507
XLOC_039622	KIAA0226	chr1	71025556	71026132	0.0	0.0	0.0	0.61907	1.47619	0.356752	0.308532	0.171062	0.752509
XLOC_039623	KIAA0226	chr1	71027377	71028406	1.65979	3.47496	1.29834	15.3977	18.3681	15.5896	16.0405	15.3635	16.2409
XLOC_039624	KIAA0226	chr1	71028564	71029404	3.17919	1.90129	3.31501	11.8703	15.0563	14.4971	15.5124	12.2666	12.3033
XLOC_039625	KIAA0226	chr1	71029629	71103119	9.29127	3.69732	4.83411	6.37115	7.85163	7.60892	7.01439	6.63515	8.05217
XLOC_039626	KIAA0226	chr1	71029629	71103119	0.0	3.81848	0.0	3.05131	3.79273	5.04492	7.17382	3.77726	6.85453
XLOC_041700	KIAA0317	chr10	86300486	86349670	48.054	37.8711	39.6483	1.78471	0.448627	1.11269	2.06449	1.39526	1.23596
XLOC_042098	KIAA0317;FCF1	chr10	86351579	86498888	1.32106	1.57322	5.1642	1.05174	1.95531	1.2689	0.831569	0.131398	1.32166
XLOC_062893	KIAA0895L	chr18	34933029	34933299	0.0	7.57795	4.95306	11.3935	11.4869	6.4175	6.86601	7.19836	8.74828
XLOC_062894	KIAA0895L	chr18	34933570	34934081	2.04618	0.0	4.79559	2.01486	2.21867	1.89689	1.0895	2.62264	1.95771
XLOC_062895	KIAA0895L	chr18	34935292	34936168	4.02652	1.80611	4.72362	1.98461	3.69491	2.50459	2.27617	1.80924	2.1093
XLOC_062896	KIAA0895L	chr18	34936279	34936623	0.0	3.46684	6.04344	2.42505	7.39937	3.94961	6.33	3.74057	6.36428
XLOC_070680	KIAA0907	chr3	14901171	14901827	0.0	0.433691	0.0	0.0	0.11291	0.0	0.130164	0.0	0.0
XLOC_070681	KIAA0907	chr3	14901885	14902783	0.0	0.0	0.0	0.175064	0.152595	0.0	0.0	0.0975624	0.0852856
XLOC_070682	KIAA0907	chr3	14904210	14904675	0.0	0.0	0.0	0.0	0.181744	0.0	0.0	0.0	0.0
XLOC_069843	KIAA0907;MIR1940	chr3	14886230	14900788	7.64848	3.23071	1.76038	8.59292	2.64711	7.53553	4.02936	9.04656	4.44781
XLOC_089480	KIAA1009	chr9	66126605	66138554	39.9975	20.9751	30.6013	42.422	49.2901	51.8846	56.0553	62.5991	66.2206
XLOC_089966	KIAA1009	chr9	66088111	66126519	15.0225	23.4014	18.5382	22.7032	23.4798	23.2843	24.247	24.4611	31.2956
XLOC_091139	KIAA1009	chr9	66138697	66139110	19.8153	34.6464	22.0975	35.9586	44.017	41.5009	46.5722	55.8551	74.6449

XLOC_041449	KIAA1191	chr10	4939218	5005613	3.37271	1.69819	2.52011	0.0	0.0	0.0	0.0	0.0325452	0.0282486
XLOC_039305	KIAA1524	chr1	53730502	53731469	0.0	0.0	0.0	0.880849	0.488884	1.48305	0.0809802	0.89337	0.0780392
XLOC_039306	KIAA1524	chr1	53738211	53739353	0.0	0.219741	0.574712	1.1195	0.402491	1.44949	0.400686	0.883194	0.321019
XLOC_039307	KIAA1524	chr1	53740417	53741650	0.0	0.0	0.526116	0.783703	0.263299	0.628747	0.734411	0.337117	0.293922
XLOC_039308	KIAA1524	chr1	53742759	53743214	0.0	0.0	0.0	0.652702	0.563208	0.749557	0.214275	2.62472	0.42237
XLOC_039309	KIAA1524	chr1	53745064	53745931	0.0	0.0	0.0	1.55299	1.59209	1.05673	1.56706	1.01772	0.355994
XLOC_065498	KIAA1715	chr2	20968971	20970230	2.14507	4.49146	2.79691	3.14073	2.18294	3.78734	2.14573	1.7913	2.43728
XLOC_086350	KIAA1797	chr8	23410928	23449817	13.3807	9.22823	10.2467	19.9729	49.1029	28.1053	53.3497	35.3877	58.8899
XLOC_066570	KIF17;SH2D5	chr2	132529205	132578241	10.6246	13.3542	13.311	6.05428	6.95862	6.97845	7.12161	5.96058	7.26569
XLOC_068648	KIF17;SH2D5	chr2	132529205	132578241	6.55852	2.93567	2.55884	3.226	3.77379	3.68882	1.99338	3.18688	1.13611
XLOC_068649	KIF17;SH2D5	chr2	132529205	132578241	2.35786	3.87731	9.21857	1.47883	1.83889	2.3197	0.744047	1.40966	1.02866
XLOC_068650	KIF17;SH2D5	chr2	132529205	132578241	13.323	5.2969	6.92508	1.9858	2.02801	3.16065	1.51419	2.55776	2.68369
XLOC_086954	KIF27	chr8	78505236	78508894	20.8826	14.8563	16.3381	65.1797	59.7783	60.5115	70.8913	71.6071	57.383
XLOC_088253	KIF27	chr8	78503776	78504091	4.58014	0.0	3.57076	0.819267	4.17933	0.930772	1.5584	4.39028	1.18579
XLOC_088254	KIF27	chr8	78505236	78508894	0.0	9.01149	14.1363	13.0002	9.12043	16.4993	12.6075	11.4414	12.4893
XLOC_088255	KIF27	chr8	78505236	78508894	7.37133	7.15808	8.63989	12.7051	15.0107	13.1141	18.0558	12.0213	13.9553
XLOC_063819	KIF2B	chr19	3851568	3894746	43.2291	37.7226	31.972	34.508	21.6977	34.7161	27.765	34.7192	21.4483
XLOC_064357	KIF2B	chr19	3851568	3894746	26.0188	10.3532	6.76679	2.34617	3.23653	6.10235	9.86789	2.41912	2.98
XLOC_064358	KIF2B	chr19	3851568	3894746	9.9035	29.5238	34.7379	13.2899	12.0217	10.5499	9.63622	6.62137	11.1014
XLOC_064359	KIF2B	chr19	3851568	3894746	16.5865	15.1429	13.6567	6.02476	5.32351	6.34043	4.27433	4.88936	4.65158
XLOC_064360	KIF2B	chr19	3851568	3894746	11.9165	7.82905	11.1677	2.77285	3.6819	3.91993	3.78416	2.34029	2.69163
XLOC_064361	KIF2B	chr19	3851568	3894746	11.1209	9.2659	11.185	4.55696	5.21996	5.44236	5.0491	2.54474	4.51147
XLOC_052106	KIF3B	chr13	62290599	62291683	0.0	0.0	0.0	1.5394	2.0155	2.26917	2.26884	1.71959	2.04635
XLOC_052107	KIF3B	chr13	62300764	62301297	0.0	0.0	0.0	1.89703	2.23999	1.98563	1.37053	0.380392	0.670473
XLOC_057903	KIFAP3	chr16	38362157	38377914	0.0	0.276881	0.0	0.254027	0.0322375	0.195975	0.171107	0.280939	0.0
XLOC_057904	KIFAP3	chr16	38458897	38494700	1.83219	0.0	0.716534	3.9909	2.07042	3.69916	1.32361	2.31955	1.69315
XLOC_058128	KIFAP3	chr16	38395999	38415499	0.918418	0.549323	1.19051	5.7989	2.79779	4.02739	2.24773	4.60623	2.8088
XLOC_058129	KIFAP3	chr16	38445384	38453566	21.8391	26.4303	24.1146	94.6717	55.4926	75.5247	82.4721	121.163	66.3514
XLOC_058130	KIFAP3	chr16	38498818	38523699	10.8361	16.8669	15.2806	20.769	26.1663	24.7117	23.7708	22.3002	23.7265
XLOC_058940	KIFAP3	chr16	38443070	38443506	0.0	0.775452	0.0	2.32372	0.200281	1.06652	0.456431	1.01754	1.5782
XLOC_058941	KIFAP3	chr16	38445098	38445262	0.0	9.95109	0.0	18.7677	7.07088	3.27683	0.0	8.60638	5.66315

XLOC_058942	KIFAP3	chr16	38458897	38494700	4.50628	2.69536	0.0	3.71565	2.25425	2.99184	2.04187	3.33399	1.25948
XLOC_058943	KIFAP3	chr16	38494918	38495592	2.80072	0.418596	1.09475	1.63075	1.30823	1.01375	0.125744	1.39156	0.610442
XLOC_058944	KIFAP3	chr16	38496297	38496883	0.0	0.504431	3.95761	0.453485	0.524436	0.871282	0.603104	0.668604	1.02907
XLOC_058945	KIFAP3	chr16	38497908	38498495	8.42028	5.53585	0.0	3.16702	4.83989	4.34637	4.36264	3.00186	4.98679
XLOC_062274	KIR2DS1	chr18	62977800	62998018	26.3067	38.6623	20.4582	23.6389	28.2828	25.0684	28.647	30.1591	38.9885
XLOC_063369	KIR2DS1	chr18	62977800	62998018	3.16888	1.42061	1.23842	0.709521	0.369512	1.63672	1.55974	1.25676	0.690252
XLOC_079763	KIT	chr6	71707971	71810756	11.1677	15.653	9.81904	1.2586	1.28284	1.15192	1.58087	1.32922	2.40163
XLOC_079764	KIT	chr6	71831059	71877841	39.2027	50.4773	35.9316	132.595	108.188	124.443	127.316	162.28	118.495
XLOC_079765	KIT	chr6	71908890	71984963	75.5079	87.5904	84.3522	127.974	83.3788	114.502	107.869	158.342	95.2579
XLOC_080152	KIT	chr6	71815108	71823922	18.0403	20.3448	13.7552	30.2368	25.3991	27.3791	32.5425	36.0829	35.0849
XLOC_080153	KIT	chr6	71879300	71908791	21.2162	19.5095	16.4134	59.4702	47.9973	55.9263	58.0927	72.3972	65.1244
XLOC_081327	KIT	chr6	71707971	71810756	2.74088	2.45963	3.21648	0.0614264	0.160957	0.284716	0.187038	0.13738	0.419284
XLOC_081328	KIT	chr6	71707971	71810756	0.0	0.726056	0.0	0.652702	0.375472	0.0	0.214275	0.0	0.0
XLOC_081329	KIT	chr6	71829874	71830197	0.0	1.30055	0.0	2.7302	0.996318	0.887179	1.48884	1.25691	0.376587
XLOC_081330	KIT	chr6	71831059	71877841	5.92362	2.83373	1.85277	2.6538	3.60354	3.3125	3.95173	2.1247	2.3775
XLOC_081331	KIT	chr6	71878041	71878225	19.7732	23.8469	0.0	18.402	8.66164	7.91454	6.11178	3.54187	10.2208
XLOC_081332	KIT	chr6	71878585	71878850	6.63604	7.91399	0.0	7.14268	13.4776	7.3663	5.49797	8.75148	11.4159
XLOC_081333	KIT	chr6	71879300	71908791	43.8523	5.23528	20.5286	13.4445	18.977	12.3392	23.5043	11.4102	15.8202
XLOC_081334	KIT	chr6	71879300	71908791	4.609	9.61952	7.18642	12.7797	9.46107	12.1748	13.3255	8.83339	14.3182
XLOC_081335	KIT	chr6	71908890	71984963	7.11574	9.72727	11.9251	2.09521	2.2227	2.4237	2.482	2.43575	2.48502
XLOC_076747	KITLG	chr5	18265058	18360369	5.8942	5.57397	4.24444	4.03879	3.24235	3.75767	3.22325	4.53937	2.70419
XLOC_063214	KLC3	chr18	53406312	53406666	25.7474	9.87549	11.4768	12.8275	14.3504	12.3864	14.5959	23.8437	25.4552
XLOC_062444	KLC3;ERCC2	chr18	53406785	53418663	33.5437	16.2153	20.1556	17.5891	25.9841	19.3561	25.1291	22.8975	31.8332
XLOC_050527	KLF6	chr13	44729788	45002029	16.08	21.8926	19.7673	54.5342	39.2908	53.9719	52.3518	72.2383	36.3415
XLOC_050825	KLF6	chr13	44729788	45002029	7.02221	4.27956	4.77674	4.48908	5.30658	4.75799	4.03806	5.14468	4.31636
XLOC_051749	KLF6	chr13	44729788	45002029	3.62863	3.66422	4.25938	9.51704	7.7212	8.96516	6.59028	5.79114	6.07127
XLOC_051750	KLF6	chr13	44729788	45002029	0.645464	1.15858	1.51507	2.02529	2.02224	2.21319	1.46931	2.71895	1.80573
XLOC_072703	KLHDC10;C4H7orf45	chr4	94735367	94874978	4.6762	4.83465	13.6348	3.7793	2.06336	3.25938	2.22253	3.67815	2.17029
XLOC_047839	KLHL1	chr12	44254574	44313772	24.9172	27.6157	24.2501	13.6961	14.5498	14.2192	13.7894	13.5472	16.3654
XLOC_047840	KLHL1	chr12	44328892	44600322	1.94151	2.34163	2.30665	0.308385	0.69626	0.62869	0.514794	0.140277	0.472145
XLOC_049294	KLHL1	chr12	44314072	44315228	0.0	1.08331	4.53328	1.03889	1.30404	0.977914	1.71229	0.65319	1.13951
XLOC_049295	KLHL1	chr12	44316794	44317756	0.0	2.95753	2.81274	0.322298	0.983801	1.11917	0.977702	0.0898862	1.17784

XLOC_049296	KLHL1	chr12	44318651	44319007	0.0	1.0863	2.84051	0.651228	1.11453	1.11496	2.20016	1.05735	3.46538
XLOC_049297	KLHL1	chr12	44622589	44623207	0.0	0.0	1.22767	0.140673	0.366335	0.162264	0.281196	1.40181	0.136852
XLOC_060312	KLHL22	chr17	74519772	74523677	0.0	0.0	0.0	3.03905	10.3162	1.47775	5.7569	5.10395	12.5576
XLOC_065535	KLHL23	chr2	26628458	26636647	1.43954	2.17388	1.87401	7.97676	8.54975	6.75256	7.44017	9.10627	8.05438
XLOC_066201	KLHL23	chr2	26636819	26640549	19.5088	10.64	10.237	109.138	94.7833	93.4993	107.422	107.092	105.688
XLOC_038443	KLHL24	chr1	84036600	84284613	2.00014	0.498744	1.04359	3.84035	3.7432	4.37282	4.0301	4.08123	4.38889
XLOC_039738	KLHL24	chr1	84036600	84284613	1.88118	0.0	0.0	0.168428	0.145889	0.387936	0.334885	0.185845	0.654884
XLOC_039739	KLHL24	chr1	84036600	84284613	0.0	0.0	0.0	1.0375	0.299451	0.398173	0.343515	0.381385	0.504188
XLOC_039740	KLHL24	chr1	84036600	84284613	0.0	0.0	0.0	0.666104	0.191525	1.01967	1.09245	0.486753	0.64648
XLOC_039741	KLHL24	chr1	84036600	84284613	0.0	0.0	0.0	0.446515	0.582714	0.773862	0.448788	0.372138	0.217376
XLOC_044241	KLHL29	chr11	75348479	75390477	2.4447	7.06836	8.5453	10.3544	10.438	9.62291	6.98337	12.0616	8.97028
XLOC_044242	KLHL29	chr11	75390669	75405183	1.52526	5.76273	5.56976	7.74621	7.48016	8.79586	7.25291	10.8604	8.09606
XLOC_044243	KLHL29	chr11	75440945	75454817	1.90065	0.0	0.012363	0.0	0.0	0.0	0.0	0.0	0.228281
XLOC_044244	KLHL29	chr11	75461622	75470006	30.7979	26.1006	37.9366	7.47325	6.20011	5.0471	9.32072	6.56641	7.43477
XLOC_044245	KLHL29	chr11	75486812	75510312	53.1554	63.6243	54.7376	128.387	111.857	121.678	131.602	160.24	98.6326
XLOC_044246	KLHL29	chr11	75512349	75513085	0.0	3.43447	3.59262	0.0	0.0	0.0	0.406343	0.452138	0.399727
XLOC_044247	KLHL29	chr11	75513125	75515684	0.0	1.15125	1.50546	0.392713	0.526326	0.0998024	0.522717	0.192302	0.588288
XLOC_044248	KLHL29	chr11	75520559	75573878	6.81683	3.15092	7.49093	3.0251	3.58884	3.56093	1.7438	2.07619	2.85229
XLOC_044249	KLHL29	chr11	75588073	75628878	115.878	97.3845	84.6603	44.3215	47.2279	48.6935	59.2838	67.4538	45.457
XLOC_044910	KLHL29	chr11	75300251	75348376	41.9988	58.2458	43.5862	174.232	147.039	163.165	171.762	224.662	154.551
XLOC_044911	KLHL29	chr11	75409907	75422564	24.8232	13.4607	14.5679	5.00778	3.69137	3.81817	4.48414	2.02604	3.39426
XLOC_044912	KLHL29	chr11	75431730	75439557	0.707807	0.211778	0.276959	0.0	0.0	0.0	0.0	0.0	0.0
XLOC_044913	KLHL29	chr11	75440945	75454817	5.01326	10.8176	8.75925	2.86891	1.80359	2.59889	1.9311	3.25078	1.81076
XLOC_046573	KLHL29	chr11	75297773	75299036	0.0	0.195704	0.511848	0.879749	0.819812	0.815671	0.595602	1.11534	0.915087
XLOC_046574	KLHL29	chr11	75300251	75348376	0.0	1.49587	1.30401	1.19537	1.68502	2.75627	0.596359	0.82631	0.5813
XLOC_046575	KLHL29	chr11	75300251	75348376	3.19261	0.952762	2.49139	1.14214	1.47055	2.28649	2.77651	3.72674	1.65959
XLOC_046576	KLHL29	chr11	75300251	75348376	0.970169	1.1605	1.51757	0.782507	1.06106	1.30781	1.84397	1.64757	0.677725
XLOC_046577	KLHL29	chr11	75405365	75405846	0.0	0.667825	0.0	2.00119	1.72914	2.07041	1.58197	3.29923	1.16608
XLOC_046578	KLHL29	chr11	75428072	75428240	0.0	61.9375	23.1393	108.018	63.2021	143.032	88.4547	100.175	90.7334
XLOC_046579	KLHL29	chr11	75459727	75461290	1.02915	0.615803	1.6106	1.79936	1.49282	1.97987	0.986342	0.827083	1.12519
XLOC_046580	KLHL29	chr11	75461622	75470006	17.8486	8.52007	8.35466	2.87294	3.55279	1.45796	4.62737	1.72894	3.08906
XLOC_046581	KLHL29	chr11	75470643	75470885	16.6024	9.9073	12.9498	5.98009	4.96277	5.84372	3.38353	4.64085	12.8369
XLOC_046582	KLHL29	chr11	75574010	75574602	3.32935	1.49243	3.90304	1.3417	1.55187	1.20312	0.297514	1.15423	0.57997

XLOC_046583	KLHL29	chr11	75574709	75574948	0.0	0.0	0.0	0.0	1.28071	4.31088	2.09271	0.0	1.47355
XLOC_046584	KLHL29	chr11	75575145	75576320	7.81888	3.82714	3.33651	1.97529	1.72498	1.18121	0.71108	0.783566	0.931902
XLOC_046585	KLHL29	chr11	75576389	75577151	5.98242	2.8618	4.67782	1.92963	1.4928	0.867257	0.215681	0.834349	0.521956
XLOC_046586	KLHL29	chr11	75577234	75577456	0.0	0.0	0.0	0.0	0.781023	1.05525	0.0	0.0	0.903563
XLOC_046587	KLHL29	chr11	75588073	75628878	148.891	186.57	179.831	497.877	456.655	512.626	481.311	489.2	655.276
XLOC_046588	KLHL29	chr11	75588073	75628878	3.7723	6.19888	4.42122	1.68867	0.585056	2.13915	1.67864	0.558956	0.820726
XLOC_046589	KLHL29	chr11	75588073	75628878	3.40561	1.27324	3.32987	0.457859	0.865466	0.441746	0.463362	0.170348	0.743758
XLOC_062249	KLK4	chr18	57354674	57390116	8.61602	8.266	5.62301	38.2488	36.3296	38.0622	40.2425	40.18	40.8983
XLOC_063278	KLK5	chr18	57390764	57393163	1.29061	2.22063	1.01009	13.8021	10.1097	11.623	13.8619	18.3241	12.3695
XLOC_076934	KLRF1	chr5	100654496	100674257	10.0451	13.6544	8.30797	17.2773	20.4839	17.1123	14.3122	9.5177	19.9314
XLOC_076935	KLRF1	chr5	100680050	100719688	1.96537	0.823778	0.239391	0.21945	0.192189	0.286658	0.168165	0.123223	0.669334
XLOC_078423	KLRF1	chr5	100654496	100674257	5.28919	0.0	2.0648	3.31253	4.48506	4.61422	1.16096	1.81222	4.13172
XLOC_078424	KLRF1	chr5	100654496	100674257	2.46446	2.20777	0.0	3.74893	4.37563	5.57054	1.73654	1.69237	2.56845
XLOC_078425	KLRF1	chr5	100654496	100674257	11.413	13.6538	0.0	15.5528	10.1424	9.14989	4.55144	13.6139	11.7609
XLOC_078426	KLRF1	chr5	100654496	100674257	8.97214	2.6782	7.00117	4.04696	10.0348	2.70414	3.637	4.99702	5.39435
XLOC_078427	KLRF1	chr5	100654496	100674257	0.0	4.03928	7.54531	1.90209	5.68956	3.18538	3.43515	1.90692	2.52094
XLOC_078428	KLRF1	chr5	100654496	100674257	0.0	2.06068	3.59262	3.91083	4.97762	3.07497	4.06343	2.26069	2.99795
XLOC_078429	KLRF1	chr5	100654496	100674257	0.0	1.07554	0.0	2.57907	3.8631	4.41639	3.73648	1.74543	5.92686
XLOC_078430	KLRF1	chr5	100654496	100674257	0.0	3.12031	4.0792	3.74671	4.75715	4.24319	2.20546	4.48745	5.86353
XLOC_078431	KLRF1	chr5	100654496	100674257	1.88964	5.36849	2.21693	4.99587	7.16099	6.56518	2.99439	3.39886	5.28062
XLOC_057891	KMO	chr16	35983314	36037627	51.69	41.1247	34.7869	35.4114	31.0953	30.9445	41.1222	46.0809	33.4321
XLOC_058907	KMO	chr16	36037822	36038446	6.20188	5.56079	4.84762	1.94414	2.89345	2.88354	3.60986	4.30614	4.32325
XLOC_058908	KMO	chr16	36038503	36039164	1.43652	3.00573	1.12297	2.05882	1.90063	1.93086	2.57811	2.28298	2.88016
XLOC_058909	KMO	chr16	36040274	36040754	2.24288	2.00967	1.75185	1.20443	1.04064	1.84599	0.793339	1.7649	0.974727
XLOC_057892	KMO;FH	chr16	36040966	36092255	10.8682	11.7708	9.59447	81.0037	79.5715	79.3944	76.6098	74.5573	94.0484
XLOC_058116	KMO;FH	chr16	36040966	36092255	9.79678	6.92624	6.19521	19.7863	27.4069	24.4937	22.2429	13.4304	29.44
XLOC_058910	KMO;FH	chr16	36040966	36092255	6.87876	6.15292	5.36204	13.5805	23.261	13.8746	5.68411	6.46827	13.6049
XLOC_070078	KNCN;MKNK1	chr3	100162480	100207785	8.30322	9.88039	10.1289	4.97637	4.19494	4.18609	5.69166	5.3225	5.86576
XLOC_038527	KPNA6;RBP1	chr1	130582490	130599788	0.0	0.0	0.0	0.548045	0.566232	1.03988	0.608198	0.446362	0.728956
XLOC_040214	KPNA6;RBP1	chr1	130607459	130608092	0.0	0.0	0.0	0.817454	0.828143	0.314385	1.36286	1.20747	0.265117
XLOC_062446	KPTN;NAPA	chr18	54888919	54992560	2.19561	1.45715	0.712476	0.690647	0.239179	0.780714	0.0975795	0.129306	0.14863

XLOC_063248	KPTN;NAPA	chr18	54888919	54992560	0.0	0.0	0.0	0.107379	0.0	0.124098	0.0	0.0	0.0
XLOC_063249	KPTN;NAPA	chr18	54888919	54992560	0.0	0.270234	0.706787	0.0	0.0	0.0939587	0.0	0.0	0.0
XLOC_063253	KPTN;NAPA	chr18	54888919	54992560	0.880409	0.0	0.688647	0.0	0.0	0.0913442	0.0	0.0880478	0.0
XLOC_063254	KPTN;NAPA	chr18	54888919	54992560	0.0	0.383044	0.0	0.0	0.0	0.0	0.0	0.0	0.0
XLOC_063256	KPTN;NAPA	chr18	54888919	54992560	0.0	0.0	0.0	0.0	0.0	0.122885	0.0	0.0	0.0
XLOC_063257	KPTN;NAPA	chr18	54888919	54992560	0.0	0.0	0.0	0.170196	0.0	0.195984	0.0	0.0	0.0
XLOC_063259	KPTN;NAPA	chr18	54888919	54992560	0.0	0.0	0.0	0.285535	0.0	0.0	0.0	0.0	0.0
XLOC_063260	KPTN;NAPA	chr18	54888919	54992560	0.0	0.0	4.2858	0.245564	0.211435	0.563106	0.0	0.0	0.0
XLOC_063261	KPTN;NAPA	chr18	54888919	54992560	6.58832	0.0	0.0	0.0	0.0	0.0	0.0	0.0	0.0
XLOC_063262	KPTN;NAPA	chr18	54888919	54992560	1.88609	0.0	0.0	0.0	0.146264	0.194468	0.0	0.0	0.0
XLOC_063263	KPTN;NAPA	chr18	54888919	54992560	0.0	0.0	0.0	0.0	0.0	0.0	0.0	0.145529	0.0
XLOC_063264	KPTN;NAPA	chr18	54888919	54992560	6.94188	0.0	0.0	0.623062	0.0	0.0	0.0	0.0	0.0
XLOC_064264	KRT20;KRT23	chr19	41729079	41802056	13.5642	16.1335	9.93408	40.9936	42.28	42.4067	49.2851	46.194	45.1763
XLOC_065159	KRT20;KRT23	chr19	41729079	41802056	6.72472	9.85685	8.41786	24.3551	22.9598	23.1239	26.3164	26.093	25.2185
XLOC_064261	KRT25;KRT26	chr19	41600317	41625582	0.0	0.0	0.193662	0.0887624	0.174977	0.257723	0.158879	0.0498754	0.0649869
XLOC_064262	KRT27;KRT28	chr19	41627241	41649555	0.0	0.291916	0.0	0.218711	0.191296	0.405913	0.133654	0.0490239	0.170697
XLOC_064263	KRT28;KRT10;KRT20;KRT20	chr19	41650689	41727132	0.0	0.627195	0.0	0.0	0.162548	0.0	0.0	0.0	0.182589
XLOC_064006	KRT31	chr19	42170604	42192278	9.29841	4.80528	8.28344	9.81136	7.57521	7.05468	9.68311	9.01355	9.63198
XLOC_064007	KRT31	chr19	42238627	42268389	0.0	0.0	0.0	0.0	0.0889227	0.118071	0.0	0.0	0.151058
XLOC_064271	KRT35;KRT32;KRT36;KRT14;KRT15;KRT19	chr19	42283070	42431763	0.0	0.540037	0.94164	1.89348	5.89875	2.97544	15.1131	5.19958	11.6039
XLOC_076777	KRT81;KRT86	chr5	27766138	27791679	23.6281	19.3787	18.3627	37.1533	32.2917	33.2089	34.8079	40.962	32.0324

XLOC_077482	KRT81;KRT86	chr5	27766138	27791679	0.0	2.57528	2.24477	3.34418	3.76197	3.53701	6.78953	2.52682	3.74113
XLOC_077483	KRT81;KRT86	chr5	27766138	27791679	1.82987	4.6516	6.44066	3.52601	4.0759	2.75228	4.55917	3.29258	4.31509
XLOC_077484	KRT81;KRT86	chr5	27766138	27791679	6.89133	3.50438	4.31313	4.88041	3.45326	3.1497	4.45155	2.97042	3.13234
XLOC_077485	KRT81;KRT86	chr5	27766138	27791679	8.4592	3.367	6.60352	3.53143	5.86104	4.91477	4.19575	3.85657	4.64735
XLOC_077481	KRT86	chr5	27765881	27766027	0.0	57.9358	50.5665	68.4945	75.6206	94.5666	46.2676	42.9759	65.6253
XLOC_076776	KRT86;KRT83	chr5	27742005	27765740	15.3037	14.6315	10.8007	31.5877	27.0092	27.4529	28.9045	36.062	25.468
XLOC_038790	KRTAP13-1	chr1	4774819	4776065	0.664441	0.993792	1.03967	2.50174	0.988651	1.93283	0.907184	1.53237	1.62635
XLOC_064265	KRTAP3-1	chr19	41862537	41896247	24.0956	28.185	26.3197	25.9221	14.8349	20.7671	22.2655	33.0783	15.4554
XLOC_065161	KRTAP3-3	chr19	41845866	41847828	8.01583	11.3926	9.72326	3.01894	3.20831	3.58684	4.39928	4.39477	4.45391
XLOC_064003	KRTAP4-7	chr19	41981385	42034652	9.48591	7.1389	12.8957	8.7486	8.34859	8.1014	6.75459	8.07362	9.14541
XLOC_064004	KRTAP9-1	chr19	42090710	42122273	17.6248	16.129	15.5121	10.3162	9.79275	9.55967	11.7552	12.1472	8.12006
XLOC_070028	KTI12;BTF3L4;TXNDC12;RAB3B	chr3	94612048	94944192	3.03755	2.41917	1.98984	1.08931	1.57644	0.841433	0.98788	0.318886	1.4342
XLOC_070029	KTI12;BTF3L4;TXNDC12;RAB3B	chr3	94612048	94944192	0.0	0.0	0.0	0.666174	0.957623	0.76483	0.0	0.0	0.431005
XLOC_070030	KTI12;BTF3L4;TXNDC12;RAB3B	chr3	94612048	94944192	22.5804	26.5	22.6876	11.3207	12.7469	12.3602	13.8838	16.1343	9.28592
XLOC_070402	KTI12;BTF3L4;TXNDC12;RAB3B	chr3	94612048	94944192	8.65671	12.8606	12.0282	1.57537	1.69868	1.19398	1.05947	1.25592	1.81137
XLOC_070403	KTI12;BTF3L4;TXNDC12;RAB3B	chr3	94612048	94944192	31.304	42.8935	51.816	16.0768	17.3905	18.6185	6.79044	12.7574	9.11213
XLOC_071695	KTI12;BTF3L4;TXNDC12;RAB3B	chr3	94612048	94944192	4.20252	4.02211	7.23209	1.05471	0.920206	1.04673	0.610041	0.420484	1.10143
XLOC_071696	KTI12;BTF3L4;TXNDC12;RAB3B	chr3	94612048	94944192	10.019	0.996538	5.21172	1.19477	1.28053	3.07282	1.15888	3.56764	2.02445
XLOC_071697	KTI12;BTF3L4;TXNDC12;RAB3B	chr3	94612048	94944192	0.0	3.27406	8.5583	0.993872	2.43585	3.29391	0.87584	0.0	0.0

XLOC_071698	KTI12;BTF3L4;TXNDC12;RAB3B	chr3	94612048	94944192	14.8548	1.47619	7.71955	2.21503	2.25406	2.51195	0.837899	0.47296	1.28096
XLOC_071699	KTI12;BTF3L4;TXNDC12;RAB3B	chr3	94612048	94944192	10.8048	12.9195	25.3282	5.8794	2.40418	3.25026	1.7298	1.98801	0.0
XLOC_071700	KTI12;BTF3L4;TXNDC12;RAB3B	chr3	94612048	94944192	8.64967	3.87545	6.75659	1.35492	2.34254	2.44845	1.14898	2.34194	1.69207
XLOC_083369	KXD1	chr7	4539779	4545689	1.22635	0.917162	1.91902	1.04448	1.10491	0.892138	0.223464	0.246092	0.536126
XLOC_041521	L2HGDH;MAP4K5;CDKL1;ATP5S	chr10	43221486	43440598	4.16189	6.08058	4.08002	7.06323	6.25069	7.32855	10.9218	9.30183	8.68538
XLOC_089843	LACE1	chr9	42254672	42264306	41.0954	28.6461	40.7792	12.9985	15.2713	13.3893	15.9911	11.8338	16.2792
XLOC_090669	LACE1	chr9	42251939	42252572	6.08448	6.8196	5.94501	3.95103	3.31257	3.45824	4.90629	4.98081	4.10931
XLOC_090670	LACE1	chr9	42252642	42253255	6.35167	1.8983	3.72338	3.6976	2.22184	2.62444	4.6892	3.3061	4.56541
XLOC_090671	LACE1	chr9	42253704	42254516	8.84057	7.93018	19.0117	4.75301	5.34656	4.80872	6.58476	4.07775	5.30463
XLOC_089344	LAMA4	chr9	38718286	38742056	26.2063	22.9603	14.8613	7.10269	8.1218	6.71015	7.32217	6.71067	14.775
XLOC_090596	LAMA4	chr9	38747939	38748691	3.64983	3.63735	2.85385	0.0	0.284562	0.125962	0.0	0.0	0.318419
XLOC_058174	LAMC1	chr16	65539039	65562851	16.0995	15.2637	11.1586	7.82346	6.28852	7.10754	8.73659	6.26351	6.42245
XLOC_079658	LAP3;MED28	chr6	38551284	38609004	50.48	44.5938	57.4504	44.5106	51.7148	50.5856	58.6061	59.1909	47.1361
XLOC_080854	LAP3;MED28	chr6	38551284	38609004	14.1924	6.52876	7.68377	8.51092	11.2468	10.8595	10.1546	6.96937	11.244
XLOC_080855	LAP3;MED28	chr6	38551284	38609004	8.74615	11.4994	25.9726	15.0371	16.9749	13.5383	14.6708	9.0005	12.1849
XLOC_080856	LAP3;MED28	chr6	38551284	38609004	11.354	6.11295	15.3957	8.27776	11.7282	12.1034	13.8247	7.2022	9.92208
XLOC_080857	LAP3;MED28	chr6	38551284	38609004	6.08686	8.48571	6.34054	14.8938	21.3743	20.2744	22.6941	10.6045	21.708
XLOC_080858	LAP3;MED28	chr6	38551284	38609004	7.64046	6.83661	0.0	9.61639	14.8813	13.8474	13.7846	7.14804	9.19312
XLOC_080859	LAP3;MED28	chr6	38551284	38609004	23.1529	20.7178	12.0363	29.1457	46.2373	26.4163	32.2544	12.99	55.0493
XLOC_080860	LAP3;MED28	chr6	38551284	38609004	11.9943	17.8776	28.045	24.7142	53.8421	29.7063	32.034	21.0042	49.5578
XLOC_080861	LAP3;MED28	chr6	38551284	38609004	5.23352	1.56016	8.15869	8.89851	16.2536	14.3208	9.70399	7.47909	14.8844
XLOC_080862	LAP3;MED28	chr6	38551284	38609004	16.7971	3.13597	6.56095	11.0888	17.3926	16.2151	14.5196	9.10268	19.3545
XLOC_080863	LAP3;MED28	chr6	38551284	38609004	0.0	5.6422	7.37712	7.60909	10.1628	8.70631	8.22673	4.59984	10.102

XLOC_080864	LAP3;MED28	chr6	38551284	38609004	0.0	4.36968	3.8087	8.30408	16.3161	14.874	14.8952	5.13639	16.4325
XLOC_080865	LAP3;MED28	chr6	38551284	38609004	2.91041	2.60626	9.0872	7.81025	8.2844	13.1228	9.15631	6.53265	10.8525
XLOC_080866	LAP3;MED28	chr6	38551284	38609004	22.633	8.45281	5.89496	10.6386	15.7965	12.446	13.2613	6.70748	16.4145
XLOC_080867	LAP3;MED28	chr6	38551284	38609004	9.31821	4.17434	7.27771	10.007	19.8022	17.0073	12.5464	9.6138	16.8006
XLOC_080868	LAP3;MED28	chr6	38551284	38609004	4.8852	5.9795	9.73106	6.4114	12.9958	10.4418	9.70102	5.04575	13.2105
XLOC_080869	LAP3;MED28	chr6	38551284	38609004	1.45385	2.60737	9.09205	1.69296	3.62033	3.30667	3.26048	2.59878	2.2811
XLOC_080870	LAP3;MED28	chr6	38551284	38609004	0.0	5.42858	3.54884	7.32745	7.962	6.01311	3.48606	2.18208	4.3212
XLOC_080871	LAP3;MED28	chr6	38551284	38609004	1.81837	3.26286	3.55572	2.36308	1.98971	1.60332	3.04812	2.45408	3.17634
XLOC_080872	LAP3;MED28	chr6	38551284	38609004	9.7878	18.1299	21.4123	22.7829	25.1856	24.4116	34.7999	27.6293	20.4386
XLOC_080873	LAP3;MED28	chr6	38551284	38609004	4.64415	4.85446	5.44084	14.7546	14.3563	14.8045	20.9148	14.1475	11.2993
XLOC_080874	LAP3;MED28	chr6	38551284	38609004	12.2743	8.90743	13.7029	12.7182	12.7957	12.4851	15.331	10.0628	9.16066
XLOC_080875	LAP3;MED28	chr6	38551284	38609004	17.9443	10.7128	21.004	12.141	17.3938	15.3235	18.1849	14.1582	13.8713
XLOC_076361	LARGE	chr5	72203331	72298721	5.34658	5.19127	7.29725	9.81127	8.58648	9.80201	7.7222	9.52782	7.26257
XLOC_076873	LARGE	chr5	72158522	72168212	7.00137	11.5375	9.51035	24.2237	23.9951	24.1551	25.4004	24.6372	29.2045
XLOC_076874	LARGE	chr5	72300057	72305148	0.38603	0.231015	0.604213	0.242317	0.212094	0.40175	0.282561	0.194205	0.304023
XLOC_076876	LARGE	chr5	72513540	72559711	13.2807	14.3349	16.2518	4.30062	6.78551	5.61085	6.5635	4.46862	4.70916
XLOC_078105	LARGE	chr5	72157678	72157842	0.0	19.9022	0.0	15.6397	7.07088	6.55367	9.87638	5.73758	8.49473
XLOC_078106	LARGE	chr5	72513540	72559711	0.0	0.778239	2.03519	0.466415	0.0	0.0	0.229	0.510545	0.226267
XLOC_078107	LARGE	chr5	72513540	72559711	3.0295	2.71643	2.36809	0.407017	0.942535	0.156541	0.271462	0.0	1.18806
XLOC_078108	LARGE	chr5	72513540	72559711	0.0	2.35574	1.5403	0.264738	0.23075	0.102099	0.0	0.1967	0.171961
XLOC_076245	LARP4;LARP4;DIP2B	chr5	29569494	29758908	25.6193	28.9806	26.5851	38.6571	30.7394	35.2076	40.8644	45.3802	34.5436
XLOC_076246	LARP4;LARP4;DIP2B	chr5	29569494	29758908	12.0253	15.55	18.4751	15.0773	14.0162	14.174	13.9542	21.9253	15.8828
XLOC_077510	LARP4;LARP4;DIP2B	chr5	29569494	29758908	3.08945	2.40295	3.86753	4.48699	3.87256	3.08219	4.05292	5.70331	3.8357
XLOC_077511	LARP4;LARP4;DIP2B	chr5	29569494	29758908	3.14691	2.50934	3.28139	4.22998	3.93091	3.80516	3.41288	4.7109	3.93752

XLOC_077512	LARP4;LARP4;DIP2B	chr5	29569494	29758908	7.10449	4.23919	11.0848	2.8589	4.35181	2.90185	2.45656	5.50659	3.99633
XLOC_077513	LARP4;LARP4;DIP2B	chr5	29569494	29758908	4.85334	11.5755	7.56656	6.9464	4.0528	2.46292	4.11207	5.10395	2.9301
XLOC_077514	LARP4;LARP4;DIP2B	chr5	29569494	29758908	5.20988	5.18942	4.07144	3.42121	3.9102	4.48059	6.04435	3.95292	4.68782
XLOC_077515	LARP4;LARP4;DIP2B	chr5	29569494	29758908	0.0	3.21699	0.0	0.964013	2.2834	3.31675	4.25352	2.63554	3.74035
XLOC_077516	LARP4;LARP4;DIP2B	chr5	29569494	29758908	0.608532	1.6384	1.9045	1.74581	2.05013	1.77082	2.4397	2.50349	1.96868
XLOC_077517	LARP4;LARP4;DIP2B	chr5	29569494	29758908	2.1689	2.59136	6.77677	2.91197	5.20234	3.57181	3.64892	2.5623	4.14843
XLOC_077518	LARP4;LARP4;DIP2B	chr5	29569494	29758908	1.06755	1.59609	3.33945	1.91326	1.33332	1.77011	1.73607	1.70427	1.58416
XLOC_077519	LARP4;LARP4;DIP2B	chr5	29569494	29758908	0.0	5.82432	0.0	7.05218	2.17587	3.91443	7.8594	5.40858	6.69862
XLOC_077520	LARP4;LARP4;DIP2B	chr5	29569494	29758908	0.0	2.39101	0.0	2.50773	2.17034	1.64927	1.5994	3.35554	2.78502
XLOC_077521	LARP4;LARP4;DIP2B	chr5	29569494	29758908	0.0	11.5636	10.0774	11.5927	12.1661	10.4423	13.9518	10.975	10.0106
XLOC_077522	LARP4;LARP4;DIP2B	chr5	29569494	29758908	12.2771	13.4476	9.59119	11.7228	13.312	9.69523	14.3451	13.1132	13.3481
XLOC_050829	LARP4B;DIP2C	chr13	46749085	46929661	2.72561	2.73654	2.80152	8.80711	10.5597	8.59421	10.7636	11.4912	8.42514
XLOC_051763	LARP4B;DIP2C	chr13	46749085	46929661	0.876151	0.262032	2.05595	1.09937	0.821959	1.27266	0.556052	0.525824	1.07144
XLOC_051764	LARP4B;DIP2C	chr13	46749085	46929661	1.01228	0.908106	0.0	1.26999	1.02768	1.3642	0.640829	0.404368	0.972156
XLOC_084946	LARS	chr7	59320872	59321958	0.778945	0.232985	1.2187	0.488754	1.27985	0.808681	0.707508	0.233989	1.49744
XLOC_044859	LBH	chr11	69599730	69603216	36.5666	43.9738	45.6271	0.835267	0.893811	0.718215	1.89524	1.70163	2.08322
XLOC_044860	LBH	chr11	69603508	69606009	32.9534	30.9846	34.0606	0.970591	0.891345	2.00779	1.46207	1.78829	2.06221
XLOC_044861	LBH	chr11	69614956	69617926	23.6919	31.4138	25.5499	0.0	0.0	0.0	0.0	0.0	0.0
XLOC_046477	LBH	chr11	69607974	69608237	6.75526	16.1129	52.6567	0.0	0.0	0.0	0.0	0.0	0.0
XLOC_080280	LDB2	chr6	116626654	116629048	8.22867	8.85285	5.14495	8.40091	7.54436	9.85647	5.29615	7.50034	7.5977
XLOC_080281	LDB2	chr6	116634179	116673820	1.15749	0.779454	0.566299	0.298489	0.113785	0.331771	0.239367	0.277426	0.582923
XLOC_082228	LDB2	chr6	116551274	116552399	27.6591	32.8691	30.9945	31.0254	24.6883	28.022	35.1241	41.8584	27.3075
XLOC_082229	LDB2	chr6	116624444	116626509	11.7536	10.3239	7.41809	6.45998	6.24975	5.4061	5.27365	8.12637	6.56945
XLOC_082230	LDB2	chr6	116633018	116633596	1.72011	1.02803	2.68852	0.154033	0.400708	0.53261	0.153555	0.510795	1.04853
XLOC_082231	LDB2	chr6	116633840	116634094	0.0	0.0	0.0	0.0	0.0	1.48015	0.0	1.37711	0.0
XLOC_082232	LDB2	chr6	116634179	116673820	0.0	0.0	0.778609	0.089217	0.233269	0.412863	0.0	0.0	0.173847

XLOC_082233	LDB2	chr6	116634179	116673820	0.0	0.395645	1.03473	0.118565	0.0	0.0	0.119008	0.0	0.115418
XLOC_082234	LDB2	chr6	116634179	116673820	1.6959	1.52038	0.0	0.0	0.0	0.0	0.151461	0.0	0.295392
XLOC_082786	LDLR	chr7	16781204	16798375	3.63943	2.44375	3.91887	23.5256	34.2994	25.3038	53.9405	35.7685	47.8098
XLOC_058728	LEFTY2	chr16	29664055	29664824	0.0	0.353634	0.0	0.423904	0.922418	0.489944	0.853061	0.58924	1.03201
XLOC_065318	LGALS3BP	chr19	54034941	54036459	1.06319	1.74944	0.831923	0.714942	0.208377	0.386919	0.339548	0.0533914	0.139481
XLOC_065319	LGALS3BP	chr19	54038849	54039845	0.862558	0.773917	0.674697	0.30924	0.539533	0.358008	0.469344	0.603958	0.602788
XLOC_065320	LGALS3BP	chr19	54044568	54045156	0.0	1.00418	0.0	0.150461	0.0	0.173455	0.150097	0.332781	0.146332
XLOC_044140	LGALSL	chr11	62820215	62841287	0.342031	0.921118	1.07074	0.797486	0.402829	0.49848	0.187921	1.17071	0.778308
XLOC_055466	LGR4	chr15	58889310	58933726	14.0759	12.9459	16.6677	8.98443	8.45122	8.66647	8.80574	8.54883	9.15396
XLOC_055778	LGR4	chr15	58889310	58933726	4.28496	6.13818	6.38225	5.08273	6.11258	5.38465	6.60412	7.53192	6.53059
XLOC_056657	LGR4	chr15	58872268	58874764	2.47414	2.86891	2.17847	2.10789	1.7974	1.57788	1.98362	2.21144	1.40768
XLOC_056658	LGR4	chr15	58874932	58875747	3.30009	0.986754	0.0	0.295709	0.515069	0.797821	0.198646	0.658281	0.288028
XLOC_056659	LGR4	chr15	58875886	58876533	0.0	0.883309	1.15504	0.132351	0.689773	0.305475	0.397479	0.0	0.386361
XLOC_056660	LGR4	chr15	58876669	58877220	1.83815	0.549224	1.43633	0.329166	0.142601	0.189582	0.327481	0.363378	0.0
XLOC_056661	LGR4	chr15	58888868	58889161	0.0	0.0	0.0	5.70158	2.81382	7.53055	1.78797	3.53817	4.11731
XLOC_056662	LGR4	chr15	58889310	58933726	1.8861	2.25418	1.47371	1.68872	2.19397	0.777906	1.6787	0.931594	1.31316
XLOC_056663	LGR4	chr15	58889310	58933726	1.70793	2.0416	2.6695	1.37654	1.98955	2.11554	1.67757	0.845419	1.78483
XLOC_056664	LGR4	chr15	58889310	58933726	3.95058	3.37682	6.18226	2.02399	2.16742	2.17079	1.49253	2.09642	2.4677
XLOC_056665	LGR4	chr15	58889310	58933726	6.90818	6.60816	6.48077	3.96058	5.05615	3.28676	5.21246	3.70781	5.54134
XLOC_056666	LGR4	chr15	58889310	58933726	7.20639	17.1921	11.2362	5.17767	10.2745	7.26253	4.15456	5.40891	8.67339
XLOC_056667	LGR4	chr15	58889310	58933726	0.786577	1.64688	0.615307	0.775575	1.2923	0.734917	0.857225	0.472514	0.893485
XLOC_056668	LGR4	chr15	58889310	58933726	1.42521	0.852093	5.57067	0.638357	0.998383	1.32633	1.15129	1.13265	1.24242
XLOC_056669	LGR4	chr15	58889310	58933726	0.546463	2.61578	2.13792	0.979906	0.856711	1.47718	1.04685	0.82311	1.29035
XLOC_056670	LGR4	chr15	58889310	58933726	0.0	1.53496	0.0	0.689989	1.50029	1.06272	0.808559	0.25546	0.671711
XLOC_056671	LGR4	chr15	58889310	58933726	3.69942	2.21358	1.24059	0.61601	1.07726	0.714386	0.530505	0.583894	0.878224
XLOC_056672	LGR4	chr15	58889310	58933726	1.09837	1.97057	3.43571	2.36211	1.20002	1.47945	1.68602	1.20504	1.15037
XLOC_056673	LGR4	chr15	58889310	58933726	2.37547	6.38472	3.71031	0.850375	1.83502	2.19777	2.30523	1.63297	1.4446
XLOC_056674	LGR4	chr15	58889310	58933726	2.82105	1.2658	1.1035	0.821908	1.10426	0.879039	0.705629	0.706823	0.616433
XLOC_056675	LGR4	chr15	58940209	58940631	0.0	1.63274	0.0	0.0	1.26392	1.96354	1.19853	1.06964	0.474544
XLOC_055779	LGR4;LIN7C	chr15	58941049	59082107	32.1146	33.4912	27.4851	49.0528	31.3611	44.797	43.2408	78.944	32.8972
XLOC_045714	LHCGR	chr11	30880554	30881307	0.0	0.363125	1.89938	0.870561	0.378785	1.25752	0.984905	0.967741	1.16558
XLOC_044008	LHCGR;GTF2A1L	chr11	30772011	30880400	28.0207	27.6015	23.0431	59.0723	42.5037	52.272	54.7367	56.6939	45.4297

XLOC_048871	LHFP	chr12	22894901	22895279	0.0	0.0	0.0	0.0	0.0	0.335348	0.0	0.0	0.0
XLOC_048873	LHFP	chr12	22898004	22898216	0.0	0.0	9.45423	2.20032	1.7872	3.63185	3.84151	7.74181	1.03793
XLOC_048875	LHFP	chr12	22903635	22904865	0.0	0.0	0.527587	0.0604534	0.158419	0.140111	0.18411	0.0676104	0.176845
XLOC_048876	LHFP	chr12	22904918	22906482	0.0	0.153841	0.0	0.0	0.040318	0.0534721	0.0469356	0.0	0.0899516
XLOC_048877	LHFP	chr12	22954076	22954924	0.0	0.0	0.819162	0.0	0.0	0.0	0.0	0.0	0.0
XLOC_048881	LHFP	chr12	22965102	22966090	0.0	0.0	0.0	0.0	0.0	0.0903596	0.0	0.0	0.0
XLOC_048883	LHFP	chr12	22967747	22968997	0.0	0.0	0.0	0.0	0.0	0.0687781	0.0	0.0	0.0
XLOC_048884	LHFP	chr12	22970376	22971080	0.0	0.0	0.0	0.0	0.0	0.136936	0.0	0.263279	0.115418
XLOC_048886	LHFP	chr12	22973598	22974071	0.0	0.0	0.0	0.205182	0.0	0.0	0.405108	0.225372	0.0
XLOC_048887	LHFP	chr12	22974234	22976997	0.0	0.0	0.0	0.0995757	0.0436114	0.0867222	0.17814	0.0	0.0971975
XLOC_048890	LHFP	chr12	22983315	22983829	0.0	0.0	0.0	0.363262	0.314328	0.0	0.0	0.600256	0.0
XLOC_048891	LHFP	chr12	22998120	23000132	0.0	0.0	0.0	0.0349712	0.0918258	0.0	0.0356794	0.0784742	0.0
XLOC_048892	LHFP	chr12	23005228	23006508	0.0	0.0	0.0	0.0	0.0504663	0.0	0.0	0.0	0.112657
XLOC_048893	LHFP	chr12	23008140	23010274	0.0	0.10949	0.0	0.0656267	0.0861744	0.114258	0.133968	0.0368261	0.128089
XLOC_072617	LHFPL3	chr4	45869079	45873822	0.0	0.0	0.0	0.0	0.0	0.0	0.58974	0.218633	0.0
XLOC_072618	LHFPL3	chr4	45877928	45885702	0.0	0.0	0.0	0.0629156	0.0549465	0.0728983	0.127677	0.0703423	0.184035
XLOC_072619	LHFPL3	chr4	45899958	45903921	1.19057	1.42384	2.79285	0.0	0.0	0.0	0.0	0.0	0.0
XLOC_072620	LHFPL3	chr4	45914820	45923731	2.42596	2.35864	2.37264	0.0	0.0	0.0	0.0	0.0	0.0
XLOC_072621	LHFPL3	chr4	45986449	46034718	20.4402	22.5918	22.5533	0.79247	1.20764	0.50392	0.514371	0.254537	0.88197
XLOC_072622	LHFPL3	chr4	46115312	46172434	29.8193	35.4551	31.0688	41.6869	39.4822	43.2013	51.3858	56.5264	47.1564
XLOC_073158	LHFPL3	chr4	45810444	45826811	0.447129	0.535124	1.0497	1.88437	2.70096	2.37231	2.08405	2.60737	3.0121
XLOC_073159	LHFPL3	chr4	46115312	46172434	7.10102	8.29488	11.4186	12.5355	13.0648	13.2318	13.4524	14.3328	16.039
XLOC_073160	LHFPL3	chr4	46174473	46196323	3.01595	4.80943	8.80638	4.79508	6.19144	4.66679	4.53015	4.60022	5.6596
XLOC_073161	LHFPL3	chr4	46379940	46389772	12.9511	13.9076	8.74007	16.0408	12.078	17.4247	13.8843	15.9988	14.9705
XLOC_074137	LHFPL3	chr4	45809838	45810307	0.0	0.0	5.44071	1.45468	1.25617	2.14904	1.23028	1.5973	1.81595
XLOC_074138	LHFPL3	chr4	45885970	45886493	0.0	0.0	0.0	0.177167	0.306709	0.0	0.351671	0.0	0.516516
XLOC_074140	LHFPL3	chr4	45965831	45966482	0.0	0.0	0.0	0.0	0.0	0.151511	0.0	0.0	0.0
XLOC_074141	LHFPL3	chr4	45966795	45967337	0.0	0.0	0.0	0.0	0.0	0.0	0.167443	0.0	0.0
XLOC_074146	LHFPL3	chr4	45972556	45974393	1.72249	1.80382	0.673976	0.0386136	0.033787	0.0	0.0	0.0433039	0.0
XLOC_074147	LHFPL3	chr4	45975779	45976123	3.87427	5.77807	0.0	0.0	0.0	0.0	0.0	0.0	0.0
XLOC_074148	LHFPL3	chr4	45978743	45979451	0.0	0.0	1.02722	0.117704	0.0	0.0	0.0	0.0	0.0
XLOC_074149	LHFPL3	chr4	46113546	46115141	5.03119	3.91366	7.48014	3.6991	2.88	3.7149	3.81243	3.08349	3.69664
XLOC_074150	LHFPL3	chr4	46115312	46172434	0.0	1.45656	0.0	1.74821	1.48328	4.958	0.827512	0.466944	0.0

XLOC_074151	LHFPL3	chr4	46115312	46172434	9.09752	1.81007	7.09983	2.98341	3.72899	1.8631	4.75978	2.65954	4.99404
XLOC_074152	LHFPL3	chr4	46115312	46172434	13.8854	4.13977	5.41143	4.98449	2.08594	2.80003	5.15928	5.21989	5.96926
XLOC_074153	LHFPL3	chr4	46115312	46172434	0.0	1.27024	0.0	0.761814	1.29826	1.3003	1.82041	1.22882	1.47155
XLOC_074154	LHFPL3	chr4	46172501	46174377	3.78764	1.38514	2.96409	1.50951	1.61812	1.70778	1.96253	1.69305	1.43608
XLOC_074155	LHFPL3	chr4	46242841	46243884	7.35096	6.59583	7.66697	13.6902	12.5854	13.5641	13.8632	18.8016	14.629
XLOC_074156	LHFPL3	chr4	46374951	46375763	1.10507	0.660848	1.72834	0.594126	0.17247	0.34348	1.19723	0.330629	0.385791
XLOC_074157	LHFPL3	chr4	46378939	46379603	0.0	0.8537	0.0	1.02332	0.778031	1.3289	0.897134	0.851134	1.49384
XLOC_057945	LHX4	chr16	62854225	62929494	11.4777	4.88044	7.23014	3.63362	1.52893	2.18146	2.51886	3.52573	3.16939
XLOC_060303	LIF;OSM;GATSL3;TBC1D10A	chr17	71365085	71475532	74.7892	111.97	88.129	40.2646	48.5014	48.9827	55.5104	43.1012	45.8898
XLOC_061892	LIF;OSM;GATSL3;TBC1D10A	chr17	71365085	71475532	14.4158	12.895	16.8543	5.82473	9.73538	4.35914	4.15516	2.70448	10.5334
XLOC_065466	LIMS2	chr2	4717868	4801971	15.2922	9.95574	12.4544	0.227598	0.547014	0.527723	0.463309	0.40766	0.413126
XLOC_042096	LIN52	chr10	85891542	85917078	23.813	27.9211	23.5704	23.8778	17.7442	21.5993	23.5548	25.7705	18.1563
XLOC_042097	LIN52	chr10	85938574	85957818	59.1163	60.8903	52.8636	94.64	84.0004	86.9651	99.2802	113.46	99.4187
XLOC_043257	LIN52	chr10	85888653	85890253	9.02476	10.0502	6.27724	10.1148	9.98623	12.5662	12.1304	14.0546	11.0515
XLOC_043258	LIN52	chr10	85890801	85891445	2.97345	1.7775	1.16216	1.46483	1.8506	2.15139	0.933015	0.885494	1.03662
XLOC_082035	LIN54	chr6	99526530	99527001	0.0	0.0	1.80203	0.0	0.0	0.0	0.0	0.0	0.200496
XLOC_083769	LIX1;RIOK2	chr7	98886491	99141195	6.08473	7.28495	4.94401	7.71734	7.57031	6.79554	7.33707	7.9664	7.37332
XLOC_084674	LMAN2	chr7	40190890	40191350	35.8672	22.8505	26.1435	66.7616	40.4474	57.5132	57.7826	84.0513	63.9791
XLOC_073334	LMBR1	chr4	119070849	119073096	0.0	0.0	0.0	1.80553	1.45958	1.00703	0.9492	0.373639	0.456361
XLOC_075050	LMBR1	chr4	119053626	119054620	0.0	0.0	0.0	1.31741	1.75765	1.61487	2.43065	2.59452	1.28398
XLOC_075051	LMBR1	chr4	119055202	119055856	0.0	0.0	0.0	2.87073	1.92712	3.76508	1.17607	1.73595	1.65075
XLOC_075052	LMBR1	chr4	119073160	119073990	0.0	0.321589	0.0	2.12021	0.671572	1.44884	0.777211	0.536492	0.375505
XLOC_089268	LMBRD1	chr9	8900428	8967626	0.871654	0.0840724	0.652549	0.125951	0.111579	0.16328	0.0471847	0.0459678	0.0198221
XLOC_090229	LMBRD1	chr9	8900428	8967626	0.0	0.165684	0.433345	0.0248273	0.0217474	0.0864902	0.0253807	0.0278915	0.0242343
XLOC_082877	LMNB1	chr7	28437374	28441539	0.0	0.0	0.0	5.2098	7.36872	2.9555	3.70086	4.17596	10.0461
XLOC_082878	LMNB1	chr7	28445916	28447142	0.0	0.314579	0.822733	0.188546	0.410651	0.109033	0.0	0.0	0.367343
XLOC_082879	LMNB1	chr7	28458050	28468219	0.0	0.587826	0.576548	3.28114	2.14159	2.78775	1.71186	2.96973	1.46179
XLOC_084483	LMNB1	chr7	28453554	28454734	0.0	0.0	0.0	0.317038	0.110748	0.293865	0.578985	0.425327	0.247295
XLOC_082876	LMNB1;MARCH3	chr7	28385675	28436842	17.6759	20.6837	18.3703	16.7212	13.8916	14.8572	17.025	21.8433	16.3582

XLOC_083499	LMNB1;MARCH3	chr7	28385675	28436842	3.50551	2.78143	1.86264	2.06168	1.68771	1.62763	2.21223	2.35442	2.55149
XLOC_083500	LMNB1;MARCH3	chr7	28385675	28436842	27.3681	24.9408	22.139	10.2465	7.9873	10.3619	9.96003	12.8878	7.45156
XLOC_084481	LMNB1;MARCH3	chr7	28385675	28436842	2.42916	1.45256	0.0	0.10882	0.284222	0.37727	0.219046	0.241935	0.0
XLOC_084482	LMNB1;MARCH3	chr7	28385675	28436842	0.0	2.171	4.54237	0.0650607	0.227321	0.226131	0.593996	0.581769	0.126866
XLOC_079754	LNX1	chr6	70722929	70726863	5.15712	1.55189	0.0	0.0	0.0	0.0	0.0	0.0	0.0
XLOC_080146	LNX1	chr6	70766415	70792058	13.5361	40.1437	27.0952	8.97726	7.75972	10.8681	6.35454	9.11211	4.36573
XLOC_081313	LNX1	chr6	70765126	70766307	8.47968	11.2028	15.4792	3.73739	3.04261	3.22937	2.18517	3.96588	2.16172
XLOC_062277	LOC100124497	chr18	64887816	65155318	4.20971	17.8792	12.3723	29.4989	19.7525	19.8643	19.9336	18.8996	18.6009
XLOC_062278	LOC100124497;LOC509810	chr18	65155512	65213498	5.71769	9.47946	9.69583	15.6296	11.2637	12.4508	9.15892	13.1704	10.2026
XLOC_066498	LOC100125266	chr2	120860181	120970746	7.43711	7.56582	7.82001	7.88752	8.51575	8.2999	10.7694	9.65986	8.68182
XLOC_070330	LOC100190915	chr3	83898216	84040127	17.1518	24.1449	20.446	17.6706	16.3877	17.1176	14.0748	15.3318	18.6895
XLOC_053723	LOC100196897;CA2	chr14	79335624	79390773	7.69738	4.94631	5.26458	22.531	28.6046	27.7194	28.5652	21.3756	30.0195
XLOC_054919	LOC100196897;CA2	chr14	79335624	79390773	0.0	0.0	0.0	4.40252	5.26226	9.88952	7.51637	2.63297	5.42185
XLOC_054920	LOC100196897;CA2	chr14	79335624	79390773	0.0	0.0	0.0	1.8519	9.82102	6.24357	5.68407	2.58731	3.54911
XLOC_054921	LOC100196897;CA2	chr14	79335624	79390773	0.0	0.514013	0.0	2.46452	1.73652	1.77537	0.921332	1.02159	1.79748
XLOC_054922	LOC100196897;CA2	chr14	79335624	79390773	0.0	0.991982	5.18799	3.27031	2.80478	3.05889	1.44233	0.968698	2.59103
XLOC_050960	LOC100196900;PIGT	chr13	74478297	74618115	2.97227	1.35684	0.433809	6.93475	5.97829	7.48994	4.93393	4.63958	5.72341
XLOC_050961	LOC100196900;PIGT	chr13	74478297	74618115	4.11935	3.07636	9.28855	11.6626	10.8695	13.6015	10.8016	7.5077	14.2484
XLOC_052517	LOC100196900;PIGT	chr13	74478297	74618115	0.0	0.246235	0.644001	0.664135	0.643911	0.512689	0.298882	0.988692	0.503495
XLOC_052518	LOC100196900;PIGT	chr13	74478297	74618115	0.0	0.0	0.0	0.346697	0.33707	0.402277	0.274931	0.172808	0.263108
XLOC_052519	LOC100196900;PIGT	chr13	74478297	74618115	0.0	0.0	0.0	0.424487	0.888199	0.884191	0.257341	0.473289	0.496351
XLOC_052520	LOC100196900;PIGT	chr13	74478297	74618115	2.0388	4.26767	3.98623	11.8758	10.0301	9.82763	12.7209	14.3499	8.98884

XLOC_052521	LOC100196900;PIGT	chr13	74478297	74618115	0.418414	0.500773	0.982317	1.57582	1.14913	1.26256	0.879989	0.715406	1.17151
XLOC_052522	LOC100196900;PIGT	chr13	74478297	74618115	0.0	0.0	0.0	2.70837	4.56021	2.44438	1.00871	0.0	5.20386
XLOC_052523	LOC100196900;PIGT	chr13	74478297	74618115	0.0	0.382367	0.0	0.114586	1.09636	0.0	0.80575	0.254574	0.334673
XLOC_052524	LOC100196900;PIGT	chr13	74478297	74618115	0.903507	0.0	0.0	0.647814	0.706201	1.12473	0.982524	0.361324	0.63131
XLOC_052525	LOC100196900;PIGT	chr13	74478297	74618115	0.0	1.25013	0.93418	3.47889	4.44377	5.14992	2.61153	2.63604	3.60177
XLOC_064243	LOC100196902;DLX4;DLX3	chr19	37269336	37344092	33.7275	38.457	32.9035	96.8827	107.373	100.284	112.55	92.7402	104.662
XLOC_055592	LOC100271685	chr15	85206014	85285980	12.979	15.0205	8.7581	21.6345	26.3244	23.8382	25.7024	22.4997	18.7571
XLOC_064055	LOC100302389;C19H17orf109;SAP30BP;RECQL5	chr19	56529157	56604217	7.28161	8.31217	8.10804	9.23425	10.2226	9.86459	10.1233	12.1413	10.3753
XLOC_064321	LOC100302389;C19H17orf109;SAP30BP;RECQL5	chr19	56529157	56604217	4.62736	3.33034	1.43857	8.50566	10.1508	8.84478	9.00138	8.67016	10.6386
XLOC_083104	LOC100616526;LOC100616526	chr7	90371461	90375923	6.35012	4.9359	2.97897	0.0	0.0	0.0	0.0	0.0	0.0
XLOC_083105	LOC100616526;LOC100616526	chr7	90443677	90504864	37.8082	36.388	44.6333	35.2915	29.3481	29.3386	39.0027	29.8032	28.1112
XLOC_083752	LOC100616526;LOC100616526	chr7	90443677	90504864	7.20658	8.6658	6.44863	16.5587	14.8036	15.6781	18.8605	18.8486	17.2582
XLOC_085264	LOC100616526;LOC100616526	chr7	90426543	90428826	0.0	0.305451	0.2663	0.0610277	0.267167	0.0354218	0.186955	0.137021	0.238242
XLOC_085265	LOC100616526;LOC100616526	chr7	90442767	90443079	0.0	1.39172	3.63895	5.42745	2.838	4.26704	2.37928	1.34115	2.01374
XLOC_085266	LOC100616526;LOC100616526	chr7	90443140	90443537	27.1767	31.5444	30.638	44.565	32.0315	40.8203	27.1289	32.9634	27.223
XLOC_068552	LOC282685	chr2	128240827	128241817	0.0	0.0	0.0	0.778667	0.339622	0.0901437	0.236336	0.434475	0.22767

XLOC_062468	LOC407171;LENG8;LILRA4;RPS9	chr18	63108248	63387393	2.059	1.18161	3.49146	0.0	0.0	0.10717	0.0692732	0.138185	0.0
XLOC_041548	LOC503858	chr10	59323270	59372307	33.3171	29.3189	30.7536	29.1954	46.6584	36.447	33.8747	14.4079	53.4636
XLOC_072734	LOC509513	chr4	106651072	106655284	1.7502	1.98781	1.64363	6.74778	7.30438	6.99247	4.70877	7.32671	7.17073
XLOC_072735	LOC509513	chr4	106733905	106805027	1.8297	0.496574	0.113549	0.174768	0.152222	0.112703	0.0	0.0	0.119989
XLOC_073269	LOC509513	chr4	106662175	106678988	11.0866	20.8376	20.6071	31.5041	32.3972	31.1243	36.7983	40.4215	42.6625
XLOC_073270	LOC509513	chr4	106733905	106805027	0.0	0.224424	0.59126	1.03523	1.52954	1.14629	1.27327	1.29813	1.40261
XLOC_074810	LOC509513	chr4	106651072	106655284	0.0	0.865648	1.13195	1.42676	1.35224	1.34736	0.909374	1.15047	1.64089
XLOC_074811	LOC509513	chr4	106651072	106655284	0.0	0.730954	1.91151	2.84747	1.88982	2.7667	2.37228	2.16161	2.12602
XLOC_074812	LOC509513	chr4	106839221	106840139	0.0	0.284408	0.743832	1.02278	1.26321	0.690397	1.20549	0.570158	0.913554
XLOC_074813	LOC509513	chr4	106840307	106841015	0.0	1.17832	0.0	1.53015	0.511781	1.08759	0.827151	0.261397	0.572916
XLOC_074814	LOC509513	chr4	106841214	106841997	0.0	0.0	0.0	1.24328	0.992158	0.838347	0.417186	0.460989	0.706307
XLOC_074815	LOC509513	chr4	106847998	106848716	0.0	0.0	0.0	1.38728	0.804391	0.267086	0.464424	0.513612	0.112548
XLOC_074816	LOC509513	chr4	106859794	106860283	0.0	0.0	0.0	2.7342	1.3505	0.673702	1.54529	0.644338	0.379387
XLOC_057754	LOC510860	chr16	4886288	4964442	39.5342	36.5775	45.0702	23.5977	24.2254	22.1499	24.8626	22.5466	22.8858
XLOC_058005	LOC510860	chr16	4886288	4964442	7.22226	7.75472	6.62757	6.57161	5.72022	5.90902	7.26105	6.8908	6.11706
XLOC_058307	LOC510860	chr16	4886288	4964442	0.0	6.47034	2.41706	2.77007	2.37943	2.21927	2.96763	3.01601	2.68406
XLOC_058308	LOC510860	chr16	4886288	4964442	8.02584	3.59647	0.0	0.179625	0.46644	0.826894	1.2473	0.593775	0.349086
XLOC_058309	LOC510860	chr16	4886288	4964442	50.9282	30.3749	28.3606	6.53496	3.82088	9.53023	5.38879	6.82286	6.25434
XLOC_058310	LOC510860	chr16	4886288	4964442	4.62939	16.5908	23.5011	3.31437	4.65123	2.85634	4.49719	6.14186	6.43623
XLOC_058311	LOC510860	chr16	4886288	4964442	6.96821	7.27654	0.0	1.24626	1.60154	0.355895	0.301514	2.02687	1.80942
XLOC_058312	LOC510860	chr16	4886288	4964442	0.0	4.68156	9.79346	1.9642	1.20488	1.60542	1.0924	1.22153	0.543713
XLOC_055864	LOC513151	chr15	79286228	79328236	3.24176	8.98431	8.15289	23.9383	20.2957	21.3595	21.1724	23.8428	19.071
XLOC_063871	LOC513767	chr19	6306667	6325871	4.64281	3.86766	3.25239	12.1495	11.1404	12.086	7.24894	10.1712	7.20065
XLOC_063872	LOC513767	chr19	6334836	6340900	24.92	29.491	26.4274	26.214	21.7884	22.7402	23.8244	28.446	19.6968
XLOC_072714	LOC514257	chr4	99767337	99831581	5.30895	6.21282	4.13793	2.90424	3.10692	4.61815	4.30294	5.60439	2.50424
XLOC_072715	LOC514257	chr4	99877818	99912745	0.438987	1.95356	2.03635	2.40134	2.92955	2.1009	3.32091	2.08053	3.86324
XLOC_073256	LOC514257	chr4	99767337	99831581	2.66192	2.71503	2.36833	3.33048	5.16838	3.90382	4.72361	4.41913	6.51935
XLOC_072716	LOC514257;CNOT4	chr4	99929354	99987364	17.7846	30.7896	27.5475	55.2099	83.0277	64.1327	63.0013	30.5966	93.9766
XLOC_055907	LOC518623	chr15	83365030	83367314	12.6185	17.7117	9.60854	22.9235	20.5496	20.6308	14.3533	15.3089	21.0397
XLOC_057310	LOC518623	chr15	83372617	83373630	3.38167	1.5171	1.9839	0.6062	0.727216	0.350931	0.536857	0.507498	0.812413
XLOC_057311	LOC518623	chr15	83374793	83375640	1.04897	0.313667	0.820349	0.281999	0.081894	0.652313	0.189605	0.209373	0.366281

XLOC_055908	LOC518623;GLYAT	chr15	83375711	83464567	48.5391	52.4648	46.3156	28.5291	24.2359	25.0788	32.6617	35.007	26.2579
XLOC_057312	LOC518623;GLYAT	chr15	83375711	83464567	10.3145	3.07975	8.05376	12.4589	9.94412	10.3255	10.6532	11.1156	8.28301
XLOC_092566	LOC520057	chrX	106243772	106485420	1.03762	1.01553	0.512839	0.857739	0.244217	1.61795	0.0150405	0.208511	0.0915234
XLOC_093511	LOC520057	chrX	106243772	106485420	27.3314	9.77703	8.52094	47.9526	49.6239	46.4968	45.0098	57.1692	61.6893
XLOC_093512	LOC520057	chrX	106243772	106485420	8.577	7.6787	13.3822	13.1395	30.0966	24.1409	20.2295	27.9266	28.7342
XLOC_093513	LOC520057	chrX	106243772	106485420	0.0	0.0	0.0	8.0998	8.34378	4.20004	1.71976	3.91492	8.35696
XLOC_069954	LOC521656;C3H1orf87;HOOK1	chr3	86224223	86783560	18.353	19.2066	19.2162	7.76088	8.15299	7.85733	7.90942	7.25888	7.79728
XLOC_071536	LOC521656;C3H1orf87;HOOK1	chr3	86224223	86783560	106.484	109.339	101.392	297.204	319.449	315.155	250.809	350.796	328.717
XLOC_082792	LOC522449	chr7	17203452	17212463	1.79505	0.266402	0.0	0.0399124	0.0	0.0	0.0	0.0	0.0
XLOC_040477	LOC525947	chr1	136776269	136776920	0.0	0.0	0.0	0.0	1.14041	0.757553	0.394343	0.0	1.27749
XLOC_040478	LOC525947	chr1	136777057	136778138	0.0	0.0	0.0	0.210599	0.857822	0.406519	0.142257	0.0784145	1.02649
XLOC_040479	LOC525947	chr1	136789394	136789518	0.0	0.0	0.0	128.071	66.5089	268.144	190.324	199.413	191.941
XLOC_039240	LOC529036	chr1	44463935	44464291	10.924	8.69042	14.2025	48.8421	46.2529	47.9433	48.4036	42.6463	51.3506
XLOC_060307	LOC531152;TOP3B;TOP3B	chr17	73883900	73950673	0.827941	0.385263	1.03402	0.269446	0.117531	0.155304	0.170919	0.0382569	0.163587
XLOC_065842	LOC614396;TSSK3;HDAC1;FAM167B;MARCKSL1;LCK;BSDC1;BSDC1	chr2	122028533	122118712	10.5294	6.32108	11.2028	11.8525	8.65097	10.3107	11.8208	15.9498	11.311
XLOC_066506	LOC614396;TSSK3;HDAC1;FAM167B;MARCKSL1;LCK;BSDC1;BSDC1	chr2	122028533	122118712	11.7832	14.5054	14.2535	13.1737	14.0109	13.5084	13.9681	14.903	15.4534
XLOC_068448	LOC614396;TSSK3;HDAC1;FAM167B;MARCKSL1;LCK;BSDC1;BSDC1	chr2	122028533	122118712	1.83815	2.74612	5.74532	1.81041	1.71121	0.947908	2.29236	1.45351	1.12001

XLOC_068449	LOC614396;TSSK3;HDAC1;FAM167B;MARCKSL1;LCK;BSDC1;BSDC1	chr2	122028533	122118712	0.0	4.34081	0.0	2.6049	0.368436	1.97033	2.05603	1.85598	0.837172
XLOC_068450	LOC614396;TSSK3;HDAC1;FAM167B;MARCKSL1;LCK;BSDC1;BSDC1	chr2	122028533	122118712	0.0	0.0	0.0	2.86033	2.34307	5.27625	2.53134	10.6616	4.51782
XLOC_065740	LOC614993	chr2	91042965	91059437	94.8007	150.661	117.632	198.458	175.813	196.695	208.046	245.268	215.664
XLOC_065741	LOC614993	chr2	91070444	91071479	0.0	0.772685	0.0	1.3791	0.231736	0.205029	0.445197	0.760508	0.890186
XLOC_065742	LOC614993	chr2	91096898	91115711	13.3609	2.52415	2.75071	0.567341	0.275264	0.219118	0.127923	0.634301	0.0
XLOC_065743	LOC614993	chr2	91118176	91120491	1.45385	6.08387	1.1365	0.0	0.0	0.0	0.0	0.0	0.0
XLOC_065744	LOC614993	chr2	91124600	91125308	4.75881	11.3504	3.70974	0.0	0.722931	0.0	0.0	0.0	0.0
XLOC_065745	LOC614993	chr2	91134216	91136488	5.58008	6.5643	0.970345	0.66253	0.180964	0.343087	0.0564814	0.542241	0.365073
XLOC_066401	LOC614993	chr2	91061508	91070227	6.98912	5.09855	4.22691	2.54287	2.93201	2.71761	2.27139	2.27633	2.71961
XLOC_068096	LOC614993	chr2	91030289	91031190	0.972779	0.0	0.0	1.30768	0.531945	1.41218	0.35216	0.58304	0.679541
XLOC_068097	LOC614993	chr2	91042965	91059437	1.74502	0.521486	1.36376	0.312517	0.406455	0.180123	0.311415	0.34537	0.151945
XLOC_068098	LOC614993	chr2	91060473	91061280	1.11358	0.998895	0.0	0.598695	1.30339	0.461475	0.70365	0.333139	0.971873
XLOC_068099	LOC614993	chr2	91061508	91070227	0.0	7.45408	0.0	0.0	0.0	1.24679	0.987494	1.13767	0.0
XLOC_068100	LOC614993	chr2	91137150	91137982	3.2169	0.961908	0.0	1.15306	0.669598	1.33345	2.03425	1.81873	0.748791
XLOC_042249	LOC616348	chr10	4888994	4889907	0.0	0.858867	1.4975	0.0	0.0	0.0	0.0	0.0	0.0
XLOC_076420	LOC618591	chr5	100762521	100821039	5.0699	4.73492	4.26033	14.7831	10.8881	10.9562	15.6664	15.8118	14.7884
XLOC_076936	LOC618591	chr5	100762521	100821039	4.36497	5.8739	2.87575	15.4699	15.2981	15.7127	15.3702	14.717	13.4963
XLOC_076937	LOC618591	chr5	100762521	100821039	7.1222	9.67855	10.5356	18.4243	22.4305	19.6132	21.9822	18.205	22.5289
XLOC_078432	LOC618591	chr5	100762521	100821039	2.09188	0.938319	2.45393	3.65539	2.85807	2.27653	3.02509	1.9834	2.6479
XLOC_078433	LOC618591	chr5	100762521	100821039	0.0	2.6486	3.46253	7.14887	6.75987	6.32131	6.43539	4.68917	6.90095
XLOC_078434	LOC618591	chr5	100762521	100821039	0.0	0.0	0.0	6.76388	6.14197	7.63914	10.6919	3.30476	6.50281
XLOC_078435	LOC618591	chr5	100762521	100821039	0.0	4.95394	0.0	1.49503	0.620346	0.834818	2.03031	2.3207	1.42642
XLOC_078436	LOC618591	chr5	100762521	100821039	3.03228	2.7152	0.0	3.25463	3.96205	3.10517	2.11553	2.36415	2.89132
XLOC_078437	LOC618591	chr5	100762521	100821039	0.0	0.0	0.0	1.94276	3.69524	4.94587	3.64932	2.0645	1.86999
XLOC_078438	LOC618591	chr5	100762521	100821039	0.0	0.855139	0.0	0.768688	0.440872	0.587207	0.501107	0.83915	1.24218
XLOC_078439	LOC618591	chr5	100762521	100821039	0.0	2.19094	0.0	5.9393	3.30549	5.18051	0.604295	0.688804	7.57747

XLOC_083520	LOC768323	chr7	29320210	29518942	10.5586	15.3737	13.5642	6.84527	5.39651	6.16881	4.52519	6.85848	3.77535
XLOC_092086	LOC768323	chrX	35887961	36076561	26.7075	18.9561	22.5915	6.57073	5.22723	6.45287	6.01736	4.74023	4.03273
XLOC_092087	LOC768323	chrX	36112101	36171260	13.5977	11.2972	15.5683	0.746316	0.287155	0.422915	0.670786	0.593531	0.622004
XLOC_092861	LOC768323	chrX	36076771	36077485	0.0	0.0	0.0	0.116437	0.101264	0.0	0.0	0.0	0.113354
XLOC_064002	LOC777598;LOC618938	chr19	41937302	41981193	10.5765	14.1316	11.1892	6.81977	7.20044	7.01382	9.08874	8.35773	7.52114
XLOC_064267	LOC777598;LOC618938	chr19	41937302	41981193	10.4946	13.8932	11.0375	35.9482	34.1225	33.1366	31.6003	35.2781	39.3567
XLOC_072732	LOC780846;PRSS58;LOC780933	chr4	106313497	106463823	22.182	26.0719	22.6534	20.011	18.9654	18.944	22.7876	21.8807	15.5322
XLOC_073267	LOC780846;PRSS58;LOC780933	chr4	106313497	106463823	4.06158	2.67296	3.68166	1.84371	2.63145	2.67845	2.96328	1.27152	1.94391
XLOC_074804	LOC780846;PRSS58;LOC780933	chr4	106313497	106463823	11.0059	9.8096	15.5272	8.54211	8.93709	8.21953	7.07668	6.34843	4.63662
XLOC_074805	LOC780846;PRSS58;LOC780933	chr4	106313497	106463823	10.2866	4.22847	3.01602	2.76495	3.70746	4.52444	3.35539	2.81501	3.14027
XLOC_074806	LOC780846;PRSS58;LOC780933	chr4	106313497	106463823	3.35513	4.00461	2.61791	4.80138	4.37408	1.71482	3.78281	2.60629	3.19599
XLOC_080200	LOC781988	chr6	86651438	86684665	111.061	187.674	157.875	175.438	223.049	260.504	232.877	216.971	295.835
XLOC_080201	LOC781988	chr6	86691710	86733379	2.79678	5.10444	4.4257	9.18989	9.0098	9.4979	9.72141	11.6148	7.99175
XLOC_081540	LOC781988	chr6	86651438	86684665	0.0	0.0	1.189	0.408727	0.354919	0.1572	0.272573	0.301867	0.0
XLOC_051740	LOC782061	chr13	43667767	43668882	0.0	0.0	0.0	0.0	0.0591104	0.0784302	0.0686379	0.151311	0.198028
XLOC_051741	LOC782061	chr13	43668940	43669345	0.0	0.0	0.0	0.0	0.449571	0.299429	0.255316	0.0	0.0
XLOC_051742	LOC782061	chr13	43669426	43670036	0.0	0.0	0.0	0.286313	0.0	0.0	0.0	0.0	0.278516
XLOC_079800	LOC785585	chr6	85587742	85779394	27.8397	37.6858	34.3543	37.7933	40.4471	40.4147	39.0534	37.7993	39.3249
XLOC_081504	LOC785585	chr6	85587742	85779394	4.52347	2.69775	10.58	6.06955	9.63454	9.19481	5.00753	10.846	10.9319
XLOC_050526	LOC785762	chr13	43785203	43803097	10.5239	6.64777	8.44518	0.0	0.162836	0.0	0.113854	0.0	0.0
XLOC_058149	LOC786597	chr16	45962858	46010415	0.52152	1.40461	1.42177	0.631721	0.710239	0.84121	0.478362	0.455624	0.390809
XLOC_065170	LOC787225	chr19	41920287	41920747	4.7823	2.14224	1.86739	3.63765	3.32445	2.45783	2.31974	3.05214	1.03862
XLOC_089588	LOC788572	chr9	104002747	104042659	3.58652	2.13109	2.32216	4.11336	4.0475	3.84933	4.07891	3.58734	3.43589
XLOC_056488	LOC788703	chr15	50649383	50652091	0.283561	0.0	0.0	0.534043	0.601421	0.324826	1.27373	0.399939	1.06738
XLOC_083449	LONP1	chr7	19808494	19822774	8.71454	7.23714	12.486	8.05561	5.9556	8.95417	14.536	16.4294	4.78268
XLOC_084327	LONP1	chr7	19808494	19822774	6.41346	0.0	10.0095	1.14718	0.984602	1.96837	3.62456	2.80697	3.0559

XLOC_062367	LONP2	chr18	16601463	16808425	53.4141	54.0738	64.0434	0.407731	0.223185	0.236701	0.115718	0.429345	0.298458
XLOC_062730	LONP2	chr18	16601463	16808425	0.0	28.3022	90.4211	0.0	0.0	0.0	0.0	0.0	0.0
XLOC_062731	LONP2	chr18	16601463	16808425	11.8415	4.71691	38.5486	0.0	0.0	0.0	0.0	0.0	0.0
XLOC_062732	LONP2	chr18	16601463	16808425	11.39	8.36	21.8644	0.0	0.0	0.107327	0.0	0.0	0.0
XLOC_062733	LONP2	chr18	16601463	16808425	5.38658	19.2688	46.1819	0.0	0.0	0.545605	0.0	0.0	0.0
XLOC_062734	LONP2	chr18	16601463	16808425	65.4408	17.7345	55.6408	0.0	0.0	0.0	0.0	0.0	0.0
XLOC_062735	LONP2	chr18	16601463	16808425	0.0	18.381	80.0793	0.0	0.0	0.0	0.0	0.0	0.0
XLOC_062736	LONP2	chr18	16601463	16808425	23.0106	12.5104	26.9933	0.0	0.0	0.0	0.0	0.0	0.0
XLOC_062737	LONP2	chr18	16601463	16808425	8.87393	7.94621	41.5581	0.264586	0.0	0.0	0.0	0.0	0.0
XLOC_062738	LONP2	chr18	16601463	16808425	20.2429	13.4964	17.0397	0.0	0.0	0.0	0.0	0.0	0.0
XLOC_062739	LONP2	chr18	16601463	16808425	23.9115	19.9942	37.3479	0.0	0.0	0.0	0.0	0.0	0.0
XLOC_062740	LONP2	chr18	16601463	16808425	41.1564	23.3597	28.9371	0.368419	0.0	0.21195	0.0	0.0	0.0
XLOC_062741	LONP2	chr18	16601463	16808425	37.6839	27.6891	61.0928	0.0	0.0	0.0	0.0	0.0	0.0
XLOC_062742	LONP2	chr18	16601463	16808425	28.1642	15.29	31.9877	0.0	0.0	0.0	0.0	0.0	0.222296
XLOC_062743	LONP2	chr18	16601463	16808425	15.813	20.4271	53.409	0.0	0.0	0.0	0.444022	0.0	0.0
XLOC_062744	LONP2	chr18	16601463	16808425	22.6394	12.8472	37.1337	0.0	0.0	0.232886	0.0	0.0	0.0
XLOC_062745	LONP2	chr18	16601463	16808425	40.3519	24.1809	105.345	0.0	0.0	0.0	0.0	0.0	0.0
XLOC_062746	LONP2	chr18	16601463	16808425	19.9462	25.7915	70.0359	0.0	0.0	0.339876	0.0	0.0	0.287893
XLOC_062747	LONP2	chr18	16601463	16808425	16.8698	15.8629	28.2878	0.0432178	0.0	0.100264	0.0440216	0.0	0.0
XLOC_062748	LONP2	chr18	16808749	16809119	30.621	26.3949	31.8551	0.60853	0.0	0.0	0.0	0.330185	0.0
XLOC_062749	LONP2	chr18	16809437	16810540	0.764938	0.686395	1.1968	0.0685675	0.0	0.079421	0.0	0.076607	0.0
XLOC_086458	LOXL2	chr8	71272721	71360973	28.1514	27.6818	25.6898	19.2382	21.036	18.5675	20.5811	16.3152	15.4713
XLOC_086459	LOXL2	chr8	71366621	71369752	15.2247	15.5522	14.1791	38.3506	34.2379	36.1439	39.1007	39.7885	38.9232
XLOC_086460	LOXL2	chr8	71371236	71373153	3.54715	4.29389	3.31424	7.69934	5.98544	6.55761	5.76105	5.86105	5.12974
XLOC_086926	LOXL2	chr8	71373778	71395796	9.65164	20.9508	17.6339	14.0108	11.8699	14.1137	6.9915	9.90696	12.1272
XLOC_088053	LOXL2	chr8	71272721	71360973	0.0	15.3625	8.60765	4.93375	8.72315	6.38111	8.88449	3.55895	5.72747
XLOC_088054	LOXL2	chr8	71373279	71373623	3.87427	1.15561	0.0	0.692873	1.1839	0.0	0.0	0.0	0.0
XLOC_088055	LOXL2	chr8	71373778	71395796	0.0	0.0	5.362	2.46919	1.55062	4.85611	0.568501	0.646928	2.95759
XLOC_088056	LOXL2	chr8	71373778	71395796	0.0	3.14688	0.0	1.886	1.42197	2.43407	2.77665	1.28991	0.228712
XLOC_071333	LPAR3	chr3	59431742	59432420	27.7919	10.3846	9.7771	18.7964	22.6119	24.4321	33.0726	30.383	20.5963
XLOC_071334	LPAR3	chr3	59432992	59433111	0.0	124.527	0.0	380.971	294.702	593.617	475.053	449.21	278.149
XLOC_071335	LPAR3	chr3	59433612	59433747	0.0	34.2192	89.6936	22.4924	15.3836	33.2296	55.9168	55.5261	28.9107
XLOC_059247	LPGAT1	chr16	73510108	73510598	0.0	0.0	0.0	0.0	0.336627	0.0	0.0	0.0	0.0

XLOC_069922	LPHN2	chr3	63289847	63381927	17.7456	17.8288	13.8586	16.7891	8.14192	13.7257	19.7072	32.372	21.4555
XLOC_071354	LPHN2	chr3	62923759	62923990	0.0	0.0	0.0	1.69841	4.20069	0.0	3.03912	1.74155	7.26728
XLOC_071355	LPHN2	chr3	63173133	63173326	16.5375	39.7615	12.992	44.228	40.0143	28.1248	47.7213	22.381	28.4451
XLOC_071356	LPHN2	chr3	63173664	63173774	0.0	0.0	0.0	636.399	288.195	143.306	0.0	344.136	636.413
XLOC_044971	LPIN1	chr11	86060987	86189999	8.25258	10.1755	9.51467	11.528	13.4346	12.888	14.0347	14.1966	17.2178
XLOC_046860	LPIN1	chr11	86058144	86058658	0.0	0.0	0.0	0.726524	0.628656	0.627043	2.52157	0.800342	0.882436
XLOC_046861	LPIN1	chr11	86059464	86059732	0.0	0.0	0.0	0.579637	4.86642	1.95793	3.75625	4.87776	5.56142
XLOC_046862	LPIN1	chr11	86060332	86060807	0.0	0.0	0.0	1.63114	1.76123	3.98365	2.61729	1.34395	2.77187
XLOC_086422	LPL	chr8	67480914	67492448	0.0	0.0	0.0	0.318657	0.398106	0.316791	0.0463481	0.0510075	0.222042
XLOC_086423	LPL	chr8	67494843	67502670	1.08067	1.13157	3.80515	2.08317	1.65194	2.30336	2.6127	1.68216	2.83538
XLOC_038430	LPP	chr1	79608220	79672000	1.1376	2.5272	0.445048	0.930551	1.32214	0.876664	0.835826	0.793896	0.967831
XLOC_038431	LPP	chr1	79704158	79761833	9.90668	12.7308	17.2205	17.1998	20.5856	18.2882	20.7446	15.0974	21.6672
XLOC_039702	LPP	chr1	79607436	79608084	1.4746	0.0	0.0	0.924573	0.803113	0.914573	0.661151	1.0248	0.257052
XLOC_039703	LPP	chr1	79699699	79700106	5.79752	1.73056	6.78807	0.518601	1.7842	3.56485	1.0135	0.282929	1.25692
XLOC_039704	LPP	chr1	79700327	79701285	0.903507	2.43188	2.82679	0.809767	1.0593	1.31218	1.14626	0.993634	1.10479
XLOC_039705	LPP	chr1	79702206	79703999	9.28636	6.21854	7.61311	9.91299	11.7955	10.0752	12.6103	11.6492	12.9993
XLOC_086571	LPPR1	chr8	92357746	92361561	0.0	0.180387	0.0	0.054061	0.118366	0.156923	0.248589	0.0607163	0.0263833
XLOC_087010	LPPR1	chr8	92326349	92356100	0.541134	1.44307	0.0	1.74271	3.84963	3.02992	3.12499	1.3171	4.0007
XLOC_088575	LPPR1	chr8	92356727	92357574	0.0	0.0	0.0	0.0939997	0.0	0.0	0.0	0.0	0.0
XLOC_088576	LPPR1	chr8	92362453	92363155	0.0	0.0	0.0	0.0	0.103472	0.137435	0.0	0.0	0.0
XLOC_088579	LPPR1	chr8	92366114	92366833	0.0	0.0	0.0	0.115401	0.0	0.0	0.0	0.0	0.0
XLOC_055904	LPXN	chr15	83227000	83251988	6.89284	2.35633	4.45147	30.3919	46.3285	35.1478	23.4164	18.0808	48.0662
XLOC_057295	LPXN	chr15	83223234	83225067	0.0	0.129152	1.01338	0.27094	0.270939	0.40419	0.276224	0.0434068	0.339895
XLOC_057296	LPXN	chr15	83226099	83226823	0.0	0.381693	0.998243	0.915072	0.895452	0.924976	0.574525	0.0	0.556809
XLOC_057297	LPXN	chr15	83227000	83251988	0.0	0.0	4.49282	3.09747	4.78943	4.082	0.482486	2.18674	4.46721
XLOC_057298	LPXN	chr15	83227000	83251988	0.0	0.0	0.0	2.58893	1.08152	2.17872	2.37391	0.676128	1.85858
XLOC_057299	LPXN	chr15	83227000	83251988	0.0	0.0	6.61773	9.17145	1.2671	3.41148	0.690479	2.36882	3.64372
XLOC_057300	LPXN	chr15	83227000	83251988	0.938689	1.12288	0.0	4.627	3.52076	3.60215	2.29522	1.87615	3.52497
XLOC_057301	LPXN	chr15	83227000	83251988	0.0	0.920409	0.0	4.13752	4.97572	2.84141	2.68697	3.0036	4.27639
XLOC_057302	LPXN	chr15	83227000	83251988	0.0	0.974167	2.5474	4.08745	3.00574	3.33865	3.11913	3.17298	2.54485
XLOC_072708	LRGUK	chr4	98726136	98736748	0.0	0.367431	0.0	224.006	211.462	243.504	290.747	314.79	210.221
XLOC_073251	LRGUK	chr4	98755086	98777301	0.0	0.262885	0.0	2.57844	2.34882	2.21556	3.46036	4.38193	3.30804
XLOC_073252	LRGUK	chr4	98778165	98817719	0.0	0.6576	0.286654	2.76156	2.58704	2.89783	3.31849	4.83063	4.64966

XLOC_074638	LRGUK	chr4	98726136	98736748	0.0	0.0	0.0	12.3222	8.11758	12.0375	13.5291	11.7762	11.2312
XLOC_074639	LRGUK	chr4	98726136	98736748	0.0	0.0	0.0	12.0914	13.1642	17.8044	15.9668	13.2596	10.4863
XLOC_074640	LRGUK	chr4	98726136	98736748	0.0	0.0	0.0	3.82832	4.83089	4.38699	5.24117	2.22894	5.69156
XLOC_074641	LRGUK	chr4	98736834	98737280	0.0	0.0	0.0	122.264	105.638	125.416	134.603	143.32	98.8421
XLOC_074642	LRGUK	chr4	98738117	98738677	0.0	0.536963	0.0	123.739	111.287	122.913	136.97	151.413	112.798
XLOC_074643	LRGUK	chr4	98739534	98739860	0.0	0.0	0.0	7.27981	7.50763	8.28263	6.95553	3.29527	9.25062
XLOC_074644	LRGUK	chr4	98740008	98740868	0.0	0.0	0.0	3.50595	2.73329	2.98815	3.25784	3.69985	3.59528
XLOC_074645	LRGUK	chr4	98741182	98741533	0.0	1.11416	0.0	4.67572	3.42727	3.42922	4.50657	1.44457	3.55348
XLOC_074646	LRGUK	chr4	98742250	98742719	0.0	0.693495	0.0	3.94841	3.58906	4.29807	2.46056	3.87916	4.64077
XLOC_074647	LRGUK	chr4	98743436	98744340	0.0	0.289738	0.0	1.56292	2.57343	1.70794	1.75385	1.83896	2.62268
XLOC_074648	LRGUK	chr4	98778165	98817719	4.97114	2.476	2.59013	2.67112	1.54485	2.22424	2.22144	2.13394	1.73199
XLOC_074649	LRGUK	chr4	98778165	98817719	3.92022	1.56259	1.02166	1.52187	1.62892	2.16349	2.35079	1.56001	1.59551
XLOC_053662	LRP12	chr14	62276223	62293003	0.0	0.359553	0.0	0.592646	0.800462	0.437207	0.7667	1.32671	1.2611
XLOC_070019	LRP8	chr3	93555049	93565009	176.993	131.913	107.059	42.0703	45.1506	41.506	46.6296	50.6249	52.1669
XLOC_071683	LRP8	chr3	93520506	93521322	3.29507	3.28419	1.71785	0.0984202	0.342863	0.113802	0.19835	0.109549	0.191728
XLOC_071684	LRP8	chr3	93521607	93522299	2.70639	0.0	0.0	0.121223	0.105387	0.0	0.0	0.134546	0.117997
XLOC_071685	LRP8	chr3	93522542	93524400	5.10365	1.27254	3.32828	0.0381369	0.233598	0.221257	0.0777701	0.256632	0.260482
XLOC_071686	LRP8	chr3	93525694	93527135	25.357	25.2865	25.1314	4.29425	4.41644	5.38923	2.87764	3.90349	3.15374
XLOC_076841	LRRC10;CCT2	chr5	43993550	44102026	0.823578	0.377601	0.736547	2.24892	1.93669	2.55084	1.35041	0.475111	1.92045
XLOC_041463	LRRC49	chr10	17722822	17724426	111.269	133.167	140.745	14.8836	9.47077	11.9217	13.6462	14.1299	18.1305
XLOC_041464	LRRC49	chr10	17796382	17843840	0.938236	0.187172	0.979091	1.09384	0.368424	0.488445	0.630391	0.566926	1.04024
XLOC_042377	LRRC49	chr10	17724537	17727086	39.9186	33.3927	27.456	4.39358	3.04029	4.03063	4.98832	4.96487	5.05604
XLOC_042378	LRRC49	chr10	17727153	17728574	0.572409	0.342487	1.34363	0.10264	0.0	0.0595026	0.260956	0.229846	0.100111
XLOC_042379	LRRC49	chr10	17728686	17730127	0.563489	0.50573	0.0	0.151562	0.132493	0.0	0.0513865	0.0	0.0
XLOC_042380	LRRC49	chr10	17796382	17843840	4.52787	4.73317	1.76826	5.87595	4.37599	6.05502	7.20437	5.78831	3.5417
XLOC_064242	LRRC59	chr19	36917591	36919760	0.0	0.279117	0.0	0.167297	0.219917	0.388159	0.19748	0.0723762	0.0629316
XLOC_053470	LRRC6;PHF20L1;KCNQ3	chr14	9550107	9870074	7.68463	2.29592	7.50531	0.0	0.297884	0.0	0.341754	0.0	0.0
XLOC_069937	LRRC7	chr3	75557129	75641329	6.29035	7.7586	7.51474	2.21585	2.49711	1.84625	1.71775	2.10165	2.86871
XLOC_070318	LRRC7	chr3	75516108	75556834	15.6998	19.0694	20.7398	12.5339	12.2707	13.2285	15.5046	18.2718	17.6596
XLOC_071453	LRRC7	chr3	75516108	75556834	0.0	2.20777	1.9245	1.32316	0.951225	1.51924	1.3024	1.20887	2.78251
XLOC_071454	LRRC7	chr3	75516108	75556834	2.49191	2.97999	6.81938	2.34421	2.0395	2.70852	1.90735	1.36452	3.04327
XLOC_071455	LRRC7	chr3	75516108	75556834	3.52593	2.8999	3.44743	1.34308	1.44713	1.37182	3.03666	1.76306	2.07891

XLOC_071456	LRRC7	chr3	75516108	75556834	6.97841	3.5774	7.01713	2.59086	3.27023	1.34364	3.87793	2.58845	2.61129
XLOC_071457	LRRC7	chr3	75516108	75556834	11.9943	0.0	18.6964	2.14907	4.977	3.6375	6.00638	1.13544	5.16231
XLOC_071458	LRRC7	chr3	75516108	75556834	7.37133	1.65187	5.75993	0.660012	1.14367	0.570178	1.64144	0.546451	0.962453
XLOC_071459	LRRC7	chr3	75516108	75556834	1.89957	3.12439	3.71428	0.936325	0.890514	0.393998	0.515973	1.32866	0.912367
XLOC_071460	LRRC7	chr3	75516108	75556834	2.40706	2.15647	1.87979	0.646209	1.11538	0.742184	0.636687	0.47263	1.67278
XLOC_071461	LRRC7	chr3	75516108	75556834	2.33156	1.13378	2.05297	0.679577	0.755462	0.789082	1.54937	0.645893	1.14806
XLOC_071462	LRRC7	chr3	75516108	75556834	2.82656	2.32511	2.76414	1.26691	0.829803	1.61468	1.92809	0.708203	1.3588
XLOC_071463	LRRC7	chr3	75516108	75556834	0.541143	1.29516	2.96399	0.194075	0.339365	0.843951	0.197477	0.380414	0.425947
XLOC_071464	LRRC7	chr3	75516108	75556834	3.35604	1.33894	1.16732	0.568466	0.76105	0.815031	0.989671	0.487836	0.717915
XLOC_071465	LRRC7	chr3	75516108	75556834	1.63089	1.94959	1.27466	0.584234	0.887124	0.842107	1.45828	0.484851	1.13655
XLOC_071466	LRRC7	chr3	75516108	75556834	1.34203	1.1245	2.52096	0.962876	0.84348	1.11817	0.836813	0.730231	0.634436
XLOC_071467	LRRC7	chr3	75641783	75642517	3.7638	2.62556	1.9619	1.79844	1.56458	1.55838	2.25915	1.24894	1.64158
XLOC_071468	LRRC7	chr3	75644512	75645970	0.0	0.0	0.0	1.79498	2.35384	2.31262	2.23176	1.73097	2.23718
XLOC_071469	LRRC7	chr3	75646280	75646501	0.0	0.0	0.0	6.76536	6.32871	7.48378	11.1048	6.87032	4.57773
XLOC_071470	LRRC7	chr3	75647726	75648380	0.0	0.435436	0.0	76.2049	75.2711	74.8498	83.3704	79.8536	87.1088
XLOC_071471	LRRC7	chr3	75649676	75650245	1.75773	0.0	0.0	9.28636	6.2767	8.52476	8.31074	7.13031	7.34621
XLOC_072564	LRRC72	chr4	24966503	25008601	0.50382	0.904409	0.78848	0.180695	0.158027	0.209581	0.0459963	0.0506191	0.132206
XLOC_073868	LRRC72	chr4	24966503	25008601	0.0	0.0	3.61676	0.0	0.180392	0.239528	0.104297	0.0	0.0
XLOC_073869	LRRC72	chr4	25008695	25009373	0.0	0.415383	0.0	0.124479	0.324572	0.287436	0.0	0.138104	0.0
XLOC_073870	LRRC72	chr4	25010385	25012501	0.369276	0.110496	0.0	0.165574	0.173928	0.0384354	0.0337978	0.074326	0.0646324
XLOC_069905	LRRC8D	chr3	53463662	53482009	3.66332	6.49232	5.31401	5.64851	5.98875	6.16867	5.13911	6.31381	5.16742
XLOC_070288	LRRC8D	chr3	53514010	53657581	1.93741	2.48769	2.19893	2.01211	1.22868	1.62934	0.775636	0.658719	1.02265
XLOC_071283	LRRC8D	chr3	53463662	53482009	1.52183	0.455191	4.76203	1.63697	0.238172	0.711046	0.760499	1.21932	0.864424
XLOC_070518	LRRFIP1	chr3	117782122	117869014	42.3989	39.718	40.1746	1.63723	1.41313	1.41416	1.36337	1.1897	2.23645
XLOC_077311	LRRIQ1;ALX1	chr5	14901018	15032847	0.0	0.0	0.0	0.0	0.0	0.0	0.0	0.157095	0.0
XLOC_044422	LRSAM1	chr11	98202375	98264549	51.7051	64.8955	62.509	54.653	45.5468	53.7113	45.8862	57.0447	37.4578
XLOC_047208	LRSAM1	chr11	98201810	98202179	6.83674	0.0	2.66706	0.917091	2.88189	0.69862	1.18462	1.32673	0.887839
XLOC_047209	LRSAM1	chr11	98202375	98264549	16.7395	8.33968	10.9053	9.62182	9.12269	11.3972	10.7733	7.07003	9.72958
XLOC_047210	LRSAM1	chr11	98202375	98264549	24.1563	8.23712	21.5389	11.1097	11.8989	17.276	10.1596	8.70264	13.1442
XLOC_047211	LRSAM1	chr11	98202375	98264549	6.81645	8.12958	15.9403	9.78613	12.296	20.6268	7.89095	8.3364	12.8965
XLOC_047212	LRSAM1	chr11	98202375	98264549	41.2717	4.10198	10.724	12.9632	8.78682	13.1809	10.7997	14.2302	11.8304
XLOC_047213	LRSAM1	chr11	98202375	98264549	8.25836	12.9223	10.9345	17.1992	11.3454	17.3606	13.6536	21.4741	9.66377

XLOC_047214	LRSAM1	chr11	98202375	98264549	5.9188	5.04023	5.34427	9.55297	7.46453	9.33047	6.32373	10.8019	5.69585
XLOC_047215	LRSAM1	chr11	98202375	98264549	3.49281	3.00455	3.41665	2.97536	3.76799	3.95187	2.1152	1.09755	3.43787
XLOC_047216	LRSAM1	chr11	98202375	98264549	4.9582	3.70585	1.93839	4.77538	7.05359	6.03099	3.23737	2.34494	3.89274
XLOC_047217	LRSAM1	chr11	98202375	98264549	10.8523	14.5578	12.6875	6.79966	11.0857	11.5404	3.19304	1.54824	11.687
XLOC_047218	LRSAM1	chr11	98202375	98264549	3.09711	1.84867	9.66823	3.32408	4.28271	3.17038	1.88849	1.50799	3.75768
XLOC_047219	LRSAM1	chr11	98202375	98264549	1.19649	1.4309	2.80669	2.35844	2.42579	2.23009	1.0784	0.595964	1.1483
XLOC_047220	LRSAM1	chr11	98202375	98264549	2.59576	1.35887	6.09261	1.80348	2.59216	1.95535	1.00445	1.10641	3.177
XLOC_037874	LSAMP;LOC538060	chr1	61638967	62216459	2.97395	4.89429	4.65488	0.100008	0.0291812	0.0	0.0680441	0.0	0.0
XLOC_039466	LSAMP;LOC538060	chr1	61638967	62216459	0.0	1.12412	0.0	0.0	0.145889	0.0	0.0	0.185845	0.163721
XLOC_039467	LSAMP;LOC538060	chr1	61638967	62216459	3.09431	5.0865	0.0	0.0	0.0	0.159857	0.0	0.0	0.0
XLOC_039468	LSAMP;LOC538060	chr1	61638967	62216459	0.0	5.06793	1.32538	0.0	0.0	0.0	0.0	0.0	0.0
XLOC_039469	LSAMP;LOC538060	chr1	61638967	62216459	0.0	1.9808	1.29506	0.0	0.0	0.171095	0.0	0.0	0.0
XLOC_039470	LSAMP;LOC538060	chr1	61638967	62216459	3.24713	1.45563	1.26894	0.0	0.0	0.0	0.0	0.0	0.0
XLOC_039471	LSAMP;LOC538060	chr1	61638967	62216459	0.0	0.0	1.21191	0.0	0.0	0.160196	0.0	0.0	0.0
XLOC_039472	LSAMP;LOC538060	chr1	61638967	62216459	1.4252	0.42601	2.22827	0.0	0.0	0.0	0.0	0.0	0.0
XLOC_039473	LSAMP;LOC538060	chr1	61638967	62216459	2.64212	1.18477	1.03284	0.0	0.0	0.0	0.0	0.0	0.0
XLOC_039474	LSAMP;LOC538060	chr1	61638967	62216459	1.68689	0.252261	1.31952	0.0755984	0.0659574	0.0	0.0	0.0	0.0
XLOC_039475	LSAMP;LOC538060	chr1	61638967	62216459	0.0	0.300184	3.14035	0.0	0.0	0.0	0.0	0.0	0.0
XLOC_064284	LSM12	chr19	44518557	44525580	0.0	0.180677	0.0	0.920494	0.851774	0.878778	0.385249	0.0606051	0.528085
XLOC_065212	LSM12	chr19	44516756	44517465	0.0	0.0	0.0	0.822438	0.613036	0.271409	0.589777	0.130465	0.915012
XLOC_062223	LSM14A	chr18	44828792	44888115	5.45733	8.5037	6.80602	9.49102	7.80407	7.81596	10.6099	10.9571	10.1789
XLOC_063081	LSM14A	chr18	44821041	44821441	2.98233	9.7918	4.65548	4.80169	3.89826	5.80429	6.50754	6.97932	7.23962
XLOC_083374	LSM4	chr7	4825633	4836151	0.731624	0.132753	0.463018	4.99325	4.65273	6.7165	3.57974	4.08967	5.48924
XLOC_084073	LSM4	chr7	4821487	4822152	0.0	0.0	0.0	0.638317	1.33117	0.736837	1.02333	0.707905	1.24242
XLOC_060528	LSM6	chr17	12324707	12325206	2.11809	0.0	0.0	0.379185	0.163952	0.218076	0.187721	0.20865	0.184181
XLOC_076750	LUM;KERA	chr5	20986524	21055742	45.1093	52.2791	46.7305	12.9121	12.4274	12.8084	17.5877	15.4893	11.3219
XLOC_065882	LUZP1	chr2	130273741	130284878	59.8265	70.7426	67.1049	190.979	160.735	180.152	240.453	230.858	216.146

XLOC_066550	LUZP1	chr2	130273741	130284878	24.4931	31.8309	26.364	65.4494	58.4687	61.5741	62.6358	68.12	70.5398
XLOC_068605	LUZP1	chr2	130273741	130284878	0.0	6.28525	12.3256	3.7737	2.39509	4.27485	2.66664	5.02242	1.81753
XLOC_068606	LUZP1	chr2	130286176	130287071	0.980694	1.17308	0.767007	0.527325	0.306422	0.711799	0.0887434	0.587727	0.342525
XLOC_068607	LUZP1	chr2	130296685	130297986	0.632481	0.378408	0.989699	0.170106	0.148632	0.197168	0.115208	0.0	0.16589
XLOC_068608	LUZP1	chr2	130302317	130302743	0.0	0.804248	0.0	0.241003	0.415163	0.0	0.0	0.0	0.0
XLOC_070226	LY9;CD244;PIFO;SLAMF7	chr3	8802197	9004485	40.5108	33.9553	22.677	172.66	159.024	160.331	155.66	175.498	177.987
XLOC_070634	LY9;CD244;PIFO;SLAMF7	chr3	8802197	9004485	21.9026	0.0	0.0	6.95496	8.93238	12.0768	12.2618	7.04952	16.9273
XLOC_070635	LY9;CD244;PIFO;SLAMF7	chr3	8802197	9004485	2.46984	1.47747	1.932	3.98481	3.02645	2.73435	2.01734	1.97752	2.73326
XLOC_070636	LY9;CD244;PIFO;SLAMF7	chr3	8802197	9004485	0.0	3.42196	3.72896	6.15287	5.28964	5.93323	5.17978	5.04969	5.49573
XLOC_066319	LYPD1	chr2	65133639	65239773	14.975	20.8394	19.439	0.148724	0.258047	0.10943	0.445266	0.329029	0.288403
XLOC_067669	LYPD1	chr2	65133639	65239773	6.47972	0.484126	3.79831	0.0	0.0	0.0	0.0	0.0	0.0
XLOC_067670	LYPD1	chr2	65133639	65239773	2.75534	1.64514	2.15104	0.0	0.0	0.0	0.0	0.0	0.0
XLOC_067671	LYPD1	chr2	65133639	65239773	1.39496	0.416984	2.18106	0.0	0.0	0.144268	0.125272	0.0	0.0
XLOC_067672	LYPD1	chr2	65133639	65239773	0.0	1.12412	2.93977	0.0	0.0	0.0	0.0	0.0	0.0
XLOC_067673	LYPD1	chr2	65133639	65239773	2.09326	0.984337	0.936191	0.0	0.0	0.0	0.0	0.0	0.0
XLOC_067674	LYPD1	chr2	65133639	65239773	1.58444	0.947071	0.0	0.0	0.0	0.0	0.0	0.0	0.0
XLOC_067675	LYPD1	chr2	65133639	65239773	0.0	0.42854	0.0	0.0	0.0	0.0	0.0	0.0	0.0
XLOC_067676	LYPD1	chr2	65133639	65239773	3.17557	1.26674	4.14136	0.0	0.0414936	0.0	0.0	0.0	0.0
XLOC_067677	LYPD1	chr2	65133639	65239773	5.33296	0.398559	5.21174	0.0	0.0	0.0	0.0	0.0	0.0
XLOC_067678	LYPD1	chr2	65133639	65239773	3.99439	1.34444	3.90703	0.0447687	0.039154	0.0519264	0.0	0.0	0.0
XLOC_067679	LYPD1	chr2	65133639	65239773	0.0	1.37279	3.5904	0.0	0.0	0.0	0.0	0.0	0.0
XLOC_067680	LYPD1	chr2	65133639	65239773	2.43413	2.00249	3.80899	0.0	0.0	0.0	0.0	0.061061	0.0
XLOC_067681	LYPD1	chr2	65133639	65239773	3.04865	0.911194	1.1915	0.0	0.0	0.0	0.0	0.0	0.0
XLOC_067682	LYPD1	chr2	65133639	65239773	4.00213	3.58098	21.8484	0.0	0.0	0.0	0.0	0.0	0.0
XLOC_067683	LYPD1	chr2	65133639	65239773	3.08112	2.76265	4.81669	0.0	0.0	0.0	0.0	0.0	0.0
XLOC_067684	LYPD1	chr2	65133639	65239773	2.08752	0.623582	1.63076	0.0	0.0	0.0	0.0	0.0	0.0
XLOC_067685	LYPD1	chr2	65133639	65239773	7.34576	2.19068	17.1814	0.0	0.0	0.0	0.0	0.0	0.0
XLOC_067686	LYPD1	chr2	65133639	65239773	5.93696	1.18245	10.8231	0.177168	0.0	0.0	0.0	0.0	0.0

XLOC_067687	LYPD1	chr2	65133639	65239773	0.0	0.947071	2.47682	0.0	0.0	0.0	0.0	0.0	0.0
XLOC_067688	LYPD1	chr2	65133639	65239773	1.28583	0.384404	0.0	0.0	0.0	0.0	0.0	0.0	0.0
XLOC_067689	LYPD1	chr2	65133639	65239773	0.545633	1.3059	2.1347	0.0489209	0.0	0.0	0.0497751	0.0	0.0
XLOC_067690	LYPD1	chr2	65133639	65239773	3.8415	0.946751	2.12249	0.0	0.0	0.0	0.0	0.0	0.0
XLOC_067691	LYPD1	chr2	65133639	65239773	4.49971	2.68788	0.0	0.0	0.0	0.0	0.0	0.0	0.0
XLOC_067692	LYPD1	chr2	65133639	65239773	2.84187	0.848342	2.2184	0.0	0.0	0.0	0.0	0.0	0.0
XLOC_067693	LYPD1	chr2	65133639	65239773	0.0	7.45107	0.0	0.0	0.0	0.631204	0.0	0.0	0.0
XLOC_067694	LYPD1	chr2	65133639	65239773	2.7268	0.407563	1.06589	0.122136	0.0	0.0	0.0	0.0	0.0
XLOC_067695	LYPD1	chr2	65133639	65239773	0.0	2.91216	0.0	0.0	0.0	0.0	0.0	0.0	0.0
XLOC_067696	LYPD1	chr2	65133639	65239773	1.11187	0.997361	1.73896	0.0	0.0	0.0	0.0	0.0	0.0
XLOC_067697	LYPD1	chr2	65133639	65239773	3.27926	1.95711	2.55882	0.0	0.0	0.0	0.0	0.0	0.0
XLOC_067698	LYPD1	chr2	65133639	65239773	0.798927	1.19525	3.12615	0.0	0.0	0.0	0.0	0.0	0.0
XLOC_067699	LYPD1	chr2	65133639	65239773	2.71656	2.03017	5.30948	0.121678	0.0	0.0	0.0	0.0	0.0
XLOC_067700	LYPD1	chr2	65133639	65239773	0.0	1.78482	7.00168	0.0	0.0	0.0	0.0	0.0	0.0
XLOC_067701	LYPD1	chr2	65133639	65239773	7.21033	2.15109	0.0	0.0	0.0	0.0	0.0	0.0	0.0
XLOC_067702	LYPD1	chr2	65133639	65239773	0.0	2.51121	0.0	0.376505	0.0	0.0	0.0	0.0	0.0
XLOC_067703	LYPD1	chr2	65133639	65239773	0.836578	0.834485	1.74607	0.0500183	0.0	0.0	0.0	0.0280953	0.0244118
XLOC_067704	LYPD1	chr2	65133639	65239773	2.54817	1.01615	3.98643	0.0	0.0	0.0	0.0	0.0	0.0
XLOC_067705	LYPD1	chr2	65133639	65239773	3.57512	1.92491	5.03444	0.0640969	0.0	0.0742616	0.0	0.0	0.0
XLOC_067707	LYPD1	chr2	65133639	65239773	2.39115	0.71408	1.86739	0.0	0.0	0.0	0.0	0.0	0.0
XLOC_067708	LYPD1	chr2	65133639	65239773	4.16302	3.10895	6.5043	0.0	0.0	0.0	0.0	0.0	0.0
XLOC_067709	LYPD1	chr2	65133639	65239773	2.66157	3.18261	5.20216	0.0	0.103662	0.0	0.0	0.0	0.0
XLOC_067710	LYPD1	chr2	65133639	65239773	2.40357	2.01355	4.13784	0.0431032	0.0	0.0	0.0	0.0	0.0
XLOC_067711	LYPD1	chr2	65133639	65239773	4.1313	2.3175	4.44496	0.0463024	0.0	0.0537004	0.0942698	0.0518764	0.0451683
XLOC_067712	LYPD1	chr2	65133639	65239773	5.14332	3.84404	4.02133	0.115197	0.0	0.0	0.115704	0.255908	0.0
XLOC_067713	LYPD1	chr2	65133639	65239773	3.43455	0.0	0.0	0.0	0.0	0.0	0.0	0.0	0.0
XLOC_067714	LYPD1	chr2	65133639	65239773	21.9026	3.27406	0.0	0.0	0.0	0.0	0.0	0.0	0.0
XLOC_067715	LYPD1	chr2	65133639	65239773	0.0	2.4826	9.73722	0.0	0.0	0.0	0.356304	0.0	0.359578
XLOC_067716	LYPD1	chr2	65133639	65239773	5.44418	2.44361	4.26081	0.0610279	0.0534336	0.177109	0.0311593	0.0685108	0.0297803
XLOC_067717	LYPD1	chr2	65133639	65239773	6.89499	1.64888	4.31228	0.123532	0.0	0.0	0.123875	0.0	0.0
XLOC_067718	LYPD1	chr2	65133639	65239773	4.62169	0.920884	3.61252	0.0	0.0	0.0	0.0	0.0	0.0
XLOC_067719	LYPD1	chr2	65133639	65239773	8.80765	3.94609	3.43985	0.19708	0.170329	0.226591	0.194857	0.0	0.0
XLOC_067720	LYPD1	chr2	65133639	65239773	4.37033	2.17718	5.69391	0.130488	0.0	0.0	0.130675	0.0	0.0

XLOC_067721	LYPD1	chr2	65133639	65239773	14.0987	4.20875	8.80468	0.0	0.217078	0.0	0.0	0.0	0.0
XLOC_067722	LYPD1	chr2	65133639	65239773	5.60411	2.98038	6.82059	0.0	0.0487807	0.0	0.0567221	0.0	0.0
XLOC_067723	LYPD1	chr2	65133639	65239773	3.73054	1.39456	4.37675	0.16717	0.0728805	0.0967314	0.337879	0.186419	0.0814497
XLOC_067724	LYPD1	chr2	65133639	65239773	4.20639	4.5619	5.34856	0.0471435	0.0412233	0.0546733	0.0479834	0.105626	0.0459877
XLOC_057844	LYPLAL1	chr16	23224738	23360344	17.126	21.3604	26.8233	5.61195	5.27255	5.24854	4.74028	2.50455	3.71344
XLOC_082826	LYRM7	chr7	24482099	24504536	20.3037	16.4258	15.8898	1.88816	3.1875	1.99579	1.65161	2.04315	3.25744
XLOC_084361	LYRM7	chr7	24482099	24504536	2.79921	3.55859	1.09496	0.0627331	0.0	0.145375	0.0	0.0	0.0
XLOC_082827	LYRM7;HINT1	chr7	24506934	24515655	6.68852	12.5318	10.6121	46.7948	25.7577	40.9458	23.8743	43.5534	25.0459
XLOC_076843	LYSB;LYZ1;LYZ2;LYZ3;LYZ;CPSF6	chr5	44472143	44799584	9.28695	3.89572	2.32905	0.0596998	0.0784316	0.0686519	0.304267	0.0	0.0
XLOC_077822	LYSB;LYZ1;LYZ2;LYZ3;LYZ;CPSF6	chr5	44472143	44799584	0.0	0.0	0.0	0.0	0.0	0.0	0.135731	0.0	0.0
XLOC_077825	LYSB;LYZ1;LYZ2;LYZ3;LYZ;CPSF6	chr5	44472143	44799584	0.0	0.316429	0.0	0.0	0.0	0.0	0.0	0.0	0.0
XLOC_050505	LYZL1	chr13	35738421	35875070	39.9657	32.8801	38.9956	31.4249	35.8421	30.9911	28.7205	31.5383	34.7426
XLOC_051572	LYZL1	chr13	35738421	35875070	0.0	0.0	0.0	5.66798	2.35819	7.92721	3.867	5.88625	4.06093
XLOC_051573	LYZL1	chr13	35738421	35875070	0.0	0.0	2.44836	5.61198	5.54184	2.2477	4.64271	3.66451	1.08743
XLOC_051574	LYZL1	chr13	35738421	35875070	8.48366	2.82029	4.79462	2.15529	2.36594	2.7454	1.63608	2.17912	1.44307
XLOC_051575	LYZL1	chr13	35738421	35875070	0.0	3.2742	0.0	0.0	0.0	1.09824	2.62752	0.0	1.88081
XLOC_051576	LYZL1	chr13	35738421	35875070	5.64614	6.07752	4.41519	1.8213	1.49777	3.15846	2.14	2.81464	1.77385
XLOC_051577	LYZL1	chr13	35738421	35875070	3.09709	5.54601	9.66823	1.38504	1.9035	2.85345	2.69784	1.50799	1.61044
XLOC_051578	LYZL1	chr13	35738421	35875070	9.0762	8.12851	7.08288	4.91412	3.38282	2.73543	2.94096	2.52607	2.33862
XLOC_051579	LYZL1	chr13	35738421	35875070	4.91765	1.26086	7.69451	1.63739	0.82501	1.4594	0.766799	1.05615	0.736854
XLOC_051580	LYZL1	chr13	35738421	35875070	0.0	3.25895	19.8835	5.86106	1.9505	4.08832	2.82878	1.40979	2.20524
XLOC_051581	LYZL1	chr13	35738421	35875070	0.0	0.526537	0.0	0.47334	0.957485	0.545508	0.628704	1.3946	0.767084
XLOC_051582	LYZL1	chr13	35738421	35875070	3.27518	1.6322	10.245	1.17392	1.44847	1.92308	0.985884	0.762275	0.381151
XLOC_051583	LYZL1	chr13	35738421	35875070	10.1775	1.5171	0.0	4.55313	1.54323	3.0962	0.859472	1.94178	0.877369
XLOC_051584	LYZL1	chr13	35738421	35875070	4.27372	3.82977	3.33844	0.765083	0.496189	0.880011	0.567963	0.420904	0.557404
XLOC_051585	LYZL1	chr13	35738421	35875070	3.72362	1.66886	1.45478	0.166702	1.29973	0.960035	0.663107	0.551924	0.0
XLOC_051586	LYZL1	chr13	35738421	35875070	5.46871	3.27216	3.85117	1.66707	0.60016	1.76259	1.29705	0.878643	1.14785
XLOC_051587	LYZL1	chr13	35738421	35875070	19.7434	14.7436	23.1237	1.78563	1.46833	0.0	0.795047	1.82421	0.847742

XLOC_051588	LYZL1	chr13	35738421	35875070	5.29865	2.21818	4.97252	1.04459	0.579112	1.20813	0.574534	0.317239	0.555036
XLOC_051589	LYZL1	chr13	35738421	35875070	1.74081	3.64135	6.80203	1.09119	1.21643	1.07796	1.24276	0.172271	0.909492
XLOC_051590	LYZL1	chr13	35738421	35875070	1.73755	1.29916	4.07733	1.09014	0.47549	0.721181	0.315115	0.608265	0.30356
XLOC_051591	LYZL1	chr13	35738421	35875070	2.69128	0.80454	3.15609	0.60274	0.209641	0.139258	0.483808	0.267615	0.469366
XLOC_051592	LYZL1	chr13	35738421	35875070	1.27052	2.09039	2.98213	0.626462	0.298578	0.594113	0.404979	0.254886	0.722006
XLOC_051593	LYZL1	chr13	35738421	35875070	8.92034	1.33314	0.0	0.532668	0.462684	0.614736	0.133288	0.147582	0.388732
XLOC_051594	LYZL1	chr13	35738421	35875070	0.0	2.84728	5.95687	0.682578	1.47787	1.17903	0.67829	1.31766	1.16105
XLOC_051595	LYZL1	chr13	35738421	35875070	2.56038	2.29354	5.99766	1.37455	1.38258	1.57757	2.25132	0.501786	1.55607
XLOC_051596	LYZL1	chr13	35738421	35875070	0.0	2.48265	3.24574	1.4888	1.26952	2.96623	0.356301	0.400651	0.0
XLOC_051597	LYZL1	chr13	35738421	35875070	0.671848	2.61265	4.20502	0.662519	0.683961	1.11676	0.428013	0.134722	0.70476
XLOC_051598	LYZL1	chr13	35738421	35875070	2.173	0.649994	2.26664	0.584376	0.453595	0.376146	0.526858	0.508037	0.253224
XLOC_051599	LYZL1	chr13	35738421	35875070	3.53657	1.52851	3.07521	0.916166	0.740186	1.26772	0.898721	0.553478	0.618933
XLOC_051600	LYZL1	chr13	35738421	35875070	0.0	1.90848	2.99473	1.60138	0.795987	0.792882	0.91924	0.508256	0.445448
XLOC_051601	LYZL1	chr13	35738421	35875070	3.55255	1.94842	2.31634	0.690086	0.649549	0.615432	0.593627	0.297115	0.776597
XLOC_051602	LYZL1	chr13	35738421	35875070	3.70594	3.69322	5.79533	0.664062	0.385219	0.895231	1.00135	0.246005	0.646584
XLOC_051603	LYZL1	chr13	35738421	35875070	0.0	0.779844	2.91379	0.93485	0.935243	1.2788	0.340742	0.749354	0.521312
XLOC_051604	LYZL1	chr13	35738421	35875070	0.0	2.07438	0.90419	0.621643	0.270615	0.359333	0.31289	0.691484	0.302704
XLOC_051605	LYZL1	chr13	35738421	35875070	2.12638	2.38561	2.07981	0.810268	0.541793	0.939679	0.485069	0.800871	1.02286
XLOC_051606	LYZL1	chr13	35738421	35875070	2.72832	2.04077	3.20253	1.14165	1.14147	1.08799	0.623275	0.63999	0.994529
XLOC_051607	LYZL1	chr13	35738421	35875070	1.54056	2.76267	2.40835	0.827888	0.838632	1.27351	0.551915	0.916959	0.80546
XLOC_051608	LYZL1	chr13	35738421	35875070	1.82002	1.36137	2.84848	0.693584	1.1065	0.899344	0.582086	0.823359	0.835929
XLOC_051609	LYZL1	chr13	35738421	35875070	6.89683	3.09144	5.38983	1.8528	2.14217	1.60166	0.923457	1.53596	1.35131
XLOC_051610	LYZL1	chr13	35738421	35875070	8.56912	3.83312	10.0226	1.5326	2.28503	3.48757	0.36608	1.64763	1.85013
XLOC_051611	LYZL1	chr13	35738421	35875070	1.1495	2.40767	4.19813	1.0308	0.721811	1.11659	0.420721	0.501206	0.871783
XLOC_051612	LYZL1	chr13	35738421	35875070	0.571053	2.05007	2.6809	0.921572	0.939857	0.415555	0.364483	0.745243	0.798997
XLOC_051613	LYZL1	chr13	35738421	35875070	6.46363	3.09172	0.0	0.463256	0.90657	1.47162	0.232617	0.771773	0.90199
XLOC_051614	LYZL1	chr13	35738421	35875070	1.26055	3.7709	1.47938	1.01708	0.444365	0.916943	0.459245	0.821931	0.881669
XLOC_051615	LYZL1	chr13	35738421	35875070	0.0	2.22234	1.93747	0.488411	0.621343	0.721032	0.58781	0.845884	0.563106
XLOC_064294	LYZL6;RDM1;GOSR2	chr19	45817104	45998676	8.84957	10.2558	7.48847	12.6767	13.8292	12.9305	16.1561	15.4755	12.3696
XLOC_061909	LZTR1;PRODH	chr17	74329382	74329829	9.99398	14.9213	7.80407	14.5317	15.0401	13.6026	13.8557	14.7005	13.0181
XLOC_070104	MACF1	chr3	107278817	107283886	15.7853	24.8698	14.349	28.0748	33.6767	31.2545	29.098	29.3014	36.7162
XLOC_070105	MACF1	chr3	107297777	107299677	4.47601	2.61647	5.20794	1.50055	1.75157	1.42094	2.3848	0.266988	1.68401

XLOC_070471	MACF1	chr3	107306329	107334907	25.8205	25.2279	32.437	29.7204	30.8793	31.3637	33.9981	30.2112	28.4469
XLOC_070472	MACF1	chr3	107445438	107448379	0.0	0.0	0.0	0.354838	0.185819	0.246569	0.143791	0.0792646	0.0
XLOC_070473	MACF1	chr3	107500370	107517814	0.0	0.0	2.68215	0.0	0.533027	0.53136	0.612808	0.0	0.0
XLOC_072038	MACF1	chr3	107285129	107285901	4.70804	2.11146	2.76107	0.316378	1.00975	0.487569	0.742863	0.234562	1.23239
XLOC_072039	MACF1	chr3	107290230	107290975	6.15554	4.04871	1.92521	0.2206	0.671812	0.382355	1.44158	0.24518	0.859189
XLOC_072040	MACF1	chr3	107293413	107294061	1.4746	1.76302	2.30539	0.132082	0.917844	0.457287	0.13223	0.0	0.514104
XLOC_072041	MACF1	chr3	107295149	107295741	4.99402	3.48233	5.20406	0.59631	1.29322	1.03125	0.446271	0.494668	1.44993
XLOC_072042	MACF1	chr3	107296320	107296744	0.0	1.62053	0.0	0.242807	1.46374	1.39218	0.713983	0.796403	0.471018
XLOC_072043	MACF1	chr3	107299750	107300385	4.54425	0.452738	1.18403	0.54269	1.29598	0.782702	1.7645	0.150313	1.32006
XLOC_072044	MACF1	chr3	107300558	107301548	0.868775	2.07864	2.03867	1.32373	1.69811	1.17187	0.551451	0.608265	1.29013
XLOC_072045	MACF1	chr3	107301744	107302340	3.29898	2.46472	6.44583	2.06808	2.05053	3.23613	1.91673	0.490246	2.5862
XLOC_072046	MACF1	chr3	107302483	107304054	2.55831	3.36776	3.20298	1.14691	1.60477	2.07512	2.52209	0.565422	1.96915
XLOC_072047	MACF1	chr3	107304246	107304892	7.40318	4.42557	5.78703	1.59146	2.87987	3.36704	2.38962	0.587956	3.74243
XLOC_072048	MACF1	chr3	107305445	107306029	3.39181	4.56114	13.2538	1.21495	3.16117	2.80104	2.27192	1.34335	3.98779
XLOC_072049	MACF1	chr3	107306329	107334907	5.45863	3.91725	5.97627	2.05436	3.3229	3.9592	3.35201	1.08896	3.14449
XLOC_072050	MACF1	chr3	107306329	107334907	49.3584	2.94869	30.832	3.57122	13.9471	10.8972	8.74552	4.56052	13.5638
XLOC_072051	MACF1	chr3	107306329	107334907	0.0	11.7112	0.0	0.0	2.8849	3.91454	4.12291	3.5654	6.7169
XLOC_072052	MACF1	chr3	107306329	107334907	8.29742	5.79001	7.9321	2.06567	2.80994	4.01637	3.84178	1.38228	2.97916
XLOC_072053	MACF1	chr3	107306329	107334907	2.47414	3.97946	5.80926	2.41298	2.52608	2.70494	2.49369	0.934411	2.30101
XLOC_072054	MACF1	chr3	107306329	107334907	0.0	15.3574	20.0737	5.41038	12.8071	12.9326	7.67327	4.78742	13.9987
XLOC_072055	MACF1	chr3	107306329	107334907	12.9659	3.87405	5.78945	2.48768	2.87361	4.01145	3.95895	1.83053	2.902
XLOC_072056	MACF1	chr3	107306329	107334907	9.83681	14.6631	19.1698	4.83995	10.8237	15.4708	9.57574	4.22937	13.1484
XLOC_072057	MACF1	chr3	107306329	107334907	4.70455	4.21497	3.6743	2.31559	2.9079	3.62765	3.73682	1.84856	2.04375
XLOC_072058	MACF1	chr3	107306329	107334907	0.0	13.2774	17.3535	5.0381	6.5821	8.90218	8.87044	3.0599	10.4863
XLOC_072059	MACF1	chr3	107306329	107334907	5.42612	4.8526	12.6878	4.8569	11.4963	7.14403	9.12298	6.19296	6.54473
XLOC_072060	MACF1	chr3	107306329	107334907	2.25236	2.29127	5.28772	2.42355	3.7459	4.21764	5.35167	1.76626	3.27104
XLOC_050434	MACROD2	chr13	8129201	8129790	0.0	1.40499	0.0	2.94711	3.08965	3.8695	4.15202	4.6214	3.06563
XLOC_050435	MACROD2	chr13	8885579	8886988	0.0	2.93017	3.99727	5.13159	7.18522	5.36054	6.22193	4.30048	5.65721
XLOC_050436	MACROD2	chr13	8887115	8891052	3.34054	2.8556	1.86716	3.40772	3.74972	3.57387	4.62694	2.87773	2.71472
XLOC_050437	MACROD2	chr13	9093727	9174587	25.9061	23.2199	19.0281	35.4856	36.1203	35.1934	38.5216	36.6441	39.2977
XLOC_050438	MACROD2	chr13	9276875	9668974	10.9955	8.98631	9.73868	5.45924	4.02275	4.82042	5.49718	4.66598	4.47944
XLOC_050730	MACROD2	chr13	8802878	8871105	10.1828	14.4758	13.0773	8.40091	8.59944	8.27306	10.8413	10.649	10.0134
XLOC_050731	MACROD2	chr13	8929513	8997057	75.3028	91.2661	76.1853	108.642	95.011	106.715	129.407	141.011	125.732

XLOC_050732	MACROD2	chr13	9684691	9765366	25.5886	21.4942	17.8237	7.95542	10.9632	9.14471	10.5022	7.5255	10.3411
XLOC_051100	MACROD2	chr13	8029513	8029923	0.0	6.84031	4.47182	10.2492	13.2262	14.9738	13.528	13.9841	11.9246
XLOC_051101	MACROD2	chr13	8030811	8030986	0.0	22.0384	0.0	11.421	19.3963	4.86083	16.7173	19.4036	12.5742
XLOC_051102	MACROD2	chr13	8128433	8128542	0.0	0.0	0.0	1176.35	824.343	1406.71	1979.98	984.169	1406.22
XLOC_051103	MACROD2	chr13	8892390	8892957	0.0	1.0556	0.0	1.7398	1.09686	0.546763	1.26038	0.349482	1.99925
XLOC_051104	MACROD2	chr13	8929513	8997057	9.83681	1.46631	3.83391	4.39996	3.73232	3.99247	2.08168	1.87972	2.96899
XLOC_051105	MACROD2	chr13	8929513	8997057	1.29042	0.771548	4.03566	1.84971	2.31262	2.93794	1.9738	1.66924	3.15135
XLOC_051106	MACROD2	chr13	8929513	8997057	7.27542	4.33926	5.67212	7.84193	5.45792	4.39857	4.19128	4.77596	8.75608
XLOC_051107	MACROD2	chr13	8929513	8997057	3.90455	4.66607	3.05065	4.36952	4.84214	4.02421	2.77674	2.31243	3.05756
XLOC_051108	MACROD2	chr13	8929513	8997057	6.31214	4.08952	0.822733	4.33655	4.51716	4.36132	6.84524	7.87404	6.24483
XLOC_051109	MACROD2	chr13	9093727	9174587	10.418	3.83507	9.40349	5.81839	7.33469	6.90454	7.85796	6.09681	8.1925
XLOC_051110	MACROD2	chr13	9093727	9174587	7.51746	6.37168	9.80287	3.98752	5.44727	4.23147	6.04853	3.45648	5.25808
XLOC_051111	MACROD2	chr13	9093727	9174587	8.3335	6.05566	6.83233	4.66164	5.23225	5.20339	6.46162	4.71088	6.70183
XLOC_051112	MACROD2	chr13	9093727	9174587	6.63285	4.57944	5.19027	3.01931	4.00064	3.34272	4.37803	2.97295	3.61488
XLOC_051113	MACROD2	chr13	9093727	9174587	8.48339	7.59427	13.2368	1.52856	8.2362	14.4995	8.97622	9.4752	5.10218
XLOC_051114	MACROD2	chr13	9093727	9174587	4.49509	3.76412	3.51611	3.38413	3.51358	4.75653	3.91081	3.86511	3.7692
XLOC_051115	MACROD2	chr13	9093727	9174587	7.29699	2.1803	1.42584	1.79674	1.98153	3.19893	2.76322	0.541033	2.85858
XLOC_051116	MACROD2	chr13	9093727	9174587	3.25067	2.26797	9.32115	2.23318	2.28339	3.48072	5.28469	2.05374	2.26997
XLOC_051117	MACROD2	chr13	9093727	9174587	8.32457	4.9652	6.4923	1.861	5.39508	2.11898	6.76972	3.60586	3.59625
XLOC_051118	MACROD2	chr13	9093727	9174587	5.30906	9.49575	12.4148	2.37567	6.4316	4.30362	6.2579	7.5818	7.32028
XLOC_051119	MACROD2	chr13	9174812	9175937	2.24263	1.78879	0.0	0.871123	0.643534	0.620987	0.203815	0.449285	0.58796
XLOC_051120	MACROD2	chr13	9223785	9224722	17.6291	17.2039	10.1601	31.932	23.0577	26.8504	31.0121	40.0621	25.1201
XLOC_051121	MACROD2	chr13	9276875	9668974	6.71401	4.23963	5.83597	1.8724	1.10928	1.54928	1.695	0.747279	1.62986
XLOC_051122	MACROD2	chr13	9276875	9668974	7.24215	5.40989	8.48877	0.972692	1.12392	1.12061	1.12953	1.7902	2.04898
XLOC_051123	MACROD2	chr13	9276875	9668974	2.64696	5.53903	8.27783	0.474259	0.515489	1.0955	0.595042	0.526559	1.03876
XLOC_051124	MACROD2	chr13	9276875	9668974	3.60502	2.96489	3.52468	0.565426	0.91579	1.02845	0.898423	0.540647	1.41692
XLOC_051125	MACROD2	chr13	9276875	9668974	0.0	5.15507	4.4927	1.03244	0.435406	0.0	0.0	0.546686	2.48179
XLOC_051126	MACROD2	chr13	9276875	9668974	4.66794	3.14204	1.82618	0.837008	1.0974	1.15237	0.797864	1.05419	0.969434
XLOC_051127	MACROD2	chr13	9276875	9668974	1.89598	6.23117	0.0	0.679001	0.588078	0.977393	0.674856	0.561821	0.495
XLOC_051128	MACROD2	chr13	9276875	9668974	1.26116	0.754079	3.9443	0.225979	0.294878	0.391626	0.45414	0.251074	0.330026
XLOC_051129	MACROD2	chr13	9276875	9668974	0.0	1.04793	4.11085	0.471045	0.272249	0.180955	0.156438	0.0	0.305355
XLOC_051130	MACROD2	chr13	9276875	9668974	2.38327	2.84693	1.86125	0.639828	0.552284	0.489986	0.0	0.468058	0.414093
XLOC_051131	MACROD2	chr13	9276875	9668974	4.34503	2.73011	4.76033	0.506498	0.647718	0.678093	0.834104	0.349537	1.02641

XLOC_051132	MACROD2	chr13	9276875	9668974	2.58545	2.31878	2.02143	0.579066	0.402908	0.936466	0.697852	0.643145	0.901989
XLOC_051133	MACROD2	chr13	9276875	9668974	1.93659	4.05008	3.02619	0.693518	1.05084	1.19769	0.688805	0.382383	0.842549
XLOC_051134	MACROD2	chr13	9276875	9668974	1.40504	1.47099	3.84726	0.18893	0.439997	0.656724	0.3195	0.563281	0.429831
XLOC_051135	MACROD2	chr13	9276875	9668974	6.62819	2.9712	10.3605	0.296791	0.514952	0.171107	0.296192	0.0	0.721662
XLOC_051136	MACROD2	chr13	9682883	9684542	0.481674	1.44112	1.13076	0.259134	0.4911	0.601187	0.263955	0.242036	0.210675
XLOC_079593	MAD2L1;LOC780876	chr6	5766409	6028469	27.9315	23.4507	29.667	0.383118	0.195462	0.352178	0.381671	0.492098	0.581734
XLOC_080425	MAD2L1;LOC780876	chr6	5766409	6028469	11.7837	7.03463	27.5929	0.263513	0.0	0.0	0.0	0.0	0.0
XLOC_080426	MAD2L1;LOC780876	chr6	5766409	6028469	5.19428	9.30542	22.3063	0.0	0.0	0.0	0.0	0.0	0.225458
XLOC_080427	MAD2L1;LOC780876	chr6	5766409	6028469	6.16857	3.38174	10.4525	0.0	0.0802776	0.106572	0.0	0.0	0.0
XLOC_080428	MAD2L1;LOC780876	chr6	5766409	6028469	10.787	3.87056	12.6536	0.0	0.0	0.0	0.0	0.0	0.0941551
XLOC_080429	MAD2L1;LOC780876	chr6	5766409	6028469	6.79181	2.70467	17.6826	0.0	0.0	0.0	0.0	0.0	0.0
XLOC_080430	MAD2L1;LOC780876	chr6	5766409	6028469	1.22072	2.55473	9.54491	0.109371	0.0	0.0	0.0	0.0	0.0
XLOC_080431	MAD2L1;LOC780876	chr6	5766409	6028469	7.5839	9.62999	23.7028	0.0	0.0	0.0	0.0	0.187273	0.0
XLOC_080432	MAD2L1;LOC780876	chr6	5766409	6028469	10.696	7.15224	10.3376	0.0	0.0	0.0	0.114615	0.126233	0.0550086
XLOC_080433	MAD2L1;LOC780876	chr6	5766409	6028469	10.1672	6.63405	11.567	0.0	0.0722326	0.0	0.251182	0.0923848	0.0807231
XLOC_080434	MAD2L1;LOC780876	chr6	5766409	6028469	12.2977	7.73974	12.2786	0.0	0.0998209	0.0	0.0775447	0.0426472	0.0742056
XLOC_080435	MAD2L1;LOC780876	chr6	5766409	6028469	6.10342	5.47615	12.9582	0.156297	0.136337	0.180939	0.237179	0.0	0.304654
XLOC_080436	MAD2L1;LOC780876	chr6	5766409	6028469	18.0152	8.61277	15.4854	0.0	0.0	0.0	0.0	0.178147	0.0
XLOC_080437	MAD2L1;LOC780876	chr6	5766409	6028469	18.6485	23.0662	24.9596	0.0	0.0	0.0	0.0	0.0	0.0
XLOC_080438	MAD2L1;LOC780876	chr6	5766409	6028469	10.5126	8.27163	13.1024	0.0349149	0.0	0.0405214	0.0	0.0	0.0681418
XLOC_080439	MAD2L1;LOC780876	chr6	5766409	6028469	5.76954	5.91637	16.1184	0.0	0.0	0.0	0.0	0.0	0.0
XLOC_080440	MAD2L1;LOC780876	chr6	5766409	6028469	4.20741	3.02009	10.5316	0.0	0.0	0.0873292	0.152687	0.0841934	0.0
XLOC_080441	MAD2L1;LOC780876	chr6	5766409	6028469	8.07552	6.94273	12.6315	0.0	0.0	0.0	0.0	0.0	0.0
XLOC_080442	MAD2L1;LOC780876	chr6	5766409	6028469	17.6518	5.55676	7.26649	0.166526	0.072601	0.0963637	0.0	0.0928542	0.0

XLOC_080443	MAD2L1;LOC780876	chr6	5766409	6028469	13.8597	4.14161	13.539	0.0	0.0	0.0	0.0	0.0	0.0
XLOC_080444	MAD2L1;LOC780876	chr6	5766409	6028469	9.54848	4.75617	13.6824	0.0	0.0	0.0	0.0	0.0	0.0
XLOC_080445	MAD2L1;LOC780876	chr6	5766409	6028469	7.36827	3.66715	21.0978	0.0	0.0	0.0	0.0	0.0	0.213317
XLOC_080446	MAD2L1;LOC780876	chr6	5766409	6028469	9.10491	1.70146	9.78976	0.101979	0.0	0.0	0.0	0.0	0.0
XLOC_080447	MAD2L1;LOC780876	chr6	5766409	6028469	12.7602	4.50886	6.34961	0.0	0.0	0.0	0.0	0.115612	0.101223
XLOC_080448	MAD2L1;LOC780876	chr6	5766409	6028469	4.42128	1.7633	5.76467	0.0	0.0	0.0	0.0	0.0	0.0
XLOC_080449	MAD2L1;LOC780876	chr6	5766409	6028469	8.6272	6.00898	26.9372	0.0	0.0	0.0	0.0	0.0	0.0
XLOC_058963	MAD2L2	chr16	42876146	42881003	2.15448	1.70414	3.25255	1.02145	1.30696	1.34728	0.890933	0.66773	1.29389
XLOC_082977	MADCAM1	chr7	44733552	44777679	18.4456	23.4857	23.3167	24.216	20.4865	19.1845	15.8124	22.0441	21.8792
XLOC_083608	MADCAM1	chr7	44733552	44777679	0.539817	1.45368	0.0	18.3985	16.6484	17.07	12.0436	13.5574	13.8751
XLOC_057037	MADD	chr15	78349780	78350683	14.5525	2.32101	3.03514	0.0	0.0	0.201202	0.0	0.0	0.0
XLOC_055524	MADD;NR1H3	chr15	78318481	78349487	19.7717	8.85229	9.21841	0.213786	0.117162	0.285324	0.0	0.0418412	0.0471854
XLOC_078707	MAFF	chr5	110475317	110475930	0.0	0.474574	0.0	1.70658	1.48122	1.31222	1.13678	2.3615	0.691729
XLOC_078708	MAFF	chr5	110476447	110476979	0.0	0.0	1.50906	0.864587	1.64698	0.59726	2.06109	1.52554	1.51257
XLOC_078709	MAFF	chr5	110480538	110481893	0.0	0.54203	0.945095	0.866347	0.993736	0.627699	1.15575	0.606051	0.580893
XLOC_092200	MAGED1	chrX	95711715	95742168	0.0	1.60905	2.10406	0.0	0.104805	0.0	0.0	0.267615	0.0
XLOC_070244	MAGI3	chr3	29951313	30009886	8.48884	3.22991	4.83424	0.153036	0.258886	0.158387	0.250261	0.218887	0.234639
XLOC_070753	MAGI3	chr3	30032390	30033119	0.0	0.0	0.0	1.24723	0.493165	0.261985	0.569615	0.0	0.551959
XLOC_053575	MAL2	chr14	47153908	47189417	18.0285	11.8691	13.4594	0.671621	0.566514	0.60659	0.432295	0.251503	0.65558
XLOC_089328	MAN1A1	chr9	32094194	32265904	22.8801	23.4295	22.1079	13.0973	12.7816	12.8129	13.2634	13.7013	12.555
XLOC_090505	MAN1A1	chr9	32094194	32265904	0.858463	1.79724	5.37197	4.53965	5.2357	5.25568	5.37228	4.3796	3.97457
XLOC_090506	MAN1A1	chr9	32094194	32265904	0.0	2.45462	0.0	2.94392	4.39443	2.5142	2.11541	3.56686	4.26671
XLOC_090507	MAN1A1	chr9	32094194	32265904	0.0	1.86003	1.62142	2.04371	2.0894	1.92376	0.736472	2.86389	2.70763
XLOC_090508	MAN1A1	chr9	32094194	32265904	0.0	1.9785	5.17259	0.595223	4.49295	5.35731	3.84889	6.25106	3.99556
XLOC_083837	MAN2A1	chr7	111471679	111779973	9.60471	7.36962	5.09701	0.329904	0.0787758	0.104444	0.431303	0.101008	0.43975
XLOC_080026	MANBA;SLC39A8;NFKB1	chr6	23410757	23839001	2.75213	6.0047	7.5309	0.0863746	0.113328	0.100195	0.219959	0.0968113	0.252808
XLOC_080580	MANBA;SLC39A8;NFKB1	chr6	23410757	23839001	1.31148	0.784124	2.05072	0.117491	0.0	0.0	0.117955	0.130465	0.114376

XLOC_080581	MANBA;SLC39A8;NFKB1	chr6	23410757	23839001	1.30911	1.56541	0.0	0.0	0.203979	0.0	0.117747	0.520931	0.11417
XLOC_050609	MANBAL;SRC	chr13	66880248	66998366	22.8005	16.8206	17.0297	11.8498	11.4138	12.6705	14.2353	15.0002	13.3924
XLOC_050610	MANBAL;SRC	chr13	66880248	66998366	1.04401	1.87407	2.45077	0.421276	0.695752	0.651372	0.666964	0.524341	0.684845
XLOC_050923	MANBAL;SRC	chr13	66880248	66998366	107.847	106.272	51.6293	140.309	141.178	143.795	157.354	148.824	190.038
XLOC_050924	MANBAL;SRC	chr13	66880248	66998366	26.7448	23.6099	25.2613	19.215	21.4419	18.5355	24.9844	21.9368	23.0304
XLOC_052359	MANBAL;SRC	chr13	66880248	66998366	1.40581	0.0	1.09902	0.0	0.437754	0.290792	0.631108	0.55875	0.612808
XLOC_052360	MANBAL;SRC	chr13	66880248	66998366	0.0	0.721248	1.88609	0.432456	1.30554	0.248265	0.212917	0.474128	1.88809
XLOC_052361	MANBAL;SRC	chr13	66880248	66998366	0.0	0.95701	2.50244	4.01544	1.72306	2.2965	5.01864	1.55943	1.66687
XLOC_052362	MANBAL;SRC	chr13	66880248	66998366	6.19418	7.39467	9.66828	2.77035	5.71028	4.12157	8.36332	5.12716	2.95247
XLOC_052363	MANBAL;SRC	chr13	66880248	66998366	4.54209	2.03489	1.77384	2.236	2.63369	0.467266	2.60942	1.56315	1.38163
XLOC_089410	MANEA	chr9	55149594	55224033	40.3422	49.9386	50.0321	115.141	101.486	107.174	137.306	155.469	124.854
XLOC_090847	MANEA	chr9	55148421	55148862	0.0	0.0	1.99219	0.0	0.19682	0.0	0.448748	0.0	0.0
XLOC_090848	MANEA	chr9	55149594	55224033	11.277	3.98529	6.4141	3.21547	2.08139	3.29434	2.13193	2.55855	2.23753
XLOC_090849	MANEA	chr9	55149594	55224033	1.53475	1.83622	3.60182	1.65087	1.74135	1.19509	1.32458	1.22958	1.47526
XLOC_090850	MANEA	chr9	55149594	55224033	0.0	0.0	0.0	0.434262	1.47394	0.985187	1.64483	0.0	1.25578
XLOC_090851	MANEA	chr9	55149594	55224033	1.55034	1.3915	5.25689	0.973051	1.01299	1.50469	1.36787	0.830607	1.62721
XLOC_090852	MANEA	chr9	55149594	55224033	5.29895	5.27801	8.28183	1.10718	1.50825	1.82255	1.57548	2.27164	1.99926
XLOC_090853	MANEA	chr9	55149594	55224033	6.29485	1.88194	4.9218	1.80472	1.86439	1.43346	1.13342	1.37854	0.87855
XLOC_090854	MANEA	chr9	55149594	55224033	4.82142	0.0	3.75845	3.0191	0.0	3.91528	3.26951	2.7665	0.415887
XLOC_090855	MANEA	chr9	55149594	55224033	12.2911	1.83213	4.78988	1.10156	0.463566	2.48398	0.512013	0.0	2.11564
XLOC_090856	MANEA	chr9	55149594	55224033	4.9852	2.97246	7.77179	2.23011	1.51281	3.03424	0.421595	0.0	0.859711
XLOC_090857	MANEA	chr9	55149594	55224033	2.15573	1.07471	2.24863	0.45092	0.337525	0.671663	0.457385	0.792054	1.00487
XLOC_090858	MANEA	chr9	55149594	55224033	3.25444	1.45892	2.54358	0.582954	1.13812	0.840239	0.582037	0.967517	1.4175
XLOC_090859	MANEA	chr9	55149594	55224033	3.29147	0.491846	3.85871	0.294807	0.639419	0.339885	0.735576	0.489152	1.43354
XLOC_090860	MANEA	chr9	55149594	55224033	2.36171	2.11836	2.77008	0.529044	0.73679	0.489155	0.95816	1.05892	1.44247
XLOC_090861	MANEA	chr9	55149594	55224033	1.28291	0.767558	4.01494	0.402559	0.753681	0.666518	1.10983	1.41544	1.28983
XLOC_090862	MANEA	chr9	55149594	55224033	0.0	16.1208	0.0	8.60502	10.9074	8.07806	19.1093	8.57285	9.24328
XLOC_090863	MANEA	chr9	55149594	55224033	0.0	2.19692	4.30899	0.98754	1.14088	0.568753	1.30992	0.726755	0.800015

XLOC_090864	MANEA	chr9	55149594	55224033	0.999672	1.19577	2.3455	0.716715	1.01503	0.51821	1.80858	0.598961	1.30924
XLOC_090865	MANEA	chr9	55149594	55224033	0.754563	2.93408	1.18058	1.96153	1.06291	1.48863	3.3598	1.73828	1.71446
XLOC_090866	MANEA	chr9	55149594	55224033	0.0	1.20456	6.30048	0.962621	0.836958	0.972682	2.29393	0.934943	1.4055
XLOC_090867	MANEA	chr9	55149594	55224033	6.52073	5.83225	15.2478	1.16964	2.45401	4.60758	3.78654	1.8443	6.17044
XLOC_090868	MANEA	chr9	55149594	55224033	3.35513	2.00232	2.61785	0.900222	1.28644	3.77262	1.45492	3.58352	2.9053
XLOC_090869	MANEA	chr9	55149594	55224033	4.11129	2.86957	6.96902	2.21137	1.39499	2.42009	2.49383	1.44249	3.47395
XLOC_090870	MANEA	chr9	55149594	55224033	22.9213	4.55784	29.7879	0.687055	1.14501	4.61583	3.13285	0.714804	5.90984
XLOC_090871	MANEA	chr9	55149594	55224033	0.0	2.01594	1.75729	1.00686	0.695973	0.462923	0.0	0.663839	0.195559
XLOC_090872	MANEA	chr9	55149594	55224033	6.58127	2.36094	2.05817	0.707539	0.717839	0.817173	0.946994	1.04746	1.60708
XLOC_090873	MANEA	chr9	55149594	55224033	0.0	0.865667	4.52781	1.6862	1.12693	1.34737	1.68884	0.719044	1.00978
XLOC_090874	MANEA	chr9	55149594	55224033	0.0	3.12694	6.54188	1.12446	0.972596	1.29351	0.92815	0.618886	1.63859
XLOC_090875	MANEA	chr9	55149594	55224033	1.65409	2.22626	5.17555	0.148279	1.03497	1.63089	1.20085	0.827638	1.51738
XLOC_090876	MANEA	chr9	55149594	55224033	15.7528	9.39819	6.14221	6.37664	3.53733	2.37812	5.8005	2.94312	2.0305
XLOC_090877	MANEA	chr9	55149594	55224033	6.14557	1.83213	9.57975	7.71004	3.24349	3.72595	7.16818	2.90526	4.23125
XLOC_076925	MANSC1;LOH12CR1	chr5	97884077	98031099	16.972	12.0461	15.9634	0.194261	0.0784151	0.103516	0.0465239	0.150167	0.0583618
XLOC_058111	MAP1LC3C	chr16	35695103	35769971	12.8761	11.8595	12.178	6.80844	5.46132	5.98079	5.53365	5.68019	7.0014
XLOC_068144	MAP2	chr2	97949123	97949703	8.55981	16.8823	12.0411	8.43176	10.7686	8.83526	9.01819	11.8633	11.0323
XLOC_041849	MAP2K1;LCTL;ZWILCH;SNAPC5;RPL4	chr10	13247046	13474196	12.9253	9.29911	7.06556	2.75273	1.06478	0.699219	1.124	1.03913	2.23192
XLOC_041848	MAP2K1;TIPIN	chr10	13212327	13245929	6.57349	4.99978	3.85876	0.319333	0.537933	0.342298	0.401807	0.24837	0.863193
XLOC_041852	MAP2K5	chr10	14402360	14545460	1.2027	0.600376	0.633043	0.288315	0.253135	0.205328	0.370714	0.365527	0.52999
XLOC_042376	MAP2K5	chr10	14567217	14567340	0.0	83.3667	0.0	449.315	380.902	372.871	320.986	456.722	862.802
XLOC_037939	MAP3K13	chr1	82316549	82444287	6.24461	4.24188	5.54598	4.51394	5.70849	4.57384	4.88008	2.03771	5.39035
XLOC_064021	MAP3K14	chr19	45552277	45660314	7.75208	9.96699	9.04857	17.8652	16.0141	18.9991	18.8191	22.7435	18.3313
XLOC_064289	MAP3K14	chr19	45552277	45660314	3.37874	1.69579	2.11544	10.2404	10.8217	9.61777	8.02687	10.5347	10.5869
XLOC_064290	MAP3K14	chr19	45552277	45660314	2.57882	1.51456	1.97801	2.07824	2.53387	2.41225	1.6138	1.60506	1.5243
XLOC_089518	MAP3K5	chr9	75625723	75637772	4.23203	9.27145	7.2753	3.56944	2.2664	2.64862	2.84025	3.13144	2.23517
XLOC_089994	MAP3K5	chr9	75573421	75611136	3.31471	5.08295	1.94551	1.04032	0.97541	1.25029	1.25038	1.1669	0.870092
XLOC_091299	MAP3K5	chr9	75565565	75566357	0.0	1.36331	1.78276	0.408555	0.266785	0.354231	0.0	0.113635	0.198951
XLOC_091300	MAP3K5	chr9	75566512	75566854	0.0	4.6721	3.05418	1.40067	0.598152	0.798262	0.336521	0.377918	1.01559
XLOC_091301	MAP3K5	chr9	75567416	75567744	0.0	1.26288	0.0	0.0	0.645466	0.0	0.362105	0.407331	1.09732

XLOC_091302	MAP3K5	chr9	75567973	75568195	21.0445	6.28936	8.22005	2.86032	0.781023	0.0	0.84378	0.969237	0.903563
XLOC_091303	MAP3K5	chr9	75568413	75571077	0.577077	2.50398	2.93583	0.232892	0.385305	0.360565	0.238034	0.494136	0.303098
XLOC_091304	MAP3K5	chr9	75571427	75571853	0.0	3.21699	2.10313	0.241003	1.24549	0.0	0.0	1.05422	0.701316
XLOC_089993	MAP3K5;MAP7	chr9	75499239	75559665	32.5354	25.5869	32.8479	31.9601	32.1883	33.8806	30.115	30.4514	39.3337
XLOC_089943	MAP3K7	chr9	60587254	60688398	7.16381	8.51759	8.28617	1.77382	1.67884	1.51204	1.74125	2.54356	1.90897
XLOC_041685	MAP3K9	chr10	82699058	82741482	20.6901	24.0876	26.7497	3.54953	4.36078	3.17657	3.79751	4.91909	4.21311
XLOC_042086	MAP3K9	chr10	82749048	82764163	15.2875	13.4534	14.0541	51.9124	50.5331	51.8171	50.7855	52.7414	55.1781
XLOC_043168	MAP3K9	chr10	82699058	82741482	2.06964	1.23649	1.61679	1.29683	0.641083	0.852621	0.917939	0.408	1.25997
XLOC_043169	MAP3K9	chr10	82741776	82742570	0.0	0.679517	0.0	0.101818	0.0886515	0.117709	0.0	0.113283	0.297493
XLOC_043170	MAP3K9	chr10	82748093	82748698	1.61631	1.93218	1.26328	0.723768	0.628068	1.33545	0.867375	0.480579	0.985634
XLOC_043171	MAP3K9	chr10	82771520	82772009	0.0	0.651741	0.0	2.1483	1.68812	1.3474	0.579485	0.644338	0.379387
XLOC_043172	MAP3K9	chr10	82777609	82778172	0.0	0.0	0.0	2.55553	2.35349	1.65629	0.477154	0.529284	0.776481
XLOC_043173	MAP3K9	chr10	82795503	82796023	1.99537	0.596108	0.0	1.07178	4.1743	2.05597	1.41791	1.18112	1.56228
XLOC_043174	MAP3K9	chr10	82804849	82805820	0.0	0.0	0.0	1.11557	0.555996	1.01461	0.56412	0.533415	0.46594
XLOC_043988	MAP4K3;CDKL4	chr11	21572032	21885004	6.27456	2.90852	2.92583	0.967424	0.813841	1.05965	1.01476	1.05339	1.36077
XLOC_042654	MAP4K5	chr10	43476952	43478991	0.384389	0.230033	0.0	1.41325	0.905121	0.880101	0.527566	0.773534	0.403643
XLOC_042655	MAP4K5	chr10	43479056	43479217	0.0	10.9318	0.0	24.1377	15.473	14.3801	2.69754	34.4812	12.4337
XLOC_089516	MAP7	chr9	75441437	75477991	44.1013	45.9007	41.3243	76.4872	66.2149	69.6391	79.9703	92.8848	82.8207
XLOC_089517	MAP7	chr9	75481609	75484897	2.95796	7.9462	13.8527	11.9064	9.78156	9.09107	9.03994	15.8681	14.8751
XLOC_089992	MAP7	chr9	75386709	75410066	81.8441	85.0256	82.9578	63.2587	57.5499	59.5298	67.8857	72.2449	67.5337
XLOC_091288	MAP7	chr9	75384623	75385542	0.949783	1.42018	1.48571	1.10656	0.445257	0.393998	0.0859954	0.379612	0.331767
XLOC_091289	MAP7	chr9	75410215	75410617	0.0	0.882911	0.0	2.64586	0.454956	0.909107	0.0	1.15404	0.256467
XLOC_091290	MAP7	chr9	75412790	75413674	0.995544	0.595413	0.0	1.0706	1.94391	1.54824	0.810562	0.596518	1.39077
XLOC_091291	MAP7	chr9	75414163	75414451	5.50705	1.64165	4.29221	4.92988	1.6661	3.34549	0.462462	0.0	1.89756
XLOC_091292	MAP7	chr9	75417778	75418385	0.0	0.961799	0.0	1.58522	0.750371	1.66195	0.431815	0.159494	0.981273
XLOC_091293	MAP7	chr9	75441437	75477991	0.0	4.90157	3.66235	6.71447	4.16681	2.65224	4.34609	1.6134	6.92682
XLOC_091294	MAP7	chr9	75479322	75479666	3.87427	2.31123	3.02172	1.73218	3.25572	1.18488	2.66526	1.12217	0.669924
XLOC_091295	MAP7	chr9	75485056	75485517	9.53309	8.54079	5.58376	8.9576	9.38881	13.2292	11.7723	17.5521	13.458
XLOC_091296	MAP7	chr9	75485608	75485998	6.22077	5.56972	4.85481	16.4134	12.1852	13.3718	13.8149	16.6566	25.0678
XLOC_091297	MAP7	chr9	75486136	75486708	1.74501	2.60721	2.72737	6.25034	6.77381	6.12269	8.71961	8.80657	14.5867
XLOC_091298	MAP7	chr9	75497972	75499101	17.8672	13.1381	15.7247	13.4135	14.1581	15.7703	13.2618	13.349	19.323
XLOC_060308	MAPK1	chr17	74017328	74047848	0.0	0.0	0.562717	0.0644788	0.0	0.0	0.0	0.0	0.0

XLOC_055842	MAPK8IP1;SLC35C1;PEX16;C15H11orf94	chr15	76720938	76818160	7.09682	11.1026	11.9464	18.137	12.4098	15.0903	10.7668	17.9689	14.5064
XLOC_056995	MAPK8IP1;SLC35C1;PEX16;C15H11orf94	chr15	76720938	76818160	3.85384	2.29901	9.01725	4.47981	2.94453	4.32215	3.64651	4.83804	4.99829
XLOC_056996	MAPK8IP1;SLC35C1;PEX16;C15H11orf94	chr15	76720938	76818160	0.0	1.07317	2.8067	3.53766	2.51911	1.48678	2.48033	2.14547	1.67032
XLOC_056997	MAPK8IP1;SLC35C1;PEX16;C15H11orf94	chr15	76720938	76818160	8.62728	1.71685	0.0	5.40214	4.20459	4.42138	4.52633	5.8959	3.49183
XLOC_076519	MAPK8IP2	chr5	120116473	120141115	30.8176	24.4059	37.5444	53.3347	32.6913	38.2314	47.0386	66.8469	36.7066
XLOC_044393	MAPKAP1	chr11	96298901	96301499	2.94208	2.3804	6.14282	0.175773	0.0	0.0	0.0968788	0.0	0.0
XLOC_044394	MAPKAP1	chr11	96303925	96305271	1.52433	1.36679	0.0	0.0	0.0	0.0	0.0	0.0	0.0
XLOC_044395	MAPKAP1	chr11	96319053	96320547	1.33079	1.61733	1.06579	0.0	0.0	0.0	0.0	0.0	0.0
XLOC_044396	MAPKAP1	chr11	96341034	96345405	0.571504	0.854866	0.44717	0.0512388	0.0	0.0	0.0	0.0	0.0
XLOC_044397	MAPKAP1	chr11	96345631	96354817	1.01951	0.670092	2.32172	0.0565734	0.0713815	0.130345	0.0	0.126665	0.0
XLOC_044398	MAPKAP1	chr11	96365477	96367678	0.0	0.1332	0.0	0.0	0.0	0.0	0.0	0.0	0.0
XLOC_045029	MAPKAP1	chr11	96345631	96354817	0.64386	1.80202	0.194678	0.0985815	0.0382126	0.101149	0.0845069	3.24673E-6	0.10615
XLOC_047100	MAPKAP1	chr11	96289682	96290052	3.40234	5.07595	7.96377	0.0	0.0	0.347702	0.0	0.0	0.0
XLOC_047101	MAPKAP1	chr11	96297357	96298625	0.0	0.584469	0.0	0.0	0.0	0.0	0.0	0.0	0.0
XLOC_047103	MAPKAP1	chr11	96309509	96310991	0.0	0.163361	0.0	0.0	0.0	0.0	0.0	0.0	0.0
XLOC_047104	MAPKAP1	chr11	96317587	96318444	0.0	1.85512	0.808633	0.0	0.0	0.0	0.0	0.0	0.0
XLOC_047105	MAPKAP1	chr11	96320684	96321422	4.98382	4.09748	1.9484	0.223257	0.0971189	0.128977	0.0	0.0	0.0
XLOC_047107	MAPKAP1	chr11	96331986	96333181	0.0	0.208523	0.0	0.0	0.0	0.0	0.0	0.0	0.0
XLOC_047108	MAPKAP1	chr11	96335788	96336616	0.0	0.0	0.0	0.0	0.0	0.0	0.0	0.107614	0.0
XLOC_047110	MAPKAP1	chr11	96341034	96345405	0.0	0.814693	0.0	0.0	0.0	0.0	0.0	0.0	0.0
XLOC_047113	MAPKAP1	chr11	96367771	96368816	0.0	0.0	0.0	0.073045	0.0	0.0	0.0	0.0	0.0
XLOC_044399	MAPKAP1;PBX3	chr11	96464592	96758675	3.26102	2.95591	3.27853	2.69987	5.29541	2.01204	2.13921	2.38605	3.22705
XLOC_047117	MAPKAP1;PBX3	chr11	96464592	96758675	0.0	0.409103	0.0	0.735585	0.213144	1.55717	0.368884	0.136049	0.0
XLOC_052173	MAPRE1	chr13	62746785	62748511	0.461098	0.137959	0.0	0.124035	0.180846	0.239825	0.0842589	0.324458	0.363044

XLOC_052174	MAPRE1	chr13	62753005	62754669	0.0	0.0	0.375668	0.430458	0.37652	0.599198	0.219239	0.144742	0.167982
XLOC_064029	MAPT	chr19	46573287	46597067	2.98365	2.94193	3.01616	7.42411	5.52368	6.79268	5.09108	5.58283	4.80477
XLOC_064030	MAPT	chr19	46599217	46657652	13.1651	14.69	18.2317	10.1005	8.13673	9.37032	9.48846	12.7049	8.98748
XLOC_064299	MAPT	chr19	46531079	46573048	10.0844	9.99287	8.38313	18.703	21.3782	19.4518	19.4031	17.6399	17.6334
XLOC_065243	MAPT	chr19	46573287	46597067	0.0	0.0	0.0	6.49812	4.56952	5.1425	6.58896	6.61919	7.92397
XLOC_064310	MARCH10	chr19	47751847	47762006	0.0	0.529089	1.38368	0.3171	0.962067	1.82696	0.0	0.0	1.07914
XLOC_064311	MARCH10	chr19	47823031	47835995	2.10405	0.314579	0.0	1.22555	1.72474	1.3084	1.14087	0.524936	1.37754
XLOC_082875	MARCH3	chr7	28350664	28352767	3.60513	2.39712	3.76176	4.23855	4.90412	4.62696	6.77849	6.48777	4.45145
XLOC_083491	MARCH3	chr7	28284652	28289025	0.0	0.279272	0.0	0.0	0.0	0.0	0.0	0.0	0.0
XLOC_083492	MARCH3	chr7	28293695	28294568	0.0	2.45731	2.00629	1.68867	2.25103	2.13915	0.335728	0.931594	0.898691
XLOC_083493	MARCH3	chr7	28294620	28302285	0.0	0.244311	0.0	0.256263	0.256304	0.212412	0.0373384	0.0821325	0.0357225
XLOC_083494	MARCH3	chr7	28305619	28312262	27.7062	34.3721	26.8362	60.7912	55.7134	55.4122	55.8325	62.834	51.9555
XLOC_083495	MARCH3	chr7	28314753	28338848	202.204	980.697	345.263	503.049	347.298	454.109	429.29	622.791	255.52
XLOC_083496	MARCH3	chr7	28361848	28363115	32.0634	40.3246	35.9152	2.74356	2.68079	3.17843	1.78875	3.43154	2.35644
XLOC_083497	MARCH3	chr7	28364197	28372772	25.57	32.0274	36.4577	2.50935	2.75716	2.10488	2.66461	2.53071	2.0415
XLOC_083498	MARCH3	chr7	28377294	28379342	84.4723	55.6918	67.245	10.3786	9.22633	10.398	9.34167	7.39608	9.36106
XLOC_084469	MARCH3	chr7	28268255	28269654	8.73829	11.1537	9.572	11.0203	9.90637	10.2335	14.2324	12.6885	11.8183
XLOC_084470	MARCH3	chr7	28277744	28278233	2.18199	3.25871	3.40878	1.7577	1.85694	1.12284	0.193162	0.429559	1.32786
XLOC_084471	MARCH3	chr7	28278362	28279211	4.18375	1.25104	2.45393	1.03101	0.489955	0.758845	1.03987	0.417551	0.273919
XLOC_084472	MARCH3	chr7	28290258	28290728	0.0	0.0	0.0	0.207147	0.178889	0.0	0.204418	0.0	0.201132
XLOC_084474	MARCH3	chr7	28294620	28302285	0.0	1.4862	0.0	0.445999	0.0	0.0	0.0	0.0	0.0
XLOC_084476	MARCH3	chr7	28353920	28354520	3.26915	1.46549	2.55506	3.07412	3.17537	2.86957	1.02303	4.04918	0.996706
XLOC_084477	MARCH3	chr7	28359542	28361266	44.7836	50.0048	45.522	2.64946	2.82479	2.92959	2.82625	3.43434	1.97909
XLOC_084478	MARCH3	chr7	28376362	28377188	38.9493	39.7918	38.0741	5.04137	4.81333	5.71768	4.78834	4.53314	4.81622
XLOC_084479	MARCH3	chr7	28380900	28382212	5.63809	2.62363	1.96055	1.6287	1.22686	2.14826	0.513555	0.502761	0.876335
XLOC_084480	MARCH3	chr7	28385351	28385550	0.0	4.45685	0.0	1.36317	3.27642	1.48626	2.33103	1.34648	2.55317
XLOC_065774	MARCH4	chr2	104886322	104975662	1.69478	3.0446	6.21031	5.80598	7.88663	5.61974	5.03507	2.6002	7.23169
XLOC_066448	MARCH4	chr2	104757549	104818676	18.0948	18.951	16.9365	18.4086	16.9913	20.5802	14.0841	12.6931	11.7585
XLOC_066449	MARCH4	chr2	104820854	104849980	24.6712	30.1581	27.0599	28.2434	18.0242	26.2721	12.9772	13.8348	16.5678
XLOC_066450	MARCH4	chr2	104999527	105017122	19.5395	19.2512	19.4131	38.9525	35.1474	39.5165	38.0913	44.9826	48.2584
XLOC_068223	MARCH4	chr2	104757549	104818676	0.0	1.30401	6.81757	4.1013	3.88269	5.61418	5.0222	3.00691	3.98357
XLOC_068224	MARCH4	chr2	104819966	104820756	1.14351	3.41905	2.68258	7.47968	6.77974	6.15928	7.63432	7.75129	8.08289
XLOC_068225	MARCH4	chr2	104820854	104849980	7.6202	12.9093	6.951	10.0128	6.23548	6.57244	4.57259	5.43542	4.09878

XLOC_068226	MARCH4	chr2	104820854	104849980	21.0315	19.3377	24.3739	20.8069	11.5796	13.4241	7.92412	9.90552	10.8224
XLOC_068227	MARCH4	chr2	104820854	104849980	21.9593	27.0422	4.28594	18.4173	8.88026	16.3301	8.66065	6.17299	8.09755
XLOC_068228	MARCH4	chr2	104820854	104849980	34.7097	28.9784	37.8804	18.6919	15.6446	15.4002	7.45249	10.4398	11.3416
XLOC_068231	MARCH4	chr2	104999527	105017122	23.8743	4.74828	0.0	3.58013	4.76364	5.60576	5.85642	3.71518	6.83821
XLOC_068232	MARCH4	chr2	104999527	105017122	3.27926	1.95723	10.2354	2.63953	4.27698	4.02426	4.27158	3.82415	3.6924
XLOC_065775	MARCH4;SHOX	chr2	104980741	104999400	5.43489	5.4007	5.77124	5.41275	6.75359	7.47501	6.30116	4.67404	6.87541
XLOC_068229	MARCH4;SHOX	chr2	104980741	104999400	9.37138	0.932348	0.0	0.838173	2.39932	1.91835	1.63192	1.82463	3.24836
XLOC_068230	MARCH4;SHOX	chr2	104980741	104999400	0.0	3.51142	0.834865	2.48724	2.08331	2.98706	2.02542	1.2782	1.67735
XLOC_038435	MASP1	chr1	80593203	80665252	9.52221	4.91906	3.38559	0.0775874	0.0	0.0	0.157001	0.0865863	0.0
XLOC_051267	MASTL	chr13	18030309	18031630	48.4854	71.778	48.6348	78.2421	100.364	88.8857	100.34	70.0956	87.5006
XLOC_052501	MATN4	chr13	74358570	74359093	0.0	6.50346	4.63845	10.4529	12.8818	7.74933	11.0776	8.20159	14.6346
XLOC_052502	MATN4	chr13	74359196	74359749	9.14426	6.55741	11.4326	10.9714	13.6215	12.6385	17.1087	15.0056	15.6013
XLOC_052503	MATN4	chr13	74359911	74360206	5.23351	4.68047	0.0	6.08841	6.73929	6.89519	4.85199	2.99163	5.86353
XLOC_052504	MATN4	chr13	74360391	74362465	3.39632	2.82291	2.06731	2.6057	3.67305	2.7493	3.10799	2.24044	3.03816
XLOC_052505	MATN4	chr13	74362782	74364535	0.906589	1.76313	1.06417	0.65033	0.853424	0.707333	0.538493	0.501282	0.872447
XLOC_052506	MATN4	chr13	74364776	74365342	1.77064	1.05818	4.15104	0.475649	0.549753	0.365391	0.947526	0.52548	0.770814
XLOC_052507	MATN4;RBPJL	chr13	74370487	74371703	0.0	1.83949	0.0	1.59256	2.35404	1.56152	1.86514	1.57543	4.00166
XLOC_037920	MB21D2	chr1	75142900	75144604	18.4548	23.763	14.427	5.08652	6.51951	4.69741	4.71479	4.93622	5.69283
XLOC_037921	MB21D2	chr1	75144666	75148119	28.748	31.6973	29.8945	7.61285	9.02505	8.07247	8.46624	6.94536	6.78861
XLOC_037922	MB21D2	chr1	75168651	75178655	3.02321	5.02599	1.70842	3.92046	3.08165	4.69202	3.73297	4.22648	4.1109
XLOC_037923	MB21D2	chr1	75180857	75200316	3.58548	6.27704	5.20747	14.2858	13.3035	13.7704	13.6678	11.4818	15.6894
XLOC_037924	MB21D2	chr1	75210438	75219363	2.47745	3.49826	3.51531	8.99538	7.59683	8.21315	7.83679	9.68882	7.01703
XLOC_038418	MB21D2	chr1	75125543	75133979	1.05817	0.316412	1.28664	0.0	0.0556802	0.0	0.0	0.105589	0.0
XLOC_038419	MB21D2	chr1	75135689	75142726	16.032	20.9872	20.8199	4.64056	5.97855	5.19976	4.88022	3.63128	4.40499
XLOC_038420	MB21D2	chr1	75168651	75178655	9.70736	12.758	9.44313	3.91812	4.38807	3.61299	5.47844	4.70085	4.98973
XLOC_038421	MB21D2	chr1	75178934	75180539	9.16956	9.82794	5.37991	3.63024	2.98966	3.41151	3.81834	3.52023	3.67259
XLOC_038422	MB21D2	chr1	75180857	75200316	6.19982	13.0095	7.86565	7.34105	8.01529	7.11193	7.16259	6.45962	5.79962
XLOC_038423	MB21D2	chr1	75200404	75209223	1.45596	1.48175	1.29183	0.740438	1.11856	0.753717	0.793293	1.02355	1.41862
XLOC_038424	MB21D2	chr1	75210438	75219363	3.33015	2.87185	2.52508	36.3357	27.7168	31.0601	35.1832	36.4975	30.0596
XLOC_082999	MBD3;MBD3	chr7	45567789	45589709	9.0755	8.08966	6.14016	38.7047	22.4767	29.9909	28.7956	37.7826	21.9997

XLOC_083639	MBD3;MBD3	chr7	45567789	45589709	0.0	0.0	0.0	0.397618	0.555809	0.493141	0.13437	0.370234	0.0830503
XLOC_047907	MBNL2;RAP2A	chr12	78225788	78627262	13.9061	9.91687	9.79818	2.85287	4.08654	3.68605	2.81185	2.17479	3.5811
XLOC_049649	MBNL2;RAP2A	chr12	78225788	78627262	0.0	14.0164	12.2167	0.350267	0.897229	2.39479	0.336592	0.0	0.0
XLOC_049650	MBNL2;RAP2A	chr12	78225788	78627262	21.3406	18.2239	17.8725	14.7452	14.3448	20.0051	14.7491	18.452	13.018
XLOC_062151	MC1R;TCF25;TUBB3;SPIRE2;SPIRE2	chr18	14706063	14761937	5.73771	11.356	5.83626	16.6157	17.2269	15.5766	21.3667	15.8969	23.5332
XLOC_044003	MCFD2	chr11	29171154	29220892	27.1306	23.483	19.5673	19.5266	19.8981	19.5632	23.0181	25.6536	23.1301
XLOC_053210	MCM4;UBE2V2;PRKDC	chr14	21039936	21448571	41.9041	49.3977	36.5477	67.363	68.0156	70.6255	64.5536	57.9522	80.423
XLOC_053498	MCM4;UBE2V2;PRKDC	chr14	21039936	21448571	11.6178	9.73011	9.14959	13.0339	14.3236	14.2321	12.7709	15.0765	19.1474
XLOC_053943	MCM4;UBE2V2;PRKDC	chr14	21039936	21448571	0.0	1.15111	4.51531	1.55212	2.3894	0.992918	0.856689	2.09216	0.502941
XLOC_053944	MCM4;UBE2V2;PRKDC	chr14	21039936	21448571	1.1694	1.39861	3.65775	0.628669	2.28023	1.09005	0.632777	0.582666	2.44897
XLOC_053945	MCM4;UBE2V2;PRKDC	chr14	21039936	21448571	1.39825	0.836545	0.547012	0.626725	1.04	0.653597	0.635991	0.700727	0.42779
XLOC_076882	MCM5;HMOX1	chr5	73976480	74068417	7.25353	6.51169	3.26671	18.967	26.2147	21.2441	25.8743	22.9282	30.6312
XLOC_050833	MCM8	chr13	48490414	48498757	6.88255	8.65996	8.48331	1.94487	1.58392	1.70507	1.50777	2.25025	1.9224
XLOC_051779	MCM8	chr13	48489644	48490272	12.2983	12.4056	18.0243	2.47838	2.51051	3.17695	4.2685	2.89765	3.34898
XLOC_051780	MCM8	chr13	48532690	48534427	5.49464	3.83595	4.29976	3.32563	5.13631	3.4295	7.90749	4.97126	6.92999
XLOC_069918	MCOLN2	chr3	59294084	59344550	0.841495	0.782728	0.900611	0.0764423	0.109092	0.101646	0.109731	0.022251	0.117606
XLOC_070302	MCOLN2	chr3	59294084	59344550	2.69074	0.541968	0.570059	0.706676	0.37343	0.541451	0.703498	0.600873	0.319867
XLOC_083111	MCTP1;ANKRD32;MCTP1	chr7	96210119	96896256	6.40455	3.22617	2.7832	13.2811	7.49498	8.88097	9.33752	11.6157	9.58243
XLOC_083112	MCTP1;ANKRD32;MCTP1	chr7	96210119	96896256	0.0	1.2372	0.0	2.5139	3.51678	4.00757	3.80915	3.4427	4.34928
XLOC_085293	MCTP1;ANKRD32;MCTP1	chr7	96210119	96896256	0.0	0.137048	0.0	3.22421	2.1234	2.1468	1.99614	2.12368	2.84688
XLOC_085294	MCTP1;ANKRD32;MCTP1	chr7	96210119	96896256	0.0	0.0	0.0	2.2255	1.47587	1.8373	2.55918	2.35696	2.37363

XLOC_085295	MCTP1;ANKRD32;MCTP1	chr7	96210119	96896256	0.0	3.13549	0.0	3.28855	2.22659	1.88643	3.22818	2.31361	3.19045
XLOC_085296	MCTP1;ANKRD32;MCTP1	chr7	96210119	96896256	0.0	0.666957	0.0	2.69828	1.65349	2.31092	1.61077	1.33459	1.55739
XLOC_085297	MCTP1;ANKRD32;MCTP1	chr7	96210119	96896256	0.0	0.368065	0.0	2.20601	1.34363	2.4216	2.43959	2.57439	2.36277
XLOC_085298	MCTP1;ANKRD32;MCTP1	chr7	96210119	96896256	0.0	0.0	0.0	1.03546	1.48435	1.19899	1.19815	0.908346	1.15346
XLOC_085299	MCTP1;ANKRD32;MCTP1	chr7	96210119	96896256	0.0	0.0	0.0	2.16976	1.63144	2.17365	3.17319	2.06854	1.05138
XLOC_085300	MCTP1;ANKRD32;MCTP1	chr7	96210119	96896256	0.0	0.0	0.0	1.31156	1.19394	1.5204	1.33296	1.40674	0.692861
XLOC_085301	MCTP1;ANKRD32;MCTP1	chr7	96210119	96896256	0.0	0.267562	0.0	2.48577	1.89418	1.86064	1.67542	2.20278	2.5818
XLOC_085302	MCTP1;ANKRD32;MCTP1	chr7	96210119	96896256	0.0	0.0	0.0	1.79758	1.08047	1.91425	1.93571	1.83776	1.74887
XLOC_085303	MCTP1;ANKRD32;MCTP1	chr7	96210119	96896256	0.0	0.0	0.0	1.66569	1.45003	1.32372	1.15268	1.73692	1.31801
XLOC_085304	MCTP1;ANKRD32;MCTP1	chr7	96210119	96896256	0.0	0.484127	1.26613	2.32124	1.5107	3.01144	2.02828	2.08708	1.97566
XLOC_085305	MCTP1;ANKRD32;MCTP1	chr7	96210119	96896256	0.0	0.0	0.462519	2.59684	1.76016	2.58052	1.778	2.19508	1.8091
XLOC_085306	MCTP1;ANKRD32;MCTP1	chr7	96210119	96896256	0.0	0.0	0.0	2.74025	2.60728	2.40967	2.22147	1.30196	2.41264
XLOC_085307	MCTP1;ANKRD32;MCTP1	chr7	96210119	96896256	0.0	0.0	0.0	4.95949	2.94754	0.792787	6.44502	0.735788	1.35364
XLOC_085308	MCTP1;ANKRD32;MCTP1	chr7	96210119	96896256	0.0	0.0	0.0	2.86892	1.96256	2.36897	1.75383	1.70985	2.59451
XLOC_085309	MCTP1;ANKRD32;MCTP1	chr7	96210119	96896256	0.0	0.0	0.0	2.09779	1.81392	2.41281	2.26542	2.93787	3.5199

XLOC_085310	MCTP1;ANKRD32;MCTP1	chr7	96210119	96896256	0.0	0.0	1.63079	3.92411	1.77788	4.51439	1.85115	3.49716	2.90469
XLOC_085311	MCTP1;ANKRD32;MCTP1	chr7	96210119	96896256	0.0	0.458493	1.19909	3.43491	1.07368	3.96286	2.88561	2.89162	2.13883
XLOC_085312	MCTP1;ANKRD32;MCTP1	chr7	96210119	96896256	0.0	0.0	0.0	5.27128	3.18784	4.60497	5.10267	2.69004	3.00144
XLOC_085313	MCTP1;ANKRD32;MCTP1	chr7	96210119	96896256	0.0	0.0	0.0	3.4606	3.39321	3.81603	3.30214	3.66059	3.80463
XLOC_085314	MCTP1;ANKRD32;MCTP1	chr7	96210119	96896256	0.0	0.566584	0.740925	2.80162	1.55435	2.16136	1.97281	3.69164	2.72993
XLOC_085315	MCTP1;ANKRD32;MCTP1	chr7	96210119	96896256	0.0	0.266036	0.0	2.76396	1.95522	2.19094	2.4172	2.47755	2.64593
XLOC_085316	MCTP1;ANKRD32;MCTP1	chr7	96210119	96896256	0.0	0.0	0.0	3.21668	1.91715	3.1204	1.69625	1.35188	3.59949
XLOC_085317	MCTP1;ANKRD32;MCTP1	chr7	96210119	96896256	0.0	0.164603	0.0	2.3185	1.63884	1.88767	2.45917	1.49167	2.30963
XLOC_085318	MCTP1;ANKRD32;MCTP1	chr7	96210119	96896256	0.0	0.237327	0.0	1.42247	0.869046	0.988438	1.00872	2.14479	1.1093
XLOC_085319	MCTP1;ANKRD32;MCTP1	chr7	96210119	96896256	0.0	0.0	0.0	1.91069	1.20109	1.22687	0.85443	1.18038	0.826957
XLOC_085320	MCTP1;ANKRD32;MCTP1	chr7	96210119	96896256	0.0	1.66884	0.0	0.666795	0.433233	0.959992	1.16044	0.551926	0.810233
XLOC_085321	MCTP1;ANKRD32;MCTP1	chr7	96210119	96896256	0.0	0.165865	0.0	1.04386	0.912589	1.09514	0.556247	0.723682	1.26062
XLOC_085322	MCTP1;ANKRD32;MCTP1	chr7	96210119	96896256	0.0	0.0	0.0	0.831304	0.889581	0.929261	1.19466	0.794949	0.871406
XLOC_085323	MCTP1;ANKRD32;MCTP1	chr7	96210119	96896256	0.0	0.0	0.0	1.37455	0.0	2.10333	2.02619	1.00357	0.88918
XLOC_085324	MCTP1;ANKRD32;MCTP1	chr7	96210119	96896256	0.0	0.591225	0.0	1.24018	0.920132	2.85504	0.527511	0.390554	0.516516

XLOC_085325	MCTP1;ANKRD32;MCTP1	chr7	96210119	96896256	0.0	0.0	1.24661	0.142847	0.371922	0.658995	0.285414	0.31623	1.11162
XLOC_085326	MCTP1;ANKRD32;MCTP1	chr7	96210119	96896256	0.0	0.33764	1.76609	1.61893	1.05724	1.40376	0.917144	1.46361	1.97095
XLOC_085327	MCTP1;ANKRD32;MCTP1	chr7	96210119	96896256	0.0	0.0	0.0	0.866784	0.819479	1.00378	0.365793	0.483983	1.4786
XLOC_085328	MCTP1;ANKRD32;MCTP1	chr7	96210119	96896256	0.0	0.0	0.0	0.775694	0.941617	1.78801	0.773122	0.51439	0.905161
XLOC_085329	MCTP1;ANKRD32;MCTP1	chr7	96210119	96896256	0.0	0.0	0.0	1.60057	0.458627	2.13846	0.260307	0.290808	3.61981
XLOC_085330	MCTP1;ANKRD32;MCTP1	chr7	96210119	96896256	0.0	0.414589	0.0	0.621209	0.647917	0.430351	0.37371	0.413533	0.846465
XLOC_085331	MCTP1;ANKRD32;MCTP1	chr7	96210119	96896256	0.0	0.0	0.0	1.08421	0.748483	0.747113	0.427182	1.66485	3.36774
XLOC_085332	MCTP1;ANKRD32;MCTP1	chr7	96210119	96896256	0.0	0.0	0.0	1.13743	0.392291	1.30549	0.89449	1.74428	0.882987
XLOC_085333	MCTP1;ANKRD32;MCTP1	chr7	96210119	96896256	0.0	0.0	0.0	1.89201	0.595773	1.7824	1.53789	2.08673	0.501529
XLOC_085334	MCTP1;ANKRD32;MCTP1	chr7	96210119	96896256	0.0	1.18715	0.0	2.84733	1.82312	2.43337	2.39187	1.9193	2.06414
XLOC_085335	MCTP1;ANKRD32;MCTP1	chr7	96210119	96896256	0.0	0.0	0.887204	0.813273	0.354058	0.58764	0.614247	1.4704	0.594056
XLOC_085336	MCTP1;ANKRD32;MCTP1	chr7	96210119	96896256	0.0	0.0	0.487193	1.06066	0.878045	0.841211	0.850827	0.999508	1.3066
XLOC_085337	MCTP1;ANKRD32;MCTP1	chr7	96210119	96896256	0.0	0.0	0.0	1.78123	1.36812	0.605562	0.843615	1.3984	1.73465
XLOC_085338	MCTP1;ANKRD32;MCTP1	chr7	96210119	96896256	1.05977	0.316882	0.0	1.13956	0.661818	1.20811	0.861803	0.528733	1.5726
XLOC_085339	MCTP1;ANKRD32;MCTP1	chr7	96210119	96896256	0.0	0.0	0.0	0.939015	0.315185	0.250952	1.24369	0.564647	0.633686

XLOC_085340	MCTP1;ANKRD32;MCTP1	chr7	96210119	96896256	0.0	0.0	0.0	0.855226	0.689291	0.83834	0.800572	0.955832	0.384833
XLOC_085341	MCTP1;ANKRD32;MCTP1	chr7	96210119	96896256	0.0	0.0	0.0	1.83241	0.752225	1.01571	0.0	0.0	2.60785
XLOC_085342	MCTP1;ANKRD32;MCTP1	chr7	96210119	96896256	0.0	0.0	0.0	0.996463	1.10519	1.15273	0.365687	1.61487	1.14731
XLOC_085343	MCTP1;ANKRD32;MCTP1	chr7	96210119	96896256	0.0	0.0	0.0	0.456577	0.393647	1.31003	0.224379	0.750107	0.664555
XLOC_085344	MCTP1;ANKRD32;MCTP1	chr7	96210119	96896256	0.0	0.0	1.58513	0.908161	1.88597	0.627067	0.720452	0.600258	1.4119
XLOC_085345	MCTP1;ANKRD32;MCTP1	chr7	96210119	96896256	0.0	0.0	0.0	2.42579	0.951231	0.759649	0.868272	1.93414	1.07019
XLOC_085346	MCTP1;ANKRD32;MCTP1	chr7	96210119	96896256	0.0	0.0	0.0	0.589455	0.339644	0.677763	0.38858	0.864197	0.954181
XLOC_085347	MCTP1;ANKRD32;MCTP1	chr7	96210119	96896256	0.0	0.0	0.0	0.576531	0.334802	0.444496	0.484347	0.534923	1.02959
XLOC_085348	MCTP1;ANKRD32;MCTP1	chr7	96210119	96896256	0.0	0.0	0.0	0.497541	1.00577	0.764109	1.31965	2.19664	1.61222
XLOC_085349	MCTP1;ANKRD32;MCTP1	chr7	96210119	96896256	0.0	0.0	0.0	1.0859	1.01083	1.59284	0.879832	1.536	1.12906
XLOC_085350	MCTP1;ANKRD32;MCTP1	chr7	96210119	96896256	0.0	0.0	0.0	1.09469	0.715441	0.633166	1.10463	1.01631	2.39959
XLOC_085351	MCTP1;ANKRD32;MCTP1	chr7	96210119	96896256	0.0	0.33764	0.0	1.41657	2.11447	1.52073	0.407621	0.675514	1.47821
XLOC_085352	MCTP1;ANKRD32;MCTP1	chr7	96210119	96896256	0.679444	0.0	0.531558	0.852706	0.744816	0.846964	0.989207	1.15794	1.60354
XLOC_085353	MCTP1;ANKRD32;MCTP1	chr7	96210119	96896256	0.0	0.0	0.0	1.39081	0.875046	1.35423	1.41323	0.498052	1.84468
XLOC_085354	MCTP1;ANKRD32;MCTP1	chr7	96210119	96896256	0.0	0.485212	0.0	1.16322	0.883197	1.17373	0.725965	0.965389	0.707166

XLOC_085355	MCTP1;ANKRD32;MCTP1	chr7	96210119	96896256	0.0	0.0	0.0	1.47899	0.636675	1.69568	0.965776	0.538762	0.956246
XLOC_085356	MCTP1;ANKRD32;MCTP1	chr7	96210119	96896256	0.0	0.0	0.0	1.72302	1.47225	2.7504	1.65751	2.23294	5.99771
XLOC_085357	MCTP1;ANKRD32;MCTP1	chr7	96210119	96896256	0.0	0.0	0.0	0.671624	0.250733	0.332885	1.25747	0.213657	0.934609
XLOC_072646	MDFIC	chr4	53713951	53762823	1.75147	2.55551	3.34198	5.99845	5.92351	6.12402	7.48799	6.33063	7.24632
XLOC_072647	MDFIC	chr4	53779708	53787910	0.0	0.431199	0.0	0.484602	0.311108	0.412492	0.0989351	0.1813	0.126114
XLOC_072648	MDFIC	chr4	53826024	53860644	7.56968	10.5894	8.38704	10.8859	9.0785	10.0528	8.22406	10.148	10.4241
XLOC_074249	MDFIC	chr4	53762959	53764439	1.64061	2.61773	2.56744	7.89408	8.18772	7.90312	8.58048	6.97423	8.13057
XLOC_074250	MDFIC	chr4	53764618	53764944	4.28456	3.83306	0.0	9.96184	11.0982	13.5138	6.95553	12.3572	12.2108
XLOC_074251	MDFIC	chr4	53765102	53765919	2.19338	0.327921	1.71525	2.84986	3.08112	3.52256	2.77278	2.9534	2.87158
XLOC_074252	MDFIC	chr4	53791169	53791381	24.1722	47.0195	28.3627	60.5087	35.7439	60.5309	41.2962	68.5703	70.5794
XLOC_089447	MDN1	chr9	61372512	61418578	82.1508	93.1236	109.726	31.7697	28.4029	33.6553	43.9781	47.543	34.5517
XLOC_091011	MDN1	chr9	61306131	61306456	0.0	0.0	0.0	5.78122	2.62634	2.19226	0.736202	1.24271	2.9775
XLOC_091012	MDN1	chr9	61322654	61323970	0.624291	0.186756	0.0	0.0559684	0.0	0.0648749	0.0	0.0	0.0
XLOC_091013	MDN1	chr9	61335906	61336514	0.0	0.479833	0.0	0.0	0.0	0.0	0.0	0.0	0.0
XLOC_091014	MDN1	chr9	61364745	61365236	0.0	0.647841	0.0	0.0	0.0	0.0	0.0	0.0	0.0
XLOC_091015	MDN1	chr9	61365870	61367274	0.0	0.520734	0.0	0.0520194	0.0909385	0.0603112	0.0528954	0.0	0.202947
XLOC_091016	MDN1	chr9	61368190	61369187	1.72306	1.28833	2.69557	0.38609	0.0673619	0.0	0.0781323	0.0	0.150518
XLOC_091017	MDN1	chr9	61369251	61369994	1.23531	0.0	0.0	0.553383	0.192595	0.0	0.222521	0.0	0.0
XLOC_091018	MDN1	chr9	61371102	61372372	0.650106	2.1392	2.03452	1.63187	1.06927	0.878106	0.947039	1.43437	0.511505
XLOC_091019	MDN1	chr9	61372512	61418578	9.3356	18.0923	18.1954	3.3403	6.03076	6.16446	5.15521	3.57654	5.23577
XLOC_091020	MDN1	chr9	61372512	61418578	6.84103	4.54713	7.73029	2.86174	2.55768	3.94628	3.10793	2.13162	2.45771
XLOC_091021	MDN1	chr9	61372512	61418578	9.9731	4.95991	2.59438	2.37864	2.5496	3.73932	2.30781	1.2917	1.1516
XLOC_091022	MDN1	chr9	61418673	61419769	0.770644	0.0	1.20572	0.898021	0.542698	1.28017	0.700077	0.077173	0.538736
XLOC_072093	MEAF6	chr3	108971098	108972010	5.75157	4.30002	6.74768	0.343636	0.524249	0.397631	0.347117	0.0957733	0.251126
XLOC_072094	MEAF6	chr3	108972306	108973132	14.065	11.6464	10.1531	0.969494	0.337778	0.672669	0.977212	0.863455	0.283307
XLOC_064827	MED11	chr19	27253393	27254192	2.25493	0.337113	0.0	0.505127	1.40747	1.86876	1.01749	0.449649	0.688761
XLOC_064828	MED11	chr19	27254313	27254766	2.44778	0.730954	0.0	0.0	0.188982	0.251518	0.215662	0.0	0.0
XLOC_040117	MED12L	chr1	117885746	117885912	30.7774	37.4933	98.054	91.1422	48.9729	43.2523	49.0196	24.406	69.3837
XLOC_040118	MED12L	chr1	117887668	117887997	4.21033	0.0	0.0	3.01202	2.56737	2.14262	2.1609	3.24064	2.18211

XLOC_059899	MED15	chr17	74489489	74500449	12.3794	11.8097	12.7131	15.2608	17.1438	14.9739	18.5098	18.8352	21.2883
XLOC_066517	MED18	chr2	125628607	125657573	2.48106	1.11354	1.61801	0.185399	0.0973465	0.129083	0.0378151	0.0415919	0.0
XLOC_057204	MED19	chr15	82355898	82357186	0.639754	1.33965	0.500536	0.745599	1.25277	0.465327	0.407811	0.962532	1.06269
XLOC_057205	MED19	chr15	82357314	82358047	0.0	1.12719	0.0	0.675584	0.881589	1.30089	0.678897	0.500429	1.42516
XLOC_057206	MED19	chr15	82358557	82359078	1.98988	0.594471	1.55464	0.8907	1.54185	0.615114	0.0	0.785299	1.03868
XLOC_044491	MED22;ABO;SURF6;RPL7A;SURF4	chr11	104231581	104330069	0.597072	1.28469	1.03739	3.74056	3.26545	3.44385	5.31296	8.87835	6.66993
XLOC_045134	MED22;ABO;SURF6;RPL7A;SURF4	chr11	104231581	104330069	1.37827	0.20614	0.0	1.54444	1.08647	1.18754	1.25396	1.17438	0.662615
XLOC_089494	MED23;ENPP3	chr9	70542472	70592304	43.4673	45.9596	34.2371	41.5193	39.1938	42.5039	54.5864	49.4138	45.7479
XLOC_091212	MED23;ENPP3	chr9	70542472	70592304	1.95756	7.60285	15.2944	4.38132	6.52396	7.86819	7.48198	5.98957	5.79092
XLOC_091213	MED23;ENPP3	chr9	70542472	70592304	12.4572	5.11931	6.08558	6.27587	6.2958	7.40047	8.78358	5.55986	7.19105
XLOC_091214	MED23;ENPP3	chr9	70542472	70592304	3.64133	4.34521	0.0	2.2793	1.67179	2.60157	2.20018	0.704897	3.15034
XLOC_091215	MED23;ENPP3	chr9	70542472	70592304	6.14167	2.74954	2.39662	1.09865	3.30341	4.08716	2.40857	2.99131	1.59692
XLOC_091216	MED23;ENPP3	chr9	70542472	70592304	1.20046	3.58911	5.632	1.82847	2.24656	2.73465	4.11124	1.07624	2.40893
XLOC_091217	MED23;ENPP3	chr9	70542472	70592304	0.0	2.37429	0.0	2.4914	3.03851	2.43332	3.07527	2.30315	1.37609
XLOC_091218	MED23;ENPP3	chr9	70542472	70592304	1.56389	2.80441	3.66711	2.10099	3.7693	2.74667	5.32029	2.32644	4.63296
XLOC_082753	MED26	chr7	6343723	6346778	0.0	0.520189	0.0	1.09118	1.21639	1.7965	0.621378	1.37817	0.606327
XLOC_082754	MED26	chr7	6349219	6353709	0.0	0.895583	0.0	1.78926	2.33908	2.38048	2.25766	1.69474	2.00473
XLOC_082755	MED26	chr7	6375520	6378527	0.0	0.845033	2.20975	3.03876	3.26859	2.90216	1.9818	4.42416	5.89302
XLOC_084114	MED26	chr7	6354359	6354846	0.0	0.0	1.71472	5.30503	3.90585	4.97007	3.88576	4.96912	5.72508
XLOC_084115	MED26	chr7	6379421	6380068	0.0	1.32496	0.0	2.38232	1.49451	1.52737	0.927451	1.32025	1.93181
XLOC_044473	MED27	chr11	102053477	102054764	0.0	0.0	0.0	0.0	0.0900536	0.0	0.0	0.0	0.0
XLOC_044474	MED27	chr11	102146788	102149384	3.03727	1.36271	4.75204	0.816767	0.415932	0.3942	0.275973	0.532344	0.928969
XLOC_044475	MED27	chr11	102189621	102194140	9.84442	7.99439	12.6421	6.49346	10.0302	7.3122	9.08101	6.56412	8.47696
XLOC_045103	MED27	chr11	101972800	101984772	11.4794	8.07362	12.5611	7.75673	8.98923	8.23297	3.66394	4.16414	5.25636
XLOC_045104	MED27	chr11	102000568	102026958	9.22976	10.7199	13.6903	2.98744	1.4316	4.45805	1.31605	1.81843	1.28451
XLOC_045105	MED27	chr11	102057024	102088486	1.30382	0.278617	0.874748	0.818556	1.59612	1.4126	0.598756	0.413236	0.766569

XLOC_045106	MED27	chr11	102106679	102107804	3.95797	0.0	0.0	0.0	0.153355	0.407859	0.0	0.0	0.344344
XLOC_045107	MED27	chr11	102152545	102157445	6.36705	8.65219	8.59708	10.9978	12.3316	11.248	11.2327	10.8575	14.2711
XLOC_045108	MED27	chr11	102163593	102185217	28.0911	40.3956	29.7847	33.4359	26.9233	33.5535	33.4145	41.0731	31.7644
XLOC_045109	MED27	chr11	102195570	102202496	22.6789	19.5652	19.1561	10.7781	11.2443	10.1126	8.57778	11.4434	10.5841
XLOC_047309	MED27	chr11	102055222	102055621	0.0	0.893835	2.33734	0.0	0.230236	0.0	0.0	0.0	0.0
XLOC_047310	MED27	chr11	102124045	102124656	17.5441	23.8332	16.2057	30.5681	24.7946	28.3359	31.8233	43.1652	40.2962
XLOC_047311	MED27	chr11	102159391	102160287	0.979366	0.87862	0.0	0.438843	0.459016	0.507743	0.443127	0.29347	0.256547
XLOC_047312	MED27	chr11	102161447	102161731	5.67609	3.38406	4.42389	2.03297	1.28678	2.87186	0.951246	1.07741	1.46645
XLOC_047313	MED27	chr11	102162165	102163485	30.485	37.2227	37.9678	44.3976	35.5303	40.7311	39.8415	53.3656	40.9593
XLOC_047314	MED27	chr11	102163593	102185217	4.6105	3.44627	1.80262	0.929492	0.539557	0.596924	1.24765	0.689311	0.603484
XLOC_083486	MEGF10	chr7	27964626	27971748	3.41837	3.05988	8.00119	3.66836	1.83393	2.44517	4.14618	3.6485	5.91893
XLOC_083487	MEGF10	chr7	27974017	27979287	0.86049	0.0	0.673076	1.00264	0.613387	0.589761	1.08236	0.706643	0.411042
XLOC_083488	MEGF10	chr7	27990212	28005353	22.6428	38.3133	29.4683	41.4214	37.4683	36.4358	52.4701	49.5788	60.733
XLOC_084433	MEGF10	chr7	27878969	27879169	0.0	4.38029	0.0	6.6955	1.07413	4.38352	3.44076	10.5975	12.5485
XLOC_084434	MEGF10	chr7	27879607	27879822	11.5748	0.0	18.1005	6.31233	3.42738	0.0	7.37871	11.675	10.9338
XLOC_084435	MEGF10	chr7	27882013	27885111	0.983805	3.75376	2.31011	4.74258	5.48869	5.02134	4.44483	3.86719	4.93117
XLOC_084436	MEGF10	chr7	27886375	27887156	0.0	1.38734	0.0	0.311816	0.361931	0.600726	0.313872	0.346835	0.809786
XLOC_084437	MEGF10	chr7	27907674	27908315	0.0	1.34138	0.0	1.6079	1.51283	1.85539	1.47502	1.18785	0.651884
XLOC_084438	MEGF10	chr7	27909081	27909999	4.75516	0.568817	2.2315	1.36371	2.3035	2.16982	3.44426	2.09058	3.15591
XLOC_084439	MEGF10	chr7	27910214	27910588	0.0	0.996538	2.60583	2.98668	3.58547	2.04848	1.73832	4.54063	5.49488
XLOC_084440	MEGF10	chr7	27911138	27911418	5.85539	15.7094	27.3817	6.81774	8.84142	7.6983	5.87457	9.43325	6.04949
XLOC_084441	MEGF10	chr7	27948936	27949795	5.15513	12.6406	16.1266	6.19035	5.1523	4.06109	7.17727	7.10136	5.76062
XLOC_084442	MEGF10	chr7	27959166	27959350	39.5464	5.96173	15.5869	3.6804	2.88721	0.0	4.58383	5.3128	3.40692
XLOC_084443	MEGF10	chr7	27959507	27959805	0.0	4.5828	3.9941	5.04351	3.1071	2.59765	2.59512	5.86405	1.76684
XLOC_084444	MEGF10	chr7	27960090	27960311	21.3237	3.18676	8.33	4.8324	4.74653	6.41467	1.70843	6.87032	6.40882
XLOC_084445	MEGF10	chr7	27961243	27962605	3.60269	4.49068	1.40941	3.22993	2.96397	2.05941	2.29821	4.15763	3.41264
XLOC_084446	MEGF10	chr7	27962883	27963078	15.9407	19.152	25.0309	13.2079	4.67957	14.3528	2.49152	21.6077	13.7057
XLOC_084447	MEGF10	chr7	27974017	27979287	0.929041	1.11135	0.0	0.166526	0.217807	0.0963608	0.336599	0.278562	0.243409
XLOC_084448	MEGF10	chr7	27982790	27984487	1.87914	0.421669	0.0	0.463358	0.663263	0.586391	0.600826	0.141658	0.945226
XLOC_084449	MEGF10	chr7	27984886	27985880	4.3231	2.58589	0.0	4.18471	3.7181	3.22974	3.842	4.75661	5.51355
XLOC_084450	MEGF10	chr7	27986917	27987916	3.43793	9.25392	8.73981	15.2529	11.8949	13.8236	17.8505	18.4847	19.1456
XLOC_084451	MEGF10	chr7	27988045	27988291	0.0	9.49598	12.4123	21.4797	14.2905	23.223	20.1717	30.4645	34.8742
XLOC_084452	MEGF10	chr7	27988394	27988815	2.74489	14.7503	8.5716	16.2072	17.7605	16.3301	23.576	27.3759	19.7676

XLOC_084453	MEGF10	chr7	27989453	27989911	7.22118	14.3764	20.6777	24.5556	17.1026	22.0181	23.9818	25.0476	33.4552
XLOC_082867	MEGF10;PRRC1	chr7	27698658	27878750	0.0	1.29517	0.0	14.3507	15.4146	14.7502	14.9306	19.8262	17.2799
XLOC_082868	MEGF10;PRRC1	chr7	27698658	27878750	0.0	0.887033	1.01616	1.61765	2.40789	1.08911	1.11428	1.6646	1.95333
XLOC_082869	MEGF10;PRRC1	chr7	27698658	27878750	16.4708	4.915	5.14083	17.6762	18.9524	17.179	17.7314	15.3635	26.8185
XLOC_082870	MEGF10;PRRC1	chr7	27698658	27878750	3.29416	1.96602	7.71124	13.8464	9.09717	15.8317	15.1575	18.2442	18.83
XLOC_082871	MEGF10;PRRC1	chr7	27698658	27878750	2.02895	4.849	11.0957	14.8937	15.7164	18.8113	19.272	33.0141	24.5317
XLOC_083485	MEGF10;PRRC1	chr7	27698658	27878750	11.388	16.6677	12.6293	13.8351	16.2119	15.6385	14.772	15.7106	24.5214
XLOC_084417	MEGF10;PRRC1	chr7	27698658	27878750	0.0	0.253156	0.0	0.303454	0.26475	0.35134	1.3053	0.508086	0.295768
XLOC_084418	MEGF10;PRRC1	chr7	27698658	27878750	0.0	0.0	0.0	8.06949	10.644	11.9717	6.61169	9.09158	16.9796
XLOC_084419	MEGF10;PRRC1	chr7	27698658	27878750	0.0	3.38413	0.0	7.62363	8.57855	6.31809	7.60997	12.9289	10.2652
XLOC_084420	MEGF10;PRRC1	chr7	27698658	27878750	0.495535	0.296521	0.77552	4.93187	3.80825	5.10209	4.43419	5.37751	5.85163
XLOC_084421	MEGF10;PRRC1	chr7	27698658	27878750	0.56525	0.676421	0.44228	0.405428	0.620225	0.52885	0.463909	0.510732	0.0988619
XLOC_084422	MEGF10;PRRC1	chr7	27698658	27878750	10.9326	16.2951	17.0419	14.6794	14.0601	14.9454	12.4007	12.993	24.4873
XLOC_084423	MEGF10;PRRC1	chr7	27698658	27878750	7.16404	8.76705	6.11454	12.1443	12.0381	10.8268	9.72442	11.3649	15.8851
XLOC_084424	MEGF10;PRRC1	chr7	27698658	27878750	3.23536	0.0	0.0	0.0	0.0	0.0	0.0	0.314568	0.0
XLOC_084425	MEGF10;PRRC1	chr7	27698658	27878750	0.0	0.491846	0.0	0.0	0.0	0.0	0.0	0.0	0.0
XLOC_084426	MEGF10;PRRC1	chr7	27698658	27878750	0.0	0.701553	0.0	1.26141	0.457503	0.1215	0.528932	0.818341	0.614207
XLOC_084427	MEGF10;PRRC1	chr7	27698658	27878750	0.0	8.84619	0.0	5.35683	4.40435	6.93458	10.3356	13.6816	10.1728
XLOC_084428	MEGF10;PRRC1	chr7	27698658	27878750	0.0	0.0	9.05024	2.10411	7.7116	4.63926	6.45637	12.7363	12.9218
XLOC_084429	MEGF10;PRRC1	chr7	27698658	27878750	0.0	4.04189	0.0	4.84554	3.63446	4.4994	7.04514	7.88885	4.39819
XLOC_084430	MEGF10;PRRC1	chr7	27698658	27878750	3.61203	3.7775	7.05634	6.46845	5.46563	4.28501	5.31208	9.82203	7.38946
XLOC_084431	MEGF10;PRRC1	chr7	27698658	27878750	0.0	4.23416	22.1342	59.4846	41.5862	43.8092	57.7891	65.4313	59.4518

XLOC_084432	MEGF10;PRRC1	chr7	27698658	27878750	2.60008	2.33288	2.03378	16.3905	15.4499	14.3886	17.9192	26.8736	26.2709
XLOC_086415	MELK	chr8	61224187	61396117	15.8692	9.69806	11.3824	6.12	7.16035	8.12188	8.46192	4.30792	3.46585
XLOC_080074	MEPE	chr6	38292051	38299238	6.45517	15.4615	3.38977	8.6754	9.26275	10.8446	9.44916	16.7772	8.15604
XLOC_080832	MEPE	chr6	38287406	38288123	1.29273	2.31878	0.0	1.62139	2.41744	2.94315	1.97725	2.05806	1.24023
XLOC_080833	MEPE	chr6	38288396	38288727	4.16224	6.20649	0.0	4.09418	6.66449	7.62707	3.56301	8.01302	5.75323
XLOC_080834	MEPE	chr6	38289384	38289588	0.0	0.0	0.0	21.2485	14.0997	15.0478	8.62181	12.4357	11.7414
XLOC_080835	MEPE	chr6	38289920	38290779	6.18616	4.6246	2.41899	5.08163	5.79633	6.30538	6.05873	7.3072	4.05043
XLOC_080836	MEPE	chr6	38290977	38291188	0.0	11.0135	19.1923	4.46832	0.906494	7.37076	4.86859	5.60808	5.26685
XLOC_080837	MEPE	chr6	38292051	38299238	2.94593	0.0	0.0	0.263512	0.453147	0.0	0.0	0.0	0.0
XLOC_076236	METAP2	chr5	25222931	25265649	17.4229	17.1537	18.0169	16.2494	17.9049	16.3593	21.3693	19.1516	21.4087
XLOC_077433	METAP2	chr5	25222931	25265649	3.12101	2.79837	3.65921	1.9567	1.09198	2.41835	0.838284	2.01198	1.76763
XLOC_077434	METAP2	chr5	25222931	25265649	0.0	4.21497	5.51131	2.7366	2.54442	0.725531	3.73682	1.38642	1.02188
XLOC_077435	METAP2	chr5	25222931	25265649	2.56947	2.30163	0.0	0.919609	0.594571	1.58299	0.45179	2.01406	1.56154
XLOC_077436	METAP2	chr5	25222931	25265649	0.0	2.31433	1.5131	0.346759	1.20096	0.399227	1.72201	0.955955	2.19063
XLOC_077437	METAP2	chr5	25265955	25266988	0.0	1.48247	1.29241	0.148091	0.646102	0.428694	0.149937	0.248011	1.01043
XLOC_077438	METAP2	chr5	25269701	25270569	1.01796	0.913214	1.59225	0.273672	0.715438	0.105526	0.184105	0.406523	1.3331
XLOC_077439	METAP2	chr5	25270620	25271027	0.0	0.865282	4.52538	0.777901	0.0	0.0	0.506749	0.282929	0.75415
XLOC_057905	METTL11B	chr16	38562419	38592826	35.766	29.0967	26.0323	60.5495	64.8913	63.6992	74.4904	78.4457	90.4237
XLOC_058132	METTL11B	chr16	38562419	38592826	3.31873	3.09712	4.82074	5.57097	5.8271	5.1317	7.08441	7.00538	8.42906
XLOC_058954	METTL11B	chr16	38562419	38592826	5.08875	6.06816	7.933	15.4806	17.7458	16.5123	25.3544	23.7868	30.7078
XLOC_058955	METTL11B	chr16	38562419	38592826	0.0	5.71657	4.98278	6.85284	8.82331	7.18624	10.5507	13.9753	14.3831
XLOC_058956	METTL11B	chr16	38562419	38592826	2.85039	1.70404	3.3424	5.10654	8.87446	6.63159	8.05871	6.08798	7.70303
XLOC_058957	METTL11B	chr16	38562419	38592826	3.05787	6.38858	0.0	5.4701	11.2793	9.3929	12.2612	8.63946	15.1073
XLOC_058958	METTL11B	chr16	38562419	38592826	3.45455	1.47651	1.15852	2.52222	2.70909	2.92559	3.33468	3.22329	5.74138
XLOC_055469	METTL15	chr15	59648280	59655790	40.5454	26.3841	40.213	9.90928	7.40855	7.93798	7.30081	9.30853	6.44507
XLOC_056682	METTL15	chr15	59646387	59647023	0.0	0.451794	1.18156	1.2185	0.70544	1.24973	0.135455	0.600018	0.131732
XLOC_056683	METTL15	chr15	59647197	59648007	1.10846	1.32574	1.73363	1.48986	0.951466	0.689051	0.200137	0.663257	0.580453
XLOC_056684	METTL15	chr15	59655929	59656675	38.0995	32.3347	44.2046	7.70791	5.55707	7.76145	8.19226	8.9341	7.29075
XLOC_055468	METTL15;KIF18A	chr15	59331738	59601749	43.9279	40.891	38.055	50.9042	53.455	50.0443	59.5895	55.5873	66.8337
XLOC_055780	METTL15;KIF18A	chr15	59331738	59601749	16.5274	20.1297	19.0728	29.6286	22.6587	27.9409	31.479	34.9365	25.3328
XLOC_056680	METTL15;KIF18A	chr15	59331738	59601749	0.0	0.73343	0.0	0.439912	0.379223	0.0	0.649118	0.481941	0.213608

XLOC_056681	METTL15;KIF18A	chr15	59331738	59601749	0.578943	1.21213	2.71736	0.41524	0.453606	0.300835	0.633245	0.406706	0.506222
XLOC_064210	METTL16	chr19	23972886	24030618	1.95203	5.0231	2.44424	0.385109	0.127992	0.212157	0.298352	0.746563	0.649273
XLOC_041924	METTL21D	chr10	43022498	43127911	5.88639	9.67691	1.15035	0.131813	0.1145	0.152122	0.131968	0.292215	0.513063
XLOC_065534	METTL5;SSB	chr2	26581105	26604208	4.21749	3.48181	4.7889	21.252	20.9034	19.8738	18.7688	21.8378	18.214
XLOC_066200	METTL5;SSB	chr2	26581105	26604208	1.17877	0.444235	2.57255	2.2809	1.8098	2.18876	1.86193	2.49913	2.45656
XLOC_065513	METTL8	chr2	25083130	25085713	0.309665	0.277993	0.242363	0.0	0.0	0.0322429	0.0	0.0	0.0
XLOC_067085	METTL8	chr2	25081827	25082657	0.0	0.321589	0.0	0.0	0.0	0.0	0.0	0.0	0.0
XLOC_083687	MFAP3;FAM114A2	chr7	67231040	67372375	33.2102	33.0895	35.3267	2.04771	2.46498	2.4839	2.77805	2.0164	1.95154
XLOC_085009	MFAP3;FAM114A2	chr7	67231040	67372375	5.53381	5.62579	8.65491	0.0	0.0863657	0.573336	0.499602	0.110376	0.579569
XLOC_085010	MFAP3;FAM114A2	chr7	67231040	67372375	6.19499	8.0274	11.3047	0.7402	0.886805	0.85618	0.56006	0.103063	0.540826
XLOC_085011	MFAP3;FAM114A2	chr7	67231040	67372375	11.9837	5.11584	16.0549	0.919829	0.797674	1.06023	0.917119	0.508427	0.745428
XLOC_085012	MFAP3;FAM114A2	chr7	67231040	67372375	10.3514	7.72685	20.2061	0.463086	0.997897	0.53138	0.454895	0.253503	1.12329
XLOC_085013	MFAP3;FAM114A2	chr7	67231040	67372375	7.27859	7.61189	14.219	0.814648	0.42356	0.563085	0.324309	0.539702	0.158402
XLOC_085014	MFAP3;FAM114A2	chr7	67231040	67372375	9.96511	6.45236	10.3843	1.18989	1.03224	0.342968	0.593717	0.822592	1.01263
XLOC_085015	MFAP3;FAM114A2	chr7	67231040	67372375	3.83513	5.16265	6.50122	0.401122	0.300401	0.332084	0.349252	0.384675	0.223527
XLOC_085016	MFAP3;FAM114A2	chr7	67231040	67372375	3.91581	2.34245	3.67587	0.0701997	0.061273	0.162608	0.0	0.0	0.0684328
XLOC_085017	MFAP3;FAM114A2	chr7	67231040	67372375	1.26116	3.77039	3.9443	0.112989	0.0982925	0.261078	0.0	0.125537	0.220017
XLOC_085018	MFAP3;FAM114A2	chr7	67231040	67372375	3.14135	2.81657	3.68301	0.42202	0.0	0.324527	0.140611	0.0	0.0
XLOC_085019	MFAP3;FAM114A2	chr7	67231040	67372375	3.80285	1.13801	2.12604	0.194889	0.149379	0.113158	0.0996282	0.0547388	0.166458
XLOC_076887	MFNG	chr5	76210964	76305127	36.7159	44.5135	56.3531	10.3188	11.3394	11.6775	12.2424	12.1315	14.9775
XLOC_078155	MFNG	chr5	76210964	76305127	59.1858	66.7975	62.3493	60.4082	52.7861	59.8849	65.1423	70.9489	60.4037
XLOC_078156	MFNG	chr5	76210964	76305127	3.45096	8.23803	8.0771	2.77779	2.90865	3.87829	4.48217	1.00429	3.28605
XLOC_078157	MFNG	chr5	76210964	76305127	4.18623	6.24318	0.0	2.24649	2.87219	0.852224	1.79108	2.82015	4.33943
XLOC_070103	MFSD2A	chr3	106684945	106735882	18.0054	10.6541	11.557	8.30792	7.83399	7.25163	6.61704	6.49029	8.29039
XLOC_072025	MFSD2A	chr3	106684945	106735882	1.90638	0.85556	0.745893	0.55554	1.08403	0.941873	0.435328	0.814297	0.500307

XLOC_072026	MFSD2A	chr3	106684945	106735882	0.0	0.335547	0.0	0.301667	0.875631	0.46503	0.303856	0.447585	0.48971
XLOC_072027	MFSD2A	chr3	106684945	106735882	1.76632	1.5834	5.52122	0.632653	0.959784	1.82254	1.10283	1.22319	1.07655
XLOC_072028	MFSD2A	chr3	106684945	106735882	1.40308	0.419408	2.19373	1.00548	1.74768	0.725501	0.503927	0.0	0.733967
XLOC_072029	MFSD2A	chr3	106684945	106735882	1.25399	0.937824	0.981124	0.618319	0.933231	0.91217	0.0571105	0.377395	0.877103
XLOC_072030	MFSD2A	chr3	106736198	106736720	0.0	4.74274	10.8527	3.19774	2.76784	1.84034	2.11561	1.56639	2.9349
XLOC_059694	MFSD8;C17H4orf29;PLK4	chr17	30060806	30211173	127.079	121.812	69.1427	34.7013	36.3078	38.1442	37.2738	48.1741	23.7562
XLOC_061084	MFSD8;C17H4orf29;PLK4	chr17	30060806	30211173	6.67935	1.99308	2.60589	1.19467	0.256105	1.70707	0.0	0.972992	0.0
XLOC_061085	MFSD8;C17H4orf29;PLK4	chr17	30060806	30211173	0.0	1.44694	11.3499	0.8683	0.0	0.492583	0.0	0.0	1.25576
XLOC_061086	MFSD8;C17H4orf29;PLK4	chr17	30060806	30211173	0.0	4.91496	5.14088	0.0	0.252699	0.336844	0.0	0.0	0.0
XLOC_061087	MFSD8;C17H4orf29;PLK4	chr17	30060806	30211173	6.43647	3.84719	20.1227	0.864663	1.50074	3.4901	1.00757	0.637976	2.10273
XLOC_061088	MFSD8;C17H4orf29;PLK4	chr17	30060806	30211173	3.26816	5.47476	9.20496	0.703164	0.972652	1.08658	0.833098	0.262198	1.19997
XLOC_061089	MFSD8;C17H4orf29;PLK4	chr17	30060806	30211173	6.63107	3.3995	8.89096	0.679179	0.4441	0.6877	0.343096	0.757259	0.496351
XLOC_061090	MFSD8;C17H4orf29;PLK4	chr17	30060806	30211173	10.4021	5.17273	10.8208	0.930224	0.796957	1.77113	0.600313	0.0	0.900433
XLOC_061091	MFSD8;C17H4orf29;PLK4	chr17	30060806	30211173	6.83459	4.76292	5.33817	0.611677	0.70449	0.234332	0.20133	0.223991	0.791962
XLOC_043937	MGAT4A	chr11	3894102	3897712	4.86765	9.46323	8.37694	1.66276	2.31454	1.27143	1.33085	1.84488	1.54108
XLOC_044624	MGAT4A	chr11	3906917	3914542	2.50813	2.10162	3.92628	1.03475	0.571521	1.01886	0.828234	0.758346	0.636804
XLOC_044625	MGAT4A	chr11	3917135	3932063	12.9393	11.1457	14.504	11.4585	13.5951	11.3725	11.359	7.86389	13.3687
XLOC_045306	MGAT4A	chr11	3897829	3898589	1.20046	7.53713	5.632	1.07557	0.468033	0.745813	0.865523	1.79374	1.1521
XLOC_045307	MGAT4A	chr11	3900021	3900313	16.0424	12.7528	25.0075	0.478601	1.21441	0.0	1.80002	3.56263	3.22536
XLOC_045308	MGAT4A	chr11	3900519	3900719	14.6028	56.9437	11.4496	2.6782	0.0	1.46117	0.0	0.0	1.25485
XLOC_066315	MGAT5	chr2	63181362	63184204	0.0	0.0	0.0	0.133743	0.0	0.0	0.0	0.0	0.0
XLOC_067606	MGAT5	chr2	63148384	63149212	0.0	0.0	0.0	0.0966606	0.0	0.111779	0.0	0.107614	0.0

XLOC_067608	MGAT5	chr2	63158090	63160567	0.0	0.0	0.0	0.0	0.0489754	0.0	0.0	0.0	0.0
XLOC_067613	MGAT5	chr2	63173556	63175410	0.0	0.0	0.0	0.0	0.0334496	0.0	0.0	0.0857445	0.0
XLOC_067614	MGAT5	chr2	63177234	63177850	0.0	0.0	0.0	0.0	0.0	0.0	0.282388	0.0	0.0
XLOC_067615	MGAT5	chr2	63177920	63178895	0.0	0.0	0.692013	0.0	0.0	0.0	0.0802007	0.0	0.0
XLOC_067616	MGAT5	chr2	63179133	63179988	0.0	0.0	0.0	0.0929226	0.0809625	0.0	0.0	0.0	0.0
XLOC_078761	MGC127055;MGC127055	chr5	113609045	113610927	0.0	0.752901	0.328198	1.01537	1.02016	0.960019	1.15044	1.72937	1.98151
XLOC_078762	MGC127055;MGC127055	chr5	113611167	113612453	2.56355	0.0	0.0	1.0342	1.55618	1.39845	1.9259	1.67132	2.68944
XLOC_078763	MGC127055;MGC127055	chr5	113612627	113612966	3.98024	0.0	0.0	2.84731	5.16547	6.48885	10.5925	10.7481	8.25655
XLOC_069940	MGC137454	chr3	79860972	79942334	12.701	10.4354	7.89474	2.38362	0.573795	1.81898	1.38507	1.20213	1.71665
XLOC_070323	MGC137454	chr3	79812374	79843221	0.937189	1.02211	2.93022	1.32746	1.87721	1.43441	0.603777	1.07704	0.753812
XLOC_069939	MGC137454;PDE4B	chr3	79628996	79632036	3.99399	3.58425	3.85847	7.28034	6.18685	5.87829	5.57419	5.87367	5.26058
XLOC_070322	MGC137454;PDE4B	chr3	79603557	79628987	18.8288	15.7261	14.517	11.3667	12.6276	11.3553	10.5887	11.5233	14.2195
XLOC_071475	MGC137454;PDE4B	chr3	79632179	79633107	2.81606	2.8072	3.67094	4.12221	4.25425	2.92066	3.23032	3.18945	3.52497
XLOC_062459	MGC139164	chr18	61196326	61211620	6.28661	7.5871	6.05872	4.55673	4.90992	4.85163	4.94263	4.53008	4.59794
XLOC_079807	MGC152010	chr6	86404017	86433228	12.0627	8.90649	12.3003	12.6217	15.5927	13.7255	12.3743	9.92157	14.1893
XLOC_079808	MGC152010	chr6	86438261	86470517	11.1244	12.0062	15.7508	9.27109	8.17155	7.92097	4.59281	5.23754	7.39789
XLOC_081525	MGC152010	chr6	86404017	86433228	0.0	5.05155	6.60449	3.02958	4.841	3.01676	0.724215	1.22199	3.65786
XLOC_050461	MGC152301	chr13	20937660	21023558	1.15076	0.0	0.0	15.9405	7.47002	14.4086	5.8935	11.9729	5.19749
XLOC_051274	MGC152301	chr13	20937660	21023558	0.0	0.0	0.0	3.95836	2.45696	1.6398	0.690497	1.5517	1.04336
XLOC_078759	MGC152344	chr5	113596808	113597285	0.0	0.0	0.0	0.202619	0.350079	1.86308	0.800485	0.890509	1.96761
XLOC_078760	MGC152344	chr5	113607229	113608336	0.0	0.0	0.0	0.61451	0.596054	0.553616	0.968913	0.762872	0.599076
XLOC_067104	MGC152346	chr2	25706776	25708144	2.98736	1.25116	1.40243	2.9461	3.93246	3.04281	2.39585	2.69816	2.24643
XLOC_062460	MGC157082	chr18	61297815	61310738	1.35573	0.405271	1.47223	5.67945	3.12933	3.73617	3.89704	4.13003	3.04904
XLOC_062461	MGC157082	chr18	61310804	61400414	0.0	0.614895	0.0	3.56876	2.68947	4.41184	3.05133	4.59431	2.68867
XLOC_063312	MGC157082	chr18	61291238	61296779	0.134339	0.321653	0.210322	1.09653	0.919209	0.728153	0.827418	0.840651	0.776583
XLOC_063313	MGC157082	chr18	61296852	61297702	1.04443	0.624619	0.0	0.748743	0.326169	0.433004	1.79359	0.93815	0.273525
XLOC_063314	MGC157082	chr18	61297815	61310738	1.02664	0.306995	0.802901	1.288	0.480988	1.06419	0.371288	1.02486	0.448139
XLOC_063315	MGC157082	chr18	61310804	61400414	0.0	0.0	0.0	0.276556	0.240104	0.319038	0.0	0.153151	0.0
XLOC_063316	MGC157082	chr18	61310804	61400414	0.0	0.0	0.0	0.0	0.258059	0.514452	0.0	0.0	0.144662
XLOC_063317	MGC157082	chr18	61310804	61400414	0.0	0.0	0.0	0.349316	0.101264	0.134495	0.233841	0.0	0.113354

XLOC_063318	MGC157082	chr18	61310804	61400414	0.0	0.344079	0.0	0.309343	0.269309	0.0	0.103809	0.458823	0.401685
XLOC_063319	MGC157082	chr18	61310804	61400414	0.0	0.0	0.0	0.148064	0.0	0.341416	0.0	0.0	0.144004
XLOC_063320	MGC157082	chr18	61310804	61400414	1.40308	0.0	0.0	0.628431	0.327685	0.2902	0.629909	0.278847	0.244648
XLOC_063321	MGC157082	chr18	61310804	61400414	0.0	0.0	0.0	0.687902	0.0855868	0.227262	0.198056	0.437544	0.574317
XLOC_063322	MGC157082	chr18	61310804	61400414	1.45596	0.0	0.0	0.391545	0.398867	0.226803	0.264737	0.072941	0.0636237
XLOC_063323	MGC157082	chr18	61310804	61400414	0.0	0.0	2.4274	0.0	0.477852	0.0	0.0	0.0	0.269546
XLOC_063324	MGC157082	chr18	61310804	61400414	0.0	0.441654	0.0	0.39706	0.0	0.305475	0.132493	0.440087	0.128787
XLOC_063325	MGC157082	chr18	61310804	61400414	0.0	0.456558	0.0	0.547274	0.356399	0.0	0.684227	0.757801	0.665576
XLOC_063326	MGC157082	chr18	61310804	61400414	0.0	0.0	0.0	0.661018	0.288199	0.382512	0.0	0.184307	0.241554
XLOC_063327	MGC157082	chr18	61310804	61400414	0.0	0.0	0.0	0.0902137	0.235845	0.313074	0.0	0.0	0.0878876
XLOC_063328	MGC157082	chr18	61310804	61400414	1.13099	0.338166	0.884418	0.405369	0.352958	0.23432	0.612364	0.902076	0.394804
XLOC_063329	MGC157082	chr18	61310804	61400414	0.0	0.649785	0.0	0.389437	0.168313	0.447802	0.57781	0.642458	0.378255
XLOC_063330	MGC157082	chr18	61310804	61400414	0.0	0.452338	0.591523	0.745578	0.295859	0.392559	0.343543	0.227205	0.0
XLOC_078666	MICALL1	chr5	110223393	110224456	4.79239	4.30017	3.74888	3.5797	3.49906	4.47726	6.59945	4.15782	4.39672
XLOC_078667	MICALL1	chr5	110224578	110227223	2.32594	1.47906	2.50313	2.21633	2.39796	1.72574	3.1446	2.31375	2.64689
XLOC_076462	MICALL1;POLR2F;C5H22orf23	chr5	110228815	110255189	31.5408	31.2868	21.0931	28.9548	28.5483	28.7306	31.8256	32.7471	32.0841
XLOC_078668	MICALL1;POLR2F;C5H22orf23	chr5	110228815	110255189	0.0	0.0	2.13483	2.93595	2.52814	3.08615	2.39706	1.60458	3.08546
XLOC_078669	MICALL1;POLR2F;C5H22orf23	chr5	110228815	110255189	4.16302	3.10895	1.62608	5.59	4.83526	4.9307	4.43044	4.30799	4.52631
XLOC_092292	MID1	chrX	139800736	139811203	0.566199	0.0846877	0.221557	0.126918	0.133405	0.058931	0.0518723	0.142577	0.0247427
XLOC_092605	MID1	chrX	139821705	139871771	7.54096	7.47489	8.74699	2.05058	1.69269	1.59231	2.00089	0.964738	1.44469
XLOC_092606	MID1	chrX	139926926	139998553	0.575117	3.9645	7.90777	11.1451	10.9152	10.9707	12.7757	11.5929	13.2641
XLOC_093678	MID1	chrX	139816317	139817117	0.0	0.0	0.0	0.0	0.0	0.233234	0.0	0.0	0.0
XLOC_093679	MID1	chrX	139818836	139819002	0.0	9.37333	0.0	0.0	0.0	0.0	0.0	0.0	0.0
XLOC_093680	MID1	chrX	139819702	139821411	0.0	0.976288	0.364777	0.125393	0.036564	0.0	0.0	0.0468561	0.0407794
XLOC_093681	MID1	chrX	139926926	139998553	1.01227	0.908115	0.791704	5.17072	3.95258	3.98766	5.67591	4.14382	5.12592
XLOC_093682	MID1	chrX	139926926	139998553	0.0	2.64994	2.31013	6.48525	5.86307	5.34581	8.87703	7.3347	7.21208
XLOC_083604	MIER2	chr7	44605659	44608502	1.83684	1.51296	0.718346	4.67891	5.97762	4.3902	3.46458	4.11109	5.30927
XLOC_083605	MIER2	chr7	44611466	44638457	0.189423	0.850508	1.3569	5.23276	6.261	3.77393	3.83284	3.7837	6.03602
XLOC_073788	MIOS	chr4	15550427	15550942	0.0	0.0	0.0	1.99236	0.313463	0.416874	1.07776	0.199539	0.175997

XLOC_050850	MIR103-2;RNF24	chr13	51700847	51793711	8.25465	8.18259	5.55681	9.46531	8.78455	9.80866	7.32968	9.95731	5.02957
XLOC_053239	MIR124A-2	chr14	30715017	30818946	14.7805	20.2824	14.745	5.06863	7.20774	5.6879	5.64574	5.09868	5.52097
XLOC_054106	MIR124A-2	chr14	30715017	30818946	7.91718	1.1807	0.0	3.89377	0.604487	0.806845	0.340019	0.763716	1.02652
XLOC_054107	MIR124A-2	chr14	30715017	30818946	2.55802	3.05894	5.00003	1.26045	0.697683	0.926621	0.57554	0.763712	0.669347
XLOC_054108	MIR124A-2	chr14	30715017	30818946	5.07581	3.03366	1.32228	1.21213	2.10259	1.22265	1.66233	0.670136	1.03147
XLOC_054109	MIR124A-2	chr14	30715017	30818946	15.1613	0.0	0.0	1.80868	1.91602	2.05018	0.0	0.0	0.871394
XLOC_062450	MIR125A;MIR99B;MIRLET7E;ZNF613;ZNF432	chr18	58008622	58210431	12.8513	12.4101	9.1782	0.209792	0.0318355	0.19559	0.161414	0.0817532	0.0236251
XLOC_055425	MIR125B-1;MIR100;MIRLET7A-2	chr15	33083186	33594472	7.41955	5.85043	7.30271	0.27891	0.0348627	0.462371	0.731059	0.106529	0.466499
XLOC_055426	MIR125B-1;MIR100;MIRLET7A-2	chr15	33083186	33594472	12.5927	6.58628	6.15179	60.1981	38.0584	50.4102	42.5504	68.367	40.0474
XLOC_056294	MIR125B-1;MIR100;MIRLET7A-2	chr15	33083186	33594472	21.8087	18.8143	15.1389	81.9656	62.4978	76.9496	68.562	101.556	59.1461
XLOC_055486	MIR129-2	chr15	74529144	74587271	0.236539	0.58466	0.771153	4.54643	5.43651	4.13548	6.49652	4.32621	5.48854
XLOC_056934	MIR129-2	chr15	74529144	74587271	0.0	0.0	0.0	0.0562109	0.540285	0.390946	0.0	0.0	0.109642
XLOC_056935	MIR129-2	chr15	74529144	74587271	0.0	0.174136	0.455443	0.574054	0.866684	0.786576	1.11436	0.934813	1.2216
XLOC_064208	MIR132;DPH1;MIR212;OVCA2	chr19	23558664	23652787	0.0	0.0	0.0	0.0	0.0	0.0961152	0.0	0.0	0.0
XLOC_064774	MIR132;DPH1;MIR212;OVCA2	chr19	23558664	23652787	318.693	368.601	287.341	777.604	716.194	781.655	743.18	789.889	875.436
XLOC_050570	MIR133A-1;MIR1-1;GATA5	chr13	55209536	55318588	41.0362	47.0784	42.0481	89.0277	78.9615	84.6005	86.2107	90.2374	84.4565
XLOC_051882	MIR133A-1;MIR1-1;GATA5	chr13	55209536	55318588	4.05183	5.36717	2.77129	14.7951	11.1916	12.3987	14.9961	11.6649	16.7144
XLOC_078472	MIR141	chr5	103858181	103859251	0.792604	0.474134	0.0	0.497317	1.24014	1.39875	0.503813	0.634805	0.969575
XLOC_072802	MIR153-2	chr4	119538160	119629697	23.3226	26.1041	28.3548	13.9061	14.8974	15.4486	19.023	19.2999	12.5161
XLOC_075076	MIR153-2	chr4	119538160	119629697	2.16242	1.93772	5.06773	3.87104	2.84452	3.78386	4.97891	5.10968	2.82008
XLOC_075077	MIR153-2	chr4	119538160	119629697	13.8838	12.4193	10.8239	1.24612	3.65042	1.40001	2.29301	7.17735	5.37233
XLOC_073205	MIR1814C	chr4	63414944	63495142	8.40701	7.57845	7.87397	5.86462	7.13494	6.65826	4.42788	4.94385	4.6477

XLOC_074394	MIR1814C	chr4	63414944	63495142	4.77015	1.06964	3.73002	1.28219	1.11595	1.48186	0.537466	1.18805	1.76882
XLOC_074395	MIR1814C	chr4	63414944	63495142	3.00224	2.15553	4.69807	1.5073	1.36437	1.43535	0.656634	0.783321	0.840036
XLOC_063932	MIR193A	chr19	18814973	18892517	0.422007	0.549689	0.16516	0.10828	0.095275	0.0631905	0.0193677	0.0	0.0
XLOC_063988	MIR196A-1;HOXB7;HOXB9;MIR10A;HOXB3;HOXB1;HOXB4;HOXB2	chr19	38481179	38726999	7.40779	12.4155	11.5243	0.753275	0.823096	0.872623	0.825493	0.732947	0.780519
XLOC_065092	MIR196A-1;HOXB7;HOXB9;MIR10A;HOXB3;HOXB1;HOXB4;HOXB2	chr19	38481179	38726999	0.0	1.46631	0.0	0.0	0.746465	1.49717	1.24901	0.469931	0.848282
XLOC_083416	MIR199A-2;YIPF2;C7H19orf52;TMED1;DNM2	chr7	16494428	16614571	45.5936	44.1816	47.8439	24.7523	20.107	23.3679	21.0978	27.1951	16.2063
XLOC_044434	MIR199B;DNM1;GOLGA2;MIR3604-2	chr11	98863785	98885048	50.5516	30.3953	92.6965	17.1092	17.2891	33.7144	9.18989	21.2674	14.494
XLOC_047237	MIR199B;DNM1;GOLGA2;MIR3604-2	chr11	98863785	98885048	1.31625	0.786975	0.0	0.589591	0.410162	0.272389	0.0	0.261865	0.229582
XLOC_086865	MIR204;TRPM3	chr8	47179873	47413400	3.66712	7.71613	7.03969	3.93384	3.26244	3.49693	4.66968	4.8372	3.09083
XLOC_086866	MIR204;TRPM3	chr8	47179873	47413400	0.0	0.0	0.0	10.6407	3.76681	6.31204	5.1996	10.6256	10.2161
XLOC_087751	MIR204;TRPM3	chr8	47179873	47413400	0.726551	0.869257	1.13677	1.10714	0.568671	0.679048	0.462371	0.655141	0.380977
XLOC_087752	MIR204;TRPM3	chr8	47179873	47413400	0.0	0.0	0.0	0.385265	0.0	0.296472	0.0	0.0	0.0
XLOC_087753	MIR204;TRPM3	chr8	47179873	47413400	0.666308	0.797229	2.6064	1.13487	0.991368	0.692198	0.30322	0.601265	0.582442
XLOC_087754	MIR204;TRPM3	chr8	47179873	47413400	0.0	1.39172	7.27813	2.92247	3.54751	2.8447	2.37928	1.34115	2.81924
XLOC_087755	MIR204;TRPM3	chr8	47179873	47413400	0.959913	0.861145	0.750785	0.172046	0.149983	0.298617	0.173785	0.383595	0.0
XLOC_087756	MIR204;TRPM3	chr8	47179873	47413400	0.81497	0.73122	0.637517	0.0	0.0	0.169171	0.0	0.0	0.0
XLOC_087757	MIR204;TRPM3	chr8	47179873	47413400	0.943632	0.846552	0.0	0.169131	0.0	0.0978606	0.0	0.0942911	0.0

XLOC_041474	MIR208B;MYH7;NGDN;MYH7	chr10	21295982	21368886	29.8579	32.527	29.0576	58.4623	54.4439	56.1791	53.4432	53.9573	52.5623
XLOC_042437	MIR208B;MYH7;NGDN;MYH7	chr10	21295982	21368886	6.81703	2.03239	5.31342	5.5047	5.63568	8.25071	2.81819	6.41261	7.03445
XLOC_042438	MIR208B;MYH7;NGDN;MYH7	chr10	21295982	21368886	2.44034	1.64254	2.86397	2.02369	2.29428	2.2825	1.66759	1.89763	1.81362
XLOC_042439	MIR208B;MYH7;NGDN;MYH7	chr10	21295982	21368886	0.369121	0.993955	3.17737	1.72109	1.76737	1.88237	1.68903	1.74577	1.64729
XLOC_042440	MIR208B;MYH7;NGDN;MYH7	chr10	21295982	21368886	3.12962	0.936201	2.44857	1.73952	1.76516	2.01632	2.10947	1.50699	0.930302
XLOC_072629	MIR2284B	chr4	47353290	47415281	33.1874	24.003	26.5866	6.3701	5.88447	6.47007	6.2989	5.93468	5.48684
XLOC_074186	MIR2284B	chr4	47353290	47415281	9.09824	5.43706	18.4847	1.14051	1.41187	1.68926	3.56724	0.539705	1.90082
XLOC_074187	MIR2284B	chr4	47353290	47415281	5.9815	4.76987	5.45777	1.16142	0.778626	0.620143	0.901845	0.497777	0.783385
XLOC_049355	MIR2284S	chr12	53250666	53252075	0.0	0.0	0.0	0.0518117	0.0	0.0600711	0.0	0.0	0.0
XLOC_055443	MIR2285B;FAR1;SPON1	chr15	39198070	39684486	2.21143	2.56833	2.83584	6.36355	6.70638	6.74203	6.38281	3.77295	5.4264
XLOC_056431	MIR2285B;FAR1;SPON1	chr15	39198070	39684486	0.0	0.0	0.0	1.50724	5.16855	3.78982	4.38391	4.25408	2.62678
XLOC_038344	MIR2286	chr1	52165709	52232696	71.9084	68.3184	66.1998	34.5271	48.6326	41.2868	56.0139	35.2568	60.7949
XLOC_039274	MIR2286	chr1	52165709	52232696	8.2976	4.95425	0.0	0.247436	1.06508	1.13468	0.969282	0.811127	4.31939
XLOC_039275	MIR2286	chr1	52165709	52232696	3.23536	3.86195	2.52466	0.578704	0.993182	0.0	0.56233	0.314568	3.08289
XLOC_039276	MIR2286	chr1	52165709	52232696	1.20046	3.2302	5.632	1.39825	1.4977	1.49163	1.29828	0.358748	1.98999
XLOC_039277	MIR2286	chr1	52165709	52232696	2.81744	2.69723	3.08632	0.555727	1.67825	1.23015	1.28466	0.169717	1.33048
XLOC_039278	MIR2286	chr1	52165709	52232696	2.03105	2.63361	4.23888	1.00177	1.06318	0.951476	1.33299	0.408956	1.18508
XLOC_039279	MIR2286	chr1	52165709	52232696	5.36151	2.24445	3.35429	0.288264	1.0881	1.88905	0.871821	0.320953	1.21679
XLOC_039280	MIR2286	chr1	52165709	52232696	7.17035	5.89198	5.60318	1.60511	1.94764	0.554804	2.07758	1.59555	2.8091
XLOC_039281	MIR2286	chr1	52165709	52232696	21.5894	12.916	14.1661	7.55418	15.6488	10.4892	15.965	7.39934	17.4099
XLOC_039282	MIR2286	chr1	52165709	52232696	24.6477	15.8612	14.8142	10.5245	17.0542	16.8109	13.1597	7.49093	22.11
XLOC_039283	MIR2286	chr1	52165709	52232696	29.2773	25.1105	37.1121	11.4539	25.1999	15.6866	26.8428	10.2705	30.394
XLOC_039284	MIR2286	chr1	52165709	52232696	25.7584	16.9531	30.9036	10.9311	19.2002	15.5302	18.6845	8.38938	20.2725
XLOC_062668	MIR2326	chr18	11975846	11976201	0.0	0.0	0.0	0.0	0.279999	0.0	0.0	0.0	0.0
XLOC_063990	MIR2340	chr19	38837011	38971056	82.3765	116.9	86.9253	238.726	183.757	217.762	249.068	300.41	244.836
XLOC_065096	MIR2340	chr19	38837011	38971056	1.22694	2.2012	2.87803	0.110639	0.382603	0.254046	0.442172	0.366542	0.214142

XLOC_065097	MIR2340	chr19	38837011	38971056	3.38648	4.04267	5.28446	51.7887	41.0175	51.9162	58.1226	67.3839	46.621
XLOC_065098	MIR2340	chr19	38837011	38971056	2.93401	1.75226	4.58024	13.6489	10.3811	9.92051	7.68912	12.5951	13.4838
XLOC_066123	MIR2350;NAB1;BIN1	chr2	5297706	5714465	4.49737	3.83153	6.99238	6.8557	5.12207	5.46312	7.89246	6.31317	8.19135
XLOC_066795	MIR2350;NAB1;BIN1	chr2	5297706	5714465	11.9112	17.8202	27.9481	3.25109	8.81051	2.38648	3.7895	2.18162	5.11453
XLOC_066796	MIR2350;NAB1;BIN1	chr2	5297706	5714465	3.47213	6.64712	6.51936	1.49404	1.46798	2.16395	3.285	1.39212	2.42795
XLOC_066797	MIR2350;NAB1;BIN1	chr2	5297706	5714465	0.0	15.0663	7.87854	0.452171	0.0	0.0	0.0	0.0	0.0
XLOC_066798	MIR2350;NAB1;BIN1	chr2	5297706	5714465	7.84775	7.81048	4.08494	0.234052	0.201742	0.80558	0.0	0.512374	0.454156
XLOC_065779	MIR2356;TNS1	chr2	106693440	106867840	2.83694	4.73174	6.30238	0.453429	0.318052	0.766433	0.481884	0.436203	0.354621
XLOC_068254	MIR2356;TNS1	chr2	106693440	106867840	0.855143	1.53517	0.669197	0.536757	0.301933	0.444863	0.117271	0.128995	0.112228
XLOC_068255	MIR2356;TNS1	chr2	106693440	106867840	3.4579	1.72313	0.901312	0.103277	0.269734	0.23877	0.207942	0.0	0.100581
XLOC_068256	MIR2356;TNS1	chr2	106693440	106867840	3.33974	0.996538	0.0	0.597336	0.512211	0.341413	0.579439	0.0	0.0
XLOC_068257	MIR2356;TNS1	chr2	106693440	106867840	1.91467	1.43144	3.74375	0.0	0.0	0.0992771	0.0	0.191296	0.0835981
XLOC_068258	MIR2356;TNS1	chr2	106693440	106867840	5.39116	1.61089	4.21281	0.0	0.139458	0.0	0.0	0.355429	0.469339
XLOC_065839	MIR2357;AK2	chr2	121488766	121542785	5.02864	10.8425	8.16306	31.8725	33.8115	31.66	34.7829	32.9647	40.1499
XLOC_068427	MIR2357;AK2	chr2	121488766	121542785	5.85543	6.98194	0.0	8.39146	4.86356	4.14561	3.91703	7.76892	14.6196
XLOC_065906	MIR2358;PADI4;PADI1;RCC2	chr2	135744075	136013255	12.6379	10.0846	9.42385	17.1462	12.918	15.2371	14.4125	16.3619	14.2784
XLOC_068680	MIR2358;PADI4;PADI1;RCC2	chr2	135744075	136013255	4.7004	0.0	7.35092	0.315865	0.0	0.243391	0.423818	0.234186	0.102535
XLOC_082770	MIR23A;MIR24-2;MIR27A	chr7	12977514	12983072	2.41378	4.36467	2.29429	6.17569	4.57615	2.60501	5.1947	7.58378	4.79795
XLOC_059553	MIR2404-2	chr17	6106529	6234351	89.6866	116.18	87.2597	161.191	144.919	171.529	169.531	199.098	123.293
XLOC_059554	MIR2404-2	chr17	6106529	6234351	5.71915	3.1922	4.32128	1.2337	0.493905	0.705857	0.545414	0.632061	0.361023
XLOC_059946	MIR2404-2	chr17	6106529	6234351	0.686922	0.204629	0.0	0.196664	0.0655149	0.390001	0.0710031	0.0758013	0.0715856
XLOC_060428	MIR2404-2	chr17	6106529	6234351	2.81973	5.05957	2.94061	0.589711	0.660994	1.55972	0.851318	0.845349	0.410411
XLOC_060429	MIR2404-2	chr17	6106529	6234351	1.42801	1.70746	2.23274	0.51175	0.111147	0.44299	0.641035	0.0	0.746919

XLOC_060430	MIR2404-2	chr17	6106529	6234351	5.94916	10.641	0.0	1.06622	0.0	0.0	0.994507	0.0	1.02426
XLOC_060431	MIR2404-2	chr17	6106529	6234351	0.0	2.842	0.0	3.43953	0.708323	0.955464	0.769665	0.0	4.90323
XLOC_060432	MIR2404-2	chr17	6106529	6234351	11.8667	6.03154	5.56746	0.956986	0.370166	0.491559	1.06994	0.354687	1.03538
XLOC_060433	MIR2404-2	chr17	6106529	6234351	0.0	6.33994	0.0	0.712592	0.0	0.272447	0.932419	0.259808	0.0
XLOC_060434	MIR2404-2	chr17	6106529	6234351	3.6123	4.01416	3.23042	0.555262	0.202306	0.697617	0.659503	0.259197	0.315952
XLOC_060435	MIR2404-2	chr17	6106529	6234351	3.47449	4.84798	5.43389	0.622708	0.542036	0.119973	0.104644	0.115429	0.202124
XLOC_060436	MIR2404-2	chr17	6106529	6234351	0.0	0.92447	0.0	0.831213	0.237928	0.0	0.270259	0.0	0.536811
XLOC_060437	MIR2404-2	chr17	6106529	6234351	0.0	2.87725	2.50835	0.479088	0.250363	0.221606	0.386523	0.213344	0.186646
XLOC_060438	MIR2404-2	chr17	6106529	6234351	1.52433	4.55604	4.7661	0.955799	0.118552	0.157545	0.0	0.302492	0.929854
XLOC_076264	MIR2426	chr5	30949944	30960812	3.78227	2.54528	2.09364	2.53442	2.03706	1.74585	1.70559	1.98521	1.95586
XLOC_076787	MIR2426	chr5	30949944	30960812	8.74026	7.40456	4.76513	11.8671	11.9729	14.824	17.611	16.1992	9.37663
XLOC_077583	MIR2426	chr5	30949944	30960812	2.4812	4.69985	2.58778	1.26021	2.71671	1.54505	1.12588	1.07593	1.66188
XLOC_076473	MIR2439	chr5	112343089	112378410	5.76623	5.30653	7.61599	7.18277	7.34578	6.51694	7.0716	9.57661	6.82366
XLOC_080293	MIR2452;AFAP1	chr6	118780184	119108336	3.52593	7.64519	11.0318	0.0790046	0.0689108	0.0914549	0.159824	0.0881522	0.0769963
XLOC_082278	MIR2452;AFAP1	chr6	118780184	119108336	0.0	1.26304	2.06464	0.0	0.0	0.0	0.0481564	0.0	0.0
XLOC_084971	MIR2461;NDST1	chr7	63886276	63890010	24.0634	21.1809	22.1596	9.5399	7.45483	9.29142	9.11565	12.2966	6.69325
XLOC_083109	MIR2464	chr7	92739027	92838280	2.71782	4.25492	2.91739	3.49046	3.21096	3.14707	4.03287	3.62044	3.96052
XLOC_083756	MIR2464	chr7	92739027	92838280	3.40654	3.20881	1.87779	3.29633	2.42029	2.61445	1.54136	1.60235	1.53378
XLOC_086387	MIR2470	chr8	40862362	40917718	2.24986	5.45378	4.65836	2.0741	1.06092	1.53862	1.60762	1.60031	0.974701
XLOC_087653	MIR2470	chr8	40862362	40917718	0.0	1.28679	0.0	0.771262	0.134565	0.892902	0.390204	0.77469	0.300679
XLOC_086848	MIR2471	chr8	40550787	40634834	9.10204	13.2055	9.63865	51.7385	59.482	52.8122	72.3024	52.529	56.0775
XLOC_087648	MIR2471	chr8	40550787	40634834	10.0194	2.24658	3.91706	3.81507	6.05234	6.09317	6.42769	4.11446	4.69775
XLOC_089340	MIR2479	chr9	37489587	37524285	11.6197	14.3095	11.7335	29.2998	21.7644	27.4951	21.5474	28.5354	23.1003
XLOC_090576	MIR2479	chr9	37489587	37524285	5.2266	3.38827	6.81686	6.56128	7.82544	7.38628	6.84804	6.52572	6.17329
XLOC_090577	MIR2479	chr9	37489587	37524285	32.8895	7.84453	20.5091	13.5686	18.8072	22.5724	17.4506	21.6963	20.3699
XLOC_090578	MIR2479	chr9	37489587	37524285	7.79003	10.4561	12.1516	5.57267	10.7106	11.117	9.7101	8.2715	9.09187
XLOC_090579	MIR2479	chr9	37489587	37524285	0.0	2.1033	4.00077	5.09998	6.9593	5.37976	5.76257	3.0774	4.13525
XLOC_090580	MIR2479	chr9	37489587	37524285	4.3378	7.77409	11.8594	9.90068	11.5794	10.0457	9.98652	8.11395	12.0682
XLOC_090581	MIR2479	chr9	37489587	37524285	4.79621	4.54446	5.63019	6.88138	8.56329	9.15034	7.93354	5.83017	6.99468
XLOC_090582	MIR2479	chr9	37489587	37524285	9.39551	7.00315	7.32465	14.2863	13.5645	16.2213	14.361	12.1443	11.7539
XLOC_090583	MIR2479	chr9	37489587	37524285	1.48367	1.33039	3.47938	4.38548	5.42513	3.06717	5.18785	4.12398	5.43113

XLOC_090584	MIR2479	chr9	37489587	37524285	2.51858	4.01745	1.97012	7.60002	5.77748	7.14424	5.48409	4.956	6.38084
XLOC_090585	MIR2479	chr9	37489587	37524285	0.0	3.51251	0.0	4.27065	8.68786	9.4105	20.5634	6.45515	4.03301
XLOC_090586	MIR2479	chr9	37489587	37524285	6.55311	3.9168	5.12174	4.84168	5.72844	5.0753	4.687	2.4349	4.70934
XLOC_090587	MIR2479	chr9	37489587	37524285	3.20398	3.3514	6.26058	4.87809	4.9806	5.79133	5.30281	6.19324	5.86171
XLOC_090588	MIR2479	chr9	37489587	37524285	4.11522	6.13654	0.0	5.15187	10.3577	4.60936	4.93548	3.17054	7.46674
XLOC_090589	MIR2479	chr9	37489587	37524285	5.6289	4.48482	4.39831	5.20776	8.0034	8.12577	6.01283	4.2636	5.22558
XLOC_090590	MIR2479	chr9	37489587	37524285	8.01553	6.61468	6.46401	13.136	11.7574	13.4855	10.0053	8.59338	10.1227
XLOC_062458	MIR292;NLRP12	chr18	61130378	61177163	18.6189	21.3432	26.7221	16.4324	12.8098	13.3201	15.698	19.6798	11.9083
XLOC_063308	MIR292;NLRP12	chr18	61130378	61177163	0.0	8.11142	2.35677	2.43082	3.01746	2.47396	3.16065	2.35453	6.80575
XLOC_050880	MIR296	chr13	58033280	58113345	0.0	0.0	0.774341	0.0	0.0	0.513269	0.179141	0.197751	0.154934
XLOC_057952	MIR29D;CD46;CD46;CD46;CD46;CD46;CD46;MIR29E	chr16	77500528	77594739	27.3864	15.1659	11.4204	0.0	0.0	0.0	0.0	0.0	0.0
XLOC_059304	MIR29D;CD46;CD46;CD46;CD46;CD46;CD46;MIR29E	chr16	77500528	77594739	8.08144	10.4552	8.41253	0.0	0.0	0.0	0.0	0.0	0.0
XLOC_059305	MIR29D;CD46;CD46;CD46;CD46;CD46;CD46;MIR29E	chr16	77500528	77594739	5.13201	4.8245	4.01484	0.0	0.0	0.0	0.0	0.0	0.0
XLOC_059306	MIR29D;CD46;CD46;CD46;CD46;CD46;CD46;MIR29E	chr16	77500528	77594739	5.15911	2.8937	7.06371	0.0	0.0	0.0	0.0	0.0	0.0
XLOC_059307	MIR29D;CD46;CD46;CD46;CD46;CD46;CD46;MIR29E	chr16	77500528	77594739	5.82814	5.92468	2.73441	0.0	0.0	0.120722	0.0	0.0	0.0
XLOC_059308	MIR29D;CD46;CD46;CD46;CD46;CD46;CD46;MIR29E	chr16	77500528	77594739	18.436	15.1164	7.18643	0.0	0.0	0.0	0.0	0.0	0.0

XLOC_059309	MIR29D;CD46;CD46;CD46;CD46;CD46;CD46;MIR29E	chr16	77500528	77594739	11.8758	11.807	12.3493	0.0	0.0	0.0	0.0	0.0	0.0
XLOC_059310	MIR29D;CD46;CD46;CD46;CD46;CD46;CD46;MIR29E	chr16	77500528	77594739	21.8286	3.55706	3.10078	0.0	0.0	0.0	0.0	0.0	0.0
XLOC_059311	MIR29D;CD46;CD46;CD46;CD46;CD46;CD46;MIR29E	chr16	77500528	77594739	3.83702	2.2942	10.0001	0.0	0.0	0.0	0.0	0.0	0.0
XLOC_059312	MIR29D;CD46;CD46;CD46;CD46;CD46;CD46;MIR29E	chr16	77500528	77594739	3.97044	4.35209	6.20837	0.0	0.0	0.0	0.0	0.0	0.0
XLOC_059313	MIR29D;CD46;CD46;CD46;CD46;CD46;CD46;MIR29E	chr16	77500528	77594739	4.00504	4.7922	8.87798	0.0	0.0	0.0693471	0.0	0.0	0.0
XLOC_059314	MIR29D;CD46;CD46;CD46;CD46;CD46;CD46;MIR29E	chr16	77500528	77594739	8.10947	4.24376	4.75669	0.0	0.0	0.0	0.0	0.0	0.0
XLOC_059315	MIR29D;CD46;CD46;CD46;CD46;CD46;CD46;MIR29E	chr16	77500528	77594739	2.75275	4.11436	8.60819	0.0	0.0	0.0	0.0	0.0	0.0
XLOC_059316	MIR29D;CD46;CD46;CD46;CD46;CD46;CD46;MIR29E	chr16	77500528	77594739	2.80152	4.46835	0.730402	0.0	0.0	0.0	0.0	0.0	0.0
XLOC_053187	MIR30D;MIR30B	chr14	8080307	8090778	7.85764	8.41302	8.342	40.8885	46.5157	41.0488	44.9696	46.2449	59.1127
XLOC_086340	MIR31	chr8	22476757	22570535	9.61751	9.9253	8.04765	29.9541	32.2824	29.0837	31.9568	24.5926	24.5276
XLOC_086341	MIR31	chr8	22476757	22570535	3.26178	4.38656	1.27466	8.90948	8.11085	10.4421	10.6454	9.05011	5.96683

XLOC_086342	MIR31	chr8	22476757	22570535	0.0	2.55538	0.0	2.68203	1.30567	2.61557	0.732161	0.823816	0.0
XLOC_087433	MIR31	chr8	22476757	22570535	5.41181	3.69988	4.83833	8.73179	8.52936	9.31085	8.0061	7.04505	6.95841
XLOC_087434	MIR31	chr8	22476757	22570535	8.93725	6.10709	8.98419	9.72263	9.3525	8.7212	11.6054	6.86146	7.12715
XLOC_083422	MIR339B;ADAMTS10;MYO1F;ACTL9	chr7	18376202	18510465	0.0	8.84821	0.0	0.0	16.8539	11.6761	15.4796	0.0	0.0
XLOC_063931	MIR365-2	chr19	18730738	18813563	0.0	0.378862	0.3303	0.0	0.0	0.0	0.0	0.0468783	0.0
XLOC_092072	MIR424;MIR450;MIR3601;MIR542;MIR450-1;PLAC1	chrX	18162405	18279564	13.9529	12.4563	9.74539	18.8593	19.8256	22.4951	21.3745	22.1029	21.1108
XLOC_072721	MIR490	chr4	101419138	101465971	6.89671	10.161	6.37055	28.5019	29.0969	25.6666	31.6574	15.258	32.8237
XLOC_074762	MIR490	chr4	101419138	101465971	0.0	0.0	0.0	3.23879	3.07134	1.6398	5.52373	1.16378	3.82587
XLOC_074763	MIR490	chr4	101419138	101465971	0.0	1.81475	0.0	1.49557	1.29872	0.941195	0.408024	0.602492	1.32291
XLOC_074764	MIR490	chr4	101419138	101465971	0.0	0.0	2.2184	0.508532	1.09382	1.16535	0.497298	0.277563	2.71163
XLOC_074765	MIR490	chr4	101419138	101465971	0.0	0.0	0.827543	1.32757	0.578286	1.20634	1.14742	0.422376	1.01614
XLOC_053682	MIR599;MIR875	chr14	66985006	67009125	23.6015	27.9322	25.2512	26.3153	34.7326	29.8003	27.6778	23.2196	42.0755
XLOC_054712	MIR599;MIR875	chr14	66985006	67009125	0.998292	1.79117	0.0	0.805167	1.32548	0.724492	0.903065	0.598146	1.3946
XLOC_054713	MIR599;MIR875	chr14	66985006	67009125	9.90317	2.95238	11.5792	0.885954	1.50271	1.50708	3.3516	1.89183	4.26973
XLOC_054714	MIR599;MIR875	chr14	66985006	67009125	5.53711	1.24139	2.16438	0.620017	1.29337	1.00221	1.11903	1.37585	0.844853
XLOC_086998	MIRLET7A-1	chr8	86841344	86885020	28.6549	25.66	23.9325	60.1423	55.5648	56.1982	62.0539	64.9852	44.2672
XLOC_078832	MIRLET7B;MIR3596	chr5	117120138	117122349	3.52192	2.9508	3.58325	1.3265	2.29501	0.98985	0.612645	0.531768	1.23292
XLOC_045325	MITD1	chr11	4305185	4305703	0.0	5.99409	0.0	0.359239	0.155448	0.62017	0.178186	0.593757	0.0
XLOC_067913	MKI67IP	chr2	73810568	73811512	0.0	0.0	0.0	0.164834	0.718679	0.953862	0.166616	0.367681	1.36534
XLOC_067914	MKI67IP	chr2	73812292	73812870	1.72011	0.0	1.34426	0.308065	1.06856	1.06522	0.61422	0.510795	1.79748
XLOC_067915	MKI67IP	chr2	73813506	73814728	0.679422	1.01618	0.531549	0.426352	1.17042	0.776376	0.370952	0.272457	0.593904
XLOC_050427	MKKS;C13H20orf94	chr13	3538139	3611205	95.5912	77.7545	77.7603	255.952	236.158	250.909	271.992	294.507	277.851
XLOC_050724	MKKS;C13H20orf94	chr13	3538139	3611205	44.7559	57.6173	50.6572	25.6599	24.8536	27.5243	25.6373	27.3727	30.8367
XLOC_051083	MKKS;C13H20orf94	chr13	3538139	3611205	20.7446	12.559	13.4785	3.00238	5.69363	3.75591	4.73837	4.47839	3.89662

XLOC_051084	MKKS;C13H20orf94	chr13	3538139	3611205	11.7728	7.91747	16.1049	3.82376	2.97699	3.4988	4.75166	4.23922	5.77196
XLOC_051085	MKKS;C13H20orf94	chr13	3538139	3611205	6.59398	6.09721	9.3805	3.22506	3.71059	3.7382	3.60589	3.12899	3.61666
XLOC_072704	MKLN1	chr4	95557502	95995128	9.14707	7.2334	6.67671	9.49256	11.1674	10.1539	9.76389	9.38393	10.8256
XLOC_072705	MKLN1	chr4	95995782	96022439	28.819	30.9408	31.8392	34.2406	40.2376	39.9513	44.2131	30.1766	42.4579
XLOC_074624	MKLN1	chr4	95995782	96022439	6.43647	3.84719	2.51534	7.78197	8.12902	9.97173	8.92417	6.37976	6.58855
XLOC_070443	MKNK1	chr3	100138100	100162429	19.0687	24.3359	19.1185	38.7602	29.5217	37.8941	38.5852	47.6413	33.497
XLOC_086382	MLANA	chr8	39005331	39165808	19.0025	19.5345	18.1378	17.2018	21.6065	17.1391	14.6838	12.3039	14.5603
XLOC_076427	MLF2	chr5	104045729	104065382	18.8308	22.4032	14.8064	56.9056	56.8018	55.7853	57.1835	60.9124	60.0785
XLOC_072624	MLL5	chr4	46489282	46503486	11.8078	44.99	35.4932	70.0037	76.1308	75.9219	71.097	77.3263	72.8557
XLOC_073162	MLL5	chr4	46507326	46536144	1.82066	0.816742	0.712029	0.571114	0.355748	1.03874	0.164967	0.273015	0.397539
XLOC_073163	MLL5	chr4	46539087	46601882	1.20085	4.04375	2.78296	0.762181	1.6354	1.74814	0.647075	0.59993	1.47138
XLOC_074176	MLL5	chr4	46473763	46475544	0.890953	0.533148	0.348607	1.27824	1.39793	1.15861	1.42499	1.07495	1.09127
XLOC_074177	MLL5	chr4	46475711	46476204	2.15597	0.643986	0.0	1.54381	0.500502	0.665775	2.86422	1.486	1.1247
XLOC_074178	MLL5	chr4	46504397	46504900	0.0	1.25077	0.0	0.187402	0.32417	0.646752	0.37126	0.206295	0.364129
XLOC_074179	MLL5	chr4	46505392	46506716	0.620009	0.556427	0.970196	0.444678	0.631438	0.77318	0.0564814	0.497628	0.271044
XLOC_074180	MLL5	chr4	46537166	46539031	0.847121	1.90099	1.32586	1.02548	1.46233	0.83734	0.774538	0.937145	1.0006
XLOC_074181	MLL5	chr4	46539087	46601882	0.0	0.591223	0.0	0.0	0.306709	0.0	0.175836	0.0	0.0
XLOC_059847	MLXIP	chr17	55445499	55514467	0.123742	0.0474812	0.24838	0.0221989	0.0124744	0.0128993	0.043733	0.0374731	0.0277886
XLOC_062632	MLYCD	chr18	10262465	10262664	118.835	98.0506	58.2484	98.1485	123.412	135.25	116.552	117.144	125.105
XLOC_062633	MLYCD	chr18	10262972	10263283	9.39551	8.40378	10.9867	6.30276	7.49619	9.06487	11.1697	8.99576	7.29551
XLOC_060578	MMAA	chr17	12819099	12819945	1.05049	0.628244	3.28616	0.0	0.0	0.217751	0.0	0.0	0.0
XLOC_060579	MMAA	chr17	12820031	12821134	0.0	1.37279	1.7952	0.137135	0.0	0.158842	0.0	0.076607	0.0668448
XLOC_060286	MMAB;MVK	chr17	65864619	65885899	53.4707	75.1619	68.4526	61.1668	51.9648	52.0739	64.0419	85.8213	73.9103
XLOC_064123	MMD	chr19	5824203	5830151	58.4968	26.3387	40.7022	6.21985	6.06294	5.01937	11.0624	4.88647	11.9206
XLOC_064448	MMD	chr19	5822212	5822935	0.0	0.382367	2.00001	0.229172	0.299007	0.0	0.34531	0.127285	0.111558
XLOC_064449	MMD	chr19	5830265	5830402	96.6635	152.147	0.0	29.8872	27.4912	0.0	14.2759	0.0	51.4704
XLOC_064450	MMD	chr19	5830582	5830695	0.0	271.519	0.0	93.6667	229.761	0.0	60.1553	0.0	227.011
XLOC_064451	MMD	chr19	5831062	5833517	24.2386	9.04288	15.5213	2.9924	3.18908	1.60598	4.00878	1.99715	4.46363
XLOC_064452	MMD	chr19	5833636	5833774	183.053	115.062	150.723	18.8009	26.0498	9.33523	13.5283	7.84287	64.9101
XLOC_064453	MMD	chr19	5833902	5834128	119.95	56.7353	132.695	20.8002	11.1454	8.02383	17.6959	12.9223	21.4596
XLOC_064454	MMD	chr19	5834540	5838286	27.006	8.80593	16.2486	2.78371	2.62979	2.01587	4.68142	2.23568	4.14724
XLOC_064455	MMD	chr19	5838827	5839313	26.423	9.20753	25.7989	3.15327	3.06589	1.81273	3.5074	2.81696	5.74238

XLOC_064456	MMD	chr19	5839433	5840883	43.0865	18.7492	29.7727	7.07383	6.88574	4.88646	8.87964	5.50575	10.0806
XLOC_056059	MMP12	chr15	6014950	6015771	0.0	0.0	0.0	0.390717	0.595527	1.35541	0.196886	0.869876	0.761157
XLOC_053421	MMP16	chr14	77036819	77111488	2.71393	7.30474	2.83032	0.891857	1.27416	0.375618	0.245929	0.271326	0.6321
XLOC_053719	MMP16	chr14	77024359	77035875	8.21017	7.17345	6.05183	5.99622	3.89557	4.23429	3.50364	4.05171	4.27984
XLOC_062161	MMP2;LPCAT2	chr18	23803804	23894030	7.7111	5.84639	6.70673	5.80974	5.8069	6.46441	7.38284	8.00401	5.55251
XLOC_058155	MMP23B;CDC2L1;SSU72;ATAD3A;TMEM88B;MXRA8;MRPL20;VWA1;AURKAIP1;C16H1orf70;ANKRD65;CCNL2;DVL1;UBE2J2	chr16	52249685	52452929	4.82879	7.43736	14.9863	0.338971	0.298113	0.395328	0.269369	0.169992	0.112485
XLOC_056064	MMP3	chr15	6027900	6028133	0.0	0.0	0.0	4.97313	4.10533	2.76677	7.43249	0.851406	5.52054
XLOC_056065	MMP3	chr15	6028536	6029581	0.0	0.0	0.0	0.803495	0.764908	1.77629	1.40543	1.22345	1.70881
XLOC_056066	MMP3	chr15	6031184	6031509	0.0	0.0	0.0	3.85415	5.90927	4.38453	6.25772	4.55659	3.72187
XLOC_056067	MMP3	chr15	6031803	6032042	0.0	0.0	0.0	4.63747	7.68424	2.58653	9.76598	3.98952	10.3148
XLOC_056068	MMP3	chr15	6032276	6034260	0.0	0.0	0.0	1.38478	1.52274	1.56584	1.26778	0.876385	1.38593
XLOC_055377	MMP7	chr15	6386617	6440707	20.4719	5.69324	7.62282	0.545552	0.166435	0.40213	0.262298	0.276445	0.240576
XLOC_056074	MMP7	chr15	6386617	6440707	0.0	0.0	0.0	0.504344	0.425765	0.570083	0.0	0.0	0.0
XLOC_056075	MMP7	chr15	6386617	6440707	3.6763	2.1969	1.43633	0.0	0.0	0.0	0.16374	0.181696	0.0
XLOC_056076	MMP7	chr15	6386617	6440707	0.0	0.0	0.0	0.0	0.183503	0.0	0.0	0.0	0.0
XLOC_056077	MMP7	chr15	6386617	6440707	8.04137	2.39895	6.27137	0.0	0.0	1.6181	0.0	0.0	0.0
XLOC_056078	MMP7	chr15	6386617	6440707	4.47499	0.0	0.874852	0.0	0.0872935	0.1159	0.0	0.0	0.195273
XLOC_089398	MMS22L	chr9	53340441	53377957	0.24939	0.858411	0.780937	0.111841	0.0490279	0.0259837	0.0114498	0.0377485	0.065522
XLOC_089898	MMS22L	chr9	53213077	53248129	27.7449	56.8271	41.7495	35.257	32.5844	32.3988	40.9433	49.9967	38.9184
XLOC_042943	MNAT1	chr10	73202633	73202846	0.0	3.56401	0.0	8.66953	3.52413	5.96621	5.68425	6.54435	5.11449
XLOC_042944	MNAT1	chr10	73299331	73299879	1.85227	0.0	0.0	1.99014	0.0	0.0	0.494868	0.0	0.161222
XLOC_042945	MNAT1	chr10	73325023	73326453	1.13672	0.850166	1.77885	0.86627	0.0445444	0.118166	0.103652	0.228232	0.0
XLOC_041936	MNS1	chr10	53904703	54075757	7.35293	4.62463	8.11741	0.981856	0.724909	0.82568	0.649959	0.610613	1.08483
XLOC_063940	MNT	chr19	23892765	23945768	4.47587	2.82903	2.46033	0.922122	2.03401	0.825521	1.17826	0.74335	1.9475
XLOC_064209	MNT	chr19	23892765	23945768	0.0	0.0025122	0.181759	0.156579	0.410278	0.199726	0.162067	0.152701	0.329749

XLOC_064789	MNT	chr19	23892765	23945768	0.0	0.0	0.0	0.281958	0.244749	0.325227	0.140895	0.0	0.411445
XLOC_064790	MNT	chr19	23892765	23945768	0.0	0.0	0.0	0.133166	0.462651	0.153671	0.266576	0.0	0.259154
XLOC_064791	MNT	chr19	23892765	23945768	0.0	0.0	0.0	0.315865	0.45824	0.0	0.105955	0.234186	0.512667
XLOC_064792	MNT	chr19	23892765	23945768	0.0	0.0	0.750341	0.300921	0.413625	0.0	0.0875804	0.0481839	0.503279
XLOC_064793	MNT	chr19	23892765	23945768	0.0	0.0	0.0	0.479162	0.553763	0.552098	1.43146	0.0	0.931777
XLOC_064794	MNT	chr19	23892765	23945768	0.0	0.237327	0.0	0.284493	0.496597	0.329476	0.288204	0.0794368	0.485318
XLOC_064795	MNT	chr19	23892765	23945768	0.0	0.0	0.0	0.125442	0.545097	0.289643	0.251488	0.278312	0.610442
XLOC_064796	MNT	chr19	23892765	23945768	0.73914	0.0	0.0	0.265027	0.520644	0.307019	0.335925	0.44428	0.645964
XLOC_064797	MNT	chr19	23892765	23945768	0.0	0.0	0.0	0.225116	0.58241	0.25841	0.0	0.0	0.218464
XLOC_064798	MNT	chr19	23892765	23945768	0.0	0.0	1.35715	0.0	0.539339	0.179224	0.0	0.515598	1.36098
XLOC_064799	MNT	chr19	23892765	23945768	0.0	0.4311	0.0	0.0	0.7857	0.149115	0.258812	0.28649	0.754328
XLOC_064800	MNT	chr19	23945831	23950326	0.833677	1.39723	1.17465	0.299104	0.734168	0.364902	0.490188	0.185041	0.540282
XLOC_080210	MOB1B	chr6	87997969	88045261	20.9972	16.512	14.1383	25.7472	27.9806	26.5374	28.9998	31.9076	21.3025
XLOC_081562	MOB1B	chr6	87977843	87978990	0.730922	0.0	0.0	0.0	0.11442	0.15181	0.0	0.0732309	0.0
XLOC_081563	MOB1B	chr6	87979465	87981398	0.407338	2.31573	3.18775	3.65266	3.6761	3.77244	2.45882	4.42522	1.71082
XLOC_081564	MOB1B	chr6	87981574	87983426	0.85362	0.383113	0.334006	0.727166	0.6028	0.710524	0.507281	0.429226	0.485463
XLOC_081565	MOB1B	chr6	87984686	87985086	2.98233	3.56066	0.0	0.800282	1.60517	2.13842	0.260302	0.58161	0.258558
XLOC_081566	MOB1B	chr6	87985763	87986040	0.0	0.0	0.0	0.537264	1.35736	1.2125	0.0	0.56768	0.0
XLOC_081567	MOB1B	chr6	87986273	87986809	0.0	0.570952	0.0	0.171093	0.592668	0.788029	0.34001	0.377447	0.997793
XLOC_081568	MOB1B	chr6	87986986	87987649	0.0	0.0	2.23707	1.28168	0.556823	0.887672	0.770453	0.710657	0.374195
XLOC_081569	MOB1B	chr6	87988041	87989722	0.0	0.426096	0.371478	0.723616	0.484035	0.543153	0.520354	0.286274	0.124583
XLOC_081570	MOB1B	chr6	87990105	87991053	0.0	0.547248	0.715628	0.410001	0.286028	0.474531	0.497363	0.182921	0.0
XLOC_081571	MOB1B	chr6	87991134	87991752	1.57067	0.0	0.0	0.140673	0.244223	0.811318	0.281196	0.155757	0.136852
XLOC_081572	MOB1B	chr6	87991832	87992614	0.0	0.34628	0.0	0.41509	0.451697	0.239909	0.20892	0.346288	0.202124
XLOC_081573	MOB1B	chr6	87993502	87994167	0.0	0.0	1.11413	0.510654	0.110931	0.58947	0.127916	0.141581	0.124242
XLOC_055461	MOGAT2;MOGAT2;MOGAT2;MARK1	chr15	55638515	55906543	0.0	1.03046	0.442626	12.7593	7.69327	10.1394	7.71863	13.4091	9.88135
XLOC_055769	MOGAT2;MOGAT2;MOGAT2;MARK1	chr15	55638515	55906543	1.6234	3.3033	2.28699	4.4549	2.42216	3.88735	2.79565	4.25009	2.95548

XLOC_056636	MOGAT2;MOGAT2;MOGAT2;MARK1	chr15	55638515	55906543	0.0	0.0	0.0	3.53669	2.71711	4.06642	3.30289	3.88888	3.81672
XLOC_056637	MOGAT2;MOGAT2;MOGAT2;MARK1	chr15	55638515	55906543	4.41479	0.0	2.3007	2.37264	3.66399	3.04243	4.09102	4.67545	4.8741
XLOC_056638	MOGAT2;MOGAT2;MOGAT2;MARK1	chr15	55638515	55906543	1.01653	0.0	0.0	2.45959	1.58765	1.36992	1.28696	2.23277	2.04123
XLOC_056639	MOGAT2;MOGAT2;MOGAT2;MARK1	chr15	55638515	55906543	0.0	0.0	0.0	2.29889	2.61135	0.435928	4.02689	5.35481	4.4403
XLOC_056640	MOGAT2;MOGAT2;MOGAT2;MARK1	chr15	55638515	55906543	0.0	0.0	0.0	1.27163	1.326	1.46794	0.254854	1.12828	0.742543
XLOC_092272	MOSPD2	chrX	135795908	135891267	17.751	21.1557	15.974	32.1834	38.8758	35.1993	33.2619	29.4342	39.0225
XLOC_093610	MOSPD2	chrX	135795908	135891267	12.0956	4.46938	6.6796	7.39866	9.07893	7.38981	10.2883	6.70246	10.0122
XLOC_093611	MOSPD2	chrX	135891331	135891904	0.0	5.20189	0.0	0.779416	0.675773	0.718602	0.310689	0.172271	0.606327
XLOC_069859	MOV10	chr3	30793104	30809881	122.908	151.022	99.8719	46.2665	61.5151	49.4063	61.1101	57.3261	64.5138
XLOC_086368	MPDZ	chr8	31150615	31161072	22.3187	15.7028	19.2472	15.3718	20.0449	17.8895	23.1141	16.8114	16.579
XLOC_086369	MPDZ	chr8	31187038	31261882	5.98315	9.22119	5.92055	3.39176	3.89299	3.26734	3.22061	3.23515	3.60792
XLOC_086834	MPDZ	chr8	31150615	31161072	33.5552	35.4063	31.7861	68.0298	61.2888	65.6812	70.8118	74.7896	52.5813
XLOC_086835	MPDZ	chr8	31161758	31169592	59.4051	61.3699	50.1669	132.31	122.723	125.783	128.933	149.344	113.12
XLOC_087567	MPDZ	chr8	31150263	31150487	10.2529	24.5079	8.00788	22.2793	19.0397	12.342	16.4724	17.9664	14.0871
XLOC_087568	MPDZ	chr8	31150615	31161072	10.5973	4.43634	5.80128	3.22873	4.38452	4.9422	3.92598	2.22068	3.42272
XLOC_087569	MPDZ	chr8	31187038	31261882	4.00215	9.54927	9.36358	1.78936	3.66572	4.07753	1.71729	0.771762	2.07536
XLOC_087570	MPDZ	chr8	31263804	31264644	0.0	0.0	0.0	0.0	0.165454	0.219653	0.0	0.0	0.0925059
XLOC_055913	MPEG1	chr15	83606683	83725002	2.15981	1.70074	1.75584	0.096841	0.0678871	0.169662	0.0251115	0.130648	0.0378076
XLOC_064283	MPP2	chr19	44394331	44407704	4.67807	3.11632	4.37072	14.862	29.1571	23.0417	20.1088	14.0705	20.7369
XLOC_042028	MPP5	chr10	79540007	79555117	14.8238	18.1442	15.9676	23.6726	18.2745	18.1098	19.4103	23.0692	18.3585
XLOC_050809	MPP7	chr13	36725726	36734407	5.60867	4.19482	4.82737	0.0	0.0	0.116614	0.0511499	0.0563109	0.0980968
XLOC_055471	MPPED2	chr15	61985346	61990271	69.334	49.1183	55.5714	9.07325	4.48521	7.16227	4.10744	19.0321	2.80737
XLOC_055472	MPPED2	chr15	62032369	62222901	5.4938	0.623319	5.27929	20.3094	16.3345	18.7072	10.4141	14.7006	12.2211

XLOC_055473	MPPED2	chr15	62032369	62222901	0.0	0.292128	0.191016	0.83172	0.594476	0.762616	0.223884	0.639545	0.363233
XLOC_055782	MPPED2	chr15	62032369	62222901	0.75852	0.0	1.21191	2.61254	2.15531	2.55242	0.207776	0.687102	1.20834
XLOC_055783	MPPED2	chr15	62032369	62222901	0.0	0.0	1.29983	0.704295	0.757635	0.728279	0.639934	0.89368	0.245149
XLOC_056687	MPPED2	chr15	61990372	61992695	1.66988	0.799494	1.04553	0.0299504	0.104898	0.104307	0.0	0.0	0.0292308
XLOC_056688	MPPED2	chr15	62032369	62222901	2.20677	0.32992	0.862853	2.57062	1.29157	2.2864	1.39469	0.990393	0.963022
XLOC_056689	MPPED2	chr15	62032369	62222901	0.0	0.356546	1.86497	2.24382	1.39493	2.59325	0.752454	1.90089	0.832398
XLOC_056690	MPPED2	chr15	62032369	62222901	0.932636	0.278912	0.0	2.84189	2.69655	2.03136	0.591293	1.30493	0.9774
XLOC_056691	MPPED2	chr15	62032369	62222901	0.0	0.588175	0.512775	0.76383	0.923952	1.22571	0.119337	0.460084	0.40108
XLOC_056692	MPPED2	chr15	62032369	62222901	0.454434	0.135966	0.0	0.325982	0.463429	0.472737	0.332209	0.411165	0.159029
XLOC_056693	MPPED2	chr15	62032369	62222901	0.0	0.0	0.206179	0.708745	0.620884	0.685893	0.217416	0.530894	0.322863
XLOC_056694	MPPED2	chr15	62032369	62222901	0.0	0.0	0.0	1.21952	0.702318	0.233607	0.200737	0.446614	0.592141
XLOC_056695	MPPED2	chr15	62032369	62222901	0.0	0.0	0.0	1.68359	0.481893	1.92651	0.273118	0.0	0.271884
XLOC_056696	MPPED2	chr15	62032369	62222901	0.0	0.0	1.38403	0.475766	0.484133	0.917887	0.0802058	0.442361	0.386398
XLOC_056697	MPPED2	chr15	62032369	62222901	0.0	0.0	0.0	0.948517	0.41239	0.410808	0.119016	0.394919	0.115429
XLOC_056698	MPPED2	chr15	62032369	62222901	0.560867	0.0	0.438852	1.60914	1.05504	0.816298	0.971851	1.18253	0.637634
XLOC_056699	MPPED2	chr15	62032369	62222901	0.0	0.0	5.74532	1.48125	1.42601	2.27498	1.96489	1.6352	1.60003
XLOC_056700	MPPED2	chr15	62032369	62222901	0.708024	0.0	0.0	1.01551	0.665135	0.882453	0.708336	0.567654	0.309427
XLOC_056701	MPPED2	chr15	62032369	62222901	0.0	0.0	0.0	1.80107	1.72914	0.920181	1.77973	1.97954	1.36045
XLOC_056702	MPPED2	chr15	62032369	62222901	1.76201	0.0	0.0	0.788893	1.64133	1.27271	0.471538	0.174325	0.613681
XLOC_056703	MPPED2	chr15	62032369	62222901	0.0	0.875774	0.0	1.04978	1.12838	2.40497	1.7941	2.57627	1.27207
XLOC_056704	MPPED2	chr15	62032369	62222901	2.66184	0.0772267	0.201986	2.52743	2.62109	1.20954	2.14583	1.14698	1.81397
XLOC_056705	MPPED2	chr15	62032369	62222901	0.0	0.557382	0.0	0.946563	0.924447	0.709961	0.396044	0.249238	0.217212
XLOC_056706	MPPED2	chr15	62032369	62222901	1.03209	0.15439	0.807598	1.11046	1.29476	0.697609	0.423916	0.881268	0.631908
XLOC_056707	MPPED2	chr15	62032369	62222901	0.0	0.0	0.892785	2.3529	1.06881	1.65567	0.824063	0.569061	0.797065
XLOC_056708	MPPED2	chr15	62032369	62222901	0.0	0.272934	0.0	1.39049	1.21257	0.94668	0.496134	0.821089	0.478255
XLOC_056709	MPPED2	chr15	62032369	62222901	0.0	0.545075	0.0	0.96629	0.411868	0.223388	0.629482	0.269714	0.375813
XLOC_056710	MPPED2	chr15	62032369	62222901	0.983361	0.0	0.769091	0.793137	0.460874	0.815683	0.266947	0.392871	0.343462
XLOC_056711	MPPED2	chr15	62032369	62222901	0.0	0.228557	0.597768	0.684951	0.239173	0.555362	0.90252	0.459161	0.267104
XLOC_056712	MPPED2	chr15	62032369	62222901	0.0	0.419469	0.0	0.502837	0.329361	0.873935	0.0637817	0.140549	0.674149
XLOC_056713	MPPED2	chr15	62032369	62222901	0.0	0.0	0.918864	0.421153	0.733184	0.973565	0.211916	0.468372	0.205077
XLOC_056714	MPPED2	chr15	62032369	62222901	0.0	0.0	0.0	1.05237	0.68562	0.759077	0.526839	0.583265	0.51204
XLOC_056715	MPPED2	chr15	62032369	62222901	2.43322	0.0	0.0	0.654018	0.379416	0.251925	0.438462	0.484672	0.21229
XLOC_056716	MPPED2	chr15	62032369	62222901	1.46563	0.0	1.1457	0.52512	0.228082	0.303021	0.262904	0.291052	0.25551

XLOC_056717	MPPED2	chr15	62032369	62222901	0.0	0.0	0.378177	0.606666	0.644345	0.60319	0.30897	0.582816	0.295933
XLOC_056718	MPPED2	chr15	62032369	62222901	0.662008	0.0	0.517935	0.949558	0.414758	0.962894	0.180785	0.597444	0.347237
XLOC_056719	MPPED2	chr15	62032369	62222901	0.0	0.20962	0.365508	0.816688	0.495413	1.1918	0.321361	0.51781	0.367975
XLOC_056720	MPPED2	chr15	62032369	62222901	0.0	0.0	0.0	0.794302	0.462823	0.613908	0.430686	0.355671	0.413177
XLOC_056721	MPPED2	chr15	62032369	62222901	0.0	1.05465	0.0	0.948329	0.541394	0.0	2.75098	0.342551	0.611889
XLOC_056722	MPPED2	chr15	62032369	62222901	0.0	0.0	0.0	1.08101	0.157013	0.625282	0.0909283	0.501875	0.0877747
XLOC_056723	MPPED2	chr15	62032369	62222901	2.06151	0.205553	1.07521	1.54003	1.02228	1.499	0.250089	0.757728	0.540599
XLOC_056724	MPPED2	chr15	62032369	62222901	0.0	0.520317	0.0	0.935599	0.77236	1.26552	0.369978	0.34916	0.506968
XLOC_056725	MPPED2	chr15	62032369	62222901	0.0	0.0	0.0	0.932163	1.15663	1.22937	0.266584	0.295165	0.0
XLOC_056726	MPPED2	chr15	62032369	62222901	0.0	0.0	0.0	0.934311	0.484875	2.1497	0.370241	0.82286	0.544647
XLOC_056727	MPPED2	chr15	62032369	62222901	0.0	0.0	0.0	2.99005	1.24069	1.66963	2.03016	0.0	1.4264
XLOC_056728	MPPED2	chr15	62032369	62222901	0.0	0.0	0.0	5.35683	0.0	0.0	1.59014	0.912104	0.0
XLOC_056729	MPPED2	chr15	62032369	62222901	1.05818	0.0	0.0	0.758592	1.07389	1.97401	0.286861	0.527969	0.277122
XLOC_056730	MPPED2	chr15	62032369	62222901	0.0	0.101626	0.797401	1.401	1.06666	0.742464	0.808627	0.923163	0.535041
XLOC_056731	MPPED2	chr15	62032369	62222901	0.0	0.0	0.0	2.03788	2.08338	1.49203	1.46869	1.01997	0.720002
XLOC_056732	MPPED2	chr15	62032369	62222901	0.0	0.0	0.0	1.34282	0.574086	1.14886	0.32348	0.725866	0.974116
XLOC_056733	MPPED2	chr15	62032369	62222901	0.979659	0.0	0.0	0.834479	0.691453	1.17173	0.357848	0.541441	0.642713
XLOC_056734	MPPED2	chr15	62032369	62222901	0.202101	0.120966	0.158194	1.01509	1.1597	1.11603	0.686541	0.509525	0.637098
XLOC_056735	MPPED2	chr15	62032369	62222901	0.0	0.0	0.373192	1.32562	1.19695	1.0417	0.871228	0.81483	0.625789
XLOC_056736	MPPED2	chr15	62032369	62222901	0.973069	0.145566	0.761441	1.48324	1.44995	1.31568	0.66649	1.12453	0.723516
XLOC_056737	MPPED2	chr15	62032369	62222901	0.831537	0.746411	0.325369	1.11847	1.33764	1.64395	0.532282	0.961811	1.12774
XLOC_056738	MPPED2	chr15	62032369	62222901	0.0	0.0	0.0	1.26981	1.06191	2.13868	2.33281	0.664098	0.0
XLOC_056739	MPPED2	chr15	62032369	62222901	0.0	0.783872	0.0	1.17448	1.01209	1.07794	0.922351	0.257068	1.13947
XLOC_056740	MPPED2	chr15	62032369	62222901	0.0	0.0	0.0	0.781199	0.506437	0.673702	0.96582	0.859118	0.0
XLOC_056741	MPPED2	chr15	62032369	62222901	0.0	1.30107	0.0	1.55958	1.01619	1.80005	0.65083	1.00864	1.0118
XLOC_056742	MPPED2	chr15	62032369	62222901	0.0	0.179792	0.783745	1.86794	1.41672	1.54401	1.0664	0.949176	1.0346
XLOC_056743	MPPED2	chr15	62032369	62222901	2.91041	0.0	2.27176	1.3017	2.91072	2.08772	1.01738	0.568056	1.51433
XLOC_056744	MPPED2	chr15	62032369	62222901	1.10676	0.330929	0.0	0.991724	0.863657	1.37601	0.499599	0.883007	1.25574
XLOC_056745	MPPED2	chr15	62032369	62222901	0.0	0.0	0.0	2.24352	0.710318	1.5326	0.718834	1.24805	0.794567
XLOC_056746	MPPED2	chr15	62032369	62222901	0.0	0.0	0.0	1.17024	1.2102	1.61116	0.689382	1.02467	0.227096
XLOC_056747	MPPED2	chr15	62032369	62222901	0.0	0.0	0.0	1.48544	1.23476	1.14691	0.644978	1.10611	0.275823
XLOC_056748	MPPED2	chr15	62032369	62222901	0.0	0.0	0.0	1.03851	0.986987	1.63786	0.38084	0.525691	0.82773
XLOC_056749	MPPED2	chr15	62032369	62222901	0.564809	0.506914	0.441935	1.16469	1.37229	1.70275	0.824093	1.30419	0.938461

XLOC_056750	MPPED2	chr15	62032369	62222901	0.0	0.0	1.41127	1.4554	2.24231	2.60827	0.482927	0.178582	1.57224
XLOC_056751	MPPED2	chr15	62032369	62222901	0.0	0.145958	0.0	1.04981	1.22429	1.97882	0.62372	1.17657	1.28023
XLOC_056752	MPPED2	chr15	62032369	62222901	0.0	0.0	0.769091	1.41002	1.15218	1.73333	1.06777	1.08039	0.772779
XLOC_056753	MPPED2	chr15	62032369	62222901	0.770644	0.691511	0.60286	1.51973	0.783896	1.60021	0.840097	0.463038	0.673427
XLOC_056754	MPPED2	chr15	62032369	62222901	1.53403	0.0	0.0	0.961775	1.19298	1.90216	0.549648	0.760951	0.401044
XLOC_056755	MPPED2	chr15	62032369	62222901	0.0	0.0	0.0	1.39195	0.668251	0.72548	0.775793	0.621979	0.54278
XLOC_056756	MPPED2	chr15	62032369	62222901	0.0	0.798319	0.0	1.19613	1.23648	0.274386	0.469301	1.04667	0.696205
XLOC_056757	MPPED2	chr15	62032369	62222901	0.0	0.0	0.0	0.909756	0.677463	0.900026	0.65083	0.432276	0.0
XLOC_056758	MPPED2	chr15	62032369	62222901	2.12121	0.824451	1.41834	2.41593	1.68525	2.00761	0.896238	0.647169	1.11094
XLOC_056759	MPPED2	chr15	62032369	62222901	0.0	0.596233	0.0	1.25076	0.545038	1.34364	0.541112	0.39822	0.522265
XLOC_089481	MRAP2;KIAA1009	chr9	66152758	66272411	6.59099	9.25299	10.3149	0.777572	0.251158	0.369069	0.156298	0.355853	0.291297
XLOC_038039	MRAS	chr1	131797428	131820936	1.82197	1.33933	2.02143	2.72385	2.87365	2.31276	1.43049	1.12598	2.36759
XLOC_038040	MRAS	chr1	131830178	131848946	21.9736	22.518	18.4864	9.74095	9.22946	8.64684	10.2222	12.402	10.8292
XLOC_040240	MRAS	chr1	131769697	131770447	1.22072	0.0	0.0	1.42182	0.380684	0.126384	0.219951	0.121571	0.106496
XLOC_040241	MRAS	chr1	131827969	131829978	1.95309	5.14269	6.41959	3.43273	2.36067	3.1708	3.1091	2.86893	3.00794
XLOC_064039	MRC2	chr19	47731658	47736853	65.2316	75.3414	62.7262	68.072	63.6273	66.5811	66.4952	84.7575	67.7338
XLOC_064309	MRC2	chr19	47737066	47746736	1.85209	0.738737	0.966058	0.830215	1.06405	0.769892	0.843646	0.55746	1.07956
XLOC_065278	MRC2	chr19	47705798	47706656	1.0325	0.0	0.0	0.0	0.806186	0.0	0.560051	0.206127	0.360551
XLOC_065772	MREG	chr2	104559711	104567945	7.54744	9.00558	2.9435	156.575	83.3748	121.63	102.061	132.766	143.926
XLOC_066445	MREG	chr2	104436961	104494458	52.552	47.1768	43.6078	5.30907	3.82873	4.64754	4.78554	4.87771	4.26732
XLOC_038570	MRPL3;NUDT16	chr1	139810995	140101841	7.66522	0.654851	2.56899	0.0981225	0.0854583	0.567301	0.0	0.109222	0.0955751
XLOC_040529	MRPL3;NUDT16	chr1	139810995	140101841	42.0028	55.1346	45.6022	106.954	93.8934	99.7435	107.04	146.872	110.899
XLOC_040530	MRPL3;NUDT16	chr1	139810995	140101841	19.0071	15.1404	28.046	50.4737	32.2058	35.0091	33.5077	48.6852	24.4251
XLOC_076224	MRPL42	chr5	23434454	23502533	18.0598	16.2415	15.9783	8.55945	10.7649	10.879	8.90908	5.1464	8.9746
XLOC_076756	MRPL42	chr5	23422753	23434400	17.5978	9.66976	6.87412	7.64651	8.13842	7.46134	9.13284	6.57374	8.23804
XLOC_077382	MRPL42	chr5	23422753	23434400	0.0	21.4531	18.6964	6.98443	13.5737	9.70001	6.00638	2.83841	7.74344
XLOC_077383	MRPL42	chr5	23422753	23434400	0.0	8.32051	13.0549	1.9947	4.29245	3.14401	3.90553	3.54108	3.38518
XLOC_077384	MRPL42	chr5	23422753	23434400	0.0	10.4034	0.0	4.01399	2.26895	6.57414	3.79436	1.42806	1.28957
XLOC_077385	MRPL42	chr5	23422753	23434400	0.0	8.98373	1.95776	2.69206	1.16088	3.09034	4.4132	2.70415	1.08859
XLOC_077386	MRPL42	chr5	23422753	23434400	0.0	5.62849	2.94351	3.37447	2.30798	3.07924	4.55051	5.10638	2.28457
XLOC_077387	MRPL42	chr5	23422753	23434400	7.05646	2.53115	3.30984	3.28691	3.40569	2.33507	3.16751	2.80447	1.84556

XLOC_077388	MRPL42	chr5	23422753	23434400	6.07172	1.36106	2.37302	2.71914	3.89602	2.66672	2.99217	1.65685	3.30706
XLOC_077389	MRPL42	chr5	23422753	23434400	7.42286	6.5144	3.48507	4.2152	4.85085	4.73486	5.51263	4.9724	4.58834
XLOC_077390	MRPL42	chr5	23434454	23502533	1.34577	5.63167	2.10406	4.82189	5.03066	4.73314	5.32189	3.88046	4.92834
XLOC_077391	MRPL42	chr5	23434454	23502533	2.91729	2.47295	4.56557	2.52853	2.85983	3.03414	3.46342	1.41695	2.50933
XLOC_077392	MRPL42	chr5	23434454	23502533	0.912712	4.36695	2.8553	0.736141	1.49788	1.51469	0.661505	1.27727	0.797079
XLOC_077393	MRPL42	chr5	23434454	23502533	4.62746	2.49096	3.61931	1.3271	1.66346	1.05592	1.42515	0.740036	1.69735
XLOC_077394	MRPL42	chr5	23434454	23502533	5.03599	1.12916	0.0	1.01514	1.2756	1.17282	0.793393	0.501317	0.768727
XLOC_037968	MRPL47;NDUFB5;ACTL6A	chr1	88095075	88219102	5.30203	2.06459	3.34964	0.355855	0.131341	0.237794	0.12255	0.0673518	0.202521
XLOC_039859	MRPL47;NDUFB5;ACTL6A	chr1	88095075	88219102	5.80048	3.25318	1.70168	0.389973	0.227015	0.225898	0.461457	0.363244	0.443584
XLOC_050553	MRPS26;PTPRA;PTPRA	chr13	52582802	52620937	5.80939	5.21085	3.6068	26.2259	20.8403	18.8745	21.5162	24.4411	22.3464
XLOC_053278	MRPS28	chr14	45277500	45280494	3.04105	3.33438	3.17113	0.272523	0.237485	0.315254	0.0	0.0	0.265504
XLOC_053279	MRPS28	chr14	45329796	45349723	106.02	166.725	145.724	230.816	308.269	263.211	308.071	270.927	435.728
XLOC_053280	MRPS28	chr14	45351408	45358144	8.0833	7.24157	6.38786	2.51414	4.05204	3.8444	3.7558	1.85434	5.01581
XLOC_053281	MRPS28	chr14	45367928	45371210	2.50486	0.748862	2.93776	1.4587	1.17142	2.20391	2.25533	0.748084	0.76475
XLOC_053282	MRPS28	chr14	45377432	45388971	12.102	7.73754	7.734	3.76205	6.24704	7.9028	11.4397	11.9997	12.3959
XLOC_053563	MRPS28	chr14	45285602	45295238	30.7936	28.2884	28.1907	38.1135	77.3789	53.1202	47.6571	23.4774	71.9545
XLOC_053564	MRPS28	chr14	45298000	45318732	44.4545	42.3247	20.7697	53.8206	105.081	71.4227	67.5435	31.7984	102.389
XLOC_053565	MRPS28	chr14	45377432	45388971	25.1054	34.5121	27.8857	69.3896	61.9797	63.3709	87.1341	74.3576	103.985
XLOC_054245	MRPS28	chr14	45280710	45282066	6.03454	4.33262	5.66584	0.0541015	0.378252	0.250871	0.10998	0.0605549	0.369352
XLOC_054246	MRPS28	chr14	45282222	45283197	4.42359	2.11675	1.38403	0.55506	0.414971	1.10146	0.160401	0.0	0.540946
XLOC_054247	MRPS28	chr14	45283396	45285430	6.16661	5.18952	3.92111	10.0574	19.6631	14.6806	12.554	5.97201	19.2578
XLOC_054248	MRPS28	chr14	45295877	45296482	0.0	0.966091	1.26328	2.75032	2.51227	2.50396	2.16844	0.640771	1.83046
XLOC_054249	MRPS28	chr14	45297055	45297624	8.78866	3.15143	4.12082	2.83313	3.0019	3.80894	2.5089	2.43474	4.59138
XLOC_054250	MRPS28	chr14	45351156	45351357	0.0	8.61207	0.0	3.9473	10.5664	2.87366	3.3866	1.30353	7.4026
XLOC_054251	MRPS28	chr14	45351408	45358144	3.01689	3.15597	1.1791	1.7564	1.29064	1.09125	2.0277	0.748487	2.23483
XLOC_054252	MRPS28	chr14	45351408	45358144	0.0	6.09113	7.96377	3.04265	3.39029	5.56323	7.0763	1.65093	3.53496
XLOC_054253	MRPS28	chr14	45358347	45358713	3.46738	4.13818	5.41039	2.17052	3.45348	1.4169	2.1011	1.34502	5.10245
XLOC_054254	MRPS28	chr14	45358993	45359567	3.47325	1.55683	4.07144	1.24408	1.21351	1.97146	1.8598	2.23426	1.20976
XLOC_054255	MRPS28	chr14	45361002	45361238	0.0	2.6476	0.0	4.79985	2.64643	5.34762	4.31827	0.0	1.52378
XLOC_054256	MRPS28	chr14	45361887	45362419	1.93142	2.30816	4.52719	1.55626	2.69506	0.796346	1.37406	0.953461	2.35288

XLOC_054257	MRPS28	chr14	45365905	45366262	3.62315	2.16179	0.0	3.23991	1.66366	3.69834	4.06685	1.40304	2.50784
XLOC_054258	MRPS28	chr14	45367928	45371210	8.3723	3.74524	16.3217	1.87169	2.55303	1.27834	1.07465	3.22283	6.50915
XLOC_054259	MRPS28	chr14	45371815	45372299	2.2154	2.64681	6.92175	1.98284	2.39894	2.73552	4.11604	0.653943	2.50349
XLOC_054260	MRPS28	chr14	45377033	45377338	9.77132	1.45656	0.0	3.05938	3.33737	2.9748	2.06878	4.2025	5.05614
XLOC_054261	MRPS28	chr14	45377432	45388971	6.86915	2.04956	2.67971	7.37158	8.42252	6.66773	5.65207	5.33108	8.91998
XLOC_054262	MRPS28	chr14	45377432	45388971	6.94196	10.3494	0.0	13.0846	15.6448	15.4002	13.1848	11.0923	13.7293
XLOC_054263	MRPS28	chr14	45445491	45446043	0.0	1.09567	0.0	2.29833	1.84918	2.26928	1.63339	0.906192	1.43638
XLOC_054264	MRPS28	chr14	45455946	45456641	0.0	2.01131	4.20812	0.120547	1.04805	0.69605	0.0	0.401423	0.469365
XLOC_053566	MRPS28;TPD52	chr14	45458713	45529799	92.9754	83.1649	83.1771	42.9149	50.2353	45.5186	56.1151	63.636	66.1583
XLOC_048831	MRPS31	chr12	21874417	21874930	2.03466	0.607824	0.0	0.728564	1.89119	1.04797	0.903	0.601904	1.41584
XLOC_048832	MRPS31	chr12	21877166	21877710	0.0	1.11831	0.0	0.502675	1.16116	1.73678	0.833027	0.739621	1.95455
XLOC_048833	MRPS31	chr12	21879037	21879640	0.0	0.0	0.0	1.59943	2.1449	1.34138	1.30673	1.60898	1.6972
XLOC_073266	MRPS33	chr4	105033528	105109836	0.352573	1.00242	0.551968	1.59698	2.10673	1.92891	1.71645	1.63608	2.08415
XLOC_043971	MRPS9	chr11	9117833	9136269	8.84362	12.229	10.138	344.015	305.536	309.03	372.171	302.714	458.226
XLOC_045461	MRPS9	chr11	9136327	9137539	0.0	0.205163	0.536587	1.16821	1.02035	0.569969	1.87213	0.962567	1.79857
XLOC_045462	MRPS9	chr11	9149700	9149977	23.9885	32.1797	9.34822	68.2325	54.747	62.4438	68.5728	53.9296	51.6226
XLOC_045463	MRPS9	chr11	9150072	9150628	34.4865	24.9478	18.4384	70.3718	78.024	73.5811	80.3915	66.0409	76.0011
XLOC_055587	MS4A13	chr15	84663208	84703223	6.18491	15.2567	12.1568	7.07075	5.40027	7.31678	7.38245	14.5196	5.64057
XLOC_057331	MS4A13	chr15	84663208	84703223	0.0	0.870378	2.27635	0.173891	0.22737	0.301803	0.351232	0.290748	0.423578
XLOC_057330	MS4A14	chr15	84553395	84557853	1.17719	1.91255	1.71132	1.68939	1.6264	1.75256	1.14328	1.98507	1.70842
XLOC_088571	MSANTD3	chr8	91796667	91797547	0.0	0.299353	0.0	0.0	0.0	0.0	0.0	0.0	0.0
XLOC_044006	MSH2;KCNK12	chr11	29678122	29777445	164.352	127.182	159.564	113.98	93.2847	110.714	119.34	113.292	98.0521
XLOC_044701	MSH2;KCNK12	chr11	29678122	29777445	7.75109	9.348	6.60447	3.51855	2.52971	3.19373	2.16941	3.45904	2.67977
XLOC_045707	MSH2;KCNK12	chr11	29678122	29777445	0.0	0.0	0.0	1.93595	1.3675	1.39727	1.09253	2.01449	1.41333
XLOC_045708	MSH2;KCNK12	chr11	29678122	29777445	12.7085	11.3885	9.92752	1.51674	1.47557	1.96271	2.06493	2.0865	2.02599
XLOC_045709	MSH2;KCNK12	chr11	29678122	29777445	17.099	13.4285	14.6339	4.50346	5.69705	5.8337	7.26453	4.40059	8.08491
XLOC_085232	MSH3	chr7	83189725	83190230	0.0	0.0	0.0	1.86326	0.161168	0.428718	0.738407	0.410276	0.543073
XLOC_071435	MSH4	chr3	69222411	69223583	0.0	0.426494	0.0	0.0	0.0	0.0	0.0	0.0	0.0
XLOC_063890	MSI2	chr19	8618106	8621361	0.0	0.0	0.0	0.0	0.0	0.0	0.0	0.0	0.077752
XLOC_063891	MSI2	chr19	8723470	8726597	33.6605	53.3288	45.4675	183.472	180.94	182.827	258.61	220.713	233.319

XLOC_064158	MSI2	chr19	8707450	8723346	163.504	124.121	116.816	137.378	165.686	148.087	200.265	155.931	134.371
XLOC_064518	MSI2	chr19	8694732	8695289	1.81054	0.0	0.0	1.29692	0.98343	0.373532	0.968171	0.0	1.57613
XLOC_064519	MSI2	chr19	8702442	8703342	0.974089	1.45649	1.52369	1.74592	2.81547	1.61608	3.70257	1.55684	2.72181
XLOC_064520	MSI2	chr19	8704929	8705092	0.0	0.0	0.0	22.6043	14.5637	23.6431	10.166	17.7185	35.0306
XLOC_064521	MSI2	chr19	8705147	8705808	2.87304	2.14695	4.49189	5.27573	4.36028	4.75289	5.41403	4.28058	5.1342
XLOC_064522	MSI2	chr19	8706058	8706648	0.0	4.49796	2.61404	4.49298	4.54694	2.93524	2.98848	5.79727	5.68071
XLOC_064523	MSI2	chr19	8706856	8707278	5.46904	2.44912	4.26965	7.0945	5.05567	4.4881	5.99265	6.15041	8.06725
XLOC_064524	MSI2	chr19	8707450	8723346	1.43367	0.85708	0.0	2.69685	1.5628	1.33412	1.92985	2.27853	0.99982
XLOC_064525	MSI2	chr19	8707450	8723346	0.0	1.16338	0.0	1.74311	0.906364	1.80608	0.692383	1.34537	1.18587
XLOC_093330	MSN	chrX	100073560	100074932	0.595504	0.356298	0.0	0.0	0.0	0.0618945	0.0542737	0.0	0.260354
XLOC_093331	MSN	chrX	100085866	100086462	1.64949	1.97178	1.28917	0.14772	0.128158	0.0	0.442322	0.0	0.0
XLOC_093332	MSN	chrX	100116445	100118252	0.0	0.39356	0.686229	0.196578	0.103198	0.091232	0.0	0.0	0.153442
XLOC_093333	MSN	chrX	100139835	100140202	0.0	1.02964	2.69237	0.0	0.26442	0.0	0.298811	0.0	0.0
XLOC_093334	MSN	chrX	100141772	100142448	0.0	0.416984	1.09053	0.0	0.108603	0.288535	0.0	0.0	0.12162
XLOC_093335	MSN	chrX	100160502	100161241	0.0	0.743716	0.972524	0.0	0.0969536	0.128757	0.0	0.0	0.0
XLOC_086772	MSRA	chr8	8617469	8661363	17.6569	11.6926	13.9632	3.85495	2.73013	4.54389	1.18826	2.12029	1.14816
XLOC_086773	MSRA	chr8	8664894	8690322	63.6763	57.883	73.5503	85.9953	86.6868	89.5083	99.8404	92.4373	77.3369
XLOC_087215	MSRA	chr8	8617469	8661363	4.3354	3.88764	4.51878	1.42391	0.112464	0.448231	0.388974	0.143526	0.251942
XLOC_087216	MSRA	chr8	8617469	8661363	3.01963	1.80254	2.35677	0.0	0.696337	3.09244	0.526775	0.588631	0.523519
XLOC_087217	MSRA	chr8	8617469	8661363	6.31684	3.39927	3.95118	0.67912	0.590775	0.523061	1.25102	0.377259	0.881599
XLOC_087218	MSRA	chr8	8617469	8661363	6.05234	2.06871	3.38154	1.62739	0.608416	1.07658	0.940897	0.778355	1.13292
XLOC_087219	MSRA	chr8	8617469	8661363	2.934	3.5031	2.29012	1.04978	0.451352	2.10435	0.512595	0.572503	0.508818
XLOC_087220	MSRA	chr8	8617469	8661363	2.5092	3.00064	9.80949	2.58525	1.46679	2.2077	0.790704	0.249787	0.984948
XLOC_087221	MSRA	chr8	8617469	8661363	1.53429	2.98408	3.00184	1.9262	0.812886	1.3972	1.54427	0.926281	0.53705
XLOC_087222	MSRA	chr8	8617469	8661363	2.16866	1.5136	2.82764	1.23122	1.24486	2.10208	0.788728	1.15893	0.884516
XLOC_087223	MSRA	chr8	8661840	8662577	0.0	0.74628	0.975877	0.559105	0.389139	0.0	0.224773	0.248514	0.0
XLOC_087224	MSRA	chr8	8662965	8664831	0.0	0.126658	0.662541	0.303667	0.531447	1.05708	0.851497	0.340582	0.296302
XLOC_062376	MT1A;MT1E;MT1E	chr18	24105939	24122024	3.9684	6.33119	6.44882	12.0341	9.50329	11.6876	11.3227	15.4217	10.0384
XLOC_055527	MTCH2	chr15	78597150	78622016	2.69229	1.10886	1.12025	0.423469	0.634613	0.350268	0.131221	0.474238	0.530063
XLOC_053697	MTERFD1;UQCRB	chr14	70305135	70351605	11.5462	15.1402	19.1423	32.3825	28.3245	25.4653	21.4313	25.7737	19.6761
XLOC_054780	MTERFD1;UQCRB	chr14	70305135	70351605	0.796094	2.14299	3.73653	4.0674	4.48409	3.9667	2.02402	4.54275	1.87811

XLOC_054781	MTERFD1;UQCRB	chr14	70305135	70351605	1.80735	1.26158	0.942747	3.7808	1.55774	2.69238	2.745	2.96232	1.68566
XLOC_070118	MTF1;YRDC;MANEAL	chr3	108653915	108722184	14.1722	15.8363	15.1	14.9415	18.3927	16.5324	14.6967	13.1706	18.0008
XLOC_070493	MTF1;YRDC;MANEAL	chr3	108653915	108722184	10.2648	11.3826	8.69015	9.27442	13.496	11.0233	12.7972	8.96268	10.0828
XLOC_060305	MTFP1;SEC14L3;SEC14L2	chr17	71569820	71598761	5.51804	3.70143	2.00038	6.79582	7.25785	6.0742	9.40076	8.19452	8.79927
XLOC_042992	MTHFD1	chr10	76881849	76882528	0.0	0.0	0.0	0.248482	0.431942	0.143445	0.124569	0.275688	0.120924
XLOC_042993	MTHFD1	chr10	76886612	76887786	0.0	0.425655	1.11326	0.701597	0.891184	0.812877	0.388238	0.142605	0.497502
XLOC_042994	MTHFD1	chr10	76889569	76890082	0.0	0.0	0.0	0.364282	1.57599	1.25757	0.0	0.200635	1.06188
XLOC_042995	MTHFD1	chr10	76891391	76892362	0.0	0.0	0.695413	0.637471	0.416997	0.830138	0.483531	0.533415	0.854224
XLOC_089555	MTHFD1L	chr9	89210990	89332880	10.3527	2.68318	2.98629	4.0773	10.1553	6.52361	8.66675	10.5314	7.41335
XLOC_048987	MTIF3	chr12	32711467	32714022	4.2232	4.78424	3.30536	2.78646	3.15116	2.76414	2.9579	2.43067	3.5383
XLOC_092858	MTM1	chrX	33505534	33507180	31.5822	22.2416	25.8542	135.665	120.795	134.07	130.901	163.865	144.891
XLOC_059889	MTMR3	chr17	71122569	71126639	3.21823	1.9236	0.0	0.144111	0.0	0.166195	0.287876	0.318988	0.280364
XLOC_059890	MTMR3	chr17	71183598	71248139	40.3512	31.9342	32.0063	4.30341	4.46317	3.75225	4.55729	4.10722	3.79255
XLOC_061890	MTMR3	chr17	71248229	71250264	23.8829	21.6691	21.4039	2.59072	2.7816	2.36529	2.67865	2.67435	2.3933
XLOC_066507	MTMR9;EIF3I	chr2	122120012	122129991	1.15995	1.20202	1.02506	3.94587	4.90625	3.85902	3.21626	2.31032	4.05812
XLOC_074749	MTPN	chr4	100567411	100568051	11.9924	9.40912	12.8895	7.2505	8.62915	8.67625	8.33049	6.99283	10.9742
XLOC_065497	MTX2;HOXD4;MIR10B	chr2	20510042	20810602	20.2232	24.9065	20.1147	72.902	70.6762	73.8325	71.3743	79.8923	56.1909
XLOC_066965	MTX2;HOXD4;MIR10B	chr2	20510042	20810602	0.0	0.0	4.23772	8.25544	5.85496	6.9609	5.23588	4.24748	7.53664
XLOC_066966	MTX2;HOXD4;MIR10B	chr2	20510042	20810602	1.35828	2.43621	3.18569	7.05731	6.87563	6.6038	4.88239	5.67182	8.29082
XLOC_066967	MTX2;HOXD4;MIR10B	chr2	20510042	20810602	2.72423	2.43994	2.12682	6.33669	6.08642	7.26615	5.01579	8.52592	6.6192
XLOC_066968	MTX2;HOXD4;MIR10B	chr2	20510042	20810602	0.0	1.16181	6.07573	7.31412	5.95035	8.33774	5.69213	7.51955	11.4495
XLOC_066969	MTX2;HOXD4;MIR10B	chr2	20510042	20810602	0.0	0.680431	1.77938	7.13624	7.39715	7.02997	5.23459	6.27176	6.53398
XLOC_066970	MTX2;HOXD4;MIR10B	chr2	20510042	20810602	19.9917	5.9722	0.0	8.13921	7.43026	12.0358	2.41309	4.61509	4.29321
XLOC_066971	MTX2;HOXD4;MIR10B	chr2	20510042	20810602	0.0	4.69909	0.0	12.7529	12.9691	9.51233	7.73402	8.82937	8.79968
XLOC_066972	MTX2;HOXD4;MIR10B	chr2	20510042	20810602	0.0	1.17435	3.07066	4.57681	5.71237	7.22249	3.38229	6.07804	6.80733

XLOC_066973	MTX2;HOXD4;MIR10B	chr2	20510042	20810602	0.0	2.05101	10.724	18.5189	11.3712	13.8746	12.5049	16.1707	13.0143
XLOC_066974	MTX2;HOXD4;MIR10B	chr2	20510042	20810602	3.1786	0.0	0.0	5.68569	4.14872	7.15516	4.14749	3.71098	6.33454
XLOC_066975	MTX2;HOXD4;MIR10B	chr2	20510042	20810602	0.0	0.0	0.0	4.65196	6.10639	5.2813	4.01317	5.43072	4.89535
XLOC_066976	MTX2;HOXD4;MIR10B	chr2	20510042	20810602	0.0	0.0	9.59618	4.46832	6.34546	13.5131	2.92117	2.24323	10.5353
XLOC_066977	MTX2;HOXD4;MIR10B	chr2	20510042	20810602	0.0	1.08212	1.88672	2.70237	3.1984	4.87186	1.73952	3.60517	4.421
XLOC_066978	MTX2;HOXD4;MIR10B	chr2	20510042	20810602	2.87573	0.0	4.48953	4.88765	5.7536	6.48451	4.02341	3.36908	6.48492
XLOC_066979	MTX2;HOXD4;MIR10B	chr2	20510042	20810602	0.0	1.91078	0.0	9.76843	5.3086	12.943	5.32349	5.44277	4.96357
XLOC_066980	MTX2;HOXD4;MIR10B	chr2	20510042	20810602	0.0	2.62277	0.0	4.32262	5.43422	6.77737	7.77151	4.10493	5.53453
XLOC_066981	MTX2;HOXD4;MIR10B	chr2	20510042	20810602	7.54744	2.25142	2.94351	5.0617	5.1929	6.92829	3.25037	2.55319	4.56956
XLOC_066982	MTX2;HOXD4;MIR10B	chr2	20510042	20810602	0.0	4.90074	0.0	4.43634	3.06968	6.60847	6.70019	0.765644	7.0571
XLOC_065901	MUL1	chr2	132686760	132750909	0.883559	1.7849	1.38226	5.86954	7.30699	4.03298	4.85823	5.89879	8.65037
XLOC_082991	MUM1	chr7	45413692	45416547	3.06454	1.65937	0.481382	1.72199	1.22325	1.59758	0.840487	1.85098	1.45273
XLOC_082992	MUM1	chr7	45418368	45421779	0.329988	0.789987	0.258258	0.295951	0.278468	0.377929	0.181334	0.199367	0.173282
XLOC_089509	MYB	chr9	74110444	74282575	0.0	0.334734	0.0	0.143123	0.058316	0.0773885	0.0	0.060234	0.032632
XLOC_053259	MYBL1	chr14	32856878	32939012	4.33638	5.2046	2.97402	0.0486984	0.0	0.0	0.0	0.163638	0.0
XLOC_076350	MYBPC1	chr5	65763513	65787399	4.71541	4.09375	8.35494	6.10764	6.92221	7.54863	7.22136	4.67839	4.07805
XLOC_076351	MYBPC1	chr5	65790059	65826915	11.5974	15.7888	10.0749	19.9296	21.8453	23.3066	27.0007	20.7823	18.6554
XLOC_078063	MYBPC1	chr5	65760659	65763389	1.68681	0.841296	0.660121	0.352985	0.198763	0.292776	0.283506	0.283236	0.172275
XLOC_078064	MYBPC1	chr5	65763513	65787399	0.0	3.22979	2.81552	2.74226	1.39802	1.48677	1.28467	1.60334	0.784167
XLOC_078065	MYBPC1	chr5	65763513	65787399	0.0	1.529	0.0	0.687272	2.17256	0.525825	0.900528	1.00359	0.222295
XLOC_078066	MYBPC1	chr5	65763513	65787399	0.0	5.83217	0.0	2.33898	0.49081	3.29111	4.32747	2.45911	0.560946
XLOC_078067	MYBPC1	chr5	65787549	65788068	0.0	0.597754	3.12645	0.358247	0.930147	1.03079	0.888554	0.197385	0.348127
XLOC_078068	MYBPC1	chr5	65788676	65788832	0.0	12.9499	0.0	4.11212	12.1345	4.2498	6.33179	7.3581	3.67846
XLOC_078069	MYBPC1	chr5	65827050	65828488	2.82404	5.57606	3.53548	11.8495	12.749	12.4477	14.4731	11.2841	9.68091
XLOC_078070	MYBPC1	chr5	65828604	65830361	4.97377	5.95258	3.18452	10.136	10.6767	11.6184	12.1067	10.0462	12.1046
XLOC_055854	MYBPC3;SPI1	chr15	78399603	78406407	6.03324	9.7963	2.76476	12.3393	6.57403	8.15148	6.52586	13.9589	8.82411
XLOC_047847	MYCBP2	chr12	52503795	52570667	34.7333	30.6204	31.6949	25.9249	29.0271	27.9316	32.4046	27.0792	37.9022

XLOC_049346	MYCBP2	chr12	52501896	52502655	0.0	0.0	0.0	0.107736	0.937607	0.249014	0.0	0.0	0.209819
XLOC_049347	MYCBP2	chr12	52503795	52570667	0.0	1.91875	12.0426	1.4949	1.30025	2.92242	3.00306	1.91595	2.12709
XLOC_049348	MYCBP2	chr12	52503795	52570667	4.92652	7.36082	7.69993	2.49985	1.91376	3.05203	3.3762	2.44032	2.71756
XLOC_049349	MYCBP2	chr12	52571006	52572213	2.75653	2.88596	2.69571	1.17377	1.94246	1.28851	1.75554	1.58883	2.52996
XLOC_049350	MYCBP2	chr12	52572307	52574443	1.82771	1.53131	3.14687	0.524483	1.3774	1.17948	1.17105	0.662202	1.98338
XLOC_049351	MYCBP2	chr12	52574615	52575194	3.43205	2.56398	2.68215	1.53668	1.19931	4.25088	1.99163	0.849347	2.68987
XLOC_049352	MYCBP2	chr12	52602630	52603375	0.0	0.368065	0.0	1.6545	0.671812	0.637259	0.11089	0.12259	0.644392
XLOC_064944	MYH10	chr19	28742462	28742593	0.0	132.77	0.0	689.14	492.388	456.696	337.588	556.188	472.572
XLOC_064945	MYH10	chr19	28763025	28763531	6.22661	9.92015	12.9714	17.0929	11.0892	16.0314	11.7819	22.7066	12.9966
XLOC_064226	MYH2;MYH1	chr19	30098415	30175589	5.35994	10.4528	5.76784	91.3324	38.4129	86.758	29.7325	49.562	22.9008
XLOC_063963	MYH3	chr19	30232941	30239672	0.0	0.536388	1.40288	0.0	0.0	0.0	0.162541	0.0	0.156646
XLOC_063962	MYH8	chr19	30030467	30093959	26.7873	24.5888	28.6245	20.3365	24.0411	21.4641	23.7242	18.2223	23.4777
XLOC_064984	MYH8	chr19	30030467	30093959	7.56471	13.5372	5.89835	3.39973	4.53478	3.80894	4.34565	2.12419	6.50169
XLOC_064985	MYH8	chr19	30030467	30093959	12.4867	7.44779	6.49148	3.34979	2.85621	2.11863	3.56301	1.6026	4.6745
XLOC_076372	MYH9	chr5	75151580	75197447	8.14529	7.07757	7.56825	1.0312	0.431559	0.757677	1.22511	0.457349	0.766687
XLOC_060267	MYL2	chr17	56954399	56959230	0.595504	0.0	0.465937	0.160168	0.139985	0.123789	0.0	0.179289	0.156213
XLOC_065714	MYO1B	chr2	80315482	80353095	25.0349	28.6665	26.364	22.0139	24.5875	23.898	26.3168	25.1861	27.3117
XLOC_068017	MYO1B	chr2	80353203	80354838	7.83197	9.37292	9.959	7.37356	8.06151	7.68745	6.7945	7.27994	8.56366
XLOC_068018	MYO1B	chr2	80356468	80356796	0.0	0.0	0.0	0.0	3.22733	0.0	0.362105	0.407331	1.82887
XLOC_064767	MYO1C	chr19	23169306	23169999	2.70134	1.61505	0.0	1.81495	0.946734	1.25753	3.52039	1.07439	2.47333
XLOC_064768	MYO1C	chr19	23170312	23171106	5.68162	3.39759	0.888582	1.32364	2.39359	2.00105	2.97342	1.81253	2.37994
XLOC_064769	MYO1C	chr19	23175413	23176113	0.0	1.19568	0.0	0.716626	1.14235	0.275875	0.119864	0.132594	0.348795
XLOC_063938	MYO1C;CRK	chr19	23134971	23168526	29.2274	26.5415	30.6228	60.0796	53.7557	57.6924	62.9948	64.1643	58.5701
XLOC_064761	MYO1C;CRK	chr19	23134971	23168526	0.0	1.78076	1.55242	6.81874	4.04023	5.70341	3.79297	3.44884	4.16301
XLOC_064762	MYO1C;CRK	chr19	23134971	23168526	0.0	0.682908	1.19055	2.66011	3.03672	1.65913	1.79754	2.43873	1.6624
XLOC_064763	MYO1C;CRK	chr19	23134971	23168526	4.18996	4.31807	5.10034	13.6921	11.1012	10.9445	9.44226	9.73364	8.18616
XLOC_064764	MYO1C;CRK	chr19	23134971	23168526	0.0	2.32675	6.0799	12.621	9.33914	5.49349	23.6213	12.388	10.7199
XLOC_064765	MYO1C;CRK	chr19	23134971	23168526	11.3454	4.41012	8.87195	19.7218	13.7197	15.6309	20.2701	12.8944	13.9604
XLOC_064766	MYO1C;CRK	chr19	23134971	23168526	22.0374	8.76388	22.9089	24.4174	19.2823	30.3432	22.3525	17.2146	15.1552
XLOC_089282	MYO6	chr9	15709609	15739028	10.172	9.27184	12.5488	0.456353	0.691411	0.454505	0.71691	0.322343	0.543429
XLOC_090299	MYO6	chr9	15739405	15742479	6.44735	6.23354	5.24051	0.24464	0.155881	0.361614	0.0909895	0.0749792	0.130263
XLOC_084984	MYOZ3	chr7	64032123	64033432	0.0	0.37578	0.0	0.900935	0.738025	0.717945	0.343251	0.126015	0.933526

XLOC_069962	MYSM1	chr3	87926744	87939548	47.4179	42.8677	32.8523	66.995	57.8348	62.0163	75.2841	92.7115	66.9021
XLOC_069963	MYSM1	chr3	87948317	87953393	2.54103	1.71031	3.97617	10.5359	7.76266	9.37343	5.38043	6.88191	12.163
XLOC_070348	MYSM1	chr3	87926744	87939548	1.17701	6.92577	6.4818	10.8858	13.0741	11.3936	7.34252	9.55536	10.5784
XLOC_071554	MYSM1	chr3	87926033	87926273	0.0	2.53142	0.0	8.40695	1.2671	5.11723	0.690479	3.948	2.91497
XLOC_071555	MYSM1	chr3	87939742	87939968	59.975	56.7353	39.0278	92.2443	57.213	80.2383	93.3056	114.454	94.4223
XLOC_071556	MYSM1	chr3	87940421	87940648	59.2301	38.3329	15.4158	66.9603	53.5862	57.4579	85.07	86.6499	55.1028
XLOC_071557	MYSM1	chr3	87940820	87941350	0.0	1.16027	4.55145	2.43382	0.451551	0.800571	0.517942	0.958461	1.18271
XLOC_071558	MYSM1	chr3	87941717	87942229	4.08081	4.87628	3.18806	2.19184	2.05447	2.9425	3.07854	1.40831	1.59728
XLOC_071559	MYSM1	chr3	87943436	87944287	3.12876	0.935579	1.63124	1.1215	0.895682	0.972862	1.13119	0.832724	1.72984
XLOC_071560	MYSM1	chr3	87944488	87946941	1.26025	2.35697	0.986339	1.07368	0.96498	1.01691	0.750492	0.698021	0.772162
XLOC_062476	MZF1	chr18	65980705	66002713	27.134	32.1202	27.0166	56.3448	66.2848	56.5156	63.3658	64.6164	76.1771
XLOC_048112	N4BP2L1	chr12	28594172	28623333	0.570152	0.682276	1.78445	0.0	0.0	0.0	0.0	0.0	0.0997174
XLOC_048113	N4BP2L1	chr12	28631053	28636258	8.28023	9.59744	9.99994	31.278	22.5461	31.6302	28.8056	30.1025	30.2966
XLOC_048961	N4BP2L1	chr12	28629549	28629994	7.54761	3.75621	3.9291	8.55442	6.98891	8.01071	5.75622	8.87979	5.4616
XLOC_048962	N4BP2L1	chr12	28631053	28636258	2.82625	2.53656	4.86508	11.5547	10.8982	10.4007	10.5669	13.3925	9.44131
XLOC_059167	NADK	chr16	52208130	52208836	0.0	1.18261	1.03096	0.236266	0.308177	0.0	0.0	0.0	0.459996
XLOC_062185	NAE1	chr18	34599886	34651934	20.1266	27.1308	17.2498	81.2337	75.1568	79.2403	94.192	110.338	95.9371
XLOC_062390	NAE1	chr18	34599886	34651934	54.5811	68.0305	61.5914	133.451	129.752	120.604	149.609	141.498	146.361
XLOC_062391	NAE1	chr18	34599886	34651934	12.9778	12.4892	9.70526	41.4705	28.4532	35.9445	36.6766	41.2225	33.5109
XLOC_062881	NAE1	chr18	34599886	34651934	8.42067	20.0897	26.2652	30.1225	27.9201	33.4249	27.7315	29.9759	32.368
XLOC_062882	NAE1	chr18	34599886	34651934	5.19658	3.09831	0.0	6.51301	6.69311	3.68709	6.57293	8.9145	6.71831
XLOC_045062	NAIF1	chr11	98704513	98729413	16.6332	25.0199	18.3404	28.3339	23.0883	24.4013	26.8327	32.2473	22.5059
XLOC_047230	NAIF1	chr11	98704513	98729413	3.46747	2.0691	0.0	2.48209	0.531305	0.708451	0.600354	0.337556	0.900433
XLOC_076938	NANOG;SLC2A3;C3AR1;FOXJ2;NECAP1	chr5	101832852	102069974	18.5877	17.5839	19.2406	7.89445	7.1693	7.52401	7.29197	8.80787	5.96628
XLOC_078445	NANOG;SLC2A3;C3AR1;FOXJ2;NECAP1	chr5	101832852	102069974	2.91041	0.868775	2.27176	5.46728	3.80633	4.1756	2.79776	6.81698	4.7953
XLOC_078446	NANOG;SLC2A3;C3AR1;FOXJ2;NECAP1	chr5	101832852	102069974	2.04746	0.306135	0.800629	3.66965	2.31825	3.07752	2.3141	4.19027	1.60875

XLOC_078447	NANOG;SLC2A3;C3AR1;FOXJ2;NECAP1	chr5	101832852	102069974	0.701159	1.04868	1.09709	1.31998	1.20766	1.31094	1.27555	1.26502	1.22572
XLOC_078448	NANOG;SLC2A3;C3AR1;FOXJ2;NECAP1	chr5	101832852	102069974	0.0	0.454652	0.0	1.63497	1.65629	1.72921	1.77172	1.66043	2.2535
XLOC_078449	NANOG;SLC2A3;C3AR1;FOXJ2;NECAP1	chr5	101832852	102069974	0.0	0.689099	1.80203	0.826055	1.2483	1.42381	2.8531	0.453784	1.60398
XLOC_078450	NANOG;SLC2A3;C3AR1;FOXJ2;NECAP1	chr5	101832852	102069974	0.0	0.562073	0.0	0.673796	1.45889	0.969951	3.01397	1.11527	1.4735
XLOC_078451	NANOG;SLC2A3;C3AR1;FOXJ2;NECAP1	chr5	101832852	102069974	12.7095	0.0	4.95306	2.27898	1.43587	2.56736	2.11262	2.4001	4.37417
XLOC_078452	NANOG;SLC2A3;C3AR1;FOXJ2;NECAP1	chr5	101832852	102069974	0.0	0.0	1.96455	1.35081	1.16482	2.32584	1.10697	1.48023	1.52926
XLOC_078453	NANOG;SLC2A3;C3AR1;FOXJ2;NECAP1	chr5	101832852	102069974	0.0	0.0	0.0	0.314735	0.808255	1.43732	0.608535	0.341322	2.74017
XLOC_078454	NANOG;SLC2A3;C3AR1;FOXJ2;NECAP1	chr5	101832852	102069974	3.51546	1.05049	4.12082	1.10185	0.955151	1.81388	1.41126	0.869737	1.53047
XLOC_078455	NANOG;SLC2A3;C3AR1;FOXJ2;NECAP1	chr5	101832852	102069974	3.04627	0.911394	0.794565	0.773904	0.995266	0.950403	0.880614	0.51013	0.621721
XLOC_078456	NANOG;SLC2A3;C3AR1;FOXJ2;NECAP1	chr5	101832852	102069974	2.76831	1.40831	1.95005	1.16689	1.00038	0.864918	1.4467	0.892556	1.04208
XLOC_076736	NAP1L1	chr5	5581118	5664428	1.60894	2.06356	1.25936	0.824588	0.668346	0.694381	0.717119	0.787824	0.744619
XLOC_044311	NBAS	chr11	82951945	82961891	2.34259	0.690845	1.22235	0.396853	0.0806105	0.216883	0.381916	0.262273	0.413225
XLOC_044312	NBAS	chr11	82980787	83032242	4.15106E-	7.28219E-	0.0	4.37561	2.35989	1.45822	0.0	5.0989	4.46853

XLOC_044313	NBAS	chr11	83065107	83214810	22.3614	5.11059	4.0185	0.864273	0.506225	0.49968	0.916075	0.989332	1.34104
XLOC_044949	NBAS	chr11	82877671	82890632	1.92643	2.42939	1.12109	8.57871	8.97563	7.12999	6.17143	4.99811	8.20893
XLOC_044950	NBAS	chr11	82900749	82908412	8.02721	5.19854	3.13205	15.6339	8.41196	12.4111	10.8556	17.3448	8.44198
XLOC_044951	NBAS	chr11	82980787	83032242	1.67784	0.944763	0.318208	19.9882	21.3567	26.5621	23.3438	10.9288	16.3443
XLOC_044952	NBAS	chr11	82980787	83032242	0.962423	0.0	0.0	0.0	0.300801	0.399209	0.0871615	0.192302	0.0840416
XLOC_046746	NBAS	chr11	82897127	82897976	0.0	0.0	0.0	0.0	0.0816591	0.0	0.0	0.208775	0.0
XLOC_046747	NBAS	chr11	82899290	82900647	0.602951	0.0	0.0	0.108113	0.0	0.187997	0.0549449	0.0605048	0.0
XLOC_046748	NBAS	chr11	82961960	82962541	3.41584	1.02076	0.0	0.0	0.0	0.0	0.457503	0.507251	0.892415
XLOC_046749	NBAS	chr11	82962732	82963188	0.0	0.0	1.89235	0.0	0.187119	0.0	0.213589	0.237835	0.0
XLOC_046750	NBAS	chr11	82964010	82964805	2.26908	0.0	0.0	0.0	0.0	0.117525	0.307123	0.113108	0.495047
XLOC_046751	NBAS	chr11	82980787	83032242	0.0	0.0	0.0	4.06906	3.74087	6.64789	2.82369	2.21149	3.37808
XLOC_046752	NBAS	chr11	82980787	83032242	0.0	1.65397	0.0	4.96717	0.839362	1.12338	0.465909	1.58146	0.477919
XLOC_046753	NBAS	chr11	82980787	83032242	0.0	0.0	0.0	1.46286	4.86156	4.08736	2.65415	3.78982	9.77474
XLOC_046754	NBAS	chr11	83035235	83036177	0.921935	0.55143	0.721097	0.330507	0.720498	0.95628	1.33627	0.276456	0.483108
XLOC_046755	NBAS	chr11	83065107	83214810	3.88358	0.580133	0.0	0.0	0.0	0.0	0.0	0.191694	0.168959
XLOC_065754	NBEAL1;CYP20A1	chr2	91730000	91910508	16.0352	21.6336	19.6706	12.4396	11.5131	12.5014	14.0039	16.7551	7.63636
XLOC_053417	NBN	chr14	76090196	76135133	12.0205	21.5926	15.0278	46.4895	47.4773	41.8806	52.3097	51.6112	53.9501
XLOC_053715	NBN	chr14	76090196	76135133	7.08962	7.80211	8.90315	14.7396	20.8026	18.203	17.9074	20.7405	22.9287
XLOC_054839	NBN	chr14	76084558	76085836	0.0	0.579256	0.0	0.405057	0.606671	0.53653	0.411407	0.064737	0.564291
XLOC_054840	NBN	chr14	76086098	76087593	3.78516	3.23548	4.23111	8.3389	10.3429	8.32077	9.86653	9.28588	13.9037
XLOC_054841	NBN	chr14	76088251	76089136	2.98253	2.08108	3.8877	8.46397	12.657	11.8536	10.5231	11.6163	16.1456
XLOC_054842	NBN	chr14	76089442	76090050	14.4499	8.15717	6.2744	19.987	22.8362	21.0604	17.9535	25.3043	35.8074
XLOC_053670	NCALD	chr14	64810796	64949876	16.9817	10.1673	16.5058	10.1398	13.4187	9.74914	10.3113	9.59502	9.96886
XLOC_054669	NCALD	chr14	64810796	64949876	5.67841	7.21386	5.54884	3.17908	3.53601	7.04611	5.60678	3.80794	5.07405
XLOC_054670	NCALD	chr14	64810796	64949876	6.68717	6.65784	3.48217	3.99008	4.65453	3.89888	4.92903	2.41218	3.10005
XLOC_054671	NCALD	chr14	64810796	64949876	9.95676	6.80285	3.33585	3.05791	4.31789	4.70657	3.44712	1.9783	1.98399
XLOC_075081	NCAPG2	chr4	120136855	120137499	2.97345	5.33249	1.16216	0.0	0.0	0.0	0.0	0.0	0.0
XLOC_038486	NCEH1	chr1	95588356	95667537	2.22291	0.478508	0.535392	0.0460066	0.0134403	0.0534543	0.0157041	0.0344971	0.0
XLOC_039986	NCEH1	chr1	95587715	95588224	0.0	0.614728	1.60761	0.184209	0.0	0.0	0.0	0.0	0.0
XLOC_076373	NCF4	chr5	75574928	75655614	8.85909	10.1301	10.1361	26.0198	27.8339	23.8596	48.4385	47.4873	42.4463
XLOC_078151	NCF4	chr5	75655733	75656681	15.5539	10.1241	13.5969	28.3721	30.748	27.7126	49.2389	51.7665	44.8289
XLOC_038055	NCK1	chr1	133350685	133360309	45.9846	12.5378	11.8415	0.0521867	0.0912298	0.0605047	0.0530639	0.0	0.0

XLOC_038056	NCK1	chr1	133363582	133380451	10.1373	12.2508	9.43592	1.59003	1.97459	2.04732	0.974502	2.37065	1.35436
XLOC_040335	NCK1	chr1	133341066	133341387	0.0	0.0	0.0	1.57902	2.01605	0.897694	1.12923	4.23811	3.81091
XLOC_038543	NCK1;TMEM22	chr1	133381891	133423979	13.8733	16.9642	18.0419	6.76634	6.082	6.32751	5.13596	8.48712	4.38623
XLOC_076774	NCKAP1L	chr5	25691216	25705951	1.59784	3.77818	1.73489	7.45772	7.97574	7.08396	7.7404	8.67128	10.1664
XLOC_077448	NCKAP1L	chr5	25667550	25667930	0.0	0.0	0.0	2.03453	2.49382	0.0	1.41178	1.26371	0.281507
XLOC_077449	NCKAP1L	chr5	25668564	25668957	6.14167	0.916513	2.39662	2.74662	2.59553	1.57199	0.267618	2.09392	1.59692
XLOC_077450	NCKAP1L	chr5	25680680	25681904	2.71259	1.01428	0.0	1.94538	1.38065	1.12717	0.925663	1.35975	1.18559
XLOC_077451	NCKAP1L	chr5	25683769	25684337	0.0	1.05303	0.0	1.26223	3.55622	4.18175	3.14352	3.31217	1.841
XLOC_077452	NCKAP1L	chr5	25685654	25686109	0.0	2.17816	0.0	1.52297	3.00378	3.24808	2.14275	1.67028	1.68948
XLOC_077453	NCKAP1L	chr5	25686820	25687258	2.57863	1.53988	0.0	5.30656	5.17094	5.29515	3.39995	3.7894	4.02957
XLOC_077454	NCKAP1L	chr5	25687607	25688074	2.33708	0.697966	7.30104	4.39217	2.52828	3.60455	1.85684	1.83695	3.24905
XLOC_077455	NCKAP1L	chr5	25688321	25688571	0.0	0.0	11.9152	4.80814	4.57884	4.61579	8.77198	6.43324	6.56645
XLOC_077456	NCKAP1L	chr5	25688769	25689673	0.96887	3.18711	4.54661	5.47022	3.4817	3.51634	4.29694	3.8715	5.07615
XLOC_077457	NCKAP1L	chr5	25690141	25690386	8.04137	16.7926	6.27137	38.3525	32.4802	32.3621	28.2518	33.0189	29.0191
XLOC_077458	NCKAP1L	chr5	25690525	25690897	10.1122	3.01733	0.0	9.6461	10.5955	11.714	5.84521	15.0527	12.5502
XLOC_077459	NCKAP1L	chr5	25691216	25705951	0.0	1.2691	0.0	2.09163	2.30195	1.74967	2.25902	1.88323	1.66246
XLOC_076253	NCKAP5L;MIR2425	chr5	30232574	30272335	7.60761	6.30285	6.47323	4.73275	5.28192	6.15144	4.04127	4.24108	3.83413
XLOC_065823	NCL	chr2	120032598	120042498	2.98435	2.66271	2.03777	2.57727	2.93003	2.28422	2.0217	3.19781	2.63622
XLOC_044234	NCOA1	chr11	74629701	74638209	0.0	0.0	0.0	0.820951	1.22388	1.08369	0.470988	1.69302	0.685022
XLOC_044235	NCOA1	chr11	74649289	74670849	20.7388	33.3357	29.2089	46.6562	26.6958	35.1791	41.0895	80.5062	26.9005
XLOC_046548	NCOA1	chr11	74610930	74611493	0.0	0.0	0.0	0.638883	1.10752	0.552097	0.0	0.0	1.55296
XLOC_046549	NCOA1	chr11	74619895	74620826	0.0	0.0	0.0	1.00561	1.3152	0.678861	0.0	1.30827	0.97991
XLOC_046550	NCOA1	chr11	74623414	74624021	0.0	0.0	0.0	0.432332	0.750371	0.498586	0.143938	0.318988	1.40182
XLOC_046551	NCOA1	chr11	74638295	74641702	0.222563	0.732661	1.04524	2.75467	2.65876	2.64323	1.20513	2.22145	2.06567
XLOC_046552	NCOA1	chr11	74649289	74670849	0.0	2.09945	0.0	3.77495	0.808254	5.74849	5.17254	5.79781	3.95802
XLOC_044904	NCOA1;ADCY3;PTRHD1;CENPO	chr11	74383481	74567242	2.47337	1.82707	3.04434	0.814908	1.41878	0.743709	0.589576	0.687794	1.43954
XLOC_048150	NDFIP2	chr12	54893332	54930424	23.9656	19.4711	21.3448	8.82198	7.12478	9.64764	15.0157	13.7704	9.29554
XLOC_048151	NDFIP2	chr12	54934854	54986429	37.6914	30.4167	30.5996	10.2281	6.25631	8.59628	13.1865	6.60697	7.66938
XLOC_052308	NDRG3	chr13	66399494	66400337	0.0	0.0	0.0	0.661833	0.494207	0.874793	0.667413	1.15819	0.460513
XLOC_052309	NDRG3	chr13	66403483	66404032	0.0	0.0	0.0	0.661689	0.859913	0.0	0.329098	0.547789	0.482439

XLOC_052310	NDRG3	chr13	66430030	66430590	0.0	4.2957	0.0	12.0682	9.34367	7.60095	7.04873	8.53029	11.577
XLOC_083673	NDST1	chr7	63873363	63876831	0.0	0.235793	0.62715	0.0717614	0.063246	0.041002	0.0	0.0396383	0.0344757
XLOC_083674	NDST1	chr7	63891397	63938303	16.2254	14.9256	22.9484	10.2972	7.10174	6.71876	9.72538	11.6801	4.46385
XLOC_084965	NDST1	chr7	63866801	63867215	0.0	0.0	2.20117	0.0	0.0	0.0	0.0	0.0	0.0
XLOC_084966	NDST1	chr7	63867868	63868864	0.0	0.515945	0.0	0.0	0.0674417	0.0	0.0	0.0	0.0753485
XLOC_084967	NDST1	chr7	63869047	63869467	2.75534	0.0	0.0	0.0	0.0	0.0	0.0	0.0	0.0
XLOC_084968	NDST1	chr7	63869571	63870666	0.771466	0.0	0.0	0.0691522	0.0	0.0	0.0700813	0.0772545	0.202241
XLOC_084970	NDST1	chr7	63872402	63872842	0.0	0.0	0.0	0.0	0.0	0.262912	0.0	0.0	0.222295
XLOC_084972	NDST1	chr7	63890060	63891344	11.5565	20.3581	20.5948	8.46092	8.39809	10.5403	9.23717	13.7805	6.00582
XLOC_084973	NDST1	chr7	63891397	63938303	2.53349	5.29544	7.9131	2.26689	1.3683	1.30096	0.445747	0.745024	0.659946
XLOC_084974	NDST1	chr7	63891397	63938303	18.365	13.7071	21.4991	0.828855	1.36849	0.922256	2.22975	0.0	0.788663
XLOC_084975	NDST1	chr7	63891397	63938303	0.895675	1.60721	8.40691	1.28441	0.980177	1.39379	0.892952	0.358229	0.782321
XLOC_084976	NDST1	chr7	63891397	63938303	5.68793	2.83236	2.96285	2.3765	1.32318	1.56381	0.84357	0.374557	0.825002
XLOC_084977	NDST1	chr7	63891397	63938303	5.0255	4.50917	6.55179	1.87684	0.720528	0.869242	0.759943	0.921886	0.658563
XLOC_084978	NDST1	chr7	63891397	63938303	23.8224	35.6401	46.5802	6.50215	3.5242	11.9324	5.68425	5.45369	2.04581
XLOC_084979	NDST1	chr7	63891397	63938303	4.88166	7.29452	2.54358	4.37187	3.54062	3.02483	2.18264	1.93504	3.11849
XLOC_084980	NDST1	chr7	63891397	63938303	5.30649	5.95392	6.57495	1.9826	1.63056	2.16225	1.29337	1.51173	1.35409
XLOC_079597	NDST3	chr6	8209032	8236996	13.0262	17.1242	16.159	16.5812	19.5578	17.2743	19.9702	17.6276	16.3416
XLOC_080474	NDST3	chr6	8238782	8239632	0.0	0.624619	0.0	0.467964	0.570795	1.19076	1.13279	0.312717	0.729401
XLOC_079600	NDST4	chr6	11907873	11935798	4.14034	5.17483	7.77338	5.15187	5.07127	5.06979	6.93891	5.79936	5.11651
XLOC_079601	NDST4	chr6	11942944	12081197	15.6794	20.8339	17.6191	17.1746	17.8682	19.3378	25.6306	23.1172	21.5755
XLOC_080009	NDST4	chr6	11900431	11907587	185.469	193.116	109.899	395.826	390.389	418.7	411.669	409.64	521.268
XLOC_080487	NDST4	chr6	11935993	11937415	0.571956	0.513325	0.0	0.461514	0.224125	0.178367	0.521504	0.172249	0.500161
XLOC_080488	NDST4	chr6	11937689	11938883	0.697777	0.626171	0.545899	0.437861	0.10926	0.362391	0.571266	0.209816	0.487926
XLOC_080489	NDST4	chr6	11939051	11939604	0.0	1.0929	0.0	0.163752	0.283781	0.565901	0.16294	0.0	0.636789
XLOC_080490	NDST4	chr6	11940664	11941374	0.0	0.0	0.0	0.0	0.305969	0.406383	0.0	0.130233	0.228341
XLOC_080491	NDST4	chr6	11941990	11942760	0.0	2.11835	1.84672	1.05803	0.644648	0.244575	0.638774	0.235317	0.3091
XLOC_080492	NDST4	chr6	11942944	12081197	0.0	7.98462	0.0	1.8018	1.00697	0.0	0.0	0.0	1.15169
XLOC_070519	NDUFA10	chr3	119716273	119783358	33.8857	32.9488	26.4132	15.6689	17.6397	18.6385	20.568	13.4296	16.5074
XLOC_072134	NDUFA10	chr3	119714174	119715603	0.0	0.850834	1.33518	0.458974	0.847006	0.591295	0.155599	0.22841	0.696378
XLOC_072135	NDUFA10	chr3	119715681	119715986	4.88566	4.36968	3.80843	1.31116	1.85409	2.9748	0.827512	0.0	2.52807
XLOC_072136	NDUFA10	chr3	119716273	119783358	6.5965	3.3818	8.84465	1.5202	2.79808	2.05241	3.24272	1.31838	1.89279
XLOC_072137	NDUFA10	chr3	119716273	119783358	7.516	5.72287	8.55297	2.81761	3.31706	3.69086	6.34148	2.19191	4.06138

XLOC_072138	NDUFA10	chr3	119716273	119783358	6.26214	4.11874	9.79252	1.79533	3.12379	1.81499	6.42769	0.872764	2.8405
XLOC_072139	NDUFA10	chr3	119716273	119783358	5.01917	3.6022	8.63596	1.52931	3.05755	2.70582	4.72153	1.20285	3.1552
XLOC_072140	NDUFA10	chr3	119716273	119783358	11.0989	6.63645	11.5709	2.09927	3.36473	2.80868	3.88763	1.71914	5.59421
XLOC_072141	NDUFA10	chr3	119716273	119783358	7.80024	2.85129	6.10135	1.78665	2.03288	2.42808	3.14372	1.12696	1.58988
XLOC_072142	NDUFA10	chr3	119716273	119783358	4.2557	5.37605	3.70022	1.61115	2.70742	2.06575	2.33249	1.66342	2.19235
XLOC_072143	NDUFA10	chr3	119716273	119783358	3.20673	9.56967	2.50238	0.573592	3.44611	1.96837	2.2305	0.623772	2.77809
XLOC_072144	NDUFA10	chr3	119716273	119783358	5.25247	7.22481	8.21539	2.72995	3.03444	3.48402	3.32281	1.36288	3.2096
XLOC_072145	NDUFA10	chr3	119716273	119783358	17.2512	8.09515	9.6225	3.74893	5.89759	2.53207	7.1632	2.41767	3.42461
XLOC_072146	NDUFA10	chr3	119716273	119783358	0.0	3.38406	0.0	0.508242	0.857855	2.29749	0.475623	0.0	3.42173
XLOC_072147	NDUFA10	chr3	119716273	119783358	6.25688	4.76467	5.7858	1.22393	1.20364	1.95127	2.28212	1.02785	1.84043
XLOC_072148	NDUFA10	chr3	119716273	119783358	12.3119	8.58182	0.0	3.49007	3.81404	4.43848	3.45952	1.4161	3.21256
XLOC_072149	NDUFA10	chr3	119716273	119783358	8.88198	5.2969	13.8501	2.38296	1.68997	5.41827	2.64982	1.7051	2.3003
XLOC_076436	NDUFA9;AKAP3	chr5	105876560	105914757	24.9104	16.2728	18.7353	11.9572	14.447	15.4755	16.8454	8.61245	11.4912
XLOC_078540	NDUFA9;AKAP3	chr5	105876560	105914757	2.93016	2.04458	0.76392	1.6631	0.915572	0.911487	0.972294	0.975624	1.27931
XLOC_078541	NDUFA9;AKAP3	chr5	105876560	105914757	0.0	3.19328	8.35057	1.19613	1.23648	1.92071	1.17321	0.261668	0.696257
XLOC_078542	NDUFA9;AKAP3	chr5	105876560	105914757	3.66835	2.92463	2.86833	2.73888	2.95524	1.39256	2.64378	0.730641	2.66692
XLOC_083633	NDUFS7;MUM1;GAMT	chr7	45421851	45434610	25.9434	27.6875	29.8822	269.15	237.922	256.359	227.681	295.997	243.864
XLOC_062138	NECAB2;DNAAF1;HSDL1;TAF1C;SLC38A8;MBTPS1	chr18	10324810	10564142	15.4175	9.65824	7.09736	1.89528	2.00658	3.21081	1.70118	0.856283	1.95269
XLOC_062635	NECAB2;DNAAF1;HSDL1;TAF1C;SLC38A8;MBTPS1	chr18	10324810	10564142	7.66685	2.28693	2.98995	0.0	1.46471	0.781753	0.659722	0.0	0.331469
XLOC_062636	NECAB2;DNAAF1;HSDL1;TAF1C;SLC38A8;MBTPS1	chr18	10324810	10564142	0.650693	2.33578	1.52726	0.291668	0.10193	0.676071	0.0	0.0652613	0.170655

XLOC_062637	NECAB2;DNAAF1;HSDL1;TAF1C;SLC38A8;MBTPS1	chr18	10324810	10564142	0.0	1.75588	1.14802	0.131547	0.799899	0.151815	0.263415	0.145826	0.128007
XLOC_062638	NECAB2;DNAAF1;HSDL1;TAF1C;SLC38A8;MBTPS1	chr18	10324810	10564142	4.67416	1.39594	1.82526	0.836604	0.722378	0.240303	0.0	0.229634	0.203066
XLOC_062639	NECAB2;DNAAF1;HSDL1;TAF1C;SLC38A8;MBTPS1	chr18	10324810	10564142	3.65052	1.63761	2.8553	0.408967	0.427972	0.757344	0.0	0.0912382	0.318831
XLOC_062640	NECAB2;DNAAF1;HSDL1;TAF1C;SLC38A8;MBTPS1	chr18	10324810	10564142	0.0	1.48701	0.0	0.0	0.0	0.0	0.0	0.0	0.216229
XLOC_062642	NECAB2;DNAAF1;HSDL1;TAF1C;SLC38A8;MBTPS1	chr18	10324810	10564142	0.0	0.0	1.32538	0.0	0.131726	0.175065	0.0	0.0	0.147696
XLOC_062643	NECAB2;DNAAF1;HSDL1;TAF1C;SLC38A8;MBTPS1	chr18	10324810	10564142	0.0	0.379024	3.96501	0.227166	0.0	0.26244	0.228236	0.0	0.221169
XLOC_062644	NECAB2;DNAAF1;HSDL1;TAF1C;SLC38A8;MBTPS1	chr18	10324810	10564142	0.0	0.0	0.0	0.286797	0.0	0.656123	0.0	0.0	0.27781
XLOC_062645	NECAB2;DNAAF1;HSDL1;TAF1C;SLC38A8;MBTPS1	chr18	10324810	10564142	6.04586	0.0	0.0	0.0	0.456056	0.0	0.0	0.0	0.0
XLOC_062646	NECAB2;DNAAF1;HSDL1;TAF1C;SLC38A8;MBTPS1	chr18	10324810	10564142	4.1348	1.44298	3.23485	0.185332	0.107919	0.214752	0.0626977	0.0690842	0.120474

XLOC_062647	NECAB2;DNAAF1;HSDL1;TAF1C;SLC38A8;MBTPS1	chr18	10324810	10564142	0.0	1.53832	4.02208	0.0	0.0	0.0	0.0	0.0	0.0
XLOC_062648	NECAB2;DNAAF1;HSDL1;TAF1C;SLC38A8;MBTPS1	chr18	10324810	10564142	0.0	1.34878	0.0	0.0	0.344117	0.0	0.0	0.0	0.0
XLOC_086947	NEFL;NEF3	chr8	72814198	73353042	13.0183	9.68919	12.9766	9.02398	9.85342	7.58189	9.67238	7.32181	8.40571
XLOC_088148	NEFL;NEF3	chr8	72814198	73353042	11.3522	6.76812	8.84785	0.508393	2.57357	1.1489	0.0	0.0	1.46645
XLOC_088149	NEFL;NEF3	chr8	72814198	73353042	0.0	1.92053	3.34834	0.383727	1.16103	0.661942	1.1393	0.0	0.372694
XLOC_088150	NEFL;NEF3	chr8	72814198	73353042	9.66294	2.88363	7.54045	1.44043	1.23609	0.329583	2.24008	0.313222	1.95321
XLOC_088151	NEFL;NEF3	chr8	72814198	73353042	3.35513	3.00341	0.0	0.600185	1.02904	1.37195	0.0	0.325776	0.871584
XLOC_088152	NEFL;NEF3	chr8	72814198	73353042	8.47235	3.16347	3.3092	1.13761	1.96742	2.18082	2.81596	0.625952	2.76271
XLOC_088153	NEFL;NEF3	chr8	72814198	73353042	1.52433	3.18918	2.38303	0.273099	2.01539	2.36285	2.04858	0.453738	1.4612
XLOC_088154	NEFL;NEF3	chr8	72814198	73353042	2.19672	3.61261	2.57679	0.885811	1.28573	1.36566	1.68605	0.328647	1.15037
XLOC_088155	NEFL;NEF3	chr8	72814198	73353042	6.64171	2.38258	6.23116	1.30903	1.8625	0.824647	1.075	0.264231	2.20096
XLOC_088156	NEFL;NEF3	chr8	72814198	73353042	0.0	5.32923	3.48371	2.79727	2.38001	0.454353	0.380987	1.2863	1.54281
XLOC_088157	NEFL;NEF3	chr8	72814198	73353042	5.68793	3.96529	0.0	2.37656	2.94039	3.32314	2.69955	0.187274	3.465
XLOC_088158	NEFL;NEF3	chr8	72814198	73353042	4.167	3.42836	3.26063	2.14831	1.67438	1.1916	1.71141	0.889467	2.27789
XLOC_088159	NEFL;NEF3	chr8	72814198	73353042	0.0	10.1522	22.1195	2.54136	3.86035	2.29765	10.9397	3.77093	5.86582
XLOC_088160	NEFL;NEF3	chr8	72814198	73353042	1.04292	5.61347	2.44688	1.96265	3.1756	1.94575	3.48791	0.936814	3.36863
XLOC_070315	NEGR1	chr3	73638347	73708398	3.10349	0.61919	0.485849	0.59382	0.422835	0.301792	0.436862	0.584184	0.289872
XLOC_044379	NEK6	chr11	95273189	95382009	18.7384	19.6783	21.2516	17.2349	12.3913	17.0635	12.4192	22.3367	13.9865
XLOC_044380	NEK6	chr11	95384672	95391776	29.5425	25.2872	23.0859	29.2025	19.6334	22.7884	29.3575	39.7738	22.5627
XLOC_045020	NEK6	chr11	95273189	95382009	29.0991	38.0581	34.3409	59.1483	56.5787	59.5833	63.221	71.2565	62.4231
XLOC_047057	NEK6	chr11	95273189	95382009	5.86814	0.0	0.0	3.93671	2.93379	2.40498	1.79408	2.57627	1.27204
XLOC_047058	NEK6	chr11	95273189	95382009	0.0	1.74567	4.56361	4.19558	4.42071	4.14525	3.91638	6.65877	6.04949
XLOC_047059	NEK6	chr11	95273189	95382009	0.0	1.39408	1.82294	0.939976	1.36374	0.965778	1.15631	1.51012	1.01711
XLOC_041702	NEK9;ACYP1	chr10	86705766	86759206	2.00313	3.20016	3.39695	2.47393	1.87981	2.67318	1.79497	1.9114	2.02509
XLOC_043265	NEK9;ACYP1	chr10	86705766	86759206	0.0	0.0	0.0	0.593114	0.170844	0.227384	0.0	0.0	0.385728
XLOC_043266	NEK9;ACYP1	chr10	86705766	86759206	0.0	0.748644	0.0	0.224422	0.0	0.257653	0.0	0.245832	0.21969

XLOC_043267	NEK9;ACYP1	chr10	86705766	86759206	30.9651	22.4905	23.5285	170.166	150.773	150.442	258.407	231.443	294.43
XLOC_076298	NELL2	chr5	35757924	35883738	0.525793	1.57394	1.61819	3.89222	8.36724	3.65843	6.94234	5.02254	16.0409
XLOC_076299	NELL2	chr5	35893362	36002227	8.33356	4.95245	4.44564	2.78892	1.72199	1.94302	2.03239	2.9219	2.56316
XLOC_076819	NELL2	chr5	35651943	35679489	9.42607	4.09453	7.13948	0.109076	0.0238817	0.0316611	0.167181	0.0306252	0.0532319
XLOC_076820	NELL2	chr5	35697069	35740139	4.83351	0.718518	1.53589	6.19799	5.73394	5.02622	4.76212	6.28121	6.51914
XLOC_076821	NELL2	chr5	36018217	36078040	7.63469	5.5333	3.96593	2.2282	1.93456	2.15909	1.77544	0.827645	2.50609
XLOC_077720	NELL2	chr5	35697069	35740139	0.0	0.0	0.0	0.790535	1.13297	0.0	0.514574	0.287378	0.510867
XLOC_077721	NELL2	chr5	35757924	35883738	0.0	0.0	0.0	1.64168	3.27163	1.13464	3.26679	2.17486	4.46876
XLOC_077722	NELL2	chr5	35757924	35883738	0.0	0.0	0.0	0.0	0.464003	0.0	0.268737	0.197764	0.864494
XLOC_077723	NELL2	chr5	35757924	35883738	0.0	0.0	0.578237	0.0	0.80989	0.153509	0.201556	0.22214	0.581371
XLOC_077724	NELL2	chr5	35883804	35884829	0.0	0.0	0.0	0.448366	1.95606	1.0383	1.51299	0.417124	3.64186
XLOC_077725	NELL2	chr5	36002358	36003488	6.6933	7.11841	1.74539	1.73329	2.21175	1.85343	1.48706	2.75648	2.33978
XLOC_077726	NELL2	chr5	36003595	36004168	3.48162	6.24227	4.08122	1.8706	1.4867	2.69476	2.48551	2.58406	3.48638
XLOC_077727	NELL2	chr5	36017248	36018086	7.43992	6.35623	2.49356	0.761933	2.24013	1.32177	0.864284	1.16659	2.04109
XLOC_042646	NEMF	chr10	42831567	42831837	0.0	17.0504	14.8592	5.12707	9.0938	10.9098	5.8097	2.39945	10.9354
XLOC_042647	NEMF	chr10	42831928	42832318	15.5519	11.1394	9.70962	7.51122	8.84026	8.27777	8.93906	8.17686	8.35593
XLOC_042648	NEMF	chr10	42832561	42832984	2.72423	2.43992	2.12681	0.487438	0.419753	0.0	0.0	0.53287	0.709162
XLOC_042649	NEMF	chr10	42833712	42835949	1.39115	0.936616	2.4497	0.249509	0.327672	0.253426	0.222907	0.280079	0.182629
XLOC_041519	NEMF;KLHDC1;<br>KLHDC2	chr10	42761662	42831428	32.4751	37.5752	42.6992	9.35414	9.30469	10.4622	10.7321	12.6603	10.6901
XLOC_057950	NENF;TMEM206	chr16	72958883	73046291	10.7508	13.4735	10.8525	2.15282	1.80624	2.07437	1.87441	3.5112	2.36846
XLOC_055456	NEU3;SLCO2B1	chr15	55014986	55212232	5.36632	6.15821	9.21005	12.9705	15.7523	15.7629	12.6906	11.7997	16.3787
XLOC_055767	NEU3;SLCO2B1	chr15	55014986	55212232	26.2183	34.9354	32.133	33.0695	29.3478	28.4445	35.1901	37.2791	34.0763
XLOC_056620	NEU3;SLCO2B1	chr15	55014986	55212232	2.28775	4.33343	5.96509	0.956911	0.537011	0.791713	0.554241	0.610935	0.466438
XLOC_062400	NFATC3	chr18	35651251	35662204	0.843818	5.12139	6.12997	1.07005	1.22647	1.81909	0.830247	0.687984	1.42905
XLOC_062401	NFATC3	chr18	35674597	35703122	8.22681	11.8725	9.0884	7.20294	8.42561	7.0466	7.33666	6.90583	9.22839
XLOC_062402	NFATC3	chr18	35713085	35716078	5.25545	1.96387	4.10888	1.29475	0.204713	0.951644	0.472658	1.82978	2.29167
XLOC_062403	NFATC3	chr18	35717150	35722797	4.09208	3.66126	9.5735	0.731813	0.31212	1.66686	0.350645	1.18253	1.4143
XLOC_062922	NFATC3	chr18	35650273	35651096	0.0	1.62484	0.849902	0.389544	0.169644	0.112613	0.0981529	0.216824	0.18972
XLOC_062923	NFATC3	chr18	35670959	35672396	4.522	4.39669	4.86508	2.78732	3.49984	2.76177	2.57727	3.17789	4.10277

XLOC_062924	NFATC3	chr18	35673116	35673330	23.4817	17.5626	45.9071	9.60895	9.55664	7.05787	17.7593	17.2137	8.06601
XLOC_062925	NFATC3	chr18	35673532	35673703	0.0	0.0	0.0	20.34	23.361	21.528	14.3256	16.6351	16.2548
XLOC_062926	NFATC3	chr18	35673867	35674226	7.1747	13.9132	8.39557	6.09504	4.66832	5.49373	4.33896	5.55887	9.00204
XLOC_066957	NFE2L2	chr2	19658633	19660919	2.37847	3.55857	2.1274	3.16897	2.02761	3.07736	2.98709	2.73658	1.7843
XLOC_073210	NFE2L3	chr4	70221140	70228715	1.09497	3.1656	1.14258	3.30579	3.81079	2.77329	3.57467	3.78356	3.64134
XLOC_073211	NFE2L3	chr4	70230639	70410437	13.0877	14.8451	12.6017	20.8376	23.1135	22.4776	25.5124	22.7589	23.8457
XLOC_074418	NFE2L3	chr4	70216382	70217255	0.0	0.604559	1.58114	0.815285	1.26307	1.04793	1.64558	1.91766	1.41208
XLOC_074419	NFE2L3	chr4	70218276	70219316	1.6391	3.18656	1.92325	3.0118	3.33326	3.23236	3.12408	3.85499	3.07892
XLOC_074420	NFE2L3	chr4	70219498	70221075	2.038	2.13406	0.797363	2.05572	2.35707	1.37758	2.93012	2.09863	1.60433
XLOC_069951	NFIA	chr3	84814555	84979806	12.3327	14.7736	12.7094	12.3095	9.26911	10.2211	13.98	15.484	12.3161
XLOC_071494	NFIA	chr3	84814555	84979806	8.94077	3.74019	15.3707	1.60115	2.35925	3.13621	4.30466	0.884273	2.33516
XLOC_071495	NFIA	chr3	84814555	84979806	7.22566	1.29588	6.77817	1.81225	1.91192	1.3447	1.55589	0.861158	2.26748
XLOC_071496	NFIA	chr3	84981473	84982546	0.0	0.945162	1.23599	0.920565	0.803466	0.574076	0.430445	0.316372	0.621262
XLOC_052291	NFS1	chr13	65546136	65546348	0.0	0.0	0.0	14.3021	8.04239	9.68494	6.72264	12.1657	13.4931
XLOC_069852	NGF	chr3	27938621	28133346	0.623958	0.700284	0.854754	1.20323	0.478501	1.02422	0.546689	0.61378	0.437116
XLOC_070742	NGF	chr3	27938621	28133346	0.0	0.0	0.0	0.0	0.0	0.170323	0.0	0.163415	0.0
XLOC_070743	NGF	chr3	27938621	28133346	0.0	0.0	0.0	0.0	0.147019	0.0	0.0	0.0	0.0
XLOC_070744	NGF	chr3	27938621	28133346	0.0	0.0	0.0	0.0	0.0	0.0	0.0	0.113283	0.0
XLOC_070745	NGF	chr3	27938621	28133346	0.0	0.0	0.0	0.247436	0.0	0.0	0.0	0.0	0.0
XLOC_070746	NGF	chr3	27938621	28133346	0.0	0.414588	1.08426	0.0	0.0	0.143445	0.0	0.0	0.0
XLOC_070747	NGF	chr3	27938621	28133346	0.0	0.0	0.0	0.0	0.154185	0.0	0.0	0.0	0.346225
XLOC_070749	NGF	chr3	27938621	28133346	0.0	0.0	0.0	0.12145	0.0	0.0	0.0	0.0	0.0
XLOC_065875	NIPAL3	chr2	129065962	129095211	4.14327	3.32828	2.04826	5.18293	5.36455	4.78826	5.41393	6.42922	6.08814
XLOC_061880	NIPSNAP1	chr17	70871741	70873131	0.0	0.526646	0.459136	3.26182	1.83933	2.9887	1.0163	3.88716	1.53936
XLOC_061881	NIPSNAP1	chr17	70873258	70873831	0.0	0.0	0.0	3.7412	1.89216	4.13196	1.55344	4.1345	2.42531
XLOC_061882	NIPSNAP1	chr17	70876175	70876311	306.738	354.653	253.475	2432.37	1968.72	2579.97	1207.14	1669.96	2171.18
XLOC_089320	NKAIN2	chr9	26999626	27030091	30.9034	34.162	18.9537	70.9379	66.1716	72.6382	69.435	72.6503	83.3162
XLOC_089321	NKAIN2	chr9	27030142	27095213	20.9091	27.7233	26.9314	31.1811	31.072	32.0256	35.861	33.3963	38.3507
XLOC_089322	NKAIN2	chr9	27299201	27349330	7.299	7.40971	9.43408	7.29095	7.55681	7.30688	5.36431	7.3368	4.19804
XLOC_089800	NKAIN2	chr9	26763280	26791837	1.86978	1.75854	2.49633	0.718689	1.75555	0.445122	0.661353	0.376778	1.16924
XLOC_089801	NKAIN2	chr9	26818096	26845685	4.14464	14.5245	8.8021	0.0	0.0	0.0	0.0	0.0	0.0
XLOC_089802	NKAIN2	chr9	26850101	26860664	3.84382	8.04392	6.01065	0.0	0.0	0.0	0.0	0.0	0.0
XLOC_089803	NKAIN2	chr9	26974891	26999555	2.7779	4.83101	3.54497	12.5461	12.358	13.0311	12.2028	12.1599	15.0338

XLOC_089804	NKAIN2	chr9	26999626	27030091	31.0347	37.7105	32.3514	21.9281	19.5186	20.5042	22.2053	24.969	19.3446
XLOC_089805	NKAIN2	chr9	27096263	27155436	14.7834	18.8179	16.3485	2.91384	2.80179	2.79331	3.83555	4.53541	4.19284
XLOC_090454	NKAIN2	chr9	26959012	26959252	0.0	5.06284	6.61755	0.0	0.0	0.0	4.14287	2.3688	2.91497
XLOC_090455	NKAIN2	chr9	26961508	26961694	0.0	57.136	0.0	19.3717	26.3329	20.8728	44.0552	25.5207	16.3309
XLOC_090456	NKAIN2	chr9	26961856	26962036	43.156	52.138	102.244	54.4831	45.5963	51.8416	44.8355	75.1148	48.3845
XLOC_090457	NKAIN2	chr9	26974152	26974663	0.0	1.22251	0.0	0.549508	0.792381	0.421532	0.907921	1.61393	0.88987
XLOC_090458	NKAIN2	chr9	27030142	27095213	8.21286	0.0	0.0	4.43634	7.36724	3.30427	8.04022	7.65644	9.87846
XLOC_090459	NKAIN2	chr9	27095266	27095567	0.0	1.49634	0.0	0.449061	1.52265	0.0	1.27282	0.958239	2.5965
XLOC_090460	NKAIN2	chr9	27095725	27096117	3.08391	1.84081	4.81359	0.551659	0.710816	0.0	1.88088	1.5018	1.33637
XLOC_090461	NKAIN2	chr9	27096263	27155436	5.30869	2.37767	0.0	2.37475	0.613796	0.817214	3.26203	1.55885	3.22525
XLOC_076418	NKG2C;KLRC1; KLRK1	chr5	99958345	100068869	25.3406	20.662	15.3809	59.4631	51.3558	54.8427	70.8314	77.7957	70.2781
XLOC_076928	NKG2C;KLRC1; KLRK1	chr5	99958345	100068869	206.146	290.059	236.979	396.253	375.737	437.972	675.263	556.471	452.789
XLOC_078405	NKG2C;KLRC1; KLRK1	chr5	99958345	100068869	7.39337	7.35922	11.5474	10.586	13.6977	10.1283	14.3265	13.7813	10.2742
XLOC_078406	NKG2C;KLRC1; KLRK1	chr5	99958345	100068869	4.88354	3.21841	4.18497	3.46735	4.63884	3.76252	4.1224	4.27101	7.40093
XLOC_078407	NKG2C;KLRC1; KLRK1	chr5	99958345	100068869	13.0316	10.1334	12.2321	11.1352	13.7887	14.6934	13.4712	9.47175	12.0666
XLOC_078408	NKG2C;KLRC1; KLRK1	chr5	99958345	100068869	0.583022	0.523251	0.912451	2.35238	1.59909	2.48469	2.33856	2.98461	1.27465
XLOC_062239	NKPD1;PPP1R37	chr18	53176315	53220786	15.86	19.924	14.0786	67.4193	66.8218	69.2406	71.0319	66.6509	82.5234
XLOC_086471	NKX3-1	chr8	71658604	71690762	106.345	47.9362	67.4519	21.4375	22.2501	18.6638	15.9678	17.6547	13.0514
XLOC_086936	NKX3-1	chr8	71658604	71690762	0.0	0.0	0.0	7.50025	9.64417	7.33947	6.4276	5.86386	6.91563
XLOC_079864	NKX3-2	chr6	113623990	113630153	5.79883	12.9137	10.082	9.29972	10.7493	9.90819	12.9072	11.6321	12.9556
XLOC_037998	NLGN1	chr1	93620024	93635763	1.37807	1.50333	1.88579	0.633826	0.465649	0.307393	0.658446	0.240605	0.411306
XLOC_037999	NLGN1	chr1	93685752	93695918	13.1973	14.251	14.2003	4.69199	4.29001	4.98988	5.53263	4.18748	3.8283
XLOC_038000	NLGN1	chr1	93754910	93789636	24.076	24.9646	22.5223	20.2274	18.3907	21.0152	19.18	23.1301	22.3761
XLOC_038001	NLGN1	chr1	93803467	93819915	6.71537	7.23592	5.85342	25.1543	22.1373	22.9881	24.5627	30.2305	22.8009
XLOC_038002	NLGN1	chr1	93825151	93985560	1.12877	2.41722	1.27922	3.73922	3.7486	3.81533	3.1142	4.12965	2.06152
XLOC_038483	NLGN1	chr1	93620024	93635763	32.3624	39.1161	30.6645	27.2179	25.3644	24.1689	21.8046	21.7503	26.6482
XLOC_038484	NLGN1	chr1	93705396	93754789	19.8963	25.1359	22.3418	25.4757	24.7681	26.2177	27.4949	24.3795	25.4848
XLOC_038485	NLGN1	chr1	93792478	93802027	30.6803	32.3358	19.2776	168.146	149.548	163.097	164.123	204.594	176.187
XLOC_039973	NLGN1	chr1	93790100	93790557	4.83019	0.721215	3.7721	1.29671	0.932531	0.992834	1.91616	0.474128	1.6783

XLOC_039974	NLGN1	chr1	93820231	93820389	0.0	12.0771	0.0	26.7698	28.3726	39.6667	20.7451	27.5522	20.5917
XLOC_039975	NLGN1	chr1	93820497	93822348	4.27062	5.49453	3.34203	15.9305	14.4757	14.3077	15.3445	20.4432	15.5066
XLOC_039976	NLGN1	chr1	93825151	93985560	0.0	0.778563	1.35748	0.855509	0.407061	0.630253	0.5508	1.21509	0.151598
XLOC_039978	NLGN1	chr1	93825151	93985560	0.0	0.0	0.0	0.0	0.0	0.0	0.0	0.0	0.0799088
XLOC_039980	NLGN1	chr1	93825151	93985560	0.0	0.0	8.67651	4.0305	3.29105	3.33832	0.887044	4.07988	10.4863
XLOC_039981	NLGN1	chr1	93825151	93985560	0.970171	0.870386	0.758785	0.608619	0.985269	1.30781	1.31712	1.25991	0.847156
XLOC_039982	NLGN1	chr1	93825151	93985560	1.21457	0.726261	2.84906	0.217642	0.662874	0.377257	0.437735	0.604839	1.37751
XLOC_039983	NLGN1	chr1	93985692	93986170	0.0	0.674062	0.0	1.41392	2.26854	2.55386	1.39666	3.55122	2.35382
XLOC_039984	NLGN1	chr1	94467508	94469703	8.51898	6.37278	6.38935	10.759	10.8404	9.23709	11.0811	11.4688	11.3697
XLOC_062266	NLRP12	chr18	61105817	61130323	25.2332	1.88779	6.17188	0.165013	0.123907	0.383264	0.0241048	0.079461	0.0920448
XLOC_057899	NME7	chr16	37776993	37836494	37.8446	14.386	20.8749	16.7377	15.5462	15.2858	22.8478	26.8298	30.2375
XLOC_058124	NME7	chr16	37647240	37654461	5.21741	2.33954	2.03952	8.98499	9.54101	9.62443	14.2755	16.8651	17.9698
XLOC_058925	NME7	chr16	37647240	37654461	0.0	2.73126	0.0	1.2289	0.348278	0.465386	2.3376	1.31708	3.95265
XLOC_058926	NME7	chr16	37647240	37654461	2.34638	0.350769	0.917375	1.26141	0.640505	0.1215	1.26944	1.16906	0.716574
XLOC_058927	NME7	chr16	37647240	37654461	3.28192	0.981591	3.85087	1.10313	1.28347	0.93674	1.78718	1.8886	1.79209
XLOC_058928	NME7	chr16	37647240	37654461	4.64415	1.38699	1.81357	2.49373	1.25617	2.14904	2.66561	5.93283	2.2195
XLOC_058929	NME7	chr16	37647240	37654461	0.0	0.893474	2.3368	1.67351	1.11042	0.852979	1.28951	1.87011	1.23997
XLOC_038041	NME9	chr1	131867167	131873842	2.34724	4.69864	6.3577	3.83028	3.88811	2.40851	5.2831	4.93273	4.9325
XLOC_038534	NME9	chr1	131867167	131873842	0.0	0.0	0.0	0.702402	0.203625	0.405654	1.17539	0.780027	1.3676
XLOC_057947	NMNAT2	chr16	65815183	65866488	5.92931	3.28835	4.7157	0.137884	0.082702	0.197319	0.100981	0.199539	0.115414
XLOC_059241	NMNAT2	chr16	65793761	65793963	14.1217	8.46796	0.0	10.3451	5.19828	4.2396	5.55664	10.2637	8.49312
XLOC_059242	NMNAT2	chr16	65815183	65866488	1.01796	0.608809	0.796126	0.0912241	0.0794931	0.105526	0.0920527	0.0	0.0888736
XLOC_059243	NMNAT2	chr16	65815183	65866488	2.30726	0.68908	0.0	0.412976	0.356658	0.23728	0.0	0.0	0.0
XLOC_059244	NMNAT2	chr16	65815183	65866488	2.31464	1.38256	0.0	0.207147	0.0	0.0	0.0	0.0	0.0
XLOC_050794	NMT2;ACBD7;RPP38	chr13	29906780	29987082	5.78571	7.47365	6.81443	13.4148	9.85421	11.6074	10.9248	15.7301	9.06721
XLOC_051500	NMT2;ACBD7;RPP38	chr13	29906780	29987082	0.0	1.31944	3.45033	1.9768	1.36671	1.59091	1.95427	3.69466	1.72793
XLOC_051501	NMT2;ACBD7;RPP38	chr13	29906780	29987082	0.0	2.04002	0.0	0.611394	2.6199	2.09586	0.592312	0.995047	1.18379
XLOC_051502	NMT2;ACBD7;RPP38	chr13	29906780	29987082	2.44144	1.45988	3.81796	0.765594	0.761368	1.26384	0.659853	0.364713	0.212993
XLOC_051503	NMT2;ACBD7;RPP38	chr13	29906780	29987082	0.0	0.602806	1.57629	0.36124	0.156301	0.83145	0.358288	0.795985	1.05306
XLOC_051504	NMT2;ACBD7;RPP38	chr13	29906780	29987082	0.0	1.87776	4.91069	1.82875	1.83168	2.10943	0.984188	1.40181	1.36852

XLOC_051505	NMT2;ACBD7;RPP38	chr13	29906780	29987082	7.9581	9.49621	6.20618	3.57994	5.95438	2.40238	2.6028	0.0	1.36761
XLOC_051506	NMT2;ACBD7;RPP38	chr13	29906780	29987082	2.71052	1.62128	7.77366	1.94344	2.25984	2.43691	1.80126	1.26463	1.42045
XLOC_051507	NMT2;ACBD7;RPP38	chr13	29906780	29987082	1.4058	2.52137	2.198	2.26672	1.96989	2.1807	1.2622	2.235	1.83841
XLOC_051508	NMT2;ACBD7;RPP38	chr13	29906780	29987082	0.0	1.4567	0.0	0.874108	2.22491	1.9832	1.24127	1.40083	0.842691
XLOC_051509	NMT2;ACBD7;RPP38	chr13	29906780	29987082	2.54775	2.85806	3.98667	2.79797	2.14533	3.11064	2.66824	1.91665	1.44782
XLOC_051510	NMT2;ACBD7;RPP38	chr13	29906780	29987082	4.44441	4.64617	3.47149	2.78449	2.23431	2.05786	3.9316	1.74906	0.965814
XLOC_051511	NMT2;ACBD7;RPP38	chr13	29906780	29987082	3.97487	4.16271	2.48855	1.74654	1.87173	2.10959	2.72705	1.59953	1.3217
XLOC_051512	NMT2;ACBD7;RPP38	chr13	29906780	29987082	5.96466	4.45091	4.65548	5.06845	2.75171	3.66587	4.68543	0.58161	2.58558
XLOC_051513	NMT2;ACBD7;RPP38	chr13	29906780	29987082	4.58129	2.05471	0.895612	2.15509	2.32302	1.77951	2.37651	1.25584	2.09887
XLOC_051514	NMT2;ACBD7;RPP38	chr13	29906780	29987082	11.4399	13.6409	8.91608	6.1464	6.48212	4.6298	3.83224	2.71332	3.44792
XLOC_051515	NMT2;ACBD7;RPP38	chr13	29906780	29987082	0.0	1.93107	0.0	3.47223	2.48295	1.32375	3.65514	2.20198	1.68158
XLOC_051516	NMT2;ACBD7;RPP38	chr13	29906780	29987082	2.36557	1.76821	3.69947	1.80159	2.58277	1.83729	4.2653	1.17848	1.96082
XLOC_051517	NMT2;ACBD7;RPP38	chr13	29906780	29987082	6.77866	3.64751	5.29955	3.52205	4.01215	3.08539	4.14244	3.50466	1.89147
XLOC_051518	NMT2;ACBD7;RPP38	chr13	29906780	29987082	4.03164	2.94866	2.8044	3.93642	3.58452	4.334	4.21673	2.387	2.23387
XLOC_051519	NMT2;ACBD7;RPP38	chr13	29906780	29987082	7.13193	6.01248	6.08715	4.24309	4.72266	5.32189	5.07681	3.25124	3.17416
XLOC_051520	NMT2;ACBD7;RPP38	chr13	29906780	29987082	0.0	2.64858	6.92506	10.3261	9.12582	9.93349	6.43529	7.67293	3.83383
XLOC_051521	NMT2;ACBD7;RPP38	chr13	29906780	29987082	0.0	7.12837	46.5802	15.1717	9.69137	14.3189	15.158	14.1794	13.2977
XLOC_051522	NMT2;ACBD7;RPP38	chr13	29906780	29987082	3.16399	4.10192	4.5389	5.24816	4.34097	3.89309	4.71592	3.54872	3.13625
XLOC_083041	NMUR2	chr7	65644910	65701923	20.0607	21.0206	23.1384	23.5299	27.6943	22.3955	22.2966	19.3942	32.3544
XLOC_084992	NMUR2	chr7	65644910	65701923	0.0	3.24269	0.0	4.21212	3.32736	3.69846	3.44119	4.20912	6.58307
XLOC_044981	NOL10	chr11	86923744	86993757	6.87466	8.00182	11.0251	3.15307	3.10665	3.44673	3.48891	2.19739	6.88337
XLOC_044982	NOL10	chr11	87034793	87074104	15.8421	19.4038	13.7259	1.41138	2.67329	1.39506	1.83576	1.1789	2.73824
XLOC_046900	NOL10	chr11	87033851	87034686	7.47287	9.89574	12.523	0.66964	2.3333	1.10632	1.92897	0.745617	2.42284
XLOC_086992	NOL8;IARS	chr8	85325364	85360562	0.395315	0.118284	0.0	0.0354489	0.0	0.0822777	0.0	0.0397707	0.0

XLOC_065746	NOP58	chr2	91202730	91236999	3.42155	2.97029	2.11363	2.63618	2.6253	2.20282	4.19133	2.77466	2.49512
XLOC_066404	NOP58	chr2	91202730	91236999	4.89209	8.58036	5.81592	6.28315	10.2821	8.00034	6.06165	5.93344	10.6139
XLOC_068101	NOP58	chr2	91202730	91236999	0.0	4.39664	2.29941	1.31756	3.17203	4.52736	1.28644	2.58639	2.55434
XLOC_062245	NOSIP;PRRG2;RRAS	chr18	56432101	56503855	3.98548	4.00388	5.79309	2.55307	1.94552	2.87563	0.939462	1.46264	0.822375
XLOC_053285	NOV	chr14	46882902	47104397	16.972	17.0212	25.4631	0.34437	0.452998	0.499228	0.25917	0.134659	0.178354
XLOC_054282	NOV	chr14	46882902	47104397	6.16857	4.61146	12.0606	0.184262	0.160555	0.106569	0.0929513	0.0	0.0
XLOC_054283	NOV	chr14	46882902	47104397	12.8839	1.92242	7.54041	0.288069	0.247218	0.0	0.0	0.0	0.0
XLOC_054284	NOV	chr14	46882902	47104397	1.37637	4.93723	6.45613	0.0	0.0	0.0	0.0	0.0	0.0
XLOC_054285	NOV	chr14	46882902	47104397	6.3239	6.61503	7.41426	0.141594	0.0	0.163318	0.141494	0.470275	0.137748
XLOC_054286	NOV	chr14	46882902	47104397	0.0	2.2081	1.44365	0.0	0.0	0.190539	0.0	0.0	0.160817
XLOC_054287	NOV	chr14	46882902	47104397	6.56796	3.48416	5.60787	0.0267739	0.117245	0.0310871	0.0547205	0.0	0.104533
XLOC_054288	NOV	chr14	46882902	47104397	2.44556	2.19347	6.69272	0.0	0.0953304	0.126596	0.0	0.0	0.0
XLOC_054289	NOV	chr14	46882902	47104397	0.0	0.0	7.25168	0.0	0.0	0.0	0.0	0.0	0.0
XLOC_054290	NOV	chr14	46882902	47104397	1.99934	2.69046	5.47284	0.0	0.0	0.0	0.0	0.0	0.0
XLOC_054291	NOV	chr14	46882902	47104397	13.0674	6.641	7.15161	0.0	0.0	0.0	0.0	0.0	0.0
XLOC_054292	NOV	chr14	46882902	47104397	5.33807	1.59506	8.34285	0.0	0.0	0.367169	0.0	0.0	0.0
XLOC_054293	NOV	chr14	46882902	47104397	3.96737	2.37327	6.20704	0.0	0.0620746	0.0	0.0	0.0	0.0
XLOC_054294	NOV	chr14	46882902	47104397	2.72413	2.71564	1.42048	0.0	0.0	0.0	0.0	0.0	0.0
XLOC_054295	NOV	chr14	46882902	47104397	0.0	0.436314	2.28215	0.0	0.0	0.150905	0.130931	0.0	0.0
XLOC_054296	NOV	chr14	46882902	47104397	1.78252	1.86584	2.7885	0.0	0.0	0.0	0.0	0.0	0.0
XLOC_054297	NOV	chr14	46882902	47104397	0.0	0.621378	3.25032	0.0	0.0	0.0	0.0	0.0	0.0
XLOC_054298	NOV	chr14	46882902	47104397	0.0	1.05818	2.76736	0.0	0.0	0.0	0.0	0.0	0.0
XLOC_054299	NOV	chr14	46882902	47104397	1.52826	1.37134	1.19554	0.0	0.0	0.0	0.0	0.0	0.0
XLOC_054300	NOV	chr14	46882902	47104397	1.34722	1.20926	2.45994	0.0402672	0.0352293	0.0	0.0	0.0	0.0
XLOC_054301	NOV	chr14	46882902	47104397	2.73195	2.04166	4.27162	0.122366	0.0	0.141296	0.0	0.0	0.0
XLOC_054302	NOV	chr14	46882902	47104397	1.50529	2.24958	2.35329	0.0	0.0	0.0	0.0	0.0	0.0
XLOC_062243	NOVA2	chr18	53862532	53917134	27.4415	17.4208	22.445	7.50735	7.04205	6.65891	11.2371	5.96656	8.72606
XLOC_063243	NOVA2	chr18	53862532	53917134	8.16501	6.50875	8.51113	1.21906	1.90756	1.26692	1.71197	0.947053	0.593297
XLOC_043960	NPAS2	chr11	5955703	5967809	29.8143	24.5122	26.3988	32.8417	31.1991	28.9752	33.1178	38.1892	32.7144
XLOC_044645	NPAS2	chr11	5871779	5878245	13.5452	15.4501	19.4145	64.4501	66.1592	67.7683	72.3066	83.7273	69.4329
XLOC_044646	NPAS2	chr11	5896637	5899050	1.03397	3.31932	17.353	14.4313	13.9905	19.5831	7.88841	11.8477	20.222
XLOC_044647	NPAS2	chr11	5900867	5903186	0.0	0.0	0.0	0.29193	0.663114	1.21802	0.415067	0.587837	0.910976

XLOC_050881	NPEPL1	chr13	58176060	58215645	34.0055	37.0168	37.8913	72.8168	59.6057	74.9701	74.1778	90.317	55.6361
XLOC_051932	NPEPL1	chr13	58216292	58217251	0.90238	0.539743	0.705816	0.323503	0.211598	0.187222	0.49065	0.451095	0.0788157
XLOC_040484	NPHP3	chr1	137901437	137903261	0.433982	1.42834	2.71693	0.0	0.0	0.0451519	0.0	0.0436399	0.0
XLOC_040485	NPHP3	chr1	137904286	137905381	0.771466	1.61525	1.81051	0.0	0.0603637	0.0	0.0	0.0	0.0
XLOC_040486	NPHP3	chr1	137905528	137906296	2.36945	1.06264	0.0	0.0	0.0	0.122685	0.106803	0.0	0.0
XLOC_040487	NPHP3	chr1	137906922	137907544	3.11429	1.39617	0.0	0.0	0.0	0.0	0.0	0.0	0.0
XLOC_040488	NPHP3	chr1	137907637	137909986	0.660027	0.790012	1.03313	0.0	0.0	0.0	0.0	0.0	0.0
XLOC_040489	NPHP3	chr1	137910355	137911010	0.0	0.434562	2.27299	0.0	0.0	0.0	0.0	0.0	0.0
XLOC_040490	NPHP3	chr1	137913746	137914110	0.0	1.0445	0.0	0.0	0.0	0.0	0.0	0.0	0.0
XLOC_040491	NPHP3	chr1	137915006	137916077	0.791736	0.710422	0.0	0.0709676	0.0	0.0	0.0	0.0	0.0
XLOC_040492	NPHP3	chr1	137916563	137917483	0.948538	0.567326	0.741883	0.0	0.0	0.0	0.0858846	0.0	0.0
XLOC_040493	NPHP3	chr1	137917777	137918544	0.0	1.41917	0.0	0.0	0.0	0.0	0.0	0.0	0.0
XLOC_040494	NPHP3	chr1	137918681	137919487	1.1153	0.333478	1.74431	0.0	0.0	0.0	0.0	0.0	0.0
XLOC_040495	NPHP3	chr1	137923444	137924521	0.786569	0.470527	0.0	0.0	0.0	0.0	0.0	0.0	0.0
XLOC_040496	NPHP3	chr1	137931984	137933322	0.0	0.916385	2.39674	0.0	0.0	0.0	0.0	0.0	0.0
XLOC_040497	NPHP3	chr1	137933492	137933907	2.80879	0.0	2.19265	0.0	0.0	0.0	0.0	0.0	0.0
XLOC_040498	NPHP3	chr1	137933980	137934477	6.3917	2.54566	3.32863	0.0	0.0	0.0	0.0	0.0	0.0
XLOC_040499	NPHP3	chr1	137934724	137934971	0.0	2.34952	6.14221	0.0	0.0	0.0	0.0	0.0	0.0
XLOC_040500	NPHP3	chr1	137935369	137936880	1.06869	0.63945	0.836224	0.0	0.0	0.0	0.0	0.0	0.0467336
XLOC_040501	NPHP3	chr1	137937024	137941165	2.17863	0.923692	0.994795	0.0	0.0	0.0189187	0.0	0.0	0.0476971
XLOC_040502	NPHP3	chr1	137941270	137943219	0.8074	0.0	0.0	0.0	0.0	0.0	0.0	0.0	0.0
XLOC_040503	NPHP3	chr1	137943290	137943880	0.0	0.999546	0.0	0.0	0.0	0.0	0.0	0.0	0.291318
XLOC_040504	NPHP3	chr1	137944229	137945501	0.648939	1.35887	1.52315	0.0	0.0	0.0	0.0	0.0	0.0
XLOC_040505	NPHP3	chr1	137946030	137946384	0.0	2.19455	2.8692	0.0	0.0	0.0	0.0	0.0	0.0
XLOC_040506	NPHP3	chr1	137947138	137947390	0.0	4.46791	5.84022	0.0	0.0	0.0	0.0	0.0	0.0
XLOC_040507	NPHP3	chr1	137947960	137948813	0.0	0.0	0.0	0.0931896	0.0	0.107787	0.0	0.0	0.0
XLOC_040508	NPHP3	chr1	137949140	137950433	2.54775	0.571609	0.996667	0.0	0.0	0.0661838	0.0	0.0	0.0
XLOC_040509	NPHP3	chr1	137950602	137952337	1.3754	1.64606	0.717536	0.0	0.0	0.0	0.0	0.0	0.0
XLOC_040510	NPHP3	chr1	137952441	137953850	1.15579	0.518655	1.35651	0.0	0.0	0.0	0.0	0.0	0.0
XLOC_040511	NPHP3	chr1	137953953	137955029	0.787426	0.471039	1.23195	0.0705816	0.0	0.0	0.0	0.0	0.0
XLOC_040512	NPHP3	chr1	137955180	137955544	0.0	1.0445	0.0	0.0	0.0	0.0	0.0	0.0	0.0
XLOC_040513	NPHP3	chr1	137955686	137956543	3.10192	0.618374	0.0	0.0	0.0	0.107174	0.0	0.0	0.0
XLOC_040514	NPHP3	chr1	137956597	137957323	0.0	0.380352	0.0	0.0	0.0	0.0	0.0	0.0	0.110972

XLOC_062438	NPHS1	chr18	46712875	46728819	3.1969	2.97748	1.57332	4.55836	3.05817	4.0947	2.67688	2.15926	3.79467
XLOC_062439	NPHS1	chr18	46733740	46742984	22.7804	30.6473	27.8898	24.0633	28.0135	25.9794	26.2605	23.0443	35.3262
XLOC_063108	NPHS1	chr18	46729566	46729911	0.0	9.19604	15.0288	0.0	0.588896	1.57163	4.97252	0.744312	1.66603
XLOC_063109	NPHS1	chr18	46730527	46731468	2.76934	0.828199	4.3321	0.910054	1.08212	0.765994	1.17071	0.461341	0.644963
XLOC_063110	NPHS1	chr18	46732069	46732997	11.2643	16.0011	14.6837	14.9746	13.7896	13.0456	13.7713	13.2268	16.3132
XLOC_063111	NPHS1	chr18	46733233	46733503	44.4833	32.2063	24.7653	27.914	37.3325	32.7293	28.5203	25.1943	34.4464
XLOC_086431	NPM2	chr8	69832576	69872890	1.90029	3.40918	0.530049	4.96314	5.98326	5.07292	1.68549	0.785251	1.47612
XLOC_087975	NPM2	chr8	69832576	69872890	3.83342	2.28695	5.97989	4.45619	4.39405	6.64493	0.659722	2.22165	2.32028
XLOC_065824	NPPC	chr2	120355201	120460073	1.25844	1.68859	2.44941	1.671	1.89407	2.30897	1.47194	1.69416	1.61024
XLOC_066493	NPPC	chr2	120355201	120460073	19.9243	27.169	25.5042	25.9995	26.1149	24.4211	28.7412	26.6881	33.9525
XLOC_072676	NPSR1	chr4	62546080	62547984	1.72421	1.03047	0.0	0.617597	0.267767	0.177955	0.461729	2.04795	0.600581
XLOC_072677	NPSR1	chr4	62583484	62690239	1.58907	2.10722	2.25826	0.940743	2.40279	1.1259	1.61534	0.697804	2.93956
XLOC_074384	NPSR1	chr4	62583484	62690239	1.25524	1.50232	1.30976	0.300155	0.919306	0.391829	0.612161	0.294578	1.39117
XLOC_041470	NPTN	chr10	20148957	20280632	11.0122	14.1758	11.6008	29.9279	28.277	30.1412	29.7384	32.34	30.0707
XLOC_042430	NPTN	chr10	20148957	20280632	0.0	0.618483	0.0	0.926424	2.24386	2.98422	2.01943	0.611979	3.78051
XLOC_042431	NPTN	chr10	20148957	20280632	0.0	1.16849	3.05429	1.05072	1.49578	0.798549	4.37477	1.13375	1.01671
XLOC_076341	NR1H4	chr5	64803398	64831590	15.8013	11.0699	11.431	13.6383	13.3961	12.5709	14.4032	18.7259	12.6596
XLOC_078030	NR1H4	chr5	64801462	64803181	0.463165	1.38577	3.26198	1.74427	0.835607	1.25266	1.05792	0.838039	0.607781
XLOC_078031	NR1H4	chr5	64803398	64831590	3.011	2.4953	4.71357	3.13674	2.96692	2.82297	3.80432	3.52568	3.42722
XLOC_078032	NR1H4	chr5	64803398	64831590	1.62204	1.94134	1.90407	2.07268	1.84574	2.19426	2.11408	2.08036	1.95154
XLOC_076772	NR2C1;NDUFA12	chr5	24862747	25100726	13.6229	18.3402	9.29627	7.91678	11.0899	9.18968	13.6186	13.299	10.3894
XLOC_077432	NR2C1;NDUFA12	chr5	24862747	25100726	0.0	0.0	0.0	1.61437	1.40636	0.549133	0.574534	1.3747	1.11007
XLOC_041861	NR2E3	chr10	18635685	18647250	2.64561	2.7699	2.06985	0.296466	0.569773	0.274862	1.20412	0.53058	0.693829
XLOC_083031	NR3C1	chr7	56204843	56231980	31.2024	42.4484	42.075	15.2023	11.4996	12.9208	15.4453	16.8146	12.0237
XLOC_070750	NRAS	chr3	28729259	28729724	0.0	0.702495	1.8371	0.0	0.0	0.0	0.0	0.0	0.0
XLOC_070751	NRAS	chr3	28729878	28731337	0.0	0.498739	0.434808	0.0	0.0	0.0	0.0	0.0557952	0.0
XLOC_072639	NRCAM	chr4	49513942	49521865	0.0	0.0	0.0017215	0.0	0.0	0.0	0.0	0.0	0.0
XLOC_073171	NRCAM	chr4	49513942	49521865	11.5806	9.89877	10.3555	22.4469	17.3767	20.9836	19.7179	22.3152	21.4611
XLOC_073172	NRCAM	chr4	49536330	49549662	0.441398	0.132067	0.345417	0.0	0.0	0.0	0.0806887	0.0	0.0772352
XLOC_073173	NRCAM	chr4	49585166	49597864	2.93323	1.70444	2.44377	0.510812	0.432257	0.801865	0.402708	0.303611	0.25669
XLOC_074218	NRCAM	chr4	49529018	49529272	0.0	4.38136	0.0	3.29961	3.30549	9.62095	2.41649	0.688555	6.31449
XLOC_074219	NRCAM	chr4	49530647	49531965	2.49286	2.42364	2.43802	1.73203	1.65995	1.29527	1.47602	1.31298	1.4167

XLOC_070035	NRD1	chr3	95111029	95122933	26.524	30.6315	22.3599	46.0653	43.1057	40.9619	43.7202	53.8422	43.6849
XLOC_070407	NRD1	chr3	95032700	95042643	100.945	129.247	106.245	148.694	124.041	137.459	135.5	180.821	140.461
XLOC_070408	NRD1	chr3	95058113	95106912	39.4543	45.1935	31.7447	17.6033	16.7956	14.9162	16.0104	19.329	16.7438
XLOC_071713	NRD1	chr3	95027765	95028333	0.0	3.15909	1.37695	0.631115	1.50456	1.81815	0.628704	1.04595	1.07392
XLOC_071714	NRD1	chr3	95031054	95031328	0.0	3.6641	4.78988	5.50707	3.24325	0.620988	2.04805	1.1621	1.0578
XLOC_092123	NRK	chrX	53434889	53443574	25.0502	33.6734	19.5691	0.0	0.0	0.0	0.0	0.0	0.0
XLOC_093033	NRK	chrX	53418328	53418969	47.8704	27.2748	25.7258	9.78136	11.6372	6.9577	19.7116	13.9573	12.5162
XLOC_051273	NRP1	chr13	19994824	19995911	32.6805	33.5137	33.4781	47.5674	45.1729	48.9536	60.8519	61.9409	50.8582
XLOC_083446	NRTN;FUT5	chr7	19716057	19749783	91.6459	84.6529	92.62	72.1907	75.2438	73.2615	39.3749	52.0212	51.554
XLOC_044017	NRXN1	chr11	32642888	32747276	39.5057	41.1791	40.2829	23.7931	19.9267	21.7922	23.5264	25.1058	21.9601
XLOC_044018	NRXN1	chr11	33039120	33039709	5.70288	10.2232	7.42656	27.4015	29.48	30.3775	24.5256	22.7183	35.733
XLOC_044019	NRXN1	chr11	33152065	33163238	16.2631	13.2424	16.3715	63.4332	45.7162	53.6444	71.8551	83.1876	66.9917
XLOC_044713	NRXN1	chr11	32642888	32747276	0.66084	0.768482	0.796336	0.717216	0.491037	0.940917	0.829808	1.06259	0.290066
XLOC_044714	NRXN1	chr11	33152065	33163238	215.883	247.315	176.992	1044.09	877.32	1016.54	1269.2	1302.2	1261.21
XLOC_044715	NRXN1	chr11	33212457	33513675	7.86154	9.41887	13.2351	23.8775	20.0275	22.6232	27.499	26.298	24.8406
XLOC_045732	NRXN1	chr11	32638697	32639537	0.0	0.316881	0.0	0.0949628	0.165454	0.109827	0.0	0.0	0.0
XLOC_045733	NRXN1	chr11	32642888	32747276	15.5801	4.64724	3.03789	1.045	4.76028	4.36785	1.00453	3.00783	1.01021
XLOC_045734	NRXN1	chr11	32909592	32910973	4.72899	4.77466	3.23758	16.111	12.8769	14.9301	16.3792	18.6879	15.9201
XLOC_045735	NRXN1	chr11	33144127	33144695	1.76201	0.526515	1.37695	0.473336	0.410334	0.36363	1.57176	0.348649	0.153416
XLOC_045736	NRXN1	chr11	33147956	33149518	1.02988	0.616241	0.805874	3.09341	3.71452	2.78449	3.57214	2.74165	3.10774
XLOC_045737	NRXN1	chr11	33149677	33150157	0.0	1.33978	0.0	6.22288	5.89697	5.76872	5.75171	5.73591	7.21298
XLOC_045738	NRXN1	chr11	33150244	33150665	0.0	2.45838	4.2858	10.8048	11.4175	9.5728	12.9908	17.4454	13.099
XLOC_045739	NRXN1	chr11	33150801	33151167	3.46738	5.17273	5.41039	22.3254	23.3774	23.3789	33.3174	35.643	27.3131
XLOC_045740	NRXN1	chr11	33151312	33151944	3.04865	3.18918	3.57451	17.4757	13.7521	19.06	23.4892	25.1068	19.1284
XLOC_045741	NRXN1	chr11	33152065	33163238	27.7675	8.28005	16.235	44.8616	41.7189	44.8005	52.166	49.5931	64.4733
XLOC_045742	NRXN1	chr11	33152065	33163238	9.93858	13.2403	9.18715	34.6582	32.7677	34.023	41.7565	43.6301	39.7733
XLOC_045743	NRXN1	chr11	33152065	33163238	25.5428	16.644	18.1359	50.2906	55.9893	49.1891	72.3814	71.4235	61.1389
XLOC_045744	NRXN1	chr11	33172879	33173442	21.4049	14.3909	18.1207	34.34	29.9032	32.3897	40.081	46.2241	37.737
XLOC_045745	NRXN1	chr11	33206932	33207696	0.0	0.713093	0.0	0.213698	1.02295	0.864418	0.537462	0.356416	1.35263
XLOC_045746	NRXN1	chr11	33208123	33208505	0.0	0.0	0.0	2.88069	1.23609	0.329493	0.279984	0.0	0.558061
XLOC_045747	NRXN1	chr11	33208911	33209859	0.0	0.273624	0.715628	0.574001	0.715069	0.0949062	0.497363	0.182921	0.319635
XLOC_045748	NRXN1	chr11	33210055	33211156	0.0	1.3757	1.19933	1.0994	0.539839	0.397943	0.905345	0.997982	0.535889
XLOC_045749	NRXN1	chr11	33212081	33212367	0.0	4.99942	4.35709	3.50351	2.53583	2.82928	9.37902	5.3095	5.77799

XLOC_045750	NRXN1	chr11	33212457	33513675	1.66468	1.49243	0.0	2.8325	2.58668	2.75003	4.01647	3.13291	2.75486
XLOC_045751	NRXN1	chr11	33212457	33513675	0.0	0.334509	0.874887	2.10516	1.8333	3.24523	2.52448	1.89644	2.53855
XLOC_045752	NRXN1	chr11	33212457	33513675	0.0	2.1751	3.79243	2.28141	1.60733	2.38534	2.29436	2.41536	1.90413
XLOC_045753	NRXN1	chr11	33212457	33513675	0.0	0.0	0.0	1.28543	0.364409	0.0	1.21868	2.74927	6.61076
XLOC_045754	NRXN1	chr11	33212457	33513675	0.0	2.089	0.0	1.2523	2.41384	3.21828	2.72602	3.05439	2.42407
XLOC_045755	NRXN1	chr11	33212457	33513675	0.0	1.14951	0.0	1.37845	1.76721	4.32207	3.31508	2.60512	2.66565
XLOC_045756	NRXN1	chr11	33212457	33513675	0.0	0.667825	0.0	1.60098	1.38362	1.61036	2.37299	0.65986	3.10956
XLOC_045757	NRXN1	chr11	33212457	33513675	0.0	0.910987	1.42959	1.52888	1.62247	2.27869	1.94229	1.46668	2.82237
XLOC_045758	NRXN1	chr11	33212457	33513675	3.32171	7.44503	2.59612	68.121	83.8693	74.9385	98.5547	89.0044	97.3573
XLOC_045759	NRXN1	chr11	33212457	33513675	0.0	0.0	3.03799	1.04493	0.893083	3.97043	2.00905	1.50393	1.34694
XLOC_045760	NRXN1	chr11	33212457	33513675	0.0	0.315956	0.0	1.98841	2.8872	1.97116	3.53283	1.58163	2.21368
XLOC_045761	NRXN1	chr11	33212457	33513675	3.09431	0.462409	2.41868	1.80143	2.16572	2.07817	4.57225	1.84163	3.37036
XLOC_045762	NRXN1	chr11	33212457	33513675	0.0	2.67049	0.0	1.33384	1.37627	3.66593	3.64427	2.61726	1.03423
XLOC_045763	NRXN1	chr11	33212457	33513675	0.0	0.944462	2.46979	1.69831	2.43045	2.26694	3.30434	1.8477	2.46786
XLOC_045764	NRXN1	chr11	33212457	33513675	28.9013	30.4609	20.2131	29.5646	18.3377	24.0505	21.3969	31.847	25.0535
XLOC_045765	NRXN1	chr11	33212457	33513675	0.0	0.0	0.0	1.05624	1.50379	2.00632	3.72057	1.51954	1.02102
XLOC_044012	NRXN1;NRXN1	chr11	32205275	32211754	5.22639	3.37023	4.89708	6.99142	6.30994	6.59743	5.75925	6.50222	6.92764
XLOC_044013	NRXN1;NRXN1	chr11	32226476	32232283	17.5832	11.3921	17.8464	13.297	13.8471	11.2531	8.4288	9.22062	8.88683
XLOC_044014	NRXN1;NRXN1	chr11	32276584	32284451	1279.13	148.111	691.898	0.0	0.0	0.0	0.0	1.10597	0.0
XLOC_044015	NRXN1;NRXN1	chr11	32347840	32424368	4.42851	2.58753	3.63145	0.96461	0.878493	0.856865	1.0647	1.06321	0.923271
XLOC_044016	NRXN1;NRXN1	chr11	32455456	32625139	31.6608	33.9647	37.1377	14.8412	15.0814	13.9412	14.6548	15.5599	12.5393
XLOC_044710	NRXN1;NRXN1	chr11	32205275	32211754	10.9322	7.22369	7.29929	2.18716	2.28678	3.24214	0.613673	0.433237	0.983172
XLOC_044711	NRXN1;NRXN1	chr11	32211926	32222251	19.1351	12.1633	14.6827	6.79778	5.09399	7.41258	2.10869	2.32722	2.9356
XLOC_044712	NRXN1;NRXN1	chr11	32232796	32267664	3.54547	5.13275	5.3288	6.88902	8.04667	6.50534	7.11697	6.09491	7.38122
XLOC_045723	NRXN1;NRXN1	chr11	32187575	32187976	0.0	0.0	0.0	0.0	0.0	0.0	0.0	0.289653	0.0
XLOC_045724	NRXN1;NRXN1	chr11	32211926	32222251	0.0	2.73453	0.0	0.819482	1.3235	0.811327	0.470157	0.0	0.341895
XLOC_045725	NRXN1;NRXN1	chr11	32211926	32222251	3.04866	3.64478	3.57451	1.50183	1.30408	0.472584	0.27313	0.302492	0.265672

XLOC_045726	NRXN1;NRXN1	chr11	32211926	32222251	3.04687	2.73402	3.57528	1.6387	0.715265	1.26542	0.069208	0.534011	0.33282
XLOC_045727	NRXN1;NRXN1	chr11	32424497	32424839	0.0	0.0	0.0	0.350167	1.1963	0.399131	1.00956	0.755835	0.677058
XLOC_045728	NRXN1;NRXN1	chr11	32424966	32426894	3.6764	3.05561	1.91805	0.732594	0.673177	0.510076	0.821905	0.780698	0.571882
XLOC_045729	NRXN1;NRXN1	chr11	32427000	32428401	2.32646	1.91396	4.09569	0.312869	0.455784	0.302281	0.63626	0.758929	0.559449
XLOC_045730	NRXN1;NRXN1	chr11	32455456	32625139	14.7049	16.2043	16.3468	6.93741	4.66283	5.22293	4.35881	4.96008	4.26066
XLOC_045731	NRXN1;NRXN1	chr11	32455456	32625139	0.0	1.91522	3.00533	0.344391	0.399379	1.06098	0.0	0.510136	0.223509
XLOC_079843	NSG1	chr6	106769177	106800089	20.6031	20.6969	18.1835	40.8527	35.5697	38.5329	45.5223	51.4361	46.8296
XLOC_080250	NSG1	chr6	106769177	106800089	27.7923	38.4867	35.6632	29.8351	27.199	26.1164	23.6313	30.1544	34.1564
XLOC_082118	NSG1	chr6	106769177	106800089	3.88022	3.74096	3.71129	1.31446	2.50322	2.28771	2.52249	1.34403	2.75366
XLOC_082119	NSG1	chr6	106769177	106800089	7.68194	4.73389	12.0605	2.39337	3.26317	2.05892	5.05169	2.457	5.44875
XLOC_082120	NSG1	chr6	107255612	107256367	2.421	3.98102	1.89303	0.433825	0.188765	0.250669	0.872605	0.0	0.316827
XLOC_082121	NSG1;HGFAC;HGFAC	chr6	107276727	107277120	6.14167	10.9982	19.173	1.37331	0.235958	0.628794	1.87332	1.49565	1.33077
XLOC_057949	NSL1;TATDN3;FAM71A;ATF3;BATF3	chr16	72663070	72956668	11.3058	17.864	13.9891	3.84057	1.91909	2.61724	2.90039	4.71989	3.82031
XLOC_059245	NSL1;TATDN3;FAM71A;ATF3;BATF3	chr16	72663070	72956668	0.0	0.0	0.0	0.198042	0.17247	0.228987	0.0	0.11021	0.0
XLOC_059246	NSL1;TATDN3;FAM71A;ATF3;BATF3	chr16	72663070	72956668	0.853398	0.765705	1.33508	0.152979	0.0667303	0.0885566	0.0	0.0853731	0.0745508
XLOC_053198	NSMCE2	chr14	16383135	16575598	23.3607	21.1748	17.8357	16.3581	21.994	18.9154	24.6469	16.8953	26.1788
XLOC_053478	NSMCE2	chr14	16383135	16575598	0.231187	0.386864	0.574932	0.649061	0.603874	0.483847	0.473451	0.60397	0.851171
XLOC_053876	NSMCE2	chr14	16383135	16575598	0.0	0.575506	0.0	0.172644	0.447998	0.198563	0.171316	0.0	0.167623
XLOC_053877	NSMCE2	chr14	16383135	16575598	0.0	0.194823	0.0	0.058449	0.0	0.0676678	0.0592953	0.0	0.0569377
XLOC_053878	NSMCE2	chr14	16383135	16575598	0.0	0.0	0.0	0.155258	0.135364	0.0	0.0785004	0.173173	0.226857
XLOC_037808	NSUN3	chr1	37998036	38019311	13.7589	19.4765	15.0663	16.5238	25.6639	28.6388	38.891	27.2318	14.7404
XLOC_037809	NSUN3	chr1	38019432	38021611	1.52433	113.246	1182.65	949.356	1304.12	180.026	1737.23	1677.53	158.34
XLOC_038323	NSUN3	chr1	37987122	37997363	39.749	32.8539	29.4889	30.6954	25.3218	31.3835	36.5487	37.1569	34.8101

XLOC_039151	NSUN3	chr1	37984284	37984509	0.0	9.07298	0.0	9.16186	4.5132	8.12473	8.13881	3.73672	13.0393
XLOC_039152	NSUN3	chr1	37984850	37985227	6.58832	7.86393	5.14083	1.76762	2.77969	3.36844	4.86184	4.80111	3.99425
XLOC_039153	NSUN3	chr1	37985547	37986621	5.52401	10.6215	8.02516	3.60751	3.76602	3.52264	4.22816	4.02939	3.99933
XLOC_039154	NSUN3	chr1	37987122	37997363	0.0	6.89476	6.76109	4.1323	2.88802	4.43859	9.08695	3.94574	6.00937
XLOC_039155	NSUN3	chr1	37987122	37997363	0.0	4.13985	0.0	6.23062	7.30081	8.40009	5.73253	10.4398	5.37233
XLOC_039156	NSUN3	chr1	37987122	37997363	1.21458	0.726265	4.74844	1.0882	2.5568	1.13177	2.07924	4.23386	2.01328
XLOC_039157	NSUN3	chr1	37987122	37997363	4.28458	5.1108	3.34088	1.53259	3.91703	2.61557	2.56256	2.47145	4.81032
XLOC_044300	NT5C1B;RDH14	chr11	80209077	80267216	17.3169	23.601	20.3552	36.0333	36.7929	35.2731	38.4181	39.834	45.3023
XLOC_089816	NT5DC1	chr9	35004861	35036249	1.39635	1.46946	1.09077	1.19271	1.29158	1.00619	1.98125	0.771573	2.06531
XLOC_090537	NT5DC1	chr9	35004861	35036249	0.0	0.0	0.0	0.123767	0.0	0.142902	0.24821	0.549305	0.240928
XLOC_090538	NT5DC1	chr9	35004861	35036249	0.0	0.0	2.13483	0.0	0.0	0.0	1.19853	0.534819	0.0
XLOC_090539	NT5DC1	chr9	35004861	35036249	0.0	0.217096	0.0	0.260243	0.284052	0.226124	0.263951	0.363605	0.190298
XLOC_090540	NT5DC1	chr9	35004861	35036249	0.0	0.240224	2.51312	0.57593	0.188484	0.416851	0.145836	0.401988	0.701756
XLOC_090541	NT5DC1	chr9	35004861	35036249	2.48619	0.4463	0.778185	0.356672	0.350935	0.258564	0.590213	0.349733	0.217471
XLOC_090542	NT5DC1	chr9	35004861	35036249	0.0	0.83508	0.873641	0.250264	0.393814	0.464303	0.356408	0.168156	0.390573
XLOC_090543	NT5DC1	chr9	35004861	35036249	1.49354	0.446737	1.1684	0.334702	0.409101	0.232631	0.882298	0.0748043	0.978924
XLOC_090544	NT5DC1	chr9	35004861	35036249	0.0	0.527073	0.0	0.737137	0.414184	0.427302	0.481794	0.589439	0.462182
XLOC_089460	NT5E;NT5E;SNX14	chr9	64811343	64919613	15.6991	12.6467	10.2927	3.64404	4.10672	3.65556	4.9624	2.86745	3.24245
XLOC_076945	NTF3	chr5	105079827	105171791	5.92005	1.2458	3.45912	2.64953	1.74297	2.12347	1.63222	2.24997	1.55469
XLOC_078513	NTF3	chr5	105079827	105171791	0.0	0.0	0.0	0.186327	0.161175	0.857436	0.923009	0.410276	0.724097
XLOC_078514	NTF3	chr5	105079827	105171791	0.0	0.0	0.0	0.0	0.250129	0.0	0.0	0.0	0.560728
XLOC_063956	NTN1	chr19	29101370	29143923	17.1451	13.8557	15.0187	32.0231	25.9554	33.5908	31.6994	40.4602	25.8682
XLOC_064956	NTN1	chr19	29144867	29145580	2.60407	0.0	1.01799	1.5164	1.21733	2.2905	0.702757	1.03632	0.794897
XLOC_064957	NTN1	chr19	29146002	29148683	0.0	0.428814	0.897248	0.950997	0.76543	0.865507	0.709318	0.981637	0.652292
XLOC_064958	NTN1	chr19	29148782	29149554	2.35402	0.703819	0.920358	0.421837	0.550774	1.46271	0.848986	0.586404	0.616197
XLOC_063957	NTN1;STX8	chr19	29251656	29310962	34.3928	33.6676	38.1245	2.70242	2.2136	2.37732	3.3244	2.441	3.513
XLOC_069876	NTNG1	chr3	36066011	36091751	7.50025	9.31583	7.97296	18.3348	11.8195	13.1815	16.5754	27.9673	17.0626
XLOC_069877	NTNG1	chr3	36151081	36164991	8.77994	8.53523	5.72388	13.0518	12.8279	11.7772	11.9726	15.3939	15.6664
XLOC_070257	NTNG1	chr3	35956264	36000664	1.45137	0.651552	1.57464	4.06975	5.35751	4.63207	6.23777	5.56919	3.60535
XLOC_070258	NTNG1	chr3	36179567	36222210	0.0	0.0	0.393965	0.0	0.0	0.0	0.0	0.0	0.0
XLOC_070259	NTNG1	chr3	36267776	36270796	0.0	0.189369	0.0	0.0	0.0	0.0	0.0	0.0	0.0
XLOC_070849	NTNG1	chr3	35956264	36000664	3.71583	0.0	0.0	3.32273	0.852551	1.51645	0.640702	1.43748	0.0

XLOC_070850	NTNG1	chr3	35956264	36000664	0.0	0.408332	0.0	1.34603	1.4892	1.27166	0.613669	1.62955	0.952844
XLOC_070851	NTNG1	chr3	36091909	36092227	0.0	2.68097	7.01007	8.84527	8.20882	5.94059	6.89205	9.91959	4.6566
XLOC_070852	NTNG1	chr3	36094404	36094707	0.0	0.0	3.85973	0.442977	1.12703	0.0	0.837899	2.36477	4.6967
XLOC_070853	NTNG1	chr3	36151081	36164991	2.58785	2.31806	0.0	0.463086	0.199579	0.53138	1.13718	1.26751	2.47124
XLOC_070854	NTNG1	chr3	36151081	36164991	0.631376	0.377748	0.987972	0.509429	0.544039	0.459258	0.690057	0.63335	0.552003
XLOC_070855	NTNG1	chr3	36166124	36166474	3.75422	6.71938	14.6417	8.72831	10.3335	14.1693	9.38033	13.7914	12.6631
XLOC_070856	NTNG1	chr3	36166656	36167071	2.80879	9.22341	4.38531	9.79948	11.2451	9.50402	12.7866	15.9172	15.8376
XLOC_070857	NTNG1	chr3	36167194	36168360	4.3029	4.07578	4.48836	5.40012	4.94017	4.39441	5.0866	5.89279	6.01726
XLOC_070858	NTNG1	chr3	36168662	36169291	4.60208	0.916984	3.59722	3.02272	5.0105	4.5969	4.80934	5.63104	3.74295
XLOC_070859	NTNG1	chr3	36169690	36170184	4.29913	0.642076	5.03736	4.8101	7.4856	6.19568	4.18886	5.29176	8.97104
XLOC_044478	NTNG2	chr11	102284852	102296160	2.19677	2.77346	6.55027	2.99095	2.80648	2.67255	3.37228	2.57082	3.14455
XLOC_045111	NTNG2	chr11	102284852	102296160	12.2345	12.7417	9.25753	17.1928	13.3025	14.8747	16.8967	20.8631	14.2809
XLOC_045112	NTNG2	chr11	102303955	102305713	3.87484	4.10283	4.45404	21.3424	17.7519	20.3122	23.804	33.5186	22.9414
XLOC_045113	NTNG2	chr11	102306994	102319619	54.2686	63.8464	59.1918	105.834	77.0983	94.3121	99.4737	145.26	92.0972
XLOC_047316	NTNG2	chr11	102284852	102296160	7.00701	14.6239	0.0	6.91823	1.57854	2.11918	3.46909	3.29121	4.21699
XLOC_047317	NTNG2	chr11	102284852	102296160	8.23497	4.0973	2.14291	2.45564	1.69148	3.09708	1.92457	1.07356	1.66714
XLOC_047318	NTNG2	chr11	102297189	102298108	0.0	1.13614	0.742856	0.851201	0.296838	0.492497	0.429977	0.664321	0.248825
XLOC_047319	NTNG2	chr11	102305903	102306603	33.331	29.0948	36.4822	47.4168	35.1012	40.5536	42.1923	58.3415	39.6463
XLOC_047320	NTNG2	chr11	102319815	102320824	0.0	1.27018	0.664404	2.58844	4.31723	1.41028	3.77538	3.05914	3.63588
XLOC_086957	NTRK2	chr8	79338650	79399511	12.8356	13.9796	15.1807	9.51957	10.9042	10.8411	11.8024	7.64501	8.72028
XLOC_088272	NTRK2	chr8	79338650	79399511	6.64171	1.98563	8.30819	1.309	1.8625	1.78665	2.86644	1.58548	1.62176
XLOC_088273	NTRK2	chr8	79338650	79399511	1.61993	2.90493	1.2661	2.61139	2.64372	1.17111	2.31803	1.28447	1.97566
XLOC_050568	NTSR1	chr13	55105270	55134406	8.11801	7.55621	8.50837	6.56543	8.35601	6.60105	6.49157	6.3412	8.08376
XLOC_051875	NTSR1	chr13	55105270	55134406	11.005	4.02136	3.82441	2.08157	2.57395	2.40534	2.09299	2.67902	1.92017
XLOC_051876	NTSR1	chr13	55105270	55134406	0.0	4.45859	0.0	0.892068	2.26904	0.0	2.10798	0.952055	0.429886
XLOC_051877	NTSR1	chr13	55134831	55135049	0.0	0.0	0.0	1.00762	3.29105	2.22554	7.09635	3.0599	1.90659
XLOC_066525	NUDC;C2H1orf172	chr2	126842777	126876266	22.3327	24.1338	31.0252	31.0273	33.8598	31.019	37.3476	26.3987	35.8116
XLOC_053646	NUDCD1	chr14	57275000	57398525	4.9033	6.1937	5.11556	3.61468	5.33836	3.9084	4.02574	3.06375	5.62236
XLOC_054503	NUDCD1	chr14	57275000	57398525	44.1084	50.7195	44.2015	107.88	91.1434	96.1017	99.0408	120.2	97.0526
XLOC_092520	NUDT10	chrX	94074440	94150629	13.211	12.2337	12.2232	11.7578	12.0144	11.5661	13.3635	13.2621	12.7039
XLOC_093261	NUDT10	chrX	94074440	94150629	0.0	2.11007	3.15352	0.542036	1.10215	0.836035	1.73231	1.10719	0.26407
XLOC_093262	NUDT10	chrX	94074440	94150629	0.0	3.95649	1.21743	1.39496	1.03497	1.45408	1.90829	1.16872	1.69984

XLOC_093263	NUDT10	chrX	94074440	94150629	8.47235	2.5308	3.30928	1.13761	1.14769	1.52657	1.68961	1.66925	1.47353
XLOC_093264	NUDT10	chrX	94074440	94150629	0.0	3.18688	8.33056	3.8662	2.37339	4.2766	5.12582	10.7964	9.15589
XLOC_093265	NUDT10	chrX	94074440	94150629	0.0	2.68791	3.51469	4.02728	3.30546	3.70334	2.58617	1.99157	1.36892
XLOC_093266	NUDT10	chrX	94074440	94150629	8.56912	2.55543	10.0228	1.5327	3.26424	2.61563	1.83062	2.05964	2.22032
XLOC_093267	NUDT10	chrX	94074440	94150629	0.0	0.0	0.0	0.761924	0.324618	0.0	0.0	0.0	0.368057
XLOC_051141	NUDT5	chr13	12259857	12260275	27.7648	18.2351	26.0099	19.6223	28.6513	21.6403	25.5365	20.3534	20.4746
XLOC_050736	NUDT5;CDC123	chr13	12186015	12258175	31.4574	51.2222	51.0524	16.2685	15.6758	14.8429	16.304	19.2986	17.9097
XLOC_059764	NUDT6	chr17	35144005	35147394	57.6457	65.3431	56.4621	61.3848	49.9558	53.4624	57.6766	74.8499	44.0368
XLOC_059765	NUDT6	chr17	35149159	35195976	2.49014	4.14933	3.94449	2.75513	2.63662	2.48349	2.93161	2.13527	2.31005
XLOC_060155	NUDT6	chr17	35149159	35195976	100.906	124.84	106.456	66.6917	56.4354	64.2292	70.4337	99.966	38.848
XLOC_061312	NUDT6	chr17	35147489	35147652	33.6176	10.2616	26.8376	35.521	7.28183	3.37759	2.54149	2.95308	23.3537
XLOC_060156	NUDT6;FGF2	chr17	35199926	35201997	16.4109	2.80487	1.83396	0.0700481	0.0	0.081129	0.0	0.0	0.0682853
XLOC_041696	NUMB	chr10	85102824	85128361	13.7224	11.8775	9.549	15.4537	14.4198	15.4163	17.1141	18.0423	17.2884
XLOC_042094	NUMB	chr10	85102824	85128361	3.76143	2.3632	1.52482	3.97499	5.12181	5.05447	6.64245	7.37181	6.92274
XLOC_042095	NUMB	chr10	85131612	85185223	21.6468	22.3765	16.1681	14.1527	13.4327	12.004	12.2118	15.2261	11.9427
XLOC_043250	NUMB	chr10	85102824	85128361	9.40216	5.61574	0.0	10.1905	6.3011	11.3286	8.35758	14.8032	15.3429
XLOC_043251	NUMB	chr10	85130502	85130962	0.0	0.71408	5.60217	0.427959	0.369383	0.737348	0.210885	1.1739	0.207724
XLOC_055859	NUP160	chr15	78756019	78773494	1.38589	1.5204	1.06852	4.08247	4.89302	3.45755	3.20347	3.89053	3.38565
XLOC_045071	NUP188	chr11	99477976	99493413	0.0	0.386303	0.0	0.0771812	0.0	0.0	0.0393441	0.0	0.0376539
XLOC_072717	NUP205;SLC13A4;CNOT4;PL-5283	chr4	100095330	100334209	0.0	0.626778	0.546426	0.250448	0.109365	0.145096	0.0	0.0700063	0.0
XLOC_073257	NUP205;SLC13A4;CNOT4;PL-5283	chr4	100095330	100334209	0.0	0.273723	0.975848	0.148617	0.0718558	0.173305	0.114409	0.125019	0.109505
XLOC_074661	NUP205;SLC13A4;CNOT4;PL-5283	chr4	100095330	100334209	0.0	0.891897	0.77754	0.0890944	0.0	0.103075	0.179882	0.19857	0.173608
XLOC_074662	NUP205;SLC13A4;CNOT4;PL-5283	chr4	100095330	100334209	0.0	1.80232	0.0	0.541674	0.0	0.0	0.504301	0.0	0.0
XLOC_074663	NUP205;SLC13A4;CNOT4;PL-5283	chr4	100095330	100334209	0.950684	0.14222	0.371966	0.0852431	0.0	0.0	0.0868387	0.0	0.0

XLOC_074664	NUP205;SLC13A4;CNOT4;PL-5283	chr4	100095330	100334209	0.0	0.0	1.30101	0.0	0.0	0.171875	0.0	0.0	0.0
XLOC_074665	NUP205;SLC13A4;CNOT4;PL-5283	chr4	100095330	100334209	0.0	0.421864	2.20656	0.0	0.109861	0.0	0.2534	0.0	0.0
XLOC_074666	NUP205;SLC13A4;CNOT4;PL-5283	chr4	100095330	100334209	1.36268	0.0	0.0	0.0	0.0	0.0707783	0.0	0.0683058	0.0595578
XLOC_074667	NUP205;SLC13A4;CNOT4;PL-5283	chr4	100095330	100334209	0.0	0.282714	0.739432	0.0847274	0.0247323	0.0983682	0.0865578	0.0	0.0
XLOC_074668	NUP205;SLC13A4;CNOT4;PL-5283	chr4	100095330	100334209	0.0	0.0	1.51554	0.0868289	0.0756892	0.100467	0.0	0.0	0.0
XLOC_074669	NUP205;SLC13A4;CNOT4;PL-5283	chr4	100095330	100334209	0.0	0.151369	1.58357	0.0	0.0	0.105229	0.092375	0.0	0.0
XLOC_074671	NUP205;SLC13A4;CNOT4;PL-5283	chr4	100095330	100334209	0.0	0.0	2.95882	0.0	0.0	0.0	0.0	0.0	0.0
XLOC_074672	NUP205;SLC13A4;CNOT4;PL-5283	chr4	100095330	100334209	0.0	0.250512	0.0	0.0	0.0	0.0	0.0	0.0	0.0
XLOC_074673	NUP205;SLC13A4;CNOT4;PL-5283	chr4	100095330	100334209	0.0	1.45658	0.0	0.0	0.370819	0.0	0.0	0.0	0.0
XLOC_074674	NUP205;SLC13A4;CNOT4;PL-5283	chr4	100095330	100334209	1.1017	0.0	2.58462	0.0987197	0.0	0.0	0.0	0.0	0.19231
XLOC_074675	NUP205;SLC13A4;CNOT4;PL-5283	chr4	100095330	100334209	0.462194	0.207478	0.361771	0.0414533	0.0181614	0.0	0.0	0.0	0.0202341
XLOC_074676	NUP205;SLC13A4;CNOT4;PL-5283	chr4	100095330	100334209	1.47761	0.0	2.31009	0.0	0.0	0.0	0.0	0.0	0.0
XLOC_074677	NUP205;SLC13A4;CNOT4;PL-5283	chr4	100095330	100334209	1.65599	0.247647	1.29537	0.0	0.0	0.0859341	0.0	0.0	0.0
XLOC_074678	NUP205;SLC13A4;CNOT4;PL-5283	chr4	100095330	100334209	0.953329	0.356619	0.186547	0.0427508	0.0	0.0248266	0.0	0.0720723	0.020867
XLOC_074679	NUP205;SLC13A4;CNOT4;PL-5283	chr4	100095330	100334209	0.757108	0.226544	0.592515	0.0339465	0.0594283	0.0393988	0.0	0.0380927	0.033127

XLOC_074680	NUP205;SLC13A4;CNOT4;PL-5283	chr4	100095330	100334209	0.0	0.0	0.0	0.0	0.0	0.0	0.0	0.156091	0.0
XLOC_074681	NUP205;SLC13A4;CNOT4;PL-5283	chr4	100095330	100334209	0.0	0.0	0.982651	0.0	0.0	0.0	0.0	0.0	0.109628
XLOC_074682	NUP205;SLC13A4;CNOT4;PL-5283	chr4	100095330	100334209	3.93717	0.0	0.0	0.352062	0.0	0.0	0.0	0.379881	0.0
XLOC_074683	NUP205;SLC13A4;CNOT4;PL-5283	chr4	100095330	100334209	0.0	0.408669	0.356285	0.204123	0.0357155	0.0	0.0	0.0	0.0
XLOC_074684	NUP205;SLC13A4;CNOT4;PL-5283	chr4	100095330	100334209	0.0	0.159629	0.417495	0.0478385	0.0	0.0	0.0	0.0	0.0466647
XLOC_074685	NUP205;SLC13A4;CNOT4;PL-5283	chr4	100095330	100334209	0.576617	0.0862765	0.451306	0.0	0.022647	0.0300232	0.0	0.0	0.0
XLOC_074687	NUP205;SLC13A4;CNOT4;PL-5283	chr4	100095330	100334209	0.937472	0.0	0.0	0.0	0.0732549	0.0	0.0849	0.0	0.0
XLOC_074688	NUP205;SLC13A4;CNOT4;PL-5283	chr4	100095330	100334209	0.0	0.245401	0.0	0.0	0.0	0.0	0.0	0.0	0.0
XLOC_074689	NUP205;SLC13A4;CNOT4;PL-5283	chr4	100095330	100334209	1.30429	0.650417	0.680456	0.0389848	0.0	0.0904662	0.0	0.0	0.0380381
XLOC_074690	NUP205;SLC13A4;CNOT4;PL-5283	chr4	100095330	100334209	0.0	0.0	0.644001	0.0737927	0.0	0.0854475	0.0	0.0	0.0
XLOC_074691	NUP205;SLC13A4;CNOT4;PL-5283	chr4	100095330	100334209	0.221608	0.198961	0.52038	0.0397516	0.0	0.0	0.0	0.0	0.0194039
XLOC_074692	NUP205;SLC13A4;CNOT4;PL-5283	chr4	100095330	100334209	0.371935	0.111293	0.582159	0.0333532	0.0583923	0.0387114	0.0	0.0	0.0
XLOC_074693	NUP205;SLC13A4;CNOT4;PL-5283	chr4	100095330	100334209	0.0	0.0	0.0	0.0	0.0	0.35424	0.0	0.0	0.0
XLOC_074694	NUP205;SLC13A4;CNOT4;PL-5283	chr4	100095330	100334209	1.65705	0.495206	1.29506	0.0	0.0	0.171095	0.0	0.0	0.0
XLOC_074696	NUP205;SLC13A4;CNOT4;PL-5283	chr4	100095330	100334209	0.314649	0.379	0.251801	0.029129	0.0	0.0167476	0.0574768	0.0	0.0

XLOC_074697	NUP205;SLC13A4;CNOT4;PL-5283	chr4	100095330	100334209	0.914937	0.0	1.43126	0.164	0.0	0.0949062	0.0	0.0	0.0
XLOC_074698	NUP205;SLC13A4;CNOT4;PL-5283	chr4	100095330	100334209	0.709567	0.106162	0.277662	0.0	0.0278537	0.0738605	0.0	0.0714221	0.0
XLOC_074699	NUP205;SLC13A4;CNOT4;PL-5283	chr4	100095330	100334209	1.60067	0.159629	1.66998	0.0478385	0.083657	0.0554773	0.0	0.0535876	0.0
XLOC_074700	NUP205;SLC13A4;CNOT4;PL-5283	chr4	100095330	100334209	2.65429	1.58496	0.0	0.0	0.0	0.0	0.0	0.0	0.0
XLOC_074701	NUP205;SLC13A4;CNOT4;PL-5283	chr4	100095330	100334209	0.0	0.0	1.32849	0.0	0.0	0.0	0.0	0.0	0.0
XLOC_074702	NUP205;SLC13A4;CNOT4;PL-5283	chr4	100095330	100334209	0.0	0.0	1.14247	0.0	0.0	0.0	0.0	0.0	0.0
XLOC_074703	NUP205;SLC13A4;CNOT4;PL-5283	chr4	100095330	100334209	0.0	0.327713	0.0	0.0491058	0.0	0.0569428	0.0	0.0549984	0.0
XLOC_074705	NUP205;SLC13A4;CNOT4;PL-5283	chr4	100095330	100334209	0.26419	0.158121	0.0	0.0236939	0.0	0.0	0.0	0.0266223	0.0
XLOC_074706	NUP205;SLC13A4;CNOT4;PL-5283	chr4	100095330	100334209	0.0	0.518419	0.677928	0.0	0.0	0.0	0.0	0.0	0.0
XLOC_074707	NUP205;SLC13A4;CNOT4;PL-5283	chr4	100095330	100334209	0.0	0.366201	0.957719	0.0	0.0	0.0	0.0	0.0	0.0
XLOC_074708	NUP205;SLC13A4;CNOT4;PL-5283	chr4	100095330	100334209	0.0	0.36374	0.0	0.0	0.0	0.0	0.0	0.0	0.0
XLOC_074709	NUP205;SLC13A4;CNOT4;PL-5283	chr4	100095330	100334209	0.0	0.26754	0.69971	0.0801762	0.0	0.0	0.0	0.0	0.0
XLOC_074710	NUP205;SLC13A4;CNOT4;PL-5283	chr4	100095330	100334209	0.0	0.417794	0.0	0.0	0.0	0.0	0.0	0.0	0.0
XLOC_074711	NUP205;SLC13A4;CNOT4;PL-5283	chr4	100095330	100334209	0.490494	0.0	1.15145	0.0439795	0.0	0.0	0.0	0.0	0.0
XLOC_074713	NUP205;SLC13A4;CNOT4;PL-5283	chr4	100095330	100334209	0.0	0.14354	0.750838	0.0	0.0	0.0	0.0	0.0	0.0

XLOC_074714	NUP205;SLC13A4;CNOT4;PL-5283	chr4	100095330	100334209	0.0	0.912666	0.0	0.273505	0.0	0.0	0.0	0.0	0.0
XLOC_074716	NUP205;SLC13A4;CNOT4;PL-5283	chr4	100095330	100334209	0.0	0.593844	0.388292	0.0444922	0.0389129	0.103213	0.0	0.0	0.0
XLOC_074719	NUP205;SLC13A4;CNOT4;PL-5283	chr4	100095330	100334209	0.0	0.783882	0.0	0.234896	0.0	0.0	0.0	0.0	0.0
XLOC_074720	NUP205;SLC13A4;CNOT4;PL-5283	chr4	100095330	100334209	0.228903	0.479518	0.0	0.0410598	0.0179892	0.0238458	0.0	0.0	0.0
XLOC_074721	NUP205;SLC13A4;CNOT4;PL-5283	chr4	100095330	100334209	0.0	0.119329	0.312098	0.0	0.0	0.0415009	0.0	0.0	0.0
XLOC_074722	NUP205;SLC13A4;CNOT4;PL-5283	chr4	100095330	100334209	0.0	0.0	0.68781	0.0788126	0.0	0.0	0.0	0.0	0.0
XLOC_074724	NUP205;SLC13A4;CNOT4;PL-5283	chr4	100095330	100334209	0.0	0.17874	0.0	0.107131	0.0	0.0620982	0.0	0.0599596	0.0
XLOC_074725	NUP205;SLC13A4;CNOT4;PL-5283	chr4	100095330	100334209	0.0	0.0732307	0.957662	0.0438932	0.0	0.0	0.0	0.0	0.0
XLOC_074726	NUP205;SLC13A4;CNOT4;PL-5283	chr4	100095330	100334209	1.12222	0.0	0.0	0.0	0.0	0.0	0.0	0.0	0.0
XLOC_074727	NUP205;SLC13A4;CNOT4;PL-5283	chr4	100095330	100334209	0.0	0.0	1.15504	0.0	0.0	0.0	0.0	0.0	0.128787
XLOC_074729	NUP205;SLC13A4;CNOT4;PL-5283	chr4	100095330	100334209	0.0	0.657689	0.0	0.0	0.0	0.226591	0.0	0.0	0.0
XLOC_074730	NUP205;SLC13A4;CNOT4;PL-5283	chr4	100095330	100334209	0.0	0.430249	1.1252	0.0	0.0	0.0	0.0	0.0	0.0
XLOC_074731	NUP205;SLC13A4;CNOT4;PL-5283	chr4	100095330	100334209	0.0	0.144072	0.376819	0.0647663	0.0	0.0	0.0	0.0242634	0.0
XLOC_074732	NUP205;SLC13A4;CNOT4;PL-5283	chr4	100095330	100334209	0.0	0.552032	0.0	0.0	0.0	0.0	0.0	0.0	0.0
XLOC_074733	NUP205;SLC13A4;CNOT4;PL-5283	chr4	100095330	100334209	1.18085	0.0	0.0	0.0	0.0	0.0	0.0	0.117659	0.103033

XLOC_074734	NUP205;SLC13A4;CNOT4;PL-5283	chr4	100095330	100334209	0.0	0.0	0.0	0.0	0.101445	0.0	0.0	0.0	0.0
XLOC_074735	NUP205;SLC13A4;CNOT4;PL-5283	chr4	100095330	100334209	1.58098	0.0	0.0	0.0	0.0	0.0	0.0	0.0	0.0
XLOC_074737	NUP205;SLC13A4;CNOT4;PL-5283	chr4	100095330	100334209	0.0	0.0	0.0	0.0	0.0	0.0	0.472613	0.0	0.0
XLOC_074738	NUP205;SLC13A4;CNOT4;PL-5283	chr4	100095330	100334209	1.49905	0.0	1.17178	0.0	0.11661	0.0	0.0	0.0	0.0
XLOC_044461	NUP214	chr11	101425197	101437259	2.85193	3.25584	2.59116	3.27367	2.89489	2.73798	2.85182	3.16165	2.0532
XLOC_045096	NUP214	chr11	101406635	101417770	10.7927	11.2683	9.42466	64.3751	52.8972	58.1888	58.0657	69.7506	65.1869
XLOC_045097	NUP214	chr11	101418766	101424992	1.35629	1.01151	2.12221	0.633186	0.683481	0.509156	0.925679	0.781753	0.71855
XLOC_045098	NUP214	chr11	101425197	101437259	2.11687E-	1.52473	1.57187	0.301086	0.670457	0.00738614	0.914691	0.378639	1.12933
XLOC_047290	NUP214	chr11	101405118	101405382	0.0	1.99616	0.0	0.600599	1.00697	1.35106	0.554336	0.0	0.575844
XLOC_066147	NUP35	chr2	13276105	13493689	1.43617	0.640731	1.70172	0.0768105	0.0385054	0.0829887	0.0732663	0.0865364	0.0803995
XLOC_081701	NUP54	chr6	92772701	92774374	16.7028	11.8508	19.0454	1.66882	1.31002	0.843837	1.17692	1.48684	2.63003
XLOC_062247	NUP62;ATF5;VRK3	chr18	56707265	56753434	35.8936	29.709	26.1601	126.585	101.783	125.045	155.514	176.873	106.684
XLOC_072566	NUPL2;GPNMB	chr4	31962489	32022559	23.4382	33.5545	23.9025	29.8482	28.7268	28.7425	33.285	34.1292	30.2061
XLOC_073903	NUPL2;GPNMB	chr4	31962489	32022559	4.4685	6.66153	6.96731	5.99391	7.82004	4.99686	8.75625	6.43149	6.55695
XLOC_073904	NUPL2;GPNMB	chr4	31962489	32022559	4.16319	6.2179	6.5043	4.28549	4.35152	4.07298	4.43044	2.25652	3.98253
XLOC_073905	NUPL2;GPNMB	chr4	31962489	32022559	4.28489	3.83306	0.0	2.29889	5.54912	3.05182	2.19648	3.29527	3.33022
XLOC_082747	NWD1	chr7	6172509	6190943	5.81163	6.49104	9.39388	13.1782	13.1097	13.0739	10.4671	11.2406	12.4448
XLOC_082748	NWD1	chr7	6222231	6238808	0.0	2.06056	3.09317	5.40895	3.46823	4.71198	3.74911	6.10043	4.39746
XLOC_084099	NWD1	chr7	6172509	6190943	0.0	5.14278	4.09364	2.94843	1.93063	1.86297	1.2229	2.09667	2.22118
XLOC_084100	NWD1	chr7	6172509	6190943	0.0	2.81079	0.816826	2.62062	2.36476	2.81454	1.69921	1.25087	1.73233
XLOC_084101	NWD1	chr7	6172509	6190943	6.49972	2.90942	0.0	4.06913	4.489	4.65358	1.97656	2.21149	2.25205
XLOC_092129	NXF3	chrX	56346384	56363024	2.29482	5.49208	5.89568	2.74614	3.80714	3.61111	2.72882	2.42328	3.07491
XLOC_093039	NXF3	chrX	56363565	56364439	3.02832	4.52787	3.94733	1.44737	3.23214	2.72083	2.09981	1.61264	3.08462
XLOC_093040	NXF3	chrX	56364699	56365665	3.57827	3.21044	4.89797	1.60352	2.30757	2.87695	2.91883	1.1628	2.96915
XLOC_092130	NXF3;NXF3	chrX	56374502	56380135	1.26972	1.21243	0.597013	1.55239	1.95631	1.74527	1.09424	0.972771	1.1569

XLOC_092131	NXF3;NXF3	chrX	56454807	56507785	15.5969	10.7941	14.0583	4.23938	5.4695	4.90939	4.13404	2.6839	4.31848
XLOC_086567	NXNL2	chr8	91122722	91134014	3.15003	3.44935	0.0	6.84836	6.61499	6.28748	6.29121	4.3331	3.5905
XLOC_087006	NXNL2	chr8	91122722	91134014	5.22672	7.66003	5.77172	15.7093	19.2236	17.7156	14.8243	13.0804	14.5949
XLOC_072550	NXPH1	chr4	17853210	18116566	1.22399	1.0988	0.239492	0.13721	0.0480659	0.0318616	0.112157	0.0308188	0.0535693
XLOC_073796	NXPH1	chr4	17853210	18116566	0.0	0.0	3.40878	0.0	0.0	0.0	0.193162	0.0	0.0
XLOC_061807	OAS1X;OAS1Z	chr17	63675276	63675666	0.0	0.0	0.0	3.61652	1.43356	1.91025	2.97969	0.90854	2.15637
XLOC_061808	OAS1X;OAS1Z	chr17	63676410	63677210	0.0	0.0	0.0	1.61389	2.19578	2.44895	2.13347	1.23464	1.37539
XLOC_061809	OAS1X;OAS1Z	chr17	63677435	63678948	0.0	0.0	0.0	0.95677	0.794741	1.38693	1.02236	1.12533	0.699971
XLOC_041931	OAZ2;ZNF609	chr10	45376559	45486510	7.93024	24.0723	18.9069	23.6372	24.1468	21.4533	25.2121	14.0567	14.367
XLOC_042679	OAZ2;ZNF609	chr10	45376559	45486510	0.0	0.397831	1.04043	0.23844	0.0	0.137686	0.0	0.132356	0.0
XLOC_042680	OAZ2;ZNF609	chr10	45376559	45486510	0.0	0.323999	0.0	0.194192	0.16914	0.224557	0.0	0.0	0.189192
XLOC_042681	OAZ2;ZNF609	chr10	45376559	45486510	0.0	0.406802	0.0	0.365723	0.211952	0.281537	0.366858	0.135295	0.118708
XLOC_042682	OAZ2;ZNF609	chr10	45376559	45486510	0.0	0.413801	1.08219	0.372014	0.107782	0.0	0.0	0.137586	0.0
XLOC_042683	OAZ2;ZNF609	chr10	45376559	45486510	0.665664	0.398251	1.56238	0.358051	0.208518	0.484095	0.0605925	0.333732	0.290977
XLOC_042684	OAZ2;ZNF609	chr10	45376559	45486510	1.29904	0.0	0.0	0.407604	0.406977	0.202459	0.236553	0.195423	0.170372
XLOC_042685	OAZ2;ZNF609	chr10	45376559	45486510	0.0	0.472508	1.23571	0.283193	0.122903	0.163317	0.565983	0.470276	0.0
XLOC_042686	OAZ2;ZNF609	chr10	45376559	45486510	0.0	0.509184	0.0	0.762923	0.793968	0.175881	0.456467	0.0	0.296836
XLOC_042687	OAZ2;ZNF609	chr10	45376559	45486510	2.85039	2.55607	3.3424	0.893648	2.66234	2.79998	3.06999	1.27423	1.98793
XLOC_065716	OBFC2A	chr2	80617216	80642926	5.60873	3.30355	4.22031	9.30585	9.10042	10.0005	9.47084	10.0326	9.20891
XLOC_065717	OBFC2A	chr2	80644050	80659299	6.72631	7.60515	7.09612	17.3651	14.464	17.6325	16.6409	21.5115	14.5492
XLOC_068020	OBFC2A	chr2	80617216	80642926	0.0	0.0	2.51347	0.576153	0.741655	1.31797	1.39993	0.626448	0.837097
XLOC_068021	OBFC2A	chr2	80617216	80642926	0.0	0.0	2.4682	1.4141	2.46391	1.3084	0.855659	1.04987	2.11222
XLOC_068022	OBFC2A	chr2	80617216	80642926	0.0	1.0945	1.43126	1.31201	1.0726	0.379625	0.331579	0.182922	1.35845
XLOC_068023	OBFC2A	chr2	80617216	80642926	2.35786	0.704965	0.921856	2.95767	1.83888	2.44179	2.23215	3.17167	1.85159
XLOC_068024	OBFC2A	chr2	80617216	80642926	1.74501	1.56433	1.36368	1.71885	1.62571	1.26056	3.58128	1.5541	1.6714
XLOC_068025	OBFC2A	chr2	80643218	80643678	2.39115	0.71408	3.73478	2.56775	1.66222	2.70361	2.10885	3.5217	1.24635

XLOC_068026	OBFC2A	chr2	80644050	80659299	0.0	1.2691	1.65944	1.52119	2.46638	1.31225	4.14153	1.67398	2.03189
XLOC_044344	ODC1;HPCAL1	chr11	87127034	87219741	27.6272	31.5718	30.0486	41.1469	41.4081	40.1047	53.1162	43.1539	45.559
XLOC_044438	ODF2	chr11	99040026	99052543	27.7981	9.00754	12.962	7.2195	12.1929	8.30501	8.63775	3.73793	17.08
XLOC_047246	ODF2	chr11	99065635	99066510	0.0	0.0	0.0	0.18067	0.314895	0.522512	0.182342	0.0	0.352039
XLOC_047247	ODF2	chr11	99066657	99067356	0.0	0.0	0.0	0.239316	0.104039	0.27638	0.0	0.0	0.349436
XLOC_070299	ODF2L	chr3	57841838	57847521	14.954	12.5199	11.5344	2.52518	2.5713	2.52039	2.9288	2.07489	2.52814
XLOC_071310	ODF2L	chr3	57840582	57841698	0.754563	1.57988	1.18058	0.202915	0.295245	0.313395	0.479971	0.30231	0.857229
XLOC_071311	ODF2L	chr3	57841838	57847521	3.63764	2.90024	4.74057	0.760489	1.03993	0.878802	0.764781	0.724639	1.05785
XLOC_092060	ODZ1	chrX	8271415	8280175	4.32683	11.7809	9.48081	0.116395	0.271585	0.0900344	0.632868	1.52285	0.757127
XLOC_092726	ODZ1	chrX	8654482	8654673	17.174	0.0	0.0	1508.96	1212.76	1288.57	1289.89	1217.51	1591.22
XLOC_092727	ODZ1	chrX	8662570	8663252	1.379	4.94663	5.39036	11.4884	9.55634	12.8368	8.05184	13.1586	12.0235
XLOC_092728	ODZ1	chrX	8663774	8663910	102.246	0.0	0.0	296.114	239.734	292.467	196.16	157.378	190.773
XLOC_092594	OFD1	chrX	136955109	136976868	17.0323	11.9925	9.39522	10.028	9.35039	9.75729	9.3772	11.7983	15.2226
XLOC_093646	OFD1	chrX	136929993	136930142	0.0	50.8801	44.3919	21.7706	15.7152	33.2824	16.3588	33.2511	14.4249
XLOC_093647	OFD1	chrX	136930388	136930530	74.6753	23.3253	61.09	30.2748	5.32792	22.778	33.2215	44.9744	26.3703
XLOC_093648	OFD1	chrX	136981417	136981810	0.0	6.41559	2.39662	0.823987	0.0	0.314397	0.535235	0.897392	0.532306
XLOC_092595	OFD1;TRAPPC2	chrX	136982058	136993024	0.0	0.147751	0.386437	0.221398	0.348548	0.154082	0.135281	0.0496228	0.0431977
XLOC_062164	OGFOD1;BBS2;<br>NUDT21	chr18	24205300	24286949	22.9552	28.3839	25.6666	24.1145	27.8183	27.0308	33.4972	29.9205	32.6946
XLOC_062804	OGFOD1;BBS2;<br>NUDT21	chr18	24205300	24286949	17.0919	5.0998	2.66706	3.97406	2.35791	3.4931	2.6654	5.30692	2.36757
XLOC_062805	OGFOD1;BBS2;<br>NUDT21	chr18	24205300	24286949	4.2238	5.0468	1.64977	1.8904	1.96177	0.869791	2.24633	1.87251	1.28553
XLOC_062806	OGFOD1;BBS2;<br>NUDT21	chr18	24205300	24286949	10.1229	15.1216	55.3387	33.8989	32.3446	41.6392	47.2051	56.985	39.1178
XLOC_050864	OGFR;C13H20orf<br>20;NTSR1	chr13	55028347	55100279	27.3091	24.9775	23.496	21.0383	20.0294	20.3726	22.5957	23.4463	25.0549
XLOC_093225	OGT	chrX	84401489	84402207	0.0	0.0	0.0	0.693641	1.91043	1.8696	0.348318	0.0	2.25097
XLOC_093226	OGT	chrX	84409426	84409893	0.0	0.0	0.0	1.46406	2.16709	0.961212	1.03158	0.459238	1.01533
XLOC_093227	OGT	chrX	84413363	84414419	0.0	0.0	0.0	0.649363	1.32231	1.58754	0.36539	0.564024	0.773645
XLOC_093228	OGT	chrX	84416825	84417261	0.0	0.0	0.0	2.78847	1.00141	1.06652	0.456431	0.763154	1.35275
XLOC_093229	OGT	chrX	84419830	84422370	0.0	0.0	0.0	1.60617	1.33514	1.86487	1.08486	0.886645	1.40828
XLOC_093230	OGT	chrX	84423075	84423489	0.0	0.0	0.0	2.7747	2.17076	2.02373	1.72765	1.65281	1.95676

XLOC_093231	OGT	chrX	84424038	84424829	0.0	0.0	0.0	4.70579	5.16592	6.38615	2.9872	2.04862	4.88196
XLOC_093232	OGT	chrX	84425027	84426579	0.0	0.0	0.0	3.30159	6.30285	4.96163	2.84014	3.07378	5.39809
XLOC_093233	OGT	chrX	84427226	84428235	0.0	0.0	0.0	0.609045	1.0627	0.793285	0.462291	0.934738	0.593613
XLOC_066990	OLA1	chr2	22588412	22590230	5669.99	6911.68	5591.18	5793.02	5050.86	5636.37	5582.42	5540.82	5996.44
XLOC_066991	OLA1	chr2	22590345	22590535	595.286	779.539	688.447	205.632	289.718	276.746	186.498	193.832	298.282
XLOC_045149	OLFM1	chr11	106654189	106750307	44.1337	26.5724	54.5844	81.2976	103.584	93.707	98.3287	79.5989	124.805
XLOC_047409	OLFM1	chr11	106654189	106750307	0.0	0.945004	0.0	2.26551	1.47483	1.30654	0.565975	1.72434	0.688722
XLOC_047410	OLFM1	chr11	106654189	106750307	0.0	0.0	0.623437	1.21442	0.810504	0.661842	0.289456	0.638268	0.348179
XLOC_047411	OLFM1	chr11	106654189	106750307	0.0	0.270546	0.0	2.27018	1.55557	2.06456	1.06569	2.17061	1.10617
XLOC_069895	OLFM3	chr3	41555687	41616966	31.9133	36.2284	35.0186	83.8807	67.6942	78.1657	93.1944	110.934	86.6775
XLOC_069896	OLFM3	chr3	41555687	41616966	0.0	4.02823	5.26567	26.0609	20.315	20.4448	20.1223	17.1663	9.87675
XLOC_070270	OLFM3	chr3	41454624	41501147	1.8616	1.92945	2.5137	30.3615	36.327	35.4465	21.34	22.1198	35.2403
XLOC_070271	OLFM3	chr3	41510303	41523279	2.20009	3.42624	4.36564	11.6752	15.1431	12.4621	9.23589	10.2602	16.2052
XLOC_070272	OLFM3	chr3	41626771	41742509	7.04678	7.47458	6.00178	5.48363	5.12509	4.96033	6.23399	6.36763	7.16984
XLOC_070929	OLFM3	chr3	41421261	41422011	1.22072	0.729924	0.0	0.656223	2.18893	1.13746	0.659853	0.243142	2.02343
XLOC_070930	OLFM3	chr3	41422711	41423696	0.0	0.0	0.0	0.313347	1.84496	0.634798	0.316988	0.611901	0.76347
XLOC_070931	OLFM3	chr3	41427863	41428528	0.0	0.0	0.0	0.38299	1.10931	0.147367	0.0	0.141581	0.621212
XLOC_070932	OLFM3	chr3	41429683	41430969	0.0	0.0	1.00285	0.172366	0.953787	0.599334	0.933771	0.0642816	1.28869
XLOC_070933	OLFM3	chr3	41431462	41432269	1.11358	0.0	1.74163	0.299347	1.73786	0.807581	0.502607	0.111046	1.74937
XLOC_070934	OLFM3	chr3	41435743	41436114	0.0	0.0	5.28446	1.21138	2.33644	2.76886	1.17419	0.328702	2.93212
XLOC_070935	OLFM3	chr3	41437395	41437671	0.0	0.0	4.7121	1.08335	6.3843	4.88869	0.0	1.71621	3.6427
XLOC_070936	OLFM3	chr3	41438103	41438616	0.0	0.0	0.0	0.728564	2.52158	1.25757	0.7224	0.200635	0.8849
XLOC_070937	OLFM3	chr3	41439260	41439901	0.0	0.0	0.0	0.535965	1.04735	1.23692	0.938646	0.148481	2.47716
XLOC_070938	OLFM3	chr3	41440930	41441642	0.0	0.779887	0.0	0.350569	2.03251	0.809858	0.938659	0.389311	1.36513
XLOC_070939	OLFM3	chr3	41442532	41443194	0.0	0.42854	1.12075	0.385265	2.67799	1.03765	0.643284	0.712041	2.12462
XLOC_070940	OLFM3	chr3	41443382	41444326	0.919591	0.0	0.719265	0.329668	1.58109	1.14463	0.499849	0.0919203	1.36534
XLOC_070941	OLFM3	chr3	41445404	41446305	0.0	0.290906	0.0	0.435895	1.36786	0.706089	1.05648	0.29152	1.7838
XLOC_070942	OLFM3	chr3	41448339	41448607	0.0	5.78178	5.0387	2.31855	2.91985	3.26321	2.14643	0.0	6.6737
XLOC_070943	OLFM3	chr3	41450470	41450859	0.0	0.932279	0.0	1.39695	2.63925	1.2789	1.35993	0.608212	2.70696
XLOC_070944	OLFM3	chr3	41451684	41452090	0.0	0.868752	0.0	1.3017	3.13463	1.49123	2.28907	0.568056	3.78576
XLOC_070945	OLFM3	chr3	41452777	41453873	0.0	0.230504	0.60286	0.414471	1.74869	0.960127	0.630069	0.231519	1.81823
XLOC_070946	OLFM3	chr3	41454624	41501147	0.0	0.863921	3.38908	1.42391	4.72351	3.13762	1.29658	0.574106	2.39346
XLOC_070947	OLFM3	chr3	41454624	41501147	0.0	1.16652	0.0	2.79649	5.90136	3.42058	4.16511	2.11973	3.56717

XLOC_070948	OLFM3	chr3	41454624	41501147	0.0	0.520189	0.0	2.18236	6.48742	5.74881	3.41758	2.41179	5.9117
XLOC_070949	OLFM3	chr3	41454624	41501147	0.0	1.38512	4.52818	5.18863	8.85325	7.0773	4.59624	1.73144	5.35629
XLOC_070950	OLFM3	chr3	41454624	41501147	0.0	0.361307	0.0	3.46481	7.34964	4.50456	3.48472	1.68518	3.58474
XLOC_070951	OLFM3	chr3	41454624	41501147	0.0	1.62951	0.0	2.93587	8.27066	3.87473	5.97069	2.5986	7.53461
XLOC_070952	OLFM3	chr3	41454624	41501147	0.0	0.0	3.36823	4.63143	9.17587	3.5508	2.86422	2.75971	5.9984
XLOC_070953	OLFM3	chr3	41454624	41501147	0.0	0.965488	0.0	2.31482	5.2142	6.28781	2.24932	1.25827	5.60529
XLOC_070954	OLFM3	chr3	41454624	41501147	0.0	0.0	3.81538	2.91458	5.69027	5.54553	2.03713	0.806264	2.835
XLOC_070955	OLFM3	chr3	41454624	41501147	0.0	1.40045	0.0	2.3081	3.44215	3.85713	2.27652	1.38205	3.66695
XLOC_070956	OLFM3	chr3	41454624	41501147	0.0	5.00769	0.0	6.80223	11.2839	7.59378	4.78468	8.59604	7.2088
XLOC_070957	OLFM3	chr3	41454624	41501147	0.0	0.0	0.0	6.95491	16.239	10.9786	4.3792	0.0	3.76169
XLOC_070958	OLFM3	chr3	41454624	41501147	0.0	0.516592	0.900743	3.14794	4.05981	4.1883	2.30907	1.56004	3.47304
XLOC_070959	OLFM3	chr3	41454624	41501147	0.0	0.450925	0.589675	2.77028	4.83701	5.16566	1.64392	1.66099	3.62297
XLOC_070960	OLFM3	chr3	41454624	41501147	0.0	1.65773	0.0	5.21605	7.05593	9.68117	2.67525	5.42758	5.78109
XLOC_070961	OLFM3	chr3	41454624	41501147	0.0	1.1807	3.08732	7.78751	9.97401	8.47118	3.05958	4.96415	4.79044
XLOC_070962	OLFM3	chr3	41454624	41501147	0.0	0.0	0.0	0.672143	1.31859	0.48615	0.4245	0.187374	0.409358
XLOC_070963	OLFM3	chr3	41504531	41506804	1.02561	0.0	0.267559	0.643819	1.15423	1.13885	0.469582	0.447415	0.38897
XLOC_070964	OLFM3	chr3	41509074	41509974	0.0	0.582594	0.761847	2.2697	2.20672	2.22211	1.05788	1.45954	1.19079
XLOC_070965	OLFM3	chr3	41510303	41523279	0.0	0.0	0.0	1.0573	5.34576	2.38719	0.98631	1.67729	2.03239
XLOC_070966	OLFM3	chr3	41510303	41523279	7.9049	0.78672	4.11461	2.3575	2.43767	1.08181	0.694153	1.28987	2.51583
XLOC_070967	OLFM3	chr3	41510303	41523279	3.20739	0.319689	2.50829	2.77832	2.42018	1.66192	1.25565	2.13344	1.39985
XLOC_070968	OLFM3	chr3	41510303	41523279	0.0	0.0	2.07098	3.55954	2.75977	3.02168	1.84019	2.29472	2.46688
XLOC_070969	OLFM3	chr3	41510303	41523279	0.0	0.0	3.26433	4.11772	4.78693	4.26112	2.86572	3.22283	2.89295
XLOC_070970	OLFM3	chr3	41510303	41523279	0.0	0.0	2.46969	5.6609	6.31804	5.82911	3.30428	2.77152	6.30675
XLOC_070971	OLFM3	chr3	41523899	41524158	0.0	0.0	0.0	7.54716	4.20944	4.23837	5.20363	3.29121	1.80728
XLOC_070972	OLFM3	chr3	41524382	41524885	2.09356	1.25077	1.63547	4.31025	2.91753	2.58701	1.8563	3.30073	0.546193
XLOC_070973	OLFM3	chr3	41525832	41526576	1.23321	1.84346	1.92849	2.09927	1.73043	1.14901	0.999677	2.33312	1.29097
XLOC_070974	OLFM3	chr3	41555687	41616966	7.5679	6.7686	5.89835	6.79946	6.80219	5.33255	8.69147	7.08065	6.50178
XLOC_070975	OLFM3	chr3	41617393	41618810	1.14845	0.858937	1.3479	2.6771	3.06014	3.58145	2.61776	2.70921	3.1635
XLOC_070976	OLFM3	chr3	41618862	41620521	0.481674	1.00878	0.753837	2.46177	2.30439	3.65722	1.49575	1.79107	2.57024
XLOC_070977	OLFM3	chr3	41620784	41621650	1.02084	0.610526	1.59674	3.20185	3.50749	3.06883	2.21538	2.34402	2.58458
XLOC_070978	OLFM3	chr3	41622693	41623309	0.0	0.471473	0.0	1.83672	2.20748	1.30372	0.988357	1.09496	1.92424
XLOC_069834	OLFML2B;NOS1AP	chr3	7366659	7553832	8.84167	13.3134	5.90473	17.772	22.5025	19.0155	15.7698	13.7792	18.8348

XLOC_070617	OLFML2B;NOS1AP	chr3	7366659	7553832	0.0	0.285534	0.0	0.256708	0.149197	0.198036	0.0	0.286195	0.333516
XLOC_070618	OLFML2B;NOS1AP	chr3	7366659	7553832	0.0	0.57704	0.0	0.0	0.149726	0.199093	0.0	0.0	0.504196
XLOC_070619	OLFML2B;NOS1AP	chr3	7366659	7553832	0.0	0.0	1.65459	0.568778	0.327904	0.218083	0.0	0.208651	0.184189
XLOC_070620	OLFML2B;NOS1AP	chr3	7366659	7553832	0.0	0.0	0.0	0.0	0.106971	0.142098	0.0	0.136557	0.0
XLOC_070621	OLFML2B;NOS1AP	chr3	7366659	7553832	0.0	0.559302	0.0	0.167616	0.0976441	0.194292	0.11354	0.0	0.0
XLOC_070622	OLFML2B;NOS1AP	chr3	7366659	7553832	1.86188	0.556281	1.45478	0.166697	0.144409	0.0	0.165777	0.183975	0.0
XLOC_070623	OLFML2B;NOS1AP	chr3	7366659	7553832	1.18477	0.0	0.0	0.0	0.0	0.0	0.0	0.0	0.206744
XLOC_069965	OMA1	chr3	88043050	88055996	5.40384	4.41469	6.26577	15.5886	14.0315	15.2656	19.9772	30.4757	16.8826
XLOC_069966	OMA1	chr3	88081997	88121045	2.74262	7.05053	3.16931	24.1044	24.2475	27.0698	19.1117	22.747	21.1837
XLOC_070351	OMA1	chr3	88056477	88058613	32.6494	48.256	29.2097	47.8164	51.0924	46.9672	53.8668	46.2007	60.076
XLOC_071562	OMA1	chr3	88043050	88055996	2.1776	0.814347	2.12988	2.34289	2.30445	2.37724	1.93688	3.11612	1.90447
XLOC_071563	OMA1	chr3	88043050	88055996	1.32766	1.78718	1.55808	3.15407	3.5351	3.65524	4.10884	4.79261	2.43732
XLOC_071564	OMA1	chr3	88043050	88055996	1.96287	0.586419	0.0	2.46018	1.97765	2.83205	1.39564	3.48703	1.36631
XLOC_041541	ONECUT1	chr10	57632104	57736427	1.72011	0.0	0.0	0.308065	0.0	0.0	0.0	0.0	0.0
XLOC_042728	ONECUT1	chr10	57736521	57737054	0.0	0.0	0.0	0.0	0.0	0.198563	0.0	0.0	0.0
XLOC_037909	OPA1	chr1	74383631	74385673	7.06206	1.05573	4.60179	1.89827	2.47848	1.34081	1.59185	1.05553	2.87559
XLOC_037910	OPA1	chr1	74391863	74397128	20.018	16.2001	19.3586	3.53209	3.17887	3.62892	3.60473	5.19006	3.48508
XLOC_037911	OPA1	chr1	74409222	74435542	1.91506	2.07664	1.42341	1.3813	1.84528	1.80165	2.14046	1.47907	2.40539
XLOC_038411	OPA1	chr1	74435608	74505927	44.9164	45.8245	45.1348	39.7424	48.0795	44.9474	56.212	53.757	57.8955
XLOC_039667	OPA1	chr1	74391283	74391720	2.58785	3.09074	0.0	2.31543	0.399159	2.12552	0.454873	1.01401	1.79727
XLOC_039668	OPA1	chr1	74391863	74397128	0.0	0.0	0.0	1.45707	1.23175	1.09908	0.456149	0.0	1.86992
XLOC_039669	OPA1	chr1	74397383	74397775	0.0	3.68162	2.4068	0.827488	0.710816	0.315712	0.537393	0.300359	0.267275
XLOC_063241	OPA3;OPA3	chr18	53580839	53581692	0.0	0.0	0.0	0.0	0.0	0.107787	0.0	0.0	0.0
XLOC_065855	OPRD1	chr2	125247129	125257733	46.6005	56.2476	39.9908	274.515	235.776	261.365	308.908	332.916	253.533
XLOC_053502	OPRK1	chr14	23367467	23425740	5.04043	4.51882	3.93925	0.0	0.130508	0.0	0.0	0.0	0.0
XLOC_083389	OR10H1	chr7	8088024	8289128	0.0	0.0	0.0	0.0	0.11709	0.0	0.0	0.0	0.0
XLOC_084165	OR10H1	chr7	8088024	8289128	1.42801	0.42685	0.0	0.383746	0.666884	0.295312	0.0	0.141856	0.0
XLOC_061633	ORAI1	chr17	55864435	55865233	10.163	2.02583	2.64912	1.92248	1.76206	2.10561	0.815237	1.46361	1.47821
XLOC_062366	ORC6;SHCBP1;VPS35	chr18	14969350	15152045	5.13534	4.47435	5.61203	1.44235	1.54855	1.96786	1.3399	0.973226	1.24089

XLOC_062719	ORC6;SHCBP1;VPS35	chr18	14969350	15152045	4.1274	6.16478	1.61219	14.7788	12.4652	14.4532	14.8295	16.6809	14.8974
XLOC_062720	ORC6;SHCBP1;VPS35	chr18	14969350	15152045	0.0	0.0	0.0	0.0	0.0	0.0509791	0.0	0.0	0.0
XLOC_062721	ORC6;SHCBP1;VPS35	chr18	14969350	15152045	0.0	0.0	0.0	0.0	0.0	0.0	0.0	0.122385	0.0
XLOC_086611	ORM1;COL27A1	chr8	105169823	105218161	63.3289	61.7924	57.2591	28.093	32.8483	29.9304	36.6168	32.8562	30.8197
XLOC_063996	ORMDL3	chr19	40911116	40918240	23.6352	33.335	29.3955	35.1141	35.2724	35.9438	45.4688	42.4815	47.1289
XLOC_065139	ORMDL3	chr19	40909285	40909652	0.0	2.05928	2.69237	2.46881	2.90863	4.23086	2.39049	2.67773	3.58478
XLOC_065140	ORMDL3	chr19	40910146	40910948	3.36666	0.671091	0.0	1.00556	0.437805	0.813803	0.911567	0.671377	0.881444
XLOC_063995	ORMDL3;GSDMB	chr19	40893890	40908323	11.3802	14.8536	19.8621	19.295	15.8736	16.9363	20.8719	20.1722	19.7969
XLOC_065137	ORMDL3;GSDMB	chr19	40893890	40908323	1.21662	2.54616	6.65898	3.59713	4.36328	5.29045	3.39803	3.99855	4.67014
XLOC_065138	ORMDL3;GSDMB	chr19	40893890	40908323	0.0	9.84993	12.8766	7.88795	7.08092	7.2487	6.01201	3.66388	5.21828
XLOC_059892	OSBP2	chr17	71782768	71857541	6.18032	5.40732	3.72144	2.23851	3.62386	2.40198	3.39141	2.76884	2.64314
XLOC_061896	OSBP2	chr17	71782768	71857541	2.19351	0.65586	1.71525	1.3758	2.13968	1.36357	2.27764	0.984466	1.72296
XLOC_050574	OSBPL2	chr13	55445498	55461318	11.9967	12.3253	11.3276	54.0146	35.0815	42.1904	47.11	65.2073	33.4117
XLOC_050575	OSBPL2	chr13	55464315	55473272	5.58377	4.27293	5.40404	2.97127	2.39932	3.05363	2.09814	3.25149	1.4842
XLOC_050868	OSBPL2	chr13	55445498	55461318	0.0	0.360493	2.1979	1.18416	1.40199	0.989732	0.712925	0.849785	0.751936
XLOC_050869	OSBPL2	chr13	55473298	55488078	14.4339	15.8897	11.6332	5.30937	4.63014	5.78648	6.07179	6.63421	5.38084
XLOC_051887	OSBPL2	chr13	55462118	55463405	0.0	0.766191	2.00392	1.2629	1.15357	0.998009	0.641406	0.513801	0.391863
XLOC_051888	OSBPL2	chr13	55463526	55464179	0.0	0.436314	0.0	1.43826	1.02228	1.05633	0.523725	1.01464	0.636179
XLOC_051889	OSBPL2	chr13	55464315	55473272	1.04443	0.624619	2.45039	1.3103	0.89697	0.757756	0.849595	1.04239	0.911751
XLOC_051890	OSBPL2	chr13	55464315	55473272	0.0	0.812068	3.18569	0.730066	0.423123	1.68608	0.854417	1.08035	0.829064
XLOC_051891	OSBPL2	chr13	55464315	55473272	10.4521	0.0	0.0	1.24625	2.66898	0.711789	0.904543	0.675614	0.0
XLOC_070036	OSBPL9	chr3	95137320	95140232	0.667333	0.500122	0.0	0.717965	0.288803	0.625264	0.489425	0.52103	0.468338
XLOC_070037	OSBPL9	chr3	95160467	95174141	24.8563	28.8838	25.2009	7.26952	7.70428	5.14409	6.74947	9.0199	8.70048
XLOC_070038	OSBPL9	chr3	95219494	95251223	80.1489	73.0505	65.4577	39.1666	37.0876	41.4037	41.141	38.0791	36.6651
XLOC_070039	OSBPL9	chr3	95254611	95296568	7.99118	5.80062	6.05499	34.4159	28.1906	33.1109	29.205	35.0824	32.8211
XLOC_070409	OSBPL9	chr3	95128760	95134990	11.0515	18.3657	9.83879	31.3355	30.4489	31.752	31.376	32.2449	38.6296
XLOC_070410	OSBPL9	chr3	95188193	95218387	11.8635	12.078	14.4606	14.5033	13.802	14.9707	15.938	16.9495	13.3723
XLOC_070411	OSBPL9	chr3	95219494	95251223	4.08784	4.00958	4.11285	7.09605	7.5528	7.15844	6.05399	6.95973	7.13038
XLOC_070412	OSBPL9	chr3	95219494	95251223	0.909038	0.957875	1.13936	1.92235	0.923972	0.449344	0.0144904	0.0	1.20698

XLOC_070413	OSBPL9	chr3	95219494	95251223	0.709623	0.95532	2.49581	4.60684	3.38597	4.5286	1.65309	1.72067	1.84786
XLOC_070414	OSBPL9	chr3	95254611	95296568	15.5477	23.0283	14.6779	43.444	37.4266	38.0723	34.7681	41.4032	36.005
XLOC_071715	OSBPL9	chr3	95135374	95136022	0.0	0.440755	0.0	0.660409	0.229461	0.304858	0.39669	0.585601	0.257052
XLOC_071716	OSBPL9	chr3	95144264	95144962	6.69086	8.40066	6.2772	8.39154	8.75531	10.9371	10.4659	10.2469	12.0194
XLOC_071717	OSBPL9	chr3	95181872	95182551	8.32159	7.04799	11.9269	8.94534	7.23503	11.7625	8.34612	8.27064	8.82742
XLOC_071718	OSBPL9	chr3	95182670	95182986	18.2065	16.2857	21.2915	24.8317	25.6167	23.1273	27.501	22.6937	28.6766
XLOC_071719	OSBPL9	chr3	95183086	95183456	6.80468	8.12151	2.65459	5.78103	7.82376	10.0833	6.19176	5.28297	7.36433
XLOC_071720	OSBPL9	chr3	95183610	95183821	12.2656	7.34235	0.0	17.8733	12.6909	9.82768	12.6583	7.85131	11.5871
XLOC_071721	OSBPL9	chr3	95183966	95184219	51.9218	17.6969	28.9158	21.3271	18.9084	17.9323	14.6301	18.0685	21.6775
XLOC_071722	OSBPL9	chr3	95184325	95184454	0.0	50.9877	0.0	33.9277	112.86	32.8198	11.7282	81.3267	114.494
XLOC_071723	OSBPL9	chr3	95184715	95185614	15.6065	14.5845	9.15449	18.5317	15.0867	18.2052	17.6547	19.0967	14.9049
XLOC_071724	OSBPL9	chr3	95185699	95185886	0.0	5.59607	14.6298	25.8546	14.9445	26.0274	30.2467	13.3471	19.1971
XLOC_071725	OSBPL9	chr3	95186333	95187037	10.5878	5.53903	6.20837	12.805	10.3098	11.6396	14.043	12.769	11.8881
XLOC_071726	OSBPL9	chr3	95219066	95219395	21.0517	11.3005	0.0	13.9306	13.7996	13.2843	24.13	16.6083	16.3658
XLOC_071727	OSBPL9	chr3	95219494	95251223	0.0	3.63536	0.0	3.8134	2.57439	3.11815	4.51337	2.96711	5.54288
XLOC_071728	OSBPL9	chr3	95219494	95251223	0.0	2.74615	1.43646	3.45648	2.28172	1.51675	1.47373	1.81693	2.24012
XLOC_071729	OSBPL9	chr3	95219494	95251223	19.2775	20.1825	20.4679	13.9485	14.1235	13.5166	14.9081	16.5635	15.5866
XLOC_071730	OSBPL9	chr3	95253474	95254463	11.3077	13.7875	12.2467	34.6923	20.6737	28.2487	25.5545	35.0604	25.9095
XLOC_066129	OSGEPL1;ASNSD1;SLC40A1;WDR75	chr2	6541515	7083686	18.0139	18.5376	16.4961	0.536331	0.460962	0.551207	0.557297	0.247416	0.627818
XLOC_066820	OSGEPL1;ASNSD1;SLC40A1;WDR75	chr2	6541515	7083686	2.38519	4.9917	2.79758	0.0	0.0	0.246977	0.0	0.0	0.10405
XLOC_066821	OSGEPL1;ASNSD1;SLC40A1;WDR75	chr2	6541515	7083686	1.22084	6.2044	4.77259	0.0	0.0	0.0	0.0	0.0	0.0
XLOC_066822	OSGEPL1;ASNSD1;SLC40A1;WDR75	chr2	6541515	7083686	4.82444	2.40494	6.91884	0.0	0.125795	0.0834625	0.0	0.0804932	0.0702542
XLOC_066823	OSGEPL1;ASNSD1;SLC40A1;WDR75	chr2	6541515	7083686	4.86228	3.87804	6.33916	0.217911	0.126775	0.0841138	0.147129	0.0	0.141606
XLOC_044292	OSR1	chr11	79339942	79581539	22.4767	23.8445	24.5796	4.70194	4.91991	4.95464	6.67977	6.65689	4.67379
XLOC_044941	OSR1	chr11	79339942	79581539	7.22475	3.02219	9.60356	0.841294	0.564566	0.524832	0.131455	0.57832	0.630291
XLOC_079609	OSTC;AGXT2L1;RPL34	chr6	17694150	17942979	5.7419	6.29374	3.94972	2.03132	1.75652	1.68804	1.63755	0.575185	1.74597

XLOC_080562	OSTC;AGXT2L1;RPL34	chr6	17694150	17942979	1.25243	0.374443	0.0	1.23429	0.976189	1.68535	2.0298	1.62085	1.311
XLOC_044224	OTOF	chr11	72999082	73008994	0.0	0.0	0.0	0.0	0.0	0.150004	0.130164	0.0	0.0
XLOC_044225	OTOF	chr11	73009217	73014368	0.876143	0.0	0.0	0.0785265	0.136993	0.0	0.158872	0.0	0.0765314
XLOC_044226	OTOF	chr11	73025892	73042051	0.0	0.694793	0.0	0.994857	0.891829	1.15537	0.662476	0.727923	0.993731
XLOC_044227	OTOF	chr11	73047688	73081733	8.75936	10.9716	8.26785	18.1431	19.6041	17.9195	20.001	20.7525	22.0622
XLOC_038497	OTOL1	chr1	106543152	106714259	3.59231	3.71655	3.32379	0.1124	0.0984755	0.182746	0.388811	0.0	0.061946
XLOC_038498	OTOL1	chr1	106723488	106796714	42.6895	31.9666	31.5482	36.8251	42.962	40.7024	48.5781	41.1271	47.2869
XLOC_040041	OTOL1	chr1	106543152	106714259	0.982026	1.46834	0.0	0.0	0.0	0.0	0.0	0.0	0.0
XLOC_040043	OTOL1	chr1	106543152	106714259	2.34465	0.0	0.0	0.0	0.0	0.0	0.0	0.0	0.0
XLOC_040046	OTOL1	chr1	106543152	106714259	0.0	0.408332	1.06791	0.0	0.106372	0.0	0.0	0.0	0.0
XLOC_040047	OTOL1	chr1	106543152	106714259	0.0	0.702677	1.83773	0.0	0.0	0.0	0.0	0.0	0.0
XLOC_040048	OTOL1	chr1	106543152	106714259	0.0	1.05465	0.0	0.0	0.0	0.0	0.0	0.0	0.0
XLOC_040049	OTOL1	chr1	106543152	106714259	0.0	0.958767	1.25378	0.0	0.0	0.0	0.0	0.0	0.0
XLOC_040050	OTOL1	chr1	106543152	106714259	1.41682	0.0	0.0	0.0	0.0	0.0	0.0	0.0	0.0
XLOC_040051	OTOL1	chr1	106543152	106714259	0.0	0.941001	0.0	0.0	0.0	0.0	0.0	0.0	0.0
XLOC_040052	OTOL1	chr1	106543152	106714259	0.0	0.186273	0.974371	0.0558239	0.0	0.0	0.0	0.0	0.0
XLOC_040053	OTOL1	chr1	106543152	106714259	0.0	0.267492	1.57416	0.0	0.0	0.0	0.0	0.0	0.0
XLOC_040054	OTOL1	chr1	106543152	106714259	0.409413	0.735007	0.961195	0.0	0.0	0.0426029	0.0	0.0	0.0
XLOC_040055	OTOL1	chr1	106543152	106714259	0.0	0.847019	1.1076	0.0	0.0	0.0	0.0	0.0	0.0
XLOC_040056	OTOL1	chr1	106543152	106714259	0.0	0.778161	0.339209	0.0	0.0	0.0	0.0	0.0435879	0.0
XLOC_040057	OTOL1	chr1	106543152	106714259	1.37376	0.410655	4.29593	0.123063	0.0	0.0	0.0	0.0	0.119781
XLOC_040058	OTOL1	chr1	106543152	106714259	0.925737	0.0	1.44884	0.0	0.0363076	0.0	0.0	0.0	0.0
XLOC_040059	OTOL1	chr1	106543152	106714259	0.862558	1.54783	0.674697	0.0	0.0	0.0	0.0	0.0	0.0
XLOC_040060	OTOL1	chr1	106543152	106714259	0.0	0.838492	0.0	0.0	0.0	0.0	0.0	0.0	0.0
XLOC_040062	OTOL1	chr1	106543152	106714259	0.354436	0.212114	1.10956	0.0	0.0	0.0	0.0	0.0	0.0
XLOC_040063	OTOL1	chr1	106543152	106714259	0.43165	0.387456	1.01338	0.0	0.0	0.0	0.0	0.0	0.0377661
XLOC_040064	OTOL1	chr1	106543152	106714259	0.441668	0.660739	2.07377	0.0	0.0	0.0	0.0403701	0.0	0.0
XLOC_040065	OTOL1	chr1	106543152	106714259	0.612138	0.366244	1.43683	0.0	0.0	0.0	0.0	0.0	0.0
XLOC_040066	OTOL1	chr1	106543152	106714259	1.45385	1.73825	1.1365	0.0	0.113135	0.0	0.0	0.0	0.0
XLOC_040067	OTOL1	chr1	106543152	106714259	0.0	0.527254	1.37897	0.0	0.0	0.0	0.0	0.0	0.0
XLOC_040068	OTOL1	chr1	106543152	106714259	1.47937	0.221331	2.31554	0.0	0.0	0.0	0.0	0.0	0.0
XLOC_040069	OTOL1	chr1	106543152	106714259	0.0	0.471473	1.23302	0.0	0.0	0.0	0.0	0.0	0.0

XLOC_040070	OTOL1	chr1	106543152	106714259	1.44177	0.862827	1.69253	0.0	0.0	0.0	0.0	0.0	0.0
XLOC_040071	OTOL1	chr1	106543152	106714259	0.0	3.7239	3.24574	0.0	0.0	0.0	0.0	0.0	0.0
XLOC_040072	OTOL1	chr1	106543152	106714259	0.0	2.4826	3.24574	0.0	0.0	0.0	0.0	0.0	0.0
XLOC_040073	OTOL1	chr1	106543152	106714259	1.03103	1.84984	0.80633	0.0	0.0	0.0	0.0	0.0	0.0
XLOC_040074	OTOL1	chr1	106543152	106714259	0.564809	0.506914	1.76774	0.0	0.0442674	0.0	0.0	0.0	0.0
XLOC_040075	OTOL1	chr1	106543152	106714259	0.0	1.45453	0.760824	0.0871789	0.0	0.0	0.0	0.0	0.0
XLOC_040076	OTOL1	chr1	106543152	106714259	1.31936	1.05268	0.688315	0.0	0.0345035	0.0	0.160795	0.0	0.0769538
XLOC_040077	OTOL1	chr1	106543152	106714259	2.46222	1.84032	2.88781	0.0	0.0	0.0	0.110894	0.0	0.0
XLOC_040078	OTOL1	chr1	106543152	106714259	1.20457	1.08156	2.09544	0.0	0.0	0.0418536	0.0123043	0.0	0.0351686
XLOC_040079	OTOL1	chr1	106717268	106718279	2.54219	0.0	0.662849	0.0	0.463851	0.175875	0.384354	0.254337	0.814311
XLOC_040080	OTOL1	chr1	106720743	106723198	4.40702	3.20269	2.4637	3.24647	4.52405	3.50694	2.85517	2.53606	3.85745
XLOC_040081	OTOL1	chr1	106723488	106796714	1.20461	3.60105	3.76716	2.26622	1.31481	0.997704	2.06231	1.4397	2.73217
XLOC_040082	OTOL1	chr1	106723488	106796714	2.36457	2.29891	1.85005	0.582964	0.787439	0.860172	0.754305	0.711918	1.08546
XLOC_040083	OTOL1	chr1	106723488	106796714	3.83238	4.5797	0.0	1.20082	0.594214	0.0	0.8522	2.64897	1.66736
XLOC_040084	OTOL1	chr1	106723488	106796714	2.88757	0.0	4.50738	1.29134	0.666466	1.18362	1.00966	0.281839	0.751172
XLOC_040085	OTOL1	chr1	106723488	106796714	3.7808	3.16637	2.36609	1.35559	1.95267	1.25619	1.44288	0.833075	1.45372
XLOC_040086	OTOL1	chr1	106723488	106796714	13.8846	22.7688	21.6457	13.0843	13.5586	7.00007	10.3186	3.91492	10.7447
XLOC_040087	OTOL1	chr1	106723488	106796714	40.3516	17.1836	13.4781	9.80815	13.9329	6.41457	8.20226	10.387	12.9053
XLOC_079844	OTOP1	chr6	107529754	107570181	1.4372	1.59756	0.482126	0.165732	0.258215	0.213916	0.226188	0.0414084	0.143826
XLOC_082122	OTOP1	chr6	107529754	107570181	0.0	0.473615	0.619346	0.28387	0.0619395	0.246569	0.0	0.0792646	0.0691798
XLOC_082123	OTOP1	chr6	107529754	107570181	0.0	0.0	0.0	0.0	0.1398	0.0	0.160584	0.0	0.0
XLOC_082124	OTOP1	chr6	107529754	107570181	0.0	0.217971	0.570083	0.130646	0.0	0.151354	0.1325	0.0	0.0636876
XLOC_082125	OTOP1	chr6	107529754	107570181	0.0	0.0	3.48365	0.0	0.0	0.0	0.0	0.0	0.0
XLOC_050813	OVOL2	chr13	38602284	38635299	3.25411	4.12314	2.03745	3.50203	3.78593	2.94748	2.98077	3.6491	4.4113
XLOC_051664	OVOL2	chr13	38600307	38601239	0.93384	0.0	0.0	0.0836927	0.802705	0.387422	0.338307	0.279985	0.407771
XLOC_038110	OXNAD1	chr1	155158703	155205499	29.1532	37.377	36.7592	11.0714	10.5906	10.2719	7.97073	8.94623	8.89502
XLOC_040665	OXNAD1	chr1	155096047	155099703	22.326	16.7657	16.5047	2.16929	0.877444	1.22767	0.854001	0.812989	1.1766
XLOC_053655	OXR1;ABRA	chr14	59646883	60178273	0.908085	1.31359	0.947819	0.0474294	0.0357051	0.11109	0.0139087	0.0	0.0265107
XLOC_054549	OXR1;ABRA	chr14	59646883	60178273	1.33324	0.0	0.0	0.716626	0.519249	0.275875	0.599322	0.265189	1.27891
XLOC_060251	P2RX4	chr17	56176188	56184243	12.2884	10.3291	10.9788	22.7815	24.6838	27.3234	25.1335	28.1343	30.9625
XLOC_061640	P2RX4	chr17	56173129	56173537	0.0	1.72368	0.0	0.258268	0.444311	0.591812	0.252415	0.0	0.250391
XLOC_061641	P2RX4	chr17	56173695	56173940	0.0	0.0	0.0	0.723632	1.20297	0.0	0.0	0.750429	0.690931
XLOC_061642	P2RX4	chr17	56174376	56175241	0.0	0.611388	0.799498	0.183221	0.31931	0.317911	0.369743	0.102056	0.178498

XLOC_060252	P2RX4;P2RX7	chr17	56186629	56218503	7.7091	8.52988	9.0627	11.6036	9.36879	10.7325	9.93944	14.1994	6.507
XLOC_060253	P2RX7	chr17	56269360	56276758	4.97059	3.75782	3.48096	1.24351	1.39768	0.899166	0.911696	0.922699	1.21386
XLOC_061643	P2RX7	chr17	56256616	56260192	7.82772	5.76152	9.10782	8.76635	6.31886	7.36186	7.63212	8.70363	6.83565
XLOC_061644	P2RX7	chr17	56260308	56260969	10.0556	3.86451	3.36892	2.05882	1.34162	1.63381	1.93358	2.14029	1.62792
XLOC_061645	P2RX7	chr17	56261086	56261741	1.45385	6.08387	5.68248	3.90678	2.2627	1.95394	3.78215	2.02127	1.64745
XLOC_061646	P2RX7	chr17	56262192	56262976	0.0	2.41623	6.31924	2.89636	1.44086	2.39148	1.97854	2.07119	1.41037
XLOC_061647	P2RX7	chr17	56263040	56263532	4.32483	10.9804	8.44569	3.09683	2.50986	2.89354	3.82993	2.76774	3.19609
XLOC_061648	P2RX7	chr17	56264925	56265577	0.0	1.31158	1.14338	0.52406	1.25195	0.604828	0.262378	0.580948	0.509967
XLOC_053378	PABPC1	chr14	65805617	65817861	14.3078	15.3464	14.9118	26.746	34.5164	30.4157	29.2756	24.8216	45.5206
XLOC_053674	PABPC1	chr14	65818989	65834115	15.0852	25.754	19.3563	96.7456	119.759	99.9965	98.4321	92.5707	167.32
XLOC_054694	PABPC1	chr14	65818670	65818877	0.0	7.80749	0.0	53.5361	59.6212	48.2793	50.5056	38.0309	87.3196
XLOC_091575	PACRG	chr9	99668785	99668990	0.0	4.03015	10.5345	1.22924	7.93227	9.42434	6.36975	3.67408	4.6216
XLOC_091576	PACRG	chr9	99669185	99669482	0.0	0.0	0.0	0.92347	1.56413	4.18483	0.870678	0.491939	2.66866
XLOC_091577	PACRG	chr9	99674128	99674901	0.0	0.0	0.0	0.210576	0.274944	0.243391	0.0	0.117093	0.102533
XLOC_089579	PACRG;PARK2	chr9	99629546	99668369	2.7504	3.78819	3.0075	4.25021	5.84005	4.33666	6.24878	4.34815	5.75488
XLOC_091574	PACRG;PARK2	chr9	99629546	99668369	0.0	0.0	0.0	0.179139	0.310049	0.0	0.177711	0.197385	0.0
XLOC_076483	PACSIN2	chr5	114362594	114421281	16.7363	19.0615	16.8937	16.262	12.9671	13.2051	13.4788	16.9613	12.5249
XLOC_076484	PACSIN2	chr5	114444272	114460663	5.70327	8.17921	6.24418	12.4804	8.5254	11.3055	10.395	13.1467	9.70915
XLOC_076999	PACSIN2	chr5	114362594	114421281	23.992	31.0108	33.8874	11.0228	9.82285	11.8274	10.6106	10.8834	11.0712
XLOC_077000	PACSIN2	chr5	114430923	114444037	2.48762	4.71863	5.60035	10.033	14.8681	12.3861	9.75985	10.0041	15.7425
XLOC_078772	PACSIN2	chr5	114359577	114360298	0.0	3.83723	3.01065	0.0	0.0	0.0	0.0	0.0	0.111952
XLOC_078773	PACSIN2	chr5	114362594	114421281	4.59257	6.40558	7.17929	1.91948	1.78577	1.26572	1.6456	1.82249	1.20056
XLOC_078774	PACSIN2	chr5	114430923	114444037	3.48216	2.49986	1.63454	4.74478	6.10686	6.2935	4.30802	2.51298	5.23513
XLOC_078775	PACSIN2	chr5	114430923	114444037	2.88343	1.14972	2.2552	2.15343	1.12635	2.09308	1.30509	1.1523	1.00716
XLOC_078776	PACSIN2	chr5	114474388	114474880	0.0	0.0	0.0	1.93552	0.501973	0.44516	0.0	0.212903	0.188006
XLOC_064211	PAFAH1B1	chr19	24094610	24225273	33.8	40.6862	43.6918	84.3462	51.7122	71.8165	70.679	117.43	49.4242
XLOC_055411	PAFAH1B2	chr15	28229429	28295449	0.182561	0.218436	0.142831	0.0327323	0.0143454	0.0190141	0.0167589	0.0736229	0.0
XLOC_076354	PAH	chr5	66745855	66999387	4.68134	0.709678	2.46996	0.426135	0.225062	0.165103	0.17481	0.347579	0.0418568
XLOC_076355	PAH	chr5	67002255	67056057	1.08935	1.17362	1.02321	0.195406	0.0684946	0.158886	0.19997	0.153769	0.152615
XLOC_078075	PAH	chr5	66745855	66999387	43.5446	68.481	37.048	36.8137	38.9893	46.3898	45.2138	46.5867	31.4798
XLOC_092143	PAK3	chrX	64496607	64497927	16.9292	6.06816	13.3903	51.5041	51.3843	45.7856	83.4353	41.1228	53.6979

XLOC_088638	PALM2	chr8	101108410	101109345	3.72095	3.89473	5.09308	4.08513	4.5797	3.95584	4.2128	4.27672	4.38699
XLOC_088639	PALM2	chr8	101109443	101110165	7.68764	8.81	9.01598	10.6753	11.482	11.9344	10.1466	8.16047	10.9519
XLOC_083154	PAM	chr7	104244501	104390173	1.20857	1.98188	2.72061	6.36631	9.75959	5.47457	4.48961	3.19168	7.5149
XLOC_083801	PAM	chr7	104244501	104390173	13.6543	9.12666	12.0555	5.8598	7.24226	6.58921	4.68009	3.26156	8.89543
XLOC_083802	PAM	chr7	104411066	104412379	1.13454	0.0	0.0	0.101659	0.0	0.0	0.204748	0.0	0.0990093
XLOC_083803	PAM	chr7	104420831	104465174	0.51192	0.303826	0.400538	0.0910449	0.238864	0.211194	0.0465455	0.0	0.0894366
XLOC_083804	PAM	chr7	104471885	104473236	0.0	0.638435	0.0	0.0	0.0	0.0	0.0	0.0	0.0
XLOC_083805	PAM	chr7	104500755	104502017	16.446	17.8879	17.5441	5.6958	4.97276	4.81265	6.79382	8.08714	5.29164
XLOC_085525	PAM	chr7	104244501	104390173	1.56728	0.468413	1.22503	0.140376	1.46222	0.323831	0.140324	0.621714	0.409672
XLOC_085526	PAM	chr7	104244501	104390173	8.01726	4.4518	9.85171	2.46297	3.21651	2.96585	1.75657	0.685015	1.99892
XLOC_085527	PAM	chr7	104409029	104409718	0.0	0.0	2.12778	0.121906	0.105976	0.140769	0.0	0.0	0.0
XLOC_085528	PAM	chr7	104409790	104410065	6.09532	3.63411	0.0	0.0	0.0	0.615999	0.0	0.0	0.0
XLOC_085529	PAM	chr7	104410162	104410911	3.66835	1.09673	0.0	0.21911	0.0953304	0.253193	0.220315	0.0	0.320027
XLOC_056850	PAMR1	chr15	66781951	66783016	3.98486	3.09884	1.24687	0.0	0.0	0.0	0.0	0.0	0.0
XLOC_077040	PANX2;MIR2894;TRABD	chr5	121153505	121191384	11.3226	14.5194	12.9748	10.3707	9.32994	9.55207	11.5397	10.739	8.66894
XLOC_041843	PAPD4	chr10	10545717	10558239	11.5764	7.26988	4.98182	0.57084	0.226713	0.180429	0.158309	0.232311	0.202461
XLOC_079614	PAPSS1	chr6	18848134	18854384	24.3701	34.7847	29.0016	32.4575	29.4201	29.1976	35.8271	39.3778	39.1239
XLOC_080567	PAPSS1	chr6	18824550	18825320	5.90427	3.88363	1.84672	7.08882	4.51254	5.01378	5.53604	5.76526	4.22437
XLOC_080568	PAPSS1	chr6	18854563	18854822	0.0	0.0	0.0	3.77358	4.73562	5.65115	4.04727	2.63297	3.01214
XLOC_062397	PARD6A;C18H16orf86;C18H16orf48;GFOD2;THAP11;NUTF2;CENPT;RANBP10;CTCF	chr18	35266029	35499159	29.4504	12.071	11.394	15.4459	20.968	15.4094	18.6129	10.0642	24.0061
XLOC_089574	PARK2	chr9	98424773	98446552	31.8524	36.7829	30.0624	3.64155	2.82862	4.21069	2.67773	8.32573	2.87593
XLOC_089575	PARK2	chr9	98563708	98601768	57.859	78.7325	61.2234	54.1352	50.1684	51.6745	58.4973	59.923	62.3324
XLOC_089576	PARK2	chr9	98986368	99002171	13.5319	14.9126	8.3577	16.4327	16.6983	15.1506	19.4336	20.3937	19.6472
XLOC_089577	PARK2	chr9	99243271	99300813	18.2524	18.8469	18.6651	48.3108	55.8604	53.9267	59.9853	44.2777	66.2141
XLOC_089578	PARK2	chr9	99575722	99585036	11.2841	8.81137	7.7659	20.3434	18.9183	17.5267	24.2184	26.3422	22.1282
XLOC_090049	PARK2	chr9	98563708	98601768	13.3208	11.8562	10.5095	10.4978	9.58994	10.3055	11.7967	13.5607	11.877
XLOC_090050	PARK2	chr9	99039139	99071808	6.25806	8.16741	8.07807	7.63318	7.21391	8.67044	9.14115	9.79222	6.0001

XLOC_090051	PARK2	chr9	99073371	99140179	5.70243	5.08722	5.70119	7.19465	5.88072	7.74625	7.69674	10.015	4.45709
XLOC_090052	PARK2	chr9	99229588	99237189	0.0	0.0	1.55038	0.355304	0.153769	0.204482	0.0	0.391598	0.345282
XLOC_090053	PARK2	chr9	99310654	99358582	13.2374	12.1349	22.3778	2.26343	3.83852	2.54935	3.20259	3.36307	4.39859
XLOC_090054	PARK2	chr9	99387387	99437232	31.7429	34.2165	28.6607	8.972	7.16396	9.07536	21.7989	26.0794	17.9477
XLOC_090055	PARK2	chr9	99477950	99568942	32.5972	42.006	33.8156	43.3541	42.1572	45.5891	46.2362	46.961	48.4023
XLOC_091557	PARK2	chr9	98864761	98866229	3.31127	1.48595	0.863647	1.53389	1.68708	1.83603	1.20813	1.82868	1.39965
XLOC_091558	PARK2	chr9	98872750	98873514	4.77015	3.20892	3.72993	3.9534	4.37077	3.82814	5.05214	5.58385	6.03481
XLOC_091559	PARK2	chr9	99034152	99034816	2.85602	0.42685	0.0	0.511661	0.222295	0.442968	0.128162	0.141856	0.37346
XLOC_091560	PARK2	chr9	99034966	99036416	2.79782	1.17182	0.437834	0.652197	0.701731	0.872582	0.459292	0.561811	0.440413
XLOC_091561	PARK2	chr9	99036566	99037177	4.78477	0.0	0.0	1.28557	0.619864	1.15321	0.285411	0.948686	1.38952
XLOC_091562	PARK2	chr9	99037437	99039087	2.42289	2.17471	2.65433	3.04145	2.81228	2.92321	2.8766	3.26272	2.6281
XLOC_091563	PARK2	chr9	99073053	99073258	13.4506	4.03015	0.0	0.0	0.0	0.0	1.06163	2.44939	1.1554
XLOC_091564	PARK2	chr9	99243271	99300813	5.6329	0.0	0.0	7.56514	6.81217	6.27091	7.55622	8.55679	4.36618
XLOC_091565	PARK2	chr9	99303526	99306323	0.547999	0.983935	0.857826	1.37611	2.15254	1.39825	1.08026	0.828213	1.27129
XLOC_091566	PARK2	chr9	99306967	99307308	3.93717	1.17433	0.0	1.05619	4.20912	1.605	2.02937	0.759755	1.36136
XLOC_091567	PARK2	chr9	99309472	99309641	0.0	17.207	0.0	5.38076	20.5101	14.1976	6.46188	2.50168	7.35372
XLOC_091568	PARK2	chr9	99309985	99310126	548.882	269.808	706.693	63.7802	50.3156	87.7625	29.044	53.9144	124.723
XLOC_091569	PARK2	chr9	99310223	99310540	22.6173	14.8364	17.6334	4.45007	5.50545	2.75844	1.15531	3.47069	5.07554
XLOC_091570	PARK2	chr9	99385361	99387270	29.3173	25.0806	21.6504	2.81405	1.68494	2.01941	7.59006	11.5046	4.87752
XLOC_091571	PARK2	chr9	99475516	99476902	0.0	1.23284	0.46063	0.686155	0.369058	0.489533	0.912254	0.354521	0.617744
XLOC_091572	PARK2	chr9	99477950	99568942	0.0	3.70279	12.9094	2.96066	5.04966	2.94957	6.37934	4.3832	1.78778
XLOC_091573	PARK2	chr9	99477950	99568942	2.77648	2.4866	2.16749	3.22898	1.71053	2.84741	3.40487	2.17103	4.3358
XLOC_079833	PARM1	chr6	91683618	91759119	24.5049	10.5375	16.325	24.149	23.4745	22.9311	30.0572	31.6325	22.5972
XLOC_076947	PARP11	chr5	106592929	106697083	64.5185	66.7961	51.3914	36.3823	36.5684	37.73	38.9649	34.4004	39.9611
XLOC_078548	PARP11	chr5	106592929	106697083	5.14059	2.45963	0.804062	0.737051	1.36472	1.06571	2.60266	1.53944	1.88491
XLOC_078549	PARP11	chr5	106592929	106697083	5.82943	2.98897	3.90848	1.4182	2.6051	1.98782	2.1158	1.33328	1.52788
XLOC_078550	PARP11	chr5	106592929	106697083	0.0	1.13225	2.95891	0.678431	0.579922	1.16069	1.63322	0.366568	2.29657
XLOC_078551	PARP11	chr5	106592929	106697083	2.82007	5.89276	4.4024	1.76573	4.12444	2.60199	4.9362	1.10188	2.93532
XLOC_078552	PARP11	chr5	106592929	106697083	3.68316	3.02917	3.60092	1.07279	2.01485	2.86522	2.83605	1.65665	1.93004
XLOC_078553	PARP11	chr5	106592929	106697083	0.0	0.924891	4.83418	1.66205	0.23793	3.80451	2.15834	1.50799	0.805412
XLOC_078554	PARP11	chr5	106592929	106697083	4.80615	4.30924	8.76477	1.86516	3.3619	3.97122	4.01297	2.69962	4.88486
XLOC_078555	PARP11	chr5	106592929	106697083	19.6214	5.86329	12.5451	5.90968	7.75267	6.0731	6.83925	4.05784	8.38611
XLOC_078556	PARP11	chr5	106592929	106697083	4.99872	3.1777	6.84416	3.36101	3.72004	3.50627	3.41494	2.50734	3.71483

XLOC_078557	PARP11	chr5	106592929	106697083	3.59296	4.94377	8.43239	4.18696	3.4312	4.55237	6.33807	3.81626	5.02437
XLOC_078558	PARP11	chr5	106592929	106697083	6.42237	6.23813	13.8037	4.16995	4.99151	6.9649	5.31427	4.45611	5.17538
XLOC_078559	PARP11	chr5	106592929	106697083	2.82113	6.118	8.82802	4.48878	5.74214	6.22645	5.96559	4.3823	5.73288
XLOC_078560	PARP11	chr5	106592929	106697083	12.2684	9.33759	6.10512	6.59579	6.788	8.31928	8.85927	4.89348	7.98167
XLOC_078561	PARP11	chr5	106592929	106697083	8.42116	7.5344	13.1327	8.28308	11.2322	11.1417	11.885	6.48128	14.184
XLOC_078562	PARP11	chr5	106592929	106697083	6.396	4.14391	9.17	3.91641	4.65979	5.08163	5.0081	4.25447	5.39692
XLOC_078563	PARP11	chr5	106592929	106697083	2.24314	2.67997	5.2556	4.61699	4.85633	4.38426	4.95842	2.42673	4.28894
XLOC_078564	PARP11	chr5	106592929	106697083	7.09753	5.23808	7.03502	5.00633	5.97505	6.59502	5.87824	4.80327	4.63594
XLOC_041863	PARP6	chr10	19000958	19015393	24.5037	14.5484	13.3795	0.945809	0.591605	0.905072	0.509667	0.451777	0.520705
XLOC_042427	PARP6	chr10	19000958	19015393	15.5031	2.85237	7.45987	0.106849	0.0929982	0.123495	0.107493	0.0	0.0
XLOC_055444	PARVA;PARVA	chr15	40655224	41103752	8.14513	12.151	11.9632	0.0170994	0.0299746	0.0397304	0.0525217	0.0192286	0.184594
XLOC_055445	PARVA;PARVA	chr15	40655224	41103752	7.55849	16.675	22.4547	12.6515	16.6513	14.2589	15.6472	14.9214	9.63149
XLOC_056436	PARVA;PARVA	chr15	40655224	41103752	4.78594	5.00815	1.87113	0.0	0.0	0.0	0.0	0.0	0.0
XLOC_056437	PARVA;PARVA	chr15	40655224	41103752	2.26394	8.79018	7.07306	0.0	0.0	0.0	0.0	0.0	0.0
XLOC_056438	PARVA;PARVA	chr15	40655224	41103752	7.62718	9.1097	5.95562	0.0	0.0	0.0	0.0	0.0	0.0
XLOC_056439	PARVA;PARVA	chr15	40655224	41103752	4.06614	3.9517	9.5401	0.091096	0.0	0.0	0.0	0.0	0.0
XLOC_056440	PARVA;PARVA	chr15	40655224	41103752	5.1687	3.09206	5.19879	0.0	0.0	0.0766767	0.0	0.0	0.0
XLOC_056441	PARVA;PARVA	chr15	40655224	41103752	5.55682	6.0878	2.89469	0.0	0.0	0.0	0.0	0.0	0.0
XLOC_056442	PARVA;PARVA	chr15	40655224	41103752	10.8582	4.32846	11.3203	0.108095	0.0	0.0	0.0	0.0	0.105258
XLOC_056443	PARVA;PARVA	chr15	40655224	41103752	8.62558	5.15945	10.1205	0.15462	0.0	0.0895021	0.0	0.0	0.0
XLOC_056444	PARVA;PARVA	chr15	40655224	41103752	3.8149	4.18443	4.97458	0.0	0.0	0.0660679	0.0579044	0.0	0.0
XLOC_056445	PARVA;PARVA	chr15	40655224	41103752	2.51588	7.51241	15.7164	0.0	0.0	0.0	0.0	0.0	0.218464
XLOC_056446	PARVA;PARVA	chr15	40655224	41103752	3.70595	4.43184	4.82944	0.0	0.0	0.0	0.0	0.0	0.0
XLOC_056447	PARVA;PARVA	chr15	40655224	41103752	5.7288	3.99747	7.46775	0.0	0.0745984	0.0	0.345766	0.0	0.0
XLOC_056448	PARVA;PARVA	chr15	40655224	41103752	6.41059	7.02119	0.0	0.0	0.0	0.0	0.0	0.0	0.0

XLOC_056449	PARVA;PARVA	chr15	40655224	41103752	7.58965	4.53687	8.30557	0.135957	0.0	0.0	0.0	0.0	0.0
XLOC_056450	PARVA;PARVA	chr15	40655224	41103752	5.28746	2.71119	5.90906	0.0	0.0591104	0.0	0.0	0.0	0.0
XLOC_056451	PARVA;PARVA	chr15	40655224	41103752	3.50253	5.23609	12.7811	0.0	0.0	0.0	0.0	0.0	0.0
XLOC_056452	PARVA;PARVA	chr15	40655224	41103752	2.15419	5.15307	5.89621	0.0	0.0	0.111614	0.0	0.0	0.0
XLOC_056453	PARVA;PARVA	chr15	40655224	41103752	4.64754	4.1687	5.45126	0.0	0.0	0.0	0.0	0.0	0.0
XLOC_056454	PARVA;PARVA	chr15	40655224	41103752	9.05269	5.95088	7.07398	0.0	0.0	0.0	0.0	0.0	0.0
XLOC_056455	PARVA;PARVA	chr15	40655224	41103752	3.77435	2.82184	1.47603	0.0	0.0	0.0	0.0	0.0	0.0
XLOC_056456	PARVA;PARVA	chr15	40655224	41103752	3.7835	3.77039	1.97215	0.0	0.0	0.0	0.0	0.0	0.0
XLOC_076498	PARVB	chr5	115506740	115555991	42.7784	38.3176	40.6494	16.6028	15.2091	14.7485	16.136	26.3846	14.3506
XLOC_076499	PARVB	chr5	115559471	115564660	94.0653	105.141	94.5403	285.111	238.784	268.755	233.781	285.834	234.532
XLOC_076500	PARVG;PARVB	chr5	115564791	115622654	56.669	65.7808	68.6566	83.8945	87.0938	85.3648	89.2906	86.8991	70.4621
XLOC_055584	PATL1	chr15	84167836	84215056	15.4679	28.8795	14.8842	129.879	107.343	124.579	125.604	145.179	120.576
XLOC_057320	PATL1	chr15	84167836	84215056	0.0	1.19424	0.780871	4.29482	3.59966	4.20314	3.82671	2.95786	5.32456
XLOC_057321	PATL1	chr15	84167836	84215056	0.0	1.30742	0.0	8.03147	6.77258	7.43285	5.23052	5.60072	9.70342
XLOC_068281	PAX3	chr2	111183019	111183311	16.0424	7.97049	4.16792	6.22181	6.88166	3.79207	7.65007	4.58053	7.83302
XLOC_068282	PAX3	chr2	111183574	111183892	4.49564	9.3834	3.50504	3.61852	3.76238	1.82787	3.82892	3.45029	2.3283
XLOC_068283	PAX3	chr2	111184103	111184576	11.4632	15.0639	8.95315	6.56583	5.13935	5.89492	7.08939	5.18356	5.57859
XLOC_072793	PAXIP1	chr4	117835015	117873510	10.3152	10.4656	8.36007	1.15483	2.32834	1.87098	1.98847	0.784583	1.06208
XLOC_073318	PAXIP1	chr4	117835015	117873510	0.800757	2.23162	1.34743	0.751481	0.948746	0.814521	0.726607	0.581708	0.543825
XLOC_045030	PBX3	chr11	96764570	96777665	8.91564	10.1571	7.7219	9.13429	5.82066	6.53901	5.07839	7.052	4.82352
XLOC_047118	PBX3	chr11	96758734	96758983	0.0	0.0	0.0	2.77578	1.15593	1.5539	0.0	0.721668	0.663243
XLOC_047119	PBX3	chr11	96762888	96763562	1.40036	1.67438	1.09475	0.501768	0.763135	0.86893	0.251488	0.417467	0.122088
XLOC_047120	PBX3	chr11	96764140	96764499	0.0	1.07024	0.0	0.320792	0.823822	0.732497	0.309925	0.34743	0.310415
XLOC_082723	PBX4	chr7	3644015	3659952	17.1612	21.6036	17.6077	25.2871	22.1866	22.2671	28.2276	32.2584	16.6438
XLOC_082724	PBX4	chr7	3677736	3701007	10.0396	10.4117	10.4656	32.6097	26.1151	29.375	40.0414	39.6201	29.5759
XLOC_083359	PBX4	chr7	3644015	3659952	1.23094	1.66312	2.45599	1.18856	1.1998	0.957632	1.08168	0.0	0.825028
XLOC_083360	PBX4	chr7	3660127	3677653	7.30368	10.4669	5.76696	7.89353	6.07588	7.50662	7.37805	8.76384	7.17476
XLOC_083361	PBX4	chr7	3677736	3701007	12.9023	13.5145	11.0929	6.43839	7.58842	6.85036	8.58765	7.36897	6.98146

XLOC_084038	PBX4	chr7	3677736	3701007	0.0	4.84879	6.33815	6.58395	8.5072	6.54001	7.96155	6.06371	5.58556
XLOC_084039	PBX4	chr7	3677736	3701007	0.0	1.83205	4.79009	4.40646	5.55992	3.10512	7.16823	1.74316	4.23122
XLOC_047924	PCCA	chr12	80790504	80880898	18.1062	18.6759	16.7887	12.1224	11.0086	11.716	7.10793	9.21267	6.77249
XLOC_047925	PCCA	chr12	80945597	80950912	41.9549	48.5618	56.4377	85.2557	93.5028	77.4547	118.237	102.806	123.853
XLOC_049777	PCCA	chr12	80790504	80880898	3.24987	2.90942	0.0	3.19712	2.74337	1.99437	3.10591	2.21149	1.40753
XLOC_049778	PCCA	chr12	80790504	80880898	2.39115	2.14224	5.60217	1.71184	0.5542	2.45783	2.9524	1.1739	2.49269
XLOC_049779	PCCA	chr12	80790504	80880898	2.57624	1.15526	2.01424	1.96182	0.70267	1.73299	0.579521	0.769067	1.12349
XLOC_049780	PCCA	chr12	80881253	80882213	2.70377	0.539071	0.0	1.2924	1.12713	1.96339	0.408374	0.81097	0.472307
XLOC_049781	PCCA	chr12	80951234	80951638	2.934	11.3851	9.16047	16.5341	19.8595	19.2398	19.2223	13.4538	17.2998
XLOC_049782	PCCA	chr12	80952141	80953813	0.0	0.857253	1.12105	2.82602	4.38211	3.02988	3.35861	3.16761	5.05465
XLOC_049783	PCCA	chr12	80954112	80954645	0.0	0.575506	1.50505	5.00127	7.01864	5.16263	5.82474	5.32549	6.03426
XLOC_049784	PCCA	chr12	80955621	80956267	0.0	1.77023	2.31481	4.37652	4.2622	3.52009	6.63785	4.85063	6.83961
XLOC_049785	PCCA	chr12	80956980	80957338	0.0	1.07554	0.0	2.90145	3.8631	4.41639	4.35922	2.44361	4.36716
XLOC_049786	PCCA	chr12	80957551	80958580	0.0	1.73748	1.94751	2.00839	3.05053	1.63648	3.69006	2.40833	4.85776
XLOC_049787	PCCA	chr12	80959717	80960942	0.677512	0.405332	0.530056	1.03252	1.27326	0.774203	1.91127	0.61132	1.89517
XLOC_049788	PCCA	chr12	81022951	81023137	0.0	0.0	0.0	1.76106	1.38594	5.69259	0.0	3.40277	1.63309
XLOC_049789	PCCA	chr12	81023399	81024141	0.0	0.369952	0.0	0.221731	0.482304	0.640505	0.66868	0.36963	0.539737
XLOC_049790	PCCA	chr12	81024193	81024501	0.0	1.42806	0.0	1.28537	2.18256	3.4039	3.65581	2.29104	1.23952
XLOC_049791	PCCA	chr12	81024614	81025073	0.0	0.0	0.0	0.644063	0.555877	0.739758	0.634665	0.471073	0.416815
XLOC_049792	PCCA	chr12	81025246	81026155	0.0	0.0	0.0	0.345007	0.751894	0.399209	0.261358	0.480755	0.0840415
XLOC_038548	PCCB	chr1	134075004	134079236	39.6112	37.2229	33.1763	10.9519	10.0113	11.9571	5.90128	6.48405	6.31742
XLOC_059644	PCDH10	chr17	25473789	25476597	0.0	0.328918	0.0	0.0	0.0	0.0	0.099323	0.0	0.0
XLOC_048081	PCDH17	chr12	5080779	5090687	0.0	0.730397	0.636759	0.0	0.191013	0.0	0.147776	0.0814727	0.0711206
XLOC_048082	PCDH17	chr12	5090964	5375266	1.77305	2.65402	1.66516	0.0636003	0.19488	0.147649	0.0649288	0.10708	0.0620688
XLOC_048419	PCDH17	chr12	5090964	5375266	1.94333	0.387554	0.506809	0.116145	0.0507361	0.201917	0.0589804	0.0	0.0
XLOC_048420	PCDH17	chr12	5090964	5375266	0.996916	0.596233	0.779682	0.0893398	0.0778626	0.103357	0.0	0.0	0.0870426
XLOC_048421	PCDH17	chr12	5090964	5375266	1.03282	0.618	0.808175	0.0	0.16196	0.107401	0.235674	0.103752	0.180672
XLOC_048422	PCDH17	chr12	5090964	5375266	0.0	1.85657	0.0	1.11277	0.716778	0.0	0.0	0.0	0.269546
XLOC_048423	PCDH17	chr12	5090964	5375266	0.0	2.9376	1.28042	0.0	0.0	0.0	0.146469	0.0	0.0
XLOC_048424	PCDH17	chr12	5090964	5375266	0.0	0.723625	0.0	0.0	0.0	0.249028	0.213589	0.237835	0.0
XLOC_079709	PCDH7	chr6	51523929	51590731	8.15706	6.36408	5.88733	34.204	31.3415	31.9697	40.0687	38.9571	43.0713
XLOC_080116	PCDH7	chr6	51856908	52013205	5.43214	5.76401	3.86561	13.3472	14.1599	12.9521	20.5834	16.9733	21.9847
XLOC_081071	PCDH7	chr6	51590785	51594342	0.638227	0.573005	0.832611	0.515182	0.535084	0.48762	0.742074	0.429027	0.484334

XLOC_081072	PCDH7	chr6	51594537	51596116	1.01757	0.152219	0.398121	0.410566	0.518626	0.529096	0.325115	0.306687	0.445021
XLOC_037836	PCNP;RG9MTD1	chr1	46340240	46373517	0.864614	0.652602	0.676302	1.02781	0.135196	0.628003	0.0784081	0.467706	0.151049
XLOC_083764	PCSK1	chr7	98123471	98178316	2.27003	1.69759	1.02147	20.5728	29.7576	20.2923	23.5101	19.086	40.7325
XLOC_050508	PCSK2	chr13	37825823	37928203	9.79355	5.26919	10.7245	10.2763	7.83716	12.7088	8.12695	10.4297	5.96107
XLOC_050509	PCSK2	chr13	37929394	37952129	3.29186	3.13858	2.54443	5.43449	5.03501	6.30725	3.97207	6.32143	3.25347
XLOC_050510	PCSK2	chr13	38037229	38061381	18.1171	21.9412	30.7271	22.1975	21.4416	19.8932	31.1424	28.7772	27.306
XLOC_050811	PCSK2	chr13	37972200	37994221	90.0393	87.2793	92.4822	75.0386	87.952	80.7535	105.914	96.1694	97.7497
XLOC_051621	PCSK2	chr13	37825823	37928203	0.738362	0.810186	0.963207	0.573912	0.44481	0.692185	0.316091	0.620163	0.387866
XLOC_051622	PCSK2	chr13	37825823	37928203	0.0	0.0	1.38574	0.555739	0.27699	0.643304	0.562084	0.708636	0.541606
XLOC_051623	PCSK2	chr13	37825823	37928203	0.0	0.542448	0.0	0.406405	0.354426	0.564467	0.328717	0.362667	0.0792097
XLOC_051624	PCSK2	chr13	37825823	37928203	0.0	0.0	0.0	1.92461	0.838237	0.667708	1.45515	0.749989	2.06222
XLOC_051625	PCSK2	chr13	37825823	37928203	0.0	0.0	0.0	0.90433	2.29925	2.56263	0.427006	1.44679	1.30709
XLOC_051626	PCSK2	chr13	37825823	37928203	0.0	0.0	0.0	3.30488	0.874992	3.2047	0.994604	1.94292	0.985988
XLOC_051627	PCSK2	chr13	37825823	37928203	0.0	0.0	1.07401	1.10756	1.06972	1.56303	1.11075	1.77524	1.07803
XLOC_051628	PCSK2	chr13	37825823	37928203	0.0	0.0	0.0	1.5058	1.40411	0.372906	0.108195	0.83708	0.733154
XLOC_051629	PCSK2	chr13	37825823	37928203	1.61275	0.96394	0.0	0.722157	0.50136	0.499687	0.721254	0.159844	0.421478
XLOC_051630	PCSK2	chr13	37825823	37928203	0.0	0.0	0.0	0.397784	0.171886	1.3719	0.589748	0.655901	0.193163
XLOC_051631	PCSK2	chr13	37825823	37928203	0.0	0.147751	0.386447	1.19555	0.658372	0.821773	0.721503	0.595474	0.56157
XLOC_051632	PCSK2	chr13	37825823	37928203	0.0	0.136051	0.355846	0.693147	0.463723	0.567641	0.373966	0.50285	0.238687
XLOC_051633	PCSK2	chr13	37825823	37928203	0.0	1.61892	2.82258	0.808557	0.0	0.745219	0.643895	0.0	0.471667
XLOC_051634	PCSK2	chr13	37825823	37928203	0.0	0.0	0.0	0.969389	0.384069	1.12156	0.622866	0.49109	0.171727
XLOC_051635	PCSK2	chr13	37825823	37928203	4.51381	0.0	1.76281	1.00994	0.0	0.464338	1.39666	0.221954	0.784608
XLOC_051636	PCSK2	chr13	37825823	37928203	0.0	1.39145	0.0	1.04239	0.360057	0.23954	0.617046	0.0	0.202417
XLOC_051637	PCSK2	chr13	37825823	37928203	0.674372	1.00861	1.05519	1.02771	0.369649	0.420332	0.245482	0.405663	0.589485
XLOC_051638	PCSK2	chr13	37825823	37928203	0.0	0.0	0.0	0.396568	0.342721	0.0	0.392012	0.0	0.0
XLOC_051639	PCSK2	chr13	37825823	37928203	0.0	0.661701	0.0	0.0	0.171369	0.22796	0.392012	0.0	0.192576
XLOC_051640	PCSK2	chr13	37825823	37928203	0.0	0.0	0.0	0.495004	0.285931	0.380118	0.328294	0.0	0.641624
XLOC_051641	PCSK2	chr13	37970853	37971990	0.0	0.662585	1.15529	0.860458	1.44476	0.996797	0.73829	0.517803	0.96796
XLOC_051642	PCSK2	chr13	37972200	37994221	1.23473	0.738737	2.41515	2.71204	4.01436	3.7853	2.13724	2.10596	3.1847
XLOC_051643	PCSK2	chr13	37972200	37994221	1.11187	1.99472	0.869478	3.58665	2.60281	3.91654	2.91073	3.43722	3.97858
XLOC_051644	PCSK2	chr13	37972200	37994221	0.0	1.00578	2.62998	4.52161	4.39327	1.72264	3.21486	2.61786	5.25355
XLOC_051645	PCSK2	chr13	38022322	38023258	0.0	3.88973	0.0	0.249789	0.798611	0.289082	0.0	0.371417	0.486819

XLOC_055413	PCSK7;TAGLN;SIDT2	chr15	28311263	28331773	0.753777	0.225462	0.0	0.0675678	0.117976	0.0782675	0.0	0.0754995	0.0
XLOC_063877	PCTP	chr19	6428042	6482459	0.937468	1.30809	2.61217	2.4159	2.09507	1.65784	1.78549	3.57665	1.57209
XLOC_070130	PDCD1	chr3	121291736	121362065	1.6317	7.32044	7.02013	10.3841	7.84909	8.04462	7.62739	9.47194	8.98136
XLOC_080163	PDCL2;NMU	chr6	72626231	72788899	2.64467	1.84149	1.45666	12.8639	10.707	13.2763	10.7456	14.6471	8.1869
XLOC_081346	PDCL2;NMU	chr6	72626231	72788899	2.99466	0.893836	0.0	1.60717	1.61168	0.920192	1.56793	1.45983	1.5577
XLOC_081347	PDCL2;NMU	chr6	72626231	72788899	0.0	0.123904	0.324066	1.26252	1.20232	1.24958	0.719506	0.791371	0.978294
XLOC_081348	PDCL2;NMU	chr6	72626231	72788899	0.0	0.692561	0.0	0.726408	0.542047	0.239909	0.62676	0.461717	0.202124
XLOC_081349	PDCL2;NMU	chr6	72626231	72788899	0.0	0.647841	0.0	0.582393	0.839107	0.446477	0.960242	0.8541	0.188566
XLOC_081350	PDCL2;NMU	chr6	72626231	72788899	0.0	0.356547	0.0	1.81643	1.11595	0.74093	0.214985	0.356416	0.312146
XLOC_081351	PDCL2;NMU	chr6	72626231	72788899	1.86661	0.0	1.45853	1.83839	0.579123	2.69477	0.33238	1.47551	0.974773
XLOC_081352	PDCL2;NMU	chr6	72626231	72788899	0.0	0.0	0.0	1.00194	0.0	0.381024	0.0	0.0	0.323045
XLOC_081353	PDCL2;NMU	chr6	72626231	72788899	0.0	0.819756	0.0	1.35113	1.28126	1.84375	0.615938	0.681511	1.19555
XLOC_081354	PDCL2;NMU	chr6	72626231	72788899	0.0	0.0	0.0	1.09777	0.0	0.83345	0.70136	0.394186	2.1215
XLOC_081355	PDCL2;NMU	chr6	72626231	72788899	0.0	0.0	0.0	0.739489	0.636693	1.41304	0.482886	0.808139	0.956246
XLOC_081356	PDCL2;NMU	chr6	72626231	72788899	0.0	0.0	0.0	2.19553	0.936501	1.6669	2.80544	2.36511	0.353585
XLOC_081357	PDCL2;NMU	chr6	72626231	72788899	0.0	0.781046	0.0	1.63834	1.61362	1.07411	0.919156	0.768501	0.908293
XLOC_081358	PDCL2;NMU	chr6	72626231	72788899	0.0	0.141106	0.0	1.14178	1.22073	0.883018	0.732404	0.711044	0.206287
XLOC_081359	PDCL2;NMU	chr6	72626231	72788899	2.4899	1.48701	1.94432	2.89636	1.53739	1.79047	0.219209	0.488379	0.432458
XLOC_081360	PDCL2;NMU	chr6	72626231	72788899	9.75032	0.0	0.0	0.881523	1.45066	0.978608	1.57188	0.0	0.83733
XLOC_081361	PDCL2;NMU	chr6	72626231	72788899	0.0	0.445289	1.16455	1.46784	0.8113	1.07789	1.33555	0.443642	1.42827
XLOC_081362	PDCL2;NMU	chr6	72626231	72788899	0.0	0.0	1.0679	0.856564	1.48922	0.98907	0.245468	0.950571	0.595528
XLOC_081363	PDCL2;NMU	chr6	72626231	72788899	0.0	0.0	0.0	1.9571	0.631983	0.280506	0.479412	0.0	0.0
XLOC_081364	PDCL2;NMU	chr6	72626231	72788899	0.0	1.73182	0.0	0.0	0.877476	2.3505	0.971983	0.55076	0.500213
XLOC_081365	PDCL2;NMU	chr6	72626231	72788899	0.0	0.0	0.0	1.0372	1.33818	0.594142	0.253375	0.848788	1.25692
XLOC_081366	PDCL2;NMU	chr6	72626231	72788899	0.0	0.315037	2.47179	0.9441	1.39824	0.655145	0.476043	0.105138	1.01166
XLOC_081367	PDCL2;NMU	chr6	72626231	72788899	0.0	0.751242	0.0	3.82698	1.35898	1.80887	0.0	0.739983	0.873858
XLOC_081368	PDCL2;NMU	chr6	72626231	72788899	0.0	0.85708	2.2415	1.28422	0.446345	1.48236	0.643284	0.712041	0.124978
XLOC_081369	PDCL2;NMU	chr6	72626231	72788899	0.0	1.0809	0.0	1.61995	1.38642	0.0	3.44118	0.35076	0.313481
XLOC_081370	PDCL2;NMU	chr6	72626231	72788899	1.20046	0.0	0.0	0.645344	0.655257	0.870115	0.757333	1.67416	0.209473
XLOC_081371	PDCL2;NMU	chr6	72626231	72788899	0.0	0.0	0.0	1.31564	0.0	0.750692	0.317302	1.4235	0.636382
XLOC_081372	PDCL2;NMU	chr6	72626231	72788899	0.0	0.289738	1.51554	0.868289	0.983968	1.10514	0.438463	0.580725	0.592218
XLOC_081373	PDCL2;NMU	chr6	72626231	72788899	0.0	0.0	0.0	3.18718	5.01516	1.54801	1.28921	1.45634	0.0

XLOC_081374	PDCL2;NMU	chr6	72626231	72788899	0.0	0.855516	0.559382	1.15374	1.34335	0.519831	0.715242	0.573217	0.499955
XLOC_081375	PDCL2;NMU	chr6	72626231	72788899	0.0	0.897538	0.0	3.22766	1.61822	3.38781	1.83646	2.05195	0.521368
XLOC_081376	PDCL2;NMU	chr6	72626231	72788899	0.0	0.0	0.0	1.18237	1.10451	1.07507	0.512009	0.282509	0.493773
XLOC_081377	PDCL2;NMU	chr6	72626231	72788899	0.0	0.0	0.0	0.919829	1.19653	1.59035	1.68136	0.847379	1.78903
XLOC_081378	PDCL2;NMU	chr6	72626231	72788899	1.46267	0.87439	0.0	1.44116	1.13815	1.36086	1.31189	0.580948	0.764951
XLOC_081379	PDCL2;NMU	chr6	72626231	72788899	0.0	0.0	0.0	7.83879	8.81543	3.24993	1.7298	1.988	3.7113
XLOC_081380	PDCL2;NMU	chr6	72626231	72788899	0.0	0.0	0.871446	1.5311	1.282	1.73845	1.32483	0.78441	0.877009
XLOC_081381	PDCL2;NMU	chr6	72626231	72788899	0.687822	0.411496	0.0	1.8498	2.0465	1.71477	1.5645	1.31004	1.50307
XLOC_081382	PDCL2;NMU	chr6	72626231	72788899	0.983361	0.588134	1.53818	3.08442	1.99713	1.52941	1.69063	1.96435	1.71727
XLOC_081383	PDCL2;NMU	chr6	72626231	72788899	0.0	0.0	0.0	11.337	5.01514	8.43753	4.10115	4.68875	7.20874
XLOC_081384	PDCL2;NMU	chr6	72626231	72788899	0.0	1.56016	4.0792	7.49343	4.36076	2.652	3.96981	5.48466	3.15729
XLOC_081385	PDCL2;NMU	chr6	72626231	72788899	0.0	2.30197	12.0359	10.4092	14.4492	3.88475	8.22169	9.38169	4.6427
XLOC_081386	PDCL2;NMU	chr6	72626231	72788899	0.0	0.393488	1.02909	4.48089	3.69147	2.5877	4.37985	3.27331	2.64019
XLOC_081387	PDCL2;NMU	chr6	72626231	72788899	0.0	0.0	0.0	9.00917	8.7949	7.29365	3.67621	5.89417	5.45677
XLOC_081388	PDCL2;NMU	chr6	72626231	72788899	0.0	0.0	0.0	2.74245	3.55562	4.72999	2.5184	2.36954	2.66369
XLOC_081389	PDCL2;NMU	chr6	72626231	72788899	2.87573	0.858427	0.0	6.17387	4.64716	7.95826	3.77194	5.05362	4.4893
XLOC_081390	PDCL2;NMU	chr6	72626231	72788899	0.0	0.0	0.0	3.80148	4.37584	3.88194	4.5815	4.17245	3.28058
XLOC_081391	PDCL2;NMU	chr6	72626231	72788899	0.0	1.38699	0.0	3.94841	4.84525	4.53686	4.71607	2.73823	2.62304
XLOC_081392	PDCL2;NMU	chr6	72626231	72788899	1.24168	0.371221	0.970857	5.45105	4.54908	4.75589	5.92649	5.5632	3.68276
XLOC_081393	PDCL2;NMU	chr6	72626231	72788899	0.0	0.825708	0.0	7.42309	6.81655	6.24074	4.84641	5.94827	4.31939
XLOC_066157	PDE11A	chr2	18842101	18998180	9.42888	8.0401	8.50792	2.48466	2.75789	2.6706	2.82437	3.59571	3.35812
XLOC_065495	PDE11A;TTC30A	chr2	19238703	19286035	0.0	0.0	0.0	0.0	0.0	0.0	0.140163	0.0	0.0674137
XLOC_065486	PDE1A	chr2	14229404	14331923	26.6813	5.60052	9.26746	0.780399	0.109259	0.618978	0.157105	0.257469	0.121722
XLOC_066902	PDE1A	chr2	14189584	14189938	7.3564	2.19455	0.0	5.59147	5.34623	2.62742	1.90381	2.84701	3.81828
XLOC_083411	PDE4A	chr7	16198573	16222061	3.29293	2.22082	3.37206	5.8868	7.04634	6.68781	5.84864	7.37419	5.20933
XLOC_069938	PDE4B	chr3	79591351	79603408	66.65	72.7343	79.2424	251.802	212.765	243.751	257.538	304.924	265.335
XLOC_070320	PDE4B	chr3	79062032	79287269	56.6842	49.8325	42.0858	6.89785	6.46237	7.49248	8.20944	8.58543	6.56296
XLOC_070321	PDE4B	chr3	79456565	79567613	16.781	17.6517	17.9015	14.5378	11.454	14.0607	13.9075	15.7387	10.6396
XLOC_071474	PDE4B	chr3	79456171	79456392	0.0	9.56028	16.66	9.6648	2.37326	8.55289	7.68795	3.92589	7.32437
XLOC_082737	PDE4C	chr7	4927009	4928693	0.0	0.0	0.866816	0.297973	0.0	0.0	0.200137	0.0	0.0967422
XLOC_082738	PDE4C	chr7	4928994	4930140	2.6714	0.0	0.0	0.119658	0.312118	0.13819	0.0	0.0	0.116479
XLOC_082739	PDE4C	chr7	4933171	4943771	0.466451	0.418679	0.365012	0.501897	0.2927	0.291121	0.2983	0.0	0.57128

XLOC_079595	PDE5A	chr6	6955981	7039708	4.73487	2.83239	1.12645	7.25449	4.42838	6.19533	5.79121	7.13587	6.63041
XLOC_080461	PDE5A	chr6	6877829	6878685	0.0	0.309629	0.0	0.0	0.0	0.0	0.0	0.0	0.0
XLOC_080462	PDE5A	chr6	7040561	7041670	2.28034	1.13678	1.78389	0.476948	0.297404	0.31569	0.483452	0.380642	0.265698
XLOC_083672	PDE6A	chr7	63098710	63212500	1.14889	1.03131	0.299708	1.27065	1.92379	1.79355	0.911082	1.19455	1.17293
XLOC_066488	PDE6D	chr2	120266298	120270127	0.0	0.0	0.60286	0.0	0.0	0.0	0.0	0.0	0.0
XLOC_066489	PDE6D	chr2	120270319	120281022	0.0	0.0	0.0	0.0	0.0527574	0.0699904	0.0	0.0	0.0
XLOC_066490	PDE6D	chr2	120299596	120302623	0.0	0.29074	0.0	0.522793	0.304845	0.151605	0.221866	0.341792	0.467527
XLOC_089511	PDE7B	chr9	75037633	75060512	23.2329	20.4248	14.492	23.4737	22.3321	24.4558	24.2016	26.3586	24.7907
XLOC_089512	PDE7B	chr9	75071537	75223866	21.0049	14.5451	14.6504	10.6642	10.5006	9.81662	15.2746	15.8922	9.39133
XLOC_091283	PDE7B	chr9	75061211	75062119	23.1289	35.7361	21.8583	39.1241	32.2989	34.1776	45.0997	51.0272	42.5815
XLOC_091284	PDE7B	chr9	75071191	75071470	0.0	1.75936	0.0	1.0573	2.67288	3.58078	3.45209	5.59095	2.03239
XLOC_056041	PDGFD	chr15	4559948	4560088	0.0	0.0	0.0	126.151	46.993	100.742	42.717	106.179	116.683
XLOC_080150	PDGFRA	chr6	71388627	71440301	15.8728	16.8881	14.2711	26.899	27.8164	29.927	29.1056	28.6223	28.4686
XLOC_081323	PDGFRA	chr6	71376853	71377607	1.21253	0.0	0.0	0.0	0.0	0.0	0.109254	0.120768	0.0
XLOC_080652	PDHA2	chr6	30064123	30065483	0.601447	1.43941	4.23526	0.593138	0.801124	0.937648	0.876949	0.905324	0.84143
XLOC_056837	PDHX	chr15	66243224	66244076	0.0	0.0	0.0	0.0	0.0813093	0.431765	0.0	0.0	0.0
XLOC_056838	PDHX	chr15	66259840	66260403	0.0	0.532997	0.0	0.0	0.138441	0.0	0.0	0.0	0.0
XLOC_056839	PDHX	chr15	66263455	66264248	0.0	0.0	1.77996	0.305935	0.266369	0.235785	0.0	0.0	0.0
XLOC_056840	PDHX	chr15	66266299	66267166	0.0	0.304833	0.0	0.0913525	0.0796043	0.211347	0.0	0.101772	0.177997
XLOC_056841	PDHX	chr15	66272056	66273828	0.44796	0.670148	0.701099	0.16067	0.210854	0.419419	0.0409391	0.0	0.195954
XLOC_056842	PDHX	chr15	66278023	66279039	0.0	0.251967	0.0	0.0	0.329405	0.524567	0.15286	0.0842904	0.220795
XLOC_056843	PDHX	chr15	66280431	66280876	0.0	0.751241	0.0	0.450233	0.582409	0.25841	0.0	0.246661	0.0
XLOC_056844	PDHX	chr15	66283080	66284859	0.0	0.533805	0.0	0.95986	0.489877	0.696023	0.0	0.0896888	0.234131
XLOC_056845	PDHX	chr15	66285122	66286131	0.0	1.01615	0.0	1.06583	0.464933	0.352571	0.231145	0.169952	0.593613
XLOC_038399	PDIA5	chr1	68039628	68078613	4.59675	6.35328	5.51073	0.0	0.0	0.0	0.0	0.0	0.0
XLOC_039596	PDIA5	chr1	68034356	68035453	3.0793	5.29595	4.21553	0.0	0.0	0.0	0.0	0.0	0.0
XLOC_039597	PDIA5	chr1	68035536	68038049	2.76332	3.95075	5.04642	0.0	0.0	0.0	0.0	0.0	0.0
XLOC_039598	PDIA5	chr1	68038688	68039568	10.0106	6.28642	5.48041	0.0	0.0	0.0	0.0	0.0	0.0
XLOC_084335	PDLIM4	chr7	23453143	23453416	6.19662	3.69457	4.8297	4.44267	6.5388	0.0	2.57978	1.75694	1.59979
XLOC_079639	PDLIM5	chr6	31398682	31512126	38.4893	32.7751	34.2833	21.9328	29.8026	24.6996	26.2736	23.3778	30.7428
XLOC_080053	PDLIM5	chr6	31517053	31563960	16.4028	13.8555	11.9149	11.8359	9.90076	12.4886	4.66219	5.23835	8.40682
XLOC_080746	PDLIM5	chr6	31512730	31512998	0.0	7.70904	0.0	0.0	1.94657	1.95793	3.75625	2.43888	2.22457
XLOC_080747	PDLIM5	chr6	31513227	31515660	2.54264	1.52171	1.74126	0.57006	1.77216	1.05893	1.54322	0.864163	1.47442

XLOC_080748	PDLIM5	chr6	31515792	31516858	1.59219	2.3811	2.49101	1.49851	1.86835	1.48751	1.44571	0.557876	2.15634
XLOC_053706	PDP1	chr14	72537784	72710695	18.6991	16.2637	13.9887	15.1734	19.0882	15.7621	15.4264	13.3577	19.5727
XLOC_054799	PDP1	chr14	72537784	72710695	7.79211	3.87775	4.0557	2.09142	2.40339	2.93292	3.19502	2.28946	2.93095
XLOC_054800	PDP1	chr14	72537784	72710695	4.27049	3.51159	5.84405	2.10461	2.49997	1.99137	3.1828	1.2782	1.95691
XLOC_089349	PDSS2	chr9	43311464	43335953	0.0	0.0	0.0	0.324461	0.347067	0.37654	0.147047	0.161735	0.175935
XLOC_089847	PDSS2	chr9	43110769	43116558	0.0	0.438766	0.466317	1.35654	1.86772	1.38847	2.12121	1.08036	1.94856
XLOC_089848	PDSS2	chr9	43123920	43205803	0.547584	0.0	0.488478	3.25305	6.26851	5.06609	3.21249	5.03361	7.10071
XLOC_089849	PDSS2	chr9	43221738	43239492	43.0288	21.7054	28.189	1.88418	2.19714	2.18542	3.15199	2.56382	4.85786
XLOC_090674	PDSS2	chr9	43108665	43109286	3.12101	0.0	0.0	0.838586	2.79062	0.806117	2.37514	1.08337	1.63165
XLOC_090675	PDSS2	chr9	43109360	43109760	0.0	1.78033	0.0	1.60056	1.37586	1.22196	3.64422	1.45402	3.87837
XLOC_090676	PDSS2	chr9	43110055	43110327	0.0	0.0	0.0	0.560036	4.70851	0.631204	4.67964	1.18062	2.15077
XLOC_090677	PDSS2	chr9	43110769	43116558	1.44867	0.216662	0.0	0.714236	0.62367	0.300896	0.592715	0.798344	0.569753
XLOC_090678	PDSS2	chr9	43220510	43221072	3.57631	0.0	2.79467	0.160115	0.0	0.0	0.0	0.0	0.155678
XLOC_090679	PDSS2	chr9	43239596	43240823	2.70498	2.62973	2.64533	0.42436	0.317722	0.140502	0.3077	0.406791	0.413794
XLOC_089846	PDSS2;SOBP	chr9	42970491	43106054	40.6642	38.2508	39.6105	12.1607	12.3764	12.1261	16.2904	14.4655	13.851
XLOC_076313	PDZRN4	chr5	39798132	39799445	86.5925	75.8639	49.8855	112.68	78.9046	107.206	99.6767	142.232	98.5897
XLOC_076836	PDZRN4	chr5	39613958	39644644	14.7842	18.4129	18.2497	0.133455	0.0388906	0.103193	0.0452859	0.149557	0.0433831
XLOC_077768	PDZRN4	chr5	39609375	39611732	1.64407	2.16463	2.05875	0.0	0.0	0.0	0.0301172	0.0	0.0
XLOC_077769	PDZRN4	chr5	39611829	39612615	1.15079	3.44079	6.29916	0.0	0.0	0.0	0.0	0.0	0.0
XLOC_077770	PDZRN4	chr5	39612702	39613619	1.90457	1.99348	1.48962	0.0	0.0	0.0987573	0.0	0.0	0.0
XLOC_077771	PDZRN4	chr5	39613958	39644644	0.0	0.0	5.08259	0.0	0.0	0.0	0.0	0.0	0.560946
XLOC_077772	PDZRN4	chr5	39613958	39644644	0.0	3.2833	0.0	0.0	0.0	0.0	0.0	0.0	0.474389
XLOC_077773	PDZRN4	chr5	39613958	39644644	0.0	0.0	21.7686	0.0	0.0	0.0	1.09429	0.0	0.0
XLOC_077774	PDZRN4	chr5	39613958	39644644	0.0	1.81706	4.75068	0.0	0.0	0.0	0.0	0.0	0.0
XLOC_077775	PDZRN4	chr5	39665101	39665923	1.08844	3.58002	2.55354	0.0	0.0	0.0	0.0	0.0	0.0
XLOC_077776	PDZRN4	chr5	39679525	39680359	3.20739	4.15596	5.85267	0.0	0.0	0.0	0.0	0.0	0.0
XLOC_077777	PDZRN4	chr5	39693035	39693617	0.0	1.52753	17.311	0.0	0.0	0.0	0.0	0.0	0.0
XLOC_077778	PDZRN4	chr5	39703731	39704515	2.30893	3.10659	11.7357	0.0	0.0	0.0	0.0	0.0	0.0
XLOC_077779	PDZRN4	chr5	39715421	39716572	8.00777	3.04852	7.40362	0.0652571	0.0	0.0	0.0	0.0	0.0
XLOC_077780	PDZRN4	chr5	39717165	39717675	0.0	6.12987	9.61834	0.183688	0.0	0.0	0.0	0.0	0.0
XLOC_077781	PDZRN4	chr5	39719496	39720322	3.24578	4.85266	12.6914	0.0969494	0.0	0.0	0.0	0.107932	0.0944357
XLOC_077782	PDZRN4	chr5	39743223	39743812	1.67625	6.01116	5.24018	0.0	0.0	0.0	0.0	0.0	0.0
XLOC_077783	PDZRN4	chr5	39743872	39744570	5.35269	6.00047	6.2772	0.119879	0.0	0.0	0.0	0.0	0.0

XLOC_077784	PDZRN4	chr5	39745710	39746785	1.57657	4.95129	6.78312	0.0	0.0	0.0	0.0	0.157845	0.0
XLOC_086448	PEBP4	chr8	70527556	70535395	30.5162	45.9802	30.2639	116.322	108.841	108.011	98.8082	92.7964	97.4946
XLOC_086449	PEBP4	chr8	70539464	70593448	11.3475	13.7382	15.0527	5.71223	7.76923	6.65715	8.09793	7.21473	5.19662
XLOC_086450	PEBP4	chr8	70594136	70597298	76.5036	118.399	105.266	94.5162	73.1914	90.4123	48.2116	81.875	43.473
XLOC_086451	PEBP4	chr8	70673260	70678858	51.4355	74.564	61.1931	147.129	157.968	155.22	140.821	117.907	109.525
XLOC_086452	PEBP4	chr8	70718555	70722245	0.0	0.764502	0.0	1.14545	3.55505	1.84039	0.225132	0.501786	1.11148
XLOC_086453	PEBP4	chr8	70730309	70749989	6.22095	10.1759	3.85815	0.526578	0.568674	0.561166	1.3572	0.732651	1.50269
XLOC_086454	PEBP4	chr8	70730309	70749989	1.57282	2.35277	5.7433	0.0940133	0.0	0.327092	0.143536	0.0526607	0.0917088
XLOC_086914	PEBP4	chr8	70597395	70608333	23.3175	31.6678	32.7016	63.2018	71.3118	69.8535	73.2424	88.6322	55.7117
XLOC_086915	PEBP4	chr8	70629359	70632605	6.40292	0.522409	1.82177	1.77435	3.0562	2.72271	3.34302	0.934813	2.59589
XLOC_086916	PEBP4	chr8	70649852	70659703	24.9947	23.5365	19.284	16.0778	18.2831	17.7826	12.6496	11.8018	15.3324
XLOC_088023	PEBP4	chr8	70539464	70593448	4.23387	3.37487	1.10355	0.505821	1.20847	0.729909	1.1403	0.701116	0.246073
XLOC_088024	PEBP4	chr8	70645909	70647752	3.00362	3.72319	4.36526	3.232	6.93542	3.83941	4.58972	3.4952	7.62111
XLOC_088025	PEBP4	chr8	70647865	70648635	7.08513	8.47338	6.46352	5.07856	8.93298	5.01378	7.23943	6.47121	12.158
XLOC_088026	PEBP4	chr8	70648820	70649168	0.0	4.52623	5.91765	4.74891	9.85864	8.51126	10.4521	7.33134	10.1695
XLOC_088027	PEBP4	chr8	70649307	70649739	15.8098	20.4547	16.4584	13.9092	24.5798	11.6294	18.7421	14.1886	19.6692
XLOC_088028	PEBP4	chr8	70679228	70679956	0.0	0.37902	0.0	0.340749	0.395217	0.524881	0.456472	0.378561	0.221169
XLOC_088029	PEBP4	chr8	70686154	70686966	0.0	0.0	0.0	0.594126	0.603644	1.14493	0.099769	0.440838	0.482239
XLOC_088030	PEBP4	chr8	70687497	70688517	0.0	0.0	0.0	0.300642	0.852508	0.78322	0.076081	0.251711	0.219774
XLOC_088031	PEBP4	chr8	70689363	70690161	0.0	0.0	0.0	1.01183	0.704823	0.467914	0.203809	0.337756	0.788379
XLOC_088032	PEBP4	chr8	70695110	70696131	0.0	0.501018	0.0	0.900883	1.37554	1.30386	0.0759943	0.335229	0.146347
XLOC_088033	PEBP4	chr8	70696919	70697320	2.9701	0.886523	0.0	2.12535	2.74068	1.82554	0.259289	1.15861	1.80256
XLOC_088034	PEBP4	chr8	70697437	70697754	0.0	2.69753	0.0	5.66373	5.16136	8.27533	1.92551	0.433837	0.0
XLOC_088035	PEBP4	chr8	70698026	70698865	5.30643	2.85611	1.65994	1.52163	2.23688	1.86977	1.4384	1.1649	0.83377
XLOC_088036	PEBP4	chr8	70699047	70700469	107.528	116.696	114.119	83.9955	91.0843	85.0811	70.4031	63.4451	71.423
XLOC_088037	PEBP4	chr8	70700521	70701189	1.41682	4.2351	0.0	7.86868	8.27146	8.35101	3.81551	1.82992	3.58197
XLOC_088038	PEBP4	chr8	70701314	70701621	4.82142	8.62462	3.75845	13.3698	12.8129	8.80933	2.45213	2.30542	6.23793
XLOC_088039	PEBP4	chr8	70701706	70702006	15.1613	0.0	0.0	9.94763	9.58008	9.73798	5.12385	1.92905	5.22836
XLOC_088040	PEBP4	chr8	70702607	70703021	0.0	0.84175	4.40234	1.51347	3.25613	2.31283	0.987228	0.550937	1.22298
XLOC_088041	PEBP4	chr8	70703467	70703848	6.47072	0.965488	0.0	1.44676	3.72443	1.32375	0.281165	0.629137	1.12105
XLOC_088042	PEBP4	chr8	70718555	70722245	11.3522	5.07609	0.0	3.04945	6.00499	3.44623	0.0	1.07741	1.95527
XLOC_088043	PEBP4	chr8	70750075	70750784	3.93444	5.09681	1.02536	0.587455	0.510863	1.08564	1.17955	0.391395	1.48689
XLOC_065849	PEF1	chr2	122630593	122632847	3.24443	5.33854	6.76977	4.50882	4.57801	4.7226	2.76263	3.69263	2.93208

XLOC_065850	PEF1	chr2	122637835	122641201	3.8234	3.61262	3.15606	1.14172	1.39039	1.25194	0.0	0.985944	0.958688
XLOC_068471	PEF1	chr2	122632932	122633482	0.0	1.65186	0.0	0.165001	0.285918	0.0	0.164144	0.182141	0.0
XLOC_044134	PELI1	chr11	62503728	62513603	1.76943	2.38134	2.07604	21.9645	17.0138	17.0727	17.4035	29.9921	21.1742
XLOC_044828	PELI1	chr11	62524902	62539525	18.8796	48.1765	11.803	98.6047	76.0711	89.1076	107.383	106.896	114.27
XLOC_046352	PELI1	chr11	62503728	62513603	0.0	1.1257	0.0	2.69958	2.59645	4.23396	3.25037	1.8237	5.87452
XLOC_046353	PELI1	chr11	62503728	62513603	0.0	0.0	0.0	11.8198	6.78681	7.88126	3.00319	10.2182	14.9706
XLOC_046354	PELI1	chr11	62513689	62513936	0.0	0.0	0.0	17.0039	11.7901	7.13425	8.37851	16.1872	12.8596
XLOC_046355	PELI1	chr11	62514369	62514609	0.0	0.0	0.0	20.6352	17.1059	14.4988	24.1667	36.3216	23.3198
XLOC_046356	PELI1	chr11	62515625	62515796	0.0	0.0	0.0	66.1051	46.7219	43.056	51.1627	133.081	76.6297
XLOC_059551	PET112	chr17	5941359	6038762	8.74434	8.97716	8.65635	4.58801	5.33984	4.74029	4.31768	4.87319	5.47928
XLOC_059552	PET112	chr17	6079783	6084898	35.3576	31.4956	18.5779	64.6927	52.7556	60.4202	63.4882	85.3182	71.1477
XLOC_059945	PET112	chr17	6041555	6079734	8.203	13.2466	13.8121	14.636	16.0862	14.8465	16.9487	15.4904	18.2258
XLOC_060416	PET112	chr17	6038921	6039908	1.74383	4.69384	8.86614	2.2663	2.18139	1.44749	1.42306	2.18015	2.81805
XLOC_060417	PET112	chr17	6040694	6041411	5.1709	7.34279	9.09645	9.26506	10.677	9.09701	10.933	6.6887	11.8386
XLOC_060418	PET112	chr17	6041555	6079734	0.736135	2.20189	2.30357	1.71567	2.07415	1.98753	1.60596	1.8437	2.05869
XLOC_060419	PET112	chr17	6041555	6079734	3.23987	2.42066	2.5323	2.03108	2.39194	1.17111	1.44877	2.40817	2.11678
XLOC_060420	PET112	chr17	6041555	6079734	2.20028	1.31666	3.09932	1.69674	1.34646	1.19033	1.85024	1.63715	1.07801
XLOC_073076	PEX1;ANKIB1;TMBIM1B;GATAD1	chr4	9450958	9669330	8.2527	5.96469	5.75478	13.1778	16.9495	14.1783	13.6382	11.7516	16.6235
XLOC_073680	PEX1;ANKIB1;TMBIM1B;GATAD1	chr4	9450958	9669330	0.786569	0.0	0.0	0.211519	0.246153	0.653252	0.0714333	0.0787524	0.206189
XLOC_073681	PEX1;ANKIB1;TMBIM1B;GATAD1	chr4	9450958	9669330	0.0	0.418603	0.0	0.125449	0.0	0.289653	0.251488	0.139156	0.488354
XLOC_073682	PEX1;ANKIB1;TMBIM1B;GATAD1	chr4	9450958	9669330	2.20677	0.659846	0.0	0.296616	0.430525	0.114327	0.0996199	0.440175	0.288904
XLOC_073683	PEX1;ANKIB1;TMBIM1B;GATAD1	chr4	9450958	9669330	0.746772	0.446741	0.584199	0.602467	0.35066	0.387723	0.542953	0.52363	0.39157
XLOC_073684	PEX1;ANKIB1;TMBIM1B;GATAD1	chr4	9450958	9669330	0.0	0.53213	0.278358	0.478433	0.796812	0.481742	0.522618	0.32285	0.420414
XLOC_073685	PEX1;ANKIB1;TMBIM1B;GATAD1	chr4	9450958	9669330	0.0	3.27412	0.0	12.9163	8.11951	8.78296	5.25504	7.0478	4.70203

XLOC_057911	PEX14	chr16	43889876	43963957	11.7197	12.0947	7.4654	9.75417	11.4378	10.8538	9.78321	8.51646	14.6565
XLOC_058135	PEX14	chr16	43861330	43889764	12.8373	11.188	10.215	16.9725	13.314	15.0913	16.1118	21.2186	15.5553
XLOC_058136	PEX14	chr16	43968814	43979728	1.81997	7.43027	5.99093	1.45636	0.842892	1.16357	1.06763	1.32594	0.638336
XLOC_059030	PEX14	chr16	43861330	43889764	2.16242	7.10499	1.68914	2.51617	3.51381	3.3387	4.02143	4.47097	3.5721
XLOC_059031	PEX14	chr16	43964012	43967013	1.01737	3.27288	1.79167	0.729946	0.69945	1.00668	0.816501	0.589552	0.578952
XLOC_059032	PEX14	chr16	43967198	43968671	2.19912	5.59225	1.72073	1.08443	0.646422	0.74306	0.802388	0.828082	0.721213
XLOC_059033	PEX14	chr16	43980362	43980603	0.0	0.0	0.0	0.755803	1.25377	0.0	0.0	0.781458	0.0
XLOC_058137	PEX14;CORT;DFFA	chr16	43980667	44016512	0.964763	0.577427	0.377561	0.187923	0.274434	0.12561	0.0221163	0.0486116	0.101919
XLOC_089536	PEX3;FUCA2;ADAT2	chr9	81863720	82026571	2.52309	4.42199	3.10297	3.03835	1.89587	3.86435	1.51776	2.24923	2.05037
XLOC_076425	PEX5;WC1.3;CD163L1;CLSTN3	chr5	103424698	103598377	23.4554	29.318	37.7103	15.9235	11.7981	14.7592	7.0566	10.0167	11.5219
XLOC_037965	PEX5L	chr1	87667973	87722726	3.50229	1.39215	1.28931	52.7157	40.6694	42.5262	35.5649	42.7745	38.2272
XLOC_037966	PEX5L	chr1	87755882	87792398	23.2635	28.2802	29.2162	24.985	19.9096	22.7696	28.9211	28.4378	24.5052
XLOC_039850	PEX5L	chr1	87723842	87725001	0.0	0.0	0.0	1.4242	1.30015	1.275	0.722289	0.506528	0.820518
XLOC_039851	PEX5L	chr1	87728283	87729280	0.861531	0.0	0.0	1.00383	0.606257	0.625772	0.234397	0.258534	0.602072
XLOC_039852	PEX5L	chr1	87755882	87792398	4.49608	16.0858	10.5155	6.43328	5.81494	6.85453	9.57229	6.03833	6.2091
XLOC_039853	PEX5L	chr1	87755882	87792398	3.30952	15.7995	15.493	132.288	88.585	82.2198	102.251	72.9763	130.686
XLOC_039854	PEX5L	chr1	87755882	87792398	20.6365	32.8156	21.4486	196.301	118.364	101.285	132.439	97.0245	169.766
XLOC_039855	PEX5L	chr1	87755882	87792398	9.09195	10.0605	10.6671	4.88919	4.90327	5.65878	7.5791	6.63517	7.38502
XLOC_039856	PEX5L	chr1	87755882	87792398	0.0	9.63352	2.29037	3.41203	2.70834	3.00621	6.40744	4.58024	6.36041
XLOC_039857	PEX5L	chr1	87792766	87793372	0.0	1.44591	0.0	0.144431	0.752023	0.333125	0.14425	0.479528	0.702463
XLOC_089519	PEX7;MAP3K5	chr9	75775377	75840289	1.45702	0.762956	0.285071	0.0653293	0.028595	0.0	0.0	0.0	0.0
XLOC_089520	PEX7;SLC35D3	chr9	75873103	75899756	2.12652	0.636269	0.998485	0.0	0.0	0.0442515	0.0	0.0	0.0
XLOC_050529	PFKP	chr13	45507475	45546428	7.04858	8.88717	8.66724	2.0314	1.97397	2.7222	2.20558	3.08563	3.21479
XLOC_051752	PFKP	chr13	45550501	45551641	3.68065	2.20188	4.03117	0.131974	0.172845	0.229331	0.334576	0.147496	0.257337
XLOC_051753	PFKP	chr13	45553240	45554393	5.81213	4.5636	3.97856	0.521008	0.284336	0.301799	0.066053	0.363967	0.317481
XLOC_041444	PGGT1B;CCDC112	chr10	3972395	4156114	2.67692	3.15118	3.49235	44.4488	43.6294	43.8595	51.0123	38.6656	42.5598
XLOC_062244	PGLYRP1;MIR769	chr18	53918406	53956128	10.2437	11.3107	10.1588	3.93243	4.6109	4.55069	6.67254	5.11708	4.64578
XLOC_082733	PGPEP1	chr7	4792459	4794050	4.99529	4.26914	3.34967	7.61245	8.99393	9.41269	9.34494	10.6553	5.36394

XLOC_083373	PGPEP1	chr7	4782323	4789870	1.18415	1.76051	1.52251	7.30191	3.44878	5.24107	3.1086	4.58957	3.07191
XLOC_084059	PGPEP1	chr7	4791509	4791753	0.0	4.84879	0.0	8.04574	1.82295	0.817471	0.663457	1.51593	4.18917
XLOC_084060	PGPEP1	chr7	4795194	4795450	7.20632	0.0	0.0	10.3549	5.94835	11.6198	7.12173	9.46559	8.67339
XLOC_084061	PGPEP1	chr7	4795830	4796074	8.12627	0.0	0.0	10.24	8.50712	5.7223	7.96148	6.06371	7.68015
XLOC_084062	PGPEP1	chr7	4796206	4796413	0.0	0.0	0.0	2.37938	3.84653	3.91454	0.0	5.94233	3.35845
XLOC_084063	PGPEP1	chr7	4797146	4797371	0.0	6.04866	0.0	4.58093	9.0264	7.10913	4.0694	4.6709	7.82355
XLOC_084064	PGPEP1	chr7	4797524	4797855	8.32448	4.96519	0.0	3.72198	8.88598	8.89825	5.70082	7.61237	5.39365
XLOC_084065	PGPEP1	chr7	4798300	4798443	0.0	0.0	0.0	21.5846	10.1678	7.23481	0.0	0.0	12.5608
XLOC_055381	PGR	chr15	8109247	8158178	29.4624	30.1079	24.255	51.2849	44.749	47.5318	52.715	59.5975	58.2407
XLOC_055382	PGR	chr15	8168751	8170389	4.32696	8.60924	6.80943	3.76318	4.04268	3.57365	3.25161	3.50838	3.17125
XLOC_055383	PGR	chr15	8171636	8247289	12.3569	15.51	18.8202	5.60973	5.91594	6.10364	5.69624	5.92447	4.43947
XLOC_055702	PGR	chr15	7873056	8099226	4.78094	12.8135	9.71453	12.1837	6.50007	12.2459	12.0857	19.7754	11.5051
XLOC_056092	PGR	chr15	7873056	8099226	5.7744	6.03288	4.50738	0.0	2.66586	1.18363	1.76691	2.25471	1.75273
XLOC_056093	PGR	chr15	7873056	8099226	1.54715	4.16168	0.0	3.04854	1.32336	3.51686	3.1867	3.06936	2.29184
XLOC_056094	PGR	chr15	7873056	8099226	5.1757	3.86343	4.04123	8.10401	5.58823	3.71966	3.86642	5.57706	6.96442
XLOC_056095	PGR	chr15	7873056	8099226	6.133	2.88235	3.42656	1.88464	2.32887	2.45441	2.38308	1.75248	1.91329
XLOC_056096	PGR	chr15	8158875	8159342	30.382	40.482	45.6315	62.9544	61.7622	62.4788	77.9874	76.6927	96.4561
XLOC_056097	PGR	chr15	8159520	8160163	0.0	1.78115	3.49364	8.67361	7.88107	9.54703	11.4857	10.6474	12.2052
XLOC_056098	PGR	chr15	8160272	8161504	2.69239	1.61077	1.05321	2.95671	2.84626	2.72709	3.18537	2.02457	3.82454
XLOC_056099	PGR	chr15	8161673	8161872	0.0	8.91369	0.0	1.36317	8.73711	4.45879	11.6552	0.0	5.10634
XLOC_056100	PGR	chr15	8162171	8162732	3.58517	1.6069	0.0	1.76562	1.94764	1.84935	2.07758	1.95012	1.71667
XLOC_056101	PGR	chr15	8171636	8247289	1.79361	1.8774	2.80577	0.401933	1.12158	0.558203	0.406413	0.627665	0.626629
XLOC_060088	PGRMC2	chr17	29839548	29888262	4.86107	6.52821	4.66566	1.41763	0.781115	0.832286	0.573043	1.57008	1.35539
XLOC_084485	PHAX	chr7	28558619	28559224	17.7794	17.8727	17.6859	26.4899	22.108	24.3719	35.1287	33.4803	20.9799
XLOC_083502	PHAX;ALDH7A1	chr7	28559330	28598112	20.896	22.2444	25.5879	39.6168	36.0047	40.7276	39.3991	50.0634	30.7669
XLOC_084486	PHAX;ALDH7A1	chr7	28559330	28598112	0.0	0.325456	0.0	0.0	0.339794	0.563909	0.196595	0.217146	0.190004
XLOC_084487	PHAX;ALDH7A1	chr7	28559330	28598112	0.804705	0.361168	0.314875	0.469036	0.56837	0.628041	0.441628	0.323802	0.457685
XLOC_084488	PHAX;ALDH7A1	chr7	28559330	28598112	0.0	0.366554	0.479349	0.27463	0.623986	0.254682	0.502372	0.430299	0.535673
XLOC_084489	PHAX;ALDH7A1	chr7	28559330	28598112	0.804955	0.481516	0.0	0.432909	0.692637	0.501329	0.803858	0.886323	0.49232
XLOC_084490	PHAX;ALDH7A1	chr7	28559330	28598112	0.0	1.65607	1.44365	0.992533	0.286638	0.381078	0.0	0.365193	0.482439

XLOC_084491	PHAX;ALDH7A1	chr7	28559330	28598112	0.0	1.28797	1.68411	0.192976	0.333668	0.665775	0.763792	0.42457	0.562348
XLOC_084492	PHAX;ALDH7A1	chr7	28559330	28598112	0.0	0.0	1.8558	0.637941	0.462708	0.737309	0.320925	0.472916	0.207077
XLOC_084493	PHAX;ALDH7A1	chr7	28559330	28598112	0.0	0.471473	2.46604	0.282572	0.613189	0.651859	0.847163	1.40781	0.412337
XLOC_084494	PHAX;ALDH7A1	chr7	28559330	28598112	0.0	0.0	0.0	0.295946	0.253824	0.0	0.287223	0.0	0.286592
XLOC_084495	PHAX;ALDH7A1	chr7	28559330	28598112	0.713613	0.640372	1.11656	1.2794	0.837944	0.592925	1.23301	1.07268	0.623714
XLOC_084496	PHAX;ALDH7A1	chr7	28559330	28598112	0.639754	0.956892	2.50268	0.91766	0.851886	0.930655	0.87388	0.705857	1.00676
XLOC_084497	PHAX;ALDH7A1	chr7	28559330	28598112	0.0	0.603716	0.789466	0.633226	0.709495	0.627884	0.456481	1.0079	0.616924
XLOC_078471	PHB2	chr5	103857454	103858037	1.69989	0.507982	0.0	1.52226	0.792126	1.22831	1.97348	1.34644	3.2569
XLOC_065833	PHC2;ZNF362	chr2	121161233	121309076	13.1736	22.5385	16.9176	13.7679	15.0575	14.7244	14.2577	14.0267	14.347
XLOC_065834	PHC2;ZNF362	chr2	121161233	121309076	5.11164	4.94196	5.13847	10.9164	9.98013	8.82965	12.0174	18.4165	7.72284
XLOC_066500	PHC2;ZNF362	chr2	121161233	121309076	7.9349	7.4442	5.7038	2.4357	2.78401	2.56364	3.25293	2.29918	2.87795
XLOC_066501	PHC2;ZNF362	chr2	121161233	121309076	30.2444	31.0863	25.8918	20.7001	18.3411	20.2508	15.26	16.8042	13.5942
XLOC_068418	PHC2;ZNF362	chr2	121161233	121309076	1.08527	2.92033	0.848738	0.87535	0.677695	0.337395	0.784069	0.433188	0.947257
XLOC_068419	PHC2;ZNF362	chr2	121161233	121309076	0.0	1.23297	3.22458	1.47823	2.3974	1.27539	1.28156	2.64487	3.05141
XLOC_068420	PHC2;ZNF362	chr2	121161233	121309076	3.30521	4.28244	6.03089	2.66562	2.40742	2.16885	1.69101	2.85702	3.26935
XLOC_068421	PHC2;ZNF362	chr2	121161233	121309076	0.0	2.45464	0.0	4.4166	3.76692	4.60958	3.87788	2.37857	4.62256
XLOC_068422	PHC2;ZNF362	chr2	121161233	121309076	1.57081	6.10258	7.36619	0.984987	1.09919	1.13593	1.12479	0.934801	0.410667
XLOC_068423	PHC2;ZNF362	chr2	121161233	121309076	1.36095	1.6272	3.19181	0.731675	0.741993	0.844684	0.978269	0.541401	0.830713
XLOC_038493	PHC3;PRKCI	chr1	97897801	98095629	23.7122	15.0268	28.209	2.47382	2.04319	2.23152	2.58286	2.46383	3.12831
XLOC_038494	PHC3;PRKCI	chr1	97897801	98095629	163.145	135.49	106.777	503.372	465.854	480.612	512.798	553.341	477.402
XLOC_091679	PHF10	chr9	105078334	105079538	2.07333	0.413461	0.0	0.185863	0.324666	0.430731	0.251501	0.207828	0.604094
XLOC_048611	PHF11	chr12	19175862	19178616	0.278537	0.0833522	0.0	1.44885	1.00653	1.27633	0.89377	1.37507	0.755889
XLOC_048612	PHF11	chr12	19179388	19180817	0.0	0.170167	0.0	0.764957	0.401214	0.591295	0.311198	0.285513	0.348189
XLOC_059691	PHF17	chr17	29369093	29379277	0.629166	0.564636	0.492253	1.00464	0.874066	1.04609	0.229213	0.494016	0.306884

XLOC_052292	PHF20	chr13	65641624	65642955	0.0	0.18437	0.964413	0.773548	0.289703	0.192146	0.280742	0.742023	0.269431
XLOC_052293	PHF20	chr13	65649253	65649536	0.0	3.41019	4.45803	14.8538	15.5571	16.2043	13.8919	17.9079	21.1801
XLOC_052294	PHF20	chr13	65661208	65661838	0.0	0.0	1.19654	0.274212	0.833337	0.316363	0.274254	0.151874	0.400186
XLOC_055516	PHF21A	chr15	76879248	76881098	4.66666	5.31798	4.37466	6.73426	6.38366	7.12294	4.95117	7.6038	4.88894
XLOC_055517	PHF21A	chr15	76887881	76890294	14.4818	14.0756	10.2648	47.6461	42.7242	50.0053	45.8736	50.7349	46.0652
XLOC_055518	PHF21A	chr15	76899707	76906330	642.736	672.498	565.204	2389.19	2081.57	2143.06	2104.71	2322.28	2056.42
XLOC_055519	PHF21A	chr15	76948230	76950974	16.4037	15.9269	12.9083	14.7783	14.0872	13.5122	18.4625	22.6952	16.2478
XLOC_055843	PHF21A	chr15	76854150	76871431	6.7785	13.7287	9.41375	6.67987	7.77205	4.81217	6.63572	9.36559	7.25845
XLOC_055844	PHF21A	chr15	76911211	76925419	2.06543	1.85349	1.61589	4.52282	6.93299	5.10884	2.44291	3.45072	3.24972
XLOC_056998	PHF21A	chr15	76851593	76852034	12.7568	7.61812	3.98438	2.28285	3.93641	2.096	2.01937	5.0007	2.21518
XLOC_056999	PHF21A	chr15	76852223	76853930	13.5358	10.6134	8.03545	4.68739	4.94249	4.56383	3.92302	4.97312	4.69568
XLOC_057000	PHF21A	chr15	76881342	76881554	0.0	7.23376	9.45423	14.3021	3.57439	13.3168	7.68302	6.63583	5.18966
XLOC_057001	PHF21A	chr15	76881656	76882208	0.0	3.83484	1.43269	2.4625	2.27592	2.8366	3.92014	3.08105	2.23437
XLOC_057002	PHF21A	chr15	76882500	76882685	0.0	11.6708	0.0	5.39967	1.41426	9.68674	2.9954	5.20663	5.0029
XLOC_057003	PHF21A	chr15	76883456	76883681	0.0	3.02433	0.0	7.32949	3.761	6.09354	3.25552	4.6709	6.08499
XLOC_057004	PHF21A	chr15	76885073	76885308	0.0	5.3564	0.0	7.28452	3.34494	3.60552	2.90959	1.66567	8.47683
XLOC_057005	PHF21A	chr15	76886933	76887162	19.2634	2.87646	0.0	4.3525	2.86685	0.966831	0.0	2.67296	1.6543
XLOC_057006	PHF21A	chr15	76890754	76891048	21.0839	20.4271	12.3251	70.7567	69.8569	83.8572	67.9353	74.799	76.3119
XLOC_057007	PHF21A	chr15	76891513	76891702	35.7085	69.8493	42.1379	155.358	116.29	135.749	117.874	110.792	158.26
XLOC_057008	PHF21A	chr15	76892204	76892481	0.0	0.0	4.67411	6.44716	1.80982	3.03125	1.00106	3.97376	2.58113
XLOC_057009	PHF21A	chr15	76894660	76895079	2.76587	2.47712	2.15923	2.47436	3.19524	4.53872	2.66553	3.24451	2.87959
XLOC_057010	PHF21A	chr15	76906477	76907252	0.0	3.15182	0.915891	4.1979	4.01957	3.39652	3.27418	5.13564	2.65727
XLOC_057011	PHF21A	chr15	76907409	76907750	0.0	0.0	0.0	4.22474	1.50326	1.605	0.338228	1.89939	2.38239
XLOC_057012	PHF21A	chr15	76907957	76908266	0.0	0.0	0.0	1.70265	2.16879	1.44953	4.44119	2.27683	0.821034
XLOC_057013	PHF21A	chr15	76910369	76910914	1.86661	1.11543	2.91706	2.00552	4.63285	2.11732	4.32094	3.13546	2.27447
XLOC_057014	PHF21A	chr15	76911211	76925419	0.0	0.0	1.37695	0.473336	0.410334	0.545445	0.157176	0.0	0.153417
XLOC_078744	PHF5A	chr5	113086067	113087744	5.23564	2.70571	4.46947	0.72552	1.11993	0.891129	0.478236	2.20051	1.12419
XLOC_078745	PHF5A	chr5	113088052	113088945	5.90017	9.11608	9.99818	2.29128	2.91887	2.03921	1.95757	4.81267	1.97486
XLOC_076735	PHLDA1	chr5	5539143	5559598	20.3952	15.0077	21.3635	31.4346	28.3933	30.5486	27.2045	31.516	34.3379
XLOC_077254	PHLDA1	chr5	5539143	5559598	0.0	0.414588	0.0	1.61513	1.5118	0.573804	0.996587	0.551385	1.3302
XLOC_072594	PHTF2	chr4	43370493	43565415	10.0707	17.8396	12.1348	4.16434	4.03523	4.53044	5.37056	9.49499	7.22674
XLOC_072595	PHTF2	chr4	43370493	43565415	17.367	16.637	20.0625	2.74749	2.18354	2.48612	1.87421	2.52299	1.71153
XLOC_072596	PHTF2	chr4	43370493	43565415	7.7002	4.93821	5.11255	7.37265	8.82958	6.83053	10.9104	8.45022	11.5046

XLOC_072597	PHTF2	chr4	43370493	43565415	17.9246	35.1809	34.2913	4.64742	6.03132	5.3121	7.59976	5.44829	7.55534
XLOC_072598	PHTF2	chr4	43565583	43585135	12.5994	11.5413	11.2427	1.31799	1.77928	1.4632	2.1137	1.33998	2.11664
XLOC_072599	PHTF2	chr4	43565583	43585135	4.54671	6.032	4.67602	4.05832	4.8188	3.73878	3.40045	3.64232	3.72555
XLOC_072600	PHTF2	chr4	43631542	43651969	3.13469	3.53399	4.34065	0.215567	0.173751	0.0716498	0.128719	0.185846	0.2952
XLOC_073141	PHTF2	chr4	43370493	43565415	60.3735	54.2921	63.8815	34.5361	20.6696	28.3984	28.6822	47.4931	20.2453
XLOC_073142	PHTF2	chr4	43370493	43565415	1.86195	0.0	2.09415	0.445455	0.0	0.0	0.0	0.0	0.0
XLOC_073143	PHTF2	chr4	43370493	43565415	0.98379	0.981385	3.51347	16.9514	20.6611	17.8502	17.8881	12.4368	19.5828
XLOC_073144	PHTF2	chr4	43370493	43565415	14.8378	11.6354	17.8288	10.622	8.3931	10.9457	18.3646	14.6697	14.052
XLOC_073145	PHTF2	chr4	43370493	43565415	59.1403	44.8017	48.4794	29.5616	19.8256	24.2098	22.0741	41.8488	17.7914
XLOC_073146	PHTF2	chr4	43565583	43585135	1.96639	3.58071	3.14246	3.15627	2.81332	3.08926	3.35334	4.72647	3.06213
XLOC_073147	PHTF2	chr4	43631542	43651969	0.0	0.0	0.0	0.170711	0.0871428	0.0990084	0.0257703	0.191287	0.0919405
XLOC_074047	PHTF2	chr4	43370493	43565415	1.58001	3.78065	1.85448	0.496519	0.742257	0.4105	0.502652	0.395723	1.51916
XLOC_074048	PHTF2	chr4	43370493	43565415	0.0	2.87647	22.5637	0.880708	0.723623	0.0	1.55912	1.78488	0.0
XLOC_074049	PHTF2	chr4	43370493	43565415	0.912628	0.818802	2.14205	0.164546	0.357327	0.0	0.662043	0.182761	0.638267
XLOC_074050	PHTF2	chr4	43370493	43565415	3.95859	4.72282	0.0	0.712106	0.0	0.0	0.682117	1.14682	0.344768
XLOC_074051	PHTF2	chr4	43370493	43565415	0.0	1.29759	2.03677	0.234232	0.339871	0.0	0.315255	0.521034	0.152173
XLOC_074052	PHTF2	chr4	43370493	43565415	12.6038	0.940369	4.91994	0.285121	0.486273	0.324209	0.276008	0.920966	0.548124
XLOC_074053	PHTF2	chr4	43370493	43565415	2.7917	0.939779	2.18509	0.594951	0.274244	0.290803	0.639223	0.35139	0.549966
XLOC_074054	PHTF2	chr4	43370493	43565415	0.0	2.6939	0.0	0.136125	0.585374	0.621851	0.270144	0.298659	0.65557
XLOC_074055	PHTF2	chr4	43370493	43565415	0.0	0.991984	2.59601	1.19269	0.512378	1.36135	0.578808	0.323951	0.577968
XLOC_074056	PHTF2	chr4	43370493	43565415	1.65326	1.97628	5.16947	0.445907	0.38658	0.513052	0.296496	0.164315	1.87315
XLOC_074057	PHTF2	chr4	43370493	43565415	1.30438	2.33966	3.06028	0.585651	0.712359	0.405662	0.118096	0.519504	1.13847
XLOC_074058	PHTF2	chr4	43370493	43565415	2.3144	2.3075	0.603988	0.899789	0.78531	0.641199	0.841431	0.772797	1.4162
XLOC_074059	PHTF2	chr4	43370493	43565415	0.938688	0.842161	0.0	0.42162	0.220755	0.389951	0.680619	0.656956	1.06631
XLOC_074060	PHTF2	chr4	43370493	43565415	0.0	2.96384	1.93923	0.0	0.576421	0.511221	1.96783	1.46105	1.0791
XLOC_074061	PHTF2	chr4	43370493	43565415	2.35786	3.52483	0.922598	0.846287	1.10421	1.22156	0.638446	0.705198	0.617978
XLOC_074062	PHTF2	chr4	43370493	43565415	0.0	9.1656	0.0	0.463877	0.0	0.0	0.0	0.978933	0.0
XLOC_074063	PHTF2	chr4	43370493	43565415	2586.21	4482.64	3551.79	29929.8	20073.4	21170.5	22517.9	18089.2	22226.6
XLOC_074064	PHTF2	chr4	43370493	43565415	0.0	1.09567	1.43385	0.822757	0.997085	0.757454	1.30778	2.17545	1.1184
XLOC_074065	PHTF2	chr4	43370493	43565415	0.0	0.653863	0.0	0.0	0.342144	0.113906	0.0	0.0	0.0
XLOC_074066	PHTF2	chr4	43370493	43565415	2.00644	2.39764	1.56881	0.0	0.62329	0.828017	0.535717	0.594401	0.0
XLOC_074067	PHTF2	chr4	43565583	43585135	3.74302	2.796	1.4623	0.670264	0.0	1.15786	0.166622	1.10943	0.325759
XLOC_074068	PHTF2	chr4	43565583	43585135	2.93419	1.7519	0.0	0.0	0.225733	0.300622	1.02522	0.286252	0.508818

XLOC_074069	PHTF2	chr4	43565583	43585135	0.0	2.93401	0.0	0.439597	0.568883	0.50472	1.08183	1.20485	1.2799
XLOC_074070	PHTF2	chr4	43565583	43585135	7.64097	0.0	5.95758	0.687002	0.0	0.769301	0.626631	1.42961	0.0
XLOC_074071	PHTF2	chr4	43565583	43585135	2.78719	1.94498	2.9066	0.582856	0.726029	0.674526	0.420757	0.371417	0.649092
XLOC_074072	PHTF2	chr4	43585256	43587360	4.83018	4.66943	4.07093	2.96538	3.9666	3.51913	2.82239	4.00084	3.31654
XLOC_050790	PHYH	chr13	28257600	28269762	91.7941	90.9289	73.0668	266.547	241.994	248.995	224.521	252.458	276.688
XLOC_086433	PHYHIP;POLR3D;MIR320-1	chr8	70024646	70072837	19.7045	19.5295	18.0949	16.3767	19.198	18.2312	31.9838	30.9239	16.5971
XLOC_086434	PHYHIP;POLR3D;MIR320-1	chr8	70024646	70072837	2.66406	2.38633	2.07997	2.62182	1.43746	2.46058	3.74111	1.82525	2.77466
XLOC_086900	PHYHIP;POLR3D;MIR320-1	chr8	70024646	70072837	10.7136	11.7727	13.0371	13.5411	14.3096	11.3002	12.2053	11.248	12.755
XLOC_087986	PHYHIP;POLR3D;MIR320-1	chr8	70024646	70072837	3.73787	5.21505	7.79358	4.46515	5.73007	3.4824	2.91712	2.35693	3.47806
XLOC_087987	PHYHIP;POLR3D;MIR320-1	chr8	70024646	70072837	12.5614	5.0032	8.17735	6.55907	6.3214	4.95847	4.64075	5.36377	4.00549
XLOC_087988	PHYHIP;POLR3D;MIR320-1	chr8	70024646	70072837	4.44441	7.9649	6.94296	1.98892	3.60936	2.5152	3.34186	1.96779	1.35222
XLOC_087989	PHYHIP;POLR3D;MIR320-1	chr8	70024646	70072837	0.0	12.098	7.90553	3.66474	0.752601	3.04696	8.13881	0.934587	1.73892
XLOC_087990	PHYHIP;POLR3D;MIR320-1	chr8	70024646	70072837	3.71617	3.61195	4.3599	2.16484	1.88766	1.25271	1.68299	1.3	1.37935
XLOC_087991	PHYHIP;POLR3D;MIR320-1	chr8	70024646	70072837	16.5211	6.56695	8.58443	3.45091	6.2481	2.23043	3.23724	2.61728	2.84653
XLOC_087992	PHYHIP;POLR3D;MIR320-1	chr8	70024646	70072837	3.31409	8.91371	11.6556	2.96791	2.8323	4.27741	2.07334	1.96986	1.87638
XLOC_087993	PHYHIP;POLR3D;MIR320-1	chr8	70024646	70072837	4.89683	2.05018	3.06388	2.36975	2.52463	2.33564	2.48151	3.22822	2.30896
XLOC_087994	PHYHIP;POLR3D;MIR320-1	chr8	70024646	70072837	3.3092	12.8373	5.16422	6.80676	7.36104	4.73701	10.0528	9.32307	5.15877
XLOC_076971	PICK1;BAIAP2L2	chr5	110361888	110448216	28.4353	53.0129	61.4681	30.7319	31.4045	25.8947	31.1154	36.6617	27.4767

XLOC_078675	PICK1;BAIAP2L2	chr5	110361888	110448216	1.23743	5.54931	10.6429	1.33044	1.06107	1.02481	0.445787	0.739265	0.863579
XLOC_078676	PICK1;BAIAP2L2	chr5	110361888	110448216	5.19393	6.21358	7.4483	0.698325	1.1506	0.718574	0.628003	0.519522	1.20989
XLOC_078677	PICK1;BAIAP2L2	chr5	110361888	110448216	0.578823	4.32905	3.62315	0.986022	0.81649	0.722003	0.369388	1.33632	0.961691
XLOC_078678	PICK1;BAIAP2L2	chr5	110361888	110448216	4.70804	7.39014	4.6018	0.632808	1.28514	1.21892	1.59185	0.234567	1.027
XLOC_078679	PICK1;BAIAP2L2	chr5	110361888	110448216	2.39384	2.50648	7.96024	1.18042	0.89095	0.248803	0.599942	1.1411	0.575617
XLOC_078680	PICK1;BAIAP2L2	chr5	110361888	110448216	1.45093	2.60219	3.40268	1.16975	1.46784	0.750022	0.520657	1.15274	1.26474
XLOC_078681	PICK1;BAIAP2L2	chr5	110361888	110448216	4.3969	1.75236	2.29141	1.05031	0.456165	0.454532	0.394343	0.873163	1.40524
XLOC_078682	PICK1;BAIAP2L2	chr5	110361888	110448216	1.642	2.94431	5.13331	1.17648	0.510335	0.339114	0.880747	0.650759	0.572117
XLOC_078683	PICK1;BAIAP2L2	chr5	110361888	110448216	1.13277	5.08047	6.20062	0.507549	0.883763	1.29077	1.12439	1.12934	0.494274
XLOC_078684	PICK1;BAIAP2L2	chr5	110361888	110448216	3.32721	4.72688	9.10927	0.745596	0.780638	0.517964	0.754779	0.582648	1.09005
XLOC_078685	PICK1;BAIAP2L2	chr5	110361888	110448216	3.66216	2.18981	0.954506	0.875018	1.14205	0.505537	0.219951	0.729431	0.745474
XLOC_078686	PICK1;BAIAP2L2	chr5	110361888	110448216	2.03296	3.44719	5.30356	1.12427	0.957755	0.705456	0.806743	1.02335	0.978608
XLOC_078687	PICK1;BAIAP2L2	chr5	110361888	110448216	2.88497	1.94234	4.7979	1.26124	1.24567	0.825805	0.990331	1.01629	0.915234
XLOC_078688	PICK1;BAIAP2L2	chr5	110361888	110448216	1.37376	0.821352	3.22196	1.10762	0.427885	1.56303	0.370247	0.955903	0.359343
XLOC_078689	PICK1;BAIAP2L2	chr5	110361888	110448216	1.66468	2.98491	2.60205	1.78901	1.93984	1.03125	2.67763	1.6489	1.30493
XLOC_078690	PICK1;BAIAP2L2	chr5	110361888	110448216	2.71402	1.62061	0.0	1.94258	0.418212	0.556872	0.237994	0.265479	0.706527
XLOC_078691	PICK1;BAIAP2L2	chr5	110361888	110448216	3.68218	3.85537	5.76195	1.26547	0.913519	0.892893	1.23008	0.862051	1.18048
XLOC_078692	PICK1;BAIAP2L2	chr5	110361888	110448216	1.56051	0.466441	4.87897	1.95677	0.970649	0.967341	3.35314	1.70245	1.9036
XLOC_078693	PICK1;BAIAP2L2	chr5	110361888	110448216	14.2121	17.0985	21.5659	14.4626	15.3101	14.9205	18.9022	23.8417	13.4058
XLOC_078694	PICK1;BAIAP2L2	chr5	110361888	110448216	0.0	0.0	32.6531	15.255	11.2547	9.73182	20.7915	16.4189	15.5147
XLOC_078695	PICK1;BAIAP2L2	chr5	110361888	110448216	9.79349	0.974259	7.64203	7.59096	7.26386	3.33865	7.65604	7.29785	7.35179
XLOC_078696	PICK1;BAIAP2L2	chr5	110361888	110448216	3.3476	2.92183	4.58522	3.00226	3.7036	2.90521	3.60614	3.09165	3.39453

XLOC_078697	PICK1;BAIAP2L2	chr5	110361888	110448216	5.06842	2.72816	2.37836	3.36116	4.82886	3.99322	5.13368	2.12539	2.47804
XLOC_078698	PICK1;BAIAP2L2	chr5	110361888	110448216	5.69766	4.68684	7.24335	6.12908	5.96563	4.95601	6.02354	4.71057	6.66062
XLOC_078699	PICK1;BAIAP2L2	chr5	110361888	110448216	9.05269	2.705	12.7332	5.83623	6.46254	4.48238	7.26128	7.33982	5.98931
XLOC_078700	PICK1;BAIAP2L2	chr5	110361888	110448216	5.00971	4.11878	1.95852	5.61045	7.02853	6.09317	7.21706	5.48595	4.26075
XLOC_041528	PIF1;PLEKHO2	chr10	45289459	45318448	1.05605	4.10773	2.47922	0.236562	0.248238	0.16452	0.0	0.37113	0.0
XLOC_041628	PIGH;PIGH	chr10	79784526	79820891	4.59083	3.66086	3.59411	10.6533	11.4019	12.9147	6.8802	8.32455	8.39706
XLOC_042032	PIGH;PIGH	chr10	79784526	79820891	0.0	0.0	0.0	0.095116	0.0831682	0.330917	0.0968024	0.106549	0.139174
XLOC_057999	PIGR	chr16	4480142	4541858	3.70131	5.48024	4.88299	9.59301	10.4069	11.997	10.0931	8.17401	8.36369
XLOC_058000	PIGR	chr16	4544899	4560813	9.72226	7.55319	7.9268	36.6019	34.3718	39.4989	36.0186	40.3261	28.9021
XLOC_058267	PIGR	chr16	4544289	4544791	0.0	0.0	0.0	0.939725	0.650191	0.0	0.186148	0.206879	0.182589
XLOC_058268	PIGR	chr16	4544899	4560813	0.0	0.0	0.0	1.82129	1.15738	2.58005	0.429785	1.45634	0.877366
XLOC_058269	PIGR	chr16	4544899	4560813	0.0	0.0	0.0	4.68344	0.792909	3.71282	2.2055	5.98327	1.35312
XLOC_052516	PIGT	chr13	74476600	74477498	0.976721	0.876249	3.05561	0.787786	1.22076	0.50638	0.883904	0.975624	1.27928
XLOC_076400	PIK3C2G	chr5	91949271	91970687	3.36159	3.30886	3.91337	2.76168	2.34702	2.76897	1.95954	3.25589	1.75602
XLOC_076401	PIK3C2G	chr5	92137884	92202863	24.4948	18.5146	21.7424	14.2568	14.2783	17.8934	20.1574	19.8898	10.0961
XLOC_076908	PIK3C2G	chr5	91949271	91970687	29.1389	25.5017	22.6924	25.199	21.5266	22.0188	20.5311	25.6937	18.5025
XLOC_076909	PIK3C2G	chr5	92003445	92042987	1.77942	3.71634	6.52333	2.606	1.24847	1.69431	2.27159	2.7483	2.07382
XLOC_076910	PIK3C2G	chr5	92051982	92137616	41.4323	49.3384	47.5407	124.444	86.4602	108.495	136.309	203.54	114.309
XLOC_076911	PIK3C2G	chr5	92226885	92245484	0.436602	0.261265	1.025	1.52683	1.06189	0.772204	1.35692	0.921941	1.10776
XLOC_076912	PIK3C2G	chr5	92247770	92250669	0.0	0.0	0.0	2.25393	2.2518	0.798453	1.03321	1.33834	2.19063
XLOC_076913	PIK3C2G	chr5	92258240	92278301	3.94608	6.95287	12.1715	1.76359	2.16716	2.5121	1.27987	0.773328	2.64653
XLOC_078311	PIK3C2G	chr5	91938305	91939164	2.06205	0.924919	4.03165	0.739146	0.966056	0.427483	0.372845	0.308755	0.270029
XLOC_078312	PIK3C2G	chr5	91944096	91945187	0.774772	1.8539	0.0	0.763931	0.969931	0.563059	0.77415	0.465494	1.21863
XLOC_078313	PIK3C2G	chr5	91948494	91949079	3.38387	1.01122	5.28913	0.909088	1.31412	1.92126	0.453354	1.17274	0.58941
XLOC_078314	PIK3C2G	chr5	91949271	91970687	0.0	2.35845	1.54195	0.706738	0.0	0.81352	2.10447	0.58427	0.858528
XLOC_078315	PIK3C2G	chr5	91970832	91971597	3.57172	1.06788	0.0	2.88017	2.22824	2.83559	2.14639	2.72809	1.66205
XLOC_078316	PIK3C2G	chr5	91971718	91972122	0.0	0.875774	0.0	1.04978	0.225676	1.20249	0.256298	1.14501	1.01764
XLOC_078317	PIK3C2G	chr5	91974116	91974532	0.0	0.0	0.0	2.50299	1.07717	1.43453	0.244993	0.546817	0.970889
XLOC_078318	PIK3C2G	chr5	91994922	91995501	0.0	0.0	1.34107	1.84401	1.46583	1.06272	0.459606	0.339739	1.1955
XLOC_078319	PIK3C2G	chr5	92043042	92043701	1.44225	0.4311	2.25489	0.516755	0.336728	0.89469	0.647031	0.28649	0.502885

XLOC_078320	PIK3C2G	chr5	92049178	92050241	0.0	1.43339	0.624813	1.28869	1.74953	1.32659	1.52295	1.03945	2.09368
XLOC_078321	PIK3C2G	chr5	92051040	92051627	0.0	2.01303	5.26454	2.26215	1.56969	2.95553	0.902614	1.6677	3.37342
XLOC_078322	PIK3C2G	chr5	92205003	92205981	1.76296	1.84539	0.0	0.632037	0.275643	0.823094	0.0799122	0.352609	0.846959
XLOC_078323	PIK3C2G	chr5	92226885	92245484	0.0	0.0	0.0	0.232456	0.404337	0.134256	0.817012	0.129083	0.339454
XLOC_038036	PIK3CB	chr1	131286209	131543940	5.06954	3.3276	3.91628	4.79173	3.46777	4.77336	6.27577	5.41628	4.58176
XLOC_038533	PIK3CB	chr1	131286209	131543940	16.9071	17.7949	27.5036	21.5372	19.3543	18.4316	21.1527	16.494	18.1068
XLOC_040234	PIK3CB	chr1	131286209	131543940	5.38676	4.81834	8.39673	2.89272	3.66906	3.27363	1.35917	1.5376	0.9282
XLOC_072636	PIK3CG	chr4	48279752	48422564	2.39523	3.26101	3.78423	1.1401	0.96279	1.32707	0.748268	0.72276	1.12865
XLOC_073168	PIK3CG	chr4	48193976	48279684	27.7255	23.8656	18.892	20.5002	19.5827	18.637	22.1066	19.5399	20.3982
XLOC_083377	PIK3R2	chr7	4992699	4996055	3.87028	3.9146	4.81774	1.21398	0.758887	0.957065	0.792181	1.06325	0.874734
XLOC_084078	PIK3R2	chr7	4992699	4996055	1.1965	1.4309	2.80671	0.750417	0.279899	0.619469	0.323521	0.357583	0.208782
XLOC_070445	PIK3R3	chr3	100563778	100685507	19.593	21.3656	19.7637	10.6213	10.7242	11.257	9.90141	9.51214	10.4738
XLOC_038599	PIK3R4	chr1	153218821	153333049	78.2189	63.4872	66.0736	94.3916	77.5881	84.1917	104.561	126.651	87.0824
XLOC_040640	PIK3R4	chr1	153218821	153333049	7.9222	2.84121	8.66889	3.97332	3.32554	3.60078	2.5523	5.02704	2.62284
XLOC_040641	PIK3R4	chr1	153218821	153333049	2.64212	2.76446	6.19704	1.89357	1.33785	1.64026	2.37593	1.18263	1.84333
XLOC_064218	PIK3R6	chr19	28923414	28929219	0.0	0.0	1.37028	0.157015	0.272248	0.0	0.156438	0.0	0.305355
XLOC_064947	PIK3R6	chr19	28913295	28914234	2.77642	0.0	0.0	0.248831	0.289297	0.191986	0.0	0.185003	0.161652
XLOC_072608	PION	chr4	44106486	44116181	0.0	0.839752	0.0	0.188747	0.329679	0.218695	0.0	0.0	0.184035
XLOC_073150	PION	chr4	44037017	44084259	2.51481	2.65671	3.60192	9.4936	11.0835	8.59471	7.42211	6.4769	8.58717
XLOC_074086	PION	chr4	44106486	44116181	0.0	0.341366	0.892785	0.0	0.0	0.0	0.0	0.0	0.0
XLOC_074087	PION	chr4	44116347	44116925	5.16032	0.0	1.34426	0.308065	0.267139	0.53261	0.30711	0.851325	0.14979
XLOC_074088	PION	chr4	44123516	44124249	1.25678	3.00584	3.9306	5.85506	5.87726	6.24427	7.35471	7.00601	5.4814
XLOC_051372	PIP4K2A	chr13	23933740	23934212	94.297	148.369	98.7971	19.5541	11.9107	14.9018	11.5808	7.23421	3.79741
XLOC_051373	PIP4K2A	chr13	23934443	23934988	95.1973	105.408	116.682	29.4143	17.3732	27.9101	17.45	11.4352	10.56
XLOC_070705	PIP5K1A	chr3	19628446	19628627	0.0	6.37033	0.0	13.7958	0.0	6.33631	1.62555	3.77017	3.6386
XLOC_064207	PITPNA;SLC43A2;INPP5K	chr19	23206922	23273950	23.8202	27.9679	23.4929	30.92	37.0294	33.6756	39.8304	33.8236	40.3336
XLOC_064772	PITPNA;SLC43A2;INPP5K	chr19	23206922	23273950	14.8381	14.0302	9.65531	14.6057	19.0883	12.9579	20.0354	8.24737	20.832
XLOC_083175	PJA2	chr7	111042544	111052375	28.0845	34.3584	38.6032	31.6845	24.3856	29.1518	25.8898	34.9905	25.9592
XLOC_083176	PJA2	chr7	111052862	111056514	12.5597	14.6561	14.7433	13.252	11.3666	11.0211	11.3315	13.7689	11.1725
XLOC_083835	PJA2	chr7	111069640	111082843	215.735	205.059	191.817	309.395	283.151	326.216	350.027	397.939	260.445

XLOC_083836	PJA2	chr7	111082904	111122717	1.12215	1.98498	3.45648	4.98912	4.72711	6.42847	3.22577	4.10214	3.28663
XLOC_085648	PJA2	chr7	111063868	111064013	0.0	20.2094	0.0	6.5265	18.5825	6.59295	77.2951	22.4332	28.6025
XLOC_085649	PJA2	chr7	111068709	111068847	0.0	0.0	0.0	18.8009	13.0249	28.0057	87.934	7.84287	16.2275
XLOC_080072	PKD2	chr6	38041589	38097093	0.826308	0.206152	0.0	10.5646	8.2931	10.8094	7.24616	8.2481	10.9651
XLOC_090466	PKIB	chr9	28995041	28995881	0.0	0.316881	0.0	0.0949628	0.0	0.109827	0.0	0.0	0.0
XLOC_089806	PKIB;SERINC1;HSF2	chr9	28996031	29412499	0.0	0.74787	0.258519	0.111767	0.0796181	0.0	0.0605079	0.126405	0.0867322
XLOC_090467	PKIB;SERINC1;HSF2	chr9	28996031	29412499	0.0	0.0	0.0	0.231104	0.0	0.0	0.0	0.0	0.0
XLOC_050631	PKIG;ADA	chr13	73733307	73819287	10.7019	12.9637	11.7058	13.7955	16.9873	13.6489	19.5599	17.2036	18.6172
XLOC_052483	PKIG;ADA	chr13	73733307	73819287	0.0	8.29747	3.616	9.12654	9.87187	6.59638	10.6435	6.2208	6.8038
XLOC_052484	PKIG;ADA	chr13	73733307	73819287	1.79135	3.48228	4.90404	3.93349	4.62081	3.53093	6.08831	4.20913	4.69391
XLOC_052485	PKIG;ADA	chr13	73733307	73819287	18.1524	5.41893	7.08301	9.00917	8.1183	10.9405	7.35241	6.73619	10.9135
XLOC_052486	PKIG;ADA	chr13	73733307	73819287	2.56255	7.27783	10.0178	5.50986	7.28858	2.51948	6.45695	5.86533	6.48176
XLOC_083394	PKN1	chr7	12416076	12422466	0.681334	1.22285	1.06609	1.55094	1.60003	1.06178	1.17848	2.52728	1.34157
XLOC_084176	PKN1	chr7	12413546	12413950	0.0	2.62732	0.0	0.524892	2.48243	3.30684	1.02519	1.14501	3.30731
XLOC_084177	PKN1	chr7	12414141	12415392	0.0	0.989251	1.03492	0.711518	0.828761	0.549724	0.782675	0.663225	0.925105
XLOC_071299	PKN2	chr3	55247180	55247519	0.0	0.0	0.0	0.0	0.303851	0.0	0.0	0.0	0.0
XLOC_076383	PKP2	chr5	77308748	77408033	58.8245	73.2282	62.7904	83.1031	70.5364	76.8766	88.9223	101.087	94.6298
XLOC_076894	PKP2	chr5	77308748	77408033	173.364	114.035	148.138	8.0293	1.8677	5.80614	9.51766	6.01321	7.35517
XLOC_065626	PKP4	chr2	37783069	38287329	0.782509	0.239542	0.0	0.105254	0.104814	0.173253	0.0357944	0.0	0.116795
XLOC_066269	PKP4	chr2	37783069	38287329	6.11896	7.9372	8.66721	6.79703	4.62407	5.63575	6.67771	6.62965	6.60564
XLOC_067374	PKP4	chr2	37783069	38287329	0.429607	0.0	0.0	0.0	0.101121	0.0446972	0.0392746	0.0432016	0.0
XLOC_080017	PLA2G12A;CASP6	chr6	16833130	16856048	0.900245	0.0	0.0	0.0804876	0.0	0.0466904	0.0	0.0	0.0392651
XLOC_080243	PLAC8;COPS4	chr6	99626793	99666811	0.979366	7.02896	6.12776	0.61438	0.841529	0.609292	1.24076	0.586941	0.256547
XLOC_084940	PLAC8L1	chr7	59308237	59309839	12.517	8.98777	10.1864	14.9492	17.3144	16.8185	15.6336	13.9347	22.0725
XLOC_038111	PLCL2	chr1	155557871	155687434	10.0613	17.6632	15.2008	23.6971	18.0085	20.3545	19.0476	23.0075	15.108
XLOC_038604	PLCL2	chr1	155778158	155812881	15.964	15.8346	16.0508	2.19104	2.48914	1.98928	2.4455	2.25171	2.7511
XLOC_040670	PLCL2	chr1	155688086	155689677	0.504523	0.60378	0.789579	0.407131	0.435179	0.629617	0.598778	0.70965	0.617823
XLOC_039988	PLD1	chr1	96645668	96646165	0.0	1.90924	1.66431	1.33495	1.31921	1.09672	0.188785	1.25909	1.66732
XLOC_057886	PLD5	chr16	35154603	35274531	2.98093	2.75782	0.68119	0.753204	0.904788	0.542158	0.0	0.559912	0.823798
XLOC_057887	PLD5	chr16	35279223	35304430	2.32388	3.57626	2.07859	0.476348	0.4953	0.345618	0.0608115	0.367678	0.319622
XLOC_057888	PLD5	chr16	35541308	35664290	14.611	12.1173	14.6303	4.0087	6.11331	4.28434	6.20024	4.50823	6.98182

XLOC_058109	PLD5	chr16	35308088	35363876	5.06811	9.12514	8.68889	9.25353	10.4625	11.657	7.98701	7.13799	13.694
XLOC_058110	PLD5	chr16	35397311	35490929	11.2482	11.8286	7.32096	8.08624	8.84617	9.553	9.66296	9.15127	8.0775
XLOC_058895	PLD5	chr16	35304699	35305880	0.70664	1.69098	0.552828	0.760147	0.663842	0.366973	0.514157	0.424916	0.617635
XLOC_058896	PLD5	chr16	35306664	35307448	0.0	0.0	0.0	0.0	0.450268	0.478296	0.208267	0.115066	0.201481
XLOC_058897	PLD5	chr16	35308088	35363876	0.0	0.0	1.45478	1.00018	1.87732	0.383988	0.0	0.183975	0.97228
XLOC_058898	PLD5	chr16	35541308	35664290	160.265	161.123	119.599	421.855	361.11	420.667	505.338	475.633	427.928
XLOC_058899	PLD5	chr16	35541308	35664290	1.52826	0.914241	0.597768	1.30141	2.09276	0.6347	1.45791	0.918341	1.26871
XLOC_042029	PLEK2;EIF2S1	chr10	79619048	79639787	7.24518	6.31611	6.41162	1.77677	2.29142	1.2488	1.2527	1.99518	1.70899
XLOC_066156	PLEKHA3	chr2	18216889	18392832	12.0205	11.1903	12.2133	3.61184	4.80053	4.18685	4.71198	4.04473	5.14108
XLOC_066954	PLEKHA3	chr2	18216889	18392832	3.7526	1.1212	4.39824	0.335984	0.873099	1.16082	1.00214	0.0	1.79629
XLOC_066955	PLEKHA3	chr2	18216889	18392832	0.0	0.0	0.0	1.23667	0.350409	0.468261	0.391888	0.0	1.59092
XLOC_066956	PLEKHA3	chr2	18216889	18392832	3.54256	4.23669	4.6168	0.846426	1.10511	0.978299	1.38401	0.588291	0.618201
XLOC_042081	PLEKHD1	chr10	81590866	81601081	6.14156	8.35647	3.09178	10.1181	10.0288	9.92255	5.44907	5.82957	7.63427
XLOC_042082	PLEKHD1	chr10	81621792	81627634	7.02458	5.60392	4.58022	14.2752	11.468	14.4206	12.1664	17.9789	8.85546
XLOC_043117	PLEKHD1	chr10	81606343	81606791	2.4899	0.0	0.0	0.891189	2.49822	1.02313	1.09604	0.732569	1.5136
XLOC_043118	PLEKHD1	chr10	81608316	81609819	0.0	0.482438	0.0	1.15665	1.68552	1.34132	0.735634	0.269913	0.564131
XLOC_089553	PLEKHG1	chr9	89066949	89067756	0.0	0.0	1.00892	0.115607	0.502744	0.267086	1.62548	0.898821	1.01294
XLOC_089554	PLEKHG1	chr9	89075248	89075953	4.49971	0.67197	0.0	1.00681	1.7397	0.462913	0.397853	0.663839	0.391094
XLOC_090028	PLEKHG1	chr9	89085234	89092676	0.0	1.37013	0.0	0.404011	0.504919	0.26847	0.438316	0.542068	0.860693
XLOC_091448	PLEKHG1	chr9	89083853	89084365	0.0	0.0	0.0	0.365307	0.316073	0.630536	2.71636	0.201187	1.06485
XLOC_042998	PLEKHG3	chr10	77191530	77191997	0.0	0.0	0.0	3.13726	0.722365	1.68212	0.412632	0.918475	0.406131
XLOC_050514	PLK1S1	chr13	40859552	40958588	3.7788	1.82475	3.64611	33.9014	30.6217	28.0009	43.4614	34.8126	37.5506
XLOC_051699	PLK1S1	chr13	40854248	40854719	0.0	0.0	0.0	2.06488	0.534987	1.66096	2.24172	1.13385	0.801984
XLOC_051700	PLK1S1	chr13	40859552	40958588	1.68015	0.0	0.0	2.55783	2.87118	2.60183	3.75242	2.99503	2.63397
XLOC_051701	PLK1S1	chr13	40859552	40958588	0.0	0.0	0.0	3.82832	2.85462	2.63219	2.4958	4.73649	2.47459
XLOC_051702	PLK1S1	chr13	40859552	40958588	5.5484	0.0	0.0	4.96717	5.87383	6.17858	3.25979	3.16292	4.30127
XLOC_051703	PLK1S1	chr13	40859552	40958588	0.0	0.0	0.0	3.66836	2.88189	5.93827	2.6654	5.30692	5.03109
XLOC_063991	PLXDC1	chr19	40222045	40271587	0.0	0.336069	0.0	1.57253	0.868746	1.06122	0.734163	0.583982	0.358383
XLOC_064250	PLXDC1	chr19	40222045	40271587	14.6394	19.6448	16.3367	65.2186	53.2596	65.8816	47.9089	51.082	44.3609
XLOC_065107	PLXDC1	chr19	40222045	40271587	6.58833	1.96599	0.0	0.294603	0.758097	3.36844	1.42995	1.60037	0.570607
XLOC_065108	PLXDC1	chr19	40222045	40271587	0.0	0.0	0.768049	0.52804	1.07392	1.3237	0.977479	0.294258	0.428736
XLOC_065109	PLXDC1	chr19	40222045	40271587	1.83349	0.0	0.0	1.14917	0.568979	1.51285	0.980034	0.362477	0.79799

XLOC_065110	PLXDC1	chr19	40222045	40271587	1.80702	0.270211	0.706698	1.21465	0.564961	1.12473	1.39188	0.632313	1.02588
XLOC_065111	PLXDC1	chr19	40222045	40271587	0.0	0.0	0.0	0.233208	0.602965	0.802723	0.229	0.0	0.678787
XLOC_065112	PLXDC1	chr19	40222045	40271587	0.0	0.0	1.61897	0.98938	1.18818	0.716521	0.502054	0.345725	0.241178
XLOC_065113	PLXDC1	chr19	40222045	40271587	2.04746	0.306129	0.0	1.10088	0.319758	1.06119	0.647948	0.613194	0.446875
XLOC_065114	PLXDC1	chr19	40291935	40293236	2.52992	3.97328	5.44334	3.51553	4.65715	3.74619	3.57146	3.67981	3.37309
XLOC_050466	PLXDC2	chr13	21715232	21812125	91.4633	92.4916	78.2232	365.075	398.349	386.794	274.683	257.347	277.88
XLOC_050467	PLXDC2	chr13	21715232	21812125	68.2103	27.8712	33.476	324.063	270.119	318.867	38.5424	126.631	119.78
XLOC_050468	PLXDC2	chr13	21944761	21947270	3.12875	3.0459	3.17726	4.34922	3.14656	3.07461	2.95076	2.83535	5.11851
XLOC_050469	PLXDC2	chr13	21949879	21958718	10.467	18.3774	8.05971	19.3703	16.7978	22.8771	24.9001	18.3226	27.5324
XLOC_050761	PLXDC2	chr13	21715232	21812125	13.0831	26.1686	14.7117	81.5746	78.5393	81.3911	110.509	93.4584	104.304
XLOC_050762	PLXDC2	chr13	21715232	21812125	18.2881	12.7689	14.0479	103.11	110.968	111.727	118.66	94.922	83.617
XLOC_050763	PLXDC2	chr13	22002364	22008154	0.0	0.200414	1.16247	1.67812	1.53516	1.78872	2.1242	1.719	2.7047
XLOC_051306	PLXDC2	chr13	21715232	21812125	0.0	0.0	0.0	1.37832	0.353539	0.281531	0.327906	0.361769	0.632099
XLOC_051307	PLXDC2	chr13	21715232	21812125	0.0	0.0	0.0	2.79716	1.02001	1.36269	1.90354	0.42877	0.385708
XLOC_051308	PLXDC2	chr13	21715232	21812125	0.0	0.142406	0.372481	1.96317	2.46385	2.7229	0.956473	0.765394	0.624552
XLOC_051309	PLXDC2	chr13	21715232	21812125	0.732196	0.328636	0.286533	0.558109	0.546047	0.419159	0.63667	0.147379	0.128154
XLOC_051310	PLXDC2	chr13	21715232	21812125	0.0	1.86278	0.0	8.96057	1.8834	5.04963	1.55989	1.18063	0.537699
XLOC_051311	PLXDC2	chr13	21715232	21812125	0.0	0.0	0.0	5.80199	5.47377	8.3003	5.20276	3.40563	3.15461
XLOC_051312	PLXDC2	chr13	21715232	21812125	40.622	57.3157	49.396	198.396	223.969	216.702	170.251	154.397	167.98
XLOC_051313	PLXDC2	chr13	21715232	21812125	0.0	0.463401	0.0	0.277734	0.482242	0.320393	0.277684	0.0	0.135103
XLOC_051314	PLXDC2	chr13	21715232	21812125	0.0	0.0	3.49302	2.20131	0.691654	0.690135	0.19775	0.219951	0.0
XLOC_051315	PLXDC2	chr13	21715232	21812125	0.0	0.311861	1.6313	0.280374	0.325702	0.108096	0.0942676	0.0	0.182089
XLOC_051316	PLXDC2	chr13	21715232	21812125	0.0	0.289739	0.75782	1.38926	2.27067	1.90887	1.2277	0.677514	1.86126
XLOC_051317	PLXDC2	chr13	21715232	21812125	0.0	1.02215	0.0	0.30632	0.334043	0.354643	0.154994	0.170947	0.373194
XLOC_051318	PLXDC2	chr13	21715232	21812125	4.16224	1.2413	3.24596	1.86099	0.95207	0.423726	1.42521	0.400656	0.359581
XLOC_051319	PLXDC2	chr13	21855073	21855321	0.0	4.65104	0.0	11.9195	2.33472	7.06273	4.4689	8.01525	8.70957
XLOC_051320	PLXDC2	chr13	21862021	21863067	0.0	0.0	0.0	1.16741	1.14608	0.929396	0.369441	1.38504	0.995689
XLOC_051321	PLXDC2	chr13	21916999	21917161	0.0	0.0	0.0	3.33582	0.0	0.0	2.61755	12.1664	6.02277
XLOC_051322	PLXDC2	chr13	21919297	21919511	11.7408	42.1502	36.7257	8.54129	6.95029	11.7631	9.347	12.9103	3.02476
XLOC_051323	PLXDC2	chr13	21924518	21924935	2.78717	0.0	2.17581	0.997351	0.0	0.571638	0.244095	0.27239	0.72539
XLOC_051324	PLXDC2	chr13	21927936	21928052	0.0	0.0	0.0	1096.6	417.532	56.2877	436.299	317.936	397.035
XLOC_051325	PLXDC2	chr13	21936953	21937095	0.0	0.0	0.0	22.7061	5.32792	15.1853	5.53691	6.42492	39.5554
XLOC_051326	PLXDC2	chr13	21940802	21940914	0.0	0.0	0.0	220.536	67.2669	0.0	70.5007	0.0	0.0

XLOC_051327	PLXDC2	chr13	21947340	21947683	0.0	0.0	3.03786	0.348292	0.892552	1.58814	2.00899	0.0	1.01021
XLOC_051328	PLXDC2	chr13	21960272	21960639	3.45089	2.05928	0.0	1.85161	0.793261	1.76286	1.49406	1.67358	1.79239
XLOC_051329	PLXDC2	chr13	21993880	21994188	0.0	1.42806	0.0	0.0	0.0	0.972543	0.0	0.916414	0.0
XLOC_051330	PLXDC2	chr13	22001251	22001988	0.0	0.0	0.0	0.0	0.680994	0.258396	0.449545	0.124257	0.217749
XLOC_051331	PLXDC2	chr13	22008282	22008876	0.0	0.0	0.0	0.296791	0.257475	0.68438	0.0	0.328298	0.432997
XLOC_057951	PLXNA2;PLXNA2	chr16	77013723	77116861	11.8809	11.4914	10.0922	3.70386	2.68908	3.51868	1.93093	1.97355	2.09385
XLOC_073248	PLXNA4	chr4	96568570	96591531	1.29217	0.777138	1.52726	0.350953	0.357557	0.559887	0.273852	0.090327	0.638153
XLOC_076227	PLXNC1	chr5	24100972	24160865	13.8652	15.8807	14.0945	31.6883	25.3853	30.2206	28.2878	29.0486	30.666
XLOC_076228	PLXNC1	chr5	24165006	24183025	1.07824	1.80424	1.68189	6.48859	6.0631	5.27639	6.85975	9.64713	6.81415
XLOC_077410	PLXNC1	chr5	24100583	24100875	5.34745	6.37639	8.33584	26.8016	21.4546	24.9193	18.9002	18.831	25.3421
XLOC_077411	PLXNC1	chr5	24100972	24160865	4.36154	3.47649	4.54599	2.47429	0.791944	1.35273	1.56503	0.721883	1.14054
XLOC_077412	PLXNC1	chr5	24100972	24160865	2.71402	3.24106	0.0	1.45684	1.04553	1.67062	0.475988	0.796403	1.17755
XLOC_077413	PLXNC1	chr5	24100972	24160865	0.0	0.0	3.12119	1.43149	3.36024	4.0774	2.74764	1.15764	0.691788
XLOC_077414	PLXNC1	chr5	24100972	24160865	1.05509	0.946487	1.65026	0.472738	1.07078	0.437397	0.953447	0.421158	0.460513
XLOC_077415	PLXNC1	chr5	24100972	24160865	9.49783	8.80509	14.8571	26.5575	18.775	22.8519	16.1671	28.376	22.726
XLOC_077416	PLXNC1	chr5	24100972	24160865	2.18345	0.0	2.56124	0.978265	0.596418	0.33936	0.985884	0.326689	0.762301
XLOC_077417	PLXNC1	chr5	24100972	24160865	0.0	0.824439	1.07807	0.741187	0.214749	0.713157	0.990995	0.274138	0.601177
XLOC_077418	PLXNC1	chr5	24100972	24160865	0.0	1.2413	12.983	2.97759	4.44299	3.38981	3.56301	2.80456	5.03407
XLOC_077419	PLXNC1	chr5	24100972	24160865	0.0	3.44851	6.0115	6.20281	7.65565	9.82271	5.30402	3.34941	5.99771
XLOC_077420	PLXNC1	chr5	24162691	24162954	0.0	0.0	0.0	0.606068	1.52362	2.04448	2.2358	3.17894	5.8098
XLOC_076863	PMCH;IGF1	chr5	66298563	66618770	13.3997	13.7219	14.6342	6.31251	7.95478	6.34129	7.46483	5.89271	7.17354
XLOC_078072	PMCH;IGF1	chr5	66298563	66618770	5.27485	2.36538	6.18576	0.354403	1.74634	1.63729	1.18585	0.787006	0.460013
XLOC_054280	PMP2	chr14	46805967	46807007	1.6391	0.980479	0.641084	0.293834	1.15382	0.680497	0.595063	0.984253	1.07404
XLOC_045133	PMPCA;CARD9;SDCCAG3;INPP5E	chr11	103821699	103954031	34.4198	27.1164	26.8687	58.8768	40.6972	45.706	34.7138	56.6697	37.9473
XLOC_065474	PMS1	chr2	6322446	6443737	24.4179	23.3413	22.2884	8.1694	8.39709	8.53157	10.444	9.67423	7.43732
XLOC_066813	PMS1	chr2	6322446	6443737	0.0	4.93733	15.4932	2.36762	1.0153	1.35341	0.574445	0.32148	0.859788
XLOC_066814	PMS1	chr2	6322446	6443737	1.19201	1.96125	2.79802	0.374041	0.326899	0.185836	0.760451	0.478495	0.416912
XLOC_066815	PMS1	chr2	6444170	6444370	0.0	30.662	11.4496	12.0519	8.59301	7.30586	8.02845	19.8704	13.8034
XLOC_066816	PMS1	chr2	6444553	6445462	11.5491	9.20997	15.8074	7.24515	5.78959	5.18972	8.10211	5.48061	4.70633
XLOC_066817	PMS1	chr2	6445581	6447758	3.22309	3.9649	3.64355	2.11958	2.13674	2.05023	3.11445	1.9825	2.25657
XLOC_066818	PMS1	chr2	6448733	6449444	5.22696	1.95324	3.06498	0.468268	1.11988	0.270436	0.940314	0.650006	1.13965

XLOC_066819	PMS1	chr2	6450116	6451572	1.11396	2.66609	2.17906	0.349561	0.523878	0.579042	0.812784	0.33554	0.5358
XLOC_041697	PNMA1;DNAL1;PTGR2	chr10	85388363	85659609	0.218911	1.06411	1.03202	3.01048	2.33282	3.34364	3.90277	5.24229	3.43302
XLOC_043254	PNMA1;DNAL1;PTGR2	chr10	85388363	85659609	0.0	0.0	0.0	0.283257	0.575742	0.327573	0.380844	0.0	1.01169
XLOC_043255	PNMA1;DNAL1;PTGR2	chr10	85388363	85659609	0.0	0.0	0.0	1.70286	2.14074	1.62632	1.31896	1.04873	1.98375
XLOC_086491	PNMA2	chr8	75033042	75052369	3.93056	7.58479	10.8837	5.37217	7.03737	7.20017	5.78137	4.38365	4.30561
XLOC_088165	PNMA2	chr8	75033042	75052369	3.97772	0.792739	1.03663	3.20711	3.61498	1.78341	3.57664	1.7144	1.50317
XLOC_088166	PNMA2	chr8	75033042	75052369	9.09682	2.7151	0.0	2.71221	2.56372	3.72621	1.85104	2.65955	1.83991
XLOC_088167	PNMA2	chr8	75033042	75052369	7.57395	5.27699	3.94278	4.74392	2.33764	3.11159	4.44262	2.96995	1.97298
XLOC_088168	PNMA2	chr8	75033042	75052369	2.54239	3.79571	3.97042	2.72983	3.13831	3.65531	2.45984	1.24592	0.220747
XLOC_088169	PNMA2	chr8	75033042	75052369	0.0	1.4856	0.0	2.52274	3.08972	2.05315	3.8505	1.80564	2.02065
XLOC_088170	PNMA2	chr8	75033042	75052369	0.0	1.42372	1.24113	2.27545	2.46872	2.29639	2.27356	1.8892	1.5218
XLOC_088171	PNMA2	chr8	75033042	75052369	0.0	1.4521	7.59478	1.08785	2.06513	1.99883	3.21414	0.477225	1.90067
XLOC_088172	PNMA2	chr8	75033042	75052369	3.77035	3.75731	2.94796	1.68897	1.9591	1.82125	1.8104	1.12597	1.31554
XLOC_088173	PNMA2	chr8	75033042	75052369	3.1969	1.43315	1.24935	0.572636	1.11821	0.990632	1.00109	1.1092	1.53184
XLOC_088174	PNMA2	chr8	75033042	75052369	3.92431	1.64327	1.22795	2.04021	2.39481	2.19995	2.63738	1.4145	1.57736
XLOC_088175	PNMA2	chr8	75033042	75052369	1.59141	1.90247	2.48771	1.71034	1.60816	1.97263	3.84482	1.26219	0.970539
XLOC_088176	PNMA2	chr8	75033042	75052369	2.09738	2.09127	2.18782	1.81751	1.6968	2.0333	1.20831	0.700727	0.733301
XLOC_088177	PNMA2	chr8	75033042	75052369	0.0	4.8545	0.0	2.18328	4.34646	2.07222	3.13962	1.17623	1.40652
XLOC_088178	PNMA2	chr8	75033042	75052369	0.0	1.83009	0.0	1.91949	0.833355	1.74	2.60542	0.759372	1.20056
XLOC_089945	PNRC1	chr9	61917226	61946820	28.5266	29.8504	27.1027	75.2769	77.9694	83.0433	105.196	81.8176	123.223
XLOC_091027	PNRC1	chr9	61917226	61946820	2.98282	3.56083	4.65548	9.33663	11.6948	13.136	18.4814	13.377	18.3576
XLOC_091028	PNRC1	chr9	61917226	61946820	4.1159	4.90948	3.20917	12.5117	11.6132	13.4091	16.9216	10.7006	18.8446
XLOC_052090	POFUT1;POFUT1	chr13	62275288	62275829	0.0	0.0	0.0	10.8075	9.21463	15.5575	10.7433	13.7876	16.5787
XLOC_052091	POFUT1;POFUT1	chr13	62275980	62276347	0.0	0.0	0.0	4.01182	2.6442	4.23086	2.6893	0.334717	2.38985
XLOC_052092	POFUT1;POFUT1	chr13	62276524	62276822	0.0	0.0	0.0	4.58501	1.55355	4.15624	2.59512	2.93203	2.20855
XLOC_052093	POFUT1;POFUT1	chr13	62276898	62277240	0.0	2.33605	0.0	9.4545	6.57968	10.7765	5.38434	3.02334	7.44764
XLOC_052094	POFUT1;POFUT1	chr13	62277683	62277918	0.0	0.0	0.0	3.23756	8.69685	10.8166	7.27397	3.33134	6.16497

XLOC_052086	POFUT1;POFUT1;POFUT1;POFUT1;POFUT1	chr13	62238153	62239116	0.0	0.0	0.0	6.03559	3.57943	4.28484	3.0109	7.36162	6.58774
XLOC_052087	POFUT1;POFUT1;POFUT1;POFUT1;POFUT1	chr13	62239301	62239760	0.0	0.0	1.87357	3.22031	3.14997	4.19196	3.17332	6.83056	7.50267
XLOC_052088	POFUT1;POFUT1;POFUT1;POFUT1;POFUT1	chr13	62240030	62241210	0.0	0.0	0.0	7.10164	4.92828	6.97928	5.98285	9.71164	8.8408
XLOC_052089	POFUT1;POFUT1;POFUT1;POFUT1;POFUT1	chr13	62245740	62246757	0.0	0.251674	0.0	0.301691	0.26322	0.0873266	0.30537	0.336774	0.882157
XLOC_076993	POLDIP3;RRP7A	chr5	113957672	113993734	23.5776	13.4223	11.5645	5.87295	3.82782	5.34345	2.67932	4.76857	4.80805
XLOC_059806	POLE	chr17	45555692	45562405	2.84831	0.710159	0.742957	0.851313	0.446802	0.592531	0.823894	1.5745	0.913608
XLOC_059807	POLE	chr17	45563974	45570391	0.871185	1.95452	1.02927	3.18427	2.35767	1.98432	2.73466	2.96206	3.51529
XLOC_060192	POLE	chr17	45543041	45549332	0.222763	0.599998	0.34873	1.14358	1.61213	0.928305	1.71818	2.40278	1.75548
XLOC_060193	POLE	chr17	45563974	45570391	239.945	245.253	221.741	881.067	693.452	796.991	872.818	1032.12	649.392
XLOC_060194	POLE	chr17	45570671	45573355	777.902	834.149	710.043	2495.19	2035.91	2396.55	2635.55	2926.51	1852.35
XLOC_061469	POLE	chr17	45541836	45542989	0.726516	0.217314	0.0	0.976891	0.909874	0.980847	1.84949	1.89263	1.7144
XLOC_061470	POLE	chr17	45562638	45563490	1.04142	1.24564	2.44335	1.02656	0.569165	0.323823	1.22373	0.935482	1.27279
XLOC_064216	POLR2A;EIF4A1;CHRNB1;AMAC1L3;ZBTB4;TNFSF12;TNFSF13;TNFSF12;SENP3;FXR2;TP53;SHBG;ATP1B2;CD68;MPDU1;SAT2;SOX15;WRAP53	chr19	27762988	28011021	2.27262	3.64665	2.80962	0.732887	1.28334	1.05941	0.899211	0.491	0.88075

XLOC_064835	POLR2A;EIF4A1;CHRNB1;AMAC1L3;ZBTB4;TNFSF12;TNFSF13;TNFSF12;SENP3;FXR2;TP53;SHBG;ATP1B2;CD68;MPDU1;SAT2;SOX15;WRAP53	chr19	27762988	28011021	0.0	0.0	0.0	0.0	0.260679	0.115369	0.0	0.0	0.291565
XLOC_064836	POLR2A;EIF4A1;CHRNB1;AMAC1L3;ZBTB4;TNFSF12;TNFSF13;TNFSF12;SENP3;FXR2;TP53;SHBG;ATP1B2;CD68;MPDU1;SAT2;SOX15;WRAP53	chr19	27762988	28011021	0.0	0.277484	0.0	0.0	0.145017	0.0962379	0.0840436	0.0	0.0810352
XLOC_064837	POLR2A;EIF4A1;CHRNB1;AMAC1L3;ZBTB4;TNFSF12;TNFSF13;TNFSF12;SENP3;FXR2;TP53;SHBG;ATP1B2;CD68;MPDU1;SAT2;SOX15;WRAP53	chr19	27762988	28011021	0.0	1.55139	2.02868	0.0	0.505422	0.402767	0.116716	0.0	0.113155

XLOC_064838	POLR2A;EIF4A1;CHRNB1;AMAC1L3;ZBTB4;TNFSF12;TNFSF13;TNFSF12;SENP3;FXR2;TP53;SHBG;ATP1B2;CD68;MPDU1;SAT2;SOX15;WRAP53	chr19	27762988	28011021	2.04788	1.42937	1.06811	0.122389	0.267254	0.0	0.0621134	0.0	0.119342
XLOC_064839	POLR2A;EIF4A1;CHRNB1;AMAC1L3;ZBTB4;TNFSF12;TNFSF13;TNFSF12;SENP3;FXR2;TP53;SHBG;ATP1B2;CD68;MPDU1;SAT2;SOX15;WRAP53	chr19	27762988	28011021	1.72734	1.68005	2.02809	0.116193	0.186676	0.17996	0.158549	0.0870829	0.113437
XLOC_064840	POLR2A;EIF4A1;CHRNB1;AMAC1L3;ZBTB4;TNFSF12;TNFSF13;TNFSF12;SENP3;FXR2;TP53;SHBG;ATP1B2;CD68;MPDU1;SAT2;SOX15;WRAP53	chr19	27762988	28011021	0.0	1.42999	4.98637	0.0	0.371918	0.658976	0.285411	0.158114	0.416862

XLOC_064841	POLR2A;EIF4A1;CHRNB1;AMAC1L3;ZBTB4;TNFSF12;TNFSF13;TNFSF12;SENP3;FXR2;TP53;SHBG;ATP1B2;CD68;MPDU1;SAT2;SOX15;WRAP53	chr19	27762988	28011021	0.0	0.509871	3.33376	0.381998	0.0	0.0884528	0.0773166	0.0	0.148929
XLOC_064842	POLR2A;EIF4A1;CHRNB1;AMAC1L3;ZBTB4;TNFSF12;TNFSF13;TNFSF12;SENP3;FXR2;TP53;SHBG;ATP1B2;CD68;MPDU1;SAT2;SOX15;WRAP53	chr19	27762988	28011021	1.13277	2.37087	2.6574	0.202999	0.0883763	0.586712	0.0	0.225866	0.296568
XLOC_064843	POLR2A;EIF4A1;CHRNB1;AMAC1L3;ZBTB4;TNFSF12;TNFSF13;TNFSF12;SENP3;FXR2;TP53;SHBG;ATP1B2;CD68;MPDU1;SAT2;SOX15;WRAP53	chr19	27762988	28011021	0.0	0.0	0.0	0.0	0.0	0.871674	0.0	0.0	2.23494

XLOC_064844	POLR2A;EIF4A1;CHRNB1;AMAC1L3;ZBTB4;TNFSF12;TNFSF13;TNFSF12;SENP3;FXR2;TP53;SHBG;ATP1B2;CD68;MPDU1;SAT2;SOX15;WRAP53	chr19	27762988	28011021	0.0	0.869127	0.0	0.0	0.22627	0.150303	0.130419	0.288753	0.126731
XLOC_064845	POLR2A;EIF4A1;CHRNB1;AMAC1L3;ZBTB4;TNFSF12;TNFSF13;TNFSF12;SENP3;FXR2;TP53;SHBG;ATP1B2;CD68;MPDU1;SAT2;SOX15;WRAP53	chr19	27762988	28011021	1.76335	1.10346	1.88226	0.244434	0.327734	0.400963	0.132564	0.14557	0.308866
XLOC_064846	POLR2A;EIF4A1;CHRNB1;AMAC1L3;ZBTB4;TNFSF12;TNFSF13;TNFSF12;SENP3;FXR2;TP53;SHBG;ATP1B2;CD68;MPDU1;SAT2;SOX15;WRAP53	chr19	27762988	28011021	0.0	5.35123	2.79852	0.320792	0.0	0.366248	0.0	0.0	0.0

XLOC_064847	POLR2A;EIF4A1;CHRNB1;AMAC1L3;ZBTB4;TNFSF12;TNFSF13;TNFSF12;SENP3;FXR2;TP53;SHBG;ATP1B2;CD68;MPDU1;SAT2;SOX15;WRAP53	chr19	27762988	28011021	0.0	5.29691	10.3876	0.794319	0.0	0.451522	0.757092	0.0	0.383395
XLOC_064848	POLR2A;EIF4A1;CHRNB1;AMAC1L3;ZBTB4;TNFSF12;TNFSF13;TNFSF12;SENP3;FXR2;TP53;SHBG;ATP1B2;CD68;MPDU1;SAT2;SOX15;WRAP53	chr19	27762988	28011021	35.2225	3.51254	0.0	3.20298	1.73757	3.52894	0.9347	0.0	1.00828
XLOC_064849	POLR2A;EIF4A1;CHRNB1;AMAC1L3;ZBTB4;TNFSF12;TNFSF13;TNFSF12;SENP3;FXR2;TP53;SHBG;ATP1B2;CD68;MPDU1;SAT2;SOX15;WRAP53	chr19	27762988	28011021	1.73247	1.55311	4.0617	0.465413	0.538064	1.43039	0.618495	0.342926	0.754305

XLOC_064850	POLR2A;EIF4A1;CHRNB1;AMAC1L3;ZBTB4;TNFSF12;TNFSF13;TNFSF12;SENP3;FXR2;TP53;SHBG;ATP1B2;CD68;MPDU1;SAT2;SOX15;WRAP53	chr19	27762988	28011021	0.0	0.628597	1.64404	0.251175	0.109681	0.291031	0.0	0.0702072	0.244908
XLOC_064851	POLR2A;EIF4A1;CHRNB1;AMAC1L3;ZBTB4;TNFSF12;TNFSF13;TNFSF12;SENP3;FXR2;TP53;SHBG;ATP1B2;CD68;MPDU1;SAT2;SOX15;WRAP53	chr19	27762988	28011021	2.75534	4.11287	2.15104	0.246496	0.212223	0.847822	0.0	0.0	0.0
XLOC_064852	POLR2A;EIF4A1;CHRNB1;AMAC1L3;ZBTB4;TNFSF12;TNFSF13;TNFSF12;SENP3;FXR2;TP53;SHBG;ATP1B2;CD68;MPDU1;SAT2;SOX15;WRAP53	chr19	27762988	28011021	0.0	1.03048	4.04237	0.154399	0.0	0.355911	0.15391	0.0	0.300295

XLOC_064853	POLR2A;EIF4A1;CHRNB1;AMAC1L3;ZBTB4;TNFSF12;TNFSF13;TNFSF12;SENP3;FXR2;TP53;SHBG;ATP1B2;CD68;MPDU1;SAT2;SOX15;WRAP53	chr19	27762988	28011021	0.0	0.711923	0.93095	0.213346	0.0	0.123287	0.214639	0.0	0.0
XLOC_064854	POLR2A;EIF4A1;CHRNB1;AMAC1L3;ZBTB4;TNFSF12;TNFSF13;TNFSF12;SENP3;FXR2;TP53;SHBG;ATP1B2;CD68;MPDU1;SAT2;SOX15;WRAP53	chr19	27762988	28011021	1.07389	1.92667	2.51945	0.0962304	0.0838229	0.222569	0.19402	0.0	0.187477
XLOC_064855	POLR2A;EIF4A1;CHRNB1;AMAC1L3;ZBTB4;TNFSF12;TNFSF13;TNFSF12;SENP3;FXR2;TP53;SHBG;ATP1B2;CD68;MPDU1;SAT2;SOX15;WRAP53	chr19	27762988	28011021	0.0	0.462413	0.0	0.0	0.240612	0.0	0.0	0.0	0.0

XLOC_064856	POLR2A;EIF4A1;CHRNB1;AMAC1L3;ZBTB4;TNFSF12;TNFSF13;TNFSF12;SENP3;FXR2;TP53;SHBG;ATP1B2;CD68;MPDU1;SAT2;SOX15;WRAP53	chr19	27762988	28011021	0.0	1.38427	2.41348	0.138275	0.240104	0.0	0.0	0.153147	0.403588
XLOC_064857	POLR2A;EIF4A1;CHRNB1;AMAC1L3;ZBTB4;TNFSF12;TNFSF13;TNFSF12;SENP3;FXR2;TP53;SHBG;ATP1B2;CD68;MPDU1;SAT2;SOX15;WRAP53	chr19	27762988	28011021	3.79341	1.13157	0.0	0.0	0.0	0.0	0.0	0.0	0.328057
XLOC_064858	POLR2A;EIF4A1;CHRNB1;AMAC1L3;ZBTB4;TNFSF12;TNFSF13;TNFSF12;SENP3;FXR2;TP53;SHBG;ATP1B2;CD68;MPDU1;SAT2;SOX15;WRAP53	chr19	27762988	28011021	0.0	1.01292	3.53215	0.0	0.264309	0.0	0.101905	0.112585	0.0985504

XLOC_064859	POLR2A;EIF4A1;CHRNB1;AMAC1L3;ZBTB4;TNFSF12;TNFSF13;TNFSF12;SENP3;FXR2;TP53;SHBG;ATP1B2;CD68;MPDU1;SAT2;SOX15;WRAP53	chr19	27762988	28011021	3.25444	0.0	0.0	0.291458	0.126451	0.168046	0.0	0.0	0.141754
XLOC_064860	POLR2A;EIF4A1;CHRNB1;AMAC1L3;ZBTB4;TNFSF12;TNFSF13;TNFSF12;SENP3;FXR2;TP53;SHBG;ATP1B2;CD68;MPDU1;SAT2;SOX15;WRAP53	chr19	27762988	28011021	2.06712	2.00994	1.6175	0.231676	0.202594	0.537387	0.518849	0.103826	0.271203
XLOC_064861	POLR2A;EIF4A1;CHRNB1;AMAC1L3;ZBTB4;TNFSF12;TNFSF13;TNFSF12;SENP3;FXR2;TP53;SHBG;ATP1B2;CD68;MPDU1;SAT2;SOX15;WRAP53	chr19	27762988	28011021	4.4899	2.01448	3.51244	0.335394	0.292816	0.932439	0.0680076	0.449746	0.0653983

XLOC_064862	POLR2A;EIF4A1;CHRNB1;AMAC1L3;ZBTB4;TNFSF12;TNFSF13;TNFSF12;SENP3;FXR2;TP53;SHBG;ATP1B2;CD68;MPDU1;SAT2;SOX15;WRAP53	chr19	27762988	28011021	2.11544	1.63553	2.75984	0.332046	0.277201	0.459264	0.469595	0.248968	0.231573
XLOC_064863	POLR2A;EIF4A1;CHRNB1;AMAC1L3;ZBTB4;TNFSF12;TNFSF13;TNFSF12;SENP3;FXR2;TP53;SHBG;ATP1B2;CD68;MPDU1;SAT2;SOX15;WRAP53	chr19	27762988	28011021	0.857445	0.769335	0.670701	0.230556	0.402268	0.444872	0.0777677	0.0857737	0.149809
XLOC_064864	POLR2A;EIF4A1;CHRNB1;AMAC1L3;ZBTB4;TNFSF12;TNFSF13;TNFSF12;SENP3;FXR2;TP53;SHBG;ATP1B2;CD68;MPDU1;SAT2;SOX15;WRAP53	chr19	27762988	28011021	1.12222	2.01328	1.75513	0.201111	0.612927	0.697545	0.708996	0.111896	0.293818

XLOC_064865	POLR2A;EIF4A1;CHRNB1;AMAC1L3;ZBTB4;TNFSF12;TNFSF13;TNFSF12;SENP3;FXR2;TP53;SHBG;ATP1B2;CD68;MPDU1;SAT2;SOX15;WRAP53	chr19	27762988	28011021	0.0	0.858783	1.12297	0.643381	0.111802	0.445584	0.773433	0.428058	0.125228
XLOC_064866	POLR2A;EIF4A1;CHRNB1;AMAC1L3;ZBTB4;TNFSF12;TNFSF13;TNFSF12;SENP3;FXR2;TP53;SHBG;ATP1B2;CD68;MPDU1;SAT2;SOX15;WRAP53	chr19	27762988	28011021	0.674983	2.22101	4.22463	0.181529	0.475698	0.771325	0.491412	0.203019	0.413025
XLOC_064867	POLR2A;EIF4A1;CHRNB1;AMAC1L3;ZBTB4;TNFSF12;TNFSF13;TNFSF12;SENP3;FXR2;TP53;SHBG;ATP1B2;CD68;MPDU1;SAT2;SOX15;WRAP53	chr19	27762988	28011021	1.9041	0.569605	3.4761	0.170703	0.298303	0.395714	0.520236	0.254664	0.277453

XLOC_064868	POLR2A;EIF4A1;CHRNB1;AMAC1L3;ZBTB4;TNFSF12;TNFSF13;TNFSF12;SENP3;FXR2;TP53;SHBG;ATP1B2;CD68;MPDU1;SAT2;SOX15;WRAP53	chr19	27762988	28011021	1.60199	1.43632	2.50421	0.0	0.373545	0.496399	0.716596	0.158801	0.139569
XLOC_064869	POLR2A;EIF4A1;CHRNB1;AMAC1L3;ZBTB4;TNFSF12;TNFSF13;TNFSF12;SENP3;FXR2;TP53;SHBG;ATP1B2;CD68;MPDU1;SAT2;SOX15;WRAP53	chr19	27762988	28011021	0.0	3.58099	9.36358	0.0	0.91643	0.815479	2.06073	1.54352	0.345904
XLOC_064870	POLR2A;EIF4A1;CHRNB1;AMAC1L3;ZBTB4;TNFSF12;TNFSF13;TNFSF12;SENP3;FXR2;TP53;SHBG;ATP1B2;CD68;MPDU1;SAT2;SOX15;WRAP53	chr19	27762988	28011021	0.763099	1.25584	2.98601	0.239505	0.509125	0.357388	0.628436	0.460709	0.367278

XLOC_064871	POLR2A;EIF4A1;CHRNB1;AMAC1L3;ZBTB4;TNFSF12;TNFSF13;TNFSF12;SENP3;FXR2;TP53;SHBG;ATP1B2;CD68;MPDU1;SAT2;SOX15;WRAP53	chr19	27762988	28011021	0.0	0.0	2.07236	0.0	0.0	0.817214	0.0	0.0	0.0
XLOC_064872	POLR2A;EIF4A1;CHRNB1;AMAC1L3;ZBTB4;TNFSF12;TNFSF13;TNFSF12;SENP3;FXR2;TP53;SHBG;ATP1B2;CD68;MPDU1;SAT2;SOX15;WRAP53	chr19	27762988	28011021	1.4196	0.848683	1.10977	0.0	0.1105	0.0	0.891988	0.282069	0.0
XLOC_064873	POLR2A;EIF4A1;CHRNB1;AMAC1L3;ZBTB4;TNFSF12;TNFSF13;TNFSF12;SENP3;FXR2;TP53;SHBG;ATP1B2;CD68;MPDU1;SAT2;SOX15;WRAP53	chr19	27762988	28011021	3.99073	4.17276	3.11784	0.357261	0.618415	0.61679	0.531715	0.196853	0.520766

XLOC_064874	POLR2A;EIF4A1;CHRNB1;AMAC1L3;ZBTB4;TNFSF12;TNFSF13;TNFSF12;SENP3;FXR2;TP53;SHBG;ATP1B2;CD68;MPDU1;SAT2;SOX15;WRAP53	chr19	27762988	28011021	0.0	2.4019	2.09395	0.319914	0.488285	0.833194	0.970583	0.178456	0.311775
XLOC_064875	POLR2A;EIF4A1;CHRNB1;AMAC1L3;ZBTB4;TNFSF12;TNFSF13;TNFSF12;SENP3;FXR2;TP53;SHBG;ATP1B2;CD68;MPDU1;SAT2;SOX15;WRAP53	chr19	27762988	28011021	0.0	1.19882	3.13511	0.359239	0.310896	1.03362	0.534558	0.593757	0.698177
XLOC_064876	POLR2A;EIF4A1;CHRNB1;AMAC1L3;ZBTB4;TNFSF12;TNFSF13;TNFSF12;SENP3;FXR2;TP53;SHBG;ATP1B2;CD68;MPDU1;SAT2;SOX15;WRAP53	chr19	27762988	28011021	0.0	0.862204	0.0	0.516755	0.0	0.0	0.129406	0.429736	0.377168

XLOC_064877	POLR2A;EIF4A1;CHRNB1;AMAC1L3;ZBTB4;TNFSF12;TNFSF13;TNFSF12;SENP3;FXR2;TP53;SHBG;ATP1B2;CD68;MPDU1;SAT2;SOX15;WRAP53	chr19	27762988	28011021	1.42884	1.14	4.09971	0.29894	0.3362	0.148619	0.0870088	0.0957369	0.29165
XLOC_064878	POLR2A;EIF4A1;CHRNB1;AMAC1L3;ZBTB4;TNFSF12;TNFSF13;TNFSF12;SENP3;FXR2;TP53;SHBG;ATP1B2;CD68;MPDU1;SAT2;SOX15;WRAP53	chr19	27762988	28011021	3.28564	0.982707	2.57016	0.220876	0.44972	0.0852544	0.223648	0.328822	0.430592
XLOC_064879	POLR2A;EIF4A1;CHRNB1;AMAC1L3;ZBTB4;TNFSF12;TNFSF13;TNFSF12;SENP3;FXR2;TP53;SHBG;ATP1B2;CD68;MPDU1;SAT2;SOX15;WRAP53	chr19	27762988	28011021	0.0	4.71395	0.0	0.0	0.0	2.13649	0.888044	1.00401	0.0
XLOC_065465	POLR2D;AMMECR1L;WDR33	chr2	4389615	4614237	11.7557	6.43675	8.4113	5.29555	5.96228	5.32323	10.2068	9.83436	7.65502
XLOC_066775	POLR2D;AMMECR1L;WDR33	chr2	4389615	4614237	2.79135	0.501048	0.873641	0.0	0.0	0.0	0.0	0.0560887	0.0
XLOC_066776	POLR2D;AMMECR1L;WDR33	chr2	4389615	4614237	2.42101	0.0	0.946513	0.0	0.0	0.0	0.0	0.0	0.0

XLOC_066777	POLR2D;AMMECR1L;WDR33	chr2	4389615	4614237	0.0	0.37508	0.980949	0.112405	0.0	0.0	0.0	0.0	0.0
XLOC_066779	POLR2D;AMMECR1L;WDR33	chr2	4389615	4614237	4.33551	3.87851	3.38048	0.387718	0.330189	0.882012	0.740287	0.0	0.374375
XLOC_066780	POLR2D;AMMECR1L;WDR33	chr2	4389615	4614237	1.20446	1.44042	3.76716	1.4029	1.2209	1.49656	1.95377	1.67973	1.261
XLOC_066781	POLR2D;AMMECR1L;WDR33	chr2	4389615	4614237	4.18618	1.24841	3.26433	0.374348	0.638258	2.55667	1.79108	1.20882	1.08486
XLOC_078670	POLR2F	chr5	110255371	110255791	13.7767	8.22572	23.6614	25.8821	19.3123	21.1956	24.6272	24.5135	30.8389
XLOC_078671	POLR2F	chr5	110256002	110257083	3.91581	7.73009	10.415	8.00277	8.82332	9.59386	8.32201	7.68462	10.8124
XLOC_083106	POLR3G;LYSMD3	chr7	92431015	92445276	0.0	0.672883	0.0	0.0	0.0	0.0	0.0	0.0	0.0655315
XLOC_082980	POLRMT	chr7	44838825	44855902	19.9708	19.2066	18.3267	16.3329	12.7297	15.2783	10.2367	16.6783	10.3452
XLOC_083610	POLRMT	chr7	44838825	44855902	3.87275	4.42489	4.51204	6.42207	4.65167	5.95799	4.96791	6.00053	4.82954
XLOC_070444	POMGNT1	chr3	100469282	100489222	10.6189	5.20988	8.85723	0.718236	0.656967	0.707495	0.783555	0.825383	0.884212
XLOC_071879	POMGNT1	chr3	100469282	100489222	0.0	0.323995	0.0	0.970945	0.16914	0.561391	0.293594	0.648549	0.0945767
XLOC_048117	POMP	chr12	31484786	31500441	12.725	15.2298	13.66	8.77076	8.17488	8.75053	8.73483	10.5485	8.17923
XLOC_047302	POMT1	chr11	101654304	101654582	5.94916	19.508	18.5468	0.0	0.0	0.0	0.0	0.0	0.51213
XLOC_047303	POMT1	chr11	101658422	101658651	28.8951	20.1353	22.5576	14.7985	12.1841	8.70148	10.1014	4.45494	8.27152
XLOC_047304	POMT1	chr11	101663122	101663815	2.70134	6.05643	3.16787	0.120997	0.0	0.0	0.121393	0.0	0.0
XLOC_073763	PON1	chr4	12408815	12409868	0.0	0.0	0.0	1.0135	0.758118	1.509	0.659873	0.565895	0.635097
XLOC_073764	PON1	chr4	12410615	12411574	0.0	0.0	0.0	0.889634	0.352663	1.12333	0.572425	0.902189	0.394078
XLOC_073765	PON1	chr4	12419238	12420175	0.0	0.0	0.0	0.332625	0.50756	1.05862	0.336174	0.556418	0.648262
XLOC_073766	PON3	chr4	12454240	12455417	0.0	0.0	0.0	0.763128	0.499824	0.51577	0.129037	0.284379	0.0620052
XLOC_089375	POPDC3	chr9	45430197	45672918	0.0	0.267397	0.0	0.961639	0.946507	1.06921	0.122516	0.269562	0.39094
XLOC_048941	POSTN	chr12	24244978	24245288	0.0	0.0	0.0	0.845812	0.0	0.480118	0.0	0.45256	0.0
XLOC_048942	POSTN	chr12	24246255	24246812	0.0	0.540989	0.0	2.59385	2.38833	2.24119	1.93634	2.32726	2.83704
XLOC_048943	POSTN	chr12	24247104	24248938	0.0	0.129075	0.0	0.309461	0.236931	0.314183	0.236623	0.130143	0.264205
XLOC_048944	POSTN	chr12	24249168	24250869	0.0	0.0	0.0	0.0420143	0.330775	0.0	0.0856118	0.18839	0.0819809
XLOC_048945	POSTN	chr12	24250936	24252468	0.0	0.0	0.0	0.0943551	0.0412527	0.0547129	0.0	0.0528511	0.0
XLOC_055725	POU2F3	chr15	31291238	31370613	1.70244	0.513132	0.679725	0.499515	0.371464	0.492834	0.472844	1.43762	0.303444
XLOC_056274	POU2F3	chr15	31291238	31370613	0.0	0.0	0.0	0.425712	0.0	0.327343	0.0	0.157095	0.0

XLOC_056275	POU2F3	chr15	31430344	31430512	0.0	0.0	0.0	8.30905	0.0	11.6761	11.0568	35.9603	2.52037
XLOC_070491	POU3F1;UTP11L	chr3	108457893	108536460	0.212555	0.826944	0.499126	0.019064	0.0167066	0.022145	0.0390222	0.0214325	0.0372237
XLOC_073219	POU6F2	chr4	82282059	82505862	17.6379	15.564	18.3824	4.94363	5.9049	5.97855	8.03221	6.25822	5.99648
XLOC_074451	POU6F2	chr4	82282059	82505862	0.0	0.17017	0.0	0.509971	0.490373	0.473036	0.466797	0.285513	0.845602
XLOC_074452	POU6F2	chr4	82282059	82505862	8.05709	5.07524	2.98671	1.33088	1.09803	1.36779	1.78351	1.83383	2.26327
XLOC_074453	POU6F2	chr4	82282059	82505862	12.6936	7.34436	6.78948	1.55593	1.84577	2.27027	3.04897	2.75188	2.55642
XLOC_074454	POU6F2	chr4	82282059	82505862	8.4549	6.53343	2.75612	1.20005	1.98574	1.31726	2.30703	1.76535	2.09384
XLOC_074455	POU6F2	chr4	82282059	82505862	0.0	0.855478	0.0	0.427282	0.745009	0.88998	1.4676	0.952741	1.49883
XLOC_074456	POU6F2	chr4	82282059	82505862	0.897031	0.268394	0.702002	0.643477	0.738902	0.979855	1.59859	0.54112	1.37337
XLOC_074457	POU6F2	chr4	82282059	82505862	0.58776	1.05501	2.75934	1.00121	1.24356	1.52731	1.66085	1.4158	1.23349
XLOC_074458	POU6F2	chr4	82282059	82505862	0.0	0.768814	0.0	0.691178	0.701366	0.931502	1.04133	1.40749	0.78505
XLOC_074459	POU6F2	chr4	82282059	82505862	0.0	2.05606	0.0	0.462098	1.06856	1.59783	2.14977	0.510796	1.34811
XLOC_074460	POU6F2	chr4	82282059	82505862	0.0	0.918937	1.20174	0.413064	0.956386	0.794237	0.413081	0.457523	2.00939
XLOC_074461	POU6F2	chr4	82282059	82505862	6.24849	1.8628	0.0	0.560036	0.941708	2.52482	5.71956	1.77094	1.07538
XLOC_074462	POU6F2	chr4	82282059	82505862	0.0	1.14827	1.50166	1.14708	1.15255	0.797704	1.33995	1.09087	0.838966
XLOC_074463	POU6F2	chr4	82282059	82505862	0.0	0.619264	4.85881	0.278369	0.889324	2.25387	0.842436	1.03355	0.903948
XLOC_074464	POU6F2	chr4	82282059	82505862	1.95383	0.974119	1.52868	1.10933	1.53026	1.15035	2.4904	1.56757	1.53727
XLOC_074465	POU6F2	chr4	82282059	82505862	0.0	0.0	0.0	2.54656	0.67726	0.900951	2.5184	1.29247	1.14158
XLOC_074466	POU6F2	chr4	82282059	82505862	0.0	0.832065	4.35184	0.748013	3.00471	1.14328	1.46457	1.36195	1.45078
XLOC_074467	POU6F2	chr4	82282059	82505862	0.0	7.02508	9.18231	2.13532	5.21273	3.52894	10.2817	2.15172	4.03301
XLOC_074468	POU6F2	chr4	82282059	82505862	2.20191	0.0	3.44002	1.57664	1.8736	1.35954	0.584567	2.16689	1.72271
XLOC_074469	POU6F2	chr4	82282059	82505862	3.48403	1.03952	5.43656	1.24625	2.66896	1.42358	1.20606	2.02684	3.31727
XLOC_074470	POU6F2	chr4	82282059	82505862	6.37293	0.634559	3.31903	0.570445	1.64426	3.06192	3.76503	1.88323	2.58605
XLOC_074471	POU6F2	chr4	82282059	82505862	0.0	1.96114	3.41924	1.37123	2.53972	2.70285	2.13095	2.36954	1.14158
XLOC_074472	POU6F2	chr4	82282059	82505862	8.4832	17.72	13.2358	4.58561	6.33552	9.38158	8.28574	3.948	2.91497
XLOC_074473	POU6F2	chr4	82282059	82505862	0.0	3.35398	2.19287	1.75888	0.648757	1.728	1.96717	0.823305	1.70559
XLOC_069998	PPAP2B	chr3	90151215	90185446	5.98864	5.72323	6.68317	1.14594	0.436314	0.493495	0.295556	0.303714	0.466315
XLOC_069999	PPAP2B	chr3	90207161	90210527	2.43322	1.81868	0.951283	3.4881	2.2765	2.77117	3.50764	3.27154	3.39647
XLOC_070000	PPAP2B	chr3	90211632	90225679	15.8795	7.0585	10.1204	5.82906	6.62745	6.23814	5.32912	7.68328	9.12954
XLOC_070001	PPAP2B	chr3	90231314	90235694	14.1817	20.4198	20.387	22.386	24.4706	25.5042	33.5473	31.5271	31.416
XLOC_070002	PPAP2B	chr3	90248204	90252892	29.3573	23.819	28.4233	14.3213	14.3202	13.972	14.9471	16.9987	17.7578
XLOC_070003	PPAP2B	chr3	90254424	90264072	21.6764	30.5376	20.9324	6.67912	6.84786	7.3631	7.74676	8.98832	6.30567

XLOC_070004	PPAP2B	chr3	90265620	90275557	1.7907	0.878288	0.823619	2.91043	3.18894	2.44331	2.93508	3.0726	2.43176
XLOC_070388	PPAP2B	chr3	90211632	90225679	0.326063	3.31062	5.31577	3.20524	6.47603	4.15701	7.03194	7.24297	4.57716
XLOC_070389	PPAP2B	chr3	90237347	90243500	0.0	0.59546	0.389346	0.936892	1.60274	1.2937	0.772328	1.34989	0.870466
XLOC_071649	PPAP2B	chr3	90207161	90210527	0.0	0.0	0.0	0.749367	1.52462	1.15633	2.18789	1.48567	1.94728
XLOC_071650	PPAP2B	chr3	90207161	90210527	5.23351	4.68047	0.0	3.27837	2.775	2.1216	3.52872	6.48187	6.31457
XLOC_071651	PPAP2B	chr3	90210631	90210806	0.0	14.6923	0.0	15.9894	14.1064	14.5825	11.1449	15.0917	16.7656
XLOC_071652	PPAP2B	chr3	90210929	90211330	2.9701	1.77305	0.0	5.57905	7.0801	4.86812	6.74151	5.50341	5.15016
XLOC_071653	PPAP2B	chr3	90225802	90226489	5.4639	0.816663	0.0	0.0	0.425487	0.282592	0.368202	0.135796	0.595528
XLOC_071654	PPAP2B	chr3	90253233	90253630	66.4319	75.7066	80.1302	35.9221	30.4067	33.3984	36.3475	32.669	42.405
XLOC_071655	PPAP2B	chr3	90254424	90264072	12.336	6.44283	4.81361	1.93081	2.84327	1.57856	1.61218	1.20144	1.87092
XLOC_082976	PPAP2C	chr7	44591687	44598831	0.0	0.543833	0.0	1.92332	1.62629	1.52831	1.34157	1.4191	0.889009
XLOC_084797	PPAP2C	chr7	44591188	44591605	0.0	0.0	0.0	0.748013	2.14622	1.14328	0.976382	0.54478	0.72539
XLOC_050861	PPDPF;SRMS;PTK6	chr13	54592983	54611291	52.4573	52.6772	47.9561	18.6752	15.3524	18.0392	14.2211	20.7645	19.8517
XLOC_076833	PPHLN1	chr5	38526094	38561603	138.285	134.528	114.848	50.1234	33.1194	44.578	56.7438	80.629	48.8709
XLOC_077762	PPHLN1	chr5	38522895	38523235	0.0	1.1807	6.17465	1.06193	0.906728	1.21017	5.7792	1.14557	1.71086
XLOC_077763	PPHLN1	chr5	38523944	38524321	3.29416	5.89795	2.57041	2.06222	1.2635	1.68422	2.8599	3.20074	2.85304
XLOC_077764	PPHLN1	chr5	38525197	38525710	0.0	3.64694	1.58956	0.910704	1.10319	0.209595	2.1672	1.40444	1.06188
XLOC_077765	PPHLN1	chr5	38561770	38562724	0.908045	0.0	0.0	2.52287	2.83889	3.20264	2.46839	2.45103	4.91715
XLOC_077766	PPHLN1	chr5	38563141	38564291	0.0	0.217971	0.570083	1.30646	2.3956	1.66489	1.1925	0.876138	1.84694
XLOC_042688	PPIB	chr10	45875285	45876068	98.2863	101.298	94.0358	511.299	489.946	516.542	401.229	411.779	593.197
XLOC_044000	PPM1B;SLC3A1	chr11	26386571	26593937	5.7381	4.70859	3.91983	22.7111	18.3853	22.1053	21.107	20.4853	20.8614
XLOC_044696	PPM1B;SLC3A1	chr11	26386571	26593937	3.98115	1.89877	1.81119	3.48992	4.6659	3.31936	2.926	2.12975	4.07841
XLOC_044897	PPM1G	chr11	72259112	72266536	1.88609	2.25408	4.42114	0.0	0.0	0.194468	0.0	0.372638	0.32829
XLOC_038012	PPM1L	chr1	107498717	107525472	23.7954	14.465	16.3585	0.0	0.0	0.213138	0.0	0.0	0.0355821
XLOC_038499	PPM1L	chr1	107498717	107525472	0.0	4.79351	0.820349	0.0	0.0	0.0	0.0948023	0.0	0.0
XLOC_060264	PPP1CC;HVCN1	chr17	56775811	56831026	1.38608	2.21237	2.16989	14.1322	11.6896	13.3588	12.7614	17.2339	12.1707
XLOC_061666	PPP1CC;HVCN1	chr17	56775811	56831026	0.0	0.0	7.28304	1.3114	2.69518	1.9276	2.51264	3.30886	2.55315
XLOC_076203	PPP1R12A	chr5	9427700	9440981	0.983361	3.5288	3.07636	0.176253	0.307249	0.407841	0.266941	0.0982177	0.25759
XLOC_065487	PPP1R1C;SSFA2;NEUROD1	chr2	14604111	14986586	6.33314	2.0451	3.28314	0.194808	0.0641064	0.282532	0.0249344	0.106481	0.0714194

XLOC_066903	PPP1R1C;SSFA2;<br>NEUROD1	chr2	14604111	14986586	1.36598	0.816663	1.0679	0.122366	0.0	0.141296	0.0	0.0	0.0
XLOC_066904	PPP1R1C;SSFA2;<br>NEUROD1	chr2	14604111	14986586	3.55224	2.1196	8.31364	0.317655	0.0	0.362731	0.0	0.0	0.30741
XLOC_066905	PPP1R1C;SSFA2;<br>NEUROD1	chr2	14604111	14986586	0.0	0.896248	1.17203	0.0	0.0586235	0.0	0.0	0.0	0.0
XLOC_066906	PPP1R1C;SSFA2;<br>NEUROD1	chr2	14604111	14986586	0.0	0.0	1.06992	0.0	0.0	0.0	0.0	0.0	0.0
XLOC_066907	PPP1R1C;SSFA2;<br>NEUROD1	chr2	14604111	14986586	0.0	0.361307	1.88987	0.0	0.0	0.0	0.0	0.0	0.0
XLOC_066908	PPP1R1C;SSFA2;<br>NEUROD1	chr2	14604111	14986586	1.66852	1.49587	1.30401	0.0	0.0	0.0	0.0	0.0	0.0
XLOC_066909	PPP1R1C;SSFA2;<br>NEUROD1	chr2	14604111	14986586	1.60633	0.480447	0.62828	1.00788	1.31939	0.750332	1.16669	1.12557	1.40351
XLOC_066910	PPP1R1C;SSFA2;<br>NEUROD1	chr2	14604111	14986586	0.0	0.666956	1.74431	0.0	0.0870257	0.115545	0.0	0.0	0.0
XLOC_066911	PPP1R1C;SSFA2;<br>NEUROD1	chr2	14604111	14986586	1.41682	0.0	3.32279	0.126914	0.0	0.0	0.0	0.0	0.0
XLOC_066912	PPP1R1C;SSFA2;<br>NEUROD1	chr2	14604111	14986586	0.0	0.0	4.70266	0.0	0.0	0.0	0.0	0.0	0.0
XLOC_066913	PPP1R1C;SSFA2;<br>NEUROD1	chr2	14604111	14986586	2.51275	0.751528	0.0	0.0	0.0	0.0	0.0	0.251422	0.0
XLOC_066914	PPP1R1C;SSFA2;<br>NEUROD1	chr2	14604111	14986586	2.22652	1.10997	2.32242	0.0	0.0	0.0770683	0.0	0.0	0.0
XLOC_066915	PPP1R1C;SSFA2;<br>NEUROD1	chr2	14604111	14986586	3.6437	0.363125	0.0	0.0	0.0	0.0	0.0	0.0	0.0
XLOC_066916	PPP1R1C;SSFA2;<br>NEUROD1	chr2	14604111	14986586	1.60633	1.20112	0.62828	0.0	0.0	0.0833702	0.291672	0.0	0.0
XLOC_066917	PPP1R1C;SSFA2;<br>NEUROD1	chr2	14604111	14986586	0.0	0.0	0.952884	0.0	0.0950123	0.126173	0.0	0.0	0.0

XLOC_066918	PPP1R1C;SSFA2;NEUROD1	chr2	14604111	14986586	2.32955	0.695723	1.8194	0.0	0.0	0.23954	0.0	0.0	0.0
XLOC_066919	PPP1R1C;SSFA2;NEUROD1	chr2	14604111	14986586	1.71489	0.256444	1.3414	0.0	0.0	0.0	0.0	0.0	0.0
XLOC_066920	PPP1R1C;SSFA2;NEUROD1	chr2	14604111	14986586	0.0	0.855387	3.35561	0.0	0.0	0.147945	0.0	0.0	0.0
XLOC_066921	PPP1R1C;SSFA2;NEUROD1	chr2	14604111	14986586	5.97145	0.446206	0.0	0.0	0.0	0.0	0.0	0.0	0.0
XLOC_037903	PPP1R2	chr1	72711253	72714368	5.20216	7.68171	5.3363	21.8505	16.586	21.8716	23.0039	26.7739	21.6201
XLOC_039653	PPP1R2	chr1	72714509	72715074	5.32496	0.530385	1.38707	3.97346	3.99535	2.56395	3.48251	2.98487	5.40886
XLOC_039654	PPP1R2	chr1	72715359	72715500	78.4118	0.0	64.2448	47.8352	39.1344	71.8057	46.4703	67.3931	76.2195
XLOC_039655	PPP1R2	chr1	72716209	72716644	2.60649	0.778239	0.0	2.09887	3.81878	2.94332	3.43499	0.765818	2.94141
XLOC_039656	PPP1R2	chr1	72716760	72717960	0.0	1.66018	0.0	0.932878	1.2493	0.504439	1.07293	0.834474	1.6373
XLOC_039657	PPP1R2	chr1	72718625	72719397	0.0	1.05573	0.920358	0.949134	0.917957	0.731354	0.31837	0.351843	0.718897
XLOC_044705	PPP1R21	chr11	30506155	30633703	0.0	2.54418	2.20118	0.762126	0.437129	1.45591	1.73976	1.38715	2.7066
XLOC_045710	PPP1R21	chr11	30506155	30633703	2.36557	5.30451	8.32381	7.41833	9.68539	8.20656	7.99744	6.83518	9.70092
XLOC_045711	PPP1R21	chr11	30649162	30649777	0.0	0.472502	0.0	0.991161	1.22902	1.14322	0.424481	0.783792	0.413233
XLOC_084934	PPP1R39	chr7	59227886	59229149	5.888	7.43674	8.70142	8.68019	7.58326	8.36063	6.4325	6.03596	8.1214
XLOC_083034	PPP1R39;PLAC8L1	chr7	59229247	59308100	24.0812	21.5182	18.1484	28.1185	27.9888	29.106	30.6217	24.6911	36.6647
XLOC_084935	PPP1R39;PLAC8L1	chr7	59229247	59308100	2.08054	4.66832	4.06992	2.47173	2.8546	3.13695	4.31981	1.93313	2.91151
XLOC_084936	PPP1R39;PLAC8L1	chr7	59229247	59308100	5.64367	2.81029	1.46989	2.02142	2.62601	1.93969	2.67908	0.929228	3.76559
XLOC_084937	PPP1R39;PLAC8L1	chr7	59229247	59308100	2.80899	2.51548	2.19265	1.00549	3.02752	4.60802	1.22948	3.01878	1.70559
XLOC_084938	PPP1R39;PLAC8L1	chr7	59229247	59308100	9.04586	3.19742	9.00577	2.43252	3.79478	4.09684	3.80627	1.97515	2.9457
XLOC_084939	PPP1R39;PLAC8L1	chr7	59229247	59308100	1.90109	0.567956	2.97062	0.681067	2.0636	1.95984	1.35314	2.25305	2.1506
XLOC_053540	PPP1R42	chr14	33154019	33185499	6.64341	1.48901	3.8941	0.446208	0.387088	0.685936	0.296851	2.46777	0.433985
XLOC_072542	PPP1R9A	chr4	12039276	12097601	5.28817	6.87584	5.17492	2.77238	3.41477	3.48966	3.83214	3.66225	4.70307
XLOC_073083	PPP1R9A	chr4	12039276	12097601	2.13433	2.15809	2.62555	1.27017	1.91163	1.20031	1.63234	1.54892	1.88609
XLOC_073084	PPP1R9A	chr4	12099213	12172112	29.8183	20.2946	16.026	34.3117	49.8062	38.0265	62.1607	38.0913	61.2525
XLOC_073749	PPP1R9A	chr4	12098034	12098607	8.70404	2.60095	5.44163	1.40295	1.75701	1.61685	2.48551	0.172271	2.12214

XLOC_073750	PPP1R9A	chr4	12099213	12172112	0.0	1.03974	0.0	0.623123	1.06763	1.77947	1.80909	0.337807	0.90471
XLOC_073751	PPP1R9A	chr4	12099213	12172112	1.2974	3.87856	2.02868	1.16228	1.11194	1.34256	1.40059	0.258166	1.24466
XLOC_073752	PPP1R9A	chr4	12099213	12172112	2.19529	0.0	1.71472	0.0	0.339666	0.0	0.0	0.0	0.190836
XLOC_073753	PPP1R9A	chr4	12099213	12172112	0.0	0.907479	1.18651	3.67083	2.00705	2.19612	1.7681	3.61494	1.71966
XLOC_073754	PPP1R9A	chr4	12099213	12172112	0.0	0.988253	0.0	3.55337	0.770704	1.02425	1.62544	3.27562	1.15203
XLOC_073755	PPP1R9A	chr4	12099213	12172112	31.3882	19.2509	21.286	0.0469047	0.0	0.0	0.0	0.0525472	0.0
XLOC_073756	PPP1R9A	chr4	12099213	12172112	39.669	27.6358	33.0369	0.0	0.0	0.0	0.0	0.258888	0.0
XLOC_073757	PPP1R9A	chr4	12099213	12172112	4.39711	2.76708	3.08912	1.24393	1.05541	1.18714	1.07835	1.28623	1.15549
XLOC_073758	PPP1R9A	chr4	12099213	12172112	0.0	1.39628	0.0	0.139464	0.121093	0.0	0.418265	0.154441	0.27136
XLOC_073759	PPP1R9A	chr4	12099213	12172112	1.86186	1.66897	0.0	0.0	0.72207	1.15196	0.165777	0.183975	0.324093
XLOC_073760	PPP1R9A	chr4	12099213	12172112	0.0	3.09092	2.02061	0.69463	1.19751	1.59414	0.68231	0.760508	1.34795
XLOC_073761	PPP1R9A	chr4	12099213	12172112	3.04869	1.82249	0.0	0.955703	0.948438	1.57521	1.22909	1.05872	0.929854
XLOC_073762	PPP1R9A	chr4	12236863	12237100	17.5409	0.0	0.0	25.3043	45.152	30.848	24.2142	8.96518	15.8202
XLOC_084882	PPP2CA	chr7	47440732	47441488	6.0424	9.75529	18.8987	6.06342	6.40738	6.50659	6.96944	3.6111	4.4282
XLOC_084883	PPP2CA	chr7	47441659	47442664	11.9476	15.8246	21.3612	7.19003	8.94187	10.4497	8.20508	4.4394	7.82783
XLOC_083670	PPP2R2B	chr7	59969864	59980791	7.62437	11.7256	12.2712	47.2787	47.4141	54.1116	62.4111	44.1121	47.6296
XLOC_083671	PPP2R2B	chr7	59983120	60005208	7.54886	12.6998	5.95985	84.3306	80.8873	87.9568	118.076	89.3456	60.6808
XLOC_084947	PPP2R2B	chr7	59966422	59967106	0.0	0.410655	1.07398	0.123063	0.213942	1.84722	0.617078	0.136557	0.479123
XLOC_084948	PPP2R2B	chr7	59967185	59967938	0.0	0.726251	0.0	0.761741	1.23105	1.00602	1.09434	0.120968	0.847697
XLOC_084949	PPP2R2B	chr7	59968164	59969770	0.0	0.298765	0.0	0.895371	1.37038	1.50587	0.638222	0.351173	1.04818
XLOC_084950	PPP2R2B	chr7	59969864	59980791	0.833934	2.49521	2.28415	9.68394	12.7278	13.6233	14.6796	8.05206	11.5652
XLOC_084951	PPP2R2B	chr7	59969864	59980791	5.1773	4.42673	5.40312	19.8779	23.6731	24.088	26.3478	15.5553	21.11
XLOC_084952	PPP2R2B	chr7	59980857	59981932	0.0	0.471552	0.616648	2.19041	3.57693	4.09165	4.00886	2.68336	2.27301
XLOC_084953	PPP2R2B	chr7	59982071	59982384	0.0	1.38291	0.0	7.05232	8.4616	6.59638	8.67248	3.55475	6.8038
XLOC_041602	PPP2R5E	chr10	75993819	76009559	0.0	0.0	0.0	1.76603	1.61657	2.44462	0.646258	2.09897	2.86167
XLOC_041603	PPP2R5E	chr10	76025898	76041547	13.9488	15.2343	11.6229	25.3431	22.968	22.8345	26.0387	33.4302	22.1112
XLOC_041999	PPP2R5E	chr10	75948083	75954671	0.517501	0.619308	0.0	1.15934	1.82529	1.82965	2.4562	1.72221	1.63065
XLOC_042000	PPP2R5E	chr10	76058526	76110090	0.0	0.116816	0.0	2.27558	0.551548	1.13762	0.857224	1.76757	0.922394
XLOC_042984	PPP2R5E	chr10	75992554	75992906	0.0	0.0	0.0	1.32909	2.84184	0.0	0.320351	0.71874	0.32141
XLOC_042985	PPP2R5E	chr10	76041653	76042994	1.22221	9.87189	10.9971	14.7377	11.6822	11.8763	11.804	20.1732	10.9535
XLOC_080588	PPP3CA	chr6	24951525	24951929	0.0	0.0	0.0	0.0	0.0	0.0	0.0	0.286252	0.0
XLOC_080589	PPP3CA	chr6	24952116	24955262	0.0	0.0724213	0.0	0.217043	0.228199	0.176457	0.133211	0.195142	0.0847516
XLOC_080590	PPP3CA	chr6	24955339	24956177	0.0	0.317812	0.0	0.380966	0.082968	0.330442	0.0960316	0.0	0.0927768

XLOC_086442	PPP3CC	chr8	70283078	70313143	4.77745	2.81248	3.73742	8.46385	8.9602	9.00012	12.0769	11.1445	9.19065
XLOC_086443	PPP3CC	chr8	70283078	70313143	28.3798	35.1922	26.5255	58.4219	63.7204	68.8964	57.3115	49.3177	48.7987
XLOC_086444	PPP3CC	chr8	70315793	70323454	1.25242	0.599536	0.0	0.534125	1.33094	1.12293	2.54575	0.678358	1.02632
XLOC_086445	PPP3CC	chr8	70329409	70345422	3.83824	3.67071	3.47202	5.43166	4.02911	5.19291	4.68204	5.26888	3.60604
XLOC_086906	PPP3CC	chr8	70283078	70313143	29.1339	29.2566	25.6448	54.3554	44.1162	53.4943	49.0363	50.9073	38.9207
XLOC_086907	PPP3CC	chr8	70329409	70345422	34.4205	41.344	34.9753	49.9275	43.6838	47.8734	46.5388	52.9957	25.9368
XLOC_087999	PPP3CC	chr8	70263301	70263679	6.55852	5.87134	7.67646	8.21164	11.5728	15.0906	2.84769	2.86812	3.97637
XLOC_088000	PPP3CC	chr8	70263771	70264304	0.0	1.15101	0.0	1.72458	1.94133	2.97844	1.19921	0.380392	0.167618
XLOC_088001	PPP3CC	chr8	70264638	70265159	0.0	1.18894	3.10928	2.85024	3.23789	3.07557	0.530305	0.981623	2.25046
XLOC_088002	PPP3CC	chr8	70266979	70267664	0.0	0.819756	0.0	2.57942	1.8151	2.41106	0.123188	1.36302	0.597774
XLOC_088003	PPP3CC	chr8	70268206	70268618	0.0	1.69668	0.0	2.79644	2.84366	0.87401	0.497297	0.832679	0.739491
XLOC_088004	PPP3CC	chr8	70268693	70269350	0.0	0.432824	0.0	0.907937	1.80299	1.79648	1.55893	0.862852	1.89333
XLOC_088005	PPP3CC	chr8	70278082	70278490	0.0	0.0	0.0	1.03307	2.22155	0.591812	0.252415	0.281839	0.500781
XLOC_088006	PPP3CC	chr8	70283078	70313143	4.609	2.74884	10.7801	2.88555	2.45306	1.40486	1.56755	3.09169	1.19319
XLOC_088007	PPP3CC	chr8	70313341	70313883	0.0	1.68617	0.0	0.505285	0.291779	0.775873	0.0	0.185845	0.327442
XLOC_045027	PPP6C	chr11	96072791	96075909	28.954	41.074	35.3198	95.0149	66.8289	93.3134	86.897	101.044	77.1964
XLOC_047082	PPP6C	chr11	96069742	96070045	0.0	1.47619	3.85973	0.442977	0.375677	1.00472	0.0	0.0	0.426973
XLOC_047083	PPP6C	chr11	96070118	96070790	0.0	0.0	0.0	1.13336	0.547192	0.14538	0.378661	0.419062	0.735363
XLOC_047084	PPP6C	chr11	96072320	96072630	0.0	0.0	0.0	0.0	0.0	0.0	0.401316	0.90512	0.0
XLOC_057877	PPPDE1	chr16	33250462	33299718	34.6196	45.8821	29.7573	35.5036	34.5675	34.2993	39.2194	47.2688	48.0194
XLOC_059862	PPTC7	chr17	56655984	56664454	1.65788	2.95736	0.0	3.46245	3.3757	1.9889	1.20807	2.15822	3.89205
XLOC_059863	PPTC7	chr17	56673235	56711888	27.4931	39.6718	35.0942	62.6401	50.9575	56.8994	59.619	73.9368	55.6148
XLOC_060261	PPTC7	chr17	56655984	56664454	0.0	0.362521	0.0	0.866862	0.278311	1.1416	0.953593	0.329878	0.489056
XLOC_089856	PRDM1	chr9	44348074	44361656	20.9947	26.105	24.5918	14.4349	12.6326	13.1713	15.231	16.3862	12.0148
XLOC_090695	PRDM1	chr9	44374594	44375253	43.2675	34.0569	22.5489	52.9674	54.2133	44.8836	44.9039	64.3171	64.1179
XLOC_089394	PRDM13	chr9	50856652	50882087	15.424	21.2846	17.8772	21.3915	18.7437	22.0789	25.6326	28.7515	19.3984
XLOC_079586	PRDM5	chr6	4509423	4537549	47.2877	16.2832	15.5938	8.56094	1.78947	5.47386	8.51072	4.17181	4.27472
XLOC_079587	PRDM5	chr6	4537667	4541582	71.6237	31.8285	33.1659	8.36064	1.62946	5.82071	9.14486	4.7737	5.85881
XLOC_079588	PRDM5	chr6	4600484	4643541	4.2231	5.86881	4.61117	3.48086	3.66694	4.22628	2.34511	2.77815	3.72223
XLOC_079999	PRDM5	chr6	4674350	4747178	12.3814	9.9861	6.32287	4.61213	10.0855	6.15307	3.06288	1.72993	6.07481
XLOC_080407	PRDM5	chr6	4505682	4506153	39.2235	13.7816	10.8122	3.92328	0.891644	2.84736	6.72516	2.72124	3.80942
XLOC_089869	PREP	chr9	45172766	45204044	0.0	0.0	0.0	0.286242	0.234557	0.534587	0.0	0.0429443	0.0748687
XLOC_090719	PREP	chr9	45254153	45255218	0.796971	0.476744	0.0	0.0	0.0	0.0	0.0	0.0797835	0.0

XLOC_053265	PREX2	chr14	34085865	34100554	0.0	0.0	0.0	8.65665	9.16646	8.22701	3.45238	3.07741	5.97913
XLOC_053266	PREX2	chr14	34237318	34310609	0.0	0.0756344	0.016582	4.52478	8.3371	3.64018	5.72156	5.13591	5.06834
XLOC_053267	PREX2	chr14	34310720	34345066	28.3936	32.2279	39.4613	153.678	171.752	159.064	158.064	177.033	168.874
XLOC_053542	PREX2	chr14	34146090	34152386	0.570602	0.0	0.0	1.07432	0.357755	0.474524	0.104057	0.572815	0.399184
XLOC_053543	PREX2	chr14	34192319	34201603	0.409233	0.428651	0.480489	0.770791	0.965024	0.724847	0.657483	0.45395	0.268759
XLOC_053544	PREX2	chr14	34237318	34310609	2.28567	1.42352	1.51361	9.43383	15.7329	9.66955	7.89778	6.71389	15.1623
XLOC_054177	PREX2	chr14	34133139	34134464	0.0	0.370633	0.0	0.499835	0.776492	0.38626	0.225734	0.186452	0.433299
XLOC_054178	PREX2	chr14	34187120	34187662	1.88118	1.12412	2.93977	0.505285	1.45889	0.581905	0.0	0.743381	0.0
XLOC_054179	PREX2	chr14	34188607	34190676	0.378356	0.679271	0.0	0.407145	0.593973	0.43316	0.138492	0.15229	0.264878
XLOC_054180	PREX2	chr14	34230671	34231089	0.0	0.828867	4.33498	1.98707	3.63487	2.27792	0.729614	1.89965	3.13141
XLOC_054181	PREX2	chr14	34231665	34233133	0.551879	0.0	0.0	1.63285	3.54719	2.40978	1.45982	1.10829	1.68924
XLOC_054182	PREX2	chr14	34233295	34233589	0.0	0.0	0.0	1.41513	4.39101	2.13649	1.77609	0.502007	3.17966
XLOC_054183	PREX2	chr14	34233823	34234232	0.0	0.0	0.0	0.514489	3.09809	0.884251	2.26317	1.68454	1.49643
XLOC_054184	PREX2	chr14	34234528	34235636	0.0	0.0	0.595254	1.02311	2.67944	0.869057	0.276545	0.838285	1.72885
XLOC_054185	PREX2	chr14	34237318	34310609	0.885827	1.05969	0.69286	0.714522	1.45417	1.01094	0.48181	1.24015	0.541606
XLOC_054186	PREX2	chr14	34237318	34310609	0.0	0.0	0.76597	0.61438	1.68306	1.01552	1.24078	1.0761	1.19722
XLOC_055570	PRG3	chr15	81872305	81915373	47.7148	55.7132	48.5408	108.275	116.072	111.672	112.057	113.544	139.346
XLOC_055888	PRG3	chr15	81865250	81872221	55.0202	44.8792	44.9064	112.749	76.8171	94.2245	108.962	143.394	91.7182
XLOC_055889	PRG3	chr15	81916435	81926260	29.2764	25.6268	17.1238	19.3099	19.0167	16.727	20.8271	26.2517	18.9882
XLOC_076311	PRICKLE1	chr5	38294497	38303979	0.660932	0.0	0.0	0.0889072	0.0259496	0.0344042	0.0302677	0.199639	0.14462
XLOC_069995	PRKAA2	chr3	90052782	90065875	27.8701	20.9129	14.3141	17.2206	17.3906	15.1628	18.0571	16.1458	20.124
XLOC_069996	PRKAA2	chr3	90079372	90118576	112.81	109.981	114.297	159.457	134.761	150.214	159.058	215.231	153.675
XLOC_069997	PRKAA2	chr3	90124868	90144320	70.0489	64.2856	67.6086	313.976	270.948	304.058	372.699	372.192	324.557
XLOC_071642	PRKAA2	chr3	90052782	90065875	3.32935	2.98486	3.90305	1.49078	3.75035	1.03125	3.27269	2.63825	2.6099
XLOC_071643	PRKAA2	chr3	90066617	90067459	2.11327	3.47552	0.0	1.51497	2.47462	1.53311	2.29155	1.05441	1.19908
XLOC_071644	PRKAA2	chr3	90067755	90068098	0.0	0.0	3.03786	1.74146	1.7851	0.397035	3.34831	0.751955	1.01021
XLOC_071645	PRKAA2	chr3	90079372	90118576	26.9164	13.391	35.0059	12.9503	8.02786	7.21103	8.00136	8.33059	7.70621
XLOC_071646	PRKAA2	chr3	90079372	90118576	7.85823	3.6943	6.58789	4.2776	4.57535	3.50113	3.99276	3.38141	4.3196
XLOC_071647	PRKAA2	chr3	90079372	90118576	9.75032	8.73649	0.0	7.9337	5.80231	5.87165	4.71564	2.7067	7.53594
XLOC_071648	PRKAA2	chr3	90079372	90118576	10.979	2.18353	11.4191	5.89057	3.91999	2.98793	4.10538	5.66741	6.64869
XLOC_076265	PRKAG1	chr5	30970056	30974756	3.67258	0.784823	1.2316	0.0	0.0411335	0.163664	0.0478797	0.0	0.0
XLOC_073169	PRKAR2B	chr4	48429218	48473485	50.6324	70.633	52.1508	57.672	40.9761	46.2626	52.7249	71.0608	54.4487
XLOC_074200	PRKAR2B	chr4	48429218	48473485	17.4287	8.65909	22.6394	11.4466	8.33553	12.3401	9.71982	7.71064	10.0042

XLOC_045694	PRKCE	chr11	28337882	28338410	7.8091	4.66607	7.62662	6.64167	4.84214	6.03631	8.67732	5.97378	5.94526
XLOC_045695	PRKCE	chr11	28338521	28338687	61.5548	18.7467	24.5135	5.88014	13.3563	9.26835	7.0028	13.5589	2.6686
XLOC_045696	PRKCE	chr11	28338782	28339938	6.519	4.76656	1.69998	2.92187	3.00495	2.70807	3.227	3.70141	3.29191
XLOC_083421	PRKCSH	chr7	17034517	17062408	1.57429	1.61627	0.880156	2.13999	2.8179	2.6374	1.54809	0.839252	1.41939
XLOC_045553	PRKD3	chr11	19720489	19721478	13.0473	10.6658	7.48409	7.25031	4.9644	4.78333	8.83366	9.74382	6.07847
XLOC_059959	PRMT10;TMEM184C;EDNRA	chr17	10597112	10840068	0.0	0.951233	4.97541	0.142528	0.123703	0.32877	0.0	0.0	0.277297
XLOC_062949	PRMT7	chr18	35809961	35810342	3.23536	2.89646	5.04931	2.89352	2.23466	1.98563	0.281165	1.88741	2.24211
XLOC_062950	PRMT7	chr18	35810537	35812353	2.61645	1.43523	1.70627	1.09486	1.50538	1.27033	1.11613	1.31548	0.839358
XLOC_062951	PRMT7	chr18	35812511	35813284	2.3502	2.10803	1.83773	0.947594	1.55801	1.94713	1.58932	1.05384	1.64053
XLOC_062952	PRMT7	chr18	35813426	35813884	2.40706	2.87528	5.63936	1.077	2.60256	2.22655	1.27337	1.65408	2.30005
XLOC_062953	PRMT7	chr18	35814100	35816400	2.02537	0.707065	0.792564	0.817339	0.848168	0.808251	0.432788	0.815631	0.70906
XLOC_062954	PRMT7	chr18	35816463	35816910	2.4985	0.746064	1.95102	0.670695	1.92822	0.769956	0.439864	0.245008	1.73575
XLOC_062955	PRMT7	chr18	35817063	35818074	0.0	1.01377	1.3257	1.06333	0.530115	0.96731	1.3068	0.423896	0.592226
XLOC_076948	PRMT8	chr5	106840516	106845414	0.0	0.0	0.0	0.0	0.482138	0.213752	0.0	0.204564	1.9856
XLOC_066122	PROC	chr2	5020556	5064080	10.0025	16.3423	17.0639	6.75684	6.51276	6.01905	4.66054	7.07733	8.59484
XLOC_050917	PROCR	chr13	65065943	65113221	27.4621	21.2217	18.8042	14.1998	16.7797	16.2593	16.1313	15.0026	22.3497
XLOC_052254	PROCR	chr13	65062849	65063891	0.817696	0.733698	0.0	0.439755	0.575614	0.594095	0.371086	0.245512	0.571531
XLOC_052255	PROCR	chr13	65064889	65065786	11.7365	10.8217	11.474	8.765	9.168	7.91015	9.73585	7.62004	11.6145
XLOC_061902	PRODH	chr17	74187035	74188480	0.561738	0.336106	2.19767	0.352546	0.176111	0.233589	0.614742	0.338387	0.196498
XLOC_061903	PRODH	chr17	74192124	74192801	4.17682	0.416182	4.35373	0.374156	0.0	0.287985	0.125036	0.138366	0.36416
XLOC_061904	PRODH	chr17	74193442	74193820	0.0	0.978557	0.0	1.17309	0.503167	0.670695	0.0	0.0	0.284027
XLOC_061905	PRODH	chr17	74194521	74196714	1.06592	0.531587	1.11228	0.637251	0.585786	0.702707	0.325277	0.393396	0.497524
XLOC_061906	PRODH	chr17	74197014	74197516	4.19928	3.76317	1.64021	1.12767	1.95057	1.94581	1.11689	0.413758	1.27812
XLOC_061907	PRODH	chr17	74198090	74198903	1.10338	0.32992	0.0	0.692091	0.861045	0.800239	0.896579	0.330131	0.481506
XLOC_061908	PRODH	chr17	74199703	74200546	2.11019	2.52397	0.82513	1.60731	1.15315	1.31219	0.476724	1.47405	1.10523
XLOC_061910	PRODH	chr17	74390570	74391406	5.32989	5.73745	5.83544	9.74325	8.40426	7.51195	10.2088	11.2743	10.6076
XLOC_060310	PRODH;AIFM3	chr17	74299481	74316657	49.5498	62.0367	41.1961	338.605	305.77	327.958	346.531	388.851	414.029
XLOC_060311	PRODH;MED15	chr17	74426778	74459146	0.670785	0.200743	0.175015	1.40378	0.474467	0.791984	0.471967	0.991876	0.60691
XLOC_061911	PRODH;MED15	chr17	74426296	74426560	0.0	0.0	0.0	1.2012	1.51046	2.02659	0.0	3.15201	1.15169

XLOC_061912	PRODH;MED15	chr17	74426778	74459146	40.0213	91.9117	62.4239	193.967	157.015	161.057	128.109	162.456	181.594
XLOC_060309	PRODH;SNAP29;PI4KA;SERPIND1	chr17	74200612	74295887	30.367	21.9191	21.2208	27.0757	21.7516	25.3711	26.852	31.2386	21.0097
XLOC_080278	PROM1	chr6	115954740	115994679	20.8271	16.2338	20.7505	0.87914	0.61328	0.603768	0.358501	0.505994	0.458526
XLOC_080279	PROM1	chr6	116021321	116193363	32.053	37.5407	26.6603	4.93411	4.95188	5.03535	5.23189	4.56604	5.20101
XLOC_082226	PROM1	chr6	116018472	116021233	3.33346	2.99261	2.82648	0.722478	0.851074	0.63645	0.967783	0.951576	0.997039
XLOC_038321	PROS1	chr1	37819593	37865103	4.32994	4.24108	2.76804	2.4011	3.01318	3.46499	2.31706	3.1689	3.92578
XLOC_051455	PRPF18	chr13	28574495	28575102	1.60912	0.961799	0.0	0.0	0.0	0.166195	0.287876	0.0	0.0
XLOC_051456	PRPF18	chr13	28584462	28585132	0.0	0.421859	0.0	0.0	0.0	0.0	0.0	0.0	0.246073
XLOC_051457	PRPF18	chr13	28585653	28586430	3.50253	0.349072	0.912938	0.0	0.0910617	0.0	0.105285	0.116348	0.0
XLOC_051458	PRPF18	chr13	28592904	28593611	2.63251	0.393487	0.0	0.0	0.0	0.0	0.118374	0.0	0.0
XLOC_051459	PRPF18	chr13	28593665	28594272	1.60912	0.961799	0.0	0.288221	0.0	0.0	0.0	0.0	0.0
XLOC_051460	PRPF18	chr13	28595952	28596962	0.0	0.761217	0.663626	0.0760414	0.0663416	0.17608	0.0769595	0.0848775	0.14823
XLOC_051461	PRPF18	chr13	28598307	28598638	4.16224	0.0	0.0	0.0	0.317357	0.0	0.0	0.0	0.359577
XLOC_051462	PRPF18	chr13	28600461	28601133	0.0	0.840443	0.0	0.0	0.0	0.0	0.252441	0.279375	0.0
XLOC_051463	PRPF18	chr13	28603415	28604822	0.578822	0.692646	0.452893	0.0518945	0.0	0.0	0.10554	0.0	0.0506153
XLOC_051464	PRPF18	chr13	28605125	28605422	0.0	0.0	4.02208	0.0	0.0	0.0	0.0	0.0	0.0
XLOC_050791	PRPF18;FRMD4A;MIR2304	chr13	28608454	28885371	12.0661	7.84624	11.132	1.04548	0.607543	0.63334	0.549219	1.1597	0.511706
XLOC_051465	PRPF18;FRMD4A;MIR2304	chr13	28608454	28885371	0.0	1.22731	4.27969	0.0	0.0	0.141561	0.122963	0.0	0.0
XLOC_051466	PRPF18;FRMD4A;MIR2304	chr13	28608454	28885371	0.0	0.557714	2.91706	0.0	0.0	0.192484	0.0	0.0	0.0
XLOC_051467	PRPF18;FRMD4A;MIR2304	chr13	28608454	28885371	0.0	4.96519	0.0	0.0	0.0	0.847453	0.0	0.0	0.0
XLOC_051468	PRPF18;FRMD4A;MIR2304	chr13	28608454	28885371	0.936265	0.559988	0.73229	0.0839092	0.146321	0.0971051	0.169586	0.374273	0.16353
XLOC_051469	PRPF18;FRMD4A;MIR2304	chr13	28608454	28885371	1.04898	1.25467	0.0	0.0939997	0.081894	0.108719	0.0948041	0.104691	0.0
XLOC_051470	PRPF18;FRMD4A;MIR2304	chr13	28608454	28885371	0.0	0.858937	2.2465	0.154448	0.0	0.059691	0.104712	0.461145	0.0502147

XLOC_051471	PRPF18;FRMD4A;MIR2304	chr13	28608454	28885371	1.4898	3.56231	2.3291	0.26688	0.347694	0.307969	0.534222	0.59153	0.0
XLOC_051472	PRPF18;FRMD4A;MIR2304	chr13	28608454	28885371	1.11703	0.0	0.0	0.0	0.0	0.115723	0.0	0.222775	0.0
XLOC_051473	PRPF18;FRMD4A;MIR2304	chr13	28608454	28885371	0.0	0.826012	0.0	0.0	0.0	0.0	0.0	0.0	0.0
XLOC_051474	PRPF18;FRMD4A;MIR2304	chr13	28608454	28885371	1.21912	0.182351	1.90771	0.109297	0.0	0.253399	0.111081	0.122328	0.0
XLOC_051475	PRPF18;FRMD4A;MIR2304	chr13	28608454	28885371	4.09021	1.22269	1.0659	0.122136	0.0	0.141032	0.0	0.0	0.356647
XLOC_051476	PRPF18;FRMD4A;MIR2304	chr13	28608454	28885371	0.0	0.584835	1.52945	0.0	0.0	0.201749	0.174003	0.386432	0.0
XLOC_051477	PRPF18;FRMD4A;MIR2304	chr13	28608454	28885371	0.0	1.31624	0.0	0.394754	0.0	0.448848	0.0	0.0	0.0
XLOC_051478	PRPF18;FRMD4A;MIR2304	chr13	28608454	28885371	0.0	0.497476	5.20406	0.0	0.129322	0.171875	0.0	0.0	0.289987
XLOC_051479	PRPF18;FRMD4A;MIR2304	chr13	28608454	28885371	1.46862	0.438968	1.14802	0.131546	0.34281	0.0	0.0	0.291639	0.128008
XLOC_051480	PRPF18;FRMD4A;MIR2304	chr13	28608454	28885371	1.41961	1.69736	3.3293	0.127163	0.0	0.146794	0.254856	0.141041	0.247516
XLOC_051481	PRPF18;FRMD4A;MIR2304	chr13	28608454	28885371	0.0	0.840443	1.099	0.0	0.109438	0.0	0.378663	0.0	0.0
XLOC_051482	PRPF18;FRMD4A;MIR2304	chr13	28608454	28885371	1.70793	0.0	0.0	0.305887	0.0	0.0	0.0	0.0	0.0
XLOC_051483	PRPF18;FRMD4A;MIR2304	chr13	28608454	28885371	0.0	0.484126	1.26611	0.0	0.0	0.334603	0.0	0.0	0.0
XLOC_051484	PRPF18;FRMD4A;MIR2304	chr13	28608454	28885371	0.0	0.507983	0.0	0.304451	0.0	0.175473	0.0	0.504924	0.0
XLOC_051485	PRPF18;FRMD4A;MIR2304	chr13	28608454	28885371	2.20678	0.32992	0.0	0.0988701	0.0861045	0.11432	0.0	0.330136	0.288905

XLOC_051486	PRPF18;FRMD4A;MIR2304	chr13	28608454	28885371	0.0	0.531688	0.0	0.159329	0.0	0.0	0.0	0.176011	0.0
XLOC_051487	PRPF18;FRMD4A;MIR2304	chr13	28608454	28885371	0.0	0.297707	3.11444	0.0	0.0	0.309647	0.0	0.695942	0.0
XLOC_051488	PRPF18;FRMD4A;MIR2304	chr13	28608454	28885371	0.0	0.0	2.15615	0.123531	0.107375	0.142632	0.247751	0.0	0.120236
XLOC_051489	PRPF18;FRMD4A;MIR2304	chr13	28608454	28885371	0.0	0.0	0.0	0.387708	0.0	0.441007	0.0	0.416607	0.0
XLOC_051490	PRPF18;FRMD4A;MIR2304	chr13	28608454	28885371	2.38329	0.711732	1.86126	0.213276	0.368189	0.244985	0.420445	0.468068	0.0
XLOC_064773	PRPF8	chr19	23343809	23344064	0.0	15.1874	5.67212	2.61398	2.72896	4.39857	2.39502	0.68228	3.12717
XLOC_084326	PRR22	chr7	19770970	19772106	0.0	0.0	0.0	0.530054	0.578493	0.230264	0.26874	0.148093	0.0645964
XLOC_077017	PRR5	chr5	116013535	116024660	18.2105	18.8637	17.2758	20.3259	23.0713	21.1559	25.064	22.302	23.7892
XLOC_084416	PRRC1	chr7	27689592	27690632	0.0	0.49024	0.0	2.93834	3.78197	2.38174	1.48766	1.72244	3.22212
XLOC_092571	PRRG1	chrX	111388134	111538116	5.38384	4.91832	4.39448	27.6829	30.3702	32.4136	31.689	19.6519	22.6042
XLOC_093531	PRRG1	chrX	111388134	111538116	3.13732	1.87263	0.0	3.64778	2.89136	3.85362	3.00412	1.22151	1.08744
XLOC_093532	PRRG1	chrX	111388134	111538116	1.74081	2.60096	1.36042	0.623534	0.946085	0.89859	1.86414	0.861362	0.757915
XLOC_093534	PRRG1	chrX	111388134	111538116	0.0	0.529101	0.0	0.0	0.0	0.0	0.0	0.0	0.0
XLOC_080473	PRSS12	chr6	7981629	7983614	69.2558	83.9553	68.1357	133.043	111.193	116.632	132.176	146.436	119.852
XLOC_086874	PSAT1	chr8	54519724	54530139	11.1331	9.26689	9.80401	24.2776	30.4586	26.9561	31.2558	21.4796	25.5475
XLOC_087796	PSAT1	chr8	54515486	54516178	0.0	2.02258	0.0	0.606115	1.05387	0.699925	0.851299	0.269093	1.17997
XLOC_087797	PSAT1	chr8	54517036	54518278	0.666891	0.997455	0.521753	0.538064	0.470026	0.900684	0.303496	0.200605	0.52468
XLOC_087798	PSAT1	chr8	54518458	54518859	2.9701	0.0	2.31822	0.265669	2.51229	1.21703	1.03716	1.15861	0.515016
XLOC_041695	PSEN1	chr10	84917669	85015709	15.2545	26.0259	20.4325	14.9185	11.943	14.3822	20.5539	24.5775	18.3736
XLOC_043246	PSEN1	chr10	85015973	85016610	18.1012	22.9935	16.5074	11.4841	9.50381	10.6008	13.9236	22.0055	15.6436
XLOC_043247	PSEN1	chr10	85016828	85018083	1.97697	1.37991	3.09346	0.590772	1.39346	1.30085	1.49973	2.51114	1.84348
XLOC_058745	PSEN2	chr16	30599566	30601369	1.75808	1.18355	1.03184	3.78347	2.48275	2.78932	2.97289	5.25907	2.57639
XLOC_058746	PSEN2	chr16	30601502	30602944	4.5044	1.17912	2.64336	3.53369	2.95672	3.57052	1.95118	5.48327	2.26499
XLOC_050870	PSMA7	chr13	55497873	55513432	36.5811	35.1174	40.5958	14.0853	15.1598	18.044	16.7681	17.4563	10.3038
XLOC_051892	PSMA7	chr13	55497873	55513432	0.0	12.1823	10.6187	2.73838	3.39039	1.73855	4.42269	3.30185	3.53488
XLOC_044381	PSMB7	chr11	95414292	95492691	21.5166	18.5867	18.9862	11.2999	11.5509	12.1938	12.1662	14.5569	14.6409
XLOC_047060	PSMB7	chr11	95414292	95492691	0.0	5.20429	10.208	2.5993	0.564552	1.80014	1.56197	2.44957	1.51769

XLOC_062441	PSMC4;FBL	chr18	49698141	49755575	3.23742	5.38355	2.56905	11.011	8.60032	10.4608	9.44322	13.0905	9.20804
XLOC_063143	PSMC4;FBL	chr18	49696433	49697989	5.17151	2.9397	1.61866	5.5642	4.62244	6.34568	5.99459	6.07813	5.56361
XLOC_065818	PSMD1	chr2	119742340	119751262	1.84087	1.65235	2.16082	7.71676	4.47645	4.97886	5.34031	12.8149	8.05226
XLOC_068381	PSMD1	chr2	119733526	119735779	3.45132	5.06043	4.5919	5.72585	4.47118	5.53289	8.31142	8.54694	6.37347
XLOC_065817	PSMD1;HTR2B	chr2	119675136	119733455	16.8766	23.4228	22.0908	23.0423	25.3473	22.7217	29.871	31.8909	24.2742
XLOC_045156	PSMD13	chr11	107078627	107131332	30.0858	46.7171	33.4138	41.9134	27.5017	42.4238	35.5914	47.558	24.1763
XLOC_047431	PSMD13	chr11	107078627	107131332	1.49684	1.79064	3.51244	1.94528	2.10828	3.10813	2.17624	1.04941	2.02729
XLOC_065620	PSMD14	chr2	35109029	35201729	11.5254	13.902	10.8604	12.7157	14.1638	12.5428	15.163	12.3281	14.5195
XLOC_066258	PSMD14	chr2	35109029	35201729	13.6413	13.7975	16.2916	9.69936	9.07902	9.93753	11.7751	12.6253	8.11143
XLOC_060242	PSMD9;HPD	chr17	55652909	55712634	2.03105	6.94272	5.35196	2.45903	2.79893	2.75846	5.26467	5.81288	4.94111
XLOC_061632	PSMD9;HPD	chr17	55652909	55712634	0.0	1.21907	0.0	0.182654	1.10625	0.840715	1.62982	1.40837	0.709903
XLOC_086395	PTAR1	chr8	46163258	46195740	6.70027	13.3925	13.3948	8.56584	13.5331	9.87639	12.7492	8.43453	11.9451
XLOC_086862	PTAR1	chr8	46033254	46151937	6.36458	8.65355	9.96305	2.65634	3.99502	3.41914	4.89854	1.72855	3.91863
XLOC_087743	PTAR1	chr8	46033254	46151937	2.81708	2.52628	3.30341	1.00939	0.767544	0.728302	1.01173	0.27991	0.491192
XLOC_082985	PTBP1	chr7	45018068	45021645	20.2225	23.4479	15.8144	8.99874	15.3623	10.926	10.4277	10.6235	12.4999
XLOC_083616	PTBP1	chr7	45002926	45016369	26.3137	25.9602	25.7565	23.6259	22.2988	23.5352	24.5863	32.0625	16.2995
XLOC_086510	PTCH1	chr8	83566709	83594492	7.48402	7.36047	5.37597	21.1068	18.6328	21.4989	20.6267	18.4817	17.3161
XLOC_086968	PTCH1	chr8	83566709	83594492	65.6743	81.1096	73.7146	124.609	174.327	144.944	140.251	110.231	134.974
XLOC_088390	PTCH1	chr8	83566709	83594492	5.12421	1.5276	0.0	1.836	2.71872	3.11718	0.865377	0.977782	0.883419
XLOC_088391	PTCH1	chr8	83566709	83594492	0.0	1.57571	0.0	2.83381	2.31966	1.81378	1.72499	1.21753	0.76523
XLOC_058984	PTCHD2	chr16	43027329	43029538	1.76268	1.7933	1.65542	0.252913	0.747296	0.513755	0.193654	0.141942	0.740473
XLOC_058985	PTCHD2	chr16	43029602	43030397	1.13454	0.678453	3.54876	0.609952	0.885137	0.822676	0.307123	0.113108	0.693065
XLOC_058986	PTCHD2	chr16	43031303	43032307	2.56322	0.511072	2.00498	0.22974	0.46766	0.620625	0.464978	0.427364	0.970302
XLOC_058987	PTCHD2	chr16	43035232	43035640	0.0	0.0	0.0	0.258268	0.888621	0.591812	0.757246	0.845516	2.7543
XLOC_058988	PTCHD2	chr16	43035878	43036452	1.73663	2.07577	1.35715	0.0	1.34835	0.537671	0.774916	0.171866	0.45366
XLOC_058989	PTCHD2	chr16	43036642	43037369	1.27002	1.51874	0.0	0.113782	0.197952	0.262898	0.571569	0.25281	1.10778
XLOC_053696	PTDSS1	chr14	70217955	70289345	9.79902	9.50593	14.1779	1.39305	3.97094	2.16497	3.28524	1.65283	5.16787
XLOC_054776	PTDSS1	chr14	70217955	70289345	2.80879	0.838492	0.0	1.7589	2.16251	0.864002	0.491793	0.274435	3.65483
XLOC_054777	PTDSS1	chr14	70300079	70300742	2.86167	0.0	1.11854	1.15351	0.445459	0.443836	0.128409	0.284263	0.498926
XLOC_054778	PTDSS1	chr14	70303414	70304356	6.45355	11.58	10.8165	22.144	18.9491	21.2294	13.5298	18.4304	14.5738
XLOC_044504	PTGDS;C11H9orf142;CLIC3	chr11	106216461	106273021	13.5856	17.3558	13.889	18.0146	16.9139	16.8896	22.7208	22.7217	20.6212

XLOC_044505	PTGDS;C11H9orf142;CLIC3	chr11	106216461	106273021	21.9007	22.5078	21.3695	45.3327	35.9691	44.3684	50.5396	58.7749	39.7802
XLOC_045143	PTGDS;C11H9orf142;CLIC3	chr11	106216461	106273021	29.0135	35.9551	29.3594	72.3283	53.3154	64.0161	78.2678	99.2476	69.9194
XLOC_045144	PTGDS;C11H9orf142;CLIC3	chr11	106216461	106273021	13.3627	16.5124	12.8674	26.6571	23.3972	29.1847	26.3103	25.5838	25.2525
XLOC_047401	PTGDS;C11H9orf142;CLIC3	chr11	106216461	106273021	1.29616	0.904603	2.02796	1.58788	0.982738	1.52786	0.592259	0.694921	0.529041
XLOC_047402	PTGDS;C11H9orf142;CLIC3	chr11	106216461	106273021	0.838421	1.00204	3.51458	1.80177	0.524017	1.04311	0.970888	0.838074	0.731736
XLOC_045064	PTGES2	chr11	98771929	98775433	31.2607	18.9806	29.6365	1.78284	1.3323	2.35783	1.80126	1.51452	1.3236
XLOC_043256	PTGR2	chr10	85659670	85660472	0.0	0.671091	0.0	2.21223	4.46561	2.55767	5.67197	4.92343	4.40722
XLOC_053763	PTK2	chr14	4032252	4032521	0.0	0.0	0.0	0.0	4.3434	0.0	1.0647	1.81426	1.10283
XLOC_053764	PTK2	chr14	4040171	4040738	0.0	0.0	1.38031	0.790816	1.23396	0.364509	1.10283	0.524224	2.15304
XLOC_088199	PTK2B	chr8	75711840	75713262	39.465	50.9903	39.8296	85.5339	90.6808	90.8483	87.2477	71.4834	76.9248
XLOC_072724	PTN	chr4	101717913	101814581	0.538007	0.321999	0.210545	0.120624	0.149707	0.0742606	0.271331	0.0542078	0.236091
XLOC_074769	PTN	chr4	101717913	101814581	0.0	0.315991	0.0	0.0	0.0	0.0	0.0963825	0.106086	0.369509
XLOC_055858	PTPMT1;CELF1	chr15	78560926	78572732	34.14	4.39353	0.884418	0.688482	0.951767	0.912461	1.07993	0.764696	1.28199
XLOC_072605	PTPN12	chr4	43875533	43944610	7.89742	7.60809	6.10046	2.83405	3.57234	3.03481	3.4473	2.97315	3.36388
XLOC_074077	PTPN12	chr4	43875533	43944610	0.0	1.88907	4.93938	1.41523	0.486003	1.2954	1.10143	1.23179	1.37103
XLOC_074078	PTPN12	chr4	43875533	43944610	2.8392	0.848746	1.10977	1.14447	0.552501	0.293608	0.764561	0.141036	0.371272
XLOC_074079	PTPN12	chr4	43875533	43944610	0.962423	0.287856	1.50546	0.603762	0.751894	0.798432	0.696956	0.384605	1.0085
XLOC_074080	PTPN12	chr4	43875533	43944610	0.0	0.0	0.0	0.239227	0.412159	0.548809	0.234643	0.0	0.464121
XLOC_043957	PTPN13	chr11	5715198	5784768	3.69211	4.78416	3.91192	6.49093	5.90102	5.46073	5.1599	6.90982	5.59652
XLOC_044642	PTPN13	chr11	5715198	5784768	12.736	10.5334	13.0552	6.04298	5.64891	5.44349	3.55212	4.76182	4.79492
XLOC_045402	PTPN13	chr11	5715198	5784768	2.47288	2.2153	1.9311	0.0	0.76353	0.254072	0.653328	0.242569	0.0
XLOC_045403	PTPN13	chr11	5715198	5784768	3.54199	1.8546	1.73238	0.635208	0.937846	0.598803	0.283268	0.178067	0.464832
XLOC_045404	PTPN13	chr11	5715198	5784768	0.0	1.22043	0.0	0.365922	0.624309	0.0	0.35068	0.394186	0.353626
XLOC_045405	PTPN13	chr11	5715198	5784768	2.61015	1.05024	2.0426	0.419858	0.136525	0.362084	0.278367	0.262466	0.380614
XLOC_050554	PTPRA;PTPRA	chr13	52622674	52646468	9.38429	1.53155	1.04288	20.0577	15.0746	16.2174	15.1997	18.8712	18.3852

XLOC_051840	PTPRA;PTPRA	chr13	52647028	52647435	8.69628	4.32641	0.0	29.0416	20.7413	28.2218	18.243	24.8978	23.3786
XLOC_051841	PTPRA;PTPRA	chr13	52647654	52649142	3.2615	1.78887	0.850672	5.36106	4.55938	3.27794	3.76881	4.85826	4.42121
XLOC_051842	PTPRA;PTPRA	chr13	52729784	52731202	0.0	0.0	0.0	0.0514419	0.0449665	0.0	0.0523142	0.0	0.100349
XLOC_076314	PTPRB;KCNMB4	chr5	43234912	43289538	14.9002	10.3378	9.3895	0.72798	0.306186	0.79515	0.442471	0.178642	0.37418
XLOC_077791	PTPRB;KCNMB4	chr5	43234912	43289538	0.856459	2.81755	4.01944	0.383806	0.0669658	0.622087	0.310708	0.0	0.299259
XLOC_077792	PTPRB;KCNMB4	chr5	43234912	43289538	1.07393	1.28445	1.67963	0.0962304	0.335292	0.222569	0.38804	0.107141	0.0937373
XLOC_077793	PTPRB;KCNMB4	chr5	43234912	43289538	4.91858	1.46633	0.0	0.0	0.373232	0.0	0.0	0.0	0.424141
XLOC_077794	PTPRB;KCNMB4	chr5	43234912	43289538	7.6847	0.57399	1.50106	0.172	0.0	0.0	0.51263	0.189703	0.334352
XLOC_077795	PTPRB;KCNMB4	chr5	43234912	43289538	3.812	0.56946	0.0	0.853216	0.295565	0.392988	0.0	0.376475	0.165864
XLOC_077796	PTPRB;KCNMB4	chr5	43234912	43289538	2.37553	0.709413	0.0	0.212577	0.0	0.0	0.209559	0.0	0.0
XLOC_077797	PTPRB;KCNMB4	chr5	43234912	43289538	5.53716	0.827598	4.32877	0.124003	0.0	0.429518	0.0	0.0	0.120693
XLOC_077798	PTPRB;KCNMB4	chr5	43234912	43289538	7.70761	9.19606	12.023	0.0	0.0	0.0	0.331501	0.0	0.333206
XLOC_077799	PTPRB;KCNMB4	chr5	43234912	43289538	2.63735	1.57682	4.12384	0.118133	0.102726	0.409322	0.118585	0.0	0.114999
XLOC_077800	PTPRB;KCNMB4	chr5	43234912	43289538	11.9294	0.89018	2.32774	0.0	0.22931	0.0	0.0	0.290805	0.258558
XLOC_077801	PTPRB;KCNMB4	chr5	43234912	43289538	6.04638	4.96974	1.18156	0.135389	0.235147	0.312432	0.27091	0.150005	0.526927
XLOC_077802	PTPRB;KCNMB4	chr5	43234912	43289538	0.747569	0.894401	4.09362	0.134019	0.0585031	0.155247	0.0679382	0.0	0.195987
XLOC_077803	PTPRB;KCNMB4	chr5	43234912	43289538	4.36122	1.15985	3.41271	0.173797	0.0760077	0.1512	0.177022	0.146092	0.127166
XLOC_077804	PTPRB;KCNMB4	chr5	43234912	43289538	1.72104	0.514722	3.36546	0.154252	0.134565	0.17858	0.234123	0.0	0.0
XLOC_077805	PTPRB;KCNMB4	chr5	43234912	43289538	13.2716	0.792491	4.14471	0.237475	0.0	0.272405	0.0	0.0	0.230374
XLOC_070086	PTPRF	chr3	102995462	103058225	230.689	278.929	216.869	305.001	301.814	308.677	360.797	389.019	379.87
XLOC_070087	PTPRF	chr3	103058292	103066058	24.4643	31.0608	29.0739	52.6125	53.0681	53.536	61.1994	59.9331	59.8201
XLOC_071892	PTPRF	chr3	102995462	103058225	5.70037	5.45351	6.23972	7.76255	9.78213	8.14731	6.99427	8.52265	9.94755
XLOC_071893	PTPRF	chr3	102995462	103058225	5.90626	0.0	4.6002	3.17189	3.56384	1.79039	4.93442	3.35457	3.04858
XLOC_071894	PTPRF	chr3	102995462	103058225	3.96653	2.07561	0.0	1.33276	2.32317	1.13073	1.7048	1.78228	2.94328

XLOC_071895	PTPRF	chr3	102995462	103058225	6.6888	4.6609	8.70551	2.19454	3.27541	1.14673	1.38128	1.09644	2.51879
XLOC_071896	PTPRF	chr3	103066173	103066700	17.618	12.8664	4.58833	26.6384	22.9097	20.7801	26.0999	30.141	28.4436
XLOC_071897	PTPRF	chr3	103066866	103067866	18.0277	16.6887	15.4444	39.0872	37.8577	37.8587	45.4694	44.0536	49.5696
XLOC_071898	PTPRF	chr3	103068271	103068655	0.0	0.0	2.49139	1.42767	2.20583	0.979925	3.05416	2.48449	2.76598
XLOC_071899	PTPRF	chr3	103068971	103070070	4.60913	2.75724	3.60564	2.96092	2.70489	1.67489	2.72172	1.38473	3.62491
XLOC_071900	PTPRF	chr3	103073605	103074439	0.0	0.319689	0.836096	0.191608	0.834544	0.553972	0.386353	0.0	0.933232
XLOC_089971	PTPRK	chr9	66955382	66975444	2.74629	7.04272	7.24384	5.84612	3.98783	4.75189	5.78045	5.9791	5.976
XLOC_089972	PTPRK	chr9	67022557	67050051	239.425	225.168	198.832	63.6406	58.4446	66.2656	62.1196	58.2674	66.6801
XLOC_089973	PTPRK	chr9	67220136	67633949	8.88678	9.57418	10.348	16.4113	12.2572	12.7771	13.2003	13.6201	10.2907
XLOC_091166	PTPRK	chr9	67020816	67022165	1.214	1.63427	3.32448	0.108838	0.190232	0.12617	0.387167	0.243632	0.371517
XLOC_091167	PTPRK	chr9	67050605	67051002	0.0	0.901269	0.0	0.810273	0.0	0.618489	0.0	0.0	0.0
XLOC_091168	PTPRK	chr9	67207124	67207935	0.0	0.330929	0.0	1.28924	1.12275	1.26134	0.199837	0.220752	0.676163
XLOC_091169	PTPRK	chr9	67212796	67214102	0.0	0.0	0.492696	0.508099	0.986591	1.17788	0.286781	0.315854	0.385395
XLOC_091170	PTPRK	chr9	67217087	67217892	1.11702	0.333993	0.0	4.00361	3.48635	3.35595	3.62972	2.78462	3.89945
XLOC_091171	PTPRK	chr9	67218145	67219230	0.0	0.932942	0.610004	2.4464	1.70829	2.34769	1.629	1.32735	1.49905
XLOC_091172	PTPRK	chr9	67219418	67219897	2.24985	1.34394	0.0	4.02722	6.08895	3.47185	2.78497	2.2128	3.71539
XLOC_077302	PTPRQ	chr5	10236450	10237255	0.0	0.0	0.0	1.0009	0.610112	0.694335	1.20991	0.891078	0.584918
XLOC_044031	PUS10	chr11	43528725	43587391	5.60734	5.25302	3.05322	3.18764	3.1035	2.84424	2.34847	5.00583	2.64701
XLOC_045866	PUS10	chr11	43587473	43587747	6.14557	1.83205	0.0	0.550707	0.926643	3.72593	2.04805	1.74316	2.11561
XLOC_076301	PUS7L;IRAK4	chr5	36868737	36955072	4.01394	6.27347	4.44951	1.19491	2.52028	1.53256	1.5617	1.16104	1.01329
XLOC_055418	PVRL1	chr15	30775167	30887244	0.0	4.90151	1.97215	0.564947	0.294877	0.652695	0.22707	0.878759	0.220017
XLOC_059805	PXMP2;POLE	chr17	45506654	45520698	3.0073	3.93407	4.7037	23.6613	16.4147	20.5287	20.494	27.6138	20.8457
XLOC_057851	PYCR2	chr16	29683425	29700454	0.0	0.0	0.0	1.27714	0.953918	0.316577	1.10463	1.82935	0.622115
XLOC_050523	PYGB;ABHD12	chr13	43180404	43197801	10.8353	9.4602	7.78508	4.89799	5.10381	3.90347	3.83665	5.30962	5.20774
XLOC_051718	PYGB;ABHD12	chr13	43180404	43197801	0.627541	0.563182	0.0	0.562596	0.58991	0.52169	0.628755	0.503626	1.04245
XLOC_051719	PYGB;ABHD12	chr13	43180404	43197801	0.0	0.0	0.0	1.40295	0.810928	1.25755	0.466033	0.344542	2.27373
XLOC_051720	PYGB;ABHD12	chr13	43180404	43197801	1.09173	0.326438	0.0	0.586959	1.02243	1.1312	0.690119	0.544482	1.42931
XLOC_050821	PYGB;ENTPD6	chr13	43151736	43171864	7.59487	9.21069	6.36058	9.33566	10.0755	9.56288	9.70369	10.993	10.0057
XLOC_090056	QKI	chr9	100458993	100466341	0.0	0.596108	0.0	0.0	0.0	0.411194	0.0	0.0	0.173587

XLOC_090057	QKI	chr9	100473126	100546120	30.5616	29.386	30.2574	13.3815	13.2177	14.2764	14.9665	14.0781	16.1524
XLOC_091579	QKI	chr9	100457507	100458777	0.650106	1.16684	0.50863	0.349687	0.152753	0.0	0.23676	0.325994	0.170502
XLOC_091580	QKI	chr9	100473126	100546120	0.0	0.491825	1.28624	1.62123	0.895094	0.849696	0.588461	0.0	0.573411
XLOC_091581	QKI	chr9	100473126	100546120	3.4323	0.512796	0.0	0.614672	0.399772	1.23984	0.76601	0.679479	0.448312
XLOC_091582	QKI	chr9	100473126	100546120	0.661497	0.59355	1.03492	0.415052	0.362583	0.687155	0.541852	0.397935	0.63601
XLOC_091583	QKI	chr9	100473126	100546120	0.0	0.597055	3.12303	0.715705	0.623755	0.517495	0.541839	0.598145	0.435812
XLOC_091584	QKI	chr9	100473126	100546120	5.95353	0.0	1.55038	1.24357	1.84523	1.84034	0.528902	2.34959	1.03585
XLOC_091585	QKI	chr9	100473126	100546120	2.29881	1.54731	0.899312	0.772855	0.720603	0.537644	1.10032	0.576883	1.10559
XLOC_091586	QKI	chr9	100473126	100546120	15.7013	0.0	4.0792	0.936681	0.0	0.530403	0.882179	1.49582	0.451041
XLOC_091587	QKI	chr9	100473126	100546120	0.0	0.879328	2.29941	0.0	0.0	0.301826	0.771861	0.287378	0.0
XLOC_091588	QKI	chr9	100473126	100546120	12.4928	2.98426	11.7061	0.894261	0.96411	1.28326	1.09966	1.22504	1.51878
XLOC_091589	QKI	chr9	100473126	100546120	2.8352	3.73194	4.43666	1.2201	1.06656	1.11995	1.08581	1.13849	1.19006
XLOC_045554	QPCT	chr11	19832543	19832871	0.0	0.0	0.0	0.378692	0.645466	0.430965	0.362105	0.0	0.0
XLOC_045555	QPCT	chr11	19833556	19834233	0.0	0.0	0.0	0.0	0.32519	0.287985	0.375109	0.415097	0.0
XLOC_045556	QPCT	chr11	19834348	19834794	2.50715	0.0	0.0	0.224338	0.193477	0.257528	0.44132	1.96665	0.217714
XLOC_045557	QPCT	chr11	19835028	19836506	0.0	0.0	0.0	1.03122	0.643985	0.284714	0.899303	1.15495	0.57479
XLOC_045558	QPCT	chr11	19836843	19837039	0.0	0.0	0.0	11.5213	11.4955	7.83172	15.922	7.0796	6.72978
XLOC_089350	QRSL1	chr9	43743891	43807564	9.45121	4.86289	6.76522	4.93614	3.67935	4.17471	5.46896	9.11572	3.184
XLOC_058172	QSOX1	chr16	62806292	62847039	16.6412	14.6745	12.0848	8.93125	8.39923	8.64349	10.0937	9.16229	10.6654
XLOC_082781	QTRT1	chr7	16399121	16429610	14.017	12.4475	12.6417	20.3198	19.6458	19.9339	20.9915	31.0006	14.7945
XLOC_084271	QTRT1	chr7	16399121	16429610	1.50598	1.80182	0.0	2.49741	1.70888	1.72012	1.57381	1.28217	1.38188
XLOC_084272	QTRT1	chr7	16399121	16429610	1.35214	1.31496	1.85191	1.2732	0.743186	1.23167	0.742956	1.32725	0.562126
XLOC_084273	QTRT1	chr7	16399121	16429610	0.0	0.924329	2.41707	1.38503	0.713793	1.90229	2.42806	0.603196	0.536815
XLOC_084274	QTRT1	chr7	16399121	16429610	0.0	2.0939	1.82527	1.67321	1.44474	0.240346	0.825264	1.83695	0.6092
XLOC_084275	QTRT1	chr7	16399121	16429610	1.20691	0.541578	2.83292	0.919726	0.756506	0.376317	0.659882	0.847769	0.422117
XLOC_084276	QTRT1	chr7	16399121	16429610	0.0	0.634548	1.65944	1.33104	2.30196	2.18712	2.82377	0.83699	1.66246
XLOC_084277	QTRT1	chr7	16399121	16429610	2.92216	0.872249	2.28091	1.82972	0.674364	0.89834	0.765948	2.28109	0.253396
XLOC_084278	QTRT1	chr7	16399121	16429610	0.0	1.89851	3.31017	1.70683	1.07389	1.75469	0.669328	1.58391	0.461857
XLOC_038377	QTRTD1	chr1	59158602	59237393	23.5708	33.9473	33.8908	18.9118	15.6735	17.6793	16.2796	22.8393	16.5702
XLOC_082986	R3HDM4	chr7	45053511	45077933	13.4928	18.4936	14.4758	40.796	32.511	36.8154	38.3018	46.0604	41.659
XLOC_083619	R3HDM4	chr7	45053511	45077933	6.31298	3.65483	5.60446	2.75875	4.27747	3.15307	3.35412	4.11435	2.01425
XLOC_044233	RAB10	chr11	73326564	73346097	0.800496	1.19713	1.41645	0.434444	0.125238	0.439074	0.164959	0.480796	0.316576

XLOC_041845	RAB11A;MIR2290	chr10	12649158	12869730	23.4437	18.9077	23.0784	14.8165	13.9733	13.3468	12.1513	10.5858	11.812
XLOC_045467	RAB11FIP5	chr11	11329914	11330462	9.26136	9.40842	11.5787	5.47289	5.60354	4.3935	5.93842	4.21023	5.96522
XLOC_072729	RAB19;MKRN1;SLC37A3	chr4	104367275	104744204	3.25359	3.35299	2.95701	7.70491	5.37614	5.28319	5.02503	4.08688	2.62794
XLOC_074797	RAB19;MKRN1;SLC37A3	chr4	104367275	104744204	0.0	0.37508	0.0	0.224805	0.0	0.389595	0.112958	0.0	0.0
XLOC_050887	RAB22A	chr13	58555450	58610886	0.0	0.0	0.0	0.114163	0.149957	0.033135	0.0291555	0.256385	0.027856
XLOC_051946	RAB22A	chr13	58553479	58553750	0.0	0.0	0.0	1.12963	0.0	0.0	0.0	1.78515	0.0
XLOC_051947	RAB22A	chr13	58554787	58555190	0.0	0.0	0.0	0.263512	0.0	0.0	0.0	0.0	0.255434
XLOC_051948	RAB22A	chr13	58555450	58610886	0.0	0.0	0.0	0.0	0.0	0.226591	0.389712	0.0	0.0
XLOC_079859	RAB28	chr6	113458724	113470360	0.0	0.32947	0.39701	0.936303	0.586562	0.993478	0.0671867	0.542628	0.180653
XLOC_079860	RAB28	chr6	113471483	113483141	0.0	0.511421	0.0	1.86138	2.47791	1.56238	1.85692	1.73934	2.35606
XLOC_079861	RAB28	chr6	113483293	113493433	7.71941	6.04291	6.91522	10.4364	15.504	11.8099	16.1545	11.2046	13.8799
XLOC_079862	RAB28	chr6	113514722	113596453	9.54945	12.1494	10.0264	27.4214	33.6759	28.3179	32.42	29.248	36.0466
XLOC_080262	RAB28	chr6	113493672	113501944	0.39167	0.52726	0.160292	1.00687	0.742547	0.917867	0.88112	1.29849	0.486794
XLOC_080263	RAB28	chr6	113514722	113596453	28.0602	23.8819	19.1538	14.9642	16.9443	14.4139	15.6273	15.7354	18.4975
XLOC_082195	RAB28	chr6	113493672	113501944	0.0	1.32746	0.0	1.98892	0.343739	0.914603	0.0	0.218633	0.0
XLOC_082196	RAB28	chr6	113514722	113596453	1.69194	2.0226	3.96685	1.66666	3.15388	1.39745	4.08019	1.0052	1.76823
XLOC_053230	RAB2A;CA8	chr14	27622508	28302918	3.41482	2.49578	1.28373	4.6651	6.55345	6.11617	8.93963	5.58156	5.97268
XLOC_054077	RAB2A;CA8	chr14	27622508	28302918	0.0	0.0	0.0	1.84463	2.26574	0.603648	3.60202	1.14951	1.27717
XLOC_059876	RAB35	chr17	64600788	64755149	27.6316	23.8559	29.7398	7.1811	6.65681	7.36444	7.32639	6.60061	6.76075
XLOC_064058	RAB37	chr19	57361219	57388832	15.6797	13.0327	15.5276	14.6917	15.9329	13.2867	16.468	17.7775	15.5595
XLOC_065390	RAB37	chr19	57359474	57360551	0.0	0.0	0.0	1.33959	0.430765	0.571591	0.2143	0.866275	0.0687296
XLOC_065391	RAB37	chr19	57361219	57388832	0.0	1.51608	0.0	0.908663	0.592825	0.787321	0.798827	0.504748	0.110584
XLOC_065392	RAB37	chr19	57361219	57388832	4.58527	1.36944	0.0	1.23109	1.94941	3.30116	1.62043	0.901489	1.79312
XLOC_065393	RAB37	chr19	57389091	57390121	3.31577	3.96683	7.13269	5.94398	6.93691	5.93621	5.56646	8.79287	6.66273
XLOC_084077	RAB3A	chr7	4943826	4944818	0.866693	0.0	1.35586	0.543762	0.271051	0.179858	0.078593	0.0	0.454251
XLOC_070031	RAB3B	chr3	94953275	94958528	4.72281	9.81456	8.63483	10.1951	8.21571	6.07089	6.44285	6.7558	7.4589
XLOC_070032	RAB3B	chr3	94974683	94976859	4.65007	7.28656	6.44979	1.96511	1.92231	1.86393	1.10697	1.3941	1.79625
XLOC_070033	RAB3B	chr3	94983657	95015372	6.11211	6.64296	6.79617	3.82886	3.35668	3.57643	3.47943	4.30918	3.14557
XLOC_070404	RAB3B	chr3	94983657	95015372	33.308	43.9494	38.565	15.9407	17.3682	18.1788	14.4157	21.8566	20.5712
XLOC_070405	RAB3B	chr3	94983657	95015372	9.01318	11.56	7.5216	4.73976	4.59098	4.95597	5.36204	5.5309	5.38417
XLOC_070406	RAB3B	chr3	94983657	95015372	9.08576	15.9176	20.9139	11.3007	9.50166	8.98488	9.82467	10.2446	7.34548

XLOC_071701	RAB3B	chr3	94944469	94944753	0.0	0.0	0.0	2.54121	4.7182	2.87186	0.0	2.15482	1.46645
XLOC_071702	RAB3B	chr3	94958713	94959626	8.61594	16.0322	8.23625	8.75114	9.72327	9.62988	6.23998	6.59971	10.0318
XLOC_071703	RAB3B	chr3	94960035	94960830	11.3454	9.49834	14.195	10.0642	12.3034	9.87211	7.57569	6.44715	8.11876
XLOC_071704	RAB3B	chr3	94977018	94977776	1.20445	4.32126	2.82537	1.72664	0.751322	0.872991	0.434171	0.4799	0.735585
XLOC_071705	RAB3B	chr3	94977993	94979091	3.84503	3.91024	2.40631	0.620386	0.661895	1.51698	1.32736	1.00114	0.806391
XLOC_071706	RAB3B	chr3	94979452	94979887	5.21299	1.55648	0.0	0.466415	0.200988	0.535149	0.0	0.510545	0.452525
XLOC_071707	RAB3B	chr3	94981454	94982059	4.84893	3.38132	3.78984	0.723768	0.628068	0.834654	0.722813	0.160193	0.844829
XLOC_071708	RAB3B	chr3	94982259	94982873	6.33776	2.36768	2.47682	1.27714	0.369505	1.30937	0.141795	0.628381	0.828267
XLOC_071709	RAB3B	chr3	94983657	95015372	15.5925	11.6276	0.0	3.50574	8.17153	5.49353	5.74572	3.64483	5.35997
XLOC_071710	RAB3B	chr3	94983657	95015372	0.0	5.45117	4.75068	1.09231	0.91929	1.84823	0.0	1.15427	2.62324
XLOC_071711	RAB3B	chr3	94983657	95015372	0.0	6.70489	4.78209	1.46123	1.26429	0.840795	1.44873	1.8111	1.59734
XLOC_071712	RAB3B	chr3	94983657	95015372	3.91512	5.26351	6.11778	1.22677	1.82064	1.00882	1.73999	2.12574	1.87359
XLOC_058694	RAB3GAP2	chr16	24379488	24380077	15.0862	13.0242	19.6507	1.65124	2.21356	1.03834	2.2464	0.996075	2.18992
XLOC_058695	RAB3GAP2	chr16	24380149	24380586	31.0542	35.5435	14.1443	3.2416	1.79622	3.98535	6.36823	4.56305	2.92056
XLOC_057846	RAB3GAP2;C16H1orf115;MARK1;MOSC2	chr16	24392573	24984240	21.8331	22.8455	32.7041	6.98779	5.29048	7.08665	5.41626	5.47887	6.00057
XLOC_058696	RAB3GAP2;C16H1orf115;MARK1;MOSC2	chr16	24392573	24984240	0.0	3.78865	0.0	0.0	0.322733	0.0	0.0	0.0	0.0
XLOC_058697	RAB3GAP2;C16H1orf115;MARK1;MOSC2	chr16	24392573	24984240	5.72024	3.20819	3.35629	1.60242	1.79113	1.85654	2.21075	0.859825	1.62485
XLOC_058698	RAB3GAP2;C16H1orf115;MARK1;MOSC2	chr16	24392573	24984240	2.48771	1.48743	1.94505	1.33725	1.4543	0.901298	1.12009	1.7337	1.51903
XLOC_058699	RAB3GAP2;C16H1orf115;MARK1;MOSC2	chr16	24392573	24984240	0.871974	1.56462	5.45609	0.859638	0.681683	0.814212	1.10683	0.610442	0.761635
XLOC_058700	RAB3GAP2;C16H1orf115;MARK1;MOSC2	chr16	24392573	24984240	8.54511	9.85397	13.3629	3.06239	2.28411	4.0443	2.41946	1.94514	3.51438

XLOC_058701	RAB3GAP2;C16H1orf115;MARK1;MOSC2	chr16	24392573	24984240	6.21612	6.50837	5.83618	2.06194	1.5583	1.48573	1.75539	2.18269	1.79349
XLOC_077811	RAB3IP	chr5	43910736	43911359	0.0	4.17954	2.42901	8.07155	8.21551	8.99009	9.32176	12.021	13.1329
XLOC_076317	RAB3IP;C5H12orf28	chr5	43868368	43910624	2.54452	1.51534	4.94505	13.1362	12.6675	10.4481	12.856	13.9955	14.9505
XLOC_038078	RAB6B	chr1	136576324	136625022	0.0	0.114282	0.298911	18.2703	10.0743	9.95319	17.0481	30.2551	12.3915
XLOC_038079	RAB6B	chr1	136625207	136656899	43.3683	53.9313	52.7978	46.384	38.0312	42.8737	50.0905	70.6282	41.8306
XLOC_040426	RAB6B	chr1	136573629	136574158	0.0	0.0	0.0	1.5688	2.11282	3.01013	2.42334	3.45953	1.18587
XLOC_040427	RAB6B	chr1	136576324	136625022	0.0	0.0	0.0	1.92677	0.935531	1.30299	1.90423	1.97703	0.730793
XLOC_040428	RAB6B	chr1	136576324	136625022	0.0	0.0	0.0	3.09507	2.42954	1.86933	2.05961	3.26102	2.29364
XLOC_040429	RAB6B	chr1	136576324	136625022	0.0	0.0	0.0	2.4942	1.78218	2.70683	1.02528	2.59723	1.71249
XLOC_040430	RAB6B	chr1	136576324	136625022	0.0	0.0	0.0	1.14237	1.10649	1.27799	1.41364	1.64672	0.796122
XLOC_040431	RAB6B	chr1	136576324	136625022	0.0	0.145958	0.0	1.00607	0.650406	1.11626	0.980127	1.71584	0.938831
XLOC_040432	RAB6B	chr1	136576324	136625022	0.0	0.0	0.0	1.06678	0.983065	1.30321	1.19194	1.6778	0.704135
XLOC_040433	RAB6B	chr1	136576324	136625022	0.0	0.0	0.0	1.94558	1.69031	1.49707	2.20848	3.01998	1.64089
XLOC_040434	RAB6B	chr1	136576324	136625022	0.0	0.0	0.0	2.19778	1.89611	1.00944	1.7309	3.13262	3.19976
XLOC_040435	RAB6B	chr1	136576324	136625022	0.0	0.0	0.0	1.23248	1.69778	2.54347	1.93154	5.65697	1.67343
XLOC_040436	RAB6B	chr1	136576324	136625022	0.0	0.0	0.0	1.10354	0.964891	1.27974	1.02536	1.98855	1.40408
XLOC_040437	RAB6B	chr1	136625207	136656899	5.34782	7.98319	10.4381	11.4829	8.24317	12.073	13.14	12.8217	11.371
XLOC_040438	RAB6B	chr1	136625207	136656899	3.10815	6.27759	3.34458	6.34083	5.70235	6.79281	9.31311	7.46624	7.6155
XLOC_040439	RAB6B	chr1	136625207	136656899	0.0	3.05988	0.0	3.36267	3.14388	2.09586	0.888467	1.32673	1.47973
XLOC_040440	RAB6B	chr1	136625207	136656899	0.0	2.11596	0.0	1.08705	1.02625	1.25751	1.91985	0.706505	1.67684
XLOC_040441	RAB6B	chr1	136625207	136656899	0.0	0.832364	0.0	1.24719	0.65038	1.00795	0.500145	1.24529	1.45664
XLOC_040442	RAB6B	chr1	136625207	136656899	0.0	0.388544	0.0	0.465747	0.607585	1.07596	0.818445	0.387934	1.24689
XLOC_058228	RAB7L1	chr16	3346379	3346735	0.0	0.0	0.0	2.60491	2.22905	1.48661	3.77171	4.93428	1.26014
XLOC_092596	RAB9A	chrX	137015177	137038288	0.0	1.99791	1.53357	2.66165	2.85894	2.91075	2.1739	2.16611	3.21936
XLOC_093652	RAB9A	chrX	137015177	137038288	2.33209	0.697669	1.36853	1.14996	1.87321	1.15144	0.74408	1.28743	1.32553
XLOC_093653	RAB9A	chrX	137015177	137038288	2.46446	2.20777	1.9245	2.86685	1.33172	2.53207	1.73654	1.20884	1.49826
XLOC_093654	RAB9A	chrX	137015177	137038288	1.76042	0.351098	0.459137	1.0522	1.56343	1.76882	1.28375	0.942343	0.76968
XLOC_093655	RAB9A	chrX	137015177	137038288	1.32834	0.794194	1.03853	1.30901	1.44861	2.19896	1.19435	1.05692	1.1584
XLOC_093656	RAB9A	chrX	137015177	137038288	0.0	0.996541	0.0	1.1947	1.79274	1.36565	1.4486	0.648661	1.44602
XLOC_063950	RABEP1	chr19	26775880	26778486	19.1797	12.2732	11.6727	0.620985	0.250603	0.553959	0.097226	0.321052	0.139788

XLOC_063949	RABEP1;NUP88;LOC788205;RPAIN;MIS12;DERL2;C1QBP;DHX33;MIR199C	chr19	26663607	26774234	26.9614	27.6261	29.8104	1.0292	0.550683	0.613247	0.783965	1.40289	0.608983
XLOC_045028	RABEPK	chr11	96097337	96112036	0.0	0.0	0.0	17.9974	10.6335	18.292	6.99041	23.489	6.64408
XLOC_047085	RABEPK	chr11	96096341	96096583	0.0	0.0	0.0	1.49502	1.86104	2.50445	0.0	0.773474	1.42632
XLOC_047086	RABEPK	chr11	96096768	96097233	0.0	0.0	0.0	0.842032	0.726976	1.6929	1.45321	2.3107	1.635
XLOC_047087	RABEPK	chr11	96097337	96112036	0.0	0.0	0.0	1.17109	2.41328	1.85678	0.876885	2.75344	0.142387
XLOC_044370	RABGAP1	chr11	94134341	94155595	2.12583	0.54922	2.71805	7.57363	12.8103	5.65459	3.47519	7.17332	7.07029
XLOC_044371	RABGAP1	chr11	94209824	94214350	11.0127	9.79726	13.4048	18.5072	22.9407	19.8665	29.8422	23.3307	20.6391
XLOC_045001	RABGAP1	chr11	94238836	94245970	0.0	1.38291	1.80838	0.0	0.0	0.0	0.0	0.0	0.302703
XLOC_047014	RABGAP1	chr11	94127953	94128711	1.20445	1.08032	0.0	0.539576	0.187831	0.0	0.434171	0.23995	0.525418
XLOC_047015	RABGAP1	chr11	94128773	94129475	1.32834	1.98548	0.0	0.237999	0.0	0.137435	0.47774	0.0	0.34752
XLOC_047016	RABGAP1	chr11	94134341	94155595	0.0	0.266879	0.0	0.55985	0.41853	1.01835	0.242646	0.624596	0.23383
XLOC_047017	RABGAP1	chr11	94158455	94159513	0.803167	0.0	0.0	0.503939	0.816764	0.750332	1.02085	0.241193	0.561405
XLOC_047018	RABGAP1	chr11	94160096	94160584	0.0	0.0	0.0	0.979447	1.86246	1.35143	1.54978	0.215412	0.761053
XLOC_047019	RABGAP1	chr11	94160688	94161737	0.0	0.0	0.634617	0.363588	1.07879	0.421038	0.441859	0.324805	0.567056
XLOC_047020	RABGAP1	chr11	94214476	94215617	5.88305	9.67842	14.9577	9.09697	12.5474	10.4622	15.9098	13.998	13.5607
XLOC_047021	RABGAP1	chr11	94215793	94216108	0.0	1.36563	0.0	0.819267	4.87589	1.39616	2.7272	0.878055	1.97632
XLOC_044369	RABGAP1;ZBTB6	chr11	93996265	94126228	52.0751	43.0529	45.7645	8.34399	9.90248	8.85377	9.83748	7.43504	11.2557
XLOC_071436	RABGGTB	chr3	69314910	69316749	5.16132	3.34597	4.03905	0.462812	0.303722	0.0895004	0.43253	0.129758	0.413947
XLOC_069935	RABGGTB;ACADM	chr3	69316906	69557393	9.96761	11.7093	13.5573	0.871723	1.14661	0.792273	1.1685	0.792349	0.90581
XLOC_088636	RAD23B	chr8	98700292	98701374	0.0	0.0	0.0	0.841486	0.489657	0.568514	0.28421	0.234991	0.410154
XLOC_041646	RAD51B	chr10	80249232	80256796	43.186	37.9238	40.8225	69.7553	67.925	74.9177	68.2915	66.6432	80.8828
XLOC_041647	RAD51B	chr10	80261216	80288404	39.4558	44.5195	36.6788	90.8521	85.1149	89.0771	92.333	100.212	80.2642
XLOC_041648	RAD51B	chr10	80292367	80295355	53.6244	52.8555	40.154	157.901	141.018	148.984	136.186	152.511	139.446
XLOC_041649	RAD51B	chr10	80301287	80307540	0.0	0.167793	0.0	0.502857	0.351678	0.58307	0.15345	0.450487	0.343339
XLOC_041650	RAD51B	chr10	80308958	80315297	0.836183	0.350357	0.261819	0.450004	0.223542	0.383427	0.0921856	0.118107	0.146461
XLOC_041651	RAD51B	chr10	80330148	80335690	3.53666	3.17369	2.76684	2.59971	3.54393	3.82024	2.70193	2.9064	1.66924
XLOC_041652	RAD51B	chr10	80356498	80365444	0.0	0.745973	0.0	3.72608	5.03716	3.95268	2.35657	2.89892	1.85148
XLOC_041653	RAD51B	chr10	80578125	80579532	1.43005	0.641637	1.11876	0.576869	0.391809	0.371308	0.260088	0.286608	0.249977

XLOC_041654	RAD51B	chr10	80602855	80615808	18.0226	19.7851	19.811	25.0742	20.8859	22.7407	21.3602	26.3431	17.4823
XLOC_041655	RAD51B	chr10	80622984	80640704	60.5879	63.8115	41.8105	72.1332	57.4044	67.1171	59.7959	86.1299	50.7147
XLOC_042043	RAD51B	chr10	80257618	80260956	8.30353	11.0959	10.3955	0.893371	0.824416	0.748162	0.252456	0.778164	0.67776
XLOC_042044	RAD51B	chr10	80317742	80320550	3.22949	4.18751	6.45907	6.85405	5.2679	5.15441	5.81416	8.37906	6.02946
XLOC_042045	RAD51B	chr10	80321132	80323751	0.0	0.549828	0.0	0.439404	0.858035	1.15905	0.279092	0.433596	0.397185
XLOC_042046	RAD51B	chr10	80380382	80387434	12.6636	20.1611	16.4538	7.31423	5.65174	6.99083	5.87745	8.67861	4.30173
XLOC_042047	RAD51B	chr10	80392514	80399251	14.0373	16.3934	12.8162	44.9851	34.9995	51.4455	39.797	49.4471	23.4117
XLOC_042048	RAD51B	chr10	80402987	80407006	99.3389	110.513	114.483	238.917	182.269	222.834	190.633	232.357	216.769
XLOC_042049	RAD51B	chr10	80409320	80433928	33.9936	49.4336	40.4293	44.9715	38.2797	40.4643	37.7294	53.6666	32.7552
XLOC_042050	RAD51B	chr10	80519559	80526325	2.01403	1.25134	0.944779	1.19267	2.18667	1.12775	1.05194	1.52449	1.85202
XLOC_042051	RAD51B	chr10	80540921	80561886	21.4022	22.1023	22.3029	19.4028	19.0085	18.9795	19.2816	27.856	17.1629
XLOC_042052	RAD51B	chr10	80565961	80577333	10.0002	8.79721	8.12989	9.81455	6.86499	8.62621	9.6605	11.1892	6.88915
XLOC_042053	RAD51B	chr10	80583577	80587861	18.3812	13.3464	15.484	33.5938	22.7837	26.2443	27.2936	33.8534	20.6493
XLOC_043056	RAD51B	chr10	80261216	80288404	1.49595	0.447252	0.0	0.535965	0.349115	1.08247	0.402277	0.594145	0.130756
XLOC_043057	RAD51B	chr10	80295477	80295931	0.0	0.0	0.0	0.654898	1.88357	0.501365	1.07483	0.718177	1.69513
XLOC_043058	RAD51B	chr10	80315380	80317598	0.70199	0.840222	0.824095	0.220333	0.303128	0.182684	0.0964069	0.211988	0.184311
XLOC_043059	RAD51B	chr10	80335759	80336594	2.1351	4.14983	6.67891	2.96555	5.33326	6.63791	4.43663	8.09527	3.26151
XLOC_043060	RAD51B	chr10	80390928	80391751	0.0	3.57465	0.0	1.46079	0.763397	0.788294	0.196306	0.650472	0.379439
XLOC_043061	RAD51B	chr10	80399672	80400200	0.0	1.74978	0.0	0.873903	0.302634	0.603631	1.04128	0.385405	0.169865
XLOC_043062	RAD51B	chr10	80587993	80589504	1.60304	2.5578	2.92678	2.68291	1.96883	2.22236	1.75519	2.84428	1.44874
XLOC_043063	RAD51B	chr10	80589616	80590147	5.80977	3.47149	13.6179	3.98773	5.2542	2.59497	3.27182	5.54454	2.35914
XLOC_043064	RAD51B	chr10	80602855	80615808	0.0	3.35969	2.92834	3.69275	2.58345	4.59548	1.6173	1.08886	1.94817
XLOC_043065	RAD51B	chr10	80622984	80640704	0.975403	1.45845	2.28873	1.13638	0.609565	1.0114	0.706186	0.38973	0.596197
XLOC_043066	RAD51B	chr10	80622984	80640704	1.9393	1.24358	3.03577	0.844773	0.652921	0.923268	0.762001	0.502429	0.873104
XLOC_043067	RAD51B	chr10	80622984	80640704	1.40915	1.8968	2.20493	1.20005	0.496435	0.585449	0.44859	0.494298	0.800585
XLOC_076447	RAD52	chr5	108206525	108232837	1.49354	4.47344	5.85016	6.74308	10.5549	7.96399	11.2114	5.31107	17.2975
XLOC_089548	RAET1G;PPP1R14C;ULBP17	chr9	88376705	88482238	2.20736	3.07224	1.99115	1.72385	1.83938	2.27232	3.65371	1.94151	3.1846
XLOC_089549	RAET1G;PPP1R14C;ULBP17	chr9	88376705	88482238	12.6542	7.01977	6.72582	10.4483	8.64562	9.69608	8.02204	10.1244	10.2046
XLOC_090024	RAET1G;PPP1R14C;ULBP17	chr9	88376705	88482238	62.7609	63.9255	69.8365	60.0064	58.066	61.4564	79.623	80.2464	67.7028

XLOC_091423	RAET1G;PPP1R14C;ULBP17	chr9	88376705	88482238	14.4938	3.46113	2.26269	1.0372	2.67631	3.56487	2.78712	1.69758	3.51967
XLOC_091424	RAET1G;PPP1R14C;ULBP17	chr9	88376705	88482238	18.1375	14.4184	9.4242	13.0002	6.38432	6.722	9.07742	9.15312	13.5307
XLOC_091425	RAET1G;PPP1R14C;ULBP17	chr9	88376705	88482238	3.48347	5.99047	4.76833	2.41968	2.99582	4.60835	4.50103	2.17755	3.95584
XLOC_091426	RAET1G;PPP1R14C;ULBP17	chr9	88376705	88482238	1.53078	3.20266	4.78616	1.50817	2.14287	2.84727	2.74254	2.27811	2.93486
XLOC_091427	RAET1G;PPP1R14C;ULBP17	chr9	88376705	88482238	10.1594	9.09416	7.92669	2.72562	4.41329	8.30662	10.8613	3.94442	7.91709
XLOC_091428	RAET1G;PPP1R14C;ULBP17	chr9	88376705	88482238	7.48391	6.39378	3.34439	3.54475	4.42309	4.32099	4.926	2.56013	5.41286
XLOC_091429	RAET1G;PPP1R14C;ULBP17	chr9	88376705	88482238	0.0	1.73057	4.52538	3.3709	1.56119	2.97073	2.027	1.41465	3.26829
XLOC_091430	RAET1G;PPP1R14C;ULBP17	chr9	88376705	88482238	0.0	6.7686	0.0	4.07968	6.23536	3.809	5.58726	4.24838	5.20216
XLOC_091431	RAET1G;PPP1R14C;ULBP17	chr9	88376705	88482238	0.0	3.22988	0.0	10.7783	10.4182	10.8332	11.2437	7.95201	4.64026
XLOC_091432	RAET1G;PPP1R14C;ULBP17	chr9	88376705	88482238	0.0	9.89761	12.9428	3.9548	5.16627	5.94464	7.27225	5.61223	7.60792
XLOC_091433	RAET1G;PPP1R14C;ULBP17	chr9	88376705	88482238	27.5475	16.4485	14.3327	22.3791	9.57913	13.8339	8.91899	16.1767	15.7739
XLOC_091434	RAET1G;PPP1R14C;ULBP17	chr9	88376705	88482238	15.4442	7.17914	8.04644	2.76602	3.99771	3.71953	3.67685	3.39739	4.18443
XLOC_091435	RAET1G;PPP1R14C;ULBP17	chr9	88376705	88482238	5.8271	6.0961	4.55513	4.69756	4.42105	5.72293	5.09631	5.35251	6.60316
XLOC_091436	RAET1G;PPP1R14C;ULBP17	chr9	88376705	88482238	5.75752	6.8839	5.62602	3.86796	3.13662	3.12525	2.45395	2.8593	5.14452
XLOC_091437	RAET1G;PPP1R14C;ULBP17	chr9	88376705	88482238	8.59307	5.13023	4.47182	3.84344	3.52699	3.22966	2.00414	2.51715	5.71416

XLOC_091438	RAET1G;PPP1R14C;ULBP17	chr9	88376705	88482238	3.78349	10.9341	10.8468	5.31051	4.91463	4.82996	5.67675	4.51933	5.61057
XLOC_091439	RAET1G;PPP1R14C;ULBP17	chr9	88376705	88482238	10.8522	3.23507	16.9167	5.82828	6.97992	5.49545	8.21068	6.70903	6.54531
XLOC_091440	RAET1G;PPP1R14C;ULBP17	chr9	88376705	88482238	4.28639	7.68213	8.37078	4.02853	3.64897	7.28071	6.07553	5.48755	3.91352
XLOC_089547	RAET1G;ULBP17	chr9	88327624	88370659	34.2698	34.7295	40.2362	0.525033	0.287248	0.533206	0.0669842	1.54823	0.657894
XLOC_091422	RAET1G;ULBP17	chr9	88370958	88372214	22.3853	29.3456	26.2705	0.295117	0.257815	0.410424	0.479481	1.12239	0.575565
XLOC_065693	RALB;TMEM185B	chr2	72297718	72442257	3.08668	4.31809	3.40871	6.62438	8.59477	6.30038	5.69667	4.81612	6.43152
XLOC_067862	RALB;TMEM185B	chr2	72297718	72442257	2.87716	5.37886	5.06446	5.93205	6.41875	5.97622	4.0551	2.81096	4.52637
XLOC_067863	RALB;TMEM185B	chr2	72297718	72442257	5.05023	4.26707	4.88271	5.11526	5.95009	5.84655	3.72998	2.5734	4.96683
XLOC_067864	RALB;TMEM185B	chr2	72297718	72442257	5.79596	4.20994	7.77221	5.34346	5.76339	5.92945	4.88394	3.06573	4.99135
XLOC_044487	RALGDS	chr11	103131319	103139223	5.50416	5.25328	4.24927	5.72549	6.01094	6.00393	5.04495	6.1996	6.6324
XLOC_047347	RALGDS	chr11	103128550	103129092	0.0	2.81029	0.0	0.505285	1.02122	0.969841	0.66977	1.11507	2.12837
XLOC_044416	RALGPS1	chr11	97662762	97683046	55.6754	65.4923	76.2744	79.9535	58.4296	54.6022	59.8414	99.5924	50.0896
XLOC_044417	RALGPS1	chr11	97684090	97688746	14.4345	13.974	18.1409	25.6733	21.6839	20.1522	26.2294	41.1578	23.6014
XLOC_044418	RALGPS1	chr11	97702667	97713564	10.9923	19.4922	15.4248	38.209	27.798	32.4785	33.1982	43.1201	35.337
XLOC_044419	RALGPS1	chr11	97798997	97836581	30.0819	21.1659	31.2911	80.6759	59.5033	70.5479	62.9907	111.46	53.9222
XLOC_045045	RALGPS1	chr11	97689870	97699562	5.18831	9.70666	6.75326	25.4561	26.1497	26.5291	27.8647	38.504	20.5893
XLOC_045046	RALGPS1	chr11	97714970	97744728	11.8857	28.5678	29.4999	31.142	23.0283	28.3343	34.5296	38.8514	17.8351
XLOC_045047	RALGPS1	chr11	97746488	97754189	11.1713	12.1666	6.64399	10.6982	9.39496	10.0877	13.6401	14.6462	11.22
XLOC_045048	RALGPS1	chr11	97756107	97766590	10.8867	11.7971	11.7028	11.3373	12.0812	10.6984	16.2637	19.8881	12.4592
XLOC_045049	RALGPS1	chr11	97771593	97775910	0.0	0.625383	0.0	0.374804	1.29668	2.15584	1.67067	2.88814	1.82064
XLOC_045050	RALGPS1	chr11	97777805	97782495	16.5815	24.9366	20.7426	55.1843	55.4915	58.8064	67.0616	73.1648	56.4516
XLOC_045052	RALGPS1	chr11	97923655	97986763	1.49211	1.19264	1.55725	4.39323	5.67814	5.25452	4.47492	3.3109	3.48471
XLOC_047170	RALGPS1	chr11	97662762	97683046	0.0	6.76861	5.89835	19.7184	6.23533	5.33252	6.20807	9.91358	5.20136
XLOC_047171	RALGPS1	chr11	97662762	97683046	0.0	4.14768	7.23112	8.2859	5.72445	6.6648	3.67952	7.96187	6.63736
XLOC_047172	RALGPS1	chr11	97662762	97683046	3.12386	4.6614	14.627	15.9253	9.83721	12.4692	15.7752	17.9425	13.5348
XLOC_047173	RALGPS1	chr11	97683107	97683640	15.4102	15.5387	18.0606	20.5225	15.0826	12.9066	17.1316	30.2412	16.259

XLOC_047174	RALGPS1	chr11	97688820	97689716	1.95873	1.75724	2.29791	7.98694	8.41529	6.60066	10.0147	13.8909	6.92678
XLOC_047175	RALGPS1	chr11	97689870	97699562	3.6437	0.0	0.949688	2.82932	0.852266	2.01204	1.96981	2.29838	1.90732
XLOC_047176	RALGPS1	chr11	97689870	97699562	1.53729	1.37839	1.20162	2.06532	2.51051	1.90617	1.92771	3.05015	1.60751
XLOC_047177	RALGPS1	chr11	97689870	97699562	0.81955	1.47072	0.641084	3.08526	2.30764	2.21162	3.0497	2.70669	2.36289
XLOC_047178	RALGPS1	chr11	97689870	97699562	6.35475	3.78898	0.0	8.54511	7.17932	8.9845	2.11262	6.5985	7.65475
XLOC_047179	RALGPS1	chr11	97699752	97702422	0.863548	1.1198	0.675881	1.08423	0.881828	0.92924	0.633251	1.04391	0.680343
XLOC_047180	RALGPS1	chr11	97702667	97713564	6.3012	9.3925	9.82255	1.69445	2.37353	2.54574	1.57208	2.97526	7.59071
XLOC_047181	RALGPS1	chr11	97702667	97713564	0.0	7.34235	19.1923	10.0537	9.97144	4.91384	7.78974	5.6081	9.48033
XLOC_047182	RALGPS1	chr11	97702667	97713564	0.0	0.0	12.3251	2.35856	1.59673	4.8071	4.88424	3.01205	2.72542
XLOC_047183	RALGPS1	chr11	97702667	97713564	5.84431	3.489	2.2809	3.39806	2.92221	3.59314	2.55316	3.70677	2.28053
XLOC_047184	RALGPS1	chr11	97713693	97714245	1.83349	1.09567	1.43269	0.656666	0.568979	0.378213	0.163339	0.724954	0.159598
XLOC_047185	RALGPS1	chr11	97745006	97745755	2.44556	5.1181	1.9122	1.42422	0.476652	0.632982	0.771101	1.09596	1.06676
XLOC_047186	RALGPS1	chr11	97745907	97746283	9.92759	13.8245	7.74634	5.91892	5.33031	7.10542	8.90391	9.32293	7.73799
XLOC_047187	RALGPS1	chr11	97776133	97776442	0.0	1.4188	3.70974	3.83096	1.80733	1.9327	2.01872	1.82147	4.10517
XLOC_047188	RALGPS1	chr11	97776819	97777159	0.0	3.54211	3.08732	3.89375	5.74261	5.64746	2.03972	3.05486	3.76389
XLOC_047189	RALGPS1	chr11	97777335	97777667	4.1386	3.70279	3.22735	5.55124	9.46811	6.3205	4.96171	7.17251	4.29068
XLOC_047190	RALGPS1	chr11	97777805	97782495	14.7403	4.40312	9.86984	19.2261	21.3547	18.6468	27.4405	30.2901	29.6649
XLOC_047191	RALGPS1	chr11	97777805	97782495	3.68774	5.23858	4.32658	25.4491	27.3069	24.4808	27.3101	28.0142	26.1684
XLOC_047192	RALGPS1	chr11	97798782	97798906	0.0	76.1976	0.0	0.0	16.6272	0.0	0.0	0.0	42.6535
XLOC_047196	RALGPS1	chr11	97923037	97923565	1.95227	0.0	0.0	2.44693	5.44741	3.62179	2.6032	2.11973	3.05756
XLOC_047197	RALGPS1	chr11	97923655	97986763	0.0	1.13294	0.0	1.358	2.79337	1.56381	1.51843	2.99637	2.145
XLOC_047198	RALGPS1	chr11	97923655	97986763	0.0	0.0	0.0	1.26666	1.2108	0.29245	1.39636	1.26443	1.23276
XLOC_052219	RALY	chr13	63955455	63956109	0.0	0.0	0.0	0.260976	0.79352	0.602413	2.22147	0.723312	0.888866
XLOC_052220	RALY	chr13	63956888	63957490	0.0	0.0	0.0	2.47739	1.64386	1.34437	3.0557	1.61253	2.12624
XLOC_052221	RALY	chr13	63957899	63958212	0.0	0.0	0.0	2.9039	3.52567	1.88468	4.33624	0.888686	1.60089
XLOC_052222	RALY	chr13	63960219	63960485	0.0	0.0	0.0	1.76982	4.94927	1.99169	3.27198	2.47958	2.26332
XLOC_052223	RALY	chr13	63961563	63962280	0.0	0.0	0.0	3.12696	4.02907	4.28095	3.95449	2.95846	5.07369
XLOC_052224	RALY	chr13	63963131	63963472	0.0	0.0	0.0	4.22474	5.71237	6.41999	3.72051	3.79878	3.74375
XLOC_052225	RALY	chr13	63963523	63964666	0.0	0.0	0.0	4.47348	5.39943	4.11544	5.87085	3.82333	6.34974
XLOC_052226	RALY	chr13	63965127	63965308	0.0	0.0	0.0	25.6208	27.6896	19.0089	27.6344	28.2763	32.7474
XLOC_052227	RALY	chr13	63965650	63965847	0.0	0.0	0.0	26.8598	31.6351	12.3105	34.9281	13.9207	31.7306
XLOC_052228	RALY	chr13	63978903	63981206	0.0	0.0	0.0	0.816197	1.37635	0.736939	0.740892	0.712683	1.12111
XLOC_054938	RALYL	chr14	80312480	80313089	0.0	0.478772	0.0	4.59114	2.36578	2.9784	3.43966	2.85842	2.51216

XLOC_065184	RAMP2	chr19	43441564	43442356	1.13991	1.02249	0.0	1.3278	0.711428	0.236154	0.822785	0.454541	0.198951
XLOC_059902	RANBP1;RANBP1;HSFY2	chr17	75005847	75391486	11.2513	7.6287	10.176	2.49332	2.90618	2.55559	3.06835	2.09448	3.5802
XLOC_044035	RANBP2	chr11	44518678	44565566	20.3432	21.7817	13.4441	17.1249	14.6734	16.1798	17.2659	14.2732	14.2696
XLOC_083443	RANBP3;CAPS;NDUFA11;VMAC	chr7	19633241	19716057	363.047	153.615	180.269	655.822	557.829	611.284	597.398	721.336	686.366
XLOC_083444	RANBP3;CAPS;NDUFA11;VMAC	chr7	19633241	19716057	6.15594	1.33157	3.48216	0.0	0.517171	0.917385	1.38013	0.877155	1.74378
XLOC_083445	RANBP3;CAPS;NDUFA11;VMAC	chr7	19633241	19716057	4.3378	5.22459	2.30872	136.678	134.334	134.019	104.483	108.951	168.396
XLOC_084324	RANBP3;CAPS;NDUFA11;VMAC	chr7	19633241	19716057	4.43283	2.20829	3.46513	0.794227	1.14962	0.305476	1.8549	0.586776	1.0303
XLOC_084325	RANBP3;CAPS;NDUFA11;VMAC	chr7	19633241	19716057	0.0	0.0	0.0	0.477003	0.0693305	0.0	0.160788	0.354747	0.232401
XLOC_070765	RAP1A	chr3	31774573	31774847	49.1646	43.9692	62.2684	18.724	21.3128	20.4926	13.3123	10.4589	19.5694
XLOC_044471	RAPGEF1	chr11	101732915	101738164	29.0815	50.8293	36.2902	69.5682	50.4173	64.8941	67.221	83.7574	59.5693
XLOC_044472	RAPGEF1	chr11	101738287	101766995	31.9772	34.5355	32.0852	38.1555	30.0494	32.8401	39.5761	49.6809	34.807
XLOC_045101	RAPGEF1	chr11	101769896	101786446	15.0987	15.7971	11.6971	44.6282	34.9009	38.7787	43.1111	72.8565	32.794
XLOC_047305	RAPGEF1	chr11	101767131	101768298	9.31372	8.14349	10.0888	9.50508	6.16913	7.21758	6.77551	9.18948	6.95058
XLOC_047306	RAPGEF1	chr11	101788277	101788673	0.0	0.0	0.0	3.25463	0.0	0.0	1.05773	0.886515	0.262844
XLOC_061418	RAPGEF2	chr17	40485436	40486644	0.0	0.0	0.0	3.82655	2.91093	2.86065	1.31542	2.48452	1.26378
XLOC_061419	RAPGEF2	chr17	40486746	40488209	0.0	0.0	0.0	3.97358	2.30144	3.68607	2.17277	2.39189	2.08328
XLOC_061420	RAPGEF2	chr17	40488472	40490222	0.0	0.0	0.0	2.80983	2.74321	3.40158	1.90899	2.55676	1.82764
XLOC_061421	RAPGEF2	chr17	40490597	40491769	0.0	0.0	0.0	5.3682	3.51591	5.10896	3.88994	2.7148	2.4924
XLOC_061422	RAPGEF2	chr17	40491918	40492508	0.0	0.0	0.0	4.49298	3.24781	2.93524	1.34482	1.822	1.74791
XLOC_061423	RAPGEF2	chr17	40493189	40493409	0.0	0.0	0.0	13.7179	10.4181	12.9997	5.1894	7.95201	8.35041
XLOC_077624	RAPGEF3	chr5	32712675	32714246	1.02332	0.765399	0.800745	0.779901	0.842503	0.425665	1.21434	0.616824	0.671302
XLOC_064259	RARA	chr19	41213721	41263878	106.263	99.1071	110.571	57.0578	57.5096	56.7059	67.6757	65.961	66.4612
XLOC_065142	RARA	chr19	41209308	41212671	5.64045	3.37598	4.06175	1.96283	2.3051	2.74998	2.44321	1.79693	2.9237
XLOC_065143	RARA	chr19	41212845	41213512	17.0352	9.75982	8.87813	7.75694	5.74601	5.13779	5.2245	5.35932	5.1978
XLOC_065144	RARA	chr19	41213721	41263878	2.31464	0.0	3.61556	2.69292	1.61	2.14226	1.83976	1.81981	2.41359
XLOC_065145	RARA	chr19	41213721	41263878	3.69692	2.20569	0.0	0.661165	1.69665	1.13166	0.0	0.357614	1.27917

XLOC_083746	RASA1	chr7	89139616	89338963	5.48012	6.45449	6.42957	8.10871	7.83919	7.97119	8.84627	8.1331	8.34496
XLOC_038524	RASA2	chr1	128331488	128417391	77.5157	85.1958	70.1316	320.576	303.379	309.523	318.196	370.949	361.75
XLOC_086496	RASEF	chr8	77549403	77581530	9.49527	10.0449	7.71866	14.7434	17.6029	16.2399	15.3696	16.2119	18.9366
XLOC_086949	RASEF	chr8	77595982	77597921	19.1853	24.5439	24.5282	19.9956	25.3934	24.1097	19.5259	22.015	20.6117
XLOC_086950	RASEF	chr8	77601483	77610805	6.4053	8.61885	8.34862	9.21493	11.9395	10.9907	11.0091	11.0859	9.41716
XLOC_088205	RASEF	chr8	77518492	77519284	0.0	0.681657	0.0	0.102139	0.0	0.118077	0.411392	0.0	0.0994755
XLOC_088206	RASEF	chr8	77520005	77520507	2.09964	1.88158	0.0	0.37589	0.0	0.216202	0.372297	0.0	0.182589
XLOC_088207	RASEF	chr8	77521274	77521823	0.0	1.10405	0.0	0.165422	0.0	0.0	0.164549	0.0	0.0
XLOC_088208	RASEF	chr8	77522162	77522599	0.0	0.0	0.0	0.231543	0.199579	0.0	0.0	0.0	0.0
XLOC_088209	RASEF	chr8	77523612	77524024	0.0	0.0	0.0	0.508443	0.0	0.0	0.0	0.0	0.0
XLOC_088210	RASEF	chr8	77524099	77524973	0.0	0.603716	0.0	0.0	0.0788328	0.0	0.0	0.10079	0.0
XLOC_086948	RASEF;DCTN3;C8H9orf23;GALT;SIGMAR1;CCL27;CCL21;CCL19;IL11RA	chr8	77250381	77517964	13.7798	12.2148	14.6913	7.09701	6.61335	7.45025	6.23415	5.79759	4.99327
XLOC_079841	RASGEF1B	chr6	97807134	98021439	1.71205	1.24732	0.981575	11.9366	10.4443	10.1365	13.6901	12.3804	20.2146
XLOC_081943	RASGEF1B	chr6	97807134	98021439	1.83918	0.0	0.0	0.0824177	0.0	0.0	0.0	0.0	0.0803139
XLOC_081944	RASGEF1B	chr6	97807134	98021439	0.267118	0.159872	0.0	0.0718692	0.0839439	0.0556408	0.0489891	0.0538321	0.116924
XLOC_081945	RASGEF1B	chr6	97807134	98021439	0.0	0.719014	0.940226	0.0	0.0	0.0	0.0	0.0	0.0
XLOC_081946	RASGEF1B	chr6	97807134	98021439	0.303047	0.453424	0.237185	0.0815333	0.0714056	0.094666	0.0277706	0.0	0.0795804
XLOC_081947	RASGEF1B	chr6	97807134	98021439	0.0	2.50385	0.0	0.0	0.626883	0.0	0.683526	0.0	0.0
XLOC_081948	RASGEF1B	chr6	97807134	98021439	0.0	0.511584	1.33791	0.0	0.0	0.0	0.152851	0.0	0.0
XLOC_081949	RASGEF1B	chr6	97807134	98021439	0.0	0.360705	0.0	0.108096	0.0	0.0	0.0	0.240345	0.0
XLOC_081950	RASGEF1B	chr6	97807134	98021439	0.0	0.217533	0.568937	0.195575	0.0	0.0	0.0661186	0.0728661	0.0
XLOC_081951	RASGEF1B	chr6	97807134	98021439	0.0	0.389242	0.0	0.116647	0.0	0.0	0.117126	0.129541	0.113557
XLOC_081952	RASGEF1B	chr6	97807134	98021439	0.0	1.0454	0.0	0.0	0.0	0.0	0.156072	0.0	0.304621
XLOC_081953	RASGEF1B	chr6	97807134	98021439	0.639189	0.764838	0.0	0.0573036	0.0	0.0	0.0	0.0	0.0
XLOC_081954	RASGEF1B	chr6	97807134	98021439	3.89501	1.16179	3.03786	0.0	0.0	0.0	0.0	0.0	0.0
XLOC_081955	RASGEF1B	chr6	97807134	98021439	0.0	4.16403	0.0	2.54241	0.0	0.0	0.0	0.0	0.0
XLOC_081956	RASGEF1B	chr6	97807134	98021439	0.0	1.4022	0.0	0.280133	0.0	0.161572	0.0	0.0	0.0
XLOC_081957	RASGEF1B	chr6	97807134	98021439	3.6782	1.09728	2.8692	0.0	0.0	0.750699	0.0	0.711752	0.0
XLOC_081958	RASGEF1B	chr6	97807134	98021439	1.42509	0.142125	0.0	0.127781	0.0745157	0.0494108	0.0	0.0	0.0

XLOC_081959	RASGEF1B	chr6	97807134	98021439	0.0	0.0	0.0	0.0	0.0	0.0	0.0	0.0	0.707167
XLOC_081960	RASGEF1B	chr6	97807134	98021439	0.0	0.8622	1.12744	0.12919	0.0	0.0	0.0	0.0	0.0
XLOC_081961	RASGEF1B	chr6	97807134	98021439	2.29792	1.0306	0.0	0.0	0.0	0.11901	0.0	0.114525	0.0
XLOC_081962	RASGEF1B	chr6	97807134	98021439	0.0	0.79623	1.19001	0.102268	0.0298387	0.158257	0.0695712	0.0	0.0
XLOC_081963	RASGEF1B	chr6	97807134	98021439	1.04443	0.936929	0.816798	0.0	0.0	0.0	0.0943994	0.0	0.0
XLOC_081964	RASGEF1B	chr6	97807134	98021439	1.29969	0.0	0.0	0.0	0.0	0.0	0.233841	0.0	0.0
XLOC_081965	RASGEF1B	chr6	97807134	98021439	0.0	0.861841	0.0	0.0	0.222155	0.0	0.0	0.0	0.0
XLOC_081966	RASGEF1B	chr6	97807134	98021439	2.32955	0.0	1.8194	0.0	0.0	0.0	0.0	0.0	0.0
XLOC_081967	RASGEF1B	chr6	97807134	98021439	1.38693	0.0	4.33706	0.124242	0.107986	0.0	0.124569	0.0	0.0
XLOC_081968	RASGEF1B	chr6	97807134	98021439	0.0	1.1495	0.0	0.344604	0.0	0.0	0.0	0.0	0.0
XLOC_081969	RASGEF1B	chr6	97807134	98021439	0.547999	0.491968	0.214457	0.0245736	0.107627	0.0856075	0.0251224	0.0	0.0479733
XLOC_081971	RASGEF1B	chr6	97807134	98021439	0.0	0.0	0.0	0.0	0.0	0.0	0.486409	0.0	0.0
XLOC_081972	RASGEF1B	chr6	97807134	98021439	0.0	0.0	0.0	0.0	0.105976	0.140771	0.0	0.270587	0.0
XLOC_081973	RASGEF1B	chr6	97807134	98021439	2.4843	0.557382	0.0	0.167041	0.0	0.064543	0.226309	0.0	0.0543017
XLOC_081974	RASGEF1B	chr6	97807134	98021439	0.0	0.389242	1.01799	0.0	0.101445	0.134738	0.0	0.0	0.113557
XLOC_081975	RASGEF1B	chr6	97807134	98021439	0.0	1.79326	0.0	0.0	0.155025	0.0	0.0	0.0	0.174064
XLOC_081976	RASGEF1B	chr6	97807134	98021439	0.0	0.0	1.26047	0.0	0.0	0.0	0.288499	0.0	0.0
XLOC_081977	RASGEF1B	chr6	97807134	98021439	0.0	0.0	0.0	0.0	0.134089	0.0	0.155535	0.0	0.0749033
XLOC_081979	RASGEF1B	chr6	97807134	98021439	0.0	0.0	0.831187	0.0	0.0	0.0	0.0	0.0	0.0
XLOC_081980	RASGEF1B	chr6	97807134	98021439	0.0	0.0	0.0	0.110489	0.0	0.0	0.0	0.0	0.0
XLOC_081981	RASGEF1B	chr6	97807134	98021439	1.16002	0.0	0.0	0.0	0.0	0.0	0.0	0.0	0.101223
XLOC_081982	RASGEF1B	chr6	97807134	98021439	0.0	0.0	0.0	0.0	0.839119	0.0	0.0	0.0	0.0
XLOC_081983	RASGEF1B	chr6	97807134	98021439	0.0	0.0	1.40427	0.160911	0.0	0.185392	0.320397	0.177714	0.0
XLOC_081984	RASGEF1B	chr6	97807134	98021439	0.0	0.328956	0.430183	0.0985851	0.0	0.0571595	0.100299	0.0	0.0
XLOC_081985	RASGEF1B	chr6	97807134	98021439	0.0	0.0	0.0	0.0	0.0	0.0	0.0	0.120768	0.105785
XLOC_081986	RASGEF1B	chr6	97807134	98021439	0.0	1.11416	0.0	0.0	0.0	0.0	0.0	0.0	0.0
XLOC_081988	RASGEF1B	chr6	97807134	98021439	0.0	0.0	0.0	0.0	0.0	0.194974	0.0	0.0	0.0
XLOC_081989	RASGEF1B	chr6	97807134	98021439	0.0	0.407563	1.06589	0.0	0.0	0.0	0.0	0.0	0.0
XLOC_081990	RASGEF1B	chr6	97807134	98021439	0.642596	0.0	0.0	0.0576089	0.0	0.0	0.0	0.0	0.056179
XLOC_081993	RASGEF1B	chr6	97807134	98021439	0.0	0.0	0.0	0.0	0.142959	0.0	0.0	0.0	0.0
XLOC_081994	RASGEF1B	chr6	97807134	98021439	0.0	0.0	0.0	0.0	0.0	0.0	0.459284	0.0	0.0
XLOC_081996	RASGEF1B	chr6	97807134	98021439	0.0	0.0	0.0	0.0	0.0	0.43365	0.0	0.0	0.183116
XLOC_081997	RASGEF1B	chr6	97807134	98021439	0.0	0.0	0.0	0.431288	0.0	0.0	0.0	0.0	0.0

XLOC_081998	RASGEF1B	chr6	97807134	98021439	0.0	0.0	1.18651	0.0	0.0	0.313734	0.0	0.150623	0.0
XLOC_041903	RASGRP1	chr10	34151336	34208232	5.69426	3.52439	2.45799	38.2469	34.7568	36.2691	42.1658	46.2156	42.5165
XLOC_041904	RASGRP1	chr10	34230919	34232954	0.0	0.0	0.0	48.2485	27.8853	28.9511	20.3062	40.1127	34.6615
XLOC_042566	RASGRP1	chr10	34228721	34229522	0.0	0.0	0.0	0.805695	0.613871	1.39724	0.507197	1.12068	0.784719
XLOC_048556	RB1	chr12	18216542	18217740	17.3774	21.6239	26.6463	21.4351	31.51	26.4254	23.3962	17.7664	38.7021
XLOC_048557	RB1	chr12	18227991	18228851	0.0	0.0	0.0	0.738095	0.160782	0.426879	0.465406	0.822189	1.25835
XLOC_092575	RBBP7	chrX	133891067	133898458	10.6065	6.08251	8.99181	2.73431	3.12044	2.98853	3.595	3.33267	3.05451
XLOC_092576	RBBP7	chrX	133937833	133950317	5.26501	5.90231	2.05817	0.117918	0.102541	0.0	0.0	0.261865	0.0
XLOC_093575	RBBP7	chrX	133936335	133937759	2.28421	5.97934	4.91499	0.0511984	0.0895089	0.0	0.0	0.0573264	0.0
XLOC_076883	RBFOX2	chr5	74205424	74531773	4.536	7.58505	3.19754	10.1182	6.27432	7.81345	7.27487	9.38523	7.13462
XLOC_076371	RBFOX2;LOC515697	chr5	74535420	74704182	54.4598	74.825	50.7003	62.0647	65.7315	59.1978	68.7139	69.3959	68.5545
XLOC_076884	RBFOX2;LOC515697	chr5	74535420	74704182	0.222533	0.298062	1.07494	3.44346	3.75017	3.99548	1.19344	1.77676	2.52632
XLOC_078135	RBFOX2;LOC515697	chr5	74535420	74704182	1.84625	1.10427	2.88807	2.15104	1.51496	2.77673	2.59228	1.01495	2.57993
XLOC_078136	RBFOX2;LOC515697	chr5	74535420	74704182	2.88454	2.1555	5.63722	2.06702	4.26523	4.17522	1.55287	1.86219	2.89171
XLOC_078137	RBFOX2;LOC515697	chr5	74535420	74704182	1.00068	2.69447	4.69816	2.24307	2.23637	2.91389	3.3804	2.21193	3.58873
XLOC_078138	RBFOX2;LOC515697	chr5	74535420	74704182	1.69595	0.506793	0.0	0.911216	1.44887	1.75065	0.302923	0.167919	0.738623
XLOC_078139	RBFOX2;LOC515697	chr5	74535420	74704182	0.606508	0.362868	0.474528	0.326242	0.760292	0.819417	0.276319	0.365144	0.530346
XLOC_078140	RBFOX2;LOC515697	chr5	74535420	74704182	0.0	0.0	0.0	0.580829	1.18047	0.44778	0.390312	0.107773	0.0943867
XLOC_078141	RBFOX2;LOC515697	chr5	74535420	74704182	0.0	0.0	0.0	0.0	1.24765	0.127452	0.0	0.0	0.3223
XLOC_064045	RBFOX3	chr19	53600397	53634831	67.6937	72.0532	64.424	82.7655	84.8588	87.6535	102.961	102.873	102.546
XLOC_064046	RBFOX3	chr19	53635369	53645388	52.3915	62.6159	41.9926	68.2411	73.3918	72.7867	85.0106	86.4827	87.7656
XLOC_064047	RBFOX3	chr19	53677225	53864749	0.178056	0.155406	0.278759	0.945843	1.00581	1.15298	1.30219	1.83172	1.79491
XLOC_064048	RBFOX3	chr19	53903230	53929968	67.8713	77.0432	60.4548	23.5724	32.9945	33.9821	27.4836	29.0119	27.4609
XLOC_064317	RBFOX3	chr19	53677225	53864749	1.46861	0.427699	0.0	0.986342	0.563221	0.352153	0.348143	0.45286	0.296132
XLOC_065304	RBFOX3	chr19	53635098	53635258	0.0	33.8807	0.0	10.7005	7.98175	14.8499	36.1619	6.46506	9.63192
XLOC_065305	RBFOX3	chr19	53645545	53646320	4.68517	2.10121	0.915891	2.30885	3.01468	3.15391	2.95732	2.91798	2.45286
XLOC_065306	RBFOX3	chr19	53646600	53647048	2.4899	0.743503	1.94432	2.45077	1.72954	3.06938	1.31525	1.95352	0.432457
XLOC_065307	RBFOX3	chr19	53647224	53647849	1.54715	0.924818	0.0	1.66284	0.721835	0.799285	0.969866	0.920808	0.539257

XLOC_065308	RBFOX3	chr19	53647966	53648430	0.0	0.704782	3.68616	1.47835	2.55256	0.485243	0.832999	0.0	0.410073
XLOC_065309	RBFOX3	chr19	53648693	53649805	0.757725	0.0	1.18552	1.29051	1.30449	1.25881	0.826207	0.607131	0.397297
XLOC_065310	RBFOX3	chr19	53677225	53864749	0.0	0.0	0.188284	0.366765	0.18903	0.20046	0.397254	0.193977	0.379103
XLOC_065311	RBFOX3	chr19	53677225	53864749	0.0	0.0	0.0	0.157053	0.273989	0.363616	0.23832	0.350496	0.459195
XLOC_065312	RBFOX3	chr19	53865027	53867074	0.0	0.0	0.0	0.995386	0.811171	0.956055	0.630418	1.07838	1.17231
XLOC_065313	RBFOX3	chr19	53930073	53930727	4.37033	3.91892	2.27756	1.56585	1.81376	1.80724	2.74417	1.1573	3.3015
XLOC_050606	RBM12;CPNE1;SPAG4;ERGIC3	chr13	65384035	65541853	0.0	0.0	0.905637	1.34904	0.542036	1.19954	1.0446	1.03886	0.808495
XLOC_050919	RBM12;CPNE1;SPAG4;ERGIC3	chr13	65384035	65541853	0.0	2.55538	7.66529	13.0844	4.70915	10.7809	6.1561	7.28201	7.4157
XLOC_052281	RBM12;CPNE1;SPAG4;ERGIC3	chr13	65384035	65541853	2.26394	0.676168	0.0	2.63405	3.67583	3.72617	3.60218	2.67153	4.5255
XLOC_052282	RBM12;CPNE1;SPAG4;ERGIC3	chr13	65384035	65541853	0.0	0.0	0.0	12.8878	10.1224	7.20714	15.6168	13.3747	14.36
XLOC_052283	RBM12;CPNE1;SPAG4;ERGIC3	chr13	65384035	65541853	1.40854	6.73662	6.6068	20.4402	17.8728	19.8098	14.1635	31.6298	28.2436
XLOC_052284	RBM12;CPNE1;SPAG4;ERGIC3	chr13	65384035	65541853	0.335525	0.2008	0.525191	0.571697	0.421533	0.349299	0.430194	0.304025	0.499226
XLOC_052285	RBM12;CPNE1;SPAG4;ERGIC3	chr13	65384035	65541853	0.0	0.0	0.0	3.27269	1.46822	1.83468	0.999186	1.54526	2.31827
XLOC_052286	RBM12;CPNE1;SPAG4;ERGIC3	chr13	65384035	65541853	0.0	0.0	0.0	1.43175	1.76669	0.470124	1.41358	0.674039	1.7875
XLOC_052287	RBM12;CPNE1;SPAG4;ERGIC3	chr13	65384035	65541853	0.0	0.0	0.0	1.63853	4.87589	3.72309	0.7792	0.0	1.58106
XLOC_052288	RBM12;CPNE1;SPAG4;ERGIC3	chr13	65384035	65541853	0.0	0.0	0.0	0.478738	0.293015	0.166554	0.389757	0.482637	0.373593
XLOC_052289	RBM12;CPNE1;SPAG4;ERGIC3	chr13	65384035	65541853	0.0	6.87343	13.4781	6.71084	3.91863	6.41457	2.89492	10.387	10.9198
XLOC_052290	RBM12;CPNE1;SPAG4;ERGIC3	chr13	65384035	65541853	0.0	0.0	0.815743	0.607565	0.653885	0.867231	1.1893	0.837745	1.23094

XLOC_074605	RBM28	chr4	93334151	93335191	2.45865	2.94144	2.56434	0.587668	0.512809	0.510373	0.595063	0.73819	0.644425
XLOC_080141	RBM47	chr6	61176457	61180271	0.0	0.0	0.0	0.0	0.0	0.0	0.0	0.095648	0.0
XLOC_065623	RBMS1	chr2	36115914	36190031	6.57975	6.27241	5.4685	6.85769	6.41546	7.56166	6.27105	8.13414	7.24918
XLOC_066262	RBMS1	chr2	36036324	36049380	30.9134	36.9747	37.5135	51.8685	48.8445	50.8161	55.4741	66.4761	49.7813
XLOC_066263	RBMS1	chr2	36082741	36105271	2.27275	1.26302	1.86591	0.564497	0.382444	0.439437	0.394316	0.85033	0.795704
XLOC_066264	RBMS1	chr2	36115914	36190031	20.0378	19.4496	18.156	11.2873	11.9646	11.6254	14.5546	11.9897	14.1732
XLOC_067323	RBMS1	chr2	35977793	35978662	2.03307	2.12784	1.59002	0.091096	0.0	0.316135	0.0919257	0.202979	0.266247
XLOC_067324	RBMS1	chr2	36081698	36082638	0.924292	1.65851	2.16881	0.579865	0.216698	0.383484	0.502361	1.01623	0.726508
XLOC_067325	RBMS1	chr2	36082741	36105271	0.0	0.0	0.0	0.164166	0.119811	0.127071	0.111828	0.276553	0.240351
XLOC_067326	RBMS1	chr2	36082741	36105271	0.0	0.0	0.0	0.243355	0.211558	0.140506	0.244119	0.40513	0.236875
XLOC_067327	RBMS1	chr2	36082741	36105271	0.0	0.0	0.0	0.200119	0.345827	0.230045	0.395493	0.219948	0.388695
XLOC_067328	RBMS1	chr2	36082741	36105271	0.0	0.356547	1.86497	0.0	0.0929952	0.0	0.0	0.0	0.0
XLOC_067329	RBMS1	chr2	36082741	36105271	0.0	0.0	0.518409	0.0	0.0	0.0	0.0	0.0	0.0
XLOC_067330	RBMS1	chr2	36082741	36105271	0.0	0.115016	0.300822	0.0	0.0	0.0	0.0	0.0	0.0
XLOC_067331	RBMS1	chr2	36082741	36105271	0.773723	0.0	0.0	0.0	0.0	0.0	0.0	0.0	0.0
XLOC_067332	RBMS1	chr2	36082741	36105271	0.0	0.944463	0.0	0.0	0.0	0.0	0.0	0.0	0.0
XLOC_067334	RBMS1	chr2	36115914	36190031	0.836615	1.2511	0.0	0.22496	0.196281	0.260493	0.0759905	0.0837114	0.21929
XLOC_067335	RBMS1	chr2	36115914	36190031	0.0	0.0	0.738978	0.254027	0.590598	0.489961	0.427863	0.283239	0.165045
XLOC_038032	RBP2	chr1	130688938	130721015	6.01498	2.41336	4.26145	2.06972	2.3677	1.56097	2.24248	1.85164	1.99357
XLOC_038528	RBP2	chr1	130688938	130721015	6.324	6.69412	6.49677	3.99708	3.72154	4.04982	3.18264	3.24718	4.1111
XLOC_040215	RBP2	chr1	130688938	130721015	1.86008	2.04023	0.485115	1.33404	1.89431	2.06181	1.01666	0.933065	1.13839
XLOC_040216	RBP2	chr1	130688938	130721015	8.67343	5.17661	6.76643	1.56348	3.88377	0.871675	2.81921	2.41928	4.46998
XLOC_040217	RBP2	chr1	130688938	130721015	1.15825	2.07768	2.71694	2.07545	1.35509	1.67936	1.88028	1.15432	2.22338
XLOC_040218	RBP2	chr1	130688938	130721015	0.0	2.04151	5.33905	1.22355	1.459	1.58663	0.610004	0.845453	2.08233
XLOC_040219	RBP2	chr1	130688938	130721015	5.79473	1.94996	1.13334	1.4934	1.36073	1.42926	0.790287	1.37897	1.64597
XLOC_050958	RBPJL	chr13	74376504	74390844	2.14956	2.56831	3.35824	5.77212	4.82405	4.86803	5.33127	3.38673	5.98069
XLOC_052508	RBPJL	chr13	74376504	74390844	0.0	0.0	0.0	1.07772	0.466343	0.62017	0.534558	0.791676	1.04726
XLOC_078740	RBX1	chr5	112692126	112693260	2.22196	0.22154	1.15883	0.265569	0.347802	0.230733	0.134641	0.296788	0.38837
XLOC_065873	RCAN3	chr2	128993157	129006805	3.73144	5.20601	3.8901	1.11437	5.62331	1.41633	2.5762	0.990688	4.99109
XLOC_065874	RCAN3	chr2	129010424	129017661	2.33816	5.1519	4.34531	1.02981	3.62728	1.58267	2.25023	0.588875	3.73539
XLOC_068572	RCAN3	chr2	129017761	129018847	0.0	0.232985	1.2187	0.69822	1.95024	0.646945	0.919761	0.155992	2.38228
XLOC_048558	RCBTB2	chr12	18338944	18339188	0.0	0.0	0.0	0.0	3.03826	3.26989	3.31728	0.757964	6.98196
XLOC_079835	RCHY1	chr6	91978953	92075856	9.81904	16.3466	22.9237	1.15514	1.86481	1.54338	0.889229	1.05341	1.40113

XLOC_058175	RCOR3;TRAF5	chr16	73895423	74046936	2.70368	1.2936	0.931309	0.105043	0.02303	0.0712394	0.156701	0.0590856	0.0602464
XLOC_043053	RDH12	chr10	80159413	80160081	0.0	1.69404	0.0	0.0	0.110286	0.146509	0.0	0.140763	0.247032
XLOC_043054	RDH12	chr10	80160191	80160665	0.0	0.0	0.0	0.0	0.176669	0.0	0.605821	0.0	0.0
XLOC_082995	REEP6;C7H19orf25	chr7	45475198	45522421	4.29854	9.06047	6.39136	6.155	7.10066	9.57031	5.65122	4.91822	9.99573
XLOC_083636	REEP6;C7H19orf25	chr7	45475198	45522421	0.739139	0.663262	0.578233	15.7691	16.1399	14.1229	10.4809	7.18253	14.8572
XLOC_083637	REEP6;C7H19orf25	chr7	45475198	45522421	35.6203	30.4563	31.1227	27.3433	29.4472	27.4293	28.6075	28.5099	27.6886
XLOC_084843	REEP6;C7H19orf25	chr7	45475198	45522421	6.96806	2.079	0.0	5.29678	4.27034	4.98253	1.80922	3.37807	6.0314
XLOC_084844	REEP6;C7H19orf25	chr7	45475198	45522421	2.5671	2.30234	2.0071	0.920023	0.700133	0.531353	0.693068	0.51092	0.55976
XLOC_079740	RELL1	chr6	58596021	58692517	12.0303	13.0207	9.38875	24.8314	24.4436	22.7357	35.8669	32.3879	33.3945
XLOC_072611	RELN	chr4	44981404	45012604	3.51559	6.38349	5.78539	7.97926	7.92202	7.894	7.68076	7.67733	7.50114
XLOC_072612	RELN	chr4	45018619	45032162	8.02643	11.4915	7.62598	19.4296	14.8272	20.4416	22.1115	29.8695	16.9957
XLOC_072613	RELN	chr4	45184392	45286131	2.08174	3.88182	2.16619	0.416693	0.219156	0.237218	0.104274	0.120935	0.486087
XLOC_072614	RELN	chr4	45297159	45316390	4.7793	3.37096	4.21792	9.26835	9.95216	9.97964	9.1238	9.40468	12.1553
XLOC_072615	RELN	chr4	45342087	45376433	5.54922	6.07033	5.84154	2.74604	2.46701	2.75154	3.16426	3.62834	2.63079
XLOC_073152	RELN	chr4	44981404	45012604	3.51158	4.58529	6.33067	1.9722	2.50801	4.01796	2.39219	1.40731	2.45948
XLOC_073153	RELN	chr4	45035114	45084592	8.04133	11.8523	8.96945	2.07355	2.56003	2.13652	2.0828	2.04765	2.81659
XLOC_073154	RELN	chr4	45105203	45108452	8.09721	13.0188	3.19046	107.822	81.4071	88.0119	109.056	127.971	131.356
XLOC_073155	RELN	chr4	45316654	45320621	16.1384	19.3072	24.583	63.8376	53.7939	58.1999	63.6439	77.1725	63.9978
XLOC_073156	RELN	chr4	45398014	45408908	17.427	24.0576	28.7709	17.2331	20.4725	20.0108	17.4788	19.205	21.7585
XLOC_073157	RELN	chr4	45423264	45529826	27.651	29.1594	30.8608	10.9337	9.10319	11.1287	7.49748	8.61294	8.52618
XLOC_074122	RELN	chr4	44981404	45012604	0.0	0.736224	1.28362	0.294194	0.834276	0.255474	0.148939	0.16424	0.215064
XLOC_074123	RELN	chr4	45032251	45032516	13.2721	3.95699	5.17258	5.95223	10.4826	5.35731	7.14736	12.5021	7.42033
XLOC_074124	RELN	chr4	45032660	45033214	1.82424	4.90568	0.0	2.94012	4.38767	5.08041	5.52644	3.78723	4.12876
XLOC_074125	RELN	chr4	45033544	45033708	0.0	19.9022	52.0495	6.25589	23.5696	16.3842	9.87638	2.86879	5.66315
XLOC_074126	RELN	chr4	45033886	45034704	8.76025	4.58396	4.28164	0.981225	1.45279	1.02114	0.889929	0.982996	2.10265
XLOC_074127	RELN	chr4	45102936	45103612	0.0	0.0	0.0	1.24959	1.73765	1.15414	0.250543	1.24765	1.2162
XLOC_074128	RELN	chr4	45376623	45377091	2.32955	2.78289	0.0	2.9187	2.88032	4.31172	3.70223	2.289	4.04834
XLOC_074129	RELN	chr4	45377213	45377491	0.0	0.0	9.27342	2.66461	2.69365	3.60893	1.49045	0.563356	3.58491
XLOC_074130	RELN	chr4	45377642	45377925	0.0	5.11529	0.0	0.5122	5.61784	4.6298	1.91612	2.17066	4.92559
XLOC_074131	RELN	chr4	45378693	45379105	2.84187	4.24171	4.43681	1.77955	3.49989	2.33069	1.98919	3.05316	2.95796

XLOC_074132	RELN	chr4	45379286	45379601	0.0	1.36563	3.57076	1.2289	4.17933	2.32693	1.948	1.75611	1.97632
XLOC_074133	RELN	chr4	45395922	45396992	5.54823	7.34907	8.06031	4.26272	5.58063	5.34815	7.05338	5.15779	5.88671
XLOC_074134	RELN	chr4	45397136	45397566	34.5058	15.8495	12.4341	15.4359	19.4369	16.8891	19.5722	13.7698	21.4248
XLOC_074135	RELN	chr4	45398014	45408908	14.4938	6.05698	4.52538	2.8523	3.34538	2.97071	1.0135	1.9805	4.27351
XLOC_089523	REPS1;ABRACL	chr9	77754541	77794522	42.7831	52.7849	52.8172	48.9865	55.3371	51.5621	64.2346	45.6309	50.257
XLOC_076403	RERGL	chr5	92430171	92435942	0.946083	1.73829	3.71013	2.82998	2.23891	2.07612	3.14294	2.89392	2.00839
XLOC_076917	RERGL	chr5	92430171	92435942	13.4044	7.79975	7.81385	12.7795	13.78	16.2846	19.3923	16.4178	13.8954
XLOC_076918	RERGL	chr5	92442961	92505742	17.7729	10.7679	13.0461	33.3675	38.6617	35.3455	36.2836	29.2757	51.1794
XLOC_078329	RERGL	chr5	92440136	92441032	0.0	0.292873	0.0	0.877686	1.14754	0.913938	0.177251	0.489117	1.02619
XLOC_078330	RERGL	chr5	92441101	92442128	6.65443	3.48295	8.4586	18.4899	24.3299	17.7834	22.0396	18.062	33.1376
XLOC_078331	RERGL	chr5	92442254	92442452	30.2271	9.07157	11.856	72.1771	57.7563	43.8519	54.495	46.5438	105.218
XLOC_078332	RERGL	chr5	92442586	92442696	0.0	914.426	1210.28	318.199	576.391	1003.14	100.867	344.136	1400.11
XLOC_057941	RFWD2	chr16	58670233	58679212	8.32892	10.2059	9.3116	18.2451	18.832	19.0685	17.3426	18.5938	17.422
XLOC_057942	RFWD2	chr16	58727939	58734205	24.1895	18.5529	19.4745	6.85616	8.65817	7.46111	9.33405	7.10029	5.90388
XLOC_059216	RFWD2	chr16	58679415	58679844	10.6563	15.1124	8.31986	19.3062	24.2297	25.6988	22.9143	17.4692	21.5029
XLOC_059217	RFWD2	chr16	58680165	58680387	0.0	6.28936	8.22005	1.90688	3.12409	5.27625	5.06268	2.90771	6.32494
XLOC_082815	RFX2	chr7	19577158	19586432	1.60628	0.662041	0.0	31.1122	22.813	24.0022	21.7384	39.7908	20.2247
XLOC_083440	RFX2	chr7	19577158	19586432	4.96408	6.89325	6.37964	11.8145	12.9755	11.6802	11.261	14.6948	10.4636
XLOC_083441	RFX2	chr7	19586600	19604689	16.4755	15.5299	16.0416	25.0941	23.0271	25.1043	27.094	30.1004	24.526
XLOC_083442	RFX2	chr7	19606217	19629388	22.6942	21.4331	21.4658	6.87406	5.26721	6.02624	6.53863	7.74397	5.86142
XLOC_093245	RGN	chrX	90744660	90744822	0.0	10.5882	55.3843	6.67164	2.50116	13.9341	10.4702	9.12481	3.01139
XLOC_057776	RGS1	chr16	13256105	13417903	21.3073	17.9926	10.937	9.55321	4.66713	8.25076	5.51145	7.67676	4.74765
XLOC_058026	RGS1	chr16	13256105	13417903	8.74993	8.67122	10.6977	2.84383	3.64375	2.67228	2.99319	3.78915	3.44354
XLOC_058423	RGS1	chr16	13256105	13417903	1.56051	1.86558	3.65922	0.419293	1.21331	0.322447	0.419142	0.464302	0.271942
XLOC_058424	RGS1	chr16	13256105	13417903	2.87304	1.28817	0.0	0.257353	0.223604	0.0	0.0	0.428058	0.125224
XLOC_058425	RGS1	chr16	13256105	13417903	0.0	0.526515	0.0	0.473336	0.547111	0.0	0.471528	0.0	0.0
XLOC_058426	RGS1	chr16	13256105	13417903	12.155	13.2137	13.0087	5.82888	5.71108	5.16768	5.43848	5.87538	4.97341
XLOC_058427	RGS1	chr16	13256105	13417903	11.5393	15.0254	20.711	5.96333	5.95295	5.42955	5.55913	5.23992	5.2808
XLOC_091473	RGS17	chr9	91220619	91221330	20.9078	24.2201	31.6714	11.0043	11.9115	12.7105	10.6961	6.63006	12.4222
XLOC_058432	RGS18	chr16	13754411	13757460	1.50072	0.374251	0.195771	6.72968	3.26243	4.1163	3.41853	7.03317	3.13142
XLOC_086607	RGS3	chr8	104540776	104550331	0.0	0.154721	1.21399	0.0463683	0.202739	0.215108	0.236008	0.0	0.0
XLOC_087040	RGS3	chr8	104511030	104534667	51.4323	75.9887	65.6436	59.2595	73.9326	65.2944	70.1122	51.7124	74.9684

XLOC_057893	RGS7	chr16	36693906	36726810	0.0	0.0	0.0	16.0294	18.2362	14.5618	19.4414	20.7352	29.2767
XLOC_058117	RGS7	chr16	36122102	36420853	84.6958	7.86208	0.0	0.259285	0.0	0.0	0.253346	0.0	0.0
XLOC_058119	RGS7	chr16	36122102	36420853	49.7024	6.24836	2.04247	0.0	0.0	0.0	0.0	0.0	0.227073
XLOC_058911	RGS7	chr16	36122102	36420853	0.0	0.0	0.0	0.0	0.0	0.0	0.0320584	0.0	0.0
XLOC_058912	RGS7	chr16	36693906	36726810	0.0	0.0	0.0	2.78534	4.07157	2.30034	4.50986	4.76054	6.00719
XLOC_058913	RGS7	chr16	36693906	36726810	0.0	0.0	1.0679	3.42625	3.82943	3.3911	4.29569	3.93808	4.52601
XLOC_066465	RHBDD1	chr2	115863092	116065021	1.06612	0.962637	1.27568	0.282899	0.23009	0.977864	0.112151	0.626434	0.127851
XLOC_068305	RHBDD1	chr2	115857393	115859736	0.0	0.0	0.0	0.0593528	0.0	0.0	0.030309	0.0	0.0
XLOC_068306	RHBDD1	chr2	115859786	115862646	0.0	0.0	0.0	0.0	0.0	0.0557431	0.0490793	0.0	0.0
XLOC_068307	RHBDD1	chr2	115863092	116065021	0.0	0.0	0.0	0.0	0.0	1.37091	0.765784	0.0	0.0
XLOC_070476	RHBDL2	chr3	107665042	107696279	44.2737	35.4673	38.4446	25.1099	43.1999	42.5898	44.606	33.7657	28.2458
XLOC_072061	RHBDL2	chr3	107657976	107658409	10.5016	12.542	8.19945	1.40938	2.22659	2.69486	0.922336	1.02827	2.27889
XLOC_072062	RHBDL2	chr3	107661028	107661476	7.46969	4.46102	1.94432	0.668392	1.72954	1.27891	0.657625	0.0	0.864914
XLOC_072063	RHBDL2	chr3	107663045	107664965	21.3378	23.8191	27.6167	17.1469	22.8628	20.4527	26.7572	19.3579	18.9577
XLOC_072064	RHBDL2	chr3	107665042	107696279	4.2995	2.56866	6.71648	1.34683	1.16466	2.213	1.90431	1.48169	1.49517
XLOC_076262	RHEBL1	chr5	30913408	30921312	3.47023	2.17951	2.17985	2.00984	1.82003	1.59368	1.78411	1.79299	1.50843
XLOC_076263	RHEBL1	chr5	30921564	30931270	1.22738	2.90168	1.52368	1.65456	2.34281	3.00124	1.31516	0.76865	2.21088
XLOC_044286	RHOB	chr11	78465582	78472282	2.86568	5.41952	8.59947	2.85451	2.45223	2.26865	1.69981	3.43988	2.91077
XLOC_086920	RHOBTB2	chr8	70835169	70839228	5.15847	7.87175	8.52823	12.5566	17.5721	15.5203	15.1956	8.3932	11.7271
XLOC_086921	RHOBTB2	chr8	70843883	70855154	1.4866	1.84669	1.18349	0.922357	1.21056	1.19254	1.44484	0.845596	1.23426
XLOC_086922	RHOBTB2	chr8	70868222	70873769	11.8622	12.5234	11.4639	64.7409	53.0459	61.8238	63.9183	67.0711	42.3886
XLOC_086923	RHOBTB2	chr8	70883584	70887876	2.22373	0.0	0.869478	3.38739	2.77633	3.45577	1.50555	2.43932	1.06742
XLOC_086924	RHOBTB2	chr8	70894769	70897519	26.5621	36.8004	23.1365	92.848	121.518	118.121	117.241	87.4829	89.2947
XLOC_088047	RHOBTB2	chr8	70839289	70839641	0.0	1.10847	2.89847	4.65182	5.96786	6.44491	3.52386	0.71874	5.46397
XLOC_088048	RHOBTB2	chr8	70843283	70843812	0.0	1.74507	0.0	0.174311	3.16923	0.802701	1.03857	0.384392	0.508232
XLOC_088049	RHOBTB2	chr8	70893543	70893891	0.0	1.13156	0.0	1.01762	2.02972	0.386875	1.95977	1.46627	0.984141
XLOC_088050	RHOBTB2	chr8	70894088	70894292	0.0	0.0	0.0	3.74973	5.03561	5.47192	2.15545	2.48714	2.34829
XLOC_041993	RHOJ	chr10	75774117	75798570	3.67319	3.83583	2.35546	3.83475	3.20192	4.62445	2.4273	2.48058	4.97656
XLOC_041994	RHOJ	chr10	75831858	75835728	0.764108	0.997383	1.19551	8.63877	8.86186	9.57642	5.67682	4.69601	6.95426
XLOC_041995	RHOJ	chr10	75858520	75860818	15.7029	15.7084	17.9567	170.325	159.647	172.222	169.953	159.803	158.061
XLOC_042978	RHOJ	chr10	75837807	75838639	0.0	0.0	0.0	0.288264	0.418499	0.222241	0.581214	0.427937	0.280797
XLOC_042979	RHOJ	chr10	75855989	75857093	0.0	0.0	0.0	1.16442	0.358759	0.476025	0.347122	0.688741	0.200323
XLOC_042980	RHOJ	chr10	75857152	75858373	0.0	0.406856	0.0	1.70701	0.852013	0.635812	0.68071	1.49991	0.891692

XLOC_044001	RHOQ;PIGF;CRIPT	chr11	28853224	28926710	10.6969	6.72293	8.37437	2.24862	0.532368	2.10267	1.47372	0.796913	0.860591
XLOC_062429	RHPN2;C18H19orf40;GPATCH1	chr18	43531529	43687705	2.80635	3.90567	4.21014	0.0838813	0.0183605	0.146153	0.0	0.0707075	0.0
XLOC_063075	RHPN2;C18H19orf40;GPATCH1	chr18	43531529	43687705	0.0	0.0	0.0	0.120324	0.0	0.0	0.0	0.0	0.0
XLOC_078830	RIBC2	chr5	116552996	116553920	0.0	0.0	0.0	0.0	0.0737291	0.0	0.0	0.0	0.0
XLOC_054592	RIMS2	chr14	62540388	62540703	0.0	2.73126	3.57076	21.7106	24.3794	26.527	27.6616	18.0001	15.0201
XLOC_054593	RIMS2	chr14	62541150	62541551	2.9701	0.0	2.31822	16.2058	19.4132	22.2108	33.9668	22.3033	12.6179
XLOC_073165	RINT1;ATXN7L1;PUS7	chr4	46663680	46991254	0.0	0.0	0.0	0.313543	0.109723	0.109187	0.384145	0.175975	0.183598
XLOC_074185	RINT1;ATXN7L1;PUS7	chr4	46663680	46991254	0.0	0.0	0.0	3.61802	2.8138	2.25209	2.22112	1.77939	5.40924
XLOC_054843	RIPK2	chr14	76237285	76237557	49.9879	20.4904	24.3508	110.887	77.6904	71.3261	49.9162	85.5952	74.2015
XLOC_070102	RLF	chr3	106547860	106575797	10.5909	9.52294	7.78664	8.29145	15.0841	9.45204	13.1651	8.39322	17.8674
XLOC_070469	RLF	chr3	106503017	106547837	10.9755	12.2096	16.6615	15.1916	18.328	15.8726	18.3672	18.2766	27.6397
XLOC_072023	RLF	chr3	106502114	106502546	0.0	0.78672	0.0	0.471499	0.609417	0.270452	0.231384	0.0	0.228712
XLOC_072024	RLF	chr3	106547860	106575797	0.0	0.614741	1.60761	0.552628	0.159363	0.63585	0.912895	0.0	1.78979
XLOC_090029	RMND1	chr9	89589592	89623020	0.163559	0.332084	0.384104	0.0440043	0.0385752	0.0511306	0.0751321	0.0329968	0.0572908
XLOC_041488	RNASE1	chr10	26229719	26272908	0.0	0.0929474	0.0	0.0	0.0487588	0.0646565	0.0284299	0.0	0.0271585
XLOC_047799	RNASEH2B	chr12	20243276	20666316	2.17535	0.649718	1.64977	5.53709	4.35247	3.1963	5.51185	2.24051	3.6016
XLOC_048099	RNASEH2B	chr12	20243276	20666316	0.0	0.0	0.0	0.0	0.0	95.7409	0.0	0.0	0.0
XLOC_048100	RNASEH2B	chr12	20243276	20666316	0.0	0.0	0.0	0.372198	3.80828	0.423726	0.356311	0.0	0.719153
XLOC_048693	RNASEH2B	chr12	20243276	20666316	0.0	6.73161	0.0	14.3087	15.0092	10.1525	17.9705	15.5014	5.7991
XLOC_048694	RNASEH2B	chr12	20243276	20666316	0.0	0.0	0.0	6.04572	11.5187	7.78941	6.20933	11.2196	8.57968
XLOC_048695	RNASEH2B	chr12	20243276	20666316	0.0	0.0	0.0	0.991748	2.54498	0.75444	1.27528	1.43046	1.91875
XLOC_048696	RNASEH2B	chr12	20243276	20666316	1.77064	0.0	0.0	0.634199	1.37438	1.46156	0.789609	0.35032	0.924977
XLOC_048697	RNASEH2B	chr12	20243276	20666316	0.0	0.0	0.0	0.768248	1.30146	1.39205	0.977251	0.91433	1.12409
XLOC_048698	RNASEH2B	chr12	20243276	20666316	0.0	0.0	0.0	0.88533	1.09143	0.560264	0.984353	0.270727	0.785271
XLOC_048699	RNASEH2B	chr12	20243276	20666316	0.0	0.4311	0.0	1.42108	1.34691	0.59646	1.55288	0.143246	0.880049
XLOC_048700	RNASEH2B	chr12	20243276	20666316	0.0	0.0	0.0	1.68955	1.45861	0.970485	1.2495	0.0	0.410073
XLOC_048701	RNASEH2B	chr12	20243276	20666316	0.0	0.0	0.0	0.670804	0.655965	0.961423	0.44245	0.575216	0.885507
XLOC_048702	RNASEH2B	chr12	20243276	20666316	0.0	0.0	0.0	0.717683	1.17275	0.830269	0.0905543	0.29989	0.961431
XLOC_048703	RNASEH2B	chr12	20243276	20666316	0.0	0.0	0.0	1.04812	0.682879	0.756035	0.393571	0.726186	1.14743

XLOC_048704	RNASEH2B	chr12	20243276	20666316	0.0	0.0	0.0	0.0	0.144044	0.383013	0.330735	0.367026	1.29307
XLOC_048705	RNASEH2B	chr12	20243276	20666316	2.43322	0.0	0.0	10.2463	8.53686	9.82506	9.31718	5.33139	9.55256
XLOC_048706	RNASEH2B	chr12	20243276	20666316	0.0	0.0	0.0	16.8329	11.9958	5.79907	9.22341	2.12273	4.96991
XLOC_048707	RNASEH2B	chr12	20243276	20666316	0.0	0.0	0.0	7.66829	2.60366	5.06595	6.61825	3.56139	2.93095
XLOC_048708	RNASEH2B	chr12	20243276	20666316	0.0	0.0	0.606086	3.75021	3.69786	5.46972	5.13754	4.26703	4.53602
XLOC_048709	RNASEH2B	chr12	20243276	20666316	0.0	0.133369	0.0	0.869026	0.279755	0.794852	0.366651	0.672254	0.6052
XLOC_048710	RNASEH2B	chr12	20243276	20666316	0.0	0.0	0.0	2.49338	1.71697	0.857457	1.70867	1.08956	1.45078
XLOC_048711	RNASEH2B	chr12	20243276	20666316	0.88172	0.923344	1.37998	5.09952	4.25425	4.90761	3.26394	4.43278	2.89279
XLOC_048712	RNASEH2B	chr12	20243276	20666316	0.0	0.0	0.0	0.496717	2.93692	2.80845	1.86275	3.16292	8.12461
XLOC_048713	RNASEH2B	chr12	20243276	20666316	0.0	0.0	7.16637	5.80199	4.10533	0.922256	6.68926	6.81125	3.94324
XLOC_048714	RNASEH2B	chr12	20243276	20666316	0.0	1.2413	0.0	0.744397	3.49092	0.847452	1.06891	0.801304	1.07873
XLOC_048715	RNASEH2B	chr12	20243276	20666316	0.0	0.613187	0.0	1.28636	1.44633	1.43865	1.21604	0.875064	0.941152
XLOC_048716	RNASEH2B	chr12	20243276	20666316	4.89267	5.84875	0.0	17.3808	11.6593	16.6737	15.6036	16.8074	12.5019
XLOC_048717	RNASEH2B	chr12	20243276	20666316	0.0	0.0	0.0	0.327174	0.0713279	0.094668	0.248067	0.182464	0.159416
XLOC_048718	RNASEH2B	chr12	20243276	20666316	0.0	0.501435	0.437158	0.901648	0.700652	0.580826	0.611454	0.392664	0.635171
XLOC_048719	RNASEH2B	chr12	20243276	20666316	0.469784	0.140556	0.0	0.631852	0.552719	0.781854	0.772491	0.708289	0.739742
XLOC_048720	RNASEH2B	chr12	20243276	20666316	0.0	0.0	0.0	5.22301	7.16712	7.73465	6.21626	3.56395	9.92583
XLOC_048721	RNASEH2B	chr12	20243276	20666316	0.0	0.0	0.812112	4.55974	4.78359	6.45799	5.53819	3.42038	4.89528
XLOC_048722	RNASEH2B	chr12	20243276	20666316	0.0	0.539743	0.0	0.566131	0.211598	0.748887	0.490653	0.541314	0.945788
XLOC_048723	RNASEH2B	chr12	20243276	20666316	0.0	0.0	0.0	0.431374	0.124788	0.33166	0.718144	0.477441	1.25885
XLOC_048724	RNASEH2B	chr12	20243276	20666316	0.0	0.0	0.0	0.278971	0.22214	0.235592	0.129619	0.170935	0.173285
XLOC_048725	RNASEH2B	chr12	20243276	20666316	0.0	0.464968	0.608039	0.975408	1.15548	0.726256	1.34142	0.700472	0.543355
XLOC_048726	RNASEH2B	chr12	20243276	20666316	0.0	0.499773	0.0	5.84087	4.93668	7.25177	5.52869	6.62546	5.68071
XLOC_048727	RNASEH2B	chr12	20243276	20666316	0.0	1.87074	0.0	7.47449	4.36388	4.9443	6.10878	5.34859	6.71708
XLOC_048728	RNASEH2B	chr12	20243276	20666316	7.27542	2.16963	5.67212	7.18843	7.64109	9.53023	4.19129	7.50508	11.2578
XLOC_048729	RNASEH2B	chr12	20243276	20666316	1.92628	0.575506	3.0101	2.58687	1.79199	1.98563	2.39843	1.71177	2.34666
XLOC_048730	RNASEH2B	chr12	20243276	20666316	0.0	0.642692	2.35331	2.19575	2.02242	1.65379	2.1601	1.68486	2.21763
XLOC_048731	RNASEH2B	chr12	20243276	20666316	0.0	0.292478	0.0	2.36655	2.3684	2.33248	2.38971	1.27001	1.87881
XLOC_048732	RNASEH2B	chr12	20243276	20666316	0.0	0.417788	0.0	2.8796	1.84979	1.73453	1.25508	1.94448	1.5841
XLOC_048733	RNASEH2B	chr12	20243276	20666316	0.0	0.356606	0.621796	2.10182	1.77717	2.10843	1.74436	1.11906	1.70336
XLOC_048734	RNASEH2B	chr12	20243276	20666316	0.0	0.0	1.69419	1.74718	0.839087	1.33943	0.960247	0.640576	2.07421
XLOC_048735	RNASEH2B	chr12	20243276	20666316	0.0	0.0	0.0	2.40886	3.12192	2.30749	1.98335	2.20612	1.36462
XLOC_048736	RNASEH2B	chr12	20243276	20666316	0.0	0.420221	0.0	1.13336	1.31326	1.16304	2.77685	1.39688	1.96097

XLOC_048737	RNASEH2B	chr12	20243276	20666316	0.644314	0.192742	0.0	0.866434	1.31212	0.803367	1.23205	0.904715	1.1829
XLOC_048738	RNASEH2B	chr12	20243276	20666316	0.0	0.467401	0.0	0.840396	2.91817	1.61569	3.08017	0.465288	3.40659
XLOC_076791	RND1	chr5	31086005	31093308	0.0	0.125484	0.328198	1.01537	1.11888	0.610921	0.345133	0.506157	0.660503
XLOC_070046	RNF11	chr3	95588396	95619256	9.85341	11.8622	7.88786	13.0954	11.8272	13.0413	11.7352	14.7656	15.059
XLOC_082981	RNF126;POLRMT;FGF22	chr7	44857053	44892326	5.57895	6.35294	7.03574	13.0983	7.46332	10.8002	12.3991	16.0624	8.11225
XLOC_083611	RNF126;POLRMT;FGF22	chr7	44857053	44892326	77.6894	86.4371	74.4938	53.7992	42.3423	49.436	52.687	67.8087	34.7032
XLOC_084816	RNF126;POLRMT;FGF22	chr7	44857053	44892326	2.1369	0.63829	6.67688	0.573945	0.661522	0.439969	0.757284	0.841808	0.929004
XLOC_038403	RNF168;UBXN7	chr1	71585244	71654506	36.1942	26.9446	26.8041	141.391	142.464	145.777	195.241	170.268	117.921
XLOC_087037	RNF183;PRPF4	chr8	104226692	104248553	4.05249	4.04258	3.97965	6.44768	8.82175	5.64166	7.4518	4.56532	6.36803
XLOC_053382	RNF19A	chr14	66177292	66281017	0.0	0.513497	0.0	0.54829	0.747077	0.801714	0.0913759	0.447099	0.374966
XLOC_053383	RNF19A	chr14	66296529	66309521	29.2371	39.079	38.6606	20.1065	31.6119	20.8123	24.7865	25.5688	42.7161
XLOC_053680	RNF19A	chr14	66177292	66281017	2.92689	3.33261	0.844829	8.08793	4.92755	7.35388	1.48483	4.07505	3.27598
XLOC_054699	RNF19A	chr14	66177292	66281017	0.0	0.150316	0.0	1.12621	1.5365	0.57475	0.183479	0.807669	1.14261
XLOC_054700	RNF19A	chr14	66296529	66309521	10.5309	9.67473	10.0036	4.51763	3.47309	2.42127	3.69124	3.0891	3.1553
XLOC_054701	RNF19A	chr14	66296529	66309521	0.0	5.0505	2.20117	3.27919	3.03906	1.15642	2.96168	1.65281	1.46757
XLOC_054702	RNF19A	chr14	66296529	66309521	23.8327	16.3698	11.1675	7.25139	6.6274	3.42979	5.04529	4.9146	7.45365
XLOC_066505	RNF19B;TMEM54;HPCA;YARS;FNDC5;S100PBP;RBBP4;SYNC	chr2	121547426	121807160	1.43515	2.32417	3.47343	1.77182	1.61636	1.20542	1.40552	0.954779	1.9657
XLOC_068433	RNF19B;TMEM54;HPCA;YARS;FNDC5;S100PBP;RBBP4;SYNC	chr2	121547426	121807160	0.726525	0.869258	0.0	0.716386	0.284336	0.980851	0.396327	0.291174	0.380977
XLOC_068434	RNF19B;TMEM54;HPCA;YARS;FNDC5;S100PBP;RBBP4;SYNC	chr2	121547426	121807160	0.780636	1.63441	2.44265	0.419835	1.03829	0.729381	0.638121	0.312652	0.818542
XLOC_068435	RNF19B;TMEM54;HPCA;YARS;FNDC5;S100PBP;RBBP4;SYNC	chr2	121547426	121807160	1.33256	0.79723	1.56382	0.477839	0.57395	0.830641	0.363872	0.467651	0.232977

XLOC_068436	RNF19B;TMEM54;HPCA;YARS;FNDC5;S100PBP;RBBP4;SYNC	chr2	121547426	121807160	0.994187	0.297299	0.0	0.890944	0.0776503	0.618453	0.269834	0.198569	0.260413
XLOC_068437	RNF19B;TMEM54;HPCA;YARS;FNDC5;S100PBP;RBBP4;SYNC	chr2	121547426	121807160	1.02519	0.613122	0.801763	1.01057	0.40026	0.743886	0.648861	0.409368	0.80551
XLOC_068438	RNF19B;TMEM54;HPCA;YARS;FNDC5;S100PBP;RBBP4;SYNC	chr2	121547426	121807160	0.0	0.0	4.93938	2.83045	0.486003	0.323854	1.37682	0.0	0.0
XLOC_086573	RNF20;TMEM246	chr8	92855347	92918434	10.0823	8.39155	2.5162	0.325295	0.599287	0.599819	0.461793	0.434619	0.457479
XLOC_055414	RNF214	chr15	28370472	28397102	0.0	0.0	1.38527	0.0	0.0	0.0613409	0.0	0.0	0.0
XLOC_048145	RNF219	chr12	54102668	54107431	0.0	0.0	0.0	0.0	0.0	0.0	2.85218	1.15861	5.40767
XLOC_049378	RNF219	chr12	54102668	54107431	0.0	0.0	0.0	0.210508	0.181744	0.725531	1.03801	0.23107	2.4525
XLOC_049379	RNF219	chr12	54102668	54107431	0.0	0.0	0.0	0.0	0.0	0.191022	0.494868	0.54916	1.61222
XLOC_070082	RNF220	chr3	102086525	102127987	5.86825	7.31194	4.58961	0.0	0.0	0.202824	0.354031	0.390771	0.0
XLOC_070083	RNF220	chr3	102133379	102204670	10.6949	16.2015	8.33584	0.234163	0.612794	0.18072	0.236927	0.683489	1.14108
XLOC_070453	RNF220	chr3	102133379	102204670	1.64239	3.14064	1.8817	17.2351	13.9931	14.4073	22.7075	25.5354	20.8935
XLOC_071887	RNF220	chr3	102133379	102204670	6.42915	4.99726	10.0533	0.34559	0.400799	0.665359	0.809965	0.25598	0.56075
XLOC_071888	RNF220	chr3	102133379	102204670	0.0	0.0	0.0	1.6699	3.71293	1.5241	0.965817	0.72249	3.55348
XLOC_071889	RNF220	chr3	102133379	102204670	0.280155	0.167674	0.219274	0.530379	1.90493	1.21852	0.956794	0.392592	1.5461
XLOC_071890	RNF220	chr3	102133379	102204670	0.653631	0.391057	0.0	0.878955	1.94531	1.29032	1.07115	0.524433	1.48568
XLOC_071891	RNF220	chr3	102133379	102204670	0.0	0.891898	0.0	1.33642	2.79542	2.37072	4.22726	3.67359	5.20825
XLOC_050551	RNF24	chr13	51645078	51687514	48.4161	52.3481	41.7337	134.443	102.476	114.598	122.772	121.15	129.687
XLOC_050849	RNF24	chr13	51645078	51687514	3.13892	1.19161	0.0	1.1675	0.515674	1.84703	0.843417	0.521992	0.551667
XLOC_051833	RNF24	chr13	51645078	51687514	7.36161	3.66817	0.959437	14.9502	19.8953	15.2428	17.7941	16.3722	19.2665
XLOC_051834	RNF24	chr13	51645078	51687514	1.87496	3.64464	1.46654	6.21747	6.37317	4.66712	6.1128	4.12223	5.64904
XLOC_051835	RNF24	chr13	51688335	51689341	0.0	1.52961	2.00025	0.381998	0.533218	0.972981	0.154633	0.170547	0.670169
XLOC_059853	RNF34	chr17	55997191	56036658	2.84902	2.89035	2.52932	5.14515	5.3412	4.79349	4.70594	5.0897	4.92067
XLOC_059854	RNF34	chr17	56040354	56057790	12.6086	16.3435	13.1523	34.2854	34.2206	32.741	34.0247	44.1603	32.0214
XLOC_060243	RNF34	chr17	55997191	56036658	3.6681	0.35594	1.86634	5.33686	6.93793	6.89796	2.13031	3.62365	2.92738

XLOC_060244	RNF34	chr17	55997191	56036658	18.5687	19.9618	19.6025	23.5493	20.2417	21.1916	22.9315	31.0682	20.1053
XLOC_086414	RNF38	chr8	60993150	61132533	11.0486	13.6519	15.8617	10.494	14.9896	12.792	11.8005	9.06663	11.0586
XLOC_086880	RNF38	chr8	60910302	60983962	17.2502	15.2645	14.5961	23.3537	28.0611	26.8043	24.4369	15.8487	21.3939
XLOC_079846	RNF4;HAUS3;MXD4	chr6	108213208	108551233	29.2055	26.1925	27.1478	15.3202	19.1895	15.3337	19.411	15.8671	16.1944
XLOC_063902	RNF43	chr19	9557793	9559166	14.9773	15.2987	16.5899	7.26837	5.93438	4.65112	9.44045	7.82818	6.8591
XLOC_064558	RNF43	chr19	9606863	9607372	2.05782	1.22946	0.0	12.7105	12.4303	10.5975	13.6934	15.4175	18.9718
XLOC_038030	RNF7	chr1	128193654	128238355	33.4682	35.3453	32.9128	19.7339	21.6197	23.648	21.8952	23.8855	22.9113
XLOC_064596	RNFT1	chr19	11121298	11122426	72.2876	84.9281	54.2187	88.7137	75.762	86.3611	112.637	118.696	83.8842
XLOC_089947	RNGTT	chr9	62057134	62122720	20.8756	25.1043	24.5707	8.39394	8.49362	7.41861	9.29052	10.1378	10.722
XLOC_038296	ROBO1	chr1	25803375	25829607	0.0	0.0	0.0	0.159564	0.0695845	0.369402	0.0	0.0890108	0.155504
XLOC_039050	ROBO1	chr1	25803375	25829607	0.0	0.0	0.0	0.120772	0.104999	0.0	0.0	0.0	0.0
XLOC_044341	ROCK2	chr11	86544923	86567193	3.5859	3.35283	4.91077	2.97424	3.05927	4.01026	3.44101	4.0559	3.52935
XLOC_046882	ROCK2	chr11	86571302	86571877	0.0	1.5531	1.3539	0.155138	0.134516	0.0	0.154624	0.0	0.0
XLOC_046883	ROCK2	chr11	86576757	86577796	3.28192	0.490795	0.641811	1.39729	0.834254	0.851581	0.819126	0.656905	0.860205
XLOC_046884	ROCK2	chr11	86577880	86578197	4.52347	2.69753	10.58	4.85463	4.12909	4.59741	2.69571	3.90453	2.73298
XLOC_046885	ROCK2	chr11	86578819	86579133	13.827	2.74843	10.7796	2.06111	2.80327	1.87304	1.95944	0.883339	1.59092
XLOC_046886	ROCK2	chr11	86579241	86580995	2.71806	2.43972	2.4815	1.90915	1.84793	1.79079	1.49028	1.32074	1.8627
XLOC_046887	ROCK2	chr11	86581378	86581544	61.5548	28.12	24.5135	17.6404	11.1302	15.4473	7.0028	18.9824	34.6919
XLOC_046888	ROCK2	chr11	86581847	86582177	8.3723	3.74524	13.0573	4.11772	3.82955	3.83501	2.86572	2.01427	2.89295
XLOC_046889	ROCK2	chr11	86582495	86582757	47.7149	22.3563	21.2537	6.11633	8.70969	4.81291	3.38183	5.77135	5.86204
XLOC_046890	ROCK2	chr11	86582945	86583570	12.3772	11.0978	14.5118	7.06708	4.57162	4.15628	2.49394	3.3763	4.44887
XLOC_044342	ROCK2;C11H2orf50;PQLC3;C11H2orf50	chr11	86583726	86690258	26.3517	18.8353	17.1997	26.7361	27.5564	27.3154	34.5004	33.3823	33.1458
XLOC_044976	ROCK2;C11H2orf50;PQLC3;C11H2orf50	chr11	86583726	86690258	65.9067	66.6434	57.878	24.2119	25.6724	27.1345	24.7149	26.4766	28.9654
XLOC_044977	ROCK2;C11H2orf50;PQLC3;C11H2orf50	chr11	86583726	86690258	306.981	348.423	293.176	836.599	771.005	813.207	845.198	884.136	900.595

XLOC_046891	ROCK2;C11H2orf50;PQLC3;C11H2orf50	chr11	86583726	86690258	5.37784	2.67817	5.60324	1.92671	3.61707	2.58909	2.39721	2.1274	3.5894
XLOC_046892	ROCK2;C11H2orf50;PQLC3;C11H2orf50	chr11	86583726	86690258	3.25557	3.24481	2.54592	1.45895	1.60923	2.6987	2.45021	1.40727	2.74682
XLOC_046893	ROCK2;C11H2orf50;PQLC3;C11H2orf50	chr11	86583726	86690258	11.2628	4.47959	2.92847	2.01543	3.7316	3.06365	3.55805	1.81466	2.59756
XLOC_044340	ROCK2;E2F6	chr11	86360067	86544891	11.8906	13.4262	13.6873	9.86906	12.9503	12.2167	11.226	8.9481	13.17
XLOC_044975	ROCK2;E2F6	chr11	86360067	86544891	42.625	43.0593	39.5003	58.6394	51.5326	55.7186	55.413	59.3155	51.4946
XLOC_046874	ROCK2;E2F6	chr11	86360067	86544891	5.51599	9.06891	8.6247	1.9765	1.61062	1.85421	1.98199	2.46724	2.044
XLOC_046875	ROCK2;E2F6	chr11	86360067	86544891	0.0	3.88852	10.1658	5.84744	0.981502	2.63289	2.16374	4.30337	1.68284
XLOC_046876	ROCK2;E2F6	chr11	86360067	86544891	4.52347	13.4879	17.6338	3.23642	2.75272	2.2987	1.15531	2.60302	4.29469
XLOC_046877	ROCK2;E2F6	chr11	86360067	86544891	4.05791	7.27359	7.92572	0.908155	2.04313	2.09014	2.70168	0.600258	3.17677
XLOC_046878	ROCK2;E2F6	chr11	86360067	86544891	8.2772	4.93732	12.9098	0.370083	3.15604	2.94957	2.48085	0.398476	2.14534
XLOC_046879	ROCK2;E2F6	chr11	86360067	86544891	0.0	5.39535	7.05377	1.21366	1.37636	4.59741	2.31061	1.73535	0.390426
XLOC_046880	ROCK2;E2F6	chr11	86360067	86544891	4.60129	3.44219	5.4018	6.07168	8.72326	8.21208	8.00756	7.31344	7.50803
XLOC_046881	ROCK2;E2F6	chr11	86360067	86544891	4.44099	1.3245	6.92545	5.95739	4.73191	5.41827	2.64982	2.55765	6.51752
XLOC_039599	ROPN1	chr1	68955669	68956577	4.81853	3.74653	1.50747	0.0	0.0	0.0	0.0	0.0	0.0
XLOC_039600	ROPN1	chr1	68986107	68986555	0.0	4.46102	0.0	0.0	0.0	0.0	0.0	0.0	0.0
XLOC_086550	ROR2	chr8	87472797	87524419	20.4308	24.7866	11.2888	13.8833	14.7692	18.7263	11.8723	18.7454	8.48915
XLOC_086999	ROR2	chr8	87468860	87472334	2.27013	6.90592	2.66493	1.86608	2.0789	2.12642	1.86964	2.28435	1.42372
XLOC_088506	ROR2	chr8	87525162	87526039	1.00523	3.3066	2.35853	0.900841	1.02059	1.14635	0.909225	0.803001	0.526595
XLOC_088507	ROR2	chr8	87529445	87530066	3.12101	4.66394	1.21974	0.139764	0.485325	0.48367	0.419142	0.154767	0.0
XLOC_053503	RP1;ATP6V1H;MRPL15;TCEA1;LYPLA1;RGS20;SOX17	chr14	23494568	24003901	9.23898	7.77752	3.61447	0.0826246	0.0	0.0	0.0	0.0	0.0
XLOC_065857	RPA2	chr2	126035510	126088321	1.72421	0.0	0.0	1.85279	0.0	0.889777	0.615638	0.341325	0.750726
XLOC_069899	RPAP2	chr3	51221259	51252079	41.4579	37.2488	45.3413	16.7523	11.8234	15.0874	16.7978	32.3878	10.5657
XLOC_071005	RPAP2	chr3	51200917	51201610	0.0	0.807524	0.0	0.725982	1.05193	0.558903	0.485571	0.268599	0.588887
XLOC_071006	RPAP2	chr3	51221259	51252079	3.48565	9.37958	8.17689	2.81085	2.53755	2.52708	2.61971	3.12646	1.01385

XLOC_071007	RPAP2	chr3	51221259	51252079	9.61632	10.677	17.1837	2.58431	3.20914	2.84188	3.9493	2.73114	1.67693
XLOC_071008	RPAP2	chr3	51221259	51252079	4.01583	5.28492	6.91108	2.30372	1.1309	1.83415	1.89587	2.49233	0.842107
XLOC_071009	RPAP2	chr3	51221259	51252079	4.30988	6.42609	6.72111	1.92707	2.62634	0.438453	1.4724	0.414235	1.48875
XLOC_071010	RPAP2	chr3	51221259	51252079	0.0	1.25561	3.28314	1.12951	1.28368	2.14262	0.720299	1.21524	0.363685
XLOC_071011	RPAP2	chr3	51221259	51252079	0.0	0.0	0.0	1.05205	0.0	0.0	0.0	0.0	0.993981
XLOC_071012	RPAP2	chr3	51221259	51252079	5.2505	2.87886	5.47587	0.470589	0.68414	0.453973	0.634732	0.875191	0.84083
XLOC_071013	RPAP2	chr3	51252440	51252744	0.0	2.93262	7.66783	0.879992	1.49293	1.49717	0.832673	0.939861	2.1207
XLOC_071014	RPAP2	chr3	51252841	51253344	6.28069	3.7523	1.63547	2.81103	1.78294	2.80259	3.34134	1.85666	2.91303
XLOC_076800	RPAP3	chr5	32789195	32854307	22.9925	24.4935	26.8889	23.3184	23.8742	23.1666	23.6674	23.3333	24.369
XLOC_077630	RPAP3	chr5	32789195	32854307	5.98994	0.893835	4.67467	4.28595	2.30244	2.7607	3.39723	2.62773	5.71181
XLOC_077631	RPAP3	chr5	32789195	32854307	2.83135	0.845033	4.41951	0.759846	1.74332	2.32184	1.98183	1.93561	1.47349
XLOC_077632	RPAP3	chr5	32789195	32854307	0.0	1.0809	2.82637	2.26814	2.21831	1.84932	1.87706	2.80612	3.76205
XLOC_077633	RPAP3	chr5	32789195	32854307	2.19568	3.93413	1.71472	2.35791	1.18879	1.12965	1.94291	2.16052	1.33603
XLOC_077634	RPAP3	chr5	32789195	32854307	4.03568	0.0	6.30512	0.722591	1.25046	0.62367	0.895748	0.398019	0.526695
XLOC_061810	RPH3A	chr17	63685309	63686507	0.0	0.0	0.0	1.43316	0.489792	1.15521	0.316165	0.209016	0.97211
XLOC_064205	RPH3AL	chr19	22993169	22996122	0.0	0.0	0.873502	1.41249	1.98923	0.636496	0.100907	0.704852	0.80216
XLOC_044737	RPIA;EIF2AK3	chr11	46979181	47379558	0.658392	0.19695	0.0	1.41656	0.67032	1.09446	0.719221	0.594207	2.30226
XLOC_041506	RPL10L	chr10	39732212	39875270	28.9724	34.0814	34.0818	19.6644	22.1757	22.8448	23.6978	20.6972	25.1284
XLOC_042604	RPL10L	chr10	39732212	39875270	0.0	0.0	3.35826	2.50125	2.99424	3.76166	4.56966	3.17522	3.17724
XLOC_042605	RPL10L	chr10	39732212	39875270	4.91441	3.92066	5.98167	3.47599	3.59547	3.1792	4.5827	3.01585	2.81753
XLOC_042606	RPL10L	chr10	39732212	39875270	3.90455	7.58236	6.10131	2.62171	4.69082	3.21937	5.90058	2.89068	3.73702
XLOC_042607	RPL10L	chr10	39732212	39875270	9.63169	8.62939	7.51929	3.482	2.86685	3.86733	7.77031	0.891656	4.96291
XLOC_042608	RPL10L	chr10	39732212	39875270	0.0	2.29901	3.00579	2.0676	2.65003	1.57163	1.32601	0.0	3.66527
XLOC_042609	RPL10L	chr10	39732212	39875270	0.0	3.39337	8.87364	3.30488	4.15611	3.49604	2.73514	2.77581	5.66943
XLOC_042610	RPL10L	chr10	39732212	39875270	4.57405	4.78667	1.7884	0.71723	2.76542	2.36895	2.1665	1.36796	1.69641
XLOC_065118	RPL19	chr19	40330364	40331102	0.0	0.0	0.0	0.558143	0.291357	0.0	0.673182	0.0	0.0
XLOC_065119	RPL19	chr19	40332575	40332959	0.0	0.0	0.0	0.57107	0.490184	0.326642	0.0	0.0	0.0
XLOC_065120	RPL19	chr19	40333024	40333495	0.0	0.0	3.60405	1.03244	0.891644	0.23728	0.407585	0.0	0.200496
XLOC_065121	RPL19	chr19	40334043	40336448	0.32179	0.57775	0.755548	0.28858	0.328512	0.335029	0.41269	0.259226	0.25349
XLOC_048990	RPL21	chr12	32852510	32853577	2.38566	0.951393	0.622067	9.40889	10.3269	9.32807	11.2643	9.07551	15.9811
XLOC_048991	RPL21	chr12	32853795	32854508	1.30203	0.778485	1.01799	0.816523	0.913001	0.808411	1.28839	0.777243	0.567783
XLOC_048992	RPL21	chr12	32854571	32856173	0.0	0.149796	1.17535	0.538709	0.706709	0.833116	0.457123	0.150917	0.613126

XLOC_048993	RPL21	chr12	32856247	32857263	0.0	0.0	0.658992	0.528573	0.329405	1.04913	0.61144	0.0842904	0.735985
XLOC_044648	RPL31;TBC1D8;NPAS2	chr11	5969483	6065654	1.51677	0.964608	0.871038	4.80681	6.16407	5.94279	6.78696	8.89608	7.73468
XLOC_041518	RPL36AL;LRR1	chr10	42679068	42702478	4.72859	4.70285	3.90944	10.9046	10.2854	8.78579	12.1884	11.3977	16.3744
XLOC_059875	RPL6	chr17	63862768	64146829	21.5854	22.6099	28.9983	23.6918	20.1879	23.0218	24.4668	26.5742	26.6373
XLOC_052355	RPN2	chr13	66849648	66850222	1.73663	2.07577	1.35715	5.13181	3.10119	4.83904	5.42441	6.35904	3.93172
XLOC_052356	RPN2	chr13	66850423	66850725	4.9852	2.97239	0.0	8.47397	9.83198	7.07985	8.01031	11.9004	10.7461
XLOC_050572	RPS21	chr13	55343866	55362977	2.15055	0.695872	0.279903	0.722031	0.576546	0.896169	0.631759	0.947896	0.815059
XLOC_050867	RPS21	chr13	55343866	55362977	16.5745	21.9887	21.3834	31.349	29.4753	27.4093	29.5842	34.834	32.7604
XLOC_051886	RPS21	chr13	55343866	55362977	0.0	0.704782	9.21541	2.53432	1.64093	1.94097	2.9155	3.94065	3.07555
XLOC_059556	RPS3A	chr17	6673173	6715409	0.504876	0.604201	0.395065	0.452683	0.47507	0.525046	0.368733	0.253622	0.309127
XLOC_089585	RPS6KA2	chr9	103007976	103097434	6.8391	1.69606	5.1783	0.0968928	0.028249	0.062175	0.0	0.0150492	0.0130614
XLOC_091630	RPS6KA2	chr9	103007976	103097434	2.5157	0.501605	0.655956	0.0	0.0	0.0	0.0	0.0	0.0
XLOC_091631	RPS6KA2	chr9	103007976	103097434	0.737812	0.110386	0.288713	0.0	0.0	0.0	0.0	0.0	0.0
XLOC_092259	RPS6KA3;SH3KBP1;HSP90AB1;EIF1AX	chrX	129971482	130461958	10.5821	19.33	16.6565	11.2083	6.93946	9.58192	11.4583	10.7278	8.39782
XLOC_093566	RPS6KA3;SH3KBP1;HSP90AB1;EIF1AX	chrX	129971482	130461958	12.8339	0.0	10.0458	2.34152	1.89484	5.14017	10.1591	0.0	3.30681
XLOC_092148	RPS6KA6	chrX	69360812	69725061	7.68298	7.26342	6.16497	0.0	0.0	0.0	0.0	0.0	0.0
XLOC_064595	RPS6KB1	chr19	11090597	11092306	3.26305	2.37098	2.91822	1.29573	0.840973	1.7456	0.340697	0.374849	0.652471
XLOC_062393	RRAD;CDH16	chr18	34733140	34751568	8.64166	13.3791	8.52704	14.0611	15.3176	12.6046	17.2441	16.9478	18.8934
XLOC_055762	RRM1;STIM1	chr15	51786590	51865541	90.5208	102.666	85.6314	117.792	121.412	137.41	135.375	145.154	116.573
XLOC_044345	RRM2;RRM2;KLF11	chr11	87405065	87555778	28.7243	40.5509	36.7222	13.8687	23.1803	16.6009	23.7185	17.2987	27.7592
XLOC_046919	RRM2;RRM2;KLF11	chr11	87405065	87555778	29.6152	11.7959	0.0	4.46402	13.9471	9.90654	0.795047	3.64842	5.93415
XLOC_046920	RRM2;RRM2;KLF11	chr11	87405065	87555778	19.2858	28.7493	26.3091	15.9575	17.9381	16.6398	19.2084	10.6049	15.3869
XLOC_046921	RRM2;RRM2;KLF11	chr11	87405065	87555778	20.8257	8.28036	0.0	4.9845	5.21486	9.8001	11.4651	11.0923	8.95388
XLOC_046922	RRM2;RRM2;KLF11	chr11	87405065	87555778	9.99598	14.9315	15.6111	16.2784	14.1175	7.02085	19.3046	10.1532	9.44223

XLOC_046923	RRM2;RRM2;KLF11	chr11	87405065	87555778	3.61206	5.93625	4.23381	3.07252	4.34447	2.98087	2.57555	5.53606	2.35834
XLOC_046924	RRM2;RRM2;KLF11	chr11	87405065	87555778	13.7559	13.01	12.5344	7.79692	7.62041	10.6109	12.1532	7.66265	8.16865
XLOC_046925	RRM2;RRM2;KLF11	chr11	87405065	87555778	16.4709	13.7623	15.4225	6.77588	7.83367	4.37897	8.29372	8.00184	6.56198
XLOC_046926	RRM2;RRM2;KLF11	chr11	87405065	87555778	30.8673	19.6715	20.8989	9.57889	9.5618	7.2063	12.7805	7.70873	8.94897
XLOC_046927	RRM2;RRM2;KLF11	chr11	87405065	87555778	9.87182	26.5393	7.7079	9.82085	15.4152	9.90654	19.0811	10.0331	11.8683
XLOC_046928	RRM2;RRM2;KLF11	chr11	87405065	87555778	12.3404	15.5755	19.2951	7.12411	9.60936	10.7789	10.4709	8.04183	5.97774
XLOC_046929	RRM2;RRM2;KLF11	chr11	87405065	87555778	8.02578	20.3801	21.9457	6.82554	8.86052	10.7496	14.9676	14.646	8.90169
XLOC_046930	RRM2;RRM2;KLF11	chr11	87405065	87555778	4.1623	8.68957	6.49148	7.07177	8.88598	2.11863	7.48233	8.01302	2.87661
XLOC_046931	RRM2;RRM2;KLF11	chr11	87405065	87555778	4.20925	6.71068	5.48434	3.26781	6.77216	3.1922	6.42507	4.32205	5.87155
XLOC_058075	RRP15	chr16	22381913	22439641	28.8737	30.6622	38.755	118.304	93.9562	99.9787	123.008	142.589	102.925
XLOC_058660	RRP15	chr16	22381913	22439641	0.0	1.77367	6.1846	4.78351	5.98083	3.87466	8.96761	4.8819	5.5095
XLOC_058661	RRP15	chr16	22381913	22439641	0.0	1.17832	1.02722	8.2393	7.88143	7.06935	6.49904	7.5805	6.875
XLOC_058662	RRP15	chr16	22381913	22439641	0.0	1.75926	4.60076	7.0291	9.27965	7.28241	5.75701	9.29874	5.46496
XLOC_058663	RRP15	chr16	22381913	22439641	0.0	2.14785	1.40427	8.85	8.08855	7.60095	6.56813	8.17486	5.16273
XLOC_058664	RRP15	chr16	22381913	22439641	2.02306	2.42066	2.63793	9.12846	8.81866	8.26653	8.65316	8.4513	8.48858
XLOC_058665	RRP15	chr16	22381913	22439641	5.05378	1.50662	0.0	12.6606	8.43047	10.763	8.11277	9.16298	11.7638
XLOC_058666	RRP15	chr16	22381913	22439641	11.6228	2.31123	6.04344	20.4397	15.6867	18.1682	20.3226	19.825	13.7334
XLOC_058667	RRP15	chr16	22381913	22439641	0.0	1.51298	1.97827	5.89392	7.42783	7.28539	6.46333	6.20841	5.27953
XLOC_058668	RRP15	chr16	22381913	22439641	1.70792	1.53113	5.33901	12.0825	10.213	9.87236	9.30256	8.11602	10.114
XLOC_058669	RRP15	chr16	22381913	22439641	0.0	3.67119	0.0	10.0537	7.25195	12.2846	9.73717	6.72969	7.37359
XLOC_058670	RRP15	chr16	22381913	22439641	0.564368	0.675359	0.88318	6.88152	6.41378	5.86697	5.30098	6.11928	6.26795
XLOC_058671	RRP15	chr16	22381913	22439641	0.0	1.87261	4.89673	2.80598	6.74651	5.13735	6.82751	5.19138	7.06827
XLOC_058672	RRP15	chr16	22381913	22439641	0.0	0.0	0.0	9.41013	7.85285	7.68328	8.16024	6.60532	9.64533
XLOC_058673	RRP15	chr16	22381913	22439641	1.9041	0.759472	0.993171	6.88503	4.87228	5.01238	6.06941	5.6026	6.65884
XLOC_058674	RRP15	chr16	22439945	22441500	0.0	0.309663	0.0	1.16004	1.54191	1.50683	0.425115	0.363907	0.724238
XLOC_063978	RSAD1;ACSF2;CHAD	chr19	36833497	36893509	69.2071	73.7342	67.445	124.748	134.15	135.743	131.746	131.186	143.628
XLOC_064241	RSAD1;ACSF2;CHAD	chr19	36833497	36893509	0.0	0.446608	0.726519	0.630157	0.889837	0.573323	0.818197	0.406721	0.77721

XLOC_089572	RSPH3	chr9	96769697	96782476	1.6756	0.181288	0.474166	0.0	0.0	0.0	0.0	0.0	0.0
XLOC_091529	RSPH3	chr9	96765839	96766218	3.2645	0.974162	0.0	0.0	0.250478	0.0	0.0	0.0	0.0
XLOC_091530	RSPH3	chr9	96769697	96782476	1.23532	0.0	1.93178	0.0	0.0	0.0	0.0	0.0	0.0
XLOC_089797	RSPO3;RSPO3	chr9	24411728	24646355	17.317	16.9564	14.6542	38.3864	40.9738	36.5687	44.8748	39.1363	42.9337
XLOC_090431	RSPO3;RSPO3	chr9	24411728	24646355	3.58517	2.67817	0.0	0.160514	0.973914	0.924673	0.799071	0.53185	0.312168
XLOC_090432	RSPO3;RSPO3	chr9	24411728	24646355	0.0	2.22187	3.48651	0.932166	1.15671	0.461012	0.799727	0.442747	0.907078
XLOC_090433	RSPO3;RSPO3	chr9	24411728	24646355	0.0	2.73093	1.7856	1.023	1.06887	0.709572	1.23608	0.796685	0.597821
XLOC_090434	RSPO3;RSPO3	chr9	24411728	24646355	1.2567	1.37872	1.31128	1.27714	0.788909	1.22043	1.11082	0.884754	0.879663
XLOC_090435	RSPO3;RSPO3	chr9	24411728	24646355	0.0	0.0	0.0	5.56984	11.4243	5.1425	4.94172	10.4016	5.28291
XLOC_062169	RSPRY1;CPNE2;FAM192A;ARL2BP;PLLP;PLLP;CCL22	chr18	25169018	25478661	8.07007	10.3313	8.18421	12.4263	13.5651	11.7552	10.8682	9.60558	12.4211
XLOC_062814	RSPRY1;CPNE2;FAM192A;ARL2BP;PLLP;PLLP;CCL22	chr18	25169018	25478661	0.0	1.10971	0.0	0.16627	0.72022	0.57452	0.661462	0.0	0.969809
XLOC_062815	RSPRY1;CPNE2;FAM192A;ARL2BP;PLLP;PLLP;CCL22	chr18	25169018	25478661	0.0	0.0	1.26781	0.290544	0.25355	0.336455	0.220686	0.405557	0.283215
XLOC_062816	RSPRY1;CPNE2;FAM192A;ARL2BP;PLLP;PLLP;CCL22	chr18	25169018	25478661	0.0	0.806021	0.0	0.72463	1.57498	0.697337	0.242344	0.670267	1.05804
XLOC_061899	RTDR1	chr17	73831402	73832093	0.0	1.6211	0.0	0.60725	1.47816	0.560981	1.70571	2.29151	0.118217
XLOC_059896	RTDR1;SNRPD3	chr17	73804264	73831202	0.843082	1.80948	1.39309	0.812584	0.883057	0.731521	0.848578	1.21002	0.732652
XLOC_041499	RTF1	chr10	36974338	37001891	74.2082	96.0688	79.4692	123.04	114.76	120.882	135.738	143.926	162.25
XLOC_079814	RUFY3	chr6	87887841	87913592	28.0837	26.6596	26.3608	38.0978	37.7134	36.618	34.4429	43.2185	39.9542
XLOC_080208	RUFY3	chr6	87818371	87839402	20.6749	19.5708	20.6992	37.3469	32.744	35.8211	37.2944	43.16	36.269
XLOC_080209	RUFY3	chr6	87843074	87851842	206.646	209.626	194.663	355.814	362.621	338.428	441.244	441.864	448.802

XLOC_081559	RUFY3	chr6	87840191	87840957	8.32032	2.13226	1.85884	2.98194	3.98577	4.55416	4.28587	3.31578	3.00753
XLOC_081560	RUFY3	chr6	87841379	87841621	0.0	2.47683	0.0	1.49502	6.20346	3.33927	2.70683	5.41432	2.85265
XLOC_081561	RUFY3	chr6	87842110	87842250	0.0	129.143	0.0	67.2806	29.3707	67.1615	54.9219	77.8644	21.8781
XLOC_068263	RUFY4	chr2	106876751	106877564	1.10338	0.32992	1.72571	0.19774	0.0	0.22864	0.39848	0.0	0.0
XLOC_065185	RUNDC1	chr19	43659568	43660066	0.0	1.2691	0.0	0.380297	0.0	0.0	0.0	0.0	0.0
XLOC_065186	RUNDC1	chr19	43662683	43663206	3.95797	2.36489	4.63845	0.354334	0.306709	0.20393	0.527507	0.97638	0.344344
XLOC_072569	RUNDC3B	chr4	32615269	32694259	87.4101	112.088	107.008	71.4018	56.1234	65.6303	74.0049	87.5694	63.1288
XLOC_072570	RUNDC3B	chr4	32694381	32776672	75.9939	103.663	97.7793	48.4882	31.7313	43.0375	47.0641	64.9097	30.1574
XLOC_072571	RUNDC3B	chr4	32799935	32801381	5.55262	16.93	6.36115	10.8045	12.8968	10.5095	10.9249	11.6429	13.8059
XLOC_073099	RUNDC3B	chr4	32803747	32817352	5.20379	6.0297	5.5595	8.83529	7.8111	8.05526	6.46621	7.94789	7.72678
XLOC_073923	RUNDC3B	chr4	32615269	32694259	0.0	0.550037	0.0	0.49384	1.42603	0.948173	0.0	0.545067	0.320053
XLOC_073924	RUNDC3B	chr4	32694381	32776672	7.49037	17.8716	23.3733	8.07774	7.29873	3.01767	9.8431	8.41733	12.8768
XLOC_073925	RUNDC3B	chr4	32776920	32777450	1.94179	7.54172	1.51715	2.43382	2.85982	1.20086	3.10765	4.40892	2.53438
XLOC_073926	RUNDC3B	chr4	32801892	32802087	0.0	4.78801	25.0309	14.6754	14.0387	12.7581	12.4576	24.4887	12.3351
XLOC_053408	RUNX1T1	chr14	74732208	74750478	65.751	70.658	46.4256	64.2948	80.058	70.0458	62.8287	51.9103	101.564
XLOC_066541	RUNX3	chr2	128612414	128630014	36.1353	48.0783	39.4295	34.8562	33.718	31.5033	38.9766	37.6659	31.7792
XLOC_066542	RUNX3	chr2	128630225	128687537	71.8538	95.1598	78.9586	56.182	61.5814	61.4781	66.8973	65.6999	55.5452
XLOC_090535	RWDD1	chr9	34579103	34579864	1.19847	3.94149	4.68557	0.0	0.0	0.0	0.0	0.0	0.0
XLOC_084241	S1PR2	chr7	15976430	15976885	0.0	0.0	0.0	0.0	0.187736	0.0	0.428551	0.238611	0.42237
XLOC_083410	S1PR2;ICAM1;MRPL4	chr7	15979948	16047941	2.76301	0.926386	2.45639	1.62759	1.70461	0.950591	2.00976	1.09402	1.55345
XLOC_084242	S1PR2;ICAM1;MRPL4	chr7	15979948	16047941	3.55224	0.0	5.54242	0.952965	0.271988	1.08822	0.0	0.0	0.0
XLOC_084243	S1PR2;ICAM1;MRPL4	chr7	15979948	16047941	0.0	0.0	0.0	0.290647	0.0	1.3296	0.282356	0.0	0.281507
XLOC_084244	S1PR2;ICAM1;MRPL4	chr7	15979948	16047941	1.12923	0.0	0.0	0.607098	0.264309	0.233964	0.407618	0.0	0.0985474
XLOC_084245	S1PR2;ICAM1;MRPL4	chr7	15979948	16047941	2.59008	0.774308	1.01252	0.464081	0.504526	0.670094	0.116512	0.257711	0.225899
XLOC_084246	S1PR2;ICAM1;MRPL4	chr7	15979948	16047941	0.0	0.0	1.25767	0.288221	0.750371	0.166206	0.575753	0.159494	0.140182
XLOC_084247	S1PR2;ICAM1;MRPL4	chr7	15979948	16047941	0.0	0.0	0.0	0.0	0.0	1.10953	0.0	0.0	0.626959
XLOC_084248	S1PR2;ICAM1;MRPL4	chr7	15979948	16047941	0.0	0.323511	0.0	0.484747	0.168889	1.23323	0.293163	0.431728	0.377743
XLOC_084249	S1PR2;ICAM1;MRPL4	chr7	15979948	16047941	2.1119	2.5234	0.0	0.56712	0.653925	0.217461	0.374388	0.62417	0.367294

XLOC_084250	S1PR2;ICAM1;MRPL4	chr7	15979948	16047941	0.0	1.05303	5.50779	0.788893	0.547111	0.727271	0.628704	0.348649	0.306833
XLOC_084251	S1PR2;ICAM1;MRPL4	chr7	15979948	16047941	0.501724	1.05075	0.3926	0.584816	0.314744	0.469603	0.320646	0.302458	0.219429
XLOC_084252	S1PR2;ICAM1;MRPL4	chr7	15979948	16047941	4.12128	2.46393	0.0	0.738375	0.534856	0.142103	0.246831	1.09245	0.359343
XLOC_084253	S1PR2;ICAM1;MRPL4	chr7	15979948	16047941	0.0	0.0	0.0	0.760465	0.189078	0.50218	0.218509	0.120768	0.105785
XLOC_055731	SAAL1	chr15	34875494	34945470	11.5115	13.9095	8.66184	6.31238	8.51009	7.09348	10.0762	6.58848	11.6594
XLOC_055732	SAAL1;SERGEF	chr15	34958915	35073194	22.701	32.4254	30.5683	13.5587	17.8892	14.0982	19.0739	17.5752	25.852
XLOC_082816	SAFB	chr7	19858109	19859098	0.0	0.0	0.0	0.358313	1.0385	0.689688	0.0	0.397783	0.581325
XLOC_083450	SAFB2	chr7	19904949	19921231	58.4987	41.8849	35.2119	17.3942	14.9773	17.5753	17.986	12.7601	26.1968
XLOC_053291	SAMD12	chr14	48120851	48261098	9.43595	13.8216	11.3302	3.47231	5.90789	4.76597	4.74764	5.97172	7.91113
XLOC_054337	SAMD12	chr14	48079132	48079844	26.0877	56.1519	41.8126	45.4571	49.9998	53.7206	42.9437	41.3967	56.3115
XLOC_054338	SAMD12;MIR2489	chr14	48094115	48094895	0.0	0.0	0.0	0.312317	0.181254	0.240674	1.15267	0.23159	1.21662
XLOC_089489	SAMD3	chr9	69289943	69379571	5.23671	4.87219	5.26923	1.22035	1.32128	1.6982	0.70947	0.6321	1.33982
XLOC_091186	SAMD3	chr9	69289943	69379571	0.0	0.855387	0.0	0.256336	0.22273	0.147945	0.128409	0.142131	0.0
XLOC_091187	SAMD3	chr9	69289943	69379571	0.0	0.0	2.23591	1.53737	0.220437	0.293604	0.501035	0.0	0.248428
XLOC_091188	SAMD3	chr9	69289943	69379571	1.43288	0.214302	0.0	0.321117	0.504747	0.148816	0.456044	0.143586	0.0626179
XLOC_091189	SAMD3	chr9	69289943	69379571	4.3231	0.517177	1.35262	0.232484	0.0	0.17943	0.0	0.0	0.226584
XLOC_091190	SAMD3	chr9	69289943	69379571	0.0	0.0	0.0	0.0	0.0971195	0.0	0.0	0.0	0.217376
XLOC_076489	SAMM50	chr5	114923256	114937686	4.41605	2.04207	1.2567	0.467995	0.756149	0.584874	0.88131	0.484629	1.96719
XLOC_076490	SAMM50	chr5	114947615	114979676	0.0	0.0	0.0	0.280419	0.218587	0.344245	0.105985	0.0	0.0915763
XLOC_077005	SAMM50	chr5	114947615	114979676	2.24199	2.01296	2.29497	5.37694	5.13833	5.40451	5.02432	5.34141	5.28639
XLOC_078785	SAMM50	chr5	114947615	114979676	0.0	0.0	0.0	0.0	0.0	0.454229	1.14212	0.0	0.771406
XLOC_083689	SAP30L	chr7	67730778	67759056	5.59226	4.99395	4.81035	3.16415	2.72497	2.88021	1.30021	1.52469	1.63748
XLOC_044492	SARDH	chr11	104584025	104587200	4.04656	1.81329	1.58068	2.89798	4.54521	3.12655	4.31104	2.79355	11.7918
XLOC_044493	SARDH	chr11	104607882	104669136	3.56418	4.34653	5.08166	0.740461	0.637095	1.15634	0.680714	1.0126	1.19423

XLOC_076335	SARNP;DNAJC14;WIBG;MMP19;MGC142702;MGC142702;ORMDL2;BLOC1S1;CD63;RDH5;ITGA7;ITGA7	chr5	57718044	58131878	5.75835	3.19356	5.01368	0.258293	0.0271483	0.24375	0.210521	0.0970231	0.101166
XLOC_077971	SARNP;DNAJC14;WIBG;MMP19;MGC142702;MGC142702;ORMDL2;BLOC1S1;CD63;RDH5;ITGA7;ITGA7	chr5	57718044	58131878	0.812186	0.485839	1.27069	0.0	0.0	0.0843018	0.0	0.0	0.0
XLOC_077972	SARNP;DNAJC14;WIBG;MMP19;MGC142702;MGC142702;ORMDL2;BLOC1S1;CD63;RDH5;ITGA7;ITGA7	chr5	57718044	58131878	0.0	1.72194	3.00219	0.0	0.0	0.0	0.0	0.0	0.0
XLOC_077973	SARNP;DNAJC14;WIBG;MMP19;MGC142702;MGC142702;ORMDL2;BLOC1S1;CD63;RDH5;ITGA7;ITGA7	chr5	57718044	58131878	0.0	1.16179	0.0	0.0	0.0	0.0	0.0	0.0	0.0
XLOC_077974	SARNP;DNAJC14;WIBG;MMP19;MGC142702;MGC142702;ORMDL2;BLOC1S1;CD63;RDH5;ITGA7;ITGA7	chr5	57718044	58131878	0.0	0.0	0.486789	0.0	0.0	0.0	0.0	0.0	0.0

XLOC_077975	SARNP;DNAJC14;WIBG;MMP19;MGC142702;MGC142702;ORMDL2;BLOC1S1;CD63;RDH5;ITGA7;ITGA7	chr5	57718044	58131878	0.0	0.0	0.0	0.0	0.0	0.191994	0.0	0.0	0.0
XLOC_077977	SARNP;DNAJC14;WIBG;MMP19;MGC142702;MGC142702;ORMDL2;BLOC1S1;CD63;RDH5;ITGA7;ITGA7	chr5	57718044	58131878	0.0	0.0	0.0	0.0	0.0	0.0	0.0	0.0	0.140805
XLOC_077978	SARNP;DNAJC14;WIBG;MMP19;MGC142702;MGC142702;ORMDL2;BLOC1S1;CD63;RDH5;ITGA7;ITGA7	chr5	57718044	58131878	0.0	0.0	0.838612	0.0	0.0	0.0	0.0	0.106985	0.0
XLOC_077979	SARNP;DNAJC14;WIBG;MMP19;MGC142702;MGC142702;ORMDL2;BLOC1S1;CD63;RDH5;ITGA7;ITGA7	chr5	57718044	58131878	0.0	0.271565	0.0	0.0	0.0	0.0941953	0.0	0.0	0.0
XLOC_077980	SARNP;DNAJC14;WIBG;MMP19;MGC142702;MGC142702;ORMDL2;BLOC1S1;CD63;RDH5;ITGA7;ITGA7	chr5	57718044	58131878	0.0	0.0	0.602888	0.0	0.0	0.0	0.0	0.0	0.0

XLOC_077981	SARNP;DNAJC14;WIBG;MMP19;MGC142702;MGC142702;ORMDL2;BLOC1S1;CD63;RDH5;ITGA7;ITGA7	chr5	57718044	58131878	0.0	0.0	0.408774	0.0	0.0	0.0	0.0	0.0	0.0
XLOC_077983	SARNP;DNAJC14;WIBG;MMP19;MGC142702;MGC142702;ORMDL2;BLOC1S1;CD63;RDH5;ITGA7;ITGA7	chr5	57718044	58131878	0.0	0.149796	0.0	0.0	0.0	0.0520697	0.0	0.0	0.0
XLOC_077984	SARNP;DNAJC14;WIBG;MMP19;MGC142702;MGC142702;ORMDL2;BLOC1S1;CD63;RDH5;ITGA7;ITGA7	chr5	57718044	58131878	0.0	0.0	1.42555	0.0	0.0	0.0	0.0	0.0	0.0
XLOC_077985	SARNP;DNAJC14;WIBG;MMP19;MGC142702;MGC142702;ORMDL2;BLOC1S1;CD63;RDH5;ITGA7;ITGA7	chr5	57718044	58131878	0.282343	0.168982	0.441981	0.0	0.0	0.0	0.0517667	0.0	0.0
XLOC_077986	SARNP;DNAJC14;WIBG;MMP19;MGC142702;MGC142702;ORMDL2;BLOC1S1;CD63;RDH5;ITGA7;ITGA7	chr5	57718044	58131878	0.0	0.546452	0.0	0.0	0.0	0.0	0.0	0.0	0.0

XLOC_077987	SARNP;DNAJC14;WIBG;MMP19;MGC142702;MGC142702;ORMDL2;BLOC1S1;CD63;RDH5;ITGA7;ITGA7	chr5	57718044	58131878	0.0	0.0	0.0	0.0	0.0	0.0	0.0	0.0641127	0.0
XLOC_077988	SARNP;DNAJC14;WIBG;MMP19;MGC142702;MGC142702;ORMDL2;BLOC1S1;CD63;RDH5;ITGA7;ITGA7	chr5	57718044	58131878	0.0	0.142219	0.371984	0.0	0.0	0.0	0.0	0.0	0.0
XLOC_077990	SARNP;DNAJC14;WIBG;MMP19;MGC142702;MGC142702;ORMDL2;BLOC1S1;CD63;RDH5;ITGA7;ITGA7	chr5	57718044	58131878	0.0	0.966091	1.26334	0.0	0.0	0.0	0.0	0.0	0.0
XLOC_077991	SARNP;DNAJC14;WIBG;MMP19;MGC142702;MGC142702;ORMDL2;BLOC1S1;CD63;RDH5;ITGA7;ITGA7	chr5	57718044	58131878	1.01796	0.304405	0.0	0.0	0.0	0.0	0.0	0.0	0.0
XLOC_077992	SARNP;DNAJC14;WIBG;MMP19;MGC142702;MGC142702;ORMDL2;BLOC1S1;CD63;RDH5;ITGA7;ITGA7	chr5	57718044	58131878	0.0	0.432825	1.13201	0.0	0.0	0.149707	0.0	0.0	0.0

XLOC_077995	SARNP;DNAJC14;WIBG;MMP19;MGC142702;MGC142702;ORMDL2;BLOC1S1;CD63;RDH5;ITGA7;ITGA7	chr5	57718044	58131878	0.0	0.315496	0.0	0.0	0.0	0.0	0.0	0.0	0.0
XLOC_077998	SARNP;DNAJC14;WIBG;MMP19;MGC142702;MGC142702;ORMDL2;BLOC1S1;CD63;RDH5;ITGA7;ITGA7	chr5	57718044	58131878	0.0	0.366197	1.91548	0.10974	0.0	0.0	0.0	0.0	0.0
XLOC_078002	SARNP;DNAJC14;WIBG;MMP19;MGC142702;MGC142702;ORMDL2;BLOC1S1;CD63;RDH5;ITGA7;ITGA7	chr5	57718044	58131878	0.0	0.409104	0.0	0.0	0.0	0.0	0.0	0.0	0.0
XLOC_078003	SARNP;DNAJC14;WIBG;MMP19;MGC142702;MGC142702;ORMDL2;BLOC1S1;CD63;RDH5;ITGA7;ITGA7	chr5	57718044	58131878	1.75654	0.0	0.0	0.0	0.0	0.0	0.0	0.0	0.0
XLOC_078004	SARNP;DNAJC14;WIBG;MMP19;MGC142702;MGC142702;ORMDL2;BLOC1S1;CD63;RDH5;ITGA7;ITGA7	chr5	57718044	58131878	0.0	0.0	1.0424	0.0	0.0	0.0	0.0	0.0	0.0

XLOC_078006	SARNP;DNAJC14;WIBG;MMP19;MGC142702;MGC142702;ORMDL2;BLOC1S1;CD63;RDH5;ITGA7;ITGA7	chr5	57718044	58131878	0.0	0.0	1.81948	0.0	0.0	0.0	0.0	0.0	0.0
XLOC_078009	SARNP;DNAJC14;WIBG;MMP19;MGC142702;MGC142702;ORMDL2;BLOC1S1;CD63;RDH5;ITGA7;ITGA7	chr5	57718044	58131878	0.0	0.0	0.799069	0.0	0.0800691	0.0	0.0	0.0512939	0.044659
XLOC_060294	SART3;ISCU;LOC533308;CMKLR1;SGSM1	chr17	66667020	67354436	8.5973	8.25881	8.90095	0.140692	0.0336358	0.0891729	0.203946	0.0699745	0.1124
XLOC_038116	SATB1	chr1	157109838	157420237	2.02765	4.0846	2.90881	11.5053	10.1867	10.6589	10.6134	9.97555	10.9305
XLOC_038612	SATB1	chr1	157109838	157420237	17.6859	20.2202	20.4035	5.77372	5.71879	6.62441	6.88669	7.43654	5.51853
XLOC_040712	SATB1	chr1	157109838	157420237	3.62784	4.49655	5.27195	2.23046	2.3568	2.53293	2.55431	1.66595	1.49588
XLOC_040713	SATB1	chr1	157109838	157420237	6.63916	4.96423	7.79003	2.75224	3.31015	3.61747	4.0666	3.73706	2.90015
XLOC_042662	SAV1	chr10	43584692	43585415	0.0	0.0	0.0	0.114586	0.0996689	0.132371	0.0	0.0	0.111558
XLOC_055758	SBF2	chr15	43021227	43170827	5.78462	9.86686	8.60223	0.0518779	0.0	0.0	0.0530228	0.0	0.0759561
XLOC_055759	SBF2	chr15	43250023	43282789	67.0509	54.7303	61.9781	178.78	154.576	180.143	182.459	215.194	209.513
XLOC_056466	SBF2	chr15	43021227	43170827	2.08584	4.36603	4.89373	0.0	0.0	0.0	0.0	0.0	0.0
XLOC_056467	SBF2	chr15	43021227	43170827	2.63251	4.72185	1.02909	0.0	0.0	0.0	0.0	0.0	0.0
XLOC_056468	SBF2	chr15	43241934	43242612	0.0	0.830767	2.17269	0.497917	1.08191	1.29346	1.62243	0.828626	0.726929
XLOC_056469	SBF2	chr15	43244256	43246857	0.591956	1.50569	0.694961	0.769773	1.30189	1.14035	1.11218	0.655897	1.29543
XLOC_056470	SBF2	chr15	43247450	43248239	0.0	0.0	1.79122	0.923611	1.25086	1.06771	1.03326	1.25584	1.89898
XLOC_056471	SBF2	chr15	43248426	43248955	0.0	0.0	3.04245	0.871555	0.905495	1.40473	1.90405	2.30635	1.35528
XLOC_056472	SBF2	chr15	43249123	43249882	2.4049	1.43803	2.82068	1.07736	1.21889	1.12057	1.84223	1.6769	2.72765
XLOC_082989	SBNO2	chr7	45215749	45239147	16.922	19.2948	13.8306	75.1026	75.829	79.2175	67.8622	86.0028	71.4657
XLOC_083626	SBNO2	chr7	45207522	45212579	1.15076	1.94588	5.03891	1.83655	2.54442	3.03063	2.68713	1.85951	2.19351
XLOC_083627	SBNO2	chr7	45215749	45239147	1.69953	0.0	0.728333	1.85185	1.60969	1.25041	0.703932	1.79252	0.90341

XLOC_084831	SBNO2	chr7	45215749	45239147	0.0	0.0	3.42134	3.13997	2.67237	2.67722	1.12289	2.94963	3.78826
XLOC_055423	SC5DL;TBCEL;SORL1	chr15	32244809	32691843	5.23298	10.7533	7.42971	0.120448	0.201398	0.229543	0.134833	0.147828	0.135078
XLOC_056289	SC5DL;TBCEL;SORL1	chr15	32244809	32691843	0.0	0.966099	2.52662	0.0	0.0	0.0	0.0	0.0	0.0
XLOC_056290	SC5DL;TBCEL;SORL1	chr15	32244809	32691843	2.19672	2.62736	0.858971	0.0	0.0	0.0	0.0	0.0	0.0
XLOC_056291	SC5DL;TBCEL;SORL1	chr15	32244809	32691843	2.25846	2.02584	1.76612	0.0	0.0	0.0	0.0	0.0	0.0
XLOC_056292	SC5DL;TBCEL;SORL1	chr15	32244809	32691843	2.51566	1.7556	1.31191	0.0	0.0	0.0	0.0	0.0	0.0
XLOC_076291	SCAF11	chr5	34370378	34488063	52.1409	61.5177	50.0412	20.5478	18.3207	19.8695	22.1924	27.7341	15.3912
XLOC_077696	SCAF11	chr5	34370378	34488063	4.12438	4.93537	7.8371	1.42625	1.47743	1.40855	1.66489	0.946154	1.75171
XLOC_077697	SCAF11	chr5	34370378	34488063	5.40852	3.41575	1.88078	0.808153	1.88344	1.24916	1.2596	1.32671	1.47129
XLOC_077698	SCAF11	chr5	34370378	34488063	1.81508	2.71172	1.41837	0.487565	0.56335	0.936147	0.9706	0.358918	0.632063
XLOC_077699	SCAF11	chr5	34370378	34488063	4.68152	1.39918	9.75792	0.838586	0.242662	0.0	1.39721	0.464302	0.543917
XLOC_077700	SCAF11	chr5	34370378	34488063	4.16302	1.24358	1.62611	0.0	0.483503	0.643076	0.184692	0.0	0.543116
XLOC_077701	SCAF11	chr5	34370378	34488063	0.0	3.53164	2.30882	0.529172	0.682435	0.303036	0.774978	0.0	1.28239
XLOC_077702	SCAF11	chr5	34370378	34488063	0.0	1.17308	0.0	0.0	0.229817	0.305057	0.266273	0.0	0.0856517
XLOC_077703	SCAF11	chr5	34370378	34488063	4.86266	6.53447	1.89873	0.870269	2.25283	3.49793	1.50003	2.62472	3.37901
XLOC_077704	SCAF11	chr5	34370378	34488063	7.13942	7.45497	5.56965	0.638432	1.91304	1.45792	0.308641	1.03736	2.78022
XLOC_044389	SCAI	chr11	96007165	96008217	0.0	1.15278	0.0	2.8501	1.12933	1.49902	0.697868	1.05905	1.34645
XLOC_045025	SCAI	chr11	95956288	96007090	13.0448	13.7913	13.9798	64.9675	49.3442	61.039	64.4578	96.969	39.9866
XLOC_047079	SCAI	chr11	96008269	96009390	0.750647	0.449054	0.0	0.336437	0.176233	0.779441	0.409298	0.902267	0.327997
XLOC_047080	SCAI	chr11	96009674	96010707	1.65221	0.247078	0.0	0.518318	0.12922	0.171478	0.0	0.413351	0.144347
XLOC_047081	SCAI	chr11	96010815	96011561	2.45803	0.36744	0.0	0.110113	0.0	0.0	0.0	0.122385	0.0
XLOC_044390	SCAI;PPP6C	chr11	96035007	96065113	11.0293	17.7217	14.2399	13.5705	12.7922	12.5049	15.9299	15.6916	10.5277
XLOC_045026	SCAI;PPP6C	chr11	96035007	96065113	52.5463	47.7455	42.8407	47.9902	36.9128	45.3545	49.961	61.4927	34.5007
XLOC_052295	SCAND1	chr13	65743354	65745386	0.0	0.577228	0.60389	0.58817	0.99934	0.602304	0.600125	0.931691	0.506435
XLOC_061601	SCARB1	chr17	53196690	53196824	114.979	36.3834	95.3853	83.868	122.372	141.202	101.673	88.3124	61.4462
XLOC_079837	SCARB2;NUP54	chr6	92774500	92943519	23.3912	14.4765	21.5066	1.51114	0.550162	1.28998	1.83908	3.76049	6.67071
XLOC_080231	SCARB2;NUP54	chr6	92774500	92943519	26.2806	21.489	25.2701	2.67395	2.5593	2.91246	2.58618	2.94455	6.06677
XLOC_081702	SCARB2;NUP54	chr6	92774500	92943519	0.0	11.0923	0.0	0.831019	0.237928	0.0	1.07914	1.2064	2.14725

XLOC_081703	SCARB2;NUP54	chr6	92774500	92943519	8.51883	4.6621	5.74781	0.504397	0.485951	0.85353	0.225053	0.552561	0.280574
XLOC_049354	SCEL	chr12	53139380	53139888	0.0	6.16478	11.2853	0.184734	0.159811	0.0	0.0	0.0	0.0
XLOC_081309	SCFD2	chr6	70274755	70274996	25.1738	40.0615	32.7275	17.3835	26.956	27.8439	26.6575	14.8477	17.301
XLOC_081310	SCFD2	chr6	70429007	70429313	0.0	1.44694	0.0	2.6049	4.78967	2.9555	2.46724	2.31998	0.837172
XLOC_081311	SCFD2	chr6	70429368	70430411	0.816773	2.4429	0.638914	1.97667	1.85268	1.61073	2.44644	3.0246	1.2845
XLOC_081312	SCFD2	chr6	70430853	70431035	0.0	18.6869	0.0	1.92559	7.52789	4.13187	9.5509	9.22821	1.77923
XLOC_042735	SCG3	chr10	58757175	58757695	3.99073	1.19222	0.0	2.14357	2.47366	1.64477	2.12686	2.16539	2.08304
XLOC_042538	SCG5	chr10	30084498	30084824	0.0	0.0	0.0	1.91574	3.59061	2.61557	2.56256	2.88336	2.22015
XLOC_042539	SCG5	chr10	30114021	30114641	0.0	0.0	0.0	0.420198	0.0	0.161569	0.56003	0.310191	1.4989
XLOC_070098	SCMH1	chr3	105704630	105717333	0.721443	0.431596	0.0	1.55212	0.73414	1.12388	0.524784	0.795186	0.945809
XLOC_070465	SCMH1	chr3	105678225	105687542	25.4906	38.8367	29.2037	35.2456	30.5375	33.643	42.6043	42.0101	35.2275
XLOC_071993	SCMH1	chr3	105669473	105672041	2.10021	3.77085	1.40895	0.107629	0.0942619	0.0937247	0.247461	0.0906604	0.078789
XLOC_071994	SCMH1	chr3	105673412	105673906	6.44869	2.56831	0.0	0.577212	0.665387	0.442549	0.571208	0.211671	0.747587
XLOC_071995	SCMH1	chr3	105676010	105676901	0.986042	0.589736	2.31356	0.883663	1.23232	0.817893	0.892186	0.984823	1.54974
XLOC_071996	SCMH1	chr3	105704630	105717333	0.0	0.0	0.0	1.30421	0.41239	0.547744	0.357025	0.13164	0.0
XLOC_071997	SCMH1	chr3	105717910	105718548	0.0	0.449916	1.17665	0.943786	0.468359	0.466716	0.269813	0.149392	0.262373
XLOC_071998	SCMH1	chr3	105721632	105722549	1.90457	0.284783	1.48962	0.597409	1.26486	0.493786	0.517307	0.1903	0.166319
XLOC_071999	SCMH1	chr3	105722900	105723802	0.971472	0.871546	0.0	0.174124	0.758909	0.503676	0.263772	0.0970444	0.424145
XLOC_072000	SCMH1	chr3	105725048	105727016	1.19839	1.79288	2.1883	0.46567	1.0032	0.415695	0.475021	0.281302	0.76899
XLOC_072001	SCMH1	chr3	105727133	105729762	8.19349	5.86697	6.41284	3.72654	4.32122	4.14417	5.49826	4.27416	5.55855
XLOC_065571	SCN1A	chr2	30300484	30359266	38.3157	50.326	42.3717	87.632	83.0156	85.0214	87.2749	95.8106	75.0452
XLOC_065572	SCN1A	chr2	30364217	30385807	3.52628	6.64041	5.19629	5.4749	4.43672	4.66685	4.35668	6.30412	5.20731
XLOC_066224	SCN1A	chr2	30300484	30359266	9.96826	12.8089	10.7976	9.20914	7.49041	7.71571	7.28884	9.62447	7.96922
XLOC_067193	SCN1A	chr2	30300484	30359266	3.9767	4.16219	2.33283	4.90019	3.88251	4.01991	3.59793	4.36882	4.51403
XLOC_067194	SCN1A	chr2	30387116	30387489	3.35513	6.00682	2.61785	0.300048	0.771783	1.02889	0.0	0.0	1.16211
XLOC_065579	SCN2A	chr2	30982295	31023071	3.95444	4.4878	3.10645	2.70033	2.4405	1.71392	2.45415	2.3876	2.70065
XLOC_066230	SCN2A	chr2	30972752	30982178	9.24415	45.2472	16.548	52.3525	31.2937	34.4523	31.5272	45.1958	38.578
XLOC_066231	SCN2A	chr2	31026472	31142002	10.9571	9.57213	6.14592	19.5766	19.8593	22.3377	23.1303	25.0767	16.8367
XLOC_067214	SCN2A	chr2	31026472	31142002	0.0	1.74455	4.5618	6.27361	8.99142	8.68372	9.19148	5.98785	7.34874
XLOC_067215	SCN2A	chr2	31026472	31142002	8.13852	2.42733	6.34679	8.00565	9.62421	9.53257	9.07012	10.194	11.6043
XLOC_067216	SCN2A	chr2	31026472	31142002	4.02945	0.803055	4.20032	4.57243	3.97529	4.16875	4.9501	3.87334	3.86528
XLOC_076241	SCN8A	chr5	28316867	28389534	28.9502	21.4451	27.1558	26.4868	32.3801	33.3672	40.2666	32.8081	27.2281

XLOC_077492	SCN8A	chr5	28316867	28389534	1.4716	0.879719	0.0	2.24083	2.97699	2.89031	3.03527	2.77605	2.69358
XLOC_077493	SCN8A	chr5	28316867	28389534	2.18199	1.30348	1.70439	2.3436	2.701	2.47024	3.09059	3.86603	3.60418
XLOC_065565	SCN9A	chr2	30061694	30152254	47.8135	48.2684	60.959	215.993	187.146	204.447	213.843	271.304	188.094
XLOC_065566	SCN9A	chr2	30061694	30152254	1.29862	1.36431	2.28261	5.53432	5.86982	5.06868	5.32579	4.71792	4.66548
XLOC_065567	SCN9A	chr2	30171416	30178479	2.30606	3.85871	3.39822	8.5426	5.628	7.3921	4.90558	8.90321	6.67677
XLOC_066220	SCN9A	chr2	30061694	30152254	3.54823	3.64868	3.28623	15.7888	15.1676	15.2711	17.1987	15.9045	17.5957
XLOC_066221	SCN9A	chr2	30061694	30152254	6.90041	9.42789	8.17141	4.36498	4.41138	3.86883	3.3029	3.80268	3.9517
XLOC_066222	SCN9A	chr2	30171416	30178479	7.08939	10.2181	8.49926	34.3753	28.4642	33.0493	32.0424	41.948	31.2552
XLOC_067180	SCN9A	chr2	30061694	30152254	0.0	0.0	0.0	7.72364	4.66364	5.78838	5.16762	4.75013	4.88739
XLOC_067181	SCN9A	chr2	30061694	30152254	0.0	0.236829	0.309708	6.88461	5.03166	5.148	5.90108	5.73323	2.70113
XLOC_067182	SCN9A	chr2	30061694	30152254	23.7789	22.5639	22.7737	209.406	173.283	200.447	219.958	292.313	211.205
XLOC_067183	SCN9A	chr2	30061694	30152254	5.89186	0.879339	4.59882	14.2296	12.915	12.3749	13.8938	12.6447	16.6035
XLOC_067184	SCN9A	chr2	30061694	30152254	0.0	0.0	0.0	2.24161	2.08185	1.47597	2.07282	1.23805	1.40126
XLOC_067185	SCN9A	chr2	30061694	30152254	2.13196	2.23124	1.66727	0.573132	0.582583	0.441944	0.0964324	0.319127	0.744493
XLOC_067186	SCN9A	chr2	30061694	30152254	1.20646	0.36071	1.88672	0.21619	0.0	0.124992	0.0	0.0	0.0
XLOC_067187	SCN9A	chr2	30061694	30152254	0.0	0.458498	0.0	0.412189	0.477348	0.158605	0.274995	0.15225	0.668527
XLOC_067188	SCN9A	chr2	30159494	30160374	4.00422	3.29289	3.13166	2.78102	2.26732	1.66054	2.80711	2.09923	2.97169
XLOC_087213	SCRG1	chr8	5639627	5641272	73.9035	73.6067	71.9078	146.219	156.833	152.516	173.038	138.763	162.043
XLOC_072688	SCRN1	chr4	66827557	66841909	1.18386	0.283427	1.11196	0.106177	0.0372129	0.0	0.195518	0.0	0.0621914
XLOC_074409	SCRN1	chr4	66841967	66842589	0.0	0.930783	1.21712	0.0	0.0	0.482639	0.0	0.0	0.0
XLOC_074410	SCRN1	chr4	66843033	66847220	0.35896	0.268573	0.140491	0.0482943	0.0141108	0.0748121	0.0659413	0.0	0.0
XLOC_066989	SCRN3	chr2	22410401	22410756	18.2983	2.18353	5.70956	3.59979	3.35999	1.86745	3.78958	4.24985	2.21623
XLOC_076856	SCYL2	chr5	64594633	64610626	0.39922	0.0	0.0	4.04611	2.112	1.34554	3.62426	3.10344	5.2255
XLOC_058126	SCYL3	chr16	38297777	38302060	0.0	0.953327	1.24659	0.714207	0.371918	0.329488	1.42705	0.316229	0.416857
XLOC_058127	SCYL3	chr16	38321380	38341800	0.0	1.64286	0.537096	0.430799	0.32252	0.356554	0.3123	0.619362	0.420049
XLOC_081698	SDAD1	chr6	92564497	92565204	18.4275	14.1655	6.17452	27.5928	30.4546	34.5934	37.7614	35.4827	29.1569
XLOC_081699	SDAD1	chr6	92565431	92565674	8.21286	24.5035	12.8114	29.5756	26.3993	23.9557	26.8007	21.438	25.4018
XLOC_044937	SDC1	chr11	78561480	78696019	15.4975	12.7663	8.19563	15.1474	14.1033	14.9469	17.7269	15.6534	14.3739
XLOC_046658	SDC1	chr11	78696186	78697316	17.8488	16.6838	22.1083	19.3996	19.382	17.9937	17.2364	15.8684	16.5085
XLOC_053509	SDCBP	chr14	26412761	26535164	34.5172	43.2329	39.2678	15.0095	16.442	15.9268	17.9807	18.8639	17.7349
XLOC_054015	SDCBP	chr14	26412761	26535164	5.54994	1.6542	21.6221	3.47702	2.51736	1.68507	0.931427	1.58196	4.30127

XLOC_060278	SDS;LHX5;SDSL;PLBD2;DTX1;IQCD;C17H12orf52;CCDC42B	chr17	63059144	63499193	10.8942	16.8119	15.6533	5.45061	3.61521	4.79575	6.69435	5.44203	2.85033
XLOC_060279	SDS;LHX5;SDSL;PLBD2;DTX1;IQCD;C17H12orf52;CCDC42B	chr17	63059144	63499193	0.0	0.0	0.0	0.297472	0.129029	0.342968	0.296851	0.0	0.144682
XLOC_061792	SDS;LHX5;SDSL;PLBD2;DTX1;IQCD;C17H12orf52;CCDC42B	chr17	63059144	63499193	1.05438	0.736293	1.37556	0.504376	0.331592	0.34794	0.387407	0.301366	0.277011
XLOC_061793	SDS;LHX5;SDSL;PLBD2;DTX1;IQCD;C17H12orf52;CCDC42B	chr17	63059144	63499193	2.5186	2.00872	1.97011	0.97822	0.525225	0.3485	0.76168	0.42	0.366725
XLOC_061794	SDS;LHX5;SDSL;PLBD2;DTX1;IQCD;C17H12orf52;CCDC42B	chr17	63059144	63499193	0.0	1.01505	0.663712	0.646428	0.266189	0.661831	0.232632	0.29853	0.333931
XLOC_061795	SDS;LHX5;SDSL;PLBD2;DTX1;IQCD;C17H12orf52;CCDC42B	chr17	63059144	63499193	1.22699	0.708559	0.694965	0.477513	0.162736	0.441475	0.576292	0.316268	0.37099
XLOC_061796	SDS;LHX5;SDSL;PLBD2;DTX1;IQCD;C17H12orf52;CCDC42B	chr17	63059144	63499193	0.946993	0.850234	1.77904	0.594562	0.416901	0.315765	0.295682	0.362957	0.514155
XLOC_061797	SDS;LHX5;SDSL;PLBD2;DTX1;IQCD;C17H12orf52;CCDC42B	chr17	63059144	63499193	1.01973	0.305081	0.398969	0.411434	0.279848	0.636254	0.279254	0.25611	0.40137
XLOC_061798	SDS;LHX5;SDSL;PLBD2;DTX1;IQCD;C17H12orf52;CCDC42B	chr17	63059144	63499193	0.822225	0.943323	2.14546	0.467088	0.323328	0.285679	0.352714	0.345769	0.444099

XLOC_061799	SDS;LHX5;SDSL;PLBD2;DTX1;IQCD;C17H12orf52;CCDC42B	chr17	63059144	63499193	1.23438	0.812675	1.93232	0.464968	0.368577	0.257151	0.430291	0.174177	0.345831
XLOC_061800	SDS;LHX5;SDSL;PLBD2;DTX1;IQCD;C17H12orf52;CCDC42B	chr17	63059144	63499193	0.751706	0.719922	2.23603	0.606821	0.248275	0.501419	0.207246	0.227564	0.355517
XLOC_061801	SDS;LHX5;SDSL;PLBD2;DTX1;IQCD;C17H12orf52;CCDC42B	chr17	63059144	63499193	0.0	1.76201	3.84025	0.880067	0.536962	0.407291	0.444309	0.588516	0.600243
XLOC_061802	SDS;LHX5;SDSL;PLBD2;DTX1;IQCD;C17H12orf52;CCDC42B	chr17	63059144	63499193	1.67495	1.14571	1.87288	0.429205	0.470076	0.598201	0.395157	0.26531	0.398049
XLOC_064051	SEC14L1	chr19	55298248	55625368	10.2372	9.67912	14.8558	7.17689	12.4987	9.79881	7.65069	10.1931	7.26
XLOC_064320	SEC14L1	chr19	55298248	55625368	12.1735	18.9325	14.5129	25.6586	25.8818	25.7021	25.0003	26.7445	23.4593
XLOC_065324	SEC14L1	chr19	55298248	55625368	1.32825	1.19224	2.42532	3.85095	2.88302	3.82311	3.39921	2.49294	2.59529
XLOC_065325	SEC14L1	chr19	55298248	55625368	1.08391	1.13496	0.848128	0.728863	0.594784	0.901559	0.840521	0.489822	0.426579
XLOC_065326	SEC14L1	chr19	55298248	55625368	2.122	1.69264	0.553374	1.01452	0.775248	0.661195	0.450322	0.425327	0.370943
XLOC_065327	SEC14L1	chr19	55298248	55625368	4.01379	1.20086	2.3556	0.854731	0.865555	0.730489	0.320646	0.856965	0.702172
XLOC_065328	SEC14L1	chr19	55298248	55625368	0.907856	0.747144	1.24356	0.712459	0.606464	0.827541	0.354078	0.457625	0.417321
XLOC_065329	SEC14L1	chr19	55298248	55625368	1.97944	1.03638	0.774464	0.443705	0.388076	0.514659	0.451855	0.348068	0.389576
XLOC_065330	SEC14L1	chr19	55298248	55625368	0.503827	0.452205	0.394244	0.406564	0.434584	0.419161	0.413966	0.55681	0.176275
XLOC_065331	SEC14L1	chr19	55298248	55625368	0.514582	0.923705	1.61061	0.3691	0.363128	0.74914	0.281812	0.516927	0.135023
XLOC_065332	SEC14L1	chr19	55298248	55625368	0.0	0.920056	0.601586	0.413591	0.120358	0.479047	0.209583	0.385052	0.268797
XLOC_065333	SEC14L1	chr19	55298248	55625368	0.396403	1.64913	0.620437	0.702545	0.907357	1.46678	0.518467	0.527455	1.2305
XLOC_065334	SEC14L1	chr19	55298248	55625368	0.725953	0.434444	0.852212	0.488249	0.484418	0.453372	0.498374	0.365317	0.254124
XLOC_059891	SEC14L4	chr17	71615812	71657486	63.409	65.3175	57.5728	121.622	101.797	119.849	150.985	149.489	132.821
XLOC_038400	SEC22A;MYLK;PDIA5;ADCY5;PTPLB	chr1	68080641	68740517	7.88254	10.796	6.16939	0.0	0.0	0.0	0.0	0.0	0.0
XLOC_043330	SEL1L	chr10	93962536	93964388	1.28043	0.766226	2.33804	0.497535	0.468845	0.577301	0.546303	0.214613	0.522806

XLOC_043331	SEL1L	chr10	93964651	93965983	2.46314	0.552639	0.963592	0.220826	0.434186	0.447961	0.336606	0.247132	0.161521
XLOC_057901	SELL;SELE	chr16	38088148	38242982	24.1893	28.7463	24.5253	10.747	11.4811	10.626	13.5377	11.2946	11.4767
XLOC_058935	SELL;SELE	chr16	38088148	38242982	6.6913	4.00032	1.0462	0.959033	0.52115	0.553776	0.481373	1.46385	0.466978
XLOC_058936	SELL;SELE	chr16	38088148	38242982	19.5812	22.1925	21.3792	13.6565	3.88799	9.57915	9.4231	7.18043	7.78676
XLOC_038014	SELT;EIF2A;SERP1	chr1	118388187	118607767	20.352	28.7099	29.9728	17.5938	16.7522	18.7821	24.8794	20.8501	16.7006
XLOC_040127	SELT;EIF2A;SERP1	chr1	118388187	118607767	7.20636	6.4469	22.4724	14.8852	11.8967	10.1674	18.992	13.5223	17.3468
XLOC_040128	SELT;EIF2A;SERP1	chr1	118388187	118607767	11.2844	1.34813	0.0	0.807957	0.52351	1.39301	0.199753	0.887817	0.196152
XLOC_040129	SELT;EIF2A;SERP1	chr1	118388187	118607767	3.89503	4.64716	3.03786	0.696591	1.48759	0.0	2.34421	1.87991	0.673472
XLOC_072580	SEMA3C	chr4	40142994	40198499	15.1577	16.2013	13.4881	31.6771	27.6567	26.5447	28.9292	32.6326	29.7028
XLOC_073111	SEMA3C	chr4	40205003	40239839	2.96274	2.20163	3.233	35.002	19.9798	25.7432	18.8208	45.0602	26.83
XLOC_073112	SEMA3C	chr4	40258737	40270295	36.002	41.5974	34.6894	47.9983	48.8811	46.6428	65.6012	68.8364	56.7025
XLOC_073113	SEMA3C	chr4	40270652	40279433	22.4278	35.4291	35.0589	74.6061	63.6271	69.3305	73.3794	89.405	71.457
XLOC_073978	SEMA3C	chr4	40204095	40204677	0.0	1.01835	0.0	6.5611	4.7638	6.33172	3.19521	8.94077	8.01293
XLOC_073979	SEMA3C	chr4	40252246	40253102	0.0	0.0	0.0	0.371159	0.565933	0.643962	1.49766	0.310064	0.813553
XLOC_073980	SEMA3C	chr4	40258737	40270295	12.6042	7.514	4.91128	11.2963	10.4435	9.54653	6.28831	7.73566	10.3017
XLOC_088551	SEMA4D	chr8	90246475	90248442	0.0	1.43509	0.625575	0.0	0.0627347	0.0415924	0.0365601	0.0804162	0.0349734
XLOC_087005	SEMA4D;CKS2	chr8	90248681	90347943	2.50799	4.38319	2.18358	0.187644	0.10925	0.217424	0.126937	0.0699272	0.0
XLOC_088552	SEMA4D;CKS2	chr8	90248681	90347943	0.0	0.0	3.48647	0.0	0.0	0.0	0.0	0.0	0.0
XLOC_088553	SEMA4D;CKS2	chr8	90248681	90347943	1.23321	0.0	0.0	0.0	0.0	0.127667	0.0	0.122796	0.0
XLOC_088555	SEMA4D;CKS2	chr8	90248681	90347943	0.0	0.470527	0.615307	0.0705049	0.0	0.0	0.0	0.0	0.0
XLOC_088556	SEMA4D;CKS2	chr8	90248681	90347943	0.657794	0.393542	0.0	0.0589698	0.0	0.0	0.0	0.0	0.0
XLOC_041567	SEMA6D	chr10	62920628	62944699	5.95273	3.45967	2.9607	8.03995	6.56396	5.72786	7.71792	10.4313	7.48562
XLOC_041969	SEMA6D	chr10	62812482	62892871	1.36141	1.54528	1.48325	191.371	207.71	193.733	242.512	204.595	180.071
XLOC_042811	SEMA6D	chr10	62812482	62892871	0.0	0.0	0.0	3.06877	2.4865	3.05789	2.02372	5.05931	3.2985
XLOC_042812	SEMA6D	chr10	62812482	62892871	0.0	0.0	0.0	4.88884	4.3933	5.57687	4.28617	5.21426	4.48158
XLOC_042813	SEMA6D	chr10	62812482	62892871	0.0	0.0	0.0	5.20852	4.36549	4.64442	5.68026	5.19048	4.90124
XLOC_042814	SEMA6D	chr10	62812482	62892871	0.0	0.0	0.0	4.66374	4.78812	6.1195	6.11965	3.89404	5.60581
XLOC_042815	SEMA6D	chr10	62812482	62892871	0.0	0.0	0.0	4.96248	6.96385	6.10836	5.61167	6.79405	7.15315

XLOC_042816	SEMA6D	chr10	62812482	62892871	0.0	0.0	0.0	7.19449	8.84005	12.721	4.95256	13.7205	5.79088
XLOC_042817	SEMA6D	chr10	62893063	62893408	0.0	0.0	0.0	1.3784	3.53338	1.96454	1.32601	2.97725	2.66565
XLOC_042818	SEMA6D	chr10	62897299	62897592	0.0	0.0	0.0	6.65184	3.21579	0.537897	0.893986	3.53817	3.20235
XLOC_042819	SEMA6D	chr10	62897999	62898499	0.0	0.0	0.0	3.59176	1.30785	3.26172	1.49755	3.3289	1.65282
XLOC_089281	SENP6	chr9	15493438	15544914	8.88514	5.22767	5.12862	5.66808	5.07095	2.312	4.25697	2.80036	2.27309
XLOC_090297	SENP6	chr9	15545035	15545358	8.7228	6.50273	6.80126	1.95015	3.65317	2.66154	2.97767	0.837938	1.88293
XLOC_037833	SENP7	chr1	46175375	46183039	0.0	1.27919	1.33823	0.0	0.0	0.0	0.0	0.0	0.0
XLOC_037834	SENP7	chr1	46221367	46233856	0.0	1.66246	2.17404	0.0498223	0.0	0.0577712	0.0	0.0	0.0
XLOC_037835	SENP7	chr1	46240778	46242243	3.29147	1.47548	11.5761	0.0	0.0	0.0	0.0	0.0	0.0
XLOC_039245	SENP7	chr1	46184029	46185114	0.0	0.699707	1.22001	0.0698972	0.0	0.0	0.0	0.0	0.0
XLOC_070296	SEP15	chr3	57287100	57295010	17.4208	9.44642	13.6312	0.439292	0.213375	0.56601	0.496635	0.382682	0.38089
XLOC_081718	SEPT11	chr6	93530697	93531051	0.0	0.0	0.0	0.0	0.562761	0.375346	4.44222	0.355876	0.0
XLOC_081719	SEPT11	chr6	93568750	93569447	0.0	0.400772	1.04814	0.0	0.208842	1.52568	1.32566	0.13332	0.818359
XLOC_081720	SEPT11	chr6	93571946	93572427	0.0	0.0	0.0	0.400238	0.518741	0.0	2.17521	0.219948	0.583042
XLOC_081721	SEPT11	chr6	93574112	93574685	0.0	0.0	0.0	0.623533	1.4867	0.718602	0.776722	0.689084	0.757909
XLOC_081722	SEPT11	chr6	93591744	93592186	0.0	0.0	0.0	0.0	0.392285	0.261093	2.01259	0.0	0.441493
XLOC_080232	SEPT11;CCNI	chr6	93597804	93648206	0.0	0.189535	0.0	4.33896	6.42832	2.97842	9.94201	4.04864	3.63676
XLOC_081723	SEPT11;CCNI	chr6	93597804	93648206	0.0	0.0	0.0	0.520904	1.35762	0.450915	2.0867	0.288753	0.380182
XLOC_081724	SEPT11;CCNI	chr6	93597804	93648206	0.0	0.0	0.0	0.86922	1.65569	0.20015	1.20853	0.191692	0.0
XLOC_081725	SEPT11;CCNI	chr6	93597804	93648206	0.0	0.0	0.0	2.01821	1.28534	0.776265	1.48098	0.149087	1.44007
XLOC_081726	SEPT11;CCNI	chr6	93597804	93648206	0.0	0.0	0.0	18.7027	12.5104	9.68498	10.5642	9.95375	17.6449
XLOC_081727	SEPT11;CCNI	chr6	93597804	93648206	0.0	0.0	0.0	1.16868	1.0918	0.579649	1.43417	1.58254	1.38286
XLOC_081728	SEPT11;CCNI	chr6	93597804	93648206	0.0	0.0	0.0	0.599396	1.87614	0.415336	0.962378	0.93154	1.40032
XLOC_081729	SEPT11;CCNI	chr6	93597804	93648206	0.0	0.0	0.0	2.58771	2.56258	1.95765	2.04345	1.84433	7.06966
XLOC_081730	SEPT11;CCNI	chr6	93597804	93648206	0.0	0.0	0.0	1.0131	1.05024	2.32886	0.400244	0.222627	1.57409
XLOC_081731	SEPT11;CCNI	chr6	93597804	93648206	0.0	0.0	0.0	1.57702	2.06758	1.70645	0.908596	1.76547	2.30717
XLOC_076475	SEPT3;WBP2NL	chr5	113490857	113551629	0.0	0.191041	0.0	0.400768	0.550252	0.265434	0.814198	0.640564	0.781655

XLOC_078755	SEPT3;WBP2NL	chr5	113490857	113551629	0.0	0.645908	1.68914	3.09683	1.50592	2.2258	2.10646	2.12903	3.00809
XLOC_059901	SEPT5;UFD1L;CLDN5;CDC45;GNB1L	chr17	74717741	74863570	12.2173	18.6095	15.2812	26.9202	21.4847	26.8964	29.1018	31.7154	25.1059
XLOC_074363	SEPT7	chr4	61671100	61672342	0.0	0.0	0.0	0.418494	0.626702	0.346417	0.485594	0.334342	0.349787
XLOC_089570	SERAC1;GTF2H5	chr9	96119381	96179704	5.16457	5.50242	5.03743	10.6951	11.2991	12.0964	12.0603	12.7407	13.8599
XLOC_055733	SERGEF	chr15	35099065	35155718	9.94179	10.4054	8.92143	4.544	6.2517	4.94763	7.10572	5.93461	7.036
XLOC_056314	SERGEF	chr15	35084357	35085543	0.0	0.0	1.65043	0.126076	0.275264	0.0730394	0.0	0.0	0.184392
XLOC_052481	SERINC3	chr13	73649619	73649907	5.50705	6.5666	0.0	9.85975	19.5767	11.1516	10.1742	7.32775	11.3853
XLOC_052482	SERINC3	chr13	73650033	73650242	0.0	0.0	0.0	8.06654	13.0666	7.59367	2.00293	1.15415	14.1118
XLOC_048754	SERPINE3	chr12	20873162	20874293	0.0	0.444444	0.0	1.06555	1.51176	1.15721	1.41803	0.818659	1.88289
XLOC_048755	SERPINE3	chr12	20875229	20875544	0.0	0.0	0.0	1.63853	3.48278	1.39616	4.6752	1.75611	1.58106
XLOC_048756	SERPINE3	chr12	20876565	20877198	0.0	0.0	0.0	3.26981	4.02241	3.14385	3.13457	0.905601	3.97675
XLOC_055575	SERPING1;YPEL4;MIR130A	chr15	82147243	82212013	71.9373	98.7477	97.9375	59.2074	69.3524	66.4716	64.396	68.2134	56.3401
XLOC_055893	SERPING1;YPEL4;MIR130A	chr15	82147243	82212013	3.22582	3.52659	5.03916	0.802647	0.304241	0.786821	0.498118	0.623682	0.432062
XLOC_055459	SERPINH1	chr15	55487934	55516209	0.0	1.42346	3.72251	0.213276	0.0	0.489969	0.0	0.0	0.0
XLOC_055460	SERPINH1	chr15	55522600	55626336	7.45908	9.35479	8.26239	17.4398	21.2335	19.4739	24.449	16.5047	22.2736
XLOC_055768	SERPINH1	chr15	55522600	55626336	15.3635	23.6266	15.2336	56.8295	49.0908	55.5313	55.4769	53.3033	63.3108
XLOC_056632	SERPINH1	chr15	55522600	55626336	2.20346	0.0	0.861639	0.592401	1.37566	0.685001	0.79577	0.439513	0.192383
XLOC_056633	SERPINH1	chr15	55522600	55626336	4.61193	1.37844	4.80661	1.10162	1.67377	1.58864	3.16695	1.67758	1.87553
XLOC_056634	SERPINH1	chr15	55522600	55626336	0.662038	1.7823	2.0718	1.54308	1.71092	1.65075	2.65145	1.65957	2.25705
XLOC_045069	SET	chr11	99218967	99231516	13.0038	12.1704	12.0574	8.75451	8.37683	8.61953	8.67563	10.6609	8.29925
XLOC_047254	SET	chr11	99218967	99231516	0.0	0.0	0.0	1.03686	0.8452	0.0	2.73119	1.04722	0.0
XLOC_047255	SET	chr11	99218967	99231516	0.70048	3.77157	1.09602	2.44896	0.932291	1.16412	1.08321	1.75518	0.795944
XLOC_060304	SF3A1;CCDC157	chr17	71496072	71526843	18.9933	27.7682	25.6063	33.5339	35.5786	34.3388	39.8719	39.3889	46.5247
XLOC_070116	SF3A3	chr3	108571691	108582068	4.557	8.2946	7.16161	11.0138	11.2044	12.2191	11.4905	17.9759	15.2705
XLOC_070492	SF3A3	chr3	108558576	108562524	18.4096	19.0651	19.3905	12.4848	13.9685	13.9408	14.6918	14.9426	16.2793
XLOC_059815	SFSWAP	chr17	46298195	46302586	1.5957	3.57952	1.24825	0.0	0.0	0.0	0.0	0.0	0.0
XLOC_060202	SFSWAP	chr17	46257839	46283066	21.9658	30.5008	47.714	25.8543	22.4423	22.9	22.0849	22.9855	15.8001
XLOC_060203	SFSWAP	chr17	46302987	46327982	1.20046	1.70748	3.5956	1.25911	1.74084	1.45267	1.06676	1.4286	0.973856

XLOC_089584	SFT2D1;BRP44L	chr9	102835181	102908480	62.7745	82.7225	65.6425	31.8188	30.9582	33.4134	30.1472	36.2306	32.6799
XLOC_041450	SFXN1	chr10	5593859	5604728	5.08771	6.58831	6.62689	0.0	0.0	0.0	0.0	0.0	0.0
XLOC_045468	SFXN5	chr11	11437216	11437635	8.2976	8.25708	8.63693	7.91796	6.81652	9.07744	7.75426	9.19277	9.59865
XLOC_045469	SFXN5	chr11	11462181	11463565	2.35871	0.882035	1.84552	0.528671	0.785522	0.55162	0.37624	0.651007	0.61875
XLOC_053260	SGK3	chr14	32995190	33014004	0.319798	0.428693	0.0	0.258559	0.150695	0.0740326	0.234372	0.143754	0.883564
XLOC_053538	SGK3	chr14	33042613	33076425	4.83392	3.97438	2.8348	0.649652	0.376905	0.500507	0.43559	0.842591	0.632599
XLOC_054165	SGK3	chr14	33014743	33015290	0.0	0.0	0.0	0.16627	0.288088	0.0	0.0	0.0	0.323267
XLOC_042002	SGPP1	chr10	76262507	76345236	11.1083	6.97542	6.14087	4.22174	3.16057	2.6071	2.35004	2.27565	2.03962
XLOC_078736	SGSM3	chr5	112227400	112227962	0.0	0.0	0.0	0.160115	0.138778	0.0	0.0	0.353709	0.0
XLOC_059873	SH2B3	chr17	57402773	57483473	7.05444	1.09774	2.42941	0.531542	0.0886659	1.2049	0.258683	0.284283	0.14824
XLOC_092375	SH2D1A	chrX	8240513	8252678	7.71445	16.5314	15.2644	25.3178	19.2153	21.3473	30.6033	34.8927	34.2607
XLOC_044426	SH2D3C	chr11	98446357	98455368	1.54742	3.8531	3.02752	31.4176	26.9861	33.9452	15.8433	25.2785	22.8338
XLOC_047228	SH2D3C	chr11	98455969	98456575	0.0	0.0	0.0	0.0	0.0	0.333125	0.0	0.319685	0.0
XLOC_068647	SH2D5	chr2	132528043	132529013	0.0	0.532442	0.0	0.159564	0.208753	0.184701	0.0	0.178022	0.155504
XLOC_092470	SH3BGRL	chrX	70459673	70477180	5.34532	4.6902	2.81118	3.42355	3.53076	3.80958	3.46201	3.89261	2.81672
XLOC_040664	SH3BP5	chr1	154096133	154096304	0.0	0.0	0.0	0.0	0.0	0.0	2.04651	14.2586	0.0
XLOC_082968	SH3BP5L	chr7	43931686	43939753	0.0	0.0	0.0	0.155454	0.0	0.0899324	0.0785906	0.173482	0.0756972
XLOC_087532	SH3GL2	chr8	26823259	26823465	119.153	99.1512	93.3026	126.964	112.283	135.18	112.964	194.219	120.542
XLOC_087533	SH3GL2	chr8	26823563	26823714	0.0	78.161	40.9067	130.023	105.377	148.43	132.471	215.452	62.0857
XLOC_093567	SH3KBP1	chrX	130462184	130463403	5.45074	2.85335	2.66525	2.50426	1.65387	1.98179	1.92193	1.98085	1.78674
XLOC_093568	SH3KBP1	chrX	130464074	130464233	0.0	46.6995	0.0	22.1521	8.2399	30.6897	2.87026	6.67108	23.2286
XLOC_093569	SH3KBP1	chrX	130465262	130466035	0.0	1.05402	4.59432	1.05288	1.37472	2.31222	0.953591	0.702558	1.2304
XLOC_041559	SHC4	chr10	61475782	61581210	3.86885	4.77489	5.29805	2.0814	2.18759	2.36437	1.66286	2.29549	2.24242
XLOC_042780	SHC4	chr10	61581286	61581960	1.40036	5.02315	4.37899	2.13251	3.05254	2.0275	1.88616	2.22649	1.22088
XLOC_042781	SHC4	chr10	61582288	61582795	4.1392	1.85472	4.85037	1.66735	1.28208	1.06574	1.65226	1.01997	1.07998
XLOC_062718	SHCBP1	chr18	14968549	14969233	0.0	1.23197	1.07398	0.123063	0.748799	0.142094	0.246831	0.546227	0.239562
XLOC_064214	SHPK;CTNS	chr19	24920210	24961628	16.7483	15.3544	9.41621	22.4264	21.1877	22.4017	22.4755	22.8346	27.1351
XLOC_081707	SHROOM3	chr6	93348025	93349017	0.0	0.259208	0.0	0.233041	0.0677627	0.269786	0.707337	0.173378	0.757086
XLOC_081708	SHROOM3	chr6	93383773	93384673	0.0	0.0	0.0	1.92052	0.532656	1.11105	1.67497	1.26494	1.53102
XLOC_038501	SIAH2	chr1	118299725	118311471	38.4671	46.8304	46.2274	53.3917	46.5313	48.6495	61.1688	67.6545	57.0403
XLOC_037860	SIDT1	chr1	58711267	58715030	4.56175	2.72993	4.76005	0.374981	0.566935	0.553897	0.278285	0.344266	0.432459
XLOC_037861	SIDT1	chr1	58725818	58754536	13.7165	11.4345	9.86314	1.32043	0.871833	1.2267	0.900089	0.492193	0.767964

XLOC_039381	SIDT1	chr1	58715550	58716818	3.25638	2.33788	4.07636	0.467088	0.204035	0.270671	0.355772	0.457207	0.227744
XLOC_039382	SIDT1	chr1	58717098	58718200	2.29725	2.51945	6.58936	0.48048	0.419433	0.397523	0.278277	0.306749	0.267662
XLOC_039383	SIDT1	chr1	58719404	58721158	1.35903	1.76202	3.1905	0.324961	0.390909	0.282757	0.165587	0.227714	0.158527
XLOC_039384	SIDT1	chr1	58721719	58722082	3.51781	1.04955	0.0	0.0	0.0	0.718561	0.304267	0.340955	0.304462
XLOC_039385	SIDT1	chr1	58722225	58723000	0.0	2.10121	1.83178	0.41979	0.365415	0.242609	0.633712	0.350157	0.306608
XLOC_039386	SIDT1	chr1	58725818	58754536	0.773117	1.15621	0.604795	0.138605	0.241971	0.0802659	0.421374	0.154836	0.0
XLOC_039387	SIDT1	chr1	58755263	58755542	41.3135	17.5936	9.19976	2.1146	1.78192	3.58078	0.493155	2.23638	1.52429
XLOC_039388	SIDT1	chr1	58756665	58757150	22.0864	11.2146	15.5265	2.56984	0.854192	2.04546	0.586281	1.73867	1.34395
XLOC_039389	SIDT1	chr1	58757338	58757699	24.8657	16.9568	5.54242	0.0	1.08795	0.725462	1.22828	1.03249	1.84446
XLOC_039390	SIDT1	chr1	58757902	58758090	18.2121	49.3435	42.9984	1.68755	5.32867	1.82201	4.23925	0.0	0.0
XLOC_039391	SIDT1	chr1	58758682	58758861	22.0732	6.66998	0.0	2.06686	1.60757	4.41983	0.0	0.0	1.90422
XLOC_055412	SIDT2	chr15	28297071	28304641	0.0	0.0	0.0	0.15081	0.0	0.0	0.0	0.16677	0.0
XLOC_083665	SIL1	chr7	51997164	52005875	1.05942	1.25533	1.65844	2.82163	2.90443	3.11296	1.99567	2.03434	2.57807
XLOC_084914	SIL1	chr7	51995846	51996508	0.0	0.0	1.12075	0.128422	0.669497	0.0	0.257313	0.0	1.49973
XLOC_045157	SIRT3	chr11	107167113	107189370	23.0236	26.063	17.2457	63.2134	55.9537	58.7519	73.4672	81.8976	68.8487
XLOC_074414	SKAP2	chr4	69691954	69692609	21.8077	29.9848	12.5015	64.7223	56.6805	66.7346	66.5137	80.9953	82.4992
XLOC_038492	SKIL;PRKCI	chr1	97820967	97897276	36.6158	36.7536	43.3588	4.76363	4.12617	4.36334	5.33615	4.97302	6.25148
XLOC_052306	SLA2	chr13	66381405	66381941	0.0	0.0	1.49314	1.36875	1.03717	2.36409	2.21007	0.943617	0.831494
XLOC_069836	SLAMF6	chr3	9251458	9284498	10.2676	10.5805	9.25843	4.44166	4.0787	4.87721	6.01025	6.20508	4.16649
XLOC_070640	SLAMF6	chr3	9284648	9286006	1.80735	1.08135	2.82821	1.45831	1.65211	2.25409	1.75679	1.26955	2.2651
XLOC_070641	SLAMF6	chr3	9286136	9286489	0.0	0.0	2.88376	0.330583	0.282775	0.37722	0.318819	0.0	0.0
XLOC_070642	SLAMF6	chr3	9286688	9287526	1.06285	0.635623	0.831187	0.285725	0.082968	0.220295	0.0960316	0.106053	0.0927768
XLOC_070643	SLAMF6	chr3	9287662	9288136	0.0	0.682563	1.78499	0.613606	0.706676	0.0	0.605821	0.0	0.0
XLOC_070644	SLAMF6	chr3	9288224	9288885	0.0	0.0	2.24595	0.257353	0.447208	0.0	0.644528	0.0	0.375673
XLOC_070645	SLAMF6	chr3	9289546	9289831	0.0	0.0	8.78048	0.504343	0.85152	2.28033	1.41679	0.0	0.485132
XLOC_070646	SLAMF6	chr3	9290047	9290699	0.0	0.0	0.0	0.131015	0.227626	0.151207	0.262378	0.290474	0.0
XLOC_081305	SLC10A4	chr6	68814253	68814813	0.0	0.0	0.0	1.60909	0.418373	0.556167	0.320397	0.355429	0.312893
XLOC_043958	SLC10A6	chr11	5788355	5800832	11.6732	17.5817	11.7178	24.7255	14.2486	20.6709	24.2912	30.535	17.9362
XLOC_044643	SLC10A6	chr11	5800951	5804545	1.65293	1.68407	1.52471	2.06069	1.77702	1.40512	0.989695	1.30973	1.68918
XLOC_059569	SLC10A7	chr17	12004092	12062633	0.663657	1.19129	1.03855	1.95702	0.935599	1.16392	0.543649	0.465779	0.92833

XLOC_062168	SLC12A3;NUP93;CES5A;BREH1;MIR138-2;HERPUD1	chr18	24709475	25133638	3.84394	2.35949	3.61368	4.22219	3.36989	4.25131	6.78501	6.1511	4.64134
XLOC_062192	SLC12A4	chr18	35553419	35557325	11.8796	17.7783	17.5311	211.62	157.392	173.097	197.461	174.446	190.672
XLOC_062901	SLC12A4	chr18	35553419	35557325	2.56038	6.88052	11.9955	38.7163	35.748	40.4885	47.9531	64.2286	39.5685
XLOC_041490	SLC12A6;LPCAT4;NOP10;C10H15orf29;C10H15orf24;CHRM5;TMEM85;LOC515694	chr10	28074750	28596318	10.6345	5.2134	12.4741	25.0822	22.2076	21.3782	22.7043	24.2607	24.8535
XLOC_050964	SLC13A3	chr13	76002695	76018685	1.37958	5.84494	2.45002	5.14109	9.26134	5.31722	9.69052	6.06866	7.57918
XLOC_052540	SLC13A3	chr13	76001196	76001730	0.0	0.0	0.0	0.172	0.595768	0.198041	0.0	0.379405	0.668705
XLOC_052541	SLC13A3	chr13	76001899	76002494	0.0	0.0	0.0	0.296114	0.899131	0.341416	0.295535	0.163781	0.432013
XLOC_052542	SLC13A3	chr13	76031857	76032268	2.85307	0.0	4.45425	0.255221	1.31752	0.584931	0.998319	0.835852	2.47459
XLOC_050965	SLC13A3;TP53RK;SLC2A10	chr13	76061194	76098663	6.5934	4.7403	3.55685	2.83184	2.87311	3.01917	1.9139	2.3118	3.464
XLOC_049751	SLC15A1	chr12	79606267	79607166	0.0	0.29169	0.0	0.0874139	1.14293	0.303421	0.353093	0.194865	0.255513
XLOC_049752	SLC15A1	chr12	79609514	79610295	0.0	0.0	0.0	0.415755	0.995312	0.600726	0.209248	0.346835	0.506116
XLOC_065803	SLC16A14	chr2	118900121	118924857	12.2806	14.1614	12.2151	2.9091	2.69782	2.88929	1.93859	3.17847	1.765
XLOC_068346	SLC16A14	chr2	118900121	118924857	1.6153	3.38192	1.89539	0.506751	0.568589	0.167667	0.366621	0.242528	0.211699
XLOC_068347	SLC16A14	chr2	118900121	118924857	4.11347	5.32845	3.2159	0.491318	0.640624	0.567308	0.615979	0.408909	0.717329
XLOC_076334	SLC16A7	chr5	54053464	54120183	22.5075	23.0468	26.2533	4.17588	4.66518	5.33332	5.9744	6.2071	4.05743
XLOC_077955	SLC16A7	chr5	53998543	54002943	0.852345	2.39786	1.86815	0.3058	0.227856	0.24871	0.469809	0.343957	0.253793
XLOC_077956	SLC16A7	chr5	54004504	54005135	0.0	1.82623	3.58205	0.410451	0.1188	0.473551	2.05268	0.151559	0.133115
XLOC_077957	SLC16A7	chr5	54005408	54006703	2.54327	2.47262	1.49237	0.285005	0.149412	0.132136	0.231618	0.063777	0.166763
XLOC_077958	SLC16A7	chr5	54007493	54007881	0.0	4.68153	0.0	0.0	0.481893	0.321084	1.3655	0.305375	0.271857
XLOC_077959	SLC16A7	chr5	54011708	54012392	1.37376	0.0	3.22194	0.0	0.0	0.284188	0.246831	0.0	0.479123
XLOC_077960	SLC16A7	chr5	54012716	54013643	0.0	1.12434	2.94056	0.168472	0.0734438	0.194963	0.0851169	0.187855	0.0820823
XLOC_077961	SLC16A7	chr5	54018472	54019183	1.30674	0.390647	0.0	0.117067	0.203615	0.270436	0.117539	0.130001	0.0
XLOC_077962	SLC16A7	chr5	54019278	54020549	1.29904	0.777194	0.508173	0.0582288	0.0	0.0	0.177412	0.195422	0.056783
XLOC_077963	SLC16A7	chr5	54023965	54024761	0.0	2.03217	0.0	0.1015	0.0883763	0.117342	0.204434	0.0	0.296565
XLOC_077964	SLC16A7	chr5	54033922	54034801	2.00489	0.599536	0.0	0.0	0.234873	0.0	0.0	0.1001	0.0875235

XLOC_077965	SLC16A7	chr5	54120246	54121289	2.45032	0.73287	1.27783	0.14642	0.127771	0.254326	0.296539	0.163492	0.356804
XLOC_090246	SLC17A5	chr9	13305329	13306560	5.3898	2.01534	2.63548	0.0	0.0	0.0699904	0.0	0.0	0.0
XLOC_090247	SLC17A5	chr9	13306936	13309260	7.01025	4.69486	3.91894	0.0898097	0.0	0.0	0.0611462	0.0336097	0.0
XLOC_090248	SLC17A5	chr9	13309459	13310458	2.57845	2.31348	6.72293	0.0	0.0	0.0	0.0779496	0.0	0.0
XLOC_090249	SLC17A5	chr9	13311112	13312226	2.26842	1.80935	8.28132	0.0	0.0	0.0785119	0.0	0.0	0.0
XLOC_090250	SLC17A5	chr9	13319914	13320630	2.59008	4.25869	5.06262	0.0	0.0	0.0	0.0	0.0	0.0
XLOC_090251	SLC17A5	chr9	13320688	13321665	3.53023	4.22318	6.21293	0.0791009	0.0	0.0	0.0800081	0.0	0.0
XLOC_090252	SLC17A5	chr9	13321809	13323470	5.29136	3.16625	4.51701	0.0	0.0	0.0	0.0	0.0	0.0420791
XLOC_086889	SLC18A1;ATP6V1B2;LZTS1	chr8	67692680	68062169	12.7192	12.7349	10.0528	0.204331	0.114036	0.337747	0.257821	0.336836	0.174809
XLOC_071688	SLC1A7	chr3	93691549	93693339	0.0	0.0	0.0	0.0397248	0.0	0.0	0.0	0.0	0.0
XLOC_044734	SLC20A1	chr11	46193728	46312300	9.02797	9.49595	6.57558	8.24834	9.13182	7.48302	9.78586	11.3688	8.47309
XLOC_045895	SLC20A1	chr11	46193728	46312300	0.0	0.0	0.0	1.62082	0.598738	0.79707	0.454873	2.28152	0.449318
XLOC_045896	SLC20A1	chr11	46193728	46312300	0.0	0.0	0.0	1.21141	1.03842	1.73054	0.880642	3.61572	1.75928
XLOC_070242	SLC22A15;NHLH2;CASQ2	chr3	27416033	27711164	33.1529	29.2763	32.831	4.91932	7.46595	7.59825	7.47479	6.81415	7.64339
XLOC_070737	SLC22A15;NHLH2;CASQ2	chr3	27416033	27711164	4.67159	0.930787	6.08572	0.976247	0.242152	0.965278	1.39431	0.617775	0.678401
XLOC_070738	SLC22A15;NHLH2;CASQ2	chr3	27416033	27711164	8.06368	2.40985	1.26061	0.288863	0.626692	0.66625	1.00985	0.319699	0.561971
XLOC_070739	SLC22A15;NHLH2;CASQ2	chr3	27416033	27711164	3.04846	0.760026	2.78298	1.09331	0.557741	0.58119	1.20592	0.765651	0.799914
XLOC_070740	SLC22A15;NHLH2;CASQ2	chr3	27416033	27711164	0.0	2.71315	1.77401	1.21952	1.05349	1.63525	1.80665	0.893247	2.17111
XLOC_070741	SLC22A15;NHLH2;CASQ2	chr3	27416033	27711164	2.4674	1.84527	1.44791	0.940109	0.918173	0.961548	1.12395	0.618881	1.72582
XLOC_090628	SLC22A16	chr9	40231986	40232151	0.0	0.0	0.0	21.2218	20.6096	34.9898	28.8011	30.6729	21.985
XLOC_090629	SLC22A16	chr9	40232879	40233036	0.0	0.0	0.0	51.5333	35.1937	32.8349	58.1778	42.7005	39.0701
XLOC_090630	SLC22A16	chr9	40233139	40233468	0.0	0.0	0.0	6.40055	5.45566	6.42787	5.7624	4.45588	5.81896
XLOC_090631	SLC22A16	chr9	40233920	40235490	0.0	0.0	0.0	7.52907	4.97828	7.3479	5.98248	7.09846	6.04599
XLOC_090632	SLC22A16	chr9	40235863	40236384	0.0	0.0	0.0	11.7572	8.78855	9.43175	8.48488	14.3317	5.88583

XLOC_090633	SLC22A16	chr9	40236589	40236865	0.0	0.0	0.0	8.66678	4.56021	4.88869	3.53011	7.43691	9.36694
XLOC_090634	SLC22A16	chr9	40237101	40237695	0.0	0.0	0.0	2.52273	2.31728	2.90862	3.5543	3.28298	3.03098
XLOC_090635	SLC22A16	chr9	40238054	40238520	0.0	0.0	0.0	11.1208	12.5004	13.5	15.3148	16.815	9.57476
XLOC_087462	SLC24A2	chr8	24533142	24533940	0.0	0.0	0.0	0.101183	0.0	0.0	0.0	0.0	0.0985474
XLOC_087463	SLC24A2	chr8	24774625	24775075	0.0	2.2153	0.0	1.10639	1.52706	0.762178	0.653328	0.0	1.07382
XLOC_041962	SLC24A5;MYEF2	chr10	62429325	62486775	55.1366	62.9588	64.7653	65.1175	64.4549	65.1871	65.3149	68.2821	65.1085
XLOC_042792	SLC24A5;MYEF2	chr10	62429325	62486775	0.531987	0.636678	1.6651	1.43095	0.625596	0.82988	0.87376	1.2289	0.511808
XLOC_042793	SLC24A5;MYEF2	chr10	62429325	62486775	2.75275	0.822997	3.22815	1.23301	0.964569	1.28167	1.48374	1.0945	0.840063
XLOC_061806	SLC24A6	chr17	63571049	63571160	0.0	379.094	0.0	131.496	319.184	118.932	83.7141	857.91	105.56
XLOC_066165	SLC25A12	chr2	24677848	24697739	3.79367	2.43206	2.96843	0.971818	1.01961	0.845211	0.296655	0.272123	0.379181
XLOC_066166	SLC25A12	chr2	24733964	24762930	2.20864	4.61779	1.72516	1.18608	1.19587	0.454547	0.0	0.652	0.0
XLOC_044428	SLC25A25;PTGES2	chr11	98741402	98771876	36.8901	39.8958	33.1972	8.48122	13.9473	10.3665	8.24867	6.80394	11.905
XLOC_045063	SLC25A25;PTGES2	chr11	98741402	98771876	29.4998	21.7696	24.8509	1.68939	1.76668	2.95366	1.46903	1.99045	1.37211
XLOC_047231	SLC25A25;PTGES2	chr11	98741402	98771876	12.2156	14.0026	9.55351	3.19284	3.41821	3.69339	2.48542	2.94729	2.22187
XLOC_060089	SLC25A31;INTU	chr17	30218956	30576217	11.4003	10.9532	13.0949	4.90164	4.55161	4.43672	5.0185	5.01876	3.25946
XLOC_086466	SLC25A37	chr8	71542825	71548028	10.0202	16.2464	14.8865	46.1061	45.4942	40.2805	36.769	34.3045	37.3098
XLOC_086467	SLC25A37	chr8	71568766	71586540	24.2623	17.3561	15.394	13.6406	16.69	14.4556	11.2924	10.731	13.8524
XLOC_073919	SLC25A40	chr4	32502099	32503165	4.77656	3.80976	3.11376	0.0713579	0.186835	0.0826397	0.289142	0.0796966	0.278238
XLOC_073920	SLC25A40	chr4	32515086	32515850	3.57761	0.713093	9.32483	0.0	0.18599	0.987906	0.214985	0.237611	0.104048
XLOC_073100	SLC25A40;RUNDC3B	chr4	32817460	32950852	2.78829	4.24717	4.70213	13.3244	10.274	8.58545	10.8898	12.6164	8.5947
XLOC_073927	SLC25A40;RUNDC3B	chr4	32817460	32950852	2.00644	1.19883	0.0	2.33505	1.24359	0.413453	2.31643	1.18752	0.872781
XLOC_069841	SLC25A44;SEMA4A	chr3	14641736	14663017	1.36679	1.00821	2.40685	7.88624	10.0004	8.10259	12.5918	10.5157	14.2138
XLOC_083018	SLC25A48	chr7	48884799	48886045	0.0	0.0	0.0	0.250755	0.218639	0.0	0.253409	0.0932093	0.0814493
XLOC_083663	SLC25A48	chr7	48866888	48882048	3.84029	3.70888	2.59556	20.0413	19.8255	23.1744	27.9147	36.5784	13.8738
XLOC_084884	SLC25A48	chr7	48865589	48866830	0.0	0.599025	0.522234	2.6928	2.30002	2.63519	3.3415	4.61814	2.39241
XLOC_084885	SLC25A48	chr7	48866888	48882048	0.0	0.0	0.0	2.13409	1.37586	0.610978	2.60305	1.45402	1.55135
XLOC_084886	SLC25A48	chr7	48882371	48883415	0.0	0.244015	0.0	0.585019	0.319069	0.338721	0.296209	0.244964	0.142561
XLOC_064313	SLC26A11	chr19	52991075	53038723	21.6525	21.5176	21.959	90.5648	81.1015	89.4732	86.8345	87.3115	88.5642

XLOC_072610	SLC26A5;RELN;PMPCB;DNAJC2;PSMC2	chr4	44756570	44933225	19.346	19.4274	18.5079	8.75276	8.80232	9.83131	10.8689	9.88329	10.1865
XLOC_074107	SLC26A5;RELN;PMPCB;DNAJC2;PSMC2	chr4	44756570	44933225	0.0	3.16531	0.0	0.0	4.82368	1.6137	1.34098	0.505473	0.914958
XLOC_074108	SLC26A5;RELN;PMPCB;DNAJC2;PSMC2	chr4	44756570	44933225	0.0	0.0	0.0	0.966543	1.58218	1.06914	1.70843	0.981512	0.0
XLOC_074109	SLC26A5;RELN;PMPCB;DNAJC2;PSMC2	chr4	44756570	44933225	16.9392	2.52581	13.2087	1.51479	0.645466	0.430976	0.362105	0.814678	1.4631
XLOC_074110	SLC26A5;RELN;PMPCB;DNAJC2;PSMC2	chr4	44756570	44933225	6.67936	0.996576	0.0	0.597356	1.02442	0.682835	1.4486	0.648674	2.89204
XLOC_074111	SLC26A5;RELN;PMPCB;DNAJC2;PSMC2	chr4	44756570	44933225	4.51375	3.37034	3.52554	2.42387	1.39603	2.78603	1.99522	1.33172	3.92304
XLOC_074112	SLC26A5;RELN;PMPCB;DNAJC2;PSMC2	chr4	44756570	44933225	1.5741	2.35226	3.69103	1.55078	1.83561	0.975685	2.81791	2.34134	2.74296
XLOC_074113	SLC26A5;RELN;PMPCB;DNAJC2;PSMC2	chr4	44756570	44933225	11.1059	9.94644	4.33501	1.49032	3.20724	3.98637	2.43205	3.25656	4.3358
XLOC_074114	SLC26A5;RELN;PMPCB;DNAJC2;PSMC2	chr4	44756570	44933225	3.82201	7.99335	7.46574	1.53985	2.96334	3.15212	2.04006	2.26469	2.66078
XLOC_074115	SLC26A5;RELN;PMPCB;DNAJC2;PSMC2	chr4	44756570	44933225	0.0	4.15804	0.0	1.55783	1.86827	1.77948	3.61817	2.70247	3.92041
XLOC_074116	SLC26A5;RELN;PMPCB;DNAJC2;PSMC2	chr4	44756570	44933225	1.86662	3.90402	1.45854	2.00553	2.89553	3.65719	1.6619	1.8444	2.92432

XLOC_074117	SLC26A5;RELN;PMPCB;DNAJC2;PSMC2	chr4	44756570	44933225	2.94097	3.07966	4.98625	1.49429	2.95988	2.70189	1.70091	2.01946	2.78695
XLOC_074118	SLC26A5;RELN;PMPCB;DNAJC2;PSMC2	chr4	44756570	44933225	19.0643	5.68354	4.95311	2.27873	3.35035	8.34277	4.22523	2.39948	5.46768
XLOC_074119	SLC26A5;RELN;PMPCB;DNAJC2;PSMC2	chr4	44756570	44933225	5.0493	4.52317	3.9428	1.12952	3.89601	2.59299	1.55492	2.22747	2.84986
XLOC_074120	SLC26A5;RELN;PMPCB;DNAJC2;PSMC2	chr4	44756570	44933225	3.81194	2.27782	7.4461	1.87709	1.77339	1.76845	2.37402	1.31767	1.99036
XLOC_074121	SLC26A5;RELN;PMPCB;DNAJC2;PSMC2	chr4	44756570	44933225	3.89112	3.78178	4.56495	2.00512	2.58373	3.63132	2.72924	1.94347	2.46334
XLOC_044437	SLC27A4;COQ4;TRUB2	chr11	98927821	98975587	14.5063	14.9978	13.2114	21.2883	21.1069	18.0443	22.3115	27.2868	18.1338
XLOC_070092	SLC2A1	chr3	103914940	103967074	14.9644	12.185	10.7967	12.4973	14.1774	14.2979	16.1803	11.3531	17.1261
XLOC_071929	SLC2A1	chr3	103914367	103914866	4.23617	11.3885	11.5821	13.6507	10.329	9.37728	9.38606	8.13736	11.7876
XLOC_050641	SLC2A10	chr13	76098752	76188780	5.49607	6.62082	5.27036	13.4911	13.0077	14.4717	16.4812	15.7355	16.1881
XLOC_052544	SLC2A10	chr13	76098752	76188780	4.4148	0.439913	2.30074	0.922694	1.603	1.36909	2.77134	1.8994	1.28266
XLOC_077786	SLC2A13	chr5	41342095	41342406	32.8843	30.8139	32.9602	129.837	111.729	112.118	111.298	117.395	137.399
XLOC_053422	SLC2A5	chr14	77156788	77722237	2.20302	4.15579	3.60622	34.3604	17.0334	27.5397	23.6092	37.7747	16.1779
XLOC_054893	SLC2A5	chr14	77156788	77722237	0.0	0.695723	0.0	1.45935	1.98032	5.03047	1.02846	1.83144	2.02421
XLOC_054894	SLC2A5	chr14	77156788	77722237	0.0	0.0	0.0	2.78011	3.38012	5.4185	1.13576	2.98437	1.15023
XLOC_054895	SLC2A5	chr14	77156788	77722237	6.02116	0.0	1.17665	2.29205	3.27858	2.02252	3.64252	2.98799	2.2302
XLOC_054896	SLC2A5	chr14	77156788	77722237	0.0	0.0	0.0	2.2205	1.57819	3.79252	0.708929	1.59431	0.357635
XLOC_045135	SLC2A6;TMEM8C;CACFD1;SURF4	chr11	104333754	104468805	40.477	41.3016	37.91	33.3717	33.7152	32.7877	33.1289	36.2595	29.9032
XLOC_047361	SLC2A6;TMEM8C;CACFD1;SURF4	chr11	104333754	104468805	2.07135	2.27186	3.24102	2.5996	3.13546	3.29913	4.27148	2.6992	2.29337
XLOC_047363	SLC2A6;TMEM8C;CACFD1;SURF4	chr11	104333754	104468805	5.92076	2.94807	1.54194	2.82696	2.75297	2.64394	4.20895	2.72658	3.26241

XLOC_047364	SLC2A6;TMEM8C;CACFD1;SURF4	chr11	104333754	104468805	0.784862	0.939013	2.45589	2.53267	2.76323	3.09622	2.63738	2.51466	1.50878
XLOC_047365	SLC2A6;TMEM8C;CACFD1;SURF4	chr11	104333754	104468805	2.16242	0.0	1.68914	0.967764	0.501973	0.22258	0.191497	0.0	0.564017
XLOC_045057	SLC2A8;RPL12	chr11	98152152	98193325	37.4632	35.2155	28.8304	30.9123	35.408	32.0739	29.4669	31.529	32.1769
XLOC_086603	SLC31A2	chr8	104101378	104144510	27.1846	12.6116	22.1956	0.0242056	0.0457817	0.0	0.0	0.0	0.0339954
XLOC_089951	SLC35A1;ORC3;RARS2;C9H6orf165	chr9	63163673	63378215	5.13499	6.78855	4.30146	6.16607	5.83419	5.63503	6.53595	5.7767	5.87537
XLOC_091043	SLC35A1;ORC3;RARS2;C9H6orf165	chr9	63163673	63378215	0.0	1.00114	5.23571	0.300048	0.0	0.0	0.0	0.0	0.0
XLOC_091044	SLC35A1;ORC3;RARS2;C9H6orf165	chr9	63163673	63378215	3.83342	0.0	2.98995	2.0567	1.75762	1.17263	0.989612	0.0	0.994406
XLOC_091045	SLC35A1;ORC3;RARS2;C9H6orf165	chr9	63163673	63378215	1.49663	1.56681	0.585408	0.939102	0.292816	0.46622	0.476059	0.449746	0.52317
XLOC_091046	SLC35A1;ORC3;RARS2;C9H6orf165	chr9	63163673	63378215	1.20646	0.360705	0.943359	0.21619	0.564423	0.749517	0.652328	0.480689	0.736807
XLOC_091047	SLC35A1;ORC3;RARS2;C9H6orf165	chr9	63163673	63378215	2.08584	0.623719	0.0	0.654206	0.814256	0.540479	0.18854	0.312271	0.182088
XLOC_091048	SLC35A1;ORC3;RARS2;C9H6orf165	chr9	63163673	63378215	3.79341	0.0	2.95883	1.35683	1.73976	1.16063	0.326658	0.366567	0.984141
XLOC_091049	SLC35A1;ORC3;RARS2;C9H6orf165	chr9	63163673	63378215	2.14319	0.0	0.0	0.76734	0.663449	1.32376	0.949318	0.422119	0.372694
XLOC_091050	SLC35A1;ORC3;RARS2;C9H6orf165	chr9	63163673	63378215	0.0	0.663888	0.0	0.497382	0.259885	0.690101	0.100228	0.11071	0.29067
XLOC_091051	SLC35A1;ORC3;RARS2;C9H6orf165	chr9	63163673	63378215	0.0	0.0	3.31373	0.759406	0.663198	0.293293	0.706291	0.778258	0.617034
XLOC_091052	SLC35A1;ORC3;RARS2;C9H6orf165	chr9	63163673	63378215	0.0	0.523966	1.37028	0.31403	0.544496	0.723772	0.782205	1.04098	0.916064
XLOC_037852	SLC35A5;ATG3;CCDC80	chr1	57774764	57946893	3.71567	3.53866	3.43284	0.484123	0.318163	0.563675	0.318552	0.203984	0.371854

XLOC_063983	SLC35B1;FAM117A;NXPH3;SPOP	chr19	37521069	37701537	11.6763	20.4261	13.0017	11.2465	9.70882	11.2955	12.2905	13.3923	16.6482
XLOC_063984	SLC35B1;FAM117A;NXPH3;SPOP	chr19	37521069	37701537	1.20415	1.25886	2.8241	0.131188	0.114839	0.195899	0.2579	0.0598632	0.0235583
XLOC_064244	SLC35B1;FAM117A;NXPH3;SPOP	chr19	37521069	37701537	0.0	0.927481	0.880931	0.0214914	0.0718074	0.0	0.0	0.032936	0.016247
XLOC_065066	SLC35B1;FAM117A;NXPH3;SPOP	chr19	37521069	37701537	0.764938	0.0	0.598429	0.0	0.0	0.158842	0.0694967	0.0	0.13369
XLOC_052535	SLC35C2	chr13	75802963	75803286	52.3368	39.0164	17.0031	94.387	80.7018	89.605	96.4022	124.434	125.78
XLOC_091307	SLC35D3	chr9	75899833	75900310	2.26394	0.0	0.0	0.0	0.0	0.0	0.0	0.0	0.196761
XLOC_076285	SLC38A4	chr5	33652962	33671984	14.9428	21.2201	13.6589	31.6952	28.0874	29.743	38.7949	40.4418	33.3862
XLOC_076803	SLC38A4	chr5	33600351	33629649	18.929	29.8288	18.664	19.765	18.826	17.3655	18.622	19.7489	17.2798
XLOC_076804	SLC38A4	chr5	33639947	33652700	44.5287	57.0096	40.8474	81.8919	89.1094	82.3248	90.8368	90.2544	102.309
XLOC_076805	SLC38A4	chr5	33652962	33671984	5.72368	7.94311	6.60524	2.48141	3.25099	2.94349	3.17647	3.43245	2.6556
XLOC_077671	SLC38A4	chr5	33639251	33639886	1.51475	1.81095	2.36806	2.57778	3.88795	1.5654	2.17169	2.10438	2.64012
XLOC_065723	SLC39A10	chr2	84734109	84739227	1.96101	3.18667	3.46518	2.34743	1.88103	2.34777	1.83277	1.89497	2.1836
XLOC_066390	SLC39A10	chr2	84740163	84743848	1.23014	0.163308	0.6407	3.94056	3.43494	3.03509	3.68927	3.27454	2.62604
XLOC_068065	SLC39A10	chr2	84739466	84739644	0.0	0.0	0.0	6.35353	18.0865	9.04703	24.2863	10.0633	11.6954
XLOC_064064	SLC39A11	chr19	58798651	58953204	1.53735	2.75684	0.942733	0.826128	0.509915	0.21001	0.550774	0.41992	0.512973
XLOC_064065	SLC39A11	chr19	58953267	58972181	0.303292	1.03506	1.17383	0.466282	0.239893	0.326557	0.375004	0.469506	0.225973
XLOC_064328	SLC39A11	chr19	58798651	58953204	3.79118	2.38827	5.7328	1.42672	2.16004	2.57206	2.57695	2.30262	2.22364
XLOC_064329	SLC39A11	chr19	58974606	59010169	4.9033	7.82362	5.96815	0.341929	0.597897	0.339861	0.546706	0.164129	0.714706
XLOC_064330	SLC39A11	chr19	59015581	59075660	6.08727	11.0324	8.33355	0.40923	0.289228	0.430336	0.325546	0.296169	0.603525
XLOC_065411	SLC39A11	chr19	58795755	58797438	4.26686	1.5603	2.96793	0.977727	1.82206	1.03557	0.822824	0.762401	2.4884
XLOC_065412	SLC39A11	chr19	58797527	58798320	3.41434	2.38205	2.66993	1.52967	1.50943	1.06103	0.821517	0.794213	1.88707
XLOC_065413	SLC39A11	chr19	58798651	58953204	0.0	1.64289	2.14838	0.307715	0.376274	0.356567	0.0624627	0.206458	0.120018
XLOC_065414	SLC39A11	chr19	58798651	58953204	4.08339	2.74741	4.79023	0.182963	0.239147	0.211643	0.369231	0.203828	0.178247
XLOC_065415	SLC39A11	chr19	58798651	58953204	0.0	5.0222	7.1641	0.410451	0.593999	0.15785	0.136845	1.06091	0.133115
XLOC_065416	SLC39A11	chr19	58953267	58972181	0.0	0.0	0.0	0.0	0.0	0.0	0.0898201	0.0	0.0
XLOC_065417	SLC39A11	chr19	58972294	58974503	2.46775	2.21526	2.48313	0.158071	0.166066	0.256877	0.0968271	0.0709712	0.30853
XLOC_065419	SLC39A11	chr19	59013475	59014983	0.0	0.160217	1.25712	0.0480155	0.0	0.0	0.0	0.0	0.0
XLOC_086904	SLC39A14	chr8	70185541	70194217	3.11558	2.21332	4.24117	2.02847	1.83514	1.07804	1.57406	2.25657	1.6391

XLOC_086441	SLC39A14;PPP3CC	chr8	70220347	70263190	25.4429	35.4255	34.1423	44.1752	52.8643	48.5422	47.2332	37.903	46.398
XLOC_086905	SLC39A14;PPP3CC	chr8	70220347	70263190	5.54357	5.05217	6.23044	8.73526	5.95358	5.50822	9.02111	11.4264	7.50287
XLOC_041676	SLC39A9	chr10	81559635	81566367	112.624	115.82	96.7428	118.821	137.513	124.52	127.65	168.891	148.527
XLOC_043116	SLC39A9	chr10	81576962	81577630	1.41682	0.42351	0.0	0.126914	0.0	0.0	0.0	0.281526	0.0
XLOC_055572	SLC43A3	chr15	81992412	82040168	27.402	31.4062	24.3267	63.2616	56.2991	62.1736	62.165	65.3215	67.0074
XLOC_055891	SLC43A3	chr15	81992412	82040168	8.61314	9.56615	7.94058	5.33904	5.31152	6.32227	6.2397	6.46714	3.68745
XLOC_057190	SLC43A3	chr15	81980526	81981565	0.820479	3.43557	0.641811	20.3711	8.6634	19.1606	12.9571	17.2438	9.17552
XLOC_057191	SLC43A3	chr15	81992412	82040168	3.35813	3.51535	3.94019	0.602056	0.787951	1.13276	0.381005	0.588208	0.3668
XLOC_082780	SLC44A2;ILF3	chr7	16326307	16387902	13.6477	21.8839	22.7302	48.3204	33.5522	48.1449	46.6625	42.7369	35.1421
XLOC_083415	SLC44A2;ILF3	chr7	16326307	16387902	65.9597	64.1683	51.6573	131.406	112.558	125.098	127.164	138.743	105.898
XLOC_084264	SLC44A2;ILF3	chr7	16326307	16387902	4.45813	3.3289	0.0	0.598571	0.344852	0.458792	2.76026	0.657866	1.16252
XLOC_084265	SLC44A2;ILF3	chr7	16326307	16387902	3.10242	2.31987	3.03368	1.80761	1.15305	1.44945	1.62039	1.63096	1.62658
XLOC_084266	SLC44A2;ILF3	chr7	16326307	16387902	1.06756	0.319238	0.834871	1.43497	1.08335	0.885104	1.54318	1.9173	0.838674
XLOC_084267	SLC44A2;ILF3	chr7	16326307	16387902	0.0	4.22522	4.41953	1.26622	1.08962	1.74142	1.48635	1.38255	0.491085
XLOC_084268	SLC44A2;ILF3	chr7	16326307	16387902	0.0	2.96246	0.0	1.77576	0.253931	0.676855	1.43611	0.964441	1.43296
XLOC_084269	SLC44A2;ILF3	chr7	16326307	16387902	0.0	1.20681	1.05204	0.964415	1.1529	1.39216	1.69333	1.47189	0.586706
XLOC_084270	SLC44A2;ILF3	chr7	16326307	16387902	0.0	0.0	0.0	0.68015	0.567085	3.04749	0.620807	2.83226	3.25085
XLOC_076278	SLC48A1;HDAC7	chr5	32610244	32686989	31.0236	37.5446	42.8199	53.067	43.6156	51.3643	85.0103	76.3588	80.5916
XLOC_065617	SLC4A10	chr2	34771998	34776464	272.732	184.125	225.946	265.297	194.446	242.331	241.522	330.128	266.929
XLOC_065618	SLC4A10	chr2	34804771	34826238	3.67798	3.2134	8.56316	2.23407	0.591664	1.68607	3.03849	3.01209	2.67795
XLOC_066253	SLC4A10	chr2	34676277	34754585	12.1954	14.4904	13.8143	24.1781	25.2017	24.0483	26.4687	26.8834	25.4551
XLOC_066254	SLC4A10	chr2	34779125	34799998	5.23508	8.69729	7.59374	18.6704	22.2237	17.6785	18.479	17.0102	21.5554
XLOC_066255	SLC4A10	chr2	34804771	34826238	28.5098	33.2805	30.6428	29.8142	29.3626	28.4981	32.8064	36.1124	34.8457
XLOC_067296	SLC4A10	chr2	34624472	34624968	6.41058	7.65948	3.33844	6.69443	4.13451	7.47945	7.00487	5.89266	5.75982
XLOC_067297	SLC4A10	chr2	34648977	34650314	18.3953	22.929	15.8319	20.01	14.8922	19.627	18.2123	26.3319	20.5872
XLOC_067298	SLC4A10	chr2	34673192	34673726	0.0	0.0	1.50106	0.688002	0.74471	0.792166	0.170877	0.569108	0.334352
XLOC_067299	SLC4A10	chr2	34673828	34674456	4.61186	0.459465	1.20162	1.1015	0.836836	1.74732	0.826162	0.610031	0.535837

XLOC_067300	SLC4A10	chr2	34676277	34754585	0.0	13.7071	0.0	0.0	6.15799	7.38033	3.71625	5.10844	4.73197
XLOC_067301	SLC4A10	chr2	34676277	34754585	0.0	5.35644	0.0	1.61878	0.668988	1.80499	1.45479	3.33134	5.39442
XLOC_067302	SLC4A10	chr2	34777066	34777959	2.95008	0.882201	0.0	2.11503	2.53481	2.95685	2.93635	1.47327	1.20209
XLOC_067303	SLC4A10	chr2	34778478	34778647	0.0	25.8105	0.0	18.8326	20.5101	17.0371	10.7698	15.0101	29.4149
XLOC_067304	SLC4A10	chr2	34778772	34779000	0.0	8.73649	7.61247	4.40761	7.97817	8.80747	9.43128	5.40858	5.02396
XLOC_067305	SLC4A10	chr2	34802018	34803109	8.52249	3.47606	3.63652	2.91683	1.758	1.85005	1.75943	1.93956	1.96335
XLOC_067306	SLC4A10	chr2	34803247	34803807	5.39112	5.36963	5.61707	2.09182	1.53404	1.48311	2.40298	1.59943	1.87736
XLOC_067307	SLC4A10	chr2	34803926	34804174	23.3888	11.6276	12.159	4.20689	5.8368	7.84748	5.10731	4.37195	7.36964
XLOC_067308	SLC4A10	chr2	34804771	34826238	11.8053	9.34755	10.8657	2.8636	4.02334	4.3279	3.85356	3.20188	2.85288
XLOC_067309	SLC4A10	chr2	34804771	34826238	7.82237	8.18999	5.61014	5.14263	4.54385	4.94418	5.87546	6.21052	5.5278
XLOC_067310	SLC4A10	chr2	34826385	34826713	4.23479	2.52577	6.60433	3.78692	0.645466	0.430965	0.72421	1.62932	1.4631
XLOC_079818	SLC4A4	chr6	88465366	88518805	10.9204	17.7896	11.564	28.6241	25.2014	27.4771	31.8175	39.104	28.0158
XLOC_080212	SLC4A4	chr6	88257285	88446569	14.457	14.3363	9.35274	20.7203	19.9104	19.7163	25.2384	28.9301	22.2176
XLOC_080213	SLC4A4	chr6	88465366	88518805	116.602	132.382	108.618	72.9855	74.0673	64.6566	74.8998	94.6489	68.9132
XLOC_081577	SLC4A4	chr6	88465366	88518805	0.0	0.736196	1.28365	0.294187	0.256746	0.681368	0.372341	0.492679	0.215055
XLOC_081578	SLC4A4	chr6	88465366	88518805	16.2526	12.122	6.33814	3.65735	5.46936	1.63593	1.99047	7.57964	2.79281
XLOC_081579	SLC4A4	chr6	88465366	88518805	0.875092	0.261718	0.0	0.627473	0.205298	0.0909046	0.396719	0.787672	0.229321
XLOC_081580	SLC4A4	chr6	88465366	88518805	4.21743	1.68089	2.19804	1.51118	1.86054	0.290936	1.38844	2.93344	1.10305
XLOC_081581	SLC4A4	chr6	88465366	88518805	0.0	1.90847	0.998285	0.800718	0.796039	0.396579	1.37888	1.52477	0.556814
XLOC_081582	SLC4A4	chr6	88465366	88518805	1.02811	1.84459	3.21619	1.75051	1.12395	1.59866	2.23085	1.64207	1.43608
XLOC_081583	SLC4A4	chr6	88465366	88518805	9.97046	7.431	15.5437	5.7981	3.40369	5.05765	1.68645	4.28416	6.8775
XLOC_081584	SLC4A4	chr6	88465366	88518805	0.0	2.42442	0.0	2.19449	1.82345	0.818462	1.32701	3.03186	2.09462
XLOC_081585	SLC4A4	chr6	88465366	88518805	2.64697	1.18694	1.03477	1.18568	0.721768	1.36953	0.952085	1.3164	0.692513
XLOC_081586	SLC4A4	chr6	88465366	88518805	0.0	0.0	0.0	0.273697	0.794996	0.316705	0.276172	1.42283	0.444372
XLOC_081587	SLC4A4	chr6	88465366	88518805	0.0	1.0556	5.52128	1.898	1.6454	0.546984	0.157572	1.04845	0.92274
XLOC_050895	SLC52A3	chr13	60842615	60864409	12.8006	15.4479	7.51685	2.40453	0.811805	1.45704	1.05519	1.529	0.906046
XLOC_055464	SLC5A12	chr15	58141352	58158644	19.7783	15.504	14.4628	11.6551	10.7987	11.3771	9.07464	6.79111	9.52509
XLOC_055776	SLC5A12	chr15	58179667	58214889	12.5925	24.4223	22.9349	0.651567	0.570878	0.682979	0.510982	0.685359	0.51023
XLOC_056653	SLC5A12	chr15	58179667	58214889	1.20646	1.44282	0.943359	0.21619	0.0	0.0	0.10872	0.0	0.0
XLOC_056654	SLC5A12	chr15	58179667	58214889	0.0	2.14933	5.62071	0.0	0.0	0.0	0.0	0.0	0.0
XLOC_076348	SLC5A8	chr5	65387847	65409203	7.8408	11.7914	5.09148	9.79419	10.2794	10.9778	14.357	8.29122	11.7918
XLOC_076858	SLC5A8	chr5	65387847	65409203	4.75831E-	0.927281	2.01589	7.04652	4.62112	3.90141	6.38227	10.5727	6.05327
XLOC_076859	SLC5A8	chr5	65413221	65435900	34.6031	22.4361	24.8721	24.0376	26.0264	25.2399	31.0546	14.0114	26.4198

XLOC_076860	SLC5A8	chr5	65449633	65493036	19.7329	31.7775	21.2956	13.6964	19.1203	13.8004	18.012	15.7939	26.2367
XLOC_078042	SLC5A8	chr5	65386709	65387643	2.7943	1.67132	0.728519	3.42257	3.20261	2.6084	3.37451	4.0029	3.98589
XLOC_078043	SLC5A8	chr5	65409584	65409805	0.0	6.37352	0.0	0.0	1.58218	4.27645	5.1253	1.96295	0.915546
XLOC_078044	SLC5A8	chr5	65410049	65410246	0.0	9.23444	0.0	4.24102	5.64912	4.61644	4.81766	5.56828	3.96632
XLOC_078045	SLC5A8	chr5	65439796	65439962	30.7774	28.12	0.0	8.82021	0.0	12.3578	9.33706	21.6942	0.0
XLOC_078046	SLC5A8	chr5	65446805	65447970	3.58931	1.71782	3.36961	0.643509	1.06768	0.670996	1.1097	0.791275	1.19209
XLOC_078047	SLC5A8	chr5	65448832	65449561	0.0	1.51343	0.0	0.907075	0.887697	0.392977	1.59492	1.13373	1.87666
XLOC_076440	SLC6A12	chr5	107597727	107661883	15.1282	10.0138	6.5426	12.9267	16.3703	14.2967	18.7848	14.702	16.1285
XLOC_078571	SLC6A12	chr5	107597727	107661883	1.59141	0.0	1.24385	0.997693	0.123703	0.493294	0.142401	0.315654	0.693349
XLOC_062163	SLC6A2	chr18	23989380	24002820	10.6956	13.8431	12.2436	12.3966	13.1659	10.6797	15.4637	18.553	14.1089
XLOC_062373	SLC6A2	chr18	23965428	23987258	1.214	3.811	0.949848	1.70204	1.38642	1.25168	1.39248	1.39351	0.95918
XLOC_062791	SLC6A2	chr18	23961607	23964820	9.46777	10.0584	7.59589	9.04334	8.92817	8.62969	9.46867	8.23149	9.40856
XLOC_062792	SLC6A2	chr18	23965428	23987258	6.529	11.6899	7.642	3.2115	3.00574	4.00638	3.11913	2.53839	1.13104
XLOC_062793	SLC6A2	chr18	23965428	23987258	0.0	0.809033	2.11586	0.727338	0.421549	0.699925	0.608071	0.538193	0.825977
XLOC_062794	SLC6A2	chr18	23965428	23987258	0.0	0.0	1.20674	0.138275	0.120052	0.0	0.138265	0.153154	0.134528
XLOC_062795	SLC6A2	chr18	23965428	23987258	1.98988	1.78341	1.55464	0.35628	0.61674	0.820152	0.353537	0.588984	0.173113
XLOC_064729	SLC6A4	chr19	21857333	21858484	3.6399	7.1858	14.8072	17.4236	14.5301	15.5738	13.3692	16.8488	16.733
XLOC_064730	SLC6A4	chr19	21861154	21861851	0.0	0.400772	0.0	1.44121	0.417684	0.416095	0.602573	0.533279	0.116908
XLOC_064731	SLC6A4	chr19	21863425	21864073	0.0	0.0	1.15269	1.32082	0.11473	0.152429	0.528921	0.292801	1.15673
XLOC_063936	SLC6A4;MIR423;NSRP1	chr19	21777550	21857175	15.0256	17.7621	18.3939	34.6239	25.4109	30.8501	26.8365	30.0179	29.5345
XLOC_048969	SLC7A1	chr12	31018686	31018815	797.602	509.877	535.223	4359.71	3408.38	4020.43	3917.21	2995.53	5223.81
XLOC_048970	SLC7A1	chr12	31018873	31019151	0.0	0.0	4.63671	5.86215	7.632	4.8119	5.96179	5.0702	5.63343
XLOC_038488	SLC7A14	chr1	97572866	97662000	18.4639	14.6203	13.0207	0.884536	1.90254	1.47586	1.28969	0.818184	1.52915
XLOC_038489	SLC7A14	chr1	97706047	97755849	9.53832	11.2041	10.7214	25.1508	17.3475	21.3222	29.0045	32.115	22.2769
XLOC_038490	SLC7A14	chr1	97756026	97759536	3.23309	3.09489	1.51771	6.08671	4.91264	4.90456	6.182	6.16092	4.23973
XLOC_039998	SLC7A14	chr1	97572866	97662000	8.04955	4.09291	7.55619	0.144303	0.125934	0.0835549	0.292312	0.16115	0.281326
XLOC_038005	SLC7A14;CLDN11	chr1	97759695	97792973	3.32427	9.73563	3.70194	4.58151	4.31826	4.10304	4.88522	6.10963	5.49416
XLOC_038491	SLC7A14;CLDN11	chr1	97759695	97792973	5.40285	3.24051	3.91843	6.62618	4.45344	5.63888	5.6648	7.23649	5.49831
XLOC_062194	SLC7A6OS;PRMT7;SLC7A6	chr18	35771581	35809121	49.0166	27.939	24.0675	22.4045	20.4831	19.7094	25.3535	23.9351	21.4197

XLOC_062927	SLC7A6OS;PRMT7;SLC7A6	chr18	35771581	35809121	15.9162	11.87	6.20616	2.14797	1.78631	4.80476	2.6028	2.97214	4.10284
XLOC_062928	SLC7A6OS;PRMT7;SLC7A6	chr18	35771581	35809121	21.7727	7.42631	2.4274	6.39845	5.01745	7.64102	7.31378	3.93701	4.85183
XLOC_062929	SLC7A6OS;PRMT7;SLC7A6	chr18	35771581	35809121	12.1399	4.8276	15.779	6.15149	8.95462	6.18275	8.67575	11.3097	4.89576
XLOC_062930	SLC7A6OS;PRMT7;SLC7A6	chr18	35771581	35809121	7.00625	6.26742	27.3087	5.66037	5.2618	5.65115	5.20363	5.92419	6.6267
XLOC_062931	SLC7A6OS;PRMT7;SLC7A6	chr18	35771581	35809121	14.7552	8.79787	11.5017	6.59994	9.33081	4.99058	5.41238	3.28951	6.78625
XLOC_062932	SLC7A6OS;PRMT7;SLC7A6	chr18	35771581	35809121	10.6928	4.10903	5.97008	2.05225	3.68279	1.8942	3.0106	2.42495	1.33115
XLOC_062933	SLC7A6OS;PRMT7;SLC7A6	chr18	35771581	35809121	22.5105	6.73165	8.79791	2.0441	0.833842	7.89642	2.69556	2.06686	1.93303
XLOC_062934	SLC7A6OS;PRMT7;SLC7A6	chr18	35771581	35809121	3.60303	3.2298	7.0388	2.09702	2.6562	1.67261	2.89052	2.49406	1.56833
XLOC_062935	SLC7A6OS;PRMT7;SLC7A6	chr18	35771581	35809121	3.55224	5.299	2.77121	1.90593	4.07982	3.26458	4.29898	2.40913	1.84446
XLOC_062936	SLC7A6OS;PRMT7;SLC7A6	chr18	35771581	35809121	20.5058	18.381	0.0	2.78491	5.3311	11.3135	8.2362	3.78239	4.4022
XLOC_062937	SLC7A6OS;PRMT7;SLC7A6	chr18	35771581	35809121	3.15096	3.76147	7.37694	3.94543	4.11349	3.54701	6.30715	2.7599	3.27632
XLOC_062938	SLC7A6OS;PRMT7;SLC7A6	chr18	35771581	35809121	20.2331	7.2414	12.6232	6.15149	6.1756	7.4193	7.28763	5.84984	3.84666
XLOC_062939	SLC7A6OS;PRMT7;SLC7A6	chr18	35771581	35809121	6.55852	5.87135	0.0	1.17309	2.26425	4.02417	6.83444	1.91208	2.55624
XLOC_062940	SLC7A6OS;PRMT7;SLC7A6	chr18	35771581	35809121	2.83076	4.22517	6.62926	2.02584	3.7044	2.03151	1.48635	3.31812	3.92868
XLOC_062941	SLC7A6OS;PRMT7;SLC7A6	chr18	35771581	35809121	4.90773	2.20091	2.87803	1.20919	1.33911	1.39725	1.43679	0.855263	1.71257

XLOC_062942	SLC7A6OS;PRMT7;SLC7A6	chr18	35771581	35809121	20.6358	8.20392	32.172	3.08649	7.75308	12.4871	11.9365	7.76192	11.8304
XLOC_062943	SLC7A6OS;PRMT7;SLC7A6	chr18	35771581	35809121	7.06989	5.27326	8.27326	4.74165	4.60185	3.60997	3.97361	2.05531	3.36522
XLOC_062944	SLC7A6OS;PRMT7;SLC7A6	chr18	35771581	35809121	15.9268	4.74789	0.0	2.37566	2.00987	1.07579	2.23497	1.51636	2.28739
XLOC_062945	SLC7A6OS;PRMT7;SLC7A6	chr18	35771581	35809121	3.93717	3.52301	3.07066	3.87268	4.20912	4.01249	4.05874	3.4189	3.74375
XLOC_062946	SLC7A6OS;PRMT7;SLC7A6	chr18	35771581	35809121	3.14819	3.76359	8.61237	4.08839	4.77259	2.60181	4.22686	3.27787	4.2516
XLOC_062947	SLC7A6OS;PRMT7;SLC7A6	chr18	35771581	35809121	0.0	8.33238	8.71418	8.50853	4.64901	7.35612	9.37902	4.77855	7.22248
XLOC_062948	SLC7A6OS;PRMT7;SLC7A6	chr18	35771581	35809121	18.0939	13.4877	14.1067	6.87739	9.97863	7.81559	10.7829	10.4121	14.4458
XLOC_041476	SLC7A7	chr10	21831289	21845170	1.07709	0.322067	0.0	0.386067	0.336282	0.0	0.0972932	1.28947	0.188031
XLOC_041868	SLC7A7	chr10	21831289	21845170	0.0	0.0	0.0	2.69732	1.10972	1.67292	1.71184	1.98967	1.05567
XLOC_042445	SLC7A7	chr10	21831289	21845170	0.0	0.171108	0.0	2.25629	1.88265	1.78367	2.34677	3.1579	1.60052
XLOC_042446	SLC7A7	chr10	21831289	21845170	0.0	0.267562	0.0	0.601396	0.456006	0.697739	0.776412	0.989003	0.352063
XLOC_042447	SLC7A7	chr10	21831289	21845170	0.0	0.0	0.0	1.01309	1.22528	0.698657	1.60097	2.22627	0.393522
XLOC_042448	SLC7A7	chr10	21846848	21848606	0.0	0.0	0.0	0.972449	0.638076	1.36325	0.49553	0.499725	0.751127
XLOC_042449	SLC7A7	chr10	21848959	21849964	0.0	0.0	0.0	1.14735	0.600573	0.708453	0.928877	0.682985	0.447305
XLOC_043990	SLC8A1	chr11	22512129	22518951	0.987229	1.16833	0.0	2.1896	2.3617	2.62964	1.86043	2.33965	3.40877
XLOC_043991	SLC8A1	chr11	22834547	22861535	0.780626	0.0	0.0	0.0	0.0	0.292628	0.585259	0.647489	0.128875
XLOC_044684	SLC8A1	chr11	22758225	22774929	0.0	0.128541	0.0	0.0385227	0.0674154	0.313442	0.196638	0.21632	0.150479
XLOC_044685	SLC8A1	chr11	22776756	22780384	0.0	0.0	0.472905	0.0541541	0.137936	0.0	0.0	0.0549978	0.264227
XLOC_044686	SLC8A1	chr11	22830198	22834404	1.89699	0.540223	0.979398	1.44187	1.55171	1.62567	3.4942	1.47784	1.97218
XLOC_044687	SLC8A1	chr11	22834547	22861535	11.9896	6.42634	10.0498	19.3228	19.9993	19.3203	24.8037	18.5462	21.1109
XLOC_045626	SLC8A1	chr11	22602826	22603239	0.0	0.0	0.0	2.78553	0.0	0.870648	0.0	0.55302	0.491085
XLOC_045627	SLC8A1	chr11	22610127	22611418	0.0	0.0	0.0	2.6885	1.39941	0.795601	1.8013	1.98401	1.17145
XLOC_045628	SLC8A1	chr11	22614051	22614597	0.0	0.0	0.0	0.166697	1.44409	0.191994	0.165777	0.0	0.162047
XLOC_045629	SLC8A1	chr11	22614806	22615508	0.0	0.0	0.0	0.475998	1.03472	0.137435	1.31378	0.26423	0.11584

XLOC_045630	SLC8A1	chr11	22621445	22621768	0.0	0.0	0.0	3.90029	2.32474	0.443589	1.11663	0.418969	1.12976
XLOC_045631	SLC8A1	chr11	22776756	22780384	0.0	0.0	0.0	0.0	0.0	0.0	0.0	0.339374	0.0
XLOC_045632	SLC8A1	chr11	22793715	22794524	0.0	0.331944	0.0	0.298429	0.259885	0.575084	0.100219	0.0	0.29067
XLOC_045633	SLC8A1	chr11	22809482	22810001	2.00089	0.0	0.0	0.0	0.465074	0.0	0.177711	0.0	0.0
XLOC_045634	SLC8A1	chr11	22810976	22811859	0.996916	0.298117	0.0	0.268019	0.0778626	0.103357	0.450922	0.0	0.174085
XLOC_045635	SLC8A1	chr11	22813492	22814678	0.0	0.0	0.550142	0.0	0.0550528	0.0	0.127923	0.0704779	0.184392
XLOC_045636	SLC8A1	chr11	22821041	22821910	1.01653	0.0	0.0	0.45548	1.11135	0.210756	0.459629	0.202979	0.443746
XLOC_045637	SLC8A1	chr11	22823658	22824315	1.44803	0.432824	0.0	0.259411	0.450748	0.299414	0.519642	0.143809	0.252444
XLOC_045638	SLC8A1	chr11	22827027	22827418	0.0	0.0	0.0	0.0	0.237928	0.634077	3.23741	0.0	0.536811
XLOC_045639	SLC8A1	chr11	22827542	22828149	0.0	0.0	0.0	0.864663	0.62531	0.830977	0.863629	0.0	0.280364
XLOC_045640	SLC8A1	chr11	22830198	22834404	0.0	0.350208	0.915891	0.314843	0.548123	0.363913	1.26742	0.817034	1.02203
XLOC_045641	SLC8A1	chr11	22834547	22861535	1.05356	1.26016	0.0	2.36036	2.30308	1.96554	2.76114	2.52332	2.02332
XLOC_043142	SLC8A3	chr10	82142116	82143655	2.09406	0.78312	2.45785	0.844895	0.656713	0.870985	0.334437	0.52585	0.549458
XLOC_043143	SLC8A3	chr10	82151944	82152631	4.09793	0.816663	5.33952	2.69206	2.76566	2.40203	2.08648	2.03694	2.97764
XLOC_043144	SLC8A3	chr10	82157535	82159800	3.43167	2.05372	2.41717	2.03111	1.8861	2.03625	1.7283	1.72733	1.68192
XLOC_043145	SLC8A3	chr10	82160959	82161269	0.0	0.0	0.0	1.69162	1.0776	2.40059	2.4079	5.43072	1.22369
XLOC_043146	SLC8A3	chr10	82161637	82162609	3.5519	1.06227	2.08368	1.43255	0.832978	2.211	0.965895	1.24312	1.70636
XLOC_043147	SLC8A3	chr10	82162906	82163576	0.0	1.26558	0.0	1.01136	1.31833	0.875653	0.886901	1.54246	1.72251
XLOC_043148	SLC8A3	chr10	82164213	82164863	0.0	1.75587	0.0	1.05237	1.1427	1.51815	2.23903	1.60398	0.896047
XLOC_043149	SLC8A3	chr10	82164921	82165347	0.0	0.804248	4.20626	1.68702	1.03791	3.04036	1.41784	2.63554	0.701316
XLOC_043150	SLC8A3	chr10	82166501	82168573	0.755526	1.13034	0.0	1.21952	1.48261	1.21882	1.14078	0.722249	1.22314
XLOC_043151	SLC8A3	chr10	82168715	82169232	0.0	0.601073	0.0	0.720473	0.779367	0.414583	1.25065	0.396912	2.27532
XLOC_043152	SLC8A3	chr10	82222728	82223609	0.0	0.59788	0.781835	0.537519	0.468457	1.24369	0.633003	0.499134	0.436412
XLOC_043153	SLC8A3	chr10	82224751	82225113	3.53494	3.16395	2.75775	6.32219	2.43627	2.88798	0.916986	5.48082	2.75336
XLOC_043154	SLC8A3	chr10	82240876	82241289	2.83076	0.845033	0.0	1.51938	1.96115	1.7413	1.73407	1.10604	2.94651
XLOC_065860	SLC9A1	chr2	126707279	126759239	45.5678	45.6103	45.6443	30.8558	31.7737	30.7355	34.2819	33.4782	32.4817
XLOC_068516	SLC9A1	chr2	126676362	126676645	5.71993	17.051	0.0	4.0976	6.04998	1.73617	5.26933	3.25599	5.41815
XLOC_068517	SLC9A1	chr2	126677077	126677787	1.30911	1.56541	6.14103	1.17279	1.12189	1.49007	1.05972	1.04186	1.82673
XLOC_068518	SLC9A1	chr2	126677929	126678561	3.04865	4.55597	1.1915	1.50182	2.72671	1.73273	4.37008	2.41993	1.19553
XLOC_068519	SLC9A1	chr2	126679456	126679948	6.48725	0.645908	3.37828	0.774207	0.167324	0.44516	0.765987	0.63871	0.752022
XLOC_068520	SLC9A1	chr2	126707279	126759239	90.5884	67.0762	44.5035	81.0134	47.336	74.0802	62.5623	92.8458	57.234
XLOC_038519	SLC9A9	chr1	125976964	126738008	17.6271	27.505	31.6958	0.0813764	0.0	0.089947	0.0150622	0.0165403	0.0355757

XLOC_080024	SLC9B2;BDH2	chr6	23072546	23133001	3.27412	3.94201	2.8442	17.5346	8.12367	11.9711	11.5355	17.9616	15.9426
XLOC_076904	SLCO1C1	chr5	89417567	89457766	0.0	0.0	0.386704	0.0	0.0387542	0.0513962	0.0	0.0	0.0432275
XLOC_038076	SLCO2A1	chr1	136415269	136440127	3.57148	4.6152	5.59002	12.5085	11.5362	11.6865	11.2872	15.8462	13.7495
XLOC_038077	SLCO2A1	chr1	136466293	136527944	7.90678	8.31536	5.10591	18.455	19.7719	19.6739	21.8151	20.6513	20.7096
XLOC_040421	SLCO2A1	chr1	136528093	136529304	1.37303	2.05358	1.61129	8.06212	9.19185	10.0552	9.55679	8.18952	10.2615
XLOC_050569	SLCO4A1	chr13	55152758	55168713	0.313285	0.513087	0.490392	0.39302	0.248019	0.423884	0.488333	0.252832	0.37142
XLOC_079666	SLIT2	chr6	41605383	41620595	6.12731	6.91287	6.96392	0.856376	0.590412	0.494261	0.350608	0.500993	0.60181
XLOC_080087	SLIT2	chr6	41620655	41623159	0.72506	1.51815	2.26891	0.0	0.0	0.0	0.0	0.0	0.0
XLOC_080088	SLIT2	chr6	41625148	41626173	0.0	0.0	0.0	0.0	0.183501	0.0	0.0	0.0	0.0
XLOC_080932	SLIT2	chr6	41253395	41253896	0.0	8.80624	4.93492	0.376982	0.978079	0.867292	0.746679	1.65973	1.0987
XLOC_080933	SLIT2	chr6	41624160	41624814	5.8271	6.53154	5.69391	0.0	0.0	0.0	0.0	0.0	0.126981
XLOC_050635	SLPI	chr13	74322595	74357702	22.5505	26.998	16.1883	24.7482	26.6108	24.7531	26.0917	27.2067	28.946
XLOC_050957	SLPI	chr13	74322595	74357702	51.8274	54.9405	52.6831	93.6091	102.573	96.835	109.671	109.922	103.62
XLOC_052499	SLPI	chr13	74322595	74357702	0.0	3.03408	0.0	2.73187	1.92889	2.06402	5.15683	1.45634	2.6321
XLOC_059965	SMAD1	chr17	12888514	12909931	8.21263	12.1184	12.6015	12.3384	13.8687	11.9933	13.6881	13.5305	14.5385
XLOC_060581	SMAD1	chr17	12888514	12909931	8.99129	10.7239	3.50519	4.82481	5.13051	5.48378	2.68024	5.17567	3.8805
XLOC_059573	SMAD1;MIR2284G	chr17	12911343	12960726	220.483	286.728	245.353	598.843	541.468	590.805	642.736	681.611	630.928
XLOC_059966	SMAD1;MIR2284G	chr17	12911343	12960726	27.5321	21.611	16.569	98.0878	78.596	85.186	91.0419	112.846	99.6
XLOC_059967	SMAD1;MIR2284G	chr17	12911343	12960726	11.6774	12.4782	11.7981	25.7362	29.5751	32.1546	21.1964	31.2377	21.5088
XLOC_060582	SMAD1;MIR2284G	chr17	12911343	12960726	0.0	1.05992	0.0	0.952965	1.08795	0.725465	1.53535	0.689002	0.0
XLOC_060583	SMAD1;MIR2284G	chr17	12911343	12960726	0.0	0.463452	0.0	2.36074	0.723363	1.12138	0.277682	1.23063	0.675516
XLOC_060584	SMAD1;MIR2284G	chr17	12911343	12960726	2.14971	1.28423	3.35932	1.73163	2.16251	1.7702	1.33282	0.847099	2.05587
XLOC_041851	SMAD3	chr10	13647048	13868970	14.4765	16.1062	9.06706	2.36735	3.67023	2.83942	3.85099	1.47704	3.14984
XLOC_042352	SMAD3	chr10	13890617	13891429	0.0	0.0	0.0	0.693147	0.34494	0.114493	0.099769	1.32251	0.482239
XLOC_041461	SMAD3;AAGAB	chr10	13965257	14151333	24.6971	26.6444	32.9437	0.773681	0.507368	0.715167	0.254318	0.485458	0.475418
XLOC_042353	SMAD3;AAGAB	chr10	13965257	14151333	1.84754	5.52034	1.44397	0.0	0.0	0.190539	0.0	0.182596	0.0
XLOC_042354	SMAD3;AAGAB	chr10	13965257	14151333	4.02734	3.81497	6.82694	0.0	0.0	0.0697316	0.0610887	0.0	0.0

XLOC_042355	SMAD3;AAGAB	chr10	13965257	14151333	9.85782	7.35934	5.77393	0.0	0.190245	0.506413	0.6512	0.0	0.0
XLOC_042356	SMAD3;AAGAB	chr10	13965257	14151333	3.16003	3.78069	7.41606	0.283252	0.0618051	0.0820109	0.0	0.0	0.0
XLOC_042357	SMAD3;AAGAB	chr10	13965257	14151333	3.89117	4.85211	8.46051	0.116333	0.101945	0.180175	0.0396844	0.0	0.170358
XLOC_042358	SMAD3;AAGAB	chr10	13965257	14151333	7.25857	5.27129	6.48777	0.27877	0.0809625	0.21496	0.0937356	0.0	0.0
XLOC_042359	SMAD3;AAGAB	chr10	13965257	14151333	2.61926	3.13413	4.61092	0.117407	0.0512844	0.0	0.0	0.0	0.228978
XLOC_042360	SMAD3;AAGAB	chr10	13965257	14151333	4.69421	4.41324	4.19735	0.0	0.0525141	0.139334	0.0	0.0672373	0.0586213
XLOC_042361	SMAD3;AAGAB	chr10	13965257	14151333	3.73054	4.18372	7.29474	0.167172	0.0	0.193463	0.0	0.0	0.0814493
XLOC_042362	SMAD3;AAGAB	chr10	13965257	14151333	0.0	4.89082	2.13203	0.0	0.106173	0.0	0.0	0.0	0.0
XLOC_042363	SMAD3;AAGAB	chr10	13965257	14151333	3.66903	4.66344	10.0443	0.0822099	0.0	0.285437	0.0	0.0	0.0801109
XLOC_042364	SMAD3;AAGAB	chr10	13965257	14151333	5.10232	5.08675	8.64755	0.0762219	0.0664966	0.0882459	0.0	0.0	0.0742887
XLOC_042365	SMAD3;AAGAB	chr10	13965257	14151333	2.5211	3.58238	6.90386	0.0	0.0493723	0.0654943	0.0	0.0	0.0551043
XLOC_042366	SMAD3;AAGAB	chr10	13965257	14151333	1.43858	3.0122	8.44088	0.0644803	0.0563048	0.0747027	0.0	0.0720759	0.251465
XLOC_042367	SMAD3;AAGAB	chr10	13965257	14151333	5.95914	4.89824	5.82299	0.133443	0.115898	0.153984	0.0	0.0	0.0
XLOC_042368	SMAD3;AAGAB	chr10	13965257	14151333	2.71147	4.05281	4.23987	0.121453	0.0	0.0	0.0	0.0	0.0
XLOC_042369	SMAD3;AAGAB	chr10	13965257	14151333	4.77862	4.28965	7.76737	0.164818	0.115423	0.15304	0.0672885	0.0369938	0.0321686
XLOC_042370	SMAD3;AAGAB	chr10	13965257	14151333	2.22373	4.32195	8.69498	0.199261	0.0	0.0	0.0	0.0	0.0
XLOC_042371	SMAD3;AAGAB	chr10	13965257	14151333	10.968	3.64354	9.52905	0.0	0.0950123	0.0	0.109794	0.242738	0.0
XLOC_042372	SMAD3;AAGAB	chr10	13965257	14151333	0.0	7.06536	3.89031	0.111439	0.0969536	0.0	0.0	0.0	0.0
XLOC_042373	SMAD3;AAGAB	chr10	13965257	14151333	5.46629	16.2953	17.0428	0.0	0.0	0.0	0.0	0.0	0.0
XLOC_042374	SMAD3;AAGAB	chr10	13965257	14151333	5.6289	3.92431	14.6611	0.167996	0.0	0.0	0.0	0.0	0.0
XLOC_042375	SMAD3;AAGAB	chr10	13965257	14151333	2.41696	2.89052	9.44954	0.0	0.0942261	0.0	0.0	0.0	0.0
XLOC_041459	SMAD6	chr10	13500948	13515146	3.52958	2.47836	0.791214	3.44949	5.06927	3.90645	3.78671	2.20852	4.60294
XLOC_041460	SMAD6	chr10	13561095	13563164	0.0	2.44076	5.09643	6.58643	6.78102	6.40139	6.96619	7.7117	9.73005

XLOC_041850	SMAD6	chr10	13524151	13553126	6.77144	10.1308	7.36024	8.49163	7.78311	9.29428	11.1506	12.1184	10.924
XLOC_042348	SMAD6	chr10	13515436	13515754	4.49564	1.34049	10.5151	2.01029	3.42034	2.74181	2.29735	2.58772	4.6566
XLOC_042349	SMAD6	chr10	13563310	13563665	0.0	0.0	0.0	2.61803	2.23999	5.97585	1.26319	4.95815	2.84944
XLOC_042350	SMAD6	chr10	13564572	13565444	0.0	0.0	0.0	0.907139	0.632414	0.524692	0.823922	0.90962	1.06053
XLOC_042351	SMAD6	chr10	13565501	13566225	0.0	0.0	0.0	0.800688	0.696463	1.05712	1.03415	0.889449	1.00226
XLOC_092784	SMARCA1	chrX	13377909	13378555	17.7676	11.949	15.0463	4.37652	11.1739	8.11151	8.76196	11.4651	12.7759
XLOC_082784	SMARCA4	chr7	16721904	16725609	0.978041	0.0	1.18156	0.0	0.0764	0.0	0.0	0.0	0.0
XLOC_082785	SMARCA4	chr7	16738295	16749621	23.2575	25.7034	18.7017	49.8763	41.1951	53.7011	37.8867	50.2704	43.3258
XLOC_083417	SMARCA4	chr7	16671804	16675665	1.00523	0.3006	0.0	0.0	0.0	0.0	0.0	0.0	0.0
XLOC_084284	SMARCA4	chr7	16671264	16671506	0.0	0.0	6.47489	0.0	0.0	0.0	0.0	0.0	0.0
XLOC_084285	SMARCA4	chr7	16725890	16726141	0.0	2.2562	0.0	0.0	0.0	0.0	0.0	0.0	0.0
XLOC_065776	SMARCAL1	chr2	105125011	105173709	28.4209	33.8278	43.6531	1.25991	0.0988973	0.6459	0.0574927	0.371718	0.0350713
XLOC_068241	SMARCAL1	chr2	105125011	105173709	6.87796	5.47777	21.4878	0.205182	0.0	0.235797	0.0	0.0	0.0
XLOC_068242	SMARCAL1	chr2	105125011	105173709	8.18624	4.16232	11.5265	0.0733754	0.0	0.0	0.0	0.0819307	0.0
XLOC_068243	SMARCAL1	chr2	105125011	105173709	4.84437	4.6358	6.06223	0.0	0.0	0.100467	0.0	0.0	0.0
XLOC_068244	SMARCAL1	chr2	105125011	105173709	7.8478	5.46732	12.255	0.0	0.0	0.0	0.0	0.0	0.0
XLOC_068245	SMARCAL1	chr2	105125011	105173709	14.4122	3.22979	8.4467	0.322618	0.0	0.185846	0.0	0.0	0.0
XLOC_063998	SMARCE1;KRT222;KRT24	chr19	41482680	41587152	46.4118	50.4182	46.127	22.4969	19.957	23.5346	26.4979	26.765	16.8205
XLOC_065149	SMARCE1;KRT222;KRT24	chr19	41482680	41587152	0.0	2.16965	11.3443	7.18977	6.54961	8.79817	4.79003	6.82282	5.00348
XLOC_065150	SMARCE1;KRT222;KRT24	chr19	41482680	41587152	6.14551	4.36563	5.40846	3.92508	4.86882	5.26406	2.51236	4.38498	3.49066
XLOC_065151	SMARCE1;KRT222;KRT24	chr19	41482680	41587152	11.3996	10.7208	8.15715	2.42439	2.6344	2.64498	2.6254	2.36129	2.85076
XLOC_065152	SMARCE1;KRT222;KRT24	chr19	41482680	41587152	4.59193	7.49426	8.49377	1.94658	2.68607	2.77998	2.02314	1.59547	2.08195
XLOC_065153	SMARCE1;KRT222;KRT24	chr19	41482680	41587152	11.9728	16.2728	20.4283	2.43851	4.16249	4.51143	2.65404	2.8229	3.13507
XLOC_065154	SMARCE1;KRT222;KRT24	chr19	41482680	41587152	9.45596	8.67742	10.6078	1.75262	2.10408	2.55984	2.02143	1.74581	2.48303

XLOC_076470	SMCR7L	chr5	111419785	111454859	3.97285	4.64825	3.10932	0.236392	0.25997	0.206803	0.242582	0.333338	0.345292
XLOC_078732	SMCR7L	chr5	111454916	111457029	3.69842	2.8773	2.89443	0.331656	0.145162	0.0769884	0.0	0.334978	0.161828
XLOC_063939	SMG6	chr19	23687403	23721756	2.42133	2.91052	2.13198	7.8542	7.12309	8.81886	7.82691	8.11016	9.32887
XLOC_064775	SMG6	chr19	23675818	23676714	76.3905	94.3052	64.3415	169.92	159.585	161.361	208.004	209.538	228.071
XLOC_064776	SMG6	chr19	23687403	23721756	0.0	0.0	0.0	2.6015	0.877425	5.87626	1.45799	2.20304	3.0014
XLOC_064777	SMG6	chr19	23687403	23721756	0.0	0.0	3.50504	1.20618	4.10441	2.28484	6.50917	2.58772	2.71646
XLOC_064778	SMG6	chr19	23722043	23724989	2.07487	1.94036	2.436	6.76884	7.19331	7.40165	7.2311	7.91941	8.8327
XLOC_064779	SMG6	chr19	23725057	23728033	2.56597	1.91971	1.80756	5.33904	5.26207	5.37198	6.26018	6.9303	7.66046
XLOC_064780	SMG6	chr19	23728308	23730969	2.59996	0.691582	1.13052	3.57529	4.0165	3.42947	4.73988	3.55038	5.6646
XLOC_064781	SMG6	chr19	23731134	23731681	1.85703	0.554855	0.0	2.82659	2.59279	1.72356	2.31511	2.20216	3.55593
XLOC_064782	SMG6	chr19	23732012	23732559	0.0	0.0	0.0	1.16389	1.44044	1.91507	0.661461	0.917565	1.77797
XLOC_064783	SMG6	chr19	23733068	23733391	0.0	0.0	0.0	3.51026	0.996318	5.76666	1.11663	1.25691	2.63611
XLOC_064784	SMG6	chr19	23760259	23761176	0.0	0.284783	0.0	0.0	0.0	0.0	0.0	0.0	0.0
XLOC_064787	SMG6	chr19	23802885	23803714	0.0	0.0	0.0	0.0965168	0.0	0.0	0.0	0.0	0.0
XLOC_063907	SMG8;GDPD1	chr19	10389432	10448967	3.11312	5.08496	4.03222	1.66307	1.44797	1.44675	1.41103	1.61626	1.30683
XLOC_043129	SMOC1	chr10	81998392	81999131	1.24381	1.85929	0.0	0.891495	2.52079	1.03005	1.68013	1.85754	1.95303
XLOC_043130	SMOC1	chr10	82008388	82008876	0.0	3.26855	1.70954	2.15478	0.677258	2.47762	0.968615	0.430825	1.52211
XLOC_043131	SMOC1	chr10	82009442	82009987	5.59984	1.67314	1.45853	0.0	0.723882	0.769935	0.83095	0.0	0.81231
XLOC_043132	SMOC1	chr10	82017793	82018569	0.0	0.699272	0.914412	1.25734	0.638453	0.847767	2.10903	0.233066	1.02038
XLOC_043133	SMOC1	chr10	82061257	82061628	6.77291	3.03139	0.0	3.02846	1.55763	3.46108	3.22902	3.94442	3.22534
XLOC_043134	SMOC1	chr10	82063674	82064263	1.67625	1.50279	0.0	1.65124	0.651048	0.519172	1.19808	0.830062	0.875968
XLOC_043135	SMOC1	chr10	82083883	82084312	0.0	1.59077	0.0	2.86017	0.821345	0.546783	0.467639	1.04294	1.38729
XLOC_043136	SMOC1	chr10	82094128	82095039	0.959868	1.43524	1.50147	0.344092	0.674922	0.497695	0.521354	0.767191	0.586735
XLOC_090068	SMOC2	chr9	104151374	104263061	21.005	20.7272	21.1234	25.0779	20.7447	25.3507	30.0324	34.1577	21.3866
XLOC_090069	SMOC2	chr9	104289843	104338206	1.29291	0.349431	0.0	1.19819	0.31057	0.582849	0.747581	1.25382	0.79826
XLOC_091676	SMOC2	chr9	104288722	104289587	0.0	0.0	0.799498	1.19094	0.239482	0.423881	0.554614	0.612338	0.98174
XLOC_050550	SMOX	chr13	51408706	51422273	13.438	4.77935	9.45155	0.541499	0.336813	0.466731	0.661672	0.274986	0.358138
XLOC_062195	SMPD3;PRMT7	chr18	35828446	35861692	14.9416	18.3306	15.4188	24.5273	19.681	25.1801	18.0515	28.3516	17.9339
XLOC_062956	SMPD3;PRMT7	chr18	35828446	35861692	1.69481	2.28098	4.63995	2.65833	1.19276	1.58287	0.99932	1.35647	0.88834
XLOC_062957	SMPD3;PRMT7	chr18	35828446	35861692	3.95396	1.97129	3.09347	2.36309	1.85795	1.36932	0.959825	0.99124	0.691306

XLOC_062958	SMPD3;PRMT7	chr18	35828446	35861692	4.91848	5.86524	0.0	4.39996	4.10556	5.9887	4.5797	2.34965	2.96899
XLOC_062959	SMPD3;PRMT7	chr18	35828446	35861692	0.0	1.8466	5.79535	1.77082	1.92596	1.91826	0.333782	1.59903	0.969876
XLOC_062960	SMPD3;PRMT7	chr18	35828446	35861692	0.0	0.985257	2.5768	1.08262	1.20002	0.569012	0.694227	1.53369	0.67105
XLOC_062961	SMPD3;PRMT7	chr18	35828446	35861692	1.79095	1.42872	1.86837	1.81973	1.26297	1.24094	0.924913	1.73739	0.730794
XLOC_062962	SMPD3;PRMT7	chr18	35828446	35861692	1.57412	0.470448	3.69104	1.12783	1.10137	1.30091	1.54985	0.780445	0.82289
XLOC_062963	SMPD3;PRMT7	chr18	35828446	35861692	7.12601	4.2601	3.34242	1.91495	1.55303	2.50525	0.767496	1.27423	1.7394
XLOC_059898	SMPD4;MZT2B;<br>PRODH	chr17	74395612	74424008	5.71241	6.46103	6.06704	10.1696	12.9779	10.9969	10.751	11.4976	11.9962
XLOC_057855	SMYD3	chr16	31663383	31872636	27.8571	29.0917	33.1202	31.5467	28.7095	32.089	34.147	36.9576	29.0212
XLOC_057856	SMYD3	chr16	31947396	31958784	15.155	19.6966	17.4546	23.9809	21.5765	23.0066	23.2897	28.2252	20.4546
XLOC_057857	SMYD3	chr16	32103549	32108113	4.20348	6.56777	5.48226	8.83642	7.94971	8.64772	7.93641	10.8446	8.04909
XLOC_057858	SMYD3	chr16	32142783	32165499	0.0452002	0.581818	0.0	0.415999	0.00455348	0.0	1.73273E-4	0.778904	0.454642
XLOC_057859	SMYD3	chr16	32246907	32257139	4.82393	5.78061	5.82171	11.1986	10.0887	10.5166	10.5361	14.0021	11.155
XLOC_057860	SMYD3	chr16	32277125	32304106	6.85133	10.4053	7.41707	27.3022	27.9507	28.0433	24.7156	28.439	30.8275
XLOC_057861	SMYD3	chr16	32331831	32387731	23.4353	24.91	22.7073	27.1743	29.428	30.5004	31.4192	25.442	25.0212
XLOC_058097	SMYD3	chr16	31962741	32003290	9.66375	12.4002	14.3849	8.06126	8.81388	8.38646	9.1244	8.80251	9.25656
XLOC_058098	SMYD3	chr16	32017173	32079347	21.5289	18.977	24.0674	24.2584	22.4522	25.7475	25.3309	24.9017	21.0978
XLOC_058099	SMYD3	chr16	32084416	32100361	28.5886	57.1559	50.4146	41.5096	49.5403	58.1438	47.745	40.8767	64.043
XLOC_058100	SMYD3	chr16	32142783	32165499	4.66922	0.941975	2.94619	0.707506	0.73538	0.783823	1.14129	0.955011	0.778768
XLOC_058101	SMYD3	chr16	32230639	32246731	6.13334	9.31669	6.64562	9.6244	9.6376	8.96723	10.5814	12.6955	8.58751
XLOC_058797	SMYD3	chr16	31663383	31872636	10.6956	7.18537	4.17526	3.11012	3.70943	2.74387	2.58126	1.04667	2.3207
XLOC_058798	SMYD3	chr16	31872815	31875024	1.41014	2.32074	3.03493	3.63563	3.54274	4.44031	3.71171	4.00987	4.10346
XLOC_058799	SMYD3	chr16	31875741	31876470	1.26558	0.378357	0.0	0.453538	1.1836	0.916947	0.455692	0.377909	0.883135
XLOC_058800	SMYD3	chr16	31876623	31877365	1.23743	1.10985	0.967539	0.886925	1.35045	0.128101	0.445787	0.61605	1.07947
XLOC_058801	SMYD3	chr16	31897590	31898143	0.0	0.0	0.0	0.0	0.567561	0.188634	0.16294	0.18079	1.59197
XLOC_058802	SMYD3	chr16	31925032	31925910	2.00767	3.00184	0.0	8.99592	8.93745	8.01339	10.0786	8.019	7.88801
XLOC_058803	SMYD3	chr16	31944269	31945562	0.0	0.381072	0.0	0.171304	0.199566	0.198551	0.11601	0.127777	0.334112
XLOC_058804	SMYD3	chr16	31959369	31960601	7.40408	8.6579	9.4789	4.28421	6.48315	4.89479	5.51314	4.25159	5.76622
XLOC_058805	SMYD3	chr16	31960757	31961027	12.7095	9.47244	4.95306	4.55739	5.26483	4.49225	5.28154	2.39945	9.29505
XLOC_058806	SMYD3	chr16	31961129	31961341	0.0	14.4675	37.8169	11.0016	9.82958	10.8956	2.88113	7.74181	5.18966

XLOC_058807	SMYD3	chr16	31961658	31962587	5.62483	9.53213	7.33236	4.11687	4.76156	3.8892	4.9242	4.59066	4.83034
XLOC_058808	SMYD3	chr16	32016221	32016928	2.63251	6.2958	8.23269	4.24505	5.63973	5.99256	6.03709	5.76102	4.24726
XLOC_058809	SMYD3	chr16	32101795	32102946	0.0	0.435503	0.569509	0.130514	0.170943	0.151202	0.132368	0.291755	0.0636237
XLOC_058810	SMYD3	chr16	32129617	32130409	0.0	0.0	0.0	0.0	0.266785	0.118077	0.0	0.227271	0.0
XLOC_058811	SMYD3	chr16	32130946	32131549	0.0	0.0	0.0	0.290805	0.252341	0.0	0.0	0.160898	0.0
XLOC_058812	SMYD3	chr16	32229725	32230526	2.24793	2.0164	2.63678	5.23702	4.1217	4.54104	6.79643	5.49132	4.61022
XLOC_058813	SMYD3	chr16	32230639	32246731	4.87973	3.54488	2.72689	4.62436	4.42081	3.62053	4.81943	4.26218	4.44805
XLOC_058814	SMYD3	chr16	32230639	32246731	2.96127	5.31069	5.78708	2.78506	4.83818	3.67335	2.52238	2.6458	2.83909
XLOC_058815	SMYD3	chr16	32277125	32304106	1.83486	2.19561	1.43562	10.2538	9.29611	8.2632	7.07718	9.02111	10.1071
XLOC_058816	SMYD3	chr16	32277125	32304106	2.81161	1.68089	1.09902	2.01486	2.07933	2.47145	1.64086	1.81594	1.22561
XLOC_058817	SMYD3	chr16	32277125	32304106	0.0	3.90374	0.0	7.13815	2.8849	3.91454	4.12291	4.75386	11.1948
XLOC_058818	SMYD3	chr16	32313755	32314614	0.0	0.0	0.0	0.739146	1.20757	1.60306	0.372845	0.411673	1.08012
XLOC_053211	SNAI2;C14H8orf22	chr14	21492030	21668698	10.5647	18.1254	8.17814	98.835	104.083	98.623	153.397	142.249	112.113
XLOC_053961	SNAI2;C14H8orf22	chr14	21492030	21668698	0.0	0.0	0.0	4.90243	10.2968	6.54779	7.4336	6.52748	6.07058
XLOC_053962	SNAI2;C14H8orf22	chr14	21492030	21668698	0.0	0.0	0.0	4.70911	4.04478	2.21927	2.42806	5.12716	1.87884
XLOC_053963	SNAI2;C14H8orf22	chr14	21492030	21668698	0.0	0.507982	0.0	1.82671	1.84829	0.526417	3.03613	2.35628	1.92453
XLOC_053964	SNAI2;C14H8orf22	chr14	21492030	21668698	0.0	1.22731	0.0	4.41588	5.02192	4.60936	7.05068	3.96318	4.62227
XLOC_053965	SNAI2;C14H8orf22	chr14	21492030	21668698	1.13102	0.338166	0.0	2.83755	3.08838	2.22604	2.14327	2.02966	2.46752
XLOC_053966	SNAI2;C14H8orf22	chr14	21492030	21668698	10.1231	3.02433	0.0	7.32949	12.7874	8.12473	5.69716	14.9469	10.4314
XLOC_053967	SNAI2;C14H8orf22	chr14	21492030	21668698	0.0	0.0	5.72722	8.57899	11.5692	6.66066	7.24947	8.95121	5.05159
XLOC_053968	SNAI2;C14H8orf22	chr14	21492030	21668698	0.0	0.0	0.0	6.02405	6.41842	5.1423	7.20299	5.26604	5.45528
XLOC_053969	SNAI2;C14H8orf22	chr14	21492030	21668698	0.0	0.0	0.0	2.14404	3.35879	1.73451	2.91169	2.74143	4.38443
XLOC_082717	SNAP47	chr7	3303491	3331084	5.54237	4.10712	2.97274	4.48114	3.28121	3.76026	4.5432	5.93579	2.78484
XLOC_082718	SNAP47	chr7	3332969	3367994	3.99025	7.04695	4.85309	9.90221	7.22929	7.65934	10.6082	17.6552	8.58243
XLOC_084031	SNAP47	chr7	3332969	3367994	0.0	0.8046	0.701444	0.803748	0.700985	0.651237	1.21915	2.06231	1.25326
XLOC_084032	SNAP47	chr7	3332969	3367994	0.0	0.0	1.62656	1.02509	0.0	0.431148	1.222	1.34934	0.453916
XLOC_074604	SND1	chr4	92807786	92808091	4.88566	16.0222	11.4253	5.24464	5.56228	2.9748	7.44761	4.2025	8.00556
XLOC_064247	SNF8;GIP	chr19	38188190	38224172	8.55059	6.5216	4.44607	10.3947	9.18224	9.76087	13.2188	15.6705	13.4473

XLOC_065076	SNF8;GIP	chr19	38188190	38224172	0.898703	0.403337	1.75819	0.846137	1.33954	1.16866	0.698109	1.12942	1.37594
XLOC_044845	SNRNP27;MXD1;PCBP1	chr11	68211441	68386745	4.53707	2.62173	1.32361	4.51211	2.3959	3.27603	4.24959	8.49386	2.46941
XLOC_046433	SNRNP27;MXD1;PCBP1	chr11	68211441	68386745	0.0	0.0	0.0	0.528649	0.0	0.596797	0.0	3.35457	0.508097
XLOC_046434	SNRNP27;MXD1;PCBP1	chr11	68211441	68386745	0.0	0.0	4.35713	0.0	0.845275	1.13171	0.468951	0.53095	0.481499
XLOC_046435	SNRNP27;MXD1;PCBP1	chr11	68211441	68386745	1.18472	0.0	0.0	0.743046	0.184783	0.122685	0.213607	1.06234	0.413478
XLOC_046436	SNRNP27;MXD1;PCBP1	chr11	68211441	68386745	0.0	1.71685	0.0	0.257245	0.0	0.0	0.251463	0.561514	0.0
XLOC_046437	SNRNP27;MXD1;PCBP1	chr11	68211441	68386745	2.35995	0.0	0.0	0.63358	0.364652	0.727864	0.62475	0.463606	0.0
XLOC_046438	SNRNP27;MXD1;PCBP1	chr11	68211441	68386745	0.77913	0.233129	0.914618	0.628804	0.305757	0.527046	0.320771	0.979876	0.545428
XLOC_046439	SNRNP27;MXD1;PCBP1	chr11	68211441	68386745	0.0	0.0	0.0	1.0991	0.544496	1.08566	0.312877	1.04098	0.152677
XLOC_046440	SNRNP27;MXD1;PCBP1	chr11	68211441	68386745	0.0	0.0	0.0	0.0	0.0	0.979925	0.0	0.931685	0.0
XLOC_062445	SNRPD2;QPCTL	chr18	53681533	53744725	29.3922	24.3447	13.3037	16.2428	18.619	15.2952	15.8888	15.7488	14.8794
XLOC_052194	SNTA1	chr13	63431475	63432049	1.73663	0.0	2.71429	6.99793	4.58437	5.19749	7.4392	4.81225	4.38538
XLOC_062407	SNTB2	chr18	36505768	36524907	21.2759	11.2401	23.3976	8.98475	10.2589	7.58297	11.7994	12.8117	13.2356
XLOC_062987	SNTB2	chr18	36557890	36558358	0.0	0.0	3.63879	0.625436	2.16024	0.95816	0.411359	1.3734	0.202417
XLOC_069845	SNX27	chr3	19188196	19219784	20.6129	20.5867	20.2763	51.0407	56.5168	53.5052	54.3287	53.5647	65.0245
XLOC_070236	SNX27	chr3	19132139	19163082	8.02291	10.0682	10.5334	22.0728	20.0227	22.2037	24.5959	26.0291	21.6344
XLOC_089844	SNX3;NR2E1	chr9	42412440	42472283	6.18617	8.22224	9.78866	10.5191	10.3227	9.62977	8.81478	9.11263	10.7592
XLOC_090672	SNX3;NR2E1	chr9	42412440	42472283	1.71286	1.79299	2.00972	1.53522	0.870555	1.24417	1.16516	1.79918	1.19704
XLOC_050513	SNX5;BANF2	chr13	38346049	38528482	2.47383	2.0613	3.51242	0.341577	0.392893	0.610145	0.281069	0.341981	0.292516
XLOC_050812	SNX5;BANF2	chr13	38346049	38528482	28.062	21.1236	20.7002	58.3101	54.4873	55.3658	61.2625	60.3378	51.7484
XLOC_051653	SNX5;BANF2	chr13	38346049	38528482	1.68915	0.505337	0.440559	0.100963	0.0	0.0585332	0.0513469	0.0	0.0
XLOC_051654	SNX5;BANF2	chr13	38346049	38528482	0.0	0.0	0.641811	0.220625	0.0	0.0	0.074466	0.0	0.0
XLOC_051655	SNX5;BANF2	chr13	38346049	38528482	2.23749	1.67254	1.7497	0.0	0.0	0.0	0.0	0.0	0.0
XLOC_051656	SNX5;BANF2	chr13	38346049	38528482	0.0	0.296483	0.775411	0.0	0.0	0.0	0.0	0.0	0.0

XLOC_051657	SNX5;BANF2	chr13	38346049	38528482	0.0	0.543811	0.711134	0.0	0.0	0.0	0.0	0.0	0.0
XLOC_051658	SNX5;BANF2	chr13	38346049	38528482	0.0	0.419407	1.09687	0.0	0.218457	0.2902	0.0	0.0	0.122324
XLOC_051659	SNX5;BANF2	chr13	38346049	38528482	1.44433	0.64804	0.564963	0.0647361	0.0	0.0	0.0	0.0	0.0
XLOC_051660	SNX5;BANF2	chr13	38346049	38528482	0.0	0.461424	2.41348	0.138275	0.0	0.0	0.0	0.0	0.0
XLOC_051661	SNX5;BANF2	chr13	38346049	38528482	0.554843	0.497974	0.434141	0.0497459	0.0434897	0.0	0.0	0.0	0.0
XLOC_051662	SNX5;BANF2	chr13	38346049	38528482	0.0	0.350529	0.458393	0.0	0.0	0.0	0.0	0.0	0.0
XLOC_051663	SNX5;BANF2	chr13	38346049	38528482	0.0	0.447128	2.33871	0.0	0.0	0.0	0.0	0.0	0.0
XLOC_089567	SNX9	chr9	95868678	95952920	12.5188	17.8862	18.2153	15.0709	14.6597	14.9908	17.695	13.6381	18.8039
XLOC_091493	SNX9	chr9	95868678	95952920	1.18279	1.76817	1.84976	4.451	4.05864	4.89944	4.9051	2.35696	5.36647
XLOC_089845	SOBP	chr9	42964855	42970233	19.6939	17.6364	17.9454	7.23142	7.23517	6.97552	8.73778	8.81838	7.73475
XLOC_090673	SOBP	chr9	42963538	42964789	12.5666	9.69466	10.8667	4.80274	4.2992	5.29109	5.1777	4.90786	5.49281
XLOC_076757	SOCS2;CRADD	chr5	23505454	23676000	33.1113	42.8163	42.1632	24.8413	22.8894	25.5119	25.1824	26.3243	22.9833
XLOC_088011	SORBS3	chr8	70382213	70384481	5.14019	3.89653	4.55927	0.676074	1.23768	0.82048	0.470688	0.448471	0.779781
XLOC_086908	SORBS3;KIAA1967;BIN3;PDLIM2	chr8	70384550	70439570	9.72812	8.97158	9.35073	1.99532	2.01732	1.64644	2.84728	2.42818	2.05604
XLOC_056293	SORL1	chr15	32692005	32696467	3.52829	4.22382	5.2607	0.0753491	0.026421	0.0	0.0308709	0.0339013	0.0588564
XLOC_064012	SOST;DUSP3;MPP3	chr19	44234318	44344781	133.116	76.6665	59.6946	110.677	146.716	151.709	128.571	106.416	124.822
XLOC_065210	SOST;DUSP3;MPP3	chr19	44234318	44344781	39.6837	42.7043	40.7446	86.126	119.467	99.7013	99.8073	80.0617	92.9075
XLOC_065211	SOST;DUSP3;MPP3	chr19	44234318	44344781	13.0842	50.7245	57.8107	79.1759	107.27	92.7102	83.7471	68.2919	94.8999
XLOC_076969	SOX10	chr5	110259654	110286223	21.5383	30.6336	29.797	49.0186	50.0935	51.0276	54.4511	52.9011	63.1274
XLOC_038049	SOX14	chr1	132417556	132467083	7.84061	10.1446	8.84776	3.39415	3.25505	2.67893	3.62954	3.69026	3.62896
XLOC_076386	SOX5	chr5	86804170	86817046	39.8703	42.4618	40.2344	42.4202	43.362	39.551	52.6571	56.6833	42.6917
XLOC_076387	SOX5	chr5	86817214	86835760	48.4767	50.8374	48.6352	44.1027	43.7558	41.1561	51.8907	58.0435	51.9847
XLOC_076899	SOX5	chr5	86890905	86906942	0.0	0.122988	0.0	0.0	0.0	0.0	0.0	0.0	0.0
XLOC_078213	SOX5	chr5	86804170	86817046	8.05553	3.09671	6.29916	7.63035	6.82249	6.55579	8.51235	5.16172	7.63202
XLOC_078214	SOX5	chr5	86804170	86817046	2.03593	5.17488	7.96126	6.38569	6.67742	5.69838	8.5609	4.67502	5.33242
XLOC_078215	SOX5	chr5	86804170	86817046	5.70614	5.11006	8.90849	5.35965	5.70924	5.84931	9.73361	2.50756	4.45426

XLOC_078216	SOX5	chr5	86835880	86837840	18.0556	13.086	17.5841	15.2194	14.8312	15.7829	17.9468	18.8094	19.2397
XLOC_078217	SOX5	chr5	86838051	86839396	1.82713	0.728789	0.476525	1.09205	1.81326	1.01274	0.943395	1.0389	2.55611
XLOC_078218	SOX5	chr5	86839670	86840282	0.0	0.951233	0.0	0.285055	1.48444	0.821925	1.42401	0.473319	2.35702
XLOC_055741	SOX6	chr15	36596038	36608992	114.764	115.71	105.558	169.898	167.054	199.41	224.546	233.356	132.15
XLOC_055742	SOX6	chr15	36772061	36773928	16.626	21.6458	24.9663	18.615	18.6479	21.9709	22.1752	24.0793	19.6168
XLOC_055743	SOX6	chr15	36774387	36795316	54.9168	48.5751	64.7992	52.6792	45.4495	48.3251	49.3745	53.2473	49.0337
XLOC_055744	SOX6	chr15	36998839	37010172	8.5799	6.9972	6.41238	38.9006	36.4422	38.7373	49.0585	53.9058	34.664
XLOC_056333	SOX6	chr15	36596038	36608992	18.3636	20.7242	15.9403	14.795	15.0135	14.9235	27.3454	17.1009	21.2971
XLOC_056334	SOX6	chr15	36596038	36608992	12.3932	12.9311	14.4891	14.994	9.8082	15.6539	14.9646	11.7129	17.0644
XLOC_056335	SOX6	chr15	36679645	36679972	4.25953	1.27024	0.0	3.42816	3.24565	0.866865	1.82041	1.63843	2.20732
XLOC_056336	SOX6	chr15	36680138	36681254	0.0	0.902789	0.59029	2.77317	2.77531	2.42881	2.74269	1.81386	2.37386
XLOC_056337	SOX6	chr15	36759279	36759646	6.90178	6.17783	2.69237	0.617203	1.58652	1.41029	2.09168	5.35547	2.09112
XLOC_056338	SOX6	chr15	36761306	36762734	1.13851	0.170301	0.0	1.58215	1.33843	1.4794	1.09004	1.82871	0.945827
XLOC_056339	SOX6	chr15	36763214	36763606	0.0	0.920404	0.0	0.275829	1.18469	1.26285	0.80609	3.30395	0.534549
XLOC_056340	SOX6	chr15	36767899	36769470	3.06997	2.2962	1.20112	0.779901	1.32393	1.11737	1.21434	0.719628	2.32718
XLOC_056341	SOX6	chr15	36769602	36770774	4.99036	2.34572	3.9041	2.74801	3.46011	2.96172	3.56578	4.50085	2.30547
XLOC_056342	SOX6	chr15	36770872	36771707	102.485	90.3385	87.6607	104.081	106.665	114.725	124.129	132.294	87.5017
XLOC_056343	SOX6	chr15	36774387	36795316	16.5097	8.6307	20.9584	8.49779	7.5111	8.92706	10.2525	8.34045	11.8461
XLOC_056344	SOX6	chr15	36774387	36795316	6.82635	8.5762	6.94273	3.18213	4.16916	2.9783	5.21753	3.55852	4.65426
XLOC_056345	SOX6	chr15	36774387	36795316	6.08706	6.66734	3.17021	3.45101	3.77194	2.92627	4.14258	2.60111	1.4119
XLOC_056346	SOX6	chr15	36774387	36795316	16.0835	7.19684	25.0855	9.40731	5.41337	8.0908	3.94214	4.50258	3.45466
XLOC_056347	SOX6	chr15	36774387	36795316	15.9834	11.5177	15.3459	5.53572	7.05152	7.84681	7.00162	5.38672	7.36556
XLOC_056348	SOX6	chr15	36774387	36795316	25.0418	17.2652	45.1516	13.1068	15.9786	13.1052	16.789	14.0745	16.0914
XLOC_056349	SOX6	chr15	36774387	36795316	5.92373	16.648	22.2331	7.53663	7.94567	6.87041	8.43748	4.83957	7.64935
XLOC_056350	SOX6	chr15	36937847	36938482	0.0	0.0	1.18403	1.89941	0.589083	0.939242	0.135731	0.300626	0.660031
XLOC_056351	SOX6	chr15	36988941	36990371	1.13672	0.340066	0.444712	0.560528	1.06907	0.886248	1.03652	0.228232	1.44137
XLOC_056352	SOX6	chr15	36991054	36991765	0.0	0.0	0.0	1.4048	2.23976	1.08175	0.940314	1.17001	1.48155
XLOC_056353	SOX6	chr15	36991976	36992775	0.0	1.01134	0.0	1.01025	2.46307	1.40157	1.32274	0.674474	1.57431
XLOC_056354	SOX6	chr15	36992940	36993389	0.0	1.48192	3.87534	2.44238	2.87287	0.509835	1.31094	0.73013	0.861973
XLOC_056355	SOX6	chr15	36993660	36994427	0.0	0.0	0.0	1.3822	1.1105	0.860194	1.2837	1.65521	1.24246
XLOC_056356	SOX6	chr15	36994602	36995076	2.28539	0.682563	0.0	2.04535	1.76669	1.41037	1.41358	0.674039	1.39028
XLOC_056357	SOX6	chr15	36995244	36995556	4.6678	2.78343	0.0	2.50498	5.32126	8.53409	3.17237	1.7882	4.43024
XLOC_056358	SOX6	chr15	36997803	36998542	0.0	0.0	0.972524	2.34018	3.49033	2.96141	2.5762	2.35288	1.51903

XLOC_056359	SOX6	chr15	36998839	37010172	0.0	2.78353	0.0	2.50508	7.80451	2.8447	7.13784	5.36459	4.43024
XLOC_056360	SOX6	chr15	36998839	37010172	2.934	1.75161	4.58024	7.87345	7.67298	6.91429	5.12595	6.01129	6.61463
XLOC_056361	SOX6	chr15	36998839	37010172	0.0	1.30844	17.1051	12.5562	13.6959	13.3861	14.2233	11.7985	14.0166
XLOC_056362	SOX6	chr15	36998839	37010172	2.36768	4.94964	7.3964	7.62784	11.8893	6.57191	10.654	9.3016	10.9022
XLOC_056363	SOX6	chr15	36998839	37010172	0.0	1.94524	0.0	3.78899	2.52901	4.20116	2.18264	1.45127	1.84274
XLOC_056364	SOX6	chr15	36998839	37010172	0.0	1.1957	0.0	3.82204	3.94629	3.86225	1.55824	1.45854	1.9765
XLOC_056365	SOX6	chr15	36998839	37010172	0.0	0.0	0.0	1.59805	2.16502	1.048	0.673122	0.250035	0.664555
XLOC_056366	SOX6	chr15	37046556	37046900	0.0	0.0	0.0	0.692873	0.0	0.0	0.333158	0.0	1.00489
XLOC_065808	SP140L	chr2	119167770	119208935	2.77919	1.17797	0.0	7.45044	6.99863	9.06258	1.26191	0.829443	3.50479
XLOC_066475	SP140L	chr2	119167770	119208935	4.99111	3.85106	1.23829	10.2394	8.01277	9.99338	9.78197	14.3973	12.3053
XLOC_050476	SPAG6	chr13	23779279	23791209	0.0	0.0	0.0	0.0568359	0.0	0.0658799	0.0577387	0.0635967	0.0
XLOC_063976	SPAG9	chr19	36348481	36460967	23.6013	25.041	25.1145	4.69208	4.301	5.79029	3.70127	3.61003	4.67588
XLOC_065034	SPAG9	chr19	36348481	36460967	1.12354	1.1764	1.3186	0.0503653	0.0	0.0583972	0.0512285	0.225591	0.0982488
XLOC_065035	SPAG9	chr19	36348481	36460967	0.978152	2.04739	0.764943	0.0876528	0.1528	0.101412	0.0885077	0.0	0.0
XLOC_065036	SPAG9	chr19	36348481	36460967	0.0	1.01618	1.32882	0.076133	0.0	0.0881428	0.0770485	0.0	0.0742017
XLOC_065038	SPAG9	chr19	36348481	36460967	0.0	0.0	1.67417	0.0	0.0	0.0	0.0	0.0	0.0
XLOC_065039	SPAG9	chr19	36348481	36460967	0.0	1.20909	1.054	0.0	0.0	0.0	0.0	0.0	0.0
XLOC_065040	SPAG9	chr19	36348481	36460967	1.01238	0.0	1.58335	0.0	0.0	0.0	0.0	0.0	0.0
XLOC_062695	SPATA2L	chr18	14619680	14620943	0.654222	0.391407	0.0	0.41055	0.563621	0.883643	0.416921	0.39365	0.743508
XLOC_062696	SPATA2L	chr18	14621026	14622515	1.62952	1.78753	0.425016	1.12011	1.40511	1.2989	0.792843	0.600011	0.712563
XLOC_065816	SPATA3	chr2	119603226	119649870	22.5609	22.6733	17.0181	97.5293	118.033	106.9	134.096	102.366	165.568
XLOC_066483	SPATA3	chr2	119603226	119649870	13.2819	16.0461	15.0857	24.4073	28.2295	25.1094	22.9685	20.0701	29.0275
XLOC_068377	SPATA3	chr2	119603226	119649870	12.9682	12.3369	8.29688	13.6264	17.1016	16.1159	14.2432	10.6898	14.8128
XLOC_068378	SPATA3	chr2	119603226	119649870	23.4818	14.0506	0.0	36.3009	38.2271	30.585	28.9759	43.0353	40.3304
XLOC_070075	SPATA6	chr3	98382154	98427927	1.14284	2.35893	3.3992	0.338033	0.470518	0.890255	0.128965	0.143155	0.211823
XLOC_071860	SPATA6	chr3	98382154	98427927	0.412684	0.864356	1.61478	0.148023	0.0323842	0.128825	0.0	0.0	0.0722184
XLOC_071861	SPATA6	chr3	98382154	98427927	1.65789	0.743781	1.94528	0.0742998	0.0648309	0.0860321	0.0	0.0	0.0
XLOC_071862	SPATA6	chr3	98382154	98427927	0.0	1.45007	2.84429	0.0	0.094539	0.0	0.0	0.0	0.105785
XLOC_071863	SPATA6	chr3	98382154	98427927	0.0	0.240491	3.14489	0.0720713	0.188692	0.0	0.0	0.0804864	0.0
XLOC_071864	SPATA6	chr3	98382154	98427927	2.38327	0.711732	1.86125	0.0	0.0	0.244985	0.0	0.0	0.0
XLOC_071865	SPATA6	chr3	98382154	98427927	2.09356	0.625383	0.0	0.0	0.162085	0.0	0.0	0.0	0.0
XLOC_071866	SPATA6	chr3	98428052	98430731	0.573644	3.08991	4.04082	0.102892	0.270366	0.209081	0.0262918	0.0866835	0.175756
XLOC_071867	SPATA6	chr3	98430901	98431959	1.60633	3.84358	3.76968	0.0719913	0.439796	0.333481	0.218754	0.0	0.140351

XLOC_083119	SPATA9	chr7	97480928	97529360	21.3101	29.8415	21.3013	39.905	42.3672	43.5459	43.2343	49.7374	49.5193
XLOC_055525	SPI1	chr15	78406706	78413885	2.86574	3.64312	2.80243	9.82617	10.2623	9.07776	8.66363	7.10804	8.64151
XLOC_076861	SPIC;ARL1	chr5	65607574	65698044	27.5958	30.1188	24.318	83.7229	75.2508	82.6139	99.047	91.8413	83.7102
XLOC_037859	SPICE1	chr1	58591142	58595946	32.1437	40.9957	29.6517	85.4742	63.3895	71.5634	78.382	77.2717	94.5153
XLOC_038374	SPICE1	chr1	58527387	58587514	6.93244	7.40648	10.3669	5.80437	5.50686	5.07011	5.77429	6.37521	6.3145
XLOC_039379	SPICE1	chr1	58588945	58589184	0.0	0.0	0.0	0.0	0.0	0.862176	0.0	0.797904	0.0
XLOC_039380	SPICE1	chr1	58589820	58590362	0.0	1.12412	0.0	0.0	0.437668	0.775873	0.334885	0.743381	0.654884
XLOC_087007	SPIN1	chr8	91217020	91313365	4.47628	7.67623	8.62792	3.19425	3.99521	4.00367	3.70483	4.27976	2.89527
XLOC_050638	SPINLW1	chr13	75089865	75134834	0.0	0.414872	0.0	0.262487	0.169404	0.224524	0.127263	0.0776351	0.107916
XLOC_063944	SPNS2	chr19	25505481	25604486	72.4537	113.915	76.8298	12.786	15.3294	14.5116	14.4332	13.9496	9.15553
XLOC_064822	SPNS2	chr19	25622271	25623660	2.93642	5.9735	4.13558	0.526526	1.05847	0.488345	0.214131	0.766271	0.564889
XLOC_055440	SPON1	chr15	39006272	39036634	5.68525	5.77575	5.33223	1.52719	2.36844	1.85023	2.0452	1.41114	2.15139
XLOC_055441	SPON1	chr15	39076911	39087069	2.71515	2.82856	2.15526	2.17557	1.37833	4.07186	0.610197	0.676467	1.18648
XLOC_055442	SPON1	chr15	39112812	39146862	38.3665	55.1574	44.0583	52.9858	40.1671	50.0057	55.1386	67.7873	62.5865
XLOC_055752	SPON1	chr15	39094828	39112685	2.46172	6.4357	8.81886	11.2505	9.69439	9.55821	12.2147	12.4413	11.2596
XLOC_056424	SPON1	chr15	38990345	38992035	4.24737	2.54157	2.95438	6.22042	4.9601	4.36887	6.5959	8.06372	5.61465
XLOC_056425	SPON1	chr15	39036980	39037647	2.8392	3.39472	2.21953	0.635815	0.773502	0.440382	1.01941	0.0	1.23757
XLOC_056426	SPON1	chr15	39037749	39038166	5.57433	1.6641	2.17581	0.748013	1.71697	0.571638	1.46457	0.27239	0.967187
XLOC_056427	SPON1	chr15	39091545	39093435	1.25235	0.87433	0.326683	2.0588	1.6706	2.47587	2.13765	2.35121	1.89932
XLOC_056428	SPON1	chr15	39093845	39094426	0.0	2.04151	1.33475	2.60004	3.84644	3.70214	4.11753	4.05801	4.01587
XLOC_056429	SPON1	chr15	39195464	39196064	4.90373	0.488496	3.83259	1.17109	0.508059	1.01279	0.58459	0.323934	0.284773
XLOC_056430	SPON1	chr15	39196283	39196968	0.0	0.819756	0.0	0.245659	1.06771	0.992789	0.615938	0.545209	0.358665
XLOC_080073	SPP1	chr6	38122976	38254086	7.31055	9.92901	8.32518	1.70515	1.82631	2.05493	1.78361	1.81357	1.10452
XLOC_080831	SPP1	chr6	38122976	38254086	0.660799	1.18602	0.0	0.177717	0.155252	0.686529	0.120311	0.463837	0.288832
XLOC_041899	SPRED1	chr10	33930161	33951218	3.37859	5.60932	8.12126	6.65002	9.5617	7.95824	6.52426	8.14859	9.71371
XLOC_041900	SPRED1	chr10	33996569	34019650	3.55481	5.10574	4.72953	7.74641	6.47471	5.95744	6.80161	6.90569	8.2754
XLOC_042558	SPRED1	chr10	33930161	33951218	0.0	1.49049	0.433152	0.595581	0.694245	0.805712	0.656385	0.389082	0.774593
XLOC_042559	SPRED1	chr10	33987861	33988733	1.01227	0.0	0.791673	0.0907139	0.237155	0.209877	0.732375	0.101069	0.618645
XLOC_042560	SPRED1	chr10	33989323	33991965	0.582187	0.435543	0.683496	0.234954	0.297248	0.424378	0.186768	0.205263	0.254816
XLOC_042561	SPRED1	chr10	33992889	33993305	2.79794	2.50578	2.1842	0.500599	0.430867	1.14762	0.489985	0.273409	1.21361
XLOC_042562	SPRED1	chr10	33993460	33995482	0.0	0.580323	0.910692	0.347837	0.426229	0.201842	0.212937	0.195138	0.441261
XLOC_042563	SPRED1	chr10	33995765	33996451	1.36856	0.409103	3.20977	0.612987	0.319712	0.849365	0.245921	0.408146	0.715979
XLOC_042564	SPRED1	chr10	33996569	34019650	1.48064	0.885115	1.15741	0.265244	0.460779	0.0	0.531028	0.146989	0.0

XLOC_059749	SPRY1	chr17	34730666	34850323	15.2171	18.3121	12.8175	8.94131	4.99652	6.56559	6.04553	13.0487	5.69694
XLOC_060139	SPRY1	chr17	34730666	34850323	0.0	0.225228	1.76727	0.202492	0.235733	0.312746	0.273706	0.226265	0.0658047
XLOC_060140	SPRY1	chr17	34730666	34850323	0.0	0.0	0.0	0.182192	0.0793997	0.105378	0.0	0.20298	0.0
XLOC_060141	SPRY1	chr17	34730666	34850323	0.0	0.140374	0.0	0.0	0.0368084	0.0	0.0428609	0.0	0.0
XLOC_060142	SPRY1	chr17	34730666	34850323	6.80872	17.5078	12.9809	19.6658	11.694	9.77042	9.58414	13.0043	11.7188
XLOC_060143	SPRY1	chr17	34730666	34850323	15.4293	0.922998	1.20705	0.0	0.0	0.0	0.0	0.0	0.0
XLOC_060144	SPRY1	chr17	34730666	34850323	0.0	1.58839	0.0	0.356999	0.206967	0.82461	0.47774	0.792692	0.69504
XLOC_048156	SPRY2	chr12	55602932	55820772	29.7604	27.5977	35.669	11.0307	12.8523	11.9627	12.9201	11.5008	12.3623
XLOC_077472	SPRYD3	chr5	27054306	27055247	0.0	0.0	0.0	0.0827322	0.0721411	0.0	0.0	0.0	0.161241
XLOC_077473	SPRYD3	chr5	27060438	27061687	0.0	0.198212	0.0	0.0594017	0.051892	0.275363	0.0	0.0	0.11585
XLOC_077474	SPRYD3	chr5	27063808	27064674	0.0	0.0	0.0	0.182963	0.159431	0.211643	0.0	0.0	0.178247
XLOC_048632	SPRYD7	chr12	19493614	19493933	0.0	0.0	0.0	0.799187	1.70001	0.454229	0.380707	3.00136	2.31422
XLOC_048633	SPRYD7	chr12	19494130	19495128	0.0	0.0	0.0	1.00264	0.538258	0.892902	0.780408	0.946843	1.20272
XLOC_048634	SPRYD7	chr12	19511197	19511670	0.0	0.0	0.0	0.615546	1.41775	0.235797	0.405108	0.901489	1.39465
XLOC_045067	SPTAN1	chr11	99145098	99149080	0.877205	0.787048	0.0	0.393108	1.16584	0.182027	0.0	0.175458	0.766239
XLOC_047248	SPTAN1	chr11	99144349	99144940	0.0	0.0	0.0	0.149421	1.29617	0.689068	0.14909	0.165262	0.435975
XLOC_062235	SPTBN4;SHKBP1	chr18	50128221	50196356	20.6435	29.3852	21.8343	33.6001	33.0914	32.9451	35.3453	34.7108	38.3167
XLOC_063147	SPTBN4;SHKBP1	chr18	50128221	50196356	70.4129	52.4435	54.0897	22.1353	29.6597	32.994	31.0417	22.2635	26.7254
XLOC_041715	SPTLC2	chr10	89744994	89820205	37.1622	32.1794	31.9909	15.6797	15.9119	16.1715	21.8219	18.2034	15.3049
XLOC_045693	SRBD1	chr11	27883110	27883348	17.3455	31.0596	13.5324	13.2896	12.9459	5.23005	13.3913	16.1275	10.4296
XLOC_050925	SRC	chr13	67001086	67006577	1.03342	2.57236	1.34798	1.16018	2.47356	1.33499	2.04481	1.44197	1.64064
XLOC_052364	SRC	chr13	66999001	67000313	1.25291	1.31182	0.0	0.673946	1.03056	0.846284	0.741801	0.502761	0.60248
XLOC_052365	SRC	chr13	67000515	67000983	4.6591	0.0	0.0	1.25087	0.36004	0.47908	0.0	1.6023	1.41692
XLOC_052366	SRC	chr13	67001086	67006577	0.0	1.17504	3.0732	0.492998	1.16796	1.46822	1.21307	1.02268	0.892517
XLOC_076848	SRGAP1	chr5	49959916	50058684	41.7275	57.4785	52.5925	15.6865	15.8357	14.3665	17.8111	14.4483	14.7572
XLOC_077867	SRGAP1	chr5	49958232	49959165	0.932636	1.11565	0.729458	0.16717	0.655918	0.580388	0.591287	0.186419	0.488696
XLOC_057748	SRGAP2	chr16	3871296	3941164	14.9855	18.0374	17.1048	16.2416	16.4183	16.6201	18.4476	23.6179	20.7835
XLOC_057992	SRGAP2	chr16	3871296	3941164	1.43546	4.39005	2.21153	0.751484	0.818502	0.98858	0.464297	0.942129	0.614401
XLOC_057993	SRGAP2	chr16	3871296	3941164	1.02769	0.516312	3.32295	1.19656	1.12065	0.732637	0.514338	0.683316	0.58744
XLOC_057994	SRGAP2	chr16	3945939	3976585	2.57693	1.56106	3.6506	0.672884	1.01121	0.929284	1.06747	1.28976	1.45688
XLOC_057995	SRGAP2	chr16	3978952	4003794	10.0372	10.0561	13.8078	9.63847	9.56919	8.47854	9.74533	10.5254	8.38581
XLOC_057996	SRGAP2	chr16	4029587	4077887	33.7609	41.973	32.849	127.335	122.359	124.238	129.08	133.531	136.335

XLOC_058240	SRGAP2	chr16	3855436	3856127	1.35573	1.6211	0.0	1.09305	1.37258	1.68294	0.974692	0.808767	0.709301
XLOC_058241	SRGAP2	chr16	3856553	3857259	0.0	1.57682	1.03096	1.0632	2.36269	1.91017	0.830094	0.524669	0.919993
XLOC_058242	SRGAP2	chr16	3861177	3863048	4.22077	4.79897	4.95456	2.08162	1.39099	1.18575	1.35073	1.74041	1.21868
XLOC_058243	SRGAP2	chr16	3863189	3864821	14.7148	17.61	22.6452	3.60632	3.7312	4.28514	2.77703	3.30231	4.16181
XLOC_058244	SRGAP2	chr16	3864928	3865177	15.4352	18.4158	12.0359	0.693944	4.04576	2.33085	5.0595	1.44334	2.65297
XLOC_058245	SRGAP2	chr16	3865476	3866158	26.201	34.2142	32.3421	5.80597	5.58348	7.84472	5.45047	5.20861	6.73318
XLOC_058246	SRGAP2	chr16	3866426	3867185	2.4049	14.7398	6.58158	1.72377	1.96897	1.7431	1.51713	1.79668	1.99328
XLOC_058247	SRGAP2	chr16	3871296	3941164	0.889068	1.32948	0.695413	0.796838	0.486497	0.737917	0.0805908	0.622318	0.621254
XLOC_058248	SRGAP2	chr16	3871296	3941164	4.64146	2.31206	0.0	0.27714	0.481223	0.159886	0.554213	0.306936	0.404443
XLOC_058249	SRGAP2	chr16	3871296	3941164	0.0	1.5722	5.4825	0.0785265	0.410978	0.181824	0.0794382	0.262872	0.382657
XLOC_058250	SRGAP2	chr16	3871296	3941164	1.19847	1.43328	0.937113	0.107379	0.467265	0.12412	0.216033	0.119387	0.31369
XLOC_058251	SRGAP2	chr16	3871296	3941164	1.45012	0.650643	2.26891	1.10492	1.19183	2.03309	1.38437	0.944434	1.39412
XLOC_058252	SRGAP2	chr16	3871296	3941164	1.03282	0.927005	1.61635	1.43537	1.41715	1.28882	1.27264	0.830018	1.49055
XLOC_058253	SRGAP2	chr16	3943995	3944599	1.61993	0.968251	2.53221	0.0	0.377674	0.334602	0.579507	1.12381	0.564473
XLOC_058254	SRGAP2	chr16	3945006	3945216	12.4499	0.0	0.0	1.13447	0.0	0.0	1.97482	1.13767	2.13845
XLOC_058255	SRGAP2	chr16	3945939	3976585	5.45894	1.08741	2.84381	0.16293	0.282373	0.187695	0.324294	0.539705	0.475204
XLOC_058256	SRGAP2	chr16	3945939	3976585	1.03694	0.0	0.810955	0.0929226	0.161925	0.322439	0.281207	0.310505	0.0905239
XLOC_058257	SRGAP2	chr16	4029587	4077887	0.0	1.72407	2.25371	1.80788	0.889164	1.18363	1.00966	0.282668	1.00158
XLOC_058258	SRGAP2	chr16	4029587	4077887	0.0	1.06028	2.77123	0.317655	0.272652	0.725469	0.61414	2.06598	0.614839
XLOC_058259	SRGAP2	chr16	4029587	4077887	0.0	4.32762	1.88606	4.32236	3.91709	1.73746	1.06453	2.37134	2.30767
XLOC_058260	SRGAP2	chr16	4081220	4083871	15.0822	13.5399	12.9396	4.08385	2.6656	3.98686	4.14732	5.5806	3.27527
XLOC_051869	SRMS	chr13	54591553	54592284	0.0	1.13112	0.986074	0.790926	0.491462	0.913774	0.681209	0.502148	0.660052
XLOC_081406	SRP72	chr6	73528917	73529072	174.675	120.819	70.2377	140.876	191.551	158.532	212.923	201.747	221.121
XLOC_092117	SRPX2	chrX	50841108	50893073	0.756897	1.43487	1.26742	3.09061	2.12154	2.7807	1.3425	1.5177	1.47078
XLOC_092434	SRPX2	chrX	50894223	50897326	0.0	0.111693	0.0	0.0334736	0.0	0.0	0.0	0.0375639	0.0
XLOC_043126	SRSF5	chr10	81864318	81864900	1.7039	1.01835	2.66322	0.457751	0.264656	1.05529	0.304306	0.843469	0.445163
XLOC_050873	SS18L1	chr13	55539179	55570919	17.5301	17.1055	14.7959	24.2237	24.8739	23.0494	17.6309	20.8781	18.9816
XLOC_051895	SS18L1	chr13	55539179	55570919	2.57864	3.07977	6.04031	2.99936	2.98333	1.8533	0.906703	1.51586	1.11933
XLOC_051896	SS18L1	chr13	55539179	55570919	2.81975	5.05052	6.60353	3.53143	3.03916	2.31283	2.22132	1.92839	1.46757
XLOC_051897	SS18L1	chr13	55539179	55570919	8.99131	0.0	0.0	2.41235	1.36829	2.28484	0.0	1.72532	1.94025
XLOC_051898	SS18L1	chr13	55539179	55570919	1.36341	2.44539	1.0659	1.58777	2.22969	1.26928	1.59264	1.49104	1.06994
XLOC_051899	SS18L1	chr13	55539179	55570919	1.54057	2.76267	6.02087	1.51779	2.03665	2.22856	3.03556	1.98681	2.14789
XLOC_051900	SS18L1	chr13	55539179	55570919	2.35715	1.64506	1.22928	1.76071	1.47534	1.14195	1.21308	1.41605	1.23579

XLOC_051901	SS18L1	chr13	55539179	55570919	5.73302	1.71287	2.9863	1.88203	1.77807	2.36409	1.19007	2.26476	1.66299
XLOC_051902	SS18L1	chr13	55539179	55570919	0.0	5.14822	2.69238	4.62902	2.64433	6.34629	1.79293	1.00429	1.49366
XLOC_051903	SS18L1	chr13	55539179	55570919	2.05538	0.614933	1.60832	1.88895	1.81305	1.97707	1.36018	1.08404	1.48315
XLOC_051904	SS18L1	chr13	55539179	55570919	4.66141	8.35909	4.85803	1.53081	2.65801	2.24752	1.39134	1.23298	2.30163
XLOC_051905	SS18L1	chr13	55539179	55570919	5.36447	2.67158	4.19202	1.92138	1.94296	2.02931	1.27549	2.29918	0.934066
XLOC_083096	SSBP2	chr7	83880402	83900344	23.5328	19.8136	20.5287	30.3219	31.8192	31.086	35.167	38.5129	43.6411
XLOC_083737	SSBP2	chr7	83755818	83757805	124.276	139.012	116.9	219.558	206.219	217.782	253.951	261.679	233.971
XLOC_083738	SSBP2	chr7	83775505	83793403	0.0	0.63723	0.0	0.127313	0.222361	0.368767	0.0	0.0	0.124132
XLOC_083739	SSBP2	chr7	83934570	84086490	0.55335	0.643197	2.76156	0.240702	0.0761753	0.242293	0.381513	0.315998	0.133296
XLOC_085236	SSBP2	chr7	83879365	83880285	0.948538	0.850989	0.741883	0.425043	0.0	0.295113	0.0858846	0.09478	0.0828332
XLOC_085237	SSBP2	chr7	83880402	83900344	0.0	0.62179	1.62657	0.0	0.161168	0.0	0.553814	0.615431	0.543073
XLOC_085238	SSBP2	chr7	83900502	83901020	0.0	0.0	0.0	1.07772	0.0	0.826894	1.06912	0.989595	0.523629
XLOC_085239	SSBP2	chr7	83932858	83933481	0.0	0.0	0.0	0.278329	0.0	0.0	0.0	0.0	0.13539
XLOC_085240	SSBP2	chr7	83933540	83934350	2.21692	0.994307	0.0	0.0	0.0	0.0	0.200137	0.0	0.580453
XLOC_070398	SSBP3	chr3	92483954	92485891	34.9515	11.799	22.0416	0.0	0.0	0.233595	0.0	0.0	0.0
XLOC_071677	SSBP3	chr3	92428063	92429425	54.0403	61.6121	64.8328	61.6917	41.5426	52.2342	55.5948	83.0321	42.9993
XLOC_044498	SSNA1;TPRN	chr11	105899700	105924303	15.4465	18.3433	16.8035	1.08061	0.843398	1.03582	0.816163	0.756227	0.871294
XLOC_055569	SSRP1	chr15	81802811	81827007	24.9252	30.3313	25.5314	13.7209	19.8992	13.84	9.57745	10.3521	13.816
XLOC_057186	SSRP1	chr15	81802811	81827007	28.025	33.4271	38.2322	6.91823	7.89324	7.06394	5.78182	2.63297	4.21699
XLOC_057932	SSU72;NADK;GNB1	chr16	52031261	52208077	12.2242	13.1954	10.6653	2.51388	3.3989	2.93668	3.37923	3.03648	2.71103
XLOC_070085	ST3GAL3	chr3	102734754	102772500	2.86101	1.31995	2.20949	2.3261	1.62284	2.04081	3.41924	2.70516	2.51829
XLOC_070454	ST3GAL3	chr3	102791062	102920640	31.6415	43.4807	35.2536	11.3384	5.1597	6.44633	13.3157	11.3314	9.59446
XLOC_044044	ST3GAL5	chr11	48837277	48850633	7.77783	12.0464	14.0311	1.69881	1.37281	1.75332	0.533803	1.23451	1.40195
XLOC_044741	ST3GAL5	chr11	48865538	48871439	9.22674	4.19507	6.12635	4.71321	4.47576	4.73072	2.38286	1.80568	4.09866
XLOC_045925	ST3GAL5	chr11	48837277	48850633	0.0	1.98224	1.03688	0.396036	0.277204	0.0459494	0.161475	0.0444086	0.193206
XLOC_045926	ST3GAL5	chr11	48850751	48851373	6.22859	6.05009	7.30269	0.557855	0.242146	0.804398	0.139422	0.772203	0.542721
XLOC_045927	ST3GAL5	chr11	48856710	48856975	6.63604	7.91399	10.3452	7.14268	4.49254	9.37529	3.84858	6.87616	5.13715
XLOC_045928	ST3GAL5	chr11	48863176	48863395	0.0	0.0	0.0	0.993559	0.811949	1.09786	0.0	0.0	0.0
XLOC_039200	ST3GAL6	chr1	42698820	42699677	1.03397	2.16431	4.8518	0.0	0.0	0.107174	0.280418	0.0	0.090266
XLOC_055449	ST5	chr15	44375167	44437971	11.689	9.64599	7.90422	11.2877	10.1092	9.70179	13.0003	14.5455	13.0109
XLOC_055760	ST5	chr15	44375167	44437971	9.55402	11.3998	10.48	26.6822	23.3806	23.9954	28.7699	28.583	33.9905
XLOC_056479	ST5	chr15	44373005	44373462	0.0	1.44243	3.7721	0.216118	0.559519	1.24104	0.638719	1.65945	0.209787

XLOC_056480	ST5	chr15	44374249	44374565	4.55163	0.0	0.0	0.814154	1.03852	1.38764	0.0	0.872834	1.17849
XLOC_064052	ST6GALNAC2;LOC100196901;LOC100196901;LOC100196901;CYGB;AANAT	chr19	55742295	55933829	11.6257	8.37867	11.2777	2.48298	2.76537	2.52827	5.20549	4.86363	2.6336
XLOC_065336	ST6GALNAC2;LOC100196901;LOC100196901;LOC100196901;CYGB;AANAT	chr19	55742295	55933829	0.440115	1.04774	2.19502	0.565435	0.576486	0.836087	0.383914	0.510299	0.490794
XLOC_065337	ST6GALNAC2;LOC100196901;LOC100196901;LOC100196901;CYGB;AANAT	chr19	55742295	55933829	0.774958	1.7391	1.81943	0.764422	0.638661	0.564554	0.389968	0.272902	0.542509
XLOC_065338	ST6GALNAC2;LOC100196901;LOC100196901;LOC100196901;CYGB;AANAT	chr19	55742295	55933829	0.0	0.331436	0.866816	0.397297	0.951466	0.114842	0.600412	0.552714	0.386969
XLOC_065339	ST6GALNAC2;LOC100196901;LOC100196901;LOC100196901;CYGB;AANAT	chr19	55742295	55933829	1.14768	1.71703	1.19756	0.171527	0.0900826	0.278702	0.210029	0.384934	0.234339
XLOC_065340	ST6GALNAC2;LOC100196901;LOC100196901;LOC100196901;CYGB;AANAT	chr19	55742295	55933829	1.55702	0.310552	2.4367	0.232673	0.284848	0.377785	0.284215	0.10427	0.544735

XLOC_065341	ST6GALNAC2;LOC100196901;LOC100196901;LOC100196901;CYGB;AANAT	chr19	55742295	55933829	0.0	0.405274	0.0	0.85015	1.16141	0.0	0.731019	0.134794	0.591084
XLOC_065342	ST6GALNAC2;LOC100196901;LOC100196901;LOC100196901;CYGB;AANAT	chr19	55742295	55933829	1.03283	0.618	1.61635	0.370417	0.36441	0.590705	0.141404	0.311257	0.316177
XLOC_065343	ST6GALNAC2;LOC100196901;LOC100196901;LOC100196901;CYGB;AANAT	chr19	55742295	55933829	2.50486	0.748862	1.9585	0.561039	0.488092	0.0	0.112767	0.124681	0.32775
XLOC_065344	ST6GALNAC2;LOC100196901;LOC100196901;LOC100196901;CYGB;AANAT	chr19	55742295	55933829	0.843736	0.252487	1.10063	0.050446	0.26512	0.0878668	0.335182	0.141673	0.221583
XLOC_065345	ST6GALNAC2;LOC100196901;LOC100196901;LOC100196901;CYGB;AANAT	chr19	55742295	55933829	0.0	0.15308	0.400373	0.13763	0.0802383	0.159624	0.0	0.051402	0.268521
XLOC_065346	ST6GALNAC2;LOC100196901;LOC100196901;LOC100196901;CYGB;AANAT	chr19	55742295	55933829	0.0	0.157421	1.64691	0.0471775	0.0825054	0.164139	0.0480176	0.0528511	0.414188

XLOC_065347	ST6GALNAC2;LOC100196901;LOC100196901;LOC100196901;CYGB;AANAT	chr19	55742295	55933829	2.24347	0.167793	1.75541	0.251428	0.307719	0.291535	0.613799	0.394176	0.29429
XLOC_065348	ST6GALNAC2;LOC100196901;LOC100196901;LOC100196901;CYGB;AANAT	chr19	55742295	55933829	4.7241	2.54297	2.95591	0.254027	0.590597	0.0979877	0.342216	0.0	0.577568
XLOC_065349	ST6GALNAC2;LOC100196901;LOC100196901;LOC100196901;CYGB;AANAT	chr19	55742295	55933829	0.77644	1.16117	2.42955	0.76557	0.3645	0.161218	0.423157	0.155495	0.339233
XLOC_065350	ST6GALNAC2;LOC100196901;LOC100196901;LOC100196901;CYGB;AANAT	chr19	55742295	55933829	0.0	0.974903	2.5497	0.194772	0.0	0.112613	0.294459	0.216824	0.18972
XLOC_065351	ST6GALNAC2;LOC100196901;LOC100196901;LOC100196901;CYGB;AANAT	chr19	55742295	55933829	0.637502	1.71634	0.498772	0.628668	0.649157	0.397451	0.174167	0.0639443	0.278671
XLOC_065352	ST6GALNAC2;LOC100196901;LOC100196901;LOC100196901;CYGB;AANAT	chr19	55742295	55933829	1.4113	0.868746	1.28761	0.147541	0.429369	0.583769	0.503387	0.165242	0.492234

XLOC_065353	ST6GALNAC2;LOC100196901;LOC100196901;LOC100196901;CYGB;AANAT	chr19	55742295	55933829	1.03993	0.310964	2.43984	0.0931896	0.24358	0.107787	0.094	0.41518	0.27235
XLOC_065354	ST6GALNAC2;LOC100196901;LOC100196901;LOC100196901;CYGB;AANAT	chr19	55742295	55933829	0.0	0.868385	0.567796	0.130121	0.0568104	0.301498	0.0659876	0.0	0.0
XLOC_065355	ST6GALNAC2;LOC100196901;LOC100196901;LOC100196901;CYGB;AANAT	chr19	55742295	55933829	0.965802	0.963345	1.00785	0.173225	0.227532	0.134071	0.294909	0.0972527	0.197246
XLOC_065356	ST6GALNAC2;LOC100196901;LOC100196901;LOC100196901;CYGB;AANAT	chr19	55742295	55933829	0.430624	1.03076	1.01096	0.115841	0.202722	0.26882	0.157469	0.0433039	0.0
XLOC_065357	ST6GALNAC2;LOC100196901;LOC100196901;LOC100196901;CYGB;AANAT	chr19	55742295	55933829	0.0	1.63034	3.9361	0.187923	0.296003	0.261672	0.306608	0.210777	0.22004
XLOC_065358	ST6GALNAC2;LOC100196901;LOC100196901;LOC100196901;CYGB;AANAT	chr19	55742295	55933829	0.346493	1.06642	1.19091	0.124312	0.159782	0.108324	0.18571	0.153346	0.177809

XLOC_065359	ST6GALNAC2;LOC100196901;LOC100196901;LOC100196901;CYGB;AANAT	chr19	55742295	55933829	1.03398	1.54594	3.23453	0.0926572	0.161466	0.321523	0.0934727	0.206418	0.45133
XLOC_065360	ST6GALNAC2;LOC100196901;LOC100196901;LOC100196901;CYGB;AANAT	chr19	55742295	55933829	1.30675	0.781294	2.04332	0.117067	0.203615	0.405655	0.235079	0.0	0.683791
XLOC_065361	ST6GALNAC2;LOC100196901;LOC100196901;LOC100196901;CYGB;AANAT	chr19	55742295	55933829	0.762523	0.912301	1.78953	0.205052	0.238671	0.0	0.27712	0.152734	0.133268
XLOC_065362	ST6GALNAC2;LOC100196901;LOC100196901;LOC100196901;CYGB;AANAT	chr19	55742295	55933829	0.955711	0.28594	0.747863	0.214234	0.149914	0.198812	0.0436468	0.0480256	0.0418016
XLOC_065363	ST6GALNAC2;LOC100196901;LOC100196901;LOC100196901;CYGB;AANAT	chr19	55742295	55933829	0.504526	0.452835	1.57916	0.180947	0.23737	0.419744	0.0460598	0.101379	0.0882604
XLOC_065364	ST6GALNAC2;LOC100196901;LOC100196901;LOC100196901;CYGB;AANAT	chr19	55742295	55933829	0.0	0.540676	0.942735	0.0	0.0472031	0.0626136	0.164699	0.0	0.263384
XLOC_045061	ST6GALNAC4	chr11	98581194	98587293	34.4992	46.9915	37.9684	8.08887	6.40423	6.88276	7.6321	9.69362	7.76819
XLOC_069861	ST7L	chr3	30855955	30897157	4.09301	1.36827	1.18851	5.29503	3.96684	4.35314	5.05137	5.39092	5.11573

XLOC_070760	ST7L	chr3	30898542	30900867	2.66934	1.7972	3.1337	1.01737	1.04801	1.49369	1.03901	1.07502	1.5478
XLOC_070761	ST7L	chr3	30901032	30903485	5.35604	3.48831	4.68511	1.97783	2.35059	2.26345	2.54013	1.93542	2.23376
XLOC_070762	ST7L	chr3	30903561	30906501	5.45827	4.27799	6.10314	2.26115	2.28721	2.38229	2.07387	1.59776	2.70785
XLOC_070247	ST7L;WNT2B	chr3	30906709	30969404	6.17236	9.28835	11.0194	3.88688	4.14487	4.45304	3.42338	3.74096	3.15426
XLOC_070763	ST7L;WNT2B	chr3	30906709	30969404	1.48367	2.21732	2.31958	0.930254	1.03887	0.613456	0.532101	0.736425	0.775876
XLOC_070764	ST7L;WNT2B	chr3	30906709	30969404	1.33133	0.99562	1.56239	0.358049	0.312787	0.553261	0.36354	0.266985	0.349144
XLOC_083152	ST8SIA4	chr7	102441509	102469099	56.9481	71.4596	49.5897	122.0	125.756	127.112	133.95	118.827	142.521
XLOC_085514	ST8SIA4	chr7	102469649	102469811	0.0	21.1763	0.0	30.0224	25.0116	17.4176	10.4702	6.0832	21.0797
XLOC_085515	ST8SIA4	chr7	102470160	102470438	0.0	1.77345	4.63671	5.32923	5.83623	5.41339	5.96179	2.25342	5.63343
XLOC_085516	ST8SIA4	chr7	102476495	102477972	1.64434	0.655919	0.42888	0.49143	1.50376	0.626844	0.899978	0.330235	0.671096
XLOC_085517	ST8SIA4	chr7	102478035	102478656	1.56051	0.932788	0.0	0.419293	1.9413	0.161223	0.838284	0.154767	1.08777
XLOC_085518	ST8SIA4	chr7	102479807	102481124	1.87126	0.373189	0.97605	0.391441	0.781822	0.518553	1.19319	0.312881	0.490818
XLOC_050799	ST8SIA6	chr13	32127906	32338681	3.75079	7.1019	7.82049	2.24028	3.11844	2.45897	3.03955	2.61384	2.61749
XLOC_050798	ST8SIA6;CUBN;VIM;TRDMT1	chr13	31819582	32127324	9.95229	13.707	11.5645	4.66449	5.699	4.24732	5.82025	6.79374	5.83501
XLOC_076359	STAB2	chr5	67607953	67612909	0.295494	0.442126	0.462551	0.0265005	0.0232101	0.0	0.0541646	0.0595299	0.0
XLOC_076866	STAB2	chr5	67618137	67847421	12.0401	12.3603	14.634	3.92097	4.02326	4.00861	2.64974	3.40966	3.45819
XLOC_078090	STAB2	chr5	67613513	67614138	0.0	0.0	0.0	0.0	0.0	0.0	0.138552	0.0	0.0
XLOC_078091	STAB2	chr5	67618137	67847421	5.42612	17.7929	0.0	2.42845	0.0	2.19816	0.456149	1.03216	1.86992
XLOC_092056	STAG2	chrX	7884043	7907027	23.984	24.681	18.0465	30.4815	25.0097	28.2526	29.4141	36.7925	28.1327
XLOC_092057	STAG2	chrX	7919845	7923471	0.0	0.476397	1.17665	4.18232	3.31146	3.47512	2.30912	4.07864	2.63921
XLOC_092363	STAG2	chrX	7884043	7907027	17.3093	16.6856	14.2672	15.641	15.5988	16.2576	17.614	20.2766	17.753
XLOC_092364	STAG2	chrX	7924246	7947819	37.4427	62.0333	51.8684	77.1386	62.0062	67.7585	64.8418	84.9454	76.3413
XLOC_092365	STAG2	chrX	7959710	7967865	1.75033	2.10311	5.60221	1.03873	0.551692	0.903226	0.108951	1.0165	0.576553
XLOC_092366	STAG2	chrX	8003884	8015534	0.0	0.0	0.0	3.81029	2.42958	3.44435	3.25848	1.80195	3.80185
XLOC_092706	STAG2	chrX	7959148	7959402	0.0	0.0	0.0	0.659922	0.0	0.740073	0.0	0.0	0.0
XLOC_080187	STAP1;CENPC1;UBA6	chr6	84617969	85069529	14.1364	17.7522	17.8505	13.087	13.9323	15.774	16.4768	14.7608	14.0694
XLOC_055764	STARD10;ATG16L2	chr15	53063307	53345988	28.687	23.0139	24.498	17.4496	16.1497	15.6392	16.1435	15.3953	16.0397
XLOC_056572	STARD10;ATG16L2	chr15	53063307	53345988	8.72805	7.82089	15.3397	2.3436	2.53219	2.91938	0.386323	1.28868	1.51764

XLOC_056573	STARD10;ATG16L2	chr15	53063307	53345988	9.2916	0.0	14.504	0.838916	1.38417	1.86607	0.751428	1.72197	1.59631
XLOC_056574	STARD10;ATG16L2	chr15	53063307	53345988	0.0	0.0	31.6044	2.45849	2.9746	4.039	0.0	3.67408	0.0
XLOC_056575	STARD10;ATG16L2	chr15	53063307	53345988	14.8506	7.9787	20.8642	3.98503	3.19746	2.43406	2.59289	3.18618	3.34773
XLOC_056576	STARD10;ATG16L2	chr15	53063307	53345988	9.18042	7.84124	12.3044	1.99735	2.45214	1.62846	0.825687	1.30465	1.14382
XLOC_056577	STARD10;ATG16L2	chr15	53063307	53345988	15.3694	10.3316	18.0129	2.92401	2.97884	1.98041	0.854384	1.89703	1.33749
XLOC_056578	STARD10;ATG16L2	chr15	53063307	53345988	0.0	5.17273	8.11583	4.96119	1.06261	1.77113	0.0	1.68127	0.600434
XLOC_056579	STARD10;ATG16L2	chr15	53063307	53345988	23.4823	14.0501	9.18225	4.27065	2.60636	2.35262	0.9347	4.30343	4.0335
XLOC_056580	STARD10;ATG16L2	chr15	53063307	53345988	8.91633	7.98925	10.4466	2.59355	1.55151	2.29346	1.97161	1.53502	1.74387
XLOC_056581	STARD10;ATG16L2	chr15	53063307	53345988	6.70023	6.90306	12.2304	1.60161	2.33055	1.93263	1.35325	1.04406	1.49643
XLOC_056582	STARD10;ATG16L2	chr15	53063307	53345988	10.5228	0.0	0.0	0.95344	1.56205	1.05525	1.68756	0.969237	0.0
XLOC_056583	STARD10;ATG16L2	chr15	53063307	53345988	1.22699	7.33634	12.4715	0.879411	0.860856	1.1432	1.32627	0.244361	0.963375
XLOC_056584	STARD10;ATG16L2	chr15	53063307	53345988	22.5111	6.73161	17.5966	3.06614	0.0	1.12806	0.898521	2.06686	0.0
XLOC_056585	STARD10;ATG16L2	chr15	53063307	53345988	6.48736	4.52135	5.06757	1.35486	1.33859	2.2258	0.957483	1.27742	1.12812
XLOC_056586	STARD10;ATG16L2	chr15	53063307	53345988	5.50557	6.99441	9.6843	1.60285	1.28607	1.99307	0.989157	0.684061	1.56016
XLOC_056587	STARD10;ATG16L2	chr15	53063307	53345988	9.83706	10.2642	11.5021	3.51997	3.73232	0.499058	1.66535	0.939861	1.27263
XLOC_056588	STARD10;ATG16L2	chr15	53063307	53345988	19.3938	19.2726	25.194	2.89818	2.91985	1.95793	3.21964	1.82916	1.11255
XLOC_056589	STARD10;ATG16L2	chr15	53063307	53345988	9.59644	7.88087	5.62088	1.50281	2.03822	2.71245	2.3271	1.17768	2.29258
XLOC_056590	STARD10;ATG16L2	chr15	53063307	53345988	3.51799	17.8424	27.4445	2.83121	4.58011	3.5928	4.25974	2.72764	2.43585
XLOC_056591	STARD10;ATG16L2	chr15	53063307	53345988	13.0234	5.8421	10.9138	2.33436	1.52657	1.54374	0.842561	1.20864	1.46237
XLOC_056592	STARD10;ATG16L2	chr15	53063307	53345988	3.69711	4.41139	34.6054	4.29757	0.848325	2.64054	2.55055	0.357614	0.959532
XLOC_056593	STARD10;ATG16L2	chr15	53063307	53345988	7.17357	10.7112	9.33713	1.49786	1.84691	2.45783	1.68708	0.939121	0.623274
XLOC_056594	STARD10;ATG16L2	chr15	53063307	53345988	6.40811	5.05402	7.74881	1.51463	2.00867	1.69549	1.59318	1.46181	1.68107

XLOC_056595	STARD10;ATG16L2	chr15	53063307	53345988	4.84649	5.789	3.78487	2.60208	2.05831	1.24514	3.41742	1.18917	2.31542
XLOC_056596	STARD10;ATG16L2	chr15	53063307	53345988	8.08157	6.43398	10.5158	1.68702	2.49098	2.76396	2.36307	0.790662	3.5067
XLOC_056597	STARD10;ATG16L2	chr15	53063307	53345988	12.9293	25.0544	0.0	4.05746	0.486642	5.22114	5.36607	0.0	2.78098
XLOC_056598	STARD10;ATG16L2	chr15	53063307	53345988	12.1909	10.9023	19.0032	5.46154	3.67715	2.464	4.06502	2.30611	2.62331
XLOC_056599	STARD10;ATG16L2	chr15	53063307	53345988	29.2489	14.5471	10.144	4.65036	3.99011	3.32395	3.95298	2.21149	3.94123
XLOC_056600	STARD10;ATG16L2	chr15	53063307	53345988	5.02507	6.2271	5.61606	1.86618	1.85439	1.64021	2.93745	1.36675	2.00777
XLOC_056601	STARD10;ATG16L2	chr15	53063307	53345988	5.34772	6.37639	16.6721	4.78601	1.61921	2.70862	7.20007	2.54474	1.84329
XLOC_056602	STARD10;ATG16L2	chr15	53063307	53345988	13.6551	4.07144	3.54891	4.47785	3.11555	2.31273	3.0987	2.6185	1.17868
XLOC_056603	STARD10;ATG16L2	chr15	53063307	53345988	7.06536	4.22239	8.28196	2.37245	2.05661	1.64029	3.15095	1.22319	1.53796
XLOC_056604	STARD10;ATG16L2	chr15	53063307	53345988	0.0	2.06989	0.0	6.23062	3.12892	5.60006	4.58602	7.17735	3.58184
XLOC_056605	STARD10;ATG16L2	chr15	53063307	53345988	15.8344	5.29251	16.0275	4.75821	3.2754	4.05752	3.45887	2.51345	4.31131
XLOC_056606	STARD10;ATG16L2	chr15	53063307	53345988	11.9295	9.7918	9.31117	8.26958	6.87929	7.02625	4.42513	5.52529	4.13705
XLOC_056607	STARD10;ATG16L2	chr15	53063307	53345988	12.116	5.789	15.139	4.98732	3.36814	4.98056	2.77665	3.56752	4.84123
XLOC_056608	STARD10;ATG16L2	chr15	53063307	53345988	7.56616	7.38278	10.041	4.98567	4.44308	4.4865	5.24277	4.30592	4.00218
XLOC_056609	STARD10;ATG16L2	chr15	53063307	53345988	10.1755	9.12228	11.9285	7.28973	6.7175	6.12728	7.87599	4.19797	4.43095
XLOC_056610	STARD10;ATG16L2	chr15	53063307	53345988	4.5237	18.8827	31.7404	14.1593	11.6991	11.0338	10.7829	7.37522	8.19914
XLOC_056611	STARD10;ATG16L2	chr15	53063307	53345988	0.0	2.90066	4.55159	3.82457	3.01034	2.20157	4.14353	1.72523	3.04134
XLOC_056612	STARD10;ATG16L2	chr15	53063307	53345988	9.75198	9.71985	11.8631	5.04892	5.49704	6.1753	5.96974	3.35083	5.39092
XLOC_056613	STARD10;ATG16L2	chr15	53063307	53345988	35.5281	13.2422	17.3129	13.1063	10.1398	12.6426	6.81383	11.9357	11.5017
XLOC_056614	STARD10;ATG16L2	chr15	53063307	53345988	9.79365	15.5866	28.0209	10.8023	10.0191	10.6837	5.67114	7.61513	7.91745
XLOC_055451	STIM1	chr15	51877337	51882810	8.12533	3.3319	9.15737	13.1469	11.92	13.3831	13.0736	14.0214	11.2411
XLOC_055452	STIM1	chr15	52039846	52045758	140.608	211.216	180.461	9.24842	4.23539	5.1109	9.10865	8.21398	6.29881
XLOC_055763	STIM1	chr15	51961190	51993704	48.2201	53.4488	50.9611	56.4092	54.9794	56.7283	73.8681	72.9727	67.0927

XLOC_056559	STIM1	chr15	51883724	51884773	0.811275	0.727941	1.26923	0.727175	1.33262	1.51574	1.39922	1.21802	1.41764
XLOC_056560	STIM1	chr15	51957529	51958295	0.0	0.0	0.929422	0.851984	1.76115	1.35394	0.428587	0.118421	0.725955
XLOC_056561	STIM1	chr15	51958562	51959037	4.55639	0.680418	1.77938	0.0	1.23286	0.468664	1.20798	0.223991	2.37589
XLOC_056562	STIM1	chr15	51959503	51959799	5.19658	1.54916	0.0	2.79008	5.11826	1.05345	6.57293	0.9905	3.5831
XLOC_056563	STIM1	chr15	51959862	51960292	0.0	0.792477	0.0	0.712426	0.613796	0.544809	1.86402	0.779424	1.15187
XLOC_056564	STIM1	chr15	51961190	51993704	13.827	9.61952	32.3389	11.9547	13.3156	10.7721	21.162	11.9251	19.8864
XLOC_056565	STIM1	chr15	51961190	51993704	22.2522	0.0	34.6989	17.9951	20.5769	20.1772	20.7259	12.509	19.7648
XLOC_056566	STIM1	chr15	51961190	51993704	22.826	13.6538	26.7673	8.29544	5.07126	4.58006	6.37198	10.4722	9.80073
XLOC_056567	STIM1	chr15	51961190	51993704	6.13899	4.59052	3.60182	5.36532	5.82441	4.86038	6.76233	5.76364	6.16924
XLOC_056568	STIM1	chr15	51961190	51993704	6.06454	5.4302	2.36661	2.98357	1.39839	1.86449	2.90876	1.47752	2.10275
XLOC_056569	STIM1	chr15	52003058	52003763	0.0	0.394923	0.0	0.473393	0.411645	0.68344	0.593981	0.262806	1.15208
XLOC_056570	STIM1	chr15	52009970	52010778	0.0	0.0	0.0	0.199259	0.954365	0.806347	0.70259	0.221756	0.388154
XLOC_049699	STK24	chr12	79431026	79432223	0.0	0.208122	0.0	1.06031	1.25289	1.73448	1.01269	0.976339	1.39875
XLOC_049700	STK24	chr12	79432827	79436034	0.23716	0.212919	0.74252	1.40384	1.47231	0.889369	1.10974	0.454218	1.16281
XLOC_049701	STK24	chr12	79436166	79437284	1.50598	0.225228	0.589062	0.472482	0.94283	0.625491	0.684265	0.603373	0.52643
XLOC_049702	STK24	chr12	79437401	79437914	0.0	0.0	1.58956	1.09285	2.04879	0.838379	0.5418	0.200635	1.06188
XLOC_049703	STK24	chr12	79438126	79438726	0.0	1.46549	1.27753	1.02471	2.66731	1.18159	1.02303	0.485902	1.99341
XLOC_049704	STK24	chr12	79439046	79439281	0.0	2.6782	0.0	4.85635	6.68988	9.91517	2.90959	2.49851	10.0181
XLOC_049705	STK24	chr12	79439483	79440669	1.40641	0.210346	0.0	1.00861	1.65158	1.75295	1.91884	0.493345	1.04489
XLOC_049706	STK24	chr12	79440923	79442367	1.68652	0.336368	0.439875	1.00806	2.02684	1.69483	1.64057	1.52392	2.06483
XLOC_049707	STK24	chr12	79443844	79444413	0.0	1.57571	1.37361	0.944375	1.3645	2.35791	2.3521	1.39128	1.83655
XLOC_049708	STK24	chr12	79446104	79446694	0.0	0.999546	2.61404	1.04836	1.0393	2.76258	2.09194	0.496909	1.89357
XLOC_049709	STK24	chr12	79447528	79448745	0.0	0.204195	0.0	0.856725	1.22937	0.850936	0.621134	0.615897	1.07406
XLOC_049710	STK24	chr12	79449493	79450966	0.0	0.82239	0.430183	1.13372	2.24093	1.25749	1.50448	0.60726	2.0194
XLOC_049711	STK24	chr12	79452100	79452461	0.0	1.0598	0.0	0.63531	1.35994	1.08819	1.84242	0.0	2.15187
XLOC_049712	STK24	chr12	79452782	79453952	0.714317	0.427336	0.55883	1.60083	2.01304	1.40958	0.779504	1.28847	2.06028
XLOC_049713	STK24	chr12	79454244	79456337	0.0	0.447234	0.0	1.10577	1.1146	1.05006	0.478753	0.639244	1.20989
XLOC_049714	STK24	chr12	79458793	79459753	0.901255	0.269536	0.0	0.646201	0.563563	1.02844	0.490049	0.180215	1.49564
XLOC_049715	STK24	chr12	79465424	79466929	0.536724	0.481722	0.419973	0.962447	1.80926	1.50675	0.783523	0.592934	1.64294
XLOC_049716	STK24	chr12	79468059	79468693	0.0	0.0	2.37302	1.35957	0.944486	0.941195	0.544031	0.451868	0.661408
XLOC_049717	STK24	chr12	79469093	79469597	0.0	0.0	1.63076	0.934311	0.6465	1.28982	0.740458	0.41143	0.907714
XLOC_049718	STK24	chr12	79471702	79472619	0.0	0.284783	0.0	1.19482	0.744034	0.790058	1.12083	0.5709	1.16423
XLOC_049719	STK24	chr12	79474352	79475074	0.0	0.383044	1.00178	0.803521	0.998436	0.928228	0.922421	0.382522	1.34105

XLOC_049720	STK24	chr12	79475390	79478193	0.0	0.245426	0.855881	1.00522	0.944976	0.968016	0.827168	0.523349	1.24448
XLOC_049721	STK24	chr12	79479026	79479516	0.0	0.0	0.0	0.973568	1.68313	0.671703	0.385207	0.642451	2.45866
XLOC_049722	STK24	chr12	79480652	79482526	0.42134	0.0	0.0	0.377816	0.89265	0.482241	0.42378	0.381378	0.847904
XLOC_053386	STK3	chr14	67715866	67744138	12.5567	10.9201	8.93713	4.63038	6.59875	5.99258	6.2092	4.98078	6.49344
XLOC_053387	STK3	chr14	67745775	67748527	2.54447	5.48943	6.83827	0.774554	1.15848	1.42156	1.30578	0.301505	1.6664
XLOC_053684	STK3	chr14	67675336	67715775	15.299	18.7124	15.105	50.4308	74.8899	55.0144	59.6742	53.259	85.5176
XLOC_053685	STK3	chr14	67751713	67776885	13.6233	20.9449	12.6293	27.3914	23.2888	28.7949	25.1901	29.4165	24.1851
XLOC_053686	STK3	chr14	67777570	67933463	18.3361	18.9342	18.2452	29.5845	27.0129	27.5468	28.6979	32.2266	30.8703
XLOC_054748	STK3	chr14	67715866	67744138	2.62514	6.36667	2.05447	1.4727	1.35283	0.819669	0.857997	1.12878	1.05019
XLOC_054749	STK3	chr14	67715866	67744138	6.52077	1.66489	9.40962	0.452037	0.592481	0.45846	1.01531	0.505805	1.04699
XLOC_054750	STK3	chr14	67715866	67744138	5.42117	4.71839	6.22504	1.06054	1.31407	1.74271	1.7551	1.78279	1.89682
XLOC_054751	STK3	chr14	67744806	67745567	3.5954	3.22485	4.68557	1.28855	1.77561	0.868684	1.29618	0.358162	1.25476
XLOC_054752	STK3	chr14	67748728	67749533	1.11702	3.00593	3.49401	0.700632	0.697271	0.694335	0.604953	0.22277	1.46229
XLOC_054753	STK3	chr14	67749968	67750941	0.886887	1.32621	2.08113	0.397443	1.10928	0.276038	0.96473	0.354747	1.162
XLOC_054754	STK3	chr14	67751141	67751502	0.0	1.0598	0.0	0.63531	1.08795	0.362731	0.61414	0.344162	2.45928
XLOC_055450	STK33	chr15	44619196	44641288	8.81662	8.99885	6.48877	6.46241	7.91955	6.73914	7.85685	6.9283	6.57741
XLOC_065542	STK39	chr2	27886418	28039205	21.5874	16.8687	22.9169	3.16025	1.60237	2.36702	3.47761	2.79053	3.07205
XLOC_050634	STK4	chr13	74155107	74167755	0.0	0.235375	0.0	0.0470271	0.0823952	0.0	0.0240439	0.02642	0.0
XLOC_052495	STK4	chr13	74131185	74132391	0.68979	0.619009	0.0	0.0	0.0	0.0	0.0	0.0	0.0
XLOC_052496	STK4	chr13	74167825	74168992	0.71644	0.642907	1.12098	0.19267	0.168249	0.0	0.130298	0.0	0.125236
XLOC_052497	STK4	chr13	74169438	74170482	0.815851	0.488029	2.55278	1.38942	1.14865	1.01616	1.18484	1.46978	1.21177
XLOC_050955	STK4;KCNS1	chr13	74170810	74213189	19.577	25.4588	19.7409	17.0007	17.5608	17.5128	20.4656	22.8652	19.3507
XLOC_066538	STMN1	chr2	127770348	127788895	6.45209	8.10126	10.0892	7.51445	9.45159	6.41005	8.12742	10.0154	11.0297
XLOC_053277	STMN2	chr14	44988626	45002636	0.378554	0.0	0.296258	0.10184	0.148571	0.0787975	0.0692819	0.114277	0.0
XLOC_050858	STMN3	chr13	54552160	54565632	38.4734	48.5647	38.3305	41.2881	30.3221	36.3708	43.0926	52.1887	30.8717
XLOC_051867	STMN3	chr13	54551819	54552016	338.376	295.502	193.102	214.878	164.954	212.356	213.182	226.907	140.143
XLOC_048104	STOML3	chr12	23253652	23315358	0.451596	0.0	0.353394	1.13382	0.531399	0.798649	0.165075	0.317809	0.4741
XLOC_048905	STOML3	chr12	23253652	23315358	0.0	0.0	0.0	0.260724	0.364627	0.423193	0.31813	0.116758	0.406872
XLOC_048906	STOML3	chr12	23253652	23315358	1.39227	0.0	0.0	1.24719	0.758777	1.8719	1.12533	0.691829	1.45664
XLOC_048907	STOML3	chr12	23253652	23315358	0.0	0.0	0.0	0.777299	1.3552	0.999346	0.348934	1.05905	0.925684
XLOC_048908	STOML3	chr12	23253652	23315358	3.15096	0.940366	0.0	0.563633	1.93576	0.322456	0.274224	0.919967	2.18421
XLOC_048909	STOML3	chr12	23253652	23315358	0.0	4.16403	0.0	7.6272	5.11579	0.0	2.18858	2.52598	0.0
XLOC_048910	STOML3	chr12	23253652	23315358	0.0	0.0	0.0	0.53743	0.516664	0.436124	0.382407	0.240625	0.524154

XLOC_043324	STON2	chr10	93746401	93747082	5.52653	18.1723	17.282	0.247534	0.0	0.142901	0.0	0.137326	0.120464
XLOC_043325	STON2	chr10	93778609	93779777	32.9236	59.3031	45.9147	65.8923	66.9532	69.949	67.8191	61.1075	61.993
XLOC_043326	STON2	chr10	93802156	93802372	0.0	0.0	0.0	4.14742	0.0	2.28747	1.82057	5.23612	2.94022
XLOC_041723	STON2;GTF2A1	chr10	93641429	93746339	12.6835	13.0461	22.3392	0.133929	0.0584974	0.0790317	0.0679329	0.0325185	0.0998759
XLOC_044372	STRBP	chr11	94328244	94342021	41.9555	45.6618	36.5014	29.5805	27.2743	31.6265	32.8826	42.4965	26.3146
XLOC_045002	STRBP	chr11	94288406	94297013	118.294	111.131	104.496	78.7172	72.7875	78.6459	114.268	109.556	101.688
XLOC_045003	STRBP	chr11	94297117	94322019	85.2738	96.6904	78.2532	80.4778	69.5132	75.4683	102.877	107.623	101.119
XLOC_045004	STRBP	chr11	94411076	94416602	1.03545	0.309629	0.0	0.278369	0.0	0.107327	0.0	0.0	0.0903948
XLOC_045005	STRBP	chr11	94416657	94425787	0.0	0.497476	0.0	0.0	0.0	0.171875	0.0	0.164889	0.0
XLOC_047022	STRBP	chr11	94281008	94281517	0.0	1.84418	4.82283	1.10526	2.23109	1.2717	0.0	0.608584	1.25286
XLOC_047023	STRBP	chr11	94281624	94282913	1.91757	0.573628	2.00038	1.94831	2.30308	2.05892	1.4552	0.5129	1.95586
XLOC_047024	STRBP	chr11	94283007	94283844	1.06441	0.318279	0.0	1.0492	1.41251	1.10308	1.53872	0.106207	1.57952
XLOC_047025	STRBP	chr11	94284405	94285143	1.24596	1.11749	3.89679	1.11629	1.5539	2.32159	1.79515	0.62023	1.73901
XLOC_047026	STRBP	chr11	94285691	94288108	2.56065	2.39451	4.75973	3.15753	3.59449	2.8993	2.28714	1.77275	2.66153
XLOC_047027	STRBP	chr11	94408857	94409458	0.0	0.974791	2.54931	0.146057	0.126732	0.0	0.0	0.0	0.142067
XLOC_045552	STRN	chr11	19416212	19417390	0.0	0.0	0.0	0.0635317	0.0554819	0.0	0.0	0.355128	0.0619446
XLOC_093744	STS	chrX	146709274	146709390	0.0	355.743	0.0	243.69	151.83	112.575	158.654	136.258	198.518
XLOC_065858	STX12	chr2	126152015	126165409	6.11267	4.49006	3.96224	9.93808	9.09241	8.88079	9.58927	8.08636	11.2978
XLOC_055586	STX3	chr15	84297962	84315846	2.36076	0.918428	1.66303	5.3775	5.02723	5.78304	8.07906	9.54209	4.46343
XLOC_057328	STX3	chr15	84297962	84315846	0.0	0.0	0.0	0.712883	0.59701	0.899482	0.696242	1.11338	0.39324
XLOC_057329	STX3	chr15	84297962	84315846	0.0	0.0	0.528079	0.726117	0.581409	0.350602	0.675692	1.21811	0.53103
XLOC_063958	STX8	chr19	29367256	29386479	13.485	17.0974	17.5479	0.338218	0.204053	0.650746	0.0	0.087171	0.274995
XLOC_064220	STX8	chr19	29321366	29325308	1.50843	0.0	0.0	1.08086	1.75996	1.24715	0.811081	0.299395	1.05167
XLOC_064221	STX8	chr19	29348597	29362317	11.6228	5.93777	6.5541	4.76903	6.71037	5.02485	6.41233	8.77247	8.64952
XLOC_064959	STX8	chr19	29311550	29313166	7.934	14.8359	12.0288	1.15601	0.93327	0.618857	1.26777	1.64425	0.954252
XLOC_064960	STX8	chr19	29367256	29386479	2.53116	3.40522	9.89529	0.113384	0.0986336	0.0	0.0	0.0	0.0
XLOC_064961	STX8	chr19	29386809	29386980	107.36	89.6113	21.3039	0.0	1.94675	0.0	0.0	0.0	0.0
XLOC_064962	STX8	chr19	29387147	29387775	7.68644	7.8109	16.8226	0.413064	0.119548	0.0	0.0	0.0	0.401878
XLOC_064963	STX8	chr19	29387829	29388048	87.6102	68.7553	51.3487	0.993559	0.0	2.19572	0.0	1.00683	0.0
XLOC_064964	STX8	chr19	29486779	29487547	1.18472	0.354213	0.0	0.0	0.0	0.0	0.0	0.0	0.0
XLOC_044423	STXBP1	chr11	98356873	98357773	0.0	0.986754	0.0	0.591418	1.20183	1.02577	0.794584	0.548567	0.960094
XLOC_044424	STXBP1	chr11	98358374	98381111	15.729	15.0103	27.5411	8.53034	8.22821	8.51869	6.7306	8.3722	6.30749

XLOC_045059	STXBP1	chr11	98388677	98400487	2.56839	2.78662	2.53281	1.93188	1.9051	1.22165	1.20573	1.26908	1.74069
XLOC_070813	STXBP3	chr3	34699403	34699986	0.0	0.0	0.0	2.28338	0.396063	1.05283	0.303613	1.34644	0.592164
XLOC_063846	STXBP4	chr19	5364677	5371542	21.9946	22.0821	16.8114	22.33	19.2411	21.6181	24.2331	29.7607	20.8213
XLOC_063847	STXBP4	chr19	5425236	5427605	19.7266	23.3002	15.8662	12.7934	11.6346	11.6411	12.6253	13.6164	10.5153
XLOC_063848	STXBP4	chr19	5447602	5455970	61.7998	54.8167	60.6369	0.8878	0.499826	0.699282	0.550289	0.238382	0.526107
XLOC_063849	STXBP4	chr19	5460602	5469026	5.69223	19.6098	17.8476	16.2919	14.4042	12.3944	13.303	13.5411	14.3446
XLOC_063850	STXBP4	chr19	5474044	5481994	16.2996	15.1126	9.75442	2.47366	1.76729	2.19414	2.25093	2.31195	2.04566
XLOC_063851	STXBP4	chr19	5489988	5494621	220.118	223.346	199.245	406.946	316.271	392.417	390.531	551.075	355.519
XLOC_064105	STXBP4	chr19	5364677	5371542	64.517	75.4643	61.5591	239.773	247.754	245.087	226.467	228.047	199.886
XLOC_064106	STXBP4	chr19	5380056	5382736	57.1914	69.6265	70.9847	65.0344	43.62	55.639	62.548	101.512	45.6662
XLOC_064107	STXBP4	chr19	5382804	5388407	54.7667	60.5209	48.3937	61.8056	39.7169	50.746	61.7197	100.165	42.8098
XLOC_064108	STXBP4	chr19	5389686	5400970	0.0	0.265593	0.0	1.55269	1.42849	1.61709	1.82655	1.02701	1.16563
XLOC_064109	STXBP4	chr19	5422698	5424732	73.6203	72.8075	82.8883	80.8955	82.4747	94.2811	80.0719	100.852	67.8373
XLOC_064110	STXBP4	chr19	5456080	5458098	8.05709	6.21717	9.29188	3.19411	2.02969	2.6032	2.4814	2.85736	1.85514
XLOC_064111	STXBP4	chr19	5460602	5469026	0.269207	0.780734	0.228951	0.912681	0.552563	0.720739	0.90194	0.890864	1.22762
XLOC_064112	STXBP4	chr19	5482765	5489242	5.05694	6.74121	6.82677	5.26387	4.59167	4.6781	5.5728	6.46068	3.79837
XLOC_064113	STXBP4	chr19	5489988	5494621	176.869	198.609	226.305	286.579	221.477	267.659	273.736	360.458	265.951
XLOC_064114	STXBP4	chr19	5495219	5510661	1.52539	1.94834	0.743311	2.58503	2.94367	2.47528	2.32972	2.10405	2.45361
XLOC_064424	STXBP4	chr19	5325081	5325245	32.6258	9.95109	26.0247	31.2795	16.4987	22.9378	17.2837	11.4752	33.9789
XLOC_064425	STXBP4	chr19	5361072	5361933	0.0	0.307431	0.0	1.01344	0.883054	0.95912	1.20837	0.821035	0.897547
XLOC_064426	STXBP4	chr19	5377749	5378869	0.751427	0.89904	0.587838	0.336787	0.940883	0.702222	1.09258	0.752663	1.11634
XLOC_064427	STXBP4	chr19	5379023	5380006	1.75229	1.57219	0.685313	1.09937	1.09594	0.45452	0.79436	0.525744	0.612251
XLOC_064428	STXBP4	chr19	5388501	5389145	13.3805	14.22	9.29725	10.2538	7.7494	8.29822	7.86399	13.5776	7.64505
XLOC_064429	STXBP4	chr19	5424815	5425019	0.0	12.2884	53.5343	12.4991	20.1425	13.6798	18.3214	29.8457	16.438
XLOC_064430	STXBP4	chr19	5435936	5436802	18.3751	25.6421	19.9593	63.7625	50.0615	53.1224	81.323	88.2576	65.5058
XLOC_064431	STXBP4	chr19	5482144	5482552	0.0	3.44736	2.25369	0.774805	0.444311	0.0	0.0	0.563678	0.500781
XLOC_064432	STXBP4	chr19	5494705	5494840	108.331	68.4384	89.6936	56.2309	46.1507	11.0765	31.9524	55.5261	28.9107
XLOC_090013	STXBP5	chr9	85687266	85750011	12.6106	12.6194	11.1941	17.9027	15.1314	17.1458	20.168	18.7827	16.2605
XLOC_091390	STXBP5	chr9	85686489	85687181	1.3532	0.404517	1.05793	0.606115	0.0	0.27997	0.608071	0.403639	0.35399
XLOC_037890	STXBP5L	chr1	66175311	66433595	25.1754	22.5973	24.8522	0.965665	1.52669	1.36185	1.73376	1.18393	1.30014
XLOC_037891	STXBP5L	chr1	66438082	66442633	10.3876	7.85379	10.8414	0.572092	0.759351	0.930519	0.669514	0.790451	0.622404
XLOC_037892	STXBP5L	chr1	66468371	66510781	0.0	0.218894	0.0	0.0656217	0.0	0.0761338	0.0	0.0	0.0563777

XLOC_047796	SUCLA2;MED4;ITM2B	chr12	17690819	18156734	17.7623	10.9516	6.38826	9.84967	11.7827	10.2213	8.2928	6.21604	12.0053
XLOC_047797	SUCLA2;MED4;ITM2B	chr12	17690819	18156734	7.50648	6.73775	5.50694	4.45896	6.88151	4.97774	3.81447	2.82931	7.10027
XLOC_047798	SUCLA2;MED4;ITM2B	chr12	17690819	18156734	3.23832	1.96896	1.58492	7.6362	6.67697	6.0611	5.11043	4.29921	6.48104
XLOC_048097	SUCLA2;MED4;ITM2B	chr12	17690819	18156734	0.0	0.0	6.27137	5.78906	1.20297	4.85431	1.86383	1.50086	4.71638
XLOC_048529	SUCLA2;MED4;ITM2B	chr12	17690819	18156734	2.79532	1.10648	1.07391	0.609133	0.566552	1.09256	0.926348	0.183585	0.483603
XLOC_048530	SUCLA2;MED4;ITM2B	chr12	17690819	18156734	0.80406	0.480981	1.88693	0.57657	0.314487	0.918087	0.72998	0.241458	0.42152
XLOC_048531	SUCLA2;MED4;ITM2B	chr12	17690819	18156734	0.618419	1.85	3.38698	0.277211	0.290689	0.835471	0.563382	0.0620451	0.59477
XLOC_048532	SUCLA2;MED4;ITM2B	chr12	17690819	18156734	0.581148	0.695427	0.0	0.36472	0.728671	0.483262	0.158939	0.349995	0.304909
XLOC_048533	SUCLA2;MED4;ITM2B	chr12	17690819	18156734	1.77499	1.06077	0.0	0.953629	1.65325	0.366278	1.89955	0.702322	2.009
XLOC_048534	SUCLA2;MED4;ITM2B	chr12	17690819	18156734	0.0	0.0	0.0	3.30198	4.39101	4.27298	4.44022	3.51405	7.72203
XLOC_048535	SUCLA2;MED4;ITM2B	chr12	17690819	18156734	0.956856	1.24075	0.748886	1.6304	1.45277	1.29511	1.31487	1.05991	1.22836
XLOC_048536	SUCLA2;MED4;ITM2B	chr12	17690819	18156734	0.0	0.11745	1.53594	0.774375	1.04745	0.61274	0.646382	0.987286	1.37392
XLOC_048537	SUCLA2;MED4;ITM2B	chr12	17690819	18156734	0.0	0.286668	1.49948	0.343636	1.0485	0.596447	1.04135	0.478866	1.17192
XLOC_048538	SUCLA2;MED4;ITM2B	chr12	17690819	18156734	0.0	0.748862	0.979252	0.673247	1.36666	1.03713	0.451066	0.374042	0.98325
XLOC_048539	SUCLA2;MED4;ITM2B	chr12	17690819	18156734	1.20445	0.72021	0.941789	0.539576	0.563492	1.49656	0.542714	0.119975	1.47117
XLOC_048540	SUCLA2;MED4;ITM2B	chr12	17690819	18156734	0.589197	0.52879	0.461005	0.316945	0.969564	0.734896	0.966694	0.709615	1.18497
XLOC_048541	SUCLA2;MED4;ITM2B	chr12	17690819	18156734	5.59984	1.11543	1.45853	1.67127	3.47463	1.73235	1.99428	1.84439	2.59939
XLOC_048542	SUCLA2;MED4;ITM2B	chr12	17690819	18156734	0.613696	1.83588	0.0	0.770268	0.625039	0.255112	0.726866	0.554174	1.71706
XLOC_048543	SUCLA2;MED4;ITM2B	chr12	17690819	18156734	0.605475	0.724521	0.0	0.868522	0.474391	0.503415	0.275861	0.303781	0.63529
XLOC_048544	SUCLA2;MED4;ITM2B	chr12	17690819	18156734	0.0	0.200786	0.0	0.722076	0.420501	0.488121	0.427621	0.336496	0.704107
XLOC_048545	SUCLA2;MED4;ITM2B	chr12	17690819	18156734	0.0	0.0	1.1457	0.0	0.114041	1.3636	0.394343	0.145526	0.0
XLOC_048546	SUCLA2;MED4;ITM2B	chr12	17690819	18156734	0.0	0.434562	0.0	1.43249	0.791944	1.35273	0.260838	0.288753	0.760361

XLOC_048547	SUCLA2;MED4;ITM2B	chr12	17690819	18156734	0.0	2.16963	0.0	1.96048	0.0	1.46619	0.598754	0.0	1.25087
XLOC_048548	SUCLA2;MED4;ITM2B	chr12	17690819	18156734	0.479756	0.430616	0.750838	0.473189	0.451526	0.748502	1.09548	0.530372	0.71345
XLOC_048549	SUCLA2;MED4;ITM2B	chr12	17690819	18156734	0.0	0.0	0.0	0.22158	0.242035	0.449486	0.619196	0.0619925	0.216096
XLOC_048550	SUCLA2;MED4;ITM2B	chr12	17690819	18156734	0.0	0.0	0.0	1.63835	1.77829	2.52033	3.41413	1.05872	3.45374
XLOC_048551	SUCLA2;MED4;ITM2B	chr12	17690819	18156734	0.0	0.0	0.595254	0.613864	0.952689	0.474031	0.483954	0.0762077	1.19689
XLOC_048552	SUCLA2;MED4;ITM2B	chr12	17690819	18156734	3.00709	0.0	0.0	4.30355	3.23639	2.15588	2.09881	2.05195	3.1282
XLOC_048553	SUCLA2;MED4;ITM2B	chr12	17690819	18156734	0.0	0.271224	0.0	0.73153	0.212651	0.0940778	0.575249	0.362666	0.237629
XLOC_048554	SUCLA2;MED4;ITM2B	chr12	17690819	18156734	0.419628	0.125556	0.328389	0.225769	0.230491	0.305637	0.652293	0.295429	0.403875
XLOC_044750	SUCLG1	chr11	50388994	50422131	5.46419	4.4725	8.18635	4.19298	4.88814	4.20123	5.27459	6.20619	5.88823
XLOC_048084	SUGT1;LECT1	chr12	11090676	11150990	1.01351	0.707603	0.264311	1.37401	0.83689	1.0551	2.22259	1.9449	1.62444
XLOC_050643	SULF2	chr13	76823031	77031892	0.777238	0.620248	0.405564	0.0232356	0.0203558	0.0269843	0.0475221	0.078326	0.136091
XLOC_079810	SULT1B1	chr6	86848265	86956457	37.8445	32.952	45.5262	11.6535	12.2134	16.9094	11.2536	14.6868	10.3943
XLOC_090530	SULT1C4	chr9	34518917	34519546	7.67013	4.58492	7.19444	0.0	0.0	0.0	0.0	0.0	0.0
XLOC_090531	SULT1C4	chr9	34520295	34521072	1.16751	3.49072	1.82588	0.0	0.0	0.0	0.0	0.0	0.0
XLOC_079813	SULT1E1	chr6	87009752	87090350	16.5651	24.4519	19.4167	63.2649	77.1865	66.2244	71.3718	78.9677	75.8811
XLOC_081544	SULT1E1	chr6	87090675	87092671	5.50731	3.41346	7.69639	3.52754	4.69287	3.80723	3.95864	3.91812	3.27014
XLOC_081545	SULT1E1	chr6	87093610	87094211	1.63089	0.487396	0.0	0.292114	0.380196	0.168421	0.583311	0.323218	0.568269
XLOC_076491	SULT4A1	chr5	115011544	115098839	44.7292	44.0715	50.1399	29.3058	21.3015	29.1693	30.3442	33.3129	22.3984
XLOC_077006	SULT4A1	chr5	115110296	115119344	6.75719	6.2668	9.67641	30.2488	30.5242	27.3321	29.8498	38.7361	31.3101
XLOC_078788	SULT4A1	chr5	115011544	115098839	0.0	2.73126	0.0	1.2289	1.74139	0.930772	0.7792	1.75611	1.18579
XLOC_078789	SULT4A1	chr5	115011544	115098839	4.6591	2.08717	3.63903	2.71022	1.98022	0.95816	2.87951	2.289	2.02417
XLOC_078790	SULT4A1	chr5	115100207	115100774	0.0	1.0556	0.0	0.158163	0.411322	0.364509	0.315095	0.698965	0.768944
XLOC_078791	SULT4A1	chr5	115103055	115103593	401.102	390.186	314.886	1271.36	1120.68	1238.62	1185.69	1271.28	1429.32
XLOC_066402	SUMO1;LOC614993	chr2	91138054	91183879	4.29901	3.43505	3.69737	2.1994	2.74586	2.14564	2.3035	3.5845	2.32018
XLOC_064323	SUMO2;ARMC7;HN1;NT5C	chr19	56912409	57008591	9.80707	8.87	9.38217	0.840487	0.4671	0.908183	0.175838	0.351568	0.515221

XLOC_065375	SUMO2;ARMC7;HN1;NT5C	chr19	56912409	57008591	0.0	0.290538	0.0	0.0	0.0	0.0	0.0	0.0	0.0
XLOC_065376	SUMO2;ARMC7;HN1;NT5C	chr19	56912409	57008591	0.0	0.0	0.0	0.0	0.0	0.149116	0.0	0.0	0.0
XLOC_065377	SUMO2;ARMC7;HN1;NT5C	chr19	56912409	57008591	0.0	0.0	0.0	0.0	0.144649	0.0	0.0	0.0	0.0
XLOC_065378	SUMO2;ARMC7;HN1;NT5C	chr19	56912409	57008591	1.32644	0.396804	0.518909	0.0	0.0519393	0.0	0.0	0.0	0.0
XLOC_065379	SUMO2;ARMC7;HN1;NT5C	chr19	56912409	57008591	0.0	0.570998	2.98636	0.171096	0.0	0.0	0.0	0.0	0.0
XLOC_065380	SUMO2;ARMC7;HN1;NT5C	chr19	56912409	57008591	0.0	0.0	0.0	0.0	0.0	0.0949069	0.0	0.0	0.0
XLOC_065381	SUMO2;ARMC7;HN1;NT5C	chr19	56912409	57008591	0.0	1.38704	0.0	0.0	0.0	0.238784	0.0	0.0	0.201773
XLOC_065382	SUMO2;ARMC7;HN1;NT5C	chr19	56912409	57008591	0.0	0.46649	0.610033	0.0	0.0	0.0	0.0	0.0	0.0
XLOC_065383	SUMO2;ARMC7;HN1;NT5C	chr19	56912409	57008591	1.54715	0.924855	0.0	0.0	0.120306	0.0	0.0	0.0	0.0
XLOC_065384	SUMO2;ARMC7;HN1;NT5C	chr19	56912409	57008591	0.0	0.418629	0.0	0.0	0.0	0.144823	0.0	0.0	0.0
XLOC_065385	SUMO2;ARMC7;HN1;NT5C	chr19	56912409	57008591	0.845417	0.75857	0.661332	0.075776	0.0	0.0877335	0.0	0.0	0.0
XLOC_065386	SUMO2;ARMC7;HN1;NT5C	chr19	56912409	57008591	4.94576	0.0	0.0	0.22128	0.0	0.0	0.0	0.0	0.0
XLOC_065387	SUMO2;ARMC7;HN1;NT5C	chr19	56912409	57008591	1.3228	0.593566	1.03495	0.0	0.0	0.0	0.0	0.0663225	0.0
XLOC_065388	SUMO2;ARMC7;HN1;NT5C	chr19	56912409	57008591	0.0	0.0	0.891422	0.10214	0.0	0.0	0.0	0.0	0.0
XLOC_065389	SUMO2;ARMC7;HN1;NT5C	chr19	56912409	57008591	0.853398	0.255255	0.0	0.15298	0.0	0.0	0.0	0.0	0.0

XLOC_078725	SUN2	chr5	110873808	110873928	684.603	559.394	1467.89	3148.24	2075.23	2883.25	1735.73	1879.15	2218.24
XLOC_076468	SUN2;APOBEC3A;CBX6;DNAL4;DNAL4;APOBEC3B;CBX7;MIR2440;RPL3;PDGFB	chr5	110879852	111278555	3.24675	2.15945	3.23957	0.408673	0.439891	0.464435	0.580206	0.643748	0.345363
XLOC_078726	SUN2;APOBEC3A;CBX6;DNAL4;DNAL4;APOBEC3B;CBX7;MIR2440;RPL3;PDGFB	chr5	110879852	111278555	1.46274	2.62317	4.57361	10.7432	9.44652	9.52607	11.2823	18.0094	10.8368
XLOC_076467	SUN2;GTPBP1	chr5	110839962	110873631	5.37293	17.5348	8.3796	44.4659	39.1947	40.8038	38.7888	41.7319	41.4156
XLOC_050906	SUN5;BPIFB2	chr13	62836883	62858763	0.0	0.0	0.0	0.254226	0.111191	0.147457	0.0863326	0.0949906	0.0826761
XLOC_042497	SUPT16H	chr10	25836939	25837224	22.5316	8.39578	4.39024	4.03474	10.2182	4.56066	3.30585	2.674	2.91079
XLOC_047359	SURF4	chr11	104330131	104332521	0.647905	0.775507	0.507082	1.83027	1.70445	1.41656	1.66179	3.39248	3.119
XLOC_047360	SURF4	chr11	104332638	104333702	1.5957	0.954539	1.24825	2.00242	1.74761	1.5736	1.37641	1.3578	1.81252
XLOC_057848	SUSD4	chr16	27388955	27452226	11.0449	8.36547	10.245	9.2602	10.5005	10.5346	11.7693	8.94673	11.7515
XLOC_041836	SV2C	chr10	7443813	7473619	4.44018	3.50859	3.82589	3.45827	4.02435	3.92034	2.12574	2.23008	3.23402
XLOC_042272	SV2C	chr10	7440286	7440973	2.73195	2.04166	0.0	1.95786	2.12743	1.41296	0.981871	0.950571	1.78658
XLOC_042273	SV2C	chr10	7441140	7442815	3.81276	1.85371	1.86473	3.58964	2.87827	2.77605	1.52365	2.20339	2.70995
XLOC_042274	SV2C	chr10	7443250	7443561	4.69776	0.0	0.0	2.10092	2.85569	2.38549	0.398917	2.24894	2.02653
XLOC_050504	SVIL	chr13	34875420	34907484	58.6326	50.359	51.728	66.5133	62.0881	63.6355	76.171	75.1429	70.1929
XLOC_050806	SVIL	chr13	34947052	34994124	13.5393	17.6666	13.2381	23.7924	20.2995	19.0636	26.3333	25.322	34.545
XLOC_051569	SVIL	chr13	34875420	34907484	12.6122	13.3458	8.34683	10.5204	11.4444	11.4685	10.1857	9.69164	13.5545
XLOC_051570	SVIL	chr13	34907693	34909280	0.0	0.605469	0.791787	0.816539	1.03147	0.526144	0.785188	0.203321	0.708055
XLOC_059880	SVOP	chr17	66318979	66319455	0.0	2.16963	5.67212	4.57446	1.09158	2.19928	2.99377	1.36456	3.12717
XLOC_072727	SVOPL	chr4	103082033	103164598	49.9915	50.4345	49.4245	22.0387	23.3081	25.6649	26.4728	24.916	25.4689
XLOC_073263	SVOPL	chr4	103082033	103164598	4.3654	4.783	5.50968	1.44728	1.99387	2.54088	1.5389	2.10961	2.25646
XLOC_074781	SVOPL	chr4	103082033	103164598	0.0	1.23517	0.807555	0.092532	0.0806391	0.428176	0.0	0.412254	0.0
XLOC_074782	SVOPL	chr4	103082033	103164598	2.22907	4.6608	1.74124	0.598526	0.689604	0.917569	0.394497	1.31573	0.775062
XLOC_074783	SVOPL	chr4	103082033	103164598	2.57234	2.05171	0.670763	0.614823	0.871597	0.444944	0.466675	0.68619	1.12357

XLOC_074784	SVOPL	chr4	103082033	103164598	0.0	7.6238	3.06732	0.702923	1.97767	2.22535	1.57025	1.74351	1.87862
XLOC_074785	SVOPL	chr4	103082033	103164598	6.21006	4.94288	10.0919	2.9707	2.8308	2.54535	4.37227	3.07326	3.20185
XLOC_074786	SVOPL	chr4	103082033	103164598	2.22424	2.8831	5.22015	1.86089	2.37909	1.46287	2.02172	1.70826	1.81426
XLOC_074787	SVOPL	chr4	103082033	103164598	6.87382	5.4851	6.40451	1.79061	3.03303	2.86257	3.02818	1.87862	2.80764
XLOC_074788	SVOPL	chr4	103164759	103165269	26.6758	20.2286	24.0459	14.695	14.9383	16.2744	21.1213	17.3978	17.8475
XLOC_074789	SVOPL	chr4	103165487	103165959	13.7996	22.6675	30.5373	12.1442	14.044	13.0095	14.6283	13.112	14.59
XLOC_074790	SVOPL	chr4	103170263	103173083	4.34612	1.86959	1.27563	2.04633	1.96327	1.78224	1.5442	1.53269	1.30788
XLOC_073264	SVOPL;ATP6V0A4	chr4	103173336	103214866	3.20739	3.99549	0.908759	2.34375	2.47172	2.40925	3.02505	1.80692	1.62496
XLOC_074791	SVOPL;ATP6V0A4	chr4	103173336	103214866	3.6763	3.29537	1.43633	1.15208	1.42601	0.947908	2.29236	0.908444	0.800005
XLOC_056473	SWAP70	chr15	43528247	43528882	0.0	0.0	2.36806	0.54269	1.17817	0.313081	0.271462	0.0	0.396018
XLOC_056474	SWAP70	chr15	43530965	43531700	1.25243	0.374431	0.0	0.336624	0.488092	0.259284	0.225533	0.249361	0.76475
XLOC_055448	SWAP70;SBF2	chr15	43477186	43527913	26.8607	25.2533	20.9258	36.971	39.7489	39.9436	44.8859	39.6589	54.8766
XLOC_053343	SYBU	chr14	56886422	56963740	38.6381	39.8431	30.0923	34.3689	46.973	41.0695	39.0569	34.1558	48.5349
XLOC_054497	SYBU	chr14	56963881	56964721	4.23892	0.950643	1.65751	1.5194	1.81999	1.09827	1.05331	0.422986	2.03513
XLOC_054498	SYBU	chr14	56965478	56966123	1.48367	2.66078	2.31955	1.9934	1.73142	1.99366	1.46324	0.147285	2.19832
XLOC_054499	SYBU	chr14	56966311	56967213	0.971472	0.290515	1.51961	0.957681	1.59371	1.41029	0.791316	0.291133	2.71453
XLOC_054500	SYBU	chr14	56968744	56969241	0.0	0.0	1.66431	0.762829	1.64901	0.658033	0.566354	0.209848	0.555773
XLOC_054501	SYBU	chr14	56969528	56970825	0.634701	0.569603	3.4761	0.341406	1.54123	0.329762	0.924863	0.127332	1.44275
XLOC_054502	SYBU	chr14	56977444	56978339	0.980694	1.46635	1.53401	0.527325	0.995873	0.610113	0.709947	0.0979545	1.54136
XLOC_069917	SYDE2;C3H1orf52;BCL10;WDR63;MCOLN3	chr3	59005337	59275942	2.60116	3.95042	3.0139	0.552987	0.712247	0.4776	0.293793	0.21089	0.438124
XLOC_086555	SYK	chr8	88521366	88592100	3.23171	6.16922	4.0364	1.05989	1.26277	1.34013	1.41184	0.840831	0.84424
XLOC_076872	SYN3	chr5	71569759	71658037	0.790247	0.766994	0.204909	0.207838	0.0929905	0.184872	0.192073	0.0596466	0.0
XLOC_050521	SYNDIG1;ENTPD6;ACSS1;C13H20orf3	chr13	42709777	43094928	0.696425	0.83328	1.63454	0.0	0.0	0.0723312	0.0633453	0.0	0.12174
XLOC_041604	SYNE2	chr10	76463181	76515863	12.07	16.7189	15.1919	43.3309	26.893	29.7069	31.7531	40.7437	28.9494
XLOC_041605	SYNE2	chr10	76531604	76553726	45.9268	44.9276	35.2957	28.8845	31.2126	33.6383	29.3589	25.1033	28.6046
XLOC_042003	SYNE2	chr10	76351055	76443675	13.8923	14.8666	13.785	28.8742	22.4649	24.9766	29.4442	38.0956	23.9335
XLOC_042004	SYNE2	chr10	76463181	76515863	0.0	0.0	2.37787	4.91358	3.59524	3.47758	3.176	4.76034	2.59341
XLOC_042005	SYNE2	chr10	76595497	76614126	26.6797	12.312	15.4179	1.54302	0.450624	1.03887	1.00638	0.603132	0.589415

XLOC_042006	SYNE2	chr10	76614338	76623311	27.9212	10.5967	18.476	2.2133	0.502937	0.778992	1.35814	1.17855	1.03111
XLOC_042986	SYNE2	chr10	76460157	76460913	0.0	2.16784	1.88987	1.08275	1.69607	1.8769	1.30677	1.32407	2.10866
XLOC_042987	SYNE2	chr10	76461258	76461990	0.0	1.12915	0.0	2.93262	2.45308	3.12753	2.83355	4.51158	2.08654
XLOC_042988	SYNE2	chr10	76462298	76462782	2.2154	0.661701	1.73044	4.75882	4.45517	4.10328	2.54802	5.23155	4.42924
XLOC_042989	SYNE2	chr10	76553907	76555176	1.95207	4.08761	2.54543	7.05836	8.05217	10.3439	6.69441	7.56981	8.47585
XLOC_042990	SYNE2	chr10	76555676	76556447	2.35786	3.17234	1.84371	3.69709	5.42469	5.12776	5.10204	3.87649	5.34904
XLOC_042991	SYNE2	chr10	76556594	76556836	8.30118	9.9073	6.47489	13.4552	16.129	21.7052	9.47389	14.696	24.9607
XLOC_041680	SYNJ2BP	chr10	82383769	82401835	0.0	0.43289	0.0	0.129731	0.0566409	0.0	0.0	0.0	0.379456
XLOC_043155	SYNJ2BP	chr10	82354143	82354662	0.0	1.79326	0.0	3.58247	2.94547	3.29854	2.84337	0.986923	2.08876
XLOC_043156	SYNJ2BP	chr10	82365768	82367647	0.420116	0.754213	0.65754	1.16783	1.02193	1.70481	0.921946	1.14083	0.73517
XLOC_043157	SYNJ2BP	chr10	82379302	82379713	0.0	2.55503	0.0	5.87009	8.12469	4.97192	5.74033	3.62203	7.67123
XLOC_070804	SYPL2	chr3	34083669	34085086	1.72268	1.03072	1.7972	2.52265	2.56512	2.98454	2.93189	2.30571	3.91671
XLOC_070805	SYPL2	chr3	34086226	34086802	3.45665	1.03293	1.35067	3.25012	4.42857	1.96214	2.93105	3.07911	2.85953
XLOC_076200	SYT1	chr5	8422871	8454282	3.52309	5.74773	6.84463	30.8141	35.3962	29.5567	25.192	25.4401	29.7054
XLOC_076201	SYT1	chr5	8664560	8997239	5.27626	2.13087	4.05414	17.9611	16.6944	16.1177	17.9726	22.0221	19.4779
XLOC_076742	SYT1	chr5	8490070	8622548	4.62083	13.77	10.0258	1.1421	1.01597	1.03142	1.03618	1.0476	0.826165
XLOC_076743	SYT1	chr5	8664560	8997239	14.275	13.9452	11.6452	13.6743	15.1593	12.8467	16.6912	15.3217	20.0512
XLOC_077278	SYT1	chr5	8422871	8454282	0.0	0.0	0.0	6.63106	9.57915	5.53413	8.17574	10.2172	4.73214
XLOC_077279	SYT1	chr5	8422871	8454282	2.55136	0.0	5.97664	11.186	11.6124	10.7421	4.48748	6.501	7.7532
XLOC_077280	SYT1	chr5	8422871	8454282	0.0	0.0	0.0	3.55924	3.03853	4.46134	4.44203	4.22258	3.44034
XLOC_077281	SYT1	chr5	8422871	8454282	0.0	0.930783	0.0	4.74181	6.29581	5.14825	2.92786	3.08886	5.96997
XLOC_077282	SYT1	chr5	8422871	8454282	6.35722	0.948594	0.0	5.40148	3.17257	6.83013	4.97699	5.87583	7.16041
XLOC_077283	SYT1	chr5	8454391	8454809	2.77648	1.65773	0.0	11.1773	16.0362	10.8201	15.0787	7.32724	13.73
XLOC_077284	SYT1	chr5	8461650	8461973	0.0	0.0	0.0	1.56012	3.65317	2.66154	1.48884	2.09484	1.12976
XLOC_077285	SYT1	chr5	8664560	8997239	0.0	0.0	0.0	0.487626	1.37769	0.563074	1.71197	0.81176	0.118659
XLOC_077286	SYT1	chr5	8664560	8997239	0.0	0.0	1.70954	1.95889	1.1852	2.92809	1.74351	1.7233	3.42474
XLOC_077287	SYT1	chr5	8664560	8997239	0.0	0.0	0.0	1.43941	2.14984	2.86965	5.1785	1.16377	3.47785
XLOC_077288	SYT1	chr5	8664560	8997239	0.0	0.322553	0.0	1.54657	2.02067	1.90025	0.876919	1.07614	1.12986
XLOC_077289	SYT1	chr5	8664560	8997239	17.2355	16.1948	35.6164	11.6918	14.0121	14.657	21.7345	23.4147	19.117
XLOC_077290	SYT1	chr5	8664560	8997239	0.0	0.876167	0.0	0.78768	0.684247	0.757553	0.657239	1.16421	0.894241
XLOC_077291	SYT1	chr5	8664560	8997239	0.0	0.663742	1.73574	0.99446	1.20309	1.60056	0.982899	1.3118	0.579488
XLOC_076378	SYT10	chr5	76657773	76856586	2.0674	2.47368	0.0	0.283861	0.557447	0.251575	0.0945333	0.104388	0.138351
XLOC_078161	SYT10	chr5	76657773	76856586	0.0	0.0	1.7738	0.0	0.0	0.467213	0.200724	0.0	0.0

XLOC_078162	SYT10	chr5	76657773	76856586	0.0	1.26288	0.0	0.378692	0.0	0.0	0.362105	0.0	0.0
XLOC_078163	SYT10	chr5	76657773	76856586	0.0	0.0	0.0	0.0	0.0	0.154933	0.0	0.0	0.130646
XLOC_055824	SYT13	chr15	76178211	76231366	43.8647	34.1991	39.2593	15.2893	13.8987	14.2859	13.2957	16.8408	12.6036
XLOC_056973	SYT13	chr15	76170751	76178014	41.1199	35.4595	39.2813	17.5384	15.4218	15.0153	14.7551	18.5603	11.9651
XLOC_089984	TAAR6	chr9	71504319	71592391	8.33888	6.2375	9.78939	0.280416	0.218227	0.294712	0.127242	0.106836	0.0928862
XLOC_076469	TAB1	chr5	111330719	111386633	34.9334	57.897	24.503	26.8372	46.8899	54.237	62.2674	65.6491	24.8162
XLOC_044348	TAF1B	chr11	87617686	87658004	1.02044	1.98442	0.0	0.457471	1.20019	0.583643	0.745197	0.307547	0.401647
XLOC_044349	TAF1B	chr11	87662659	87664977	1.1623	0.173854	0.0	0.183876	0.136624	0.333489	0.0375065	0.0	0.14354
XLOC_046933	TAF1B	chr11	87658198	87659092	0.0	0.881004	0.0	0.352027	0.920506	0.610936	0.444309	0.0980859	0.171494
XLOC_091532	TAGAP	chr9	96807247	96807890	0.0	0.0	0.0	1.3344	0.231796	0.461953	0.53422	0.295762	0.129843
XLOC_065621	TANK	chr2	35256351	35306607	33.5338	17.1904	15.9801	6.32216	3.69878	4.12878	4.70889	3.99166	5.6714
XLOC_066259	TANK	chr2	35315322	35337016	9.22703	7.9592	8.93234	3.22111	7.98878	5.14491	4.7851	3.87123	8.78056
XLOC_067315	TANK	chr2	35307012	35308058	8.14015	5.35625	7.00434	2.11592	1.33709	1.68981	1.2561	1.05914	1.84914
XLOC_067316	TANK	chr2	35313984	35315230	7.30885	3.97517	1.03967	2.26348	4.52698	1.72575	1.75389	2.19861	6.67966
XLOC_067317	TANK	chr2	35340346	35341534	13.335	13.8559	15.3741	68.8926	63.683	70.93	69.4565	73.6484	75.0249
XLOC_076926	TAS2R42	chr5	98930007	99036960	0.172832	0.206901	0.405865	0.108513	0.0407665	0.018011	0.0793833	0.0697428	0.075679
XLOC_078395	TAS2R42	chr5	98930007	99036960	18.7344	19.6015	14.6468	81.8774	70.0425	79.4667	76.7496	88.4963	71.6496
XLOC_073209	TAX1BP1;HIBADH	chr4	68826826	68954193	0.0	0.289738	0.0	0.347316	0.0	0.502334	0.0	0.38715	0.0846025
XLOC_079741	TBC1D1	chr6	58831481	58919738	9.85575	12.2288	8.49078	5.98126	5.75349	4.4051	5.31759	4.73486	3.96461
XLOC_080139	TBC1D1	chr6	58953457	58956820	0.0	0.819756	1.07195	0.368489	0.106771	0.141827	0.369563	0.272604	0.0
XLOC_081181	TBC1D1	chr6	58919922	58920542	0.0	1.4022	0.0	0.560264	1.09431	0.807844	1.96011	1.39586	0.681318
XLOC_081182	TBC1D1	chr6	58935263	58935794	0.0	0.0	0.0	0.0	0.15012	0.399227	0.344402	0.191191	0.16851
XLOC_064041	TBC1D16	chr19	53140473	53247168	17.7292	19.1712	15.9296	18.9528	21.0086	19.6483	21.3865	17.7952	24.8884
XLOC_080097	TBC1D19	chr6	47530154	47595328	6.37726	9.26577	7.85386	49.7377	51.6484	56.8892	67.7603	70.503	47.0871
XLOC_080982	TBC1D19	chr6	47530154	47595328	0.0	6.54815	0.0	17.8841	20.2987	18.6636	7.00672	11.0751	20.6889
XLOC_080983	TBC1D19	chr6	47530154	47595328	4.7823	0.0	3.73525	0.641941	1.10815	1.4747	3.16328	1.40868	1.6618
XLOC_080984	TBC1D19	chr6	47530154	47595328	6.04633	1.35538	1.18185	2.84318	3.40962	2.81189	1.89637	1.95006	3.29329
XLOC_080985	TBC1D19	chr6	47530154	47595328	0.935315	1.25928	1.46392	1.76118	1.90745	2.33498	2.81976	2.39734	2.37284
XLOC_080986	TBC1D19	chr6	47595462	47596095	0.0	0.0	0.0	1.63491	1.30137	0.62877	0.681429	0.452801	0.397675
XLOC_080987	TBC1D19	chr6	47597075	47598547	0.0	0.329206	0.0	1.13459	0.819421	0.514816	0.50187	0.165742	0.433056
XLOC_080988	TBC1D19	chr6	47626712	47626969	0.0	0.0	5.56505	3.20494	0.0	0.719508	0.588292	2.68022	0.613726
XLOC_076514	TBC1D22A	chr5	118170047	118185163	0.0	1.06438	1.8558	0.425294	0.925416	0.860194	0.320925	0.472916	0.828308

XLOC_076515	TBC1D22A	chr5	118245229	118263630	4.30274	5.22211	4.67755	4.03052	3.58783	4.08366	3.81608	4.48172	3.95558
XLOC_077023	TBC1D22A	chr5	118063118	118155542	11.1636	10.9726	9.07012	9.16644	8.68564	8.42404	9.77161	10.2302	10.7114
XLOC_077024	TBC1D22A	chr5	118191689	118242963	1.86158	1.81057	1.86177	1.64871	2.21131	1.8169	1.43283	1.35858	2.09549
XLOC_078850	TBC1D22A	chr5	118186046	118186834	0.0	1.02897	0.897028	0.205572	0.984362	0.356462	0.620921	0.457385	0.300312
XLOC_078851	TBC1D22A	chr5	118188885	118189475	1.67237	0.999546	2.61404	0.748829	1.42904	1.89927	0.298848	0.496909	0.0
XLOC_078852	TBC1D22A	chr5	118191076	118191468	0.0	0.920404	7.22039	1.65498	2.84327	0.947137	0.80609	2.70323	3.74185
XLOC_078853	TBC1D22A	chr5	118191689	118242963	0.0	1.43803	0.0	0.215472	0.187521	0.373522	0.0	0.718671	0.52455
XLOC_078854	TBC1D22A	chr5	118263724	118264145	0.0	6.55568	4.2858	1.47338	2.53722	1.12621	1.44343	2.9523	5.47776
XLOC_078855	TBC1D22A	chr5	118264199	118264591	0.0	0.920404	2.4068	1.65498	2.36939	0.947137	1.07479	0.600718	1.87092
XLOC_078856	TBC1D22A	chr5	118264763	118265267	0.0	3.74149	0.0	1.4949	1.9395	0.429939	2.22137	0.617145	2.36006
XLOC_078857	TBC1D22A	chr5	118265328	118266320	2.60008	2.07366	0.677928	1.32056	1.89736	1.79858	2.2006	1.47371	2.11984
XLOC_078858	TBC1D22A	chr5	118278342	118278617	0.0	0.0	0.0	3.82308	4.59643	1.232	0.508128	0.576527	2.62305
XLOC_038112	TBC1D5	chr1	156006324	156065899	87.2209	91.364	79.4773	64.7578	60.6312	57.0547	72.7929	76.2542	73.5391
XLOC_038605	TBC1D5	chr1	155844650	155973191	26.9661	30.121	26.2612	6.61102	9.15504	7.19516	7.34887	6.0048	7.16339
XLOC_038606	TBC1D5	chr1	156006324	156065899	22.7819	24.3333	18.8717	4.94054	7.28008	5.20249	6.75121	6.29899	6.30838
XLOC_038607	TBC1D5	chr1	156255388	156303406	10.6798	21.7054	18.7158	2.24368	1.214	1.93649	1.37789	1.46556	0.812073
XLOC_040671	TBC1D5	chr1	156004676	156005276	9.80745	6.83894	8.94272	1.02471	1.01612	0.506395	1.46147	0.485902	0.996706
XLOC_040672	TBC1D5	chr1	156005523	156005872	0.0	5.62849	2.9435	0.0	0.865483	1.15472	1.95022	0.729482	0.0
XLOC_040673	TBC1D5	chr1	156006324	156065899	1.65032	1.72758	1.93649	0.443787	0.387222	0.599536	0.524206	0.0825769	0.288373
XLOC_040674	TBC1D5	chr1	156006324	156065899	0.0	1.71095	0.745883	0.34185	0.670507	0.69226	0.863313	0.285822	0.582888
XLOC_040675	TBC1D5	chr1	156006324	156065899	4.24196	2.02991	1.99096	1.82502	1.12781	0.968486	2.23184	1.01853	1.33407
XLOC_040676	TBC1D5	chr1	156006324	156065899	17.3934	23.2357	20.4197	13.6208	14.6848	13.9707	17.5588	17.8611	20.3086
XLOC_040677	TBC1D5	chr1	156006324	156065899	0.492164	1.47249	0.770292	1.67692	1.38947	1.74037	2.11227	1.92874	1.89425
XLOC_040678	TBC1D5	chr1	156066043	156067296	20.466	16.9841	21.1772	20.8923	19.182	22.0861	29.027	27.805	25.9135
XLOC_040679	TBC1D5	chr1	156067443	156067692	23.1529	23.0197	30.0898	11.797	12.1373	16.316	11.3839	25.9801	19.234
XLOC_040680	TBC1D5	chr1	156068062	156069103	6.54898	8.32465	12.8072	10.4193	9.34824	13.3397	17.1633	12.6171	14.0898
XLOC_040681	TBC1D5	chr1	156075974	156076751	1.16751	0.698145	0.0	0.0	0.0910617	0.0	0.0	0.0	0.0
XLOC_040682	TBC1D5	chr1	156076989	156077474	0.0	0.659685	0.0	0.0	0.0	0.0	0.0	0.0	0.0
XLOC_040683	TBC1D5	chr1	156077658	156078808	0.728713	2.61565	0.570083	0.130646	0.0	0.0756768	0.0	0.0730115	0.0
XLOC_044649	TBC1D8	chr11	6105757	6200426	14.1804	13.773	18.5214	21.4678	20.469	19.846	29.7037	32.3304	24.6945
XLOC_044650	TBC1D8	chr11	6105757	6200426	114.671	190.909	95.9172	376.661	247.987	359.42	222.717	353.782	407.598
XLOC_045406	TBC1D8	chr11	6098724	6099691	0.893461	1.33603	1.39769	0.240232	0.558724	0.648836	0.404901	0.625359	1.09255
XLOC_045407	TBC1D8	chr11	6100248	6101484	0.670602	0.802401	0.0	0.360704	0.945254	0.557338	0.67136	0.403424	0.234485

XLOC_045408	TBC1D8	chr11	6102699	6104013	1.25074	1.68371	0.48929	0.953107	0.68586	0.779839	0.341785	0.501898	1.09353
XLOC_045409	TBC1D8	chr11	6104517	6105341	2.17035	0.973442	2.54588	1.36136	1.27044	1.12446	1.27411	0.541257	0.94718
XLOC_045410	TBC1D8	chr11	6105757	6200426	6.20838	2.96976	8.73771	3.89371	2.8069	2.95671	2.90738	3.09076	1.84141
XLOC_041841	TBCA	chr10	8723199	8945320	10.2501	7.02148	10.0716	7.88284	6.41485	7.1051	3.07	4.28587	3.03966
XLOC_042306	TBCA	chr10	8723199	8945320	0.963996	0.144209	0.0	0.0	0.0	0.050134	0.0	0.0	0.0
XLOC_055728	TBCEL	chr15	32240581	32244663	4.36053	3.41148	2.96113	0.485979	0.374223	0.221834	0.441303	0.2117	0.506877
XLOC_092303	TBL1X	chrX	144471055	144477124	0.0	0.0	0.0	0.0969436	0.084954	0.0675654	0.138895	0.108983	0.0946434
XLOC_037975	TBL1XR1	chr1	90603433	90613457	24.8319	34.5273	29.3668	33.3602	29.0992	31.742	35.4652	41.5211	36.7168
XLOC_089594	TBP;TBP;PSMB1	chr9	105642998	105688042	9.65678	15.1576	13.6481	18.141	13.4014	15.1755	15.8474	21.7432	14.1356
XLOC_089508	TBPL1	chr9	73102177	73123002	30.6434	25.1102	18.3539	108.06	77.0083	95.6626	98.0066	106.315	97.7722
XLOC_091273	TBPL1	chr9	73102177	73123002	0.0	0.0	0.972535	3.00889	2.61775	1.15889	0.896068	1.73373	2.4956
XLOC_091274	TBPL1	chr9	73102177	73123002	0.0	0.0	0.0	0.873373	0.941785	2.00562	0.214966	0.718238	1.90714
XLOC_091275	TBPL1	chr9	73102177	73123002	0.0	0.822952	1.43489	1.64423	1.0753	1.04666	0.664804	0.733541	0.801152
XLOC_091276	TBPL1	chr9	73102177	73123002	0.0	2.32552	6.07955	7.01205	2.33472	0.785248	4.4689	2.91482	4.02017
XLOC_091277	TBPL1	chr9	73123212	73123500	0.0	4.92495	4.29221	8.87378	8.3305	9.47889	7.3994	7.32775	4.74389
XLOC_091278	TBPL1	chr9	73126934	73127445	0.0	0.611256	1.59853	0.732677	0.633905	0.210766	0.544752	0.806967	2.13569
XLOC_091279	TBPL1	chr9	73129555	73130191	1.51158	0.0	1.18156	0.812336	0.70544	0.468649	1.35455	0.300009	0.395195
XLOC_069850	TBX15	chr3	24332875	24333554	252.949	265.81	213.198	791.957	695.285	770.917	681.652	717.526	911.691
XLOC_089473	TBX18	chr9	65587089	65595198	0.973156	1.44708	0.852501	17.9779	17.0061	15.0447	19.0333	16.2957	16.4266
XLOC_089962	TBX18	chr9	65554250	65580058	24.8442	20.2753	28.5946	3.67367	3.9504	3.52907	3.27503	4.84673	3.27627
XLOC_091112	TBX18	chr9	65595739	65596275	1.91101	0.570952	0.0	13.3453	15.7057	17.1396	15.6405	14.1543	13.6365
XLOC_063918	TBX2	chr19	11912557	11945228	30.9356	34.2064	30.0699	19.1542	18.3544	18.4875	20.2745	23.0583	16.2798
XLOC_073194	TBX20	chr4	62192196	62209163	17.7096	13.3119	18.3746	7.03372	8.09285	7.14029	4.26159	5.46124	7.85107
XLOC_074367	TBX20	chr4	62192196	62209163	3.1786	9.48594	2.48049	1.70571	0.488085	0.650469	0.829498	2.16474	0.550794
XLOC_064600	TBX4	chr19	11877932	11878391	7.19724	9.31375	7.49428	7.72875	7.78228	6.16465	11.8471	12.4834	8.75311
XLOC_063992	TCAP;STARD3;PGAP3;PPP1R1B;PNMT	chr19	40659466	40740014	9.14187	4.28387	7.53312	0.107891	0.108231	0.047842	0.0275574	0.0462327	0.0
XLOC_092136	TCEAL8;BEX4	chrX	57409073	57488770	9.047	15.1512	11.2149	2.66834	0.540293	0.716347	2.17672	1.05188	1.30387
XLOC_092284	TCEANC	chrX	137049430	137075764	0.923963	4.1788	3.19069	3.06542	3.98621	4.7772	4.64352	2.60062	3.56859
XLOC_041536	TCF12	chr10	53021895	53108791	1.3301	0.0	0.0	0.17886	0.0	0.207279	0.0	0.0666854	0.0581373
XLOC_041933	TCF12	chr10	53276736	53358397	4.85445	4.47467	3.79985	0.609564	0.946401	0.566518	0.909351	0.567935	0.816186

XLOC_042700	TCF12	chr10	53108841	53110087	1.99332	0.0	0.0	0.0	0.260171	0.0690298	0.060479	0.0666246	0.0
XLOC_042701	TCF12	chr10	53110184	53111373	0.0	0.419469	2.19417	0.188564	0.109787	0.218484	0.0	0.0	0.0
XLOC_042702	TCF12	chr10	53273459	53276515	2.24564	1.49339	1.17179	0.201403	0.509752	0.389846	0.389096	0.22633	0.480595
XLOC_042703	TCF12	chr10	53276736	53358397	6.02116	5.39899	7.05988	10.2468	11.1235	13.3792	11.602	14.3416	13.5122
XLOC_042704	TCF12	chr10	53378643	53379420	0.0	1.39629	0.912938	0.104609	0.273185	0.0	0.21057	0.0	0.0
XLOC_076987	TCF20;MIR2442;CYP2D14;MGC127055;MGC127055	chr5	113613046	113661641	0.779341	0.775517	0.546208	6.2383	7.43445	6.39939	10.6806	10.3974	14.2193
XLOC_089507	TCF21	chr9	73044355	73096599	0.0	2.46115	1.01698	33.2812	31.5322	34.1547	23.5569	24.8186	30.4257
XLOC_044218	TCF23	chr11	72441089	72489703	24.7085	26.6522	26.6614	78.8344	66.5908	82.2277	93.9602	114.133	50.3541
XLOC_044900	TCF23	chr11	72441089	72489703	1.87684	1.4382	1.36033	1.97812	1.83209	1.65583	1.97634	2.51775	1.55308
XLOC_083000	TCF3	chr7	45627503	45644326	3.70778	3.09271	3.33996	1.41184	1.6157	1.96442	0.667644	1.46798	0.721239
XLOC_083640	TCF3	chr7	45627503	45644326	0.0	0.20397	0.514247	0.222869	0.301543	0.227938	0.181596	0.146151	0.0680441
XLOC_084846	TCF3	chr7	45619067	45619493	0.0	0.0	0.0	0.72301	2.07582	0.276396	0.0	0.0	0.0
XLOC_060283	TCHP;GIT2	chr17	65579961	65614310	146.691	129.418	126.024	119.395	106.392	112.491	135.784	140.24	136.155
XLOC_060263	TCTN1;HVCN1	chr17	56751086	56757561	0.637498	1.33493	0.0	3.88631	3.14592	3.24585	2.67056	4.15638	3.17686
XLOC_063945	TEKT1	chr19	25672584	25673587	0.0	12.5781	14.9497	0.0	0.0	0.0	0.0	0.0	0.331469
XLOC_063946	TEKT1	chr19	25673887	25678983	8.26541	8.65438	14.6556	0.0529428	0.138837	0.0	0.0	0.0	0.0
XLOC_041489	TEP1;TTC5;CCNB1IP1	chr10	26753439	26918764	26.6827	22.5534	14.2756	1.358	2.53386	1.70538	1.57122	1.04841	2.63696
XLOC_062199	TERF2;COG8;TMED6;VPS4A;NIP7;SNTB2	chr18	36559068	36641539	29.0541	34.1062	22.9254	30.8422	34.0179	35.3252	35.9747	33.6821	34.0969
XLOC_062988	TERF2;COG8;TMED6;VPS4A;NIP7;SNTB2	chr18	36559068	36641539	19.2297	15.2859	4.99553	16.6638	20.7518	16.1788	19.6976	22.9806	16.5425
XLOC_062989	TERF2;COG8;TMED6;VPS4A;NIP7;SNTB2	chr18	36559068	36641539	32.5657	46.8042	34.5046	75.3088	72.0065	76.3219	78.3234	86.8048	99.3141
XLOC_072642	TES	chr4	52438976	52488365	38.2785	58.9845	38.0194	97.3741	98.6966	94.4905	124.828	114.668	146.942
XLOC_074230	TES	chr4	52495674	52495832	39.3982	0.0	0.0	7.64852	8.51178	3.96667	2.96358	3.44403	0.0
XLOC_074231	TES	chr4	52496984	52497187	0.0	8.32807	10.8843	6.356	8.18526	1.39026	4.37715	10.1039	7.16061

XLOC_047817	TEX26	chr12	29937993	30017796	16.8042	18.574	20.4193	2.97982	2.76344	3.19295	2.06557	1.2672	2.03907
XLOC_038081	TF	chr1	136683359	136686865	0.0	0.375731	0.0	1.12597	1.27341	0.780533	1.01834	1.6264	0.877024
XLOC_038082	TF	chr1	136695692	136697075	0.0	0.353276	0.891379	4.39155	2.12729	3.29456	1.82714	2.447	3.29924
XLOC_040446	TF	chr1	136683359	136686865	0.0	0.0	0.0	2.10225	0.420433	1.11783	0.160972	2.14299	0.943335
XLOC_040447	TF	chr1	136688762	136689010	0.0	0.0	6.07949	19.6322	12.2573	14.9102	10.2146	16.7592	17.4191
XLOC_040448	TF	chr1	136689166	136689443	0.0	3.57552	4.67411	5.37264	8.14418	10.9125	0.500531	9.65056	5.67848
XLOC_040449	TF	chr1	136697633	136697864	9.40216	0.0	0.0	8.49206	5.60092	4.72027	3.03912	6.09541	3.2299
XLOC_040450	TF	chr1	136698442	136698685	0.0	0.0	0.0	7.39389	6.13937	7.43453	4.02011	2.29693	0.705605
XLOC_040451	TF	chr1	136698815	136699443	0.0	0.0	0.0	3.4422	2.63006	3.97118	3.44234	3.81269	2.00939
XLOC_040452	TF	chr1	136699655	136699927	6.24849	0.0	0.0	6.72043	6.12106	10.7305	5.71956	7.08374	3.22615
XLOC_040453	TF	chr1	136700680	136701239	0.0	0.0	0.0	3.38749	2.3766	2.60184	1.44526	1.78147	1.4115
XLOC_040454	TF	chr1	136701634	136701952	0.0	0.0	0.0	0.0	2.39424	1.37091	0.382892	1.72515	0.0
XLOC_040455	TF	chr1	136704677	136704901	0.0	6.12698	0.0	11.1396	5.3311	5.1425	1.64724	7.56479	1.76088
XLOC_040456	TF	chr1	136706183	136706329	0.0	0.0	0.0	24.9071	44.4827	44.1311	46.2676	37.6039	21.8751
XLOC_040457	TF	chr1	136706777	136707197	0.0	0.0	0.0	7.39489	7.42779	8.76083	6.03607	5.92635	3.82498
XLOC_040458	TF	chr1	136707310	136707485	0.0	0.0	0.0	34.263	10.5798	12.1521	3.71495	10.7798	4.1914
XLOC_040459	TF	chr1	136708734	136708887	0.0	14.4617	0.0	55.3485	67.4488	33.177	31.6373	49.0133	36.9443
XLOC_040460	TF	chr1	136709292	136710271	1.76082	0.78992	0.0	5.83923	5.09323	5.48065	3.99083	4.66644	3.30682
XLOC_040461	TF	chr1	136710993	136711246	0.0	0.0	0.0	8.66413	7.78582	6.72461	2.43834	7.64436	1.91272
XLOC_040462	TF	chr1	136711466	136711762	5.19658	0.0	0.0	10.6953	11.0239	6.84745	5.25835	6.9335	3.13521
XLOC_040463	TF	chr1	136713521	136714178	1.44803	0.0	0.0	5.05851	4.39479	4.19179	2.20848	3.59522	2.77689
XLOC_040464	TF	chr1	136714406	136715778	0.0	0.0	0.0	3.14996	3.033	3.3423	2.38804	2.15147	1.51005
XLOC_040465	TF	chr1	136716974	136718600	0.0	0.147349	0.385385	2.56123	1.58352	3.07328	1.75391	1.7321	1.37857
XLOC_040466	TF	chr1	136719337	136720331	0.0	0.0	0.676308	3.40977	2.56887	1.88401	2.43065	1.29726	1.13292
XLOC_058096	TFB2M	chr16	31545052	31577578	0.0	0.0	0.0	0.0728809	0.157144	0.051642	0.0453392	0.0	0.0434346
XLOC_058776	TFB2M	chr16	31545052	31577578	0.0	0.0	0.0	0.0640333	0.0	0.0	0.0649587	0.0	0.0
XLOC_058778	TFB2M	chr16	31545052	31577578	0.0	0.0	0.0	0.0574045	0.0	0.0	0.0	0.0	0.0
XLOC_058782	TFB2M	chr16	31545052	31577578	0.0	0.0	0.0	0.0	0.0	0.0	0.111919	0.0	0.0537035
XLOC_058783	TFB2M	chr16	31545052	31577578	0.0	0.0	0.0	0.0	0.0	0.944054	0.0	0.0	0.0
XLOC_058787	TFB2M	chr16	31545052	31577578	0.0	0.0	0.0	0.0594562	0.0	0.0	0.0	0.0	0.0
XLOC_058788	TFB2M	chr16	31545052	31577578	0.0	0.0	0.0	0.110488	0.0961351	0.0	0.111075	0.245592	0.0
XLOC_058789	TFB2M	chr16	31545052	31577578	0.0	0.0	0.0	0.0	0.0	0.196494	0.0	0.0	0.0
XLOC_058790	TFB2M	chr16	31545052	31577578	0.0	0.0	0.340842	0.0	0.0	0.0453146	0.0	0.13139	0.0

XLOC_058791	TFB2M	chr16	31545052	31577578	0.0	0.0	0.0	1.12007	0.0	0.0	0.0	0.0	0.0
XLOC_058792	TFB2M	chr16	31545052	31577578	0.0	0.0	0.0	0.0	0.0	0.430965	0.0	0.0	0.0
XLOC_058793	TFB2M	chr16	31545052	31577578	0.0	0.0	0.0	0.0	0.0	0.0714486	0.0	0.0	0.0
XLOC_058794	TFB2M	chr16	31545052	31577578	0.0	0.0	0.0	0.0	0.540759	0.726241	0.0	0.0	0.0
XLOC_058796	TFB2M	chr16	31545052	31577578	0.0	0.0	0.0	0.0	0.0	0.0	0.0915469	0.0	0.0883779
XLOC_066343	TFCP2L1	chr2	73342979	73386475	1.3021	0.694019	1.73193	0.170179	0.313006	0.184394	0.0796773	0.200759	0.193742
XLOC_066344	TFCP2L1	chr2	73431984	73440076	3.47108	1.92862	4.1823	1.02258	1.63963	1.41498	2.17429	1.9531	1.48759
XLOC_067888	TFCP2L1	chr2	73429384	73431741	1.97288	0.885531	0.772032	0.294876	0.697145	0.718883	0.752931	0.39729	0.719486
XLOC_066345	TFCP2L1;CLASP1	chr2	73440126	73528824	4.25952	5.21356	6.59809	1.70739	0.724459	5.20967	0.508967	1.21191	0.655154
XLOC_067890	TFCP2L1;CLASP1	chr2	73440126	73528824	0.0	0.0	0.0	0.112794	0.0	0.0	0.453367	0.0	0.0
XLOC_067891	TFCP2L1;CLASP1	chr2	73440126	73528824	0.797851	0.238635	0.624125	0.0	0.0624148	0.0	0.0	0.0	0.0697129
XLOC_067892	TFCP2L1;CLASP1	chr2	73440126	73528824	1.21661	0.0	0.0	0.0	0.0	0.0	0.0	0.0	0.0
XLOC_067893	TFCP2L1;CLASP1	chr2	73440126	73528824	1.53403	0.458492	0.0	0.0	0.0	0.0	0.0	0.0	0.0
XLOC_067894	TFCP2L1;CLASP1	chr2	73440126	73528824	0.0	0.0	0.0	0.0	0.0492017	0.0	0.0572086	0.0630075	0.0
XLOC_067895	TFCP2L1;CLASP1	chr2	73440126	73528824	0.0	0.443534	0.0	0.199381	0.0	0.230969	0.0673888	0.0	0.064795
XLOC_067896	TFCP2L1;CLASP1	chr2	73440126	73528824	6.40918	0.0	0.0	0.0	0.4826	1.2943	0.0	0.0	0.0
XLOC_067897	TFCP2L1;CLASP1	chr2	73440126	73528824	4.70181	0.0	3.67505	0.561477	0.365551	0.809577	0.140302	0.0	0.273115
XLOC_067898	TFCP2L1;CLASP1	chr2	73440126	73528824	0.0	0.0	0.0	0.258894	0.0	0.14941	0.129658	0.0	0.125972
XLOC_067899	TFCP2L1;CLASP1	chr2	73440126	73528824	0.0	0.186756	0.0	0.167906	0.0489059	0.12975	0.170601	0.125259	0.109165
XLOC_067900	TFCP2L1;CLASP1	chr2	73440126	73528824	0.0	0.0	0.299232	0.13715	0.120047	0.0795872	0.209919	0.153893	0.167297
XLOC_067901	TFCP2L1;CLASP1	chr2	73440126	73528824	0.0	0.0	0.0	0.229173	0.199338	0.264743	0.0	0.127285	0.0
XLOC_067902	TFCP2L1;CLASP1	chr2	73440126	73528824	0.0	0.0	0.565527	0.324004	0.169754	0.0750744	0.0657274	0.362164	0.0631801
XLOC_067903	TFCP2L1;CLASP1	chr2	73440126	73528824	0.0	0.0	0.0	0.237014	0.137822	0.0914549	0.159824	0.264457	0.0769968
XLOC_067904	TFCP2L1;CLASP1	chr2	73440126	73528824	0.0	0.344079	0.0	0.103114	0.179539	0.357589	0.207618	0.22941	0.0

XLOC_067905	TFCP2L1;CLASP 1	chr2	73440126	73528824	0.0	0.0	0.620704	0.0711237	0.0620746	0.247107	0.216153	0.0	0.138663
XLOC_067906	TFCP2L1;CLASP 1	chr2	73440126	73528824	0.0	0.0	1.36698	0.469909	0.271599	0.36102	0.156072	0.173087	0.0
XLOC_067907	TFCP2L1;CLASP 1	chr2	73440126	73528824	0.0	0.450925	1.17935	0.270272	0.353928	0.31307	0.136994	0.301998	0.263489
XLOC_067908	TFCP2L1;CLASP 1	chr2	73440126	73528824	0.0	0.0	0.0	0.0	0.135799	0.0	0.78036	0.173087	0.152311
XLOC_067909	TFCP2L1;CLASP 1	chr2	73440126	73528824	0.0	0.394618	0.0	0.118262	0.361596	0.0685282	0.120086	0.132285	0.230645
XLOC_067910	TFCP2L1;CLASP 1	chr2	73440126	73528824	0.0	0.0	0.0	0.424784	0.122902	0.0	0.141494	0.313517	0.0
XLOC_048174	TGDS;GPR180	chr12	69584981	69681654	15.6575	18.9726	18.6437	2.92334	3.32275	3.1911	2.20435	1.73674	2.8072
XLOC_063180	TGFB1	chr18	50771599	50772872	0.0	0.0	0.0	0.52312	0.40625	1.07785	0.472261	0.520199	0.680177
XLOC_063181	TGFB1	chr18	50782237	50782842	0.0	0.0	0.0	1.01328	0.879296	1.16852	0.0	0.320386	1.54885
XLOC_057838	TGFB2	chr16	22548276	22549867	0.0	0.357725	0.0	1.60803	0.932998	0.867257	0.754882	0.715157	2.401
XLOC_058076	TGFB2	chr16	22576730	22622306	0.971472	0.58103	0.0	0.609434	0.0758909	0.302205	0.0	0.0970444	0.084829
XLOC_058675	TGFB2	chr16	22528549	22528817	0.0	0.0	5.0387	2.89818	1.45993	3.26321	2.14643	2.43888	1.66843
XLOC_041708	TGFB3	chr10	88201117	88245969	16.0281	19.5873	14.2557	23.8641	26.6518	23.692	24.3551	23.2494	35.8037
XLOC_086883	TGFBR1	chr8	64592508	64693073	109.661	125.445	149.98	1.99904	1.46778	1.64669	3.35715	1.55609	2.89485
XLOC_072669	THAP5;DNAJB9	chr4	59964272	60086337	0.0	0.0	0.0	58.1711	45.5235	97.275	29.7206	24.145	24.6333
XLOC_081678	THAP6	chr6	92088578	92091293	1.97949	0.761606	1.54931	0.50722	0.422067	0.559531	0.57032	0.484316	0.321815
XLOC_079836	THAP6;CDKL2	chr6	92091674	92168865	13.0376	14.7104	12.8722	14.7077	12.1797	14.1354	15.2771	14.8085	12.0634
XLOC_081679	THAP6;CDKL2	chr6	92091674	92168865	2.45389	3.66817	2.87804	1.20923	1.33911	2.28642	1.21578	0.977445	1.81961
XLOC_081680	THAP6;CDKL2	chr6	92091674	92168865	2.73644	2.45597	3.42585	3.14041	1.5444	2.7312	1.59758	1.42887	1.91452
XLOC_081681	THAP6;CDKL2	chr6	92091674	92168865	0.836635	1.50132	0.0	1.12483	0.915976	1.56284	1.44227	0.418558	1.90032
XLOC_081682	THAP6;CDKL2	chr6	92091674	92168865	0.0	0.0	4.85485	0.834676	0.716778	0.955158	1.35448	0.302851	0.269559
XLOC_081683	THAP6;CDKL2	chr6	92091674	92168865	8.9914	2.68097	0.0	1.60837	5.81458	6.3976	2.68035	0.862579	4.65662
XLOC_081684	THAP6;CDKL2	chr6	92091674	92168865	2.74483	1.0945	2.86253	0.328029	0.286028	1.04398	1.16054	0.274382	1.27855
XLOC_081685	THAP6;CDKL2	chr6	92091674	92168865	0.0	2.36768	1.23843	0.283857	0.369505	1.30939	0.992604	0.628383	0.276096

XLOC_081686	THAP6;CDKL2	chr6	92091674	92168865	0.0	8.9388	14.0224	2.14924	3.61963	2.42506	2.5028	2.27073	3.09738
XLOC_081687	THAP6;CDKL2	chr6	92091674	92168865	2.09361	1.25077	0.0	0.374868	0.64834	0.215605	0.556944	0.0	1.63859
XLOC_081688	THAP6;CDKL2	chr6	92091674	92168865	4.28725	1.53867	0.670711	0.537991	1.07271	0.978726	1.011	0.600417	1.79768
XLOC_081689	THAP6;CDKL2	chr6	92091674	92168865	0.0	1.65142	2.15927	1.4847	0.426033	1.41838	2.6656	0.540755	2.63964
XLOC_081690	THAP6;CDKL2	chr6	92091674	92168865	0.900156	0.538401	1.40813	0.645425	0.562866	0.933801	0.489473	0.809973	0.628965
XLOC_081691	THAP6;CDKL2	chr6	92091674	92168865	3.72097	0.834585	5.82068	0.833729	1.09041	1.06133	1.17961	0.650807	1.21861
XLOC_081692	THAP6;CDKL2	chr6	92091674	92168865	0.0	0.0	6.14231	2.83422	2.35801	7.92702	3.22269	2.20735	2.03049
XLOC_081693	THAP6;CDKL2	chr6	92091674	92168865	4.29254	4.70463	4.47416	1.79439	2.11593	1.03563	1.28413	2.2741	3.61722
XLOC_080242	THAP9;SCD5;SEC31A	chr6	99076021	99520637	12.8042	18.8804	22.2451	0.98482	1.24718	1.41024	0.579329	0.473709	0.616739
XLOC_082001	THAP9;SCD5;SEC31A	chr6	99076021	99520637	0.0	1.05048	1.37361	0.0	0.0	0.181378	0.0	0.0	0.0
XLOC_082002	THAP9;SCD5;SEC31A	chr6	99076021	99520637	1.46563	1.75232	1.1457	0.0	0.228082	0.0	0.0	0.43658	0.127749
XLOC_082003	THAP9;SCD5;SEC31A	chr6	99076021	99520637	1.79803	0.0	1.40637	0.0	0.140543	0.0932638	0.0	0.0	0.0785229
XLOC_082004	THAP9;SCD5;SEC31A	chr6	99076021	99520637	0.0	0.0	0.0	0.0	0.152125	0.0	0.0	0.0	0.170781
XLOC_082005	THAP9;SCD5;SEC31A	chr6	99076021	99520637	0.0	2.30221	0.0	0.0	0.1198	0.0	0.137979	0.152828	0.0
XLOC_082006	THAP9;SCD5;SEC31A	chr6	99076021	99520637	0.80946	1.45263	0.633198	0.0725549	0.0	0.16804	0.146962	0.0	0.141448
XLOC_082007	THAP9;SCD5;SEC31A	chr6	99076021	99520637	1.816	0.633996	0.947549	0.0814307	0.0	0.0945468	0.0	0.0	0.0
XLOC_082008	THAP9;SCD5;SEC31A	chr6	99076021	99520637	2.35402	2.46337	11.0443	0.0	0.367183	0.0	0.106123	0.117282	0.0
XLOC_082009	THAP9;SCD5;SEC31A	chr6	99076021	99520637	1.98018	4.44117	6.96915	0.0	0.0	0.0	0.0895783	0.0	0.172898
XLOC_082010	THAP9;SCD5;SEC31A	chr6	99076021	99520637	2.08151	7.46149	3.25215	0.0	0.0	0.0	0.0	0.0	0.181025
XLOC_082011	THAP9;SCD5;SEC31A	chr6	99076021	99520637	5.1569	0.77116	4.70605	0.0	0.0	0.267554	0.0	0.0	0.0750809
XLOC_082012	THAP9;SCD5;SEC31A	chr6	99076021	99520637	0.0	4.23919	2.77121	0.0	0.0	0.0	0.0	0.0	0.0
XLOC_082013	THAP9;SCD5;SEC31A	chr6	99076021	99520637	3.10192	2.16431	3.23453	0.0926572	0.0	0.0	0.0	0.0	0.0902663

XLOC_082014	THAP9;SCD5;SEC31A	chr6	99076021	99520637	3.24093	4.19937	5.06897	0.0	0.0	0.0	0.0	0.0	0.0
XLOC_082015	THAP9;SCD5;SEC31A	chr6	99076021	99520637	0.0	2.73982	2.20485	0.0631606	0.0	0.0	0.0	0.0	0.0615836
XLOC_082016	THAP9;SCD5;SEC31A	chr6	99076021	99520637	4.58129	1.36978	11.6429	0.0	0.0	0.0	0.0	0.0	0.0
XLOC_082017	THAP9;SCD5;SEC31A	chr6	99076021	99520637	3.4052	4.30205	4.14544	0.0678575	0.0296986	0.0393782	0.0	0.0	0.0993292
XLOC_082018	THAP9;SCD5;SEC31A	chr6	99076021	99520637	5.93469	5.76503	5.79888	0.0	0.115428	0.0	0.0	0.0	0.129313
XLOC_082019	THAP9;SCD5;SEC31A	chr6	99076021	99520637	2.91982	3.84337	3.65531	0.0	0.0457616	0.0606994	0.0	0.0	0.0
XLOC_082020	THAP9;SCD5;SEC31A	chr6	99076021	99520637	1.6948	4.56196	1.3257	0.227857	0.0	0.0	0.0	0.0	0.0740285
XLOC_082021	THAP9;SCD5;SEC31A	chr6	99076021	99520637	9.2125	14.6642	16.7764	0.0	0.0	0.0	0.0	0.0	0.0
XLOC_082022	THAP9;SCD5;SEC31A	chr6	99076021	99520637	2.67391	1.59664	2.08763	0.0	0.0	0.0	0.0	0.0	0.0
XLOC_082023	THAP9;SCD5;SEC31A	chr6	99076021	99520637	5.37732	5.97401	5.40846	0.206576	0.0601088	0.0	0.0	0.0	0.134256
XLOC_082024	THAP9;SCD5;SEC31A	chr6	99076021	99520637	7.24459	5.05592	11.3342	0.0	0.125934	0.0	0.146156	0.0805756	0.140663
XLOC_082025	THAP9;SCD5;SEC31A	chr6	99076021	99520637	7.29176	6.31583	5.40618	0.0	0.0602457	0.0798821	0.0351155	0.0386158	0.201501
XLOC_082026	THAP9;SCD5;SEC31A	chr6	99076021	99520637	4.14272	5.62615	7.98108	0.0571567	0.0750774	0.0331787	0.0291938	0.0641809	0.111571
XLOC_082027	THAP9;SCD5;SEC31A	chr6	99076021	99520637	2.12882	4.13762	6.65927	0.0953816	0.0	0.0	0.0961702	0.0	0.092913
XLOC_082028	THAP9;SCD5;SEC31A	chr6	99076021	99520637	5.54378	4.56136	10.1225	0.0828484	0.108715	0.0961133	0.168838	0.0	0.0404155
XLOC_082029	THAP9;SCD5;SEC31A	chr6	99076021	99520637	5.29895	7.91698	20.7046	0.158163	0.137107	0.182254	0.315095	0.0	0.0
XLOC_082030	THAP9;SCD5;SEC31A	chr6	99076021	99520637	9.47623	7.4046	10.9195	0.118038	0.0620433	0.383821	0.0482794	0.132627	0.161313
XLOC_089590	THBS2	chr9	104569879	105019792	0.0	0.0	0.0	0.0536541	0.0	0.0	0.0	0.0	0.0
XLOC_089483	THEMIS	chr9	66713682	66766846	0.777792	0.0775823	0.405851	1.32238	1.31251	3.34963	0.841944	0.793831	1.68995
XLOC_089969	THEMIS	chr9	66777408	66834716	1.33225	1.01497	0.700093	0.392654	0.20419	0.569843	0.372654	0.158186	0.546985
XLOC_089970	THEMIS	chr9	66845306	66919742	40.6223	32.4888	32.1061	10.9548	25.6562	18.332	18.9076	20.4236	19.0443
XLOC_091161	THEMIS	chr9	66775120	66777172	1.14525	0.114227	0.298759	0.0342331	0.269679	0.278115	0.0698629	0.0384125	0.167032
XLOC_091162	THEMIS	chr9	66777408	66834716	29.8904	22.2125	18.4201	12.7452	10.5486	11.0874	15.1843	16.7525	13.0533
XLOC_091163	THEMIS	chr9	66777408	66834716	0.0	0.111982	0.292889	0.0335602	0.0881307	0.116854	0.102748	0.0	0.196508

XLOC_091164	THEMIS	chr9	66843471	66845193	12.4815	3.59609	3.97922	4.51812	5.76555	3.99124	7.60252	5.11159	6.30882
XLOC_092044	THOC2	chrX	7641106	7651955	0.90577	0.0	0.708465	0.162359	0.283183	0.563764	0.0820772	0.271662	0.158222
XLOC_092045	THOC2	chrX	7657379	7667379	0.0	0.333459	4.34287	1.29454	1.60565	1.11048	1.25403	1.73657	1.09312
XLOC_092360	THOC2	chrX	7558832	7605532	9.11823	11.5682	8.41389	8.65183	6.21867	7.56168	6.92365	11.7375	7.5597
XLOC_092361	THOC2	chrX	7668426	7671482	4.14791	3.16912	2.43286	29.8528	26.8734	28.2996	26.4943	29.7119	25.3589
XLOC_092696	THOC2	chrX	7558832	7605532	0.305351	1.00511	1.67292	0.438147	0.263808	0.413329	0.447692	0.338298	0.320738
XLOC_092697	THOC2	chrX	7558832	7605532	0.0	1.73182	0.0	0.0	0.438756	0.587626	0.486062	2.2031	0.500211
XLOC_092698	THOC2	chrX	7558832	7605532	0.0	0.0	0.0	0.701012	0.606895	0.0	0.348024	0.579657	0.681285
XLOC_092699	THOC2	chrX	7558832	7605532	0.0	0.450636	0.392882	0.495188	0.590557	0.417711	0.458388	0.353117	0.263497
XLOC_092700	THOC2	chrX	7652040	7653407	0.0	0.894425	0.0	1.0722	1.26505	0.559345	0.381469	0.360049	0.83657
XLOC_092701	THOC2	chrX	7657379	7667379	1.93659	0.0	1.5131	0.0	0.15012	0.0	0.0	0.0	0.16851
XLOC_092702	THOC2	chrX	7667487	7668191	11.9113	3.5608	5.17364	18.1404	20.9288	18.8972	16.5422	15.9284	19.1594
XLOC_041824	THOC3	chr10	5021987	5204097	2.73926	2.80153	3.89157	0.642704	0.269788	0.338893	0.179133	0.372571	0.277229
XLOC_042252	THOC3	chr10	5021987	5204097	0.0	0.0	0.0	0.17962	0.0	0.0	0.0	0.0	0.174544
XLOC_042253	THOC3	chr10	5021987	5204097	0.0	0.459533	0.0	0.137688	0.0	0.158847	0.0	0.152508	0.0
XLOC_042254	THOC3	chr10	5021987	5204097	0.0	0.0	5.83296	0.0	0.0	0.0	0.0	0.0	0.21623
XLOC_042255	THOC3	chr10	5021987	5204097	0.0	0.0	0.724789	0.08305	0.0	0.0961152	0.0	0.0	0.0
XLOC_042256	THOC3	chr10	5021987	5204097	0.0	0.746335	0.0	0.0	0.0	0.0	0.0	0.0	0.0
XLOC_042257	THOC3	chr10	5021987	5204097	0.0	0.487468	1.27466	0.0	0.253464	0.0	0.0	0.0	0.0
XLOC_042258	THOC3	chr10	5021987	5204097	0.0	1.14363	0.0	0.342784	0.0	0.0	0.0	0.0	0.0
XLOC_042259	THOC3	chr10	5021987	5204097	0.0	0.0	0.0	0.0	0.0	0.451522	0.0	0.0	0.0
XLOC_060299	THOC5	chr17	70844139	70854498	41.4219	46.4822	50.8572	120.283	108.844	104.856	123.012	147.201	110.831
XLOC_061866	THOC5	chr17	70837203	70837529	4.28456	1.27769	0.0	1.53259	0.979256	1.30778	2.19648	4.11908	2.22015
XLOC_061867	THOC5	chr17	70838073	70838359	0.0	0.0	0.0	2.00201	4.64901	1.13171	0.0	3.71665	1.926
XLOC_061868	THOC5	chr17	70838573	70839000	0.0	0.0	2.09535	1.44067	2.48193	1.10155	1.41283	3.6765	2.09622
XLOC_061869	THOC5	chr17	70839532	70840008	2.27104	0.678286	0.0	1.82928	1.93137	0.467213	1.40507	0.446614	0.986869
XLOC_061870	THOC5	chr17	70840103	70840505	2.95796	1.76582	0.0	1.32293	2.95722	1.81821	2.06627	0.865531	0.512933
XLOC_061871	THOC5	chr17	70842551	70842880	4.21033	3.76682	6.56629	0.753006	3.53013	1.28557	1.4406	2.0254	1.81843
XLOC_061872	THOC5	chr17	70843055	70844072	0.841482	1.25837	0.658225	1.73472	2.6322	1.74653	1.22148	1.2629	1.39675
XLOC_061873	THOC5	chr17	70844139	70854498	5.1922	4.96828	8.12112	7.25836	5.1079	4.73586	6.00753	6.21887	4.98594
XLOC_061874	THOC5	chr17	70844139	70854498	6.6325	8.29728	6.60464	8.59498	7.0391	8.58519	6.86811	8.8337	7.17
XLOC_061875	THOC5	chr17	70844139	70854498	0.0	2.29334	2.24923	4.725	3.29528	2.38579	4.5993	4.78866	2.17642
XLOC_061876	THOC5	chr17	70855946	70856298	7.43166	1.10847	2.89847	5.98091	1.42092	3.79112	3.20351	5.03118	2.57128

XLOC_061877	THOC5	chr17	70856866	70857632	1.18862	1.06613	2.78827	1.59747	1.01961	1.7232	1.39291	1.77631	1.3482
XLOC_061878	THOC5	chr17	70863886	70864618	0.0	0.376384	0.98436	1.01514	0.784985	1.17282	0.340026	1.37854	1.208
XLOC_061879	THOC5	chr17	70864811	70865928	0.0	0.676387	0.589675	2.0946	1.88761	2.19149	1.43843	0.981493	1.44919
XLOC_070501	THRAP3	chr3	110135335	110142953	0.0	0.365578	0.0	0.0	0.0	0.0	0.0	0.0	0.0
XLOC_041466	THSD4	chr10	18318749	18534133	13.9087	10.5902	13.5357	7.65506	8.59262	7.4084	7.63858	7.56953	6.79661
XLOC_041857	THSD4	chr10	18540128	18546021	1.71028	1.40702	1.19101	1.01183	1.08064	1.42425	1.07755	1.11568	0.97266
XLOC_042386	THSD4	chr10	18318749	18534133	2.32185	2.5464	4.84354	1.24873	1.63502	2.08914	1.12485	1.0075	1.21733
XLOC_042387	THSD4	chr10	18318749	18534133	2.61598	0.781097	0.0	0.936195	1.2102	0.80558	2.29789	0.768501	2.72488
XLOC_042388	THSD4	chr10	18318749	18534133	10.1696	0.759192	13.8965	1.13742	1.17685	0.783278	0.670865	0.747549	1.32448
XLOC_042389	THSD4	chr10	18318749	18534133	1.62726	2.91784	6.35902	1.31156	1.89676	1.17632	0.873057	0.806264	1.134
XLOC_042390	THSD4	chr10	18318749	18534133	1.13627	1.81329	3.26056	0.611357	1.01081	0.867226	1.07443	0.762244	0.795464
XLOC_042391	THSD4	chr10	18318749	18534133	2.96871	1.10999	5.22547	0.997927	0.755109	0.770683	0.944397	0.37174	0.908052
XLOC_042392	THSD4	chr10	18318749	18534133	2.21695	1.6572	0.866859	0.794594	0.951466	1.26326	0.900618	0.7738	1.06416
XLOC_042393	THSD4	chr10	18318749	18534133	7.88321	1.17835	4.10893	1.17704	0.921207	0.815695	2.0088	1.30698	1.48958
XLOC_042394	THSD4	chr10	18318749	18534133	0.0	1.14354	2.99009	0.342784	0.0	0.0	0.659725	1.11082	0.0
XLOC_042395	THSD4	chr10	18318749	18534133	0.0	1.30634	2.27762	0.782927	0.34008	0.602413	0.26135	0.289325	0.253962
XLOC_042396	THSD4	chr10	18318749	18534133	0.0	1.94257	4.3549	0.249501	0.436526	0.48241	0.508446	0.0932342	0.243424
XLOC_042397	THSD4	chr10	18318749	18534133	3.50093	0.0	2.73136	0.626126	0.0	0.0	0.0	0.339374	0.0
XLOC_042398	THSD4	chr10	18318749	18534133	0.0	0.0	2.25495	0.516755	0.224486	0.0	0.517625	0.429736	0.251443
XLOC_042399	THSD4	chr10	18318749	18534133	1.48983	0.445318	2.32915	0.53376	0.231796	0.153984	0.133556	0.147881	0.51937
XLOC_042400	THSD4	chr10	18318749	18534133	0.0	0.0	4.30218	0.0	0.424445	0.0	0.965773	0.26938	0.956246
XLOC_042401	THSD4	chr10	18318749	18534133	2.15101	0.643198	1.68217	0.192747	0.755518	0.445796	0.0971521	0.64379	0.0938762
XLOC_042402	THSD4	chr10	18318749	18534133	2.17691	0.976391	3.40476	0.195065	0.679587	0.225564	0.393191	0.0	0.0
XLOC_042403	THSD4	chr10	18318749	18534133	0.0	1.50931	0.0	0.180922	0.0788329	0.627884	0.182593	0.10079	0.176264
XLOC_042404	THSD4	chr10	18318749	18534133	0.0	2.13006	2.78494	0.0	0.0	0.364481	0.0	0.0	0.308905
XLOC_042405	THSD4	chr10	18318749	18534133	0.0	0.403789	0.0	0.483988	0.631156	0.279451	0.242786	0.134299	0.235555
XLOC_042406	THSD4	chr10	18318749	18534133	0.0	0.714127	1.86748	0.0	0.369383	0.0	0.421773	0.46956	0.0
XLOC_042407	THSD4	chr10	18318749	18534133	2.47294	1.47691	1.93116	0.0	0.381765	0.0	0.0	0.0	0.0
XLOC_042408	THSD4	chr10	18318749	18534133	1.52185	1.36559	2.38105	0.136414	0.0595431	0.237015	0.345682	0.152415	0.0664941
XLOC_042409	THSD4	chr10	18318749	18534133	0.0	2.12679	1.39054	0.477986	0.138105	0.0	0.0	0.0	0.929499
XLOC_042410	THSD4	chr10	18318749	18534133	0.0	0.0	0.0	0.0	0.470851	0.0	0.0	0.0	0.537692
XLOC_042411	THSD4	chr10	18318749	18534133	1.19061	0.355984	3.72385	1.06673	0.928434	1.47944	1.07319	0.355838	0.727145
XLOC_042412	THSD4	chr10	18318749	18534133	0.9608	0.862385	1.87961	0.344595	0.715862	0.449696	0.219384	0.289676	0.588326

XLOC_042413	THSD4	chr10	18318749	18534133	2.16896	0.647884	1.69428	0.776524	0.67127	0.0	0.768195	0.42705	0.188565
XLOC_042414	THSD4	chr10	18318749	18534133	0.0	2.03045	0.0	0.0	0.260792	1.0431	0.589694	0.990556	1.47287
XLOC_042415	THSD4	chr10	18318749	18534133	2.13692	0.0	0.0	0.191269	0.496141	0.439968	1.13593	0.0	0.371602
XLOC_042416	THSD4	chr10	18318749	18534133	1.20851	0.361331	0.94498	0.108275	0.376905	0.500507	0.217796	0.36111	0.210866
XLOC_042417	THSD4	chr10	18318749	18534133	1.49976	0.672898	3.51975	0.739395	0.58684	0.545046	0.40888	0.225335	0.458721
XLOC_042418	THSD4	chr10	18318749	18534133	2.11814	1.26543	4.96384	0.189593	0.163952	0.0	0.563165	0.0	0.552543
XLOC_042419	THSD4	chr10	18318749	18534133	1.11361	0.998917	0.870859	0.598695	0.173786	0.576843	0.201044	0.555232	0.583124
XLOC_042420	THSD4	chr10	18318749	18534133	0.0	0.865677	2.26396	0.389116	0.450748	0.299414	0.0	0.719044	0.126222
XLOC_042421	THSD4	chr10	18318749	18534133	1.12225	1.67775	2.63274	0.201111	0.350244	0.232515	0.202571	0.0	0.391753
XLOC_042422	THSD4	chr10	18318749	18534133	2.06125	0.92503	2.41936	1.01647	0.565654	0.750204	0.705525	0.362361	0.721148
XLOC_072551	THSD7A	chr4	19575078	19608052	5.4518	5.45	5.4051	1.80871	2.22326	2.67301	2.0446	1.71415	4.37926
XLOC_073091	THSD7A	chr4	19522376	19570112	19.2901	13.9625	12.9136	14.003	14.7785	12.0667	16.4877	12.8083	19.0598
XLOC_073092	THSD7A	chr4	19609467	19678436	23.2543	22.9984	24.1431	26.5498	35.0193	27.8705	26.718	26.831	34.0005
XLOC_073800	THSD7A	chr4	19519250	19520325	4.7297	7.54483	9.86636	7.06584	10.2991	10.2291	9.73579	7.26085	13.2937
XLOC_073801	THSD7A	chr4	19521493	19522245	12.1661	5.81977	8.56155	10.1373	10.2442	10.7068	16.2228	9.32994	16.8762
XLOC_073802	THSD7A	chr4	19575078	19608052	6.6462	3.30851	0.0	1.78456	1.88488	2.73552	0.784007	0.435974	3.65894
XLOC_073803	THSD7A	chr4	19608572	19609242	4.23387	6.74975	3.30984	9.48147	13.6227	9.92407	12.9234	8.69384	13.9031
XLOC_073804	THSD7A	chr4	19694414	19695086	5.62322	0.840443	0.0	0.629645	0.218877	0.581519	0.252441	0.419062	0.490242
XLOC_073805	THSD7A	chr4	19698762	19700078	1.24858	0.747022	2.44223	0.839526	0.440153	1.10287	0.398068	0.876816	0.327494
XLOC_073806	THSD7A	chr4	19705237	19706007	2.36171	0.353058	1.84672	0.846426	0.460463	0.244575	0.425849	0.352975	0.515167
XLOC_073807	THSD7A	chr4	19707136	19708528	1.75757	0.876322	2.29196	0.840396	0.780455	1.2788	0.373828	1.05843	1.07581
XLOC_073808	THSD7A	chr4	19708838	19708967	0.0	0.0	0.0	84.8193	11.286	49.2297	11.7282	13.5545	28.6236
XLOC_044683	THUMPD2;TMEM178	chr11	22005563	22287055	11.9145	8.9298	9.54493	12.5235	12.1635	11.2137	12.9135	12.504	12.0022
XLOC_041820	TICAM2	chr10	4374270	4387592	2.13686	1.52404	2.98946	3.91273	2.16526	1.92871	1.2457	2.41213	1.01322
XLOC_042223	TICAM2	chr10	4374270	4387592	0.883636	1.58563	1.38234	0.871172	0.483546	1.00845	0.881147	0.441826	1.15776
XLOC_042224	TICAM2	chr10	4374270	4387592	2.72423	4.87986	6.38044	1.94975	1.25926	1.6768	1.19424	0.799305	0.945549
XLOC_042225	TICAM2	chr10	4374270	4387592	0.0	2.37088	4.42901	0.405999	0.618634	1.64279	1.32882	1.0164	0.19771
XLOC_042226	TICAM2	chr10	4374270	4387592	0.0	2.31807	2.02062	1.85235	0.598738	1.32845	0.909747	0.0	0.673976
XLOC_042227	TICAM2	chr10	4374270	4387592	0.0	3.90401	0.0	2.33977	1.44776	0.962418	1.82809	1.10663	1.78708
XLOC_042228	TICAM2	chr10	4388242	4389547	15.1266	11.6898	14.7938	4.9724	6.02342	4.71559	6.37203	4.23611	5.84106
XLOC_042229	TICAM2	chr10	4389649	4390680	10.7639	12.6298	13.6014	3.63652	4.33873	4.12484	6.08614	4.72288	4.91901
XLOC_042230	TICAM2	chr10	4390969	4391204	53.8328	37.4948	35.0059	9.71269	10.7038	8.11241	13.0931	10.8269	6.93559

XLOC_071921	TIE1	chr3	103278825	103279658	3.21214	0.640323	0.837333	1.53513	1.33723	0.887658	1.06401	0.53414	0.467304
XLOC_071922	TIE1	chr3	103279877	103280156	11.8038	0.0	0.0	7.40109	1.33644	2.38719	0.493155	2.23638	1.52429
XLOC_070089	TIE1;TMEM125;<br>C3H1orf210	chr3	103280238	103357161	28.0711	30.9402	27.5759	32.1307	27.6632	30.0777	34.4452	36.3221	30.6687
XLOC_085029	TIMD4	chr7	70340940	70341791	0.0	0.31186	0.0	0.0	0.0	0.0	0.0	0.0	0.0
XLOC_085031	TIMD4	chr7	70342744	70343094	0.0	1.1199	0.0	0.0	0.0	0.0	0.0	0.0	0.0
XLOC_085032	TIMD4	chr7	70344082	70345027	0.918423	0.274665	0.0	0.0	0.0	0.0	0.0	0.0	0.0
XLOC_085033	TIMD4	chr7	70345105	70346704	0.0	0.150108	0.0	0.0	0.0	0.0	0.0	0.0	0.0
XLOC_085034	TIMD4	chr7	70346757	70350957	0.178903	0.053542	0.0	0.0	0.0	0.0	0.0164325	0.0	0.0
XLOC_040089	TIPARP	chr1	111855352	111856765	0.0	0.689337	0.45073	0.103293	0.180579	0.239522	0.367642	0.0	0.151122
XLOC_042346	TIPIN	chr10	13208335	13209343	3.40155	2.03468	4.6563	0.0762201	0.0	0.0882459	0.0771376	0.255225	0.371443
XLOC_042347	TIPIN	chr10	13209970	13212241	3.42193	1.84311	1.07125	0.030687	0.188076	0.14249	0.0940047	0.034449	0.419287
XLOC_086392	TJP2	chr8	45627730	45826999	7.32988	11.6822	8.95798	0.321673	0.158591	0.403322	0.178676	0.103324	0.202897
XLOC_087725	TJP2	chr8	45627730	45826999	0.0	0.0	0.0	0.289352	0.0	0.0	0.0	0.0	0.0
XLOC_087727	TJP2	chr8	45627730	45826999	0.0	0.563524	0.0	0.0	0.0	0.0	0.0	0.0	0.0
XLOC_087728	TJP2	chr8	45627730	45826999	3.71583	1.10848	0.0	0.0	0.0	0.0	0.0	0.0	0.0
XLOC_087729	TJP2	chr8	45627730	45826999	0.0	3.31938	0.0	0.0	0.0	0.0	0.0	0.0	0.0
XLOC_087730	TJP2	chr8	45627730	45826999	0.586807	1.93104	1.83654	0.0526099	0.0	0.121988	0.0	0.0	0.0
XLOC_087731	TJP2	chr8	45627730	45826999	3.09709	2.77299	2.41706	0.0	0.0	0.0	0.0	0.0	0.0
XLOC_087732	TJP2	chr8	45627730	45826999	1.48893	0.890718	2.32959	0.0	0.0582636	0.231916	0.0	0.0	0.0
XLOC_087733	TJP2	chr8	45627730	45826999	0.851388	1.27317	1.9979	0.0763098	0.0	0.0	0.0	0.0	0.0
XLOC_087734	TJP2	chr8	45627730	45826999	1.88472	0.0	0.737054	0.0	0.0	0.0977339	0.0	0.0	0.0
XLOC_087735	TJP2	chr8	45627730	45826999	0.0	0.0	2.8692	0.0	0.0	0.0	0.0	0.0	0.0
XLOC_087737	TJP2	chr8	45627730	45826999	0.848392	0.761219	0.0	0.0	0.0663416	0.0	0.0	0.0	0.0
XLOC_087738	TJP2	chr8	45627730	45826999	0.0	0.863372	0.967766	0.0	0.0	0.0	0.0	0.0	0.0
XLOC_087739	TJP2	chr8	45627730	45826999	0.0	0.671975	0.0	0.0	0.0	0.0	0.0	0.0	0.0
XLOC_050962	TKDP1	chr13	74693432	74798996	13.2062	9.59264	10.2016	23.6118	22.5345	20.7126	20.4321	24.8962	24.8195
XLOC_052527	TKDP1	chr13	74802448	74803613	0.0	0.214728	0.0	0.257404	0.168581	0.0	0.0652767	0.0719341	0.188226
XLOC_052528	TKDP1	chr13	74806775	74808809	0.385413	0.345968	0.603247	1.00228	0.998278	0.561553	0.634754	1.04704	0.876886
XLOC_064038	TLK2	chr19	47580115	47643503	45.242	51.0238	41.8656	23.2967	24.8551	25.405	26.5633	30.1414	32.9334
XLOC_064307	TLK2	chr19	47645581	47661112	2.45088	1.64567	1.74075	0.777697	1.04441	0.841806	0.342133	0.340066	0.725434
XLOC_064308	TLK2	chr19	47667932	47668820	0.0	0.618239	0.0	0.370523	0.320521	0.213148	0.183585	0.0	0.359992

XLOC_065276	TLK2	chr19	47579636	47579864	9.75032	14.5608	0.0	3.52609	3.62644	4.89304	0.0	2.70429	4.18663
XLOC_065277	TLK2	chr19	47643622	47645364	5.02086	4.09699	7.14367	7.28513	9.16616	9.02158	7.79933	7.80057	11.101
XLOC_086654	TLR4	chr8	108794604	108948440	3.82661	7.81342	9.04579	5.05906	2.8013	3.60134	1.7999	2.22716	1.07218
XLOC_070334	TM2D1	chr3	84495455	84582930	9.88315	12.1722	11.951	12.2627	10.8069	11.5404	11.4445	13.8508	15.151
XLOC_038507	TM4SF1;TM4SF18;TM4SF18	chr1	119652275	119837394	14.404	20.0322	17.8991	9.09426	9.3069	8.01697	11.1454	10.0096	12.4998
XLOC_040156	TM4SF1;TM4SF18;TM4SF18	chr1	119652275	119837394	0.0	1.85367	1.21191	1.11094	1.20561	0.961282	0.833048	1.07662	1.89142
XLOC_040157	TM4SF1;TM4SF18;TM4SF18	chr1	119652275	119837394	3.98024	3.56164	9.31251	1.77958	1.51928	2.83913	3.07526	1.1518	1.03207
XLOC_040158	TM4SF1;TM4SF18;TM4SF18	chr1	119652275	119837394	3.68774	0.551475	0.721098	0.495763	0.864603	1.33885	1.67034	0.368661	1.28829
XLOC_040159	TM4SF1;TM4SF18;TM4SF18	chr1	119652275	119837394	2.934	2.62747	9.16048	3.67425	3.38516	4.50951	1.53779	0.858921	3.56172
XLOC_040160	TM4SF1;TM4SF18;TM4SF18	chr1	119652275	119837394	4.80572	2.01205	6.7656	1.03365	1.72708	2.49183	1.30509	1.15235	1.42681
XLOC_040161	TM4SF1;TM4SF18;TM4SF18	chr1	119652275	119837394	3.40671	4.07625	9.59491	0.794034	1.76057	1.91106	2.23193	0.956312	1.78674
XLOC_063951	TM4SF5;CXCL16	chr19	27216840	27249952	2.43993	1.12284	1.08913	1.22886	4.8491	2.87364	3.71174	0.339785	3.25858
XLOC_092075	TM9SF2	chrX	20550477	20562038	0.0	0.361655	0.0	0.0	0.0	0.0	0.0550811	0.0	0.0
XLOC_092818	TM9SF2	chrX	20521891	20522318	0.0	0.0	0.0	0.240112	2.2751	1.10155	0.0	0.525215	0.232913
XLOC_092819	TM9SF2	chrX	20528317	20528454	0.0	30.4294	79.7296	59.7743	27.4912	29.5999	92.7931	24.8225	42.892
XLOC_092074	TM9SF2;MIR2484;RBMX;ARHGEF6	chrX	20364548	20520076	40.3594	41.6985	33.3887	48.6623	39.8093	48.4309	40.715	31.3061	42.6252
XLOC_092817	TM9SF2;MIR2484;RBMX;ARHGEF6	chrX	20364548	20520076	4.51596	3.9184	2.4738	7.65326	6.80191	9.63077	7.79977	7.35502	11.1414
XLOC_076254	TMBIM6;FMNL3	chr5	30281419	30366518	0.343656	0.259696	0.0	0.277708	0.782493	0.500847	0.472026	0.484343	0.495617
XLOC_050854	TMC2;SNRPB;TGM3	chr13	53109257	53286364	1.45319	0.730215	1.30004	6.25087	6.57008	6.35642	9.91058	11.5425	7.91758
XLOC_051844	TMC2;SNRPB;TGM3	chr13	53109257	53286364	0.736881	0.220412	0.0	1.38714	0.865102	1.30086	1.5406	1.18116	0.708395

XLOC_051845	TMC2;SNRPB;TGM3	chr13	53109257	53286364	0.0	0.0	0.0	5.13173	3.01746	3.40169	2.37049	5.592	2.6176
XLOC_051846	TMC2;SNRPB;TGM3	chr13	53109257	53286364	0.0	0.0	0.0	8.81417	3.95705	4.53445	3.44974	5.91294	2.44874
XLOC_051847	TMC2;SNRPB;TGM3	chr13	53109257	53286364	0.0	0.0	0.634618	7.0536	2.7287	3.19989	2.2093	4.22246	2.19734
XLOC_051848	TMC2;SNRPB;TGM3	chr13	53109257	53286364	0.0	0.0	0.0	4.57681	3.60781	4.01249	1.69114	2.27927	3.40341
XLOC_051849	TMC2;SNRPB;TGM3	chr13	53109257	53286364	0.0	0.0	0.0	3.19599	1.64808	1.68228	1.25055	2.59556	2.12408
XLOC_051850	TMC2;SNRPB;TGM3	chr13	53109257	53286364	0.0	0.134529	0.0	2.29808	1.16401	1.16939	1.15054	1.71778	1.33745
XLOC_051851	TMC2;SNRPB;TGM3	chr13	53109257	53286364	1.7841	0.266902	0.349037	1.11984	0.769806	1.06723	0.611458	0.582977	0.702393
XLOC_051852	TMC2;SNRPB;TGM3	chr13	53109257	53286364	0.0	0.195276	0.0	0.848583	0.563698	0.645439	0.717304	0.78851	0.628298
XLOC_051853	TMC2;SNRPB;TGM3	chr13	53109257	53286364	0.0	0.0	0.483442	1.49566	0.726104	0.963184	0.281453	0.619925	0.486215
XLOC_076232	TMCC3	chr5	24452507	24502092	24.2129	32.274	20.0372	14.3632	14.2232	16.464	14.6623	17.4672	11.356
XLOC_077425	TMCC3	chr5	24502440	24505343	0.526841	0.472978	0.206179	0.448872	0.765757	0.493843	0.289887	0.318536	0.507352
XLOC_070101	TMCO2;RLF	chr3	106482376	106501764	1.14532	3.08933	1.55046	0.524906	0.734946	0.827068	0.191272	0.240388	0.695424
XLOC_072022	TMCO2;RLF	chr3	106482376	106501764	0.0	0.0	0.0	0.0	0.0773339	0.0	0.179157	0.0	0.0
XLOC_041703	TMED10	chr10	86775924	86776909	0.0	1.77162	1.54448	0.0	0.539871	0.307126	0.178676	0.0986151	0.603496
XLOC_041704	TMED10	chr10	86779188	86835222	24.5662	16.9168	18.3006	1.77844	1.51173	1.9417	2.37229	2.74284	2.74632
XLOC_041822	TMED7	chr10	4407841	4422787	11.5027	16.6803	24.0666	13.4649	16.8223	14.6389	11.9122	11.3036	15.5669
XLOC_042242	TMED7	chr10	4407841	4422787	0.0	1.72368	0.0	1.29134	0.222155	2.95907	1.26208	0.845516	0.500791
XLOC_042243	TMED7	chr10	4407841	4422787	2.01202	3.00537	3.14381	3.24213	4.83207	4.35312	2.32263	3.37376	2.80041
XLOC_043295	TMED8	chr10	89638720	89639301	3.41584	2.04151	0.0	2.29415	2.38745	3.17326	3.20252	2.87442	2.82598
XLOC_041714	TMED8;C10H14orf133	chr10	89639385	89689412	0.0	0.0	0.0	0.0	0.198921	0.0	0.0	0.0	0.0
XLOC_042110	TMED8;C10H14orf133	chr10	89639385	89689412	22.9615	26.4758	24.4974	16.8027	15.7991	16.7157	16.4547	15.8951	17.1102
XLOC_068033	TMEFF2	chr2	81006673	81007623	0.0	0.0	0.0	1.55408	1.14124	1.79869	0.909569	0.912321	1.43474
XLOC_068034	TMEFF2	chr2	81008583	81009793	0.0	0.0	0.0	0.739216	0.107609	0.499667	0.50017	0.206653	0.300329
XLOC_063869	TMEM100	chr19	6241656	6262796	17.9752	22.858	18.9672	16.4532	10.895	12.3053	11.8675	22.64	11.0089
XLOC_073093	TMEM106B	chr4	19877403	19922621	1.18271	0.943696	0.0	0.0707046	0.0309413	0.0	0.0	0.0	0.0689949
XLOC_076277	TMEM106C;COL2A1;COL2A1	chr5	32459174	32516448	92.8853	101.748	88.6934	148.154	146.118	148.193	176.309	168.535	159.863

XLOC_077614	TMEM106C;COL2A1;COL2A1	chr5	32459174	32516448	5.15726	2.30981	0.0	1.61504	1.98882	2.11806	5.21325	1.51576	1.11933
XLOC_077615	TMEM106C;COL2A1;COL2A1	chr5	32459174	32516448	0.0	3.97981	3.90304	1.3417	1.42255	2.40624	2.23135	0.989336	1.59492
XLOC_077616	TMEM106C;COL2A1;COL2A1	chr5	32459174	32516448	5.82537	5.22119	1.51715	2.43382	3.16086	3.80271	1.72647	1.15015	1.85855
XLOC_077617	TMEM106C;COL2A1;COL2A1	chr5	32459174	32516448	0.0	3.76928	5.91417	1.3554	3.50641	2.07439	3.33196	1.48498	1.97298
XLOC_077618	TMEM106C;COL2A1;COL2A1	chr5	32459174	32516448	2.08151	0.62179	1.62608	1.11795	1.93401	1.71487	0.184602	0.410276	0.543073
XLOC_077619	TMEM106C;COL2A1;COL2A1	chr5	32459174	32516448	3.11976	1.24385	3.25311	0.559137	1.2179	1.07787	1.41	0.62277	1.36175
XLOC_077620	TMEM106C;COL2A1;COL2A1	chr5	32459174	32516448	1.85227	2.21375	4.34203	0.66338	1.43681	0.955108	0.329912	0.54916	2.09589
XLOC_077621	TMEM106C;COL2A1;COL2A1	chr5	32459174	32516448	1.17457	1.40553	1.83804	0.579179	0.96643	0.915647	0.428261	0.176832	0.821657
XLOC_077622	TMEM106C;COL2A1;COL2A1	chr5	32459174	32516448	1.50216	2.6939	3.52262	0.672735	1.51904	0.465755	0.269268	0.596348	0.523662
XLOC_076300	TMEM117	chr5	36464959	36471751	38.6333	43.5262	40.3624	92.2425	82.3105	92.3227	94.1063	123.174	106.532
XLOC_076822	TMEM117	chr5	36153828	36397917	12.1737	2.42449	4.75532	0.726524	0.314328	1.04507	0.360224	0.0	0.705949
XLOC_076823	TMEM117	chr5	36474409	36484081	11.2517	7.27012	12.4178	3.90711	4.18586	3.66842	4.09709	4.33546	3.39558
XLOC_076824	TMEM117	chr5	36490499	36593265	5.58019	8.46085	4.44165	6.79753	8.5814	9.63842	8.28239	6.67991	4.37139
XLOC_076825	TMEM117	chr5	36600401	36673274	18.0198	17.1791	17.6457	7.67009	10.9373	8.32462	10.4364	8.5593	10.4162
XLOC_076826	TMEM117	chr5	36794033	36796309	60.6415	53.6176	45.324	83.4149	73.5597	78.6032	95.7762	117.373	100.755
XLOC_076827	TMEM117	chr5	36806967	36830640	6.12042	5.14872	4.00122	4.64749	4.38421	4.01811	3.64837	3.67383	4.65604
XLOC_077729	TMEM117	chr5	36153828	36397917	0.0	0.0	0.0	0.449173	0.156582	0.0	0.18135	0.0	0.875235
XLOC_077730	TMEM117	chr5	36153828	36397917	0.0	0.0	0.0	1.25531	0.273185	0.0	0.42114	0.116348	0.203748
XLOC_077731	TMEM117	chr5	36153828	36397917	0.0	0.0	0.0	3.28157	1.4292	1.33937	2.04316	3.22368	2.91448
XLOC_077732	TMEM117	chr5	36153828	36397917	0.0	0.0	0.0	0.468098	0.6051	0.268527	2.29789	0.0	0.454146
XLOC_077733	TMEM117	chr5	36472188	36473056	3.05389	4.87047	2.38838	4.01386	3.73618	4.74865	3.3139	1.93098	4.26593
XLOC_077734	TMEM117	chr5	36473146	36474248	9.95473	7.78738	8.98549	4.32432	4.79352	5.0883	5.07855	4.2178	4.61716

XLOC_077735	TMEM117	chr5	36474409	36484081	1.63104	3.89918	1.27466	0.584229	0.506928	0.842107	0.583311	1.13126	0.710337
XLOC_077736	TMEM117	chr5	36485089	36486803	0.929306	0.834131	0.363606	0.541627	0.692493	0.628338	0.254709	0.560477	0.121946
XLOC_077737	TMEM117	chr5	36487159	36488166	0.0	0.509269	0.0	0.0	0.266297	0.176698	0.077227	0.170348	0.0743758
XLOC_077738	TMEM117	chr5	36490499	36593265	0.0	3.11062	1.16218	1.99749	1.041	1.8441	1.5995	1.62341	0.259159
XLOC_077739	TMEM117	chr5	36600401	36673274	45.8014	15.0219	39.2784	2.45798	4.52761	2.32693	1.5584	1.75611	7.90531
XLOC_077740	TMEM117	chr5	36600401	36673274	10.2484	12.2208	35.9469	0.458698	3.88388	4.15624	0.865041	2.44335	3.9754
XLOC_077741	TMEM117	chr5	36600401	36673274	8.2772	18.514	6.4547	9.25222	10.7305	11.7983	13.8219	9.96182	9.29648
XLOC_077742	TMEM117	chr5	36693168	36693739	0.0	0.0	1.36698	0.313272	0.407398	0.18051	0.312144	0.173087	0.15231
XLOC_077743	TMEM117	chr5	36694788	36695492	5.29392	4.35209	5.17364	0.829952	1.03098	0.821617	1.66612	0.263279	0.57709
XLOC_077744	TMEM117	chr5	36745725	36746088	3.51781	0.0	0.0	0.629158	0.808254	0.0	3.04267	0.0	1.21785
XLOC_077745	TMEM117	chr5	36747087	36748680	8.06113	7.53674	5.12512	5.3305	4.38525	5.08233	4.9676	5.66934	5.02385
XLOC_077746	TMEM117	chr5	36790935	36791755	0.0	0.979314	0.0	0.195653	0.0852026	0.22624	0.295765	0.0	0.0952877
XLOC_077747	TMEM117	chr5	36791921	36792887	0.894566	0.535074	0.69971	0.320705	0.279705	0.649634	0.405394	0.0894463	0.234406
XLOC_077748	TMEM117	chr5	36796457	36796955	53.1077	60.2821	48.1236	132.723	101.286	111.541	93.9375	155.052	127.825
XLOC_077749	TMEM117	chr5	36806452	36806878	0.0	0.0	0.0	0.964013	0.415163	0.276396	0.0	0.0	0.233772
XLOC_062236	TMEM145;PRR19;CEACAM1;LIPE;CNFN	chr18	51117803	51305122	0.547584	0.819325	0.428588	0.0	0.0	0.0	0.0	0.0275863	0.0
XLOC_063202	TMEM145;PRR19;CEACAM1;LIPE;CNFN	chr18	51117803	51305122	0.0	0.0	0.0	0.233208	0.0	0.0	0.0	0.0	0.0
XLOC_063203	TMEM145;PRR19;CEACAM1;LIPE;CNFN	chr18	51117803	51305122	0.0	0.0	1.0876	0.0	0.0	0.0722007	0.0	0.0	0.0
XLOC_063204	TMEM145;PRR19;CEACAM1;LIPE;CNFN	chr18	51117803	51305122	1.78374	0.0	0.0	0.0	0.0	0.0	0.0	0.0	0.0
XLOC_083102	TMEM161B	chr7	90130203	90175037	38.087	37.8278	35.6092	29.098	25.1306	28.3414	39.5672	36.8558	27.7862
XLOC_083103	TMEM161B	chr7	90175325	90208958	75.1254	64.8836	55.6791	57.8729	47.501	55.1766	67.1091	74.0604	53.7245
XLOC_083751	TMEM161B	chr7	90124777	90129928	18.6712	4.17515	7.27789	3.33997	1.06425	3.79293	2.37928	4.91754	5.63848
XLOC_085262	TMEM161B	chr7	90124777	90129928	1.59044	1.18924	0.0	0.356397	0.808733	0.577845	0.144414	0.398049	0.833798
XLOC_066314	TMEM163	chr2	62787937	62877318	0.0	0.0	0.0	0.095102	0.0	0.0	0.0	0.0	0.0
XLOC_067592	TMEM163	chr2	62995976	62996979	0.0	0.0	0.0	0.0	0.0	0.0	0.0	0.0	0.0747266

XLOC_067600	TMEM163	chr2	63098785	63099953	0.0	0.0	0.0	0.0	0.0	0.0	0.0	0.0717224	0.0
XLOC_067601	TMEM163	chr2	63102600	63103500	0.0	0.0	0.0	0.0872962	0.0	0.0	0.0	0.0	0.0
XLOC_079770	TMEM165	chr6	72538274	72562984	16.7852	15.5692	16.8726	22.807	19.5005	24.2318	8.14076	12.7582	13.5238
XLOC_068187	TMEM169	chr2	104589461	104589626	0.0	9.65524	0.0	18.1901	27.4795	9.54266	2.40009	19.5191	0.0
XLOC_068188	TMEM169	chr2	104590543	104592057	7.99745	4.62576	5.42344	6.97927	8.06697	8.70354	9.53511	9.15667	7.9738
XLOC_068189	TMEM169	chr2	104592658	104592955	5.16015	4.61491	0.0	6.46429	5.08342	5.75415	4.78873	2.45969	2.22389
XLOC_068190	TMEM169	chr2	104593469	104593797	4.23479	3.78865	6.60433	7.57385	11.6184	9.91219	9.77684	8.14662	6.94971
XLOC_068191	TMEM169	chr2	104595052	104595264	0.0	14.4675	47.2711	18.7027	14.2976	20.5805	15.366	7.74181	9.34139
XLOC_068192	TMEM169	chr2	104595344	104595823	4.49971	1.34394	7.02915	4.22859	5.2191	7.40661	5.17209	6.19583	3.12875
XLOC_068193	TMEM169	chr2	104596053	104596632	3.43205	3.07677	8.04644	4.91737	3.9977	4.95936	5.82168	4.75635	6.57524
XLOC_068194	TMEM169	chr2	104596809	104597445	4.53475	2.71076	5.90779	4.33246	4.35021	4.68649	7.58548	4.65014	3.42503
XLOC_068195	TMEM169	chr2	104598140	104598559	2.76587	3.30283	2.15923	3.46411	5.96446	5.6734	7.51194	3.51488	4.31939
XLOC_068196	TMEM169	chr2	104598960	104599202	0.0	2.47683	6.47489	9.71765	13.6476	5.84372	10.8273	9.28169	2.85265
XLOC_068197	TMEM169	chr2	104605110	104605840	5.05347	0.755394	0.987794	1.58461	2.26464	2.22301	3.1844	2.01205	2.5346
XLOC_068198	TMEM169	chr2	104607257	104607508	0.0	11.281	0.0	2.71978	5.66848	3.04716	9.93291	4.24838	3.90102
XLOC_068199	TMEM169	chr2	104609215	104610317	0.765748	2.06136	4.19323	2.12784	2.39676	3.3392	3.82631	1.91718	1.94055
XLOC_068200	TMEM169	chr2	104610394	104611694	1.8991	2.65117	4.95282	3.06459	3.17359	2.96009	4.49701	2.3495	2.49052
XLOC_068201	TMEM169	chr2	104612039	104613710	5.73425	2.00158	4.48718	2.18518	2.43611	3.03188	3.84092	2.11313	3.21872
XLOC_068202	TMEM169	chr2	104614669	104615099	2.65429	0.792477	4.14471	0.712426	1.63679	1.36202	3.26203	1.03923	1.61262
XLOC_068203	TMEM169	chr2	104615345	104615588	16.4257	2.45035	0.0	3.69695	7.36724	6.60847	13.4004	4.59387	3.52802
XLOC_068204	TMEM169	chr2	104616726	104616970	0.0	2.42439	6.33787	2.92572	6.07651	1.63494	3.98074	2.27389	2.79278
XLOC_068205	TMEM169	chr2	104617288	104617519	9.40216	0.0	0.0	3.39682	5.60092	5.66432	3.03912	1.74155	4.03738
XLOC_068206	TMEM169	chr2	104618557	104619655	0.0	1.15007	3.00789	1.10291	1.86534	1.75651	2.16569	1.38619	1.47838
XLOC_068207	TMEM169	chr2	104619839	104620160	8.82829	1.31624	3.44165	1.18426	2.01605	1.79539	3.7641	1.69524	1.52436
XLOC_068208	TMEM169	chr2	104620356	104620832	0.0	4.06972	0.0	1.82928	2.28253	0.70082	4.61665	2.67969	2.76323
XLOC_068209	TMEM169	chr2	104621724	104622577	2.07984	1.55482	4.06639	1.58422	1.86745	2.04795	3.76	1.14174	2.1788
XLOC_068210	TMEM169	chr2	104623178	104624358	2.12199	2.96211	1.10674	2.15586	2.5472	1.91012	3.15225	1.48865	1.79289
XLOC_068211	TMEM169	chr2	104628119	104629190	0.791736	1.18404	1.85804	1.20645	1.61043	1.72598	1.29412	1.26823	1.79867
XLOC_065773	TMEM169;XRCC5	chr2	104629711	104665496	11.7522	12.6694	10.1777	19.8043	20.8155	22.6408	23.8937	27.9953	21.8129
XLOC_068212	TMEM169;XRCC5	chr2	104629711	104665496	7.00625	4.17827	10.9235	4.40251	4.20944	4.23837	2.89091	3.29125	2.40971
XLOC_068213	TMEM169;XRCC5	chr2	104629711	104665496	1.86181	3.89396	4.36435	1.83367	2.16614	1.15196	2.65243	1.65578	2.26865

XLOC_068214	TMEM169;XRCC5	chr2	104629711	104665496	1.63458	0.0	5.11013	1.46387	1.90522	2.02558	1.46147	0.809844	0.996706
XLOC_068215	TMEM169;XRCC5	chr2	104629711	104665496	7.1747	3.21074	5.59706	3.20792	2.19686	4.76123	5.88858	1.04231	4.0354
XLOC_068216	TMEM169;XRCC5	chr2	104629711	104665496	0.977012	1.46155	0.764527	1.09503	1.57074	1.16857	0.892218	0.4909	1.49561
XLOC_068217	TMEM169;XRCC5	chr2	104629711	104665496	0.0	1.75866	0.0	0.527023	1.35944	1.2073	0.771861	0.287391	0.766301
XLOC_068218	TMEM169;XRCC5	chr2	104629711	104665496	1.65326	1.48221	3.87633	1.33251	1.28447	1.87779	0.886605	0.327571	1.15203
XLOC_068219	TMEM169;XRCC5	chr2	104629711	104665496	4.21151	0.629018	0.0	2.0734	2.60821	1.95141	1.49336	1.45227	1.0987
XLOC_044124	TMEM17	chr11	60931089	60952772	59.3178	49.0612	54.0664	19.224	14.6286	14.8854	23.3023	25.028	16.6086
XLOC_065690	TMEM177	chr2	71872521	71900682	22.2418	33.1199	28.6091	139.368	127.05	125.095	129.909	123.834	139.439
XLOC_078711	TMEM184B	chr5	110486065	110487176	0.0	0.22688	1.18677	0.747919	0.652925	0.315031	0.275687	1.21552	1.12685
XLOC_086303	TMEM215;NDUFB6;MIR2469;TOPORS;LOC100616098	chr8	11198320	11448389	57.6234	42.8695	56.9899	2.26512	1.69995	1.99047	2.9332	1.76261	3.91894
XLOC_086777	TMEM215;NDUFB6;MIR2469;TOPORS;LOC100616098	chr8	11198320	11448389	158.258	94.8036	137.129	6.49021	4.85923	6.68264	9.82994	5.64813	11.5197
XLOC_041624	TMEM229B	chr10	79689148	79695618	2.37507	1.43891	1.43488	2.31948	3.54795	1.62	2.63576	1.43187	2.35949
XLOC_041625	TMEM229B	chr10	79704465	79711970	115.258	109.182	85.6674	262.453	301.984	277.314	266.533	229.155	324.071
XLOC_043025	TMEM229B	chr10	79712209	79713609	0.0	0.696546	1.36633	1.25248	1.2316	0.968075	0.583702	0.584258	1.018
XLOC_043026	TMEM229B	chr10	79713821	79714187	0.0	0.0	0.0	0.310075	0.531305	0.354225	0.0	0.336255	1.20058
XLOC_066508	TMEM234;DCDC2B;EIF3I;CCDC28B;IQCC	chr2	122130384	122186183	6.11741	1.92323	0.457282	3.06732	2.38936	3.66019	2.25561	0.879902	2.6608
XLOC_068451	TMEM234;DCDC2B;EIF3I;CCDC28B;IQCC	chr2	122130384	122186183	1.0787	0.0	0.84357	0.773284	1.01034	0.558896	0.194871	0.215228	0.0941551
XLOC_068452	TMEM234;DCDC2B;EIF3I;CCDC28B;IQCC	chr2	122130384	122186183	1.51545	1.13321	1.77829	1.22259	1.06731	0.550729	0.413104	0.455348	0.595946

XLOC_068453	TMEM234;DCDC2B;EIF3I;CCDC28B;IQCC	chr2	122130384	122186183	0.0	1.25649	0.0	1.12963	0.574084	1.08876	0.189875	0.104837	0.550217
XLOC_068454	TMEM234;DCDC2B;EIF3I;CCDC28B;IQCC	chr2	122130384	122186183	0.0	1.44042	1.88358	0.971236	1.50264	2.36955	0.976885	0.4799	0.210167
XLOC_068455	TMEM234;DCDC2B;EIF3I;CCDC28B;IQCC	chr2	122130384	122186183	2.39115	1.42816	1.86739	1.49786	0.554074	2.45783	0.843542	0.23478	0.830898
XLOC_068456	TMEM234;DCDC2B;EIF3I;CCDC28B;IQCC	chr2	122130384	122186183	0.0	0.795034	1.03967	0.655219	0.572377	1.72575	0.907184	0.266499	0.406588
XLOC_086572	TMEM246	chr8	92827293	92853482	43.1725	27.9665	17.0992	5.09228	4.67594	4.13897	4.19977	2.93609	5.82389
XLOC_092583	TMEM27	chrX	134944526	134964747	42.7374	70.76	50.7739	0.834628	0.0797982	0.303959	0.248213	0.306987	0.0857909
XLOC_093596	TMEM27;AP1S2	chrX	134972610	134973987	2.96531	3.54838	4.64028	0.159511	0.325298	0.308208	0.0540537	0.178559	0.207431
XLOC_089744	TMEM30A	chr9	15051846	15112162	0.0	0.0840398	0.65781	0.0125924	0.0	0.0	0.0	0.0238667	0.0
XLOC_037828	TMEM30C	chr1	44147733	44181228	3.25713	3.75374	4.22881	9.89125	10.5411	9.53494	8.06538	6.30478	9.00928
XLOC_039239	TMEM30C	chr1	44147733	44181228	2.74038	1.36652	0.714818	1.39242	1.39729	0.760194	1.62762	0.734637	0.839092
XLOC_037827	TMEM30C;C1H3orf26	chr1	44106920	44144747	2.52493	3.56714	3.88272	5.94819	8.05848	6.26803	7.12912	6.05672	8.74818
XLOC_038337	TMEM30C;C1H3orf26	chr1	44106920	44144747	9.2696	6.48917	6.76546	13.5328	18.8461	17.8466	21.7625	16.5209	21.4626
XLOC_082751	TMEM38A	chr7	6286586	6289474	0.0	7.51455	15.2563	9.21694	5.52007	5.81204	5.43762	5.07548	5.0029
XLOC_082752	TMEM38A	chr7	6290642	6305372	6.83695	9.05448	11.4584	5.7771	5.49333	5.26624	3.80068	6.34146	4.86125
XLOC_084107	TMEM38A	chr7	6286586	6289474	2.61592	0.0	0.0	0.234049	0.4034	0.537053	1.14895	0.512334	0.454146
XLOC_084108	TMEM38A	chr7	6286586	6289474	0.0	0.0	0.0	0.770829	0.0	0.0	0.0	0.414235	0.0
XLOC_084110	TMEM38A	chr7	6290642	6305372	6.40918	3.82148	0.0	0.0	0.0	0.647152	0.0	0.604753	0.551417
XLOC_084111	TMEM38A	chr7	6306100	6306222	0.0	91.572	0.0	92.7223	59.588	29.2124	0.0	95.242	127.981
XLOC_084112	TMEM38A	chr7	6306540	6307205	2.85039	8.09419	6.6848	4.08523	5.54654	4.56839	4.73289	4.10585	5.09394
XLOC_084113	TMEM38A	chr7	6307520	6307897	0.0	0.0	0.0	0.294603	0.505398	0.336844	1.14396	0.960221	0.285304
XLOC_088634	TMEM38B	chr8	97164396	97164756	3.56971	5.32498	5.5696	12.7686	7.92545	11.6634	5.24435	10.0279	5.5603
XLOC_038436	TMEM41A	chr1	82233001	82290992	4.50884	3.34883	3.52974	0.352023	0.282496	0.470133	0.130612	0.109625	0.160923
XLOC_037830	TMEM45A	chr1	45311440	45349194	95.9165	27.4059	32.5512	14.0747	2.92836	10.1785	11.5989	6.60906	9.20024

XLOC_053712	TMEM55A	chr14	75702395	75754524	12.9429	14.9009	14.096	12.261	15.727	12.7556	15.0707	12.2201	18.2831
XLOC_053713	TMEM55A	chr14	75761067	75787027	4.97865	5.60881	5.0896	8.65519	15.3952	8.86027	14.1052	6.90252	15.2375
XLOC_054831	TMEM55A	chr14	75759860	75760770	3.84457	2.58686	6.01386	6.89098	9.31119	6.7776	9.30962	4.60919	10.0716
XLOC_065869	TMEM57	chr2	128125961	128160079	14.165	16.4037	13.6716	14.1109	10.6762	12.5316	7.39556	9.41553	7.44982
XLOC_068549	TMEM57	chr2	128125961	128160079	6.03233	5.77128	4.71685	4.64808	4.3273	4.24726	2.718	1.44207	2.73673
XLOC_068550	TMEM57	chr2	128125961	128160079	3.05791	4.56327	2.38667	2.73505	1.41005	3.44402	0.266547	0.893737	2.12038
XLOC_068551	TMEM57	chr2	128160145	128162756	5.30589	5.99821	9.4591	4.62625	3.86662	4.38939	2.37745	3.5334	2.94156
XLOC_083366	TMEM59L	chr7	4504578	4509058	2.0404	0.609535	0.0	8.40207	11.2206	8.19697	4.70836	7.04153	17.2151
XLOC_083367	TMEM59L	chr7	4509432	4513839	0.0	0.786288	0.0	9.90103	14.9552	9.63979	4.39589	7.88594	25.5291
XLOC_072602	TMEM60	chr4	43685889	43690780	1.24643	2.61007	3.41323	0.893952	0.878796	0.453344	0.39739	0.375136	0.980791
XLOC_053707	TMEM67	chr14	72820124	72841423	0.0	0.0	0.0	2.14588	0.722191	0.799016	0.309021	1.41787	1.03749
XLOC_054801	TMEM67	chr14	72820124	72841423	0.0	0.0	0.0	0.104949	0.0913539	0.0	0.0	0.11672	0.0
XLOC_054802	TMEM67	chr14	72820124	72841423	0.0	0.0	0.0	0.0	0.0	0.0	0.341695	0.0	0.0
XLOC_054803	TMEM67	chr14	72820124	72841423	0.0	0.0	0.0	0.0	0.416682	0.832237	0.237147	0.0	0.234637
XLOC_054804	TMEM67	chr14	72820124	72841423	0.0	0.391353	0.0	1.29007	0.407959	1.35461	0.706482	0.651164	0.456682
XLOC_065047	TMEM92	chr19	37022848	37023854	0.852392	1.27467	0.0	0.152799	0.133304	0.265358	0.154633	0.255821	0.297853
XLOC_092413	TMLHE;TMLHE	chrX	38376971	38456565	79.8814	57.1896	60.587	23.2116	32.4597	27.9223	28.399	20.1648	41.4836
XLOC_092890	TMLHE;TMLHE	chrX	38376971	38456565	1.59573	0.715904	0.0	0.500606	1.06105	0.662569	0.362213	0.399353	0.766837
XLOC_092891	TMLHE;TMLHE	chrX	38376971	38456565	1.2005	0.717822	1.87742	1.07557	1.31049	0.870115	0.973713	0.717496	1.25684
XLOC_092892	TMLHE;TMLHE	chrX	38376971	38456565	0.864652	0.517177	1.35268	0.387474	0.473213	0.35886	0.548856	0.691871	0.830809
XLOC_092893	TMLHE;TMLHE	chrX	38376971	38456565	0.0	0.417788	0.0	0.3756	0.217622	0.722721	0.502029	0.416674	1.09668
XLOC_092894	TMLHE;TMLHE	chrX	38376971	38456565	0.0	4.58256	7.19008	0.549325	2.12362	3.77277	1.87332	0.598262	3.72614
XLOC_092895	TMLHE;TMLHE	chrX	38376971	38456565	4.28218	2.30527	4.0195	1.07466	1.27235	1.51078	1.08748	0.25702	1.64593
XLOC_092896	TMLHE;TMLHE	chrX	38376971	38456565	1.10343	2.96928	4.31434	0.494351	1.9804	1.82912	1.59392	0.330131	1.82972
XLOC_041547	TMOD3;LEO1	chr10	58499316	58576241	10.0346	8.53247	6.88381	11.5638	13.2695	12.8706	12.1909	10.1205	16.4024
XLOC_041929	TMX1	chr10	44004777	44148841	0.0	1.28036	6.69662	0.0	0.0	0.0	0.0	0.0	0.0
XLOC_055897	TMX2	chr15	82371513	82372526	3.36811	0.503259	0.0	0.452431	0.392424	0.34771	0.0	0.0	0.293341
XLOC_086632	TNC	chr8	105973370	105984102	0.0	0.0	0.0	3.6134	2.54561	6.17758	0.124105	0.137326	2.14791

XLOC_086633	TNC	chr8	106009619	106053842	3.44531	2.90022	1.77533	13.1099	12.262	12.9953	13.0862	12.5147	11.0214
XLOC_086634	TNC	chr8	106053892	106130535	24.3606	26.3848	25.9681	27.5351	31.8682	31.0737	33.6838	25.6594	26.2416
XLOC_087059	TNC	chr8	105939433	105966106	12.2669	10.7157	10.8155	14.1412	13.7688	13.8924	9.11286	8.96645	6.88014
XLOC_087060	TNC	chr8	105973370	105984102	8.2584	10.1387	10.0174	3.48307	1.80846	1.35255	2.2278	1.30972	1.76791
XLOC_088696	TNC	chr8	105984477	105985464	0.0	0.0	0.0	0.937777	0.954356	1.26655	0.158118	0.261618	0.304654
XLOC_088697	TNC	chr8	106053892	106130535	0.0	0.931938	0.0	0.977507	0.914177	0.404341	0.636757	0.311985	0.340326
XLOC_064202	TNFAIP1;IFT20	chr19	20373427	20390982	33.0538	25.814	23.6598	4.15642	0.627418	3.23472	4.55183	2.58706	4.27798
XLOC_066280	TNFAIP6	chr2	44564312	44927685	24.9332	19.9038	10.2547	9.52524	8.95979	9.22638	9.46407	7.54568	9.10157
XLOC_067443	TNFAIP6	chr2	44564312	44927685	2.758	2.0611	0.0	0.123531	0.644248	1.28368	0.743247	0.0	0.360706
XLOC_067444	TNFAIP6	chr2	44564312	44927685	1.29904	1.94299	2.03271	0.232915	0.50872	0.269945	0.295687	0.260563	0.170349
XLOC_067445	TNFAIP6	chr2	44564312	44927685	0.553568	1.49049	1.73259	0.397054	0.260342	0.517958	0.353438	0.444665	0.532526
XLOC_067446	TNFAIP6	chr2	44564312	44927685	3.14792	2.51143	3.69481	1.31714	1.02834	1.47298	1.19699	0.948576	1.33074
XLOC_086457	TNFRSF10D	chr8	70903699	70917391	17.5433	22.0455	21.497	18.2707	14.4707	17.0936	15.7542	20.5514	8.85561
XLOC_088051	TNFRSF10D	chr8	70903699	70917391	3.43455	7.17344	2.68014	2.76428	1.31607	2.45653	0.892433	2.33248	1.784
XLOC_088052	TNFRSF10D	chr8	70920549	70922160	0.0	1.04208	0.38936	0.0892292	0.0390195	0.0517483	0.22716	0.0999921	0.043524
XLOC_049058	TNFRSF19	chr12	34750813	34751857	2.44755	2.44015	3.82916	0.0731273	0.0	0.0	0.0	0.0	0.0
XLOC_048467	TNFSF11	chr12	12779664	12780149	0.0	0.0	0.0	0.9884	2.05006	1.59091	0.977135	1.08667	0.959962
XLOC_048468	TNFSF11	chr12	12780761	12781512	0.0	0.0	0.0	0.327559	1.61521	0.378519	0.219589	0.364108	0.637906
XLOC_082797	TNFSF14	chr7	19076559	19083721	24.2555	24.2743	24.0622	19.5633	15.627	17.9015	16.2496	19.7075	14.7432
XLOC_084306	TNFSF14	chr7	19076559	19083721	2.57863	7.6995	10.0673	8.07556	5.37012	9.53161	8.38676	7.32652	4.70127
XLOC_086625	TNFSF15	chr8	105695029	105758701	0.0	0.877511	0.445718	1.68589	2.14857	1.6934	1.56446	1.55938	0.615392
XLOC_086626	TNFSF15	chr8	105695029	105758701	0.0	1.60725	0.0	3.13616	3.92713	2.77076	2.5633	1.518	2.77179
XLOC_087054	TNFSF15	chr8	105695029	105758701	14.8913	21.7928	14.8597	14.3729	18.8741	12.4237	12.9736	15.8348	13.7032
XLOC_087055	TNFSF15	chr8	105695029	105758701	6.11335	11.2974	11.1299	11.5131	10.9428	10.977	9.65721	10.1404	8.1569
XLOC_088684	TNFSF15	chr8	105695029	105758701	0.0	1.49256	1.95175	0.559106	0.486424	0.646025	0.112387	0.124293	0.108875
XLOC_088685	TNFSF15	chr8	105695029	105758701	0.0	1.00578	2.62999	1.20577	0.516855	1.03368	0.876782	0.98179	0.0
XLOC_086627	TNFSF8	chr8	105843861	105857288	1.60217	3.94059	3.35943	2.83168	3.0736	3.48408	2.25824	3.82394	2.15582
XLOC_086628	TNFSF8	chr8	105860357	105878151	3.15606	5.87224	1.44993	9.69098	7.3615	10.2599	6.4805	7.92241	6.2129
XLOC_087057	TNFSF8	chr8	105843861	105857288	14.8769	20.4428	19.0767	38.2799	41.1546	33.6181	35.9439	36.9822	32.0967
XLOC_088689	TNFSF8	chr8	105841809	105842689	2.00211	1.49677	0.782916	0.717683	0.4691	0.622702	0.45276	0.199927	0.262208
XLOC_088690	TNFSF8	chr8	105860357	105878151	4.75042	4.73617	4.33542	7.16773	5.07904	5.34232	4.60132	4.51808	3.32063
XLOC_088691	TNFSF8	chr8	105860357	105878151	5.87268	2.33938	0.0	5.25759	4.55161	4.03497	3.82799	4.05746	2.21418

XLOC_088692	TNFSF8	chr8	105860357	105878151	0.808554	2.66019	1.89747	2.39164	2.34013	2.93743	2.2754	2.10423	1.76612
XLOC_088693	TNFSF8	chr8	105860357	105878151	3.27926	1.95718	5.11764	0.293286	0.754759	1.34139	0.569537	0.956059	1.13611
XLOC_088694	TNFSF8	chr8	105860357	105878151	16.771	4.99952	8.71418	11.5116	8.45276	9.61955	6.09637	9.02618	10.1115
XLOC_079845	TNIP2	chr6	108064973	108077188	3.63547	3.08079	2.68489	3.86282	3.80952	3.40379	3.73738	4.19248	3.57807
XLOC_080251	TNIP2	chr6	108108235	108185564	8.19924	7.56671	6.41574	8.95256	5.34262	5.95833	8.29871	8.9824	7.79161
XLOC_082126	TNIP2	chr6	108106694	108107173	4.49971	2.01591	1.75729	1.61089	1.39176	1.15728	2.18819	1.1064	0.195547
XLOC_082127	TNIP2	chr6	108107636	108108093	12.0755	5.76972	3.7721	7.34801	4.10314	6.20521	7.45172	6.87485	6.08383
XLOC_082128	TNIP2	chr6	108108235	108185564	135.328	128.212	102.438	537.432	467.932	499.236	426.094	469.844	470.279
XLOC_064103	TOM1L1	chr19	5133141	5272669	13.199	16.5474	14.1893	5.88402	6.66064	6.51151	5.93904	4.91686	4.47176
XLOC_063974	TOP3A;SHMT1	chr19	34922338	34975647	17.461	21.5834	22.6695	47.539	46.6918	50.8136	60.226	59.0255	63.1367
XLOC_038564	TOPBP1;CDV3	chr1	136842495	136953869	9.93007	13.2999	16.3441	38.8794	38.7169	37.5765	47.5765	52.4389	46.8034
XLOC_045073	TOR1A;PTGES;C11H9orf78;USP20	chr11	100140748	100262072	10.9106	0.908569	2.91807	13.6334	23.6937	19.3288	18.9844	11.7996	26.4553
XLOC_047260	TOR1A;PTGES;C11H9orf78;USP20	chr11	100140748	100262072	8.69628	9.51812	4.52539	25.93	24.0867	19.9038	27.3645	26.3124	30.6687
XLOC_044425	TOR2A;TTC16;SH2D3C	chr11	98412455	98439397	5.16396	8.16473	8.94628	1.65207	1.6146	1.27833	1.65715	2.42154	1.03279
XLOC_045060	TOR2A;TTC16;SH2D3C	chr11	98412455	98439397	6.82805	6.85694	4.38734	12.1002	10.8734	12.021	11.4073	13.4446	11.2244
XLOC_053221	TOX	chr14	26579326	26631386	15.3899	14.4595	15.7008	22.469	27.774	25.1905	21.448	23.7789	32.7975
XLOC_053222	TOX	chr14	26637337	26657200	50.0909	41.4235	40.2598	41.3008	53.6132	46.7835	47.1166	42.9017	54.3903
XLOC_053223	TOX	chr14	26716641	26724619	0.961144	1.14972	0.0	0.94751	0.750903	0.897035	1.13107	1.05627	1.34288
XLOC_053224	TOX	chr14	26856132	26894540	1.06894	0.315409	1.3027	30.2471	22.9679	26.478	15.875	24.473	31.4556
XLOC_053225	TOX	chr14	26911224	26915273	1.94528	1.55829	2.54224	1.8932	2.77387	2.23504	2.53443	2.19551	3.73447
XLOC_053510	TOX	chr14	26664788	26670168	11.2498	12.3204	11.0536	17.3885	21.3297	18.7249	17.2609	18.602	24.6451
XLOC_053511	TOX	chr14	26911224	26915273	0.614896	0.198978	4.11635	0.0	1.6553	2.45938E-4	0.0	6.07302E-4	5.98371E-4
XLOC_053512	TOX	chr14	26919461	26970572	31.4581	45.6494	27.2894	34.5148	50.3719	39.5139	42.9623	30.9629	60.5532
XLOC_054016	TOX	chr14	26631530	26631772	8.30118	0.0	12.9498	13.4552	17.99	5.84372	10.1506	7.73474	21.3949
XLOC_054017	TOX	chr14	26632576	26633045	0.0	2.08048	0.0	0.207811	2.33289	1.43269	0.820186	1.5973	3.22836
XLOC_054018	TOX	chr14	26633883	26634295	5.68374	0.848342	2.2184	1.01689	3.28114	0.291337	2.73513	2.22048	3.94395
XLOC_054019	TOX	chr14	26635233	26636237	2.56322	0.766609	0.0	1.1487	1.60341	1.32991	1.31744	0.683783	2.23916
XLOC_054020	TOX	chr14	26636530	26637154	1.55047	1.3902	1.21191	0.972068	2.04953	0.961179	0.971887	0.461372	1.08081

XLOC_054021	TOX	chr14	26658098	26658292	0.0	0.0	12.7498	4.48752	16.6724	4.87229	2.53483	0.0	1.39599
XLOC_054022	TOX	chr14	26658451	26658782	12.4867	2.4826	9.73722	1.86099	5.0777	2.96608	1.78151	2.40391	3.59577
XLOC_054023	TOX	chr14	26659243	26659750	2.0696	6.18239	1.61679	1.85262	1.76286	1.91833	1.65226	1.01997	1.79996
XLOC_054024	TOX	chr14	26660213	26660382	28.3084	0.0	0.0	8.07113	8.20404	8.51856	4.30792	10.0067	26.9636
XLOC_054025	TOX	chr14	26661586	26662273	2.73195	2.44999	3.20371	2.08023	2.87204	2.11944	1.35007	0.814775	3.69227
XLOC_054026	TOX	chr14	26662862	26663861	2.57845	2.05643	1.34459	2.31104	4.43539	2.94309	3.89748	1.80548	5.18057
XLOC_054027	TOX	chr14	26663948	26664676	3.80339	1.13706	0.991253	3.06674	5.03901	3.54295	3.42354	3.15467	5.41864
XLOC_054028	TOX	chr14	26848929	26850173	0.665664	0.199124	0.0	1.1935	0.990461	0.622407	0.302945	0.333731	0.698288
XLOC_054029	TOX	chr14	26853565	26854386	0.0	0.0	0.0	2.63734	1.95673	2.03311	0.885985	2.39216	2.66405
XLOC_054030	TOX	chr14	26854535	26855996	0.0	0.331983	0.0	2.33806	2.34845	1.96122	0.860305	2.00557	1.60125
XLOC_054031	TOX	chr14	26856132	26894540	0.0	0.0	0.0	3.53524	3.53779	2.02106	1.14398	0.320074	2.56773
XLOC_054032	TOX	chr14	26856132	26894540	0.0	0.0	3.13844	6.11747	6.75663	6.96914	2.41665	5.43094	7.65127
XLOC_054033	TOX	chr14	26856132	26894540	0.0	0.0	0.0	4.93838	5.68559	3.46865	5.68409	4.52779	8.87279
XLOC_054034	TOX	chr14	26911224	26915273	3.0295	6.79108	3.55209	1.35672	1.53161	1.5654	1.62877	1.05219	2.50812
XLOC_054035	TOX	chr14	26917432	26918389	2.71391	1.35273	0.70758	0.40539	0.777786	0.469219	0.983715	0.271326	1.10617
XLOC_054036	TOX	chr14	26918934	26919365	7.93378	7.10629	12.3888	12.5403	17.3286	10.5856	15.789	10.8733	15.8382
XLOC_055813	TP53I11	chr15	75883395	75885383	1.20289	0.539777	1.41175	1.13236	0.848249	0.937648	0.164428	0.301775	0.420715
XLOC_056961	TP53I11	chr15	75876190	75876527	0.0	0.0	0.0	3.59851	3.37831	1.22985	1.72617	3.87925	5.56456
XLOC_072751	TPK1	chr4	108564822	108614648	12.1372	15.6213	11.041	47.412	40.4731	46.1917	44.508	52.134	46.4583
XLOC_072752	TPK1	chr4	108619369	108634234	0.0	2.089	0.0	1.25246	1.60906	1.43057	0.30305	1.35776	0.909276
XLOC_072753	TPK1	chr4	108619369	108634234	15.0126	18.2219	16.914	21.8301	18.8332	19.2648	20.823	22.5977	20.044
XLOC_072754	TPK1	chr4	108819062	108856643	3.02124	3.94692	4.16977	3.30943	4.55826	4.10124	4.589	3.22408	4.1661
XLOC_072755	TPK1	chr4	108857925	108862251	33.6779	22.1109	23.1314	32.3215	48.7625	36.4889	48.9043	30.7449	53.1778
XLOC_072756	TPK1	chr4	108879031	108929517	4.40583	3.07278	5.7401	2.10474	1.59978	1.51823	1.44878	2.47628	1.9201
XLOC_073285	TPK1	chr4	108564822	108614648	24.6703	29.6566	24.5354	54.8837	50.4467	51.77	64.2375	64.4856	67.288
XLOC_073286	TPK1	chr4	108638507	108780609	14.4797	17.4912	14.6412	4.09792	4.09602	4.33849	5.23922	4.08321	3.4533
XLOC_073287	TPK1	chr4	108814644	108818867	7.75633	5.67187	6.11573	6.65003	6.74162	6.9631	7.39759	7.39049	8.09457
XLOC_073288	TPK1	chr4	108819062	108856643	8.10729	5.96051	21.2911	10.9032	7.4394	10.681	12.0617	9.40672	10.3291
XLOC_074891	TPK1	chr4	108559654	108560755	2.29968	2.29283	1.19933	1.85524	1.6795	2.30807	2.08926	0.537375	2.07657
XLOC_074892	TPK1	chr4	108561022	108561506	4.4308	1.9851	0.0	2.57769	4.45517	1.59572	1.96002	1.74385	3.08121
XLOC_074893	TPK1	chr4	108561979	108562646	1.4196	0.42434	0.0	1.90745	2.76251	2.4955	4.20509	2.11552	3.71272
XLOC_074894	TPK1	chr4	108563295	108564539	3.32832	3.38511	2.60397	3.1031	2.24157	2.76625	2.96886	1.53516	3.31687
XLOC_074895	TPK1	chr4	108618403	108618767	3.50084	1.0445	0.0	0.939189	0.536302	1.7879	1.51442	0.678748	0.909027

XLOC_074896	TPK1	chr4	108619369	108634234	5.30894	3.16525	4.13793	1.42572	5.22589	5.91724	4.47018	1.51676	4.57516
XLOC_074897	TPK1	chr4	108634801	108635501	5.33296	2.78991	6.25409	1.43325	1.86929	1.1035	0.839051	1.45854	2.3253
XLOC_074898	TPK1	chr4	108635702	108636066	14.0034	0.0	5.46245	1.25225	1.34076	0.0	0.302884	0.678748	0.909027
XLOC_074899	TPK1	chr4	108636546	108637460	1.91212	2.28729	1.49552	0.514093	1.64332	1.38805	0.865538	0.573138	0.83488
XLOC_074900	TPK1	chr4	108638058	108638441	9.62019	4.78484	5.00476	2.00757	1.23075	3.93674	6.41269	2.1832	1.94466
XLOC_074901	TPK1	chr4	108812535	108813517	1.75441	1.31175	1.37229	0.550351	0.891525	0.455068	0.954369	0.526375	0.996111
XLOC_074902	TPK1	chr4	108819062	108856643	2.90186	5.2043	3.40266	2.46934	1.01619	2.10006	2.47312	2.01729	2.15006
XLOC_074903	TPK1	chr4	108819062	108856643	13.058	5.84498	2.54733	6.71495	1.50287	4.67411	1.9849	2.53838	1.69657
XLOC_074904	TPK1	chr4	108819062	108856643	3.12251	1.8679	2.44265	1.67934	2.32088	1.29667	1.84344	1.25061	0.954965
XLOC_074905	TPK1	chr4	108819062	108856643	8.65411	10.3594	8.65532	9.87451	6.97055	8.21341	9.43949	11.4128	6.65091
XLOC_072750	TPK1;ARHGEF5	chr4	108460129	108559414	24.4734	36.3224	27.4287	22.9167	25.4763	30.2655	31.2086	23.3336	22.0241
XLOC_062191	TPPP3;ATP6V0D1;HSD11B2;AGRP;ZDHHC1;CTCF	chr18	35118884	35255348	68.1334	62.8021	63.6421	157.219	146.153	154.062	169.964	203.079	168.299
XLOC_037937	TPRG1	chr1	78657892	78706901	80.3851	94.9902	80.5446	66.1687	74.1302	71.9272	73.9961	63.9656	72.7365
XLOC_039695	TPRG1	chr1	78604707	78605733	0.0	0.0	0.0	0.149283	0.130255	0.518558	0.0	0.0	0.0727536
XLOC_039696	TPRG1	chr1	78657892	78706901	14.67	15.7639	13.7407	23.6207	27.3077	22.5466	24.092	26.3362	19.0811
XLOC_039697	TPRG1	chr1	78657892	78706901	8.30215	8.68014	6.48569	12.4485	12.8576	11.5426	13.6228	12.07	13.1774
XLOC_039698	TPRG1	chr1	78657892	78706901	1.73663	8.82202	6.78574	6.99827	6.87709	9.49885	8.52408	7.0471	6.20028
XLOC_039699	TPRG1	chr1	78657892	78706901	0.0	4.91806	7.1454	6.22294	4.54105	3.77267	3.42174	4.52039	4.77618
XLOC_050593	TPX2;BCL2L1;COX4I2;FOXS1;MYLK2;PDRG1;XKR7;POFUT1;POFUT1;HCK;TM9SF4;POFUT1;HCK;POFUT1;TTLL9;POFUT1	chr13	61745935	62237095	1.91214	2.00138	0.747762	4.54116	4.78056	5.25477	4.41424	7.06871	5.84416

XLOC_050594	TPX2;BCL2L1;COX4I2;FOXS1;MYLK2;PDRG1;XKR7;POFUT1;POFUT1;HCK;TM9SF4;POFUT1;HCK;POFUT1;TTLL9;POFUT1	chr13	61745935	62237095	3.59773	0.896893	2.18837	68.376	123.981	160.827	127.055	160.473	201.109
XLOC_050595	TPX2;BCL2L1;COX4I2;FOXS1;MYLK2;PDRG1;XKR7;POFUT1;POFUT1;HCK;TM9SF4;POFUT1;HCK;POFUT1;TTLL9;POFUT1	chr13	61745935	62237095	0.0	1.225	0.0	7.49024	7.09904	5.22648	5.01091	7.95597	5.38193
XLOC_050596	TPX2;BCL2L1;COX4I2;FOXS1;MYLK2;PDRG1;XKR7;POFUT1;POFUT1;HCK;TM9SF4;POFUT1;HCK;POFUT1;TTLL9;POFUT1	chr13	61745935	62237095	0.0	0.202825	0.0	1.10222	1.65228	0.983714	1.66546	0.4615	1.41975
XLOC_050597	TPX2;BCL2L1;COX4I2;FOXS1;MYLK2;PDRG1;XKR7;POFUT1;POFUT1;HCK;TM9SF4;POFUT1;HCK;POFUT1;TTLL9;POFUT1	chr13	61745935	62237095	1.45387	0.0	0.0	10.939	4.18599	5.56122	5.73844	8.37385	6.96998

XLOC_050598	TPX2;BCL2L1;COX4I2;FOXS1;MYLK2;PDRG1;XKR7;POFUT1;POFUT1;HCK;TM9SF4;POFUT1;HCK;POFUT1;TTLL9;POFUT1	chr13	61745935	62237095	3.91515	1.7476	4.58833	31.1498	26.7485	27.6804	27.7094	36.6977	34.2234
XLOC_050900	TPX2;BCL2L1;COX4I2;FOXS1;MYLK2;PDRG1;XKR7;POFUT1;POFUT1;HCK;TM9SF4;POFUT1;HCK;POFUT1;TTLL9;POFUT1	chr13	61745935	62237095	0.0	0.0	0.0	7.30839	6.80714	8.07368	9.98859	12.3183	7.12177
XLOC_050901	TPX2;BCL2L1;COX4I2;FOXS1;MYLK2;PDRG1;XKR7;POFUT1;POFUT1;HCK;TM9SF4;POFUT1;HCK;POFUT1;TTLL9;POFUT1	chr13	61745935	62237095	0.0	0.0	0.917375	4.12623	1.88725	3.6467	4.47134	2.98604	4.23457
XLOC_051978	TPX2;BCL2L1;COX4I2;FOXS1;MYLK2;PDRG1;XKR7;POFUT1;POFUT1;HCK;TM9SF4;POFUT1;HCK;POFUT1;TTLL9;POFUT1	chr13	61745935	62237095	2.83078	0.564368	2.21404	2.70609	2.35933	1.85935	2.39244	3.11161	3.13129

XLOC_051979	TPX2;BCL2L1;COX4I2;FOXS1;MYLK2;PDRG1;XKR7;POFUT1;POFUT1;HCK;TM9SF4;POFUT1;HCK;POFUT1;TTLL9;POFUT1	chr13	61745935	62237095	0.0	0.0	0.0	8.74937	7.04991	17.7837	5.38864	17.4101	9.39314
XLOC_051980	TPX2;BCL2L1;COX4I2;FOXS1;MYLK2;PDRG1;XKR7;POFUT1;POFUT1;HCK;TM9SF4;POFUT1;HCK;POFUT1;TTLL9;POFUT1	chr13	61745935	62237095	1.45451	0.652598	1.70681	2.34689	2.56158	3.09654	2.97534	3.20611	3.75003
XLOC_051981	TPX2;BCL2L1;COX4I2;FOXS1;MYLK2;PDRG1;XKR7;POFUT1;POFUT1;HCK;TM9SF4;POFUT1;HCK;POFUT1;TTLL9;POFUT1	chr13	61745935	62237095	1.40857	0.421039	0.0	1.00939	2.19299	1.31094	1.64398	2.37924	1.47358
XLOC_051982	TPX2;BCL2L1;COX4I2;FOXS1;MYLK2;PDRG1;XKR7;POFUT1;POFUT1;HCK;TM9SF4;POFUT1;HCK;POFUT1;TTLL9;POFUT1	chr13	61745935	62237095	0.699138	0.627381	1.09391	2.00552	1.58732	2.10591	1.14471	1.68175	1.83325

XLOC_051983	TPX2;BCL2L1;COX4I2;FOXS1;MYLK2;PDRG1;XKR7;POFUT1;POFUT1;HCK;TM9SF4;POFUT1;HCK;POFUT1;TTLL9;POFUT1	chr13	61745935	62237095	0.0	1.17602	0.0	0.352409	0.457606	0.202833	0.174914	0.388484	1.02745
XLOC_051984	TPX2;BCL2L1;COX4I2;FOXS1;MYLK2;PDRG1;XKR7;POFUT1;POFUT1;HCK;TM9SF4;POFUT1;HCK;POFUT1;TTLL9;POFUT1	chr13	61745935	62237095	1.95759	0.0	0.0	0.701011	0.0	0.403497	1.74	0.193219	0.851606
XLOC_051985	TPX2;BCL2L1;COX4I2;FOXS1;MYLK2;PDRG1;XKR7;POFUT1;POFUT1;HCK;TM9SF4;POFUT1;HCK;POFUT1;TTLL9;POFUT1	chr13	61745935	62237095	1.84757	0.0	1.44365	1.98507	1.57651	1.90539	2.13914	1.46078	1.92976
XLOC_051986	TPX2;BCL2L1;COX4I2;FOXS1;MYLK2;PDRG1;XKR7;POFUT1;POFUT1;HCK;TM9SF4;POFUT1;HCK;POFUT1;TTLL9;POFUT1	chr13	61745935	62237095	0.0	1.69498	0.0	1.35446	1.75969	1.75474	1.68288	0.560392	1.64571

XLOC_051987	TPX2;BCL2L1;COX4I2;FOXS1;MYLK2;PDRG1;XKR7;POFUT1;POFUT1;HCK;TM9SF4;POFUT1;HCK;POFUT1;TTLL9;POFUT1	chr13	61745935	62237095	0.0	0.540227	0.0	0.485699	0.896118	0.563055	0.713108	1.02688	0.789496
XLOC_051988	TPX2;BCL2L1;COX4I2;FOXS1;MYLK2;PDRG1;XKR7;POFUT1;POFUT1;HCK;TM9SF4;POFUT1;HCK;POFUT1;TTLL9;POFUT1	chr13	61745935	62237095	0.0	0.0	0.0	1.62732	2.79675	0.621033	0.793299	0.295516	1.05138
XLOC_051989	TPX2;BCL2L1;COX4I2;FOXS1;MYLK2;PDRG1;XKR7;POFUT1;POFUT1;HCK;TM9SF4;POFUT1;HCK;POFUT1;TTLL9;POFUT1	chr13	61745935	62237095	0.0	1.84346	0.964243	13.1481	15.2855	13.6604	15.8838	17.9282	20.8707
XLOC_051990	TPX2;BCL2L1;COX4I2;FOXS1;MYLK2;PDRG1;XKR7;POFUT1;POFUT1;HCK;TM9SF4;POFUT1;HCK;POFUT1;TTLL9;POFUT1	chr13	61745935	62237095	0.0	0.347951	0.910003	0.834183	1.08926	1.80794	1.3644	0.695876	1.62476

XLOC_051991	TPX2;BCL2L1;COX4I2;FOXS1;MYLK2;PDRG1;XKR7;POFUT1;POFUT1;HCK;TM9SF4;POFUT1;HCK;POFUT1;TTLL9;POFUT1	chr13	61745935	62237095	0.0	0.0	0.0	0.703367	1.70957	1.62264	0.562394	0.623032	0.68426
XLOC_051992	TPX2;BCL2L1;COX4I2;FOXS1;MYLK2;PDRG1;XKR7;POFUT1;POFUT1;HCK;TM9SF4;POFUT1;HCK;POFUT1;TTLL9;POFUT1	chr13	61745935	62237095	0.0	0.0	0.0	0.845997	1.22841	1.30463	0.663616	0.523437	1.37355
XLOC_051993	TPX2;BCL2L1;COX4I2;FOXS1;MYLK2;PDRG1;XKR7;POFUT1;POFUT1;HCK;TM9SF4;POFUT1;HCK;POFUT1;TTLL9;POFUT1	chr13	61745935	62237095	0.0	0.0	0.0	0.803772	1.11625	0.712819	1.7805	0.741525	1.33752
XLOC_051994	TPX2;BCL2L1;COX4I2;FOXS1;MYLK2;PDRG1;XKR7;POFUT1;POFUT1;HCK;TM9SF4;POFUT1;HCK;POFUT1;TTLL9;POFUT1	chr13	61745935	62237095	4.37721	0.0	0.0	1.37123	1.69315	1.35143	1.35606	1.07707	1.71237

XLOC_051995	TPX2;BCL2L1;COX4I2;FOXS1;MYLK2;PDRG1;XKR7;POFUT1;POFUT1;HCK;TM9SF4;POFUT1;HCK;POFUT1;TTLL9;POFUT1	chr13	61745935	62237095	0.0	0.0	3.61593	2.48906	2.82055	5.65404	1.97102	1.33305	1.20067
XLOC_051996	TPX2;BCL2L1;COX4I2;FOXS1;MYLK2;PDRG1;XKR7;POFUT1;POFUT1;HCK;TM9SF4;POFUT1;HCK;POFUT1;TTLL9;POFUT1	chr13	61745935	62237095	0.0	0.979404	0.512311	1.23276	1.48725	2.04102	1.19227	1.31335	1.77458
XLOC_051997	TPX2;BCL2L1;COX4I2;FOXS1;MYLK2;PDRG1;XKR7;POFUT1;POFUT1;HCK;TM9SF4;POFUT1;HCK;POFUT1;TTLL9;POFUT1	chr13	61745935	62237095	0.502778	0.300841	0.0	1.9835	2.12894	1.93459	1.42294	1.66697	1.45124
XLOC_051998	TPX2;BCL2L1;COX4I2;FOXS1;MYLK2;PDRG1;XKR7;POFUT1;POFUT1;HCK;TM9SF4;POFUT1;HCK;POFUT1;TTLL9;POFUT1	chr13	61745935	62237095	0.0	0.0	0.0	2.48577	1.25223	2.85634	1.22651	0.909911	1.40793

XLOC_051999	TPX2;BCL2L1;COX4I2;FOXS1;MYLK2;PDRG1;XKR7;POFUT1;POFUT1;HCK;TM9SF4;POFUT1;HCK;POFUT1;TTLL9;POFUT1	chr13	61745935	62237095	0.0	0.0	3.17911	2.54997	2.20639	1.25757	1.0836	2.40762	1.59282
XLOC_052000	TPX2;BCL2L1;COX4I2;FOXS1;MYLK2;PDRG1;XKR7;POFUT1;POFUT1;HCK;TM9SF4;POFUT1;HCK;POFUT1;TTLL9;POFUT1	chr13	61745935	62237095	0.0	0.165232	0.0	1.13892	1.12558	1.09097	0.705274	0.887309	1.11092
XLOC_052001	TPX2;BCL2L1;COX4I2;FOXS1;MYLK2;PDRG1;XKR7;POFUT1;POFUT1;HCK;TM9SF4;POFUT1;HCK;POFUT1;TTLL9;POFUT1	chr13	61745935	62237095	0.0	0.462485	0.0	1.7325	1.45181	2.24745	1.61527	1.16127	1.21603
XLOC_052002	TPX2;BCL2L1;COX4I2;FOXS1;MYLK2;PDRG1;XKR7;POFUT1;POFUT1;HCK;TM9SF4;POFUT1;HCK;POFUT1;TTLL9;POFUT1	chr13	61745935	62237095	0.0	1.44243	0.0	1.51283	3.54363	1.48925	1.70325	2.60771	1.25872

XLOC_052003	TPX2;BCL2L1;COX4I2;FOXS1;MYLK2;PDRG1;XKR7;POFUT1;POFUT1;HCK;TM9SF4;POFUT1;HCK;POFUT1;TTLL9;POFUT1	chr13	61745935	62237095	0.0	0.655843	0.0	1.17925	1.71174	1.59084	0.891252	1.42201	1.24435
XLOC_052004	TPX2;BCL2L1;COX4I2;FOXS1;MYLK2;PDRG1;XKR7;POFUT1;POFUT1;HCK;TM9SF4;POFUT1;HCK;POFUT1;TTLL9;POFUT1	chr13	61745935	62237095	0.0	2.374	0.0	7.15989	2.38178	0.800794	2.60281	1.4861	0.683807
XLOC_052005	TPX2;BCL2L1;COX4I2;FOXS1;MYLK2;PDRG1;XKR7;POFUT1;POFUT1;HCK;TM9SF4;POFUT1;HCK;POFUT1;TTLL9;POFUT1	chr13	61745935	62237095	0.0	0.0	0.0	1.05528	2.76324	1.62959	0.926647	1.25733	1.16587
XLOC_052006	TPX2;BCL2L1;COX4I2;FOXS1;MYLK2;PDRG1;XKR7;POFUT1;POFUT1;HCK;TM9SF4;POFUT1;HCK;POFUT1;TTLL9;POFUT1	chr13	61745935	62237095	0.0	0.406035	0.0	1.70349	1.58669	1.40506	1.83089	0.810266	0.710626

XLOC_052007	TPX2;BCL2L1;COX4I2;FOXS1;MYLK2;PDRG1;XKR7;POFUT1;POFUT1;HCK;TM9SF4;POFUT1;HCK;POFUT1;TTLL9;POFUT1	chr13	61745935	62237095	0.0	0.0	0.0	3.21794	2.68944	4.14414	2.97852	2.0599	2.04789
XLOC_052008	TPX2;BCL2L1;COX4I2;FOXS1;MYLK2;PDRG1;XKR7;POFUT1;POFUT1;HCK;TM9SF4;POFUT1;HCK;POFUT1;TTLL9;POFUT1	chr13	61745935	62237095	0.0	0.0	1.52532	1.74781	1.66449	1.20726	3.12384	4.04676	2.54797
XLOC_052009	TPX2;BCL2L1;COX4I2;FOXS1;MYLK2;PDRG1;XKR7;POFUT1;POFUT1;HCK;TM9SF4;POFUT1;HCK;POFUT1;TTLL9;POFUT1	chr13	61745935	62237095	0.0	0.564368	0.0	2.62153	2.87544	2.25079	2.13611	2.64015	1.48324
XLOC_052010	TPX2;BCL2L1;COX4I2;FOXS1;MYLK2;PDRG1;XKR7;POFUT1;POFUT1;HCK;TM9SF4;POFUT1;HCK;POFUT1;TTLL9;POFUT1	chr13	61745935	62237095	0.0	0.0	0.0	4.4122	2.48794	5.07443	3.79721	4.01014	2.60886

XLOC_052011	TPX2;BCL2L1;COX4I2;FOXS1;MYLK2;PDRG1;XKR7;POFUT1;POFUT1;HCK;TM9SF4;POFUT1;HCK;POFUT1;TTLL9;POFUT1	chr13	61745935	62237095	0.0	0.936307	0.0	1.40299	2.16853	5.45843	1.3655	3.96989	2.17485
XLOC_052012	TPX2;BCL2L1;COX4I2;FOXS1;MYLK2;PDRG1;XKR7;POFUT1;POFUT1;HCK;TM9SF4;POFUT1;HCK;POFUT1;TTLL9;POFUT1	chr13	61745935	62237095	0.0	0.0	0.0	1.00994	1.74504	2.32169	1.19713	1.10977	1.17691
XLOC_052013	TPX2;BCL2L1;COX4I2;FOXS1;MYLK2;PDRG1;XKR7;POFUT1;POFUT1;HCK;TM9SF4;POFUT1;HCK;POFUT1;TTLL9;POFUT1	chr13	61745935	62237095	0.0	0.0	0.0	2.61306	1.46357	1.40323	1.50613	2.39068	2.18192
XLOC_052014	TPX2;BCL2L1;COX4I2;FOXS1;MYLK2;PDRG1;XKR7;POFUT1;POFUT1;HCK;TM9SF4;POFUT1;HCK;POFUT1;TTLL9;POFUT1	chr13	61745935	62237095	0.0	0.37902	0.0	1.24941	0.889242	1.18098	1.71177	1.51425	0.552922

XLOC_052015	TPX2;BCL2L1;COX4I2;FOXS1;MYLK2;PDRG1;XKR7;POFUT1;POFUT1;HCK;TM9SF4;POFUT1;HCK;POFUT1;TTLL9;POFUT1	chr13	61745935	62237095	10.805	3.22988	0.0	36.2544	16.8293	32.4993	20.7576	49.7001	39.8964
XLOC_052016	TPX2;BCL2L1;COX4I2;FOXS1;MYLK2;PDRG1;XKR7;POFUT1;POFUT1;HCK;TM9SF4;POFUT1;HCK;POFUT1;TTLL9;POFUT1	chr13	61745935	62237095	0.0	0.274666	0.0	6.33805	6.10108	6.57333	5.9075	8.17063	6.33677
XLOC_052017	TPX2;BCL2L1;COX4I2;FOXS1;MYLK2;PDRG1;XKR7;POFUT1;POFUT1;HCK;TM9SF4;POFUT1;HCK;POFUT1;TTLL9;POFUT1	chr13	61745935	62237095	2.33712	0.0	0.0	4.18302	4.3342	5.76727	5.57053	6.65896	4.67051
XLOC_052018	TPX2;BCL2L1;COX4I2;FOXS1;MYLK2;PDRG1;XKR7;POFUT1;POFUT1;HCK;TM9SF4;POFUT1;HCK;POFUT1;TTLL9;POFUT1	chr13	61745935	62237095	0.0	0.0	0.716534	6.1578	4.86859	5.79657	3.56887	4.7619	4.9606

XLOC_052019	TPX2;BCL2L1;COX4I2;FOXS1;MYLK2;PDRG1;XKR7;POFUT1;POFUT1;HCK;TM9SF4;POFUT1;HCK;POFUT1;TTLL9;POFUT1	chr13	61745935	62237095	0.0	0.79285	0.0	3.74231	2.80217	4.19929	4.70455	3.12282	3.93888
XLOC_052020	TPX2;BCL2L1;COX4I2;FOXS1;MYLK2;PDRG1;XKR7;POFUT1;POFUT1;HCK;TM9SF4;POFUT1;HCK;POFUT1;TTLL9;POFUT1	chr13	61745935	62237095	0.0	0.672134	0.878926	4.43132	4.2971	4.89035	5.27485	4.25858	4.02168
XLOC_052021	TPX2;BCL2L1;COX4I2;FOXS1;MYLK2;PDRG1;XKR7;POFUT1;POFUT1;HCK;TM9SF4;POFUT1;HCK;POFUT1;TTLL9;POFUT1	chr13	61745935	62237095	0.0	0.0	0.0	0.870501	4.3003	0.0	9.32437	0.891021	0.827152
XLOC_052022	TPX2;BCL2L1;COX4I2;FOXS1;MYLK2;PDRG1;XKR7;POFUT1;POFUT1;HCK;TM9SF4;POFUT1;HCK;POFUT1;TTLL9;POFUT1	chr13	61745935	62237095	0.0	0.0	0.0	1.01309	0.700167	0.465771	2.60158	0.890517	0.983805

XLOC_052023	TPX2;BCL2L1;COX4I2;FOXS1;MYLK2;PDRG1;XKR7;POFUT1;POFUT1;HCK;TM9SF4;POFUT1;HCK;POFUT1;TTLL9;POFUT1	chr13	61745935	62237095	0.0	1.43744	0.0	1.29385	3.66085	2.44704	1.22607	0.0	1.24759
XLOC_052024	TPX2;BCL2L1;COX4I2;FOXS1;MYLK2;PDRG1;XKR7;POFUT1;POFUT1;HCK;TM9SF4;POFUT1;HCK;POFUT1;TTLL9;POFUT1	chr13	61745935	62237095	0.0	0.0	0.0	0.266761	1.37587	1.22196	2.60302	0.872426	1.55135
XLOC_052025	TPX2;BCL2L1;COX4I2;FOXS1;MYLK2;PDRG1;XKR7;POFUT1;POFUT1;HCK;TM9SF4;POFUT1;HCK;POFUT1;TTLL9;POFUT1	chr13	61745935	62237095	0.0	0.0	0.0	0.0	0.422657	0.0	5.15847	0.53097	0.962998
XLOC_052026	TPX2;BCL2L1;COX4I2;FOXS1;MYLK2;PDRG1;XKR7;POFUT1;POFUT1;HCK;TM9SF4;POFUT1;HCK;POFUT1;TTLL9;POFUT1	chr13	61745935	62237095	0.0	0.0	0.0	1.31867	1.14653	0.692219	1.20297	0.266159	0.816852

XLOC_052027	TPX2;BCL2L1;COX4I2;FOXS1;MYLK2;PDRG1;XKR7;POFUT1;POFUT1;HCK;TM9SF4;POFUT1;HCK;POFUT1;TTLL9;POFUT1	chr13	61745935	62237095	0.0	1.0445	0.0	1.56532	3.48598	1.07274	1.21154	0.339387	2.12106
XLOC_052028	TPX2;BCL2L1;COX4I2;FOXS1;MYLK2;PDRG1;XKR7;POFUT1;POFUT1;HCK;TM9SF4;POFUT1;HCK;POFUT1;TTLL9;POFUT1	chr13	61745935	62237095	0.0	0.0	0.0	1.1044	2.01081	0.928629	1.01132	0.33518	1.66238
XLOC_052029	TPX2;BCL2L1;COX4I2;FOXS1;MYLK2;PDRG1;XKR7;POFUT1;POFUT1;HCK;TM9SF4;POFUT1;HCK;POFUT1;TTLL9;POFUT1	chr13	61745935	62237095	0.0	0.12831	0.0	1.07669	2.28801	1.78471	2.9796	0.776254	2.21369
XLOC_052030	TPX2;BCL2L1;COX4I2;FOXS1;MYLK2;PDRG1;XKR7;POFUT1;POFUT1;HCK;TM9SF4;POFUT1;HCK;POFUT1;TTLL9;POFUT1	chr13	61745935	62237095	0.0	0.0	0.0	0.908538	2.59605	2.07665	2.05483	0.0	0.586425

XLOC_052031	TPX2;BCL2L1;COX4I2;FOXS1;MYLK2;PDRG1;XKR7;POFUT1;POFUT1;HCK;TM9SF4;POFUT1;HCK;POFUT1;TTLL9;POFUT1	chr13	61745935	62237095	0.0	0.0	0.0	5.06223	4.752	4.57766	4.92644	7.57797	9.98364
XLOC_052032	TPX2;BCL2L1;COX4I2;FOXS1;MYLK2;PDRG1;XKR7;POFUT1;POFUT1;HCK;TM9SF4;POFUT1;HCK;POFUT1;TTLL9;POFUT1	chr13	61745935	62237095	0.0	0.0	0.0	1.10714	1.28626	1.49408	1.02389	0.822193	1.70776
XLOC_052033	TPX2;BCL2L1;COX4I2;FOXS1;MYLK2;PDRG1;XKR7;POFUT1;POFUT1;HCK;TM9SF4;POFUT1;HCK;POFUT1;TTLL9;POFUT1	chr13	61745935	62237095	18.442	16.1975	25.2367	5.26712	5.84424	5.61109	5.92636	4.82518	6.63832
XLOC_052034	TPX2;BCL2L1;COX4I2;FOXS1;MYLK2;PDRG1;XKR7;POFUT1;POFUT1;HCK;TM9SF4;POFUT1;HCK;POFUT1;TTLL9;POFUT1	chr13	61745935	62237095	0.0	4.16404	0.0	6.356	12.2779	11.1221	7.66002	10.104	14.3212

XLOC_052035	TPX2;BCL2L1;COX4I2;FOXS1;MYLK2;PDRG1;XKR7;POFUT1;POFUT1;HCK;TM9SF4;POFUT1;HCK;POFUT1;TTLL9;POFUT1	chr13	61745935	62237095	3.12385	0.0	0.0	1.11756	1.91947	2.55779	0.81596	2.43286	0.812089
XLOC_052036	TPX2;BCL2L1;COX4I2;FOXS1;MYLK2;PDRG1;XKR7;POFUT1;POFUT1;HCK;TM9SF4;POFUT1;HCK;POFUT1;TTLL9;POFUT1	chr13	61745935	62237095	0.480083	0.0	0.0	0.860915	0.90365	0.848863	1.05235	1.0132	0.9239
XLOC_052037	TPX2;BCL2L1;COX4I2;FOXS1;MYLK2;PDRG1;XKR7;POFUT1;POFUT1;HCK;TM9SF4;POFUT1;HCK;POFUT1;TTLL9;POFUT1	chr13	61745935	62237095	0.38542	0.115323	0.0	1.58982	1.57305	1.36377	0.705282	1.43483	1.2816
XLOC_052038	TPX2;BCL2L1;COX4I2;FOXS1;MYLK2;PDRG1;XKR7;POFUT1;POFUT1;HCK;TM9SF4;POFUT1;HCK;POFUT1;TTLL9;POFUT1	chr13	61745935	62237095	0.0	0.0	1.09687	0.628425	1.31075	0.725501	1.3858	0.418269	0.978592

XLOC_052039	TPX2;BCL2L1;COX4I2;FOXS1;MYLK2;PDRG1;XKR7;POFUT1;POFUT1;HCK;TM9SF4;POFUT1;HCK;POFUT1;TTLL9;POFUT1	chr13	61745935	62237095	0.0	0.0	0.0	1.87691	2.36006	2.049	0.839628	0.927833	2.03095
XLOC_052040	TPX2;BCL2L1;COX4I2;FOXS1;MYLK2;PDRG1;XKR7;POFUT1;POFUT1;HCK;TM9SF4;POFUT1;HCK;POFUT1;TTLL9;POFUT1	chr13	61745935	62237095	0.0	0.0	0.0	0.87303	0.758782	0.863954	0.625183	0.138371	0.849708
XLOC_052041	TPX2;BCL2L1;COX4I2;FOXS1;MYLK2;PDRG1;XKR7;POFUT1;POFUT1;HCK;TM9SF4;POFUT1;HCK;POFUT1;TTLL9;POFUT1	chr13	61745935	62237095	0.0	0.572463	1.49709	1.71546	2.67397	2.76532	1.87484	1.32449	1.66736
XLOC_052042	TPX2;BCL2L1;COX4I2;FOXS1;MYLK2;PDRG1;XKR7;POFUT1;POFUT1;HCK;TM9SF4;POFUT1;HCK;POFUT1;TTLL9;POFUT1	chr13	61745935	62237095	0.0	0.0	0.0	3.76471	2.88947	3.60455	2.06316	0.688865	2.63985

XLOC_052043	TPX2;BCL2L1;COX4I2;FOXS1;MYLK2;PDRG1;XKR7;POFUT1;POFUT1;HCK;TM9SF4;POFUT1;HCK;POFUT1;TTLL9;POFUT1	chr13	61745935	62237095	0.237788	1.20971	0.93059	2.72975	2.39178	2.32834	2.15985	2.22911	2.16518
XLOC_052044	TPX2;BCL2L1;COX4I2;FOXS1;MYLK2;PDRG1;XKR7;POFUT1;POFUT1;HCK;TM9SF4;POFUT1;HCK;POFUT1;TTLL9;POFUT1	chr13	61745935	62237095	0.0	0.0	0.0	1.4903	1.06909	2.8474	0.97282	1.89966	1.92702
XLOC_052045	TPX2;BCL2L1;COX4I2;FOXS1;MYLK2;PDRG1;XKR7;POFUT1;POFUT1;HCK;TM9SF4;POFUT1;HCK;POFUT1;TTLL9;POFUT1	chr13	61745935	62237095	0.0	0.565844	2.21983	1.44137	1.18273	2.06042	0.856639	1.13442	1.15664
XLOC_052046	TPX2;BCL2L1;COX4I2;FOXS1;MYLK2;PDRG1;XKR7;POFUT1;POFUT1;HCK;TM9SF4;POFUT1;HCK;POFUT1;TTLL9;POFUT1	chr13	61745935	62237095	0.0	0.621791	1.62608	0.186326	3.06219	0.214359	0.92301	0.410284	1.44819

XLOC_052047	TPX2;BCL2L1;COX4I2;FOXS1;MYLK2;PDRG1;XKR7;POFUT1;POFUT1;HCK;TM9SF4;POFUT1;HCK;POFUT1;TTLL9;POFUT1	chr13	61745935	62237095	0.0	0.0	0.0	1.34097	1.05976	0.563074	0.489135	0.947058	0.711957
XLOC_052048	TPX2;BCL2L1;COX4I2;FOXS1;MYLK2;PDRG1;XKR7;POFUT1;POFUT1;HCK;TM9SF4;POFUT1;HCK;POFUT1;TTLL9;POFUT1	chr13	61745935	62237095	0.0	0.539072	1.40987	7.18898	6.19919	6.91862	4.98217	7.92948	6.84845
XLOC_052049	TPX2;BCL2L1;COX4I2;FOXS1;MYLK2;PDRG1;XKR7;POFUT1;POFUT1;HCK;TM9SF4;POFUT1;HCK;POFUT1;TTLL9;POFUT1	chr13	61745935	62237095	0.0	0.0	0.0	3.17452	2.6548	4.27736	2.91597	1.99232	6.6883
XLOC_052050	TPX2;BCL2L1;COX4I2;FOXS1;MYLK2;PDRG1;XKR7;POFUT1;POFUT1;HCK;TM9SF4;POFUT1;HCK;POFUT1;TTLL9;POFUT1	chr13	61745935	62237095	0.0	0.0	0.0	1.66123	2.04232	1.92341	0.611441	0.758617	1.39837

XLOC_052051	TPX2;BCL2L1;COX4I2;FOXS1;MYLK2;PDRG1;XKR7;POFUT1;POFUT1;HCK;TM9SF4;POFUT1;HCK;POFUT1;TTLL9;POFUT1	chr13	61745935	62237095	0.0	0.0	0.0	1.43149	3.66573	3.66966	10.6471	1.54354	3.45894
XLOC_052052	TPX2;BCL2L1;COX4I2;FOXS1;MYLK2;PDRG1;XKR7;POFUT1;POFUT1;HCK;TM9SF4;POFUT1;HCK;POFUT1;TTLL9;POFUT1	chr13	61745935	62237095	0.0	0.0	0.0	1.18825	1.17616	0.977379	0.337429	1.49819	0.989999
XLOC_052053	TPX2;BCL2L1;COX4I2;FOXS1;MYLK2;PDRG1;XKR7;POFUT1;POFUT1;HCK;TM9SF4;POFUT1;HCK;POFUT1;TTLL9;POFUT1	chr13	61745935	62237095	0.0	0.0	0.0	0.855467	0.592675	0.788029	0.680021	0.943624	1.16409
XLOC_052054	TPX2;BCL2L1;COX4I2;FOXS1;MYLK2;PDRG1;XKR7;POFUT1;POFUT1;HCK;TM9SF4;POFUT1;HCK;POFUT1;TTLL9;POFUT1	chr13	61745935	62237095	0.0	0.0	0.0	0.317954	0.485315	0.36805	0.241183	0.532125	0.232401

XLOC_052055	TPX2;BCL2L1;COX4I2;FOXS1;MYLK2;PDRG1;XKR7;POFUT1;POFUT1;HCK;TM9SF4;POFUT1;HCK;POFUT1;TTLL9;POFUT1	chr13	61745935	62237095	0.0	0.0	0.0	0.0	0.888869	0.709608	0.0	1.35642	0.999318
XLOC_052056	TPX2;BCL2L1;COX4I2;FOXS1;MYLK2;PDRG1;XKR7;POFUT1;POFUT1;HCK;TM9SF4;POFUT1;HCK;POFUT1;TTLL9;POFUT1	chr13	61745935	62237095	0.0	0.0	0.0	1.76374	0.471272	2.03502	2.57889	3.75783	2.90414
XLOC_052057	TPX2;BCL2L1;COX4I2;FOXS1;MYLK2;PDRG1;XKR7;POFUT1;POFUT1;HCK;TM9SF4;POFUT1;HCK;POFUT1;TTLL9;POFUT1	chr13	61745935	62237095	0.0	0.0	0.0	1.03083	0.345813	1.46862	1.20301	1.50403	1.08189
XLOC_052058	TPX2;BCL2L1;COX4I2;FOXS1;MYLK2;PDRG1;XKR7;POFUT1;POFUT1;HCK;TM9SF4;POFUT1;HCK;POFUT1;TTLL9;POFUT1	chr13	61745935	62237095	16.8493	11.3385	19.1108	3.39797	3.88699	4.19653	4.5858	3.20304	4.48951

XLOC_052059	TPX2;BCL2L1;COX4I2;FOXS1;MYLK2;PDRG1;XKR7;POFUT1;POFUT1;HCK;TM9SF4;POFUT1;HCK;POFUT1;TTLL9;POFUT1	chr13	61745935	62237095	0.0	0.623583	0.0	8.03508	8.40451	6.01915	8.88549	9.66861	6.89863
XLOC_052060	TPX2;BCL2L1;COX4I2;FOXS1;MYLK2;PDRG1;XKR7;POFUT1;POFUT1;HCK;TM9SF4;POFUT1;HCK;POFUT1;TTLL9;POFUT1	chr13	61745935	62237095	0.0	0.947072	0.0	6.38569	7.14377	7.20156	8.93306	9.11152	8.00658
XLOC_052061	TPX2;BCL2L1;COX4I2;FOXS1;MYLK2;PDRG1;XKR7;POFUT1;POFUT1;HCK;TM9SF4;POFUT1;HCK;POFUT1;TTLL9;POFUT1	chr13	61745935	62237095	1.08025	1.18521	0.845423	8.42788	9.58302	9.37016	9.49307	10.4739	9.92651
XLOC_052062	TPX2;BCL2L1;COX4I2;FOXS1;MYLK2;PDRG1;XKR7;POFUT1;POFUT1;HCK;TM9SF4;POFUT1;HCK;POFUT1;TTLL9;POFUT1	chr13	61745935	62237095	1.46334	1.96967	0.0	7.28015	7.55931	6.76237	8.64688	8.06348	7.67336

XLOC_052063	TPX2;BCL2L1;COX4I2;FOXS1;MYLK2;PDRG1;XKR7;POFUT1;POFUT1;HCK;TM9SF4;POFUT1;HCK;POFUT1;TTLL9;POFUT1	chr13	61745935	62237095	1.33011	1.39259	0.520315	4.35227	3.74989	4.14558	3.32936	3.26759	3.48824
XLOC_052064	TPX2;BCL2L1;COX4I2;FOXS1;MYLK2;PDRG1;XKR7;POFUT1;POFUT1;HCK;TM9SF4;POFUT1;HCK;POFUT1;TTLL9;POFUT1	chr13	61745935	62237095	1.27485	0.534109	0.199566	2.49251	2.50419	2.81503	2.26843	2.49249	2.43313
XLOC_052065	TPX2;BCL2L1;COX4I2;FOXS1;MYLK2;PDRG1;XKR7;POFUT1;POFUT1;HCK;TM9SF4;POFUT1;HCK;POFUT1;TTLL9;POFUT1	chr13	61745935	62237095	0.0	0.0	0.0	2.84912	2.70979	1.87848	1.7645	3.15658	1.98009
XLOC_052066	TPX2;BCL2L1;COX4I2;FOXS1;MYLK2;PDRG1;XKR7;POFUT1;POFUT1;HCK;TM9SF4;POFUT1;HCK;POFUT1;TTLL9;POFUT1	chr13	61745935	62237095	0.0	0.0	0.0	2.39208	2.88571	3.06717	2.26137	3.68213	1.81038

XLOC_052067	TPX2;BCL2L1;COX4I2;FOXS1;MYLK2;PDRG1;XKR7;POFUT1;POFUT1;HCK;TM9SF4;POFUT1;HCK;POFUT1;TTLL9;POFUT1	chr13	61745935	62237095	0.0	1.13706	0.0	2.61241	3.06293	4.19905	1.71177	2.27137	2.8752
XLOC_052068	TPX2;BCL2L1;COX4I2;FOXS1;MYLK2;PDRG1;XKR7;POFUT1;POFUT1;HCK;TM9SF4;POFUT1;HCK;POFUT1;TTLL9;POFUT1	chr13	61745935	62237095	0.376785	0.0	0.0	2.70299	2.60262	2.35287	1.93086	2.65402	2.20912
XLOC_052069	TPX2;BCL2L1;COX4I2;FOXS1;MYLK2;PDRG1;XKR7;POFUT1;POFUT1;HCK;TM9SF4;POFUT1;HCK;POFUT1;TTLL9;POFUT1	chr13	61745935	62237095	0.0	0.661702	3.46087	3.37083	2.74165	3.87532	2.54802	2.61578	3.46636
XLOC_052070	TPX2;BCL2L1;COX4I2;FOXS1;MYLK2;PDRG1;XKR7;POFUT1;POFUT1;HCK;TM9SF4;POFUT1;HCK;POFUT1;TTLL9;POFUT1	chr13	61745935	62237095	0.689331	1.10022	1.43881	2.41115	2.65448	3.15963	1.93983	3.49776	2.65564

XLOC_052071	TPX2;BCL2L1;COX4I2;FOXS1;MYLK2;PDRG1;XKR7;POFUT1;POFUT1;HCK;TM9SF4;POFUT1;HCK;POFUT1;TTLL9;POFUT1	chr13	61745935	62237095	0.0	0.0	0.0	3.51228	9.47292	3.85488	8.12721	3.51328	3.3068
XLOC_052072	TPX2;BCL2L1;COX4I2;FOXS1;MYLK2;PDRG1;XKR7;POFUT1;POFUT1;HCK;TM9SF4;POFUT1;HCK;POFUT1;TTLL9;POFUT1	chr13	61745935	62237095	0.0	1.03436	0.676308	3.25478	3.17729	3.14002	2.66587	3.11342	2.19032
XLOC_052073	TPX2;BCL2L1;COX4I2;FOXS1;MYLK2;PDRG1;XKR7;POFUT1;POFUT1;HCK;TM9SF4;POFUT1;HCK;POFUT1;TTLL9;POFUT1	chr13	61745935	62237095	4.55171	0.0	7.09717	4.88493	3.11556	3.23783	2.32403	3.05493	4.71397
XLOC_052074	TPX2;BCL2L1;COX4I2;FOXS1;MYLK2;PDRG1;XKR7;POFUT1;POFUT1;HCK;TM9SF4;POFUT1;HCK;POFUT1;TTLL9;POFUT1	chr13	61745935	62237095	0.0	0.394205	1.03096	1.0632	1.33544	2.18305	0.94868	1.57401	1.37999

XLOC_052075	TPX2;BCL2L1;COX4I2;FOXS1;MYLK2;PDRG1;XKR7;POFUT1;POFUT1;HCK;TM9SF4;POFUT1;HCK;POFUT1;TTLL9;POFUT1	chr13	61745935	62237095	0.0	0.58642	0.0	0.527182	2.43401	0.809157	1.22119	0.581179	0.853901
XLOC_052076	TPX2;BCL2L1;COX4I2;FOXS1;MYLK2;PDRG1;XKR7;POFUT1;POFUT1;HCK;TM9SF4;POFUT1;HCK;POFUT1;TTLL9;POFUT1	chr13	61745935	62237095	0.0	0.310518	0.812112	0.651391	1.13509	1.07633	0.469339	0.310947	1.3598
XLOC_052077	TPX2;BCL2L1;COX4I2;FOXS1;MYLK2;PDRG1;XKR7;POFUT1;POFUT1;HCK;TM9SF4;POFUT1;HCK;POFUT1;TTLL9;POFUT1	chr13	61745935	62237095	0.703901	0.210551	1.10135	0.694091	1.15723	1.09665	0.320114	0.493824	0.553713
XLOC_052078	TPX2;BCL2L1;COX4I2;FOXS1;MYLK2;PDRG1;XKR7;POFUT1;POFUT1;HCK;TM9SF4;POFUT1;HCK;POFUT1;TTLL9;POFUT1	chr13	61745935	62237095	1.08846	0.325457	0.0	2.92597	1.69897	1.57894	1.37617	0.434296	1.04502

XLOC_052079	TPX2;BCL2L1;COX4I2;FOXS1;MYLK2;PDRG1;XKR7;POFUT1;POFUT1;HCK;TM9SF4;POFUT1;HCK;POFUT1;TTLL9;POFUT1	chr13	61745935	62237095	7.30046	7.79649	3.26249	12.8972	10.0968	10.4853	11.3119	16.5504	14.385
XLOC_052080	TPX2;BCL2L1;COX4I2;FOXS1;MYLK2;PDRG1;XKR7;POFUT1;POFUT1;HCK;TM9SF4;POFUT1;HCK;POFUT1;TTLL9;POFUT1	chr13	61745935	62237095	0.0	0.0	0.0	1.52604	1.13825	1.51155	0.438456	1.69636	1.69823
XLOC_052081	TPX2;BCL2L1;COX4I2;FOXS1;MYLK2;PDRG1;XKR7;POFUT1;POFUT1;HCK;TM9SF4;POFUT1;HCK;POFUT1;TTLL9;POFUT1	chr13	61745935	62237095	0.0	0.144594	0.378177	1.60333	1.17498	1.7593	0.971041	1.84559	1.26827
XLOC_052082	TPX2;BCL2L1;COX4I2;FOXS1;MYLK2;PDRG1;XKR7;POFUT1;POFUT1;HCK;TM9SF4;POFUT1;HCK;POFUT1;TTLL9;POFUT1	chr13	61745935	62237095	2.52469	0.753858	0.0	2.0331	0.584411	1.29649	1.55492	1.23749	1.97298

XLOC_052083	TPX2;BCL2L1;COX4I2;FOXS1;MYLK2;PDRG1;XKR7;POFUT1;POFUT1;HCK;TM9SF4;POFUT1;HCK;POFUT1;TTLL9;POFUT1	chr13	61745935	62237095	0.0	0.0	0.0	1.5628	2.42798	0.644885	0.897577	1.73665	1.41294
XLOC_052084	TPX2;BCL2L1;COX4I2;FOXS1;MYLK2;PDRG1;XKR7;POFUT1;POFUT1;HCK;TM9SF4;POFUT1;HCK;POFUT1;TTLL9;POFUT1	chr13	61745935	62237095	0.375027	0.224429	0.293494	1.68149	1.05976	1.99063	1.3728	1.54728	1.50964
XLOC_052085	TPX2;BCL2L1;COX4I2;FOXS1;MYLK2;PDRG1;XKR7;POFUT1;POFUT1;HCK;TM9SF4;POFUT1;HCK;POFUT1;TTLL9;POFUT1	chr13	61745935	62237095	0.0	1.45699	0.0	2.61959	2.07194	3.25887	2.36463	2.39393	2.54269
XLOC_059248	TRAF5	chr16	73894357	73895019	4.30102	0.42854	1.12075	0.0	0.0	0.0	0.0	0.0	0.0
XLOC_061814	TRAFD1	chr17	64316260	64318487	1.39787	1.25485	2.18803	0.0940178	0.137188	0.21827	0.127987	0.0703565	0.30585
XLOC_061815	TRAFD1	chr17	64318933	64319843	1.92229	1.72458	3.00693	0.172274	0.150181	0.199341	0.348023	0.0960248	0.16786
XLOC_061816	TRAFD1	chr17	64320774	64321529	1.2105	1.44764	3.78605	0.108456	0.377529	0.376004	0.654454	0.0	0.0
XLOC_061817	TRAFD1	chr17	64323454	64323633	44.1465	6.66998	17.4403	0.0	1.60757	0.0	16.9694	0.0	0.0
XLOC_061818	TRAFD1	chr17	64329575	64331231	4.34374	4.90961	2.64371	0.302928	0.264966	0.301194	0.661197	0.0970071	0.126658
XLOC_066397	TRAK2;STRADB	chr2	90357298	90449862	6.73327	2.87147	2.43766	4.63736	1.9558	2.14756	2.25213	2.27391	3.58418
XLOC_068080	TRAK2;STRADB	chr2	90357298	90449862	4.94569	1.47992	2.32243	2.72028	1.9418	1.75064	1.60055	1.99186	1.84692

XLOC_092283	TRAPPC2	chrX	136993217	136994775	0.0	0.0	0.946513	0.433829	1.09554	0.905154	0.501427	0.589148	1.15727
XLOC_093649	TRAPPC2	chrX	136995211	136996614	0.0	0.868586	1.81739	0.832978	1.09213	1.02611	0.423499	0.29143	0.710885
XLOC_062443	TRAPPC6A	chr18	53220954	53222020	3.0582	5.487	0.797247	14.0683	11.9406	11.307	13.0896	15.3676	12.5488
XLOC_053765	TRAPPC9	chr14	4503960	4504708	0.0	0.0	0.0	0.658443	0.572942	0.634046	1.21374	0.975813	1.81654
XLOC_053766	TRAPPC9	chr14	4511502	4512422	0.0	0.0	0.741883	1.36014	2.8904	2.06579	2.57654	1.80082	1.82233
XLOC_038356	TRAT1	chr1	53930877	54040521	3.44787	4.3544	5.35926	0.230284	0.204671	0.0	0.466063	0.171346	0.152425
XLOC_053344	TRHR	chr14	57423789	57517479	8.24703	9.23524	8.041	8.08442	10.5582	9.03995	8.03208	7.37123	12.5927
XLOC_054504	TRHR	chr14	57517755	57518328	3.48162	1.04038	2.72081	1.40295	2.16247	1.97615	1.3981	1.55044	2.72847
XLOC_054505	TRHR	chr14	57523517	57524600	3.12588	0.934957	0.611321	9.10625	4.21876	7.38274	5.39424	8.60713	3.14112
XLOC_044970	TRIB2	chr11	85190081	85235437	49.9894	51.5148	38.43	33.6262	30.4529	32.7012	38.333	40.4099	33.3327
XLOC_048635	TRIM13	chr12	19553913	19554621	0.0	0.0	0.0	0.470817	1.33063	0.407847	0.472658	0.130698	0.6875
XLOC_064515	TRIM25	chr19	7959786	7959905	1140.09	1618.85	1307.42	6645.83	4688.44	5421.7	4219.59	3625.77	4832.84
XLOC_055420	TRIM29	chr15	31224287	31274346	11.1384	5.15009	3.16937	4.22174	3.96996	4.58065	2.81939	2.64501	3.36561
XLOC_041443	TRIM36	chr10	3740384	3966972	3.24105	2.6791	3.39885	6.26837	2.63189	4.30517	2.1336	3.10752	1.81973
XLOC_063905	TRIM37	chr19	10041621	10196935	40.7646	31.2449	28.7146	25.8084	32.3595	26.8488	36.5483	27.2857	25.4583
XLOC_063906	TRIM37	chr19	10199001	10224526	4.82957	6.13633	5.7936	12.4371	10.6729	11.8418	11.8658	13.2981	13.5915
XLOC_064171	TRIM37	chr19	10041621	10196935	40.8813	49.4319	45.1309	29.0356	33.2218	34.1129	27.4059	24.3923	23.7357
XLOC_064172	TRIM37	chr19	10199001	10224526	21.4124	29.2597	19.847	59.8668	49.7292	54.3711	60.0793	70.7282	64.1948
XLOC_064173	TRIM37	chr19	10199001	10224526	1.84129	1.7908	2.45558	4.33576	2.7196	4.05628	2.16998	5.68456	1.4741
XLOC_064174	TRIM37	chr19	10224584	10240652	1.92624	4.33438	0.0	8.84924	6.329	7.64912	5.14357	11.0371	1.42442
XLOC_064175	TRIM37	chr19	10245265	10304842	0.664657	1.63138	1.51179	0.536429	0.530352	0.349041	0.0733227	0.19921	0.104818
XLOC_064568	TRIM37	chr19	10041621	10196935	4.45102	3.54771	4.63911	1.06315	0.577141	0.613434	1.06417	0.147285	0.517251
XLOC_064569	TRIM37	chr19	10041621	10196935	6.00576	6.00245	8.22383	1.99436	2.99631	2.43564	2.14901	1.36886	1.75731
XLOC_064570	TRIM37	chr19	10199001	10224526	0.0	2.9379	1.28049	3.52152	2.41868	0.50753	1.61117	1.78559	2.1408
XLOC_064571	TRIM37	chr19	10224584	10240652	0.0	1.17923	0.0	1.94353	0.152943	1.42366	0.350746	1.94756	0.0
XLOC_064572	TRIM37	chr19	10224584	10240652	0.0	0.0	0.0	0.238875	0.0	0.275875	0.359593	0.265189	0.116265
XLOC_064573	TRIM37	chr19	10245265	10304842	0.0	0.0	0.0	0.0	0.0	0.0	0.125271	0.0	0.0
XLOC_064574	TRIM37	chr19	10245265	10304842	0.0	0.0	0.0	0.0	0.0	0.0923505	0.0	0.0	0.0
XLOC_064575	TRIM37	chr19	10245265	10304842	0.980694	0.0	0.0	0.0	0.0766079	0.0	0.0	0.0	0.0
XLOC_064576	TRIM37	chr19	10245265	10304842	0.0	0.543129	0.710242	0.162769	0.0	0.0	0.0	0.0	0.0793088
XLOC_064577	TRIM37	chr19	10245265	10304842	1.00803	0.301438	0.0	0.180673	0.0	0.209005	0.0911712	0.0	0.0880097
XLOC_056851	TRIM44	chr15	67048037	67049128	0.0	0.463475	0.606086	0.625034	0.303104	1.44787	0.422264	0.0775824	0.677017

XLOC_044898	TRIM54;DNAJC5G	chr11	72344703	72361861	0.0	1.02456	2.66421	5.9428	7.0256	7.37894	5.50711	8.24666	8.16654
XLOC_053255	TRIM55	chr14	32159774	32177529	18.8991	21.305	12.0471	38.856	37.798	33.8387	35.3276	41.7616	49.1092
XLOC_054140	TRIM55	chr14	32159774	32177529	0.0	0.0	1.83712	1.68406	1.99918	0.725531	1.24561	0.924281	3.27
XLOC_053256	TRIM55;CRH	chr14	32180134	32299624	18.0951	23.5595	20.1417	24.8245	27.7331	25.4096	27.9144	29.539	35.9401
XLOC_053535	TRIM55;CRH	chr14	32180134	32299624	21.0252	15.042	48.0215	103.649	80.1386	81.4895	98.8508	122.113	94.5938
XLOC_054141	TRIM55;CRH	chr14	32180134	32299624	3.52531	3.22209	3.6475	2.3943	5.00587	4.73396	4.52878	3.15096	3.14819
XLOC_054142	TRIM55;CRH	chr14	32180134	32299624	6.01934	2.99704	1.56758	5.20904	4.66347	3.72103	4.45472	3.76057	5.41083
XLOC_054143	TRIM55;CRH	chr14	32180134	32299624	0.0	1.36084	7.11753	2.65069	4.40311	2.109	5.03333	4.25595	5.14775
XLOC_054144	TRIM55;CRH	chr14	32180134	32299624	0.0	4.34081	3.78333	4.34168	9.57943	9.3591	6.16826	6.96018	7.11596
XLOC_054145	TRIM55;CRH	chr14	32180134	32299624	12.4159	12.3426	9.6821	9.25222	16.7271	12.641	16.3029	6.77425	16.8052
XLOC_054146	TRIM55;CRH	chr14	32180134	32299624	2.16893	0.647841	5.0826	2.91205	5.70583	2.90211	4.41719	3.84356	3.7713
XLOC_054147	TRIM55;CRH	chr14	32180134	32299624	5.85548	3.49097	0.0	5.24463	9.2836	10.067	8.32249	6.10416	8.06598
XLOC_054148	TRIM55;CRH	chr14	32180134	32299624	0.0	9.3108	0.0	7.52613	7.71211	12.5006	6.66898	5.74419	9.81051
XLOC_054149	TRIM55;CRH	chr14	32180134	32299624	4.54594	2.44715	5.68909	4.56321	6.53761	5.84741	5.0253	4.54464	6.82911
XLOC_054150	TRIM55;CRH	chr14	32180134	32299624	15.2663	9.10223	19.8326	9.56173	13.8881	9.2881	18.4788	11.1655	14.0379
XLOC_054151	TRIM55;CRH	chr14	32180134	32299624	3.41586	4.08302	8.00853	2.44716	5.96865	4.05473	6.1001	1.69093	5.05702
XLOC_054152	TRIM55;CRH	chr14	32180134	32299624	11.0214	4.11286	10.7552	2.71156	2.54672	3.67391	4.5875	2.15518	5.25935
XLOC_054153	TRIM55;CRH	chr14	32180134	32299624	0.0	0.0	0.0	2.4108	5.29985	2.72805	3.17156	1.02525	2.7846
XLOC_054154	TRIM55;CRH	chr14	32180134	32299624	12.4501	0.0	38.9682	5.67284	9.19755	7.48076	3.95001	9.10197	7.48459
XLOC_065835	TRIM62	chr2	121357631	121363747	0.646036	0.193253	0.0	17.9485	20.2379	21.6002	10.6449	14.2163	24.5778
XLOC_066502	TRIM62	chr2	121310812	121355332	9.14271	8.62837	6.90835	5.87574	7.2641	7.04277	6.17563	7.2986	7.99756
XLOC_041525	TRIM9	chr10	43873972	44004716	3.42078	2.03023	0.897312	7.88557	8.6339	7.08701	8.51852	7.4792	7.33615
XLOC_041927	TRIM9	chr10	43873972	44004716	6.91384	8.43651	5.91142	13.0121	10.4497	11.1448	11.3412	12.1527	11.439
XLOC_041928	TRIM9	chr10	43873972	44004716	3.64977	4.09793	4.79402	1.37544	1.00582	0.858279	0.626267	1.04173	0.492474

XLOC_042663	TRIM9	chr10	43873972	44004716	0.0	1.36117	3.55991	1.32572	1.86459	2.12207	1.23228	1.58843	1.29116
XLOC_042664	TRIM9	chr10	43873972	44004716	0.0	0.485839	0.0	0.509594	0.38118	0.252912	0.147449	0.406458	0.354807
XLOC_042665	TRIM9	chr10	43873972	44004716	0.0	0.589613	0.0	0.883423	0.458843	1.83044	0.0	0.779023	0.858528
XLOC_078662	TRIOBP	chr5	110093527	110093885	3.60516	0.0	0.0	4.51337	0.275936	0.736065	0.622746	1.39635	0.93582
XLOC_076453	TRIOBP;NOL12;LGALS1;MIR2438;PDXP;GGA1;CDC42EP1;TUBA8;PEX26;LGALS2;LGALS2	chr5	109723863	110086945	0.0	1.16138	0.0	3.04176	3.8941	3.20237	7.92948	3.27148	4.09885
XLOC_076454	TRIOBP;NOL12;LGALS1;MIR2438;PDXP;GGA1;CDC42EP1;TUBA8;PEX26;LGALS2;LGALS2	chr5	109723863	110086945	0.0	0.0	0.0	4.99801	7.93998	9.39083	11.351	10.5416	10.132
XLOC_076455	TRIOBP;NOL12;LGALS1;MIR2438;PDXP;GGA1;CDC42EP1;TUBA8;PEX26;LGALS2;LGALS2	chr5	109723863	110086945	0.0	0.0	0.0	5.95683	7.43228	7.30176	8.24599	6.95367	9.65095
XLOC_076456	TRIOBP;NOL12;LGALS1;MIR2438;PDXP;GGA1;CDC42EP1;TUBA8;PEX26;LGALS2;LGALS2	chr5	109723863	110086945	0.0	0.678061	0.0	2.65428	3.47487	2.71237	2.57691	3.51964	3.22399
XLOC_076965	TRIOBP;NOL12;LGALS1;MIR2438;PDXP;GGA1;CDC42EP1;TUBA8;PEX26;LGALS2;LGALS2	chr5	109723863	110086945	0.0	0.0	0.0	3.15789	5.2105	3.36612	4.56123	2.70808	4.33274

XLOC_076966	TRIOBP;NOL12;LGALS1;MIR2438;PDXP;GGA1;CDC42EP1;TUBA8;PEX26;LGALS2;LGALS2	chr5	109723863	110086945	0.0	0.0	0.0	9.01875	7.0816	7.5948	11.727	15.9993	12.1154
XLOC_078616	TRIOBP;NOL12;LGALS1;MIR2438;PDXP;GGA1;CDC42EP1;TUBA8;PEX26;LGALS2;LGALS2	chr5	109723863	110086945	0.0	0.0	0.0	1.01744	1.25665	0.588691	1.19929	0.37814	1.07403
XLOC_078617	TRIOBP;NOL12;LGALS1;MIR2438;PDXP;GGA1;CDC42EP1;TUBA8;PEX26;LGALS2;LGALS2	chr5	109723863	110086945	0.0	0.0	0.0	1.1536	2.78436	2.11806	2.94662	0.505253	2.68638
XLOC_078618	TRIOBP;NOL12;LGALS1;MIR2438;PDXP;GGA1;CDC42EP1;TUBA8;PEX26;LGALS2;LGALS2	chr5	109723863	110086945	0.0	0.0	0.0	0.96939	0.691313	0.509802	0.533882	0.0982177	0.686907
XLOC_078619	TRIOBP;NOL12;LGALS1;MIR2438;PDXP;GGA1;CDC42EP1;TUBA8;PEX26;LGALS2;LGALS2	chr5	109723863	110086945	0.0	2.39499	0.0	1.43536	1.64864	1.64632	1.87714	1.04667	2.78473

XLOC_078620	TRIOBP;NOL12;LGALS1;MIR2438;PDXP;GGA1;CDC42EP1;TUBA8;PEX26;LGALS2;LGALS2	chr5	109723863	110086945	0.591123	0.176847	0.925023	1.00694	1.25064	1.59746	0.862062	0.711918	1.08546
XLOC_078621	TRIOBP;NOL12;LGALS1;MIR2438;PDXP;GGA1;CDC42EP1;TUBA8;PEX26;LGALS2;LGALS2	chr5	109723863	110086945	1.1751	4.21608	1.83773	5.26441	5.31558	5.598	5.08582	6.08884	6.35707
XLOC_078622	TRIOBP;NOL12;LGALS1;MIR2438;PDXP;GGA1;CDC42EP1;TUBA8;PEX26;LGALS2;LGALS2	chr5	109723863	110086945	0.839528	0.0	0.0	1.42971	1.18176	1.13262	0.76168	1.092	0.73343
XLOC_078623	TRIOBP;NOL12;LGALS1;MIR2438;PDXP;GGA1;CDC42EP1;TUBA8;PEX26;LGALS2;LGALS2	chr5	109723863	110086945	0.0	0.0	0.0	0.567123	1.14437	0.652344	2.24633	0.208057	3.4893
XLOC_078624	TRIOBP;NOL12;LGALS1;MIR2438;PDXP;GGA1;CDC42EP1;TUBA8;PEX26;LGALS2;LGALS2	chr5	109723863	110086945	0.0	0.0	0.0	0.882105	0.760987	1.26603	2.82187	0.483534	2.14038

XLOC_078625	TRIOBP;NOL12;LGALS1;MIR2438;PDXP;GGA1;CDC42EP1;TUBA8;PEX26;LGALS2;LGALS2	chr5	109723863	110086945	0.0	0.0	0.0	0.369891	0.643039	1.1389	2.72018	0.684061	2.04013
XLOC_078626	TRIOBP;NOL12;LGALS1;MIR2438;PDXP;GGA1;CDC42EP1;TUBA8;PEX26;LGALS2;LGALS2	chr5	109723863	110086945	0.0	0.0	0.0	1.67693	1.28935	1.50051	3.32283	1.02569	3.07741
XLOC_078627	TRIOBP;NOL12;LGALS1;MIR2438;PDXP;GGA1;CDC42EP1;TUBA8;PEX26;LGALS2;LGALS2	chr5	109723863	110086945	0.0	0.0	0.0	0.0	5.77967	0.0	0.0	0.0	0.0
XLOC_078628	TRIOBP;NOL12;LGALS1;MIR2438;PDXP;GGA1;CDC42EP1;TUBA8;PEX26;LGALS2;LGALS2	chr5	109723863	110086945	0.0	0.0	0.0	1.403	2.40948	1.92651	3.82341	1.83225	3.26228
XLOC_078629	TRIOBP;NOL12;LGALS1;MIR2438;PDXP;GGA1;CDC42EP1;TUBA8;PEX26;LGALS2;LGALS2	chr5	109723863	110086945	0.0	0.0	0.0	2.04411	3.3354	3.38418	8.98521	3.10029	5.79911

XLOC_078630	TRIOBP;NOL12;LGALS1;MIR2438;PDXP;GGA1;CDC42EP1;TUBA8;PEX26;LGALS2;LGALS2	chr5	109723863	110086945	0.0	0.0	0.0	8.13898	4.21525	4.1002	5.97783	4.82262	8.71394
XLOC_078631	TRIOBP;NOL12;LGALS1;MIR2438;PDXP;GGA1;CDC42EP1;TUBA8;PEX26;LGALS2;LGALS2	chr5	109723863	110086945	0.0	2.9862	0.0	1.80873	10.4024	2.00596	3.21743	4.61509	1.71678
XLOC_078632	TRIOBP;NOL12;LGALS1;MIR2438;PDXP;GGA1;CDC42EP1;TUBA8;PEX26;LGALS2;LGALS2	chr5	109723863	110086945	0.0	0.0	0.0	6.34906	9.02626	6.41604	11.0807	7.96917	6.08027
XLOC_078633	TRIOBP;NOL12;LGALS1;MIR2438;PDXP;GGA1;CDC42EP1;TUBA8;PEX26;LGALS2;LGALS2	chr5	109723863	110086945	0.0	0.0	0.0	4.13125	2.79807	3.10085	7.48381	5.34381	3.83995
XLOC_078634	TRIOBP;NOL12;LGALS1;MIR2438;PDXP;GGA1;CDC42EP1;TUBA8;PEX26;LGALS2;LGALS2	chr5	109723863	110086945	0.0	0.0	0.0	7.03948	6.82527	7.59849	9.96775	6.66525	8.98791

XLOC_078635	TRIOBP;NOL12;LGALS1;MIR2438;PDXP;GGA1;CDC42EP1;TUBA8;PEX26;LGALS2;LGALS2	chr5	109723863	110086945	0.770644	0.230514	0.0	1.45065	1.0251	2.16028	1.61018	0.848903	1.48152
XLOC_078636	TRIOBP;NOL12;LGALS1;MIR2438;PDXP;GGA1;CDC42EP1;TUBA8;PEX26;LGALS2;LGALS2	chr5	109723863	110086945	0.0	0.0	0.0	5.82466	5.94838	3.6312	4.15434	2.02834	3.09764
XLOC_078637	TRIOBP;NOL12;LGALS1;MIR2438;PDXP;GGA1;CDC42EP1;TUBA8;PEX26;LGALS2;LGALS2	chr5	109723863	110086945	0.0	0.487418	1.27466	0.584231	0.633665	2.3579	1.16662	1.45448	1.13654
XLOC_078638	TRIOBP;NOL12;LGALS1;MIR2438;PDXP;GGA1;CDC42EP1;TUBA8;PEX26;LGALS2;LGALS2	chr5	109723863	110086945	1.05202	3.1458	1.64547	9.14447	6.57042	7.74135	6.17973	6.92916	8.72439
XLOC_078639	TRIOBP;NOL12;LGALS1;MIR2438;PDXP;GGA1;CDC42EP1;TUBA8;PEX26;LGALS2;LGALS2	chr5	109723863	110086945	3.15096	0.0	2.45898	4.50907	4.8394	2.9021	4.93603	4.90649	5.46053

XLOC_078640	TRIOBP;NOL12;LGALS1;MIR2438;PDXP;GGA1;CDC42EP1;TUBA8;PEX26;LGALS2;LGALS2	chr5	109723863	110086945	0.0	1.10852	0.0	7.97455	7.67297	10.236	7.36807	7.54677	8.99948
XLOC_078641	TRIOBP;NOL12;LGALS1;MIR2438;PDXP;GGA1;CDC42EP1;TUBA8;PEX26;LGALS2;LGALS2	chr5	109723863	110086945	0.0	0.794211	0.0	9.87697	7.13959	6.73431	8.95762	7.39845	10.7731
XLOC_078642	TRIOBP;NOL12;LGALS1;MIR2438;PDXP;GGA1;CDC42EP1;TUBA8;PEX26;LGALS2;LGALS2	chr5	109723863	110086945	0.0	7.68651	10.0458	40.9767	27.4714	28.2691	19.3021	24.5927	34.1703
XLOC_078643	TRIOBP;NOL12;LGALS1;MIR2438;PDXP;GGA1;CDC42EP1;TUBA8;PEX26;LGALS2;LGALS2	chr5	109723863	110086945	0.0	1.37428	0.0	2.06112	1.40165	4.68261	3.52699	2.20835	2.7841
XLOC_078644	TRIOBP;NOL12;LGALS1;MIR2438;PDXP;GGA1;CDC42EP1;TUBA8;PEX26;LGALS2;LGALS2	chr5	109723863	110086945	0.0	2.15706	0.940226	3.23208	3.37539	4.60677	4.55138	3.71313	4.61602

XLOC_078645	TRIOBP;NOL12;LGALS1;MIR2438;PDXP;GGA1;CDC42EP1;TUBA8;PEX26;LGALS2;LGALS2	chr5	109723863	110086945	1.25243	0.748879	0.979252	4.71273	3.90474	3.75961	4.0596	5.73531	5.35325
XLOC_078646	TRIOBP;NOL12;LGALS1;MIR2438;PDXP;GGA1;CDC42EP1;TUBA8;PEX26;LGALS2;LGALS2	chr5	109723863	110086945	1.53078	0.915066	1.19654	2.1937	2.38097	1.89818	1.37127	1.97436	1.73414
XLOC_078647	TRIOBP;NOL12;LGALS1;MIR2438;PDXP;GGA1;CDC42EP1;TUBA8;PEX26;LGALS2;LGALS2	chr5	109723863	110086945	0.0	2.38446	0.0	1.60768	1.08223	1.02798	2.83581	0.787413	1.21511
XLOC_078648	TRIOBP;NOL12;LGALS1;MIR2438;PDXP;GGA1;CDC42EP1;TUBA8;PEX26;LGALS2;LGALS2	chr5	109723863	110086945	0.947295	2.54964	2.22274	3.65059	2.88665	2.84904	2.23013	2.17712	3.14356
XLOC_078649	TRIOBP;NOL12;LGALS1;MIR2438;PDXP;GGA1;CDC42EP1;TUBA8;PEX26;LGALS2;LGALS2	chr5	109723863	110086945	0.0	0.0	2.50421	1.43473	0.871609	2.31653	1.57651	0.952807	1.53521

XLOC_078650	TRIOBP;NOL12;LGALS1;MIR2438;PDXP;GGA1;CDC42EP1;TUBA8;PEX26;LGALS2;LGALS2	chr5	109723863	110086945	0.0	3.98241	5.20722	3.38117	3.4374	2.97246	1.57264	2.40496	2.31795
XLOC_078651	TRIOBP;NOL12;LGALS1;MIR2438;PDXP;GGA1;CDC42EP1;TUBA8;PEX26;LGALS2;LGALS2	chr5	109723863	110086945	0.0	2.32563	6.07949	3.50575	2.33474	2.35424	7.02255	1.45732	3.34984
XLOC_078652	TRIOBP;NOL12;LGALS1;MIR2438;PDXP;GGA1;CDC42EP1;TUBA8;PEX26;LGALS2;LGALS2	chr5	109723863	110086945	0.0	0.326946	0.0	1.17569	0.426655	1.58606	1.67849	1.52682	1.24061
XLOC_078653	TRIOBP;NOL12;LGALS1;MIR2438;PDXP;GGA1;CDC42EP1;TUBA8;PEX26;LGALS2;LGALS2	chr5	109723863	110086945	1.4746	0.88153	1.15269	1.05666	1.4915	0.914573	1.05784	1.0248	0.771156
XLOC_078654	TRIOBP;NOL12;LGALS1;MIR2438;PDXP;GGA1;CDC42EP1;TUBA8;PEX26;LGALS2;LGALS2	chr5	109723863	110086945	0.0	1.45702	0.0	0.218303	0.565078	0.0	2.14966	0.239392	0.635675

XLOC_078655	TRIOBP;NOL12;LGALS1;MIR2438;PDXP;GGA1;CDC42EP1;TUBA8;PEX26;LGALS2;LGALS2	chr5	109723863	110086945	0.0	0.0	0.0	0.0	0.0	0.0	1.07773	0.0	1.17415
XLOC_078656	TRIOBP;NOL12;LGALS1;MIR2438;PDXP;GGA1;CDC42EP1;TUBA8;PEX26;LGALS2;LGALS2	chr5	109723863	110086945	0.0	1.37859	1.44219	4.79236	3.81864	4.30326	3.25716	3.77823	3.94538
XLOC_078657	TRIOBP;NOL12;LGALS1;MIR2438;PDXP;GGA1;CDC42EP1;TUBA8;PEX26;LGALS2;LGALS2	chr5	109723863	110086945	0.0	0.502114	0.0	0.752305	1.17458	1.04073	0.750485	0.665561	0.585328
XLOC_078658	TRIOBP;NOL12;LGALS1;MIR2438;PDXP;GGA1;CDC42EP1;TUBA8;PEX26;LGALS2;LGALS2	chr5	109723863	110086945	0.0	0.0	0.0	2.20426	1.27135	1.69085	1.09248	0.6069	0.713902
XLOC_078659	TRIOBP;NOL12;LGALS1;MIR2438;PDXP;GGA1;CDC42EP1;TUBA8;PEX26;LGALS2;LGALS2	chr5	109723863	110086945	0.0	0.0	0.0	12.2374	7.39219	7.6304	5.26974	12.7143	10.2895

XLOC_078660	TRIOBP;NOL12;LGALS1;MIR2438;PDXP;GGA1;CDC42EP1;TUBA8;PEX26;LGALS2;LGALS2	chr5	109723863	110086945	0.0	0.0	0.0	2.69437	3.42597	5.09092	1.27282	4.31208	0.865503
XLOC_078661	TRIOBP;NOL12;LGALS1;MIR2438;PDXP;GGA1;CDC42EP1;TUBA8;PEX26;LGALS2;LGALS2	chr5	109723863	110086945	0.0	1.43317	0.0	2.29051	1.9879	1.81615	1.43012	2.05994	1.94961
XLOC_065798	TRIP12	chr2	118598228	118641691	35.9991	37.2728	27.7203	130.717	119.598	121.211	147.043	155.969	154.095
XLOC_065799	TRIP12	chr2	118663566	118697239	6.8521	9.59542	12.7734	1.41664	1.42586	2.17782	0.921314	1.54405	0.98913
XLOC_066471	TRIP12	chr2	118718075	118723798	0.0	1.13887	0.496217	0.113604	0.0496311	0.263809	0.0	0.190668	0.0
XLOC_068334	TRIP12	chr2	118641849	118643310	1.10969	0.331983	1.30242	1.29339	1.30469	1.09597	0.556668	0.501392	1.35863
XLOC_068335	TRIP12	chr2	118643690	118643995	9.77132	5.82624	0.0	0.874107	4.07901	4.958	0.413756	0.933889	2.10673
XLOC_068336	TRIP12	chr2	118644112	118644517	5.84431	0.872249	4.5618	2.87528	2.92221	1.79657	3.06379	1.71081	3.54749
XLOC_068337	TRIP12	chr2	118663566	118697239	0.0	1.71685	0.0	0.514489	0.0	0.589501	1.00585	0.280757	0.0
XLOC_068338	TRIP12	chr2	118697327	118697997	7.05645	5.90603	6.61967	1.89629	0.439443	0.875653	0.633501	1.12179	0.492146
XLOC_089319	TRMT11	chr9	25481610	25604107	7.41826	5.61489	4.14022	1.84947	1.36618	1.15471	1.29931	1.74029	1.06511
XLOC_051776	TRMT6	chr13	48484776	48485559	3.46893	5.53164	5.42514	1.65771	1.26275	0.958111	0.625779	1.49822	0.706307
XLOC_051777	TRMT6	chr13	48485723	48486254	3.87318	6.94299	4.53929	0.520138	0.7506	1.39729	0.688805	0.382382	2.02212
XLOC_077564	TROAP	chr5	30658616	30658724	0.0	1410.59	0.0	494.119	0.0	220.6	0.0	176.179	0.0
XLOC_077565	TROAP	chr5	30658886	30659851	8.95674	8.30391	13.3109	3.37157	2.59045	3.53093	2.67886	3.58224	2.58165
XLOC_038025	TRPC1;PLS1;PCOLCE2	chr1	127204992	127483006	11.8435	11.2814	7.46843	3.72361	5.17469	4.68115	4.89311	3.91179	4.26265
XLOC_048925	TRPC4	chr12	24043752	24043934	0.0	0.0	0.0	11.5535	7.52789	6.1978	4.77545	11.0739	8.89613
XLOC_048926	TRPC4	chr12	24044405	24044815	0.0	1.71008	2.23591	5.38081	7.71526	8.2209	10.0207	6.71239	9.93714
XLOC_048927	TRPC4	chr12	24058660	24061794	0.0	0.21814	0.0	0.523003	0.534606	0.658052	0.445818	0.514306	0.489285
XLOC_048928	TRPC4	chr12	24067835	24068324	0.0	0.0	0.0	0.976499	1.01287	1.3474	2.12478	2.57735	0.758775
XLOC_048929	TRPC4	chr12	24072918	24073052	229.958	0.0	95.3853	167.736	106.056	200.036	93.1998	137.375	286.749
XLOC_048930	TRPC4	chr12	24079341	24080397	0.0	0.0	0.0	0.577211	0.503736	0.33422	0.876936	0.402874	0.562651
XLOC_048931	TRPC4	chr12	24085646	24086043	3.01963	1.80254	2.35677	3.24109	4.41013	4.63867	3.95081	5.88631	4.71167

[truncated: 161,405 more chars]
